# Identification of Genes Associated with the Impairment of Olfactory and Gustatory Functions in COVID-19 via Machine-Learning Methods

**DOI:** 10.3390/life13030798

**Published:** 2023-03-15

**Authors:** Jingxin Ren, Yuhang Zhang, Wei Guo, Kaiyan Feng, Ye Yuan, Tao Huang, Yu-Dong Cai

**Affiliations:** 1 School of Life Sciences, Shanghai University, Shanghai 200444, China; 2 Channing Division of Network Medicine, Brigham and Women’s Hospital, Harvard Medical School, Boston, MA 02115, USA; 3 Key Laboratory of Stem Cell Biology, Shanghai Jiao Tong University School of Medicine (SJTUSM) & Shanghai Institutes for Biological Sciences (SIBS), Chinese Academy of Sciences (CAS), Shanghai 200030, China; 4 Department of Computer Science, Guangdong AIB Polytechnic College, Guangzhou 510507, China; 5 Institute of Image Processing and Pattern Recognition, Shanghai Jiao Tong University, and Key Laboratory of System Control and Information Processing, Ministry of Education of China, Shanghai 200240, China; 6 Bio-Med Big Data Center, CAS Key Laboratory of Computational Biology, Shanghai Institute of Nutrition and Health, University of Chinese Academy of Sciences, Chinese Academy of Sciences, Shanghai 200031, China; 7 CAS Key Laboratory of Tissue Microenvironment and Tumor, Shanghai Institute of Nutrition and Health, University of Chinese Academy of Sciences, Chinese Academy of Sciences, Shanghai 200031, China

**Keywords:** COVID-19, olfactory function, gustatory function, gene identification, machine learning

## Abstract

The coronavirus disease 2019 (COVID-19), as a severe respiratory disease, affects many parts of the body, and approximately 20–85% of patients exhibit functional impairment of the senses of smell and taste, some of whom even experience the permanent loss of these senses. These symptoms are not life-threatening but severely affect patients’ quality of life and increase the risk of depression and anxiety. The pathological mechanisms of these symptoms have not been fully identified. In the current study, we aimed to identify the important biomarkers at the expression level associated with the severe acute respiratory syndrome coronavirus 2 (SARS-CoV-2) infection-mediated loss of taste or olfactory ability, and we have suggested the potential pathogenetic mechanisms of COVID-19 complications. We designed a machine-learning-based approach to analyze the transcriptome of 577 COVID-19 patient samples, including 84 COVID-19 samples with a decreased ability to taste or smell and 493 COVID-19 samples without impairment. Each sample was represented by 58,929 gene expression levels. The features were analyzed and sorted by three feature selection methods (least absolute shrinkage and selection operator, light gradient boosting machine, and Monte Carlo feature selection). The optimal feature sets were obtained through incremental feature selection using two classification algorithms: decision tree (DT) and random forest (RF). The top genes identified by these multiple methods (H3-5, NUDT5, and AOC1) are involved in olfactory and gustatory impairments. Meanwhile, a high-performance RF classifier was developed in this study, and three sets of quantitative rules that describe the impairment of olfactory and gustatory functions were obtained based on the optimal DT classifiers. In summary, this study provides a new computation analysis and suggests the latent biomarkers (genes and rules) for predicting olfactory and gustatory impairment caused by COVID-19 complications.

## 1. Introduction

The coronavirus disease 2019, which is also known as COVID-19, is a respiratory infectious disease caused by the severe acute respiratory syndrome coronavirus 2 (SARS-CoV-2) virus and has spread worldwide [1]. With more than 500 million confirmed cases and 6 million deaths, COVID-19 has been one of the most severe and life-threatening infectious diseases [1,2]. In general, the specific symptoms of COVID-19 may appear 2–14 days after infection by the virus, including fever, chills, cough, fatigue, and loss of taste or smell [3,4]. One of the most typical symptoms and the long-term sequela of COVID-19 is a reduced sense of smell or taste (also known as hypogeusia/ageusia or hyposmia/anosmia) [5,6], which has been widely observed in patients with COVID-19 worldwide.

Early in May 2020, researchers from the Guy’s and St. Thomas’ Hospitals observed the renewed onset of smell and taste disturbances in patients with COVID-19 [5]; of the 382 patients, 11.5% suffered from severe loss of smell after treatment; after 1 week, more than 82% recovered, but 18% reported persistent and complete loss of smell or taste [5]. A follow-up study with systematic analyses conducted in July 2020 on JAMA Otolaryngology Head Neck Surg reported that 12 out of 202 patients had unchanged or worsening loss of taste or smell 4 weeks after their infection [6]. Therefore, a decreased sense of taste or smell in patients with COVID-19 during the course of the disease or even after their recovery is a common complication.

As for the pathological mechanisms underlying the loss of taste or smell in COVID-19, a review conducted by Brazilian researchers summarized the potential interactions between the inflammatory reactions triggered by a viral infection and the olfactory system [7]. According to their analyses, SARS-CoV-2 may target horizontal basal cells, Bowmans’ glands, and nonneuronal supporting cells but not olfactory sensory neurons [7]. Given the long recovery time for the ability to taste or smell, transmission from olfactory sensory neurons to the central nervous system may be disrupted by infection. Another review summary of COVID-19 showed reduced smell and taste and speculated that the loss of the dendrite layer of the olfactory sensory neurons (OSN: neurons that sense the odorant information and transmit it to the central neural system) might directly cause temporary or permanent loss of smell (anosmia) in patients with COVID-19 [8]. As for the loss of taste (ageusia), the taste buds, which are the fundamental units for taste, have high expression levels of ACE2, which is the major target for SARS-CoV-2 infection recognition [8]. Therefore, it has been speculated that SARS-CoV-2 may act as a probable source of taste dysfunctions [9].

Approximately 10–20% of patients with COVID-19 suffer from permanent taste or smell loss as a major complication. Some studies have summarized and speculated the potential pathological mechanisms for taste or smell loss. However, analyses were performed at the cell level rather than at the gene level in most reviews. In the present study, according to the transcriptomic profiles of 577 patients with COVID-19, we attempted to identify the key pathogenic regulatory factors of taste or smell loss at the gene level by using multiple machine learning algorithms, including three feature selection methods: least absolute shrinkage and selection operator (LASSO) [10], Monte Carlo feature selection (MCFS) [11], and light gradient boosting machine (LightGBM) [12], and two classification algorithms: decision tree (DT) [13] and random forest (RF) [14]. The feature selection methods were used to deeply analyze the transcriptomic profiles, yielding three feature lists. Then, each list was fed into an incremental feature selection (IFS) [15] method to screen out the essential biomarker genes, construct efficient classifiers and classification rules. Some genes (e.g., H3-5, NUDT5, and AOC1) and rules can be confirmed to be associated with a decreased sense of taste and smell during SARS-CoV-2 infection. This study demonstrated a novel potential pathological mechanism for viral infection complications.

## 2. Materials and Methods

### 2.1. Data

Serum transcriptome data from 577 COVID-19-positive patients were obtained from the Gene Expression Omnibus (GEO) database under registration number GSE198449 [16,17]. The patients were divided into two groups according to whether the patients had impaired olfactory and gustatory function. A total of 84 patients showed a reduced sense of taste or smell. Each sample patient had 58,929 gene expression levels.

### 2.2. Feature Selection Methods

In recent years, several feature selection methods have been proposed to analyze the complicated dataset. Their purpose was to screen out essential features that played important roles in classifying samples. However, each method has advantages and disadvantages. Given a dataset, it is impossible to extract all essential features by using one single feature selection method. To mine essential features as completely as possible, the usage of multiple feature selection methods is a feasible scheme. Thus, we employed three feature selection methods: LASSO [10], MCFS [11], and LightGBM [12] to analyze the investigated transcriptome data mentioned in Section 2.1. These methods have different principles, which were helpful to overview the data from different points of view, thereby extracting more essential features hidden in different ways.

**LASSO.** Based on the Nonnegative Garrote proposed by Leobreiman [18], the LASSO algorithm was first proposed by Robert Tibshirani [10] in 1996 to obtain a statistical regression model and construct a first-order penalty function. Overfitting can be effectively reduced by regularizing the coefficients of some variables to zero, and features that contribute less to prediction are ignored. The absolute values of the coefficients are proportional to the importance of the features. The ranking of relevant features can be obtained based on such values. This study adopted the LASSO program in SCIKIT-LEARN [19], which was performed with default parameters.

**LightGBM.** The LightGBM [12] introduces one-sided gradient sampling, exclusive feature bundling, and histogram algorithms compared with the traditional gradient-boosting DT framework. The samples are segmented according to the outputs of an ensemble of classifiers, and the importance of each feature is estimated according to the number of times it is involved in the building of all DTs. LightGBM has a fast training speed and small memory footprint and is suitable for handling large-scale data while ensuring high accuracy. Features can be ranked in a list with the decreasing order of the above times. The LightGBM program sourced from https://lightgbm.readthedocs.io/en/latest/ accessed on 10 May 2020 was used in this study. It was also executed with default parameters.

**MCFS.** The MCFS was originally developed by Draminski et al. [11]. It selects some features randomly and repeatedly many times to form the *p* subsets of features. For each subset of features, t trees are constructed by randomly splitting the dataset into a training set and a test set t times. Therefore, *p* × *t* trees are constructed, and their performance is assessed. The importance of each feature is determined according to its involvement in these DT classifiers. A feature is considered important if it is involved in the splitting of tree nodes. This importance is defined as the relative importance (RI) score, which is calculated as follows:

(1) $$RI_{g}=\sum_{\tau =1}^{p\times t}{\left(\omega A_{CC}\right)}^{u}\sum_{ng\left(\tau \right)}IG\left(ng\left(\tau \right)\right){\left(\frac{no.in\;ng\left(\tau \right)}{no.in\;\tau }\right)}^{v}$$

In the formula, $\omega A_{CC}$ is the weighted accuracy of the tree τ, ng(τ) is a node in tree τ, its information gain is denoted as IG(ng(τ)), and no.in ng(τ)/no.in τ denotes the sample size of ng(τ)/τ; u and v are two positive reals weighting the $\omega A_{CC}$ and the ratio no.in ng(τ)/no.in τ, respectively. Accordingly, we ranked features in a list according to the decreasing order of their RI scores. Here, we used the MCFS program retrieved from http://www.ipipan.eu/staff/m.draminski/mcfs.html accessed on 4 June 2019. Likewise, it was also executed with default parameters.

Three feature selection methods were applied to the transcriptome data. Each can generate a feature list. For easy descriptions, these lists were called LASSO, LightGBM, and MCFS feature lists.

### 2.3. Incremental Feature Selection

Although the above three feature selection methods can sort features in lists according to their importance, it is still not adequate to determine the optimal feature subspace for classification. In the present study, IFS [15] was used to complete the task of extracting optimal subspace. From each feature list, a series of feature subsets were constructed by setting the step size at 5; that is, each feature subset has five more features than the previous subset. For each feature subset, samples were represented by features in this subset, on which a classifier was built with a given classification algorithm. All classifiers were evaluated through cross-validation [20]. By comparing the performance of all classifiers, the classifier with the best performance can be obtained, which is called the optimal classifier. The feature subset of such a classifier was termed the optimal feature subset.

### 2.4. Synthetic Minority Oversampling Technique

The number of samples in two classes in the dataset differed significantly, which may produce preferences for major classes if the classifier was directly based on such a dataset. To solve this problem, the synthetic minority oversampling technique (SMOTE) method [21] was employed in this study. It randomly selects a sample in the minority class, and Euclidean distances with other samples in the same class are calculated for the determination of k-nearest neighbors. A point lying on the line between the selected sample and one of its k-nearest neighbors is randomly selected as a new sample, which is put into the minority class. This process is repeated for the generation of new samples until a dataset balanced in number is obtained. We used the SMOTE package obtained from https://github.com/scikit-learn-contrib/imbalanced-learn accessed on 24 March 2020. Default parameters were used.

### 2.5. Classification Algorithm

To execute the IFS method, one classification algorithm is necessary. In this study, we attempted two classification algorithms: DT [13] and RF [14].

**Decision tree.** The DT algorithm constructs a tree-like structure in which each internal node holds a test on a feature, branches hold the conclusion of the test, and each leaf node holds a class label. A tree is grown according to selected features and conditions used in splitting based on information gain. During classification, starting from the root node, a sample is allocated to a sub-node or child node along the branch that satisfies the test of a node until it reaches a leaf node where a class label is assigned to the sample [13]. A DT can produce a group of classification rules that are easy to interpret and provide insights into biological mechanisms. Each rule represents a path from the root to one leaf. In this study, we used the CART algorithm with node ranking by the Gini coefficient. The program was taken from the SCIKIT-LEARN [19] package, and it was executed with default parameters.

**Random forest.** The RF algorithm is an ensemble learning based on DT algorithms [14,22,23,24,25,26] and creates a number of independent DT classifiers that do not interfere with one another. These classifiers were constructed by randomly taking samples from the training set and features. By combining the prediction results of all the DTs, the final classification decision is the class label that receives the most votes. As an integrated algorithm, RF tends to have higher accuracy than DT and can effectively prevent overfitting. The RF program in the SCIKIT-LEARN [19] package was used in this study and executed with default parameters.

### 2.6. Performance Evaluation

In the IFS method, lots of classifiers were set up. These classifiers were evaluated by 10-fold cross-validation. To evaluate the prediction quality, several measurements were adopted in this study. Generally, the F1-measure is a widely used measurement in binary classification [27,28,29,30]. The calculation procedure is as follows:

(2) $$\mathrm{Precision}=\frac{TP}{TP+FP}\;$$

(3) $$\mathrm{Recall}=\frac{TP}{TP+FN}\;$$

(4) $$F1-\mathrm{measure}=\frac{2\times \left(Recall\times Precision\right)}{\;Recall+Precision}$$

where TP is the true positive, FP is the false positive, and FN is the false negative. Classifier performance increases with F1-measure.

In addition, we further employed two other measurements: prediction accuracy (ACC) and Matthew correlation coefficient (MCC) [31]. ACC is defined as the proportion of correctly predicted samples, and MCC can be computed by

(5) $$\mathrm{MCC}=\frac{TP\times TN-FP\times FN}{\sqrt{\left(TP+FP\right)\times \left(TP+FN\right)\times \left(TN+FP\right)\times \left(TN+FN\right)}}$$

where *TN* represents the true negative.

## 3. Results

**Figure 1:**
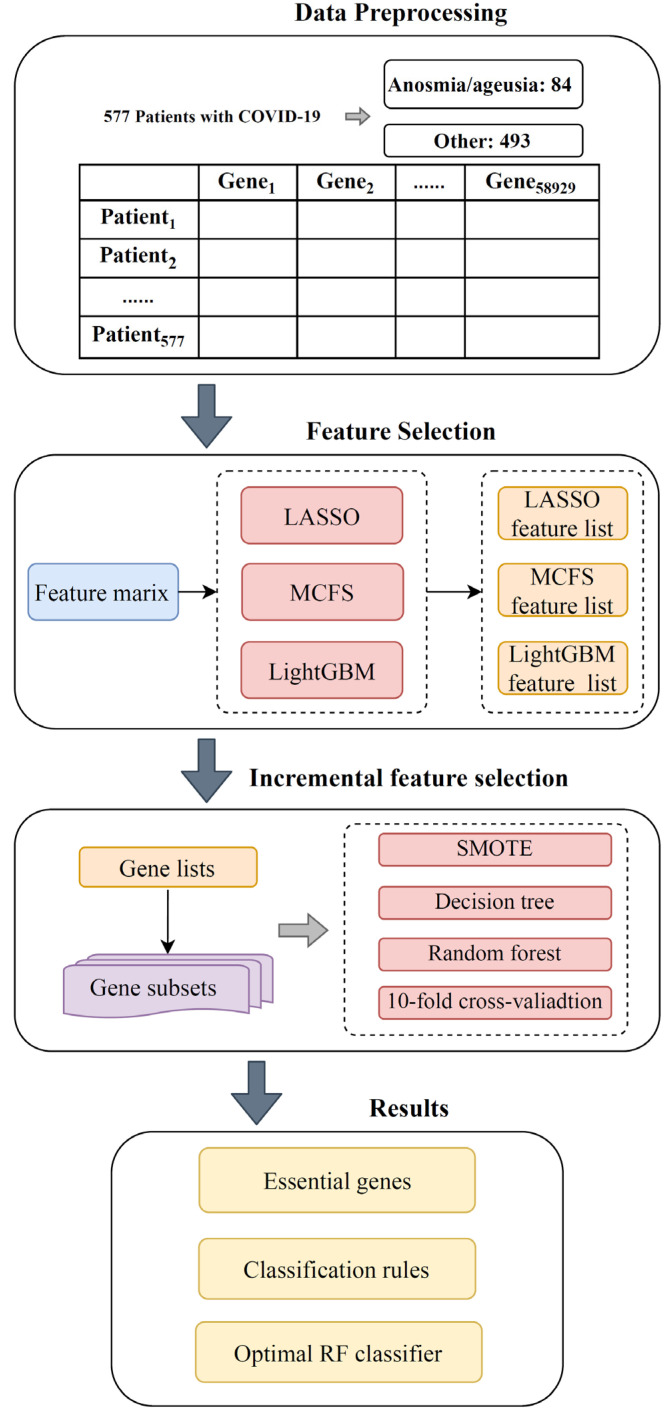
Flow chart of the entire computational analysis. A total of 58,929 gene features from 577 COVID-19 samples were ranked by feature importance using three feature selection methods: LASSO, LightGBM, and MCFS. The three feature lists were fed into the IFS computational framework, containing two classification algorithms. Finally, on the basis of the IFS results and the white-box algorithm DT, the optimal feature subset, classification rules, and optimal RF classifier were obtained.

The whole workflow of the computational analysis is shown in Figure 1. We screened and extracted the key features to distinguish the COVID-19 samples with olfactory and gustatory impairment from the other COVID-19 samples. The quantitative classification rules were obtained, and a high-performance RF classifier was built. The results of each stage are summarized in this section.

### 3.1. Feature Ranking Results

A total of 577 samples were used in this study, each of which was represented by 58,929 gene expression levels. The full sets of gene features were ranked using three feature selection methods, yielding three feature lists (the LASSO, LightGBM, and MCFS feature lists), which are shown in Table S1. Table 1 shows the top 10 gene features in the three feature lists. These were considered to be the most essential genes.

### 3.2. Results of Incremental Feature Selection

**Figure 2:**
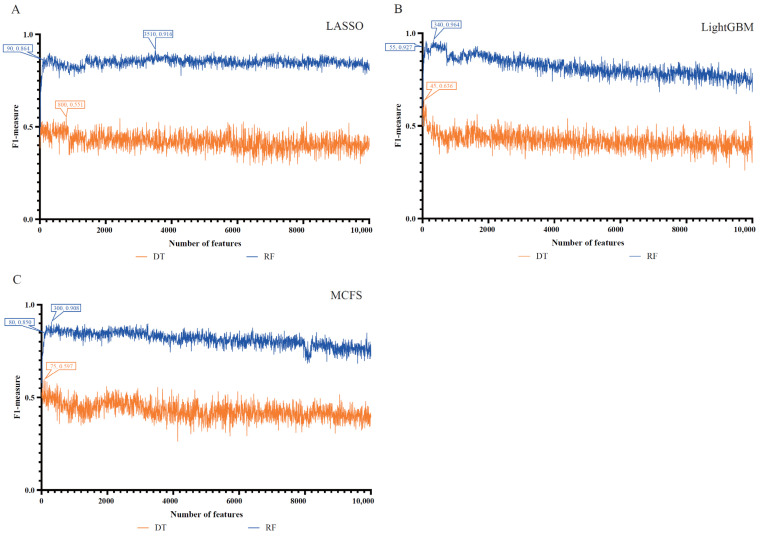
IFS curves of decision tree (DT) and random forest (RF) from different feature lists. (**A**) IFS curves based on the LASSO feature list. (**B**) IFS curves based on the LightGBM feature list. (**C**) IFS curves based on the MCFS feature list. RF classifiers always provide better performance than the DT classifiers.

According to the three lists, a number of feature subsets were constructed using the IFS method, and the step size was 5. In order to save time, we used the first 10,000 genes from each list to construct the feature subsets. After balancing the training set with the SMOTE, the RF and DT classifiers were built on each feature subset. The performance of all classifiers was evaluated through 10-fold cross-validation, and the F1-measure was selected as the major measurement. The detailed evaluation results are shown in Table S2. The IFS curves were plotted for visualization, as shown in Figure 2, where the number of features was used as the horizontal co-ordinate, and the F1-measure was used as the vertical co-ordinate.

For the LASSO feature list, the IFS curves of DT and RF are illustrated in Figure 2A. When the top 3510 features in this list were used, the RF yielded the highest F1-measure of 0.916. Accordingly, the optimal RF classifier can be built with these features. The ACC and MCC of such a classifier are listed in Table 2. For DT, its best performance was obtained by using the top 800 features in the list. Then, the optimal DT classifier was built using these features. The F1-measure of this classifier was 0.551. Clearly, such a classifier was far inferior to the optimal RF classifier.

For the LightGBM feature list, the two IFS curves are shown in Figure 2B. By using the same operation, the optimal RF and DT classifiers were built using the top 340 and 45 features in the list. They generated an F1-measure of 0.964 and 0.636, respectively. Evidently, the optimal RF classifier was much better than the optimal DT classifier. The detailed performance of the optimal RF classifier is listed in Table 2. Clearly, it yielded better performance when compared to the optimal RF classifier from the LASSO feature list.

For the MCFS feature list, the IFS curves of DT and RF are shown in Figure 2C. RF generated the highest F1-measure (0.908) when the top 300 features were adopted, whereas the highest F1-measure for DT was 0.597 when the top 75 features were used. Thus, the optimal DT and RF classifiers can be set up with corresponding optimal features. Again, the optimal RF classifier was far superior to the optimal DT classifier. The detailed performance of the optimal RF classifier is listed in Table 2. When comparing the performance of the optimal RF classifiers with the other two feature lists, the optimal classifier from the MCFS feature list was almost equal to that of the LASSO feature list and slightly weaker than that of the LightGBM feature list. Thus, the optimal RF classifiers from the LightGBM feature list can be a latent useful tool to identify COVID-19 samples with olfactory and gustatory impairment from other COVID-19 samples.

### 3.3. Intersection of Essential Features on Different Feature Lists

**Figure 3:**
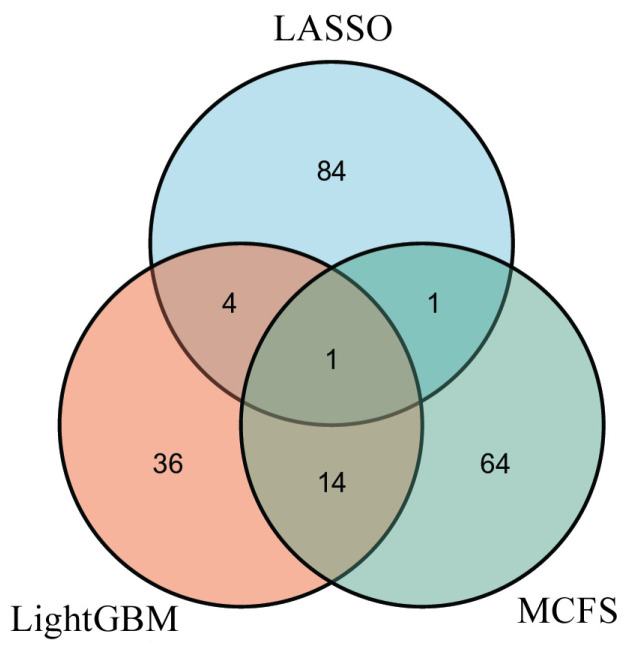
Venn diagram of the feature subsets used in the three feasible random forest classifiers from the three feature lists that were obtained by LASSO, LightGBM, and MCFS, respectively. The overlapping circles indicate the number of genes that were deemed to be essential by multiple feature selection methods.

In Section 3.2, three optimal RF classifiers were built based on different feature lists. However, the numbers of optimal features used in these classifiers were generally large. Thus, to improve the analytical process, the essential features among these optimal features should be extracted. By checking the IFS results with RF on each feature list, we can find out an RF classifier that used much fewer features and provided a slightly lower performance. From the LASSO feature list, such an RF classifier adopted the top 90 features, which yielded an F1-measure of 0.864. The RF classifiers on the other two feature lists adopted the top 55 (LightGBM feature list) and 80 (MCFS feature list) features. The detailed performance of the above three RF classifiers is listed in Table 2. Evidently, much fewer features were involved in these classifiers. However, their performance was a little lower than that of the optimal RF classifiers. These results indicated that these features were essential among the optimal features. The above RF classifiers were called feasible RF classifiers for convenience. Three feature subsets were constructed, which consisted of those features used by the three feasible RF classifiers. In order to show the relationship between these feature subsets, a Venn diagram was plotted, as shown in Figure 3. The detailed results of the intersection set are provided in Table S3. It can be observed that one gene feature was included in all three of the feature subsets, and 19 gene features belonged to two exact subsets, indicating that these genes were identified to be essential by multiple feature selection methods. Some genes can be confirmed to be associated with the impairment of gustatory and olfactory functions in patients with COVID-19, which is discussed in Section 4.

### 3.4. Classification Rules

**Figure 4:**
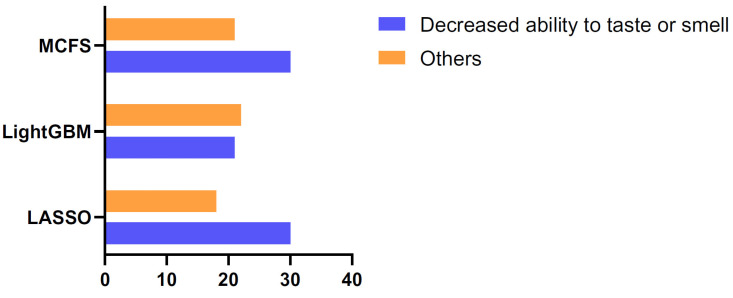
Bar chart showing the number of rules for the two classes of COVID-19 samples. Three optimal DT classifiers were constructed on the LASSO, LightGBM, and MCFS feature lists, and different numbers of classification rules were obtained by these classifiers. The number of rules for the COVID-19 samples with a reduced sense of taste or smell and other COVID-19 samples is plotted in the figure.

Although DT gave a lower performance than RF in the IFS method, it has a great advantage that RF does not own. As DT is a white-box algorithm, it can provide clues that are easy to interpret and analyze, thereby providing useful insights into understanding the essential expression differences between the COVID-19 samples with a reduced sense of taste or smell and those without impairment. According to the IFS results using DT on the three feature lists, the optimal DT classifiers adopted the top 800 features in the LASSO list, the top 45 features in the LightGBM list, and the top 75 features in the MCFS list. All samples were represented by these features, and three trees learned from these representations. Then, three rule groups were obtained, which are provided in Table S4. A total of 48, 43, and 51 classification rules were included in the three rule groups. The number of rules for the two classes in each group is shown in Figure 4. Each rule contains the tests of the expression levels of several genes (quantitatively), and the impairment of the olfactory and gustatory functions of the samples can be predicted according to the division of these rules. Some gene rules that contribute significantly to the prediction will be discussed in detail in Section 4.

## 4. Discussion

We identified the various biomarkers associated with the sequelae (loss of smell and taste) in COVID-19 infection. LASSO [10], MCFS [11], and LightGBM [12] enabled us not only to identify some of the potential biomarkers associated with COVID-19-induced taste or smell loss at the circulating transcriptomics level but also helped us to establish the quantitative rules for patient clustering on the basis of the optimal DT classifiers. A detailed discussion of some of the top gene features and quantitative rules is provided below.

### 4.1. Relationships of Top Features in Different Lists

**Figure 5:**
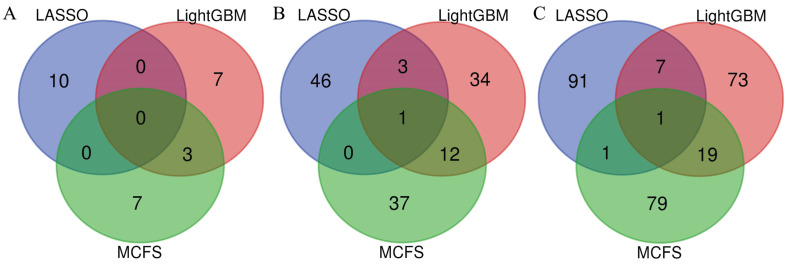
Venn diagram to show the relationship between the top features in the three feature lists. (**A**) Venn diagram for the top ten features in the three lists. (**B**) Venn diagram for the top 50 features in the three lists. (**C**) Venn diagram for the top 100 features in the three lists. The top features identified by different methods were quite different.

Three feature selection methods (LASSO, LightGBM, and MCFS) were used to analyze the expression data. As these methods have different principles, they can theoretically screen out different essential features. In order to confirm this, we selected the top 10, 50, and 100 features in the three lists and investigated their relationship. Three Venn diagrams were plotted for the top 10, 50, and 100 features, as shown in Figure 5. It can be observed that only a few features were deemed to be important by the multiple feature selection methods. There was only zero or one feature identified by all three methods when different top genes were selected in the three lists. The number of features identified by the two methods was also low. This result proved that LASSO, LightGBM, and MCFS could find the different essential gene features, thereby increasing the probability of extracting all the latent essential genes. Furthermore, the common features identified by LightGBM and MCFS were evidently more than those identified by LASSO and LightGBM or LASSO and MCFS. As LightGBM and MCFS are both DT-based methods, the features identified by them were more similar to those identified by LASSO.

### 4.2. Biomarkers Predicted by One or More Feature Selection Methods

**The biomarkers that were predicted by all three feature selection methods.** Only one biomarker (**ENSG00000234134.1**) was recognized by all three methods. This gene is a novel gene without a functional description in the protein products from the Ensemble dataset [32]. However, at the transcriptomics level, the gene is associated with an effective lncRNA and regulates the functional gene EDRF1, which is a specific gene for erythroid cell differentiation, thus contributing to alpha and gamma-globin regulation [33,34]. Erythroid membrane antigens are associated with Type II congenital smell loss [35]. In 2022, the down-regulation of the odor pathway has been reported to be associated with COVID-19-induced anosmia [36]. The dysfunction of non-neuron-related SARS-CoV-2 entry genes may help explain the smell or taste loss in patients with COVID-19 [37]. These publications indicate that the taste or smell loss in COVID-19 patients is related to in situ odor dysfunction, which the gene EDRF1 also participated in through the GATA-1-mediated pathway [38].

**The biomarkers that were predicted by the two feature selection methods.** Few biomarkers were predicted by the two machine learning models. **KRT38** (**ENSG00000171360.3**) was recognized by LightGBM and MCFS. The keratin gene family has tissue-specific patterns, especially during inflammation and particularly the regional inflammation caused by SARS-CoV-2 infection [39]. A recently published abstract described the association between alveolar regeneration and the Keratin gene family during COVID-19 pathogenic progression [40]. Early in 2021, researchers recognized that, as the main target of SARS-CoV-2, alveolar cells might transmit the effects of viral infection to nearly all cells and may eventually initiate the dysfunction of olfactory cells [41,42]. **H3-5** (**ENSG00000188375.5**) was predicted by LightGBM and LASSO. Histone modification and DNA methylation initiate the impairment of the sense of smell during SARS-CoV-2 infection [43]. Therefore, we speculated that H3-5 should be one of the key biomarkers for predicting smell or taste loss in patients with COVID-19. **NUDT5** (**ENSG00000165609.13**) was predicted by LightGBM and LASSO. It is reported to participate in the viral infection associated with signal transmission [44,45]. As for its specific role in association with COVID-19 compliments, the low expression level of the gene has been observed in severely infected cells [46]. The gene is involved in signal transmission in the respiratory system in COVID-19, indicating its potential olfactory regulatory effects [47]. These biomarkers, according to publications, may be associated with pathological decreases in smell or taste in COVID-19.

**The biomarkers that were predicted by only one feature selection method.** Some biomarkers were recognized by a single machine-learning model. These biomarkers may be associated with taste or smell loss during COVID-19 pathogenesis. The first predicted gene is **BTN2A3P** (ENSG00000124549.14, predicted by MCFS), which is reported to be associated with immune recognition and signal transduction [48]. Given that the loss of taste or smell is highly correlated with abnormal immune responses in the olfactory regulatory networks [49], the gene could participate in the regulation of olfactory impairment through these responses. **AOC1** (ENSG00000002726.20, predicted by LASSO) and another epidemiological factor, NOS2, are the two major factors reported to be associated with the neurological disorders causing taste and smell loss [50], validating the efficacy and accuracy of our prediction. **CDC42BPB** (ENSG00000259515.1, predicted by LightGBM) could be related to a viral infection that decreases the sense of smell or taste. CDC42BPB is a binding protein kinase of the functional protein CDC42, which interacts with Rac1 and PAK1 to regulate hormone olfactory neuroblasts [51] and plays a specific role in smelling capacity.

### 4.3. Quantitative Rules for COVID-19 Patient Clustering

By using three optimal DT classifiers, we established a series of quantitative rules that contributed to the distinction of patients with or without smell or taste dysfunction.

First, for the rule group in the LASSO feature list, the top rules involved the specific gene **BRCA1 (ENSG00000012048.22)**. According to the rule, the low expression of the gene may indicate a loss of smell or taste. BRCA1 is reported to be a specific proliferative regulator for taste bud cells in the taste epithelium [52]. Therefore, the gene is a quantitative parameter for COVID-19 complement prediction. Another rule-associated gene is **KATNIP (ENSG00000047578.13)**, which is said to be associated with microtubule function and lysosome delivery and has a low expression level in patients with a decreased sense of taste or smell [53]. Lysosome delivery is associated with decreased smell and taste capacities [54], and the low expression of KATNIP may contribute to patient clustering. Similarly, a low **IL23R (ENSG00000162594.15)** expression level can indicate patients without dysfunctions, corresponding to an enhanced inflammation-induced decrease in the sense of smell or taste in COVID-19 [7,55,56].

Moreover, we built another rule group for the MCFS feature list for the accurate clustering of patients with COVID-19. **CDC42EP2 (ENSG00000149798.5)**, which is reported to be functionally associated with CDC42, which is similar to CDC42BPB, is highly expressed in patients with taste or smell dysfunction but has low expression in patients without these complications. CDC42 may interact with Rac1 and PAK1 and thereby regulate hormone olfactory neuroblasts [51]. When highly expressed, it may inhibit the function of hormone olfactory neuroblasts. This feature validates our rules. **PBXIP1 (ENSG00000163346.17)** and **DHRS9 (ENSG00000073737.16)** are both reported to be quantitative biomarkers with high expression levels in patients with smell or taste dysfunction. Both genes are negatively associated with taste bud functioning [57,58].

The rules established for the LightGBM feature list are similar to those established for the MCFS feature list. The top prediction gene is **CDC42EP2 (ENSG00000149798.5)**. The expression of **ADNP2 (ENSG00000101544.9)** and **CLPP (ENSG00000125656.10)** might contribute to the prediction of patients with smell or taste dysfunctions. The associations between the two genes, the regulatory directions of the genes, and their olfactory functions have been validated [59,60]. Therefore, both rules can facilitate the identification of patients with a decreased sense of taste or smell.

The above biomarkers, especially those predicted by more than one algorithm, are functionally related to a decreased sense of taste or smell during viral infections, especially SARS-CoV-2 infection. By using multiple machine-learning algorithms, we were able to identify the potential regulatory factors as completely as possible. As for the quantitative rules built for the effective and accurate clustering of patients, the top rule-associated genes at the transcriptomics level were functionally associated with COVID-19 and taste or smell regulation, validating the efficacy and accuracy of our analysis.

### 4.4. Limitation of This Study

In this study, we designed a computational analysis to investigate the serum transcriptome data of COVID-19 samples associated with a reduced ability to taste or smell, trying to discover the latent biomarkers for such patients. Some discovered biomarkers were confirmed to have associations with a decreased sense of taste or smell during viral infections. However, some limitations exist in this study. First, the definition of smell or taste loss was based on the original questionnaire from previous papers [16,17], which has not been widely accepted. Second, the discussion on the discovered genes was only based on a literature review. This study did not provide solid evidence (through wet experiments) to link the identified genes with a decreased sense of taste or smell. Third, three feature selection methods were adopted to analyze the serum transcriptome data. They can investigate the data from different points of view. However, it was not clear whether these methods could cover all points of view. Adding other feature methods might produce additional biomarker genes.

## 5. Conclusions

We applied a set of advanced machine-learning methods to the analysis of serum transcriptomic data from patients with COVID-19 to reveal the genes associated with olfactory and gustatory impairments in COVID-19. First, we ranked all genes according to their importance by using three methods. The top genes in the ranked list might increase our understanding of the underlying mechanisms of olfactory and gustatory impairments. Subsequently, by using two classification algorithms, we identified the best-performing classifier that helped us screen patients with COVID-19 and olfactory and gustatory impairments. The quantitative rules obtained by using the DT classifiers facilitated the determination of the functional impairment profile.

## Figures and Tables

**Table 1:** Top 10 gene features in the three feature lists.

Rank	LASSO Feature List	LightGBM Feature List	MCFS Feature List
1	ENSG00000254624.1 (OR4R3P)	ENSG00000225611.1 (CCDC13-AS2)	ENSG00000171360.3 (KRT38)
2	ENSG00000223379.1 (Lnc-SPATA31A7-1)	ENSG00000259515.1 (Antisense to CDC42BPB)	ENSG00000149798.5 (CDC42EP2)
3	ENSG00000168528.12 (SERINC2)	ENSG00000104973.18 (MED25)	ENSG00000124549.14 (BTN2A3P)
4	ENSG00000278931.1 (ANKRD20A3 Pseudogene)	ENSG00000010361.13 (FUZ)	ENSG00000230526.1 (Lnc-CDH23-3)
5	ENSG00000203581.7 (OR1F2P)	ENSG00000163032.11 (VSNL1)	ENSG00000225611.1 (CCDC13-AS2)
6	ENSG00000234134.1 (EDRF1-AS1)	ENSG00000236496.2 (GPS2P1)	ENSG00000010361.13 (FUZ)
7	ENSG00000229596.3 (MYL12BP3)	ENSG00000149798.5 (CDC42EP2)	ENSG00000092054.13 (MYH7)
8	ENSG00000261707.1 (Lnc-ADAMTS18-3)	ENSG00000101544.9 (ADNP2)	ENSG00000131370.16 (SH3BP5)
9	ENSG00000229190.1 (Lnc-HACD1-2)	ENSG00000259674.1 (RPL7AP75)	ENSG00000158710.14 (TAGLN2)
10	ENSG00000183562.3 (Lnc-SLC22A18-2)	ENSG00000165406.16 (MARCHF8)	ENSG00000264204.2 (AGAP7P)

**Table 2:** Performance of the optimal and feasible random forest classifiers on the three feature lists.

Feature Lists	Number of Features	F1 Measure	MCC ^a^	ACC ^b^
LASSO feature list	90	0.864	0.841	0.960
	3510	0.916	0.902	0.976
LightGBM feature list	55	0.927	0.915	0.979
	340	0.964	0.958	0.990
MCFS feature list	80	0.850	0.825	0.957
	300	0.908	0.893	0.974

^a^: Mathew’s correlation coefficient; ^b^: Prediction accuracy.

## Data Availability

The data presented in this study are openly available in Gene Expression Omnibus at https://www.ncbi.nlm.nih.gov/geo/query/acc.cgi?acc=GSE198449 accessed on 21 April 2022, reference number [16].

## Supplementary Materials

The following supporting information can be downloaded at: https://www.mdpi.com/article/10.3390/life13030798/s1, Table S1: Feature lists obtained using LASSO, LightGBM, and MCFS. Table S2: IFS results with different classification algorithms on three feature lists. Table S3: Intersection of the feature subsets used in the feasible random forest classifiers on three feature lists. The features that appear in the 3, 2, and 1 feature subsets are shown. Table S4: Classification rules generated by the optimal DT classifiers on three feature lists.
