# Supplementary material for: Identification of Genes Associated with the Impairment of Olfactory and Gustatory Functions in COVID-19 via Machine-Learning Methods
Source: Life (Basel). 2023 Mar 15;13(3):798. doi: 10.3390/life13030798 (PMC10051382; doi:10.3390/life13030798)
Supplement: Supplementary file 1 [file life-13-00798-s001.zip › Table S1.pdf]

Supplementary Table S1: Feature-ranking results obtained using LASSO, LightGBM, and MCFS.

Rank	List yielded by LASSO	List yielded by LightGBM	List yielded by MCFS
1	ENSG00000254624.1	ENSG00000225611.1	ENSG00000171360.3
2	ENSG00000223379.1	ENSG00000259515.1	ENSG00000149798.5
3	ENSG00000168528.12	ENSG00000104973.18	ENSG00000124549.14
4	ENSG00000278931.1	ENSG00000010361.13	ENSG00000230526.1
5	ENSG00000203581.7	ENSG00000163032.11	ENSG00000225611.1
6	ENSG00000234134.1	ENSG00000236496.2	ENSG00000010361.13
7	ENSG00000229596.3	ENSG00000149798.5	ENSG00000092054.13
8	ENSG00000261707.1	ENSG00000101544.9	ENSG00000131370.16
9	ENSG00000229190.1	ENSG00000259674.1	ENSG00000158710.14
10	ENSG00000183562.3	ENSG00000165406.16	ENSG00000264204.2
11	ENSG00000188375.5	ENSG00000258926.1	ENSG00000130522.5
12	ENSG00000180104.16	ENSG00000234134.1	ENSG00000104973.18
13	ENSG00000199719.1	ENSG00000140941.13	ENSG00000261402.1
14	ENSG00000260135.6	ENSG00000260693.1	ENSG00000176009.3
15	ENSG00000250781.1	ENSG00000229007.1	ENSG00000012048.22
16	ENSG00000279315.1	ENSG00000260231.2	ENSG00000104904.12
17	ENSG00000240445.3	ENSG00000210191.1	ENSG00000185551.15
18	ENSG00000111305.19	ENSG00000196933.5	ENSG00000254990.5
19	ENSG00000269886.1	ENSG00000149260.17	ENSG00000224328.1
20	ENSG00000267708.1	ENSG00000022556.16	ENSG00000185177.12
21	ENSG00000162594.15	ENSG00000270157.1	ENSG00000234134.1
22	ENSG00000267643.1	ENSG00000267598.1	ENSG00000271412.1
23	ENSG00000189184.11	ENSG00000267226.2	ENSG00000175398.2
24	ENSG00000259895.1	ENSG00000211689.7	ENSG00000235975.1
25	ENSG00000122786.20	ENSG00000172425.10	ENSG00000198858.10
26	ENSG00000237310.1	ENSG00000163121.10	ENSG00000165406.16
27	ENSG00000273680.1	ENSG00000158710.14	ENSG00000214374.2
28	ENSG00000165609.13	ENSG00000125656.10	ENSG00000026950.17
29	ENSG00000260693.1	ENSG00000267984.1	ENSG00000173825.7
30	ENSG00000286220.1	ENSG00000244734.4	ENSG00000182809.10
31	ENSG00000167985.6	ENSG00000240137.5	ENSG00000277288.4
32	ENSG00000233834.6	ENSG00000239975.2	ENSG00000258763.5
33	ENSG00000272274.1	ENSG00000171360.3	ENSG00000286058.1
34	ENSG00000273262.1	ENSG00000164308.16	ENSG00000244411.3
35	ENSG00000248468.1	ENSG00000124839.13	ENSG00000073737.16
36	ENSG00000143297.19	ENSG00000105889.15	ENSG00000271071.1
37	ENSG00000101294.17	ENSG00000054179.12	ENSG00000227621.1
38	ENSG00000210191.1	ENSG00000250067.12	ENSG00000201988.2
39	ENSG00000170959.14	ENSG00000235052.1	ENSG00000101544.9
40	ENSG00000166450.13	ENSG00000226855.1	ENSG00000285000.1
41	ENSG00000105976.15	ENSG00000197111.15	ENSG00000240403.5
42	ENSG00000126266.3	ENSG00000165730.16	ENSG00000253204.1
43	ENSG00000228742.10	ENSG00000150045.12	ENSG00000099250.18
44	ENSG00000269982.1	ENSG00000137267.6	ENSG00000155307.18
45	ENSG00000278084.1	ENSG00000286122.1	ENSG00000149260.17
46	ENSG00000254911.3	ENSG00000267082.1	ENSG00000233456.1
47	ENSG00000258926.1	ENSG00000264204.2	ENSG00000258819.1
48	ENSG00000106341.11	ENSG00000240403.5	ENSG00000096088.16
49	ENSG00000255355.1	ENSG00000239203.1	ENSG00000241935.9
50	ENSG00000196972.9	ENSG00000230433.1	ENSG00000260231.2

51	ENSG00000178860.8	ENSG00000197182.14	ENSG00000133606.11
52	ENSG00000188037.11	ENSG00000188375.5	ENSG00000233242.2
53	ENSG00000279859.2	ENSG00000185551.15	ENSG00000235399.1
54	ENSG00000226650.5	ENSG00000184361.13	ENSG00000189430.12
55	ENSG00000247151.7	ENSG00000184292.6	ENSG00000165495.16
56	ENSG00000271787.1	ENSG00000182957.16	ENSG00000128283.7
57	ENSG00000048540.15	ENSG00000178752.16	ENSG00000180304.14
58	ENSG00000267023.5	ENSG00000174600.14	ENSG00000163032.11
59	ENSG00000253475.1	ENSG00000173825.7	ENSG00000257951.2
60	ENSG00000236204.6	ENSG00000165949.12	ENSG00000238243.3
61	ENSG00000267749.1	ENSG00000153157.13	ENSG00000173239.13
62	ENSG00000242580.1	ENSG00000141933.9	ENSG00000196998.18
63	ENSG00000285918.1	ENSG00000133816.15	ENSG00000196553.15
64	ENSG00000000003.14	ENSG00000124102.5	ENSG00000255356.2
65	ENSG00000000005.6	ENSG00000086548.9	ENSG00000259341.1
66	ENSG00000000419.12	ENSG00000066827.16	ENSG00000198963.11
67	ENSG00000000457.14	ENSG00000023171.18	ENSG00000283202.1
68	ENSG00000000460.17	ENSG00000285627.1	ENSG00000273776.1
69	ENSG00000000938.13	ENSG00000285448.1	ENSG00000253677.1
70	ENSG00000000971.15	ENSG00000272305.5	ENSG00000198901.14
71	ENSG00000001036.13	ENSG00000269886.1	ENSG00000163346.17
72	ENSG00000001084.12	ENSG00000261461.1	ENSG00000239590.1
73	ENSG00000001167.14	ENSG00000261253.2	ENSG00000250961.1
74	ENSG00000001460.18	ENSG00000260135.6	ENSG00000103381.12
75	ENSG00000001461.17	ENSG00000258819.1	ENSG00000254664.1
76	ENSG00000001497.16	ENSG00000255737.2	ENSG00000285685.1
77	ENSG00000001561.7	ENSG00000255508.7	ENSG00000229807.11
78	ENSG00000001617.12	ENSG00000249923.1	ENSG00000285064.1
79	ENSG00000001626.15	ENSG00000238243.3	ENSG00000165609.13
80	ENSG00000001629.10	ENSG00000237988.5	ENSG00000013306.16
81	ENSG00000001630.17	ENSG00000233695.2	ENSG00000165238.16
82	ENSG00000001631.15	ENSG00000231313.2	ENSG00000116337.16
83	ENSG00000002016.17	ENSG00000229368.1	ENSG00000255671.1
84	ENSG00000002079.14	ENSG00000229203.1	ENSG00000256508.2
85	ENSG00000002330.13	ENSG00000227398.3	ENSG00000234854.1
86	ENSG00000002549.12	ENSG00000223379.1	ENSG00000226752.9
87	ENSG00000002586.20	ENSG00000198858.10	ENSG00000237514.3
88	ENSG00000002586.20_PAR_Y	ENSG00000198626.16	ENSG00000253883.1
89	ENSG00000002587.10	ENSG00000196998.18	ENSG00000259398.1
90	ENSG00000002726.20	ENSG00000196517.11	ENSG00000158481.13
91	ENSG00000002745.13	ENSG00000182512.5	ENSG00000265319.2
92	ENSG00000002746.15	ENSG00000181826.10	ENSG00000251548.1
93	ENSG00000002822.15	ENSG00000179862.6	ENSG00000280083.1
94	ENSG00000002834.18	ENSG00000175746.6	ENSG00000285080.1
95	ENSG00000002919.14	ENSG00000172890.12	ENSG00000164694.17
96	ENSG00000002933.9	ENSG00000170509.12	ENSG00000185519.9
97	ENSG00000003056.8	ENSG00000169813.16	ENSG00000119950.21
98	ENSG00000003096.14	ENSG00000161939.19	ENSG00000241878.11
99	ENSG00000003137.8	ENSG00000157870.16	ENSG00000165801.10
100	ENSG00000003147.19	ENSG00000140853.15	ENSG00000226631.1
101	ENSG00000003249.13	ENSG00000133112.16	ENSG00000272772.1
102	ENSG00000003393.15	ENSG00000129595.13	ENSG00000231458.2
103	ENSG00000003400.14	ENSG00000127377.9	ENSG00000225988.1

104	ENSG00000003402.20	ENSG00000124557.12	ENSG00000182271.12
105	ENSG00000003436.16	ENSG00000124549.14	ENSG00000251584.1
106	ENSG00000003509.16	ENSG00000120645.11	ENSG00000139263.11
107	ENSG00000003756.16	ENSG00000116337.16	ENSG00000234325.1
108	ENSG00000003987.14	ENSG00000115155.17	ENSG00000278370.1
109	ENSG00000003989.17	ENSG00000107736.20	ENSG00000242779.7
110	ENSG00000004059.11	ENSG00000106245.11	ENSG00000214922.9
111	ENSG00000004139.14	ENSG00000105219.10	ENSG00000198690.9
112	ENSG00000004142.12	ENSG00000104522.16	ENSG00000156599.11
113	ENSG00000004399.12	ENSG00000092098.17	ENSG00000141401.12
114	ENSG00000004455.16	ENSG00000088387.19	ENSG00000095794.19
115	ENSG00000004468.13	ENSG00000285799.1	ENSG00000250482.3
116	ENSG00000004478.8	ENSG00000284600.1	ENSG00000255858.1
117	ENSG00000004487.16	ENSG00000280739.2	ENSG00000267441.1
118	ENSG00000004534.15	ENSG00000279148.1	ENSG00000250037.1
119	ENSG00000004660.14	ENSG00000278900.1	ENSG00000147050.14
120	ENSG00000004700.16	ENSG00000278376.1	ENSG00000204852.15
121	ENSG00000004766.17	ENSG00000277678.1	ENSG00000154760.14
122	ENSG00000004776.13	ENSG00000277200.1	ENSG00000163431.13
123	ENSG00000004777.18	ENSG00000275183.1	ENSG00000248510.2
124	ENSG00000004779.10	ENSG00000274845.1	ENSG00000161281.11
125	ENSG00000004799.8	ENSG00000274286.2	ENSG00000216368.2
126	ENSG00000004809.14	ENSG00000272489.1	ENSG00000196872.12
127	ENSG00000004838.14	ENSG00000272417.1	ENSG00000238244.3
128	ENSG00000004846.16	ENSG00000272264.1	ENSG00000284709.1
129	ENSG00000004848.8	ENSG00000271787.1	ENSG00000283380.1
130	ENSG00000004864.13	ENSG00000270882.2	ENSG00000166106.3
131	ENSG00000004866.20	ENSG00000270532.1	ENSG00000225155.4
132	ENSG00000004897.12	ENSG00000270147.1	ENSG00000232197.2
133	ENSG00000004939.15	ENSG00000269194.1	ENSG00000250640.2
134	ENSG00000004948.14	ENSG00000267475.1	ENSG00000237406.1
135	ENSG00000004961.15	ENSG00000267275.1	ENSG00000270462.1
136	ENSG00000004975.12	ENSG00000266821.1	ENSG00000130528.12
137	ENSG00000005001.10	ENSG00000265579.1	ENSG00000279939.1
138	ENSG00000005007.12	ENSG00000262823.1	ENSG00000185158.12
139	ENSG00000005020.13	ENSG00000260634.1	ENSG00000114737.15
140	ENSG00000005022.6	ENSG00000259804.1	ENSG00000206181.5
141	ENSG00000005059.16	ENSG00000259687.1	ENSG00000174678.9
142	ENSG00000005073.5	ENSG00000258499.1	ENSG00000121236.21
143	ENSG00000005075.15	ENSG00000258366.8	ENSG00000179695.2
144	ENSG00000005100.13	ENSG00000254602.1	ENSG00000054179.12
145	ENSG00000005102.13	ENSG00000253364.2	ENSG00000133816.15
146	ENSG00000005108.16	ENSG00000253204.1	ENSG00000091704.10
147	ENSG00000005156.11	ENSG00000250535.1	ENSG00000162366.8
148	ENSG00000005175.10	ENSG00000250159.6	ENSG00000227773.1
149	ENSG00000005187.12	ENSG00000249240.2	ENSG00000126733.21
150	ENSG00000005189.19	ENSG00000246889.2	ENSG00000269107.1
151	ENSG00000005194.15	ENSG00000245694.9	ENSG00000233730.1
152	ENSG00000005206.17	ENSG00000243896.4	ENSG00000235315.2
153	ENSG00000005238.19	ENSG00000243004.5	ENSG00000117154.12
154	ENSG00000005243.10	ENSG00000242611.2	ENSG00000256827.1
155	ENSG00000005249.13	ENSG00000241889.1	ENSG00000121904.17
156	ENSG00000005302.18	ENSG00000241015.2	ENSG00000235812.4

157	ENSG00000005339.14	ENSG00000239521.8	ENSG00000074211.14
158	ENSG00000005379.17	ENSG00000235554.1	ENSG00000261866.2
159	ENSG00000005381.7	ENSG00000235453.10	ENSG00000100024.14
160	ENSG00000005421.8	ENSG00000234711.1	ENSG00000285540.1
161	ENSG00000005436.13	ENSG00000234607.2	ENSG00000228496.1
162	ENSG00000005448.16	ENSG00000234432.4	ENSG00000065609.14
163	ENSG00000005469.11	ENSG00000233077.1	ENSG00000134762.17
164	ENSG00000005471.17	ENSG00000233072.1	ENSG00000262662.1
165	ENSG00000005483.20	ENSG00000231426.6	ENSG00000147119.3
166	ENSG00000005486.17	ENSG00000231249.1	ENSG00000176533.13
167	ENSG00000005513.10	ENSG00000231006.1	ENSG00000187589.8
168	ENSG00000005700.15	ENSG00000229473.2	ENSG00000102384.13
169	ENSG00000005801.18	ENSG00000228315.12	ENSG00000226386.1
170	ENSG00000005810.18	ENSG00000225930.4	ENSG00000228372.7
171	ENSG00000005812.11	ENSG00000225778.5	ENSG00000100218.11
172	ENSG00000005844.17	ENSG00000224950.2	ENSG00000267610.2
173	ENSG00000005882.11	ENSG00000223804.5	ENSG00000272937.2
174	ENSG00000005884.18	ENSG00000219392.1	ENSG00000227474.1
175	ENSG00000005889.15	ENSG00000213988.11	ENSG00000217874.2
176	ENSG00000005893.15	ENSG00000213492.2	ENSG00000241884.2
177	ENSG00000005961.18	ENSG00000211779.3	ENSG00000145293.16
178	ENSG00000005981.13	ENSG00000205913.6	ENSG00000277313.1
179	ENSG00000006007.12	ENSG00000204673.10	ENSG00000229116.2
180	ENSG00000006015.17	ENSG00000204516.10	ENSG00000243687.1
181	ENSG00000006016.11	ENSG00000204116.11	ENSG00000270098.1
182	ENSG00000006025.12	ENSG00000198353.8	ENSG00000242321.1
183	ENSG00000006042.12	ENSG00000197888.2	ENSG00000283599.2
184	ENSG00000006047.13	ENSG00000197847.12	ENSG00000181433.9
185	ENSG00000006059.3	ENSG00000196912.12	ENSG00000285614.1
186	ENSG00000006062.16	ENSG00000196169.15	ENSG00000273777.4
187	ENSG00000006071.13	ENSG00000189129.13	ENSG00000274835.1
188	ENSG00000006116.3	ENSG00000185198.11	ENSG00000250327.1
189	ENSG00000006118.14	ENSG00000182687.4	ENSG00000160445.11
190	ENSG00000006125.18	ENSG00000182158.15	ENSG00000254205.1
191	ENSG00000006128.12	ENSG00000181029.9	ENSG00000163798.13
192	ENSG00000006194.10	ENSG00000180767.9	ENSG00000225083.1
193	ENSG00000006210.7	ENSG00000180316.12	ENSG00000280650.1
194	ENSG00000006282.21	ENSG00000180304.14	ENSG00000278090.2
195	ENSG00000006327.13	ENSG00000180071.20	ENSG00000260616.6
196	ENSG00000006377.11	ENSG00000178814.17	ENSG00000149124.11
197	ENSG00000006432.15	ENSG00000177138.16	ENSG00000228536.2
198	ENSG00000006451.8	ENSG00000175800.5	ENSG00000169676.5
199	ENSG00000006453.14	ENSG00000173890.17	ENSG00000124835.2
200	ENSG00000006459.11	ENSG00000171552.13	ENSG00000278900.1
201	ENSG00000006468.14	ENSG00000170776.21	ENSG00000256465.1
202	ENSG00000006530.17	ENSG00000169871.13	ENSG00000245526.10
203	ENSG00000006534.16	ENSG00000168228.15	ENSG00000272024.1
204	ENSG00000006555.11	ENSG00000168152.13	ENSG00000173218.15
205	ENSG00000006576.16	ENSG00000168079.17	ENSG00000183347.14
206	ENSG00000006606.8	ENSG00000168062.10	ENSG00000277024.1
207	ENSG00000006607.14	ENSG00000167208.15	ENSG00000236252.2
208	ENSG00000006611.16	ENSG00000167207.13	ENSG00000088992.18
209	ENSG00000006625.18	ENSG00000166527.8	ENSG00000156313.13

210	ENSG00000006634.8	ENSG00000166188.2	ENSG00000184908.17
211	ENSG00000006638.11	ENSG00000165609.13	ENSG00000127124.16
212	ENSG00000006652.14	ENSG00000164970.15	ENSG00000272963.1
213	ENSG00000006659.13	ENSG00000164930.12	ENSG00000213287.3
214	ENSG00000006695.11	ENSG00000164691.17	ENSG00000198108.4
215	ENSG00000006712.15	ENSG00000163904.13	ENSG00000130950.13
216	ENSG00000006715.16	ENSG00000163346.17	ENSG00000143786.8
217	ENSG00000006740.17	ENSG00000163171.7	ENSG00000274584.1
218	ENSG00000006744.19	ENSG00000162804.14	ENSG00000130234.10
219	ENSG00000006747.15	ENSG00000162366.8	ENSG00000239690.3
220	ENSG00000006756.16	ENSG00000160194.18	ENSG00000185074.7
221	ENSG00000006757.12	ENSG00000154760.14	ENSG00000115504.14
222	ENSG00000006788.14	ENSG00000154330.13	ENSG00000229207.1
223	ENSG00000006831.10	ENSG00000153944.11	ENSG00000270882.2
224	ENSG00000006837.12	ENSG00000152154.11	ENSG00000116014.10
225	ENSG00000007001.12	ENSG00000151465.14	ENSG00000254571.1
226	ENSG00000007038.11	ENSG00000151445.16	ENSG00000244752.3
227	ENSG00000007047.15	ENSG00000149591.16	ENSG00000280172.1
228	ENSG00000007062.11	ENSG00000148488.17	ENSG00000196110.7
229	ENSG00000007080.11	ENSG00000143862.8	ENSG00000254641.1
230	ENSG00000007129.18	ENSG00000143476.18	ENSG00000226649.1
231	ENSG00000007168.13	ENSG00000143321.19	ENSG00000172530.20
232	ENSG00000007171.17	ENSG00000143185.4	ENSG00000267239.1
233	ENSG00000007174.18	ENSG00000143164.15	ENSG00000283928.1
234	ENSG00000007202.15	ENSG00000139970.17	ENSG00000215223.3
235	ENSG00000007216.14	ENSG00000138629.16	ENSG00000262304.2
236	ENSG00000007237.18	ENSG00000135926.15	ENSG00000073969.18
237	ENSG00000007255.10	ENSG00000134574.11	ENSG00000218586.3
238	ENSG00000007264.15	ENSG00000133169.6	ENSG00000258707.2
239	ENSG00000007306.15	ENSG00000132801.7	ENSG00000068024.16
240	ENSG00000007312.12	ENSG00000130830.15	ENSG00000273591.1
241	ENSG00000007314.12	ENSG00000129691.15	ENSG00000206936.1
242	ENSG00000007341.19	ENSG00000128254.13	ENSG00000225637.1
243	ENSG00000007350.17	ENSG00000126010.5	ENSG00000197852.11
244	ENSG00000007372.22	ENSG00000125818.18	ENSG00000238094.1
245	ENSG00000007376.8	ENSG00000118307.19	ENSG00000168077.14
246	ENSG00000007384.15	ENSG00000117266.15	ENSG00000167612.13
247	ENSG00000007392.16	ENSG00000115604.10	ENSG00000260928.2
248	ENSG00000007402.11	ENSG00000114650.20	ENSG00000227531.1
249	ENSG00000007516.13	ENSG00000111012.10	ENSG00000150045.12
250	ENSG00000007520.4	ENSG00000109046.15	ENSG00000280013.1
251	ENSG00000007541.16	ENSG00000108523.15	ENSG00000197111.15
252	ENSG00000007545.15	ENSG00000108219.15	ENSG00000254607.2
253	ENSG00000007866.21	ENSG00000107957.16	ENSG00000112333.11
254	ENSG00000007908.16	ENSG00000104728.16	ENSG00000270474.2
255	ENSG00000007923.16	ENSG00000104490.18	ENSG00000196652.11
256	ENSG00000007933.13	ENSG00000103381.12	ENSG00000178776.5
257	ENSG00000007944.15	ENSG00000101940.18	ENSG00000169398.19
258	ENSG00000007952.18	ENSG00000100784.12	ENSG00000066135.13
259	ENSG00000007968.7	ENSG00000100764.14	ENSG00000179820.16
260	ENSG00000008018.9	ENSG00000100605.17	ENSG00000239203.1
261	ENSG00000008056.14	ENSG00000100281.14	ENSG00000069812.11
262	ENSG00000008083.14	ENSG00000085832.17	ENSG00000183389.5

263	ENSG00000008086.11	ENSG00000073111.14	ENSG00000163947.11
264	ENSG00000008118.10	ENSG00000070614.15	ENSG00000170965.10
265	ENSG00000008128.23	ENSG00000070526.15	ENSG00000054690.13
266	ENSG00000008130.15	ENSG00000068971.14	ENSG00000282413.1
267	ENSG00000008196.13	ENSG00000068912.14	ENSG00000278973.1
268	ENSG00000008197.4	ENSG00000064419.13	ENSG00000264958.2
269	ENSG00000008226.19	ENSG00000064115.11	ENSG00000253276.3
270	ENSG00000008256.16	ENSG00000063438.17	ENSG00000164970.15
271	ENSG00000008277.14	ENSG00000057593.13	ENSG00000236180.2
272	ENSG00000008282.8	ENSG00000052344.16	ENSG00000143994.14
273	ENSG00000008283.16	ENSG00000048471.14	ENSG00000277901.1
274	ENSG00000008294.21	ENSG00000028277.21	ENSG00000283355.1
275	ENSG00000008300.17	ENSG00000022840.16	ENSG00000282041.1
276	ENSG00000008311.15	ENSG00000009844.16	ENSG00000226869.6
277	ENSG00000008323.15	ENSG00000007372.22	ENSG00000220515.2
278	ENSG00000008324.11	ENSG00000000003.14	ENSG00000236887.1
279	ENSG00000008382.15	ENSG00000286220.1	ENSG00000230562.3
280	ENSG00000008394.13	ENSG00000286075.1	ENSG00000223584.1
281	ENSG00000008405.12	ENSG00000286009.1	ENSG00000285117.1
282	ENSG00000008438.5	ENSG00000285994.1	ENSG00000177464.5
283	ENSG00000008441.16	ENSG00000285918.1	ENSG00000206977.1
284	ENSG00000008513.16	ENSG00000285906.1	ENSG00000248773.1
285	ENSG00000008516.17	ENSG00000285737.1	ENSG00000241809.1
286	ENSG00000008517.16	ENSG00000285696.1	ENSG00000249650.1
287	ENSG00000008710.19	ENSG00000285647.1	ENSG00000087903.13
288	ENSG00000008735.14	ENSG00000285641.1	ENSG00000257557.2
289	ENSG00000008838.20	ENSG00000285106.1	ENSG00000233926.1
290	ENSG00000008853.16	ENSG00000285006.1	ENSG00000269194.1
291	ENSG00000008869.12	ENSG00000284987.1	ENSG00000168528.12
292	ENSG00000008952.17	ENSG00000284931.1	ENSG00000130055.13
293	ENSG00000008988.9	ENSG00000284826.1	ENSG00000279778.1
294	ENSG00000009307.16	ENSG00000284697.1	ENSG00000141255.12
295	ENSG00000009335.18	ENSG00000284693.1	ENSG00000239828.6
296	ENSG00000009413.15	ENSG00000284633.1	ENSG00000258583.6
297	ENSG00000009694.13	ENSG00000284543.1	ENSG00000239912.1
298	ENSG00000009709.12	ENSG00000284294.1	ENSG00000146809.13
299	ENSG00000009724.16	ENSG00000283907.1	ENSG00000235480.1
300	ENSG00000009765.14	ENSG00000283234.1	ENSG00000241353.3
301	ENSG00000009780.15	ENSG00000283064.1	ENSG00000259515.1
302	ENSG00000009790.15	ENSG00000281849.3	ENSG00000243328.1
303	ENSG00000009830.11	ENSG00000281469.1	ENSG00000225005.1
304	ENSG00000009844.16	ENSG00000281344.1	ENSG00000253528.2
305	ENSG00000009950.16	ENSG00000281103.1	ENSG00000187045.18
306	ENSG00000009954.11	ENSG00000280789.2	ENSG00000224789.1
307	ENSG00000010017.13	ENSG00000280721.1	ENSG00000224523.1
308	ENSG00000010030.14	ENSG00000280417.1	ENSG00000252656.1
309	ENSG00000010072.16	ENSG00000280392.1	ENSG00000265334.1
310	ENSG00000010165.20	ENSG00000280374.1	ENSG00000254673.1
311	ENSG00000010219.13	ENSG00000280161.1	ENSG00000226247.1
312	ENSG00000010244.18	ENSG00000280160.1	ENSG00000214860.5
313	ENSG00000010256.11	ENSG00000279880.1	ENSG00000237222.3
314	ENSG00000010270.13	ENSG00000279863.1	ENSG00000221507.1
315	ENSG00000010278.14	ENSG00000279838.1	ENSG00000280250.1

316	ENSG00000010282.14	ENSG00000279812.1	ENSG00000254814.1
317	ENSG00000010292.13	ENSG00000279805.1	ENSG00000250682.5
318	ENSG00000010295.19	ENSG00000279766.1	ENSG00000257271.1
319	ENSG00000010310.9	ENSG00000279753.1	ENSG00000185565.12
320	ENSG00000010318.21	ENSG00000279667.1	ENSG00000250808.1
321	ENSG00000010319.6	ENSG00000279557.1	ENSG00000213667.3
322	ENSG00000010322.16	ENSG00000279453.1	ENSG00000236045.1
323	ENSG00000010327.10	ENSG00000279444.1	ENSG00000135519.8
324	ENSG00000010361.13	ENSG00000279386.1	ENSG00000164849.10
325	ENSG00000010379.16	ENSG00000279357.1	ENSG00000197044.11
326	ENSG00000010404.18	ENSG00000279315.1	ENSG00000231625.4
327	ENSG00000010438.16	ENSG00000279058.2	ENSG00000231542.1
328	ENSG00000010539.11	ENSG00000278983.1	ENSG00000131480.9
329	ENSG00000010610.10	ENSG00000278962.1	ENSG00000109046.15
330	ENSG00000010626.15	ENSG00000278942.1	ENSG00000266780.1
331	ENSG00000010671.15	ENSG00000278931.1	ENSG00000224256.1
332	ENSG00000010704.18	ENSG00000278784.1	ENSG00000285597.1
333	ENSG00000010803.16	ENSG00000278601.1	ENSG00000225196.2
334	ENSG00000010810.17	ENSG00000278576.1	ENSG00000241828.2
335	ENSG00000010818.10	ENSG00000278480.1	ENSG00000136231.14
336	ENSG00000010932.17	ENSG00000278431.1	ENSG00000216364.1
337	ENSG00000011007.12	ENSG00000278299.5	ENSG00000223511.7
338	ENSG00000011009.11	ENSG00000278144.1	ENSG00000255513.1
339	ENSG00000011021.23	ENSG00000278090.2	ENSG00000222352.1
340	ENSG00000011028.14	ENSG00000277925.1	ENSG00000256975.1
341	ENSG00000011052.21	ENSG00000277825.1	ENSG00000275293.1
342	ENSG00000011083.9	ENSG00000277767.1	ENSG00000251739.1
343	ENSG00000011105.14	ENSG00000277715.1	ENSG00000187080.9
344	ENSG00000011114.15	ENSG00000277402.1	ENSG00000224101.1
345	ENSG00000011132.12	ENSG00000277324.1	ENSG00000225235.1
346	ENSG00000011143.16	ENSG00000277310.1	ENSG00000275553.3
347	ENSG00000011198.9	ENSG00000277290.1	ENSG00000267337.1
348	ENSG00000011201.12	ENSG00000277287.1	ENSG00000090013.10
349	ENSG00000011243.18	ENSG00000277224.2	ENSG00000233276.5
350	ENSG00000011258.16	ENSG00000277147.6	ENSG00000230360.1
351	ENSG00000011260.14	ENSG00000277034.1	ENSG00000271642.1
352	ENSG00000011275.18	ENSG00000276566.1	ENSG00000259232.2
353	ENSG00000011295.16	ENSG00000276136.1	ENSG00000200408.1
354	ENSG00000011304.20	ENSG00000276014.1	ENSG00000267984.1
355	ENSG00000011332.19	ENSG00000275720.1	ENSG00000230829.3
356	ENSG00000011347.9	ENSG00000275676.1	ENSG00000231752.6
357	ENSG00000011376.11	ENSG00000275493.2	ENSG00000243483.1
358	ENSG00000011405.13	ENSG00000275441.1	ENSG00000260135.6
359	ENSG00000011422.12	ENSG00000275329.1	ENSG00000134539.17
360	ENSG00000011426.11	ENSG00000275063.1	ENSG00000156103.16
361	ENSG00000011451.19	ENSG00000275034.2	ENSG00000139350.11
362	ENSG00000011454.17	ENSG00000274987.1	ENSG00000231901.1
363	ENSG00000011465.17	ENSG00000274898.1	ENSG00000168148.3
364	ENSG00000011478.12	ENSG00000274810.4	ENSG00000141756.18
365	ENSG00000011485.14	ENSG00000274667.1	ENSG00000237730.1
366	ENSG00000011523.14	ENSG00000274471.2	ENSG00000216867.2
367	ENSG00000011566.15	ENSG00000274270.1	ENSG00000198153.8
368	ENSG00000011590.14	ENSG00000274114.2	ENSG00000266433.6

369	ENSG00000011600.11	ENSG00000274020.3	ENSG00000170613.4
370	ENSG00000011638.10	ENSG00000273514.1	ENSG00000227078.1
371	ENSG00000011677.13	ENSG00000273254.1	ENSG00000272479.1
372	ENSG00000012048.22	ENSG00000273245.1	ENSG00000022556.16
373	ENSG00000012061.15	ENSG00000273243.1	ENSG00000047621.12
374	ENSG00000012124.17	ENSG00000273189.1	ENSG00000101890.4
375	ENSG00000012171.19	ENSG00000273149.1	ENSG00000284385.1
376	ENSG00000012174.12	ENSG00000273133.1	ENSG00000223611.5
377	ENSG00000012211.13	ENSG00000273106.1	ENSG00000181260.8
378	ENSG00000012223.12	ENSG00000273056.1	ENSG00000169071.15
379	ENSG00000012232.8	ENSG00000273008.1	ENSG00000131482.9
380	ENSG00000012504.15	ENSG00000272986.1	ENSG00000227581.2
381	ENSG00000012660.14	ENSG00000272980.4	ENSG00000265702.1
382	ENSG00000012779.11	ENSG00000272887.1	ENSG00000072080.11
383	ENSG00000012817.15	ENSG00000272733.1	ENSG00000070269.14
384	ENSG00000012822.16	ENSG00000272716.1	ENSG00000284252.1
385	ENSG00000012963.15	ENSG00000272668.2	ENSG00000239201.1
386	ENSG00000012983.11	ENSG00000272620.1	ENSG00000140941.13
387	ENSG00000013016.16	ENSG00000272384.1	ENSG00000242137.1
388	ENSG00000013275.7	ENSG00000272360.1	ENSG00000130675.15
389	ENSG00000013288.8	ENSG00000272282.1	ENSG00000238193.1
390	ENSG00000013293.6	ENSG00000272269.1	ENSG00000255314.3
391	ENSG00000013297.11	ENSG00000272205.1	ENSG00000198892.6
392	ENSG00000013306.16	ENSG00000272181.1	ENSG00000086159.13
393	ENSG00000013364.19	ENSG00000272148.1	ENSG00000276348.1
394	ENSG00000013374.16	ENSG00000272037.1	ENSG00000280620.1
395	ENSG00000013375.16	ENSG00000271936.1	ENSG00000227682.2
396	ENSG00000013392.8	ENSG00000271870.1	ENSG00000274641.1
397	ENSG00000013441.16	ENSG00000271851.1	ENSG00000224155.2
398	ENSG00000013503.10	ENSG00000271789.1	ENSG00000131471.7
399	ENSG00000013523.9	ENSG00000271780.1	ENSG00000252558.1
400	ENSG00000013561.18	ENSG00000271736.1	ENSG00000226650.5
401	ENSG00000013563.14	ENSG00000271643.2	ENSG00000242307.1
402	ENSG00000013573.17	ENSG00000271580.1	ENSG00000183791.4
403	ENSG00000013583.10	ENSG00000271567.1	ENSG00000152822.14
404	ENSG00000013588.8	ENSG00000270883.1	ENSG00000232624.7
405	ENSG00000013619.14	ENSG00000270720.1	ENSG00000115649.16
406	ENSG00000013725.14	ENSG00000270638.1	ENSG00000253448.1
407	ENSG00000013810.19	ENSG00000270339.3	ENSG00000199719.1
408	ENSG00000014123.10	ENSG00000270082.1	ENSG00000107262.22
409	ENSG00000014138.9	ENSG00000270020.1	ENSG00000258033.1
410	ENSG00000014164.7	ENSG00000269985.1	ENSG00000264174.1
411	ENSG00000014216.15	ENSG00000269946.2	ENSG00000237447.1
412	ENSG00000014257.16	ENSG00000269910.1	ENSG00000249988.1
413	ENSG00000014641.18	ENSG00000269904.2	ENSG00000284615.1
414	ENSG00000014824.14	ENSG00000269891.2	ENSG00000140264.19
415	ENSG00000014914.21	ENSG00000269858.6	ENSG00000261714.2
416	ENSG00000014919.12	ENSG00000269681.1	ENSG00000255669.2
417	ENSG00000015133.19	ENSG00000268883.2	ENSG00000215174.2
418	ENSG00000015153.14	ENSG00000268738.3	ENSG00000264058.2
419	ENSG00000015171.19	ENSG00000268203.1	ENSG00000104413.17
420	ENSG00000015285.10	ENSG00000268047.1	ENSG00000219776.2
421	ENSG00000015413.9	ENSG00000267868.1	ENSG00000278745.1

422	ENSG00000015475.18	ENSG00000267767.2	ENSG00000125492.10
423	ENSG00000015479.18	ENSG00000267751.5	ENSG00000236496.2
424	ENSG00000015520.14	ENSG00000267733.5	ENSG00000266846.1
425	ENSG00000015532.9	ENSG00000267719.1	ENSG00000280243.1
426	ENSG00000015568.13	ENSG00000267666.2	ENSG00000140379.8
427	ENSG00000015592.16	ENSG00000267650.1	ENSG00000125675.18
428	ENSG00000015676.18	ENSG00000267640.6	ENSG00000272602.6
429	ENSG00000016082.15	ENSG00000267551.3	ENSG00000154451.14
430	ENSG00000016391.11	ENSG00000267519.6	ENSG00000224690.2
431	ENSG00000016402.13	ENSG00000267453.7	ENSG00000235546.2
432	ENSG00000016490.15	ENSG00000267423.1	ENSG00000231148.1
433	ENSG00000016602.9	ENSG00000267390.1	ENSG00000100084.14
434	ENSG00000016864.18	ENSG00000267359.1	ENSG00000240625.3
435	ENSG00000017260.19	ENSG00000267344.1	ENSG00000143190.23
436	ENSG00000017427.16	ENSG00000267336.1	ENSG00000255627.1
437	ENSG00000017483.15	ENSG00000267125.2	ENSG00000251468.2
438	ENSG00000017797.13	ENSG00000267084.1	ENSG00000242550.6
439	ENSG00000018189.13	ENSG00000267009.6	ENSG00000275994.1
440	ENSG00000018236.15	ENSG00000266933.2	ENSG00000157514.16
441	ENSG00000018280.17	ENSG00000266709.1	ENSG00000230444.1
442	ENSG00000018408.14	ENSG00000266708.1	ENSG00000185774.16
443	ENSG00000018510.15	ENSG00000266604.1	ENSG00000276418.5
444	ENSG00000018607.6	ENSG00000266075.2	ENSG00000274583.1
445	ENSG00000018610.14	ENSG00000265939.1	ENSG00000251260.2
446	ENSG00000018625.14	ENSG00000265298.1	ENSG00000236303.3
447	ENSG00000018699.13	ENSG00000265018.6	ENSG00000271488.1
448	ENSG00000018869.16	ENSG00000264343.6	ENSG00000183607.10
449	ENSG00000019102.12	ENSG00000264207.1	ENSG00000223905.1
450	ENSG00000019144.19	ENSG00000263753.7	ENSG00000254781.1
451	ENSG00000019169.10	ENSG00000263731.1	ENSG00000239149.1
452	ENSG00000019186.10	ENSG00000263718.2	ENSG00000259459.5
453	ENSG00000019485.13	ENSG00000263590.2	ENSG00000134070.5
454	ENSG00000019505.8	ENSG00000263321.1	ENSG00000130830.15
455	ENSG00000019549.12	ENSG00000263020.6	ENSG00000179073.6
456	ENSG00000019582.15	ENSG00000262873.1	ENSG00000228668.1
457	ENSG00000019991.17	ENSG00000262721.1	ENSG00000213726.5
458	ENSG00000019995.6	ENSG00000262500.1	ENSG00000147650.11
459	ENSG00000020129.16	ENSG00000262319.1	ENSG00000211689.7
460	ENSG00000020181.17	ENSG00000262049.1	ENSG00000285799.1
461	ENSG00000020219.9	ENSG00000261898.2	ENSG00000138835.22
462	ENSG00000020256.20	ENSG00000261777.5	ENSG00000239519.1
463	ENSG00000020426.11	ENSG00000261740.6	ENSG00000126005.17
464	ENSG00000020577.13	ENSG00000261737.1	ENSG00000138823.13
465	ENSG00000020633.18	ENSG00000261693.1	ENSG00000104870.12
466	ENSG00000020922.12	ENSG00000261654.1	ENSG00000235670.1
467	ENSG00000021300.14	ENSG00000261572.1	ENSG00000249506.3
468	ENSG00000021355.13	ENSG00000261386.2	ENSG00000197119.13
469	ENSG00000021461.16	ENSG00000261351.2	ENSG00000267709.1
470	ENSG00000021488.13	ENSG00000261279.5	ENSG00000134198.10
471	ENSG00000021574.12	ENSG00000261229.6	ENSG00000261664.5
472	ENSG00000021645.18	ENSG00000261147.1	ENSG00000254016.3
473	ENSG00000021762.20	ENSG00000261118.1	ENSG00000212135.1
474	ENSG00000021776.11	ENSG00000261096.1	ENSG00000259094.1

475	ENSG00000021826.15	ENSG00000261064.1	ENSG00000115593.15
476	ENSG00000021852.13	ENSG00000260979.1	ENSG00000282419.2
477	ENSG00000022267.17	ENSG00000260899.1	ENSG00000251775.1
478	ENSG00000022277.12	ENSG00000260772.1	ENSG00000227583.1
479	ENSG00000022355.17	ENSG00000260651.1	ENSG00000186150.4
480	ENSG00000022556.16	ENSG00000260613.1	ENSG00000240089.2
481	ENSG00000022567.9	ENSG00000260592.1	ENSG00000116133.13
482	ENSG00000022840.16	ENSG00000260534.1	ENSG00000253517.1
483	ENSG00000022976.15	ENSG00000260493.1	ENSG00000233542.1
484	ENSG00000023041.11	ENSG00000260475.1	ENSG00000135298.14
485	ENSG00000023171.18	ENSG00000260448.5	ENSG00000226045.1
486	ENSG00000023191.17	ENSG00000260439.1	ENSG00000132670.20
487	ENSG00000023228.14	ENSG00000260361.1	ENSG00000140853.15
488	ENSG00000023287.13	ENSG00000260092.1	ENSG00000253574.5
489	ENSG00000023318.8	ENSG00000260078.3	ENSG00000274181.1
490	ENSG00000023330.15	ENSG00000259887.1	ENSG00000074966.11
491	ENSG00000023445.14	ENSG00000259868.2	ENSG00000233511.1
492	ENSG00000023516.9	ENSG00000259746.1	ENSG00000258223.6
493	ENSG00000023572.9	ENSG00000259564.2	ENSG00000285713.1
494	ENSG00000023608.5	ENSG00000259531.2	ENSG00000159713.11
495	ENSG00000023697.13	ENSG00000259483.1	ENSG00000243267.3
496	ENSG00000023734.11	ENSG00000259448.2	ENSG00000100325.15
497	ENSG00000023839.11	ENSG00000259394.2	ENSG00000223379.1
498	ENSG00000023892.11	ENSG00000259316.11	ENSG00000210191.1
499	ENSG00000023902.14	ENSG00000259232.2	ENSG00000166171.13
500	ENSG00000023909.10	ENSG00000259225.6	ENSG00000103342.13
501	ENSG00000024048.10	ENSG00000259138.1	ENSG00000185000.12
502	ENSG00000024422.12	ENSG00000259004.1	ENSG00000172020.12
503	ENSG00000024526.17	ENSG00000258810.1	ENSG00000227972.1
504	ENSG00000024862.17	ENSG00000258704.6	ENSG00000213201.3
505	ENSG00000025039.14	ENSG00000258651.1	ENSG00000196166.4
506	ENSG00000025156.12	ENSG00000258572.1	ENSG00000188783.6
507	ENSG00000025293.17	ENSG00000258568.1	ENSG00000242512.8
508	ENSG00000025423.11	ENSG00000258539.1	ENSG00000279829.1
509	ENSG00000025434.19	ENSG00000258449.1	ENSG00000228476.1
510	ENSG00000025708.14	ENSG00000258172.1	ENSG00000269321.1
511	ENSG00000025770.19	ENSG00000257907.2	ENSG00000135917.15
512	ENSG00000025772.8	ENSG00000257613.1	ENSG00000253619.1
513	ENSG00000025796.14	ENSG00000257576.1	ENSG00000283511.1
514	ENSG00000025800.14	ENSG00000257527.1	ENSG00000115956.10
515	ENSG00000026025.16	ENSG00000257390.5	ENSG00000167332.8
516	ENSG00000026036.22	ENSG00000257354.2	ENSG00000106245.11
517	ENSG00000026103.22	ENSG00000257017.8	ENSG00000251138.6
518	ENSG00000026297.15	ENSG00000256980.5	ENSG00000182362.14
519	ENSG00000026508.18	ENSG00000256518.3	ENSG00000183395.5
520	ENSG00000026559.14	ENSG00000256427.2	ENSG00000248848.1
521	ENSG00000026652.14	ENSG00000256269.9	ENSG00000250725.2
522	ENSG00000026751.17	ENSG00000256007.1	ENSG00000230406.2
523	ENSG00000026950.17	ENSG00000255920.2	ENSG00000263655.1
524	ENSG00000027001.10	ENSG00000255725.2	ENSG00000206885.1
525	ENSG00000027075.15	ENSG00000255435.6	ENSG00000259674.1
526	ENSG00000027644.5	ENSG00000255237.1	ENSG00000200792.1
527	ENSG00000027697.14	ENSG00000254814.1	ENSG00000240270.1

528	ENSG00000027847.14	ENSG00000254810.1	ENSG00000279269.1
529	ENSG00000027869.11	ENSG00000254718.6	ENSG00000124839.13
530	ENSG00000028116.17	ENSG00000254703.2	ENSG00000149926.13
531	ENSG00000028137.19	ENSG00000254690.1	ENSG00000282173.1
532	ENSG00000028203.18	ENSG00000254528.7	ENSG00000185176.12
533	ENSG00000028277.21	ENSG00000254400.1	ENSG00000261273.1
534	ENSG00000028310.18	ENSG00000254395.1	ENSG00000270751.1
535	ENSG00000028528.15	ENSG00000253982.1	ENSG00000223963.2
536	ENSG00000028839.10	ENSG00000253967.1	ENSG00000246394.7
537	ENSG00000029153.14	ENSG00000253854.1	ENSG00000249923.1
538	ENSG00000029363.16	ENSG00000253819.1	ENSG00000125046.14
539	ENSG00000029364.12	ENSG00000253676.1	ENSG00000269139.2
540	ENSG00000029534.20	ENSG00000253540.5	ENSG00000259935.1
541	ENSG00000029559.7	ENSG00000253517.1	ENSG00000108439.10
542	ENSG00000029639.11	ENSG00000253409.1	ENSG00000257179.1
543	ENSG00000029725.17	ENSG00000253214.1	ENSG00000239473.1
544	ENSG00000029993.15	ENSG00000253144.1	ENSG00000085840.13
545	ENSG00000030066.13	ENSG00000251600.7	ENSG00000247699.2
546	ENSG00000030110.13	ENSG00000251576.1	ENSG00000223511.7
547	ENSG00000030304.13	ENSG00000251442.5	ENSG00000227398.3
548	ENSG00000030419.16	ENSG00000251405.3	ENSG00000266412.5
549	ENSG00000030582.18	ENSG00000251379.1	ENSG00000259038.1
550	ENSG00000031003.10	ENSG00000251102.1	ENSG00000239183.1
551	ENSG00000031081.10	ENSG00000251062.1	ENSG00000158373.8
552	ENSG00000031691.7	ENSG00000251050.1	ENSG00000198646.14
553	ENSG00000031698.13	ENSG00000250999.1	ENSG00000265630.2
554	ENSG00000031823.14	ENSG00000250764.1	ENSG00000257219.5
555	ENSG00000032219.18	ENSG00000250651.1	ENSG00000129173.13
556	ENSG00000032389.12	ENSG00000250616.2	ENSG00000284722.2
557	ENSG00000032444.16	ENSG00000250374.4	ENSG00000267423.1
558	ENSG00000032742.17	ENSG00000250312.8	ENSG00000212856.6
559	ENSG00000033011.12	ENSG00000250274.1	ENSG00000250347.1
560	ENSG00000033030.13	ENSG00000250240.5	ENSG00000225180.7
561	ENSG00000033050.9	ENSG00000250144.1	ENSG00000105889.15
562	ENSG00000033100.16	ENSG00000250091.3	ENSG00000162456.9
563	ENSG00000033122.19	ENSG00000250045.2	ENSG00000277053.4
564	ENSG00000033170.16	ENSG00000249931.4	ENSG00000129990.15
565	ENSG00000033178.13	ENSG00000249572.1	ENSG00000243478.9
566	ENSG00000033327.13	ENSG00000249565.2	ENSG00000207344.1
567	ENSG00000033627.16	ENSG00000249437.7	ENSG00000173641.17
568	ENSG00000033800.13	ENSG00000249412.1	ENSG00000100784.12
569	ENSG00000033867.16	ENSG00000249042.5	ENSG00000270387.1
570	ENSG00000034053.14	ENSG00000248873.1	ENSG00000119973.6
571	ENSG00000034152.18	ENSG00000248590.2	ENSG00000259007.1
572	ENSG00000034239.11	ENSG00000248492.1	ENSG00000259665.1
573	ENSG00000034510.6	ENSG00000247934.4	ENSG00000276251.1
574	ENSG00000034533.11	ENSG00000247853.2	ENSG00000251001.1
575	ENSG00000034677.13	ENSG00000247315.3	ENSG00000267218.2
576	ENSG00000034693.15	ENSG00000245534.6	ENSG00000276763.1
577	ENSG00000034713.8	ENSG00000245466.1	ENSG00000251473.2
578	ENSG00000034971.17	ENSG00000245317.2	ENSG00000267658.1
579	ENSG00000035115.21	ENSG00000244733.5	ENSG00000257564.1
580	ENSG00000035141.8	ENSG00000244649.4	ENSG00000153363.12

581	ENSG00000035403.17	ENSG00000244219.7	ENSG00000253199.1
582	ENSG00000035499.13	ENSG00000244161.1	ENSG00000234756.1
583	ENSG00000035664.11	ENSG00000243859.3	ENSG00000232059.3
584	ENSG00000035681.9	ENSG00000243830.2	ENSG00000256789.1
585	ENSG00000035687.10	ENSG00000243708.10	ENSG00000252211.1
586	ENSG00000035720.8	ENSG00000243250.1	ENSG00000186973.11
587	ENSG00000035862.12	ENSG00000243193.4	ENSG00000101040.19
588	ENSG00000035928.16	ENSG00000243104.1	ENSG00000118816.10
589	ENSG00000036054.13	ENSG00000242736.1	ENSG00000123374.11
590	ENSG00000036257.13	ENSG00000242574.9	ENSG00000108219.15
591	ENSG00000036448.10	ENSG00000242571.1	ENSG00000258282.2
592	ENSG00000036473.8	ENSG00000242550.6	ENSG00000182950.3
593	ENSG00000036530.8	ENSG00000242220.7	ENSG00000214269.3
594	ENSG00000036549.13	ENSG00000241954.1	ENSG00000275084.3
595	ENSG00000036565.15	ENSG00000241772.2	ENSG00000176970.8
596	ENSG00000036672.16	ENSG00000241599.1	ENSG00000249526.1
597	ENSG00000036828.16	ENSG00000241120.1	ENSG00000172775.17
598	ENSG00000037042.9	ENSG00000240950.1	ENSG00000272748.1
599	ENSG00000037241.7	ENSG00000240893.1	ENSG00000274425.1
600	ENSG00000037280.16	ENSG00000240809.1	ENSG00000212424.1
601	ENSG00000037474.15	ENSG00000240771.7	ENSG00000260836.2
602	ENSG00000037637.11	ENSG00000240674.1	ENSG00000196961.12
603	ENSG00000037749.12	ENSG00000240625.3	ENSG00000123243.15
604	ENSG00000037757.14	ENSG00000240583.12	ENSG00000172137.19
605	ENSG00000037897.17	ENSG00000240509.1	ENSG00000197332.8
606	ENSG00000037965.6	ENSG00000240440.1	ENSG00000114113.6
607	ENSG00000038002.9	ENSG00000240395.1	ENSG00000263551.5
608	ENSG00000038210.13	ENSG00000240370.6	ENSG00000171903.16
609	ENSG00000038219.13	ENSG00000240311.1	ENSG00000279936.1
610	ENSG00000038274.17	ENSG00000240291.1	ENSG00000262358.1
611	ENSG00000038295.8	ENSG00000239868.3	ENSG00000217646.1
612	ENSG00000038358.15	ENSG00000239713.9	ENSG00000260460.1
613	ENSG00000038382.20	ENSG00000239605.11	ENSG00000236216.5
614	ENSG00000038427.16	ENSG00000239528.1	ENSG00000224365.1
615	ENSG00000038532.16	ENSG00000239470.3	ENSG00000256037.1
616	ENSG00000038945.15	ENSG00000239335.4	ENSG00000285866.1
617	ENSG00000039068.19	ENSG00000239282.7	ENSG00000255021.1
618	ENSG00000039123.16	ENSG00000238221.1	ENSG00000232527.7
619	ENSG00000039139.9	ENSG00000238133.6	ENSG00000227211.2
620	ENSG00000039319.17	ENSG00000237529.2	ENSG00000137673.9
621	ENSG00000039523.20	ENSG00000237372.3	ENSG00000278931.1
622	ENSG00000039537.13	ENSG00000237214.3	ENSG00000237361.2
623	ENSG00000039560.14	ENSG00000237036.4	ENSG00000224647.2
624	ENSG00000039600.11	ENSG00000236942.1	ENSG00000229618.2
625	ENSG00000039650.12	ENSG00000236753.6	ENSG00000228770.1
626	ENSG00000039987.6	ENSG00000236735.3	ENSG00000226851.1
627	ENSG00000040199.18	ENSG00000236653.2	ENSG00000228966.2
628	ENSG00000040275.17	ENSG00000236577.1	ENSG00000285950.1
629	ENSG00000040341.18	ENSG00000236345.1	ENSG00000271983.1
630	ENSG00000040487.13	ENSG00000236327.2	ENSG00000183049.12
631	ENSG00000040531.14	ENSG00000236296.8	ENSG00000006283.18
632	ENSG00000040608.14	ENSG00000236283.4	ENSG00000189212.12
633	ENSG00000040633.13	ENSG00000236266.1	ENSG00000232073.1

634	ENSG00000040731.10	ENSG00000236204.6	ENSG00000278097.1
635	ENSG00000040933.15	ENSG00000236156.2	ENSG00000140830.9
636	ENSG00000041353.10	ENSG00000236085.1	ENSG00000173250.2
637	ENSG00000041357.16	ENSG00000235782.1	ENSG00000260634.1
638	ENSG00000041515.16	ENSG00000235750.10	ENSG00000130821.16
639	ENSG00000041802.11	ENSG00000235663.1	ENSG00000285957.1
640	ENSG00000041880.14	ENSG00000235505.7	ENSG00000180758.12
641	ENSG00000041982.16	ENSG00000235313.1	ENSG00000171435.13
642	ENSG00000041988.15	ENSG00000235272.2	ENSG00000286241.1
643	ENSG00000042062.12	ENSG00000235119.1	ENSG00000198128.4
644	ENSG00000042088.13	ENSG00000235079.1	ENSG00000233115.4
645	ENSG00000042286.15	ENSG00000234964.4	ENSG00000250613.1
646	ENSG00000042304.11	ENSG00000234945.7	ENSG00000197182.14
647	ENSG00000042317.17	ENSG00000234911.2	ENSG00000139970.17
648	ENSG00000042429.12	ENSG00000234902.6	ENSG00000271969.1
649	ENSG00000042445.14	ENSG00000234648.1	ENSG00000184792.16
650	ENSG00000042493.16	ENSG00000234518.2	ENSG00000089356.18
651	ENSG00000042753.11	ENSG00000234431.2	ENSG00000256442.1
652	ENSG00000042781.13	ENSG00000234323.6	ENSG00000205669.3
653	ENSG00000042813.8	ENSG00000234282.1	ENSG00000100206.10
654	ENSG00000042832.12	ENSG00000234199.2	ENSG00000137285.10
655	ENSG00000042980.12	ENSG00000234093.3	ENSG00000237642.1
656	ENSG00000043039.6	ENSG00000234005.3	ENSG00000285988.1
657	ENSG00000043093.14	ENSG00000233822.4	ENSG00000228219.1
658	ENSG00000043143.20	ENSG00000233820.2	ENSG00000162722.9
659	ENSG00000043355.12	ENSG00000233730.1	ENSG00000145335.15
660	ENSG00000043462.12	ENSG00000233622.3	ENSG00000163132.7
661	ENSG00000043514.16	ENSG00000233597.3	ENSG00000162981.14
662	ENSG00000043591.5	ENSG00000233588.1	ENSG00000237613.2
663	ENSG00000044012.4	ENSG00000233554.5	ENSG00000188782.8
664	ENSG00000044090.8	ENSG00000233426.3	ENSG00000254023.1
665	ENSG00000044115.21	ENSG00000233392.5	ENSG00000131019.11
666	ENSG00000044446.11	ENSG00000233327.10	ENSG00000179626.4
667	ENSG00000044459.15	ENSG00000233175.2	ENSG00000149923.14
668	ENSG00000044524.11	ENSG00000233029.3	ENSG00000267034.1
669	ENSG00000044574.8	ENSG00000232774.7	ENSG00000237775.1
670	ENSG00000046604.13	ENSG00000232587.1	ENSG00000215378.3
671	ENSG00000046647.13	ENSG00000232533.1	ENSG00000206337.10
672	ENSG00000046651.15	ENSG00000232273.1	ENSG00000228786.5
673	ENSG00000046653.15	ENSG00000232065.1	ENSG00000243568.2
674	ENSG00000046774.10	ENSG00000231964.1	ENSG00000223663.2
675	ENSG00000046889.19	ENSG00000231948.2	ENSG00000236510.1
676	ENSG00000047056.15	ENSG00000231527.6	ENSG00000179826.6
677	ENSG00000047188.16	ENSG00000231256.7	ENSG00000228874.1
678	ENSG00000047230.15	ENSG00000231167.3	ENSG00000106341.11
679	ENSG00000047249.18	ENSG00000231105.1	ENSG00000279780.1
680	ENSG00000047315.16	ENSG00000231007.5	ENSG00000206650.1
681	ENSG00000047346.12	ENSG00000230896.1	ENSG00000234230.1
682	ENSG00000047365.12	ENSG00000230492.1	ENSG00000186567.12
683	ENSG00000047410.14	ENSG00000230399.1	ENSG00000173868.11
684	ENSG00000047457.14	ENSG00000230366.9	ENSG00000181826.10
685	ENSG00000047578.13	ENSG00000230304.1	ENSG00000197261.11
686	ENSG00000047579.19	ENSG00000230042.1	ENSG00000274317.1

687	ENSG00000047597.7	ENSG00000229859.9	ENSG00000241954.1
688	ENSG00000047617.15	ENSG00000229692.3	ENSG00000224172.1
689	ENSG00000047621.12	ENSG00000229414.2	ENSG00000230188.1
690	ENSG00000047634.15	ENSG00000229344.1	ENSG00000185236.12
691	ENSG00000047644.18	ENSG00000229299.2	ENSG00000228452.1
692	ENSG00000047648.22	ENSG00000229127.1	ENSG00000105675.8
693	ENSG00000047662.4	ENSG00000229056.2	ENSG00000267245.1
694	ENSG00000047849.21	ENSG00000229043.2	ENSG00000171759.10
695	ENSG00000047932.14	ENSG00000228817.4	ENSG00000184258.7
696	ENSG00000047936.10	ENSG00000228782.7	ENSG00000267084.1
697	ENSG00000048028.11	ENSG00000228677.1	ENSG00000257735.1
698	ENSG00000048052.21	ENSG00000228672.3	ENSG00000255092.1
699	ENSG00000048140.18	ENSG00000228638.1	ENSG00000234135.2
700	ENSG00000048162.20	ENSG00000228327.3	ENSG00000152670.18
701	ENSG00000048342.16	ENSG00000228037.1	ENSG00000231468.2
702	ENSG00000048392.11	ENSG00000227533.5	ENSG00000235254.3
703	ENSG00000048405.10	ENSG00000227359.1	ENSG00000227722.2
704	ENSG00000048462.11	ENSG00000227345.8	ENSG00000233045.1
705	ENSG00000048471.14	ENSG00000227081.5	ENSG00000137094.14
706	ENSG00000048544.6	ENSG00000227039.6	ENSG00000262211.1
707	ENSG00000048545.13	ENSG00000227009.1	ENSG00000237492.1
708	ENSG00000048649.13	ENSG00000226986.4	ENSG00000226535.1
709	ENSG00000048707.15	ENSG00000226891.8	ENSG00000165949.12
710	ENSG00000048740.18	ENSG00000226822.2	ENSG00000268598.1
711	ENSG00000048828.17	ENSG00000226761.3	ENSG00000215720.4
712	ENSG00000048991.16	ENSG00000226751.2	ENSG00000269097.1
713	ENSG00000049089.15	ENSG00000226380.9	ENSG00000249774.1
714	ENSG00000049130.15	ENSG00000226252.1	ENSG00000202187.1
715	ENSG00000049167.14	ENSG00000226009.1	ENSG00000241400.1
716	ENSG00000049192.15	ENSG00000225854.2	ENSG00000224061.1
717	ENSG00000049239.12	ENSG00000225831.1	ENSG00000240891.7
718	ENSG00000049245.13	ENSG00000225793.2	ENSG00000260765.2
719	ENSG00000049246.14	ENSG00000225756.1	ENSG00000085999.12
720	ENSG00000049247.13	ENSG00000225513.1	ENSG00000178033.6
721	ENSG00000049249.8	ENSG00000225195.2	ENSG00000250535.1
722	ENSG00000049283.18	ENSG00000225137.1	ENSG00000237999.2
723	ENSG00000049323.16	ENSG00000224934.4	ENSG00000004948.14
724	ENSG00000049449.9	ENSG00000224839.1	ENSG00000228510.2
725	ENSG00000049540.17	ENSG00000224672.4	ENSG00000284638.1
726	ENSG00000049541.11	ENSG00000224628.2	ENSG00000281477.1
727	ENSG00000049618.23	ENSG00000224533.4	ENSG00000225681.2
728	ENSG00000049656.14	ENSG00000224387.1	ENSG00000183735.10
729	ENSG00000049759.18	ENSG00000224376.1	ENSG00000255382.1
730	ENSG00000049768.15	ENSG00000224165.5	ENSG00000241947.1
731	ENSG00000049769.13	ENSG00000224080.1	ENSG00000207129.1
732	ENSG00000049860.14	ENSG00000223820.5	ENSG00000253915.1
733	ENSG00000049883.15	ENSG00000223773.7	ENSG00000230228.2
734	ENSG00000050030.15	ENSG00000223711.1	ENSG00000280182.1
735	ENSG00000050130.18	ENSG00000223552.1	ENSG00000144635.9
736	ENSG00000050165.17	ENSG00000222328.1	ENSG00000233416.1
737	ENSG00000050327.15	ENSG00000221983.7	ENSG00000233096.1
738	ENSG00000050344.9	ENSG00000221859.2	ENSG00000122035.6
739	ENSG00000050393.11	ENSG00000220848.5	ENSG00000051180.17

740	ENSG00000050405.13	ENSG00000220744.1	ENSG00000199266.1
741	ENSG00000050426.16	ENSG00000220201.7	ENSG00000141030.13
742	ENSG00000050438.17	ENSG00000219773.1	ENSG00000267035.1
743	ENSG00000050555.18	ENSG00000219451.3	ENSG00000260413.1
744	ENSG00000050628.20	ENSG00000219355.2	ENSG00000242509.3
745	ENSG00000050730.16	ENSG00000218537.1	ENSG00000271989.1
746	ENSG00000050748.17	ENSG00000218418.2	ENSG00000002079.14
747	ENSG00000050767.17	ENSG00000218283.2	ENSG00000207165.1
748	ENSG00000050820.17	ENSG00000217083.1	ENSG00000135926.15
749	ENSG00000051009.10	ENSG00000215895.4	ENSG00000142449.13
750	ENSG00000051108.15	ENSG00000215817.7	ENSG00000259948.2
751	ENSG00000051128.19	ENSG00000215811.6	ENSG00000136834.3
752	ENSG00000051180.17	ENSG00000215464.4	ENSG00000102359.7
753	ENSG00000051341.14	ENSG00000215440.12	ENSG00000272023.1
754	ENSG00000051382.8	ENSG00000214517.10	ENSG00000148948.8
755	ENSG00000051523.11	ENSG00000214465.3	ENSG00000267147.2
756	ENSG00000051596.9	ENSG00000214362.2	ENSG00000184033.13
757	ENSG00000051620.11	ENSG00000214263.2	ENSG00000213750.4
758	ENSG00000051825.14	ENSG00000213889.10	ENSG00000153071.15
759	ENSG00000052126.14	ENSG00000213865.7	ENSG00000266801.1
760	ENSG00000052344.16	ENSG00000213862.4	ENSG00000166104.15
761	ENSG00000052723.11	ENSG00000213753.11	ENSG00000259941.1
762	ENSG00000052749.13	ENSG00000213740.2	ENSG00000181609.5
763	ENSG00000052795.13	ENSG00000213700.3	ENSG00000168779.19
764	ENSG00000052802.13	ENSG00000213672.8	ENSG00000267338.1
765	ENSG00000052841.15	ENSG00000213609.3	ENSG00000137142.5
766	ENSG00000052850.7	ENSG00000213453.3	ENSG00000256159.1
767	ENSG00000053108.17	ENSG00000213445.10	ENSG00000226900.1
768	ENSG00000053254.15	ENSG00000213432.2	ENSG00000102104.8
769	ENSG00000053328.8	ENSG00000213398.7	ENSG00000268157.1
770	ENSG00000053371.12	ENSG00000213376.4	ENSG00000251431.1
771	ENSG00000053372.5	ENSG00000213214.4	ENSG00000261315.2
772	ENSG00000053438.10	ENSG00000213085.10	ENSG00000197241.3
773	ENSG00000053501.13	ENSG00000213047.13	ENSG00000159247.13
774	ENSG00000053524.12	ENSG00000212961.4	ENSG00000236474.1
775	ENSG00000053702.15	ENSG00000212864.3	ENSG00000175894.17
776	ENSG00000053747.16	ENSG00000211973.2	ENSG00000088325.16
777	ENSG00000053770.12	ENSG00000211896.7	ENSG00000258404.1
778	ENSG00000053900.10	ENSG00000211802.3	ENSG00000065911.12
779	ENSG00000053918.16	ENSG00000211725.3	ENSG00000277986.1
780	ENSG00000054116.12	ENSG00000211720.3	ENSG00000231521.1
781	ENSG00000054118.15	ENSG00000211668.2	ENSG00000244043.1
782	ENSG00000054148.17	ENSG00000211642.3	ENSG00000121753.12
783	ENSG00000054179.12	ENSG00000211584.14	ENSG00000079308.19
784	ENSG00000054219.11	ENSG00000209082.1	ENSG00000225746.11
785	ENSG00000054267.22	ENSG00000206344.7	ENSG00000274666.1
786	ENSG00000054277.14	ENSG00000206177.7	ENSG00000265018.6
787	ENSG00000054282.16	ENSG00000205809.9	ENSG00000149596.6
788	ENSG00000054356.14	ENSG00000205795.4	ENSG00000164007.10
789	ENSG00000054392.13	ENSG00000205560.12	ENSG00000278431.1
790	ENSG00000054523.17	ENSG00000205537.2	ENSG00000224687.1
791	ENSG00000054598.8	ENSG00000205414.1	ENSG00000244710.3
792	ENSG00000054611.14	ENSG00000205307.12	ENSG00000222808.1

793	ENSG00000054654.16	ENSG00000205085.11	ENSG00000241307.1
794	ENSG00000054690.13	ENSG00000204758.7	ENSG00000284154.1
795	ENSG00000054793.13	ENSG00000204710.2	ENSG00000254984.1
796	ENSG00000054796.12	ENSG00000204564.12	ENSG00000259496.2
797	ENSG00000054803.3	ENSG00000204366.3	ENSG00000157184.7
798	ENSG00000054938.15	ENSG00000204305.14	ENSG00000180644.8
799	ENSG00000054965.10	ENSG00000204272.12	ENSG00000236404.9
800	ENSG00000054967.13	ENSG00000204264.10	ENSG00000253390.1
801	ENSG00000054983.17	ENSG00000204188.7	ENSG00000278496.1
802	ENSG00000055044.11	ENSG00000204104.12	ENSG00000204434.5
803	ENSG00000055070.17	ENSG00000203876.9	ENSG00000244391.3
804	ENSG00000055118.14	ENSG00000203791.15	ENSG00000243499.1
805	ENSG00000055130.16	ENSG00000203734.11	ENSG00000273186.1
806	ENSG00000055147.19	ENSG00000198947.15	ENSG00000229007.1
807	ENSG00000055163.20	ENSG00000198938.2	ENSG00000134874.17
808	ENSG00000055208.19	ENSG00000198920.10	ENSG00000234356.1
809	ENSG00000055211.14	ENSG00000198892.6	ENSG00000260326.1
810	ENSG00000055332.18	ENSG00000198890.8	ENSG00000179562.3
811	ENSG00000055483.19	ENSG00000198876.13	ENSG00000263958.1
812	ENSG00000055609.18	ENSG00000198825.13	ENSG00000269540.1
813	ENSG00000055732.13	ENSG00000198818.10	ENSG00000172244.9
814	ENSG00000055813.6	ENSG00000198792.13	ENSG00000107736.20
815	ENSG00000055917.15	ENSG00000198785.5	ENSG00000214796.8
816	ENSG00000055950.16	ENSG00000198723.11	ENSG00000113327.16
817	ENSG00000055955.16	ENSG00000198658.4	ENSG00000235617.1
818	ENSG00000055957.11	ENSG00000198569.9	ENSG00000234664.1
819	ENSG00000056050.7	ENSG00000198538.11	ENSG00000234181.1
820	ENSG00000056097.16	ENSG00000198467.14	ENSG00000007001.12
821	ENSG00000056277.16	ENSG00000198342.10	ENSG00000100380.14
822	ENSG00000056291.17	ENSG00000198336.9	ENSG00000204673.10
823	ENSG00000056487.16	ENSG00000198237.8	ENSG00000153767.10
824	ENSG00000056558.11	ENSG00000198146.4	ENSG00000279127.1
825	ENSG00000056586.16	ENSG00000198108.4	ENSG00000130711.3
826	ENSG00000056736.10	ENSG00000198087.7	ENSG00000214561.3
827	ENSG00000056972.19	ENSG00000198081.11	ENSG00000259738.1
828	ENSG00000057019.16	ENSG00000198056.14	ENSG00000267225.1
829	ENSG00000057149.16	ENSG00000197965.12	ENSG00000218186.2
830	ENSG00000057252.13	ENSG00000197852.11	ENSG00000259054.1
831	ENSG00000057294.14	ENSG00000197798.9	ENSG00000256001.1
832	ENSG00000057468.7	ENSG00000197794.2	ENSG00000278480.1
833	ENSG00000057593.13	ENSG00000197712.12	ENSG00000182389.19
834	ENSG00000057608.17	ENSG00000197506.7	ENSG00000219329.1
835	ENSG00000057657.17	ENSG00000197223.11	ENSG00000105357.16
836	ENSG00000057663.16	ENSG00000197191.5	ENSG00000280259.1
837	ENSG00000057704.13	ENSG00000197165.11	ENSG00000132855.4
838	ENSG00000057757.10	ENSG00000197099.8	ENSG00000122735.16
839	ENSG00000057935.13	ENSG00000197063.11	ENSG00000255255.1
840	ENSG00000058056.9	ENSG00000196872.12	ENSG00000270040.1
841	ENSG00000058063.16	ENSG00000196862.9	ENSG00000282300.1
842	ENSG00000058085.14	ENSG00000196604.12	ENSG00000151702.17
843	ENSG00000058091.17	ENSG00000196581.10	ENSG00000269736.1
844	ENSG00000058262.10	ENSG00000196535.16	ENSG00000224674.1
845	ENSG00000058272.19	ENSG00000196526.10	ENSG00000214925.3

846	ENSG00000058335.15	ENSG00000196436.8	ENSG00000274044.4
847	ENSG00000058404.20	ENSG00000196372.13	ENSG00000277639.2
848	ENSG00000058453.17	ENSG00000196357.11	ENSG00000117560.8
849	ENSG00000058600.16	ENSG00000196110.7	ENSG00000224906.3
850	ENSG00000058668.14	ENSG00000189369.8	ENSG00000241362.2
851	ENSG00000058673.16	ENSG00000189266.13	ENSG00000047188.16
852	ENSG00000058729.11	ENSG00000189042.13	ENSG00000108231.13
853	ENSG00000058799.15	ENSG00000189014.7	ENSG00000231668.2
854	ENSG00000058804.12	ENSG00000188997.8	ENSG00000267069.1
855	ENSG00000058866.15	ENSG00000188732.11	ENSG00000234986.1
856	ENSG00000059122.16	ENSG00000188295.14	ENSG00000164669.13
857	ENSG00000059145.18	ENSG00000188199.10	ENSG00000255296.1
858	ENSG00000059377.17	ENSG00000188185.11	ENSG00000214305.4
859	ENSG00000059378.12	ENSG00000188086.13	ENSG00000254453.1
860	ENSG00000059573.9	ENSG00000188038.8	ENSG00000225026.1
861	ENSG00000059588.10	ENSG00000188026.13	ENSG00000167117.9
862	ENSG00000059691.12	ENSG00000188021.8	ENSG00000139648.6
863	ENSG00000059728.11	ENSG00000187951.11	ENSG00000260123.1
864	ENSG00000059758.8	ENSG00000187902.11	ENSG00000211825.1
865	ENSG00000059769.20	ENSG00000187870.7	ENSG00000227038.3
866	ENSG00000059804.16	ENSG00000187837.3	ENSG00000241278.1
867	ENSG00000059915.17	ENSG00000187790.11	ENSG00000233392.5
868	ENSG00000060069.16	ENSG00000187775.16	ENSG00000144278.15
869	ENSG00000060138.13	ENSG00000187741.15	ENSG00000171243.8
870	ENSG00000060140.9	ENSG00000186710.11	ENSG00000235763.1
871	ENSG00000060237.17	ENSG00000186704.9	ENSG00000057593.13
872	ENSG00000060303.5	ENSG00000186648.15	ENSG00000179520.10
873	ENSG00000060339.14	ENSG00000186522.15	ENSG00000239880.1
874	ENSG00000060491.16	ENSG00000186453.13	ENSG00000197614.11
875	ENSG00000060558.3	ENSG00000186310.9	ENSG00000259755.1
876	ENSG00000060566.14	ENSG00000186184.17	ENSG00000278869.1
877	ENSG00000060642.10	ENSG00000186174.12	ENSG00000248468.1
878	ENSG00000060656.20	ENSG00000186020.13	ENSG00000184489.11
879	ENSG00000060688.13	ENSG00000185883.12	ENSG00000268361.1
880	ENSG00000060709.15	ENSG00000185837.3	ENSG00000227496.2
881	ENSG00000060718.22	ENSG00000185736.16	ENSG00000220131.1
882	ENSG00000060749.15	ENSG00000185532.17	ENSG00000271495.1
883	ENSG00000060762.19	ENSG00000185112.5	ENSG00000186103.4
884	ENSG00000060971.18	ENSG00000185043.11	ENSG00000283554.1
885	ENSG00000060982.15	ENSG00000185022.12	ENSG00000278599.5
886	ENSG00000061273.18	ENSG00000184979.9	ENSG00000007908.16
887	ENSG00000061337.15	ENSG00000184898.7	ENSG00000273925.1
888	ENSG00000061455.11	ENSG00000184557.4	ENSG00000177752.14
889	ENSG00000061492.11	ENSG00000184497.12	ENSG00000270708.1
890	ENSG00000061656.9	ENSG00000184489.11	ENSG00000257138.1
891	ENSG00000061676.15	ENSG00000184434.7	ENSG00000132026.13
892	ENSG00000061794.13	ENSG00000184432.10	ENSG00000125818.18
893	ENSG00000061918.13	ENSG00000184363.10	ENSG00000177447.6
894	ENSG00000061936.9	ENSG00000184313.20	ENSG00000152402.10
895	ENSG00000061938.18	ENSG00000184221.13	ENSG00000199273.1
896	ENSG00000061987.16	ENSG00000184209.14	ENSG00000212251.1
897	ENSG00000062038.14	ENSG00000183971.8	ENSG00000219074.1
898	ENSG00000062096.14	ENSG00000183726.11	ENSG00000248916.1

899	ENSG00000062194.16	ENSG00000183508.5	ENSG00000256481.1
900	ENSG00000062282.15	ENSG00000183255.12	ENSG00000280498.1
901	ENSG00000062370.16	ENSG00000183196.10	ENSG00000241907.1
902	ENSG00000062485.19	ENSG00000183087.15	ENSG00000123689.6
903	ENSG00000062524.16	ENSG00000182752.10	ENSG00000229765.2
904	ENSG00000062582.14	ENSG00000182732.18	ENSG00000141314.13
905	ENSG00000062598.18	ENSG00000182580.3	ENSG00000232608.1
906	ENSG00000062650.19	ENSG00000182534.13	ENSG00000234632.1
907	ENSG00000062716.13	ENSG00000182473.22	ENSG00000185888.5
908	ENSG00000062725.10	ENSG00000182389.19	ENSG00000206897.1
909	ENSG00000062822.13	ENSG00000182362.14	ENSG00000254388.1
910	ENSG00000063015.19	ENSG00000182327.8	ENSG00000226842.1
911	ENSG00000063046.17	ENSG00000182326.15	ENSG00000255448.1
912	ENSG00000063127.15	ENSG00000182240.16	ENSG00000261501.1
913	ENSG00000063169.10	ENSG00000182118.8	ENSG00000138629.16
914	ENSG00000063176.15	ENSG00000181982.18	ENSG00000240521.1
915	ENSG00000063177.13	ENSG00000181938.13	ENSG00000143549.20
916	ENSG00000063180.9	ENSG00000181908.5	ENSG00000261457.3
917	ENSG00000063241.8	ENSG00000181741.7	ENSG00000151365.2
918	ENSG00000063244.12	ENSG00000181588.16	ENSG00000260047.2
919	ENSG00000063245.14	ENSG00000181481.14	ENSG00000229977.2
920	ENSG00000063322.14	ENSG00000181467.4	ENSG00000056998.20
921	ENSG00000063438.17	ENSG00000181350.11	ENSG00000279044.1
922	ENSG00000063515.2	ENSG00000181322.14	ENSG00000163781.13
923	ENSG00000063587.14	ENSG00000181264.8	ENSG00000230635.2
924	ENSG00000063601.17	ENSG00000181026.15	ENSG00000254329.1
925	ENSG00000063660.9	ENSG00000180815.14	ENSG00000272130.1
926	ENSG00000063761.16	ENSG00000180758.12	ENSG00000219222.1
927	ENSG00000063854.13	ENSG00000180739.14	ENSG00000214549.2
928	ENSG00000063978.16	ENSG00000180644.8	ENSG00000135502.17
929	ENSG00000064012.21	ENSG00000180596.7	ENSG00000117594.10
930	ENSG00000064042.18	ENSG00000180385.8	ENSG00000089505.17
931	ENSG00000064102.15	ENSG00000180015.12	ENSG00000269296.1
932	ENSG00000064115.11	ENSG00000179978.11	ENSG00000047365.12
933	ENSG00000064195.7	ENSG00000179967.11	ENSG00000235052.1
934	ENSG00000064199.6	ENSG00000179918.18	ENSG00000232480.1
935	ENSG00000064201.15	ENSG00000179914.5	ENSG00000183160.9
936	ENSG00000064205.10	ENSG00000179820.16	ENSG00000267104.2
937	ENSG00000064218.5	ENSG00000179761.12	ENSG00000268174.1
938	ENSG00000064225.12	ENSG00000179698.13	ENSG00000278719.1
939	ENSG00000064270.12	ENSG00000179632.10	ENSG00000169347.16
940	ENSG00000064300.9	ENSG00000179526.17	ENSG00000275803.1
941	ENSG00000064309.14	ENSG00000179455.8	ENSG00000227173.1
942	ENSG00000064313.12	ENSG00000179361.17	ENSG00000204482.10
943	ENSG00000064393.16	ENSG00000179344.16	ENSG00000178522.14
944	ENSG00000064419.13	ENSG00000179304.16	ENSG00000273877.4
945	ENSG00000064489.22	ENSG00000179057.13	ENSG00000259770.2
946	ENSG00000064490.14	ENSG00000178860.8	ENSG00000218632.3
947	ENSG00000064545.14	ENSG00000178719.17	ENSG00000132874.14
948	ENSG00000064547.14	ENSG00000178623.12	ENSG00000116176.6
949	ENSG00000064601.18	ENSG00000178573.7	ENSG00000256651.1
950	ENSG00000064607.17	ENSG00000178458.5	ENSG00000150722.10
951	ENSG00000064651.14	ENSG00000178425.14	ENSG00000203782.6

952	ENSG00000064652.11	ENSG00000177990.11	ENSG00000225739.1
953	ENSG00000064655.19	ENSG00000177989.13	ENSG00000233708.1
954	ENSG00000064666.15	ENSG00000177889.10	ENSG00000226263.1
955	ENSG00000064687.12	ENSG00000177873.13	ENSG00000142186.17
956	ENSG00000064692.19	ENSG00000177807.9	ENSG00000176029.13
957	ENSG00000064703.12	ENSG00000177370.5	ENSG00000279440.1
958	ENSG00000064726.9	ENSG00000177324.13	ENSG00000267638.1
959	ENSG00000064763.11	ENSG00000177272.9	ENSG00000231840.1
960	ENSG00000064787.13	ENSG00000176788.9	ENSG00000255331.1
961	ENSG00000064835.10	ENSG00000176731.12	ENSG00000281990.1
962	ENSG00000064886.14	ENSG00000176720.6	ENSG00000113209.8
963	ENSG00000064932.16	ENSG00000176595.4	ENSG00000043039.6
964	ENSG00000064933.17	ENSG00000176428.6	ENSG00000115648.14
965	ENSG00000064961.19	ENSG00000176386.9	ENSG00000182050.13
966	ENSG00000064989.13	ENSG00000176236.6	ENSG00000223519.8
967	ENSG00000064995.17	ENSG00000176092.15	ENSG00000164778.4
968	ENSG00000064999.15	ENSG00000176049.16	ENSG00000203387.2
969	ENSG00000065000.17	ENSG00000175895.4	ENSG00000236792.1
970	ENSG00000065029.15	ENSG00000175894.17	ENSG00000169291.10
971	ENSG00000065054.14	ENSG00000175874.10	ENSG00000106615.10
972	ENSG00000065057.8	ENSG00000175741.5	ENSG00000197965.12
973	ENSG00000065060.17	ENSG00000175505.11	ENSG00000104946.13
974	ENSG00000065135.11	ENSG00000175352.11	ENSG00000244039.1
975	ENSG00000065150.19	ENSG00000175166.17	ENSG00000189195.13
976	ENSG00000065154.11	ENSG00000175164.15	ENSG00000223849.1
977	ENSG00000065183.16	ENSG00000175029.17	ENSG00000229965.1
978	ENSG00000065243.20	ENSG00000174951.11	ENSG00000258609.3
979	ENSG00000065268.10	ENSG00000174607.11	ENSG00000216360.1
980	ENSG00000065308.5	ENSG00000174527.9	ENSG00000020219.9
981	ENSG00000065320.9	ENSG00000174469.22	ENSG00000149557.13
982	ENSG00000065325.13	ENSG00000174403.15	ENSG00000232754.1
983	ENSG00000065328.16	ENSG00000174177.13	ENSG00000258052.1
984	ENSG00000065357.20	ENSG00000174173.7	ENSG00000222268.1
985	ENSG00000065361.15	ENSG00000174059.16	ENSG00000243730.2
986	ENSG00000065371.17	ENSG00000173928.2	ENSG00000106366.8
987	ENSG00000065413.20	ENSG00000173868.11	ENSG00000258500.2
988	ENSG00000065427.14	ENSG00000173821.19	ENSG00000278581.1
989	ENSG00000065457.10	ENSG00000173812.11	ENSG00000189332.5
990	ENSG00000065485.20	ENSG00000173811.10	ENSG00000186399.10
991	ENSG00000065491.8	ENSG00000173581.7	ENSG00000251309.1
992	ENSG00000065518.8	ENSG00000173567.15	ENSG00000143416.21
993	ENSG00000065526.11	ENSG00000173207.13	ENSG00000113712.17
994	ENSG00000065534.18	ENSG00000173065.13	ENSG00000204839.9
995	ENSG00000065548.18	ENSG00000173039.19	ENSG00000270934.1
996	ENSG00000065559.15	ENSG00000172965.15	ENSG00000212154.1
997	ENSG00000065600.12	ENSG00000172661.18	ENSG00000259155.2
998	ENSG00000065609.14	ENSG00000172572.6	ENSG00000265790.1
999	ENSG00000065613.14	ENSG00000172432.19	ENSG00000284146.1
1000	ENSG00000065615.14	ENSG00000172361.6	ENSG00000257542.5
1001	ENSG00000065618.20	ENSG00000172346.15	ENSG00000255621.1
1002	ENSG00000065621.15	ENSG00000172296.13	ENSG00000229601.1
1003	ENSG00000065665.21	ENSG00000172250.16	ENSG00000215113.6
1004	ENSG00000065675.14	ENSG00000172236.17	ENSG00000232400.1

1005	ENSG00000065717.15	ENSG00000171497.5	ENSG00000149485.18
1006	ENSG00000065802.12	ENSG00000171316.12	ENSG00000232332.1
1007	ENSG00000065809.13	ENSG00000171241.9	ENSG00000279085.1
1008	ENSG00000065833.9	ENSG00000171202.7	ENSG00000006377.11
1009	ENSG00000065882.16	ENSG00000171174.15	ENSG00000142621.19
1010	ENSG00000065883.16	ENSG00000171132.14	ENSG00000256379.2
1011	ENSG00000065911.12	ENSG00000170837.2	ENSG00000152467.9
1012	ENSG00000065923.10	ENSG00000170827.9	ENSG00000253408.5
1013	ENSG00000065970.9	ENSG00000170502.13	ENSG00000180573.9
1014	ENSG00000065978.19	ENSG00000170122.5	ENSG00000237441.10
1015	ENSG00000065989.16	ENSG00000170075.9	ENSG00000279714.1
1016	ENSG00000066027.12	ENSG00000170027.7	ENSG00000228943.1
1017	ENSG00000066032.18	ENSG00000169877.10	ENSG00000234753.5
1018	ENSG00000066044.15	ENSG00000169744.13	ENSG00000181359.5
1019	ENSG00000066056.14	ENSG00000169629.11	ENSG00000229896.2
1020	ENSG00000066084.13	ENSG00000169570.10	ENSG00000111666.11
1021	ENSG00000066117.14	ENSG00000169432.15	ENSG00000230284.1
1022	ENSG00000066135.13	ENSG00000169242.12	ENSG00000253532.1
1023	ENSG00000066136.20	ENSG00000169194.9	ENSG00000043462.12
1024	ENSG00000066185.13	ENSG00000169087.11	ENSG00000279889.1
1025	ENSG00000066230.11	ENSG00000168918.14	ENSG00000199839.1
1026	ENSG00000066248.15	ENSG00000168246.6	ENSG00000258474.1
1027	ENSG00000066279.18	ENSG00000168036.18	ENSG00000166741.7
1028	ENSG00000066294.14	ENSG00000168014.16	ENSG00000223460.1
1029	ENSG00000066322.15	ENSG00000167987.11	ENSG00000166793.11
1030	ENSG00000066336.11	ENSG00000167912.5	ENSG00000151963.4
1031	ENSG00000066379.15	ENSG00000167779.9	ENSG00000005483.20
1032	ENSG00000066382.16	ENSG00000167775.11	ENSG00000261011.1
1033	ENSG00000066405.13	ENSG00000167552.14	ENSG00000260581.1
1034	ENSG00000066422.4	ENSG00000167483.18	ENSG00000183239.5
1035	ENSG00000066427.22	ENSG00000167461.12	ENSG00000261734.1
1036	ENSG00000066455.13	ENSG00000167216.16	ENSG00000177602.5
1037	ENSG00000066468.22	ENSG00000167202.11	ENSG00000232832.1
1038	ENSG00000066557.6	ENSG00000167191.12	ENSG00000248473.1
1039	ENSG00000066583.12	ENSG00000166949.16	ENSG00000217159.2
1040	ENSG00000066629.17	ENSG00000166856.3	ENSG00000285448.1
1041	ENSG00000066651.20	ENSG00000166664.13	ENSG00000268643.1
1042	ENSG00000066654.14	ENSG00000166548.15	ENSG00000249908.2
1043	ENSG00000066697.14	ENSG00000166526.17	ENSG00000265813.2
1044	ENSG00000066735.14	ENSG00000166454.10	ENSG00000263707.1
1045	ENSG00000066739.12	ENSG00000166444.19	ENSG00000242818.3
1046	ENSG00000066777.9	ENSG00000166394.15	ENSG00000253376.1
1047	ENSG00000066813.14	ENSG00000166086.12	ENSG00000230480.1
1048	ENSG00000066827.16	ENSG00000166016.6	ENSG00000259831.1
1049	ENSG00000066855.16	ENSG00000165832.6	ENSG00000232310.7
1050	ENSG00000066923.17	ENSG00000165804.16	ENSG00000231192.2
1051	ENSG00000066926.11	ENSG00000165801.10	ENSG00000244748.3
1052	ENSG00000066933.16	ENSG00000165752.17	ENSG00000269420.5
1053	ENSG00000067048.17	ENSG00000165661.16	ENSG00000256199.1
1054	ENSG00000067057.17	ENSG00000165475.15	ENSG00000275591.4
1055	ENSG00000067064.11	ENSG00000165304.8	ENSG00000274966.1
1056	ENSG00000067066.17	ENSG00000165175.15	ENSG00000229356.1
1057	ENSG00000067082.15	ENSG00000165113.13	ENSG00000244585.1

1058	ENSG00000067113.17	ENSG00000164967.10	ENSG00000086205.18
1059	ENSG00000067141.17	ENSG00000164929.17	ENSG00000237708.1
1060	ENSG00000067167.8	ENSG00000164885.13	ENSG00000176134.5
1061	ENSG00000067177.15	ENSG00000164867.11	ENSG00000229197.1
1062	ENSG00000067182.7	ENSG00000164741.15	ENSG00000236459.1
1063	ENSG00000067191.16	ENSG00000164669.13	ENSG00000279463.1
1064	ENSG00000067208.14	ENSG00000164610.9	ENSG00000255203.1
1065	ENSG00000067221.14	ENSG00000164190.18	ENSG00000230333.6
1066	ENSG00000067225.18	ENSG00000164167.12	ENSG00000275017.1
1067	ENSG00000067248.10	ENSG00000164120.14	ENSG00000274937.1
1068	ENSG00000067334.14	ENSG00000164061.5	ENSG00000236317.1
1069	ENSG00000067365.14	ENSG00000163959.10	ENSG00000254246.1
1070	ENSG00000067369.13	ENSG00000163902.12	ENSG00000281955.1
1071	ENSG00000067445.20	ENSG00000163870.15	ENSG00000258204.1
1072	ENSG00000067533.6	ENSG00000163833.8	ENSG00000243008.2
1073	ENSG00000067560.11	ENSG00000163810.11	ENSG00000165023.7
1074	ENSG00000067596.11	ENSG00000163754.17	ENSG00000170537.13
1075	ENSG00000067601.8	ENSG00000163734.4	ENSG00000213698.2
1076	ENSG00000067606.17	ENSG00000163719.19	ENSG00000285528.1
1077	ENSG00000067646.12	ENSG00000163689.20	ENSG00000185811.18
1078	ENSG00000067704.10	ENSG00000163558.13	ENSG00000072501.17
1079	ENSG00000067715.14	ENSG00000163017.13	ENSG00000223974.1
1080	ENSG00000067798.16	ENSG00000162929.13	ENSG00000129682.16
1081	ENSG00000067829.19	ENSG00000162873.14	ENSG00000255355.1
1082	ENSG00000067836.12	ENSG00000162757.4	ENSG00000227738.1
1083	ENSG00000067840.12	ENSG00000162641.19	ENSG00000277224.2
1084	ENSG00000067842.17	ENSG00000162623.16	ENSG00000242042.1
1085	ENSG00000067900.8	ENSG00000162407.9	ENSG00000275512.1
1086	ENSG00000067955.14	ENSG00000162174.12	ENSG00000188155.11
1087	ENSG00000067992.14	ENSG00000161973.11	ENSG00000073792.15
1088	ENSG00000068001.14	ENSG00000161920.10	ENSG00000236965.4
1089	ENSG00000068024.16	ENSG00000161526.15	ENSG00000203441.2
1090	ENSG00000068028.17	ENSG00000161277.10	ENSG00000141933.9
1091	ENSG00000068079.7	ENSG00000161013.17	ENSG00000238961.1
1092	ENSG00000068097.14	ENSG00000160838.14	ENSG00000255361.1
1093	ENSG00000068120.15	ENSG00000160781.17	ENSG00000284353.1
1094	ENSG00000068137.15	ENSG00000160716.5	ENSG00000258355.1
1095	ENSG00000068305.17	ENSG00000160703.16	ENSG00000259813.1
1096	ENSG00000068308.13	ENSG00000160226.16	ENSG00000256350.1
1097	ENSG00000068323.17	ENSG00000160188.10	ENSG00000269813.1
1098	ENSG00000068354.16	ENSG00000160161.9	ENSG00000274286.2
1099	ENSG00000068366.19	ENSG00000159915.12	ENSG00000234396.3
1100	ENSG00000068383.19	ENSG00000159871.15	ENSG00000175676.15
1101	ENSG00000068394.11	ENSG00000159461.15	ENSG00000278626.1
1102	ENSG00000068400.13	ENSG00000159450.12	ENSG00000131355.14
1103	ENSG00000068438.15	ENSG00000159403.17	ENSG00000189266.13
1104	ENSG00000068489.12	ENSG00000159259.8	ENSG00000275636.1
1105	ENSG00000068615.18	ENSG00000159247.13	ENSG00000240480.1
1106	ENSG00000068650.18	ENSG00000159216.18	ENSG00000183169.6
1107	ENSG00000068654.16	ENSG00000159212.12	ENSG00000104804.7
1108	ENSG00000068697.7	ENSG00000159202.18	ENSG00000231610.1
1109	ENSG00000068724.16	ENSG00000158882.15	ENSG00000233921.2
1110	ENSG00000068745.15	ENSG00000158859.10	ENSG00000253541.1

1111	ENSG00000068781.21	ENSG00000158793.14	ENSG00000259439.2
1112	ENSG00000068784.13	ENSG00000158792.16	ENSG00000233343.1
1113	ENSG00000068796.16	ENSG00000158748.4	ENSG00000070182.20
1114	ENSG00000068831.19	ENSG00000158714.11	ENSG00000138075.12
1115	ENSG00000068878.15	ENSG00000158525.15	ENSG00000187323.12
1116	ENSG00000068885.15	ENSG00000158402.20	ENSG00000235005.1
1117	ENSG00000068903.20	ENSG00000158373.8	ENSG00000275981.1
1118	ENSG00000068912.14	ENSG00000158186.13	ENSG00000259746.1
1119	ENSG00000068971.14	ENSG00000158163.15	ENSG00000232437.1
1120	ENSG00000068976.14	ENSG00000158156.7	ENSG00000284672.1
1121	ENSG00000068985.5	ENSG00000158062.20	ENSG00000285970.1
1122	ENSG00000069011.16	ENSG00000157823.17	ENSG00000221866.9
1123	ENSG00000069018.18	ENSG00000157350.13	ENSG00000215115.6
1124	ENSG00000069020.18	ENSG00000157168.19	ENSG00000115604.10
1125	ENSG00000069122.19	ENSG00000157107.14	ENSG00000157193.16
1126	ENSG00000069188.17	ENSG00000156515.23	ENSG00000250107.1
1127	ENSG00000069206.15	ENSG00000155957.17	ENSG00000242507.6
1128	ENSG00000069248.12	ENSG00000155926.14	ENSG00000231966.1
1129	ENSG00000069275.13	ENSG00000155760.2	ENSG00000229081.1
1130	ENSG00000069329.17	ENSG00000154734.15	ENSG00000226597.1
1131	ENSG00000069345.12	ENSG00000154642.11	ENSG00000180613.11
1132	ENSG00000069399.15	ENSG00000154639.19	ENSG00000002746.15
1133	ENSG00000069424.14	ENSG00000154479.13	ENSG00000141371.13
1134	ENSG00000069431.11	ENSG00000154451.14	ENSG00000123411.15
1135	ENSG00000069482.7	ENSG00000154240.17	ENSG00000259058.1
1136	ENSG00000069493.15	ENSG00000154165.5	ENSG00000254480.1
1137	ENSG00000069509.6	ENSG00000154127.10	ENSG00000270050.1
1138	ENSG00000069535.14	ENSG00000153902.14	ENSG00000240790.1
1139	ENSG00000069667.16	ENSG00000153885.14	ENSG00000228525.1
1140	ENSG00000069696.6	ENSG00000153563.15	ENSG00000277752.1
1141	ENSG00000069702.11	ENSG00000153395.10	ENSG00000189030.9
1142	ENSG00000069764.9	ENSG00000153234.14	ENSG00000260162.2
1143	ENSG00000069812.11	ENSG00000153029.14	ENSG00000235182.1
1144	ENSG00000069849.11	ENSG00000152944.9	ENSG00000259493.2
1145	ENSG00000069869.16	ENSG00000152766.6	ENSG00000277084.1
1146	ENSG00000069943.10	ENSG00000152763.17	ENSG00000164500.6
1147	ENSG00000069956.12	ENSG00000152454.4	ENSG00000184188.6
1148	ENSG00000069966.18	ENSG00000152377.14	ENSG00000229261.1
1149	ENSG00000069974.16	ENSG00000152315.5	ENSG00000226276.1
1150	ENSG00000069998.12	ENSG00000152104.12	ENSG00000250816.1
1151	ENSG00000070010.19	ENSG00000151963.4	ENSG00000146360.8
1152	ENSG00000070018.9	ENSG00000151702.17	ENSG00000274493.1
1153	ENSG00000070019.5	ENSG00000151650.8	ENSG00000201358.1
1154	ENSG00000070031.4	ENSG00000151240.17	ENSG00000262492.1
1155	ENSG00000070047.12	ENSG00000151116.17	ENSG00000237584.1
1156	ENSG00000070061.15	ENSG00000151014.6	ENSG00000259346.2
1157	ENSG00000070081.16	ENSG00000150656.15	ENSG00000224953.1
1158	ENSG00000070087.14	ENSG00000148985.19	ENSG00000268797.1
1159	ENSG00000070159.14	ENSG00000148908.15	ENSG00000131242.18
1160	ENSG00000070182.20	ENSG00000148719.15	ENSG00000238247.2
1161	ENSG00000070190.13	ENSG00000148429.14	ENSG00000226521.7
1162	ENSG00000070193.4	ENSG00000148408.13	ENSG00000233030.2
1163	ENSG00000070214.16	ENSG00000148180.19	ENSG00000259113.1

1164	ENSG00000070269.14	ENSG00000148153.14	ENSG00000260498.5
1165	ENSG00000070366.14	ENSG00000147874.11	ENSG00000237766.2
1166	ENSG00000070367.16	ENSG00000147813.16	ENSG00000223873.1
1167	ENSG00000070371.16	ENSG00000147804.9	ENSG00000229357.1
1168	ENSG00000070388.11	ENSG00000147650.11	ENSG00000165474.7
1169	ENSG00000070404.10	ENSG00000147604.14	ENSG00000254420.1
1170	ENSG00000070413.20	ENSG00000147454.14	ENSG00000228181.1
1171	ENSG00000070423.18	ENSG00000147231.14	ENSG00000233593.9
1172	ENSG00000070444.15	ENSG00000147174.11	ENSG00000134193.15
1173	ENSG00000070476.15	ENSG00000146950.13	ENSG00000278389.1
1174	ENSG00000070495.14	ENSG00000146425.11	ENSG00000278492.1
1175	ENSG00000070501.12	ENSG00000145990.11	ENSG00000198353.8
1176	ENSG00000070526.15	ENSG00000145945.6	ENSG00000225267.1
1177	ENSG00000070540.13	ENSG00000145868.16	ENSG00000278400.1
1178	ENSG00000070601.10	ENSG00000145428.14	ENSG00000254510.1
1179	ENSG00000070610.14	ENSG00000145244.12	ENSG00000257070.1
1180	ENSG00000070614.15	ENSG00000145220.14	ENSG00000167157.11
1181	ENSG00000070669.17	ENSG00000145014.17	ENSG00000271894.1
1182	ENSG00000070718.12	ENSG00000144668.12	ENSG00000167850.3
1183	ENSG00000070729.13	ENSG00000144567.11	ENSG00000137491.14
1184	ENSG00000070731.10	ENSG00000144395.18	ENSG00000215562.2
1185	ENSG00000070748.18	ENSG00000144283.21	ENSG00000251353.1
1186	ENSG00000070756.16	ENSG00000143878.9	ENSG00000228677.1
1187	ENSG00000070759.17	ENSG00000143869.7	ENSG00000199879.1
1188	ENSG00000070761.8	ENSG00000143851.15	ENSG00000181467.4
1189	ENSG00000070770.9	ENSG00000143786.8	ENSG00000280234.1
1190	ENSG00000070778.13	ENSG00000143774.16	ENSG00000225183.1
1191	ENSG00000070785.16	ENSG00000143771.12	ENSG00000263627.1
1192	ENSG00000070808.15	ENSG00000143643.13	ENSG00000226413.2
1193	ENSG00000070814.19	ENSG00000143603.19	ENSG00000177553.6
1194	ENSG00000070831.16	ENSG00000143549.20	ENSG00000111305.19
1195	ENSG00000070882.13	ENSG00000143324.13	ENSG00000233372.1
1196	ENSG00000070886.12	ENSG00000143257.11	ENSG00000166130.15
1197	ENSG00000070915.9	ENSG00000143079.15	ENSG00000237428.1
1198	ENSG00000070950.10	ENSG00000142621.19	ENSG00000283973.1
1199	ENSG00000070961.15	ENSG00000142330.20	ENSG00000257636.6
1200	ENSG00000070985.13	ENSG00000142082.15	ENSG00000285968.1
1201	ENSG00000071051.14	ENSG00000142039.4	ENSG00000066926.11
1202	ENSG00000071054.16	ENSG00000141736.13	ENSG00000226277.1
1203	ENSG00000071073.13	ENSG00000141664.9	ENSG00000204577.11
1204	ENSG00000071082.11	ENSG00000141568.21	ENSG00000243101.1
1205	ENSG00000071127.17	ENSG00000141543.11	ENSG00000259992.1
1206	ENSG00000071189.21	ENSG00000141391.14	ENSG00000075413.18
1207	ENSG00000071203.9	ENSG00000141371.13	ENSG00000231903.1
1208	ENSG00000071205.11	ENSG00000140830.9	ENSG00000229808.1
1209	ENSG00000071242.12	ENSG00000140807.7	ENSG00000118972.2
1210	ENSG00000071243.16	ENSG00000140488.16	ENSG00000136688.11
1211	ENSG00000071246.11	ENSG00000140379.8	ENSG00000230101.5
1212	ENSG00000071282.12	ENSG00000139714.12	ENSG00000285567.1
1213	ENSG00000071462.11	ENSG00000139641.12	ENSG00000241537.1
1214	ENSG00000071537.14	ENSG00000138835.22	ENSG00000178381.11
1215	ENSG00000071539.14	ENSG00000138600.10	ENSG00000235728.1
1216	ENSG00000071553.17	ENSG00000138035.15	ENSG00000248027.1

1217	ENSG00000071564.15	ENSG00000138028.16	ENSG00000243388.1
1218	ENSG00000071575.11	ENSG00000137970.7	ENSG00000254636.1
1219	ENSG00000071626.16	ENSG00000137875.4	ENSG00000248610.1
1220	ENSG00000071655.18	ENSG00000137726.17	ENSG00000274309.1
1221	ENSG00000071677.1	ENSG00000137216.19	ENSG00000235085.3
1222	ENSG00000071794.16	ENSG00000137198.9	ENSG00000259920.1
1223	ENSG00000071859.15	ENSG00000137101.12	ENSG00000132522.16
1224	ENSG00000071889.16	ENSG00000137094.14	ENSG00000255434.1
1225	ENSG00000071894.17	ENSG00000137055.15	ENSG00000216718.7
1226	ENSG00000071909.18	ENSG00000136869.15	ENSG00000258926.1
1227	ENSG00000071967.12	ENSG00000136856.17	ENSG00000227709.4
1228	ENSG00000071991.8	ENSG00000136840.19	ENSG00000230160.1
1229	ENSG00000071994.10	ENSG00000136783.10	ENSG00000267708.1
1230	ENSG00000072041.17	ENSG00000136754.17	ENSG00000099810.20
1231	ENSG00000072042.13	ENSG00000136634.6	ENSG00000275248.1
1232	ENSG00000072062.13	ENSG00000136521.13	ENSG00000279775.1
1233	ENSG00000072071.16	ENSG00000136379.12	ENSG00000232727.2
1234	ENSG00000072080.11	ENSG00000136280.16	ENSG00000213180.4
1235	ENSG00000072110.13	ENSG00000136231.14	ENSG00000259792.1
1236	ENSG00000072121.16	ENSG00000136014.12	ENSG00000164961.16
1237	ENSG00000072133.11	ENSG00000135953.11	ENSG00000276961.1
1238	ENSG00000072134.15	ENSG00000135801.9	ENSG00000248568.1
1239	ENSG00000072135.13	ENSG00000135503.13	ENSG00000198283.2
1240	ENSG00000072163.19	ENSG00000135486.17	ENSG00000264187.1
1241	ENSG00000072182.12	ENSG00000135407.10	ENSG00000237011.1
1242	ENSG00000072195.15	ENSG00000135336.14	ENSG00000117592.9
1243	ENSG00000072201.13	ENSG00000135298.14	ENSG00000242159.1
1244	ENSG00000072210.18	ENSG00000135069.14	ENSG00000263873.1
1245	ENSG00000072274.13	ENSG00000135052.16	ENSG00000272305.5
1246	ENSG00000072310.16	ENSG00000134627.12	ENSG00000224070.1
1247	ENSG00000072315.3	ENSG00000134594.5	ENSG00000242140.1
1248	ENSG00000072364.13	ENSG00000134548.11	ENSG00000151812.14
1249	ENSG00000072401.15	ENSG00000134545.13	ENSG00000263946.1
1250	ENSG00000072415.8	ENSG00000134504.13	ENSG00000187244.11
1251	ENSG00000072422.17	ENSG00000134480.15	ENSG00000285859.1
1252	ENSG00000072501.17	ENSG00000134287.10	ENSG00000242087.2
1253	ENSG00000072506.12	ENSG00000134285.11	ENSG00000283283.2
1254	ENSG00000072518.20	ENSG00000134247.10	ENSG00000284118.1
1255	ENSG00000072571.20	ENSG00000134215.16	ENSG00000068078.18
1256	ENSG00000072609.17	ENSG00000134198.10	ENSG00000187950.8
1257	ENSG00000072657.8	ENSG00000134070.5	ENSG00000253139.1
1258	ENSG00000072682.18	ENSG00000134001.13	ENSG00000153993.13
1259	ENSG00000072694.20	ENSG00000133958.13	ENSG00000228016.1
1260	ENSG00000072736.19	ENSG00000133874.2	ENSG00000259196.1
1261	ENSG00000072756.17	ENSG00000133639.5	ENSG00000112029.10
1262	ENSG00000072778.20	ENSG00000133606.11	ENSG00000213370.3
1263	ENSG00000072786.13	ENSG00000133069.17	ENSG00000241825.2
1264	ENSG00000072803.17	ENSG00000133048.13	ENSG00000285947.1
1265	ENSG00000072818.12	ENSG00000132965.9	ENSG00000279450.1
1266	ENSG00000072832.14	ENSG00000132963.8	ENSG00000211581.1
1267	ENSG00000072840.13	ENSG00000132881.12	ENSG00000275692.1
1268	ENSG00000072849.11	ENSG00000132832.10	ENSG00000264660.2
1269	ENSG00000072858.10	ENSG00000132825.7	ENSG00000273950.1

1270	ENSG00000072864.15	ENSG00000132646.11	ENSG00000180767.9
1271	ENSG00000072952.18	ENSG00000132549.18	ENSG00000133639.5
1272	ENSG00000072954.7	ENSG00000132386.11	ENSG00000250677.1
1273	ENSG00000072958.8	ENSG00000132256.18	ENSG00000234577.1
1274	ENSG00000073008.15	ENSG00000131981.16	ENSG00000284130.1
1275	ENSG00000073050.11	ENSG00000131979.19	ENSG00000261628.1
1276	ENSG00000073060.16	ENSG00000131969.15	ENSG00000285546.1
1277	ENSG00000073067.14	ENSG00000131504.17	ENSG00000269898.1
1278	ENSG00000073111.14	ENSG00000131400.8	ENSG00000211764.1
1279	ENSG00000073146.16	ENSG00000131370.16	ENSG00000078114.18
1280	ENSG00000073150.13	ENSG00000131355.14	ENSG00000237347.2
1281	ENSG00000073169.14	ENSG00000131351.15	ENSG00000211829.9
1282	ENSG00000073282.13	ENSG00000131148.9	ENSG00000268475.1
1283	ENSG00000073331.18	ENSG00000131115.16	ENSG00000236044.1
1284	ENSG00000073350.13	ENSG00000130940.15	ENSG00000234281.5
1285	ENSG00000073417.15	ENSG00000130561.17	ENSG00000183695.2
1286	ENSG00000073464.12	ENSG00000130518.17	ENSG00000262408.1
1287	ENSG00000073536.18	ENSG00000130332.15	ENSG00000234915.1
1288	ENSG00000073578.17	ENSG00000130270.16	ENSG00000129195.16
1289	ENSG00000073584.20	ENSG00000130165.10	ENSG00000196862.9
1290	ENSG00000073598.6	ENSG00000129933.21	ENSG00000225752.1
1291	ENSG00000073605.18	ENSG00000129467.13	ENSG00000273901.1
1292	ENSG00000073614.12	ENSG00000129465.16	ENSG00000202252.1
1293	ENSG00000073670.14	ENSG00000129422.14	ENSG00000274848.1
1294	ENSG00000073711.11	ENSG00000129116.19	ENSG00000285905.1
1295	ENSG00000073712.15	ENSG00000128965.13	ENSG00000058673.16
1296	ENSG00000073734.9	ENSG00000128656.14	ENSG00000204949.8
1297	ENSG00000073737.16	ENSG00000128654.14	ENSG00000112936.18
1298	ENSG00000073754.6	ENSG00000128595.17	ENSG00000286043.1
1299	ENSG00000073756.12	ENSG00000128581.15	ENSG00000224121.1
1300	ENSG00000073792.15	ENSG00000128272.14	ENSG00000251400.1
1301	ENSG00000073803.14	ENSG00000127870.17	ENSG00000278586.1
1302	ENSG00000073849.15	ENSG00000127364.3	ENSG00000180909.2
1303	ENSG00000073861.3	ENSG00000127334.10	ENSG00000255319.5
1304	ENSG00000073905.8	ENSG00000126432.14	ENSG00000242860.3
1305	ENSG00000073910.21	ENSG00000125843.11	ENSG00000273736.1
1306	ENSG00000073921.18	ENSG00000125551.18	ENSG00000276261.1
1307	ENSG00000073969.18	ENSG00000125355.15	ENSG00000168785.8
1308	ENSG00000074047.21	ENSG00000125266.7	ENSG00000179412.10
1309	ENSG00000074054.18	ENSG00000125122.15	ENSG00000252755.1
1310	ENSG00000074071.14	ENSG00000125046.14	ENSG00000226562.3
1311	ENSG00000074181.9	ENSG00000124831.19	ENSG00000244593.1
1312	ENSG00000074201.8	ENSG00000124615.20	ENSG00000268292.1
1313	ENSG00000074211.14	ENSG00000124613.8	ENSG00000257284.1
1314	ENSG00000074219.13	ENSG00000124334.17	ENSG00000226915.1
1315	ENSG00000074266.20	ENSG00000124302.13	ENSG00000148702.15
1316	ENSG00000074276.10	ENSG00000124275.14	ENSG00000279124.1
1317	ENSG00000074317.11	ENSG00000124172.10	ENSG00000163412.13
1318	ENSG00000074319.13	ENSG00000123728.10	ENSG00000282121.1
1319	ENSG00000074356.17	ENSG00000123444.14	ENSG00000123219.13
1320	ENSG00000074370.18	ENSG00000123191.14	ENSG00000260230.3
1321	ENSG00000074410.14	ENSG00000123143.12	ENSG00000243777.1
1322	ENSG00000074416.14	ENSG00000122386.10	ENSG00000269237.1

1323	ENSG00000074527.12	ENSG00000121898.13	ENSG00000163507.14
1324	ENSG00000074582.14	ENSG00000121743.4	ENSG00000261524.1
1325	ENSG00000074590.13	ENSG00000121716.20	ENSG00000241570.8
1326	ENSG00000074603.19	ENSG00000121413.12	ENSG00000225099.1
1327	ENSG00000074621.14	ENSG00000120949.15	ENSG00000280397.1
1328	ENSG00000074657.13	ENSG00000120437.8	ENSG00000204815.10
1329	ENSG00000074660.16	ENSG00000120334.15	ENSG00000254338.1
1330	ENSG00000074695.6	ENSG00000120215.9	ENSG00000270797.1
1331	ENSG00000074696.13	ENSG00000120162.10	ENSG00000280953.2
1332	ENSG00000074706.13	ENSG00000119979.18	ENSG00000265342.1
1333	ENSG00000074755.15	ENSG00000119946.11	ENSG00000223393.1
1334	ENSG00000074771.3	ENSG00000119900.9	ENSG00000184678.10
1335	ENSG00000074800.15	ENSG00000119681.12	ENSG00000165730.16
1336	ENSG00000074803.18	ENSG00000118965.14	ENSG00000221044.2
1337	ENSG00000074842.7	ENSG00000117592.9	ENSG00000221859.2
1338	ENSG00000074855.11	ENSG00000117481.10	ENSG00000261630.1
1339	ENSG00000074935.14	ENSG00000117226.12	ENSG00000203814.6
1340	ENSG00000074964.17	ENSG00000117153.16	ENSG00000238285.1
1341	ENSG00000074966.11	ENSG00000117115.13	ENSG00000237911.2
1342	ENSG00000075035.10	ENSG00000116962.15	ENSG00000271286.1
1343	ENSG00000075043.18	ENSG00000116824.5	ENSG00000136732.16
1344	ENSG00000075073.14	ENSG00000116791.14	ENSG00000232382.2
1345	ENSG00000075089.9	ENSG00000116783.15	ENSG00000224689.9
1346	ENSG00000075131.10	ENSG00000116761.11	ENSG00000270929.1
1347	ENSG00000075142.13	ENSG00000116750.13	ENSG00000234005.3
1348	ENSG00000075151.20	ENSG00000116675.16	ENSG00000226161.1
1349	ENSG00000075188.9	ENSG00000116525.14	ENSG00000263271.1
1350	ENSG00000075213.11	ENSG00000116497.18	ENSG00000139574.8
1351	ENSG00000075218.19	ENSG00000116176.6	ENSG00000278139.1
1352	ENSG00000075223.14	ENSG00000116014.10	ENSG00000099326.8
1353	ENSG00000075234.17	ENSG00000115816.15	ENSG00000211974.3
1354	ENSG00000075239.13	ENSG00000115649.16	ENSG00000223714.1
1355	ENSG00000075240.17	ENSG00000115596.4	ENSG00000280739.2
1356	ENSG00000075275.16	ENSG00000115524.16	ENSG00000224950.2
1357	ENSG00000075290.7	ENSG00000115504.14	ENSG00000202217.1
1358	ENSG00000075292.19	ENSG00000115486.12	ENSG00000232653.8
1359	ENSG00000075303.13	ENSG00000115459.18	ENSG00000187164.20
1360	ENSG00000075336.12	ENSG00000115414.19	ENSG00000272574.1
1361	ENSG00000075340.23	ENSG00000115363.14	ENSG00000136098.17
1362	ENSG00000075388.4	ENSG00000115339.13	ENSG00000207171.1
1363	ENSG00000075391.16	ENSG00000114670.14	ENSG00000269416.5
1364	ENSG00000075399.14	ENSG00000114626.18	ENSG00000252311.1
1365	ENSG00000075407.18	ENSG00000114395.10	ENSG00000205015.1
1366	ENSG00000075413.18	ENSG00000114378.17	ENSG00000162852.14
1367	ENSG00000075415.12	ENSG00000114107.9	ENSG00000264365.1
1368	ENSG00000075420.13	ENSG00000113758.13	ENSG00000089250.19
1369	ENSG00000075426.12	ENSG00000113369.9	ENSG00000134748.13
1370	ENSG00000075429.8	ENSG00000113356.12	ENSG00000285953.1
1371	ENSG00000075461.6	ENSG00000112902.12	ENSG00000268223.5
1372	ENSG00000075539.14	ENSG00000112812.16	ENSG00000237090.1
1373	ENSG00000075568.17	ENSG00000112796.10	ENSG00000220412.1
1374	ENSG00000075618.18	ENSG00000112146.16	ENSG00000172986.12
1375	ENSG00000075624.15	ENSG00000111912.20	ENSG00000140835.9

1376	ENSG00000075643.6	ENSG00000111877.17	ENSG00000253404.1
1377	ENSG00000075651.16	ENSG00000111725.11	ENSG00000230863.3
1378	ENSG00000075673.11	ENSG00000111666.11	ENSG00000186952.15
1379	ENSG00000075702.18	ENSG00000111602.12	ENSG00000264978.2
1380	ENSG00000075711.20	ENSG00000111348.9	ENSG00000261216.1
1381	ENSG00000075785.12	ENSG00000111254.8	ENSG00000272795.1
1382	ENSG00000075790.10	ENSG00000111077.17	ENSG00000235806.1
1383	ENSG00000075826.17	ENSG00000110497.14	ENSG00000138759.19
1384	ENSG00000075856.12	ENSG00000110492.15	ENSG00000100281.14
1385	ENSG00000075884.14	ENSG00000110324.10	ENSG00000216740.2
1386	ENSG00000075886.11	ENSG00000110274.16	ENSG00000173610.12
1387	ENSG00000075891.21	ENSG00000109927.10	ENSG00000213307.4
1388	ENSG00000075914.13	ENSG00000109475.16	ENSG00000223806.7
1389	ENSG00000075945.13	ENSG00000109270.13	ENSG00000223450.1
1390	ENSG00000075975.16	ENSG00000109265.14	ENSG00000271367.1
1391	ENSG00000076003.5	ENSG00000108825.17	ENSG00000257624.1
1392	ENSG00000076043.10	ENSG00000108515.17	ENSG00000259783.5
1393	ENSG00000076053.10	ENSG00000108384.15	ENSG00000271201.1
1394	ENSG00000076067.13	ENSG00000108312.15	ENSG00000129744.2
1395	ENSG00000076108.11	ENSG00000108106.14	ENSG00000226261.1
1396	ENSG00000076201.15	ENSG00000107954.10	ENSG00000223540.1
1397	ENSG00000076242.14	ENSG00000107897.19	ENSG00000065328.16
1398	ENSG00000076248.10	ENSG00000107862.4	ENSG00000253842.1
1399	ENSG00000076258.10	ENSG00000107537.14	ENSG00000234084.1
1400	ENSG00000076321.11	ENSG00000107521.19	ENSG00000233110.1
1401	ENSG00000076344.16	ENSG00000107371.13	ENSG00000256134.1
1402	ENSG00000076351.13	ENSG00000107263.18	ENSG00000249590.7
1403	ENSG00000076356.7	ENSG00000106799.13	ENSG00000254596.1
1404	ENSG00000076382.17	ENSG00000106785.15	ENSG00000264071.2
1405	ENSG00000076513.16	ENSG00000106367.15	ENSG00000225769.1
1406	ENSG00000076554.15	ENSG00000106341.11	ENSG00000270140.1
1407	ENSG00000076555.15	ENSG00000106049.9	ENSG00000285122.1
1408	ENSG00000076604.15	ENSG00000105976.15	ENSG00000152284.5
1409	ENSG00000076641.4	ENSG00000105778.19	ENSG00000259352.1
1410	ENSG00000076650.6	ENSG00000105613.10	ENSG00000284430.1
1411	ENSG00000076662.10	ENSG00000105583.11	ENSG00000276103.1
1412	ENSG00000076685.18	ENSG00000105523.3	ENSG00000180596.7
1413	ENSG00000076706.17	ENSG00000105427.10	ENSG00000222069.1
1414	ENSG00000076716.9	ENSG00000105137.13	ENSG00000199436.1
1415	ENSG00000076770.14	ENSG00000104974.11	ENSG00000120149.9
1416	ENSG00000076826.9	ENSG00000104970.11	ENSG00000269978.1
1417	ENSG00000076864.19	ENSG00000104921.15	ENSG00000240677.1
1418	ENSG00000076924.12	ENSG00000104918.8	ENSG00000222078.1
1419	ENSG00000076928.17	ENSG00000104915.15	ENSG00000201041.1
1420	ENSG00000076944.16	ENSG00000104904.12	ENSG00000214546.3
1421	ENSG00000076984.17	ENSG00000104804.7	ENSG00000144136.11
1422	ENSG00000077009.13	ENSG00000104549.12	ENSG00000205414.1
1423	ENSG00000077044.10	ENSG00000103353.16	ENSG00000147596.3
1424	ENSG00000077063.11	ENSG00000103168.16	ENSG00000178401.16
1425	ENSG00000077080.9	ENSG00000102996.5	ENSG00000204740.11
1426	ENSG00000077092.19	ENSG00000102854.16	ENSG00000262721.1
1427	ENSG00000077097.15	ENSG00000102554.14	ENSG00000265485.6
1428	ENSG00000077147.16	ENSG00000102524.11	ENSG00000257017.8

1429	ENSG00000077150.19	ENSG00000102309.13	ENSG00000133519.12
1430	ENSG00000077152.10	ENSG00000102145.14	ENSG00000227040.4
1431	ENSG00000077157.22	ENSG00000102109.9	ENSG00000232739.1
1432	ENSG00000077232.18	ENSG00000102003.10	ENSG00000230969.2
1433	ENSG00000077235.18	ENSG00000101608.12	ENSG00000198876.13
1434	ENSG00000077238.14	ENSG00000101574.15	ENSG00000153292.16
1435	ENSG00000077254.14	ENSG00000101470.10	ENSG00000184321.1
1436	ENSG00000077264.15	ENSG00000101307.15	ENSG00000165124.18
1437	ENSG00000077274.9	ENSG00000101294.17	ENSG00000275950.1
1438	ENSG00000077279.18	ENSG00000101278.6	ENSG00000262920.5
1439	ENSG00000077312.9	ENSG00000101160.14	ENSG00000271064.1
1440	ENSG00000077327.16	ENSG00000100918.13	ENSG00000259039.2
1441	ENSG00000077348.9	ENSG00000100902.10	ENSG00000178187.7
1442	ENSG00000077380.15	ENSG00000100614.18	ENSG00000143631.10
1443	ENSG00000077420.16	ENSG00000100558.9	ENSG00000280160.1
1444	ENSG00000077454.16	ENSG00000100427.15	ENSG00000049540.17
1445	ENSG00000077458.13	ENSG00000100276.10	ENSG00000145908.12
1446	ENSG00000077463.15	ENSG00000100225.18	ENSG00000073331.18
1447	ENSG00000077498.9	ENSG00000100220.12	ENSG00000076321.11
1448	ENSG00000077514.8	ENSG00000100211.10	ENSG00000260922.2
1449	ENSG00000077522.13	ENSG00000099860.9	ENSG00000275666.1
1450	ENSG00000077549.18	ENSG00000099834.18	ENSG00000179632.10
1451	ENSG00000077585.14	ENSG00000097021.19	ENSG00000164308.16
1452	ENSG00000077616.11	ENSG00000096696.14	ENSG00000282306.1
1453	ENSG00000077684.16	ENSG00000096080.11	ENSG00000261765.1
1454	ENSG00000077713.19	ENSG00000096063.16	ENSG00000143520.6
1455	ENSG00000077721.16	ENSG00000095587.9	ENSG00000226339.1
1456	ENSG00000077782.20	ENSG00000095464.9	ENSG00000130287.14
1457	ENSG00000077800.13	ENSG00000094804.11	ENSG00000233978.1
1458	ENSG00000077935.17	ENSG00000093100.13	ENSG00000122966.16
1459	ENSG00000077942.19	ENSG00000092108.20	ENSG00000166090.8
1460	ENSG00000077943.8	ENSG00000091010.6	ENSG00000198445.4
1461	ENSG00000077984.6	ENSG00000090989.18	ENSG00000165244.7
1462	ENSG00000078018.19	ENSG00000090857.13	ENSG00000256006.1
1463	ENSG00000078043.16	ENSG00000090659.18	ENSG00000243968.2
1464	ENSG00000078053.17	ENSG00000090487.11	ENSG00000239622.1
1465	ENSG00000078061.13	ENSG00000090061.17	ENSG00000225606.1
1466	ENSG00000078070.13	ENSG00000090013.10	ENSG00000237897.2
1467	ENSG00000078081.8	ENSG00000089692.9	ENSG00000234861.2
1468	ENSG00000078098.14	ENSG00000088727.12	ENSG00000237879.1
1469	ENSG00000078124.12	ENSG00000088298.13	ENSG00000188086.13
1470	ENSG00000078140.14	ENSG00000087088.20	ENSG00000242899.1
1471	ENSG00000078142.13	ENSG00000087086.15	ENSG00000227055.1
1472	ENSG00000078177.14	ENSG00000086570.12	ENSG00000224476.3
1473	ENSG00000078237.7	ENSG00000086300.15	ENSG00000160469.16
1474	ENSG00000078246.17	ENSG00000086289.12	ENSG00000130540.14
1475	ENSG00000078269.15	ENSG00000085998.14	ENSG00000172689.2
1476	ENSG00000078295.16	ENSG00000084444.14	ENSG00000230090.5
1477	ENSG00000078304.19	ENSG00000082898.16	ENSG00000207392.1
1478	ENSG00000078319.9	ENSG00000082146.13	ENSG00000277526.4
1479	ENSG00000078328.20	ENSG00000081870.11	ENSG00000100225.18
1480	ENSG00000078369.18	ENSG00000081386.12	ENSG00000254251.1
1481	ENSG00000078399.18	ENSG00000081307.12	ENSG00000265417.1

1482	ENSG00000078401.7	ENSG00000081014.11	ENSG00000219159.4
1483	ENSG00000078403.17	ENSG00000079385.22	ENSG00000250667.1
1484	ENSG00000078487.17	ENSG00000079308.19	ENSG00000264876.1
1485	ENSG00000078549.14	ENSG00000078814.15	ENSG00000261938.1
1486	ENSG00000078579.9	ENSG00000078114.18	ENSG00000122872.8
1487	ENSG00000078589.13	ENSG00000078081.8	ENSG00000253292.1
1488	ENSG00000078596.11	ENSG00000077585.14	ENSG00000238063.2
1489	ENSG00000078618.21	ENSG00000077152.10	ENSG00000236662.1
1490	ENSG00000078668.14	ENSG00000077097.15	ENSG00000250227.1
1491	ENSG00000078674.17	ENSG00000076984.17	ENSG00000256458.1
1492	ENSG00000078687.17	ENSG00000076944.16	ENSG00000227852.1
1493	ENSG00000078699.21	ENSG00000076864.19	ENSG00000242979.1
1494	ENSG00000078725.13	ENSG00000076770.14	ENSG00000167476.10
1495	ENSG00000078747.14	ENSG00000076641.4	ENSG00000256756.1
1496	ENSG00000078795.16	ENSG00000075413.18	ENSG00000267881.1
1497	ENSG00000078804.13	ENSG00000075035.10	ENSG00000021645.18
1498	ENSG00000078808.16	ENSG00000073861.3	ENSG00000236673.5
1499	ENSG00000078814.15	ENSG00000073737.16	ENSG00000164520.11
1500	ENSG00000078898.7	ENSG00000073008.15	ENSG00000241499.1
1501	ENSG00000078900.15	ENSG00000072818.12	ENSG00000137808.12
1502	ENSG00000078902.16	ENSG00000072778.20	ENSG00000152049.6
1503	ENSG00000078967.13	ENSG00000072121.16	ENSG00000274060.1
1504	ENSG00000079101.16	ENSG00000071626.16	ENSG00000234578.1
1505	ENSG00000079102.16	ENSG00000071575.11	ENSG00000229246.1
1506	ENSG00000079112.10	ENSG00000069956.12	ENSG00000265366.6
1507	ENSG00000079134.12	ENSG00000069943.10	ENSG00000274012.1
1508	ENSG00000079150.18	ENSG00000068308.13	ENSG00000264257.1
1509	ENSG00000079156.17	ENSG00000065978.19	ENSG00000269720.2
1510	ENSG00000079215.14	ENSG00000065911.12	ENSG00000230823.3
1511	ENSG00000079246.16	ENSG00000065154.11	ENSG00000137766.17
1512	ENSG00000079257.8	ENSG00000064933.17	ENSG00000145685.14
1513	ENSG00000079263.19	ENSG00000060971.18	ENSG00000231464.1
1514	ENSG00000079277.20	ENSG00000058668.14	ENSG00000229920.2
1515	ENSG00000079308.19	ENSG00000054598.8	ENSG00000229586.2
1516	ENSG00000079313.15	ENSG00000054219.11	ENSG00000244308.2
1517	ENSG00000079332.15	ENSG00000050130.18	ENSG00000143185.4
1518	ENSG00000079335.20	ENSG00000049249.8	ENSG00000156140.10
1519	ENSG00000079337.16	ENSG00000047617.15	ENSG00000179144.5
1520	ENSG00000079385.22	ENSG00000047579.19	ENSG00000234676.1
1521	ENSG00000079387.14	ENSG00000041802.11	ENSG00000253550.1
1522	ENSG00000079393.20	ENSG00000035687.10	ENSG00000127947.16
1523	ENSG00000079432.7	ENSG00000034152.18	ENSG00000258761.1
1524	ENSG00000079435.10	ENSG00000029534.20	ENSG00000143878.9
1525	ENSG00000079459.13	ENSG00000028528.15	ENSG00000189229.10
1526	ENSG00000079462.7	ENSG00000026950.17	ENSG00000197992.7
1527	ENSG00000079482.13	ENSG00000026751.17	ENSG00000255856.2
1528	ENSG00000079557.5	ENSG00000025156.12	ENSG00000241890.1
1529	ENSG00000079616.12	ENSG00000024422.12	ENSG00000258694.1
1530	ENSG00000079689.14	ENSG00000023839.11	ENSG00000232638.1
1531	ENSG00000079691.18	ENSG00000019995.6	ENSG00000259976.3
1532	ENSG00000079739.17	ENSG00000015568.13	ENSG00000146276.11
1533	ENSG00000079785.15	ENSG00000015520.14	ENSG00000104490.18
1534	ENSG00000079805.16	ENSG00000013297.11	ENSG00000232927.1

1535	ENSG00000079819.19	ENSG00000012174.12	ENSG00000259705.1
1536	ENSG00000079841.18	ENSG00000012124.17	ENSG00000178458.5
1537	ENSG00000079931.15	ENSG00000010610.10	ENSG00000239472.3
1538	ENSG00000079950.14	ENSG00000010270.13	ENSG00000169432.15
1539	ENSG00000079974.17	ENSG00000007129.18	ENSG00000226082.2
1540	ENSG00000079999.14	ENSG00000006831.10	ENSG00000285709.1
1541	ENSG00000080007.8	ENSG00000005381.7	ENSG00000106852.15
1542	ENSG00000080031.10	ENSG00000005206.17	ENSG00000226429.2
1543	ENSG00000080166.16	ENSG00000005175.10	ENSG00000166530.8
1544	ENSG00000080189.15	ENSG00000286272.1	ENSG00000100121.13
1545	ENSG00000080200.10	ENSG00000286271.1	ENSG00000275675.1
1546	ENSG00000080224.17	ENSG00000286270.1	ENSG00000199218.1
1547	ENSG00000080293.9	ENSG00000286269.1	ENSG00000258870.1
1548	ENSG00000080298.15	ENSG00000286268.1	ENSG00000144115.16
1549	ENSG00000080345.17	ENSG00000286267.1	ENSG00000185742.6
1550	ENSG00000080371.6	ENSG00000286266.1	ENSG00000114982.18
1551	ENSG00000080493.17	ENSG00000286265.1	ENSG00000214511.3
1552	ENSG00000080503.24	ENSG00000286264.1	ENSG00000228127.2
1553	ENSG00000080511.4	ENSG00000286263.1	ENSG00000226745.2
1554	ENSG00000080546.13	ENSG00000286262.1	ENSG00000282432.1
1555	ENSG00000080561.13	ENSG00000286261.1	ENSG00000122188.12
1556	ENSG00000080572.12	ENSG00000286260.1	ENSG00000254833.1
1557	ENSG00000080573.7	ENSG00000286259.1	ENSG00000120913.23
1558	ENSG00000080603.17	ENSG00000286258.1	ENSG00000285652.1
1559	ENSG00000080608.10	ENSG00000286257.1	ENSG00000264107.1
1560	ENSG00000080618.15	ENSG00000286256.1	ENSG00000145741.15
1561	ENSG00000080644.16	ENSG00000286255.1	ENSG00000273234.1
1562	ENSG00000080709.15	ENSG00000286254.1	ENSG00000229901.1
1563	ENSG00000080802.18	ENSG00000286253.1	ENSG00000285948.1
1564	ENSG00000080815.19	ENSG00000286252.1	ENSG00000120457.12
1565	ENSG00000080819.8	ENSG00000286251.1	ENSG00000147113.17
1566	ENSG00000080822.17	ENSG00000286250.1	ENSG00000179133.13
1567	ENSG00000080823.23	ENSG00000286249.1	ENSG00000241220.1
1568	ENSG00000080824.18	ENSG00000286248.1	ENSG00000257893.2
1569	ENSG00000080839.11	ENSG00000286247.1	ENSG00000270896.1
1570	ENSG00000080845.17	ENSG00000286246.1	ENSG00000182077.11
1571	ENSG00000080854.14	ENSG00000286245.1	ENSG00000234318.1
1572	ENSG00000080910.13	ENSG00000286244.1	ENSG00000231952.3
1573	ENSG00000080947.14	ENSG00000286243.1	ENSG00000113525.10
1574	ENSG00000080986.13	ENSG00000286242.1	ENSG00000210117.1
1575	ENSG00000081014.11	ENSG00000286241.1	ENSG00000228862.4
1576	ENSG00000081019.13	ENSG00000286240.1	ENSG00000188315.8
1577	ENSG00000081026.19	ENSG00000286239.1	ENSG00000266835.5
1578	ENSG00000081041.9	ENSG00000286238.1	ENSG00000269806.1
1579	ENSG00000081051.7	ENSG00000286237.1	ENSG00000250483.1
1580	ENSG00000081052.12	ENSG00000286236.1	ENSG00000236799.1
1581	ENSG00000081059.20	ENSG00000286235.1	ENSG00000255129.5
1582	ENSG00000081087.15	ENSG00000286234.1	ENSG00000234175.1
1583	ENSG00000081138.14	ENSG00000286233.1	ENSG00000266803.1
1584	ENSG00000081148.12	ENSG00000286232.1	ENSG00000135517.8
1585	ENSG00000081154.11	ENSG00000286231.1	ENSG00000207368.1
1586	ENSG00000081177.18	ENSG00000286230.1	ENSG00000235112.1
1587	ENSG00000081181.8	ENSG00000286229.1	ENSG00000254458.1

1588	ENSG00000081189.15	ENSG00000286228.1	ENSG00000275625.1
1589	ENSG00000081237.20	ENSG00000286227.1	ENSG00000265095.1
1590	ENSG00000081248.11	ENSG00000286226.1	ENSG00000107331.17
1591	ENSG00000081277.12	ENSG00000286225.1	ENSG00000260454.1
1592	ENSG00000081307.12	ENSG00000286224.1	ENSG00000116981.3
1593	ENSG00000081320.11	ENSG00000286223.1	ENSG00000259570.1
1594	ENSG00000081377.16	ENSG00000286222.1	ENSG00000230321.1
1595	ENSG00000081386.12	ENSG00000286221.1	ENSG00000238024.1
1596	ENSG00000081479.14	ENSG00000286219.1	ENSG00000106789.13
1597	ENSG00000081665.14	ENSG00000286218.1	ENSG00000205488.9
1598	ENSG00000081692.12	ENSG00000286217.1	ENSG00000229738.1
1599	ENSG00000081721.11	ENSG00000286216.1	ENSG00000238210.3
1600	ENSG00000081760.17	ENSG00000286215.1	ENSG00000176399.4
1601	ENSG00000081791.9	ENSG00000286214.1	ENSG00000090020.11
1602	ENSG00000081800.9	ENSG00000286213.1	ENSG00000221957.8
1603	ENSG00000081803.16	ENSG00000286212.1	ENSG00000186723.4
1604	ENSG00000081818.3	ENSG00000286211.1	ENSG00000229072.1
1605	ENSG00000081842.18	ENSG00000286210.1	ENSG00000188388.10
1606	ENSG00000081853.15	ENSG00000286209.1	ENSG00000236982.1
1607	ENSG00000081870.11	ENSG00000286208.1	ENSG00000219806.1
1608	ENSG00000081913.14	ENSG00000286207.1	ENSG00000267938.1
1609	ENSG00000081923.13	ENSG00000286206.1	ENSG00000275839.1
1610	ENSG00000081985.11	ENSG00000286205.1	ENSG00000164647.9
1611	ENSG00000082014.16	ENSG00000286204.1	ENSG00000243720.1
1612	ENSG00000082068.8	ENSG00000286203.1	ENSG00000254706.2
1613	ENSG00000082074.17	ENSG00000286202.1	ENSG00000249064.1
1614	ENSG00000082126.18	ENSG00000286201.1	ENSG00000277412.1
1615	ENSG00000082146.13	ENSG00000286199.1	ENSG00000223635.1
1616	ENSG00000082153.18	ENSG00000286198.1	ENSG00000259684.1
1617	ENSG00000082175.14	ENSG00000286197.1	ENSG00000233597.3
1618	ENSG00000082196.20	ENSG00000286196.1	ENSG00000135048.14
1619	ENSG00000082212.13	ENSG00000286195.1	ENSG00000268601.1
1620	ENSG00000082213.18	ENSG00000286194.1	ENSG00000225511.7
1621	ENSG00000082258.13	ENSG00000286193.1	ENSG00000285875.1
1622	ENSG00000082269.16	ENSG00000286192.1	ENSG00000254864.1
1623	ENSG00000082293.13	ENSG00000286191.1	ENSG00000108242.13
1624	ENSG00000082397.17	ENSG00000286190.1	ENSG00000270804.1
1625	ENSG00000082438.16	ENSG00000286189.1	ENSG00000185275.6
1626	ENSG00000082458.12	ENSG00000286188.1	ENSG00000263955.2
1627	ENSG00000082482.13	ENSG00000286187.1	ENSG00000271072.1
1628	ENSG00000082497.12	ENSG00000286186.1	ENSG00000226668.5
1629	ENSG00000082512.15	ENSG00000286185.1	ENSG00000261434.1
1630	ENSG00000082515.18	ENSG00000286183.1	ENSG00000207697.1
1631	ENSG00000082516.9	ENSG00000286182.1	ENSG00000069122.19
1632	ENSG00000082556.11	ENSG00000286181.1	ENSG00000120088.14
1633	ENSG00000082641.16	ENSG00000286180.1	ENSG00000181264.8
1634	ENSG00000082684.15	ENSG00000286179.1	ENSG00000254495.1
1635	ENSG00000082701.15	ENSG00000286178.1	ENSG00000258379.1
1636	ENSG00000082781.12	ENSG00000286177.1	ENSG00000270591.1
1637	ENSG00000082805.20	ENSG00000286176.1	ENSG00000178726.6
1638	ENSG00000082898.16	ENSG00000286175.1	ENSG00000230702.1
1639	ENSG00000082929.8	ENSG00000286174.1	ENSG00000226056.1
1640	ENSG00000082996.19	ENSG00000286173.1	ENSG00000114279.13

1641	ENSG00000083067.22	ENSG00000286172.1	ENSG00000285668.1
1642	ENSG00000083093.9	ENSG00000286171.1	ENSG00000285385.1
1643	ENSG00000083097.14	ENSG00000286170.1	ENSG00000226194.5
1644	ENSG00000083099.11	ENSG00000286169.1	ENSG00000143771.12
1645	ENSG00000083123.15	ENSG00000286168.1	ENSG00000202513.1
1646	ENSG00000083168.11	ENSG00000286167.1	ENSG00000234200.2
1647	ENSG00000083223.18	ENSG00000286165.1	ENSG00000250564.1
1648	ENSG00000083290.20	ENSG00000286164.1	ENSG00000279087.1
1649	ENSG00000083307.11	ENSG00000286163.1	ENSG00000237346.1
1650	ENSG00000083312.17	ENSG00000286162.1	ENSG00000266770.2
1651	ENSG00000083444.17	ENSG00000286161.1	ENSG00000241723.1
1652	ENSG00000083454.22	ENSG00000286160.1	ENSG00000154310.17
1653	ENSG00000083457.12	ENSG00000286159.1	ENSG00000259535.1
1654	ENSG00000083520.15	ENSG00000286158.1	ENSG00000253395.1
1655	ENSG00000083535.16	ENSG00000286157.1	ENSG00000165899.11
1656	ENSG00000083544.14	ENSG00000286156.1	ENSG00000235920.1
1657	ENSG00000083622.8	ENSG00000286155.1	ENSG00000110514.19
1658	ENSG00000083635.8	ENSG00000286154.1	ENSG00000227050.1
1659	ENSG00000083642.19	ENSG00000286153.1	ENSG00000234055.1
1660	ENSG00000083720.13	ENSG00000286152.1	ENSG00000233287.1
1661	ENSG00000083750.12	ENSG00000286151.1	ENSG00000182534.13
1662	ENSG00000083782.8	ENSG00000286150.1	ENSG00000262003.1
1663	ENSG00000083799.17	ENSG00000286149.1	ENSG00000254387.1
1664	ENSG00000083807.10	ENSG00000286148.1	ENSG00000259723.1
1665	ENSG00000083812.12	ENSG00000286147.1	ENSG00000044524.11
1666	ENSG00000083814.13	ENSG00000286146.1	ENSG00000227959.1
1667	ENSG00000083817.9	ENSG00000286145.1	ENSG00000277984.1
1668	ENSG00000083828.16	ENSG00000286144.1	ENSG00000215367.10
1669	ENSG00000083838.15	ENSG00000286143.1	ENSG00000267373.1
1670	ENSG00000083844.10	ENSG00000286142.1	ENSG00000188280.11
1671	ENSG00000083845.9	ENSG00000286141.1	ENSG00000254057.1
1672	ENSG00000083857.14	ENSG00000286140.1	ENSG00000277310.1
1673	ENSG00000083896.12	ENSG00000286138.1	ENSG00000007341.19
1674	ENSG00000083937.9	ENSG00000286137.1	ENSG00000258473.1
1675	ENSG00000084070.12	ENSG00000286136.1	ENSG00000085514.16
1676	ENSG00000084072.16	ENSG00000286135.1	ENSG00000206129.4
1677	ENSG00000084073.9	ENSG00000286134.1	ENSG00000249948.6
1678	ENSG00000084090.13	ENSG00000286133.1	ENSG00000272622.1
1679	ENSG00000084092.7	ENSG00000286132.1	ENSG00000142552.8
1680	ENSG00000084093.17	ENSG00000286131.1	ENSG00000131910.5
1681	ENSG00000084110.11	ENSG00000286130.1	ENSG00000186684.12
1682	ENSG00000084112.15	ENSG00000286129.1	ENSG00000236572.1
1683	ENSG00000084207.16	ENSG00000286128.1	ENSG00000260046.1
1684	ENSG00000084234.17	ENSG00000286127.1	ENSG00000145147.20
1685	ENSG00000084444.14	ENSG00000286126.1	ENSG00000224777.3
1686	ENSG00000084453.16	ENSG00000286125.1	ENSG00000168434.13
1687	ENSG00000084463.8	ENSG00000286124.1	ENSG00000168329.13
1688	ENSG00000084623.11	ENSG00000286123.1	ENSG00000275345.1
1689	ENSG00000084628.10	ENSG00000286121.1	ENSG00000225868.7
1690	ENSG00000084636.18	ENSG00000286120.1	ENSG00000227188.1
1691	ENSG00000084652.16	ENSG00000286119.1	ENSG00000133048.13
1692	ENSG00000084674.14	ENSG00000286118.1	ENSG00000260737.5
1693	ENSG00000084676.15	ENSG00000286117.1	ENSG00000272485.1

1694	ENSG00000084693.16	ENSG00000286116.1	ENSG00000004846.16
1695	ENSG00000084710.14	ENSG00000286115.1	ENSG00000147041.12
1696	ENSG00000084731.15	ENSG00000286114.1	ENSG00000273172.1
1697	ENSG00000084733.11	ENSG00000286113.1	ENSG00000240975.1
1698	ENSG00000084734.9	ENSG00000286112.1	ENSG00000233653.3
1699	ENSG00000084754.12	ENSG00000286111.1	ENSG00000181626.11
1700	ENSG00000084764.11	ENSG00000286110.1	ENSG00000156802.13
1701	ENSG00000084774.14	ENSG00000286109.1	ENSG00000203805.10
1702	ENSG00000085063.16	ENSG00000286108.1	ENSG00000163873.10
1703	ENSG00000085117.12	ENSG00000286107.1	ENSG00000278530.4
1704	ENSG00000085185.15	ENSG00000286106.1	ENSG00000259680.5
1705	ENSG00000085224.22	ENSG00000286105.1	ENSG00000276746.1
1706	ENSG00000085231.14	ENSG00000286104.1	ENSG00000241912.1
1707	ENSG00000085265.11	ENSG00000286103.1	ENSG00000277467.1
1708	ENSG00000085274.15	ENSG00000286102.1	ENSG00000271185.1
1709	ENSG00000085276.18	ENSG00000286101.1	ENSG00000167325.15
1710	ENSG00000085365.18	ENSG00000286100.1	ENSG00000112238.11
1711	ENSG00000085377.14	ENSG00000286099.1	ENSG00000135902.10
1712	ENSG00000085382.12	ENSG00000286098.1	ENSG00000273014.1
1713	ENSG00000085415.16	ENSG00000286097.1	ENSG00000204071.10
1714	ENSG00000085433.15	ENSG00000286096.1	ENSG00000275374.1
1715	ENSG00000085449.15	ENSG00000286095.1	ENSG00000284823.1
1716	ENSG00000085465.12	ENSG00000286094.1	ENSG00000271005.1
1717	ENSG00000085491.17	ENSG00000286093.1	ENSG00000264554.2
1718	ENSG00000085511.20	ENSG00000286092.1	ENSG00000269637.1
1719	ENSG00000085514.16	ENSG00000286091.1	ENSG00000168615.12
1720	ENSG00000085552.17	ENSG00000286090.1	ENSG00000285323.1
1721	ENSG00000085563.14	ENSG00000286089.1	ENSG00000142273.12
1722	ENSG00000085644.13	ENSG00000286088.1	ENSG00000241179.1
1723	ENSG00000085662.14	ENSG00000286087.1	ENSG00000203436.2
1724	ENSG00000085719.13	ENSG00000286086.1	ENSG00000132975.8
1725	ENSG00000085721.12	ENSG00000286085.1	ENSG00000183918.16
1726	ENSG00000085733.16	ENSG00000286084.1	ENSG00000201302.1
1727	ENSG00000085741.13	ENSG00000286083.1	ENSG00000248717.1
1728	ENSG00000085760.14	ENSG00000286082.1	ENSG00000196747.4
1729	ENSG00000085788.13	ENSG00000286081.1	ENSG00000203908.4
1730	ENSG00000085831.15	ENSG00000286080.1	ENSG00000274357.1
1731	ENSG00000085832.17	ENSG00000286079.1	ENSG00000248792.1
1732	ENSG00000085840.13	ENSG00000286078.1	ENSG00000120645.11
1733	ENSG00000085871.9	ENSG00000286077.1	ENSG00000271086.6
1734	ENSG00000085872.15	ENSG00000286076.1	ENSG00000267593.1
1735	ENSG00000085978.22	ENSG00000286074.1	ENSG00000240138.1
1736	ENSG00000085982.13	ENSG00000286073.1	ENSG00000229497.1
1737	ENSG00000085998.14	ENSG00000286072.1	ENSG00000227409.1
1738	ENSG00000085999.12	ENSG00000286071.1	ENSG00000225876.1
1739	ENSG00000086015.21	ENSG00000286070.1	ENSG00000189056.14
1740	ENSG00000086061.16	ENSG00000286069.1	ENSG00000236577.1
1741	ENSG00000086062.12	ENSG00000286068.1	ENSG00000237621.3
1742	ENSG00000086065.14	ENSG00000286067.1	ENSG00000267924.1
1743	ENSG00000086102.19	ENSG00000286066.1	ENSG00000280025.1
1744	ENSG00000086159.13	ENSG00000286065.1	ENSG00000240160.3
1745	ENSG00000086189.10	ENSG00000286064.1	ENSG00000255186.1
1746	ENSG00000086200.16	ENSG00000286063.1	ENSG00000163635.18

1747	ENSG00000086205.18	ENSG00000286062.1	ENSG00000244294.3
1748	ENSG00000086232.13	ENSG00000286061.1	ENSG00000223027.1
1749	ENSG00000086288.11	ENSG00000286060.1	ENSG00000283453.2
1750	ENSG00000086289.12	ENSG00000286059.1	ENSG00000251538.6
1751	ENSG00000086300.15	ENSG00000286058.1	ENSG00000236969.2
1752	ENSG00000086475.15	ENSG00000286057.1	ENSG00000135077.9
1753	ENSG00000086504.16	ENSG00000286056.1	ENSG00000197353.3
1754	ENSG00000086506.3	ENSG00000286055.1	ENSG00000041802.11
1755	ENSG00000086544.3	ENSG00000286054.1	ENSG00000183597.15
1756	ENSG00000086548.9	ENSG00000286053.1	ENSG00000248242.1
1757	ENSG00000086570.12	ENSG00000286052.1	ENSG00000279036.1
1758	ENSG00000086589.12	ENSG00000286051.1	ENSG00000274447.1
1759	ENSG00000086598.11	ENSG00000286050.1	ENSG00000170776.21
1760	ENSG00000086619.13	ENSG00000286049.1	ENSG00000235387.4
1761	ENSG00000086666.19	ENSG00000286048.1	ENSG00000259473.1
1762	ENSG00000086696.11	ENSG00000286047.1	ENSG00000233367.1
1763	ENSG00000086712.13	ENSG00000286046.1	ENSG00000263683.1
1764	ENSG00000086717.18	ENSG00000286045.1	ENSG00000236209.1
1765	ENSG00000086730.17	ENSG00000286044.1	ENSG00000244345.1
1766	ENSG00000086758.16	ENSG00000286043.1	ENSG00000271142.1
1767	ENSG00000086827.9	ENSG00000286042.1	ENSG00000274308.1
1768	ENSG00000086848.15	ENSG00000286041.1	ENSG00000230197.6
1769	ENSG00000086967.10	ENSG00000286040.1	ENSG00000260596.5
1770	ENSG00000086991.13	ENSG00000286039.1	ENSG00000176381.6
1771	ENSG00000087008.16	ENSG00000286038.1	ENSG00000226476.3
1772	ENSG00000087053.18	ENSG00000286037.1	ENSG00000213328.3
1773	ENSG00000087074.8	ENSG00000286036.1	ENSG00000245059.2
1774	ENSG00000087076.9	ENSG00000286035.1	ENSG00000105792.19
1775	ENSG00000087077.14	ENSG00000286034.1	ENSG00000247131.5
1776	ENSG00000087085.15	ENSG00000286033.1	ENSG00000263369.6
1777	ENSG00000087086.15	ENSG00000286032.1	ENSG00000282988.2
1778	ENSG00000087087.20	ENSG00000286031.1	ENSG00000048540.15
1779	ENSG00000087088.20	ENSG00000286030.1	ENSG00000257159.1
1780	ENSG00000087095.13	ENSG00000286029.1	ENSG00000279965.1
1781	ENSG00000087111.21	ENSG00000286028.1	ENSG00000248150.1
1782	ENSG00000087116.16	ENSG00000286027.1	ENSG00000054611.14
1783	ENSG00000087128.10	ENSG00000286026.1	ENSG00000229498.1
1784	ENSG00000087152.15	ENSG00000286025.1	ENSG00000219088.1
1785	ENSG00000087157.19	ENSG00000286024.1	ENSG00000130164.13
1786	ENSG00000087191.13	ENSG00000286023.1	ENSG00000258948.2
1787	ENSG00000087206.17	ENSG00000286022.1	ENSG00000279507.1
1788	ENSG00000087237.12	ENSG00000286021.1	ENSG00000179399.15
1789	ENSG00000087245.13	ENSG00000286020.1	ENSG00000229569.1
1790	ENSG00000087250.8	ENSG00000286019.1	ENSG00000128617.2
1791	ENSG00000087253.13	ENSG00000286018.1	ENSG00000280032.1
1792	ENSG00000087258.15	ENSG00000286017.1	ENSG00000168938.5
1793	ENSG00000087263.16	ENSG00000286016.1	ENSG00000124098.10
1794	ENSG00000087266.16	ENSG00000286015.1	ENSG00000187918.5
1795	ENSG00000087269.16	ENSG00000286014.1	ENSG00000187955.12
1796	ENSG00000087274.17	ENSG00000286013.1	ENSG00000228316.1
1797	ENSG00000087299.12	ENSG00000286012.1	ENSG00000198221.8
1798	ENSG00000087301.9	ENSG00000286011.1	ENSG00000198774.5
1799	ENSG00000087302.9	ENSG00000286010.1	ENSG00000240440.1

1800	ENSG00000087303.18	ENSG00000286008.1	ENSG00000262154.1
1801	ENSG00000087338.5	ENSG00000286007.1	ENSG00000212195.2
1802	ENSG00000087365.15	ENSG00000286006.1	ENSG00000179152.20
1803	ENSG00000087448.11	ENSG00000286005.1	ENSG00000270528.1
1804	ENSG00000087460.24	ENSG00000286004.1	ENSG00000249341.1
1805	ENSG00000087470.17	ENSG00000286003.1	ENSG00000223787.2
1806	ENSG00000087494.15	ENSG00000286002.1	ENSG00000213262.3
1807	ENSG00000087495.17	ENSG00000286001.1	ENSG00000231165.3
1808	ENSG00000087502.18	ENSG00000286000.1	ENSG00000237787.5
1809	ENSG00000087510.7	ENSG00000285999.1	ENSG00000242889.3
1810	ENSG00000087586.17	ENSG00000285998.1	ENSG00000179270.7
1811	ENSG00000087589.16	ENSG00000285997.1	ENSG00000158156.7
1812	ENSG00000087842.11	ENSG00000285996.1	ENSG00000169994.18
1813	ENSG00000087884.14	ENSG00000285993.1	ENSG00000286054.1
1814	ENSG00000087903.13	ENSG00000285992.1	ENSG00000228992.2
1815	ENSG00000087995.15	ENSG00000285991.1	ENSG00000124212.6
1816	ENSG00000088002.11	ENSG00000285990.1	ENSG00000123983.14
1817	ENSG00000088035.17	ENSG00000285989.1	ENSG00000250584.2
1818	ENSG00000088038.19	ENSG00000285988.1	ENSG00000273394.1
1819	ENSG00000088053.11	ENSG00000285987.1	ENSG00000244101.1
1820	ENSG00000088179.9	ENSG00000285986.1	ENSG00000254616.1
1821	ENSG00000088205.13	ENSG00000285985.1	ENSG00000258375.2
1822	ENSG00000088247.17	ENSG00000285984.1	ENSG00000189050.16
1823	ENSG00000088256.9	ENSG00000285982.1	ENSG00000226890.1
1824	ENSG00000088280.19	ENSG00000285981.1	ENSG00000262899.1
1825	ENSG00000088298.13	ENSG00000285980.1	ENSG00000026751.17
1826	ENSG00000088305.18	ENSG00000285979.1	ENSG00000202360.1
1827	ENSG00000088320.4	ENSG00000285978.1	ENSG00000226358.1
1828	ENSG00000088325.16	ENSG00000285977.1	ENSG00000239504.3
1829	ENSG00000088340.16	ENSG00000285976.1	ENSG00000225991.1
1830	ENSG00000088356.6	ENSG00000285975.1	ENSG00000087086.15
1831	ENSG00000088367.22	ENSG00000285974.1	ENSG00000229190.1
1832	ENSG00000088386.17	ENSG00000285973.1	ENSG00000143891.17
1833	ENSG00000088387.19	ENSG00000285972.1	ENSG00000244485.1
1834	ENSG00000088448.14	ENSG00000285971.1	ENSG00000259521.1
1835	ENSG00000088451.11	ENSG00000285970.1	ENSG00000284479.1
1836	ENSG00000088538.13	ENSG00000285969.1	ENSG00000180089.5
1837	ENSG00000088543.15	ENSG00000285968.1	ENSG00000286037.1
1838	ENSG00000088682.14	ENSG00000285967.1	ENSG00000282390.1
1839	ENSG00000088726.16	ENSG00000285966.1	ENSG00000134215.16
1840	ENSG00000088727.12	ENSG00000285964.1	ENSG00000265939.1
1841	ENSG00000088756.12	ENSG00000285963.1	ENSG00000249574.1
1842	ENSG00000088766.11	ENSG00000285961.1	ENSG00000121335.12
1843	ENSG00000088782.4	ENSG00000285960.1	ENSG00000165304.8
1844	ENSG00000088808.17	ENSG00000285959.1	ENSG00000255381.1
1845	ENSG00000088812.18	ENSG00000285958.1	ENSG00000267519.6
1846	ENSG00000088826.18	ENSG00000285957.1	ENSG00000272094.1
1847	ENSG00000088827.12	ENSG00000285956.1	ENSG00000270761.1
1848	ENSG00000088832.17	ENSG00000285955.1	ENSG00000267659.5
1849	ENSG00000088833.17	ENSG00000285954.1	ENSG00000250015.1
1850	ENSG00000088836.13	ENSG00000285953.1	ENSG00000279490.1
1851	ENSG00000088854.12	ENSG00000285952.1	ENSG00000252149.1
1852	ENSG00000088876.11	ENSG00000285950.1	ENSG00000235146.2

1853	ENSG00000088881.20	ENSG00000285948.1	ENSG00000198742.9
1854	ENSG00000088882.8	ENSG00000285947.1	ENSG00000224967.1
1855	ENSG00000088888.18	ENSG00000285946.1	ENSG00000233610.1
1856	ENSG00000088899.15	ENSG00000285945.1	ENSG00000276693.1
1857	ENSG00000088926.13	ENSG00000285944.2	ENSG00000080644.16
1858	ENSG00000088930.8	ENSG00000285943.1	ENSG00000234274.1
1859	ENSG00000088970.16	ENSG00000285942.1	ENSG00000090674.16
1860	ENSG00000088986.11	ENSG00000285941.1	ENSG00000176183.8
1861	ENSG00000088992.18	ENSG00000285940.1	ENSG00000274051.1
1862	ENSG00000089006.16	ENSG00000285939.2	ENSG00000270641.1
1863	ENSG00000089009.15	ENSG00000285938.1	ENSG00000003137.8
1864	ENSG00000089012.14	ENSG00000285937.1	ENSG00000280331.1
1865	ENSG00000089022.14	ENSG00000285936.1	ENSG00000113924.12
1866	ENSG00000089041.17	ENSG00000285935.1	ENSG00000275774.2
1867	ENSG00000089048.14	ENSG00000285934.1	ENSG00000223509.8
1868	ENSG00000089050.16	ENSG00000285933.1	ENSG00000231057.3
1869	ENSG00000089053.13	ENSG00000285932.1	ENSG00000274594.1
1870	ENSG00000089057.15	ENSG00000285931.1	ENSG00000264317.2
1871	ENSG00000089060.11	ENSG00000285930.1	ENSG00000174885.12
1872	ENSG00000089063.15	ENSG00000285929.1	ENSG00000278722.1
1873	ENSG00000089091.16	ENSG00000285928.1	ENSG00000198824.7
1874	ENSG00000089094.19	ENSG00000285927.1	ENSG00000157399.14
1875	ENSG00000089101.18	ENSG00000285926.1	ENSG00000251556.1
1876	ENSG00000089116.4	ENSG00000285925.1	ENSG00000250333.2
1877	ENSG00000089123.16	ENSG00000285923.1	ENSG00000232136.2
1878	ENSG00000089127.13	ENSG00000285922.1	ENSG00000273965.1
1879	ENSG00000089154.11	ENSG00000285921.1	ENSG00000223822.2
1880	ENSG00000089157.16	ENSG00000285920.1	ENSG00000255870.1
1881	ENSG00000089159.16	ENSG00000285919.1	ENSG00000277738.1
1882	ENSG00000089163.4	ENSG00000285917.1	ENSG00000229688.7
1883	ENSG00000089169.15	ENSG00000285915.1	ENSG00000223442.1
1884	ENSG00000089177.18	ENSG00000285914.1	ENSG00000262500.1
1885	ENSG00000089195.15	ENSG00000285913.1	ENSG00000274670.1
1886	ENSG00000089199.10	ENSG00000285912.1	ENSG00000163050.17
1887	ENSG00000089220.5	ENSG00000285911.1	ENSG00000274164.1
1888	ENSG00000089225.19	ENSG00000285910.1	ENSG00000131981.16
1889	ENSG00000089234.16	ENSG00000285909.1	ENSG00000283958.1
1890	ENSG00000089248.7	ENSG00000285908.1	ENSG00000166188.2
1891	ENSG00000089250.19	ENSG00000285907.1	ENSG00000189196.4
1892	ENSG00000089280.18	ENSG00000285905.1	ENSG00000237187.8
1893	ENSG00000089289.16	ENSG00000285904.1	ENSG00000267706.3
1894	ENSG00000089327.14	ENSG00000285902.1	ENSG00000200788.1
1895	ENSG00000089335.21	ENSG00000285901.1	ENSG00000249820.1
1896	ENSG00000089351.14	ENSG00000285900.1	ENSG00000249409.1
1897	ENSG00000089356.18	ENSG00000285899.1	ENSG00000162687.17
1898	ENSG00000089472.16	ENSG00000285898.1	ENSG00000270133.1
1899	ENSG00000089486.17	ENSG00000285897.1	ENSG00000154342.6
1900	ENSG00000089505.17	ENSG00000285896.1	ENSG00000272137.1
1901	ENSG00000089558.9	ENSG00000285895.1	ENSG00000239480.1
1902	ENSG00000089597.18	ENSG00000285894.1	ENSG00000170236.14
1903	ENSG00000089639.11	ENSG00000285892.1	ENSG00000257084.1
1904	ENSG00000089682.16	ENSG00000285891.1	ENSG00000157613.10
1905	ENSG00000089685.15	ENSG00000285890.1	ENSG00000160951.4

1906	ENSG00000089692.9	ENSG00000285889.1	ENSG00000235454.1
1907	ENSG00000089693.10	ENSG00000285888.1	ENSG00000253314.6
1908	ENSG00000089723.10	ENSG00000285887.1	ENSG00000197462.3
1909	ENSG00000089737.17	ENSG00000285886.1	ENSG00000266302.6
1910	ENSG00000089775.11	ENSG00000285885.1	ENSG00000164796.18
1911	ENSG00000089818.18	ENSG00000285884.1	ENSG00000168297.15
1912	ENSG00000089820.15	ENSG00000285883.1	ENSG00000119938.9
1913	ENSG00000089847.12	ENSG00000285882.1	ENSG00000258921.1
1914	ENSG00000089876.11	ENSG00000285881.1	ENSG00000271283.1
1915	ENSG00000089902.10	ENSG00000285880.1	ENSG00000240661.3
1916	ENSG00000089916.17	ENSG00000285879.1	ENSG00000236883.1
1917	ENSG00000090006.17	ENSG00000285878.1	ENSG00000222032.1
1918	ENSG00000090013.10	ENSG00000285877.1	ENSG00000229887.4
1919	ENSG00000090020.11	ENSG00000285876.1	ENSG00000136574.17
1920	ENSG00000090054.15	ENSG00000285875.1	ENSG00000214525.4
1921	ENSG00000090060.17	ENSG00000285873.1	ENSG00000203910.9
1922	ENSG00000090061.17	ENSG00000285872.1	ENSG00000099991.18
1923	ENSG00000090097.21	ENSG00000285871.1	ENSG00000255007.1
1924	ENSG00000090104.12	ENSG00000285870.1	ENSG00000165325.13
1925	ENSG00000090238.11	ENSG00000285869.1	ENSG00000000419.12
1926	ENSG00000090263.16	ENSG00000285868.1	ENSG00000069431.11
1927	ENSG00000090266.13	ENSG00000285867.1	ENSG00000145861.8
1928	ENSG00000090273.14	ENSG00000285866.1	ENSG00000171126.8
1929	ENSG00000090316.16	ENSG00000285865.1	ENSG00000258400.1
1930	ENSG00000090339.9	ENSG00000285864.1	ENSG00000221496.2
1931	ENSG00000090372.15	ENSG00000285863.1	ENSG00000253238.2
1932	ENSG00000090376.10	ENSG00000285862.1	ENSG00000139734.18
1933	ENSG00000090382.6	ENSG00000285861.1	ENSG00000230902.1
1934	ENSG00000090402.8	ENSG00000285860.1	ENSG00000270857.1
1935	ENSG00000090432.7	ENSG00000285859.1	ENSG00000244558.5
1936	ENSG00000090447.12	ENSG00000285858.1	ENSG00000264207.1
1937	ENSG00000090470.15	ENSG00000285857.1	ENSG00000090061.17
1938	ENSG00000090487.11	ENSG00000285856.1	ENSG00000226038.5
1939	ENSG00000090512.11	ENSG00000285855.1	ENSG00000273971.1
1940	ENSG00000090520.11	ENSG00000285854.1	ENSG00000273398.6
1941	ENSG00000090530.10	ENSG00000285853.1	ENSG00000170426.2
1942	ENSG00000090534.19	ENSG00000285852.1	ENSG00000259504.2
1943	ENSG00000090539.15	ENSG00000285851.1	ENSG00000117859.19
1944	ENSG00000090554.13	ENSG00000285850.1	ENSG00000267749.1
1945	ENSG00000090565.16	ENSG00000285849.1	ENSG00000266605.1
1946	ENSG00000090581.10	ENSG00000285848.1	ENSG00000213078.3
1947	ENSG00000090612.21	ENSG00000285847.1	ENSG00000145626.11
1948	ENSG00000090615.15	ENSG00000285846.1	ENSG00000112562.18
1949	ENSG00000090621.14	ENSG00000285845.1	ENSG00000239350.2
1950	ENSG00000090659.18	ENSG00000285844.1	ENSG00000233647.2
1951	ENSG00000090661.12	ENSG00000285843.1	ENSG00000241032.3
1952	ENSG00000090674.16	ENSG00000285842.1	ENSG00000244932.2
1953	ENSG00000090686.15	ENSG00000285841.1	ENSG00000231651.1
1954	ENSG00000090776.6	ENSG00000285840.1	ENSG00000258501.1
1955	ENSG00000090857.13	ENSG00000285839.1	ENSG00000257647.1
1956	ENSG00000090861.15	ENSG00000285838.1	ENSG00000254401.2
1957	ENSG00000090863.11	ENSG00000285837.1	ENSG00000159592.11
1958	ENSG00000090889.12	ENSG00000285836.1	ENSG00000205231.1

1959	ENSG00000090905.19	ENSG00000285835.1	ENSG00000126890.13
1960	ENSG00000090924.15	ENSG00000285834.1	ENSG00000113368.12
1961	ENSG00000090932.10	ENSG00000285833.1	ENSG00000101003.10
1962	ENSG00000090971.5	ENSG00000285831.1	ENSG00000257137.6
1963	ENSG00000090975.12	ENSG00000285830.1	ENSG00000154839.10
1964	ENSG00000090989.18	ENSG00000285829.1	ENSG00000146950.13
1965	ENSG00000091009.8	ENSG00000285827.1	ENSG00000261461.1
1966	ENSG00000091010.6	ENSG00000285826.1	ENSG00000255649.1
1967	ENSG00000091039.17	ENSG00000285825.1	ENSG00000269811.2
1968	ENSG00000091073.19	ENSG00000285824.1	ENSG00000234648.1
1969	ENSG00000091106.19	ENSG00000285823.1	ENSG00000223174.1
1970	ENSG00000091127.14	ENSG00000285822.1	ENSG00000115194.10
1971	ENSG00000091128.13	ENSG00000285821.1	ENSG00000235701.1
1972	ENSG00000091129.20	ENSG00000285820.1	ENSG00000254273.1
1973	ENSG00000091136.14	ENSG00000285819.1	ENSG00000269181.1
1974	ENSG00000091137.12	ENSG00000285818.1	ENSG00000169744.13
1975	ENSG00000091138.12	ENSG00000285817.1	ENSG00000159556.10
1976	ENSG00000091140.14	ENSG00000285816.1	ENSG00000218305.4
1977	ENSG00000091157.13	ENSG00000285815.1	ENSG00000284418.1
1978	ENSG00000091164.13	ENSG00000285814.1	ENSG00000278990.1
1979	ENSG00000091181.19	ENSG00000285813.1	ENSG00000228923.1
1980	ENSG00000091262.15	ENSG00000285812.1	ENSG00000235266.1
1981	ENSG00000091317.8	ENSG00000285811.1	ENSG00000276867.1
1982	ENSG00000091409.15	ENSG00000285810.1	ENSG00000256021.1
1983	ENSG00000091428.18	ENSG00000285809.1	ENSG00000237588.2
1984	ENSG00000091436.17	ENSG00000285808.1	ENSG00000163749.17
1985	ENSG00000091482.7	ENSG00000285807.1	ENSG00000244399.3
1986	ENSG00000091483.6	ENSG00000285806.1	ENSG00000227695.5
1987	ENSG00000091490.11	ENSG00000285805.1	ENSG00000274269.1
1988	ENSG00000091513.16	ENSG00000285804.1	ENSG00000275372.1
1989	ENSG00000091527.15	ENSG00000285803.1	ENSG00000213414.3
1990	ENSG00000091536.18	ENSG00000285802.1	ENSG00000213205.3
1991	ENSG00000091542.9	ENSG00000285801.1	ENSG00000189431.7
1992	ENSG00000091583.11	ENSG00000285800.1	ENSG00000234231.4
1993	ENSG00000091592.16	ENSG00000285798.1	ENSG00000239713.9
1994	ENSG00000091622.16	ENSG00000285797.1	ENSG00000214695.3
1995	ENSG00000091640.8	ENSG00000285796.1	ENSG00000254052.1
1996	ENSG00000091651.9	ENSG00000285794.1	ENSG00000231228.4
1997	ENSG00000091656.17	ENSG00000285793.1	ENSG00000216915.2
1998	ENSG00000091664.8	ENSG00000285792.1	ENSG00000171855.7
1999	ENSG00000091704.10	ENSG00000285791.1	ENSG00000185863.7
2000	ENSG00000091732.16	ENSG00000285790.1	ENSG00000184788.13
2001	ENSG00000091831.23	ENSG00000285789.1	ENSG00000236885.2
2002	ENSG00000091844.8	ENSG00000285788.1	ENSG00000256737.1
2003	ENSG00000091879.13	ENSG00000285786.1	ENSG00000258592.1
2004	ENSG00000091947.10	ENSG00000285785.1	ENSG00000244681.1
2005	ENSG00000091972.18	ENSG00000285784.1	ENSG00000220685.3
2006	ENSG00000091986.15	ENSG00000285783.1	ENSG00000274070.1
2007	ENSG00000092009.10	ENSG00000285782.1	ENSG00000148156.7
2008	ENSG00000092010.15	ENSG00000285781.1	ENSG00000272627.1
2009	ENSG00000092020.10	ENSG00000285780.1	ENSG00000229855.8
2010	ENSG00000092036.18	ENSG00000285779.1	ENSG00000101311.15
2011	ENSG00000092051.17	ENSG00000285778.1	ENSG00000122733.12

2012	ENSG00000092067.5	ENSG00000285777.1	ENSG00000163453.11
2013	ENSG00000092068.20	ENSG00000285776.1	ENSG00000167194.7
2014	ENSG00000092094.11	ENSG00000285775.1	ENSG00000204993.3
2015	ENSG00000092096.16	ENSG00000285774.1	ENSG00000197454.2
2016	ENSG00000092098.17	ENSG00000285773.1	ENSG00000204183.2
2017	ENSG00000092108.20	ENSG00000285772.1	ENSG00000261813.2
2018	ENSG00000092140.16	ENSG00000285771.1	ENSG00000222465.1
2019	ENSG00000092148.13	ENSG00000285770.1	ENSG00000232998.2
2020	ENSG00000092199.17	ENSG00000285769.1	ENSG00000228766.3
2021	ENSG00000092200.12	ENSG00000285768.1	ENSG00000261241.6
2022	ENSG00000092201.10	ENSG00000285766.1	ENSG00000232486.1
2023	ENSG00000092203.14	ENSG00000285765.1	ENSG00000241395.3
2024	ENSG00000092208.17	ENSG00000285764.1	ENSG00000233250.1
2025	ENSG00000092295.12	ENSG00000285763.1	ENSG00000182931.9
2026	ENSG00000092330.17	ENSG00000285762.1	ENSG00000205358.3
2027	ENSG00000092345.13	ENSG00000285761.1	ENSG00000233766.7
2028	ENSG00000092377.14	ENSG00000285760.1	ENSG00000249207.1
2029	ENSG00000092421.16	ENSG00000285759.1	ENSG00000259931.2
2030	ENSG00000092439.15	ENSG00000285758.1	ENSG00000173120.15
2031	ENSG00000092445.12	ENSG00000285757.1	ENSG00000173812.11
2032	ENSG00000092470.12	ENSG00000285756.1	ENSG00000279834.1
2033	ENSG00000092529.24	ENSG00000285755.1	ENSG00000235330.2
2034	ENSG00000092531.10	ENSG00000285754.1	ENSG00000274893.1
2035	ENSG00000092607.15	ENSG00000285753.1	ENSG00000259805.1
2036	ENSG00000092621.12	ENSG00000285752.1	ENSG00000143476.18
2037	ENSG00000092758.17	ENSG00000285751.1	ENSG00000164609.10
2038	ENSG00000092820.18	ENSG00000285750.1	ENSG00000259773.1
2039	ENSG00000092841.18	ENSG00000285749.1	ENSG00000270739.1
2040	ENSG00000092847.12	ENSG00000285748.1	ENSG00000182158.15
2041	ENSG00000092850.12	ENSG00000285747.1	ENSG00000285212.1
2042	ENSG00000092853.14	ENSG00000285746.1	ENSG00000230327.1
2043	ENSG00000092871.16	ENSG00000285745.1	ENSG00000278791.1
2044	ENSG00000092929.11	ENSG00000285744.1	ENSG00000255804.2
2045	ENSG00000092931.11	ENSG00000285743.1	ENSG00000277350.1
2046	ENSG00000092964.18	ENSG00000285742.1	ENSG00000232862.5
2047	ENSG00000092969.12	ENSG00000285741.1	ENSG00000233723.8
2048	ENSG00000092978.11	ENSG00000285740.1	ENSG00000226031.5
2049	ENSG00000093000.19	ENSG00000285739.1	ENSG00000240720.8
2050	ENSG00000093009.9	ENSG00000285738.1	ENSG00000219027.2
2051	ENSG00000093010.13	ENSG00000285736.1	ENSG00000286013.1
2052	ENSG00000093072.17	ENSG00000285735.1	ENSG00000264169.2
2053	ENSG00000093100.13	ENSG00000285734.1	ENSG00000270672.1
2054	ENSG00000093134.14	ENSG00000285733.1	ENSG00000236800.1
2055	ENSG00000093144.19	ENSG00000285732.1	ENSG00000263612.1
2056	ENSG00000093167.18	ENSG00000285731.1	ENSG00000171724.3
2057	ENSG00000093183.14	ENSG00000285730.1	ENSG00000101292.7
2058	ENSG00000093217.10	ENSG00000285729.1	ENSG00000285853.1
2059	ENSG00000094631.19	ENSG00000285728.1	ENSG00000259771.1
2060	ENSG00000094661.3	ENSG00000285727.1	ENSG00000197245.6
2061	ENSG00000094755.17	ENSG00000285726.1	ENSG00000258534.1
2062	ENSG00000094796.5	ENSG00000285725.1	ENSG00000227071.1
2063	ENSG00000094804.11	ENSG00000285724.1	ENSG00000131263.12
2064	ENSG00000094841.14	ENSG00000285723.1	ENSG00000229697.2

2065	ENSG00000094880.11	ENSG00000285722.1	ENSG00000279186.1
2066	ENSG00000094914.13	ENSG00000285720.1	ENSG00000168447.11
2067	ENSG00000094916.16	ENSG00000285719.1	ENSG00000277958.1
2068	ENSG00000094963.14	ENSG00000285718.1	ENSG00000224412.1
2069	ENSG00000094975.14	ENSG00000285717.1	ENSG00000100197.22
2070	ENSG00000095002.14	ENSG00000285716.1	ENSG00000078328.20
2071	ENSG00000095015.6	ENSG00000285715.1	ENSG00000141349.8
2072	ENSG00000095059.16	ENSG00000285714.1	ENSG00000261838.5
2073	ENSG00000095066.11	ENSG00000285713.1	ENSG00000283818.1
2074	ENSG00000095110.8	ENSG00000285712.1	ENSG00000237057.2
2075	ENSG00000095139.14	ENSG00000285711.1	ENSG00000250135.1
2076	ENSG00000095203.14	ENSG00000285710.1	ENSG00000234711.1
2077	ENSG00000095209.12	ENSG00000285709.1	ENSG00000176988.9
2078	ENSG00000095261.14	ENSG00000285708.1	ENSG00000170748.6
2079	ENSG00000095303.16	ENSG00000285707.1	ENSG00000261646.1
2080	ENSG00000095319.14	ENSG00000285706.1	ENSG00000104499.6
2081	ENSG00000095321.17	ENSG00000285705.1	ENSG00000250899.3
2082	ENSG00000095370.20	ENSG00000285704.1	ENSG00000134363.12
2083	ENSG00000095380.11	ENSG00000285703.1	ENSG00000250596.2
2084	ENSG00000095383.20	ENSG00000285702.1	ENSG00000227233.1
2085	ENSG00000095397.14	ENSG00000285701.1	ENSG00000225710.1
2086	ENSG00000095464.9	ENSG00000285700.1	ENSG00000056487.16
2087	ENSG00000095485.18	ENSG00000285699.1	ENSG00000237851.1
2088	ENSG00000095539.15	ENSG00000285698.1	ENSG00000235438.7
2089	ENSG00000095564.14	ENSG00000285697.1	ENSG00000170289.12
2090	ENSG00000095574.11	ENSG00000285695.1	ENSG00000172362.3
2091	ENSG00000095585.16	ENSG00000285694.1	ENSG00000257671.1
2092	ENSG00000095587.9	ENSG00000285693.1	ENSG00000272908.1
2093	ENSG00000095596.12	ENSG00000285692.1	ENSG00000135454.14
2094	ENSG00000095627.9	ENSG00000285691.1	ENSG00000252877.1
2095	ENSG00000095637.22	ENSG00000285690.1	ENSG00000272324.5
2096	ENSG00000095713.14	ENSG00000285689.1	ENSG00000278449.1
2097	ENSG00000095739.11	ENSG00000285688.1	ENSG00000273481.1
2098	ENSG00000095752.7	ENSG00000285687.1	ENSG00000135919.13
2099	ENSG00000095777.16	ENSG00000285686.1	ENSG00000266192.3
2100	ENSG00000095787.22	ENSG00000285685.1	ENSG00000124217.4
2101	ENSG00000095794.19	ENSG00000285684.1	ENSG00000284652.1
2102	ENSG00000095906.17	ENSG00000285683.1	ENSG00000274656.1
2103	ENSG00000095917.14	ENSG00000285681.1	ENSG00000204092.2
2104	ENSG00000095932.6	ENSG00000285680.1	ENSG00000271415.1
2105	ENSG00000095951.17	ENSG00000285679.1	ENSG00000243532.3
2106	ENSG00000095970.16	ENSG00000285677.1	ENSG00000224016.2
2107	ENSG00000095981.10	ENSG00000285676.1	ENSG00000240484.1
2108	ENSG00000096006.11	ENSG00000285675.1	ENSG00000225355.1
2109	ENSG00000096060.14	ENSG00000285674.1	ENSG00000202461.1
2110	ENSG00000096063.16	ENSG00000285673.1	ENSG00000173810.9
2111	ENSG00000096070.19	ENSG00000285672.1	ENSG00000123146.20
2112	ENSG00000096080.11	ENSG00000285671.1	ENSG00000179604.10
2113	ENSG00000096092.6	ENSG00000285670.1	ENSG00000240553.1
2114	ENSG00000096093.16	ENSG00000285669.1	ENSG00000242241.3
2115	ENSG00000096264.14	ENSG00000285668.1	ENSG00000230942.1
2116	ENSG00000096384.20	ENSG00000285667.1	ENSG00000189013.14
2117	ENSG00000096395.11	ENSG00000285666.1	ENSG00000270799.1

2118	ENSG00000096401.8	ENSG00000285665.1	ENSG00000237643.1
2119	ENSG00000096433.11	ENSG00000285664.1	ENSG00000180525.12
2120	ENSG00000096654.15	ENSG00000285663.1	ENSG00000237801.6
2121	ENSG00000096696.14	ENSG00000285662.1	ENSG00000235192.1
2122	ENSG00000096717.12	ENSG00000285661.1	ENSG00000143556.9
2123	ENSG00000096746.17	ENSG00000285660.1	ENSG00000243297.2
2124	ENSG00000096872.16	ENSG00000285659.1	ENSG00000269944.1
2125	ENSG00000096968.13	ENSG00000285658.1	ENSG00000241243.3
2126	ENSG00000096996.15	ENSG00000285657.1	ENSG00000281028.1
2127	ENSG00000097007.18	ENSG00000285656.1	ENSG00000234394.8
2128	ENSG00000097021.19	ENSG00000285655.1	ENSG00000107186.16
2129	ENSG00000097033.14	ENSG00000285654.1	ENSG00000225813.1
2130	ENSG00000097046.13	ENSG00000285653.1	ENSG00000269387.1
2131	ENSG00000097096.9	ENSG00000285652.1	ENSG00000260912.1
2132	ENSG00000099139.13	ENSG00000285651.1	ENSG00000259353.1
2133	ENSG00000099194.6	ENSG00000285650.1	ENSG00000252947.1
2134	ENSG00000099203.7	ENSG00000285649.1	ENSG00000162951.11
2135	ENSG00000099204.20	ENSG00000285648.1	ENSG00000226432.3
2136	ENSG00000099219.14	ENSG00000285646.1	ENSG00000131951.11
2137	ENSG00000099246.16	ENSG00000285645.1	ENSG00000277562.1
2138	ENSG00000099250.18	ENSG00000285644.1	ENSG00000250699.1
2139	ENSG00000099251.14	ENSG00000285643.1	ENSG00000198160.14
2140	ENSG00000099256.19	ENSG00000285642.1	ENSG00000100557.9
2141	ENSG00000099260.11	ENSG00000285640.1	ENSG00000253586.1
2142	ENSG00000099282.10	ENSG00000285639.1	ENSG00000272905.1
2143	ENSG00000099284.14	ENSG00000285638.1	ENSG00000066629.17
2144	ENSG00000099290.17	ENSG00000285637.1	ENSG00000154127.10
2145	ENSG00000099308.10	ENSG00000285636.1	ENSG00000187773.8
2146	ENSG00000099326.8	ENSG00000285635.1	ENSG00000243018.1
2147	ENSG00000099330.9	ENSG00000285634.1	ENSG00000237024.1
2148	ENSG00000099331.13	ENSG00000285633.1	ENSG00000254090.1
2149	ENSG00000099337.5	ENSG00000285632.1	ENSG00000235731.2
2150	ENSG00000099338.23	ENSG00000285631.1	ENSG00000275833.1
2151	ENSG00000099341.11	ENSG00000285630.1	ENSG00000171004.18
2152	ENSG00000099364.16	ENSG00000285629.1	ENSG00000135097.7
2153	ENSG00000099365.11	ENSG00000285628.1	ENSG00000167774.2
2154	ENSG00000099377.14	ENSG00000285626.1	ENSG00000224177.6
2155	ENSG00000099381.18	ENSG00000285625.1	ENSG00000198569.9
2156	ENSG00000099385.11	ENSG00000285624.1	ENSG00000162849.16
2157	ENSG00000099399.6	ENSG00000285623.1	ENSG00000225078.2
2158	ENSG00000099617.4	ENSG00000285622.1	ENSG00000274658.1
2159	ENSG00000099622.14	ENSG00000285621.1	ENSG00000183955.13
2160	ENSG00000099624.8	ENSG00000285620.1	ENSG00000213697.3
2161	ENSG00000099625.13	ENSG00000285619.1	ENSG00000176043.5
2162	ENSG00000099715.14	ENSG00000285618.1	ENSG00000278171.1
2163	ENSG00000099721.14	ENSG00000285616.1	ENSG00000260514.1
2164	ENSG00000099725.14	ENSG00000285615.1	ENSG00000160190.14
2165	ENSG00000099769.5	ENSG00000285614.1	ENSG00000165194.15
2166	ENSG00000099783.12	ENSG00000285613.1	ENSG00000234419.1
2167	ENSG00000099785.10	ENSG00000285612.1	ENSG00000228140.1
2168	ENSG00000099795.7	ENSG00000285611.1	ENSG00000021852.13
2169	ENSG00000099797.15	ENSG00000285610.1	ENSG00000264553.1
2170	ENSG00000099800.8	ENSG00000285609.1	ENSG00000241577.1

2171	ENSG00000099804.9	ENSG00000285608.1	ENSG00000266963.1
2172	ENSG00000099810.20	ENSG00000285607.1	ENSG00000271443.1
2173	ENSG00000099812.9	ENSG00000285606.1	ENSG00000189167.11
2174	ENSG00000099814.16	ENSG00000285605.1	ENSG00000263715.7
2175	ENSG00000099817.12	ENSG00000285604.1	ENSG00000215765.3
2176	ENSG00000099821.14	ENSG00000285603.1	ENSG00000197771.12
2177	ENSG00000099822.3	ENSG00000285602.1	ENSG00000177047.6
2178	ENSG00000099834.18	ENSG00000285601.1	ENSG00000263597.1
2179	ENSG00000099840.13	ENSG00000285600.1	ENSG00000279317.2
2180	ENSG00000099849.15	ENSG00000285599.1	ENSG00000178965.14
2181	ENSG00000099860.9	ENSG00000285598.1	ENSG00000262445.3
2182	ENSG00000099864.18	ENSG00000285597.1	ENSG00000154265.16
2183	ENSG00000099866.15	ENSG00000285596.1	ENSG00000278250.1
2184	ENSG00000099869.7	ENSG00000285595.1	ENSG00000146826.17
2185	ENSG00000099875.14	ENSG00000285594.1	ENSG00000267461.1
2186	ENSG00000099889.14	ENSG00000285593.1	ENSG00000084070.12
2187	ENSG00000099899.15	ENSG00000285592.1	ENSG00000261775.1
2188	ENSG00000099901.17	ENSG00000285591.1	ENSG00000213058.3
2189	ENSG00000099904.15	ENSG00000285590.1	ENSG00000239985.2
2190	ENSG00000099910.17	ENSG00000285589.1	ENSG00000082146.13
2191	ENSG00000099917.17	ENSG00000285588.1	ENSG00000254551.1
2192	ENSG00000099937.11	ENSG00000285587.1	ENSG00000261803.1
2193	ENSG00000099940.12	ENSG00000285586.1	ENSG00000279513.1
2194	ENSG00000099942.13	ENSG00000285585.1	ENSG00000109705.7
2195	ENSG00000099949.20	ENSG00000285584.1	ENSG00000163612.10
2196	ENSG00000099953.10	ENSG00000285583.1	ENSG00000271707.1
2197	ENSG00000099954.18	ENSG00000285582.1	ENSG00000253983.2
2198	ENSG00000099956.19	ENSG00000285581.1	ENSG00000146122.17
2199	ENSG00000099957.16	ENSG00000285580.1	ENSG00000165804.16
2200	ENSG00000099958.14	ENSG00000285579.1	ENSG00000102109.9
2201	ENSG00000099960.13	ENSG00000285578.1	ENSG00000279400.1
2202	ENSG00000099968.17	ENSG00000285577.1	ENSG00000249920.2
2203	ENSG00000099974.8	ENSG00000285576.1	ENSG00000269615.1
2204	ENSG00000099977.14	ENSG00000285575.1	ENSG00000254751.3
2205	ENSG00000099984.11	ENSG00000285574.1	ENSG00000172789.3
2206	ENSG00000099985.4	ENSG00000285573.1	ENSG00000229424.1
2207	ENSG00000099991.18	ENSG00000285572.1	ENSG00000262115.1
2208	ENSG00000099992.15	ENSG00000285571.1	ENSG00000220154.2
2209	ENSG00000099994.11	ENSG00000285570.1	ENSG00000229221.1
2210	ENSG00000099995.19	ENSG00000285569.1	ENSG00000261603.2
2211	ENSG00000099998.17	ENSG00000285568.1	ENSG00000270764.1
2212	ENSG00000099999.14	ENSG00000285567.1	ENSG00000154162.14
2213	ENSG00000100003.18	ENSG00000285566.1	ENSG00000268333.1
2214	ENSG00000100012.11	ENSG00000285565.1	ENSG00000286122.1
2215	ENSG00000100014.20	ENSG00000285564.1	ENSG00000217896.2
2216	ENSG00000100023.19	ENSG00000285563.1	ENSG00000084764.11
2217	ENSG00000100024.14	ENSG00000285562.1	ENSG00000285644.1
2218	ENSG00000100027.15	ENSG00000285561.1	ENSG00000117226.12
2219	ENSG00000100028.12	ENSG00000285560.1	ENSG00000207304.1
2220	ENSG00000100029.18	ENSG00000285559.1	ENSG00000166947.13
2221	ENSG00000100030.14	ENSG00000285558.1	ENSG00000170522.9
2222	ENSG00000100031.19	ENSG00000285557.1	ENSG00000230923.2
2223	ENSG00000100033.16	ENSG00000285556.1	ENSG00000231956.1

2224	ENSG00000100034.14	ENSG00000285555.1	ENSG00000117691.10
2225	ENSG00000100036.13	ENSG00000285554.1	ENSG00000249410.1
2226	ENSG00000100038.19	ENSG00000285553.1	ENSG00000257576.1
2227	ENSG00000100053.10	ENSG00000285552.1	ENSG00000259775.1
2228	ENSG00000100055.21	ENSG00000285551.1	ENSG00000220105.2
2229	ENSG00000100056.12	ENSG00000285550.1	ENSG00000237452.2
2230	ENSG00000100058.12	ENSG00000285549.1	ENSG00000240074.1
2231	ENSG00000100060.17	ENSG00000285548.1	ENSG00000273024.6
2232	ENSG00000100065.15	ENSG00000285547.1	ENSG00000259692.5
2233	ENSG00000100068.12	ENSG00000285546.1	ENSG00000137501.17
2234	ENSG00000100075.10	ENSG00000285545.1	ENSG00000187037.8
2235	ENSG00000100077.15	ENSG00000285544.1	ENSG00000152583.12
2236	ENSG00000100078.4	ENSG00000285543.1	ENSG00000240441.1
2237	ENSG00000100079.7	ENSG00000285542.1	ENSG00000106299.8
2238	ENSG00000100083.19	ENSG00000285541.1	ENSG00000276076.4
2239	ENSG00000100084.14	ENSG00000285540.1	ENSG00000106631.8
2240	ENSG00000100092.22	ENSG00000285539.1	ENSG00000163040.14
2241	ENSG00000100095.19	ENSG00000285538.1	ENSG00000235081.1
2242	ENSG00000100097.12	ENSG00000285537.1	ENSG00000265590.9
2243	ENSG00000100099.20	ENSG00000285536.1	ENSG00000274759.1
2244	ENSG00000100100.12	ENSG00000285535.1	ENSG00000212348.1
2245	ENSG00000100101.15	ENSG00000285534.1	ENSG00000246366.6
2246	ENSG00000100104.13	ENSG00000285533.1	ENSG00000250218.1
2247	ENSG00000100105.18	ENSG00000285532.1	ENSG00000275628.2
2248	ENSG00000100106.21	ENSG00000285531.1	ENSG00000213303.3
2249	ENSG00000100109.17	ENSG00000285530.1	ENSG00000183098.11
2250	ENSG00000100116.16	ENSG00000285529.1	ENSG00000228703.1
2251	ENSG00000100121.13	ENSG00000285528.1	ENSG00000110680.12
2252	ENSG00000100122.7	ENSG00000285527.1	ENSG00000226761.3
2253	ENSG00000100124.15	ENSG00000285526.1	ENSG00000172460.16
2254	ENSG00000100129.18	ENSG00000285525.1	ENSG00000279096.2
2255	ENSG00000100138.15	ENSG00000285524.1	ENSG00000225933.1
2256	ENSG00000100139.13	ENSG00000285523.1	ENSG00000230683.1
2257	ENSG00000100142.15	ENSG00000285522.1	ENSG00000107187.16
2258	ENSG00000100146.17	ENSG00000285521.1	ENSG00000224820.2
2259	ENSG00000100147.13	ENSG00000285520.1	ENSG00000104938.16
2260	ENSG00000100150.18	ENSG00000285519.1	ENSG00000172380.6
2261	ENSG00000100151.16	ENSG00000285518.1	ENSG00000283635.1
2262	ENSG00000100154.14	ENSG00000285517.1	ENSG00000233005.1
2263	ENSG00000100156.10	ENSG00000285513.1	ENSG00000266283.1
2264	ENSG00000100162.15	ENSG00000285509.1	ENSG00000256364.1
2265	ENSG00000100167.20	ENSG00000285508.1	ENSG00000234906.10
2266	ENSG00000100170.9	ENSG00000285505.1	ENSG00000234080.3
2267	ENSG00000100181.22	ENSG00000285498.1	ENSG00000221491.2
2268	ENSG00000100191.5	ENSG00000285492.1	ENSG00000250577.1
2269	ENSG00000100196.11	ENSG00000285491.1	ENSG00000084628.10
2270	ENSG00000100197.22	ENSG00000285486.1	ENSG00000223915.1
2271	ENSG00000100201.21	ENSG00000285483.1	ENSG00000279967.1
2272	ENSG00000100206.10	ENSG00000285480.1	ENSG00000258028.2
2273	ENSG00000100207.18	ENSG00000285476.1	ENSG00000214875.2
2274	ENSG00000100209.10	ENSG00000285473.1	ENSG00000153303.17
2275	ENSG00000100211.10	ENSG00000285472.1	ENSG00000268133.1
2276	ENSG00000100216.6	ENSG00000285471.1	ENSG00000198203.10

2277	ENSG00000100218.11	ENSG00000285470.1	ENSG00000170927.14
2278	ENSG00000100219.16	ENSG00000285467.1	ENSG00000186470.14
2279	ENSG00000100220.12	ENSG00000285462.1	ENSG00000237329.2
2280	ENSG00000100221.10	ENSG00000285458.1	ENSG00000183396.3
2281	ENSG00000100225.18	ENSG00000285454.1	ENSG00000163762.7
2282	ENSG00000100226.16	ENSG00000285447.1	ENSG00000200243.1
2283	ENSG00000100227.18	ENSG00000285446.1	ENSG00000152779.14
2284	ENSG00000100228.12	ENSG00000285445.1	ENSG00000285454.1
2285	ENSG00000100234.11	ENSG00000285444.1	ENSG00000131386.19
2286	ENSG00000100239.16	ENSG00000285441.1	ENSG00000115705.21
2287	ENSG00000100241.21	ENSG00000285437.1	ENSG00000285717.1
2288	ENSG00000100242.15	ENSG00000285427.1	ENSG00000254866.2
2289	ENSG00000100243.21	ENSG00000285424.1	ENSG00000138271.5
2290	ENSG00000100246.13	ENSG00000285420.1	ENSG00000238111.1
2291	ENSG00000100249.4	ENSG00000285417.1	ENSG00000235843.1
2292	ENSG00000100253.13	ENSG00000285416.1	ENSG00000264451.1
2293	ENSG00000100258.18	ENSG00000285413.1	ENSG00000196169.15
2294	ENSG00000100263.14	ENSG00000285412.1	ENSG00000197385.5
2295	ENSG00000100266.19	ENSG00000285410.1	ENSG00000230312.2
2296	ENSG00000100271.17	ENSG00000285409.1	ENSG00000267598.1
2297	ENSG00000100276.10	ENSG00000285407.1	ENSG00000278818.1
2298	ENSG00000100280.16	ENSG00000285405.1	ENSG00000282057.1
2299	ENSG00000100281.14	ENSG00000285404.1	ENSG00000226972.2
2300	ENSG00000100284.21	ENSG00000285402.1	ENSG00000217414.2
2301	ENSG00000100285.9	ENSG00000285401.1	ENSG00000198626.16
2302	ENSG00000100288.19	ENSG00000285399.2	ENSG00000187847.4
2303	ENSG00000100290.3	ENSG00000285398.1	ENSG00000242853.3
2304	ENSG00000100292.17	ENSG00000285388.1	ENSG00000266775.1
2305	ENSG00000100294.12	ENSG00000285385.1	ENSG00000262075.3
2306	ENSG00000100296.13	ENSG00000285382.1	ENSG00000260919.1
2307	ENSG00000100297.16	ENSG00000285374.1	ENSG00000185823.3
2308	ENSG00000100298.15	ENSG00000285373.1	ENSG00000232792.2
2309	ENSG00000100299.18	ENSG00000285370.1	ENSG00000256813.1
2310	ENSG00000100300.18	ENSG00000285367.1	ENSG00000005379.17
2311	ENSG00000100302.7	ENSG00000285366.1	ENSG00000229019.1
2312	ENSG00000100304.13	ENSG00000285361.1	ENSG00000147873.5
2313	ENSG00000100307.13	ENSG00000285354.1	ENSG00000251002.7
2314	ENSG00000100311.16	ENSG00000285347.1	ENSG00000254285.3
2315	ENSG00000100312.10	ENSG00000285338.1	ENSG00000189337.17
2316	ENSG00000100314.4	ENSG00000285336.1	ENSG00000226617.1
2317	ENSG00000100316.16	ENSG00000285331.2	ENSG00000234832.1
2318	ENSG00000100319.12	ENSG00000285330.1	ENSG00000231888.1
2319	ENSG00000100320.23	ENSG00000285329.1	ENSG00000249286.1
2320	ENSG00000100321.15	ENSG00000285325.1	ENSG00000258053.1
2321	ENSG00000100324.14	ENSG00000285323.1	ENSG00000213048.3
2322	ENSG00000100325.15	ENSG00000285314.1	ENSG00000233990.1
2323	ENSG00000100330.15	ENSG00000285304.1	ENSG00000275131.3
2324	ENSG00000100335.15	ENSG00000285303.1	ENSG00000243967.4
2325	ENSG00000100336.17	ENSG00000285300.1	ENSG00000231424.2
2326	ENSG00000100341.11	ENSG00000285294.1	ENSG00000204421.3
2327	ENSG00000100342.20	ENSG00000285292.1	ENSG00000253811.1
2328	ENSG00000100344.10	ENSG00000285287.1	ENSG00000244004.1
2329	ENSG00000100345.21	ENSG00000285283.1	ENSG00000073150.13

2330	ENSG00000100346.17	ENSG00000285280.1	ENSG00000171791.13
2331	ENSG00000100347.15	ENSG00000285278.1	ENSG00000168542.14
2332	ENSG00000100348.10	ENSG00000285269.2	ENSG00000160202.7
2333	ENSG00000100350.14	ENSG00000285268.1	ENSG00000284498.1
2334	ENSG00000100351.16	ENSG00000285258.1	ENSG00000117054.13
2335	ENSG00000100353.18	ENSG00000285257.1	ENSG00000236641.2
2336	ENSG00000100354.21	ENSG00000285254.1	ENSG00000107164.16
2337	ENSG00000100359.21	ENSG00000285253.1	ENSG00000224969.1
2338	ENSG00000100360.15	ENSG00000285245.1	ENSG00000249451.1
2339	ENSG00000100362.13	ENSG00000285244.1	ENSG00000169900.7
2340	ENSG00000100364.18	ENSG00000285239.1	ENSG00000285547.1
2341	ENSG00000100365.15	ENSG00000285238.2	ENSG00000160796.17
2342	ENSG00000100368.14	ENSG00000285237.1	ENSG00000212855.5
2343	ENSG00000100372.15	ENSG00000285231.1	ENSG00000196411.10
2344	ENSG00000100373.10	ENSG00000285230.1	ENSG00000211584.14
2345	ENSG00000100376.12	ENSG00000285228.1	ENSG00000232981.1
2346	ENSG00000100379.17	ENSG00000285220.1	ENSG00000250767.1
2347	ENSG00000100380.14	ENSG00000285219.1	ENSG00000223784.2
2348	ENSG00000100385.14	ENSG00000285218.1	ENSG00000100605.17
2349	ENSG00000100387.8	ENSG00000285216.1	ENSG00000279827.1
2350	ENSG00000100393.13	ENSG00000285215.2	ENSG00000246695.7
2351	ENSG00000100395.15	ENSG00000285212.1	ENSG00000167987.11
2352	ENSG00000100399.16	ENSG00000285210.1	ENSG00000261053.1
2353	ENSG00000100401.19	ENSG00000285205.2	ENSG00000238008.1
2354	ENSG00000100403.12	ENSG00000285204.1	ENSG00000274624.1
2355	ENSG00000100410.8	ENSG00000285201.1	ENSG00000231705.1
2356	ENSG00000100412.16	ENSG00000285191.1	ENSG00000260064.1
2357	ENSG00000100413.17	ENSG00000285190.1	ENSG00000234793.1
2358	ENSG00000100416.14	ENSG00000285188.1	ENSG00000285616.1
2359	ENSG00000100417.12	ENSG00000285184.2	ENSG00000258390.1
2360	ENSG00000100418.8	ENSG00000285179.1	ENSG00000238133.6
2361	ENSG00000100422.14	ENSG00000285177.1	ENSG00000277864.1
2362	ENSG00000100425.18	ENSG00000285171.1	ENSG00000224189.7
2363	ENSG00000100426.7	ENSG00000285169.1	ENSG00000163600.12
2364	ENSG00000100427.15	ENSG00000285165.1	ENSG00000232490.6
2365	ENSG00000100429.18	ENSG00000285163.1	ENSG00000183324.11
2366	ENSG00000100433.15	ENSG00000285162.1	ENSG00000226367.5
2367	ENSG00000100439.10	ENSG00000285160.1	ENSG00000170959.14
2368	ENSG00000100441.10	ENSG00000285159.1	ENSG00000186471.12
2369	ENSG00000100442.11	ENSG00000285155.1	ENSG00000176681.14
2370	ENSG00000100445.17	ENSG00000285151.1	ENSG00000267583.5
2371	ENSG00000100448.4	ENSG00000285144.1	ENSG00000229757.2
2372	ENSG00000100450.13	ENSG00000285142.1	ENSG00000157870.16
2373	ENSG00000100453.13	ENSG00000285135.1	ENSG00000284547.1
2374	ENSG00000100461.18	ENSG00000285133.1	ENSG00000224745.1
2375	ENSG00000100462.16	ENSG00000285130.2	ENSG00000023697.13
2376	ENSG00000100473.17	ENSG00000285128.1	ENSG00000226158.1
2377	ENSG00000100478.14	ENSG00000285122.1	ENSG00000266446.1
2378	ENSG00000100479.13	ENSG00000285117.1	ENSG00000263316.1
2379	ENSG00000100483.14	ENSG00000285116.1	ENSG00000276578.1
2380	ENSG00000100485.12	ENSG00000285115.1	ENSG00000248909.1
2381	ENSG00000100490.9	ENSG00000285108.1	ENSG00000232415.1
2382	ENSG00000100503.23	ENSG00000285103.2	ENSG00000175520.9

2383	ENSG00000100504.17	ENSG00000285102.1	ENSG00000248079.2
2384	ENSG00000100505.13	ENSG00000285100.1	ENSG00000233540.1
2385	ENSG00000100519.12	ENSG00000285095.1	ENSG00000253348.1
2386	ENSG00000100522.10	ENSG00000285094.1	ENSG00000171298.13
2387	ENSG00000100523.14	ENSG00000285091.1	ENSG00000249709.8
2388	ENSG00000100526.20	ENSG00000285090.1	ENSG00000278610.1
2389	ENSG00000100528.12	ENSG00000285085.1	ENSG00000118094.11
2390	ENSG00000100532.12	ENSG00000285082.1	ENSG00000135824.12
2391	ENSG00000100554.12	ENSG00000285081.1	ENSG00000183508.5
2392	ENSG00000100557.9	ENSG00000285080.1	ENSG00000177725.5
2393	ENSG00000100558.9	ENSG00000285079.1	ENSG00000167670.16
2394	ENSG00000100564.9	ENSG00000285077.2	ENSG00000258605.1
2395	ENSG00000100565.15	ENSG00000285076.1	ENSG00000205267.5
2396	ENSG00000100567.13	ENSG00000285068.1	ENSG00000279720.1
2397	ENSG00000100568.11	ENSG00000285064.1	ENSG00000283061.1
2398	ENSG00000100575.14	ENSG00000285062.1	ENSG00000279428.1
2399	ENSG00000100577.19	ENSG00000285057.1	ENSG00000212541.1
2400	ENSG00000100578.15	ENSG00000285053.1	ENSG00000237007.4
2401	ENSG00000100580.8	ENSG00000285051.1	ENSG00000196814.15
2402	ENSG00000100583.4	ENSG00000285045.1	ENSG00000255581.1
2403	ENSG00000100591.8	ENSG00000285043.1	ENSG00000237531.6
2404	ENSG00000100592.15	ENSG00000285042.1	ENSG00000183145.9
2405	ENSG00000100593.18	ENSG00000285041.1	ENSG00000265126.1
2406	ENSG00000100596.6	ENSG00000285040.1	ENSG00000118307.19
2407	ENSG00000100599.16	ENSG00000285035.1	ENSG00000267618.5
2408	ENSG00000100600.15	ENSG00000285030.1	ENSG00000252714.1
2409	ENSG00000100601.10	ENSG00000285025.1	ENSG00000255346.10
2410	ENSG00000100603.13	ENSG00000285020.1	ENSG00000255307.1
2411	ENSG00000100604.13	ENSG00000285018.1	ENSG00000250910.8
2412	ENSG00000100605.17	ENSG00000285016.1	ENSG00000251988.1
2413	ENSG00000100612.14	ENSG00000285012.1	ENSG00000226810.3
2414	ENSG00000100614.18	ENSG00000285010.1	ENSG00000274420.1
2415	ENSG00000100625.9	ENSG00000285000.1	ENSG00000237470.3
2416	ENSG00000100626.17	ENSG00000284999.1	ENSG00000147432.6
2417	ENSG00000100628.11	ENSG00000284994.1	ENSG00000235400.1
2418	ENSG00000100629.17	ENSG00000284989.1	ENSG00000260971.4
2419	ENSG00000100632.11	ENSG00000284988.1	ENSG00000258096.1
2420	ENSG00000100644.17	ENSG00000284986.1	ENSG00000261736.1
2421	ENSG00000100647.8	ENSG00000284985.1	ENSG00000185631.7
2422	ENSG00000100650.15	ENSG00000284981.1	ENSG00000187908.18
2423	ENSG00000100652.5	ENSG00000284977.1	ENSG00000203727.4
2424	ENSG00000100664.11	ENSG00000284976.1	ENSG00000181029.9
2425	ENSG00000100665.12	ENSG00000284969.1	ENSG00000223502.1
2426	ENSG00000100678.18	ENSG00000284968.1	ENSG00000168743.12
2427	ENSG00000100697.14	ENSG00000284966.2	ENSG00000198133.8
2428	ENSG00000100711.13	ENSG00000284964.1	ENSG00000135116.9
2429	ENSG00000100714.16	ENSG00000284962.1	ENSG00000263232.2
2430	ENSG00000100721.11	ENSG00000284959.1	ENSG00000277253.1
2431	ENSG00000100722.20	ENSG00000284957.1	ENSG00000115459.18
2432	ENSG00000100726.15	ENSG00000284956.1	ENSG00000240540.2
2433	ENSG00000100731.15	ENSG00000284954.1	ENSG00000258234.1
2434	ENSG00000100739.11	ENSG00000284952.1	ENSG00000284188.2
2435	ENSG00000100744.15	ENSG00000284951.1	ENSG00000206727.1

2436	ENSG00000100749.8	ENSG00000284948.1	ENSG00000257400.1
2437	ENSG00000100764.14	ENSG00000284946.1	ENSG00000182963.10
2438	ENSG00000100767.16	ENSG00000284934.1	ENSG00000159733.13
2439	ENSG00000100784.12	ENSG00000284932.1	ENSG00000256577.2
2440	ENSG00000100796.17	ENSG00000284930.1	ENSG00000180104.16
2441	ENSG00000100802.15	ENSG00000284928.1	ENSG00000277604.1
2442	ENSG00000100804.18	ENSG00000284922.1	ENSG00000188729.6
2443	ENSG00000100811.13	ENSG00000284917.1	ENSG00000253188.1
2444	ENSG00000100813.14	ENSG00000284914.1	ENSG00000232606.1
2445	ENSG00000100814.18	ENSG00000284906.1	ENSG00000269564.1
2446	ENSG00000100815.12	ENSG00000284902.1	ENSG00000279217.1
2447	ENSG00000100823.12	ENSG00000284895.1	ENSG00000277865.4
2448	ENSG00000100836.10	ENSG00000284892.1	ENSG00000253006.1
2449	ENSG00000100842.13	ENSG00000284883.1	ENSG00000284624.1
2450	ENSG00000100852.13	ENSG00000284882.1	ENSG00000252620.1
2451	ENSG00000100865.15	ENSG00000284879.1	ENSG00000138111.14
2452	ENSG00000100867.14	ENSG00000284876.1	ENSG00000256357.1
2453	ENSG00000100883.12	ENSG00000284874.1	ENSG00000228650.2
2454	ENSG00000100884.9	ENSG00000284873.1	ENSG00000277971.1
2455	ENSG00000100888.13	ENSG00000284867.1	ENSG00000273372.1
2456	ENSG00000100889.11	ENSG00000284862.2	ENSG00000135697.10
2457	ENSG00000100890.15	ENSG00000284858.1	ENSG00000092470.12
2458	ENSG00000100897.17	ENSG00000284848.1	ENSG00000187553.9
2459	ENSG00000100902.10	ENSG00000284847.1	ENSG00000212175.1
2460	ENSG00000100906.10	ENSG00000284846.1	ENSG00000241532.1
2461	ENSG00000100908.14	ENSG00000284844.1	ENSG00000232096.1
2462	ENSG00000100911.16	ENSG00000284842.1	ENSG00000249026.2
2463	ENSG00000100916.14	ENSG00000284840.1	ENSG00000253507.5
2464	ENSG00000100918.13	ENSG00000284839.1	ENSG00000266928.1
2465	ENSG00000100926.15	ENSG00000284837.1	ENSG00000199906.1
2466	ENSG00000100934.15	ENSG00000284834.1	ENSG00000181123.8
2467	ENSG00000100938.17	ENSG00000284830.1	ENSG00000272922.1
2468	ENSG00000100941.9	ENSG00000284829.1	ENSG00000226908.1
2469	ENSG00000100949.14	ENSG00000284825.1	ENSG00000237813.3
2470	ENSG00000100968.14	ENSG00000284824.1	ENSG00000243438.1
2471	ENSG00000100979.15	ENSG00000284823.1	ENSG00000146535.14
2472	ENSG00000100982.12	ENSG00000284820.1	ENSG00000008441.16
2473	ENSG00000100983.11	ENSG00000284809.1	ENSG00000251155.2
2474	ENSG00000100985.7	ENSG00000284808.1	ENSG00000204296.11
2475	ENSG00000100987.14	ENSG00000284803.1	ENSG00000182351.7
2476	ENSG00000100991.12	ENSG00000284800.1	ENSG00000249234.1
2477	ENSG00000100994.12	ENSG00000284797.1	ENSG00000235623.2
2478	ENSG00000100997.19	ENSG00000284791.1	ENSG00000235064.1
2479	ENSG00000101000.6	ENSG00000284788.1	ENSG00000275327.1
2480	ENSG00000101003.10	ENSG00000284783.1	ENSG00000279486.5
2481	ENSG00000101004.15	ENSG00000284779.1	ENSG00000217385.1
2482	ENSG00000101017.13	ENSG00000284776.1	ENSG00000221083.1
2483	ENSG00000101019.22	ENSG00000284773.1	ENSG00000189398.5
2484	ENSG00000101040.19	ENSG00000284772.1	ENSG00000233929.1
2485	ENSG00000101049.15	ENSG00000284770.2	ENSG00000253584.1
2486	ENSG00000101052.12	ENSG00000284763.1	ENSG00000255655.1
2487	ENSG00000101057.16	ENSG00000284762.1	ENSG00000239576.1
2488	ENSG00000101074.4	ENSG00000284753.1	ENSG00000139874.6

2489	ENSG00000101076.16	ENSG00000284748.1	ENSG00000242242.5
2490	ENSG00000101079.21	ENSG00000284747.1	ENSG00000125633.11
2491	ENSG00000101082.14	ENSG00000284746.1	ENSG00000177685.17
2492	ENSG00000101084.18	ENSG00000284745.1	ENSG00000275040.1
2493	ENSG00000101096.20	ENSG00000284744.1	ENSG00000232411.1
2494	ENSG00000101098.12	ENSG00000284743.1	ENSG00000259843.2
2495	ENSG00000101104.12	ENSG00000284742.1	ENSG00000258987.1
2496	ENSG00000101109.12	ENSG00000284741.1	ENSG00000234607.2
2497	ENSG00000101115.13	ENSG00000284740.1	ENSG00000273974.1
2498	ENSG00000101126.17	ENSG00000284739.1	ENSG00000259724.1
2499	ENSG00000101132.10	ENSG00000284738.1	ENSG00000269371.1
2500	ENSG00000101134.11	ENSG00000284736.1	ENSG00000196517.11
2501	ENSG00000101138.11	ENSG00000284735.1	ENSG00000248585.2
2502	ENSG00000101144.13	ENSG00000284734.1	ENSG00000218813.1
2503	ENSG00000101146.13	ENSG00000284733.1	ENSG00000270257.1
2504	ENSG00000101150.17	ENSG00000284732.1	ENSG00000243762.1
2505	ENSG00000101152.11	ENSG00000284731.1	ENSG00000258554.1
2506	ENSG00000101158.14	ENSG00000284730.1	ENSG00000224532.2
2507	ENSG00000101160.14	ENSG00000284728.1	ENSG00000273035.1
2508	ENSG00000101161.8	ENSG00000284727.1	ENSG00000225091.3
2509	ENSG00000101162.3	ENSG00000284726.1	ENSG00000231096.1
2510	ENSG00000101166.16	ENSG00000284725.1	ENSG00000257531.2
2511	ENSG00000101180.16	ENSG00000284724.1	ENSG00000121764.11
2512	ENSG00000101181.17	ENSG00000284723.1	ENSG00000166173.10
2513	ENSG00000101182.15	ENSG00000284722.2	ENSG00000213669.2
2514	ENSG00000101187.16	ENSG00000284721.1	ENSG00000197360.9
2515	ENSG00000101188.5	ENSG00000284720.1	ENSG00000279303.1
2516	ENSG00000101189.7	ENSG00000284719.1	ENSG00000268623.2
2517	ENSG00000101190.12	ENSG00000284718.1	ENSG00000165694.9
2518	ENSG00000101191.16	ENSG00000284717.1	ENSG00000212935.1
2519	ENSG00000101193.8	ENSG00000284716.1	ENSG00000250071.1
2520	ENSG00000101194.18	ENSG00000284715.1	ENSG00000162927.14
2521	ENSG00000101197.13	ENSG00000284713.1	ENSG00000272915.1
2522	ENSG00000101198.15	ENSG00000284711.1	ENSG00000271332.1
2523	ENSG00000101199.13	ENSG00000284710.1	ENSG00000230483.1
2524	ENSG00000101200.5	ENSG00000284709.1	ENSG00000270480.1
2525	ENSG00000101203.16	ENSG00000284708.1	ENSG00000092853.14
2526	ENSG00000101204.17	ENSG00000284707.1	ENSG00000285679.1
2527	ENSG00000101210.12	ENSG00000284706.1	ENSG00000257569.1
2528	ENSG00000101213.7	ENSG00000284705.1	ENSG00000258742.5
2529	ENSG00000101216.11	ENSG00000284704.1	ENSG00000237341.1
2530	ENSG00000101220.17	ENSG00000284703.1	ENSG00000159882.13
2531	ENSG00000101222.12	ENSG00000284702.1	ENSG00000228403.1
2532	ENSG00000101224.17	ENSG00000284701.1	ENSG00000232393.1
2533	ENSG00000101230.6	ENSG00000284700.1	ENSG00000282840.1
2534	ENSG00000101236.17	ENSG00000284699.1	ENSG00000249697.1
2535	ENSG00000101246.20	ENSG00000284698.1	ENSG00000198028.3
2536	ENSG00000101247.18	ENSG00000284696.1	ENSG00000277483.1
2537	ENSG00000101251.12	ENSG00000284695.1	ENSG00000224971.1
2538	ENSG00000101255.11	ENSG00000284694.1	ENSG00000241162.3
2539	ENSG00000101265.16	ENSG00000284692.2	ENSG00000226949.1
2540	ENSG00000101266.19	ENSG00000284691.1	ENSG00000276014.1
2541	ENSG00000101276.16	ENSG00000284690.2	ENSG00000272672.1

2542	ENSG00000101278.6	ENSG00000284689.1	ENSG00000255690.2
2543	ENSG00000101280.8	ENSG00000284688.1	ENSG00000174448.8
2544	ENSG00000101282.9	ENSG00000284687.1	ENSG00000198658.4
2545	ENSG00000101290.14	ENSG00000284686.1	ENSG00000257704.3
2546	ENSG00000101292.7	ENSG00000284685.1	ENSG00000180329.14
2547	ENSG00000101298.15	ENSG00000284684.1	ENSG00000223718.3
2548	ENSG00000101306.10	ENSG00000284682.1	ENSG00000165891.16
2549	ENSG00000101307.15	ENSG00000284680.1	ENSG00000243489.4
2550	ENSG00000101310.16	ENSG00000284679.1	ENSG00000249921.2
2551	ENSG00000101311.15	ENSG00000284678.1	ENSG00000227437.1
2552	ENSG00000101323.5	ENSG00000284677.1	ENSG00000007968.7
2553	ENSG00000101327.9	ENSG00000284676.1	ENSG00000167491.17
2554	ENSG00000101331.15	ENSG00000284675.1	ENSG00000230053.2
2555	ENSG00000101333.16	ENSG00000284674.1	ENSG00000132692.19
2556	ENSG00000101335.10	ENSG00000284673.1	ENSG00000182870.13
2557	ENSG00000101336.14	ENSG00000284672.1	ENSG00000241135.5
2558	ENSG00000101337.15	ENSG00000284671.1	ENSG00000052344.16
2559	ENSG00000101342.10	ENSG00000284670.1	ENSG00000260197.1
2560	ENSG00000101343.14	ENSG00000284669.1	ENSG00000251611.1
2561	ENSG00000101346.14	ENSG00000284668.1	ENSG00000225133.1
2562	ENSG00000101347.9	ENSG00000284667.1	ENSG00000182459.5
2563	ENSG00000101349.16	ENSG00000284666.1	ENSG00000222594.1
2564	ENSG00000101350.8	ENSG00000284665.1	ENSG00000154529.14
2565	ENSG00000101353.14	ENSG00000284664.1	ENSG00000253898.1
2566	ENSG00000101361.17	ENSG00000284663.1	ENSG00000137207.12
2567	ENSG00000101363.12	ENSG00000284662.1	ENSG00000225357.3
2568	ENSG00000101365.21	ENSG00000284661.1	ENSG00000242062.1
2569	ENSG00000101367.9	ENSG00000284660.1	ENSG00000254632.1
2570	ENSG00000101384.12	ENSG00000284659.1	ENSG00000260014.1
2571	ENSG00000101391.21	ENSG00000284658.1	ENSG00000254397.1
2572	ENSG00000101400.5	ENSG00000284657.1	ENSG00000200530.1
2573	ENSG00000101405.3	ENSG00000284656.1	ENSG00000260723.1
2574	ENSG00000101407.13	ENSG00000284655.1	ENSG00000138316.11
2575	ENSG00000101412.13	ENSG00000284654.1	ENSG00000280474.1
2576	ENSG00000101413.11	ENSG00000284653.1	ENSG00000229502.5
2577	ENSG00000101417.12	ENSG00000284652.1	ENSG00000152661.8
2578	ENSG00000101421.4	ENSG00000284651.1	ENSG00000230356.1
2579	ENSG00000101425.13	ENSG00000284650.1	ENSG00000272721.5
2580	ENSG00000101435.5	ENSG00000284649.1	ENSG00000282863.1
2581	ENSG00000101438.4	ENSG00000284648.1	ENSG00000106483.12
2582	ENSG00000101439.9	ENSG00000284646.1	ENSG00000258876.1
2583	ENSG00000101440.9	ENSG00000284645.1	ENSG00000266341.1
2584	ENSG00000101441.4	ENSG00000284644.1	ENSG00000175928.5
2585	ENSG00000101442.10	ENSG00000284643.1	ENSG00000243772.7
2586	ENSG00000101443.18	ENSG00000284642.1	ENSG00000283941.1
2587	ENSG00000101444.13	ENSG00000284641.1	ENSG00000226641.1
2588	ENSG00000101445.10	ENSG00000284640.1	ENSG00000286033.1
2589	ENSG00000101446.7	ENSG00000284639.1	ENSG00000223629.1
2590	ENSG00000101447.15	ENSG00000284638.1	ENSG00000260033.1
2591	ENSG00000101448.13	ENSG00000284637.1	ENSG00000273976.1
2592	ENSG00000101452.15	ENSG00000284636.1	ENSG00000102055.6
2593	ENSG00000101457.13	ENSG00000284635.1	ENSG00000271584.2
2594	ENSG00000101460.13	ENSG00000284634.1	ENSG00000258137.5

2595	ENSG00000101463.6	ENSG00000284632.1	ENSG00000240549.2
2596	ENSG00000101464.10	ENSG00000284631.1	ENSG00000186448.15
2597	ENSG00000101470.10	ENSG00000284630.1	ENSG00000225602.5
2598	ENSG00000101473.17	ENSG00000284629.1	ENSG00000230178.1
2599	ENSG00000101474.12	ENSG00000284628.1	ENSG00000261243.1
2600	ENSG00000101489.20	ENSG00000284627.1	ENSG00000227765.4
2601	ENSG00000101493.11	ENSG00000284626.1	ENSG00000109654.15
2602	ENSG00000101542.10	ENSG00000284625.1	ENSG00000248103.1
2603	ENSG00000101544.9	ENSG00000284624.1	ENSG00000259584.1
2604	ENSG00000101546.13	ENSG00000284623.1	ENSG00000265982.1
2605	ENSG00000101557.15	ENSG00000284622.2	ENSG00000231359.3
2606	ENSG00000101558.13	ENSG00000284621.1	ENSG00000274333.4
2607	ENSG00000101574.15	ENSG00000284620.2	ENSG00000131943.17
2608	ENSG00000101577.9	ENSG00000284619.1	ENSG00000122863.6
2609	ENSG00000101596.15	ENSG00000284618.1	ENSG00000120451.10
2610	ENSG00000101605.12	ENSG00000284617.1	ENSG00000271402.1
2611	ENSG00000101608.12	ENSG00000284616.1	ENSG00000279426.1
2612	ENSG00000101624.10	ENSG00000284615.1	ENSG00000174236.3
2613	ENSG00000101638.13	ENSG00000284614.1	ENSG00000260782.1
2614	ENSG00000101639.18	ENSG00000284613.1	ENSG00000253921.1
2615	ENSG00000101654.17	ENSG00000284612.1	ENSG00000115816.15
2616	ENSG00000101665.9	ENSG00000284611.1	ENSG00000274226.5
2617	ENSG00000101670.12	ENSG00000284610.1	ENSG00000149300.10
2618	ENSG00000101680.15	ENSG00000284609.1	ENSG00000250496.2
2619	ENSG00000101695.9	ENSG00000284608.1	ENSG00000278292.1
2620	ENSG00000101745.17	ENSG00000284607.1	ENSG00000285040.1
2621	ENSG00000101746.15	ENSG00000284606.1	ENSG00000228600.1
2622	ENSG00000101751.10	ENSG00000284605.1	ENSG00000266497.1
2623	ENSG00000101752.11	ENSG00000284604.1	ENSG00000259423.2
2624	ENSG00000101773.19	ENSG00000284603.1	ENSG00000261697.5
2625	ENSG00000101782.15	ENSG00000284602.1	ENSG00000282022.1
2626	ENSG00000101811.13	ENSG00000284601.1	ENSG00000253112.1
2627	ENSG00000101812.12	ENSG00000284596.1	ENSG00000060138.13
2628	ENSG00000101825.8	ENSG00000284595.1	ENSG00000228151.1
2629	ENSG00000101842.13	ENSG00000284594.1	ENSG00000263096.1
2630	ENSG00000101843.19	ENSG00000284592.2	ENSG00000244044.3
2631	ENSG00000101844.18	ENSG00000284591.1	ENSG00000249896.2
2632	ENSG00000101846.6	ENSG00000284587.1	ENSG00000226040.3
2633	ENSG00000101849.16	ENSG00000284586.1	ENSG00000197826.11
2634	ENSG00000101850.13	ENSG00000284585.1	ENSG00000175773.13
2635	ENSG00000101856.10	ENSG00000284584.1	ENSG00000167393.17
2636	ENSG00000101868.10	ENSG00000284583.1	ENSG00000233235.1
2637	ENSG00000101871.14	ENSG00000284575.1	ENSG00000146376.11
2638	ENSG00000101882.10	ENSG00000284574.1	ENSG00000242396.1
2639	ENSG00000101883.4	ENSG00000284572.1	ENSG00000232267.1
2640	ENSG00000101888.11	ENSG00000284570.1	ENSG00000216854.2
2641	ENSG00000101890.4	ENSG00000284568.1	ENSG00000233237.7
2642	ENSG00000101892.12	ENSG00000284567.1	ENSG00000277794.1
2643	ENSG00000101898.6	ENSG00000284565.1	ENSG00000251569.1
2644	ENSG00000101901.11	ENSG00000284564.1	ENSG00000213046.4
2645	ENSG00000101911.12	ENSG00000284561.1	ENSG00000277235.1
2646	ENSG00000101916.11	ENSG00000284558.1	ENSG00000234108.1
2647	ENSG00000101928.13	ENSG00000284557.1	ENSG00000185825.16

2648	ENSG00000101935.10	ENSG00000284554.2	ENSG00000275337.1
2649	ENSG00000101938.14	ENSG00000284553.1	ENSG00000267341.1
2650	ENSG00000101940.18	ENSG00000284550.1	ENSG00000198125.13
2651	ENSG00000101945.16	ENSG00000284547.1	ENSG00000196182.10
2652	ENSG00000101951.16	ENSG00000284546.1	ENSG00000232568.2
2653	ENSG00000101955.15	ENSG00000284544.1	ENSG00000227568.1
2654	ENSG00000101958.14	ENSG00000284542.1	ENSG00000146038.12
2655	ENSG00000101966.12	ENSG00000284541.1	ENSG00000147586.10
2656	ENSG00000101972.18	ENSG00000284540.1	ENSG00000234788.1
2657	ENSG00000101974.14	ENSG00000284538.1	ENSG00000206172.8
2658	ENSG00000101977.21	ENSG00000284536.1	ENSG00000255686.1
2659	ENSG00000101981.11	ENSG00000284535.1	ENSG00000278713.1
2660	ENSG00000101986.12	ENSG00000284534.1	ENSG00000146938.16
2661	ENSG00000101997.13	ENSG00000284532.1	ENSG00000268883.2
2662	ENSG00000102001.12	ENSG00000284526.1	ENSG00000170381.14
2663	ENSG00000102003.10	ENSG00000284525.1	ENSG00000204603.6
2664	ENSG00000102007.11	ENSG00000284523.1	ENSG00000255811.1
2665	ENSG00000102010.15	ENSG00000284522.1	ENSG00000230176.2
2666	ENSG00000102021.11	ENSG00000284520.1	ENSG00000250831.1
2667	ENSG00000102024.18	ENSG00000284519.1	ENSG00000164334.15
2668	ENSG00000102030.16	ENSG00000284518.1	ENSG00000247228.2
2669	ENSG00000102032.13	ENSG00000284517.1	ENSG00000118492.17
2670	ENSG00000102034.16	ENSG00000284516.1	ENSG00000268884.1
2671	ENSG00000102038.15	ENSG00000284513.1	ENSG00000234371.6
2672	ENSG00000102043.16	ENSG00000284512.1	ENSG00000107562.16
2673	ENSG00000102048.15	ENSG00000284508.1	ENSG00000233086.7
2674	ENSG00000102053.12	ENSG00000284505.1	ENSG00000261253.2
2675	ENSG00000102054.17	ENSG00000284503.1	ENSG00000248954.1
2676	ENSG00000102055.6	ENSG00000284500.2	ENSG00000267219.1
2677	ENSG00000102057.10	ENSG00000284499.1	ENSG00000143952.20
2678	ENSG00000102076.9	ENSG00000284498.1	ENSG00000284525.1
2679	ENSG00000102078.16	ENSG00000284491.2	ENSG00000244295.2
2680	ENSG00000102081.14	ENSG00000284490.1	ENSG00000257202.1
2681	ENSG00000102096.9	ENSG00000284489.1	ENSG00000206159.11
2682	ENSG00000102098.18	ENSG00000284488.1	ENSG00000239944.1
2683	ENSG00000102100.16	ENSG00000284485.1	ENSG00000253336.1
2684	ENSG00000102103.16	ENSG00000284484.1	ENSG00000241881.1
2685	ENSG00000102104.8	ENSG00000284482.1	ENSG00000281450.1
2686	ENSG00000102109.9	ENSG00000284481.1	ENSG00000165105.10
2687	ENSG00000102119.10	ENSG00000284479.1	ENSG00000237801.6
2688	ENSG00000102125.16	ENSG00000284474.1	ENSG00000183929.7
2689	ENSG00000102128.8	ENSG00000284473.1	ENSG00000053747.16
2690	ENSG00000102144.15	ENSG00000284471.1	ENSG00000179058.7
2691	ENSG00000102145.14	ENSG00000284469.1	ENSG00000258628.1
2692	ENSG00000102158.19	ENSG00000284465.1	ENSG00000143889.16
2693	ENSG00000102172.16	ENSG00000284464.1	ENSG00000267251.2
2694	ENSG00000102174.9	ENSG00000284463.1	ENSG00000224982.4
2695	ENSG00000102178.13	ENSG00000284461.2	ENSG00000134333.14
2696	ENSG00000102181.21	ENSG00000284459.1	ENSG00000250282.1
2697	ENSG00000102189.16	ENSG00000284458.1	ENSG00000236472.1
2698	ENSG00000102195.9	ENSG00000284453.1	ENSG00000250928.1
2699	ENSG00000102218.6	ENSG00000284452.1	ENSG00000207014.1
2700	ENSG00000102221.14	ENSG00000284450.1	ENSG00000168481.9

2701	ENSG00000102225.16	ENSG00000284448.1	ENSG00000111799.21
2702	ENSG00000102226.9	ENSG00000284447.1	ENSG00000201965.1
2703	ENSG00000102230.13	ENSG00000284446.1	ENSG00000275146.1
2704	ENSG00000102239.5	ENSG00000284443.1	ENSG00000168269.10
2705	ENSG00000102241.12	ENSG00000284442.1	ENSG00000235175.1
2706	ENSG00000102243.13	ENSG00000284440.1	ENSG00000240667.1
2707	ENSG00000102245.7	ENSG00000284439.1	ENSG00000198033.12
2708	ENSG00000102265.12	ENSG00000284438.1	ENSG00000170525.21
2709	ENSG00000102271.14	ENSG00000284436.1	ENSG00000282834.1
2710	ENSG00000102287.19	ENSG00000284435.1	ENSG00000061492.11
2711	ENSG00000102290.22	ENSG00000284433.1	ENSG00000282787.1
2712	ENSG00000102302.8	ENSG00000284431.1	ENSG00000269431.1
2713	ENSG00000102309.13	ENSG00000284430.1	ENSG00000129749.3
2714	ENSG00000102312.21	ENSG00000284428.1	ENSG00000213896.4
2715	ENSG00000102313.8	ENSG00000284427.1	ENSG00000272639.1
2716	ENSG00000102316.17	ENSG00000284425.1	ENSG00000129595.13
2717	ENSG00000102317.18	ENSG00000284424.1	ENSG00000224570.1
2718	ENSG00000102349.18	ENSG00000284421.1	ENSG00000166169.17
2719	ENSG00000102359.7	ENSG00000284419.1	ENSG00000231296.1
2720	ENSG00000102362.15	ENSG00000284418.1	ENSG00000260185.1
2721	ENSG00000102383.14	ENSG00000284416.1	ENSG00000283536.2
2722	ENSG00000102385.12	ENSG00000284415.1	ENSG00000270095.1
2723	ENSG00000102387.15	ENSG00000284412.1	ENSG00000263212.2
2724	ENSG00000102390.10	ENSG00000284411.1	ENSG00000078579.9
2725	ENSG00000102393.10	ENSG00000284410.1	ENSG00000283737.1
2726	ENSG00000102401.20	ENSG00000284407.1	ENSG00000099617.4
2727	ENSG00000102409.10	ENSG00000284402.1	ENSG00000206561.13
2728	ENSG00000102445.18	ENSG00000284399.1	ENSG00000196866.2
2729	ENSG00000102452.17	ENSG00000284395.1	ENSG00000284733.1
2730	ENSG00000102466.15	ENSG00000284394.1	ENSG00000213365.3
2731	ENSG00000102468.10	ENSG00000284393.1	ENSG00000270405.1
2732	ENSG00000102471.14	ENSG00000284391.1	ENSG00000271257.1
2733	ENSG00000102524.11	ENSG00000284388.1	ENSG00000273416.1
2734	ENSG00000102531.16	ENSG00000284387.1	ENSG00000280444.1
2735	ENSG00000102539.5	ENSG00000284386.1	ENSG00000199933.1
2736	ENSG00000102543.14	ENSG00000284385.1	ENSG00000278077.1
2737	ENSG00000102547.19	ENSG00000284380.1	ENSG00000226501.2
2738	ENSG00000102554.14	ENSG00000284378.1	ENSG00000261038.1
2739	ENSG00000102572.14	ENSG00000284377.1	ENSG00000186994.11
2740	ENSG00000102575.11	ENSG00000284376.1	ENSG00000227186.1
2741	ENSG00000102580.15	ENSG00000284375.1	ENSG00000237529.2
2742	ENSG00000102595.20	ENSG00000284373.1	ENSG00000272034.1
2743	ENSG00000102606.18	ENSG00000284372.1	ENSG00000229954.1
2744	ENSG00000102678.6	ENSG00000284370.1	ENSG00000231971.5
2745	ENSG00000102683.7	ENSG00000284368.1	ENSG00000111801.16
2746	ENSG00000102699.6	ENSG00000284364.1	ENSG00000267781.1
2747	ENSG00000102710.20	ENSG00000284363.1	ENSG00000270990.1
2748	ENSG00000102738.7	ENSG00000284362.1	ENSG00000140478.15
2749	ENSG00000102743.15	ENSG00000284361.1	ENSG00000242472.1
2750	ENSG00000102753.10	ENSG00000284360.1	ENSG00000183943.5
2751	ENSG00000102755.12	ENSG00000284357.1	ENSG00000121211.7
2752	ENSG00000102760.13	ENSG00000284356.1	ENSG00000236823.1
2753	ENSG00000102763.18	ENSG00000284353.1	ENSG00000248693.1

2754	ENSG00000102780.16	ENSG00000284351.1	ENSG00000235967.1
2755	ENSG00000102781.14	ENSG00000284346.1	ENSG00000282051.1
2756	ENSG00000102786.15	ENSG00000284344.1	ENSG00000223572.9
2757	ENSG00000102794.9	ENSG00000284343.1	ENSG00000226954.1
2758	ENSG00000102796.11	ENSG00000284341.1	ENSG00000277610.1
2759	ENSG00000102802.10	ENSG00000284337.1	ENSG00000262681.2
2760	ENSG00000102804.15	ENSG00000284336.1	ENSG00000244229.1
2761	ENSG00000102805.15	ENSG00000284334.1	ENSG00000277297.1
2762	ENSG00000102837.7	ENSG00000284332.1	ENSG00000254502.1
2763	ENSG00000102854.16	ENSG00000284331.1	ENSG00000230710.1
2764	ENSG00000102858.13	ENSG00000284329.1	ENSG00000166546.14
2765	ENSG00000102870.6	ENSG00000284328.1	ENSG00000232283.1
2766	ENSG00000102871.16	ENSG00000284327.1	ENSG00000213050.5
2767	ENSG00000102878.16	ENSG00000284325.1	ENSG00000110900.15
2768	ENSG00000102879.15	ENSG00000284324.1	ENSG00000234999.1
2769	ENSG00000102882.12	ENSG00000284321.1	ENSG00000173702.7
2770	ENSG00000102886.15	ENSG00000284317.1	ENSG00000086827.9
2771	ENSG00000102890.15	ENSG00000284311.1	ENSG00000275898.1
2772	ENSG00000102891.3	ENSG00000284310.1	ENSG00000243227.3
2773	ENSG00000102893.16	ENSG00000284309.1	ENSG00000260172.1
2774	ENSG00000102897.10	ENSG00000284308.1	ENSG00000234210.1
2775	ENSG00000102898.12	ENSG00000284306.1	ENSG00000268896.1
2776	ENSG00000102900.13	ENSG00000284305.1	ENSG00000283491.1
2777	ENSG00000102901.13	ENSG00000284300.1	ENSG00000233702.1
2778	ENSG00000102904.14	ENSG00000284299.1	ENSG00000146477.6
2779	ENSG00000102908.21	ENSG00000284293.1	ENSG00000213735.2
2780	ENSG00000102910.13	ENSG00000284292.1	ENSG00000233410.1
2781	ENSG00000102921.8	ENSG00000284291.1	ENSG00000172365.3
2782	ENSG00000102924.12	ENSG00000284289.1	ENSG00000213962.2
2783	ENSG00000102931.8	ENSG00000284288.1	ENSG00000202522.1
2784	ENSG00000102934.10	ENSG00000284286.1	ENSG00000260473.2
2785	ENSG00000102935.11	ENSG00000284284.1	ENSG00000268416.1
2786	ENSG00000102962.5	ENSG00000284283.1	ENSG00000072121.16
2787	ENSG00000102967.12	ENSG00000284280.1	ENSG00000269172.1
2788	ENSG00000102970.10	ENSG00000284277.1	ENSG00000163914.4
2789	ENSG00000102974.16	ENSG00000284276.1	ENSG00000275869.1
2790	ENSG00000102977.15	ENSG00000284272.1	ENSG00000267526.1
2791	ENSG00000102978.13	ENSG00000284269.1	ENSG00000227640.2
2792	ENSG00000102981.9	ENSG00000284268.1	ENSG00000267409.1
2793	ENSG00000102984.15	ENSG00000284266.1	ENSG00000242770.2
2794	ENSG00000102996.5	ENSG00000284265.1	ENSG00000236973.2
2795	ENSG00000103005.11	ENSG00000284263.1	ENSG00000227438.1
2796	ENSG00000103018.16	ENSG00000284261.1	ENSG00000280560.1
2797	ENSG00000103021.9	ENSG00000284259.1	ENSG00000230980.1
2798	ENSG00000103023.11	ENSG00000284258.1	ENSG00000227803.2
2799	ENSG00000103024.7	ENSG00000284256.1	ENSG00000233203.6
2800	ENSG00000103034.14	ENSG00000284253.1	ENSG00000249106.1
2801	ENSG00000103035.11	ENSG00000284252.1	ENSG00000236003.1
2802	ENSG00000103037.11	ENSG00000284251.1	ENSG00000249283.3
2803	ENSG00000103042.9	ENSG00000284250.1	ENSG00000280171.1
2804	ENSG00000103043.15	ENSG00000284247.1	ENSG00000233684.2
2805	ENSG00000103044.11	ENSG00000284246.1	ENSG00000235872.2
2806	ENSG00000103047.8	ENSG00000284242.1	ENSG00000174899.11

2807	ENSG00000103051.19	ENSG00000284240.1	ENSG00000164532.10
2808	ENSG00000103056.12	ENSG00000284239.1	ENSG00000271045.1
2809	ENSG00000103061.13	ENSG00000284237.1	ENSG00000236447.2
2810	ENSG00000103064.15	ENSG00000284234.1	ENSG00000186469.8
2811	ENSG00000103066.13	ENSG00000284233.1	ENSG00000226488.1
2812	ENSG00000103067.13	ENSG00000284231.1	ENSG00000085741.13
2813	ENSG00000103089.9	ENSG00000284229.1	ENSG00000213085.10
2814	ENSG00000103091.15	ENSG00000284224.1	ENSG00000279764.1
2815	ENSG00000103111.14	ENSG00000284221.1	ENSG00000251072.2
2816	ENSG00000103121.8	ENSG00000284219.1	ENSG00000228291.1
2817	ENSG00000103126.15	ENSG00000284216.1	ENSG00000237330.3
2818	ENSG00000103145.10	ENSG00000284214.1	ENSG00000215869.4
2819	ENSG00000103148.15	ENSG00000284209.1	ENSG00000259592.1
2820	ENSG00000103150.6	ENSG00000284204.1	ENSG00000266840.1
2821	ENSG00000103152.11	ENSG00000284203.1	ENSG00000177586.6
2822	ENSG00000103154.9	ENSG00000284202.1	ENSG00000162771.7
2823	ENSG00000103160.12	ENSG00000284200.1	ENSG00000270880.1
2824	ENSG00000103168.16	ENSG00000284197.1	ENSG00000104921.15
2825	ENSG00000103174.13	ENSG00000284196.2	ENSG00000221025.1
2826	ENSG00000103175.11	ENSG00000284195.1	ENSG00000145934.16
2827	ENSG00000103184.12	ENSG00000284194.1	ENSG00000225720.6
2828	ENSG00000103187.8	ENSG00000284193.1	ENSG00000230102.7
2829	ENSG00000103194.15	ENSG00000284191.1	ENSG00000286057.1
2830	ENSG00000103196.12	ENSG00000284190.1	ENSG00000268731.1
2831	ENSG00000103197.18	ENSG00000284188.2	ENSG00000144597.14
2832	ENSG00000103199.13	ENSG00000284186.1	ENSG00000267246.1
2833	ENSG00000103200.6	ENSG00000284185.1	ENSG00000257976.1
2834	ENSG00000103202.13	ENSG00000284184.1	ENSG00000196277.16
2835	ENSG00000103222.19	ENSG00000284182.1	ENSG00000230648.1
2836	ENSG00000103226.18	ENSG00000284180.1	ENSG00000196109.8
2837	ENSG00000103227.18	ENSG00000284179.1	ENSG00000134160.13
2838	ENSG00000103241.7	ENSG00000284176.1	ENSG00000234572.1
2839	ENSG00000103245.14	ENSG00000284175.1	ENSG00000275489.1
2840	ENSG00000103248.18	ENSG00000284173.1	ENSG00000280180.1
2841	ENSG00000103249.18	ENSG00000284172.1	ENSG00000284541.1
2842	ENSG00000103253.18	ENSG00000284171.1	ENSG00000254174.1
2843	ENSG00000103254.10	ENSG00000284167.1	ENSG00000260395.1
2844	ENSG00000103257.9	ENSG00000284163.1	ENSG00000275942.1
2845	ENSG00000103260.9	ENSG00000284162.1	ENSG00000251054.1
2846	ENSG00000103264.18	ENSG00000284160.1	ENSG00000255825.1
2847	ENSG00000103266.11	ENSG00000284159.1	ENSG00000284642.1
2848	ENSG00000103269.13	ENSG00000284158.1	ENSG00000259129.5
2849	ENSG00000103274.11	ENSG00000284157.1	ENSG00000165215.6
2850	ENSG00000103275.20	ENSG00000284155.1	ENSG00000175311.7
2851	ENSG00000103310.10	ENSG00000284154.1	ENSG00000107984.10
2852	ENSG00000103313.12	ENSG00000284152.1	ENSG00000240673.1
2853	ENSG00000103316.11	ENSG00000284149.1	ENSG00000261177.1
2854	ENSG00000103319.12	ENSG00000284148.1	ENSG00000158716.8
2855	ENSG00000103326.12	ENSG00000284147.1	ENSG00000243853.1
2856	ENSG00000103335.22	ENSG00000284146.1	ENSG00000227278.1
2857	ENSG00000103342.13	ENSG00000284144.1	ENSG00000205644.5
2858	ENSG00000103343.13	ENSG00000284143.1	ENSG00000265094.1
2859	ENSG00000103351.13	ENSG00000284142.1	ENSG00000140988.16

2860	ENSG00000103353.16	ENSG00000284140.1	ENSG00000237517.9
2861	ENSG00000103355.13	ENSG00000284139.1	ENSG00000259261.2
2862	ENSG00000103356.15	ENSG00000284138.1	ENSG00000225756.1
2863	ENSG00000103363.14	ENSG00000284135.1	ENSG00000278696.1
2864	ENSG00000103365.15	ENSG00000284134.1	ENSG00000101200.5
2865	ENSG00000103375.11	ENSG00000284130.1	ENSG00000228974.5
2866	ENSG00000103381.12	ENSG00000284129.1	ENSG00000207119.2
2867	ENSG00000103404.14	ENSG00000284128.1	ENSG00000241926.1
2868	ENSG00000103415.12	ENSG00000284125.1	ENSG00000269699.6
2869	ENSG00000103423.14	ENSG00000284122.1	ENSG00000142513.5
2870	ENSG00000103426.12	ENSG00000284121.1	ENSG00000198792.13
2871	ENSG00000103429.11	ENSG00000284118.1	ENSG00000186092.6
2872	ENSG00000103449.11	ENSG00000284117.1	ENSG00000277402.1
2873	ENSG00000103460.17	ENSG00000284116.1	ENSG00000230433.1
2874	ENSG00000103472.10	ENSG00000284114.1	ENSG00000256061.7
2875	ENSG00000103479.16	ENSG00000284112.1	ENSG00000227257.1
2876	ENSG00000103485.18	ENSG00000284108.1	ENSG00000259443.1
2877	ENSG00000103489.11	ENSG00000284107.1	ENSG00000281938.2
2878	ENSG00000103490.14	ENSG00000284098.1	ENSG00000126545.14
2879	ENSG00000103494.14	ENSG00000284095.1	ENSG00000104765.16
2880	ENSG00000103495.14	ENSG00000284094.1	ENSG00000226128.1
2881	ENSG00000103496.15	ENSG00000284092.1	ENSG00000196832.4
2882	ENSG00000103502.14	ENSG00000284087.1	ENSG00000240210.3
2883	ENSG00000103507.14	ENSG00000284085.1	ENSG00000232466.1
2884	ENSG00000103510.20	ENSG00000284084.1	ENSG00000104723.20
2885	ENSG00000103512.15	ENSG00000284082.1	ENSG00000229065.1
2886	ENSG00000103522.16	ENSG00000284081.1	ENSG00000257272.1
2887	ENSG00000103528.17	ENSG00000284079.1	ENSG00000258960.1
2888	ENSG00000103534.17	ENSG00000284078.1	ENSG00000083067.22
2889	ENSG00000103540.16	ENSG00000284074.1	ENSG00000280321.1
2890	ENSG00000103544.14	ENSG00000284071.1	ENSG00000232760.1
2891	ENSG00000103546.18	ENSG00000284070.1	ENSG00000234271.1
2892	ENSG00000103549.21	ENSG00000284067.1	ENSG00000238755.3
2893	ENSG00000103550.14	ENSG00000284065.1	ENSG00000250374.4
2894	ENSG00000103569.9	ENSG00000284064.1	ENSG00000261760.8
2895	ENSG00000103591.12	ENSG00000284062.1	ENSG00000228941.1
2896	ENSG00000103599.20	ENSG00000284060.1	ENSG00000254127.1
2897	ENSG00000103642.12	ENSG00000284059.1	ENSG00000110497.14
2898	ENSG00000103647.12	ENSG00000284057.1	ENSG00000256155.1
2899	ENSG00000103653.16	ENSG00000284055.1	ENSG00000099804.9
2900	ENSG00000103657.14	ENSG00000284054.1	ENSG00000271065.1
2901	ENSG00000103671.9	ENSG00000284052.1	ENSG00000275769.1
2902	ENSG00000103707.10	ENSG00000284049.1	ENSG00000214896.4
2903	ENSG00000103710.11	ENSG00000284048.2	ENSG00000225392.1
2904	ENSG00000103723.13	ENSG00000284047.1	ENSG00000215475.5
2905	ENSG00000103740.10	ENSG00000284043.1	ENSG00000172186.7
2906	ENSG00000103742.12	ENSG00000284042.1	ENSG00000205364.4
2907	ENSG00000103769.10	ENSG00000284041.1	ENSG00000236523.2
2908	ENSG00000103811.16	ENSG00000284040.1	ENSG00000172818.10
2909	ENSG00000103832.10	ENSG00000284038.1	ENSG00000254740.2
2910	ENSG00000103852.13	ENSG00000284035.1	ENSG00000172724.12
2911	ENSG00000103855.18	ENSG00000284034.1	ENSG00000254048.1
2912	ENSG00000103876.12	ENSG00000284032.1	ENSG00000206698.1

2913	ENSG00000103888.17	ENSG00000284031.1	ENSG00000228550.1
2914	ENSG00000103932.12	ENSG00000284029.1	ENSG00000254500.1
2915	ENSG00000103942.13	ENSG00000284028.1	ENSG00000225084.1
2916	ENSG00000103966.11	ENSG00000284027.1	ENSG00000227710.1
2917	ENSG00000103978.15	ENSG00000284024.2	ENSG00000279137.1
2918	ENSG00000103994.17	ENSG00000284020.1	ENSG00000253421.1
2919	ENSG00000103995.14	ENSG00000284018.1	ENSG00000228247.1
2920	ENSG00000104043.14	ENSG00000284015.1	ENSG00000186960.11
2921	ENSG00000104044.16	ENSG00000284012.1	ENSG00000181001.2
2922	ENSG00000104047.15	ENSG00000284011.1	ENSG00000164638.10
2923	ENSG00000104055.15	ENSG00000284010.1	ENSG00000170807.12
2924	ENSG00000104059.4	ENSG00000284008.1	ENSG00000230295.1
2925	ENSG00000104064.17	ENSG00000284005.1	ENSG00000110148.10
2926	ENSG00000104067.16	ENSG00000284003.1	ENSG00000271095.1
2927	ENSG00000104081.14	ENSG00000284000.1	ENSG00000232406.6
2928	ENSG00000104093.13	ENSG00000283999.1	ENSG00000275025.1
2929	ENSG00000104112.9	ENSG00000283998.1	ENSG00000274364.1
2930	ENSG00000104129.10	ENSG00000283994.1	ENSG00000276704.1
2931	ENSG00000104131.13	ENSG00000283992.2	ENSG00000283608.1
2932	ENSG00000104133.15	ENSG00000283991.1	ENSG00000127663.15
2933	ENSG00000104140.7	ENSG00000283990.1	ENSG00000215070.4
2934	ENSG00000104142.11	ENSG00000283988.1	ENSG00000236434.2
2935	ENSG00000104147.9	ENSG00000283982.1	ENSG00000230299.1
2936	ENSG00000104154.7	ENSG00000283980.1	ENSG00000164287.12
2937	ENSG00000104164.10	ENSG00000283978.1	ENSG00000101327.9
2938	ENSG00000104177.18	ENSG00000283977.1	ENSG00000188755.10
2939	ENSG00000104205.14	ENSG00000283973.1	ENSG00000217275.2
2940	ENSG00000104213.12	ENSG00000283972.1	ENSG00000136827.12
2941	ENSG00000104218.14	ENSG00000283971.1	ENSG00000167393.17
2942	ENSG00000104219.13	ENSG00000283969.1	ENSG00000251634.2
2943	ENSG00000104221.12	ENSG00000283967.1	ENSG00000231812.1
2944	ENSG00000104228.13	ENSG00000283959.1	ENSG00000268081.1
2945	ENSG00000104231.11	ENSG00000283958.1	ENSG00000285603.1
2946	ENSG00000104237.10	ENSG00000283956.1	ENSG00000205639.10
2947	ENSG00000104267.10	ENSG00000283952.1	ENSG00000235419.5
2948	ENSG00000104290.11	ENSG00000283950.1	ENSG00000227748.1
2949	ENSG00000104299.15	ENSG00000283948.1	ENSG00000213331.4
2950	ENSG00000104312.8	ENSG00000283945.1	ENSG00000175264.8
2951	ENSG00000104313.19	ENSG00000283944.1	ENSG00000214617.9
2952	ENSG00000104320.13	ENSG00000283941.1	ENSG00000229991.1
2953	ENSG00000104321.11	ENSG00000283940.1	ENSG00000213701.4
2954	ENSG00000104324.16	ENSG00000283938.1	ENSG00000162989.5
2955	ENSG00000104325.7	ENSG00000283936.1	ENSG00000277379.1
2956	ENSG00000104327.7	ENSG00000283935.1	ENSG00000230550.1
2957	ENSG00000104331.9	ENSG00000283933.1	ENSG00000276591.1
2958	ENSG00000104332.12	ENSG00000283932.1	ENSG00000279401.1
2959	ENSG00000104341.16	ENSG00000283931.1	ENSG00000215456.6
2960	ENSG00000104343.20	ENSG00000283930.1	ENSG00000272411.1
2961	ENSG00000104356.11	ENSG00000283929.1	ENSG00000148408.13
2962	ENSG00000104361.10	ENSG00000283928.1	ENSG00000168014.16
2963	ENSG00000104365.15	ENSG00000283927.1	ENSG00000049249.8
2964	ENSG00000104368.17	ENSG00000283926.1	ENSG00000225930.4
2965	ENSG00000104369.5	ENSG00000283923.1	ENSG00000269886.1

2966	ENSG00000104371.5	ENSG00000283921.1	ENSG00000228540.1
2967	ENSG00000104375.17	ENSG00000283920.1	ENSG00000286171.1
2968	ENSG00000104381.12	ENSG00000283914.1	ENSG00000226453.1
2969	ENSG00000104388.15	ENSG00000283913.1	ENSG00000197457.10
2970	ENSG00000104408.9	ENSG00000283906.1	ENSG00000054938.15
2971	ENSG00000104412.8	ENSG00000283904.1	ENSG00000167680.16
2972	ENSG00000104413.17	ENSG00000283900.1	ENSG00000238290.1
2973	ENSG00000104415.14	ENSG00000283899.1	ENSG00000187959.9
2974	ENSG00000104419.14	ENSG00000283897.1	ENSG00000224861.1
2975	ENSG00000104427.12	ENSG00000283894.1	ENSG00000215760.2
2976	ENSG00000104432.14	ENSG00000283891.1	ENSG00000233583.1
2977	ENSG00000104435.13	ENSG00000283888.1	ENSG00000219294.6
2978	ENSG00000104442.10	ENSG00000283886.2	ENSG00000255909.1
2979	ENSG00000104447.12	ENSG00000283885.1	ENSG00000267206.6
2980	ENSG00000104450.12	ENSG00000283881.1	ENSG00000256512.1
2981	ENSG00000104472.10	ENSG00000283880.1	ENSG00000256980.5
2982	ENSG00000104490.18	ENSG00000283879.1	ENSG00000279993.1
2983	ENSG00000104497.14	ENSG00000283877.1	ENSG00000260345.1
2984	ENSG00000104499.6	ENSG00000283876.1	ENSG00000248254.1
2985	ENSG00000104517.13	ENSG00000283874.1	ENSG00000117625.13
2986	ENSG00000104518.11	ENSG00000283873.1	ENSG00000207359.1
2987	ENSG00000104522.16	ENSG00000283871.1	ENSG00000171016.12
2988	ENSG00000104524.14	ENSG00000283867.1	ENSG00000204091.7
2989	ENSG00000104529.17	ENSG00000283865.1	ENSG00000188064.10
2990	ENSG00000104537.17	ENSG00000283863.1	ENSG00000242229.1
2991	ENSG00000104549.12	ENSG00000283858.1	ENSG00000042781.13
2992	ENSG00000104611.12	ENSG00000283857.1	ENSG00000273272.2
2993	ENSG00000104613.12	ENSG00000283856.1	ENSG00000076382.17
2994	ENSG00000104626.14	ENSG00000283853.1	ENSG00000261379.1
2995	ENSG00000104635.14	ENSG00000283849.1	ENSG00000229840.2
2996	ENSG00000104643.10	ENSG00000283848.1	ENSG00000234702.1
2997	ENSG00000104660.18	ENSG00000283845.1	ENSG00000189184.11
2998	ENSG00000104671.8	ENSG00000283844.1	ENSG00000259735.1
2999	ENSG00000104679.11	ENSG00000283842.1	ENSG00000218730.1
3000	ENSG00000104687.14	ENSG00000283840.1	ENSG00000119718.11
3001	ENSG00000104689.9	ENSG00000283839.1	ENSG00000154864.12
3002	ENSG00000104691.15	ENSG00000283836.1	ENSG00000253515.1
3003	ENSG00000104695.13	ENSG00000283829.1	ENSG00000211731.1
3004	ENSG00000104714.14	ENSG00000283828.1	ENSG00000262339.5
3005	ENSG00000104722.14	ENSG00000283824.1	ENSG00000188316.14
3006	ENSG00000104723.20	ENSG00000283822.1	ENSG00000235881.2
3007	ENSG00000104728.16	ENSG00000283821.1	ENSG00000273727.1
3008	ENSG00000104731.14	ENSG00000283819.1	ENSG00000272703.1
3009	ENSG00000104738.17	ENSG00000283818.1	ENSG00000260809.1
3010	ENSG00000104755.15	ENSG00000283815.1	ENSG00000250111.3
3011	ENSG00000104756.16	ENSG00000283813.1	ENSG00000160948.14
3012	ENSG00000104760.17	ENSG00000283809.1	ENSG00000285777.1
3013	ENSG00000104763.19	ENSG00000283805.1	ENSG00000263167.1
3014	ENSG00000104765.16	ENSG00000283803.1	ENSG00000269927.1
3015	ENSG00000104774.13	ENSG00000283801.1	ENSG00000211821.2
3016	ENSG00000104783.13	ENSG00000283799.1	ENSG00000228789.7
3017	ENSG00000104804.7	ENSG00000283798.1	ENSG00000244671.3
3018	ENSG00000104805.16	ENSG00000283797.1	ENSG00000185127.6

3019	ENSG00000104808.8	ENSG00000283796.1	ENSG00000229525.1
3020	ENSG00000104812.15	ENSG00000283795.1	ENSG00000250769.2
3021	ENSG00000104814.13	ENSG00000283793.1	ENSG00000274723.1
3022	ENSG00000104818.14	ENSG00000283792.1	ENSG00000237833.1
3023	ENSG00000104823.9	ENSG00000283791.1	ENSG00000270409.1
3024	ENSG00000104824.17	ENSG00000283789.1	ENSG00000278459.1
3025	ENSG00000104825.17	ENSG00000283788.1	ENSG00000217767.2
3026	ENSG00000104826.13	ENSG00000283787.1	ENSG00000229668.1
3027	ENSG00000104827.12	ENSG00000283785.1	ENSG00000284969.1
3028	ENSG00000104833.12	ENSG00000283783.1	ENSG00000117122.13
3029	ENSG00000104835.14	ENSG00000283782.2	ENSG00000146013.11
3030	ENSG00000104848.1	ENSG00000283776.1	ENSG00000285606.1
3031	ENSG00000104852.15	ENSG00000283775.1	ENSG00000104974.11
3032	ENSG00000104853.16	ENSG00000283774.1	ENSG00000231880.2
3033	ENSG00000104856.14	ENSG00000283773.1	ENSG00000243250.1
3034	ENSG00000104859.15	ENSG00000283772.1	ENSG00000232111.2
3035	ENSG00000104863.12	ENSG00000283770.1	ENSG00000228809.2
3036	ENSG00000104866.11	ENSG00000283769.1	ENSG00000232039.2
3037	ENSG00000104870.12	ENSG00000283768.1	ENSG00000227297.1
3038	ENSG00000104872.11	ENSG00000283766.1	ENSG00000197213.9
3039	ENSG00000104879.5	ENSG00000283765.1	ENSG00000153157.13
3040	ENSG00000104880.17	ENSG00000283764.1	ENSG00000252212.1
3041	ENSG00000104881.16	ENSG00000283762.1	ENSG00000101624.10
3042	ENSG00000104883.8	ENSG00000283761.1	ENSG00000256349.1
3043	ENSG00000104884.15	ENSG00000283759.1	ENSG00000249238.1
3044	ENSG00000104885.18	ENSG00000283758.2	ENSG00000284902.1
3045	ENSG00000104886.11	ENSG00000283757.1	ENSG00000233851.1
3046	ENSG00000104888.10	ENSG00000283755.2	ENSG00000260371.1
3047	ENSG00000104889.6	ENSG00000283752.1	ENSG00000182521.5
3048	ENSG00000104892.17	ENSG00000283751.1	ENSG00000152229.18
3049	ENSG00000104894.12	ENSG00000283749.1	ENSG00000258422.5
3050	ENSG00000104897.10	ENSG00000283745.1	ENSG00000259383.1
3051	ENSG00000104899.7	ENSG00000283744.1	ENSG00000284607.1
3052	ENSG00000104901.6	ENSG00000283743.2	ENSG00000236107.9
3053	ENSG00000104903.4	ENSG00000283740.1	ENSG00000237531.6
3054	ENSG00000104904.12	ENSG00000283737.1	ENSG00000237636.2
3055	ENSG00000104907.12	ENSG00000283736.1	ENSG00000197595.4
3056	ENSG00000104915.15	ENSG00000283734.1	ENSG00000169439.12
3057	ENSG00000104918.8	ENSG00000283733.1	ENSG00000058063.16
3058	ENSG00000104921.15	ENSG00000283728.1	ENSG00000225173.1
3059	ENSG00000104936.17	ENSG00000283726.1	ENSG00000232215.2
3060	ENSG00000104938.16	ENSG00000283724.1	ENSG00000134216.19
3061	ENSG00000104941.8	ENSG00000283721.1	ENSG00000232273.1
3062	ENSG00000104946.13	ENSG00000283717.1	ENSG00000277494.2
3063	ENSG00000104951.16	ENSG00000283712.1	ENSG00000219188.1
3064	ENSG00000104953.20	ENSG00000283710.1	ENSG00000284662.1
3065	ENSG00000104957.14	ENSG00000283709.1	ENSG00000243888.1
3066	ENSG00000104960.15	ENSG00000283706.2	ENSG00000087128.10
3067	ENSG00000104964.14	ENSG00000283705.1	ENSG00000243014.1
3068	ENSG00000104967.7	ENSG00000283704.1	ENSG00000186431.19
3069	ENSG00000104969.10	ENSG00000283703.2	ENSG00000250983.1
3070	ENSG00000104970.11	ENSG00000283701.1	ENSG00000268095.1
3071	ENSG00000104972.15	ENSG00000283699.1	ENSG00000204065.3

3072	ENSG00000104973.18	ENSG00000283698.1	ENSG00000215796.3
3073	ENSG00000104974.11	ENSG00000283697.2	ENSG00000279795.1
3074	ENSG00000104976.12	ENSG00000283696.1	ENSG00000253079.1
3075	ENSG00000104979.9	ENSG00000283695.1	ENSG00000077274.9
3076	ENSG00000104980.8	ENSG00000283694.1	ENSG00000126012.11
3077	ENSG00000104983.8	ENSG00000283692.1	ENSG00000133136.4
3078	ENSG00000104998.4	ENSG00000283691.1	ENSG00000269274.1
3079	ENSG00000105011.9	ENSG00000283690.1	ENSG00000278099.1
3080	ENSG00000105048.17	ENSG00000283689.1	ENSG00000169083.16
3081	ENSG00000105053.11	ENSG00000283688.1	ENSG00000128923.11
3082	ENSG00000105058.12	ENSG00000283686.2	ENSG00000243870.3
3083	ENSG00000105063.19	ENSG00000283685.1	ENSG00000235058.1
3084	ENSG00000105072.9	ENSG00000283684.1	ENSG00000242531.1
3085	ENSG00000105085.10	ENSG00000283683.1	ENSG00000149346.15
3086	ENSG00000105088.8	ENSG00000283682.1	ENSG00000254129.1
3087	ENSG00000105122.13	ENSG00000283680.2	ENSG00000250387.2
3088	ENSG00000105127.9	ENSG00000283679.1	ENSG00000227877.6
3089	ENSG00000105131.8	ENSG00000283678.1	ENSG00000153923.10
3090	ENSG00000105135.16	ENSG00000283677.1	ENSG00000233916.1
3091	ENSG00000105136.20	ENSG00000283676.1	ENSG00000255713.2
3092	ENSG00000105137.13	ENSG00000283675.1	ENSG00000226191.3
3093	ENSG00000105141.6	ENSG00000283674.2	ENSG00000253485.2
3094	ENSG00000105143.12	ENSG00000283673.1	ENSG00000248854.1
3095	ENSG00000105146.12	ENSG00000283672.1	ENSG00000276019.1
3096	ENSG00000105171.10	ENSG00000283669.1	ENSG00000179751.6
3097	ENSG00000105173.14	ENSG00000283667.1	ENSG00000066827.16
3098	ENSG00000105176.18	ENSG00000283666.1	ENSG00000276662.1
3099	ENSG00000105185.12	ENSG00000283665.1	ENSG00000224785.1
3100	ENSG00000105186.16	ENSG00000283664.1	ENSG00000231995.2
3101	ENSG00000105193.9	ENSG00000283663.1	ENSG00000285846.1
3102	ENSG00000105197.11	ENSG00000283662.1	ENSG00000265684.2
3103	ENSG00000105198.11	ENSG00000283659.1	ENSG00000258907.2
3104	ENSG00000105202.9	ENSG00000283657.1	ENSG00000256944.1
3105	ENSG00000105204.14	ENSG00000283656.1	ENSG00000241739.1
3106	ENSG00000105205.7	ENSG00000283654.3	ENSG00000279154.1
3107	ENSG00000105219.10	ENSG00000283653.1	ENSG00000225442.2
3108	ENSG00000105220.16	ENSG00000283648.1	ENSG00000225505.1
3109	ENSG00000105221.17	ENSG00000283647.1	ENSG00000280200.1
3110	ENSG00000105223.20	ENSG00000283646.1	ENSG00000214362.2
3111	ENSG00000105227.14	ENSG00000283645.1	ENSG00000007062.11
3112	ENSG00000105229.7	ENSG00000283644.1	ENSG00000143412.10
3113	ENSG00000105245.9	ENSG00000283639.1	ENSG00000123685.9
3114	ENSG00000105246.6	ENSG00000283638.2	ENSG00000111218.11
3115	ENSG00000105248.16	ENSG00000283637.1	ENSG00000005844.17
3116	ENSG00000105251.10	ENSG00000283636.1	ENSG00000169618.6
3117	ENSG00000105254.12	ENSG00000283635.1	ENSG00000223931.2
3118	ENSG00000105255.11	ENSG00000283634.1	ENSG00000242590.1
3119	ENSG00000105258.9	ENSG00000283633.1	ENSG00000275722.5
3120	ENSG00000105261.7	ENSG00000283632.2	ENSG00000156042.17
3121	ENSG00000105270.15	ENSG00000283631.1	ENSG00000133937.4
3122	ENSG00000105278.11	ENSG00000283629.1	ENSG00000260234.5
3123	ENSG00000105281.12	ENSG00000283627.1	ENSG00000182134.16
3124	ENSG00000105287.12	ENSG00000283626.1	ENSG00000197653.15

3125	ENSG00000105289.15	ENSG00000283625.1	ENSG00000184905.9
3126	ENSG00000105290.12	ENSG00000283622.1	ENSG00000267791.1
3127	ENSG00000105298.14	ENSG00000283621.1	ENSG00000233108.1
3128	ENSG00000105321.14	ENSG00000283618.1	ENSG00000207175.1
3129	ENSG00000105323.16	ENSG00000283616.1	ENSG00000235821.1
3130	ENSG00000105325.14	ENSG00000283615.1	ENSG00000228386.2
3131	ENSG00000105327.17	ENSG00000283614.1	ENSG00000234315.1
3132	ENSG00000105329.9	ENSG00000283613.1	ENSG00000150750.7
3133	ENSG00000105339.10	ENSG00000283612.1	ENSG00000254694.1
3134	ENSG00000105341.18	ENSG00000283611.2	ENSG00000254221.2
3135	ENSG00000105352.10	ENSG00000283609.1	ENSG00000186452.11
3136	ENSG00000105355.9	ENSG00000283608.1	ENSG00000259293.1
3137	ENSG00000105357.16	ENSG00000283607.2	ENSG00000255063.1
3138	ENSG00000105364.13	ENSG00000283605.1	ENSG00000234025.1
3139	ENSG00000105366.15	ENSG00000283604.1	ENSG00000232542.1
3140	ENSG00000105369.9	ENSG00000283603.1	ENSG00000229368.1
3141	ENSG00000105370.7	ENSG00000283602.1	ENSG00000238278.3
3142	ENSG00000105371.10	ENSG00000283601.1	ENSG00000123999.5
3143	ENSG00000105372.7	ENSG00000283599.2	ENSG00000204711.9
3144	ENSG00000105373.19	ENSG00000283598.1	ENSG00000276788.1
3145	ENSG00000105374.10	ENSG00000283597.2	ENSG00000260545.1
3146	ENSG00000105376.5	ENSG00000283594.1	ENSG00000130702.15
3147	ENSG00000105379.9	ENSG00000283592.1	ENSG00000211979.2
3148	ENSG00000105383.15	ENSG00000283591.1	ENSG00000179420.11
3149	ENSG00000105388.15	ENSG00000283588.1	ENSG00000150977.10
3150	ENSG00000105392.16	ENSG00000283587.1	ENSG00000143125.6
3151	ENSG00000105393.16	ENSG00000283586.1	ENSG00000267689.1
3152	ENSG00000105397.13	ENSG00000283584.1	ENSG00000162946.22
3153	ENSG00000105398.4	ENSG00000283583.1	ENSG00000260493.1
3154	ENSG00000105401.9	ENSG00000283582.1	ENSG00000270068.1
3155	ENSG00000105402.8	ENSG00000283580.3	ENSG00000267535.1
3156	ENSG00000105404.11	ENSG00000283579.1	ENSG00000285543.1
3157	ENSG00000105409.18	ENSG00000283578.1	ENSG00000259547.1
3158	ENSG00000105419.17	ENSG00000283576.1	ENSG00000198844.12
3159	ENSG00000105426.16	ENSG00000283575.1	ENSG00000277693.1
3160	ENSG00000105427.10	ENSG00000283573.1	ENSG00000173421.17
3161	ENSG00000105428.5	ENSG00000283572.1	ENSG00000264662.1
3162	ENSG00000105429.13	ENSG00000283571.1	ENSG00000171928.14
3163	ENSG00000105438.9	ENSG00000283569.1	ENSG00000229228.1
3164	ENSG00000105443.15	ENSG00000283568.1	ENSG00000111245.14
3165	ENSG00000105447.12	ENSG00000283567.1	ENSG00000183580.10
3166	ENSG00000105464.3	ENSG00000283566.2	ENSG00000268987.1
3167	ENSG00000105467.8	ENSG00000283564.1	ENSG00000101812.12
3168	ENSG00000105472.13	ENSG00000283563.1	ENSG00000268066.5
3169	ENSG00000105479.15	ENSG00000283562.1	ENSG00000163331.11
3170	ENSG00000105483.18	ENSG00000283561.1	ENSG00000253666.1
3171	ENSG00000105486.14	ENSG00000283559.1	ENSG00000229694.6
3172	ENSG00000105492.16	ENSG00000283558.1	ENSG00000169562.10
3173	ENSG00000105497.8	ENSG00000283556.1	ENSG00000259262.1
3174	ENSG00000105499.14	ENSG00000283555.1	ENSG00000226397.8
3175	ENSG00000105501.12	ENSG00000283554.1	ENSG00000257987.5
3176	ENSG00000105507.2	ENSG00000283551.1	ENSG00000226212.2
3177	ENSG00000105509.10	ENSG00000283550.1	ENSG00000204588.5

3178	ENSG00000105514.8	ENSG00000283549.1	ENSG00000159958.6
3179	ENSG00000105516.10	ENSG00000283547.1	ENSG00000241963.3
3180	ENSG00000105518.14	ENSG00000283546.1	ENSG00000163530.4
3181	ENSG00000105519.16	ENSG00000283545.1	ENSG00000265888.1
3182	ENSG00000105520.10	ENSG00000283544.1	ENSG00000151150.22
3183	ENSG00000105523.3	ENSG00000283542.1	ENSG00000261334.1
3184	ENSG00000105538.10	ENSG00000283541.1	ENSG00000197575.6
3185	ENSG00000105549.10	ENSG00000283540.1	ENSG00000227240.1
3186	ENSG00000105550.9	ENSG00000283538.2	ENSG00000251633.3
3187	ENSG00000105552.15	ENSG00000283537.2	ENSG00000230552.5
3188	ENSG00000105556.11	ENSG00000283536.2	ENSG00000229732.1
3189	ENSG00000105559.12	ENSG00000283535.1	ENSG00000112787.13
3190	ENSG00000105568.18	ENSG00000283534.1	ENSG00000114738.11
3191	ENSG00000105576.15	ENSG00000283532.1	ENSG00000270157.1
3192	ENSG00000105583.11	ENSG00000283529.1	ENSG00000265443.1
3193	ENSG00000105605.7	ENSG00000283528.2	ENSG00000105701.16
3194	ENSG00000105607.13	ENSG00000283527.1	ENSG00000264589.3
3195	ENSG00000105609.16	ENSG00000283526.1	ENSG00000259407.1
3196	ENSG00000105610.5	ENSG00000283525.1	ENSG00000224157.1
3197	ENSG00000105612.9	ENSG00000283524.2	ENSG00000130054.4
3198	ENSG00000105613.10	ENSG00000283523.1	ENSG00000286075.1
3199	ENSG00000105617.3	ENSG00000283522.1	ENSG00000126778.10
3200	ENSG00000105618.14	ENSG00000283519.1	ENSG00000117155.16
3201	ENSG00000105619.13	ENSG00000283518.1	ENSG00000269994.2
3202	ENSG00000105639.18	ENSG00000283517.1	ENSG00000206625.1
3203	ENSG00000105640.13	ENSG00000283516.1	ENSG00000225297.1
3204	ENSG00000105641.4	ENSG00000283515.1	ENSG00000232549.1
3205	ENSG00000105642.15	ENSG00000283514.1	ENSG00000277559.1
3206	ENSG00000105643.10	ENSG00000283513.1	ENSG00000160191.18
3207	ENSG00000105647.17	ENSG00000283511.1	ENSG00000231212.1
3208	ENSG00000105649.9	ENSG00000283509.1	ENSG00000258626.2
3209	ENSG00000105650.22	ENSG00000283507.1	ENSG00000279064.1
3210	ENSG00000105655.18	ENSG00000283506.1	ENSG00000152953.13
3211	ENSG00000105656.13	ENSG00000283505.1	ENSG00000145087.12
3212	ENSG00000105662.16	ENSG00000283504.1	ENSG00000266397.1
3213	ENSG00000105664.11	ENSG00000283503.1	ENSG00000238934.1
3214	ENSG00000105668.7	ENSG00000283502.1	ENSG00000249558.1
3215	ENSG00000105669.14	ENSG00000283499.1	ENSG00000229089.8
3216	ENSG00000105671.12	ENSG00000283498.1	ENSG00000253534.1
3217	ENSG00000105672.14	ENSG00000283497.1	ENSG00000240996.1
3218	ENSG00000105675.8	ENSG00000283496.1	ENSG00000243905.3
3219	ENSG00000105676.14	ENSG00000283493.1	ENSG00000267800.1
3220	ENSG00000105677.12	ENSG00000283492.1	ENSG00000139219.19
3221	ENSG00000105679.9	ENSG00000283491.1	ENSG00000240163.1
3222	ENSG00000105694.3	ENSG00000283490.1	ENSG00000284842.1
3223	ENSG00000105695.15	ENSG00000283489.1	ENSG00000213540.3
3224	ENSG00000105696.9	ENSG00000283487.1	ENSG00000206693.1
3225	ENSG00000105697.9	ENSG00000283486.2	ENSG00000237557.1
3226	ENSG00000105698.16	ENSG00000283484.1	ENSG00000214297.3
3227	ENSG00000105699.16	ENSG00000283483.1	ENSG00000279746.1
3228	ENSG00000105700.11	ENSG00000283481.1	ENSG00000263335.1
3229	ENSG00000105701.16	ENSG00000283480.1	ENSG00000210195.2
3230	ENSG00000105705.16	ENSG00000283479.1	ENSG00000261070.1

3231	ENSG00000105707.13	ENSG00000283477.1	ENSG00000173065.13
3232	ENSG00000105708.9	ENSG00000283476.1	ENSG00000272360.1
3233	ENSG00000105711.12	ENSG00000283475.1	ENSG00000271654.1
3234	ENSG00000105717.14	ENSG00000283474.1	ENSG00000133112.16
3235	ENSG00000105722.10	ENSG00000283473.3	ENSG00000259258.1
3236	ENSG00000105723.12	ENSG00000283471.1	ENSG00000278570.4
3237	ENSG00000105726.17	ENSG00000283469.1	ENSG00000232536.1
3238	ENSG00000105732.13	ENSG00000283468.1	ENSG00000257096.1
3239	ENSG00000105737.9	ENSG00000283464.1	ENSG00000135111.16
3240	ENSG00000105738.11	ENSG00000283463.1	ENSG00000274330.1
3241	ENSG00000105750.15	ENSG00000283462.1	ENSG00000162814.11
3242	ENSG00000105755.8	ENSG00000283461.1	ENSG00000260425.1
3243	ENSG00000105767.3	ENSG00000283459.1	ENSG00000244743.1
3244	ENSG00000105771.14	ENSG00000283458.1	ENSG00000235070.3
3245	ENSG00000105778.19	ENSG00000283457.1	ENSG00000284948.1
3246	ENSG00000105784.15	ENSG00000283456.1	ENSG00000233871.2
3247	ENSG00000105792.19	ENSG00000283455.1	ENSG00000220643.1
3248	ENSG00000105793.15	ENSG00000283453.2	ENSG00000276168.1
3249	ENSG00000105808.17	ENSG00000283452.1	ENSG00000186496.12
3250	ENSG00000105810.9	ENSG00000283451.1	ENSG00000149474.13
3251	ENSG00000105819.14	ENSG00000283450.1	ENSG00000261537.1
3252	ENSG00000105821.15	ENSG00000283446.1	ENSG00000149435.12
3253	ENSG00000105825.13	ENSG00000283445.1	ENSG00000203818.7
3254	ENSG00000105829.13	ENSG00000283444.1	ENSG00000273025.1
3255	ENSG00000105835.12	ENSG00000283443.1	ENSG00000187957.8
3256	ENSG00000105849.6	ENSG00000283442.1	ENSG00000273321.1
3257	ENSG00000105851.11	ENSG00000283441.1	ENSG00000241313.2
3258	ENSG00000105852.11	ENSG00000283440.1	ENSG00000240253.6
3259	ENSG00000105854.13	ENSG00000283439.3	ENSG00000230613.1
3260	ENSG00000105855.10	ENSG00000283438.1	ENSG00000272741.1
3261	ENSG00000105856.14	ENSG00000283436.1	ENSG00000268651.3
3262	ENSG00000105865.11	ENSG00000283435.1	ENSG00000148584.15
3263	ENSG00000105866.15	ENSG00000283434.1	ENSG00000267686.1
3264	ENSG00000105875.14	ENSG00000283433.1	ENSG00000233694.5
3265	ENSG00000105877.18	ENSG00000283432.1	ENSG00000269307.1
3266	ENSG00000105879.12	ENSG00000283431.1	ENSG00000198312.4
3267	ENSG00000105880.7	ENSG00000283429.1	ENSG00000215274.5
3268	ENSG00000105887.11	ENSG00000283428.1	ENSG00000261723.1
3269	ENSG00000105889.15	ENSG00000283427.1	ENSG00000225098.1
3270	ENSG00000105894.11	ENSG00000283426.1	ENSG00000273093.1
3271	ENSG00000105926.15	ENSG00000283423.1	ENSG00000078304.19
3272	ENSG00000105928.15	ENSG00000283422.1	ENSG00000253943.1
3273	ENSG00000105929.16	ENSG00000283421.1	ENSG00000121068.14
3274	ENSG00000105939.13	ENSG00000283420.1	ENSG00000264914.1
3275	ENSG00000105948.13	ENSG00000283419.1	ENSG00000232164.1
3276	ENSG00000105953.15	ENSG00000283418.1	ENSG00000233073.1
3277	ENSG00000105954.2	ENSG00000283417.1	ENSG00000168405.17
3278	ENSG00000105963.15	ENSG00000283416.1	ENSG00000145730.20
3279	ENSG00000105967.16	ENSG00000283415.1	ENSG00000232467.2
3280	ENSG00000105968.18	ENSG00000283414.1	ENSG00000187372.11
3281	ENSG00000105971.15	ENSG00000283413.1	ENSG00000099984.11
3282	ENSG00000105974.12	ENSG00000283412.1	ENSG00000236512.1
3283	ENSG00000105982.16	ENSG00000283411.1	ENSG00000272798.1

3284	ENSG00000105983.21	ENSG00000283409.1	ENSG00000239528.1
3285	ENSG00000105988.6	ENSG00000283408.1	ENSG00000237623.1
3286	ENSG00000105989.9	ENSG00000283405.1	ENSG00000232463.1
3287	ENSG00000105991.8	ENSG00000283403.1	ENSG00000252289.1
3288	ENSG00000105993.15	ENSG00000283402.1	ENSG00000265010.1
3289	ENSG00000105996.7	ENSG00000283400.1	ENSG00000265844.1
3290	ENSG00000105997.22	ENSG00000283399.1	ENSG00000188306.6
3291	ENSG00000106003.13	ENSG00000283398.1	ENSG00000237125.9
3292	ENSG00000106004.5	ENSG00000283394.1	ENSG00000206749.1
3293	ENSG00000106006.6	ENSG00000283393.1	ENSG00000250706.1
3294	ENSG00000106009.16	ENSG00000283392.1	ENSG00000198555.7
3295	ENSG00000106012.18	ENSG00000283390.1	ENSG00000230342.2
3296	ENSG00000106013.15	ENSG00000283389.1	ENSG00000268364.1
3297	ENSG00000106018.14	ENSG00000283386.1	ENSG00000276500.1
3298	ENSG00000106025.8	ENSG00000283385.1	ENSG00000280639.1
3299	ENSG00000106028.11	ENSG00000283384.1	ENSG00000080823.23
3300	ENSG00000106031.8	ENSG00000283383.1	ENSG00000257674.1
3301	ENSG00000106034.18	ENSG00000283381.1	ENSG00000259687.1
3302	ENSG00000106038.13	ENSG00000283380.1	ENSG00000220694.2
3303	ENSG00000106049.9	ENSG00000283379.1	ENSG00000279960.1
3304	ENSG00000106052.13	ENSG00000283378.1	ENSG00000172318.5
3305	ENSG00000106066.15	ENSG00000283377.1	ENSG00000224040.1
3306	ENSG00000106069.22	ENSG00000283376.1	ENSG00000123171.6
3307	ENSG00000106070.19	ENSG00000283375.1	ENSG00000125975.13
3308	ENSG00000106077.18	ENSG00000283372.1	ENSG00000234485.5
3309	ENSG00000106078.19	ENSG00000283371.1	ENSG00000165119.21
3310	ENSG00000106080.10	ENSG00000283369.1	ENSG00000236710.1
3311	ENSG00000106086.20	ENSG00000283367.1	ENSG00000100568.11
3312	ENSG00000106089.12	ENSG00000283366.2	ENSG00000186190.7
3313	ENSG00000106100.11	ENSG00000283365.1	ENSG00000235077.1
3314	ENSG00000106105.14	ENSG00000283364.1	ENSG00000206739.1
3315	ENSG00000106113.19	ENSG00000283361.2	ENSG00000239465.1
3316	ENSG00000106123.12	ENSG00000283360.1	ENSG00000236683.3
3317	ENSG00000106125.14	ENSG00000283359.1	ENSG00000286067.1
3318	ENSG00000106128.19	ENSG00000283356.1	ENSG00000251196.1
3319	ENSG00000106133.18	ENSG00000283355.1	ENSG00000260954.1
3320	ENSG00000106144.20	ENSG00000283354.1	ENSG00000181017.5
3321	ENSG00000106153.13	ENSG00000283352.1	ENSG00000285373.1
3322	ENSG00000106178.6	ENSG00000283351.1	ENSG00000214584.3
3323	ENSG00000106211.9	ENSG00000283349.3	ENSG00000176406.22
3324	ENSG00000106236.4	ENSG00000283347.1	ENSG00000234956.6
3325	ENSG00000106244.13	ENSG00000283346.1	ENSG00000266957.1
3326	ENSG00000106245.11	ENSG00000283345.1	ENSG00000176269.3
3327	ENSG00000106246.17	ENSG00000283343.1	ENSG00000235256.1
3328	ENSG00000106258.15	ENSG00000283342.1	ENSG00000265929.1
3329	ENSG00000106261.17	ENSG00000283341.1	ENSG00000080618.15
3330	ENSG00000106263.18	ENSG00000283340.1	ENSG00000226516.7
3331	ENSG00000106266.11	ENSG00000283339.1	ENSG00000260860.2
3332	ENSG00000106268.15	ENSG00000283338.1	ENSG00000266821.1
3333	ENSG00000106278.12	ENSG00000283337.1	ENSG00000201659.1
3334	ENSG00000106290.15	ENSG00000283335.1	ENSG00000138658.15
3335	ENSG00000106299.8	ENSG00000283334.1	ENSG00000064195.7
3336	ENSG00000106302.9	ENSG00000283333.1	ENSG00000248358.2

3337	ENSG00000106304.15	ENSG00000283330.1	ENSG00000200651.1
3338	ENSG00000106305.10	ENSG00000283329.1	ENSG00000233340.1
3339	ENSG00000106327.13	ENSG00000283327.1	ENSG00000213130.3
3340	ENSG00000106328.9	ENSG00000283326.1	ENSG00000128965.13
3341	ENSG00000106330.12	ENSG00000283324.1	ENSG00000242279.1
3342	ENSG00000106331.16	ENSG00000283321.1	ENSG00000236794.5
3343	ENSG00000106333.13	ENSG00000283320.1	ENSG00000076067.13
3344	ENSG00000106336.13	ENSG00000283317.1	ENSG00000285649.1
3345	ENSG00000106344.8	ENSG00000283314.1	ENSG00000161800.13
3346	ENSG00000106346.11	ENSG00000283313.1	ENSG00000188603.19
3347	ENSG00000106348.18	ENSG00000283312.1	ENSG00000226081.2
3348	ENSG00000106351.13	ENSG00000283311.1	ENSG00000271507.1
3349	ENSG00000106355.10	ENSG00000283307.1	ENSG00000258886.2
3350	ENSG00000106366.8	ENSG00000283304.1	ENSG00000272071.1
3351	ENSG00000106367.15	ENSG00000283303.1	ENSG00000233852.1
3352	ENSG00000106384.12	ENSG00000283301.1	ENSG00000206932.1
3353	ENSG00000106392.10	ENSG00000283300.1	ENSG00000172425.10
3354	ENSG00000106397.12	ENSG00000283298.1	ENSG00000283563.1
3355	ENSG00000106399.11	ENSG00000283297.1	ENSG00000224545.1
3356	ENSG00000106400.12	ENSG00000283296.1	ENSG00000255079.1
3357	ENSG00000106404.13	ENSG00000283294.1	ENSG00000234115.2
3358	ENSG00000106410.15	ENSG00000283293.1	ENSG00000161179.13
3359	ENSG00000106415.13	ENSG00000283291.1	ENSG00000267193.5
3360	ENSG00000106436.6	ENSG00000283290.1	ENSG00000143153.12
3361	ENSG00000106443.16	ENSG00000283289.1	ENSG00000225952.1
3362	ENSG00000106459.15	ENSG00000283288.1	ENSG00000230524.8
3363	ENSG00000106460.19	ENSG00000283286.1	ENSG00000275785.1
3364	ENSG00000106462.10	ENSG00000283285.1	ENSG00000278690.1
3365	ENSG00000106477.19	ENSG00000283283.2	ENSG00000267168.1
3366	ENSG00000106479.11	ENSG00000283281.1	ENSG00000281344.1
3367	ENSG00000106483.12	ENSG00000283279.1	ENSG00000279226.1
3368	ENSG00000106484.15	ENSG00000283278.1	ENSG00000264296.1
3369	ENSG00000106511.6	ENSG00000283275.1	ENSG00000271986.2
3370	ENSG00000106524.9	ENSG00000283274.1	ENSG00000010017.13
3371	ENSG00000106526.10	ENSG00000283273.1	ENSG00000249545.1
3372	ENSG00000106536.19	ENSG00000283271.1	ENSG00000264243.1
3373	ENSG00000106537.8	ENSG00000283270.1	ENSG00000285630.1
3374	ENSG00000106538.10	ENSG00000283269.1	ENSG00000277677.1
3375	ENSG00000106540.4	ENSG00000283268.1	ENSG00000167800.9
3376	ENSG00000106541.12	ENSG00000283267.1	ENSG00000166796.12
3377	ENSG00000106546.14	ENSG00000283265.1	ENSG00000129988.6
3378	ENSG00000106554.13	ENSG00000283262.1	ENSG00000228844.1
3379	ENSG00000106560.11	ENSG00000283259.1	ENSG00000188153.13
3380	ENSG00000106565.18	ENSG00000283258.1	ENSG00000201533.1
3381	ENSG00000106571.14	ENSG00000283257.1	ENSG00000229922.5
3382	ENSG00000106588.11	ENSG00000283256.1	ENSG00000286091.1
3383	ENSG00000106591.4	ENSG00000283255.1	ENSG00000267394.1
3384	ENSG00000106603.18	ENSG00000283251.1	ENSG00000184985.16
3385	ENSG00000106605.11	ENSG00000283249.1	ENSG00000267205.1
3386	ENSG00000106608.16	ENSG00000283247.2	ENSG00000229822.2
3387	ENSG00000106609.16	ENSG00000283246.1	ENSG00000240873.1
3388	ENSG00000106610.15	ENSG00000283242.1	ENSG00000283829.1
3389	ENSG00000106615.10	ENSG00000283240.1	ENSG00000066279.18

3390	ENSG00000106617.14	ENSG00000283239.1	ENSG00000239736.2
3391	ENSG00000106624.11	ENSG00000283238.1	ENSG00000271137.1
3392	ENSG00000106628.10	ENSG00000283237.1	ENSG00000164619.10
3393	ENSG00000106631.8	ENSG00000283236.1	ENSG00000231804.1
3394	ENSG00000106633.16	ENSG00000283235.1	ENSG00000219700.1
3395	ENSG00000106635.8	ENSG00000283232.1	ENSG00000009765.14
3396	ENSG00000106636.8	ENSG00000283230.1	ENSG00000185739.13
3397	ENSG00000106638.16	ENSG00000283228.1	ENSG00000277637.1
3398	ENSG00000106648.14	ENSG00000283227.1	ENSG00000231300.1
3399	ENSG00000106665.15	ENSG00000283225.1	ENSG00000250127.1
3400	ENSG00000106682.14	ENSG00000283222.1	ENSG00000226048.1
3401	ENSG00000106683.15	ENSG00000283221.1	ENSG00000243234.1
3402	ENSG00000106686.16	ENSG00000283219.1	ENSG00000234944.1
3403	ENSG00000106688.12	ENSG00000283218.1	ENSG00000267396.1
3404	ENSG00000106689.11	ENSG00000283217.1	ENSG00000173404.5
3405	ENSG00000106692.14	ENSG00000283215.2	ENSG00000239468.3
3406	ENSG00000106701.12	ENSG00000283214.1	ENSG00000280420.1
3407	ENSG00000106714.17	ENSG00000283213.1	ENSG00000138050.15
3408	ENSG00000106723.17	ENSG00000283212.1	ENSG00000234208.1
3409	ENSG00000106733.21	ENSG00000283211.1	ENSG00000273133.1
3410	ENSG00000106771.13	ENSG00000283210.1	ENSG00000068781.21
3411	ENSG00000106772.18	ENSG00000283209.1	ENSG00000241069.1
3412	ENSG00000106780.9	ENSG00000283208.2	ENSG00000230221.2
3413	ENSG00000106785.15	ENSG00000283207.1	ENSG00000266222.1
3414	ENSG00000106789.13	ENSG00000283206.1	ENSG00000163288.13
3415	ENSG00000106799.13	ENSG00000283205.1	ENSG00000124172.10
3416	ENSG00000106803.10	ENSG00000283204.1	ENSG00000104852.15
3417	ENSG00000106804.7	ENSG00000283203.1	ENSG00000272265.1
3418	ENSG00000106809.11	ENSG00000283202.1	ENSG00000260576.1
3419	ENSG00000106819.12	ENSG00000283201.1	ENSG00000261193.1
3420	ENSG00000106823.12	ENSG00000283200.1	ENSG00000264281.3
3421	ENSG00000106829.18	ENSG00000283199.2	ENSG00000128313.2
3422	ENSG00000106852.15	ENSG00000283197.1	ENSG00000272281.6
3423	ENSG00000106853.20	ENSG00000283196.2	ENSG00000197905.9
3424	ENSG00000106868.16	ENSG00000283195.1	ENSG00000257119.1
3425	ENSG00000106927.12	ENSG00000283193.1	ENSG00000241572.1
3426	ENSG00000106948.16	ENSG00000283189.2	ENSG00000162039.15
3427	ENSG00000106952.7	ENSG00000283188.1	ENSG00000257150.3
3428	ENSG00000106976.20	ENSG00000283183.1	ENSG00000178860.8
3429	ENSG00000106991.13	ENSG00000283180.1	ENSG00000250325.1
3430	ENSG00000106992.19	ENSG00000283178.1	ENSG00000253600.1
3431	ENSG00000106993.12	ENSG00000283176.1	ENSG00000273637.1
3432	ENSG00000107014.9	ENSG00000283175.1	ENSG00000259163.1
3433	ENSG00000107018.8	ENSG00000283174.1	ENSG00000224520.2
3434	ENSG00000107020.10	ENSG00000283172.1	ENSG00000235955.1
3435	ENSG00000107021.16	ENSG00000283170.1	ENSG00000214514.8
3436	ENSG00000107036.12	ENSG00000283167.2	ENSG00000268357.1
3437	ENSG00000107077.18	ENSG00000283166.1	ENSG00000134594.5
3438	ENSG00000107099.15	ENSG00000283165.1	ENSG00000256288.1
3439	ENSG00000107104.18	ENSG00000283164.1	ENSG00000188707.6
3440	ENSG00000107105.15	ENSG00000283162.1	ENSG00000265840.1
3441	ENSG00000107130.10	ENSG00000283160.1	ENSG00000261612.1
3442	ENSG00000107140.16	ENSG00000283159.1	ENSG00000138483.2

3443	ENSG00000107147.13	ENSG00000283157.1	ENSG00000219755.1
3444	ENSG00000107159.13	ENSG00000283156.1	ENSG00000240990.10
3445	ENSG00000107164.16	ENSG00000283155.1	ENSG00000249372.1
3446	ENSG00000107165.12	ENSG00000283154.2	ENSG00000245928.2
3447	ENSG00000107175.12	ENSG00000283152.1	ENSG00000149792.8
3448	ENSG00000107185.9	ENSG00000283149.1	ENSG00000275649.1
3449	ENSG00000107186.16	ENSG00000283148.1	ENSG00000158828.8
3450	ENSG00000107187.16	ENSG00000283146.1	ENSG00000234736.5
3451	ENSG00000107201.10	ENSG00000283145.1	ENSG00000285245.1
3452	ENSG00000107223.13	ENSG00000283142.1	ENSG00000224276.1
3453	ENSG00000107242.18	ENSG00000283141.1	ENSG00000079739.17
3454	ENSG00000107249.23	ENSG00000283138.1	ENSG00000187922.13
3455	ENSG00000107262.22	ENSG00000283136.1	ENSG00000268673.3
3456	ENSG00000107263.18	ENSG00000283133.1	ENSG00000202532.1
3457	ENSG00000107281.10	ENSG00000283132.1	ENSG00000232228.1
3458	ENSG00000107282.8	ENSG00000283131.1	ENSG00000167178.16
3459	ENSG00000107290.14	ENSG00000283130.1	ENSG00000238901.1
3460	ENSG00000107295.10	ENSG00000283128.1	ENSG00000272895.1
3461	ENSG00000107317.13	ENSG00000283126.1	ENSG00000102575.11
3462	ENSG00000107331.17	ENSG00000283125.1	ENSG00000226549.3
3463	ENSG00000107338.10	ENSG00000283123.1	ENSG00000111817.17
3464	ENSG00000107341.5	ENSG00000283122.1	ENSG00000145075.13
3465	ENSG00000107362.13	ENSG00000283118.1	ENSG00000248240.1
3466	ENSG00000107371.13	ENSG00000283117.1	ENSG00000188786.10
3467	ENSG00000107372.13	ENSG00000283110.1	ENSG00000109610.6
3468	ENSG00000107404.20	ENSG00000283108.1	ENSG00000204889.10
3469	ENSG00000107438.9	ENSG00000283103.2	ENSG00000187758.8
3470	ENSG00000107443.16	ENSG00000283101.1	ENSG00000258896.1
3471	ENSG00000107447.8	ENSG00000283098.1	ENSG00000246982.6
3472	ENSG00000107485.17	ENSG00000283097.1	ENSG00000152766.6
3473	ENSG00000107518.18	ENSG00000283096.1	ENSG00000188827.11
3474	ENSG00000107521.19	ENSG00000283095.1	ENSG00000232908.3
3475	ENSG00000107537.14	ENSG00000283093.1	ENSG00000106588.11
3476	ENSG00000107551.21	ENSG00000283088.1	ENSG00000229654.1
3477	ENSG00000107554.17	ENSG00000283084.1	ENSG00000119547.6
3478	ENSG00000107560.12	ENSG00000283083.1	ENSG00000226279.2
3479	ENSG00000107562.16	ENSG00000283078.1	ENSG00000279081.2
3480	ENSG00000107566.14	ENSG00000283076.1	ENSG00000259362.2
3481	ENSG00000107581.13	ENSG00000283075.1	ENSG00000227399.1
3482	ENSG00000107593.17	ENSG00000283072.2	ENSG00000122176.12
3483	ENSG00000107611.15	ENSG00000283071.1	ENSG00000268777.1
3484	ENSG00000107614.22	ENSG00000283069.1	ENSG00000241067.2
3485	ENSG00000107625.13	ENSG00000283065.1	ENSG00000241294.1
3486	ENSG00000107643.16	ENSG00000283063.1	ENSG00000210176.1
3487	ENSG00000107651.13	ENSG00000283061.1	ENSG00000239608.1
3488	ENSG00000107669.17	ENSG00000283058.1	ENSG00000225530.1
3489	ENSG00000107672.15	ENSG00000283057.1	ENSG00000227430.1
3490	ENSG00000107679.14	ENSG00000283053.1	ENSG00000236319.2
3491	ENSG00000107719.9	ENSG00000283052.1	ENSG00000285833.1
3492	ENSG00000107731.12	ENSG00000283051.1	ENSG00000278794.1
3493	ENSG00000107736.20	ENSG00000283050.2	ENSG00000265693.1
3494	ENSG00000107738.20	ENSG00000283047.1	ENSG00000267653.1
3495	ENSG00000107742.13	ENSG00000283045.1	ENSG00000286009.1

3496	ENSG00000107745.19	ENSG00000283044.1	ENSG00000224129.1
3497	ENSG00000107758.15	ENSG00000283043.1	ENSG00000273537.1
3498	ENSG00000107771.17	ENSG00000283041.1	ENSG00000271926.1
3499	ENSG00000107779.13	ENSG00000283040.1	ENSG00000236111.5
3500	ENSG00000107789.16	ENSG00000283039.1	ENSG00000266850.1
3501	ENSG00000107796.13	ENSG00000283036.2	ENSG00000275693.1
3502	ENSG00000107798.18	ENSG00000283033.1	ENSG00000240888.1
3503	ENSG00000107807.13	ENSG00000283031.1	ENSG00000273946.1
3504	ENSG00000107815.10	ENSG00000283025.1	ENSG00000238221.1
3505	ENSG00000107816.17	ENSG00000283023.1	ENSG00000231953.2
3506	ENSG00000107819.13	ENSG00000283020.1	ENSG00000145824.12
3507	ENSG00000107821.14	ENSG00000283016.1	ENSG00000219993.1
3508	ENSG00000107829.14	ENSG00000283010.1	ENSG00000063438.17
3509	ENSG00000107831.12	ENSG00000283005.1	ENSG00000267582.1
3510	ENSG00000107833.10	ENSG00000283003.1	ENSG00000231028.8
3511	ENSG00000107854.6	ENSG00000283001.1	ENSG00000260335.1
3512	ENSG00000107859.10	ENSG00000283000.1	ENSG00000257884.2
3513	ENSG00000107862.4	ENSG00000282998.1	ENSG00000259022.2
3514	ENSG00000107863.18	ENSG00000282997.1	ENSG00000274845.1
3515	ENSG00000107864.15	ENSG00000282996.1	ENSG00000179131.7
3516	ENSG00000107872.12	ENSG00000282995.1	ENSG00000203496.9
3517	ENSG00000107874.11	ENSG00000282994.1	ENSG00000274023.1
3518	ENSG00000107882.11	ENSG00000282993.1	ENSG00000113889.13
3519	ENSG00000107890.16	ENSG00000282989.1	ENSG00000255164.1
3520	ENSG00000107897.19	ENSG00000282988.2	ENSG00000203469.2
3521	ENSG00000107902.14	ENSG00000282987.1	ENSG00000270104.1
3522	ENSG00000107929.14	ENSG00000282980.1	ENSG00000188176.12
3523	ENSG00000107937.19	ENSG00000282978.1	ENSG00000267192.1
3524	ENSG00000107938.18	ENSG00000282977.1	ENSG00000238118.1
3525	ENSG00000107949.17	ENSG00000282975.1	ENSG00000229203.1
3526	ENSG00000107951.14	ENSG00000282973.1	ENSG00000224973.5
3527	ENSG00000107954.10	ENSG00000282968.1	ENSG00000261707.1
3528	ENSG00000107957.16	ENSG00000282965.1	ENSG00000267606.1
3529	ENSG00000107959.16	ENSG00000282964.1	ENSG00000225496.1
3530	ENSG00000107960.11	ENSG00000282961.1	ENSG00000272397.1
3531	ENSG00000107968.10	ENSG00000282952.1	ENSG00000231184.3
3532	ENSG00000107984.10	ENSG00000282951.1	ENSG00000242325.1
3533	ENSG00000108001.13	ENSG00000282950.1	ENSG00000266931.2
3534	ENSG00000108010.12	ENSG00000282949.1	ENSG00000243022.1
3535	ENSG00000108018.15	ENSG00000282946.1	ENSG00000214843.3
3536	ENSG00000108021.20	ENSG00000282944.1	ENSG00000119965.13
3537	ENSG00000108039.18	ENSG00000282943.1	ENSG00000239742.3
3538	ENSG00000108055.9	ENSG00000282939.1	ENSG00000255487.1
3539	ENSG00000108061.11	ENSG00000282936.1	ENSG00000187857.4
3540	ENSG00000108064.11	ENSG00000282935.1	ENSG00000260118.1
3541	ENSG00000108091.11	ENSG00000282933.2	ENSG00000187912.11
3542	ENSG00000108094.14	ENSG00000282927.1	ENSG00000251648.2
3543	ENSG00000108100.18	ENSG00000282925.1	ENSG00000260011.2
3544	ENSG00000108106.14	ENSG00000282924.1	ENSG00000105143.12
3545	ENSG00000108107.14	ENSG00000282921.1	ENSG00000269085.1
3546	ENSG00000108175.17	ENSG00000282917.1	ENSG00000278872.1
3547	ENSG00000108176.15	ENSG00000282916.1	ENSG00000213917.2
3548	ENSG00000108179.14	ENSG00000282915.1	ENSG00000263776.1

3549	ENSG00000108187.16	ENSG00000282914.1	ENSG00000163016.10
3550	ENSG00000108219.15	ENSG00000282912.1	ENSG00000276266.1
3551	ENSG00000108231.13	ENSG00000282911.1	ENSG00000234504.2
3552	ENSG00000108239.8	ENSG00000282909.2	ENSG00000267226.2
3553	ENSG00000108242.13	ENSG00000282907.1	ENSG00000261444.1
3554	ENSG00000108244.16	ENSG00000282906.1	ENSG00000254867.1
3555	ENSG00000108255.7	ENSG00000282904.1	ENSG00000258774.1
3556	ENSG00000108256.9	ENSG00000282898.1	ENSG00000231612.1
3557	ENSG00000108262.15	ENSG00000282897.1	ENSG00000262038.1
3558	ENSG00000108298.11	ENSG00000282894.1	ENSG00000182824.7
3559	ENSG00000108306.13	ENSG00000282890.1	ENSG00000235071.1
3560	ENSG00000108309.14	ENSG00000282887.2	ENSG00000239122.1
3561	ENSG00000108312.15	ENSG00000282886.1	ENSG00000099998.17
3562	ENSG00000108342.12	ENSG00000282885.1	ENSG00000236032.3
3563	ENSG00000108344.15	ENSG00000282882.1	ENSG00000173930.9
3564	ENSG00000108349.17	ENSG00000282881.2	ENSG00000134775.15
3565	ENSG00000108352.12	ENSG00000282879.1	ENSG00000268193.5
3566	ENSG00000108370.17	ENSG00000282876.1	ENSG00000274067.1
3567	ENSG00000108375.12	ENSG00000282875.1	ENSG00000140600.17
3568	ENSG00000108379.10	ENSG00000282872.1	ENSG00000244791.2
3569	ENSG00000108381.11	ENSG00000282870.1	ENSG00000224007.1
3570	ENSG00000108384.15	ENSG00000282865.1	ENSG00000219608.3
3571	ENSG00000108387.14	ENSG00000282864.1	ENSG00000232162.1
3572	ENSG00000108389.9	ENSG00000282863.1	ENSG00000137168.8
3573	ENSG00000108395.14	ENSG00000282860.1	ENSG00000186523.14
3574	ENSG00000108405.4	ENSG00000282859.1	ENSG00000234277.2
3575	ENSG00000108406.10	ENSG00000282855.1	ENSG00000157152.17
3576	ENSG00000108417.3	ENSG00000282852.1	ENSG00000259474.1
3577	ENSG00000108423.15	ENSG00000282851.2	ENSG00000235044.1
3578	ENSG00000108424.10	ENSG00000282850.2	ENSG00000248521.1
3579	ENSG00000108433.16	ENSG00000282849.1	ENSG00000261291.1
3580	ENSG00000108439.10	ENSG00000282843.1	ENSG00000100292.17
3581	ENSG00000108442.2	ENSG00000282842.1	ENSG00000205126.2
3582	ENSG00000108443.14	ENSG00000282840.1	ENSG00000241913.1
3583	ENSG00000108448.21	ENSG00000282836.1	ENSG00000241469.8
3584	ENSG00000108452.1	ENSG00000282834.1	ENSG00000120471.15
3585	ENSG00000108465.15	ENSG00000282828.1	ENSG00000078269.15
3586	ENSG00000108468.15	ENSG00000282826.2	ENSG00000248265.2
3587	ENSG00000108469.15	ENSG00000282816.1	ENSG00000196584.3
3588	ENSG00000108474.16	ENSG00000282815.1	ENSG00000284730.1
3589	ENSG00000108479.11	ENSG00000282807.3	ENSG00000173482.16
3590	ENSG00000108506.12	ENSG00000282804.1	ENSG00000197616.12
3591	ENSG00000108509.21	ENSG00000282798.1	ENSG00000215908.10
3592	ENSG00000108510.10	ENSG00000282793.1	ENSG00000234393.1
3593	ENSG00000108511.10	ENSG00000282787.1	ENSG00000249094.2
3594	ENSG00000108515.17	ENSG00000282785.1	ENSG00000242634.1
3595	ENSG00000108518.7	ENSG00000282780.1	ENSG00000225371.1
3596	ENSG00000108523.15	ENSG00000282772.1	ENSG00000259337.4
3597	ENSG00000108528.14	ENSG00000282759.1	ENSG00000216977.1
3598	ENSG00000108551.5	ENSG00000282757.3	ENSG00000224292.1
3599	ENSG00000108556.9	ENSG00000282742.1	ENSG00000270617.1
3600	ENSG00000108557.19	ENSG00000282740.1	ENSG00000227659.1
3601	ENSG00000108559.12	ENSG00000282738.1	ENSG00000269846.1

3602	ENSG00000108561.8	ENSG00000282732.1	ENSG00000238015.2
3603	ENSG00000108576.10	ENSG00000282728.1	ENSG00000285020.1
3604	ENSG00000108578.15	ENSG00000282718.1	ENSG00000248113.2
3605	ENSG00000108582.12	ENSG00000282697.1	ENSG00000242017.1
3606	ENSG00000108587.15	ENSG00000282692.1	ENSG00000228709.1
3607	ENSG00000108588.14	ENSG00000282686.1	ENSG00000261559.1
3608	ENSG00000108590.11	ENSG00000282668.1	ENSG00000265437.1
3609	ENSG00000108591.10	ENSG00000282651.2	ENSG00000229337.1
3610	ENSG00000108592.17	ENSG00000282639.1	ENSG00000171804.10
3611	ENSG00000108599.15	ENSG00000282625.1	ENSG00000251118.1
3612	ENSG00000108602.18	ENSG00000282608.1	ENSG00000226698.1
3613	ENSG00000108604.15	ENSG00000282602.1	ENSG00000201512.1
3614	ENSG00000108622.11	ENSG00000282600.2	ENSG00000254443.1
3615	ENSG00000108639.7	ENSG00000282599.1	ENSG00000263070.1
3616	ENSG00000108641.15	ENSG00000282591.1	ENSG00000229237.2
3617	ENSG00000108651.10	ENSG00000282572.2	ENSG00000079387.14
3618	ENSG00000108654.14	ENSG00000282564.1	ENSG00000213900.2
3619	ENSG00000108666.10	ENSG00000282556.2	ENSG00000226767.1
3620	ENSG00000108669.16	ENSG00000282542.1	ENSG00000227067.3
3621	ENSG00000108671.11	ENSG00000282535.1	ENSG00000180116.15
3622	ENSG00000108679.13	ENSG00000282527.1	ENSG00000259225.6
3623	ENSG00000108688.11	ENSG00000282520.1	ENSG00000234225.2
3624	ENSG00000108691.9	ENSG00000282511.1	ENSG00000137434.11
3625	ENSG00000108700.5	ENSG00000282508.1	ENSG00000240215.4
3626	ENSG00000108702.3	ENSG00000282502.1	ENSG00000198090.3
3627	ENSG00000108733.10	ENSG00000282501.1	ENSG00000213867.4
3628	ENSG00000108759.3	ENSG00000282499.1	ENSG00000248599.1
3629	ENSG00000108771.13	ENSG00000282478.1	ENSG00000251254.1
3630	ENSG00000108773.11	ENSG00000282458.1	ENSG00000171561.4
3631	ENSG00000108774.14	ENSG00000282440.1	ENSG00000259404.5
3632	ENSG00000108784.10	ENSG00000282432.1	ENSG00000259093.1
3633	ENSG00000108785.7	ENSG00000282431.1	ENSG00000266994.1
3634	ENSG00000108786.10	ENSG00000282420.1	ENSG00000276174.1
3635	ENSG00000108788.11	ENSG00000282419.2	ENSG00000236431.1
3636	ENSG00000108797.12	ENSG00000282418.1	ENSG00000271880.1
3637	ENSG00000108798.9	ENSG00000282416.1	ENSG00000237595.4
3638	ENSG00000108799.13	ENSG00000282413.1	ENSG00000246740.2
3639	ENSG00000108813.11	ENSG00000282408.1	ENSG00000183726.11
3640	ENSG00000108819.11	ENSG00000282393.1	ENSG00000274775.2
3641	ENSG00000108821.13	ENSG00000282390.1	ENSG00000240211.1
3642	ENSG00000108823.16	ENSG00000282386.1	ENSG00000237158.1
3643	ENSG00000108825.17	ENSG00000282381.1	ENSG00000237418.1
3644	ENSG00000108826.16	ENSG00000282375.1	ENSG00000213973.9
3645	ENSG00000108828.16	ENSG00000282358.1	ENSG00000269365.2
3646	ENSG00000108829.10	ENSG00000282339.1	ENSG00000105370.7
3647	ENSG00000108830.10	ENSG00000282327.1	ENSG00000262117.5
3648	ENSG00000108839.12	ENSG00000282321.1	ENSG00000235376.5
3649	ENSG00000108840.15	ENSG00000282320.1	ENSG00000267586.6
3650	ENSG00000108846.16	ENSG00000282317.1	ENSG00000063854.13
3651	ENSG00000108848.16	ENSG00000282308.1	ENSG00000066248.15
3652	ENSG00000108849.7	ENSG00000282306.1	ENSG00000156575.2
3653	ENSG00000108852.14	ENSG00000282304.1	ENSG00000250501.2
3654	ENSG00000108854.16	ENSG00000282301.2	ENSG00000112742.10

3655	ENSG00000108861.9	ENSG00000282300.1	ENSG00000225462.1
3656	ENSG00000108878.5	ENSG00000282299.1	ENSG00000265490.1
3657	ENSG00000108883.13	ENSG00000282278.1	ENSG00000218976.2
3658	ENSG00000108924.14	ENSG00000282277.1	ENSG00000219150.2
3659	ENSG00000108932.12	ENSG00000282268.1	ENSG00000253853.1
3660	ENSG00000108946.14	ENSG00000282265.1	ENSG00000220343.5
3661	ENSG00000108947.5	ENSG00000282246.1	ENSG00000230174.1
3662	ENSG00000108950.12	ENSG00000282222.1	ENSG00000169403.12
3663	ENSG00000108953.17	ENSG00000282221.1	ENSG00000238116.2
3664	ENSG00000108958.4	ENSG00000282218.1	ENSG00000228879.1
3665	ENSG00000108960.9	ENSG00000282206.1	ENSG00000272715.1
3666	ENSG00000108961.14	ENSG00000282199.1	ENSG00000258798.1
3667	ENSG00000108963.17	ENSG00000282173.1	ENSG00000268289.1
3668	ENSG00000108984.15	ENSG00000282164.3	ENSG00000266955.1
3669	ENSG00000109016.17	ENSG00000282160.1	ENSG00000141622.14
3670	ENSG00000109046.15	ENSG00000282143.1	ENSG00000211633.3
3671	ENSG00000109047.7	ENSG00000282142.1	ENSG00000237756.1
3672	ENSG00000109061.10	ENSG00000282137.1	ENSG00000231007.5
3673	ENSG00000109062.12	ENSG00000282133.1	ENSG00000115607.9
3674	ENSG00000109063.15	ENSG00000282122.1	ENSG00000103363.14
3675	ENSG00000109065.11	ENSG00000282121.1	ENSG00000267090.1
3676	ENSG00000109066.13	ENSG00000282111.1	ENSG00000205439.10
3677	ENSG00000109072.14	ENSG00000282100.1	ENSG00000260328.1
3678	ENSG00000109079.10	ENSG00000282097.1	ENSG00000234155.1
3679	ENSG00000109083.13	ENSG00000282089.1	ENSG00000261430.1
3680	ENSG00000109084.14	ENSG00000282080.1	ENSG00000227056.2
3681	ENSG00000109089.7	ENSG00000282059.1	ENSG00000203531.3
3682	ENSG00000109099.15	ENSG00000282057.1	ENSG00000196787.3
3683	ENSG00000109101.7	ENSG00000282051.1	ENSG00000240328.1
3684	ENSG00000109103.11	ENSG00000282048.1	ENSG00000258837.1
3685	ENSG00000109107.14	ENSG00000282041.1	ENSG00000181227.3
3686	ENSG00000109111.15	ENSG00000282034.1	ENSG00000240518.2
3687	ENSG00000109113.20	ENSG00000282033.1	ENSG00000277476.1
3688	ENSG00000109118.14	ENSG00000282024.1	ENSG00000271414.1
3689	ENSG00000109132.6	ENSG00000282022.1	ENSG00000255710.2
3690	ENSG00000109133.13	ENSG00000282021.1	ENSG00000269926.1
3691	ENSG00000109158.11	ENSG00000282012.1	ENSG00000261812.6
3692	ENSG00000109163.6	ENSG00000281991.1	ENSG00000130294.16
3693	ENSG00000109171.15	ENSG00000281990.1	ENSG00000200830.1
3694	ENSG00000109180.14	ENSG00000281974.1	ENSG00000203489.3
3695	ENSG00000109181.12	ENSG00000281969.1	ENSG00000285441.1
3696	ENSG00000109182.12	ENSG00000281961.1	ENSG00000253961.1
3697	ENSG00000109184.15	ENSG00000281958.1	ENSG00000226102.3
3698	ENSG00000109189.13	ENSG00000281955.1	ENSG00000227757.3
3699	ENSG00000109193.12	ENSG00000281941.1	ENSG00000237212.1
3700	ENSG00000109205.16	ENSG00000281938.2	ENSG00000199744.1
3701	ENSG00000109208.4	ENSG00000281920.1	ENSG00000128655.18
3702	ENSG00000109220.11	ENSG00000281912.1	ENSG00000280345.1
3703	ENSG00000109255.11	ENSG00000281910.1	ENSG00000269271.1
3704	ENSG00000109265.14	ENSG00000281909.1	ENSG00000222810.1
3705	ENSG00000109270.13	ENSG00000281904.1	ENSG00000236768.1
3706	ENSG00000109272.4	ENSG00000281903.2	ENSG00000105261.7
3707	ENSG00000109320.12	ENSG00000281896.1	ENSG00000184368.16

3708	ENSG00000109321.11	ENSG00000281887.3	ENSG00000065978.19
3709	ENSG00000109323.9	ENSG00000281883.1	ENSG00000177984.7
3710	ENSG00000109332.20	ENSG00000281880.2	ENSG00000255693.2
3711	ENSG00000109339.22	ENSG00000281863.1	ENSG00000248956.1
3712	ENSG00000109381.19	ENSG00000281859.1	ENSG00000270876.1
3713	ENSG00000109390.12	ENSG00000281856.1	ENSG00000230662.1
3714	ENSG00000109424.4	ENSG00000281852.1	ENSG00000139318.8
3715	ENSG00000109436.8	ENSG00000281849.3_PAR_Y	ENSG00000285042.1
3716	ENSG00000109445.11	ENSG00000281842.2	ENSG00000231301.1
3717	ENSG00000109452.12	ENSG00000281832.1	ENSG00000237027.1
3718	ENSG00000109458.8	ENSG00000281831.1	ENSG00000239577.3
3719	ENSG00000109466.14	ENSG00000281825.1	ENSG00000267748.4
3720	ENSG00000109471.4	ENSG00000281809.1	ENSG00000213352.3
3721	ENSG00000109472.14	ENSG00000281808.1	ENSG00000270555.1
3722	ENSG00000109475.16	ENSG00000281780.1	ENSG00000228656.1
3723	ENSG00000109501.13	ENSG00000281778.1	ENSG00000205177.6
3724	ENSG00000109511.11	ENSG00000281772.1	ENSG00000226473.2
3725	ENSG00000109519.13	ENSG00000281769.1	ENSG00000172399.5
3726	ENSG00000109534.17	ENSG00000281756.1	ENSG00000279807.1
3727	ENSG00000109536.12	ENSG00000281741.2	ENSG00000252745.1
3728	ENSG00000109572.13	ENSG00000281732.1	ENSG00000089692.9
3729	ENSG00000109576.14	ENSG00000281731.2	ENSG00000113555.5
3730	ENSG00000109586.12	ENSG00000281721.1	ENSG00000125337.18
3731	ENSG00000109606.13	ENSG00000281720.1	ENSG00000261670.1
3732	ENSG00000109610.6	ENSG00000281710.1	ENSG00000196141.14
3733	ENSG00000109618.12	ENSG00000281708.1	ENSG00000260601.1
3734	ENSG00000109625.19	ENSG00000281706.2	ENSG00000283235.1
3735	ENSG00000109654.15	ENSG00000281696.1	ENSG00000258791.8
3736	ENSG00000109667.12	ENSG00000281692.1	ENSG00000286119.1
3737	ENSG00000109670.15	ENSG00000281691.1	ENSG00000283516.1
3738	ENSG00000109674.4	ENSG00000281664.1	ENSG00000259670.1
3739	ENSG00000109680.11	ENSG00000281655.1	ENSG00000254672.1
3740	ENSG00000109684.15	ENSG00000281652.1	ENSG00000285870.1
3741	ENSG00000109685.18	ENSG00000281649.1	ENSG00000238055.1
3742	ENSG00000109686.18	ENSG00000281641.2	ENSG00000129474.16
3743	ENSG00000109689.16	ENSG00000281627.1	ENSG00000213051.3
3744	ENSG00000109705.7	ENSG00000281613.2	ENSG00000186094.17
3745	ENSG00000109736.15	ENSG00000281593.1	ENSG00000224763.1
3746	ENSG00000109738.11	ENSG00000281591.1	ENSG00000102302.8
3747	ENSG00000109743.11	ENSG00000281571.2	ENSG00000100344.10
3748	ENSG00000109756.9	ENSG00000281566.3	ENSG00000237709.1
3749	ENSG00000109758.8	ENSG00000281560.1	ENSG00000176887.7
3750	ENSG00000109762.16	ENSG00000281550.1	ENSG00000197757.8
3751	ENSG00000109771.15	ENSG00000281548.1	ENSG00000235062.4
3752	ENSG00000109775.11	ENSG00000281538.1	ENSG00000283766.1
3753	ENSG00000109787.13	ENSG00000281530.1	ENSG00000091128.13
3754	ENSG00000109790.16	ENSG00000281516.1	ENSG00000268659.2
3755	ENSG00000109794.13	ENSG00000281501.1	ENSG00000277911.1
3756	ENSG00000109805.10	ENSG00000281491.1	ENSG00000208772.1
3757	ENSG00000109814.12	ENSG00000281490.1	ENSG00000286180.1
3758	ENSG00000109819.9	ENSG00000281477.1	ENSG00000197372.9
3759	ENSG00000109832.14	ENSG00000281473.1	ENSG00000243969.1
3760	ENSG00000109846.8	ENSG00000281468.1	ENSG00000236993.2

3761	ENSG00000109851.6	ENSG00000281460.1	ENSG00000232036.2
3762	ENSG00000109854.13	ENSG00000281453.1	ENSG00000279822.1
3763	ENSG00000109861.16	ENSG00000281450.1	ENSG00000224834.1
3764	ENSG00000109881.17	ENSG00000281426.1	ENSG00000226646.1
3765	ENSG00000109906.13	ENSG00000281420.1	ENSG00000188931.3
3766	ENSG00000109911.19	ENSG00000281406.2	ENSG00000237273.1
3767	ENSG00000109917.11	ENSG00000281404.1	ENSG00000186185.13
3768	ENSG00000109919.10	ENSG00000281398.4	ENSG00000226666.1
3769	ENSG00000109920.12	ENSG00000281394.1	ENSG00000217130.1
3770	ENSG00000109927.10	ENSG00000281392.1	ENSG00000100101.15
3771	ENSG00000109929.10	ENSG00000281386.1	ENSG00000273818.1
3772	ENSG00000109943.9	ENSG00000281383.1	ENSG00000144567.11
3773	ENSG00000109944.10	ENSG00000281379.2	ENSG00000282911.1
3774	ENSG00000109956.13	ENSG00000281376.1	ENSG00000259686.1
3775	ENSG00000109971.14	ENSG00000281371.1	ENSG00000278537.1
3776	ENSG00000109991.8	ENSG00000281369.1	ENSG00000187653.11
3777	ENSG00000110002.15	ENSG00000281365.1	ENSG00000258035.1
3778	ENSG00000110011.13	ENSG00000281358.1	ENSG00000170743.17
3779	ENSG00000110013.12	ENSG00000281357.2	ENSG00000108599.15
3780	ENSG00000110025.13	ENSG00000281348.1	ENSG00000198597.9
3781	ENSG00000110031.12	ENSG00000281347.2	ENSG00000205846.3
3782	ENSG00000110042.8	ENSG00000281333.1	ENSG00000227080.2
3783	ENSG00000110046.13	ENSG00000281332.1	ENSG00000283347.1
3784	ENSG00000110047.18	ENSG00000281327.1	ENSG00000228089.1
3785	ENSG00000110048.12	ENSG00000281311.1	ENSG00000204960.6
3786	ENSG00000110057.8	ENSG00000281295.1	ENSG00000241246.3
3787	ENSG00000110060.9	ENSG00000281269.1	ENSG00000226421.1
3788	ENSG00000110063.10	ENSG00000281264.1	ENSG00000228928.1
3789	ENSG00000110066.15	ENSG00000281248.1	ENSG00000147676.14
3790	ENSG00000110074.10	ENSG00000281228.1	ENSG00000198842.9
3791	ENSG00000110075.14	ENSG00000281219.2	ENSG00000228834.1
3792	ENSG00000110076.18	ENSG00000281207.1	ENSG00000226144.2
3793	ENSG00000110077.14	ENSG00000281202.2	ENSG00000226430.6
3794	ENSG00000110079.18	ENSG00000281196.1	ENSG00000275155.1
3795	ENSG00000110080.18	ENSG00000281195.1	ENSG00000199785.1
3796	ENSG00000110090.13	ENSG00000281189.1	ENSG00000276771.1
3797	ENSG00000110092.3	ENSG00000281186.1	ENSG00000255492.1
3798	ENSG00000110104.13	ENSG00000281183.1	ENSG00000262020.1
3799	ENSG00000110107.9	ENSG00000281181.1	ENSG00000185112.5
3800	ENSG00000110108.10	ENSG00000281179.1	ENSG00000228171.1
3801	ENSG00000110148.10	ENSG00000281167.1	ENSG00000243635.1
3802	ENSG00000110169.11	ENSG00000281162.2	ENSG00000230387.3
3803	ENSG00000110171.20	ENSG00000281160.1	ENSG00000285871.1
3804	ENSG00000110172.12	ENSG00000281159.1	ENSG00000001167.14
3805	ENSG00000110195.13	ENSG00000281156.1	ENSG00000118990.5
3806	ENSG00000110200.8	ENSG00000281133.1	ENSG00000226636.1
3807	ENSG00000110203.9	ENSG00000281131.1	ENSG00000260482.3
3808	ENSG00000110218.9	ENSG00000281128.1	ENSG00000249426.1
3809	ENSG00000110237.4	ENSG00000281120.1	ENSG00000279208.1
3810	ENSG00000110243.11	ENSG00000281113.1	ENSG00000224936.1
3811	ENSG00000110244.7	ENSG00000281112.1	ENSG00000286221.1
3812	ENSG00000110245.12	ENSG00000281106.4	ENSG00000169813.16
3813	ENSG00000110274.16	ENSG00000281100.1	ENSG00000229690.1

3814	ENSG00000110315.7	ENSG00000281097.1	ENSG00000285278.1
3815	ENSG00000110318.13	ENSG00000281091.2	ENSG00000242757.1
3816	ENSG00000110321.17	ENSG00000281087.1	ENSG00000251292.1
3817	ENSG00000110324.10	ENSG00000281072.1	ENSG00000269765.2
3818	ENSG00000110328.6	ENSG00000281058.1	ENSG00000171889.3
3819	ENSG00000110330.8	ENSG00000281039.1	ENSG00000232848.1
3820	ENSG00000110344.9	ENSG00000281028.1	ENSG00000223548.1
3821	ENSG00000110367.13	ENSG00000281026.1	ENSG00000261123.1
3822	ENSG00000110375.3	ENSG00000281021.1	ENSG00000159261.11
3823	ENSG00000110395.6	ENSG00000281016.1	ENSG00000139572.4
3824	ENSG00000110400.11	ENSG00000281010.1	ENSG00000236875.3
3825	ENSG00000110422.12	ENSG00000281008.1	ENSG00000158125.10
3826	ENSG00000110427.15	ENSG00000281005.1	ENSG00000280224.1
3827	ENSG00000110429.14	ENSG00000281000.1	ENSG00000278041.1
3828	ENSG00000110435.11	ENSG00000280997.1	ENSG00000280773.2
3829	ENSG00000110436.13	ENSG00000280991.1	ENSG00000276845.1
3830	ENSG00000110442.12	ENSG00000280989.2	ENSG00000269186.2
3831	ENSG00000110446.11	ENSG00000280987.4	ENSG00000271399.1
3832	ENSG00000110448.11	ENSG00000280969.1	ENSG00000279017.1
3833	ENSG00000110455.13	ENSG00000280968.1	ENSG00000233365.2
3834	ENSG00000110484.7	ENSG00000280961.1	ENSG00000261857.7
3835	ENSG00000110492.15	ENSG00000280958.1	ENSG00000219410.6
3836	ENSG00000110497.14	ENSG00000280953.2	ENSG00000237551.1
3837	ENSG00000110514.19	ENSG00000280936.1	ENSG00000284325.1
3838	ENSG00000110536.14	ENSG00000280927.1	ENSG00000250473.1
3839	ENSG00000110583.13	ENSG00000280924.1	ENSG00000286074.1
3840	ENSG00000110619.17	ENSG00000280920.1	ENSG00000233625.1
3841	ENSG00000110628.14	ENSG00000280916.2	ENSG00000229815.1
3842	ENSG00000110651.11	ENSG00000280913.2	ENSG00000198826.11
3843	ENSG00000110660.15	ENSG00000280904.1	ENSG00000113851.14
3844	ENSG00000110665.11	ENSG00000280894.1	ENSG00000271153.1
3845	ENSG00000110675.13	ENSG00000280893.1	ENSG00000270983.1
3846	ENSG00000110680.12	ENSG00000280890.1	ENSG00000214814.7
3847	ENSG00000110693.17	ENSG00000280881.1	ENSG00000108381.11
3848	ENSG00000110696.10	ENSG00000280878.1	ENSG00000237077.1
3849	ENSG00000110697.13	ENSG00000280870.2	ENSG00000230086.1
3850	ENSG00000110700.7	ENSG00000280852.2	ENSG00000220256.4
3851	ENSG00000110711.10	ENSG00000280850.1	ENSG00000227214.2
3852	ENSG00000110713.17	ENSG00000280837.1	ENSG00000258545.5
3853	ENSG00000110717.13	ENSG00000280836.1	ENSG00000119125.16
3854	ENSG00000110719.10	ENSG00000280832.1	ENSG00000272505.1
3855	ENSG00000110721.12	ENSG00000280828.1	ENSG00000211697.4
3856	ENSG00000110723.12	ENSG00000280809.3	ENSG00000167671.12
3857	ENSG00000110756.17	ENSG00000280800.1	ENSG00000226288.2
3858	ENSG00000110768.12	ENSG00000280799.1	ENSG00000242759.6
3859	ENSG00000110777.12	ENSG00000280798.1	ENSG00000264177.1
3860	ENSG00000110786.18	ENSG00000280780.2	ENSG00000158480.11
3861	ENSG00000110799.13	ENSG00000280778.1	ENSG00000246548.3
3862	ENSG00000110801.13	ENSG00000280776.1	ENSG00000219797.2
3863	ENSG00000110811.20	ENSG00000280773.2	ENSG00000230993.1
3864	ENSG00000110841.14	ENSG00000280767.3_PAR_Y	ENSG00000257431.1
3865	ENSG00000110844.13	ENSG00000280767.3	ENSG00000236713.1
3866	ENSG00000110848.8	ENSG00000280758.1	ENSG00000200278.1

3867	ENSG00000110851.12	ENSG00000280757.1	ENSG00000228397.2
3868	ENSG00000110852.4	ENSG00000280752.1	ENSG00000124092.12
3869	ENSG00000110871.15	ENSG00000280744.1	ENSG00000229146.2
3870	ENSG00000110876.9	ENSG00000280734.2	ENSG00000276545.1
3871	ENSG00000110880.10	ENSG00000280725.2	ENSG00000220600.2
3872	ENSG00000110881.11	ENSG00000280719.1	ENSG00000279359.1
3873	ENSG00000110887.8	ENSG00000280711.1	ENSG00000253132.1
3874	ENSG00000110888.17	ENSG00000280710.3	ENSG00000235189.1
3875	ENSG00000110900.15	ENSG00000280709.2	ENSG00000282912.1
3876	ENSG00000110906.13	ENSG00000280707.2	ENSG00000240622.1
3877	ENSG00000110911.15	ENSG00000280704.1	ENSG00000134398.15
3878	ENSG00000110917.8	ENSG00000280703.1	ENSG00000227392.1
3879	ENSG00000110921.14	ENSG00000280693.2	ENSG00000005889.15
3880	ENSG00000110925.6	ENSG00000280683.1	ENSG00000172890.12
3881	ENSG00000110931.19	ENSG00000280670.3	ENSG00000226209.1
3882	ENSG00000110934.11	ENSG00000280665.1	ENSG00000254957.1
3883	ENSG00000110944.9	ENSG00000280660.1	ENSG00000258249.1
3884	ENSG00000110955.9	ENSG00000280655.1	ENSG00000170165.5
3885	ENSG00000110958.16	ENSG00000280650.1	ENSG00000231744.1
3886	ENSG00000110975.8	ENSG00000280649.2	ENSG00000254245.2
3887	ENSG00000110987.8	ENSG00000280646.2	ENSG00000165655.17
3888	ENSG00000111011.18	ENSG00000280639.1	ENSG00000129910.7
3889	ENSG00000111012.10	ENSG00000280636.1	ENSG00000121152.10
3890	ENSG00000111046.4	ENSG00000280634.1	ENSG00000277914.1
3891	ENSG00000111049.4	ENSG00000280623.1	ENSG00000259375.1
3892	ENSG00000111052.7	ENSG00000280620.1	ENSG00000100842.13
3893	ENSG00000111057.11	ENSG00000280614.1	ENSG00000279667.1
3894	ENSG00000111058.8	ENSG00000280607.1	ENSG00000275562.1
3895	ENSG00000111077.17	ENSG00000280604.1	ENSG00000236467.8
3896	ENSG00000111087.10	ENSG00000280594.1	ENSG00000263698.1
3897	ENSG00000111110.12	ENSG00000280587.1	ENSG00000197769.5
3898	ENSG00000111142.14	ENSG00000280580.1	ENSG00000280255.1
3899	ENSG00000111144.10	ENSG00000280571.2	ENSG00000268734.1
3900	ENSG00000111145.8	ENSG00000280561.1	ENSG00000225064.1
3901	ENSG00000111181.12	ENSG00000280560.1	ENSG00000235840.1
3902	ENSG00000111186.13	ENSG00000280554.1	ENSG00000112186.12
3903	ENSG00000111196.10	ENSG00000280543.1	ENSG00000265794.5
3904	ENSG00000111199.10	ENSG00000280537.2	ENSG00000257956.1
3905	ENSG00000111203.13	ENSG00000280515.1	ENSG00000222973.1
3906	ENSG00000111206.12	ENSG00000280511.1	ENSG00000248817.1
3907	ENSG00000111215.12	ENSG00000280502.1	ENSG00000189233.12
3908	ENSG00000111218.11	ENSG00000280498.1	ENSG00000204814.5
3909	ENSG00000111224.14	ENSG00000280496.1	ENSG00000206356.5
3910	ENSG00000111229.16	ENSG00000280494.2	ENSG00000263667.2
3911	ENSG00000111231.9	ENSG00000280486.1	ENSG00000212342.1
3912	ENSG00000111237.18	ENSG00000280485.1	ENSG00000236804.1
3913	ENSG00000111241.2	ENSG00000280474.1	ENSG00000259868.2
3914	ENSG00000111245.14	ENSG00000280466.1	ENSG00000168992.4
3915	ENSG00000111247.14	ENSG00000280457.1	ENSG00000215572.2
3916	ENSG00000111249.14	ENSG00000280455.1	ENSG00000284703.1
3917	ENSG00000111252.10	ENSG00000280454.1	ENSG00000259939.1
3918	ENSG00000111254.8	ENSG00000280453.1	ENSG00000242562.1
3919	ENSG00000111261.14	ENSG00000280451.1	ENSG00000272425.1

3920	ENSG00000111262.6	ENSG00000280450.1	ENSG00000250493.2
3921	ENSG00000111266.8	ENSG00000280449.1	ENSG00000185290.4
3922	ENSG00000111269.3	ENSG00000280446.1	ENSG00000273553.1
3923	ENSG00000111271.14	ENSG00000280445.1	ENSG00000179083.6
3924	ENSG00000111275.13	ENSG00000280444.1	ENSG00000267766.1
3925	ENSG00000111276.11	ENSG00000280443.1	ENSG00000227523.1
3926	ENSG00000111291.8	ENSG00000280441.3	ENSG00000125686.12
3927	ENSG00000111300.10	ENSG00000280440.1	ENSG00000250623.1
3928	ENSG00000111319.13	ENSG00000280439.1	ENSG00000225727.3
3929	ENSG00000111321.11	ENSG00000280438.1	ENSG00000285914.1
3930	ENSG00000111325.16	ENSG00000280436.1	ENSG00000268458.1
3931	ENSG00000111328.7	ENSG00000280435.1	ENSG00000188833.9
3932	ENSG00000111331.13	ENSG00000280434.1	ENSG00000253341.1
3933	ENSG00000111335.12	ENSG00000280433.1	ENSG00000254315.1
3934	ENSG00000111339.12	ENSG00000280432.1	ENSG00000269068.1
3935	ENSG00000111341.9	ENSG00000280431.1	ENSG00000253384.1
3936	ENSG00000111344.11	ENSG00000280430.1	ENSG00000234919.1
3937	ENSG00000111348.9	ENSG00000280429.1	ENSG00000257883.1
3938	ENSG00000111358.14	ENSG00000280426.1	ENSG00000162490.7
3939	ENSG00000111361.12	ENSG00000280425.2	ENSG00000277568.1
3940	ENSG00000111364.16	ENSG00000280424.1	ENSG00000280277.1
3941	ENSG00000111371.15	ENSG00000280423.1	ENSG00000187848.13
3942	ENSG00000111404.6	ENSG00000280422.1	ENSG00000023330.15
3943	ENSG00000111405.9	ENSG00000280420.1	ENSG00000242019.1
3944	ENSG00000111412.6	ENSG00000280419.1	ENSG00000215893.3
3945	ENSG00000111424.10	ENSG00000280418.1	ENSG00000226766.1
3946	ENSG00000111432.4	ENSG00000280416.1	ENSG00000187546.14
3947	ENSG00000111445.14	ENSG00000280415.1	ENSG00000214881.4
3948	ENSG00000111450.14	ENSG00000280414.1	ENSG00000127980.16
3949	ENSG00000111452.13	ENSG00000280412.1	ENSG00000261173.1
3950	ENSG00000111481.10	ENSG00000280411.1	ENSG00000152193.8
3951	ENSG00000111490.13	ENSG00000280409.1	ENSG00000123240.17
3952	ENSG00000111530.13	ENSG00000280408.1	ENSG00000157103.12
3953	ENSG00000111536.5	ENSG00000280407.2	ENSG00000228002.2
3954	ENSG00000111537.5	ENSG00000280406.1	ENSG00000227245.1
3955	ENSG00000111540.16	ENSG00000280405.1	ENSG00000173124.14
3956	ENSG00000111554.14	ENSG00000280404.1	ENSG00000254099.1
3957	ENSG00000111581.10	ENSG00000280402.1	ENSG00000272473.1
3958	ENSG00000111596.12	ENSG00000280401.1	ENSG00000198633.10
3959	ENSG00000111602.12	ENSG00000280400.1	ENSG00000113448.19
3960	ENSG00000111605.18	ENSG00000280399.1	ENSG00000284952.1
3961	ENSG00000111615.14	ENSG00000280398.1	ENSG00000173432.12
3962	ENSG00000111639.8	ENSG00000280397.1	ENSG00000142794.18
3963	ENSG00000111640.15	ENSG00000280396.1	ENSG00000104067.16
3964	ENSG00000111641.11	ENSG00000280395.1	ENSG00000259251.2
3965	ENSG00000111642.15	ENSG00000280394.1	ENSG00000222614.1
3966	ENSG00000111644.8	ENSG00000280393.1	ENSG00000270285.1
3967	ENSG00000111647.13	ENSG00000280390.1	ENSG00000255192.5
3968	ENSG00000111652.10	ENSG00000280388.1	ENSG00000140505.7
3969	ENSG00000111653.20	ENSG00000280387.1	ENSG00000261653.1
3970	ENSG00000111664.10	ENSG00000280385.1	ENSG00000279072.1
3971	ENSG00000111665.11	ENSG00000280384.1	ENSG00000285082.1
3972	ENSG00000111666.11	ENSG00000280383.1	ENSG00000184486.9

3973	ENSG00000111667.13	ENSG00000280382.1	ENSG00000186479.4
3974	ENSG00000111669.15	ENSG00000280381.1	ENSG00000205922.4
3975	ENSG00000111670.16	ENSG00000280380.1	ENSG00000165685.8
3976	ENSG00000111671.9	ENSG00000280379.1	ENSG00000271687.1
3977	ENSG00000111674.9	ENSG00000280378.1	ENSG00000237041.1
3978	ENSG00000111676.15	ENSG00000280377.1	ENSG00000254291.1
3979	ENSG00000111678.11	ENSG00000280376.1	ENSG00000244436.2
3980	ENSG00000111679.17	ENSG00000280375.1	ENSG00000267285.1
3981	ENSG00000111684.11	ENSG00000280373.1	ENSG00000227068.1
3982	ENSG00000111696.12	ENSG00000280372.1	ENSG00000279171.1
3983	ENSG00000111700.12	ENSG00000280371.1	ENSG00000162616.9
3984	ENSG00000111701.7	ENSG00000280369.1	ENSG00000265289.1
3985	ENSG00000111704.11	ENSG00000280368.1	ENSG00000264549.1
3986	ENSG00000111707.12	ENSG00000280367.1	ENSG00000272162.1
3987	ENSG00000111711.10	ENSG00000280366.1	ENSG00000258867.5
3988	ENSG00000111713.2	ENSG00000280365.1	ENSG00000270036.1
3989	ENSG00000111716.12	ENSG00000280364.1	ENSG00000172782.11
3990	ENSG00000111725.11	ENSG00000280362.1	ENSG00000239801.1
3991	ENSG00000111726.13	ENSG00000280360.1	ENSG00000257771.5
3992	ENSG00000111727.12	ENSG00000280359.1	ENSG00000280270.1
3993	ENSG00000111728.10	ENSG00000280357.1	ENSG00000177133.10
3994	ENSG00000111729.14	ENSG00000280356.1	ENSG00000124159.15
3995	ENSG00000111731.12	ENSG00000280355.1	ENSG00000258245.1
3996	ENSG00000111732.11	ENSG00000280354.1	ENSG00000285498.1
3997	ENSG00000111737.12	ENSG00000280353.1	ENSG00000234148.2
3998	ENSG00000111752.11	ENSG00000280352.1	ENSG00000227200.1
3999	ENSG00000111775.3	ENSG00000280351.2	ENSG00000227758.1
4000	ENSG00000111780.8	ENSG00000280350.1	ENSG00000116039.13
4001	ENSG00000111783.12	ENSG00000280349.1	ENSG00000205693.3
4002	ENSG00000111785.20	ENSG00000280348.1	ENSG00000183313.4
4003	ENSG00000111786.9	ENSG00000280347.1	ENSG00000257813.2
4004	ENSG00000111788.10	ENSG00000280346.1	ENSG00000285755.1
4005	ENSG00000111790.14	ENSG00000280345.1	ENSG00000226309.1
4006	ENSG00000111796.3	ENSG00000280344.1	ENSG00000196301.3
4007	ENSG00000111799.21	ENSG00000280341.1	ENSG00000224019.1
4008	ENSG00000111801.16	ENSG00000280340.1	ENSG00000049449.9
4009	ENSG00000111802.14	ENSG00000280339.1	ENSG00000224272.2
4010	ENSG00000111816.8	ENSG00000280338.1	ENSG00000284600.1
4011	ENSG00000111817.17	ENSG00000280337.1	ENSG00000239466.3
4012	ENSG00000111832.13	ENSG00000280336.1	ENSG00000181938.13
4013	ENSG00000111834.12	ENSG00000280335.1	ENSG00000255277.3
4014	ENSG00000111837.11	ENSG00000280334.1	ENSG00000244234.2
4015	ENSG00000111843.14	ENSG00000280333.1	ENSG00000238622.1
4016	ENSG00000111845.5	ENSG00000280332.1	ENSG00000232872.2
4017	ENSG00000111846.17	ENSG00000280331.1	ENSG00000147647.13
4018	ENSG00000111850.11	ENSG00000280330.1	ENSG00000230274.1
4019	ENSG00000111859.17	ENSG00000280327.1	ENSG00000249856.1
4020	ENSG00000111860.14	ENSG00000280326.1	ENSG00000253910.2
4021	ENSG00000111863.12	ENSG00000280325.1	ENSG00000261002.5
4022	ENSG00000111875.8	ENSG00000280323.1	ENSG00000267279.1
4023	ENSG00000111877.17	ENSG00000280322.1	ENSG00000253745.1
4024	ENSG00000111879.19	ENSG00000280321.1	ENSG00000251148.1
4025	ENSG00000111880.16	ENSG00000280320.1	ENSG00000176998.4

4026	ENSG00000111885.7	ENSG00000280319.1	ENSG00000157214.14
4027	ENSG00000111886.10	ENSG00000280318.1	ENSG00000235246.1
4028	ENSG00000111897.7	ENSG00000280317.1	ENSG00000235339.1
4029	ENSG00000111906.17	ENSG00000280314.3	ENSG00000237453.1
4030	ENSG00000111907.21	ENSG00000280312.1	ENSG00000264963.2
4031	ENSG00000111911.7	ENSG00000280311.1	ENSG00000259511.2
4032	ENSG00000111912.20	ENSG00000280310.1	ENSG00000176771.17
4033	ENSG00000111913.18	ENSG00000280309.1	ENSG00000254900.1
4034	ENSG00000111961.18	ENSG00000280307.1	ENSG00000283757.1
4035	ENSG00000111962.8	ENSG00000280306.1	ENSG00000150201.15
4036	ENSG00000111981.5	ENSG00000280305.1	ENSG00000279091.1
4037	ENSG00000112029.10	ENSG00000280304.1	ENSG00000196933.5
4038	ENSG00000112031.16	ENSG00000280303.2	ENSG00000286224.1
4039	ENSG00000112033.14	ENSG00000280302.1	ENSG00000282556.2
4040	ENSG00000112038.18	ENSG00000280300.1	ENSG00000253182.1
4041	ENSG00000112039.4	ENSG00000280299.1	ENSG00000178279.4
4042	ENSG00000112041.13	ENSG00000280296.1	ENSG00000280710.3
4043	ENSG00000112053.13	ENSG00000280295.1	ENSG00000275239.4
4044	ENSG00000112062.10	ENSG00000280294.1	ENSG00000237667.6
4045	ENSG00000112077.16	ENSG00000280291.1	ENSG00000200741.1
4046	ENSG00000112078.14	ENSG00000280288.1	ENSG00000233754.2
4047	ENSG00000112079.9	ENSG00000280287.1	ENSG00000274747.1
4048	ENSG00000112081.17	ENSG00000280286.1	ENSG00000133488.14
4049	ENSG00000112096.18	ENSG00000280285.1	ENSG00000174876.16
4050	ENSG00000112110.10	ENSG00000280284.1	ENSG00000273046.1
4051	ENSG00000112115.6	ENSG00000280283.1	ENSG00000260871.1
4052	ENSG00000112116.9	ENSG00000280282.1	ENSG00000230116.1
4053	ENSG00000112118.19	ENSG00000280281.1	ENSG00000254545.1
4054	ENSG00000112130.17	ENSG00000280280.1	ENSG00000182218.9
4055	ENSG00000112137.17	ENSG00000280279.1	ENSG00000236700.5
4056	ENSG00000112139.16	ENSG00000280278.1	ENSG00000260668.1
4057	ENSG00000112144.15	ENSG00000280277.1	ENSG00000181903.5
4058	ENSG00000112146.16	ENSG00000280276.1	ENSG00000241651.4
4059	ENSG00000112149.10	ENSG00000280275.1	ENSG00000131797.12
4060	ENSG00000112159.12	ENSG00000280274.1	ENSG00000061455.11
4061	ENSG00000112164.6	ENSG00000280273.2	ENSG00000165806.19
4062	ENSG00000112167.10	ENSG00000280272.1	ENSG00000224388.1
4063	ENSG00000112175.8	ENSG00000280270.1	ENSG00000188660.4
4064	ENSG00000112182.15	ENSG00000280269.1	ENSG00000235358.1
4065	ENSG00000112183.15	ENSG00000280268.1	ENSG00000231394.1
4066	ENSG00000112186.12	ENSG00000280267.4	ENSG00000238178.6
4067	ENSG00000112195.9	ENSG00000280266.1	ENSG00000215349.2
4068	ENSG00000112200.17	ENSG00000280265.1	ENSG00000231029.2
4069	ENSG00000112208.11	ENSG00000280263.1	ENSG00000233176.3
4070	ENSG00000112210.12	ENSG00000280262.1	ENSG00000259635.1
4071	ENSG00000112212.12	ENSG00000280261.1	ENSG00000273063.1
4072	ENSG00000112214.10	ENSG00000280259.1	ENSG00000230289.1
4073	ENSG00000112218.9	ENSG00000280257.1	ENSG00000225857.5
4074	ENSG00000112232.9	ENSG00000280255.1	ENSG00000276380.2
4075	ENSG00000112234.9	ENSG00000280254.1	ENSG00000114698.15
4076	ENSG00000112237.13	ENSG00000280251.1	ENSG00000198554.12
4077	ENSG00000112238.11	ENSG00000280250.1	ENSG00000120594.17
4078	ENSG00000112242.15	ENSG00000280249.1	ENSG00000237206.1

4079	ENSG00000112245.11	ENSG00000280248.1	ENSG00000188816.3
4080	ENSG00000112246.9	ENSG00000280247.1	ENSG00000255652.3
4081	ENSG00000112249.14	ENSG00000280245.1	ENSG00000180432.5
4082	ENSG00000112273.6	ENSG00000280244.1	ENSG00000243844.1
4083	ENSG00000112276.14	ENSG00000280243.1	ENSG00000253087.1
4084	ENSG00000112280.16	ENSG00000280242.1	ENSG00000239572.2
4085	ENSG00000112282.18	ENSG00000280241.3	ENSG00000239194.1
4086	ENSG00000112290.13	ENSG00000280239.1	ENSG00000250616.2
4087	ENSG00000112293.15	ENSG00000280238.1	ENSG00000266654.1
4088	ENSG00000112294.12	ENSG00000280237.3	ENSG00000256075.1
4089	ENSG00000112297.15	ENSG00000280236.3	ENSG00000255198.4
4090	ENSG00000112299.8	ENSG00000280234.1	ENSG00000262554.1
4091	ENSG00000112303.14	ENSG00000280233.1	ENSG00000243385.2
4092	ENSG00000112304.11	ENSG00000280232.1	ENSG00000277618.1
4093	ENSG00000112305.14	ENSG00000280231.1	ENSG00000211698.2
4094	ENSG00000112306.8	ENSG00000280229.1	ENSG00000165794.10
4095	ENSG00000112308.13	ENSG00000280228.1	ENSG00000231351.2
4096	ENSG00000112309.10	ENSG00000280227.1	ENSG00000275061.2
4097	ENSG00000112312.10	ENSG00000280225.1	ENSG00000132718.9
4098	ENSG00000112319.19	ENSG00000280224.1	ENSG00000219146.1
4099	ENSG00000112320.12	ENSG00000280223.1	ENSG00000226074.4
4100	ENSG00000112333.11	ENSG00000280222.1	ENSG00000251249.1
4101	ENSG00000112335.15	ENSG00000280220.1	ENSG00000236972.2
4102	ENSG00000112337.10	ENSG00000280219.1	ENSG00000185303.16
4103	ENSG00000112339.14	ENSG00000280217.1	ENSG00000275140.1
4104	ENSG00000112343.11	ENSG00000280216.1	ENSG00000271523.1
4105	ENSG00000112357.13	ENSG00000280215.1	ENSG00000237259.1
4106	ENSG00000112365.4	ENSG00000280214.1	ENSG00000213384.2
4107	ENSG00000112367.10	ENSG00000280213.1	ENSG00000260635.1
4108	ENSG00000112378.12	ENSG00000280212.1	ENSG00000213413.2
4109	ENSG00000112379.9	ENSG00000280211.1	ENSG00000228051.1
4110	ENSG00000112394.17	ENSG00000280208.1	ENSG00000227619.2
4111	ENSG00000112406.5	ENSG00000280207.1	ENSG00000223537.2
4112	ENSG00000112414.14	ENSG00000280206.1	ENSG00000235907.2
4113	ENSG00000112419.14	ENSG00000280205.1	ENSG00000248334.6
4114	ENSG00000112425.15	ENSG00000280204.3	ENSG00000135773.13
4115	ENSG00000112462.8	ENSG00000280202.1	ENSG00000264529.1
4116	ENSG00000112473.18	ENSG00000280201.1	ENSG00000278594.1
4117	ENSG00000112486.16	ENSG00000280200.1	ENSG00000271931.1
4118	ENSG00000112494.10	ENSG00000280199.1	ENSG00000242097.1
4119	ENSG00000112499.13	ENSG00000280198.1	ENSG00000235027.1
4120	ENSG00000112511.17	ENSG00000280197.1	ENSG00000223991.1
4121	ENSG00000112514.15	ENSG00000280196.1	ENSG00000237928.5
4122	ENSG00000112530.11	ENSG00000280195.1	ENSG00000165084.16
4123	ENSG00000112531.17	ENSG00000280194.1	ENSG00000199733.1
4124	ENSG00000112539.15	ENSG00000280193.1	ENSG00000280360.1
4125	ENSG00000112541.14	ENSG00000280191.3	ENSG00000258657.5
4126	ENSG00000112559.14	ENSG00000280190.1	ENSG00000257900.2
4127	ENSG00000112561.18	ENSG00000280189.1	ENSG00000266667.1
4128	ENSG00000112562.18	ENSG00000280187.1	ENSG00000163083.6
4129	ENSG00000112576.12	ENSG00000280186.1	ENSG00000236712.1
4130	ENSG00000112578.10	ENSG00000280185.1	ENSG00000134824.14
4131	ENSG00000112584.13	ENSG00000280184.2	ENSG00000100453.13

4132	ENSG00000112592.14	ENSG00000280183.1	ENSG00000254911.3
4133	ENSG00000112599.9	ENSG00000280182.1	ENSG00000255451.1
4134	ENSG00000112619.8	ENSG00000280181.1	ENSG00000267478.1
4135	ENSG00000112624.12	ENSG00000280180.1	ENSG00000122852.14
4136	ENSG00000112640.15	ENSG00000280179.1	ENSG00000257658.1
4137	ENSG00000112651.12	ENSG00000280177.1	ENSG00000137267.6
4138	ENSG00000112655.16	ENSG00000280176.1	ENSG00000230039.1
4139	ENSG00000112658.8	ENSG00000280173.1	ENSG00000225316.2
4140	ENSG00000112659.14	ENSG00000280172.1	ENSG00000235213.1
4141	ENSG00000112667.12	ENSG00000280171.1	ENSG00000207357.1
4142	ENSG00000112679.14	ENSG00000280169.1	ENSG00000271892.1
4143	ENSG00000112685.14	ENSG00000280168.1	ENSG00000232015.2
4144	ENSG00000112695.11	ENSG00000280167.1	ENSG00000233778.3
4145	ENSG00000112697.16	ENSG00000280166.1	ENSG00000059804.16
4146	ENSG00000112699.11	ENSG00000280165.1	ENSG00000170423.12
4147	ENSG00000112701.18	ENSG00000280164.1	ENSG00000254506.1
4148	ENSG00000112706.12	ENSG00000280163.1	ENSG00000237073.1
4149	ENSG00000112715.21	ENSG00000280162.1	ENSG00000251287.8
4150	ENSG00000112739.17	ENSG00000280159.1	ENSG00000240627.1
4151	ENSG00000112742.10	ENSG00000280157.1	ENSG00000172935.9
4152	ENSG00000112759.18	ENSG00000280155.1	ENSG00000221643.1
4153	ENSG00000112761.20	ENSG00000280154.1	ENSG00000280228.1
4154	ENSG00000112763.17	ENSG00000280153.1	ENSG00000248673.1
4155	ENSG00000112769.19	ENSG00000280152.1	ENSG00000242888.1
4156	ENSG00000112773.16	ENSG00000280150.1	ENSG00000223060.1
4157	ENSG00000112782.17	ENSG00000280149.1	ENSG00000182383.8
4158	ENSG00000112787.13	ENSG00000280148.1	ENSG00000286232.1
4159	ENSG00000112796.10	ENSG00000280147.1	ENSG00000224678.3
4160	ENSG00000112799.9	ENSG00000280145.3	ENSG00000279971.1
4161	ENSG00000112812.16	ENSG00000280144.1	ENSG00000213126.2
4162	ENSG00000112818.10	ENSG00000280143.1	ENSG00000253752.1
4163	ENSG00000112837.17	ENSG00000280142.1	ENSG00000213265.8
4164	ENSG00000112851.14	ENSG00000280140.1	ENSG00000237845.1
4165	ENSG00000112852.6	ENSG00000280139.1	ENSG00000248319.1
4166	ENSG00000112855.16	ENSG00000280138.1	ENSG00000226328.6
4167	ENSG00000112874.10	ENSG00000280137.1	ENSG00000258373.1
4168	ENSG00000112877.8	ENSG00000280136.2	ENSG00000251992.1
4169	ENSG00000112893.9	ENSG00000280135.1	ENSG00000260239.1
4170	ENSG00000112902.12	ENSG00000280134.1	ENSG00000285190.1
4171	ENSG00000112936.18	ENSG00000280132.1	ENSG00000238003.1
4172	ENSG00000112941.14	ENSG00000280131.1	ENSG00000143164.15
4173	ENSG00000112964.14	ENSG00000280129.1	ENSG00000248187.1
4174	ENSG00000112972.15	ENSG00000280128.1	ENSG00000280703.1
4175	ENSG00000112977.15	ENSG00000280126.2	ENSG00000157227.13
4176	ENSG00000112981.5	ENSG00000280125.1	ENSG00000154330.13
4177	ENSG00000112983.17	ENSG00000280124.1	ENSG00000254450.1
4178	ENSG00000112984.12	ENSG00000280122.1	ENSG00000282033.1
4179	ENSG00000112992.17	ENSG00000280121.1	ENSG00000226772.1
4180	ENSG00000112996.11	ENSG00000280120.1	ENSG00000267522.1
4181	ENSG00000113013.14	ENSG00000280119.1	ENSG00000237166.1
4182	ENSG00000113048.16	ENSG00000280118.2	ENSG00000164900.4
4183	ENSG00000113068.9	ENSG00000280117.1	ENSG00000256732.1
4184	ENSG00000113070.8	ENSG00000280116.2	ENSG00000229129.1

4185	ENSG00000113073.15	ENSG00000280115.1	ENSG00000213435.3
4186	ENSG00000113083.14	ENSG00000280114.1	ENSG00000213727.3
4187	ENSG00000113088.5	ENSG00000280113.2	ENSG00000236326.1
4188	ENSG00000113100.10	ENSG00000280110.1	ENSG00000168828.5
4189	ENSG00000113108.19	ENSG00000280109.3	ENSG00000273680.1
4190	ENSG00000113119.12	ENSG00000280108.2	ENSG00000244623.1
4191	ENSG00000113140.11	ENSG00000280107.1	ENSG00000260536.1
4192	ENSG00000113141.18	ENSG00000280106.1	ENSG00000151418.11
4193	ENSG00000113161.16	ENSG00000280105.1	ENSG00000188525.3
4194	ENSG00000113163.16	ENSG00000280104.1	ENSG00000238129.5
4195	ENSG00000113194.13	ENSG00000280103.1	ENSG00000232006.8
4196	ENSG00000113196.3	ENSG00000280099.1	ENSG00000110324.10
4197	ENSG00000113205.5	ENSG00000280098.1	ENSG00000175097.7
4198	ENSG00000113209.8	ENSG00000280097.1	ENSG00000267550.1
4199	ENSG00000113211.5	ENSG00000280096.1	ENSG00000068784.13
4200	ENSG00000113212.6	ENSG00000280095.1	ENSG00000215441.3
4201	ENSG00000113231.13	ENSG00000280094.2	ENSG00000253606.2
4202	ENSG00000113240.13	ENSG00000280093.1	ENSG00000253709.1
4203	ENSG00000113248.6	ENSG00000280092.1	ENSG00000213791.4
4204	ENSG00000113249.12	ENSG00000280091.1	ENSG00000205403.13
4205	ENSG00000113262.16	ENSG00000280090.2	ENSG00000283051.1
4206	ENSG00000113263.12	ENSG00000280089.1	ENSG00000253822.1
4207	ENSG00000113269.14	ENSG00000280088.1	ENSG00000180777.13
4208	ENSG00000113272.14	ENSG00000280087.1	ENSG00000121853.3
4209	ENSG00000113273.17	ENSG00000280086.1	ENSG00000236646.1
4210	ENSG00000113282.13	ENSG00000280085.1	ENSG00000110427.15
4211	ENSG00000113296.14	ENSG00000280083.1	ENSG00000257198.6
4212	ENSG00000113300.12	ENSG00000280082.1	ENSG00000285686.1
4213	ENSG00000113302.4	ENSG00000280081.4	ENSG00000240023.1
4214	ENSG00000113303.11	ENSG00000280080.2	ENSG00000233311.1
4215	ENSG00000113312.11	ENSG00000280079.1	ENSG00000255160.5
4216	ENSG00000113318.10	ENSG00000280078.1	ENSG00000259999.1
4217	ENSG00000113319.13	ENSG00000280077.1	ENSG00000285908.1
4218	ENSG00000113327.16	ENSG00000280076.1	ENSG00000242387.1
4219	ENSG00000113328.19	ENSG00000280073.1	ENSG00000173627.8
4220	ENSG00000113356.12	ENSG00000280071.3	ENSG00000166220.12
4221	ENSG00000113360.16	ENSG00000280070.1	ENSG00000125445.11
4222	ENSG00000113361.13	ENSG00000280069.1	ENSG00000236327.2
4223	ENSG00000113368.12	ENSG00000280068.1	ENSG00000227805.1
4224	ENSG00000113369.9	ENSG00000280067.1	ENSG00000197408.10
4225	ENSG00000113384.14	ENSG00000280064.1	ENSG00000164093.16
4226	ENSG00000113387.12	ENSG00000280063.1	ENSG00000201564.1
4227	ENSG00000113389.16	ENSG00000280062.1	ENSG00000271787.1
4228	ENSG00000113391.19	ENSG00000280061.1	ENSG00000218991.1
4229	ENSG00000113396.13	ENSG00000280060.1	ENSG00000251553.1
4230	ENSG00000113407.13	ENSG00000280059.1	ENSG00000240373.1
4231	ENSG00000113430.10	ENSG00000280058.1	ENSG00000273164.1
4232	ENSG00000113441.16	ENSG00000280057.1	ENSG00000235060.1
4233	ENSG00000113448.19	ENSG00000280056.1	ENSG00000251705.1
4234	ENSG00000113456.19	ENSG00000280055.1	ENSG00000285551.1
4235	ENSG00000113460.12	ENSG00000280054.1	ENSG00000006116.3
4236	ENSG00000113492.14	ENSG00000280053.1	ENSG00000244332.1
4237	ENSG00000113494.17	ENSG00000280051.1	ENSG00000229348.1

4238	ENSG00000113504.21	ENSG00000280049.1	ENSG00000184906.11
4239	ENSG00000113520.11	ENSG00000280048.1	ENSG00000198134.3
4240	ENSG00000113522.14	ENSG00000280047.1	ENSG00000249961.9
4241	ENSG00000113525.10	ENSG00000280046.1	ENSG00000231482.3
4242	ENSG00000113532.13	ENSG00000280044.1	ENSG00000096401.8
4243	ENSG00000113552.16	ENSG00000280043.1	ENSG00000230731.2
4244	ENSG00000113555.5	ENSG00000280042.1	ENSG00000271543.1
4245	ENSG00000113558.18	ENSG00000280040.1	ENSG00000232768.1
4246	ENSG00000113569.16	ENSG00000280039.1	ENSG00000273091.1
4247	ENSG00000113575.10	ENSG00000280038.1	ENSG00000183586.8
4248	ENSG00000113578.18	ENSG00000280037.1	ENSG00000244040.6
4249	ENSG00000113580.15	ENSG00000280036.1	ENSG00000259396.1
4250	ENSG00000113583.8	ENSG00000280035.1	ENSG00000260628.5
4251	ENSG00000113593.12	ENSG00000280033.1	ENSG00000266308.2
4252	ENSG00000113594.10	ENSG00000280032.1	ENSG00000260612.1
4253	ENSG00000113595.15	ENSG00000280029.3	ENSG00000228742.10
4254	ENSG00000113597.18	ENSG00000280028.1	ENSG00000233509.2
4255	ENSG00000113600.11	ENSG00000280027.1	ENSG00000100577.19
4256	ENSG00000113615.13	ENSG00000280026.1	ENSG00000186231.17
4257	ENSG00000113621.14	ENSG00000280025.1	ENSG00000260578.1
4258	ENSG00000113638.14	ENSG00000280024.1	ENSG00000237629.1
4259	ENSG00000113643.9	ENSG00000280023.1	ENSG00000248385.7
4260	ENSG00000113645.14	ENSG00000280022.1	ENSG00000213972.3
4261	ENSG00000113648.16	ENSG00000280021.1	ENSG00000164188.8
4262	ENSG00000113649.11	ENSG00000280020.1	ENSG00000198520.12
4263	ENSG00000113657.13	ENSG00000280019.1	ENSG00000162998.5
4264	ENSG00000113658.18	ENSG00000280018.3	ENSG00000273423.1
4265	ENSG00000113712.17	ENSG00000280017.1	ENSG00000232756.1
4266	ENSG00000113716.13	ENSG00000280016.1	ENSG00000199753.1
4267	ENSG00000113719.16	ENSG00000280015.1	ENSG00000203661.4
4268	ENSG00000113721.14	ENSG00000280014.1	ENSG00000135392.16
4269	ENSG00000113722.16	ENSG00000280013.1	ENSG00000285886.1
4270	ENSG00000113732.9	ENSG00000280012.2	ENSG00000213117.4
4271	ENSG00000113734.17	ENSG00000280011.1	ENSG00000186825.5
4272	ENSG00000113739.10	ENSG00000280010.1	ENSG00000254553.1
4273	ENSG00000113742.13	ENSG00000280009.1	ENSG00000258420.1
4274	ENSG00000113749.7	ENSG00000280007.1	ENSG00000223342.2
4275	ENSG00000113758.13	ENSG00000280005.1	ENSG00000254844.4
4276	ENSG00000113761.12	ENSG00000280004.1	ENSG00000279418.1
4277	ENSG00000113763.12	ENSG00000280003.1	ENSG00000255991.2
4278	ENSG00000113790.11	ENSG00000280002.1	ENSG00000147036.11
4279	ENSG00000113805.8	ENSG00000280000.1	ENSG00000266439.2
4280	ENSG00000113810.16	ENSG00000279998.1	ENSG00000227454.2
4281	ENSG00000113811.11	ENSG00000279997.1	ENSG00000157873.17
4282	ENSG00000113812.14	ENSG00000279996.1	ENSG00000235462.1
4283	ENSG00000113838.13	ENSG00000279995.1	ENSG00000100678.18
4284	ENSG00000113845.9	ENSG00000279994.1	ENSG00000260604.2
4285	ENSG00000113851.14	ENSG00000279993.1	ENSG00000272254.1
4286	ENSG00000113889.13	ENSG00000279991.1	ENSG00000148600.15
4287	ENSG00000113905.4	ENSG00000279990.1	ENSG00000227533.5
4288	ENSG00000113916.18	ENSG00000279989.1	ENSG00000248870.1
4289	ENSG00000113924.12	ENSG00000279987.1	ENSG00000185056.10
4290	ENSG00000113946.3	ENSG00000279985.1	ENSG00000260420.2

4291	ENSG00000113966.10	ENSG00000279983.1	ENSG00000227942.1
4292	ENSG00000113971.20	ENSG00000279982.1	ENSG00000196083.10
4293	ENSG00000114013.16	ENSG00000279981.1	ENSG00000206976.1
4294	ENSG00000114019.14	ENSG00000279980.1	ENSG00000171345.13
4295	ENSG00000114021.12	ENSG00000279979.1	ENSG00000283084.1
4296	ENSG00000114023.15	ENSG00000279977.1	ENSG00000077454.16
4297	ENSG00000114026.21	ENSG00000279976.1	ENSG00000226971.1
4298	ENSG00000114030.13	ENSG00000279973.2	ENSG00000172519.9
4299	ENSG00000114054.14	ENSG00000279972.1	ENSG00000251661.3
4300	ENSG00000114062.19	ENSG00000279971.1	ENSG00000273486.1
4301	ENSG00000114098.17	ENSG00000279970.1	ENSG00000152669.9
4302	ENSG00000114107.9	ENSG00000279968.2	ENSG00000251149.1
4303	ENSG00000114113.6	ENSG00000279967.1	ENSG00000269275.1
4304	ENSG00000114115.9	ENSG00000279966.2	ENSG00000224683.1
4305	ENSG00000114120.12	ENSG00000279965.1	ENSG00000181195.11
4306	ENSG00000114124.2	ENSG00000279964.1	ENSG00000186191.7
4307	ENSG00000114125.14	ENSG00000279962.1	ENSG00000231509.1
4308	ENSG00000114126.17	ENSG00000279961.2	ENSG00000140795.13
4309	ENSG00000114127.10	ENSG00000279960.1	ENSG00000261795.1
4310	ENSG00000114166.8	ENSG00000279959.1	ENSG00000239344.1
4311	ENSG00000114200.10	ENSG00000279958.1	ENSG00000207313.1
4312	ENSG00000114204.14	ENSG00000279957.1	ENSG00000276043.5
4313	ENSG00000114209.15	ENSG00000279956.1	ENSG00000013588.8
4314	ENSG00000114248.9	ENSG00000279955.3	ENSG00000104760.17
4315	ENSG00000114251.14	ENSG00000279954.1	ENSG00000034693.15
4316	ENSG00000114268.12	ENSG00000279953.1	ENSG00000254060.1
4317	ENSG00000114270.17	ENSG00000279952.1	ENSG00000237483.1
4318	ENSG00000114279.13	ENSG00000279951.1	ENSG00000276063.1
4319	ENSG00000114302.16	ENSG00000279949.1	ENSG00000184115.17
4320	ENSG00000114315.4	ENSG00000279948.1	ENSG00000104738.17
4321	ENSG00000114316.12	ENSG00000279946.1	ENSG00000142973.13
4322	ENSG00000114331.14	ENSG00000279945.1	ENSG00000235790.7
4323	ENSG00000114346.13	ENSG00000279943.1	ENSG00000262519.1
4324	ENSG00000114349.9	ENSG00000279942.1	ENSG00000131495.8
4325	ENSG00000114353.17	ENSG00000279940.1	ENSG00000254101.6
4326	ENSG00000114354.14	ENSG00000279939.1	ENSG00000223813.2
4327	ENSG00000114374.13	ENSG00000279936.1	ENSG00000139211.6
4328	ENSG00000114378.17	ENSG00000279935.1	ENSG00000259115.1
4329	ENSG00000114383.10	ENSG00000279933.1	ENSG00000188375.5
4330	ENSG00000114388.12	ENSG00000279932.1	ENSG00000213264.3
4331	ENSG00000114391.13	ENSG00000279931.1	ENSG00000274351.1
4332	ENSG00000114395.10	ENSG00000279930.1	ENSG00000159131.17
4333	ENSG00000114405.10	ENSG00000279929.1	ENSG00000248564.1
4334	ENSG00000114416.18	ENSG00000279928.2	ENSG00000273565.1
4335	ENSG00000114423.21	ENSG00000279927.1	ENSG00000223573.7
4336	ENSG00000114439.19	ENSG00000279926.1	ENSG00000242600.8
4337	ENSG00000114446.5	ENSG00000279925.1	ENSG00000236703.1
4338	ENSG00000114450.10	ENSG00000279924.1	ENSG00000134551.12
4339	ENSG00000114455.13	ENSG00000279923.1	ENSG00000244482.10
4340	ENSG00000114473.14	ENSG00000279920.1	ENSG00000165192.13
4341	ENSG00000114480.13	ENSG00000279919.1	ENSG00000196390.6
4342	ENSG00000114487.9	ENSG00000279918.1	ENSG00000236058.3
4343	ENSG00000114491.14	ENSG00000279917.1	ENSG00000272995.1

4344	ENSG00000114503.11	ENSG00000279914.1	ENSG00000233849.1
4345	ENSG00000114520.11	ENSG00000279913.1	ENSG00000239510.2
4346	ENSG00000114529.12	ENSG00000279912.1	ENSG00000284675.1
4347	ENSG00000114541.15	ENSG00000279910.1	ENSG00000252821.1
4348	ENSG00000114544.16	ENSG00000279908.1	ENSG00000231978.2
4349	ENSG00000114547.10	ENSG00000279907.1	ENSG00000258826.5
4350	ENSG00000114554.11	ENSG00000279905.1	ENSG00000276642.1
4351	ENSG00000114573.10	ENSG00000279904.1	ENSG00000262098.1
4352	ENSG00000114626.18	ENSG00000279903.1	ENSG00000070729.13
4353	ENSG00000114631.11	ENSG00000279901.1	ENSG00000234238.1
4354	ENSG00000114638.8	ENSG00000279900.1	ENSG00000272141.1
4355	ENSG00000114646.10	ENSG00000279897.2	ENSG00000263081.1
4356	ENSG00000114648.12	ENSG00000279895.1	ENSG00000204614.9
4357	ENSG00000114650.20	ENSG00000279894.1	ENSG00000178882.14
4358	ENSG00000114654.7	ENSG00000279891.1	ENSG00000284721.1
4359	ENSG00000114656.11	ENSG00000279889.1	ENSG00000186335.9
4360	ENSG00000114670.14	ENSG00000279887.1	ENSG00000276107.1
4361	ENSG00000114686.9	ENSG00000279886.1	ENSG00000254432.1
4362	ENSG00000114698.15	ENSG00000279885.1	ENSG00000269758.1
4363	ENSG00000114735.9	ENSG00000279884.1	ENSG00000144645.13
4364	ENSG00000114737.15	ENSG00000279883.1	ENSG00000225471.5
4365	ENSG00000114738.11	ENSG00000279882.1	ENSG00000215168.2
4366	ENSG00000114739.14	ENSG00000279881.1	ENSG00000268985.1
4367	ENSG00000114742.14	ENSG00000279879.1	ENSG00000225000.1
4368	ENSG00000114744.9	ENSG00000279878.1	ENSG00000272660.1
4369	ENSG00000114745.13	ENSG00000279876.1	ENSG00000269902.1
4370	ENSG00000114757.19	ENSG00000279875.1	ENSG00000141446.11
4371	ENSG00000114767.7	ENSG00000279874.1	ENSG00000266743.1
4372	ENSG00000114770.17	ENSG00000279873.2	ENSG00000253325.1
4373	ENSG00000114771.14	ENSG00000279872.1	ENSG00000175745.13
4374	ENSG00000114779.19	ENSG00000279869.1	ENSG00000229473.2
4375	ENSG00000114784.4	ENSG00000279865.1	ENSG00000224346.1
4376	ENSG00000114786.16	ENSG00000279864.2	ENSG00000164691.17
4377	ENSG00000114790.13	ENSG00000279862.1	ENSG00000274885.1
4378	ENSG00000114796.16	ENSG00000279861.1	ENSG00000277684.1
4379	ENSG00000114805.17	ENSG00000279860.1	ENSG00000179141.9
4380	ENSG00000114812.13	ENSG00000279859.2	ENSG00000229092.2
4381	ENSG00000114841.17	ENSG00000279858.1	ENSG00000218819.4
4382	ENSG00000114850.6	ENSG00000279856.1	ENSG00000188558.6
4383	ENSG00000114853.14	ENSG00000279855.1	ENSG00000241319.1
4384	ENSG00000114854.7	ENSG00000279853.1	ENSG00000170549.4
4385	ENSG00000114857.18	ENSG00000279852.1	ENSG00000237049.1
4386	ENSG00000114859.16	ENSG00000279851.2	ENSG00000230062.5
4387	ENSG00000114861.20	ENSG00000279848.1	ENSG00000170442.11
4388	ENSG00000114867.20	ENSG00000279847.3	ENSG00000277581.1
4389	ENSG00000114902.14	ENSG00000279846.1	ENSG00000259426.5
4390	ENSG00000114904.13	ENSG00000279845.1	ENSG00000239932.3
4391	ENSG00000114923.17	ENSG00000279844.1	ENSG00000285253.1
4392	ENSG00000114933.16	ENSG00000279842.1	ENSG00000156453.13
4393	ENSG00000114942.14	ENSG00000279841.1	ENSG00000240294.2
4394	ENSG00000114948.13	ENSG00000279840.1	ENSG00000130643.9
4395	ENSG00000114956.20	ENSG00000279839.1	ENSG00000259020.3
4396	ENSG00000114978.18	ENSG00000279837.1	ENSG00000284310.1

4397	ENSG00000114982.18	ENSG00000279836.1	ENSG00000205076.4
4398	ENSG00000114988.11	ENSG00000279835.1	ENSG00000127080.10
4399	ENSG00000114993.16	ENSG00000279834.1	ENSG00000254406.1
4400	ENSG00000114999.8	ENSG00000279833.1	ENSG00000255236.2
4401	ENSG00000115008.5	ENSG00000279829.1	ENSG00000226862.1
4402	ENSG00000115009.13	ENSG00000279827.1	ENSG00000274874.1
4403	ENSG00000115020.17	ENSG00000279825.1	ENSG00000137166.14
4404	ENSG00000115041.13	ENSG00000279822.1	ENSG00000106689.11
4405	ENSG00000115042.10	ENSG00000279821.1	ENSG00000284601.1
4406	ENSG00000115053.16	ENSG00000279819.1	ENSG00000177800.2
4407	ENSG00000115073.7	ENSG00000279818.1	ENSG00000200327.1
4408	ENSG00000115084.14	ENSG00000279817.1	ENSG00000279485.1
4409	ENSG00000115085.13	ENSG00000279815.1	ENSG00000129691.15
4410	ENSG00000115091.11	ENSG00000279814.1	ENSG00000261509.6
4411	ENSG00000115107.20	ENSG00000279813.1	ENSG00000270722.1
4412	ENSG00000115109.14	ENSG00000279811.1	ENSG00000210825.1
4413	ENSG00000115112.8	ENSG00000279810.1	ENSG00000202056.1
4414	ENSG00000115128.7	ENSG00000279809.1	ENSG00000283044.1
4415	ENSG00000115129.14	ENSG00000279807.1	ENSG00000219547.1
4416	ENSG00000115137.11	ENSG00000279806.1	ENSG00000270343.1
4417	ENSG00000115138.11	ENSG00000279804.2	ENSG00000249494.5
4418	ENSG00000115145.10	ENSG00000279803.1	ENSG00000100934.15
4419	ENSG00000115155.17	ENSG00000279801.1	ENSG00000010165.20
4420	ENSG00000115159.16	ENSG00000279800.2	ENSG00000271318.1
4421	ENSG00000115163.15	ENSG00000279799.1	ENSG00000242849.2
4422	ENSG00000115165.10	ENSG00000279798.1	ENSG00000267343.2
4423	ENSG00000115170.13	ENSG00000279797.1	ENSG00000214067.2
4424	ENSG00000115183.15	ENSG00000279796.1	ENSG00000229474.6
4425	ENSG00000115194.10	ENSG00000279795.1	ENSG00000172167.8
4426	ENSG00000115204.15	ENSG00000279794.1	ENSG00000286052.1
4427	ENSG00000115207.13	ENSG00000279793.1	ENSG00000124334.17
4428	ENSG00000115211.15	ENSG00000279792.1	ENSG00000277453.1
4429	ENSG00000115216.14	ENSG00000279791.1	ENSG00000233706.1
4430	ENSG00000115221.12	ENSG00000279790.1	ENSG00000273176.1
4431	ENSG00000115226.10	ENSG00000279789.1	ENSG00000267044.1
4432	ENSG00000115232.14	ENSG00000279788.1	ENSG00000243494.1
4433	ENSG00000115233.12	ENSG00000279786.1	ENSG00000040731.10
4434	ENSG00000115234.11	ENSG00000279785.1	ENSG00000268593.3
4435	ENSG00000115239.21	ENSG00000279784.1	ENSG00000285810.1
4436	ENSG00000115241.11	ENSG00000279783.1	ENSG00000167633.18
4437	ENSG00000115252.18	ENSG00000279782.1	ENSG00000262904.1
4438	ENSG00000115255.11	ENSG00000279781.1	ENSG00000230287.3
4439	ENSG00000115257.15	ENSG00000279780.1	ENSG00000230849.2
4440	ENSG00000115263.15	ENSG00000279779.1	ENSG00000212451.1
4441	ENSG00000115266.11	ENSG00000279778.1	ENSG00000171357.6
4442	ENSG00000115267.7	ENSG00000279775.1	ENSG00000257058.1
4443	ENSG00000115268.9	ENSG00000279774.1	ENSG00000256030.1
4444	ENSG00000115271.11	ENSG00000279773.1	ENSG00000188693.7
4445	ENSG00000115274.15	ENSG00000279772.1	ENSG00000278068.1
4446	ENSG00000115275.12	ENSG00000279771.1	ENSG00000280330.1
4447	ENSG00000115282.19	ENSG00000279770.1	ENSG00000244061.1
4448	ENSG00000115286.20	ENSG00000279769.1	ENSG00000280081.4
4449	ENSG00000115289.14	ENSG00000279765.3	ENSG00000279801.1

4450	ENSG00000115290.9	ENSG00000279764.1	ENSG00000227356.2
4451	ENSG00000115295.20	ENSG00000279762.3	ENSG00000255074.1
4452	ENSG00000115297.11	ENSG00000279761.2	ENSG00000262576.3
4453	ENSG00000115306.16	ENSG00000279759.1	ENSG00000226933.1
4454	ENSG00000115307.17	ENSG00000279758.1	ENSG00000124097.7
4455	ENSG00000115310.18	ENSG00000279757.1	ENSG00000235629.1
4456	ENSG00000115317.11	ENSG00000279756.1	ENSG00000261467.2
4457	ENSG00000115318.12	ENSG00000279755.1	ENSG00000267886.1
4458	ENSG00000115325.13	ENSG00000279754.1	ENSG00000102935.11
4459	ENSG00000115339.13	ENSG00000279752.1	ENSG00000247416.3
4460	ENSG00000115350.11	ENSG00000279751.1	ENSG00000128581.15
4461	ENSG00000115353.11	ENSG00000279750.3	ENSG00000233603.1
4462	ENSG00000115355.16	ENSG00000279749.1	ENSG00000229258.5
4463	ENSG00000115361.8	ENSG00000279748.1	ENSG00000196361.10
4464	ENSG00000115363.14	ENSG00000279747.1	ENSG00000181036.14
4465	ENSG00000115364.14	ENSG00000279746.1	ENSG00000260272.1
4466	ENSG00000115365.11	ENSG00000279744.1	ENSG00000207153.1
4467	ENSG00000115368.10	ENSG00000279743.1	ENSG00000241082.3
4468	ENSG00000115380.20	ENSG00000279742.1	ENSG00000274589.1
4469	ENSG00000115386.6	ENSG00000279741.1	ENSG00000270734.1
4470	ENSG00000115392.11	ENSG00000279739.1	ENSG00000263465.4
4471	ENSG00000115414.19	ENSG00000279738.1	ENSG00000125944.20
4472	ENSG00000115415.18	ENSG00000279737.1	ENSG00000266296.1
4473	ENSG00000115419.12	ENSG00000279735.2	ENSG00000132854.19
4474	ENSG00000115421.13	ENSG00000279734.1	ENSG00000271798.1
4475	ENSG00000115423.18	ENSG00000279733.1	ENSG00000259322.1
4476	ENSG00000115425.14	ENSG00000279732.1	ENSG00000271996.1
4477	ENSG00000115446.11	ENSG00000279730.2	ENSG00000249791.1
4478	ENSG00000115457.10	ENSG00000279729.1	ENSG00000279063.1
4479	ENSG00000115459.18	ENSG00000279728.1	ENSG00000200779.1
4480	ENSG00000115461.5	ENSG00000279727.1	ENSG00000278773.1
4481	ENSG00000115464.14	ENSG00000279726.1	ENSG00000236679.2
4482	ENSG00000115468.12	ENSG00000279725.1	ENSG00000215221.2
4483	ENSG00000115474.6	ENSG00000279724.1	ENSG00000260695.1
4484	ENSG00000115484.15	ENSG00000279722.1	ENSG00000188133.6
4485	ENSG00000115486.12	ENSG00000279721.1	ENSG00000282816.1
4486	ENSG00000115488.3	ENSG00000279720.1	ENSG00000248517.1
4487	ENSG00000115504.14	ENSG00000279719.1	ENSG00000136160.16
4488	ENSG00000115507.10	ENSG00000279718.1	ENSG00000229282.2
4489	ENSG00000115514.12	ENSG00000279717.1	ENSG00000250186.3
4490	ENSG00000115520.8	ENSG00000279716.1	ENSG00000276532.1
4491	ENSG00000115523.16	ENSG00000279715.1	ENSG00000213558.3
4492	ENSG00000115524.16	ENSG00000279714.1	ENSG00000167037.18
4493	ENSG00000115525.18	ENSG00000279713.1	ENSG00000266050.3
4494	ENSG00000115526.11	ENSG00000279712.1	ENSG00000120324.8
4495	ENSG00000115539.14	ENSG00000279711.1	ENSG00000243316.7
4496	ENSG00000115540.15	ENSG00000279709.1	ENSG00000243838.1
4497	ENSG00000115541.11	ENSG00000279708.1	ENSG00000166415.15
4498	ENSG00000115548.17	ENSG00000279707.1	ENSG00000272887.1
4499	ENSG00000115556.14	ENSG00000279706.1	ENSG00000283338.1
4500	ENSG00000115561.16	ENSG00000279705.1	ENSG00000226149.5
4501	ENSG00000115568.15	ENSG00000279704.1	ENSG00000269425.1
4502	ENSG00000115590.14	ENSG00000279703.1	ENSG00000260721.1

4503	ENSG00000115592.11	ENSG00000279702.1	ENSG00000237417.1
4504	ENSG00000115593.15	ENSG00000279701.1	ENSG00000137872.16
4505	ENSG00000115594.12	ENSG00000279700.1	ENSG00000139767.10
4506	ENSG00000115596.4	ENSG00000279699.1	ENSG00000268083.5
4507	ENSG00000115598.10	ENSG00000279698.1	ENSG00000123358.20
4508	ENSG00000115602.16	ENSG00000279697.1	ENSG00000124116.19
4509	ENSG00000115604.10	ENSG00000279696.1	ENSG00000254689.2
4510	ENSG00000115607.9	ENSG00000279695.1	ENSG00000196796.5
4511	ENSG00000115616.2	ENSG00000279694.1	ENSG00000128422.16
4512	ENSG00000115641.19	ENSG00000279693.1	ENSG00000258450.1
4513	ENSG00000115648.14	ENSG00000279692.1	ENSG00000271218.1
4514	ENSG00000115649.16	ENSG00000279691.1	ENSG00000284633.1
4515	ENSG00000115652.14	ENSG00000279690.1	ENSG00000180934.6
4516	ENSG00000115657.14	ENSG00000279689.1	ENSG00000263011.1
4517	ENSG00000115661.14	ENSG00000279688.1	ENSG00000248993.1
4518	ENSG00000115665.8	ENSG00000279687.1	ENSG00000258982.1
4519	ENSG00000115677.17	ENSG00000279686.1	ENSG00000228768.3
4520	ENSG00000115685.15	ENSG00000279685.2	ENSG00000262686.1
4521	ENSG00000115687.13	ENSG00000279684.1	ENSG00000198915.11
4522	ENSG00000115694.15	ENSG00000279683.1	ENSG00000236924.1
4523	ENSG00000115705.21	ENSG00000279682.1	ENSG00000237853.6
4524	ENSG00000115718.17	ENSG00000279681.1	ENSG00000248837.6
4525	ENSG00000115738.10	ENSG00000279679.1	ENSG00000231449.1
4526	ENSG00000115750.17	ENSG00000279678.1	ENSG00000226180.3
4527	ENSG00000115756.13	ENSG00000279677.1	ENSG00000200755.1
4528	ENSG00000115758.13	ENSG00000279676.1	ENSG00000241111.1
4529	ENSG00000115760.14	ENSG00000279675.1	ENSG00000243005.3
4530	ENSG00000115761.16	ENSG00000279674.1	ENSG00000021461.16
4531	ENSG00000115762.16	ENSG00000279673.1	ENSG00000276680.1
4532	ENSG00000115806.13	ENSG00000279672.1	ENSG00000285904.1
4533	ENSG00000115808.12	ENSG00000279671.1	ENSG00000258925.2
4534	ENSG00000115816.15	ENSG00000279670.1	ENSG00000268234.1
4535	ENSG00000115825.10	ENSG00000279669.1	ENSG00000136982.6
4536	ENSG00000115827.14	ENSG00000279668.2	ENSG00000280177.1
4537	ENSG00000115828.17	ENSG00000279666.1	ENSG00000164211.13
4538	ENSG00000115839.17	ENSG00000279665.1	ENSG00000262136.1
4539	ENSG00000115840.14	ENSG00000279663.2	ENSG00000267696.6
4540	ENSG00000115841.20	ENSG00000279662.1	ENSG00000248866.1
4541	ENSG00000115844.11	ENSG00000279660.1	ENSG00000235903.8
4542	ENSG00000115850.10	ENSG00000279659.1	ENSG00000234537.1
4543	ENSG00000115866.11	ENSG00000279658.1	ENSG00000272982.1
4544	ENSG00000115875.19	ENSG00000279657.1	ENSG00000160446.19
4545	ENSG00000115884.11	ENSG00000279656.1	ENSG00000133958.13
4546	ENSG00000115896.16	ENSG00000279655.1	ENSG00000254621.1
4547	ENSG00000115902.11	ENSG00000279653.1	ENSG00000224706.1
4548	ENSG00000115904.12	ENSG00000279652.1	ENSG00000266538.1
4549	ENSG00000115919.15	ENSG00000279650.1	ENSG00000277304.1
4550	ENSG00000115934.11	ENSG00000279649.1	ENSG00000225889.7
4551	ENSG00000115935.17	ENSG00000279648.1	ENSG00000074054.18
4552	ENSG00000115942.9	ENSG00000279647.1	ENSG00000104435.13
4553	ENSG00000115944.15	ENSG00000279645.1	ENSG00000254460.1
4554	ENSG00000115946.8	ENSG00000279642.1	ENSG00000199480.1
4555	ENSG00000115947.13	ENSG00000279641.1	ENSG00000196139.14

4556	ENSG00000115956.10	ENSG00000279640.1	ENSG00000119139.19
4557	ENSG00000115963.13	ENSG00000279639.2	ENSG00000240758.2
4558	ENSG00000115966.17	ENSG00000279638.1	ENSG00000255072.1
4559	ENSG00000115970.18	ENSG00000279637.1	ENSG00000224100.1
4560	ENSG00000115977.19	ENSG00000279636.2	ENSG00000199420.1
4561	ENSG00000115993.13	ENSG00000279635.1	ENSG00000285836.1
4562	ENSG00000115998.7	ENSG00000279633.1	ENSG00000198146.4
4563	ENSG00000116001.16	ENSG00000279632.1	ENSG00000239602.1
4564	ENSG00000116005.12	ENSG00000279631.1	ENSG00000128951.14
4565	ENSG00000116014.10	ENSG00000279630.1	ENSG00000174792.10
4566	ENSG00000116016.14	ENSG00000279629.1	ENSG00000259003.1
4567	ENSG00000116017.11	ENSG00000279628.1	ENSG00000267667.1
4568	ENSG00000116030.16	ENSG00000279626.1	ENSG00000228262.9
4569	ENSG00000116031.9	ENSG00000279625.1	ENSG00000257864.1
4570	ENSG00000116032.5	ENSG00000279623.1	ENSG00000026297.15
4571	ENSG00000116035.4	ENSG00000279622.2	ENSG00000200090.1
4572	ENSG00000116039.13	ENSG00000279621.1	ENSG00000254582.1
4573	ENSG00000116044.16	ENSG00000279620.1	ENSG00000259376.1
4574	ENSG00000116062.15	ENSG00000279619.1	ENSG00000117281.15
4575	ENSG00000116095.11	ENSG00000279617.1	ENSG00000285602.1
4576	ENSG00000116096.6	ENSG00000279616.1	ENSG00000214776.12
4577	ENSG00000116106.12	ENSG00000279615.1	ENSG00000234263.1
4578	ENSG00000116117.18	ENSG00000279613.1	ENSG00000224799.1
4579	ENSG00000116120.10	ENSG00000279611.1	ENSG00000275191.1
4580	ENSG00000116127.18	ENSG00000279610.1	ENSG00000267551.3
4581	ENSG00000116128.11	ENSG00000279609.1	ENSG00000100626.17
4582	ENSG00000116132.12	ENSG00000279608.1	ENSG00000253706.5
4583	ENSG00000116133.13	ENSG00000279607.1	ENSG00000278998.1
4584	ENSG00000116138.13	ENSG00000279606.1	ENSG00000228144.2
4585	ENSG00000116141.15	ENSG00000279605.1	ENSG00000284049.1
4586	ENSG00000116147.17	ENSG00000279604.1	ENSG00000271178.1
4587	ENSG00000116151.14	ENSG00000279603.1	ENSG00000268729.1
4588	ENSG00000116157.6	ENSG00000279602.1	ENSG00000231704.5
4589	ENSG00000116161.17	ENSG00000279601.1	ENSG00000218793.1
4590	ENSG00000116171.18	ENSG00000279600.1	ENSG00000274135.1
4591	ENSG00000116176.6	ENSG00000279599.1	ENSG00000228723.6
4592	ENSG00000116183.11	ENSG00000279598.1	ENSG00000243099.1
4593	ENSG00000116191.17	ENSG00000279597.1	ENSG00000259425.5
4594	ENSG00000116194.13	ENSG00000279595.1	ENSG00000267275.1
4595	ENSG00000116198.13	ENSG00000279594.1	ENSG00000233163.1
4596	ENSG00000116199.12	ENSG00000279593.1	ENSG00000239696.3
4597	ENSG00000116205.14	ENSG00000279592.1	ENSG00000269460.1
4598	ENSG00000116209.11	ENSG00000279591.1	ENSG00000220494.4
4599	ENSG00000116212.15	ENSG00000279590.1	ENSG00000169763.14
4600	ENSG00000116213.16	ENSG00000279589.1	ENSG00000181733.3
4601	ENSG00000116218.12	ENSG00000279588.2	ENSG00000240808.1
4602	ENSG00000116221.15	ENSG00000279587.1	ENSG00000250749.1
4603	ENSG00000116237.16	ENSG00000279586.1	ENSG00000175426.11
4604	ENSG00000116251.11	ENSG00000279585.1	ENSG00000249816.6
4605	ENSG00000116254.18	ENSG00000279584.1	ENSG00000232716.2
4606	ENSG00000116260.17	ENSG00000279583.1	ENSG00000266901.1
4607	ENSG00000116266.11	ENSG00000279581.1	ENSG00000257239.1
4608	ENSG00000116273.6	ENSG00000279579.3	ENSG00000224079.1

4609	ENSG00000116285.13	ENSG00000279578.1	ENSG00000219487.2
4610	ENSG00000116288.13	ENSG00000279575.1	ENSG00000225928.2
4611	ENSG00000116299.17	ENSG00000279573.1	ENSG00000258835.1
4612	ENSG00000116329.11	ENSG00000279572.1	ENSG00000271164.1
4613	ENSG00000116337.16	ENSG00000279571.3	ENSG00000267857.2
4614	ENSG00000116350.17	ENSG00000279570.1	ENSG00000180016.2
4615	ENSG00000116353.15	ENSG00000279569.1	ENSG00000258524.1
4616	ENSG00000116396.14	ENSG00000279568.1	ENSG00000232430.1
4617	ENSG00000116406.19	ENSG00000279567.1	ENSG00000242510.1
4618	ENSG00000116455.14	ENSG00000279565.1	ENSG00000263841.2
4619	ENSG00000116459.11	ENSG00000279562.1	ENSG00000120539.14
4620	ENSG00000116473.14	ENSG00000279561.2	ENSG00000232963.1
4621	ENSG00000116478.12	ENSG00000279559.1	ENSG00000153246.13
4622	ENSG00000116489.13	ENSG00000279558.1	ENSG00000166073.10
4623	ENSG00000116497.18	ENSG00000279555.1	ENSG00000225655.5
4624	ENSG00000116514.16	ENSG00000279554.1	ENSG00000164924.18
4625	ENSG00000116521.11	ENSG00000279551.1	ENSG00000226289.1
4626	ENSG00000116525.14	ENSG00000279550.1	ENSG00000279859.2
4627	ENSG00000116539.13	ENSG00000279549.1	ENSG00000243854.3
4628	ENSG00000116544.12	ENSG00000279548.1	ENSG00000245688.1
4629	ENSG00000116560.11	ENSG00000279544.1	ENSG00000281039.1
4630	ENSG00000116574.6	ENSG00000279543.1	ENSG00000235429.1
4631	ENSG00000116580.18	ENSG00000279542.1	ENSG00000270268.1
4632	ENSG00000116584.18	ENSG00000279541.1	ENSG00000278385.1
4633	ENSG00000116586.11	ENSG00000279539.1	ENSG00000174231.17
4634	ENSG00000116604.18	ENSG00000279537.1	ENSG00000199405.1
4635	ENSG00000116641.17	ENSG00000279536.1	ENSG00000283538.2
4636	ENSG00000116649.10	ENSG00000279534.1	ENSG00000261465.6
4637	ENSG00000116652.6	ENSG00000279532.1	ENSG00000200656.1
4638	ENSG00000116661.11	ENSG00000279531.1	ENSG00000255094.1
4639	ENSG00000116663.11	ENSG00000279530.2	ENSG00000231357.1
4640	ENSG00000116667.14	ENSG00000279529.1	ENSG00000001036.13
4641	ENSG00000116668.13	ENSG00000279528.1	ENSG00000215841.3
4642	ENSG00000116670.15	ENSG00000279527.1	ENSG00000163660.11
4643	ENSG00000116675.16	ENSG00000279526.1	ENSG00000230495.1
4644	ENSG00000116678.19	ENSG00000279525.1	ENSG00000225383.7
4645	ENSG00000116679.16	ENSG00000279523.1	ENSG00000241950.1
4646	ENSG00000116685.16	ENSG00000279521.1	ENSG00000271034.1
4647	ENSG00000116688.16	ENSG00000279520.1	ENSG00000210100.1
4648	ENSG00000116690.12	ENSG00000279519.1	ENSG00000121578.13
4649	ENSG00000116691.11	ENSG00000279518.1	ENSG00000237963.1
4650	ENSG00000116698.21	ENSG00000279516.2	ENSG00000176753.5
4651	ENSG00000116701.14	ENSG00000279514.2	ENSG00000183571.11
4652	ENSG00000116703.13	ENSG00000279513.1	ENSG00000182585.9
4653	ENSG00000116704.8	ENSG00000279512.1	ENSG00000204138.13
4654	ENSG00000116711.10	ENSG00000279511.1	ENSG00000059915.17
4655	ENSG00000116717.13	ENSG00000279509.1	ENSG00000271763.1
4656	ENSG00000116721.9	ENSG00000279507.1	ENSG00000236124.1
4657	ENSG00000116726.4	ENSG00000279505.1	ENSG00000243398.3
4658	ENSG00000116729.14	ENSG00000279504.1	ENSG00000273542.1
4659	ENSG00000116731.22	ENSG00000279503.1	ENSG00000143321.19
4660	ENSG00000116741.8	ENSG00000279502.1	ENSG00000145692.15
4661	ENSG00000116745.7	ENSG00000279501.1	ENSG00000232905.2

4662	ENSG00000116747.12	ENSG00000279500.1	ENSG00000176635.17
4663	ENSG00000116748.21	ENSG00000279499.1	ENSG00000180178.11
4664	ENSG00000116750.13	ENSG00000279498.1	ENSG00000235415.1
4665	ENSG00000116752.6	ENSG00000279497.1	ENSG00000255421.1
4666	ENSG00000116754.13	ENSG00000279495.1	ENSG00000238113.6
4667	ENSG00000116761.11	ENSG00000279494.1	ENSG00000230913.1
4668	ENSG00000116771.6	ENSG00000279493.1	ENSG00000256902.1
4669	ENSG00000116774.12	ENSG00000279491.1	ENSG00000253430.1
4670	ENSG00000116783.15	ENSG00000279490.1	ENSG00000258153.1
4671	ENSG00000116785.13	ENSG00000279489.1	ENSG00000264618.2
4672	ENSG00000116786.13	ENSG00000279488.1	ENSG00000175595.14
4673	ENSG00000116791.14	ENSG00000279486.5	ENSG00000279946.1
4674	ENSG00000116793.16	ENSG00000279485.1	ENSG00000270512.1
4675	ENSG00000116809.11	ENSG00000279484.1	ENSG00000086506.3
4676	ENSG00000116815.16	ENSG00000279483.2	ENSG00000236189.2
4677	ENSG00000116819.8	ENSG00000279482.1	ENSG00000244703.3
4678	ENSG00000116824.5	ENSG00000279481.1	ENSG00000086589.12
4679	ENSG00000116830.12	ENSG00000279480.1	ENSG00000158488.16
4680	ENSG00000116833.14	ENSG00000279479.1	ENSG00000272784.1
4681	ENSG00000116852.14	ENSG00000279478.1	ENSG00000256195.2
4682	ENSG00000116857.17	ENSG00000279477.1	ENSG00000211674.2
4683	ENSG00000116863.11	ENSG00000279476.1	ENSG00000273580.1
4684	ENSG00000116871.15	ENSG00000279475.1	ENSG00000281974.1
4685	ENSG00000116874.11	ENSG00000279474.1	ENSG00000168246.6
4686	ENSG00000116882.14	ENSG00000279472.1	ENSG00000228037.1
4687	ENSG00000116883.8	ENSG00000279470.1	ENSG00000204710.2
4688	ENSG00000116885.18	ENSG00000279469.1	ENSG00000142609.18
4689	ENSG00000116898.12	ENSG00000279467.1	ENSG00000275901.1
4690	ENSG00000116903.7	ENSG00000279466.1	ENSG00000232070.8
4691	ENSG00000116906.13	ENSG00000279465.1	ENSG00000166527.8
4692	ENSG00000116918.14	ENSG00000279464.1	ENSG00000237001.6
4693	ENSG00000116922.14	ENSG00000279463.1	ENSG00000270604.5
4694	ENSG00000116954.8	ENSG00000279462.1	ENSG00000248492.1
4695	ENSG00000116962.15	ENSG00000279461.2	ENSG00000258435.1
4696	ENSG00000116977.18	ENSG00000279460.1	ENSG00000278324.1
4697	ENSG00000116981.3	ENSG00000279459.1	ENSG00000213480.3
4698	ENSG00000116983.13	ENSG00000279458.2	ENSG00000266504.1
4699	ENSG00000116984.13	ENSG00000279457.4	ENSG00000121361.4
4700	ENSG00000116985.12	ENSG00000279456.1	ENSG00000207016.1
4701	ENSG00000116990.11	ENSG00000279455.1	ENSG00000079112.10
4702	ENSG00000116991.10	ENSG00000279454.1	ENSG00000285692.1
4703	ENSG00000116996.9	ENSG00000279452.1	ENSG00000180284.5
4704	ENSG00000117000.9	ENSG00000279450.1	ENSG00000218187.2
4705	ENSG00000117009.12	ENSG00000279447.1	ENSG00000224887.1
4706	ENSG00000117010.17	ENSG00000279446.1	ENSG00000270077.1
4707	ENSG00000117013.16	ENSG00000279445.2	ENSG00000271816.1
4708	ENSG00000117016.10	ENSG00000279443.1	ENSG00000266893.1
4709	ENSG00000117020.17	ENSG00000279442.1	ENSG00000188517.16
4710	ENSG00000117036.12	ENSG00000279441.1	ENSG00000064201.15
4711	ENSG00000117054.13	ENSG00000279440.1	ENSG00000275853.1
4712	ENSG00000117069.15	ENSG00000279439.1	ENSG00000229278.2
4713	ENSG00000117090.15	ENSG00000279438.1	ENSG00000212330.1
4714	ENSG00000117091.10	ENSG00000279437.1	ENSG00000278475.1

4715	ENSG00000117114.19	ENSG00000279434.1	ENSG00000104267.10
4716	ENSG00000117115.13	ENSG00000279433.1	ENSG00000087074.8
4717	ENSG00000117118.9	ENSG00000279432.1	ENSG00000259336.1
4718	ENSG00000117122.13	ENSG00000279431.1	ENSG00000120436.3
4719	ENSG00000117133.11	ENSG00000279430.1	ENSG00000173039.19
4720	ENSG00000117139.17	ENSG00000279429.1	ENSG00000225082.2
4721	ENSG00000117143.13	ENSG00000279428.1	ENSG00000226237.1
4722	ENSG00000117148.8	ENSG00000279427.1	ENSG00000271532.1
4723	ENSG00000117151.13	ENSG00000279426.1	ENSG00000229794.2
4724	ENSG00000117152.13	ENSG00000279425.1	ENSG00000249302.2
4725	ENSG00000117153.16	ENSG00000279423.1	ENSG00000238090.1
4726	ENSG00000117154.12	ENSG00000279422.1	ENSG00000273274.2
4727	ENSG00000117155.16	ENSG00000279421.1	ENSG00000250403.1
4728	ENSG00000117174.11	ENSG00000279420.1	ENSG00000237456.3
4729	ENSG00000117215.14	ENSG00000279419.1	ENSG00000257307.1
4730	ENSG00000117222.14	ENSG00000279418.1	ENSG00000280115.1
4731	ENSG00000117226.12	ENSG00000279417.1	ENSG00000183032.11
4732	ENSG00000117228.10	ENSG00000279416.1	ENSG00000260165.1
4733	ENSG00000117242.7	ENSG00000279415.1	ENSG00000248893.3
4734	ENSG00000117245.12	ENSG00000279414.1	ENSG00000273544.1
4735	ENSG00000117262.19	ENSG00000279413.1	ENSG00000223779.6
4736	ENSG00000117266.15	ENSG00000279412.1	ENSG00000103196.12
4737	ENSG00000117280.13	ENSG00000279411.1	ENSG00000258588.3
4738	ENSG00000117281.15	ENSG00000279410.1	ENSG00000234795.10
4739	ENSG00000117298.16	ENSG00000279409.1	ENSG00000233828.3
4740	ENSG00000117305.15	ENSG00000279408.3	ENSG00000234964.4
4741	ENSG00000117308.15	ENSG00000279407.1	ENSG00000278848.2
4742	ENSG00000117318.9	ENSG00000279406.1	ENSG00000249617.1
4743	ENSG00000117322.17	ENSG00000279405.1	ENSG00000232882.1
4744	ENSG00000117335.19	ENSG00000279404.1	ENSG00000227924.2
4745	ENSG00000117360.13	ENSG00000279403.1	ENSG00000205457.11
4746	ENSG00000117362.13	ENSG00000279401.1	ENSG00000255663.1
4747	ENSG00000117385.15	ENSG00000279400.1	ENSG00000243918.1
4748	ENSG00000117394.21	ENSG00000279399.1	ENSG00000272829.1
4749	ENSG00000117395.12	ENSG00000279398.1	ENSG00000276092.1
4750	ENSG00000117399.14	ENSG00000279397.1	ENSG00000232519.2
4751	ENSG00000117400.17	ENSG00000279395.3	ENSG00000285374.1
4752	ENSG00000117407.17	ENSG00000279394.1	ENSG00000268324.2
4753	ENSG00000117408.11	ENSG00000279393.1	ENSG00000276832.1
4754	ENSG00000117410.14	ENSG00000279392.1	ENSG00000267467.4
4755	ENSG00000117411.16	ENSG00000279391.1	ENSG00000137558.9
4756	ENSG00000117419.15	ENSG00000279390.1	ENSG00000257384.1
4757	ENSG00000117425.14	ENSG00000279388.1	ENSG00000201699.1
4758	ENSG00000117448.13	ENSG00000279387.1	ENSG00000277985.1
4759	ENSG00000117450.14	ENSG00000279384.1	ENSG00000207041.1
4760	ENSG00000117461.15	ENSG00000279382.1	ENSG00000224312.1
4761	ENSG00000117472.10	ENSG00000279381.1	ENSG00000254341.2
4762	ENSG00000117475.14	ENSG00000279379.1	ENSG00000231586.2
4763	ENSG00000117477.12	ENSG00000279378.1	ENSG00000276292.1
4764	ENSG00000117479.14	ENSG00000279377.1	ENSG00000270421.1
4765	ENSG00000117480.16	ENSG00000279376.1	ENSG00000243902.6
4766	ENSG00000117481.10	ENSG00000279375.1	ENSG00000149488.13
4767	ENSG00000117500.13	ENSG00000279373.1	ENSG00000114115.9

4768	ENSG00000117501.14	ENSG00000279372.1	ENSG00000259388.1
4769	ENSG00000117505.13	ENSG00000279370.1	ENSG00000259820.1
4770	ENSG00000117507.6	ENSG00000279369.1	ENSG00000225612.1
4771	ENSG00000117519.16	ENSG00000279368.1	ENSG00000188124.3
4772	ENSG00000117523.16	ENSG00000279367.1	ENSG00000273331.1
4773	ENSG00000117525.14	ENSG00000279366.1	ENSG00000243915.1
4774	ENSG00000117528.13	ENSG00000279365.1	ENSG00000146828.18
4775	ENSG00000117533.15	ENSG00000279364.1	ENSG00000133055.9
4776	ENSG00000117543.21	ENSG00000279361.1	ENSG00000254190.1
4777	ENSG00000117560.8	ENSG00000279360.1	ENSG00000143367.16
4778	ENSG00000117569.18	ENSG00000279359.1	ENSG00000275720.1
4779	ENSG00000117586.11	ENSG00000279358.1	ENSG00000132164.10
4780	ENSG00000117592.9	ENSG00000279356.1	ENSG00000280247.1
4781	ENSG00000117593.11	ENSG00000279355.1	ENSG00000231890.7
4782	ENSG00000117594.10	ENSG00000279354.1	ENSG00000253978.1
4783	ENSG00000117595.12	ENSG00000279353.1	ENSG00000232342.7
4784	ENSG00000117597.18	ENSG00000279352.1	ENSG00000246863.2
4785	ENSG00000117598.12	ENSG00000279349.1	ENSG00000236608.1
4786	ENSG00000117600.12	ENSG00000279348.1	ENSG00000266976.2
4787	ENSG00000117601.13	ENSG00000279347.1	ENSG00000237938.5
4788	ENSG00000117602.12	ENSG00000279345.1	ENSG00000205879.5
4789	ENSG00000117614.10	ENSG00000279344.1	ENSG00000182264.8
4790	ENSG00000117616.18	ENSG00000279343.1	ENSG00000231244.1
4791	ENSG00000117620.15	ENSG00000279342.2	ENSG00000157150.5
4792	ENSG00000117625.13	ENSG00000279341.1	ENSG00000265519.1
4793	ENSG00000117632.23	ENSG00000279340.1	ENSG00000238390.1
4794	ENSG00000117640.18	ENSG00000279339.1	ENSG00000052850.7
4795	ENSG00000117643.14	ENSG00000279337.1	ENSG00000161940.10
4796	ENSG00000117650.13	ENSG00000279336.1	ENSG00000250853.1
4797	ENSG00000117676.14	ENSG00000279334.1	ENSG00000233460.1
4798	ENSG00000117682.17	ENSG00000279333.1	ENSG00000175354.20
4799	ENSG00000117691.10	ENSG00000279332.1	ENSG00000260552.1
4800	ENSG00000117697.15	ENSG00000279331.1	ENSG00000213574.2
4801	ENSG00000117707.15	ENSG00000279330.1	ENSG00000237262.1
4802	ENSG00000117713.20	ENSG00000279329.1	ENSG00000072778.20
4803	ENSG00000117724.13	ENSG00000279328.1	ENSG00000240385.1
4804	ENSG00000117748.10	ENSG00000279326.1	ENSG00000270702.1
4805	ENSG00000117751.18	ENSG00000279325.1	ENSG00000256081.2
4806	ENSG00000117758.14	ENSG00000279324.1	ENSG00000277531.2
4807	ENSG00000117791.16	ENSG00000279322.1	ENSG00000285336.1
4808	ENSG00000117834.12	ENSG00000279321.1	ENSG00000260830.1
4809	ENSG00000117859.19	ENSG00000279320.1	ENSG00000224815.3
4810	ENSG00000117862.12	ENSG00000279319.1	ENSG00000233401.1
4811	ENSG00000117868.16	ENSG00000279317.2	ENSG00000215866.7
4812	ENSG00000117877.10	ENSG00000279314.1	ENSG00000252274.1
4813	ENSG00000117899.11	ENSG00000279313.1	ENSG00000229766.6
4814	ENSG00000117906.14	ENSG00000279311.1	ENSG00000259288.6
4815	ENSG00000117971.12	ENSG00000279310.1	ENSG00000120696.8
4816	ENSG00000117983.17	ENSG00000279307.2	ENSG00000064205.10
4817	ENSG00000117984.14	ENSG00000279306.1	ENSG00000213026.4
4818	ENSG00000118004.17	ENSG00000279305.1	ENSG00000280206.1
4819	ENSG00000118007.13	ENSG00000279304.1	ENSG00000225711.1
4820	ENSG00000118017.3	ENSG00000279303.1	ENSG00000260092.1

4821	ENSG00000118046.15	ENSG00000279302.3	ENSG00000218198.3
4822	ENSG00000118058.21	ENSG00000279301.4	ENSG00000226428.1
4823	ENSG00000118094.11	ENSG00000279300.1	ENSG00000283123.1
4824	ENSG00000118096.7	ENSG00000279299.1	ENSG00000249971.1
4825	ENSG00000118113.12	ENSG00000279298.1	ENSG00000233547.1
4826	ENSG00000118137.9	ENSG00000279297.1	ENSG00000178363.4
4827	ENSG00000118156.12	ENSG00000279296.1	ENSG00000181577.16
4828	ENSG00000118160.14	ENSG00000279295.1	ENSG00000112297.15
4829	ENSG00000118162.14	ENSG00000279294.1	ENSG00000030304.13
4830	ENSG00000118181.11	ENSG00000279289.1	ENSG00000231095.1
4831	ENSG00000118193.12	ENSG00000279288.1	ENSG00000236078.1
4832	ENSG00000118194.19	ENSG00000279286.2	ENSG00000263280.1
4833	ENSG00000118197.14	ENSG00000279285.1	ENSG00000139921.13
4834	ENSG00000118200.14	ENSG00000279284.1	ENSG00000276758.1
4835	ENSG00000118217.6	ENSG00000279283.1	ENSG00000099985.4
4836	ENSG00000118231.5	ENSG00000279281.1	ENSG00000273584.1
4837	ENSG00000118242.16	ENSG00000279278.1	ENSG00000248167.7
4838	ENSG00000118245.2	ENSG00000279277.2	ENSG00000104369.5
4839	ENSG00000118246.14	ENSG00000279276.1	ENSG00000232978.2
4840	ENSG00000118257.16	ENSG00000279275.1	ENSG00000184795.9
4841	ENSG00000118260.15	ENSG00000279274.2	ENSG00000259891.1
4842	ENSG00000118263.15	ENSG00000279273.1	ENSG00000164483.17
4843	ENSG00000118271.10	ENSG00000279271.1	ENSG00000173401.9
4844	ENSG00000118276.11	ENSG00000279270.2	ENSG00000225976.5
4845	ENSG00000118292.9	ENSG00000279269.1	ENSG00000267589.1
4846	ENSG00000118298.12	ENSG00000279268.1	ENSG00000157211.11
4847	ENSG00000118307.19	ENSG00000279267.1	ENSG00000284748.1
4848	ENSG00000118308.15	ENSG00000279266.1	ENSG00000262514.1
4849	ENSG00000118322.14	ENSG00000279265.1	ENSG00000145888.10
4850	ENSG00000118363.12	ENSG00000279264.1	ENSG00000133884.10
4851	ENSG00000118369.13	ENSG00000279263.1	ENSG00000241163.7
4852	ENSG00000118402.6	ENSG00000279262.1	ENSG00000226009.1
4853	ENSG00000118407.15	ENSG00000279261.2	ENSG00000199370.2
4854	ENSG00000118412.12	ENSG00000279259.1	ENSG00000271913.6
4855	ENSG00000118418.14	ENSG00000279256.1	ENSG00000216475.1
4856	ENSG00000118420.17	ENSG00000279255.1	ENSG00000224725.3
4857	ENSG00000118432.12	ENSG00000279254.1	ENSG00000253545.1
4858	ENSG00000118434.9	ENSG00000279253.1	ENSG00000225349.2
4859	ENSG00000118454.13	ENSG00000279251.1	ENSG00000242602.1
4860	ENSG00000118473.22	ENSG00000279250.1	ENSG00000256843.1
4861	ENSG00000118482.11	ENSG00000279249.2	ENSG00000279022.1
4862	ENSG00000118491.9	ENSG00000279248.1	ENSG00000259476.1
4863	ENSG00000118492.17	ENSG00000279246.1	ENSG00000222036.7
4864	ENSG00000118495.19	ENSG00000279245.1	ENSG00000140534.13
4865	ENSG00000118496.5	ENSG00000279243.2	ENSG00000223622.2
4866	ENSG00000118503.15	ENSG00000279240.1	ENSG00000258789.1
4867	ENSG00000118507.17	ENSG00000279239.1	ENSG00000022567.9
4868	ENSG00000118508.5	ENSG00000279237.1	ENSG00000139209.16
4869	ENSG00000118513.19	ENSG00000279236.1	ENSG00000200953.1
4870	ENSG00000118514.14	ENSG00000279235.1	ENSG00000231404.2
4871	ENSG00000118515.11	ENSG00000279233.1	ENSG00000225982.1
4872	ENSG00000118518.15	ENSG00000279232.2	ENSG00000278338.4
4873	ENSG00000118520.14	ENSG00000279231.1	ENSG00000225398.3

4874	ENSG00000118523.6	ENSG00000279228.2	ENSG00000273957.1
4875	ENSG00000118526.6	ENSG00000279227.1	ENSG00000226556.2
4876	ENSG00000118557.15	ENSG00000279226.1	ENSG00000173826.14
4877	ENSG00000118564.14	ENSG00000279225.1	ENSG00000251691.2
4878	ENSG00000118579.13	ENSG00000279223.1	ENSG00000236453.5
4879	ENSG00000118596.12	ENSG00000279222.1	ENSG00000271425.8
4880	ENSG00000118600.11	ENSG00000279221.1	ENSG00000234324.1
4881	ENSG00000118620.13	ENSG00000279220.3	ENSG00000236039.2
4882	ENSG00000118640.11	ENSG00000279218.1	ENSG00000187763.3
4883	ENSG00000118655.6	ENSG00000279217.1	ENSG00000253645.1
4884	ENSG00000118680.13	ENSG00000279214.1	ENSG00000137941.17
4885	ENSG00000118689.15	ENSG00000279213.1	ENSG00000232752.1
4886	ENSG00000118690.13	ENSG00000279212.1	ENSG00000135953.11
4887	ENSG00000118702.9	ENSG00000279211.1	ENSG00000270894.1
4888	ENSG00000118705.17	ENSG00000279209.1	ENSG00000165059.7
4889	ENSG00000118707.10	ENSG00000279208.1	ENSG00000237260.1
4890	ENSG00000118729.11	ENSG00000279207.1	ENSG00000275449.1
4891	ENSG00000118733.16	ENSG00000279206.1	ENSG00000277112.3
4892	ENSG00000118762.8	ENSG00000279205.1	ENSG00000219133.2
4893	ENSG00000118777.12	ENSG00000279204.1	ENSG00000274281.1
4894	ENSG00000118785.14	ENSG00000279203.1	ENSG00000271329.1
4895	ENSG00000118804.8	ENSG00000279202.1	ENSG00000233977.1
4896	ENSG00000118816.10	ENSG00000279201.1	ENSG00000163754.17
4897	ENSG00000118849.10	ENSG00000279200.1	ENSG00000229046.1
4898	ENSG00000118855.19	ENSG00000279199.1	ENSG00000265386.2
4899	ENSG00000118873.16	ENSG00000279198.1	ENSG00000250349.3
4900	ENSG00000118894.14	ENSG00000279197.1	ENSG00000242439.1
4901	ENSG00000118898.16	ENSG00000279196.1	ENSG00000280924.1
4902	ENSG00000118900.14	ENSG00000279193.1	ENSG00000235990.2
4903	ENSG00000118903.6	ENSG00000279192.1	ENSG00000283458.1
4904	ENSG00000118922.17	ENSG00000279191.1	ENSG00000233786.1
4905	ENSG00000118939.17	ENSG00000279190.1	ENSG00000246777.1
4906	ENSG00000118946.12	ENSG00000279189.1	ENSG00000115380.20
4907	ENSG00000118960.13	ENSG00000279187.1	ENSG00000275674.1
4908	ENSG00000118961.15	ENSG00000279186.1	ENSG00000251451.1
4909	ENSG00000118965.14	ENSG00000279182.1	ENSG00000263718.2
4910	ENSG00000118971.8	ENSG00000279181.1	ENSG00000214045.3
4911	ENSG00000118972.2	ENSG00000279180.1	ENSG00000184344.4
4912	ENSG00000118976.5	ENSG00000279179.1	ENSG00000229567.1
4913	ENSG00000118985.16	ENSG00000279177.1	ENSG00000225744.1
4914	ENSG00000118990.5	ENSG00000279176.1	ENSG00000267780.2
4915	ENSG00000118997.14	ENSG00000279175.1	ENSG00000240041.1
4916	ENSG00000119004.15	ENSG00000279174.1	ENSG00000246331.2
4917	ENSG00000119013.9	ENSG00000279173.1	ENSG00000260212.1
4918	ENSG00000119041.11	ENSG00000279172.1	ENSG00000224730.1
4919	ENSG00000119042.17	ENSG00000279171.1	ENSG00000255530.1
4920	ENSG00000119048.7	ENSG00000279170.2	ENSG00000253366.3
4921	ENSG00000119121.22	ENSG00000279169.2	ENSG00000197565.15
4922	ENSG00000119125.16	ENSG00000279168.2	ENSG00000237353.6
4923	ENSG00000119138.4	ENSG00000279167.1	ENSG00000249493.1
4924	ENSG00000119139.19	ENSG00000279166.1	ENSG00000217060.1
4925	ENSG00000119147.10	ENSG00000279165.1	ENSG00000201876.1
4926	ENSG00000119185.12	ENSG00000279164.1	ENSG00000168209.5

4927	ENSG00000119203.14	ENSG00000279163.1	ENSG00000183911.7
4928	ENSG00000119227.8	ENSG00000279162.1	ENSG00000214424.5
4929	ENSG00000119231.11	ENSG00000279161.1	ENSG00000255008.2
4930	ENSG00000119242.8	ENSG00000279160.1	ENSG00000234147.1
4931	ENSG00000119280.16	ENSG00000279159.1	ENSG00000267480.1
4932	ENSG00000119283.15	ENSG00000279157.1	ENSG00000285840.1
4933	ENSG00000119285.11	ENSG00000279156.1	ENSG00000276410.3
4934	ENSG00000119314.15	ENSG00000279155.1	ENSG00000188505.5
4935	ENSG00000119318.13	ENSG00000279154.1	ENSG00000255520.1
4936	ENSG00000119321.9	ENSG00000279153.1	ENSG00000176973.8
4937	ENSG00000119326.15	ENSG00000279152.1	ENSG00000253848.1
4938	ENSG00000119328.12	ENSG00000279151.1	ENSG00000256797.1
4939	ENSG00000119333.11	ENSG00000279149.1	ENSG00000219902.1
4940	ENSG00000119335.16	ENSG00000279147.1	ENSG00000241478.1
4941	ENSG00000119383.19	ENSG00000279146.1	ENSG00000263756.1
4942	ENSG00000119392.14	ENSG00000279145.2	ENSG00000280913.2
4943	ENSG00000119396.11	ENSG00000279144.1	ENSG00000215151.4
4944	ENSG00000119397.16	ENSG00000279143.1	ENSG00000114346.13
4945	ENSG00000119401.10	ENSG00000279142.1	ENSG00000279493.1
4946	ENSG00000119402.17	ENSG00000279141.3	ENSG00000224473.1
4947	ENSG00000119403.15	ENSG00000279140.1	ENSG00000261407.1
4948	ENSG00000119408.16	ENSG00000279139.1	ENSG00000225230.1
4949	ENSG00000119411.11	ENSG00000279138.1	ENSG00000256810.1
4950	ENSG00000119414.11	ENSG00000279137.1	ENSG00000236956.2
4951	ENSG00000119421.7	ENSG00000279136.1	ENSG00000180264.11
4952	ENSG00000119431.9	ENSG00000279135.1	ENSG00000015475.18
4953	ENSG00000119440.8	ENSG00000279134.1	ENSG00000249346.6
4954	ENSG00000119446.14	ENSG00000279133.1	ENSG00000253618.1
4955	ENSG00000119457.8	ENSG00000279130.1	ENSG00000243546.3
4956	ENSG00000119471.15	ENSG00000279129.1	ENSG00000255539.1
4957	ENSG00000119487.17	ENSG00000279128.1	ENSG00000267734.1
4958	ENSG00000119508.18	ENSG00000279127.1	ENSG00000162641.19
4959	ENSG00000119509.13	ENSG00000279125.1	ENSG00000237592.2
4960	ENSG00000119514.7	ENSG00000279124.1	ENSG00000122986.13
4961	ENSG00000119522.16	ENSG00000279123.1	ENSG00000263669.2
4962	ENSG00000119523.10	ENSG00000279122.1	ENSG00000233799.1
4963	ENSG00000119535.17	ENSG00000279121.1	ENSG00000273433.1
4964	ENSG00000119537.17	ENSG00000279120.1	ENSG00000258847.1
4965	ENSG00000119541.10	ENSG00000279119.1	ENSG00000196240.4
4966	ENSG00000119547.6	ENSG00000279118.1	ENSG00000260351.1
4967	ENSG00000119559.16	ENSG00000279117.1	ENSG00000271682.1
4968	ENSG00000119574.13	ENSG00000279116.2	ENSG00000226605.1
4969	ENSG00000119596.18	ENSG00000279115.2	ENSG00000283342.1
4970	ENSG00000119599.16	ENSG00000279114.1	ENSG00000259986.1
4971	ENSG00000119608.12	ENSG00000279113.1	ENSG00000233588.1
4972	ENSG00000119614.3	ENSG00000279111.2	ENSG00000233771.2
4973	ENSG00000119616.11	ENSG00000279110.1	ENSG00000231755.1
4974	ENSG00000119630.14	ENSG00000279108.1	ENSG00000182685.7
4975	ENSG00000119632.4	ENSG00000279107.1	ENSG00000226340.1
4976	ENSG00000119636.16	ENSG00000279106.1	ENSG00000230449.1
4977	ENSG00000119638.13	ENSG00000279104.1	ENSG00000228481.1
4978	ENSG00000119640.9	ENSG00000279103.1	ENSG00000229455.2
4979	ENSG00000119650.12	ENSG00000279100.1	ENSG00000133392.18

4980	ENSG00000119655.10	ENSG00000279099.1	ENSG00000013297.11
4981	ENSG00000119660.4	ENSG00000279098.1	ENSG00000236307.2
4982	ENSG00000119661.15	ENSG00000279097.1	ENSG00000138592.14
4983	ENSG00000119669.5	ENSG00000279096.2	ENSG00000113649.11
4984	ENSG00000119673.14	ENSG00000279095.1	ENSG00000114107.9
4985	ENSG00000119681.12	ENSG00000279094.3	ENSG00000277948.1
4986	ENSG00000119682.17	ENSG00000279093.1	ENSG00000182952.5
4987	ENSG00000119684.15	ENSG00000279092.1	ENSG00000112137.17
4988	ENSG00000119685.20	ENSG00000279091.1	ENSG00000256351.1
4989	ENSG00000119686.10	ENSG00000279090.1	ENSG00000259921.1
4990	ENSG00000119688.21	ENSG00000279089.1	ENSG00000268993.1
4991	ENSG00000119689.15	ENSG00000279088.1	ENSG00000254295.1
4992	ENSG00000119698.12	ENSG00000279087.1	ENSG00000277741.4
4993	ENSG00000119699.7	ENSG00000279086.1	ENSG00000277651.1
4994	ENSG00000119703.14	ENSG00000279085.1	ENSG00000229742.1
4995	ENSG00000119705.9	ENSG00000279083.1	ENSG00000279128.1
4996	ENSG00000119707.14	ENSG00000279082.3	ENSG00000250604.1
4997	ENSG00000119711.12	ENSG00000279081.2	ENSG00000121318.2
4998	ENSG00000119714.11	ENSG00000279080.1	ENSG00000263717.1
4999	ENSG00000119715.15	ENSG00000279078.1	ENSG00000236268.5
5000	ENSG00000119718.11	ENSG00000279077.1	ENSG00000227232.5
5001	ENSG00000119720.18	ENSG00000279076.1	ENSG00000173320.12
5002	ENSG00000119723.16	ENSG00000279075.1	ENSG00000004059.11
5003	ENSG00000119725.18	ENSG00000279074.1	ENSG00000251066.1
5004	ENSG00000119729.12	ENSG00000279073.3	ENSG00000103540.16
5005	ENSG00000119737.5	ENSG00000279072.1	ENSG00000130822.15
5006	ENSG00000119760.16	ENSG00000279071.1	ENSG00000261390.5
5007	ENSG00000119771.15	ENSG00000279070.2	ENSG00000207051.1
5008	ENSG00000119772.16	ENSG00000279069.1	ENSG00000242537.1
5009	ENSG00000119777.20	ENSG00000279068.1	ENSG00000260369.2
5010	ENSG00000119778.15	ENSG00000279067.1	ENSG00000108651.10
5011	ENSG00000119782.14	ENSG00000279066.1	ENSG00000232663.2
5012	ENSG00000119787.14	ENSG00000279065.1	ENSG00000251576.1
5013	ENSG00000119801.13	ENSG00000279064.1	ENSG00000226241.2
5014	ENSG00000119812.19	ENSG00000279063.1	ENSG00000277721.1
5015	ENSG00000119820.11	ENSG00000279062.1	ENSG00000108342.12
5016	ENSG00000119844.15	ENSG00000279061.1	ENSG00000228348.1
5017	ENSG00000119862.13	ENSG00000279059.1	ENSG00000283392.1
5018	ENSG00000119865.8	ENSG00000279057.1	ENSG00000224282.3
5019	ENSG00000119866.21	ENSG00000279056.1	ENSG00000285736.1
5020	ENSG00000119878.5	ENSG00000279051.4	ENSG00000255107.1
5021	ENSG00000119888.10	ENSG00000279050.1	ENSG00000283791.1
5022	ENSG00000119899.13	ENSG00000279049.1	ENSG00000095713.14
5023	ENSG00000119900.9	ENSG00000279048.1	ENSG00000068745.15
5024	ENSG00000119906.13	ENSG00000279047.3	ENSG00000253957.1
5025	ENSG00000119912.16	ENSG00000279046.1	ENSG00000267388.1
5026	ENSG00000119913.5	ENSG00000279045.1	ENSG00000233608.4
5027	ENSG00000119915.5	ENSG00000279044.1	ENSG00000216306.3
5028	ENSG00000119917.14	ENSG00000279043.1	ENSG00000224739.2
5029	ENSG00000119919.11	ENSG00000279042.1	ENSG00000225358.1
5030	ENSG00000119922.10	ENSG00000279041.1	ENSG00000221102.1
5031	ENSG00000119927.14	ENSG00000279040.1	ENSG00000242299.1
5032	ENSG00000119929.13	ENSG00000279039.1	ENSG00000217447.1

5033	ENSG00000119938.9	ENSG00000279036.1	ENSG00000105443.15
5034	ENSG00000119943.13	ENSG00000279035.1	ENSG00000268677.1
5035	ENSG00000119946.11	ENSG00000279034.1	ENSG00000264423.2
5036	ENSG00000119950.21	ENSG00000279033.1	ENSG00000200785.1
5037	ENSG00000119953.12	ENSG00000279032.1	ENSG00000202406.1
5038	ENSG00000119965.13	ENSG00000279031.1	ENSG00000234060.1
5039	ENSG00000119969.15	ENSG00000279030.1	ENSG00000259097.1
5040	ENSG00000119973.6	ENSG00000279029.1	ENSG00000001561.7
5041	ENSG00000119977.21	ENSG00000279028.1	ENSG00000259119.1
5042	ENSG00000119979.18	ENSG00000279026.1	ENSG00000230157.1
5043	ENSG00000119986.7	ENSG00000279025.1	ENSG00000261602.1
5044	ENSG00000120008.16	ENSG00000279024.1	ENSG00000212440.1
5045	ENSG00000120029.13	ENSG00000279023.1	ENSG00000229611.1
5046	ENSG00000120049.19	ENSG00000279022.1	ENSG00000267504.1
5047	ENSG00000120051.15	ENSG00000279021.1	ENSG00000213820.3
5048	ENSG00000120053.12	ENSG00000279020.1	ENSG00000188467.10
5049	ENSG00000120054.11	ENSG00000279019.1	ENSG00000240305.1
5050	ENSG00000120055.7	ENSG00000279017.1	ENSG00000230707.1
5051	ENSG00000120057.5	ENSG00000279016.1	ENSG00000237586.3
5052	ENSG00000120063.10	ENSG00000279015.1	ENSG00000214124.3
5053	ENSG00000120068.6	ENSG00000279014.1	ENSG00000279206.1
5054	ENSG00000120071.14	ENSG00000279013.1	ENSG00000256393.1
5055	ENSG00000120075.5	ENSG00000279012.2	ENSG00000219384.1
5056	ENSG00000120088.14	ENSG00000279011.1	ENSG00000183696.14
5057	ENSG00000120093.11	ENSG00000279009.1	ENSG00000197608.11
5058	ENSG00000120094.8	ENSG00000279006.1	ENSG00000224551.1
5059	ENSG00000120129.6	ENSG00000279005.1	ENSG00000226578.1
5060	ENSG00000120137.7	ENSG00000279004.1	ENSG00000262560.1
5061	ENSG00000120149.9	ENSG00000279002.1	ENSG00000178750.3
5062	ENSG00000120156.21	ENSG00000279001.1	ENSG00000225945.1
5063	ENSG00000120158.12	ENSG00000279000.3	ENSG00000055813.6
5064	ENSG00000120159.13	ENSG00000278999.1	ENSG00000271742.1
5065	ENSG00000120160.11	ENSG00000278998.1	ENSG00000164744.13
5066	ENSG00000120162.10	ENSG00000278997.1	ENSG00000277690.2
5067	ENSG00000120210.8	ENSG00000278996.1	ENSG00000224827.1
5068	ENSG00000120211.4	ENSG00000278995.1	ENSG00000279980.1
5069	ENSG00000120215.9	ENSG00000278994.1	ENSG00000275520.1
5070	ENSG00000120217.14	ENSG00000278993.1	ENSG00000188818.12
5071	ENSG00000120235.4	ENSG00000278992.1	ENSG00000225639.1
5072	ENSG00000120242.3	ENSG00000278991.1	ENSG00000227791.3
5073	ENSG00000120251.20	ENSG00000278990.1	ENSG00000196620.10
5074	ENSG00000120253.14	ENSG00000278989.1	ENSG00000215326.3
5075	ENSG00000120254.15	ENSG00000278988.1	ENSG00000254780.1
5076	ENSG00000120256.10	ENSG00000278987.1	ENSG00000225907.1
5077	ENSG00000120262.10	ENSG00000278986.1	ENSG00000257870.1
5078	ENSG00000120265.17	ENSG00000278985.1	ENSG00000224972.1
5079	ENSG00000120278.16	ENSG00000278982.1	ENSG00000185182.14
5080	ENSG00000120279.6	ENSG00000278981.1	ENSG00000238171.1
5081	ENSG00000120280.5	ENSG00000278980.1	ENSG00000113946.3
5082	ENSG00000120289.11	ENSG00000278979.1	ENSG00000086548.9
5083	ENSG00000120306.11	ENSG00000278977.1	ENSG00000177910.7
5084	ENSG00000120314.18	ENSG00000278975.1	ENSG00000242559.3
5085	ENSG00000120318.16	ENSG00000278974.1	ENSG00000232662.2

5086	ENSG00000120322.3	ENSG00000278973.1	ENSG00000283906.1
5087	ENSG00000120324.8	ENSG00000278972.1	ENSG00000230046.1
5088	ENSG00000120327.6	ENSG00000278971.1	ENSG00000159214.13
5089	ENSG00000120328.6	ENSG00000278970.2	ENSG00000255251.1
5090	ENSG00000120329.6	ENSG00000278969.1	ENSG00000212163.5
5091	ENSG00000120332.15	ENSG00000278967.1	ENSG00000102290.22
5092	ENSG00000120333.4	ENSG00000278966.2	ENSG00000266477.2
5093	ENSG00000120334.15	ENSG00000278965.1	ENSG00000232077.1
5094	ENSG00000120337.8	ENSG00000278964.1	ENSG00000127366.5
5095	ENSG00000120341.18	ENSG00000278963.1	ENSG00000260671.2
5096	ENSG00000120370.12	ENSG00000278961.2	ENSG00000253900.1
5097	ENSG00000120436.3	ENSG00000278960.1	ENSG00000260410.2
5098	ENSG00000120437.8	ENSG00000278959.1	ENSG00000168703.5
5099	ENSG00000120438.12	ENSG00000278958.1	ENSG00000124508.16
5100	ENSG00000120440.15	ENSG00000278957.1	ENSG00000258016.1
5101	ENSG00000120451.10	ENSG00000278955.3	ENSG00000204532.6
5102	ENSG00000120457.12	ENSG00000278954.1	ENSG00000272866.1
5103	ENSG00000120458.11	ENSG00000278953.1	ENSG00000266074.8
5104	ENSG00000120471.15	ENSG00000278952.1	ENSG00000133059.17
5105	ENSG00000120498.13	ENSG00000278950.1	ENSG00000254029.1
5106	ENSG00000120500.17	ENSG00000278949.1	ENSG00000158458.20
5107	ENSG00000120509.10	ENSG00000278948.1	ENSG00000279050.1
5108	ENSG00000120519.14	ENSG00000278946.1	ENSG00000270269.1
5109	ENSG00000120526.11	ENSG00000278945.1	ENSG00000273720.1
5110	ENSG00000120533.13	ENSG00000278944.1	ENSG00000186526.12
5111	ENSG00000120539.14	ENSG00000278943.1	ENSG00000171848.15
5112	ENSG00000120549.18	ENSG00000278941.1	ENSG00000268660.1
5113	ENSG00000120555.13	ENSG00000278936.1	ENSG00000171552.13
5114	ENSG00000120563.9	ENSG00000278935.1	ENSG00000254486.1
5115	ENSG00000120594.17	ENSG00000278934.1	ENSG00000224221.1
5116	ENSG00000120616.15	ENSG00000278933.1	ENSG00000214815.2
5117	ENSG00000120645.11	ENSG00000278932.3	ENSG00000264982.1
5118	ENSG00000120647.10	ENSG00000278929.1	ENSG00000258920.1
5119	ENSG00000120656.11	ENSG00000278928.1	ENSG00000019582.15
5120	ENSG00000120658.13	ENSG00000278927.1	ENSG00000077080.9
5121	ENSG00000120659.15	ENSG00000278926.1	ENSG00000261692.1
5122	ENSG00000120662.16	ENSG00000278925.1	ENSG00000251014.1
5123	ENSG00000120664.10	ENSG00000278924.1	ENSG00000257738.2
5124	ENSG00000120669.16	ENSG00000278923.2	ENSG00000279537.1
5125	ENSG00000120675.6	ENSG00000278922.1	ENSG00000177418.2
5126	ENSG00000120685.20	ENSG00000278921.2	ENSG00000143627.19
5127	ENSG00000120686.12	ENSG00000278920.1	ENSG00000188712.4
5128	ENSG00000120688.9	ENSG00000278918.1	ENSG00000159164.10
5129	ENSG00000120690.16	ENSG00000278917.1	ENSG00000253653.1
5130	ENSG00000120693.13	ENSG00000278916.1	ENSG00000163689.20
5131	ENSG00000120694.19	ENSG00000278915.1	ENSG00000144908.13
5132	ENSG00000120696.8	ENSG00000278914.1	ENSG00000238188.1
5133	ENSG00000120697.9	ENSG00000278913.1	ENSG00000027869.11
5134	ENSG00000120699.13	ENSG00000278912.1	ENSG00000235828.5
5135	ENSG00000120705.13	ENSG00000278911.1	ENSG00000067064.11
5136	ENSG00000120708.17	ENSG00000278910.4	ENSG00000186472.20
5137	ENSG00000120709.11	ENSG00000278909.1	ENSG00000270773.1
5138	ENSG00000120725.13	ENSG00000278908.1	ENSG00000282879.1

5139	ENSG00000120727.13	ENSG00000278907.1	ENSG00000231419.6
5140	ENSG00000120729.9	ENSG00000278905.1	ENSG00000146416.18
5141	ENSG00000120733.14	ENSG00000278903.3	ENSG00000214369.2
5142	ENSG00000120738.8	ENSG00000278902.1	ENSG00000167323.11
5143	ENSG00000120742.11	ENSG00000278901.1	ENSG00000243437.3
5144	ENSG00000120756.13	ENSG00000278899.1	ENSG00000172322.14
5145	ENSG00000120784.16	ENSG00000278898.1	ENSG00000162496.9
5146	ENSG00000120798.17	ENSG00000278897.1	ENSG00000271697.1
5147	ENSG00000120800.5	ENSG00000278896.1	ENSG00000277049.1
5148	ENSG00000120802.13	ENSG00000278895.1	ENSG00000188869.13
5149	ENSG00000120805.14	ENSG00000278894.1	ENSG00000229914.1
5150	ENSG00000120820.12	ENSG00000278893.1	ENSG00000267765.1
5151	ENSG00000120832.10	ENSG00000278892.1	ENSG00000198452.7
5152	ENSG00000120833.14	ENSG00000278891.1	ENSG00000224513.2
5153	ENSG00000120837.8	ENSG00000278890.1	ENSG00000176198.3
5154	ENSG00000120860.10	ENSG00000278889.4	ENSG00000224826.1
5155	ENSG00000120868.13	ENSG00000278887.2	ENSG00000215464.4
5156	ENSG00000120875.9	ENSG00000278886.1	ENSG00000250678.1
5157	ENSG00000120885.21	ENSG00000278885.1	ENSG00000272567.1
5158	ENSG00000120889.13	ENSG00000278884.1	ENSG00000270457.1
5159	ENSG00000120896.13	ENSG00000278881.2	ENSG00000104299.15
5160	ENSG00000120899.18	ENSG00000278880.1	ENSG00000233363.1
5161	ENSG00000120903.13	ENSG00000278879.1	ENSG00000278655.1
5162	ENSG00000120907.17	ENSG00000278878.1	ENSG00000279334.1
5163	ENSG00000120910.14	ENSG00000278877.1	ENSG00000231313.2
5164	ENSG00000120913.23	ENSG00000278876.1	ENSG00000285803.1
5165	ENSG00000120915.13	ENSG00000278875.1	ENSG00000254298.1
5166	ENSG00000120925.16	ENSG00000278874.1	ENSG00000132639.12
5167	ENSG00000120937.9	ENSG00000278873.1	ENSG00000286262.1
5168	ENSG00000120942.13	ENSG00000278872.1	ENSG00000117899.11
5169	ENSG00000120948.17	ENSG00000278870.2	ENSG00000279527.1
5170	ENSG00000120949.15	ENSG00000278869.1	ENSG00000170633.16
5171	ENSG00000120952.4	ENSG00000278867.1	ENSG00000127377.9
5172	ENSG00000120963.12	ENSG00000278866.1	ENSG00000183647.10
5173	ENSG00000120992.18	ENSG00000278865.1	ENSG00000278083.1
5174	ENSG00000121005.9	ENSG00000278864.1	ENSG00000276667.1
5175	ENSG00000121022.14	ENSG00000278863.1	ENSG00000254818.1
5176	ENSG00000121039.10	ENSG00000278862.1	ENSG00000286107.1
5177	ENSG00000121053.6	ENSG00000278861.1	ENSG00000188937.5
5178	ENSG00000121057.13	ENSG00000278860.1	ENSG00000273403.1
5179	ENSG00000121058.5	ENSG00000278859.1	ENSG00000230317.1
5180	ENSG00000121060.18	ENSG00000278857.1	ENSG00000197110.8
5181	ENSG00000121064.12	ENSG00000278854.1	ENSG00000228889.6
5182	ENSG00000121067.18	ENSG00000278852.1	ENSG00000165188.13
5183	ENSG00000121068.14	ENSG00000278851.1	ENSG00000270066.3
5184	ENSG00000121073.14	ENSG00000278849.1	ENSG00000106571.14
5185	ENSG00000121075.10	ENSG00000278848.2	ENSG00000228513.1
5186	ENSG00000121089.4	ENSG00000278847.1	ENSG00000227660.1
5187	ENSG00000121101.15	ENSG00000278845.5	ENSG00000251508.1
5188	ENSG00000121104.8	ENSG00000278842.1	ENSG00000272417.1
5189	ENSG00000121152.10	ENSG00000278840.1	ENSG00000170185.9
5190	ENSG00000121207.12	ENSG00000278838.1	ENSG00000271974.1
5191	ENSG00000121210.16	ENSG00000278837.1	ENSG00000262470.2

5192	ENSG00000121236.21	ENSG00000278834.1	ENSG00000249102.2
5193	ENSG00000121270.15	ENSG00000278831.1	ENSG00000261267.1
5194	ENSG00000121274.12	ENSG00000278830.1	ENSG00000224797.1
5195	ENSG00000121281.12	ENSG00000278829.1	ENSG00000227910.1
5196	ENSG00000121289.18	ENSG00000278828.1	ENSG00000270385.1
5197	ENSG00000121297.7	ENSG00000278825.1	ENSG00000273933.1
5198	ENSG00000121310.17	ENSG00000278824.1	ENSG00000267325.1
5199	ENSG00000121314.2	ENSG00000278822.1	ENSG00000240733.3
5200	ENSG00000121316.11	ENSG00000278819.1	ENSG00000250412.1
5201	ENSG00000121318.2	ENSG00000278818.1	ENSG00000232194.1
5202	ENSG00000121335.12	ENSG00000278817.1	ENSG00000227018.1
5203	ENSG00000121350.16	ENSG00000278816.1	ENSG00000228028.2
5204	ENSG00000121351.7	ENSG00000278815.1	ENSG00000243160.1
5205	ENSG00000121361.4	ENSG00000278813.1	ENSG00000261740.6
5206	ENSG00000121377.2	ENSG00000278811.4	ENSG00000267620.1
5207	ENSG00000121380.12	ENSG00000278803.2	ENSG00000278546.1
5208	ENSG00000121381.4	ENSG00000278802.1	ENSG00000186704.9
5209	ENSG00000121388.5	ENSG00000278799.1	ENSG00000150394.14
5210	ENSG00000121390.18	ENSG00000278797.1	ENSG00000274799.1
5211	ENSG00000121406.9	ENSG00000278794.1	ENSG00000105583.11
5212	ENSG00000121410.11	ENSG00000278793.1	ENSG00000249717.1
5213	ENSG00000121413.12	ENSG00000278791.1	ENSG00000280496.1
5214	ENSG00000121417.14	ENSG00000278790.1	ENSG00000283611.2
5215	ENSG00000121440.15	ENSG00000278785.1	ENSG00000276054.1
5216	ENSG00000121446.20	ENSG00000278783.1	ENSG00000279134.1
5217	ENSG00000121454.6	ENSG00000278782.1	ENSG00000250366.2
5218	ENSG00000121481.11	ENSG00000278778.1	ENSG00000225814.1
5219	ENSG00000121486.12	ENSG00000278775.1	ENSG00000146143.18
5220	ENSG00000121542.12	ENSG00000278774.1	ENSG00000183840.7
5221	ENSG00000121552.4	ENSG00000278773.1	ENSG00000240268.6
5222	ENSG00000121570.12	ENSG00000278771.1	ENSG00000264491.1
5223	ENSG00000121577.13	ENSG00000278770.1	ENSG00000261063.1
5224	ENSG00000121578.13	ENSG00000278769.1	ENSG00000259278.1
5225	ENSG00000121579.13	ENSG00000278768.2	ENSG00000235500.2
5226	ENSG00000121594.12	ENSG00000278766.2	ENSG00000267587.1
5227	ENSG00000121621.7	ENSG00000278765.1	ENSG00000224649.1
5228	ENSG00000121634.5	ENSG00000278764.1	ENSG00000267567.1
5229	ENSG00000121644.19	ENSG00000278763.1	ENSG00000184967.7
5230	ENSG00000121653.11	ENSG00000278761.1	ENSG00000280354.1
5231	ENSG00000121671.11	ENSG00000278757.1	ENSG00000265646.2
5232	ENSG00000121680.16	ENSG00000278756.1	ENSG00000224844.1
5233	ENSG00000121690.11	ENSG00000278754.1	ENSG00000273679.1
5234	ENSG00000121691.6	ENSG00000278752.1	ENSG00000184012.12
5235	ENSG00000121716.20	ENSG00000278746.1	ENSG00000211900.2
5236	ENSG00000121741.16	ENSG00000278745.1	ENSG00000270981.1
5237	ENSG00000121742.18	ENSG00000278744.1	ENSG00000204261.8
5238	ENSG00000121743.4	ENSG00000278743.1	ENSG00000261695.1
5239	ENSG00000121749.15	ENSG00000278740.1	ENSG00000248840.2
5240	ENSG00000121753.12	ENSG00000278739.1	ENSG00000253632.1
5241	ENSG00000121764.11	ENSG00000278737.1	ENSG00000243711.1
5242	ENSG00000121766.15	ENSG00000278736.1	ENSG00000234415.1
5243	ENSG00000121769.7	ENSG00000278734.1	ENSG00000214146.2
5244	ENSG00000121774.18	ENSG00000278733.1	ENSG00000251127.2

5245	ENSG00000121775.18	ENSG00000278732.1	ENSG00000270141.3
5246	ENSG00000121797.10	ENSG00000278730.1	ENSG00000279180.1
5247	ENSG00000121807.5	ENSG00000278727.1	ENSG00000253823.1
5248	ENSG00000121851.13	ENSG00000278725.1	ENSG00000258728.1
5249	ENSG00000121853.3	ENSG00000278724.1	ENSG00000213383.2
5250	ENSG00000121858.11	ENSG00000278722.1	ENSG00000223431.1
5251	ENSG00000121864.10	ENSG00000278720.1	ENSG00000248256.1
5252	ENSG00000121871.4	ENSG00000278719.1	ENSG00000200463.1
5253	ENSG00000121879.5	ENSG00000278716.1	ENSG00000177993.3
5254	ENSG00000121892.15	ENSG00000278715.1	ENSG00000251533.2
5255	ENSG00000121895.8	ENSG00000278713.1	ENSG00000261575.2
5256	ENSG00000121897.14	ENSG00000278709.2	ENSG00000236975.1
5257	ENSG00000121898.13	ENSG00000278708.1	ENSG00000173988.12
5258	ENSG00000121900.19	ENSG00000278705.1	ENSG00000213866.3
5259	ENSG00000121903.14	ENSG00000278704.1	ENSG00000230219.3
5260	ENSG00000121904.17	ENSG00000278703.1	ENSG00000272359.1
5261	ENSG00000121905.10	ENSG00000278702.1	ENSG00000263257.2
5262	ENSG00000121931.16	ENSG00000278701.1	ENSG00000223443.2
5263	ENSG00000121933.19	ENSG00000278700.1	ENSG00000230637.2
5264	ENSG00000121940.15	ENSG00000278698.1	ENSG00000286215.1
5265	ENSG00000121957.14	ENSG00000278696.1	ENSG00000174233.11
5266	ENSG00000121964.14	ENSG00000278694.1	ENSG00000269095.1
5267	ENSG00000121966.6	ENSG00000278690.1	ENSG00000137875.4
5268	ENSG00000121988.18	ENSG00000278687.1	ENSG00000188800.6
5269	ENSG00000121989.15	ENSG00000278685.4	ENSG00000126266.3
5270	ENSG00000122008.15	ENSG00000278684.1	ENSG00000242641.5
5271	ENSG00000122012.14	ENSG00000278683.1	ENSG00000265737.1
5272	ENSG00000122025.14	ENSG00000278681.2	ENSG00000130363.11
5273	ENSG00000122026.10	ENSG00000278678.1	ENSG00000236060.2
5274	ENSG00000122033.14	ENSG00000278677.1	ENSG00000277009.1
5275	ENSG00000122034.15	ENSG00000278674.1	ENSG00000229086.4
5276	ENSG00000122035.6	ENSG00000278673.1	ENSG00000284613.1
5277	ENSG00000122042.9	ENSG00000278672.1	ENSG00000272055.1
5278	ENSG00000122043.10	ENSG00000278668.1	ENSG00000272854.1
5279	ENSG00000122068.13	ENSG00000278667.1	ENSG00000249669.9
5280	ENSG00000122085.17	ENSG00000278665.1	ENSG00000156689.7
5281	ENSG00000122121.11	ENSG00000278664.1	ENSG00000233217.2
5282	ENSG00000122122.10	ENSG00000278662.4	ENSG00000236495.2
5283	ENSG00000122126.17	ENSG00000278661.1	ENSG00000232642.1
5284	ENSG00000122133.17	ENSG00000278658.1	ENSG00000230628.1
5285	ENSG00000122136.13	ENSG00000278657.1	ENSG00000213538.5
5286	ENSG00000122140.11	ENSG00000278655.1	ENSG00000225647.1
5287	ENSG00000122145.15	ENSG00000278654.1	ENSG00000223741.1
5288	ENSG00000122176.12	ENSG00000278650.1	ENSG00000224094.1
5289	ENSG00000122180.5	ENSG00000278647.1	ENSG00000182574.8
5290	ENSG00000122188.12	ENSG00000278646.1	ENSG00000250519.6
5291	ENSG00000122194.18	ENSG00000278643.1	ENSG00000235244.3
5292	ENSG00000122203.15	ENSG00000278642.1	ENSG00000227042.1
5293	ENSG00000122218.15	ENSG00000278641.1	ENSG00000167034.10
5294	ENSG00000122223.13	ENSG00000278640.1	ENSG00000172382.10
5295	ENSG00000122224.18	ENSG00000278638.1	ENSG00000060709.15
5296	ENSG00000122254.7	ENSG00000278637.1	ENSG00000083457.12
5297	ENSG00000122257.20	ENSG00000278636.1	ENSG00000232265.7

5298	ENSG00000122299.12	ENSG00000278635.1	ENSG00000284686.1
5299	ENSG00000122304.10	ENSG00000278633.1	ENSG00000224655.6
5300	ENSG00000122335.16	ENSG00000278631.1	ENSG00000230246.7
5301	ENSG00000122359.18	ENSG00000278630.1	ENSG00000255152.8
5302	ENSG00000122367.19	ENSG00000278627.1	ENSG00000258481.1
5303	ENSG00000122375.12	ENSG00000278626.1	ENSG00000249848.1
5304	ENSG00000122376.11	ENSG00000278625.1	ENSG00000233129.1
5305	ENSG00000122378.14	ENSG00000278621.1	ENSG00000216588.9
5306	ENSG00000122386.10	ENSG00000278619.5	ENSG00000053524.12
5307	ENSG00000122390.19	ENSG00000278618.1	ENSG00000231202.1
5308	ENSG00000122406.13	ENSG00000278617.1	ENSG00000233351.1
5309	ENSG00000122417.15	ENSG00000278616.1	ENSG00000235486.1
5310	ENSG00000122420.10	ENSG00000278615.4	ENSG00000237379.1
5311	ENSG00000122432.17	ENSG00000278611.1	ENSG00000268199.2
5312	ENSG00000122435.10	ENSG00000278610.1	ENSG00000268267.1
5313	ENSG00000122477.12	ENSG00000278607.1	ENSG00000179256.2
5314	ENSG00000122481.17	ENSG00000278604.1	ENSG00000269376.1
5315	ENSG00000122482.21	ENSG00000278603.1	ENSG00000256533.1
5316	ENSG00000122483.17	ENSG00000278602.1	ENSG00000270620.1
5317	ENSG00000122484.9	ENSG00000278600.1	ENSG00000226711.6
5318	ENSG00000122490.19	ENSG00000278599.5	ENSG00000198758.10
5319	ENSG00000122507.20	ENSG00000278598.1	ENSG00000262660.1
5320	ENSG00000122512.16	ENSG00000278596.1	ENSG00000275263.1
5321	ENSG00000122515.15	ENSG00000278595.1	ENSG00000245857.2
5322	ENSG00000122543.10	ENSG00000278594.1	ENSG00000167768.4
5323	ENSG00000122545.19	ENSG00000278593.1	ENSG00000262980.1
5324	ENSG00000122547.11	ENSG00000278592.1	ENSG00000113648.16
5325	ENSG00000122548.5	ENSG00000278591.1	ENSG00000164385.8
5326	ENSG00000122550.18	ENSG00000278590.1	ENSG00000274973.1
5327	ENSG00000122557.10	ENSG00000278589.1	ENSG00000224807.5
5328	ENSG00000122565.19	ENSG00000278588.1	ENSG00000249272.1
5329	ENSG00000122566.21	ENSG00000278587.1	ENSG00000236257.1
5330	ENSG00000122574.10	ENSG00000278586.1	ENSG00000260357.1
5331	ENSG00000122584.13	ENSG00000278585.1	ENSG00000092009.10
5332	ENSG00000122585.8	ENSG00000278582.1	ENSG00000255775.1
5333	ENSG00000122591.11	ENSG00000278581.1	ENSG00000255372.1
5334	ENSG00000122592.8	ENSG00000278580.1	ENSG00000103248.18
5335	ENSG00000122641.11	ENSG00000278577.1	ENSG00000268282.1
5336	ENSG00000122642.11	ENSG00000278573.1	ENSG00000273249.1
5337	ENSG00000122643.20	ENSG00000278572.1	ENSG00000182263.14
5338	ENSG00000122644.13	ENSG00000278571.1	ENSG00000275128.1
5339	ENSG00000122674.11	ENSG00000278570.4	ENSG00000268926.3
5340	ENSG00000122678.17	ENSG00000278569.1	ENSG00000238205.3
5341	ENSG00000122679.8	ENSG00000278561.1	ENSG00000283050.2
5342	ENSG00000122687.17	ENSG00000278558.4	ENSG00000164104.11
5343	ENSG00000122691.13	ENSG00000278554.1	ENSG00000239930.2
5344	ENSG00000122692.9	ENSG00000278552.1	ENSG00000229758.2
5345	ENSG00000122694.16	ENSG00000278551.1	ENSG00000181378.14
5346	ENSG00000122696.14	ENSG00000278549.1	ENSG00000229750.1
5347	ENSG00000122705.17	ENSG00000278546.1	ENSG00000261578.1
5348	ENSG00000122707.12	ENSG00000278543.1	ENSG00000155428.12
5349	ENSG00000122711.9	ENSG00000278541.1	ENSG00000228081.2
5350	ENSG00000122728.6	ENSG00000278540.5	ENSG00000228360.1

5351	ENSG00000122729.19	ENSG00000278537.1	ENSG00000178429.9
5352	ENSG00000122733.12	ENSG00000278535.5	ENSG00000224515.1
5353	ENSG00000122735.16	ENSG00000278532.1	ENSG00000234042.1
5354	ENSG00000122741.16	ENSG00000278531.1	ENSG00000248373.5
5355	ENSG00000122756.15	ENSG00000278530.4	ENSG00000255366.1
5356	ENSG00000122778.9	ENSG00000278529.1	ENSG00000119913.5
5357	ENSG00000122779.18	ENSG00000278528.1	ENSG00000255393.2
5358	ENSG00000122783.16	ENSG00000278527.1	ENSG00000104903.4
5359	ENSG00000122787.15	ENSG00000278526.1	ENSG00000198648.11
5360	ENSG00000122824.11	ENSG00000278525.1	ENSG00000234933.1
5361	ENSG00000122852.14	ENSG00000278524.1	ENSG00000276214.1
5362	ENSG00000122859.4	ENSG00000278523.1	ENSG00000230045.4
5363	ENSG00000122861.16	ENSG00000278522.4	ENSG00000143995.20
5364	ENSG00000122862.5	ENSG00000278520.1	ENSG00000253821.1
5365	ENSG00000122863.6	ENSG00000278518.1	ENSG00000254846.1
5366	ENSG00000122870.11	ENSG00000278517.1	ENSG00000234835.1
5367	ENSG00000122872.8	ENSG00000278514.1	ENSG00000262539.1
5368	ENSG00000122873.12	ENSG00000278513.1	ENSG00000112964.14
5369	ENSG00000122877.16	ENSG00000278510.1	ENSG00000250510.8
5370	ENSG00000122882.10	ENSG00000278505.4	ENSG00000256176.1
5371	ENSG00000122884.12	ENSG00000278502.1	ENSG00000260381.2
5372	ENSG00000122912.15	ENSG00000278500.1	ENSG00000200601.1
5373	ENSG00000122952.17	ENSG00000278499.2	ENSG00000270361.1
5374	ENSG00000122958.15	ENSG00000278498.1	ENSG00000285814.1
5375	ENSG00000122965.11	ENSG00000278497.1	ENSG00000271047.1
5376	ENSG00000122966.16	ENSG00000278496.1	ENSG00000243176.5
5377	ENSG00000122970.16	ENSG00000278493.1	ENSG00000181374.8
5378	ENSG00000122971.9	ENSG00000278492.1	ENSG00000224521.1
5379	ENSG00000122986.13	ENSG00000278487.1	ENSG00000280394.1
5380	ENSG00000123009.4	ENSG00000278486.1	ENSG00000239744.3
5381	ENSG00000123064.12	ENSG00000278485.1	ENSG00000270909.1
5382	ENSG00000123066.8	ENSG00000278484.1	ENSG00000175497.16
5383	ENSG00000123080.11	ENSG00000278483.1	ENSG00000260186.5
5384	ENSG00000123091.5	ENSG00000278482.1	ENSG00000169174.10
5385	ENSG00000123094.15	ENSG00000278478.1	ENSG00000274183.1
5386	ENSG00000123095.6	ENSG00000278477.1	ENSG00000270225.1
5387	ENSG00000123096.11	ENSG00000278475.1	ENSG00000157045.9
5388	ENSG00000123104.12	ENSG00000278473.1	ENSG00000246820.2
5389	ENSG00000123106.10	ENSG00000278472.1	ENSG00000242034.1
5390	ENSG00000123119.12	ENSG00000278469.1	ENSG00000180913.3
5391	ENSG00000123124.13	ENSG00000278467.1	ENSG00000006659.13
5392	ENSG00000123130.17	ENSG00000278465.1	ENSG00000159197.3
5393	ENSG00000123131.13	ENSG00000278464.1	ENSG00000162595.6
5394	ENSG00000123136.14	ENSG00000278463.1	ENSG00000285712.1
5395	ENSG00000123143.12	ENSG00000278462.1	ENSG00000147168.12
5396	ENSG00000123144.11	ENSG00000278459.1	ENSG00000251733.1
5397	ENSG00000123146.20	ENSG00000278457.1	ENSG00000281560.1
5398	ENSG00000123154.12	ENSG00000278456.1	ENSG00000278275.1
5399	ENSG00000123159.16	ENSG00000278455.1	ENSG00000229625.3
5400	ENSG00000123165.8	ENSG00000278454.1	ENSG00000230355.1
5401	ENSG00000123171.6	ENSG00000278449.1	ENSG00000156110.13
5402	ENSG00000123178.15	ENSG00000278447.1	ENSG00000140285.10
5403	ENSG00000123179.14	ENSG00000278446.1	ENSG00000265494.1

5404	ENSG00000123191.14	ENSG00000278445.1	ENSG00000240527.1
5405	ENSG00000123200.16	ENSG00000278441.1	ENSG00000240238.1
5406	ENSG00000123201.14	ENSG00000278438.1	ENSG00000260156.1
5407	ENSG00000123213.23	ENSG00000278434.1	ENSG00000284966.2
5408	ENSG00000123240.17	ENSG00000278433.1	ENSG00000182070.5
5409	ENSG00000123243.15	ENSG00000278428.1	ENSG00000285829.1
5410	ENSG00000123268.9	ENSG00000278424.1	ENSG00000252118.1
5411	ENSG00000123297.18	ENSG00000278422.1	ENSG00000232335.1
5412	ENSG00000123307.4	ENSG00000278421.1	ENSG00000260029.2
5413	ENSG00000123329.18	ENSG00000278420.1	ENSG00000006282.21
5414	ENSG00000123338.13	ENSG00000278419.1	ENSG00000233896.2
5415	ENSG00000123342.16	ENSG00000278418.1	ENSG00000204637.4
5416	ENSG00000123349.14	ENSG00000278416.1	ENSG00000271765.1
5417	ENSG00000123352.17	ENSG00000278413.1	ENSG00000259732.1
5418	ENSG00000123353.10	ENSG00000278412.1	ENSG00000197889.10
5419	ENSG00000123358.20	ENSG00000278410.1	ENSG00000224809.2
5420	ENSG00000123360.12	ENSG00000278408.1	ENSG00000255568.3
5421	ENSG00000123374.11	ENSG00000278406.1	ENSG00000232496.2
5422	ENSG00000123384.13	ENSG00000278404.1	ENSG00000240963.1
5423	ENSG00000123388.4	ENSG00000278400.1	ENSG00000277475.1
5424	ENSG00000123395.14	ENSG00000278399.1	ENSG00000229646.2
5425	ENSG00000123405.14	ENSG00000278396.1	ENSG00000106992.19
5426	ENSG00000123407.4	ENSG00000278395.1	ENSG00000003756.16
5427	ENSG00000123411.15	ENSG00000278391.1	ENSG00000271817.2
5428	ENSG00000123415.15	ENSG00000278390.4	ENSG00000228604.3
5429	ENSG00000123416.15	ENSG00000278389.1	ENSG00000117010.17
5430	ENSG00000123427.17	ENSG00000278388.1	ENSG00000012779.11
5431	ENSG00000123444.14	ENSG00000278385.1	ENSG00000259867.5
5432	ENSG00000123447.6	ENSG00000278384.1	ENSG00000232953.1
5433	ENSG00000123453.18	ENSG00000278383.1	ENSG00000094916.16
5434	ENSG00000123454.11	ENSG00000278382.1	ENSG00000275799.1
5435	ENSG00000123472.12	ENSG00000278381.1	ENSG00000277147.6
5436	ENSG00000123473.15	ENSG00000278374.1	ENSG00000270823.1
5437	ENSG00000123485.12	ENSG00000278370.1	ENSG00000095596.12
5438	ENSG00000123496.8	ENSG00000278367.1	ENSG00000284724.1
5439	ENSG00000123500.10	ENSG00000278359.1	ENSG00000229083.1
5440	ENSG00000123505.16	ENSG00000278358.1	ENSG00000236611.1
5441	ENSG00000123545.6	ENSG00000278356.1	ENSG00000234185.2
5442	ENSG00000123552.17	ENSG00000278351.1	ENSG00000248327.1
5443	ENSG00000123560.14	ENSG00000278349.1	ENSG00000219274.1
5444	ENSG00000123561.15	ENSG00000278348.1	ENSG00000270090.5
5445	ENSG00000123562.17	ENSG00000278346.1	ENSG00000232110.7
5446	ENSG00000123569.8	ENSG00000278344.1	ENSG00000267627.5
5447	ENSG00000123570.4	ENSG00000278343.1	ENSG00000227227.1
5448	ENSG00000123572.17	ENSG00000278342.1	ENSG00000232557.3
5449	ENSG00000123575.9	ENSG00000278341.1	ENSG00000256381.1
5450	ENSG00000123576.5	ENSG00000278340.1	ENSG00000112304.11
5451	ENSG00000123584.7	ENSG00000278338.4	ENSG00000255964.1
5452	ENSG00000123594.5	ENSG00000278337.1	ENSG00000226664.1
5453	ENSG00000123595.7	ENSG00000278334.1	ENSG00000170324.21
5454	ENSG00000123600.19	ENSG00000278333.1	ENSG00000270996.1
5455	ENSG00000123607.15	ENSG00000278332.1	ENSG00000257411.1
5456	ENSG00000123609.10	ENSG00000278331.1	ENSG00000242593.6

5457	ENSG00000123610.5	ENSG00000278330.1	ENSG00000164604.12
5458	ENSG00000123612.16	ENSG00000278328.1	ENSG00000235493.2
5459	ENSG00000123636.18	ENSG00000278324.1	ENSG00000120669.16
5460	ENSG00000123643.13	ENSG00000278319.1	ENSG00000265257.5
5461	ENSG00000123684.13	ENSG00000278318.5	ENSG00000159450.12
5462	ENSG00000123685.9	ENSG00000278317.1	ENSG00000253445.1
5463	ENSG00000123689.6	ENSG00000278313.1	ENSG00000239005.1
5464	ENSG00000123700.4	ENSG00000278311.5	ENSG00000244757.1
5465	ENSG00000123728.10	ENSG00000278305.1	ENSG00000225761.1
5466	ENSG00000123737.12	ENSG00000278301.1	ENSG00000138231.12
5467	ENSG00000123739.11	ENSG00000278294.1	ENSG00000117153.16
5468	ENSG00000123810.8	ENSG00000278292.1	ENSG00000101940.18
5469	ENSG00000123815.12	ENSG00000278291.1	ENSG00000138696.10
5470	ENSG00000123836.15	ENSG00000278289.4	ENSG00000284678.1
5471	ENSG00000123838.11	ENSG00000278287.1	ENSG00000184761.8
5472	ENSG00000123843.13	ENSG00000278283.1	ENSG00000224751.2
5473	ENSG00000123870.10	ENSG00000278281.1	ENSG00000273225.4
5474	ENSG00000123892.12	ENSG00000278278.1	ENSG00000258603.3
5475	ENSG00000123901.9	ENSG00000278276.1	ENSG00000185477.5
5476	ENSG00000123908.12	ENSG00000278275.1	ENSG00000254211.5
5477	ENSG00000123933.17	ENSG00000278274.1	ENSG00000256100.1
5478	ENSG00000123965.13	ENSG00000278273.1	ENSG00000277039.1
5479	ENSG00000123975.5	ENSG00000278272.1	ENSG00000153930.11
5480	ENSG00000123977.10	ENSG00000278267.1	ENSG00000123977.10
5481	ENSG00000123983.14	ENSG00000278266.1	ENSG00000232581.1
5482	ENSG00000123989.14	ENSG00000278265.1	ENSG00000229921.6
5483	ENSG00000123992.19	ENSG00000278264.1	ENSG00000258666.1
5484	ENSG00000123999.5	ENSG00000278263.2	ENSG00000260148.1
5485	ENSG00000124003.12	ENSG00000278261.1	ENSG00000112116.9
5486	ENSG00000124006.15	ENSG00000278259.4	ENSG00000226792.7
5487	ENSG00000124019.10	ENSG00000278256.1	ENSG00000224908.1
5488	ENSG00000124067.17	ENSG00000278255.1	ENSG00000167858.13
5489	ENSG00000124074.12	ENSG00000278254.4	ENSG00000269980.1
5490	ENSG00000124089.4	ENSG00000278250.1	ENSG00000260132.1
5491	ENSG00000124091.9	ENSG00000278249.1	ENSG00000229604.2
5492	ENSG00000124092.12	ENSG00000278242.1	ENSG00000262302.1
5493	ENSG00000124097.7	ENSG00000278239.1	ENSG00000157734.14
5494	ENSG00000124098.10	ENSG00000278238.1	ENSG00000226003.2
5495	ENSG00000124102.5	ENSG00000278237.1	ENSG00000253414.2
5496	ENSG00000124103.9	ENSG00000278236.1	ENSG00000235508.3
5497	ENSG00000124104.19	ENSG00000278234.1	ENSG00000204709.4
5498	ENSG00000124107.5	ENSG00000278233.1	ENSG00000214278.4
5499	ENSG00000124116.19	ENSG00000278231.1	ENSG00000249189.1
5500	ENSG00000124120.11	ENSG00000278226.1	ENSG00000274252.1
5501	ENSG00000124126.14	ENSG00000278224.6	ENSG00000199546.1
5502	ENSG00000124134.9	ENSG00000278223.1	ENSG00000279584.1
5503	ENSG00000124140.13	ENSG00000278222.1	ENSG00000200170.1
5504	ENSG00000124143.10	ENSG00000278221.1	ENSG00000206899.1
5505	ENSG00000124145.6	ENSG00000278218.1	ENSG00000260265.1
5506	ENSG00000124151.19	ENSG00000278217.1	ENSG00000245330.4
5507	ENSG00000124155.18	ENSG00000278215.1	ENSG00000235142.9
5508	ENSG00000124157.6	ENSG00000278214.1	ENSG00000168913.7
5509	ENSG00000124159.15	ENSG00000278213.1	ENSG00000230730.1

5510	ENSG00000124160.12	ENSG00000278212.2	ENSG00000260814.2
5511	ENSG00000124164.15	ENSG00000278206.1	ENSG00000156219.16
5512	ENSG00000124171.9	ENSG00000278205.1	ENSG00000227118.3
5513	ENSG00000124172.10	ENSG00000278204.1	ENSG00000211676.2
5514	ENSG00000124177.15	ENSG00000278202.1	ENSG00000261359.2
5515	ENSG00000124181.14	ENSG00000278200.1	ENSG00000267656.1
5516	ENSG00000124191.18	ENSG00000278198.1	ENSG00000257121.1
5517	ENSG00000124193.15	ENSG00000278197.1	ENSG00000241073.1
5518	ENSG00000124194.16	ENSG00000278196.3	ENSG00000260892.1
5519	ENSG00000124196.5	ENSG00000278195.2	ENSG00000224004.2
5520	ENSG00000124198.9	ENSG00000278193.1	ENSG00000113811.11
5521	ENSG00000124201.15	ENSG00000278192.1	ENSG00000258915.1
5522	ENSG00000124203.6	ENSG00000278189.1	ENSG00000234159.1
5523	ENSG00000124205.17	ENSG00000278188.1	ENSG00000280268.1
5524	ENSG00000124207.17	ENSG00000278184.1	ENSG00000171346.16
5525	ENSG00000124208.16	ENSG00000278182.1	ENSG00000240012.1
5526	ENSG00000124209.4	ENSG00000278181.1	ENSG00000261647.1
5527	ENSG00000124212.6	ENSG00000278180.1	ENSG00000225533.1
5528	ENSG00000124214.19	ENSG00000278177.1	ENSG00000267359.1
5529	ENSG00000124215.16	ENSG00000278175.3	ENSG00000253399.1
5530	ENSG00000124216.4	ENSG00000278172.1	ENSG00000270294.1
5531	ENSG00000124217.4	ENSG00000278171.1	ENSG00000243024.6
5532	ENSG00000124222.22	ENSG00000278160.1	ENSG00000248202.1
5533	ENSG00000124224.17	ENSG00000278159.1	ENSG00000267353.1
5534	ENSG00000124225.16	ENSG00000278158.1	ENSG00000270302.1
5535	ENSG00000124226.11	ENSG00000278156.1	ENSG00000234423.1
5536	ENSG00000124227.5	ENSG00000278153.1	ENSG00000249616.1
5537	ENSG00000124228.14	ENSG00000278151.1	ENSG00000271519.1
5538	ENSG00000124232.11	ENSG00000278147.1	ENSG00000230507.5
5539	ENSG00000124233.12	ENSG00000278146.1	ENSG00000174992.7
5540	ENSG00000124237.5	ENSG00000278143.1	ENSG00000168398.6
5541	ENSG00000124243.17	ENSG00000278139.1	ENSG00000248729.1
5542	ENSG00000124249.7	ENSG00000278138.2	ENSG00000168081.9
5543	ENSG00000124251.11	ENSG00000278135.1	ENSG00000206941.1
5544	ENSG00000124253.11	ENSG00000278133.1	ENSG00000231397.1
5545	ENSG00000124256.15	ENSG00000278131.1	ENSG00000278701.1
5546	ENSG00000124257.6	ENSG00000278130.1	ENSG00000258884.1
5547	ENSG00000124260.12	ENSG00000278129.2	ENSG00000213755.3
5548	ENSG00000124275.14	ENSG00000278128.1	ENSG00000229145.1
5549	ENSG00000124279.12	ENSG00000278126.1	ENSG00000182667.14
5550	ENSG00000124299.14	ENSG00000278124.1	ENSG00000104371.5
5551	ENSG00000124302.13	ENSG00000278123.1	ENSG00000180532.10
5552	ENSG00000124313.15	ENSG00000278122.1	ENSG00000271134.1
5553	ENSG00000124333.16	ENSG00000278113.1	ENSG00000248588.2
5554	ENSG00000124333.16_PAR_Y	ENSG00000278112.1	ENSG00000245479.2
5555	ENSG00000124334.17	ENSG00000278110.1	ENSG00000184210.5
5556	ENSG00000124334.17_PAR_Y	ENSG00000278109.1	ENSG00000249407.1
5557	ENSG00000124343.13	ENSG00000278108.1	ENSG00000095203.14
5558	ENSG00000124356.16	ENSG00000278107.1	ENSG00000215899.3
5559	ENSG00000124357.13	ENSG00000278106.1	ENSG00000198589.12
5560	ENSG00000124370.11	ENSG00000278104.1	ENSG00000244582.2
5561	ENSG00000124374.9	ENSG00000278103.1	ENSG00000170044.8
5562	ENSG00000124380.11	ENSG00000278102.1	ENSG00000272366.1

5563	ENSG00000124383.9	ENSG00000278100.1	ENSG00000120160.11
5564	ENSG00000124391.4	ENSG00000278099.1	ENSG00000233924.1
5565	ENSG00000124399.4	ENSG00000278098.1	ENSG00000240471.1
5566	ENSG00000124406.16	ENSG00000278097.1	ENSG00000171227.7
5567	ENSG00000124422.12	ENSG00000278095.1	ENSG00000255459.1
5568	ENSG00000124429.18	ENSG00000278089.1	ENSG00000213216.2
5569	ENSG00000124440.15	ENSG00000278085.4	ENSG00000224407.1
5570	ENSG00000124444.15	ENSG00000278084.1	ENSG00000251571.1
5571	ENSG00000124449.7	ENSG00000278083.1	ENSG00000161649.13
5572	ENSG00000124459.12	ENSG00000278078.1	ENSG00000240893.1
5573	ENSG00000124466.9	ENSG00000278077.1	ENSG00000268842.1
5574	ENSG00000124467.18	ENSG00000278075.1	ENSG00000248394.1
5575	ENSG00000124469.12	ENSG00000278073.1	ENSG00000235267.1
5576	ENSG00000124479.10	ENSG00000278072.1	ENSG00000278941.1
5577	ENSG00000124486.13	ENSG00000278071.1	ENSG00000134574.11
5578	ENSG00000124490.14	ENSG00000278069.1	ENSG00000226468.2
5579	ENSG00000124491.15	ENSG00000278068.1	ENSG00000273049.1
5580	ENSG00000124493.13	ENSG00000278066.1	ENSG00000167100.14
5581	ENSG00000124496.12	ENSG00000278060.1	ENSG00000258378.1
5582	ENSG00000124507.11	ENSG00000278058.1	ENSG00000236403.1
5583	ENSG00000124508.16	ENSG00000278057.4	ENSG00000271396.1
5584	ENSG00000124523.15	ENSG00000278055.1	ENSG00000156671.14
5585	ENSG00000124532.14	ENSG00000278054.1	ENSG00000136315.4
5586	ENSG00000124535.15	ENSG00000278053.5	ENSG00000236187.1
5587	ENSG00000124541.7	ENSG00000278052.1	ENSG00000242419.5
5588	ENSG00000124549.14	ENSG00000278050.1	ENSG00000277186.1
5589	ENSG00000124557.12	ENSG00000278048.1	ENSG00000120937.9
5590	ENSG00000124562.10	ENSG00000278045.1	ENSG00000250039.3
5591	ENSG00000124564.17	ENSG00000278044.1	ENSG00000136944.17
5592	ENSG00000124568.10	ENSG00000278041.1	ENSG00000267056.2
5593	ENSG00000124570.19	ENSG00000278040.1	ENSG00000232883.1
5594	ENSG00000124571.18	ENSG00000278039.1	ENSG00000238207.1
5595	ENSG00000124574.15	ENSG00000278038.1	ENSG00000224563.1
5596	ENSG00000124575.6	ENSG00000278035.1	ENSG00000235424.1
5597	ENSG00000124587.14	ENSG00000278034.1	ENSG00000157927.16
5598	ENSG00000124588.20	ENSG00000278030.1	ENSG00000215452.5
5599	ENSG00000124593.16	ENSG00000278028.1	ENSG00000244193.1
5600	ENSG00000124596.17	ENSG00000278026.1	ENSG00000242993.1
5601	ENSG00000124602.10	ENSG00000278023.6	ENSG00000223484.7
5602	ENSG00000124608.5	ENSG00000278022.1	ENSG00000148215.4
5603	ENSG00000124610.4	ENSG00000278020.1	ENSG00000198574.6
5604	ENSG00000124613.8	ENSG00000278017.1	ENSG00000236527.1
5605	ENSG00000124614.15	ENSG00000278013.1	ENSG00000272894.5
5606	ENSG00000124615.20	ENSG00000278012.1	ENSG00000226544.4
5607	ENSG00000124635.8	ENSG00000278011.1	ENSG00000249379.1
5608	ENSG00000124641.16	ENSG00000278010.1	ENSG00000258562.2
5609	ENSG00000124657.1	ENSG00000278009.1	ENSG00000214891.9
5610	ENSG00000124659.6	ENSG00000278008.1	ENSG00000263426.2
5611	ENSG00000124664.11	ENSG00000278007.1	ENSG00000234213.1
5612	ENSG00000124678.18	ENSG00000278004.1	ENSG00000253844.1
5613	ENSG00000124688.13	ENSG00000278002.1	ENSG00000260602.2
5614	ENSG00000124701.5	ENSG00000278001.1	ENSG00000228728.1
5615	ENSG00000124702.18	ENSG00000278000.1	ENSG00000248100.2

5616	ENSG00000124713.6	ENSG00000277999.1	ENSG00000138100.13
5617	ENSG00000124721.17	ENSG00000277998.1	ENSG00000251273.3
5618	ENSG00000124731.13	ENSG00000277997.1	ENSG00000225937.2
5619	ENSG00000124733.4	ENSG00000277994.1	ENSG00000228407.2
5620	ENSG00000124743.6	ENSG00000277991.4	ENSG00000280176.1
5621	ENSG00000124749.17	ENSG00000277988.1	ENSG00000232736.1
5622	ENSG00000124762.13	ENSG00000277987.1	ENSG00000253161.5
5623	ENSG00000124766.6	ENSG00000277986.1	ENSG00000239910.3
5624	ENSG00000124767.7	ENSG00000277985.1	ENSG00000173699.16
5625	ENSG00000124772.12	ENSG00000277984.1	ENSG00000222501.1
5626	ENSG00000124780.14	ENSG00000277981.1	ENSG00000233979.1
5627	ENSG00000124782.20	ENSG00000277978.1	ENSG00000253111.2
5628	ENSG00000124783.14	ENSG00000277977.1	ENSG00000243305.1
5629	ENSG00000124784.9	ENSG00000277975.1	ENSG00000234720.3
5630	ENSG00000124785.9	ENSG00000277973.1	ENSG00000261779.1
5631	ENSG00000124786.11	ENSG00000277972.1	ENSG00000261737.1
5632	ENSG00000124787.13	ENSG00000277971.1	ENSG00000237385.1
5633	ENSG00000124788.18	ENSG00000277969.1	ENSG00000272021.1
5634	ENSG00000124789.11	ENSG00000277968.1	ENSG00000237738.1
5635	ENSG00000124795.16	ENSG00000277967.1	ENSG00000228423.2
5636	ENSG00000124802.12	ENSG00000277966.1	ENSG00000229142.1
5637	ENSG00000124812.14	ENSG00000277965.1	ENSG00000273262.1
5638	ENSG00000124813.22	ENSG00000277959.1	ENSG00000074317.11
5639	ENSG00000124818.15	ENSG00000277958.1	ENSG00000185972.5
5640	ENSG00000124827.6	ENSG00000277957.1	ENSG00000218153.2
5641	ENSG00000124831.19	ENSG00000277954.1	ENSG00000228275.1
5642	ENSG00000124835.2	ENSG00000277952.1	ENSG00000224593.1
5643	ENSG00000124839.13	ENSG00000277950.1	ENSG00000233822.4
5644	ENSG00000124875.10	ENSG00000277948.1	ENSG00000197617.8
5645	ENSG00000124882.4	ENSG00000277947.1	ENSG00000212182.2
5646	ENSG00000124900.12	ENSG00000277946.1	ENSG00000272912.1
5647	ENSG00000124915.10	ENSG00000277945.1	ENSG00000262179.2
5648	ENSG00000124920.13	ENSG00000277942.1	ENSG00000260133.1
5649	ENSG00000124935.4	ENSG00000277941.1	ENSG00000187068.3
5650	ENSG00000124939.6	ENSG00000277938.1	ENSG00000173285.4
5651	ENSG00000124942.14	ENSG00000277935.1	ENSG00000140374.16
5652	ENSG00000125037.12	ENSG00000277932.2	ENSG00000250273.1
5653	ENSG00000125046.14	ENSG00000277930.1	ENSG00000270299.1
5654	ENSG00000125084.11	ENSG00000277927.1	ENSG00000277534.1
5655	ENSG00000125089.17	ENSG00000277922.1	ENSG00000158022.6
5656	ENSG00000125107.18	ENSG00000277920.1	ENSG00000267262.1
5657	ENSG00000125122.15	ENSG00000277919.1	ENSG00000132840.10
5658	ENSG00000125124.12	ENSG00000277918.1	ENSG00000231927.1
5659	ENSG00000125144.13	ENSG00000277916.1	ENSG00000267432.5
5660	ENSG00000125148.7	ENSG00000277914.1	ENSG00000262172.1
5661	ENSG00000125149.11	ENSG00000277913.1	ENSG00000271394.1
5662	ENSG00000125166.13	ENSG00000277912.1	ENSG00000260112.1
5663	ENSG00000125170.11	ENSG00000277911.1	ENSG00000257582.5
5664	ENSG00000125207.7	ENSG00000277907.1	ENSG00000248824.2
5665	ENSG00000125245.12	ENSG00000277904.1	ENSG00000255054.3
5666	ENSG00000125246.15	ENSG00000277903.1	ENSG00000141570.11
5667	ENSG00000125247.15	ENSG00000277901.1	ENSG00000245571.6
5668	ENSG00000125249.7	ENSG00000277900.1	ENSG00000035664.11

5669	ENSG00000125255.6	ENSG00000277899.1	ENSG00000283462.1
5670	ENSG00000125257.15	ENSG00000277895.1	ENSG00000249870.1
5671	ENSG00000125266.7	ENSG00000277893.2	ENSG00000254850.2
5672	ENSG00000125285.5	ENSG00000277892.1	ENSG00000048544.6
5673	ENSG00000125304.9	ENSG00000277890.1	ENSG00000134339.8
5674	ENSG00000125319.14	ENSG00000277889.1	ENSG00000277184.1
5675	ENSG00000125337.18	ENSG00000277888.1	ENSG00000270314.1
5676	ENSG00000125347.14	ENSG00000277887.1	ENSG00000253737.1
5677	ENSG00000125351.11	ENSG00000277883.1	ENSG00000207248.1
5678	ENSG00000125352.5	ENSG00000277882.1	ENSG00000203709.11
5679	ENSG00000125354.23	ENSG00000277881.1	ENSG00000166450.13
5680	ENSG00000125355.15	ENSG00000277880.1	ENSG00000278935.1
5681	ENSG00000125356.7	ENSG00000277879.1	ENSG00000258038.5
5682	ENSG00000125363.14	ENSG00000277878.1	ENSG00000256204.1
5683	ENSG00000125375.14	ENSG00000277876.1	ENSG00000205327.3
5684	ENSG00000125378.16	ENSG00000277873.1	ENSG00000285984.1
5685	ENSG00000125384.7	ENSG00000277872.1	ENSG00000233229.1
5686	ENSG00000125385.9	ENSG00000277870.5	ENSG00000238137.2
5687	ENSG00000125386.15	ENSG00000277869.1	ENSG00000226674.10
5688	ENSG00000125388.20	ENSG00000277867.1	ENSG00000250714.3
5689	ENSG00000125398.7	ENSG00000277866.1	ENSG00000276839.1
5690	ENSG00000125409.13	ENSG00000277865.4	ENSG00000276272.1
5691	ENSG00000125414.19	ENSG00000277864.1	ENSG00000271499.1
5692	ENSG00000125430.9	ENSG00000277863.1	ENSG00000256672.1
5693	ENSG00000125434.11	ENSG00000277862.1	ENSG00000232830.5
5694	ENSG00000125445.11	ENSG00000277859.1	ENSG00000131096.10
5695	ENSG00000125447.17	ENSG00000277858.1	ENSG00000218347.2
5696	ENSG00000125449.7	ENSG00000277856.1	ENSG00000253824.1
5697	ENSG00000125450.11	ENSG00000277855.1	ENSG00000257336.1
5698	ENSG00000125454.12	ENSG00000277852.1	ENSG00000274503.1
5699	ENSG00000125457.14	ENSG00000277851.1	ENSG00000225499.1
5700	ENSG00000125458.7	ENSG00000277846.1	ENSG00000237409.2
5701	ENSG00000125459.15	ENSG00000277842.1	ENSG00000135625.7
5702	ENSG00000125462.17	ENSG00000277841.1	ENSG00000104728.16
5703	ENSG00000125482.13	ENSG00000277840.1	ENSG00000233251.7
5704	ENSG00000125484.12	ENSG00000277837.1	ENSG00000207507.1
5705	ENSG00000125485.17	ENSG00000277836.1	ENSG00000213157.3
5706	ENSG00000125492.10	ENSG00000277831.1	ENSG00000256091.1
5707	ENSG00000125498.20	ENSG00000277830.1	ENSG00000232369.1
5708	ENSG00000125503.13	ENSG00000277829.1	ENSG00000266076.1
5709	ENSG00000125505.17	ENSG00000277828.1	ENSG00000250585.2
5710	ENSG00000125508.3	ENSG00000277826.1	ENSG00000213729.3
5711	ENSG00000125510.16	ENSG00000277818.1	ENSG00000234050.1
5712	ENSG00000125514.9	ENSG00000277817.1	ENSG00000267261.5
5713	ENSG00000125520.14	ENSG00000277809.1	ENSG00000238199.1
5714	ENSG00000125522.3	ENSG00000277806.1	ENSG00000219642.2
5715	ENSG00000125531.7	ENSG00000277803.1	ENSG00000125872.7
5716	ENSG00000125533.5	ENSG00000277801.1	ENSG00000154975.14
5717	ENSG00000125534.10	ENSG00000277797.1	ENSG00000284605.1
5718	ENSG00000125538.11	ENSG00000277795.1	ENSG00000134297.6
5719	ENSG00000125551.18	ENSG00000277794.1	ENSG00000285919.1
5720	ENSG00000125571.9	ENSG00000277791.5	ENSG00000243664.1
5721	ENSG00000125611.15	ENSG00000277790.1	ENSG00000196119.7

5722	ENSG00000125618.17	ENSG00000277785.1	ENSG00000257453.1
5723	ENSG00000125629.15	ENSG00000277784.1	ENSG00000258660.1
5724	ENSG00000125630.15	ENSG00000277782.1	ENSG00000239921.3
5725	ENSG00000125631.7	ENSG00000277778.2	ENSG00000088386.17
5726	ENSG00000125633.11	ENSG00000277777.1	ENSG00000196564.4
5727	ENSG00000125637.15	ENSG00000277775.1	ENSG00000255081.1
5728	ENSG00000125648.15	ENSG00000277774.1	ENSG00000263846.1
5729	ENSG00000125650.4	ENSG00000277771.1	ENSG00000227370.1
5730	ENSG00000125651.14	ENSG00000277770.1	ENSG00000285926.1
5731	ENSG00000125652.8	ENSG00000277769.1	ENSG00000224144.1
5732	ENSG00000125656.10	ENSG00000277766.1	ENSG00000200204.1
5733	ENSG00000125657.5	ENSG00000277764.1	ENSG00000215498.9
5734	ENSG00000125675.18	ENSG00000277763.1	ENSG00000268955.2
5735	ENSG00000125676.20	ENSG00000277762.1	ENSG00000235643.1
5736	ENSG00000125686.12	ENSG00000277761.1	ENSG00000267920.1
5737	ENSG00000125691.13	ENSG00000277759.1	ENSG00000179978.11
5738	ENSG00000125695.12	ENSG00000277758.4	ENSG00000278147.1
5739	ENSG00000125703.15	ENSG00000277757.4	ENSG00000221819.6
5740	ENSG00000125726.11	ENSG00000277755.1	ENSG00000254854.1
5741	ENSG00000125730.16	ENSG00000277754.1	ENSG00000239040.1
5742	ENSG00000125731.13	ENSG00000277752.1	ENSG00000283646.1
5743	ENSG00000125733.18	ENSG00000277749.1	ENSG00000254638.1
5744	ENSG00000125734.15	ENSG00000277748.1	ENSG00000138594.14
5745	ENSG00000125735.10	ENSG00000277747.1	ENSG00000226348.1
5746	ENSG00000125740.14	ENSG00000277746.1	ENSG00000087245.13
5747	ENSG00000125741.4	ENSG00000277745.1	ENSG00000223492.1
5748	ENSG00000125743.10	ENSG00000277744.1	ENSG00000272657.1
5749	ENSG00000125744.12	ENSG00000277743.1	ENSG00000267024.1
5750	ENSG00000125746.16	ENSG00000277741.4	ENSG00000117834.12
5751	ENSG00000125753.14	ENSG00000277740.1	ENSG00000273325.1
5752	ENSG00000125755.18	ENSG00000277739.1	ENSG00000265817.3
5753	ENSG00000125772.12	ENSG00000277738.1	ENSG00000125510.16
5754	ENSG00000125775.15	ENSG00000277737.3	ENSG00000261127.2
5755	ENSG00000125779.22	ENSG00000277734.8	ENSG00000233874.2
5756	ENSG00000125780.12	ENSG00000277732.1	ENSG00000259368.1
5757	ENSG00000125787.11	ENSG00000277728.1	ENSG00000207034.1
5758	ENSG00000125788.6	ENSG00000277726.4	ENSG00000226876.3
5759	ENSG00000125798.14	ENSG00000277723.1	ENSG00000152082.14
5760	ENSG00000125804.13	ENSG00000277721.1	ENSG00000279951.1
5761	ENSG00000125810.10	ENSG00000277720.1	ENSG00000183432.6
5762	ENSG00000125812.16	ENSG00000277718.1	ENSG00000235597.1
5763	ENSG00000125813.13	ENSG00000277717.1	ENSG00000259631.1
5764	ENSG00000125814.17	ENSG00000277713.1	ENSG00000139514.13
5765	ENSG00000125815.8	ENSG00000277711.1	ENSG00000174498.14
5766	ENSG00000125816.5	ENSG00000277710.2	ENSG00000251073.1
5767	ENSG00000125817.8	ENSG00000277708.1	ENSG00000282625.1
5768	ENSG00000125818.18	ENSG00000277705.1	ENSG00000260579.1
5769	ENSG00000125820.5	ENSG00000277704.1	ENSG00000230080.2
5770	ENSG00000125821.12	ENSG00000277702.1	ENSG00000118322.14
5771	ENSG00000125823.12	ENSG00000277701.4	ENSG00000057468.7
5772	ENSG00000125826.20	ENSG00000277698.1	ENSG00000177462.7
5773	ENSG00000125827.9	ENSG00000277697.1	ENSG00000273998.1
5774	ENSG00000125831.10	ENSG00000277695.1	ENSG00000206680.1

5775	ENSG00000125834.12	ENSG00000277694.1	ENSG00000005421.8
5776	ENSG00000125835.18	ENSG00000277693.1	ENSG00000241997.3
5777	ENSG00000125841.13	ENSG00000277692.1	ENSG00000154096.13
5778	ENSG00000125843.11	ENSG00000277690.2	ENSG00000122121.11
5779	ENSG00000125844.15	ENSG00000277689.1	ENSG00000241180.1
5780	ENSG00000125845.7	ENSG00000277688.1	ENSG00000212498.1
5781	ENSG00000125846.15	ENSG00000277687.1	ENSG00000236529.1
5782	ENSG00000125848.10	ENSG00000277684.1	ENSG00000231566.1
5783	ENSG00000125850.11	ENSG00000277681.1	ENSG00000249639.1
5784	ENSG00000125851.10	ENSG00000277677.1	ENSG00000199347.1
5785	ENSG00000125861.14	ENSG00000277675.1	ENSG00000281491.1
5786	ENSG00000125863.20	ENSG00000277673.1	ENSG00000257360.1
5787	ENSG00000125864.14	ENSG00000277672.1	ENSG00000250197.1
5788	ENSG00000125868.16	ENSG00000277671.1	ENSG00000260386.6
5789	ENSG00000125869.10	ENSG00000277670.1	ENSG00000244573.3
5790	ENSG00000125870.11	ENSG00000277669.1	ENSG00000278967.1
5791	ENSG00000125871.14	ENSG00000277668.1	ENSG00000154319.16
5792	ENSG00000125872.7	ENSG00000277666.1	ENSG00000259728.5
5793	ENSG00000125875.14	ENSG00000277662.1	ENSG00000259838.1
5794	ENSG00000125877.13	ENSG00000277661.4	ENSG00000264769.1
5795	ENSG00000125878.6	ENSG00000277655.1	ENSG00000239791.1
5796	ENSG00000125879.4	ENSG00000277654.6	ENSG00000233325.3
5797	ENSG00000125885.13	ENSG00000277653.1	ENSG00000251108.1
5798	ENSG00000125888.14	ENSG00000277651.1	ENSG00000277597.1
5799	ENSG00000125895.5	ENSG00000277647.1	ENSG00000231378.1
5800	ENSG00000125898.13	ENSG00000277646.1	ENSG00000249203.1
5801	ENSG00000125899.7	ENSG00000277640.1	ENSG00000197888.2
5802	ENSG00000125900.13	ENSG00000277639.2	ENSG00000214110.3
5803	ENSG00000125901.6	ENSG00000277637.1	ENSG00000240787.1
5804	ENSG00000125903.4	ENSG00000277636.1	ENSG00000188523.8
5805	ENSG00000125910.5	ENSG00000277635.1	ENSG00000271398.1
5806	ENSG00000125912.11	ENSG00000277634.1	ENSG00000272004.1
5807	ENSG00000125931.11	ENSG00000277632.2	ENSG00000233956.1
5808	ENSG00000125944.20	ENSG00000277631.4	ENSG00000261401.1
5809	ENSG00000125945.15	ENSG00000277630.4	ENSG00000155087.4
5810	ENSG00000125952.19	ENSG00000277628.1	ENSG00000243781.1
5811	ENSG00000125954.12	ENSG00000277621.1	ENSG00000262966.2
5812	ENSG00000125962.14	ENSG00000277619.1	ENSG00000267563.1
5813	ENSG00000125965.9	ENSG00000277618.1	ENSG00000268088.1
5814	ENSG00000125966.10	ENSG00000277617.1	ENSG00000225056.1
5815	ENSG00000125967.16	ENSG00000277613.1	ENSG00000167617.3
5816	ENSG00000125968.9	ENSG00000277611.1	ENSG00000218772.2
5817	ENSG00000125970.12	ENSG00000277610.1	ENSG00000156858.11
5818	ENSG00000125971.16	ENSG00000277608.1	ENSG00000285645.1
5819	ENSG00000125975.13	ENSG00000277605.1	ENSG00000254333.1
5820	ENSG00000125977.7	ENSG00000277604.1	ENSG00000254180.1
5821	ENSG00000125991.19	ENSG00000277602.1	ENSG00000207217.1
5822	ENSG00000125995.16	ENSG00000277599.1	ENSG00000279221.1
5823	ENSG00000125997.5	ENSG00000277598.1	ENSG00000100731.15
5824	ENSG00000125998.8	ENSG00000277597.1	ENSG00000124564.17
5825	ENSG00000125999.11	ENSG00000277595.1	ENSG00000253140.1
5826	ENSG00000126001.16	ENSG00000277592.1	ENSG00000220586.2
5827	ENSG00000126003.7	ENSG00000277591.1	ENSG00000224682.1

5828	ENSG00000126005.17	ENSG00000277590.1	ENSG00000231421.7
5829	ENSG00000126010.5	ENSG00000277589.1	ENSG00000234405.1
5830	ENSG00000126012.11	ENSG00000277588.1	ENSG00000144130.11
5831	ENSG00000126016.15	ENSG00000277587.1	ENSG00000274162.1
5832	ENSG00000126062.4	ENSG00000277586.2	ENSG00000235209.1
5833	ENSG00000126067.12	ENSG00000277583.1	ENSG00000224255.2
5834	ENSG00000126070.20	ENSG00000277582.1	ENSG00000280023.1
5835	ENSG00000126088.14	ENSG00000277581.1	ENSG00000198846.6
5836	ENSG00000126091.20	ENSG00000277579.1	ENSG00000135336.14
5837	ENSG00000126106.14	ENSG00000277578.1	ENSG00000233295.3
5838	ENSG00000126107.15	ENSG00000277577.1	ENSG00000253274.1
5839	ENSG00000126214.21	ENSG00000277576.1	ENSG00000269895.1
5840	ENSG00000126215.14	ENSG00000277575.1	ENSG00000256708.1
5841	ENSG00000126216.15	ENSG00000277572.1	ENSG00000053770.12
5842	ENSG00000126217.21	ENSG00000277569.1	ENSG00000226599.1
5843	ENSG00000126218.11	ENSG00000277568.1	ENSG00000205898.3
5844	ENSG00000126226.21	ENSG00000277566.1	ENSG00000180785.10
5845	ENSG00000126231.14	ENSG00000277565.1	ENSG00000240031.1
5846	ENSG00000126233.2	ENSG00000277563.1	ENSG00000188612.12
5847	ENSG00000126243.8	ENSG00000277562.1	ENSG00000183034.12
5848	ENSG00000126246.10	ENSG00000277561.5	ENSG00000279949.1
5849	ENSG00000126247.10	ENSG00000277559.1	ENSG00000239961.3
5850	ENSG00000126249.8	ENSG00000277558.1	ENSG00000261115.6
5851	ENSG00000126251.6	ENSG00000277556.1	ENSG00000196071.5
5852	ENSG00000126254.12	ENSG00000277553.1	ENSG00000189326.4
5853	ENSG00000126259.19	ENSG00000277550.1	ENSG00000145287.10
5854	ENSG00000126261.13	ENSG00000277549.1	ENSG00000205318.5
5855	ENSG00000126262.4	ENSG00000277548.1	ENSG00000259563.1
5856	ENSG00000126264.9	ENSG00000277545.1	ENSG00000259993.1
5857	ENSG00000126267.10	ENSG00000277544.1	ENSG00000263508.6
5858	ENSG00000126337.13	ENSG00000277543.1	ENSG00000229820.2
5859	ENSG00000126351.12	ENSG00000277542.1	ENSG00000285960.1
5860	ENSG00000126353.3	ENSG00000277541.1	ENSG00000258851.1
5861	ENSG00000126368.6	ENSG00000277535.2	ENSG00000235313.1
5862	ENSG00000126391.14	ENSG00000277534.1	ENSG00000136770.11
5863	ENSG00000126432.14	ENSG00000277533.1	ENSG00000251517.1
5864	ENSG00000126453.9	ENSG00000277532.1	ENSG00000172005.11
5865	ENSG00000126456.16	ENSG00000277531.2	ENSG00000258749.1
5866	ENSG00000126457.21	ENSG00000277529.1	ENSG00000286064.1
5867	ENSG00000126458.4	ENSG00000277527.1	ENSG00000254343.2
5868	ENSG00000126460.11	ENSG00000277526.4	ENSG00000257452.1
5869	ENSG00000126461.15	ENSG00000277524.1	ENSG00000253552.7
5870	ENSG00000126464.14	ENSG00000277521.1	ENSG00000261469.1
5871	ENSG00000126467.11	ENSG00000277519.1	ENSG00000241187.1
5872	ENSG00000126500.3	ENSG00000277516.1	ENSG00000254352.1
5873	ENSG00000126522.16	ENSG00000277515.1	ENSG00000273870.1
5874	ENSG00000126524.10	ENSG00000277514.1	ENSG00000157021.9
5875	ENSG00000126545.14	ENSG00000277513.1	ENSG00000270168.2
5876	ENSG00000126549.9	ENSG00000277512.1	ENSG00000268053.1
5877	ENSG00000126550.8	ENSG00000277511.1	ENSG00000072041.17
5878	ENSG00000126561.16	ENSG00000277510.1	ENSG00000174516.15
5879	ENSG00000126562.17	ENSG00000277509.1	ENSG00000163808.17
5880	ENSG00000126581.13	ENSG00000277508.1	ENSG00000257859.1

5881	ENSG00000126583.11	ENSG00000277506.1	ENSG00000112796.10
5882	ENSG00000126602.11	ENSG00000277505.1	ENSG00000272502.1
5883	ENSG00000126603.8	ENSG00000277504.1	ENSG00000224232.1
5884	ENSG00000126653.18	ENSG00000277502.1	ENSG00000243811.10
5885	ENSG00000126698.11	ENSG00000277501.1	ENSG00000212933.1
5886	ENSG00000126705.14	ENSG00000277500.1	ENSG00000268279.4
5887	ENSG00000126709.15	ENSG00000277499.1	ENSG00000224855.5
5888	ENSG00000126733.21	ENSG00000277498.1	ENSG00000278299.5
5889	ENSG00000126746.17	ENSG00000277496.1	ENSG00000197106.7
5890	ENSG00000126749.16	ENSG00000277494.2	ENSG00000253304.2
5891	ENSG00000126752.7	ENSG00000277493.1	ENSG00000172061.8
5892	ENSG00000126756.12	ENSG00000277492.1	ENSG00000282860.1
5893	ENSG00000126759.13	ENSG00000277491.1	ENSG00000141934.10
5894	ENSG00000126767.18	ENSG00000277490.1	ENSG00000279393.1
5895	ENSG00000126768.12	ENSG00000277488.1	ENSG00000265008.1
5896	ENSG00000126773.13	ENSG00000277486.1	ENSG00000253175.1
5897	ENSG00000126775.8	ENSG00000277483.1	ENSG00000240063.1
5898	ENSG00000126777.18	ENSG00000277482.1	ENSG00000177673.3
5899	ENSG00000126778.10	ENSG00000277481.1	ENSG00000148965.10
5900	ENSG00000126785.13	ENSG00000277479.1	ENSG00000104059.4
5901	ENSG00000126787.13	ENSG00000277478.1	ENSG00000185883.12
5902	ENSG00000126790.12	ENSG00000277476.1	ENSG00000083290.20
5903	ENSG00000126803.9	ENSG00000277475.1	ENSG00000267112.1
5904	ENSG00000126804.13	ENSG00000277474.1	ENSG00000269385.1
5905	ENSG00000126814.6	ENSG00000277473.1	ENSG00000230079.1
5906	ENSG00000126821.8	ENSG00000277469.1	ENSG00000259774.1
5907	ENSG00000126822.17	ENSG00000277468.1	ENSG00000254856.1
5908	ENSG00000126838.10	ENSG00000277467.1	ENSG00000232360.1
5909	ENSG00000126856.14	ENSG00000277466.1	ENSG00000233467.2
5910	ENSG00000126858.18	ENSG00000277465.1	ENSG00000285589.1
5911	ENSG00000126860.11	ENSG00000277464.1	ENSG00000241461.3
5912	ENSG00000126861.5	ENSG00000277463.1	ENSG00000144481.17
5913	ENSG00000126870.16	ENSG00000277462.2	ENSG00000254197.1
5914	ENSG00000126878.13	ENSG00000277460.1	ENSG00000106006.6
5915	ENSG00000126882.13	ENSG00000277459.1	ENSG00000227462.1
5916	ENSG00000126883.17	ENSG00000277458.1	ENSG00000285287.1
5917	ENSG00000126890.13	ENSG00000277455.1	ENSG00000261341.6
5918	ENSG00000126895.14	ENSG00000277453.1	ENSG00000224107.5
5919	ENSG00000126903.16	ENSG00000277452.1	ENSG00000264346.1
5920	ENSG00000126934.13	ENSG00000277450.1	ENSG00000237548.1
5921	ENSG00000126945.9	ENSG00000277449.1	ENSG00000240395.1
5922	ENSG00000126947.12	ENSG00000277448.1	ENSG00000226355.1
5923	ENSG00000126950.8	ENSG00000277447.1	ENSG00000282977.1
5924	ENSG00000126952.16	ENSG00000277446.1	ENSG00000237058.1
5925	ENSG00000126953.7	ENSG00000277444.1	ENSG00000272197.2
5926	ENSG00000126970.16	ENSG00000277443.3	ENSG00000273906.1
5927	ENSG00000127022.15	ENSG00000277440.1	ENSG00000175202.4
5928	ENSG00000127054.20	ENSG00000277438.1	ENSG00000285662.1
5929	ENSG00000127074.14	ENSG00000277437.1	ENSG00000227666.1
5930	ENSG00000127080.10	ENSG00000277436.1	ENSG00000240606.3
5931	ENSG00000127081.14	ENSG00000277435.1	ENSG00000102974.16
5932	ENSG00000127083.7	ENSG00000277428.1	ENSG00000116574.6
5933	ENSG00000127084.19	ENSG00000277426.1	ENSG00000252904.1

5934	ENSG00000127124.16	ENSG00000277425.1	ENSG00000280310.1
5935	ENSG00000127125.9	ENSG00000277423.1	ENSG00000259303.5
5936	ENSG00000127129.10	ENSG00000277422.1	ENSG00000182957.16
5937	ENSG00000127152.18	ENSG00000277418.1	ENSG00000242814.2
5938	ENSG00000127184.13	ENSG00000277413.1	ENSG00000165953.9
5939	ENSG00000127191.18	ENSG00000277412.1	ENSG00000278637.1
5940	ENSG00000127220.6	ENSG00000277411.1	ENSG00000271314.1
5941	ENSG00000127241.16	ENSG00000277410.1	ENSG00000185736.16
5942	ENSG00000127249.15	ENSG00000277408.1	ENSG00000261082.1
5943	ENSG00000127252.6	ENSG00000277406.2	ENSG00000087494.15
5944	ENSG00000127311.9	ENSG00000277400.1	ENSG00000258488.2
5945	ENSG00000127314.18	ENSG00000277399.4	ENSG00000241932.1
5946	ENSG00000127318.11	ENSG00000277397.1	ENSG00000230684.1
5947	ENSG00000127324.9	ENSG00000277396.1	ENSG00000231130.1
5948	ENSG00000127325.19	ENSG00000277391.1	ENSG00000272958.1
5949	ENSG00000127328.21	ENSG00000277388.1	ENSG00000204540.10
5950	ENSG00000127329.15	ENSG00000277387.1	ENSG00000187172.15
5951	ENSG00000127334.10	ENSG00000277386.1	ENSG00000136167.14
5952	ENSG00000127337.7	ENSG00000277385.1	ENSG00000176695.8
5953	ENSG00000127362.2	ENSG00000277383.1	ENSG00000229327.1
5954	ENSG00000127364.3	ENSG00000277382.1	ENSG00000253475.1
5955	ENSG00000127366.5	ENSG00000277379.1	ENSG00000181449.4
5956	ENSG00000127377.9	ENSG00000277374.1	ENSG00000232937.1
5957	ENSG00000127399.15	ENSG00000277373.1	ENSG00000261051.1
5958	ENSG00000127412.6	ENSG00000277371.1	ENSG00000278863.1
5959	ENSG00000127415.13	ENSG00000277370.1	ENSG00000141338.14
5960	ENSG00000127418.15	ENSG00000277369.1	ENSG00000227045.1
5961	ENSG00000127419.17	ENSG00000277368.1	ENSG00000260066.1
5962	ENSG00000127423.10	ENSG00000277367.1	ENSG00000174482.10
5963	ENSG00000127445.13	ENSG00000277363.5	ENSG00000169599.12
5964	ENSG00000127452.8	ENSG00000277358.1	ENSG00000183186.8
5965	ENSG00000127463.15	ENSG00000277357.1	ENSG00000040341.18
5966	ENSG00000127472.11	ENSG00000277352.1	ENSG00000242290.2
5967	ENSG00000127481.15	ENSG00000277351.1	ENSG00000236398.2
5968	ENSG00000127483.18	ENSG00000277350.1	ENSG00000216990.2
5969	ENSG00000127507.18	ENSG00000277349.1	ENSG00000259967.1
5970	ENSG00000127511.9	ENSG00000277344.1	ENSG00000274091.1
5971	ENSG00000127515.2	ENSG00000277342.1	ENSG00000280028.1
5972	ENSG00000127526.15	ENSG00000277341.1	ENSG00000279683.1
5973	ENSG00000127527.14	ENSG00000277340.1	ENSG00000227375.5
5974	ENSG00000127528.5	ENSG00000277332.1	ENSG00000205108.5
5975	ENSG00000127529.7	ENSG00000277327.1	ENSG00000279928.2
5976	ENSG00000127530.3	ENSG00000277322.1	ENSG00000225595.2
5977	ENSG00000127533.3	ENSG00000277320.1	ENSG00000228055.2
5978	ENSG00000127540.12	ENSG00000277319.1	ENSG00000232139.5
5979	ENSG00000127554.13	ENSG00000277315.1	ENSG00000260171.1
5980	ENSG00000127561.15	ENSG00000277313.1	ENSG00000113302.4
5981	ENSG00000127564.17	ENSG00000277311.1	ENSG00000279442.1
5982	ENSG00000127578.7	ENSG00000277308.1	ENSG00000166211.7
5983	ENSG00000127580.17	ENSG00000277306.1	ENSG00000261513.1
5984	ENSG00000127585.12	ENSG00000277304.1	ENSG00000155097.12
5985	ENSG00000127586.17	ENSG00000277301.1	ENSG00000250342.1
5986	ENSG00000127588.5	ENSG00000277299.1	ENSG00000227394.1

5987	ENSG00000127589.4	ENSG00000277297.1	ENSG00000267232.1
5988	ENSG00000127603.25	ENSG00000277295.1	ENSG00000226557.1
5989	ENSG00000127616.18	ENSG00000277289.1	ENSG00000199568.1
5990	ENSG00000127663.15	ENSG00000277288.4	ENSG00000236468.1
5991	ENSG00000127666.9	ENSG00000277283.1	ENSG00000270993.1
5992	ENSG00000127720.8	ENSG00000277282.1	ENSG00000174837.15
5993	ENSG00000127743.6	ENSG00000277277.3	ENSG00000266467.2
5994	ENSG00000127774.7	ENSG00000277274.1	ENSG00000272514.6
5995	ENSG00000127780.3	ENSG00000277270.1	ENSG00000257105.1
5996	ENSG00000127804.13	ENSG00000277269.1	ENSG00000235734.4
5997	ENSG00000127824.14	ENSG00000277268.1	ENSG00000125827.9
5998	ENSG00000127831.10	ENSG00000277265.1	ENSG00000153113.23
5999	ENSG00000127837.9	ENSG00000277264.1	ENSG00000273492.5
6000	ENSG00000127838.14	ENSG00000277260.1	ENSG00000227110.6
6001	ENSG00000127863.15	ENSG00000277258.5	ENSG00000253389.2
6002	ENSG00000127870.17	ENSG00000277255.1	ENSG00000229236.2
6003	ENSG00000127884.5	ENSG00000277253.1	ENSG00000186136.1
6004	ENSG00000127903.14	ENSG00000277252.1	ENSG00000251616.1
6005	ENSG00000127914.16	ENSG00000277250.1	ENSG00000228401.4
6006	ENSG00000127920.5	ENSG00000277249.1	ENSG00000228187.1
6007	ENSG00000127922.9	ENSG00000277248.1	ENSG00000162909.18
6008	ENSG00000127928.12	ENSG00000277247.1	ENSG00000236508.1
6009	ENSG00000127946.17	ENSG00000277246.1	ENSG00000234177.5
6010	ENSG00000127947.16	ENSG00000277245.1	ENSG00000284956.1
6011	ENSG00000127948.16	ENSG00000277243.1	ENSG00000232977.6
6012	ENSG00000127951.7	ENSG00000277241.1	ENSG00000250312.8
6013	ENSG00000127952.17	ENSG00000277235.1	ENSG00000258273.1
6014	ENSG00000127954.12	ENSG00000277234.1	ENSG00000187013.4
6015	ENSG00000127955.17	ENSG00000277233.1	ENSG00000251435.1
6016	ENSG00000127957.18	ENSG00000277232.2	ENSG00000139178.11
6017	ENSG00000127980.16	ENSG00000277229.1	ENSG00000124334.17
6018	ENSG00000127989.13	ENSG00000277228.1	ENSG00000207186.1
6019	ENSG00000127990.17	ENSG00000277227.1	ENSG00000165097.15
6020	ENSG00000127993.16	ENSG00000277223.1	ENSG00000244346.1
6021	ENSG00000127995.17	ENSG00000277218.1	ENSG00000251429.1
6022	ENSG00000128000.16	ENSG00000277217.1	ENSG00000240135.1
6023	ENSG00000128011.4	ENSG00000277215.1	ENSG00000265303.1
6024	ENSG00000128016.6	ENSG00000277214.1	ENSG00000239250.3
6025	ENSG00000128039.11	ENSG00000277213.1	ENSG00000167910.4
6026	ENSG00000128040.11	ENSG00000277211.1	ENSG00000267072.2
6027	ENSG00000128045.7	ENSG00000277210.3	ENSG00000252233.1
6028	ENSG00000128050.8	ENSG00000277209.1	ENSG00000250332.1
6029	ENSG00000128052.9	ENSG00000277206.1	ENSG00000225284.1
6030	ENSG00000128059.8	ENSG00000277203.1	ENSG00000254978.2
6031	ENSG00000128159.12	ENSG00000277202.1	ENSG00000154654.15
6032	ENSG00000128165.9	ENSG00000277199.1	ENSG00000139351.15
6033	ENSG00000128185.9	ENSG00000277198.1	ENSG00000238041.3
6034	ENSG00000128191.16	ENSG00000277196.4	ENSG00000165948.11
6035	ENSG00000128203.7	ENSG00000277194.1	ENSG00000224427.1
6036	ENSG00000128218.8	ENSG00000277191.1	ENSG00000158092.7
6037	ENSG00000128228.5	ENSG00000277188.1	ENSG00000285412.1
6038	ENSG00000128242.13	ENSG00000277186.1	ENSG00000137699.17
6039	ENSG00000128245.15	ENSG00000277184.1	ENSG00000253183.1

6040	ENSG00000128250.5	ENSG00000277182.1	ENSG00000106178.6
6041	ENSG00000128253.14	ENSG00000277174.1	ENSG00000151465.14
6042	ENSG00000128254.13	ENSG00000277173.1	ENSG00000236913.1
6043	ENSG00000128262.8	ENSG00000277172.1	ENSG00000238000.1
6044	ENSG00000128266.9	ENSG00000277171.1	ENSG00000283948.1
6045	ENSG00000128268.12	ENSG00000277170.1	ENSG00000275493.2
6046	ENSG00000128271.22	ENSG00000277168.1	ENSG00000280278.1
6047	ENSG00000128272.14	ENSG00000277162.1	ENSG00000254589.1
6048	ENSG00000128274.17	ENSG00000277161.2	ENSG00000162062.15
6049	ENSG00000128276.10	ENSG00000277159.1	ENSG00000218749.1
6050	ENSG00000128283.7	ENSG00000277157.1	ENSG00000280042.1
6051	ENSG00000128284.19	ENSG00000277156.1	ENSG00000257355.1
6052	ENSG00000128285.4	ENSG00000277152.1	ENSG00000253873.6
6053	ENSG00000128294.16	ENSG00000277151.1	ENSG00000227868.6
6054	ENSG00000128298.17	ENSG00000277150.1	ENSG00000236816.2
6055	ENSG00000128309.16	ENSG00000277149.5	ENSG00000235820.1
6056	ENSG00000128310.2	ENSG00000277146.1	ENSG00000263986.1
6057	ENSG00000128311.14	ENSG00000277144.1	ENSG00000187664.9
6058	ENSG00000128313.2	ENSG00000277143.1	ENSG00000239455.1
6059	ENSG00000128322.7	ENSG00000277142.1	ENSG00000185640.5
6060	ENSG00000128335.14	ENSG00000277141.1	ENSG00000175772.10
6061	ENSG00000128340.15	ENSG00000277138.1	ENSG00000165202.3
6062	ENSG00000128342.5	ENSG00000277136.1	ENSG00000237882.1
6063	ENSG00000128346.10	ENSG00000277135.1	ENSG00000276925.1
6064	ENSG00000128383.13	ENSG00000277133.1	ENSG00000239335.4
6065	ENSG00000128394.17	ENSG00000277130.1	ENSG00000255680.1
6066	ENSG00000128408.8	ENSG00000277129.1	ENSG00000229173.1
6067	ENSG00000128422.16	ENSG00000277128.2	ENSG00000231416.1
6068	ENSG00000128438.10	ENSG00000277127.1	ENSG00000270231.4
6069	ENSG00000128463.13	ENSG00000277125.1	ENSG00000236942.1
6070	ENSG00000128482.16	ENSG00000277124.1	ENSG00000236204.6
6071	ENSG00000128487.16	ENSG00000277120.5_PAR_Y	ENSG00000229267.2
6072	ENSG00000128510.12	ENSG00000277120.5	ENSG00000249035.6
6073	ENSG00000128512.21	ENSG00000277119.1	ENSG00000130226.17
6074	ENSG00000128513.15	ENSG00000277118.1	ENSG00000273999.1
6075	ENSG00000128519.3	ENSG00000277117.4	ENSG00000224718.1
6076	ENSG00000128524.5	ENSG00000277112.3	ENSG00000237265.6
6077	ENSG00000128534.8	ENSG00000277109.1	ENSG00000271657.1
6078	ENSG00000128536.16	ENSG00000277108.1	ENSG00000189410.12
6079	ENSG00000128563.13	ENSG00000277103.1	ENSG00000163378.14
6080	ENSG00000128564.7	ENSG00000277099.1	ENSG00000239224.3
6081	ENSG00000128567.17	ENSG00000277096.1	ENSG00000285230.1
6082	ENSG00000128573.25	ENSG00000277095.1	ENSG00000279747.1
6083	ENSG00000128578.10	ENSG00000277089.4	ENSG00000181781.9
6084	ENSG00000128581.15	ENSG00000277087.1	ENSG00000259617.1
6085	ENSG00000128585.18	ENSG00000277084.1	ENSG00000243904.1
6086	ENSG00000128590.5	ENSG00000277083.1	ENSG00000234928.1
6087	ENSG00000128591.15	ENSG00000277081.1	ENSG00000235347.1
6088	ENSG00000128594.8	ENSG00000277077.1	ENSG00000215186.6
6089	ENSG00000128595.17	ENSG00000277075.2	ENSG00000231476.1
6090	ENSG00000128596.17	ENSG00000277073.1	ENSG00000126785.13
6091	ENSG00000128602.10	ENSG00000277072.5	ENSG00000189057.11
6092	ENSG00000128604.20	ENSG00000277067.4	ENSG00000251396.6

6093	ENSG00000128606.13	ENSG00000277066.1	ENSG00000251158.1
6094	ENSG00000128607.14	ENSG00000277063.1	ENSG00000267271.1
6095	ENSG00000128609.15	ENSG00000277062.1	ENSG00000261898.2
6096	ENSG00000128610.12	ENSG00000277058.2	ENSG00000237955.1
6097	ENSG00000128617.2	ENSG00000277057.1	ENSG00000279384.1
6098	ENSG00000128626.11	ENSG00000277056.1	ENSG00000244712.1
6099	ENSG00000128641.19	ENSG00000277053.4	ENSG00000224426.1
6100	ENSG00000128645.15	ENSG00000277052.1	ENSG00000181852.17
6101	ENSG00000128652.11	ENSG00000277051.1	ENSG00000250027.1
6102	ENSG00000128654.14	ENSG00000277050.1	ENSG00000254346.2
6103	ENSG00000128655.18	ENSG00000277049.1	ENSG00000286095.1
6104	ENSG00000128656.14	ENSG00000277047.4	ENSG00000105219.10
6105	ENSG00000128683.14	ENSG00000277043.1	ENSG00000165621.8
6106	ENSG00000128692.8	ENSG00000277041.1	ENSG00000244239.1
6107	ENSG00000128694.12	ENSG00000277040.1	ENSG00000274049.4
6108	ENSG00000128699.14	ENSG00000277039.1	ENSG00000280012.2
6109	ENSG00000128708.13	ENSG00000277035.1	ENSG00000153814.13
6110	ENSG00000128709.13	ENSG00000277031.1	ENSG00000254799.1
6111	ENSG00000128710.5	ENSG00000277030.1	ENSG00000256568.1
6112	ENSG00000128713.13	ENSG00000277029.1	ENSG00000286003.1
6113	ENSG00000128714.6	ENSG00000277027.1	ENSG00000178412.4
6114	ENSG00000128731.17	ENSG00000277024.1	ENSG00000259084.6
6115	ENSG00000128739.22	ENSG00000277022.1	ENSG00000255418.5
6116	ENSG00000128789.21	ENSG00000277020.4	ENSG00000283438.1
6117	ENSG00000128791.12	ENSG00000277017.1	ENSG00000112706.12
6118	ENSG00000128805.14	ENSG00000277014.1	ENSG00000006042.12
6119	ENSG00000128815.19	ENSG00000277013.1	ENSG00000233741.1
6120	ENSG00000128829.12	ENSG00000277011.1	ENSG00000178814.17
6121	ENSG00000128833.13	ENSG00000277010.1	ENSG00000256739.1
6122	ENSG00000128849.10	ENSG00000277009.1	ENSG00000213892.12
6123	ENSG00000128872.10	ENSG00000277008.1	ENSG00000239215.1
6124	ENSG00000128881.17	ENSG00000277007.1	ENSG00000261834.2
6125	ENSG00000128886.12	ENSG00000277004.1	ENSG00000260298.2
6126	ENSG00000128891.15	ENSG00000277003.1	ENSG00000262855.1
6127	ENSG00000128908.16	ENSG00000277001.1	ENSG00000177465.4
6128	ENSG00000128915.12	ENSG00000277000.1	ENSG00000284276.1
6129	ENSG00000128917.8	ENSG00000276998.1	ENSG00000264522.6
6130	ENSG00000128918.15	ENSG00000276997.4	ENSG00000262786.1
6131	ENSG00000128923.11	ENSG00000276993.1	ENSG00000279567.1
6132	ENSG00000128928.9	ENSG00000276992.1	ENSG00000240534.1
6133	ENSG00000128944.13	ENSG00000276991.1	ENSG00000241293.1
6134	ENSG00000128951.14	ENSG00000276988.1	ENSG00000229402.1
6135	ENSG00000128965.13	ENSG00000276984.1	ENSG00000186818.12
6136	ENSG00000128973.13	ENSG00000276980.1	ENSG00000255232.1
6137	ENSG00000128989.10	ENSG00000276975.3	ENSG00000225531.1
6138	ENSG00000129003.17	ENSG00000276972.1	ENSG00000228807.1
6139	ENSG00000129007.14	ENSG00000276968.1	ENSG00000229660.1
6140	ENSG00000129028.9	ENSG00000276966.2	ENSG00000237360.2
6141	ENSG00000129038.16	ENSG00000276965.1	ENSG00000250260.1
6142	ENSG00000129048.6	ENSG00000276964.1	ENSG00000256589.2
6143	ENSG00000129055.12	ENSG00000276963.1	ENSG00000273175.1
6144	ENSG00000129071.9	ENSG00000276961.1	ENSG00000260969.1
6145	ENSG00000129083.12	ENSG00000276960.1	ENSG00000245385.2

6146	ENSG00000129084.17	ENSG00000276958.1	ENSG00000169330.8
6147	ENSG00000129103.18	ENSG00000276957.1	ENSG00000237734.1
6148	ENSG00000129116.19	ENSG00000276956.1	ENSG00000239393.1
6149	ENSG00000129128.13	ENSG00000276955.1	ENSG00000257829.1
6150	ENSG00000129151.9	ENSG00000276953.1	ENSG00000236259.1
6151	ENSG00000129152.4	ENSG00000276952.1	ENSG00000278084.1
6152	ENSG00000129158.11	ENSG00000276951.1	ENSG00000258586.1
6153	ENSG00000129159.7	ENSG00000276950.5	ENSG00000196196.3
6154	ENSG00000129167.9	ENSG00000276949.1	ENSG00000237289.9
6155	ENSG00000129170.9	ENSG00000276945.1	ENSG00000272679.1
6156	ENSG00000129173.13	ENSG00000276941.1	ENSG00000065371.17
6157	ENSG00000129187.14	ENSG00000276940.1	ENSG00000235433.1
6158	ENSG00000129194.7	ENSG00000276934.1	ENSG00000150175.13
6159	ENSG00000129195.16	ENSG00000276932.1	ENSG00000213661.3
6160	ENSG00000129197.14	ENSG00000276931.1	ENSG00000266959.1
6161	ENSG00000129204.16	ENSG00000276929.1	ENSG00000234183.1
6162	ENSG00000129214.15	ENSG00000276928.1	ENSG00000231845.3
6163	ENSG00000129219.14	ENSG00000276926.1	ENSG00000216813.1
6164	ENSG00000129221.15	ENSG00000276925.1	ENSG00000183798.5
6165	ENSG00000129226.14	ENSG00000276923.1	ENSG00000200075.1
6166	ENSG00000129235.10	ENSG00000276919.1	ENSG00000186184.17
6167	ENSG00000129244.9	ENSG00000276918.1	ENSG00000275265.1
6168	ENSG00000129245.11	ENSG00000276917.1	ENSG00000264215.1
6169	ENSG00000129250.12	ENSG00000276916.1	ENSG00000215112.6
6170	ENSG00000129255.16	ENSG00000276915.1	ENSG00000168734.14
6171	ENSG00000129292.20	ENSG00000276908.1	ENSG00000202125.1
6172	ENSG00000129295.9	ENSG00000276904.1	ENSG00000247993.2
6173	ENSG00000129315.11	ENSG00000276903.1	ENSG00000186212.3
6174	ENSG00000129317.14	ENSG00000276902.1	ENSG00000279315.1
6175	ENSG00000129347.20	ENSG00000276900.1	ENSG00000286165.1
6176	ENSG00000129351.17	ENSG00000276898.1	ENSG00000252361.1
6177	ENSG00000129353.15	ENSG00000276897.1	ENSG00000259002.2
6178	ENSG00000129355.6	ENSG00000276894.1	ENSG00000213440.2
6179	ENSG00000129422.14	ENSG00000276892.1	ENSG00000111780.8
6180	ENSG00000129437.10	ENSG00000276891.1	ENSG00000102539.5
6181	ENSG00000129450.8	ENSG00000276890.1	ENSG00000230698.1
6182	ENSG00000129451.12	ENSG00000276888.1	ENSG00000212588.1
6183	ENSG00000129455.15	ENSG00000276884.1	ENSG00000263218.2
6184	ENSG00000129460.16	ENSG00000276878.1	ENSG00000139352.4
6185	ENSG00000129465.16	ENSG00000276874.1	ENSG00000169789.10
6186	ENSG00000129467.13	ENSG00000276873.1	ENSG00000282692.1
6187	ENSG00000129472.15	ENSG00000276872.1	ENSG00000236094.1
6188	ENSG00000129473.9	ENSG00000276871.1	ENSG00000218502.3
6189	ENSG00000129474.16	ENSG00000276869.1	ENSG00000163283.7
6190	ENSG00000129480.13	ENSG00000276868.1	ENSG00000228242.6
6191	ENSG00000129484.13	ENSG00000276867.1	ENSG00000235478.4
6192	ENSG00000129493.15	ENSG00000276866.1	ENSG00000109163.6
6193	ENSG00000129514.6	ENSG00000276863.1	ENSG00000133195.11
6194	ENSG00000129515.19	ENSG00000276861.1	ENSG00000115165.10
6195	ENSG00000129518.9	ENSG00000276859.1	ENSG00000228307.2
6196	ENSG00000129521.13	ENSG00000276857.1	ENSG00000283579.1
6197	ENSG00000129534.14	ENSG00000276855.1	ENSG00000237382.2
6198	ENSG00000129535.12	ENSG00000276854.1	ENSG00000182093.15

6199	ENSG00000129538.14	ENSG00000276853.1	ENSG00000229716.2
6200	ENSG00000129559.13	ENSG00000276851.1	ENSG00000159399.9
6201	ENSG00000129562.11	ENSG00000276850.4	ENSG00000158077.4
6202	ENSG00000129566.13	ENSG00000276846.1	ENSG00000266987.1
6203	ENSG00000129595.13	ENSG00000276845.1	ENSG00000269842.5
6204	ENSG00000129596.5	ENSG00000276844.1	ENSG00000130159.14
6205	ENSG00000129625.13	ENSG00000276842.1	ENSG00000279172.1
6206	ENSG00000129636.12	ENSG00000276841.1	ENSG00000108932.12
6207	ENSG00000129646.14	ENSG00000276840.1	ENSG00000235248.3
6208	ENSG00000129654.8	ENSG00000276839.1	ENSG00000231549.1
6209	ENSG00000129657.16	ENSG00000276835.2	ENSG00000174791.11
6210	ENSG00000129667.12	ENSG00000276832.1	ENSG00000207523.1
6211	ENSG00000129673.9	ENSG00000276831.1	ENSG00000182836.10
6212	ENSG00000129675.16	ENSG00000276830.1	ENSG00000260639.1
6213	ENSG00000129680.16	ENSG00000276829.1	ENSG00000277771.1
6214	ENSG00000129691.15	ENSG00000276828.1	ENSG00000226443.3
6215	ENSG00000129696.12	ENSG00000276824.1	ENSG00000232328.2
6216	ENSG00000129744.2	ENSG00000276823.1	ENSG00000231749.3
6217	ENSG00000129749.3	ENSG00000276822.1	ENSG00000266664.1
6218	ENSG00000129757.13	ENSG00000276819.1	ENSG00000251579.1
6219	ENSG00000129810.15	ENSG00000276814.1	ENSG00000248626.1
6220	ENSG00000129816.5	ENSG00000276810.1	ENSG00000260120.1
6221	ENSG00000129824.16	ENSG00000276809.1	ENSG00000125844.15
6222	ENSG00000129845.5	ENSG00000276807.1	ENSG00000236872.1
6223	ENSG00000129862.6	ENSG00000276805.2	ENSG00000224227.3
6224	ENSG00000129864.6	ENSG00000276800.1	ENSG00000215268.3
6225	ENSG00000129873.7	ENSG00000276791.1	ENSG00000285918.1
6226	ENSG00000129910.7	ENSG00000276790.1	ENSG00000067646.12
6227	ENSG00000129911.9	ENSG00000276789.1	ENSG00000134200.3
6228	ENSG00000129925.11	ENSG00000276788.1	ENSG00000205105.6
6229	ENSG00000129932.8	ENSG00000276786.1	ENSG00000229465.1
6230	ENSG00000129933.21	ENSG00000276784.1	ENSG00000286082.1
6231	ENSG00000129946.10	ENSG00000276782.1	ENSG00000165478.7
6232	ENSG00000129951.18	ENSG00000276778.1	ENSG00000115652.14
6233	ENSG00000129965.15	ENSG00000276775.1	ENSG00000278992.1
6234	ENSG00000129968.15	ENSG00000276772.1	ENSG00000285010.1
6235	ENSG00000129988.6	ENSG00000276771.1	ENSG00000280365.1
6236	ENSG00000129990.15	ENSG00000276770.1	ENSG00000232994.1
6237	ENSG00000129991.12	ENSG00000276769.1	ENSG00000255860.3
6238	ENSG00000129993.14	ENSG00000276768.1	ENSG00000284688.1
6239	ENSG00000130005.12	ENSG00000276765.1	ENSG00000255537.1
6240	ENSG00000130021.13	ENSG00000276764.1	ENSG00000229704.1
6241	ENSG00000130023.16	ENSG00000276763.1	ENSG00000261205.2
6242	ENSG00000130024.15	ENSG00000276760.4	ENSG00000257666.1
6243	ENSG00000130032.16	ENSG00000276759.2	ENSG00000278732.1
6244	ENSG00000130035.7	ENSG00000276758.1	ENSG00000243642.3
6245	ENSG00000130037.4	ENSG00000276757.1	ENSG00000105409.18
6246	ENSG00000130038.9	ENSG00000276756.4	ENSG00000179455.8
6247	ENSG00000130045.16	ENSG00000276755.1	ENSG00000286056.1
6248	ENSG00000130052.13	ENSG00000276754.1	ENSG00000186480.13
6249	ENSG00000130054.4	ENSG00000276753.1	ENSG00000256670.1
6250	ENSG00000130055.13	ENSG00000276747.1	ENSG00000166035.11
6251	ENSG00000130066.16	ENSG00000276746.1	ENSG00000090539.15

6252	ENSG00000130119.16	ENSG00000276744.1	ENSG00000279642.1
6253	ENSG00000130147.16	ENSG00000276742.1	ENSG00000230427.1
6254	ENSG00000130150.12	ENSG00000276740.1	ENSG00000249437.7
6255	ENSG00000130158.14	ENSG00000276738.1	ENSG00000249807.1
6256	ENSG00000130159.14	ENSG00000276735.1	ENSG00000279251.1
6257	ENSG00000130164.13	ENSG00000276733.1	ENSG00000258759.1
6258	ENSG00000130165.10	ENSG00000276730.1	ENSG00000237589.1
6259	ENSG00000130167.13	ENSG00000276729.1	ENSG00000215837.7
6260	ENSG00000130173.13	ENSG00000276728.1	ENSG00000261634.3
6261	ENSG00000130175.9	ENSG00000276727.1	ENSG00000196503.4
6262	ENSG00000130176.8	ENSG00000276724.1	ENSG00000214188.9
6263	ENSG00000130177.16	ENSG00000276723.1	ENSG00000266696.1
6264	ENSG00000130182.8	ENSG00000276722.1	ENSG00000242329.1
6265	ENSG00000130193.8	ENSG00000276721.1	ENSG00000185203.12
6266	ENSG00000130202.10	ENSG00000276718.1	ENSG00000244113.1
6267	ENSG00000130203.10	ENSG00000276717.1	ENSG00000231384.1
6268	ENSG00000130204.13	ENSG00000276715.4	ENSG00000243859.3
6269	ENSG00000130208.9	ENSG00000276713.1	ENSG00000258300.2
6270	ENSG00000130222.11	ENSG00000276712.1	ENSG00000127472.11
6271	ENSG00000130224.15	ENSG00000276711.1	ENSG00000277423.1
6272	ENSG00000130226.17	ENSG00000276710.4	ENSG00000171864.5
6273	ENSG00000130227.17	ENSG00000276707.1	ENSG00000259071.1
6274	ENSG00000130234.10	ENSG00000276706.1	ENSG00000235445.1
6275	ENSG00000130244.12	ENSG00000276704.1	ENSG00000228050.1
6276	ENSG00000130254.12	ENSG00000276703.1	ENSG00000227517.6
6277	ENSG00000130255.13	ENSG00000276702.1	ENSG00000112280.16
6278	ENSG00000130270.16	ENSG00000276700.1	ENSG00000125522.3
6279	ENSG00000130283.9	ENSG00000276699.1	ENSG00000263029.1
6280	ENSG00000130287.14	ENSG00000276698.1	ENSG00000225579.1
6281	ENSG00000130294.16	ENSG00000276696.1	ENSG00000138688.15
6282	ENSG00000130299.17	ENSG00000276693.1	ENSG00000261117.1
6283	ENSG00000130300.9	ENSG00000276692.1	ENSG00000128872.10
6284	ENSG00000130303.13	ENSG00000276691.1	ENSG00000264273.1
6285	ENSG00000130304.17	ENSG00000276690.1	ENSG00000286089.1
6286	ENSG00000130305.16	ENSG00000276689.1	ENSG00000132694.18
6287	ENSG00000130307.11	ENSG00000276688.1	ENSG00000146776.14
6288	ENSG00000130309.11	ENSG00000276687.1	ENSG00000282591.1
6289	ENSG00000130311.11	ENSG00000276685.1	ENSG00000285867.1
6290	ENSG00000130312.6	ENSG00000276680.1	ENSG00000275014.1
6291	ENSG00000130313.7	ENSG00000276678.1	ENSG00000277034.1
6292	ENSG00000130332.15	ENSG00000276675.1	ENSG00000230400.2
6293	ENSG00000130338.13	ENSG00000276674.1	ENSG00000236900.1
6294	ENSG00000130340.15	ENSG00000276673.1	ENSG00000256263.1
6295	ENSG00000130347.12	ENSG00000276672.1	ENSG00000230728.1
6296	ENSG00000130348.11	ENSG00000276670.1	ENSG00000124380.11
6297	ENSG00000130349.10	ENSG00000276669.1	ENSG00000270265.1
6298	ENSG00000130363.11	ENSG00000276668.1	ENSG00000268423.4
6299	ENSG00000130368.5	ENSG00000276667.1	ENSG00000226338.1
6300	ENSG00000130377.13	ENSG00000276664.1	ENSG00000250302.2
6301	ENSG00000130382.9	ENSG00000276663.1	ENSG00000234199.2
6302	ENSG00000130383.7	ENSG00000276662.1	ENSG00000282100.1
6303	ENSG00000130385.5	ENSG00000276656.1	ENSG00000149295.14
6304	ENSG00000130396.20	ENSG00000276653.1	ENSG00000224331.2

6305	ENSG00000130402.12	ENSG00000276651.1	ENSG00000259696.2
6306	ENSG00000130413.15	ENSG00000276650.1	ENSG00000273549.1
6307	ENSG00000130414.12	ENSG00000276649.1	ENSG00000237178.2
6308	ENSG00000130427.3	ENSG00000276647.1	ENSG00000254894.1
6309	ENSG00000130429.14	ENSG00000276645.1	ENSG00000235779.7
6310	ENSG00000130433.7	ENSG00000276644.5	ENSG00000253481.2
6311	ENSG00000130449.6	ENSG00000276643.1	ENSG00000261456.5
6312	ENSG00000130475.14	ENSG00000276642.1	ENSG00000242436.3
6313	ENSG00000130477.15	ENSG00000276641.1	ENSG00000270723.1
6314	ENSG00000130479.11	ENSG00000276639.1	ENSG00000267515.1
6315	ENSG00000130487.8	ENSG00000276638.1	ENSG00000284341.1
6316	ENSG00000130489.14	ENSG00000276637.1	ENSG00000231035.1
6317	ENSG00000130508.11	ENSG00000276633.1	ENSG00000242441.8
6318	ENSG00000130511.16	ENSG00000276632.1	ENSG00000279484.1
6319	ENSG00000130513.6	ENSG00000276631.1	ENSG00000250345.2
6320	ENSG00000130517.14	ENSG00000276627.2	ENSG00000135220.11
6321	ENSG00000130518.17	ENSG00000276626.1	ENSG00000241640.2
6322	ENSG00000130520.10	ENSG00000276623.1	ENSG00000279757.1
6323	ENSG00000130522.5	ENSG00000276622.1	ENSG00000228838.1
6324	ENSG00000130528.12	ENSG00000276620.1	ENSG00000244144.1
6325	ENSG00000130529.16	ENSG00000276612.3	ENSG00000240021.10
6326	ENSG00000130538.5	ENSG00000276610.1	ENSG00000285771.1
6327	ENSG00000130540.14	ENSG00000276609.1	ENSG00000227351.2
6328	ENSG00000130544.12	ENSG00000276605.1	ENSG00000222714.1
6329	ENSG00000130545.16	ENSG00000276603.1	ENSG00000230735.1
6330	ENSG00000130558.19	ENSG00000276601.1	ENSG00000226919.3
6331	ENSG00000130559.19	ENSG00000276600.5	ENSG00000236452.1
6332	ENSG00000130560.8	ENSG00000276599.1	ENSG00000242659.1
6333	ENSG00000130561.17	ENSG00000276598.1	ENSG00000252577.1
6334	ENSG00000130584.11	ENSG00000276597.1	ENSG00000255928.1
6335	ENSG00000130589.16	ENSG00000276596.1	ENSG00000249853.7
6336	ENSG00000130590.14	ENSG00000276593.1	ENSG00000241697.5
6337	ENSG00000130592.15	ENSG00000276592.1	ENSG00000100393.13
6338	ENSG00000130595.19	ENSG00000276591.1	ENSG00000131668.14
6339	ENSG00000130598.16	ENSG00000276588.1	ENSG00000212402.1
6340	ENSG00000130600.18	ENSG00000276586.1	ENSG00000272984.1
6341	ENSG00000130612.15	ENSG00000276584.1	ENSG00000226278.1
6342	ENSG00000130635.15	ENSG00000276581.2	ENSG00000235096.2
6343	ENSG00000130638.17	ENSG00000276580.1	ENSG00000280140.1
6344	ENSG00000130640.13	ENSG00000276578.1	ENSG00000260021.1
6345	ENSG00000130643.9	ENSG00000276577.1	ENSG00000275969.2
6346	ENSG00000130649.10	ENSG00000276576.1	ENSG00000204961.6
6347	ENSG00000130653.16	ENSG00000276575.1	ENSG00000236894.3
6348	ENSG00000130656.5	ENSG00000276573.1	ENSG00000240299.3
6349	ENSG00000130669.17	ENSG00000276571.1	ENSG00000107281.10
6350	ENSG00000130675.15	ENSG00000276570.1	ENSG00000253967.1
6351	ENSG00000130684.14	ENSG00000276569.1	ENSG00000221983.7
6352	ENSG00000130695.15	ENSG00000276568.1	ENSG00000246379.7
6353	ENSG00000130699.18	ENSG00000276564.1	ENSG00000250202.2
6354	ENSG00000130700.7	ENSG00000276563.1	ENSG00000236947.5
6355	ENSG00000130701.4	ENSG00000276562.1	ENSG00000223462.2
6356	ENSG00000130702.15	ENSG00000276557.1	ENSG00000256385.1
6357	ENSG00000130703.16	ENSG00000276556.1	ENSG00000153822.13

6358	ENSG00000130706.13	ENSG00000276550.4	ENSG00000273353.1
6359	ENSG00000130707.17	ENSG00000276548.1	ENSG00000285520.1
6360	ENSG00000130711.3	ENSG00000276547.1	ENSG00000228466.1
6361	ENSG00000130713.16	ENSG00000276546.1	ENSG00000258990.2
6362	ENSG00000130714.16	ENSG00000276545.1	ENSG00000224093.5
6363	ENSG00000130717.13	ENSG00000276544.1	ENSG00000255750.5
6364	ENSG00000130720.13	ENSG00000276542.1	ENSG00000242085.1
6365	ENSG00000130723.20	ENSG00000276540.1	ENSG00000278012.1
6366	ENSG00000130724.8	ENSG00000276538.1	ENSG00000095739.11
6367	ENSG00000130725.7	ENSG00000276535.1	ENSG00000223653.5
6368	ENSG00000130726.12	ENSG00000276533.1	ENSG00000234112.2
6369	ENSG00000130731.16	ENSG00000276532.1	ENSG00000277371.1
6370	ENSG00000130733.10	ENSG00000276531.1	ENSG00000237970.1
6371	ENSG00000130734.10	ENSG00000276530.1	ENSG00000130600.18
6372	ENSG00000130741.11	ENSG00000276529.1	ENSG00000109193.12
6373	ENSG00000130748.7	ENSG00000276528.1	ENSG00000238825.1
6374	ENSG00000130749.10	ENSG00000276527.1	ENSG00000283016.1
6375	ENSG00000130751.9	ENSG00000276525.1	ENSG00000282199.1
6376	ENSG00000130755.13	ENSG00000276524.1	ENSG00000255741.1
6377	ENSG00000130758.8	ENSG00000276523.1	ENSG00000276581.2
6378	ENSG00000130762.15	ENSG00000276522.1	ENSG00000162685.6
6379	ENSG00000130764.10	ENSG00000276521.1	ENSG00000260932.1
6380	ENSG00000130766.5	ENSG00000276520.1	ENSG00000161638.11
6381	ENSG00000130768.15	ENSG00000276519.1	ENSG00000164270.17
6382	ENSG00000130770.18	ENSG00000276517.1	ENSG00000256474.1
6383	ENSG00000130772.14	ENSG00000276514.1	ENSG00000257474.5
6384	ENSG00000130775.16	ENSG00000276509.1	ENSG00000186628.12
6385	ENSG00000130779.20	ENSG00000276508.1	ENSG00000123933.17
6386	ENSG00000130783.13	ENSG00000276507.1	ENSG00000255491.2
6387	ENSG00000130787.14	ENSG00000276505.1	ENSG00000249263.2
6388	ENSG00000130803.15	ENSG00000276502.1	ENSG00000100427.15
6389	ENSG00000130810.20	ENSG00000276500.1	ENSG00000222747.1
6390	ENSG00000130811.12	ENSG00000276496.1	ENSG00000255262.3
6391	ENSG00000130812.10	ENSG00000276494.1	ENSG00000257878.1
6392	ENSG00000130813.18	ENSG00000276493.1	ENSG00000240237.1
6393	ENSG00000130816.16	ENSG00000276490.1	ENSG00000227541.1
6394	ENSG00000130818.12	ENSG00000276489.1	ENSG00000230758.1
6395	ENSG00000130821.16	ENSG00000276488.1	ENSG00000123473.15
6396	ENSG00000130822.15	ENSG00000276487.1	ENSG00000166435.15
6397	ENSG00000130826.18	ENSG00000276486.1	ENSG00000177640.15
6398	ENSG00000130827.6	ENSG00000276484.1	ENSG00000228208.6
6399	ENSG00000130829.18	ENSG00000276479.1	ENSG00000239797.1
6400	ENSG00000130830.15	ENSG00000276478.1	ENSG00000251652.1
6401	ENSG00000130844.17	ENSG00000276476.2	ENSG00000256674.1
6402	ENSG00000130856.16	ENSG00000276475.1	ENSG00000273734.1
6403	ENSG00000130876.11	ENSG00000276474.1	ENSG00000162654.9
6404	ENSG00000130881.14	ENSG00000276473.1	ENSG00000174371.17
6405	ENSG00000130921.7	ENSG00000276471.1	ENSG00000127329.15
6406	ENSG00000130935.10	ENSG00000276470.1	ENSG00000278060.1
6407	ENSG00000130939.19	ENSG00000276467.1	ENSG00000235366.2
6408	ENSG00000130940.15	ENSG00000276462.1	ENSG00000207088.1
6409	ENSG00000130943.6	ENSG00000276460.1	ENSG00000214646.8
6410	ENSG00000130948.10	ENSG00000276457.1	ENSG00000176956.12

6411	ENSG00000130956.14	ENSG00000276454.1	ENSG00000256361.1
6412	ENSG00000130957.5	ENSG00000276449.1	ENSG00000237646.1
6413	ENSG00000130958.13	ENSG00000276445.1	ENSG00000117600.12
6414	ENSG00000130962.17	ENSG00000276442.1	ENSG00000285837.1
6415	ENSG00000130985.17	ENSG00000276436.1	ENSG00000228559.1
6416	ENSG00000130988.13	ENSG00000276434.1	ENSG00000198580.7
6417	ENSG00000130997.16	ENSG00000276431.1	ENSG00000250329.1
6418	ENSG00000131002.12	ENSG00000276430.2	ENSG00000196542.8
6419	ENSG00000131007.9	ENSG00000276428.1	ENSG00000230239.1
6420	ENSG00000131013.3	ENSG00000276427.1	ENSG00000268618.1
6421	ENSG00000131015.5	ENSG00000276426.1	ENSG00000101126.17
6422	ENSG00000131016.17	ENSG00000276422.1	ENSG00000131238.17
6423	ENSG00000131018.23	ENSG00000276418.5	ENSG00000227850.1
6424	ENSG00000131019.11	ENSG00000276417.1	ENSG00000149021.7
6425	ENSG00000131023.13	ENSG00000276413.1	ENSG00000259767.1
6426	ENSG00000131037.15	ENSG00000276412.2	ENSG00000151208.17
6427	ENSG00000131042.14	ENSG00000276411.1	ENSG00000253708.1
6428	ENSG00000131043.12	ENSG00000276410.3	ENSG00000267207.1
6429	ENSG00000131044.17	ENSG00000276409.5	ENSG00000231519.1
6430	ENSG00000131050.11	ENSG00000276408.1	ENSG00000232579.1
6431	ENSG00000131051.22	ENSG00000276407.4	ENSG00000142615.8
6432	ENSG00000131055.4	ENSG00000276406.1	ENSG00000276735.1
6433	ENSG00000131059.11	ENSG00000276405.1	ENSG00000149212.11
6434	ENSG00000131061.14	ENSG00000276404.1	ENSG00000101605.12
6435	ENSG00000131067.17	ENSG00000276403.1	ENSG00000278948.1
6436	ENSG00000131068.3	ENSG00000276400.1	ENSG00000286266.1
6437	ENSG00000131069.20	ENSG00000276399.1	ENSG00000228629.2
6438	ENSG00000131080.15	ENSG00000276398.1	ENSG00000228019.1
6439	ENSG00000131089.15	ENSG00000276397.1	ENSG00000232846.1
6440	ENSG00000131094.4	ENSG00000276396.1	ENSG00000239544.1
6441	ENSG00000131095.13	ENSG00000276393.1	ENSG00000258517.1
6442	ENSG00000131096.10	ENSG00000276391.1	ENSG00000250280.2
6443	ENSG00000131097.7	ENSG00000276390.1	ENSG00000275538.1
6444	ENSG00000131100.13	ENSG00000276388.1	ENSG00000284280.1
6445	ENSG00000131115.16	ENSG00000276386.1	ENSG00000226957.1
6446	ENSG00000131116.12	ENSG00000276385.1	ENSG00000271029.1
6447	ENSG00000131126.18	ENSG00000276384.1	ENSG00000261131.1
6448	ENSG00000131127.14	ENSG00000276380.2	ENSG00000258927.1
6449	ENSG00000131142.13	ENSG00000276376.1	ENSG00000250041.2
6450	ENSG00000131143.8	ENSG00000276375.1	ENSG00000268810.1
6451	ENSG00000131148.9	ENSG00000276368.1	ENSG00000228034.1
6452	ENSG00000131149.19	ENSG00000276366.1	ENSG00000244301.6
6453	ENSG00000131152.4	ENSG00000276365.1	ENSG00000012232.8
6454	ENSG00000131153.9	ENSG00000276362.1	ENSG00000204904.7
6455	ENSG00000131165.15	ENSG00000276359.1	ENSG00000230362.1
6456	ENSG00000131171.13	ENSG00000276353.1	ENSG00000270259.2
6457	ENSG00000131174.6	ENSG00000276352.2	ENSG00000262693.1
6458	ENSG00000131183.11	ENSG00000276351.1	ENSG00000274693.1
6459	ENSG00000131187.9	ENSG00000276350.1	ENSG00000196247.11
6460	ENSG00000131188.12	ENSG00000276348.1	ENSG00000113396.13
6461	ENSG00000131196.17	ENSG00000276345.1	ENSG00000196535.16
6462	ENSG00000131203.13	ENSG00000276337.1	ENSG00000267592.1
6463	ENSG00000131233.9	ENSG00000276334.1	ENSG00000241449.5

6464	ENSG00000131236.17	ENSG00000276332.1	ENSG00000128346.10
6465	ENSG00000131238.17	ENSG00000276326.1	ENSG00000006071.13
6466	ENSG00000131242.18	ENSG00000276319.1	ENSG00000264272.1
6467	ENSG00000131263.12	ENSG00000276318.1	ENSG00000256590.2
6468	ENSG00000131264.3	ENSG00000276317.1	ENSG00000238531.1
6469	ENSG00000131269.17	ENSG00000276316.1	ENSG00000286132.1
6470	ENSG00000131323.14	ENSG00000276314.1	ENSG00000232643.1
6471	ENSG00000131351.15	ENSG00000276312.1	ENSG00000253572.2
6472	ENSG00000131355.14	ENSG00000276311.1	ENSG00000260389.1
6473	ENSG00000131368.8	ENSG00000276309.1	ENSG00000261033.1
6474	ENSG00000131370.16	ENSG00000276308.1	ENSG00000236173.1
6475	ENSG00000131373.14	ENSG00000276304.1	ENSG00000184924.5
6476	ENSG00000131374.14	ENSG00000276302.1	ENSG00000244266.1
6477	ENSG00000131375.10	ENSG00000276298.1	ENSG00000254968.6
6478	ENSG00000131378.14	ENSG00000276294.1	ENSG00000279735.2
6479	ENSG00000131379.9	ENSG00000276293.4	ENSG00000128973.13
6480	ENSG00000131381.12	ENSG00000276292.1	ENSG00000215102.2
6481	ENSG00000131386.19	ENSG00000276291.5	ENSG00000106541.12
6482	ENSG00000131389.17	ENSG00000276290.1	ENSG00000286190.1
6483	ENSG00000131398.13	ENSG00000276289.4	ENSG00000241282.1
6484	ENSG00000131400.8	ENSG00000276282.1	ENSG00000235555.1
6485	ENSG00000131401.11	ENSG00000276281.1	ENSG00000251656.1
6486	ENSG00000131408.14	ENSG00000276278.1	ENSG00000201861.1
6487	ENSG00000131409.13	ENSG00000276277.1	ENSG00000034713.8
6488	ENSG00000131435.13	ENSG00000276272.1	ENSG00000159217.10
6489	ENSG00000131437.15	ENSG00000276270.1	ENSG00000279681.1
6490	ENSG00000131446.16	ENSG00000276269.1	ENSG00000227339.1
6491	ENSG00000131459.13	ENSG00000276266.1	ENSG00000258215.1
6492	ENSG00000131462.8	ENSG00000276261.1	ENSG00000224743.6
6493	ENSG00000131467.10	ENSG00000276259.1	ENSG00000277479.1
6494	ENSG00000131469.14	ENSG00000276257.1	ENSG00000253358.1
6495	ENSG00000131470.14	ENSG00000276256.1	ENSG00000266313.1
6496	ENSG00000131471.7	ENSG00000276255.2	ENSG00000213864.3
6497	ENSG00000131473.17	ENSG00000276251.1	ENSG00000226548.1
6498	ENSG00000131475.7	ENSG00000276250.1	ENSG00000210156.1
6499	ENSG00000131477.11	ENSG00000276248.1	ENSG00000130377.13
6500	ENSG00000131480.9	ENSG00000276241.1	ENSG00000240098.3
6501	ENSG00000131482.9	ENSG00000276240.2	ENSG00000142512.15
6502	ENSG00000131484.4	ENSG00000276234.5	ENSG00000236761.5
6503	ENSG00000131495.8	ENSG00000276233.1	ENSG00000233579.1
6504	ENSG00000131503.21	ENSG00000276232.1	ENSG00000284736.1
6505	ENSG00000131504.17	ENSG00000276231.4	ENSG00000229404.5
6506	ENSG00000131507.11	ENSG00000276229.1	ENSG00000256034.1
6507	ENSG00000131508.16	ENSG00000276225.1	ENSG00000145975.14
6508	ENSG00000131538.7	ENSG00000276223.1	ENSG00000266495.1
6509	ENSG00000131548.7	ENSG00000276221.1	ENSG00000228961.1
6510	ENSG00000131558.15	ENSG00000276216.1	ENSG00000253042.1
6511	ENSG00000131584.19	ENSG00000276214.1	ENSG00000154678.17
6512	ENSG00000131591.17	ENSG00000276213.1	ENSG00000279679.1
6513	ENSG00000131620.17	ENSG00000276210.2	ENSG00000211591.1
6514	ENSG00000131626.18	ENSG00000276208.1	ENSG00000213730.3
6515	ENSG00000131634.14	ENSG00000276206.1	ENSG00000255508.7
6516	ENSG00000131650.14	ENSG00000276205.1	ENSG00000113552.16

6517	ENSG00000131652.14	ENSG00000276204.1	ENSG00000260750.5
6518	ENSG00000131653.13	ENSG00000276203.4	ENSG00000183230.17
6519	ENSG00000131668.14	ENSG00000276202.1	ENSG00000250902.1
6520	ENSG00000131669.10	ENSG00000276200.1	ENSG00000213272.5
6521	ENSG00000131686.15	ENSG00000276197.1	ENSG00000188938.17
6522	ENSG00000131697.18	ENSG00000276193.1	ENSG00000170262.12
6523	ENSG00000131711.15	ENSG00000276189.1	ENSG00000204572.9
6524	ENSG00000131721.5	ENSG00000276188.1	ENSG00000286247.1
6525	ENSG00000131724.11	ENSG00000276185.1	ENSG00000148154.10
6526	ENSG00000131725.14	ENSG00000276183.1	ENSG00000259845.1
6527	ENSG00000131730.16	ENSG00000276182.1	ENSG00000267374.1
6528	ENSG00000131732.12	ENSG00000276181.1	ENSG00000245322.6
6529	ENSG00000131737.5	ENSG00000276180.1	ENSG00000249148.2
6530	ENSG00000131738.10	ENSG00000276179.1	ENSG00000270388.1
6531	ENSG00000131746.13	ENSG00000276178.1	ENSG00000267462.1
6532	ENSG00000131747.15	ENSG00000276176.1	ENSG00000255350.1
6533	ENSG00000131748.16	ENSG00000276174.1	ENSG00000239332.5
6534	ENSG00000131759.18	ENSG00000276172.1	ENSG00000215208.3
6535	ENSG00000131771.14	ENSG00000276170.4	ENSG00000280294.1
6536	ENSG00000131773.14	ENSG00000276169.1	ENSG00000227887.1
6537	ENSG00000131778.19	ENSG00000276168.1	ENSG00000269580.2
6538	ENSG00000131779.11	ENSG00000276166.1	ENSG00000198685.3
6539	ENSG00000131781.13	ENSG00000276164.1	ENSG00000108523.15
6540	ENSG00000131788.16	ENSG00000276162.1	ENSG00000171681.12
6541	ENSG00000131791.8	ENSG00000276161.1	ENSG00000115598.10
6542	ENSG00000131797.12	ENSG00000276160.1	ENSG00000219391.1
6543	ENSG00000131808.10	ENSG00000276158.2	ENSG00000235369.1
6544	ENSG00000131828.13	ENSG00000276156.1	ENSG00000095777.16
6545	ENSG00000131831.18	ENSG00000276150.1	ENSG00000283259.1
6546	ENSG00000131844.16	ENSG00000276149.1	ENSG00000240103.2
6547	ENSG00000131845.14	ENSG00000276148.1	ENSG00000198089.15
6548	ENSG00000131848.9	ENSG00000276147.1	ENSG00000248530.1
6549	ENSG00000131849.12	ENSG00000276141.4	ENSG00000276702.1
6550	ENSG00000131864.10	ENSG00000276140.1	ENSG00000238082.1
6551	ENSG00000131871.15	ENSG00000276138.1	ENSG00000184060.11
6552	ENSG00000131873.7	ENSG00000276137.1	ENSG00000118231.5
6553	ENSG00000131876.17	ENSG00000276131.1	ENSG00000244050.2
6554	ENSG00000131885.17	ENSG00000276128.1	ENSG00000271109.1
6555	ENSG00000131899.11	ENSG00000276127.1	ENSG00000242696.3
6556	ENSG00000131910.5	ENSG00000276124.1	ENSG00000272589.1
6557	ENSG00000131914.11	ENSG00000276123.1	ENSG00000278857.1
6558	ENSG00000131931.8	ENSG00000276122.1	ENSG00000263499.1
6559	ENSG00000131941.8	ENSG00000276121.1	ENSG00000278771.1
6560	ENSG00000131943.17	ENSG00000276119.1	ENSG00000236686.1
6561	ENSG00000131944.9	ENSG00000276118.1	ENSG00000269266.1
6562	ENSG00000131951.11	ENSG00000276116.2	ENSG00000273342.1
6563	ENSG00000131966.14	ENSG00000276115.1	ENSG00000227083.1
6564	ENSG00000131969.15	ENSG00000276110.1	ENSG00000251748.1
6565	ENSG00000131979.19	ENSG00000276109.1	ENSG00000168259.15
6566	ENSG00000131981.16	ENSG00000276107.1	ENSG00000232709.1
6567	ENSG00000131982.5	ENSG00000276105.1	ENSG00000214376.5
6568	ENSG00000132000.13	ENSG00000276103.1	ENSG00000279199.1
6569	ENSG00000132002.8	ENSG00000276102.1	ENSG00000186814.14

6570	ENSG00000132003.9	ENSG00000276101.1	ENSG00000229679.1
6571	ENSG00000132004.13	ENSG00000276100.1	ENSG00000141437.8
6572	ENSG00000132005.9	ENSG00000276098.1	ENSG00000130829.18
6573	ENSG00000132010.16	ENSG00000276097.1	ENSG00000259618.1
6574	ENSG00000132016.11	ENSG00000276096.1	ENSG00000177294.7
6575	ENSG00000132017.10	ENSG00000276095.2	ENSG00000279444.1
6576	ENSG00000132024.17	ENSG00000276094.1	ENSG00000224904.1
6577	ENSG00000132026.13	ENSG00000276093.1	ENSG00000277611.1
6578	ENSG00000132031.13	ENSG00000276092.1	ENSG00000241598.5
6579	ENSG00000132109.10	ENSG00000276089.1	ENSG00000286115.1
6580	ENSG00000132122.12	ENSG00000276088.1	ENSG00000218416.4
6581	ENSG00000132128.16	ENSG00000276087.2	ENSG00000286076.1
6582	ENSG00000132141.14	ENSG00000276085.1	ENSG00000226539.1
6583	ENSG00000132153.15	ENSG00000276083.1	ENSG00000224236.3
6584	ENSG00000132155.11	ENSG00000276081.1	ENSG00000233888.2
6585	ENSG00000132164.10	ENSG00000276077.4	ENSG00000264985.3
6586	ENSG00000132170.21	ENSG00000276076.4	ENSG00000166923.11
6587	ENSG00000132182.12	ENSG00000276075.1	ENSG00000248227.1
6588	ENSG00000132185.16	ENSG00000276074.1	ENSG00000230549.3
6589	ENSG00000132196.14	ENSG00000276071.1	ENSG00000255248.8
6590	ENSG00000132199.20	ENSG00000276070.5	ENSG00000169607.13
6591	ENSG00000132204.13	ENSG00000276067.2	ENSG00000179674.3
6592	ENSG00000132205.11	ENSG00000276064.1	ENSG00000272234.1
6593	ENSG00000132207.17	ENSG00000276063.1	ENSG00000277870.5
6594	ENSG00000132254.12	ENSG00000276058.1	ENSG00000231702.2
6595	ENSG00000132256.18	ENSG00000276057.1	ENSG00000226380.9
6596	ENSG00000132259.12	ENSG00000276055.1	ENSG00000228010.5
6597	ENSG00000132274.16	ENSG00000276054.1	ENSG00000101134.11
6598	ENSG00000132275.11	ENSG00000276050.1	ENSG00000254208.1
6599	ENSG00000132286.12	ENSG00000276048.1	ENSG00000280190.1
6600	ENSG00000132294.15	ENSG00000276047.1	ENSG00000271227.1
6601	ENSG00000132297.11	ENSG00000276046.1	ENSG00000232775.6
6602	ENSG00000132300.19	ENSG00000276045.3	ENSG00000204118.2
6603	ENSG00000132305.20	ENSG00000276043.5	ENSG00000279499.1
6604	ENSG00000132313.15	ENSG00000276040.4	ENSG00000235042.1
6605	ENSG00000132321.17	ENSG00000276039.1	ENSG00000272745.1
6606	ENSG00000132323.9	ENSG00000276036.1	ENSG00000259179.1
6607	ENSG00000132326.12	ENSG00000276031.1	ENSG00000279665.1
6608	ENSG00000132329.11	ENSG00000276030.1	ENSG00000185518.11
6609	ENSG00000132330.17	ENSG00000276029.1	ENSG00000266826.2
6610	ENSG00000132334.16	ENSG00000276027.1	ENSG00000186862.19
6611	ENSG00000132341.12	ENSG00000276026.1	ENSG00000231531.5
6612	ENSG00000132356.11	ENSG00000276023.5	ENSG00000261934.2
6613	ENSG00000132357.14	ENSG00000276019.1	ENSG00000181358.3
6614	ENSG00000132359.15	ENSG00000276017.1	ENSG00000219135.1
6615	ENSG00000132361.17	ENSG00000276015.1	ENSG00000165323.15
6616	ENSG00000132376.20	ENSG00000276012.1	ENSG00000203620.2
6617	ENSG00000132382.14	ENSG00000276007.1	ENSG00000136929.13
6618	ENSG00000132383.11	ENSG00000276006.1	ENSG00000261170.1
6619	ENSG00000132386.11	ENSG00000276003.1	ENSG00000143297.19
6620	ENSG00000132388.12	ENSG00000276002.1	ENSG00000182477.5
6621	ENSG00000132394.11	ENSG00000275997.1	ENSG00000187003.6
6622	ENSG00000132405.19	ENSG00000275996.1	ENSG00000248871.1

6623	ENSG00000132406.12	ENSG00000275995.1	ENSG00000161180.11
6624	ENSG00000132423.12	ENSG00000275994.1	ENSG00000227113.2
6625	ENSG00000132424.16	ENSG00000275993.2	ENSG00000163710.9
6626	ENSG00000132429.10	ENSG00000275992.1	ENSG00000225493.1
6627	ENSG00000132432.14	ENSG00000275989.2	ENSG00000259356.1
6628	ENSG00000132434.9	ENSG00000275987.1	ENSG00000272446.5
6629	ENSG00000132436.11	ENSG00000275982.1	ENSG00000248979.2
6630	ENSG00000132437.18	ENSG00000275981.1	ENSG00000100565.15
6631	ENSG00000132446.7	ENSG00000275980.1	ENSG00000261816.1
6632	ENSG00000132463.14	ENSG00000275978.1	ENSG00000275552.1
6633	ENSG00000132464.11	ENSG00000275976.5	ENSG00000230646.1
6634	ENSG00000132465.11	ENSG00000275975.1	ENSG00000236811.1
6635	ENSG00000132466.18	ENSG00000275971.1	ENSG00000225650.2
6636	ENSG00000132467.4	ENSG00000275969.2	ENSG00000227950.1
6637	ENSG00000132470.14	ENSG00000275968.1	ENSG00000100227.18
6638	ENSG00000132471.12	ENSG00000275967.1	ENSG00000248223.1
6639	ENSG00000132475.10	ENSG00000275966.1	ENSG00000258018.1
6640	ENSG00000132478.10	ENSG00000275965.1	ENSG00000207112.1
6641	ENSG00000132481.7	ENSG00000275964.1	ENSG00000081803.16
6642	ENSG00000132485.14	ENSG00000275963.1	ENSG00000271919.1
6643	ENSG00000132507.18	ENSG00000275961.1	ENSG00000116698.21
6644	ENSG00000132510.10	ENSG00000275959.1	ENSG00000162591.16
6645	ENSG00000132514.13	ENSG00000275958.1	ENSG00000261488.1
6646	ENSG00000132517.15	ENSG00000275956.1	ENSG00000102265.12
6647	ENSG00000132518.6	ENSG00000275954.5	ENSG00000271819.1
6648	ENSG00000132522.16	ENSG00000275953.1	ENSG00000271992.1
6649	ENSG00000132530.17	ENSG00000275950.1	ENSG00000277352.1
6650	ENSG00000132535.19	ENSG00000275948.1	ENSG00000106540.4
6651	ENSG00000132541.11	ENSG00000275945.1	ENSG00000260555.1
6652	ENSG00000132549.18	ENSG00000275944.1	ENSG00000228546.2
6653	ENSG00000132554.20	ENSG00000275942.1	ENSG00000236581.8
6654	ENSG00000132561.14	ENSG00000275940.1	ENSG00000149503.13
6655	ENSG00000132563.16	ENSG00000275939.1	ENSG00000169807.10
6656	ENSG00000132570.14	ENSG00000275936.1	ENSG00000224488.1
6657	ENSG00000132581.9	ENSG00000275933.1	ENSG00000138771.16
6658	ENSG00000132589.16	ENSG00000275930.1	ENSG00000224764.1
6659	ENSG00000132591.12	ENSG00000275929.1	ENSG00000249089.1
6660	ENSG00000132600.16	ENSG00000275928.2	ENSG00000109743.11
6661	ENSG00000132603.15	ENSG00000275927.1	ENSG00000079393.20
6662	ENSG00000132604.11	ENSG00000275924.1	ENSG00000258448.1
6663	ENSG00000132612.16	ENSG00000275923.1	ENSG00000231355.1
6664	ENSG00000132613.15	ENSG00000275919.1	ENSG00000196472.4
6665	ENSG00000132622.11	ENSG00000275915.1	ENSG00000233278.1
6666	ENSG00000132623.16	ENSG00000275910.1	ENSG00000214289.2
6667	ENSG00000132631.5	ENSG00000275909.1	ENSG00000251399.1
6668	ENSG00000132635.17	ENSG00000275908.1	ENSG00000007952.18
6669	ENSG00000132639.12	ENSG00000275906.1	ENSG00000259421.1
6670	ENSG00000132640.15	ENSG00000275905.1	ENSG00000184566.3
6671	ENSG00000132646.11	ENSG00000275904.1	ENSG00000123728.10
6672	ENSG00000132661.4	ENSG00000275902.1	ENSG00000277631.4
6673	ENSG00000132664.12	ENSG00000275901.1	ENSG00000230370.1
6674	ENSG00000132669.13	ENSG00000275900.1	ENSG00000090104.12
6675	ENSG00000132670.20	ENSG00000275898.1	ENSG00000171133.3

6676	ENSG00000132671.5	ENSG00000275897.1	ENSG00000274356.1
6677	ENSG00000132676.16	ENSG00000275896.5	ENSG00000169379.15
6678	ENSG00000132677.13	ENSG00000275895.7	ENSG00000257764.2
6679	ENSG00000132680.11	ENSG00000275894.1	ENSG00000258428.5
6680	ENSG00000132681.16	ENSG00000275888.1	ENSG00000232892.1
6681	ENSG00000132688.11	ENSG00000275882.1	ENSG00000056277.16
6682	ENSG00000132692.19	ENSG00000275881.1	ENSG00000278952.1
6683	ENSG00000132693.12	ENSG00000275880.1	ENSG00000226790.3
6684	ENSG00000132694.18	ENSG00000275878.1	ENSG00000235619.1
6685	ENSG00000132698.15	ENSG00000275877.1	ENSG00000270954.1
6686	ENSG00000132702.13	ENSG00000275875.1	ENSG00000224916.9
6687	ENSG00000132703.4	ENSG00000275874.1	ENSG00000166128.13
6688	ENSG00000132704.16	ENSG00000275871.1	ENSG00000185215.9
6689	ENSG00000132716.18	ENSG00000275869.1	ENSG00000256988.1
6690	ENSG00000132718.9	ENSG00000275868.1	ENSG00000204599.14
6691	ENSG00000132740.8	ENSG00000275866.1	ENSG00000253796.1
6692	ENSG00000132744.8	ENSG00000275863.1	ENSG00000224812.2
6693	ENSG00000132746.14	ENSG00000275862.1	ENSG00000256625.1
6694	ENSG00000132749.11	ENSG00000275860.1	ENSG00000253771.6
6695	ENSG00000132763.15	ENSG00000275859.1	ENSG00000266853.1
6696	ENSG00000132768.14	ENSG00000275857.1	ENSG00000111678.11
6697	ENSG00000132773.12	ENSG00000275856.1	ENSG00000172037.14
6698	ENSG00000132780.17	ENSG00000275854.1	ENSG00000274017.1
6699	ENSG00000132781.17	ENSG00000275853.1	ENSG00000249505.1
6700	ENSG00000132792.19	ENSG00000275852.1	ENSG00000179172.9
6701	ENSG00000132793.11	ENSG00000275850.1	ENSG00000106013.15
6702	ENSG00000132801.7	ENSG00000275846.1	ENSG00000132386.11
6703	ENSG00000132819.17	ENSG00000275842.1	ENSG00000171475.14
6704	ENSG00000132821.12	ENSG00000275840.1	ENSG00000099817.12
6705	ENSG00000132823.11	ENSG00000275839.1	ENSG00000235092.5
6706	ENSG00000132824.14	ENSG00000275836.1	ENSG00000233148.2
6707	ENSG00000132825.7	ENSG00000275835.5	ENSG00000242358.1
6708	ENSG00000132832.10	ENSG00000275834.1	ENSG00000229740.2
6709	ENSG00000132837.14	ENSG00000275833.1	ENSG00000224810.1
6710	ENSG00000132840.10	ENSG00000275832.5	ENSG00000276241.1
6711	ENSG00000132842.13	ENSG00000275830.1	ENSG00000204113.6
6712	ENSG00000132846.6	ENSG00000275828.1	ENSG00000267273.1
6713	ENSG00000132849.20	ENSG00000275827.1	ENSG00000261480.1
6714	ENSG00000132854.19	ENSG00000275826.1	ENSG00000207067.1
6715	ENSG00000132855.4	ENSG00000275823.1	ENSG00000249192.1
6716	ENSG00000132872.12	ENSG00000275818.1	ENSG00000255772.5
6717	ENSG00000132879.14	ENSG00000275816.1	ENSG00000125878.6
6718	ENSG00000132881.12	ENSG00000275812.1	ENSG00000270978.1
6719	ENSG00000132906.18	ENSG00000275811.1	ENSG00000197562.10
6720	ENSG00000132911.4	ENSG00000275810.1	ENSG00000085644.13
6721	ENSG00000132912.12	ENSG00000275808.1	ENSG00000229043.2
6722	ENSG00000132915.11	ENSG00000275807.1	ENSG00000160791.13
6723	ENSG00000132932.17	ENSG00000275805.1	ENSG00000180389.7
6724	ENSG00000132938.20	ENSG00000275803.1	ENSG00000227230.1
6725	ENSG00000132950.19	ENSG00000275801.1	ENSG00000168702.17
6726	ENSG00000132952.12	ENSG00000275800.1	ENSG00000270839.1
6727	ENSG00000132953.16	ENSG00000275799.1	ENSG00000213706.2
6728	ENSG00000132958.17	ENSG00000275793.1	ENSG00000227197.1

6729	ENSG00000132963.8	ENSG00000275791.1	ENSG00000202441.2
6730	ENSG00000132964.12	ENSG00000275789.1	ENSG00000235013.1
6731	ENSG00000132965.9	ENSG00000275788.1	ENSG00000206249.3
6732	ENSG00000132967.9	ENSG00000275787.1	ENSG00000257512.1
6733	ENSG00000132970.12	ENSG00000275785.1	ENSG00000202347.1
6734	ENSG00000132972.19	ENSG00000275778.2	ENSG00000279650.1
6735	ENSG00000132975.8	ENSG00000275776.1	ENSG00000224271.6
6736	ENSG00000133019.11	ENSG00000275774.2	ENSG00000198944.5
6737	ENSG00000133020.4	ENSG00000275773.1	ENSG00000217004.1
6738	ENSG00000133026.12	ENSG00000275772.1	ENSG00000231150.5
6739	ENSG00000133027.18	ENSG00000275771.1	ENSG00000226881.5
6740	ENSG00000133028.12	ENSG00000275770.1	ENSG00000233432.2
6741	ENSG00000133030.21	ENSG00000275769.1	ENSG00000226808.2
6742	ENSG00000133048.13	ENSG00000275768.1	ENSG00000105875.14
6743	ENSG00000133055.9	ENSG00000275767.1	ENSG00000248966.1
6744	ENSG00000133056.13	ENSG00000275765.5	ENSG00000164621.5
6745	ENSG00000133059.17	ENSG00000275764.1	ENSG00000128052.9
6746	ENSG00000133063.16	ENSG00000275763.3	ENSG00000259414.1
6747	ENSG00000133065.11	ENSG00000275759.1	ENSG00000279113.1
6748	ENSG00000133067.17	ENSG00000275757.1	ENSG00000261842.1
6749	ENSG00000133069.17	ENSG00000275756.1	ENSG00000250379.1
6750	ENSG00000133083.14	ENSG00000275747.1	ENSG00000201998.1
6751	ENSG00000133101.10	ENSG00000275745.1	ENSG00000270791.1
6752	ENSG00000133103.17	ENSG00000275743.1	ENSG00000255060.1
6753	ENSG00000133104.14	ENSG00000275741.1	ENSG00000127423.10
6754	ENSG00000133105.8	ENSG00000275740.1	ENSG00000156026.14
6755	ENSG00000133106.14	ENSG00000275734.2	ENSG00000242488.2
6756	ENSG00000133107.14	ENSG00000275733.1	ENSG00000180458.2
6757	ENSG00000133110.15	ENSG00000275728.1	ENSG00000273596.1
6758	ENSG00000133111.3	ENSG00000275726.1	ENSG00000207547.1
6759	ENSG00000133112.16	ENSG00000275725.1	ENSG00000119283.15
6760	ENSG00000133114.17	ENSG00000275722.5	ENSG00000217315.1
6761	ENSG00000133115.11	ENSG00000275719.1	ENSG00000254942.1
6762	ENSG00000133116.8	ENSG00000275718.2	ENSG00000128276.10
6763	ENSG00000133119.13	ENSG00000275716.1	ENSG00000263924.1
6764	ENSG00000133121.21	ENSG00000275714.1	ENSG00000268764.1
6765	ENSG00000133124.11	ENSG00000275713.2	ENSG00000274993.1
6766	ENSG00000133131.15	ENSG00000275711.1	ENSG00000148908.15
6767	ENSG00000133134.11	ENSG00000275710.1	ENSG00000265799.1
6768	ENSG00000133135.14	ENSG00000275709.1	ENSG00000170276.6
6769	ENSG00000133136.4	ENSG00000275708.1	ENSG00000206965.1
6770	ENSG00000133138.20	ENSG00000275703.1	ENSG00000233834.6
6771	ENSG00000133142.17	ENSG00000275700.5	ENSG00000182853.12
6772	ENSG00000133169.6	ENSG00000275696.1	ENSG00000177084.16
6773	ENSG00000133193.12	ENSG00000275695.1	ENSG00000113384.14
6774	ENSG00000133195.11	ENSG00000275693.1	ENSG00000127928.12
6775	ENSG00000133216.16	ENSG00000275692.1	ENSG00000265942.2
6776	ENSG00000133226.17	ENSG00000275691.2	ENSG00000214253.8
6777	ENSG00000133243.8	ENSG00000275689.1	ENSG00000255310.2
6778	ENSG00000133246.12	ENSG00000275688.4	ENSG00000231715.1
6779	ENSG00000133247.14	ENSG00000275681.1	ENSG00000234629.1
6780	ENSG00000133250.14	ENSG00000275680.1	ENSG00000084453.16
6781	ENSG00000133256.12	ENSG00000275678.1	ENSG00000241388.5

6782	ENSG00000133265.11	ENSG00000275675.1	ENSG00000228038.1
6783	ENSG00000133275.16	ENSG00000275674.1	ENSG00000244694.7
6784	ENSG00000133302.13	ENSG00000275672.1	ENSG00000239856.3
6785	ENSG00000133313.15	ENSG00000275670.1	ENSG00000272944.1
6786	ENSG00000133315.11	ENSG00000275669.1	ENSG00000255958.1
6787	ENSG00000133316.15	ENSG00000275667.1	ENSG00000267393.1
6788	ENSG00000133317.14	ENSG00000275666.1	ENSG00000272600.1
6789	ENSG00000133318.13	ENSG00000275665.1	ENSG00000280167.1
6790	ENSG00000133321.11	ENSG00000275664.1	ENSG00000236130.2
6791	ENSG00000133328.4	ENSG00000275663.1	ENSG00000173231.6
6792	ENSG00000133392.18	ENSG00000275662.1	ENSG00000158786.4
6793	ENSG00000133393.13	ENSG00000275661.1	ENSG00000133878.9
6794	ENSG00000133398.4	ENSG00000275656.1	ENSG00000255763.1
6795	ENSG00000133401.16	ENSG00000275655.1	ENSG00000010704.18
6796	ENSG00000133422.13	ENSG00000275654.1	ENSG00000183578.7
6797	ENSG00000133424.20	ENSG00000275652.1	ENSG00000247763.2
6798	ENSG00000133433.10	ENSG00000275651.1	ENSG00000231507.1
6799	ENSG00000133454.15	ENSG00000275649.1	ENSG00000236849.5
6800	ENSG00000133460.19	ENSG00000275647.1	ENSG00000141736.13
6801	ENSG00000133466.14	ENSG00000275646.1	ENSG00000227653.1
6802	ENSG00000133475.17	ENSG00000275645.1	ENSG00000233061.1
6803	ENSG00000133477.17	ENSG00000275643.1	ENSG00000200597.1
6804	ENSG00000133488.14	ENSG00000275642.1	ENSG00000183137.14
6805	ENSG00000133519.12	ENSG00000275640.1	ENSG00000186318.16
6806	ENSG00000133561.15	ENSG00000275638.1	ENSG00000207166.1
6807	ENSG00000133574.10	ENSG00000275636.1	ENSG00000270296.1
6808	ENSG00000133597.11	ENSG00000275635.1	ENSG00000264148.1
6809	ENSG00000133606.11	ENSG00000275634.1	ENSG00000233473.2
6810	ENSG00000133612.19	ENSG00000275632.1	ENSG00000233419.3
6811	ENSG00000133619.17	ENSG00000275631.1	ENSG00000204403.9
6812	ENSG00000133624.13	ENSG00000275630.1	ENSG00000126259.19
6813	ENSG00000133627.18	ENSG00000275628.2	ENSG00000117245.12
6814	ENSG00000133636.11	ENSG00000275625.1	ENSG00000241464.3
6815	ENSG00000133639.5	ENSG00000275620.1	ENSG00000178075.20
6816	ENSG00000133640.20	ENSG00000275616.1	ENSG00000223754.1
6817	ENSG00000133641.18	ENSG00000275613.1	ENSG00000221676.1
6818	ENSG00000133657.15	ENSG00000275612.1	ENSG00000188997.8
6819	ENSG00000133661.16	ENSG00000275610.1	ENSG00000164627.18
6820	ENSG00000133665.13	ENSG00000275609.1	ENSG00000280191.3
6821	ENSG00000133678.14	ENSG00000275607.1	ENSG00000170312.15
6822	ENSG00000133687.16	ENSG00000275601.1	ENSG00000285570.1
6823	ENSG00000133703.11	ENSG00000275598.1	ENSG00000253869.1
6824	ENSG00000133704.10	ENSG00000275597.1	ENSG00000276015.1
6825	ENSG00000133706.18	ENSG00000275592.1	ENSG00000283176.1
6826	ENSG00000133731.10	ENSG00000275591.4	ENSG00000282222.1
6827	ENSG00000133739.16	ENSG00000275589.1	ENSG00000200156.1
6828	ENSG00000133740.11	ENSG00000275588.1	ENSG00000254692.1
6829	ENSG00000133742.13	ENSG00000275586.1	ENSG00000183310.3
6830	ENSG00000133773.12	ENSG00000275585.2	ENSG00000172139.15
6831	ENSG00000133789.15	ENSG00000275582.1	ENSG00000258323.1
6832	ENSG00000133794.17	ENSG00000275580.1	ENSG00000105393.16
6833	ENSG00000133800.8	ENSG00000275578.1	ENSG00000231344.1
6834	ENSG00000133805.15	ENSG00000275576.1	ENSG00000213318.4

6835	ENSG00000133812.15	ENSG00000275572.1	ENSG00000224000.1
6836	ENSG00000133816.15	ENSG00000275569.1	ENSG00000260577.2
6837	ENSG00000133818.14	ENSG00000275567.1	ENSG00000274904.1
6838	ENSG00000133835.15	ENSG00000275563.1	ENSG00000223692.1
6839	ENSG00000133858.15	ENSG00000275562.1	ENSG00000202324.1
6840	ENSG00000133863.8	ENSG00000275560.1	ENSG00000236862.1
6841	ENSG00000133872.13	ENSG00000275559.1	ENSG00000266184.1
6842	ENSG00000133874.2	ENSG00000275558.1	ENSG00000151388.11
6843	ENSG00000133878.9	ENSG00000275557.1	ENSG00000272788.1
6844	ENSG00000133884.10	ENSG00000275553.3	ENSG00000225523.2
6845	ENSG00000133895.14	ENSG00000275552.1	ENSG00000249359.2
6846	ENSG00000133935.6	ENSG00000275550.1	ENSG00000273513.1
6847	ENSG00000133937.4	ENSG00000275549.1	ENSG00000224958.5
6848	ENSG00000133943.20	ENSG00000275547.1	ENSG00000224846.2
6849	ENSG00000133958.13	ENSG00000275542.1	ENSG00000283636.1
6850	ENSG00000133961.20	ENSG00000275540.1	ENSG00000237786.1
6851	ENSG00000133962.7	ENSG00000275538.1	ENSG00000286201.1
6852	ENSG00000133980.5	ENSG00000275532.1	ENSG00000280097.1
6853	ENSG00000133983.15	ENSG00000275529.1	ENSG00000279656.1
6854	ENSG00000133985.3	ENSG00000275527.1	ENSG00000099725.14
6855	ENSG00000133997.11	ENSG00000275524.1	ENSG00000257675.1
6856	ENSG00000134001.13	ENSG00000275523.1	ENSG00000239435.2
6857	ENSG00000134007.4	ENSG00000275520.1	ENSG00000275572.1
6858	ENSG00000134013.15	ENSG00000275519.1	ENSG00000268309.1
6859	ENSG00000134014.17	ENSG00000275518.1	ENSG00000148377.6
6860	ENSG00000134020.8	ENSG00000275516.1	ENSG00000198759.12
6861	ENSG00000134028.14	ENSG00000275515.1	ENSG00000253218.1
6862	ENSG00000134030.14	ENSG00000275512.1	ENSG00000231122.5
6863	ENSG00000134042.13	ENSG00000275508.1	ENSG00000254897.1
6864	ENSG00000134046.12	ENSG00000275506.1	ENSG00000163364.9
6865	ENSG00000134049.6	ENSG00000275504.1	ENSG00000240265.1
6866	ENSG00000134056.12	ENSG00000275503.1	ENSG00000273171.1
6867	ENSG00000134057.15	ENSG00000275502.1	ENSG00000230815.1
6868	ENSG00000134058.12	ENSG00000275497.1	ENSG00000254862.5
6869	ENSG00000134061.5	ENSG00000275496.4	ENSG00000275515.1
6870	ENSG00000134070.5	ENSG00000275494.1	ENSG00000262898.2
6871	ENSG00000134072.11	ENSG00000275491.1	ENSG00000096006.11
6872	ENSG00000134077.16	ENSG00000275490.1	ENSG00000212951.5
6873	ENSG00000134086.7	ENSG00000275489.1	ENSG00000185697.16
6874	ENSG00000134107.4	ENSG00000275488.1	ENSG00000273866.1
6875	ENSG00000134108.13	ENSG00000275485.1	ENSG00000164049.14
6876	ENSG00000134109.11	ENSG00000275484.1	ENSG00000124440.15
6877	ENSG00000134115.13	ENSG00000275481.1	ENSG00000140254.12
6878	ENSG00000134121.10	ENSG00000275480.1	ENSG00000272707.1
6879	ENSG00000134138.20	ENSG00000275479.1	ENSG00000103200.6
6880	ENSG00000134146.12	ENSG00000275476.1	ENSG00000249271.2
6881	ENSG00000134152.11	ENSG00000275475.1	ENSG00000213956.4
6882	ENSG00000134153.10	ENSG00000275469.1	ENSG00000242262.1
6883	ENSG00000134160.13	ENSG00000275468.1	ENSG00000134533.6
6884	ENSG00000134183.11	ENSG00000275467.1	ENSG00000276408.1
6885	ENSG00000134184.12	ENSG00000275465.6	ENSG00000228000.1
6886	ENSG00000134186.12	ENSG00000275464.4	ENSG00000253620.2
6887	ENSG00000134193.15	ENSG00000275461.1	ENSG00000284677.1

6888	ENSG00000134198.10	ENSG00000275460.1	ENSG00000223819.2
6889	ENSG00000134200.3	ENSG00000275458.1	ENSG00000256734.1
6890	ENSG00000134201.12	ENSG00000275457.1	ENSG00000175766.13
6891	ENSG00000134202.10	ENSG00000275455.1	ENSG00000276934.1
6892	ENSG00000134207.16	ENSG00000275454.1	ENSG00000256283.1
6893	ENSG00000134215.16	ENSG00000275453.2	ENSG00000233098.8
6894	ENSG00000134216.19	ENSG00000275451.1	ENSG00000168746.8
6895	ENSG00000134222.16	ENSG00000275450.1	ENSG00000104043.14
6896	ENSG00000134240.11	ENSG00000275449.1	ENSG00000280078.1
6897	ENSG00000134242.16	ENSG00000275448.1	ENSG00000196843.16
6898	ENSG00000134243.12	ENSG00000275445.1	ENSG00000267320.5
6899	ENSG00000134245.18	ENSG00000275443.1	ENSG00000270171.1
6900	ENSG00000134247.10	ENSG00000275438.1	ENSG00000259099.2
6901	ENSG00000134248.13	ENSG00000275437.1	ENSG00000273792.1
6902	ENSG00000134249.6	ENSG00000275432.1	ENSG00000220875.1
6903	ENSG00000134250.20	ENSG00000275431.1	ENSG00000256150.2
6904	ENSG00000134253.10	ENSG00000275429.1	ENSG00000227215.1
6905	ENSG00000134255.14	ENSG00000275427.1	ENSG00000185862.7
6906	ENSG00000134256.12	ENSG00000275426.1	ENSG00000233569.1
6907	ENSG00000134258.17	ENSG00000275423.1	ENSG00000188460.4
6908	ENSG00000134259.3	ENSG00000275419.1	ENSG00000259066.5
6909	ENSG00000134262.13	ENSG00000275418.1	ENSG00000257507.1
6910	ENSG00000134265.13	ENSG00000275417.1	ENSG00000151631.8
6911	ENSG00000134278.15	ENSG00000275414.1	ENSG00000205129.8
6912	ENSG00000134283.17	ENSG00000275413.1	ENSG00000224437.2
6913	ENSG00000134285.11	ENSG00000275411.1	ENSG00000230832.3
6914	ENSG00000134287.10	ENSG00000275410.5	ENSG00000163406.11
6915	ENSG00000134291.12	ENSG00000275409.1	ENSG00000265394.1
6916	ENSG00000134294.14	ENSG00000275406.1	ENSG00000113194.13
6917	ENSG00000134297.6	ENSG00000275405.1	ENSG00000258599.2
6918	ENSG00000134308.14	ENSG00000275401.1	ENSG00000261963.3
6919	ENSG00000134313.15	ENSG00000275400.1	ENSG00000219693.3
6920	ENSG00000134317.18	ENSG00000275395.5	ENSG00000225339.3
6921	ENSG00000134318.14	ENSG00000275393.1	ENSG00000108953.17
6922	ENSG00000134321.12	ENSG00000275392.1	ENSG00000142484.7
6923	ENSG00000134323.12	ENSG00000275391.1	ENSG00000224843.7
6924	ENSG00000134324.11	ENSG00000275390.1	ENSG00000264754.1
6925	ENSG00000134326.11	ENSG00000275389.1	ENSG00000226131.1
6926	ENSG00000134330.18	ENSG00000275387.1	ENSG00000253230.8
6927	ENSG00000134333.14	ENSG00000275386.1	ENSG00000263974.2
6928	ENSG00000134339.8	ENSG00000275385.1	ENSG00000103023.11
6929	ENSG00000134343.13	ENSG00000275383.1	ENSG00000224555.3
6930	ENSG00000134352.20	ENSG00000275381.1	ENSG00000223298.1
6931	ENSG00000134363.12	ENSG00000275379.1	ENSG00000243370.3
6932	ENSG00000134365.13	ENSG00000275377.1	ENSG00000163291.14
6933	ENSG00000134369.15	ENSG00000275374.1	ENSG00000175826.12
6934	ENSG00000134371.12	ENSG00000275373.1	ENSG00000269288.1
6935	ENSG00000134375.11	ENSG00000275372.1	ENSG00000166448.14
6936	ENSG00000134376.16	ENSG00000275371.1	ENSG00000232381.1
6937	ENSG00000134389.9	ENSG00000275367.1	ENSG00000262001.1
6938	ENSG00000134398.15	ENSG00000275363.1	ENSG00000281386.1
6939	ENSG00000134419.15	ENSG00000275362.1	ENSG00000222898.1
6940	ENSG00000134438.9	ENSG00000275361.1	ENSG00000274698.1

6941	ENSG00000134440.12	ENSG00000275360.1	ENSG00000169826.8
6942	ENSG00000134443.10	ENSG00000275359.1	ENSG00000069482.7
6943	ENSG00000134444.14	ENSG00000275358.1	ENSG00000214244.4
6944	ENSG00000134452.19	ENSG00000275356.4	ENSG00000226757.2
6945	ENSG00000134453.16	ENSG00000275355.1	ENSG00000214908.3
6946	ENSG00000134460.17	ENSG00000275352.1	ENSG00000175121.11
6947	ENSG00000134461.16	ENSG00000275350.1	ENSG00000109846.8
6948	ENSG00000134463.15	ENSG00000275348.1	ENSG00000167654.18
6949	ENSG00000134470.21	ENSG00000275345.1	ENSG00000276588.1
6950	ENSG00000134480.15	ENSG00000275344.1	ENSG00000179580.10
6951	ENSG00000134489.7	ENSG00000275343.1	ENSG00000176857.5
6952	ENSG00000134490.14	ENSG00000275342.4	ENSG00000211626.2
6953	ENSG00000134504.13	ENSG00000275340.1	ENSG00000254170.1
6954	ENSG00000134508.12	ENSG00000275339.1	ENSG00000277083.1
6955	ENSG00000134516.16	ENSG00000275337.1	ENSG00000248809.6
6956	ENSG00000134531.10	ENSG00000275335.1	ENSG00000258740.1
6957	ENSG00000134532.17	ENSG00000275334.1	ENSG00000232625.1
6958	ENSG00000134533.6	ENSG00000275332.1	ENSG00000258380.1
6959	ENSG00000134538.2	ENSG00000275328.1	ENSG00000204909.8
6960	ENSG00000134539.17	ENSG00000275327.1	ENSG00000223688.2
6961	ENSG00000134545.13	ENSG00000275325.4	ENSG00000239983.1
6962	ENSG00000134548.11	ENSG00000275322.1	ENSG00000226989.1
6963	ENSG00000134551.12	ENSG00000275320.1	ENSG00000234985.2
6964	ENSG00000134569.10	ENSG00000275319.1	ENSG00000152254.10
6965	ENSG00000134571.10	ENSG00000275318.1	ENSG00000261093.1
6966	ENSG00000134574.11	ENSG00000275315.1	ENSG00000253931.1
6967	ENSG00000134575.10	ENSG00000275314.1	ENSG00000282968.1
6968	ENSG00000134588.12	ENSG00000275312.1	ENSG00000274038.1
6969	ENSG00000134590.13	ENSG00000275310.1	ENSG00000188831.4
6970	ENSG00000134594.5	ENSG00000275307.1	ENSG00000221716.1
6971	ENSG00000134595.8	ENSG00000275305.1	ENSG00000177338.13
6972	ENSG00000134597.16	ENSG00000275302.2	ENSG00000207524.1
6973	ENSG00000134602.16	ENSG00000275297.1	ENSG00000127318.11
6974	ENSG00000134612.12	ENSG00000275296.1	ENSG00000267507.1
6975	ENSG00000134627.12	ENSG00000275295.1	ENSG00000216937.13
6976	ENSG00000134640.2	ENSG00000275294.4	ENSG00000183454.17
6977	ENSG00000134644.15	ENSG00000275293.1	ENSG00000100302.7
6978	ENSG00000134668.12	ENSG00000275291.1	ENSG00000254934.5
6979	ENSG00000134684.10	ENSG00000275287.5_PAR_Y	ENSG00000224707.1
6980	ENSG00000134686.18	ENSG00000275287.5	ENSG00000235482.1
6981	ENSG00000134690.11	ENSG00000275286.1	ENSG00000264801.1
6982	ENSG00000134697.13	ENSG00000275285.1	ENSG00000163702.20
6983	ENSG00000134698.11	ENSG00000275280.1	ENSG00000182492.16
6984	ENSG00000134709.11	ENSG00000275278.1	ENSG00000178234.13
6985	ENSG00000134716.11	ENSG00000275273.1	ENSG00000178795.9
6986	ENSG00000134717.18	ENSG00000275268.1	ENSG00000259013.2
6987	ENSG00000134744.14	ENSG00000275266.1	ENSG00000258785.5
6988	ENSG00000134748.13	ENSG00000275265.1	ENSG00000286240.1
6989	ENSG00000134755.16	ENSG00000275263.1	ENSG00000279263.1
6990	ENSG00000134757.5	ENSG00000275259.1	ENSG00000250030.2
6991	ENSG00000134758.14	ENSG00000275256.1	ENSG00000104522.16
6992	ENSG00000134759.14	ENSG00000275254.1	ENSG00000126261.13
6993	ENSG00000134760.6	ENSG00000275250.1	ENSG00000237631.2

6994	ENSG00000134762.17	ENSG00000275249.1	ENSG00000132589.16
6995	ENSG00000134765.9	ENSG00000275248.1	ENSG00000256640.1
6996	ENSG00000134769.21	ENSG00000275243.1	ENSG00000204661.9
6997	ENSG00000134775.15	ENSG00000275239.4	ENSG00000249715.12
6998	ENSG00000134779.15	ENSG00000275238.1	ENSG00000196172.9
6999	ENSG00000134780.10	ENSG00000275236.1	ENSG00000227123.1
7000	ENSG00000134802.17	ENSG00000275234.1	ENSG00000249633.1
7001	ENSG00000134809.9	ENSG00000275232.1	ENSG00000250562.1
7002	ENSG00000134812.8	ENSG00000275231.1	ENSG00000238151.1
7003	ENSG00000134815.19	ENSG00000275230.1	ENSG00000182000.9
7004	ENSG00000134817.10	ENSG00000275229.1	ENSG00000280269.1
7005	ENSG00000134824.14	ENSG00000275228.1	ENSG00000235181.1
7006	ENSG00000134825.15	ENSG00000275227.1	ENSG00000216819.1
7007	ENSG00000134827.8	ENSG00000275226.1	ENSG00000014216.15
7008	ENSG00000134830.5	ENSG00000275223.1	ENSG00000017260.19
7009	ENSG00000134851.13	ENSG00000275222.1	ENSG00000256372.1
7010	ENSG00000134852.15	ENSG00000275221.1	ENSG00000163060.8
7011	ENSG00000134853.12	ENSG00000275219.1	ENSG00000227105.1
7012	ENSG00000134864.10	ENSG00000275216.2	ENSG00000279836.1
7013	ENSG00000134871.18	ENSG00000275215.1	ENSG00000163632.13
7014	ENSG00000134873.10	ENSG00000275213.1	ENSG00000253851.1
7015	ENSG00000134874.17	ENSG00000275212.2	ENSG00000254418.1
7016	ENSG00000134882.15	ENSG00000275210.1	ENSG00000215790.7
7017	ENSG00000134884.15	ENSG00000275208.1	ENSG00000263887.7
7018	ENSG00000134897.14	ENSG00000275207.1	ENSG00000264186.1
7019	ENSG00000134899.20	ENSG00000275206.1	ENSG00000232949.1
7020	ENSG00000134900.12	ENSG00000275202.1	ENSG00000242444.3
7021	ENSG00000134901.13	ENSG00000275201.1	ENSG00000130165.10
7022	ENSG00000134905.17	ENSG00000275198.1	ENSG00000234352.9
7023	ENSG00000134909.18	ENSG00000275197.1	ENSG00000253125.1
7024	ENSG00000134910.13	ENSG00000275194.1	ENSG00000273006.1
7025	ENSG00000134917.10	ENSG00000275191.1	ENSG00000277893.2
7026	ENSG00000134940.13	ENSG00000275186.1	ENSG00000130173.13
7027	ENSG00000134954.14	ENSG00000275185.1	ENSG00000268738.3
7028	ENSG00000134955.11	ENSG00000275180.1	ENSG00000228124.1
7029	ENSG00000134962.7	ENSG00000275178.1	ENSG00000166111.9
7030	ENSG00000134970.14	ENSG00000275175.1	ENSG00000273675.1
7031	ENSG00000134982.17	ENSG00000275174.1	ENSG00000255386.1
7032	ENSG00000134986.13	ENSG00000275173.1	ENSG00000263969.2
7033	ENSG00000134987.11	ENSG00000275170.1	ENSG00000214940.8
7034	ENSG00000134996.11	ENSG00000275167.1	ENSG00000237951.1
7035	ENSG00000135002.12	ENSG00000275166.1	ENSG00000241472.6
7036	ENSG00000135018.14	ENSG00000275163.1	ENSG00000280395.1
7037	ENSG00000135040.15	ENSG00000275162.1	ENSG00000100191.5
7038	ENSG00000135045.7	ENSG00000275161.1	ENSG00000203855.7
7039	ENSG00000135046.14	ENSG00000275160.1	ENSG00000253147.5
7040	ENSG00000135047.15	ENSG00000275158.1	ENSG00000236274.1
7041	ENSG00000135048.14	ENSG00000275157.1	ENSG00000199809.1
7042	ENSG00000135049.15	ENSG00000275155.1	ENSG00000237009.2
7043	ENSG00000135052.16	ENSG00000275154.1	ENSG00000250362.1
7044	ENSG00000135063.19	ENSG00000275152.5	ENSG00000220744.1
7045	ENSG00000135069.14	ENSG00000275146.1	ENSG00000204382.11
7046	ENSG00000135070.15	ENSG00000275144.1	ENSG00000261915.6

7047	ENSG00000135074.15	ENSG00000275143.1	ENSG00000269099.2
7048	ENSG00000135077.9	ENSG00000275141.1	ENSG00000261318.1
7049	ENSG00000135083.15	ENSG00000275140.1	ENSG00000242958.1
7050	ENSG00000135090.14	ENSG00000275139.1	ENSG00000270533.2
7051	ENSG00000135093.13	ENSG00000275138.1	ENSG00000277579.1
7052	ENSG00000135094.11	ENSG00000275134.1	ENSG00000244183.1
7053	ENSG00000135097.7	ENSG00000275132.1	ENSG00000263905.2
7054	ENSG00000135100.17	ENSG00000275131.3	ENSG00000239908.3
7055	ENSG00000135108.14	ENSG00000275130.1	ENSG00000214204.4
7056	ENSG00000135111.16	ENSG00000275129.1	ENSG00000224349.2
7057	ENSG00000135114.12	ENSG00000275128.1	ENSG00000213512.3
7058	ENSG00000135116.9	ENSG00000275127.1	ENSG00000066405.13
7059	ENSG00000135119.14	ENSG00000275126.1	ENSG00000273706.4
7060	ENSG00000135124.14	ENSG00000275121.1	ENSG00000087460.24
7061	ENSG00000135127.11	ENSG00000275120.2	ENSG00000124785.9
7062	ENSG00000135144.7	ENSG00000275119.1	ENSG00000210196.2
7063	ENSG00000135148.12	ENSG00000275115.1	ENSG00000229873.1
7064	ENSG00000135164.18	ENSG00000275113.1	ENSG00000265123.2
7065	ENSG00000135175.5	ENSG00000275111.5	ENSG00000272545.1
7066	ENSG00000135185.12	ENSG00000275110.1	ENSG00000184557.4
7067	ENSG00000135205.15	ENSG00000275109.1	ENSG00000229238.3
7068	ENSG00000135211.6	ENSG00000275108.1	ENSG00000262294.1
7069	ENSG00000135218.19	ENSG00000275107.1	ENSG00000248458.2
7070	ENSG00000135220.11	ENSG00000275106.1	ENSG00000188820.13
7071	ENSG00000135222.6	ENSG00000275103.1	ENSG00000203645.2
7072	ENSG00000135226.17	ENSG00000275101.1	ENSG00000230140.6
7073	ENSG00000135241.17	ENSG00000275097.1	ENSG00000228791.7
7074	ENSG00000135245.10	ENSG00000275094.1	ENSG00000180019.5
7075	ENSG00000135248.15	ENSG00000275092.1	ENSG00000124678.18
7076	ENSG00000135249.8	ENSG00000275091.1	ENSG00000091138.12
7077	ENSG00000135250.17	ENSG00000275090.1	ENSG00000258260.1
7078	ENSG00000135253.13	ENSG00000275088.1	ENSG00000236152.1
7079	ENSG00000135269.18	ENSG00000275084.3	ENSG00000108799.13
7080	ENSG00000135272.10	ENSG00000275078.1	ENSG00000259276.1
7081	ENSG00000135297.15	ENSG00000275075.1	ENSG00000234806.1
7082	ENSG00000135298.14	ENSG00000275074.2	ENSG00000278983.1
7083	ENSG00000135299.17	ENSG00000275072.1	ENSG00000148735.15
7084	ENSG00000135312.6	ENSG00000275071.1	ENSG00000262583.1
7085	ENSG00000135314.12	ENSG00000275070.1	ENSG00000185203.12
7086	ENSG00000135315.12	ENSG00000275069.1	ENSG00000278635.1
7087	ENSG00000135316.17	ENSG00000275068.1	ENSG00000284523.1
7088	ENSG00000135317.13	ENSG00000275067.1	ENSG00000278920.1
7089	ENSG00000135318.12	ENSG00000275066.5	ENSG00000269742.1
7090	ENSG00000135324.6	ENSG00000275064.1	ENSG00000201096.1
7091	ENSG00000135333.14	ENSG00000275061.2	ENSG00000182329.13
7092	ENSG00000135334.9	ENSG00000275060.1	ENSG00000107807.13
7093	ENSG00000135336.14	ENSG00000275056.1	ENSG00000158055.15
7094	ENSG00000135338.14	ENSG00000275055.1	ENSG00000244227.7
7095	ENSG00000135341.18	ENSG00000275054.1	ENSG00000135374.9
7096	ENSG00000135346.8	ENSG00000275052.5	ENSG00000279588.2
7097	ENSG00000135355.4	ENSG00000275048.1	ENSG00000278518.1
7098	ENSG00000135362.14	ENSG00000275046.1	ENSG00000256612.7
7099	ENSG00000135363.12	ENSG00000275043.1	ENSG00000225170.2

7100	ENSG00000135365.15	ENSG00000275041.1	ENSG00000225407.3
7101	ENSG00000135372.9	ENSG00000275040.1	ENSG00000237916.1
7102	ENSG00000135373.12	ENSG00000275038.2	ENSG00000215246.5
7103	ENSG00000135374.9	ENSG00000275036.1	ENSG00000100526.20
7104	ENSG00000135378.4	ENSG00000275033.1	ENSG00000229276.1
7105	ENSG00000135387.21	ENSG00000275030.1	ENSG00000252539.1
7106	ENSG00000135390.19	ENSG00000275029.1	ENSG00000253733.3
7107	ENSG00000135392.16	ENSG00000275026.1	ENSG00000255966.1
7108	ENSG00000135404.11	ENSG00000275025.1	ENSG00000244053.1
7109	ENSG00000135406.14	ENSG00000275024.1	ENSG00000280408.1
7110	ENSG00000135407.10	ENSG00000275023.4	ENSG00000236890.2
7111	ENSG00000135409.11	ENSG00000275022.1	ENSG00000241868.3
7112	ENSG00000135413.9	ENSG00000275017.1	ENSG00000176826.15
7113	ENSG00000135414.9	ENSG00000275016.4	ENSG00000227813.1
7114	ENSG00000135423.13	ENSG00000275015.1	ENSG00000254027.1
7115	ENSG00000135424.16	ENSG00000275014.1	ENSG00000224318.5
7116	ENSG00000135426.16	ENSG00000275012.1	ENSG00000167355.8
7117	ENSG00000135436.8	ENSG00000275011.1	ENSG00000214980.4
7118	ENSG00000135437.9	ENSG00000275006.1	ENSG00000143382.14
7119	ENSG00000135439.11	ENSG00000275005.1	ENSG00000007171.17
7120	ENSG00000135441.7	ENSG00000275004.4	ENSG00000280377.1
7121	ENSG00000135443.8	ENSG00000275002.1	ENSG00000235183.3
7122	ENSG00000135446.17	ENSG00000275001.1	ENSG00000241218.1
7123	ENSG00000135447.17	ENSG00000274999.1	ENSG00000283440.1
7124	ENSG00000135451.13	ENSG00000274998.1	ENSG00000239486.1
7125	ENSG00000135452.10	ENSG00000274997.1	ENSG00000157212.18
7126	ENSG00000135454.14	ENSG00000274996.1	ENSG00000112144.15
7127	ENSG00000135457.9	ENSG00000274995.1	ENSG00000162391.12
7128	ENSG00000135469.13	ENSG00000274994.1	ENSG00000200783.1
7129	ENSG00000135472.9	ENSG00000274993.1	ENSG00000197233.7
7130	ENSG00000135473.15	ENSG00000274988.1	ENSG00000207561.1
7131	ENSG00000135476.11	ENSG00000274986.1	ENSG00000272516.1
7132	ENSG00000135477.11	ENSG00000274985.1	ENSG00000225751.2
7133	ENSG00000135480.16	ENSG00000274984.1	ENSG00000212479.2
7134	ENSG00000135482.7	ENSG00000274979.1	ENSG00000253754.1
7135	ENSG00000135486.17	ENSG00000274978.1	ENSG00000070614.15
7136	ENSG00000135502.17	ENSG00000274977.1	ENSG00000236090.2
7137	ENSG00000135503.13	ENSG00000274976.1	ENSG00000184459.8
7138	ENSG00000135506.16	ENSG00000274975.1	ENSG00000236182.1
7139	ENSG00000135517.8	ENSG00000274973.1	ENSG00000204429.4
7140	ENSG00000135519.8	ENSG00000274970.1	ENSG00000237512.6
7141	ENSG00000135521.9	ENSG00000274969.1	ENSG00000236048.2
7142	ENSG00000135525.18	ENSG00000274967.1	ENSG00000256069.7
7143	ENSG00000135535.17	ENSG00000274966.1	ENSG00000283805.1
7144	ENSG00000135537.16	ENSG00000274964.1	ENSG00000260500.1
7145	ENSG00000135540.11	ENSG00000274963.1	ENSG00000258498.8
7146	ENSG00000135541.21	ENSG00000274962.1	ENSG00000265967.1
7147	ENSG00000135547.9	ENSG00000274956.2	ENSG00000079102.16
7148	ENSG00000135549.15	ENSG00000274954.1	ENSG00000285972.1
7149	ENSG00000135569.4	ENSG00000274949.1	ENSG00000225934.2
7150	ENSG00000135577.4	ENSG00000274944.4	ENSG00000276956.1
7151	ENSG00000135587.9	ENSG00000274943.1	ENSG00000197008.9
7152	ENSG00000135596.18	ENSG00000274940.1	ENSG00000215704.9

7153	ENSG00000135597.18	ENSG00000274937.1	ENSG00000267717.1
7154	ENSG00000135604.9	ENSG00000274934.1	ENSG00000282917.1
7155	ENSG00000135605.13	ENSG00000274933.5	ENSG00000180182.11
7156	ENSG00000135617.4	ENSG00000274932.1	ENSG00000075218.19
7157	ENSG00000135622.13	ENSG00000274930.1	ENSG00000263503.1
7158	ENSG00000135624.16	ENSG00000274929.1	ENSG00000231231.5
7159	ENSG00000135625.7	ENSG00000274928.6	ENSG00000240925.1
7160	ENSG00000135631.16	ENSG00000274927.1	ENSG00000136813.14
7161	ENSG00000135632.12	ENSG00000274925.1	ENSG00000249031.1
7162	ENSG00000135636.14	ENSG00000274923.1	ENSG00000100802.15
7163	ENSG00000135637.13	ENSG00000274922.1	ENSG00000101294.17
7164	ENSG00000135638.13	ENSG00000274919.1	ENSG00000231550.1
7165	ENSG00000135643.5	ENSG00000274918.1	ENSG00000270136.6
7166	ENSG00000135655.16	ENSG00000274917.1	ENSG00000281106.4
7167	ENSG00000135677.11	ENSG00000274915.1	ENSG00000105472.13
7168	ENSG00000135678.12	ENSG00000274904.1	ENSG00000255120.5
7169	ENSG00000135679.24	ENSG00000274903.1	ENSG00000284999.1
7170	ENSG00000135686.13	ENSG00000274902.1	ENSG00000176896.8
7171	ENSG00000135697.10	ENSG00000274901.2	ENSG00000146049.1
7172	ENSG00000135698.10	ENSG00000274899.1	ENSG00000101849.16
7173	ENSG00000135702.14	ENSG00000274897.3	ENSG00000137573.14
7174	ENSG00000135709.12	ENSG00000274895.1	ENSG00000249906.1
7175	ENSG00000135720.13	ENSG00000274893.1	ENSG00000187979.4
7176	ENSG00000135722.9	ENSG00000274892.1	ENSG00000286015.1
7177	ENSG00000135723.14	ENSG00000274886.1	ENSG00000280053.1
7178	ENSG00000135736.6	ENSG00000274885.1	ENSG00000186842.4
7179	ENSG00000135740.17	ENSG00000274884.1	ENSG00000248943.1
7180	ENSG00000135744.8	ENSG00000274883.1	ENSG00000274892.1
7181	ENSG00000135747.11	ENSG00000274879.1	ENSG00000154997.9
7182	ENSG00000135749.19	ENSG00000274878.1	ENSG00000100053.10
7183	ENSG00000135750.14	ENSG00000274874.1	ENSG00000284197.1
7184	ENSG00000135763.10	ENSG00000274868.1	ENSG00000164451.13
7185	ENSG00000135766.8	ENSG00000274867.1	ENSG00000226621.1
7186	ENSG00000135773.13	ENSG00000274866.1	ENSG00000250195.1
7187	ENSG00000135775.14	ENSG00000274864.1	ENSG00000237338.1
7188	ENSG00000135776.5	ENSG00000274863.1	ENSG00000259750.1
7189	ENSG00000135778.12	ENSG00000274862.1	ENSG00000273132.1
7190	ENSG00000135801.9	ENSG00000274860.1	ENSG00000229414.2
7191	ENSG00000135821.18	ENSG00000274859.1	ENSG00000255767.1
7192	ENSG00000135823.14	ENSG00000274855.1	ENSG00000174576.10
7193	ENSG00000135824.12	ENSG00000274852.1	ENSG00000229321.1
7194	ENSG00000135828.11	ENSG00000274849.1	ENSG00000265043.1
7195	ENSG00000135829.17	ENSG00000274848.1	ENSG00000258001.1
7196	ENSG00000135835.12	ENSG00000274847.1	ENSG00000225222.2
7197	ENSG00000135837.16	ENSG00000274844.1	ENSG00000236053.1
7198	ENSG00000135838.13	ENSG00000274841.1	ENSG00000224142.1
7199	ENSG00000135842.17	ENSG00000274840.4	ENSG00000227560.1
7200	ENSG00000135845.10	ENSG00000274838.1	ENSG00000234066.1
7201	ENSG00000135862.6	ENSG00000274837.1	ENSG00000277900.1
7202	ENSG00000135870.11	ENSG00000274836.1	ENSG00000248881.1
7203	ENSG00000135898.10	ENSG00000274835.1	ENSG00000089472.16
7204	ENSG00000135899.17	ENSG00000274834.1	ENSG00000237036.4
7205	ENSG00000135900.4	ENSG00000274833.1	ENSG00000260430.1

7206	ENSG00000135902.10	ENSG00000274832.1	ENSG00000269900.3
7207	ENSG00000135903.19	ENSG00000274828.1	ENSG00000237674.1
7208	ENSG00000135905.19	ENSG00000274827.4	ENSG00000240747.7
7209	ENSG00000135912.11	ENSG00000274825.1	ENSG00000257611.1
7210	ENSG00000135913.11	ENSG00000274824.1	ENSG00000153002.12
7211	ENSG00000135914.6	ENSG00000274823.1	ENSG00000100399.16
7212	ENSG00000135916.16	ENSG00000274822.1	ENSG00000208892.1
7213	ENSG00000135917.15	ENSG00000274820.1	ENSG00000264781.1
7214	ENSG00000135919.13	ENSG00000274819.1	ENSG00000164729.7
7215	ENSG00000135924.15	ENSG00000274818.1	ENSG00000185522.9
7216	ENSG00000135925.9	ENSG00000274817.1	ENSG00000205927.5
7217	ENSG00000135926.15	ENSG00000274816.1	ENSG00000182310.14
7218	ENSG00000135929.9	ENSG00000274814.1	ENSG00000242628.5
7219	ENSG00000135930.14	ENSG00000274813.1	ENSG00000285619.1
7220	ENSG00000135931.17	ENSG00000274809.1	ENSG00000271848.1
7221	ENSG00000135932.11	ENSG00000274808.5	ENSG00000214192.3
7222	ENSG00000135940.6	ENSG00000274805.1	ENSG00000264655.1
7223	ENSG00000135945.10	ENSG00000274800.1	ENSG00000259545.2
7224	ENSG00000135951.15	ENSG00000274799.1	ENSG00000205502.4
7225	ENSG00000135953.11	ENSG00000274798.1	ENSG00000144406.18
7226	ENSG00000135956.8	ENSG00000274797.1	ENSG00000253952.1
7227	ENSG00000135960.10	ENSG00000274792.1	ENSG00000276645.1
7228	ENSG00000135966.13	ENSG00000274791.1	ENSG00000230097.2
7229	ENSG00000135968.20	ENSG00000274790.1	ENSG00000270354.1
7230	ENSG00000135972.9	ENSG00000274777.1	ENSG00000213522.4
7231	ENSG00000135973.2	ENSG00000274776.1	ENSG00000275812.1
7232	ENSG00000135974.10	ENSG00000274775.2	ENSG00000213176.4
7233	ENSG00000135976.19	ENSG00000274772.1	ENSG00000254369.6
7234	ENSG00000135999.12	ENSG00000274770.1	ENSG00000117616.18
7235	ENSG00000136002.18	ENSG00000274769.1	ENSG00000261431.1
7236	ENSG00000136003.15	ENSG00000274767.1	ENSG00000198857.3
7237	ENSG00000136010.14	ENSG00000274766.1	ENSG00000264520.1
7238	ENSG00000136011.15	ENSG00000274765.1	ENSG00000115226.10
7239	ENSG00000136014.12	ENSG00000274764.5	ENSG00000248785.1
7240	ENSG00000136021.18	ENSG00000274760.1	ENSG00000175886.10
7241	ENSG00000136026.14	ENSG00000274759.1	ENSG00000255471.1
7242	ENSG00000136040.9	ENSG00000274758.1	ENSG00000239831.1
7243	ENSG00000136044.12	ENSG00000274756.1	ENSG00000283464.1
7244	ENSG00000136045.12	ENSG00000274755.1	ENSG00000280242.1
7245	ENSG00000136048.14	ENSG00000274752.1	ENSG00000166492.9
7246	ENSG00000136051.14	ENSG00000274751.1	ENSG00000101138.11
7247	ENSG00000136052.9	ENSG00000274750.2	ENSG00000200057.1
7248	ENSG00000136059.14	ENSG00000274749.1	ENSG00000224215.1
7249	ENSG00000136068.14	ENSG00000274748.1	ENSG00000272321.1
7250	ENSG00000136098.17	ENSG00000274747.1	ENSG00000260394.2
7251	ENSG00000136099.13	ENSG00000274744.1	ENSG00000213979.3
7252	ENSG00000136100.14	ENSG00000274742.1	ENSG00000198049.7
7253	ENSG00000136104.20	ENSG00000274740.1	ENSG00000230371.1
7254	ENSG00000136108.15	ENSG00000274737.1	ENSG00000156509.14
7255	ENSG00000136110.13	ENSG00000274736.5	ENSG00000131711.15
7256	ENSG00000136111.13	ENSG00000274732.1	ENSG00000227481.1
7257	ENSG00000136114.17	ENSG00000274730.1	ENSG00000228554.1
7258	ENSG00000136122.16	ENSG00000274727.1	ENSG00000278455.1

7259	ENSG00000136141.15	ENSG00000274723.1	ENSG00000230667.5
7260	ENSG00000136143.15	ENSG00000274721.1	ENSG00000157470.12
7261	ENSG00000136144.12	ENSG00000274719.1	ENSG00000236394.2
7262	ENSG00000136146.14	ENSG00000274718.1	ENSG00000258667.1
7263	ENSG00000136147.18	ENSG00000274717.1	ENSG00000084754.12
7264	ENSG00000136149.6	ENSG00000274716.1	ENSG00000201518.1
7265	ENSG00000136152.15	ENSG00000274713.1	ENSG00000267573.1
7266	ENSG00000136153.20	ENSG00000274712.1	ENSG00000226465.2
7267	ENSG00000136155.17	ENSG00000274711.1	ENSG00000243254.3
7268	ENSG00000136156.14	ENSG00000274709.1	ENSG00000182512.5
7269	ENSG00000136158.12	ENSG00000274705.2	ENSG00000257905.1
7270	ENSG00000136159.4	ENSG00000274704.1	ENSG00000275930.1
7271	ENSG00000136160.16	ENSG00000274702.1	ENSG00000262967.1
7272	ENSG00000136161.12	ENSG00000274701.1	ENSG00000237436.1
7273	ENSG00000136167.14	ENSG00000274698.1	ENSG00000276375.1
7274	ENSG00000136169.16	ENSG00000274697.1	ENSG00000277051.1
7275	ENSG00000136193.17	ENSG00000274695.1	ENSG00000199585.1
7276	ENSG00000136197.12	ENSG00000274693.1	ENSG00000234800.2
7277	ENSG00000136205.17	ENSG00000274691.1	ENSG00000199730.1
7278	ENSG00000136206.4	ENSG00000274688.1	ENSG00000064692.19
7279	ENSG00000136213.10	ENSG00000274686.1	ENSG00000227256.1
7280	ENSG00000136231.14	ENSG00000274685.1	ENSG00000233081.1
7281	ENSG00000136235.16	ENSG00000274682.1	ENSG00000230231.1
7282	ENSG00000136237.18	ENSG00000274680.1	ENSG00000244130.1
7283	ENSG00000136238.18	ENSG00000274678.1	ENSG00000258597.3
7284	ENSG00000136240.10	ENSG00000274677.1	ENSG00000232767.1
7285	ENSG00000136243.17	ENSG00000274670.1	ENSG00000284393.1
7286	ENSG00000136244.12	ENSG00000274666.1	ENSG00000278964.1
7287	ENSG00000136247.14	ENSG00000274664.1	ENSG00000206127.10
7288	ENSG00000136250.11	ENSG00000274663.1	ENSG00000254444.1
7289	ENSG00000136261.15	ENSG00000274662.1	ENSG00000185269.12
7290	ENSG00000136267.13	ENSG00000274660.1	ENSG00000214106.8
7291	ENSG00000136270.14	ENSG00000274659.1	ENSG00000171169.9
7292	ENSG00000136271.10	ENSG00000274658.1	ENSG00000276664.1
7293	ENSG00000136273.13	ENSG00000274656.1	ENSG00000100867.14
7294	ENSG00000136274.9	ENSG00000274655.1	ENSG00000273846.1
7295	ENSG00000136275.10	ENSG00000274654.1	ENSG00000233932.6
7296	ENSG00000136279.20	ENSG00000274653.1	ENSG00000172283.10
7297	ENSG00000136280.16	ENSG00000274652.1	ENSG00000105976.15
7298	ENSG00000136286.16	ENSG00000274649.1	ENSG00000165507.8
7299	ENSG00000136295.15	ENSG00000274647.1	ENSG00000229771.2
7300	ENSG00000136297.14	ENSG00000274642.1	ENSG00000259236.1
7301	ENSG00000136305.11	ENSG00000274641.1	ENSG00000232234.3
7302	ENSG00000136315.4	ENSG00000274640.1	ENSG00000286123.1
7303	ENSG00000136319.12	ENSG00000274637.1	ENSG00000219699.2
7304	ENSG00000136327.6	ENSG00000274632.1	ENSG00000240767.3
7305	ENSG00000136352.17	ENSG00000274631.1	ENSG00000254400.1
7306	ENSG00000136367.14	ENSG00000274630.1	ENSG00000198358.4
7307	ENSG00000136371.10	ENSG00000274629.1	ENSG00000237249.1
7308	ENSG00000136378.15	ENSG00000274628.4	ENSG00000159761.15
7309	ENSG00000136379.12	ENSG00000274625.1	ENSG00000265296.1
7310	ENSG00000136381.13	ENSG00000274624.1	ENSG00000181616.9
7311	ENSG00000136383.6	ENSG00000274621.1	ENSG00000232298.2

7312	ENSG00000136404.15	ENSG00000274620.1	ENSG00000188856.6
7313	ENSG00000136425.13	ENSG00000274618.1	ENSG00000204379.10
7314	ENSG00000136436.14	ENSG00000274617.1	ENSG00000225918.1
7315	ENSG00000136444.10	ENSG00000274615.1	ENSG00000271749.1
7316	ENSG00000136448.12	ENSG00000274614.1	ENSG00000133710.15
7317	ENSG00000136449.14	ENSG00000274612.1	ENSG00000278654.1
7318	ENSG00000136450.13	ENSG00000274611.3	ENSG00000270987.1
7319	ENSG00000136451.9	ENSG00000274607.1	ENSG00000096696.14
7320	ENSG00000136457.10	ENSG00000274606.1	ENSG00000261588.1
7321	ENSG00000136463.8	ENSG00000274605.1	ENSG00000279098.1
7322	ENSG00000136478.7	ENSG00000274603.1	ENSG00000258177.1
7323	ENSG00000136485.15	ENSG00000274602.5	ENSG00000258864.1
7324	ENSG00000136487.18	ENSG00000274601.1	ENSG00000164761.9
7325	ENSG00000136488.15	ENSG00000274600.1	ENSG00000253525.1
7326	ENSG00000136490.9	ENSG00000274599.2	ENSG00000244307.3
7327	ENSG00000136492.8	ENSG00000274598.1	ENSG00000177519.4
7328	ENSG00000136504.12	ENSG00000274594.1	ENSG00000232824.1
7329	ENSG00000136514.3	ENSG00000274591.1	ENSG00000111783.12
7330	ENSG00000136518.17	ENSG00000274589.1	ENSG00000240474.3
7331	ENSG00000136521.13	ENSG00000274588.2	ENSG00000282965.1
7332	ENSG00000136522.13	ENSG00000274585.1	ENSG00000277806.1
7333	ENSG00000136527.18	ENSG00000274584.1	ENSG00000259144.2
7334	ENSG00000136531.16	ENSG00000274583.1	ENSG00000167600.14
7335	ENSG00000136535.15	ENSG00000274582.1	ENSG00000146090.16
7336	ENSG00000136536.15	ENSG00000274579.1	ENSG00000275426.1
7337	ENSG00000136541.14	ENSG00000274578.1	ENSG00000279070.2
7338	ENSG00000136542.9	ENSG00000274576.2	ENSG00000279590.1
7339	ENSG00000136546.15	ENSG00000274572.1	ENSG00000224409.1
7340	ENSG00000136560.13	ENSG00000274570.4	ENSG00000237821.1
7341	ENSG00000136573.14	ENSG00000274569.1	ENSG00000225053.1
7342	ENSG00000136574.17	ENSG00000274568.1	ENSG00000212153.1
7343	ENSG00000136603.14	ENSG00000274565.1	ENSG00000271524.1
7344	ENSG00000136628.18	ENSG00000274562.1	ENSG00000235937.1
7345	ENSG00000136630.13	ENSG00000274561.1	ENSG00000196979.1
7346	ENSG00000136631.14	ENSG00000274560.1	ENSG00000200320.1
7347	ENSG00000136634.6	ENSG00000274559.3	ENSG00000275915.1
7348	ENSG00000136636.13	ENSG00000274554.1	ENSG00000260282.1
7349	ENSG00000136643.12	ENSG00000274553.1	ENSG00000280346.1
7350	ENSG00000136682.14	ENSG00000274552.1	ENSG00000197421.9
7351	ENSG00000136688.11	ENSG00000274551.1	ENSG00000159403.17
7352	ENSG00000136689.18	ENSG00000274549.1	ENSG00000130300.9
7353	ENSG00000136694.8	ENSG00000274547.1	ENSG00000231654.1
7354	ENSG00000136695.14	ENSG00000274544.1	ENSG00000228237.5
7355	ENSG00000136696.10	ENSG00000274541.1	ENSG00000164118.13
7356	ENSG00000136697.12	ENSG00000274536.6	ENSG00000269469.1
7357	ENSG00000136698.8	ENSG00000274535.1	ENSG00000264421.1
7358	ENSG00000136699.19	ENSG00000274532.1	ENSG00000148488.17
7359	ENSG00000136709.12	ENSG00000274529.5	ENSG00000285218.1
7360	ENSG00000136710.10	ENSG00000274528.1	ENSG00000100852.13
7361	ENSG00000136715.18	ENSG00000274525.1	ENSG00000243095.1
7362	ENSG00000136717.15	ENSG00000274523.5	ENSG00000253696.2
7363	ENSG00000136718.9	ENSG00000274520.1	ENSG00000257286.1
7364	ENSG00000136720.7	ENSG00000274516.2	ENSG00000283483.1

7365	ENSG00000136731.12	ENSG00000274515.1	ENSG00000206531.10
7366	ENSG00000136732.16	ENSG00000274514.1	ENSG00000213424.9
7367	ENSG00000136738.15	ENSG00000274512.5	ENSG00000228133.2
7368	ENSG00000136750.13	ENSG00000274510.1	ENSG00000233993.1
7369	ENSG00000136754.17	ENSG00000274508.1	ENSG00000259385.1
7370	ENSG00000136758.18	ENSG00000274507.1	ENSG00000205111.8
7371	ENSG00000136770.11	ENSG00000274505.1	ENSG00000259185.1
7372	ENSG00000136783.10	ENSG00000274503.1	ENSG00000185610.6
7373	ENSG00000136802.11	ENSG00000274502.1	ENSG00000221837.5
7374	ENSG00000136807.13	ENSG00000274501.1	ENSG00000255104.8
7375	ENSG00000136810.13	ENSG00000274500.1	ENSG00000234685.1
7376	ENSG00000136811.16	ENSG00000274499.1	ENSG00000259604.5
7377	ENSG00000136813.14	ENSG00000274494.1	ENSG00000279589.1
7378	ENSG00000136816.16	ENSG00000274493.1	ENSG00000244381.1
7379	ENSG00000136819.15	ENSG00000274492.1	ENSG00000182103.5
7380	ENSG00000136824.19	ENSG00000274491.1	ENSG00000145002.12
7381	ENSG00000136826.15	ENSG00000274489.1	ENSG00000270099.1
7382	ENSG00000136827.12	ENSG00000274487.2	ENSG00000196712.17
7383	ENSG00000136828.19	ENSG00000274486.1	ENSG00000110887.8
7384	ENSG00000136830.12	ENSG00000274484.1	ENSG00000240970.1
7385	ENSG00000136834.3	ENSG00000274481.1	ENSG00000136939.1
7386	ENSG00000136839.1	ENSG00000274478.1	ENSG00000161270.19
7387	ENSG00000136840.19	ENSG00000274475.1	ENSG00000151233.11
7388	ENSG00000136842.14	ENSG00000274472.1	ENSG00000276778.1
7389	ENSG00000136848.17	ENSG00000274469.1	ENSG00000273982.1
7390	ENSG00000136854.21	ENSG00000274468.1	ENSG00000184785.6
7391	ENSG00000136856.17	ENSG00000274467.1	ENSG00000270835.2
7392	ENSG00000136859.10	ENSG00000274466.1	ENSG00000256053.7
7393	ENSG00000136861.18	ENSG00000274461.1	ENSG00000238103.4
7394	ENSG00000136866.13	ENSG00000274460.1	ENSG00000213231.13
7395	ENSG00000136867.11	ENSG00000274459.1	ENSG00000243592.1
7396	ENSG00000136868.11	ENSG00000274458.1	ENSG00000249375.7
7397	ENSG00000136869.15	ENSG00000274455.1	ENSG00000225762.1
7398	ENSG00000136870.10	ENSG00000274452.1	ENSG00000237176.4
7399	ENSG00000136872.19	ENSG00000274451.1	ENSG00000116044.16
7400	ENSG00000136874.11	ENSG00000274450.1	ENSG00000007372.22
7401	ENSG00000136875.12	ENSG00000274447.1	ENSG00000275467.1
7402	ENSG00000136877.14	ENSG00000274446.1	ENSG00000224273.2
7403	ENSG00000136878.13	ENSG00000274445.1	ENSG00000227845.2
7404	ENSG00000136881.11	ENSG00000274444.1	ENSG00000171431.3
7405	ENSG00000136883.14	ENSG00000274443.4	ENSG00000163026.12
7406	ENSG00000136888.7	ENSG00000274441.1	ENSG00000237115.2
7407	ENSG00000136891.14	ENSG00000274432.1	ENSG00000204287.14
7408	ENSG00000136895.19	ENSG00000274430.1	ENSG00000236444.4
7409	ENSG00000136897.8	ENSG00000274428.1	ENSG00000172379.21
7410	ENSG00000136908.17	ENSG00000274427.1	ENSG00000267773.1
7411	ENSG00000136918.7	ENSG00000274425.1	ENSG00000226012.1
7412	ENSG00000136925.15	ENSG00000274424.1	ENSG00000227431.5
7413	ENSG00000136928.7	ENSG00000274423.1	ENSG00000285766.1
7414	ENSG00000136929.13	ENSG00000274422.1	ENSG00000285707.1
7415	ENSG00000136930.13	ENSG00000274421.1	ENSG00000271022.1
7416	ENSG00000136931.10	ENSG00000274420.1	ENSG00000272320.1
7417	ENSG00000136932.13	ENSG00000274419.6	ENSG00000171931.12

7418	ENSG00000136933.16	ENSG00000274417.1	ENSG00000185070.11
7419	ENSG00000136935.13	ENSG00000274415.1	ENSG00000205795.4
7420	ENSG00000136936.10	ENSG00000274414.1	ENSG00000225420.1
7421	ENSG00000136937.13	ENSG00000274409.1	ENSG00000154143.2
7422	ENSG00000136938.9	ENSG00000274408.1	ENSG00000226251.5
7423	ENSG00000136939.1	ENSG00000274403.1	ENSG00000237649.8
7424	ENSG00000136940.13	ENSG00000274400.1	ENSG00000261293.1
7425	ENSG00000136942.15	ENSG00000274398.1	ENSG00000249193.1
7426	ENSG00000136943.11	ENSG00000274397.1	ENSG00000284233.1
7427	ENSG00000136944.17	ENSG00000274396.1	ENSG00000229292.1
7428	ENSG00000136950.13	ENSG00000274391.4	ENSG00000144355.15
7429	ENSG00000136960.12	ENSG00000274390.1	ENSG00000256804.1
7430	ENSG00000136982.6	ENSG00000274387.1	ENSG00000228886.1
7431	ENSG00000136986.10	ENSG00000274386.5	ENSG00000248302.3
7432	ENSG00000136997.18	ENSG00000274385.1	ENSG00000215771.2
7433	ENSG00000136999.5	ENSG00000274383.1	ENSG00000281097.1
7434	ENSG00000137033.11	ENSG00000274381.1	ENSG00000272407.1
7435	ENSG00000137038.8	ENSG00000274380.1	ENSG00000211739.4
7436	ENSG00000137040.9	ENSG00000274379.1	ENSG00000185607.5
7437	ENSG00000137054.16	ENSG00000274378.1	ENSG00000229163.2
7438	ENSG00000137055.15	ENSG00000274376.4	ENSG00000261548.1
7439	ENSG00000137070.17	ENSG00000274374.1	ENSG00000258558.1
7440	ENSG00000137073.21	ENSG00000274373.1	ENSG00000225724.1
7441	ENSG00000137074.18	ENSG00000274372.4	ENSG00000241456.1
7442	ENSG00000137075.18	ENSG00000274370.1	ENSG00000262413.1
7443	ENSG00000137076.21	ENSG00000274369.1	ENSG00000072315.3
7444	ENSG00000137077.8	ENSG00000274367.1	ENSG00000165682.14
7445	ENSG00000137078.9	ENSG00000274365.1	ENSG00000285955.1
7446	ENSG00000137080.4	ENSG00000274364.1	ENSG00000276612.3
7447	ENSG00000137090.12	ENSG00000274363.1	ENSG00000176125.5
7448	ENSG00000137094.14	ENSG00000274357.1	ENSG00000167182.15
7449	ENSG00000137098.13	ENSG00000274356.1	ENSG00000279370.1
7450	ENSG00000137100.16	ENSG00000274355.1	ENSG00000244722.1
7451	ENSG00000137101.12	ENSG00000274354.1	ENSG00000249463.1
7452	ENSG00000137103.20	ENSG00000274353.1	ENSG00000258782.3
7453	ENSG00000137106.18	ENSG00000274351.1	ENSG00000227848.1
7454	ENSG00000137124.8	ENSG00000274350.1	ENSG00000102890.15
7455	ENSG00000137133.11	ENSG00000274349.4	ENSG00000241597.2
7456	ENSG00000137135.18	ENSG00000274347.1	ENSG00000275491.1
7457	ENSG00000137142.5	ENSG00000274346.1	ENSG00000239726.3
7458	ENSG00000137145.20	ENSG00000274344.1	ENSG00000250210.5
7459	ENSG00000137154.12	ENSG00000274343.1	ENSG00000274428.1
7460	ENSG00000137161.16	ENSG00000274342.1	ENSG00000145451.13
7461	ENSG00000137166.14	ENSG00000274341.1	ENSG00000101438.4
7462	ENSG00000137168.8	ENSG00000274340.1	ENSG00000272554.1
7463	ENSG00000137171.15	ENSG00000274333.4	ENSG00000249395.2
7464	ENSG00000137177.20	ENSG00000274331.1	ENSG00000204713.11
7465	ENSG00000137185.12	ENSG00000274330.1	ENSG00000171989.5
7466	ENSG00000137193.14	ENSG00000274328.1	ENSG00000232554.8
7467	ENSG00000137198.9	ENSG00000274326.1	ENSG00000227854.1
7468	ENSG00000137200.13	ENSG00000274322.1	ENSG00000213557.4
7469	ENSG00000137203.12	ENSG00000274321.1	ENSG00000149571.12
7470	ENSG00000137204.14	ENSG00000274319.1	ENSG00000145506.13

7471	ENSG00000137207.12	ENSG00000274317.1	ENSG00000232675.7
7472	ENSG00000137210.13	ENSG00000274316.1	ENSG00000227238.1
7473	ENSG00000137216.19	ENSG00000274315.1	ENSG00000230023.2
7474	ENSG00000137218.10	ENSG00000274314.1	ENSG00000152931.7
7475	ENSG00000137221.14	ENSG00000274312.1	ENSG00000001617.12
7476	ENSG00000137225.13	ENSG00000274310.1	ENSG00000224715.1
7477	ENSG00000137251.16	ENSG00000274309.1	ENSG00000281641.2
7478	ENSG00000137252.9	ENSG00000274308.1	ENSG00000213295.3
7479	ENSG00000137261.14	ENSG00000274307.1	ENSG00000203729.8
7480	ENSG00000137265.15	ENSG00000274306.1	ENSG00000286143.1
7481	ENSG00000137266.14	ENSG00000274303.1	ENSG00000224114.1
7482	ENSG00000137267.6	ENSG00000274300.1	ENSG00000260422.1
7483	ENSG00000137269.15	ENSG00000274299.1	ENSG00000251583.1
7484	ENSG00000137270.11	ENSG00000274297.1	ENSG00000117174.11
7485	ENSG00000137273.5	ENSG00000274295.1	ENSG00000235994.4
7486	ENSG00000137274.13	ENSG00000274294.1	ENSG00000116857.17
7487	ENSG00000137275.14	ENSG00000274293.1	ENSG00000226153.1
7488	ENSG00000137285.10	ENSG00000274292.1	ENSG00000225380.4
7489	ENSG00000137288.10	ENSG00000274290.2	ENSG00000042753.11
7490	ENSG00000137309.19	ENSG00000274284.1	ENSG00000261026.1
7491	ENSG00000137310.12	ENSG00000274282.1	ENSG00000221475.1
7492	ENSG00000137312.15	ENSG00000274281.1	ENSG00000259546.1
7493	ENSG00000137331.12	ENSG00000274280.1	ENSG00000286176.1
7494	ENSG00000137337.15	ENSG00000274279.1	ENSG00000257097.1
7495	ENSG00000137338.5	ENSG00000274276.4	ENSG00000100505.13
7496	ENSG00000137343.18	ENSG00000274275.1	ENSG00000250948.1
7497	ENSG00000137364.5	ENSG00000274274.1	ENSG00000101938.14
7498	ENSG00000137392.9	ENSG00000274272.1	ENSG00000166573.5
7499	ENSG00000137393.9	ENSG00000274269.1	ENSG00000215183.5
7500	ENSG00000137404.14	ENSG00000274267.1	ENSG00000220748.2
7501	ENSG00000137409.19	ENSG00000274266.1	ENSG00000136449.14
7502	ENSG00000137411.17	ENSG00000274265.4	ENSG00000229324.1
7503	ENSG00000137413.16	ENSG00000274263.1	ENSG00000253536.1
7504	ENSG00000137414.6	ENSG00000274261.1	ENSG00000125551.18
7505	ENSG00000137434.11	ENSG00000274259.2	ENSG00000235028.3
7506	ENSG00000137440.4	ENSG00000274258.1	ENSG00000120498.13
7507	ENSG00000137441.8	ENSG00000274256.1	ENSG00000225674.1
7508	ENSG00000137449.15	ENSG00000274253.4	ENSG00000244158.1
7509	ENSG00000137460.8	ENSG00000274252.1	ENSG00000164841.5
7510	ENSG00000137462.7	ENSG00000274251.1	ENSG00000231402.1
7511	ENSG00000137463.5	ENSG00000274248.1	ENSG00000117650.13
7512	ENSG00000137473.17	ENSG00000274244.1	ENSG00000166926.8
7513	ENSG00000137474.21	ENSG00000274242.1	ENSG00000258011.2
7514	ENSG00000137478.15	ENSG00000274238.1	ENSG00000253663.1
7515	ENSG00000137486.17	ENSG00000274235.1	ENSG00000100593.18
7516	ENSG00000137491.14	ENSG00000274234.1	ENSG00000181109.3
7517	ENSG00000137492.8	ENSG00000274231.1	ENSG00000268650.3
7518	ENSG00000137494.13	ENSG00000274230.1	ENSG00000164674.15
7519	ENSG00000137496.17	ENSG00000274228.1	ENSG00000158639.12
7520	ENSG00000137497.17	ENSG00000274227.1	ENSG00000205809.9
7521	ENSG00000137500.9	ENSG00000274226.5	ENSG00000285648.1
7522	ENSG00000137501.17	ENSG00000274225.1	ENSG00000260107.1
7523	ENSG00000137502.10	ENSG00000274220.1	ENSG00000252042.1

7524	ENSG00000137504.13	ENSG00000274219.1	ENSG00000233186.2
7525	ENSG00000137507.11	ENSG00000274216.1	ENSG00000169894.18
7526	ENSG00000137509.11	ENSG00000274215.1	ENSG00000283982.1
7527	ENSG00000137513.10	ENSG00000274214.1	ENSG00000272043.1
7528	ENSG00000137522.18	ENSG00000274213.1	ENSG00000240764.3
7529	ENSG00000137547.8	ENSG00000274212.1	ENSG00000257004.1
7530	ENSG00000137558.9	ENSG00000274211.5	ENSG00000127083.7
7531	ENSG00000137561.4	ENSG00000274210.1	ENSG00000275834.1
7532	ENSG00000137563.12	ENSG00000274209.4	ENSG00000261418.1
7533	ENSG00000137571.11	ENSG00000274206.1	ENSG00000214070.3
7534	ENSG00000137573.14	ENSG00000274204.1	ENSG00000249784.1
7535	ENSG00000137574.10	ENSG00000274202.1	ENSG00000179698.13
7536	ENSG00000137575.12	ENSG00000274199.1	ENSG00000254649.1
7537	ENSG00000137601.16	ENSG00000274198.1	ENSG00000143333.7
7538	ENSG00000137628.17	ENSG00000274197.1	ENSG00000069020.18
7539	ENSG00000137634.9	ENSG00000274196.5	ENSG00000271736.1
7540	ENSG00000137642.13	ENSG00000274191.1	ENSG00000248936.1
7541	ENSG00000137648.17	ENSG00000274186.1	ENSG00000177666.17
7542	ENSG00000137656.12	ENSG00000274184.1	ENSG00000226913.1
7543	ENSG00000137672.13	ENSG00000274183.1	ENSG00000271180.1
7544	ENSG00000137673.9	ENSG00000274181.1	ENSG00000260877.2
7545	ENSG00000137674.4	ENSG00000274180.1	ENSG00000285891.1
7546	ENSG00000137675.4	ENSG00000274178.2	ENSG00000280466.1
7547	ENSG00000137691.13	ENSG00000274177.1	ENSG00000282012.1
7548	ENSG00000137692.12	ENSG00000274175.1	ENSG00000281593.1
7549	ENSG00000137693.14	ENSG00000274173.1	ENSG00000255299.5
7550	ENSG00000137699.17	ENSG00000274172.1	ENSG00000266066.1
7551	ENSG00000137700.18	ENSG00000274170.1	ENSG00000278572.1
7552	ENSG00000137707.13	ENSG00000274168.1	ENSG00000251434.1
7553	ENSG00000137709.10	ENSG00000274167.4	ENSG00000229926.1
7554	ENSG00000137710.15	ENSG00000274164.1	ENSG00000253220.2
7555	ENSG00000137713.15	ENSG00000274162.1	ENSG00000254572.2
7556	ENSG00000137714.3	ENSG00000274159.1	ENSG00000265460.6
7557	ENSG00000137720.8	ENSG00000274157.1	ENSG00000110375.3
7558	ENSG00000137726.17	ENSG00000274156.1	ENSG00000255647.3
7559	ENSG00000137727.12	ENSG00000274154.1	ENSG00000254708.1
7560	ENSG00000137731.14	ENSG00000274150.1	ENSG00000254164.1
7561	ENSG00000137745.12	ENSG00000274149.1	ENSG00000178997.11
7562	ENSG00000137747.16	ENSG00000274139.1	ENSG00000203307.2
7563	ENSG00000137752.24	ENSG00000274135.1	ENSG00000203435.2
7564	ENSG00000137757.11	ENSG00000274134.1	ENSG00000267156.1
7565	ENSG00000137760.14	ENSG00000274133.1	ENSG00000272667.1
7566	ENSG00000137764.20	ENSG00000274128.1	ENSG00000184254.17
7567	ENSG00000137766.17	ENSG00000274127.1	ENSG00000266282.1
7568	ENSG00000137767.14	ENSG00000274124.1	ENSG00000255035.2
7569	ENSG00000137770.14	ENSG00000274121.1	ENSG00000272371.1
7570	ENSG00000137776.17	ENSG00000274118.1	ENSG00000267159.2
7571	ENSG00000137801.10	ENSG00000274116.1	ENSG00000254213.2
7572	ENSG00000137802.14	ENSG00000274115.1	ENSG00000110446.11
7573	ENSG00000137804.13	ENSG00000274111.1	ENSG00000241933.1
7574	ENSG00000137806.9	ENSG00000274105.1	ENSG00000273484.1
7575	ENSG00000137807.15	ENSG00000274104.1	ENSG00000233859.2
7576	ENSG00000137808.12	ENSG00000274102.2	ENSG00000240216.7

7577	ENSG00000137809.17	ENSG00000274099.1	ENSG00000250711.1
7578	ENSG00000137812.19	ENSG00000274098.1	ENSG00000249534.1
7579	ENSG00000137814.11	ENSG00000274097.1	ENSG00000278778.1
7580	ENSG00000137815.14	ENSG00000274093.1	ENSG00000169213.7
7581	ENSG00000137817.17	ENSG00000274092.1	ENSG00000260036.1
7582	ENSG00000137818.12	ENSG00000274091.1	ENSG00000261729.1
7583	ENSG00000137819.13	ENSG00000274090.1	ENSG00000118689.15
7584	ENSG00000137821.11	ENSG00000274086.1	ENSG00000218565.2
7585	ENSG00000137822.12	ENSG00000274080.1	ENSG00000214190.2
7586	ENSG00000137824.16	ENSG00000274079.1	ENSG00000167619.12
7587	ENSG00000137825.11	ENSG00000274076.1	ENSG00000278276.1
7588	ENSG00000137831.15	ENSG00000274075.1	ENSG00000155959.11
7589	ENSG00000137834.15	ENSG00000274072.1	ENSG00000203446.2
7590	ENSG00000137841.12	ENSG00000274070.1	ENSG00000257663.1
7591	ENSG00000137842.7	ENSG00000274068.1	ENSG00000270690.1
7592	ENSG00000137843.11	ENSG00000274067.1	ENSG00000284770.2
7593	ENSG00000137845.15	ENSG00000274066.1	ENSG00000248245.1
7594	ENSG00000137857.17	ENSG00000274064.1	ENSG00000249894.1
7595	ENSG00000137860.12	ENSG00000274062.1	ENSG00000234770.1
7596	ENSG00000137868.19	ENSG00000274060.1	ENSG00000268983.1
7597	ENSG00000137869.14	ENSG00000274059.1	ENSG00000205704.6
7598	ENSG00000137871.20	ENSG00000274056.1	ENSG00000264167.1
7599	ENSG00000137872.16	ENSG00000274055.1	ENSG00000196230.13
7600	ENSG00000137875.4	ENSG00000274054.1	ENSG00000284977.1
7601	ENSG00000137876.10	ENSG00000274052.2	ENSG00000228014.1
7602	ENSG00000137877.10	ENSG00000274051.1	ENSG00000276842.1
7603	ENSG00000137878.17	ENSG00000274049.4	ENSG00000118137.9
7604	ENSG00000137880.6	ENSG00000274046.1	ENSG00000171819.5
7605	ENSG00000137936.18	ENSG00000274044.4	ENSG00000008869.12
7606	ENSG00000137942.16	ENSG00000274038.1	ENSG00000260615.1
7607	ENSG00000137944.18	ENSG00000274034.1	ENSG00000260372.6
7608	ENSG00000137947.12	ENSG00000274033.1	ENSG00000215088.3
7609	ENSG00000137948.18	ENSG00000274031.1	ENSG00000253505.2
7610	ENSG00000137955.16	ENSG00000274029.1	ENSG00000199202.1
7611	ENSG00000137959.16	ENSG00000274028.1	ENSG00000239351.1
7612	ENSG00000137960.6	ENSG00000274026.2	ENSG00000237686.6
7613	ENSG00000137962.13	ENSG00000274025.1	ENSG00000270469.1
7614	ENSG00000137965.11	ENSG00000274024.1	ENSG00000253993.1
7615	ENSG00000137968.16	ENSG00000274023.1	ENSG00000139835.13
7616	ENSG00000137970.7	ENSG00000274022.1	ENSG00000266891.1
7617	ENSG00000137975.8	ENSG00000274021.1	ENSG00000214255.5
7618	ENSG00000137976.7	ENSG00000274019.1	ENSG00000285906.1
7619	ENSG00000137992.14	ENSG00000274017.1	ENSG00000267890.1
7620	ENSG00000137996.12	ENSG00000274015.1	ENSG00000285106.1
7621	ENSG00000138002.15	ENSG00000274012.1	ENSG00000267577.1
7622	ENSG00000138018.18	ENSG00000274011.1	ENSG00000214759.3
7623	ENSG00000138028.16	ENSG00000274010.1	ENSG00000213443.2
7624	ENSG00000138029.14	ENSG00000274008.1	ENSG00000257763.1
7625	ENSG00000138030.13	ENSG00000274006.1	ENSG00000231073.1
7626	ENSG00000138031.14	ENSG00000274004.1	ENSG00000108602.18
7627	ENSG00000138032.21	ENSG00000274002.1	ENSG00000204054.13
7628	ENSG00000138035.15	ENSG00000274001.1	ENSG00000181323.8
7629	ENSG00000138036.18	ENSG00000273999.1	ENSG00000279883.1

7630	ENSG00000138039.15	ENSG00000273998.1	ENSG00000158714.11
7631	ENSG00000138050.15	ENSG00000273997.1	ENSG00000267576.1
7632	ENSG00000138061.12	ENSG00000273994.1	ENSG00000163898.10
7633	ENSG00000138068.10	ENSG00000273989.1	ENSG00000143398.20
7634	ENSG00000138069.18	ENSG00000273988.1	ENSG00000200890.1
7635	ENSG00000138071.13	ENSG00000273987.1	ENSG00000260776.5
7636	ENSG00000138073.14	ENSG00000273986.1	ENSG00000196972.9
7637	ENSG00000138074.15	ENSG00000273983.1	ENSG00000225043.1
7638	ENSG00000138075.12	ENSG00000273982.1	ENSG00000234229.8
7639	ENSG00000138078.15	ENSG00000273981.1	ENSG00000272970.1
7640	ENSG00000138079.13	ENSG00000273980.1	ENSG00000198523.6
7641	ENSG00000138080.13	ENSG00000273979.1	ENSG00000260792.1
7642	ENSG00000138081.20	ENSG00000273978.1	ENSG00000157554.19
7643	ENSG00000138083.5	ENSG00000273976.1	ENSG00000205622.10
7644	ENSG00000138085.16	ENSG00000273975.1	ENSG00000153029.14
7645	ENSG00000138092.11	ENSG00000273974.1	ENSG00000186732.14
7646	ENSG00000138095.19	ENSG00000273973.1	ENSG00000219361.1
7647	ENSG00000138100.13	ENSG00000273972.1	ENSG00000206647.1
7648	ENSG00000138101.18	ENSG00000273971.1	ENSG00000173213.9
7649	ENSG00000138107.13	ENSG00000273966.1	ENSG00000285522.1
7650	ENSG00000138109.11	ENSG00000273965.1	ENSG00000137098.13
7651	ENSG00000138111.14	ENSG00000273964.1	ENSG00000224025.2
7652	ENSG00000138115.14	ENSG00000273963.1	ENSG00000234142.1
7653	ENSG00000138119.17	ENSG00000273962.1	ENSG00000226138.4
7654	ENSG00000138131.4	ENSG00000273961.1	ENSG00000262188.1
7655	ENSG00000138134.12	ENSG00000273958.1	ENSG00000084731.15
7656	ENSG00000138135.6	ENSG00000273957.1	ENSG00000200834.1
7657	ENSG00000138136.6	ENSG00000273956.1	ENSG00000258262.1
7658	ENSG00000138138.13	ENSG00000273951.1	ENSG00000267009.6
7659	ENSG00000138152.8	ENSG00000273950.1	ENSG00000261523.1
7660	ENSG00000138160.6	ENSG00000273948.1	ENSG00000134627.12
7661	ENSG00000138161.14	ENSG00000273946.1	ENSG00000226233.1
7662	ENSG00000138162.19	ENSG00000273945.1	ENSG00000207475.1
7663	ENSG00000138166.6	ENSG00000273942.1	ENSG00000254270.1
7664	ENSG00000138172.11	ENSG00000273940.1	ENSG00000090372.15
7665	ENSG00000138175.9	ENSG00000273937.1	ENSG00000281128.1
7666	ENSG00000138180.15	ENSG00000273933.1	ENSG00000214745.2
7667	ENSG00000138182.14	ENSG00000273932.1	ENSG00000100985.7
7668	ENSG00000138185.20	ENSG00000273927.1	ENSG00000278685.4
7669	ENSG00000138190.16	ENSG00000273925.1	ENSG00000271882.1
7670	ENSG00000138193.15	ENSG00000273923.1	ENSG00000242661.1
7671	ENSG00000138207.14	ENSG00000273920.1	ENSG00000228411.1
7672	ENSG00000138231.12	ENSG00000273919.1	ENSG00000064419.13
7673	ENSG00000138246.17	ENSG00000273913.1	ENSG00000129255.16
7674	ENSG00000138271.5	ENSG00000273912.1	ENSG00000228115.1
7675	ENSG00000138279.16	ENSG00000273908.1	ENSG00000236780.6
7676	ENSG00000138286.14	ENSG00000273907.1	ENSG00000216639.1
7677	ENSG00000138303.17	ENSG00000273906.1	ENSG00000279571.3
7678	ENSG00000138308.5	ENSG00000273904.1	ENSG00000224525.2
7679	ENSG00000138311.17	ENSG00000273901.1	ENSG00000244218.3
7680	ENSG00000138315.13	ENSG00000273900.1	ENSG00000255526.6
7681	ENSG00000138316.11	ENSG00000273899.5	ENSG00000270665.1
7682	ENSG00000138326.19	ENSG00000273898.1	ENSG00000233033.1

7683	ENSG00000138336.9	ENSG00000273897.1	ENSG00000257568.1
7684	ENSG00000138346.15	ENSG00000273894.1	ENSG00000285444.1
7685	ENSG00000138347.15	ENSG00000273893.1	ENSG00000258800.1
7686	ENSG00000138356.14	ENSG00000273891.1	ENSG00000232517.2
7687	ENSG00000138363.15	ENSG00000273890.1	ENSG00000127780.3
7688	ENSG00000138375.12	ENSG00000273888.1	ENSG00000241434.1
7689	ENSG00000138376.11	ENSG00000273886.1	ENSG00000239524.2
7690	ENSG00000138378.19	ENSG00000273885.1	ENSG00000280222.1
7691	ENSG00000138379.4	ENSG00000273882.1	ENSG00000232087.1
7692	ENSG00000138380.18	ENSG00000273877.4	ENSG00000146278.10
7693	ENSG00000138381.10	ENSG00000273876.1	ENSG00000167311.13
7694	ENSG00000138382.15	ENSG00000273874.1	ENSG00000245937.7
7695	ENSG00000138385.16	ENSG00000273872.1	ENSG00000256928.1
7696	ENSG00000138386.17	ENSG00000273870.1	ENSG00000201428.1
7697	ENSG00000138395.15	ENSG00000273866.1	ENSG00000238085.1
7698	ENSG00000138398.16	ENSG00000273863.1	ENSG00000230596.3
7699	ENSG00000138399.18	ENSG00000273860.1	ENSG00000255409.1
7700	ENSG00000138400.13	ENSG00000273858.1	ENSG00000104313.19
7701	ENSG00000138411.13	ENSG00000273855.1	ENSG00000286189.1
7702	ENSG00000138413.13	ENSG00000273853.1	ENSG00000178977.3
7703	ENSG00000138430.16	ENSG00000273851.1	ENSG00000278236.1
7704	ENSG00000138433.16	ENSG00000273849.1	ENSG00000183625.15
7705	ENSG00000138434.17	ENSG00000273846.1	ENSG00000130649.10
7706	ENSG00000138435.15	ENSG00000273844.1	ENSG00000233339.1
7707	ENSG00000138439.12	ENSG00000273843.1	ENSG00000229817.1
7708	ENSG00000138442.9	ENSG00000273841.5	ENSG00000104517.13
7709	ENSG00000138443.16	ENSG00000273840.1	ENSG00000135362.14
7710	ENSG00000138448.12	ENSG00000273838.1	ENSG00000236680.1
7711	ENSG00000138449.10	ENSG00000273837.1	ENSG00000255815.3
7712	ENSG00000138459.9	ENSG00000273836.1	ENSG00000228217.1
7713	ENSG00000138463.9	ENSG00000273835.1	ENSG00000283945.1
7714	ENSG00000138468.16	ENSG00000273830.1	ENSG00000231333.2
7715	ENSG00000138472.11	ENSG00000273829.1	ENSG00000226210.3
7716	ENSG00000138483.2	ENSG00000273828.2	ENSG00000228643.1
7717	ENSG00000138495.6	ENSG00000273825.1	ENSG00000256045.2
7718	ENSG00000138496.16	ENSG00000273824.1	ENSG00000096070.19
7719	ENSG00000138587.6	ENSG00000273821.1	ENSG00000177363.5
7720	ENSG00000138592.14	ENSG00000273820.1	ENSG00000279020.1
7721	ENSG00000138593.9	ENSG00000273819.1	ENSG00000274340.1
7722	ENSG00000138594.14	ENSG00000273818.1	ENSG00000259050.2
7723	ENSG00000138600.10	ENSG00000273816.1	ENSG00000231967.1
7724	ENSG00000138604.10	ENSG00000273813.1	ENSG00000168490.14
7725	ENSG00000138606.19	ENSG00000273812.2	ENSG00000240870.2
7726	ENSG00000138613.14	ENSG00000273806.1	ENSG00000276531.1
7727	ENSG00000138614.15	ENSG00000273805.1	ENSG00000240125.1
7728	ENSG00000138615.6	ENSG00000273804.1	ENSG00000144550.13
7729	ENSG00000138617.15	ENSG00000273802.2	ENSG00000182575.7
7730	ENSG00000138621.12	ENSG00000273800.1	ENSG00000273819.1
7731	ENSG00000138622.4	ENSG00000273797.1	ENSG00000236229.1
7732	ENSG00000138623.10	ENSG00000273796.1	ENSG00000111445.14
7733	ENSG00000138629.16	ENSG00000273792.1	ENSG00000139292.13
7734	ENSG00000138639.18	ENSG00000273791.1	ENSG00000250634.5
7735	ENSG00000138640.15	ENSG00000273788.1	ENSG00000185306.12

7736	ENSG00000138641.17	ENSG00000273786.1	ENSG00000265801.1
7737	ENSG00000138642.14	ENSG00000273784.4	ENSG00000252916.1
7738	ENSG00000138646.9	ENSG00000273783.1	ENSG00000170745.12
7739	ENSG00000138650.9	ENSG00000273782.1	ENSG00000224099.1
7740	ENSG00000138653.9	ENSG00000273778.1	ENSG00000254756.1
7741	ENSG00000138658.15	ENSG00000273777.4	ENSG00000118113.12
7742	ENSG00000138660.11	ENSG00000273776.1	ENSG00000179403.12
7743	ENSG00000138663.9	ENSG00000273773.1	ENSG00000237373.1
7744	ENSG00000138668.19	ENSG00000273771.1	ENSG00000253270.1
7745	ENSG00000138669.9	ENSG00000273769.5	ENSG00000145794.17
7746	ENSG00000138670.17	ENSG00000273768.1	ENSG00000254538.1
7747	ENSG00000138674.17	ENSG00000273767.1	ENSG00000234019.1
7748	ENSG00000138675.16	ENSG00000273765.1	ENSG00000213650.3
7749	ENSG00000138678.11	ENSG00000273763.2	ENSG00000135100.17
7750	ENSG00000138684.8	ENSG00000273762.1	ENSG00000187762.5
7751	ENSG00000138685.15	ENSG00000273760.1	ENSG00000269136.1
7752	ENSG00000138686.9	ENSG00000273759.1	ENSG00000134207.16
7753	ENSG00000138688.15	ENSG00000273758.1	ENSG00000133475.17
7754	ENSG00000138696.10	ENSG00000273756.4	ENSG00000268434.5
7755	ENSG00000138698.14	ENSG00000273750.1	ENSG00000226912.1
7756	ENSG00000138709.19	ENSG00000273749.5	ENSG00000136938.9
7757	ENSG00000138722.10	ENSG00000273748.1	ENSG00000150783.9
7758	ENSG00000138735.16	ENSG00000273747.1	ENSG00000119321.9
7759	ENSG00000138738.10	ENSG00000273746.1	ENSG00000116675.16
7760	ENSG00000138741.11	ENSG00000273745.1	ENSG00000225486.1
7761	ENSG00000138744.15	ENSG00000273744.1	ENSG00000244124.1
7762	ENSG00000138750.15	ENSG00000273742.1	ENSG00000280780.2
7763	ENSG00000138755.5	ENSG00000273739.1	ENSG00000151575.14
7764	ENSG00000138756.17	ENSG00000273736.1	ENSG00000262708.1
7765	ENSG00000138757.14	ENSG00000273734.1	ENSG00000259479.6
7766	ENSG00000138758.11	ENSG00000273733.1	ENSG00000188883.4
7767	ENSG00000138759.19	ENSG00000273732.1	ENSG00000232172.1
7768	ENSG00000138760.10	ENSG00000273731.1	ENSG00000223808.1
7769	ENSG00000138764.15	ENSG00000273730.1	ENSG00000106025.8
7770	ENSG00000138767.13	ENSG00000273729.1	ENSG00000228470.1
7771	ENSG00000138768.14	ENSG00000273727.1	ENSG00000213950.2
7772	ENSG00000138769.11	ENSG00000273725.1	ENSG00000229842.2
7773	ENSG00000138771.16	ENSG00000273724.1	ENSG00000283541.1
7774	ENSG00000138772.13	ENSG00000273723.1	ENSG00000171489.10
7775	ENSG00000138777.20	ENSG00000273721.1	ENSG00000215311.3
7776	ENSG00000138778.11	ENSG00000273720.1	ENSG00000237294.1
7777	ENSG00000138780.14	ENSG00000273719.1	ENSG00000112041.13
7778	ENSG00000138785.15	ENSG00000273717.1	ENSG00000121892.15
7779	ENSG00000138792.10	ENSG00000273716.2	ENSG00000256452.1
7780	ENSG00000138794.10	ENSG00000273712.1	ENSG00000252965.1
7781	ENSG00000138795.10	ENSG00000273711.2	ENSG00000167195.7
7782	ENSG00000138796.16	ENSG00000273710.1	ENSG00000261192.1
7783	ENSG00000138798.12	ENSG00000273709.1	ENSG00000241607.1
7784	ENSG00000138801.9	ENSG00000273706.4	ENSG00000148688.13
7785	ENSG00000138802.11	ENSG00000273704.1	ENSG00000201185.1
7786	ENSG00000138813.10	ENSG00000273703.1	ENSG00000280339.1
7787	ENSG00000138814.17	ENSG00000273702.1	ENSG00000258949.1
7788	ENSG00000138821.13	ENSG00000273701.1	ENSG00000249244.1

7789	ENSG00000138823.13	ENSG00000273700.1	ENSG00000189037.7
7790	ENSG00000138829.12	ENSG00000273698.1	ENSG00000230718.1
7791	ENSG00000138834.12	ENSG00000273696.4	ENSG00000286090.1
7792	ENSG00000138835.22	ENSG00000273694.1	ENSG00000153574.9
7793	ENSG00000138867.16	ENSG00000273693.1	ENSG00000235545.1
7794	ENSG00000138892.11	ENSG00000273692.1	ENSG00000232431.3
7795	ENSG00000138942.16	ENSG00000273691.1	ENSG00000273717.1
7796	ENSG00000138944.8	ENSG00000273687.1	ENSG00000215016.2
7797	ENSG00000138964.17	ENSG00000273682.1	ENSG00000205420.11
7798	ENSG00000139044.11	ENSG00000273680.1	ENSG00000253886.1
7799	ENSG00000139053.3	ENSG00000273679.1	ENSG00000237596.6
7800	ENSG00000139055.7	ENSG00000273677.1	ENSG00000168453.15
7801	ENSG00000139083.10	ENSG00000273675.1	ENSG00000268707.1
7802	ENSG00000139112.11	ENSG00000273674.4	ENSG00000223440.1
7803	ENSG00000139116.18	ENSG00000273669.1	ENSG00000039523.20
7804	ENSG00000139117.14	ENSG00000273668.1	ENSG00000223544.1
7805	ENSG00000139131.12	ENSG00000273664.1	ENSG00000214280.3
7806	ENSG00000139132.14	ENSG00000273659.1	ENSG00000250582.1
7807	ENSG00000139133.7	ENSG00000273658.1	ENSG00000204933.3
7808	ENSG00000139144.9	ENSG00000273657.1	ENSG00000232053.6
7809	ENSG00000139146.14	ENSG00000273654.1	ENSG00000171815.5
7810	ENSG00000139151.15	ENSG00000273650.1	ENSG00000235787.1
7811	ENSG00000139154.15	ENSG00000273648.1	ENSG00000147408.14
7812	ENSG00000139155.8	ENSG00000273644.1	ENSG00000007933.13
7813	ENSG00000139160.13	ENSG00000273643.1	ENSG00000174132.9
7814	ENSG00000139163.15	ENSG00000273640.1	ENSG00000266968.2
7815	ENSG00000139168.8	ENSG00000273639.6	ENSG00000213269.2
7816	ENSG00000139173.10	ENSG00000273637.1	ENSG00000211693.2
7817	ENSG00000139174.12	ENSG00000273634.1	ENSG00000273413.1
7818	ENSG00000139178.11	ENSG00000273629.1	ENSG00000232282.1
7819	ENSG00000139180.11	ENSG00000273628.1	ENSG00000186163.9
7820	ENSG00000139182.14	ENSG00000273627.1	ENSG00000204616.11
7821	ENSG00000139187.10	ENSG00000273623.1	ENSG00000256683.7
7822	ENSG00000139190.16	ENSG00000273621.1	ENSG00000240935.6
7823	ENSG00000139192.12	ENSG00000273619.1	ENSG00000202025.1
7824	ENSG00000139193.3	ENSG00000273618.1	ENSG00000237971.1
7825	ENSG00000139194.8	ENSG00000273614.1	ENSG00000180745.5
7826	ENSG00000139197.10	ENSG00000273613.1	ENSG00000285750.1
7827	ENSG00000139200.13	ENSG00000273612.1	ENSG00000257691.2
7828	ENSG00000139209.16	ENSG00000273611.5	ENSG00000214770.3
7829	ENSG00000139211.6	ENSG00000273610.1	ENSG00000175325.2
7830	ENSG00000139218.18	ENSG00000273609.1	ENSG00000279686.1
7831	ENSG00000139219.19	ENSG00000273606.1	ENSG00000092377.14
7832	ENSG00000139220.16	ENSG00000273604.1	ENSG00000147655.11
7833	ENSG00000139223.2	ENSG00000273600.1	ENSG00000273301.1
7834	ENSG00000139233.7	ENSG00000273599.1	ENSG00000256824.1
7835	ENSG00000139239.7	ENSG00000273598.1	ENSG00000197921.6
7836	ENSG00000139263.11	ENSG00000273597.1	ENSG00000231981.3
7837	ENSG00000139266.6	ENSG00000273596.1	ENSG00000119919.11
7838	ENSG00000139269.3	ENSG00000273595.1	ENSG00000213194.3
7839	ENSG00000139278.10	ENSG00000273593.1	ENSG00000269982.1
7840	ENSG00000139287.13	ENSG00000273591.1	ENSG00000116833.14
7841	ENSG00000139289.13	ENSG00000273590.4	ENSG00000131747.15

7842	ENSG00000139291.13	ENSG00000273589.1	ENSG00000231093.1
7843	ENSG00000139292.13	ENSG00000273588.1	ENSG00000241155.1
7844	ENSG00000139304.14	ENSG00000273587.1	ENSG00000261490.1
7845	ENSG00000139318.8	ENSG00000273586.2	ENSG00000233388.2
7846	ENSG00000139323.14	ENSG00000273585.1	ENSG00000163719.19
7847	ENSG00000139324.12	ENSG00000273584.1	ENSG00000265369.3
7848	ENSG00000139329.5	ENSG00000273582.1	ENSG00000242296.2
7849	ENSG00000139330.5	ENSG00000273580.1	ENSG00000215515.2
7850	ENSG00000139343.10	ENSG00000273576.1	ENSG00000257639.1
7851	ENSG00000139344.8	ENSG00000273573.1	ENSG00000255364.1
7852	ENSG00000139350.11	ENSG00000273571.1	ENSG00000086289.12
7853	ENSG00000139351.15	ENSG00000273570.1	ENSG00000230630.5
7854	ENSG00000139352.4	ENSG00000273569.1	ENSG00000285079.1
7855	ENSG00000139354.11	ENSG00000273568.1	ENSG00000213174.3
7856	ENSG00000139364.10	ENSG00000273567.1	ENSG00000106819.12
7857	ENSG00000139370.12	ENSG00000273566.1	ENSG00000270301.1
7858	ENSG00000139372.15	ENSG00000273565.1	ENSG00000267927.1
7859	ENSG00000139405.16	ENSG00000273559.5	ENSG00000253355.1
7860	ENSG00000139410.15	ENSG00000273558.1	ENSG00000250057.1
7861	ENSG00000139428.12	ENSG00000273557.1	ENSG00000267420.1
7862	ENSG00000139433.10	ENSG00000273555.1	ENSG00000253833.1
7863	ENSG00000139436.21	ENSG00000273554.4	ENSG00000218996.1
7864	ENSG00000139437.18	ENSG00000273553.1	ENSG00000165029.16
7865	ENSG00000139438.5	ENSG00000273552.1	ENSG00000093100.13
7866	ENSG00000139445.18	ENSG00000273551.1	ENSG00000173976.15
7867	ENSG00000139496.16	ENSG00000273550.1	ENSG00000282697.1
7868	ENSG00000139505.11	ENSG00000273549.1	ENSG00000260342.2
7869	ENSG00000139508.14	ENSG00000273544.1	ENSG00000241169.1
7870	ENSG00000139514.13	ENSG00000273542.1	ENSG00000107159.13
7871	ENSG00000139515.6	ENSG00000273541.1	ENSG00000135248.15
7872	ENSG00000139517.9	ENSG00000273540.4	ENSG00000181786.4
7873	ENSG00000139531.13	ENSG00000273537.1	ENSG00000125998.8
7874	ENSG00000139537.11	ENSG00000273536.1	ENSG00000177803.7
7875	ENSG00000139540.12	ENSG00000273532.1	ENSG00000275676.1
7876	ENSG00000139546.10	ENSG00000273524.1	ENSG00000261668.1
7877	ENSG00000139547.7	ENSG00000273523.1	ENSG00000213368.3
7878	ENSG00000139549.4	ENSG00000273521.1	ENSG00000215785.2
7879	ENSG00000139567.12	ENSG00000273520.5	ENSG00000271826.5
7880	ENSG00000139572.4	ENSG00000273516.1	ENSG00000257894.2
7881	ENSG00000139574.8	ENSG00000273515.1	ENSG00000274827.4
7882	ENSG00000139579.13	ENSG00000273513.1	ENSG00000261396.1
7883	ENSG00000139597.18	ENSG00000273512.1	ENSG00000229696.1
7884	ENSG00000139610.1	ENSG00000273509.1	ENSG00000280169.1
7885	ENSG00000139613.12	ENSG00000273507.4	ENSG00000160973.7
7886	ENSG00000139618.14	ENSG00000273500.1	ENSG00000223576.2
7887	ENSG00000139620.12	ENSG00000273499.1	ENSG00000119660.4
7888	ENSG00000139624.12	ENSG00000273497.1	ENSG00000253623.1
7889	ENSG00000139625.13	ENSG00000273496.1	ENSG00000238405.1
7890	ENSG00000139626.16	ENSG00000273493.1	ENSG00000109906.13
7891	ENSG00000139629.16	ENSG00000273492.5	ENSG00000256087.7
7892	ENSG00000139631.18	ENSG00000273489.1	ENSG00000223313.1
7893	ENSG00000139636.15	ENSG00000273487.1	ENSG00000276853.1
7894	ENSG00000139637.14	ENSG00000273486.1	ENSG00000213757.3

7895	ENSG00000139641.12	ENSG00000273485.1	ENSG00000154165.5
7896	ENSG00000139644.13	ENSG00000273484.1	ENSG00000226814.2
7897	ENSG00000139645.10	ENSG00000273483.1	ENSG00000267089.1
7898	ENSG00000139648.6	ENSG00000273481.1	ENSG00000199525.1
7899	ENSG00000139651.11	ENSG00000273476.1	ENSG00000165972.13
7900	ENSG00000139656.6	ENSG00000273474.1	ENSG00000273444.1
7901	ENSG00000139668.9	ENSG00000273473.1	ENSG00000104415.14
7902	ENSG00000139675.12	ENSG00000273472.1	ENSG00000232876.1
7903	ENSG00000139679.15	ENSG00000273471.1	ENSG00000279774.1
7904	ENSG00000139684.14	ENSG00000273466.1	ENSG00000230148.8
7905	ENSG00000139687.15	ENSG00000273464.1	ENSG00000243193.4
7906	ENSG00000139697.14	ENSG00000273461.5	ENSG00000267062.1
7907	ENSG00000139714.12	ENSG00000273456.1	ENSG00000160472.5
7908	ENSG00000139718.10	ENSG00000273455.1	ENSG00000228413.1
7909	ENSG00000139719.10	ENSG00000273454.1	ENSG00000268751.1
7910	ENSG00000139722.7	ENSG00000273451.1	ENSG00000231201.1
7911	ENSG00000139725.8	ENSG00000273450.1	ENSG00000176320.2
7912	ENSG00000139726.11	ENSG00000273449.1	ENSG00000206990.1
7913	ENSG00000139734.18	ENSG00000273448.1	ENSG00000272822.1
7914	ENSG00000139737.22	ENSG00000273447.1	ENSG00000257199.2
7915	ENSG00000139746.15	ENSG00000273445.1	ENSG00000255353.1
7916	ENSG00000139767.10	ENSG00000273444.1	ENSG00000171840.11
7917	ENSG00000139780.7	ENSG00000273443.1	ENSG00000253103.1
7918	ENSG00000139793.18	ENSG00000273442.1	ENSG00000267011.5
7919	ENSG00000139797.7	ENSG00000273437.1	ENSG00000227012.2
7920	ENSG00000139800.8	ENSG00000273434.1	ENSG00000214264.4
7921	ENSG00000139826.6	ENSG00000273433.1	ENSG00000271779.1
7922	ENSG00000139832.5	ENSG00000273432.1	ENSG00000259505.1
7923	ENSG00000139835.13	ENSG00000273428.3	ENSG00000261491.1
7924	ENSG00000139842.15	ENSG00000273424.1	ENSG00000228948.2
7925	ENSG00000139865.16	ENSG00000273423.1	ENSG00000172346.15
7926	ENSG00000139874.6	ENSG00000273420.1	ENSG00000269583.1
7927	ENSG00000139880.19	ENSG00000273419.1	ENSG00000122859.4
7928	ENSG00000139890.10	ENSG00000273416.1	ENSG00000251639.2
7929	ENSG00000139899.10	ENSG00000273415.2	ENSG00000233252.1
7930	ENSG00000139908.14	ENSG00000273413.1	ENSG00000257543.1
7931	ENSG00000139910.20	ENSG00000273409.2	ENSG00000247193.2
7932	ENSG00000139914.6	ENSG00000273408.1	ENSG00000242737.1
7933	ENSG00000139915.20	ENSG00000273407.1	ENSG00000278647.1
7934	ENSG00000139921.13	ENSG00000273406.1	ENSG00000229814.1
7935	ENSG00000139926.15	ENSG00000273403.1	ENSG00000163885.12
7936	ENSG00000139946.10	ENSG00000273402.1	ENSG00000240750.3
7937	ENSG00000139970.17	ENSG00000273399.2	ENSG00000101638.13
7938	ENSG00000139971.15	ENSG00000273398.6	ENSG00000274620.1
7939	ENSG00000139973.16	ENSG00000273396.2	ENSG00000114374.13
7940	ENSG00000139974.15	ENSG00000273394.1	ENSG00000277087.1
7941	ENSG00000139977.14	ENSG00000273391.1	ENSG00000284424.1
7942	ENSG00000139985.6	ENSG00000273388.1	ENSG00000275026.1
7943	ENSG00000139988.9	ENSG00000273387.1	ENSG00000226375.1
7944	ENSG00000139990.17	ENSG00000273384.1	ENSG00000260287.4
7945	ENSG00000139998.15	ENSG00000273382.1	ENSG00000274797.1
7946	ENSG00000140006.11	ENSG00000273381.1	ENSG00000257550.1
7947	ENSG00000140009.18	ENSG00000273377.1	ENSG00000236384.7

7948	ENSG00000140015.20	ENSG00000273375.1	ENSG00000248969.1
7949	ENSG00000140022.12	ENSG00000273374.1	ENSG00000266908.1
7950	ENSG00000140025.16	ENSG00000273373.1	ENSG00000204849.8
7951	ENSG00000140030.6	ENSG00000273372.1	ENSG00000271130.1
7952	ENSG00000140043.11	ENSG00000273370.1	ENSG00000232493.2
7953	ENSG00000140044.13	ENSG00000273369.1	ENSG00000271454.1
7954	ENSG00000140057.9	ENSG00000273368.1	ENSG00000206828.1
7955	ENSG00000140067.6	ENSG00000273367.1	ENSG00000159374.17
7956	ENSG00000140090.17	ENSG00000273365.1	ENSG00000183185.9
7957	ENSG00000140092.14	ENSG00000273363.1	ENSG00000163904.13
7958	ENSG00000140093.10	ENSG00000273362.1	ENSG00000286168.1
7959	ENSG00000140104.14	ENSG00000273361.1	ENSG00000077522.13
7960	ENSG00000140105.18	ENSG00000273360.1	ENSG00000260266.1
7961	ENSG00000140107.11	ENSG00000273356.1	ENSG00000180660.7
7962	ENSG00000140153.17	ENSG00000273355.1	ENSG00000267786.1
7963	ENSG00000140157.14	ENSG00000273353.1	ENSG00000250796.1
7964	ENSG00000140199.11	ENSG00000273350.1	ENSG00000077254.14
7965	ENSG00000140254.12	ENSG00000273348.1	ENSG00000258792.4
7966	ENSG00000140259.7	ENSG00000273345.5	ENSG00000218574.1
7967	ENSG00000140262.17	ENSG00000273344.1	ENSG00000183853.18
7968	ENSG00000140263.14	ENSG00000273343.1	ENSG00000177736.4
7969	ENSG00000140264.19	ENSG00000273342.1	ENSG00000285770.1
7970	ENSG00000140265.12	ENSG00000273341.1	ENSG00000215006.4
7971	ENSG00000140274.13	ENSG00000273340.1	ENSG00000243930.1
7972	ENSG00000140279.12	ENSG00000273338.1	ENSG00000261481.1
7973	ENSG00000140280.14	ENSG00000273336.1	ENSG00000225169.1
7974	ENSG00000140284.11	ENSG00000273335.1	ENSG00000260100.1
7975	ENSG00000140285.10	ENSG00000273333.2	ENSG00000172146.2
7976	ENSG00000140287.11	ENSG00000273331.1	ENSG00000215492.6
7977	ENSG00000140297.13	ENSG00000273330.1	ENSG00000169247.12
7978	ENSG00000140299.11	ENSG00000273329.1	ENSG00000233930.3
7979	ENSG00000140307.11	ENSG00000273328.5	ENSG00000225473.1
7980	ENSG00000140319.10	ENSG00000273327.1	ENSG00000214078.12
7981	ENSG00000140320.12	ENSG00000273325.1	ENSG00000237475.2
7982	ENSG00000140323.6	ENSG00000273321.1	ENSG00000278595.1
7983	ENSG00000140326.13	ENSG00000273320.1	ENSG00000283286.1
7984	ENSG00000140332.15	ENSG00000273319.1	ENSG00000261143.1
7985	ENSG00000140350.15	ENSG00000273314.1	ENSG00000280433.1
7986	ENSG00000140365.15	ENSG00000273313.1	ENSG00000242206.2
7987	ENSG00000140367.11	ENSG00000273312.2	ENSG00000240729.1
7988	ENSG00000140368.12	ENSG00000273311.1	ENSG00000267640.6
7989	ENSG00000140374.16	ENSG00000273308.1	ENSG00000145864.12
7990	ENSG00000140379.8	ENSG00000273306.1	ENSG00000186051.6
7991	ENSG00000140382.15	ENSG00000273305.1	ENSG00000108587.15
7992	ENSG00000140386.13	ENSG00000273302.1	ENSG00000201778.1
7993	ENSG00000140391.14	ENSG00000273301.1	ENSG00000234026.1
7994	ENSG00000140395.9	ENSG00000273300.1	ENSG00000264007.1
7995	ENSG00000140396.13	ENSG00000273299.1	ENSG00000102595.20
7996	ENSG00000140398.13	ENSG00000273297.1	ENSG00000249019.2
7997	ENSG00000140400.17	ENSG00000273295.1	ENSG00000170608.3
7998	ENSG00000140403.12	ENSG00000273294.1	ENSG00000250076.1
7999	ENSG00000140406.3	ENSG00000273293.1	ENSG00000232316.1
8000	ENSG00000140416.21	ENSG00000273291.5	ENSG00000130299.17

8001	ENSG00000140443.14	ENSG00000273289.1	ENSG00000118785.14
8002	ENSG00000140450.9	ENSG00000273287.2	ENSG00000279502.1
8003	ENSG00000140451.13	ENSG00000273284.1	ENSG00000188536.13
8004	ENSG00000140455.17	ENSG00000273275.1	ENSG00000162897.15
8005	ENSG00000140459.18	ENSG00000273274.2	ENSG00000144452.15
8006	ENSG00000140463.14	ENSG00000273272.2	ENSG00000138162.19
8007	ENSG00000140464.19	ENSG00000273271.1	ENSG00000172551.11
8008	ENSG00000140465.14	ENSG00000273270.1	ENSG00000143217.9
8009	ENSG00000140470.14	ENSG00000273269.3	ENSG00000254177.1
8010	ENSG00000140471.17	ENSG00000273267.1	ENSG00000203914.4
8011	ENSG00000140474.14	ENSG00000273265.1	ENSG00000224280.1
8012	ENSG00000140478.15	ENSG00000273264.1	ENSG00000225916.1
8013	ENSG00000140479.17	ENSG00000273262.1	ENSG00000211448.11
8014	ENSG00000140481.15	ENSG00000273261.1	ENSG00000256968.1
8015	ENSG00000140488.16	ENSG00000273259.3	ENSG00000073584.20
8016	ENSG00000140497.16	ENSG00000273258.1	ENSG00000197217.13
8017	ENSG00000140505.7	ENSG00000273257.1	ENSG00000249478.1
8018	ENSG00000140506.16	ENSG00000273255.1	ENSG00000243410.1
8019	ENSG00000140511.11	ENSG00000273253.2	ENSG00000262372.1
8020	ENSG00000140519.14	ENSG00000273252.1	ENSG00000272209.1
8021	ENSG00000140521.13	ENSG00000273249.1	ENSG00000106591.4
8022	ENSG00000140522.12	ENSG00000273248.1	ENSG00000237781.3
8023	ENSG00000140525.17	ENSG00000273247.5	ENSG00000270809.1
8024	ENSG00000140526.18	ENSG00000273244.1	ENSG00000200814.1
8025	ENSG00000140527.15	ENSG00000273240.1	ENSG00000207233.1
8026	ENSG00000140534.13	ENSG00000273238.2	ENSG00000261716.2
8027	ENSG00000140538.16	ENSG00000273234.1	ENSG00000262081.2
8028	ENSG00000140543.14	ENSG00000273233.1	ENSG00000275005.1
8029	ENSG00000140545.15	ENSG00000273232.1	ENSG00000239467.5
8030	ENSG00000140548.10	ENSG00000273230.1	ENSG00000270966.1
8031	ENSG00000140553.17	ENSG00000273228.1	ENSG00000232979.1
8032	ENSG00000140557.12	ENSG00000273226.1	ENSG00000133026.12
8033	ENSG00000140563.15	ENSG00000273225.4	ENSG00000213711.3
8034	ENSG00000140564.12	ENSG00000273221.1	ENSG00000269476.1
8035	ENSG00000140575.13	ENSG00000273219.1	ENSG00000283142.1
8036	ENSG00000140577.16	ENSG00000273218.1	ENSG00000217030.1
8037	ENSG00000140598.15	ENSG00000273217.1	ENSG00000232422.1
8038	ENSG00000140600.17	ENSG00000273216.1	ENSG00000227513.1
8039	ENSG00000140612.14	ENSG00000273214.1	ENSG00000275801.1
8040	ENSG00000140623.13	ENSG00000273213.2	ENSG00000278770.1
8041	ENSG00000140632.17	ENSG00000273212.1	ENSG00000255326.1
8042	ENSG00000140650.12	ENSG00000273211.1	ENSG00000092607.15
8043	ENSG00000140675.13	ENSG00000273210.1	ENSG00000256206.2
8044	ENSG00000140678.16	ENSG00000273209.1	ENSG00000217539.2
8045	ENSG00000140682.19	ENSG00000273204.1	ENSG00000159166.13
8046	ENSG00000140688.17	ENSG00000273203.1	ENSG00000251221.1
8047	ENSG00000140691.16	ENSG00000273199.1	ENSG00000231194.1
8048	ENSG00000140694.17	ENSG00000273198.1	ENSG00000236154.1
8049	ENSG00000140718.21	ENSG00000273196.1	ENSG00000186207.5
8050	ENSG00000140740.10	ENSG00000273192.1	ENSG00000136943.11
8051	ENSG00000140743.8	ENSG00000273188.1	ENSG00000247675.6
8052	ENSG00000140749.9	ENSG00000273186.1	ENSG00000130477.15
8053	ENSG00000140750.17	ENSG00000273184.1	ENSG00000234818.1

8054	ENSG00000140795.13	ENSG00000273183.1	ENSG00000214301.4
8055	ENSG00000140798.16	ENSG00000273181.1	ENSG00000165204.3
8056	ENSG00000140807.7	ENSG00000273180.1	ENSG00000183317.17
8057	ENSG00000140829.12	ENSG00000273179.1	ENSG00000213996.13
8058	ENSG00000140830.9	ENSG00000273177.1	ENSG00000175183.10
8059	ENSG00000140832.9	ENSG00000273176.1	ENSG00000286022.1
8060	ENSG00000140835.9	ENSG00000273175.1	ENSG00000255556.2
8061	ENSG00000140836.17	ENSG00000273174.1	ENSG00000227582.2
8062	ENSG00000140839.11	ENSG00000273173.5	ENSG00000235397.1
8063	ENSG00000140848.17	ENSG00000273172.1	ENSG00000278878.1
8064	ENSG00000140853.15	ENSG00000273171.1	ENSG00000227578.1
8065	ENSG00000140854.13	ENSG00000273167.1	ENSG00000204718.3
8066	ENSG00000140859.16	ENSG00000273165.1	ENSG00000224184.5
8067	ENSG00000140873.16	ENSG00000273164.1	ENSG00000267703.1
8068	ENSG00000140876.11	ENSG00000273162.1	ENSG00000244502.2
8069	ENSG00000140905.10	ENSG00000273160.1	ENSG00000137960.6
8070	ENSG00000140931.20	ENSG00000273156.2	ENSG00000251297.1
8071	ENSG00000140932.10	ENSG00000273155.1	ENSG00000286080.1
8072	ENSG00000140937.14	ENSG00000273154.3	ENSG00000104967.7
8073	ENSG00000140939.14	ENSG00000273153.1	ENSG00000249755.2
8074	ENSG00000140941.13	ENSG00000273151.1	ENSG00000081853.15
8075	ENSG00000140943.17	ENSG00000273148.1	ENSG00000250516.1
8076	ENSG00000140945.17	ENSG00000273145.1	ENSG00000261439.1
8077	ENSG00000140948.11	ENSG00000273143.1	ENSG00000272057.1
8078	ENSG00000140950.16	ENSG00000273142.1	ENSG00000167994.12
8079	ENSG00000140955.10	ENSG00000273141.1	ENSG00000226543.3
8080	ENSG00000140961.13	ENSG00000273139.1	ENSG00000272469.2
8081	ENSG00000140968.11	ENSG00000273138.1	ENSG00000152093.8
8082	ENSG00000140983.14	ENSG00000273137.1	ENSG00000139990.17
8083	ENSG00000140986.8	ENSG00000273136.7	ENSG00000254931.2
8084	ENSG00000140987.20	ENSG00000273132.1	ENSG00000235020.4
8085	ENSG00000140988.16	ENSG00000273129.1	ENSG00000104341.16
8086	ENSG00000140990.15	ENSG00000273125.1	ENSG00000151967.18
8087	ENSG00000140992.19	ENSG00000273124.1	ENSG00000225670.4
8088	ENSG00000140993.11	ENSG00000273123.1	ENSG00000224323.2
8089	ENSG00000140995.16	ENSG00000273119.1	ENSG00000201616.1
8090	ENSG00000141002.20	ENSG00000273118.1	ENSG00000007080.11
8091	ENSG00000141012.13	ENSG00000273117.1	ENSG00000275924.1
8092	ENSG00000141013.17	ENSG00000273115.1	ENSG00000218048.2
8093	ENSG00000141026.6	ENSG00000273113.1	ENSG00000230515.1
8094	ENSG00000141027.21	ENSG00000273112.1	ENSG00000224995.1
8095	ENSG00000141028.6	ENSG00000273111.6	ENSG00000259102.2
8096	ENSG00000141030.13	ENSG00000273110.1	ENSG00000284360.1
8097	ENSG00000141034.9	ENSG00000273108.1	ENSG00000271784.1
8098	ENSG00000141040.15	ENSG00000273107.1	ENSG00000235644.1
8099	ENSG00000141052.17	ENSG00000273104.1	ENSG00000254484.1
8100	ENSG00000141068.14	ENSG00000273102.1	ENSG00000101000.6
8101	ENSG00000141076.18	ENSG00000273100.1	ENSG00000270181.3
8102	ENSG00000141084.11	ENSG00000273098.2	ENSG00000253924.1
8103	ENSG00000141086.18	ENSG00000273096.1	ENSG00000229536.2
8104	ENSG00000141096.5	ENSG00000273093.1	ENSG00000110195.13
8105	ENSG00000141098.13	ENSG00000273091.1	ENSG00000130518.17
8106	ENSG00000141101.13	ENSG00000273090.1	ENSG00000004848.8

8107	ENSG00000141127.15	ENSG00000273088.1	ENSG00000171773.2
8108	ENSG00000141161.11	ENSG00000273085.1	ENSG00000163874.11
8109	ENSG00000141179.13	ENSG00000273084.1	ENSG00000270048.1
8110	ENSG00000141194.6	ENSG00000273082.1	ENSG00000167634.12
8111	ENSG00000141198.16	ENSG00000273080.1	ENSG00000236136.1
8112	ENSG00000141200.7	ENSG00000273079.5	ENSG00000271525.1
8113	ENSG00000141219.15	ENSG00000273077.1	ENSG00000143340.6
8114	ENSG00000141232.5	ENSG00000273076.1	ENSG00000188801.9
8115	ENSG00000141252.20	ENSG00000273073.1	ENSG00000137814.11
8116	ENSG00000141255.12	ENSG00000273069.1	ENSG00000254946.1
8117	ENSG00000141258.13	ENSG00000273068.1	ENSG00000127533.3
8118	ENSG00000141279.16	ENSG00000273066.5	ENSG00000235538.1
8119	ENSG00000141293.16	ENSG00000273064.1	ENSG00000232347.1
8120	ENSG00000141294.10	ENSG00000273063.1	ENSG00000225953.2
8121	ENSG00000141295.14	ENSG00000273062.1	ENSG00000234750.1
8122	ENSG00000141298.19	ENSG00000273061.1	ENSG00000231701.1
8123	ENSG00000141314.13	ENSG00000273059.1	ENSG00000130707.17
8124	ENSG00000141337.12	ENSG00000273058.2	ENSG00000229239.2
8125	ENSG00000141338.14	ENSG00000273055.1	ENSG00000234715.1
8126	ENSG00000141349.8	ENSG00000273051.1	ENSG00000221977.2
8127	ENSG00000141367.11	ENSG00000273049.1	ENSG00000247775.2
8128	ENSG00000141371.13	ENSG00000273047.1	ENSG00000258212.1
8129	ENSG00000141376.22	ENSG00000273046.1	ENSG00000106823.12
8130	ENSG00000141378.14	ENSG00000273045.6	ENSG00000225146.1
8131	ENSG00000141380.14	ENSG00000273044.1	ENSG00000207118.1
8132	ENSG00000141384.12	ENSG00000273041.1	ENSG00000231726.2
8133	ENSG00000141385.9	ENSG00000273038.2	ENSG00000145388.15
8134	ENSG00000141391.14	ENSG00000273036.3	ENSG00000244155.1
8135	ENSG00000141401.12	ENSG00000273035.1	ENSG00000120337.8
8136	ENSG00000141404.16	ENSG00000273033.2	ENSG00000042317.17
8137	ENSG00000141424.13	ENSG00000273032.2	ENSG00000137968.16
8138	ENSG00000141425.18	ENSG00000273027.1	ENSG00000239710.3
8139	ENSG00000141428.17	ENSG00000273026.1	ENSG00000266613.1
8140	ENSG00000141429.13	ENSG00000273025.1	ENSG00000239648.1
8141	ENSG00000141431.12	ENSG00000273024.6	ENSG00000200674.1
8142	ENSG00000141433.13	ENSG00000273018.6	ENSG00000160801.14
8143	ENSG00000141434.11	ENSG00000273017.1	ENSG00000225418.1
8144	ENSG00000141437.8	ENSG00000273015.2	ENSG00000148357.16
8145	ENSG00000141441.16	ENSG00000273014.1	ENSG00000286226.1
8146	ENSG00000141446.11	ENSG00000273013.1	ENSG00000269964.3
8147	ENSG00000141447.18	ENSG00000273012.1	ENSG00000285823.1
8148	ENSG00000141448.9	ENSG00000273011.1	ENSG00000227888.4
8149	ENSG00000141449.14	ENSG00000273010.1	ENSG00000285974.1
8150	ENSG00000141452.9	ENSG00000273007.1	ENSG00000273885.1
8151	ENSG00000141456.14	ENSG00000273006.1	ENSG00000276729.1
8152	ENSG00000141458.13	ENSG00000273004.1	ENSG00000159961.2
8153	ENSG00000141469.18	ENSG00000273003.1	ENSG00000280245.1
8154	ENSG00000141480.18	ENSG00000273002.1	ENSG00000133317.14
8155	ENSG00000141485.16	ENSG00000273001.1	ENSG00000176040.13
8156	ENSG00000141497.14	ENSG00000272995.1	ENSG00000183878.15
8157	ENSG00000141499.16	ENSG00000272994.1	ENSG00000253958.1
8158	ENSG00000141503.16	ENSG00000272991.1	ENSG00000007516.13
8159	ENSG00000141504.11	ENSG00000272990.1	ENSG00000258454.1

8160	ENSG00000141505.12	ENSG00000272989.1	ENSG00000261745.2
8161	ENSG00000141506.13	ENSG00000272988.1	ENSG00000153233.13
8162	ENSG00000141510.17	ENSG00000272987.1	ENSG00000225272.2
8163	ENSG00000141519.15	ENSG00000272984.1	ENSG00000124788.18
8164	ENSG00000141522.12	ENSG00000272983.1	ENSG00000058866.15
8165	ENSG00000141524.15	ENSG00000272982.1	ENSG00000280064.1
8166	ENSG00000141526.16	ENSG00000272979.1	ENSG00000218350.1
8167	ENSG00000141527.18	ENSG00000272977.1	ENSG00000229468.1
8168	ENSG00000141540.11	ENSG00000272975.1	ENSG00000263301.1
8169	ENSG00000141542.11	ENSG00000272973.1	ENSG00000259064.2
8170	ENSG00000141543.11	ENSG00000272971.1	ENSG00000285673.1
8171	ENSG00000141551.14	ENSG00000272970.1	ENSG00000132780.17
8172	ENSG00000141552.17	ENSG00000272969.1	ENSG00000229153.5
8173	ENSG00000141556.21	ENSG00000272968.5	ENSG00000198914.3
8174	ENSG00000141560.15	ENSG00000272967.1	ENSG00000280060.1
8175	ENSG00000141562.18	ENSG00000272966.1	ENSG00000275954.5
8176	ENSG00000141564.15	ENSG00000272963.1	ENSG00000134873.10
8177	ENSG00000141568.21	ENSG00000272958.1	ENSG00000239570.1
8178	ENSG00000141569.12	ENSG00000272954.1	ENSG00000215397.4
8179	ENSG00000141570.11	ENSG00000272953.1	ENSG00000094661.3
8180	ENSG00000141574.7	ENSG00000272950.1	ENSG00000132005.9
8181	ENSG00000141576.16	ENSG00000272949.1	ENSG00000240861.1
8182	ENSG00000141577.14	ENSG00000272948.2	ENSG00000065413.20
8183	ENSG00000141579.7	ENSG00000272945.1	ENSG00000125571.9
8184	ENSG00000141580.16	ENSG00000272944.1	ENSG00000224746.1
8185	ENSG00000141582.15	ENSG00000272942.1	ENSG00000213131.3
8186	ENSG00000141622.14	ENSG00000272941.1	ENSG00000234745.11
8187	ENSG00000141627.13	ENSG00000272940.1	ENSG00000243225.2
8188	ENSG00000141639.12	ENSG00000272937.2	ENSG00000197766.7
8189	ENSG00000141642.9	ENSG00000272936.1	ENSG00000139610.1
8190	ENSG00000141644.17	ENSG00000272934.1	ENSG00000267506.5
8191	ENSG00000141646.13	ENSG00000272933.1	ENSG00000279177.1
8192	ENSG00000141655.17	ENSG00000272931.1	ENSG00000268866.1
8193	ENSG00000141664.9	ENSG00000272927.1	ENSG00000260613.1
8194	ENSG00000141665.12	ENSG00000272923.1	ENSG00000179997.8
8195	ENSG00000141668.10	ENSG00000272922.1	ENSG00000258150.6
8196	ENSG00000141682.11	ENSG00000272921.1	ENSG00000253540.5
8197	ENSG00000141696.13	ENSG00000272920.1	ENSG00000281021.1
8198	ENSG00000141698.16	ENSG00000272918.1	ENSG00000213754.2
8199	ENSG00000141699.11	ENSG00000272917.1	ENSG00000269867.1
8200	ENSG00000141736.13	ENSG00000272916.5	ENSG00000233873.1
8201	ENSG00000141738.14	ENSG00000272915.1	ENSG00000249628.3
8202	ENSG00000141741.12	ENSG00000272914.1	ENSG00000120729.9
8203	ENSG00000141744.3	ENSG00000272913.1	ENSG00000237186.1
8204	ENSG00000141748.12	ENSG00000272912.1	ENSG00000132514.13
8205	ENSG00000141750.7	ENSG00000272911.1	ENSG00000114547.10
8206	ENSG00000141753.7	ENSG00000272910.1	ENSG00000286216.1
8207	ENSG00000141759.15	ENSG00000272909.1	ENSG00000179431.6
8208	ENSG00000141837.19	ENSG00000272908.1	ENSG00000230769.1
8209	ENSG00000141854.9	ENSG00000272906.1	ENSG00000250170.1
8210	ENSG00000141858.11	ENSG00000272905.1	ENSG00000203663.4
8211	ENSG00000141867.17	ENSG00000272904.1	ENSG00000173389.16
8212	ENSG00000141873.11	ENSG00000272902.2	ENSG00000184530.9

8213	ENSG00000141905.19	ENSG00000272900.1	ENSG00000257703.5
8214	ENSG00000141933.9	ENSG00000272899.4	ENSG00000259753.1
8215	ENSG00000141934.10	ENSG00000272897.5	ENSG00000094963.14
8216	ENSG00000141946.1	ENSG00000272896.1	ENSG00000152086.9
8217	ENSG00000141956.13	ENSG00000272895.1	ENSG00000108819.11
8218	ENSG00000141959.17	ENSG00000272894.5	ENSG00000240567.1
8219	ENSG00000141965.4	ENSG00000272892.1	ENSG00000106384.12
8220	ENSG00000141968.8	ENSG00000272888.6	ENSG00000266941.1
8221	ENSG00000141971.13	ENSG00000272886.6	ENSG00000169271.3
8222	ENSG00000141977.9	ENSG00000272885.1	ENSG00000268951.1
8223	ENSG00000141979.4	ENSG00000272884.1	ENSG00000262316.1
8224	ENSG00000141985.9	ENSG00000272882.1	ENSG00000142149.9
8225	ENSG00000141994.16	ENSG00000272880.1	ENSG00000157542.11
8226	ENSG00000142002.17	ENSG00000272874.1	ENSG00000226298.1
8227	ENSG00000142025.16	ENSG00000272872.1	ENSG00000251356.1
8228	ENSG00000142039.4	ENSG00000272871.1	ENSG00000280997.1
8229	ENSG00000142046.15	ENSG00000272870.2	ENSG00000133110.15
8230	ENSG00000142065.14	ENSG00000272866.1	ENSG00000205240.4
8231	ENSG00000142082.15	ENSG00000272865.1	ENSG00000278865.1
8232	ENSG00000142089.16	ENSG00000272864.1	ENSG00000179363.7
8233	ENSG00000142102.15	ENSG00000272862.1	ENSG00000236861.6
8234	ENSG00000142149.9	ENSG00000272861.1	ENSG00000226803.8
8235	ENSG00000142156.14	ENSG00000272858.1	ENSG00000259332.3
8236	ENSG00000142163.8	ENSG00000272856.1	ENSG00000251363.3
8237	ENSG00000142166.13	ENSG00000272855.1	ENSG00000234816.2
8238	ENSG00000142168.14	ENSG00000272854.1	ENSG00000261544.1
8239	ENSG00000142173.15	ENSG00000272853.1	ENSG00000255983.1
8240	ENSG00000142178.8	ENSG00000272851.1	ENSG00000277591.1
8241	ENSG00000142182.8	ENSG00000272849.1	ENSG00000242856.3
8242	ENSG00000142185.16	ENSG00000272848.2	ENSG00000204022.9
8243	ENSG00000142186.17	ENSG00000272844.1	ENSG00000255343.1
8244	ENSG00000142188.17	ENSG00000272843.1	ENSG00000134242.16
8245	ENSG00000142192.21	ENSG00000272842.1	ENSG00000185433.9
8246	ENSG00000142197.12	ENSG00000272841.1	ENSG00000185532.17
8247	ENSG00000142207.7	ENSG00000272840.1	ENSG00000168631.12
8248	ENSG00000142208.16	ENSG00000272839.1	ENSG00000150753.12
8249	ENSG00000142224.15	ENSG00000272837.1	ENSG00000100385.14
8250	ENSG00000142227.11	ENSG00000272836.1	ENSG00000227755.1
8251	ENSG00000142230.11	ENSG00000272834.1	ENSG00000127540.12
8252	ENSG00000142233.11	ENSG00000272832.1	ENSG00000225854.2
8253	ENSG00000142235.9	ENSG00000272831.1	ENSG00000232383.1
8254	ENSG00000142252.10	ENSG00000272829.1	ENSG00000237434.1
8255	ENSG00000142273.12	ENSG00000272825.1	ENSG00000236846.1
8256	ENSG00000142279.12	ENSG00000272824.1	ENSG00000251163.1
8257	ENSG00000142303.14	ENSG00000272823.1	ENSG00000242578.1
8258	ENSG00000142319.18	ENSG00000272822.1	ENSG00000196660.10
8259	ENSG00000142327.13	ENSG00000272821.1	ENSG00000255472.1
8260	ENSG00000142330.20	ENSG00000272817.1	ENSG00000267557.1
8261	ENSG00000142347.17	ENSG00000272815.1	ENSG00000276040.4
8262	ENSG00000142396.10	ENSG00000272814.1	ENSG00000285623.1
8263	ENSG00000142405.21	ENSG00000272812.1	ENSG00000235426.2
8264	ENSG00000142408.4	ENSG00000272810.1	ENSG00000274290.2
8265	ENSG00000142409.6	ENSG00000272808.4	ENSG00000119669.5

8266	ENSG00000142444.7	ENSG00000272807.1	ENSG00000266365.1
8267	ENSG00000142449.13	ENSG00000272804.3	ENSG00000257924.1
8268	ENSG00000142453.11	ENSG00000272801.1	ENSG00000226235.1
8269	ENSG00000142459.8	ENSG00000272800.2	ENSG00000228914.2
8270	ENSG00000142484.7	ENSG00000272799.1	ENSG00000229386.3
8271	ENSG00000142494.13	ENSG00000272798.1	ENSG00000225218.1
8272	ENSG00000142507.10	ENSG00000272797.1	ENSG00000249454.1
8273	ENSG00000142511.4	ENSG00000272795.1	ENSG00000269502.5
8274	ENSG00000142512.15	ENSG00000272791.1	ENSG00000258064.1
8275	ENSG00000142513.5	ENSG00000272789.1	ENSG00000258930.1
8276	ENSG00000142515.15	ENSG00000272788.1	ENSG00000229154.1
8277	ENSG00000142528.16	ENSG00000272787.1	ENSG00000230047.1
8278	ENSG00000142530.10	ENSG00000272784.1	ENSG00000196260.5
8279	ENSG00000142534.7	ENSG00000272783.1	ENSG00000241668.2
8280	ENSG00000142538.1	ENSG00000272779.1	ENSG00000215548.2
8281	ENSG00000142539.9	ENSG00000272777.1	ENSG00000173848.19
8282	ENSG00000142541.17	ENSG00000272774.1	ENSG00000248964.6
8283	ENSG00000142544.7	ENSG00000272772.1	ENSG00000235563.1
8284	ENSG00000142546.13	ENSG00000272770.1	ENSG00000181101.7
8285	ENSG00000142549.9	ENSG00000272769.1	ENSG00000258928.1
8286	ENSG00000142552.8	ENSG00000272768.1	ENSG00000285012.1
8287	ENSG00000142556.19	ENSG00000272767.1	ENSG00000138400.13
8288	ENSG00000142583.17	ENSG00000272764.1	ENSG00000255946.1
8289	ENSG00000142599.18	ENSG00000272763.1	ENSG00000260840.2
8290	ENSG00000142606.16	ENSG00000272762.5	ENSG00000229405.1
8291	ENSG00000142609.18	ENSG00000272760.1	ENSG00000242931.1
8292	ENSG00000142611.17	ENSG00000272758.5	ENSG00000070808.15
8293	ENSG00000142615.8	ENSG00000272755.1	ENSG00000140297.13
8294	ENSG00000142619.4	ENSG00000272754.1	ENSG00000172340.14
8295	ENSG00000142621.19	ENSG00000272752.6	ENSG00000101180.16
8296	ENSG00000142623.11	ENSG00000272750.1	ENSG00000203437.3
8297	ENSG00000142627.13	ENSG00000272748.1	ENSG00000204422.7
8298	ENSG00000142632.17	ENSG00000272746.1	ENSG00000242199.1
8299	ENSG00000142634.13	ENSG00000272745.1	ENSG00000285887.1
8300	ENSG00000142655.13	ENSG00000272744.1	ENSG00000262292.2
8301	ENSG00000142657.21	ENSG00000272742.1	ENSG00000272489.1
8302	ENSG00000142661.19	ENSG00000272741.1	ENSG00000260675.1
8303	ENSG00000142669.15	ENSG00000272736.5	ENSG00000254092.1
8304	ENSG00000142675.18	ENSG00000272735.1	ENSG00000255276.1
8305	ENSG00000142676.14	ENSG00000272734.1	ENSG00000268535.1
8306	ENSG00000142677.4	ENSG00000272732.1	ENSG00000268931.1
8307	ENSG00000142684.9	ENSG00000272729.1	ENSG00000228366.1
8308	ENSG00000142686.8	ENSG00000272727.1	ENSG00000253239.1
8309	ENSG00000142687.18	ENSG00000272721.5	ENSG00000263648.1
8310	ENSG00000142694.6	ENSG00000272720.1	ENSG00000251544.1
8311	ENSG00000142698.15	ENSG00000272719.1	ENSG00000233293.1
8312	ENSG00000142700.12	ENSG00000272717.1	ENSG00000198911.12
8313	ENSG00000142731.11	ENSG00000272715.1	ENSG00000258942.1
8314	ENSG00000142733.16	ENSG00000272714.1	ENSG00000243672.1
8315	ENSG00000142748.12	ENSG00000272711.1	ENSG00000230927.3
8316	ENSG00000142751.15	ENSG00000272710.2	ENSG00000229780.1
8317	ENSG00000142765.18	ENSG00000272707.1	ENSG00000243016.1
8318	ENSG00000142784.16	ENSG00000272703.1	ENSG00000231769.2

8319	ENSG00000142789.19	ENSG00000272702.1	ENSG00000104044.16
8320	ENSG00000142794.18	ENSG00000272701.3	ENSG00000271236.1
8321	ENSG00000142798.20	ENSG00000272699.1	ENSG00000134201.12
8322	ENSG00000142856.16	ENSG00000272696.1	ENSG00000275976.5
8323	ENSG00000142864.14	ENSG00000272695.1	ENSG00000258284.1
8324	ENSG00000142867.13	ENSG00000272694.1	ENSG00000224164.1
8325	ENSG00000142871.17	ENSG00000272693.4	ENSG00000112246.9
8326	ENSG00000142875.19	ENSG00000272692.1	ENSG00000169436.17
8327	ENSG00000142892.15	ENSG00000272691.1	ENSG00000273791.1
8328	ENSG00000142910.16	ENSG00000272690.5	ENSG00000259607.1
8329	ENSG00000142920.17	ENSG00000272689.1	ENSG00000182195.7
8330	ENSG00000142937.12	ENSG00000272688.1	ENSG00000170890.14
8331	ENSG00000142945.13	ENSG00000272686.1	ENSG00000150471.16
8332	ENSG00000142949.17	ENSG00000272685.1	ENSG00000173253.15
8333	ENSG00000142959.5	ENSG00000272682.1	ENSG00000223391.2
8334	ENSG00000142961.14	ENSG00000272681.2	ENSG00000156970.12
8335	ENSG00000142973.13	ENSG00000272679.1	ENSG00000285184.2
8336	ENSG00000143001.5	ENSG00000272678.1	ENSG00000245662.3
8337	ENSG00000143006.7	ENSG00000272677.1	ENSG00000237988.5
8338	ENSG00000143013.13	ENSG00000272676.1	ENSG00000236844.1
8339	ENSG00000143028.9	ENSG00000272674.3	ENSG00000285935.1
8340	ENSG00000143032.8	ENSG00000272672.1	ENSG00000255115.2
8341	ENSG00000143033.18	ENSG00000272669.1	ENSG00000238110.1
8342	ENSG00000143036.17	ENSG00000272667.1	ENSG00000256657.1
8343	ENSG00000143061.17	ENSG00000272666.1	ENSG00000167992.13
8344	ENSG00000143067.5	ENSG00000272664.1	ENSG00000163273.4
8345	ENSG00000143079.15	ENSG00000272663.1	ENSG00000257488.5
8346	ENSG00000143093.15	ENSG00000272662.1	ENSG00000132932.17
8347	ENSG00000143105.7	ENSG00000272661.1	ENSG00000155918.7
8348	ENSG00000143106.13	ENSG00000272660.1	ENSG00000242858.1
8349	ENSG00000143107.9	ENSG00000272657.1	ENSG00000285594.1
8350	ENSG00000143110.11	ENSG00000272656.1	ENSG00000263574.1
8351	ENSG00000143119.14	ENSG00000272655.2	ENSG00000138180.15
8352	ENSG00000143125.6	ENSG00000272654.1	ENSG00000205710.4
8353	ENSG00000143126.8	ENSG00000272650.1	ENSG00000272293.1
8354	ENSG00000143127.13	ENSG00000272647.3	ENSG00000250641.1
8355	ENSG00000143147.14	ENSG00000272646.1	ENSG00000155100.10
8356	ENSG00000143149.12	ENSG00000272645.3	ENSG00000274659.1
8357	ENSG00000143153.12	ENSG00000272644.1	ENSG00000174473.16
8358	ENSG00000143155.13	ENSG00000272642.2	ENSG00000273507.4
8359	ENSG00000143156.14	ENSG00000272639.1	ENSG00000115252.18
8360	ENSG00000143157.11	ENSG00000272638.1	ENSG00000278842.1
8361	ENSG00000143158.11	ENSG00000272636.4	ENSG00000248967.1
8362	ENSG00000143162.9	ENSG00000272635.1	ENSG00000259201.1
8363	ENSG00000143164.15	ENSG00000272634.2	ENSG00000235436.10
8364	ENSG00000143167.12	ENSG00000272632.1	ENSG00000258647.5
8365	ENSG00000143171.13	ENSG00000272631.1	ENSG00000143473.13
8366	ENSG00000143178.13	ENSG00000272630.1	ENSG00000235076.2
8367	ENSG00000143179.16	ENSG00000272627.1	ENSG00000173575.21
8368	ENSG00000143183.16	ENSG00000272626.1	ENSG00000212126.3
8369	ENSG00000143184.5	ENSG00000272625.1	ENSG00000248557.1
8370	ENSG00000143190.23	ENSG00000272622.1	ENSG00000261635.2
8371	ENSG00000143194.13	ENSG00000272619.1	ENSG00000236709.1

8372	ENSG00000143195.13	ENSG00000272617.3	ENSG00000225554.1
8373	ENSG00000143196.5	ENSG00000272610.1	ENSG00000254604.1
8374	ENSG00000143198.13	ENSG00000272609.1	ENSG00000223885.4
8375	ENSG00000143199.17	ENSG00000272606.1	ENSG00000079335.20
8376	ENSG00000143207.21	ENSG00000272604.1	ENSG00000254550.1
8377	ENSG00000143217.9	ENSG00000272602.6	ENSG00000109255.11
8378	ENSG00000143222.12	ENSG00000272601.1	ENSG00000274570.4
8379	ENSG00000143224.18	ENSG00000272600.1	ENSG00000236770.1
8380	ENSG00000143226.13	ENSG00000272599.2	ENSG00000124469.12
8381	ENSG00000143228.13	ENSG00000272597.1	ENSG00000263829.1
8382	ENSG00000143248.13	ENSG00000272595.2	ENSG00000233996.1
8383	ENSG00000143252.14	ENSG00000272593.1	ENSG00000253741.1
8384	ENSG00000143256.5	ENSG00000272592.1	ENSG00000064835.10
8385	ENSG00000143257.11	ENSG00000272589.1	ENSG00000204025.7
8386	ENSG00000143258.16	ENSG00000272588.1	ENSG00000270542.1
8387	ENSG00000143278.4	ENSG00000272583.1	ENSG00000226856.6
8388	ENSG00000143294.15	ENSG00000272582.1	ENSG00000257545.5
8389	ENSG00000143303.12	ENSG00000272578.5	ENSG00000249334.1
8390	ENSG00000143314.12	ENSG00000272576.1	ENSG00000248596.7
8391	ENSG00000143315.7	ENSG00000272575.6	ENSG00000251046.2
8392	ENSG00000143318.13	ENSG00000272574.1	ENSG00000161011.20
8393	ENSG00000143319.16	ENSG00000272573.6	ENSG00000227408.1
8394	ENSG00000143320.9	ENSG00000272572.1	ENSG00000011465.17
8395	ENSG00000143321.19	ENSG00000272569.1	ENSG00000211973.2
8396	ENSG00000143322.20	ENSG00000272568.5	ENSG00000224516.5
8397	ENSG00000143324.13	ENSG00000272567.1	ENSG00000237257.1
8398	ENSG00000143333.7	ENSG00000272566.1	ENSG00000205274.3
8399	ENSG00000143337.18	ENSG00000272564.1	ENSG00000271776.1
8400	ENSG00000143340.6	ENSG00000272563.1	ENSG00000136758.18
8401	ENSG00000143341.12	ENSG00000272562.1	ENSG00000164929.17
8402	ENSG00000143344.15	ENSG00000272559.1	ENSG00000143774.16
8403	ENSG00000143353.12	ENSG00000272558.1	ENSG00000232623.1
8404	ENSG00000143355.16	ENSG00000272556.2	ENSG00000165076.13
8405	ENSG00000143363.17	ENSG00000272555.1	ENSG00000279575.1
8406	ENSG00000143365.18	ENSG00000272554.1	ENSG00000259906.1
8407	ENSG00000143367.16	ENSG00000272551.1	ENSG00000232388.4
8408	ENSG00000143368.10	ENSG00000272549.1	ENSG00000235621.8
8409	ENSG00000143369.15	ENSG00000272545.1	ENSG00000245067.6
8410	ENSG00000143373.18	ENSG00000272543.1	ENSG00000157538.14
8411	ENSG00000143374.17	ENSG00000272542.1	ENSG00000230911.1
8412	ENSG00000143375.15	ENSG00000272541.1	ENSG00000279040.1
8413	ENSG00000143376.14	ENSG00000272540.1	ENSG00000261385.1
8414	ENSG00000143379.12	ENSG00000272537.1	ENSG00000244065.1
8415	ENSG00000143382.14	ENSG00000272536.2	ENSG00000279354.1
8416	ENSG00000143384.13	ENSG00000272533.1	ENSG00000167749.11
8417	ENSG00000143387.13	ENSG00000272529.1	ENSG00000114019.14
8418	ENSG00000143390.17	ENSG00000272525.1	ENSG00000267723.1
8419	ENSG00000143393.16	ENSG00000272523.1	ENSG00000279872.1
8420	ENSG00000143398.20	ENSG00000272519.1	ENSG00000249834.3
8421	ENSG00000143401.15	ENSG00000272518.1	ENSG00000207425.1
8422	ENSG00000143409.15	ENSG00000272516.1	ENSG00000270381.1
8423	ENSG00000143412.10	ENSG00000272515.1	ENSG00000258780.1
8424	ENSG00000143416.21	ENSG00000272514.6	ENSG00000225117.2

8425	ENSG00000143418.19	ENSG00000272512.1	ENSG00000250174.5
8426	ENSG00000143420.18	ENSG00000272510.1	ENSG00000232190.2
8427	ENSG00000143429.10	ENSG00000272509.1	ENSG00000285675.1
8428	ENSG00000143434.15	ENSG00000272508.1	ENSG00000259342.1
8429	ENSG00000143436.11	ENSG00000272507.1	ENSG00000241622.1
8430	ENSG00000143437.21	ENSG00000272506.1	ENSG00000254132.1
8431	ENSG00000143442.22	ENSG00000272505.1	ENSG00000128285.4
8432	ENSG00000143443.9	ENSG00000272502.1	ENSG00000273112.1
8433	ENSG00000143450.16	ENSG00000272501.1	ENSG00000211961.3
8434	ENSG00000143452.16	ENSG00000272498.1	ENSG00000263177.1
8435	ENSG00000143457.11	ENSG00000272494.1	ENSG00000178462.12
8436	ENSG00000143458.12	ENSG00000272491.1	ENSG00000235578.1
8437	ENSG00000143469.19	ENSG00000272485.1	ENSG00000283422.1
8438	ENSG00000143473.13	ENSG00000272483.1	ENSG00000136152.15
8439	ENSG00000143476.18	ENSG00000272482.1	ENSG00000213935.3
8440	ENSG00000143479.16	ENSG00000272480.1	ENSG00000121101.15
8441	ENSG00000143486.16	ENSG00000272479.1	ENSG00000253621.1
8442	ENSG00000143493.13	ENSG00000272478.1	ENSG00000226863.1
8443	ENSG00000143494.15	ENSG00000272477.1	ENSG00000254488.1
8444	ENSG00000143498.18	ENSG00000272476.1	ENSG00000244314.3
8445	ENSG00000143499.14	ENSG00000272474.1	ENSG00000160013.9
8446	ENSG00000143502.15	ENSG00000272473.1	ENSG00000255000.1
8447	ENSG00000143507.18	ENSG00000272472.1	ENSG00000254522.1
8448	ENSG00000143512.13	ENSG00000272469.2	ENSG00000271676.1
8449	ENSG00000143514.17	ENSG00000272468.1	ENSG00000072952.18
8450	ENSG00000143515.17	ENSG00000272465.1	ENSG00000255351.1
8451	ENSG00000143520.6	ENSG00000272463.1	ENSG00000175390.14
8452	ENSG00000143536.7	ENSG00000272462.2	ENSG00000277327.1
8453	ENSG00000143537.13	ENSG00000272461.1	ENSG00000254743.1
8454	ENSG00000143543.15	ENSG00000272460.1	ENSG00000183979.8
8455	ENSG00000143545.8	ENSG00000272459.1	ENSG00000279806.1
8456	ENSG00000143546.10	ENSG00000272458.1	ENSG00000204173.11
8457	ENSG00000143549.20	ENSG00000272457.1	ENSG00000248618.1
8458	ENSG00000143552.9	ENSG00000272456.1	ENSG00000222383.1
8459	ENSG00000143553.10	ENSG00000272455.1	ENSG00000196274.5
8460	ENSG00000143554.14	ENSG00000272449.2	ENSG00000163792.6
8461	ENSG00000143556.9	ENSG00000272447.1	ENSG00000224448.1
8462	ENSG00000143569.19	ENSG00000272446.5	ENSG00000234880.1
8463	ENSG00000143570.18	ENSG00000272445.1	ENSG00000242163.1
8464	ENSG00000143575.14	ENSG00000272444.1	ENSG00000124568.10
8465	ENSG00000143578.16	ENSG00000272443.1	ENSG00000279815.1
8466	ENSG00000143590.14	ENSG00000272442.2	ENSG00000258308.5
8467	ENSG00000143595.13	ENSG00000272440.1	ENSG00000244055.1
8468	ENSG00000143603.19	ENSG00000272439.1	ENSG00000222854.1
8469	ENSG00000143612.20	ENSG00000272438.1	ENSG00000277144.1
8470	ENSG00000143614.10	ENSG00000272436.1	ENSG00000035862.12
8471	ENSG00000143621.17	ENSG00000272435.1	ENSG00000224895.1
8472	ENSG00000143622.11	ENSG00000272434.1	ENSG00000223336.1
8473	ENSG00000143624.14	ENSG00000272432.1	ENSG00000236863.2
8474	ENSG00000143627.19	ENSG00000272431.1	ENSG00000230189.7
8475	ENSG00000143630.10	ENSG00000272430.1	ENSG00000188662.6
8476	ENSG00000143631.10	ENSG00000272428.1	ENSG00000142731.11
8477	ENSG00000143632.14	ENSG00000272426.1	ENSG00000232696.1

8478	ENSG00000143633.13	ENSG00000272425.1	ENSG00000242431.2
8479	ENSG00000143641.10	ENSG00000272420.1	ENSG00000266369.1
8480	ENSG00000143643.13	ENSG00000272419.6	ENSG00000237975.6
8481	ENSG00000143653.10	ENSG00000272418.1	ENSG00000276519.1
8482	ENSG00000143669.13	ENSG00000272416.1	ENSG00000236263.1
8483	ENSG00000143674.11	ENSG00000272414.6	ENSG00000233524.1
8484	ENSG00000143702.16	ENSG00000272412.2	ENSG00000124749.17
8485	ENSG00000143727.16	ENSG00000272411.1	ENSG00000202255.1
8486	ENSG00000143740.14	ENSG00000272410.5	ENSG00000259415.1
8487	ENSG00000143742.13	ENSG00000272407.1	ENSG00000184423.5
8488	ENSG00000143748.18	ENSG00000272406.1	ENSG00000249736.1
8489	ENSG00000143751.10	ENSG00000272405.1	ENSG00000171459.4
8490	ENSG00000143753.13	ENSG00000272402.1	ENSG00000258618.2
8491	ENSG00000143756.12	ENSG00000272400.1	ENSG00000121005.9
8492	ENSG00000143761.16	ENSG00000272398.6	ENSG00000279652.1
8493	ENSG00000143768.13	ENSG00000272397.1	ENSG00000234848.1
8494	ENSG00000143771.12	ENSG00000272396.1	ENSG00000183154.1
8495	ENSG00000143772.9	ENSG00000272395.7	ENSG00000272372.1
8496	ENSG00000143774.16	ENSG00000272393.1	ENSG00000239533.6
8497	ENSG00000143776.18	ENSG00000272391.6	ENSG00000164076.17
8498	ENSG00000143786.8	ENSG00000272387.2	ENSG00000279289.1
8499	ENSG00000143793.13	ENSG00000272386.1	ENSG00000256050.2
8500	ENSG00000143797.12	ENSG00000272383.1	ENSG00000259982.1
8501	ENSG00000143799.13	ENSG00000272382.1	ENSG00000229044.1
8502	ENSG00000143801.17	ENSG00000272381.1	ENSG00000278505.4
8503	ENSG00000143811.19	ENSG00000272380.1	ENSG00000110719.10
8504	ENSG00000143815.15	ENSG00000272379.1	ENSG00000189375.10
8505	ENSG00000143816.8	ENSG00000272375.1	ENSG00000278733.1
8506	ENSG00000143819.12	ENSG00000272374.1	ENSG00000234584.1
8507	ENSG00000143839.15	ENSG00000272372.1	ENSG00000165480.16
8508	ENSG00000143842.15	ENSG00000272371.1	ENSG00000227133.1
8509	ENSG00000143845.15	ENSG00000272370.1	ENSG00000081985.11
8510	ENSG00000143847.15	ENSG00000272369.1	ENSG00000257979.1
8511	ENSG00000143850.15	ENSG00000272368.2	ENSG00000272483.1
8512	ENSG00000143851.15	ENSG00000272366.1	ENSG00000270212.1
8513	ENSG00000143858.12	ENSG00000272362.1	ENSG00000267692.1
8514	ENSG00000143862.8	ENSG00000272361.2	ENSG00000260211.2
8515	ENSG00000143867.7	ENSG00000272359.1	ENSG00000261065.1
8516	ENSG00000143869.7	ENSG00000272356.1	ENSG00000135924.15
8517	ENSG00000143870.12	ENSG00000272354.1	ENSG00000129354.11
8518	ENSG00000143878.9	ENSG00000272351.1	ENSG00000240003.2
8519	ENSG00000143882.12	ENSG00000272347.1	ENSG00000108840.15
8520	ENSG00000143889.16	ENSG00000272346.2	ENSG00000101251.12
8521	ENSG00000143891.17	ENSG00000272345.1	ENSG00000262837.1
8522	ENSG00000143919.15	ENSG00000272344.1	ENSG00000094804.11
8523	ENSG00000143921.8	ENSG00000272343.1	ENSG00000228901.3
8524	ENSG00000143924.19	ENSG00000272342.1	ENSG00000261393.1
8525	ENSG00000143933.17	ENSG00000272341.1	ENSG00000089159.16
8526	ENSG00000143942.5	ENSG00000272338.2	ENSG00000207332.1
8527	ENSG00000143947.13	ENSG00000272337.1	ENSG00000248469.1
8528	ENSG00000143951.16	ENSG00000272335.1	ENSG00000276470.1
8529	ENSG00000143952.20	ENSG00000272334.1	ENSG00000260865.1
8530	ENSG00000143954.13	ENSG00000272333.5	ENSG00000159231.6

8531	ENSG00000143970.16	ENSG00000272330.1	ENSG00000264391.2
8532	ENSG00000143971.8	ENSG00000272329.1	ENSG00000138356.14
8533	ENSG00000143977.14	ENSG00000272328.1	ENSG00000228919.5
8534	ENSG00000143994.14	ENSG00000272325.2	ENSG00000227170.1
8535	ENSG00000143995.20	ENSG00000272324.5	ENSG00000166206.15
8536	ENSG00000144010.9	ENSG00000272323.1	ENSG00000230204.1
8537	ENSG00000144015.4	ENSG00000272321.1	ENSG00000226419.7
8538	ENSG00000144021.2	ENSG00000272320.1	ENSG00000232387.2
8539	ENSG00000144026.12	ENSG00000272319.2	ENSG00000150676.12
8540	ENSG00000144028.15	ENSG00000272316.1	ENSG00000140299.11
8541	ENSG00000144029.12	ENSG00000272312.1	ENSG00000267429.1
8542	ENSG00000144031.12	ENSG00000272311.1	ENSG00000137193.14
8543	ENSG00000144034.14	ENSG00000272308.1	ENSG00000234235.1
8544	ENSG00000144035.3	ENSG00000272304.1	ENSG00000120262.10
8545	ENSG00000144036.15	ENSG00000272301.1	ENSG00000183850.14
8546	ENSG00000144040.12	ENSG00000272298.1	ENSG00000163661.4
8547	ENSG00000144043.12	ENSG00000272297.2	ENSG00000103507.14
8548	ENSG00000144045.14	ENSG00000272296.1	ENSG00000206624.1
8549	ENSG00000144048.10	ENSG00000272293.1	ENSG00000265222.1
8550	ENSG00000144057.15	ENSG00000272288.5	ENSG00000261235.1
8551	ENSG00000144061.12	ENSG00000272281.6	ENSG00000115414.19
8552	ENSG00000144063.3	ENSG00000272279.1	ENSG00000260412.1
8553	ENSG00000144115.16	ENSG00000272277.1	ENSG00000250476.1
8554	ENSG00000144118.14	ENSG00000272275.1	ENSG00000197444.10
8555	ENSG00000144119.4	ENSG00000272274.1	ENSG00000264125.1
8556	ENSG00000144120.13	ENSG00000272273.1	ENSG00000237676.1
8557	ENSG00000144130.11	ENSG00000272267.2	ENSG00000267520.2
8558	ENSG00000144134.18	ENSG00000272265.1	ENSG00000186019.10
8559	ENSG00000144136.11	ENSG00000272263.1	ENSG00000274471.2
8560	ENSG00000144152.13	ENSG00000272262.1	ENSG00000272695.1
8561	ENSG00000144158.4	ENSG00000272259.5	ENSG00000139364.10
8562	ENSG00000144161.13	ENSG00000272256.1	ENSG00000263563.6
8563	ENSG00000144182.17	ENSG00000272255.1	ENSG00000267784.1
8564	ENSG00000144188.9	ENSG00000272254.1	ENSG00000270789.1
8565	ENSG00000144199.11	ENSG00000272253.1	ENSG00000224014.1
8566	ENSG00000144214.9	ENSG00000272249.1	ENSG00000280036.1
8567	ENSG00000144218.18	ENSG00000272248.1	ENSG00000254786.1
8568	ENSG00000144224.17	ENSG00000272247.1	ENSG00000130035.7
8569	ENSG00000144227.5	ENSG00000272243.5	ENSG00000128335.14
8570	ENSG00000144228.8	ENSG00000272240.1	ENSG00000238149.1
8571	ENSG00000144229.12	ENSG00000272239.1	ENSG00000235411.1
8572	ENSG00000144230.16	ENSG00000272237.1	ENSG00000258485.2
8573	ENSG00000144231.11	ENSG00000272236.1	ENSG00000278151.1
8574	ENSG00000144233.9	ENSG00000272235.1	ENSG00000198822.10
8575	ENSG00000144278.15	ENSG00000272234.1	ENSG00000108639.7
8576	ENSG00000144283.21	ENSG00000272232.2	ENSG00000257258.1
8577	ENSG00000144285.19	ENSG00000272230.1	ENSG00000103175.11
8578	ENSG00000144290.17	ENSG00000272226.1	ENSG00000240520.6
8579	ENSG00000144306.14	ENSG00000272223.1	ENSG00000230623.5
8580	ENSG00000144320.14	ENSG00000272221.1	ENSG00000275609.1
8581	ENSG00000144331.20	ENSG00000272220.1	ENSG00000273573.1
8582	ENSG00000144339.12	ENSG00000272219.1	ENSG00000146426.18
8583	ENSG00000144354.14	ENSG00000272218.1	ENSG00000261248.1

8584	ENSG00000144355.15	ENSG00000272217.1	ENSG00000275381.1
8585	ENSG00000144357.16	ENSG00000272215.1	ENSG00000215580.11
8586	ENSG00000144362.12	ENSG00000272211.1	ENSG00000236937.2
8587	ENSG00000144366.16	ENSG00000272209.1	ENSG00000286030.1
8588	ENSG00000144369.13	ENSG00000272203.1	ENSG00000144834.14
8589	ENSG00000144381.17	ENSG00000272202.1	ENSG00000276692.1
8590	ENSG00000144395.18	ENSG00000272197.2	ENSG00000253120.1
8591	ENSG00000144401.14	ENSG00000272196.2	ENSG00000241183.1
8592	ENSG00000144406.18	ENSG00000272195.1	ENSG00000228293.1
8593	ENSG00000144407.9	ENSG00000272192.1	ENSG00000175336.10
8594	ENSG00000144410.4	ENSG00000272189.1	ENSG00000254639.1
8595	ENSG00000144426.18	ENSG00000272186.1	ENSG00000172995.16
8596	ENSG00000144445.17	ENSG00000272183.1	ENSG00000187242.5
8597	ENSG00000144451.19	ENSG00000272182.1	ENSG00000278399.1
8598	ENSG00000144452.15	ENSG00000272180.1	ENSG00000181392.16
8599	ENSG00000144455.14	ENSG00000272175.1	ENSG00000082126.18
8600	ENSG00000144460.12	ENSG00000272173.1	ENSG00000250507.1
8601	ENSG00000144468.17	ENSG00000272172.1	ENSG00000254680.1
8602	ENSG00000144476.6	ENSG00000272170.1	ENSG00000215301.10
8603	ENSG00000144481.17	ENSG00000272168.7	ENSG00000077616.11
8604	ENSG00000144485.11	ENSG00000272167.2	ENSG00000286254.1
8605	ENSG00000144488.15	ENSG00000272166.1	ENSG00000109851.6
8606	ENSG00000144504.15	ENSG00000272164.1	ENSG00000278267.1
8607	ENSG00000144524.17	ENSG00000272163.1	ENSG00000110583.13
8608	ENSG00000144535.19	ENSG00000272162.1	ENSG00000235151.1
8609	ENSG00000144550.13	ENSG00000272161.1	ENSG00000165197.5
8610	ENSG00000144554.10	ENSG00000272160.1	ENSG00000274008.1
8611	ENSG00000144559.10	ENSG00000272159.1	ENSG00000239453.1
8612	ENSG00000144560.15	ENSG00000272158.1	ENSG00000211827.1
8613	ENSG00000144566.11	ENSG00000272157.1	ENSG00000256603.1
8614	ENSG00000144567.11	ENSG00000272156.1	ENSG00000066455.13
8615	ENSG00000144579.7	ENSG00000272155.1	ENSG00000267356.1
8616	ENSG00000144580.13	ENSG00000272154.4	ENSG00000260528.4
8617	ENSG00000144583.5	ENSG00000272153.1	ENSG00000248830.1
8618	ENSG00000144589.22	ENSG00000272150.5	ENSG00000281189.1
8619	ENSG00000144591.18	ENSG00000272149.1	ENSG00000198414.5
8620	ENSG00000144596.13	ENSG00000272146.5	ENSG00000271078.1
8621	ENSG00000144597.14	ENSG00000272145.1	ENSG00000259277.1
8622	ENSG00000144619.15	ENSG00000272144.1	ENSG00000237861.1
8623	ENSG00000144635.9	ENSG00000272143.1	ENSG00000283697.2
8624	ENSG00000144642.21	ENSG00000272142.1	ENSG00000166927.13
8625	ENSG00000144644.14	ENSG00000272141.1	ENSG00000204516.10
8626	ENSG00000144645.13	ENSG00000272140.2	ENSG00000255014.1
8627	ENSG00000144647.6	ENSG00000272139.1	ENSG00000066651.20
8628	ENSG00000144648.16	ENSG00000272138.1	ENSG00000259502.1
8629	ENSG00000144649.9	ENSG00000272137.1	ENSG00000248930.1
8630	ENSG00000144655.15	ENSG00000272134.1	ENSG00000215057.4
8631	ENSG00000144659.12	ENSG00000272130.1	ENSG00000225779.1
8632	ENSG00000144668.12	ENSG00000272129.1	ENSG00000140995.16
8633	ENSG00000144671.10	ENSG00000272128.1	ENSG00000196376.11
8634	ENSG00000144674.16	ENSG00000272123.1	ENSG00000132122.12
8635	ENSG00000144677.15	ENSG00000272121.1	ENSG00000182871.15
8636	ENSG00000144681.10	ENSG00000272115.1	ENSG00000213293.4

8637	ENSG00000144711.16	ENSG00000272114.1	ENSG00000062716.13
8638	ENSG00000144712.12	ENSG00000272113.2	ENSG00000249230.1
8639	ENSG00000144713.12	ENSG00000272112.1	ENSG00000233003.4
8640	ENSG00000144724.20	ENSG00000272109.1	ENSG00000149554.12
8641	ENSG00000144730.18	ENSG00000272108.1	ENSG00000266909.1
8642	ENSG00000144736.14	ENSG00000272106.1	ENSG00000274341.1
8643	ENSG00000144741.17	ENSG00000272104.1	ENSG00000168118.11
8644	ENSG00000144744.17	ENSG00000272103.2	ENSG00000229792.1
8645	ENSG00000144746.7	ENSG00000272100.1	ENSG00000255500.2
8646	ENSG00000144747.17	ENSG00000272097.1	ENSG00000254898.1
8647	ENSG00000144749.13	ENSG00000272096.2	ENSG00000254992.1
8648	ENSG00000144771.8	ENSG00000272094.1	ENSG00000232751.1
8649	ENSG00000144785.8	ENSG00000272092.1	ENSG00000215146.5
8650	ENSG00000144791.10	ENSG00000272088.1	ENSG00000230490.2
8651	ENSG00000144792.9	ENSG00000272087.1	ENSG00000226352.2
8652	ENSG00000144802.11	ENSG00000272086.1	ENSG00000243979.2
8653	ENSG00000144810.16	ENSG00000272085.1	ENSG00000283544.1
8654	ENSG00000144815.16	ENSG00000272084.1	ENSG00000240199.3
8655	ENSG00000144820.8	ENSG00000272081.1	ENSG00000272646.1
8656	ENSG00000144821.9	ENSG00000272080.1	ENSG00000184844.6
8657	ENSG00000144824.20	ENSG00000272079.2	ENSG00000123338.13
8658	ENSG00000144827.9	ENSG00000272078.1	ENSG00000214435.8
8659	ENSG00000144834.14	ENSG00000272077.1	ENSG00000242706.2
8660	ENSG00000144837.9	ENSG00000272076.1	ENSG00000250304.2
8661	ENSG00000144840.9	ENSG00000272075.2	ENSG00000244604.1
8662	ENSG00000144843.11	ENSG00000272072.1	ENSG00000273138.1
8663	ENSG00000144847.12	ENSG00000272071.1	ENSG00000275945.1
8664	ENSG00000144848.10	ENSG00000272070.1	ENSG00000232837.1
8665	ENSG00000144852.17	ENSG00000272068.1	ENSG00000259467.1
8666	ENSG00000144857.14	ENSG00000272057.1	ENSG00000215005.2
8667	ENSG00000144867.11	ENSG00000272056.1	ENSG00000141431.12
8668	ENSG00000144868.13	ENSG00000272055.1	ENSG00000232177.1
8669	ENSG00000144891.17	ENSG00000272054.1	ENSG00000003147.19
8670	ENSG00000144893.12	ENSG00000272051.1	ENSG00000207635.1
8671	ENSG00000144895.12	ENSG00000272050.1	ENSG00000271664.1
8672	ENSG00000144908.13	ENSG00000272049.1	ENSG00000266824.1
8673	ENSG00000144909.8	ENSG00000272047.2	ENSG00000260284.1
8674	ENSG00000144935.15	ENSG00000272046.1	ENSG00000285641.1
8675	ENSG00000144959.10	ENSG00000272043.1	ENSG00000234650.5
8676	ENSG00000144962.7	ENSG00000272040.1	ENSG00000282668.1
8677	ENSG00000145002.12	ENSG00000272036.1	ENSG00000226024.1
8678	ENSG00000145012.13	ENSG00000272034.1	ENSG00000277952.1
8679	ENSG00000145014.17	ENSG00000272033.1	ENSG00000255367.3
8680	ENSG00000145016.16	ENSG00000272031.3	ENSG00000264701.1
8681	ENSG00000145020.15	ENSG00000272030.1	ENSG00000233602.1
8682	ENSG00000145022.4	ENSG00000272028.1	ENSG00000016602.9
8683	ENSG00000145029.13	ENSG00000272027.2	ENSG00000219201.4
8684	ENSG00000145040.4	ENSG00000272025.1	ENSG00000257851.2
8685	ENSG00000145041.15	ENSG00000272024.1	ENSG00000155975.10
8686	ENSG00000145050.16	ENSG00000272023.1	ENSG00000167618.10
8687	ENSG00000145063.14	ENSG00000272021.1	ENSG00000272568.5
8688	ENSG00000145075.13	ENSG00000272020.1	ENSG00000270610.1
8689	ENSG00000145087.12	ENSG00000272015.1	ENSG00000058404.20

8690	ENSG00000145088.9	ENSG00000272010.1	ENSG00000259215.1
8691	ENSG00000145103.14	ENSG00000272009.1	ENSG00000170498.9
8692	ENSG00000145107.15	ENSG00000272008.1	ENSG00000114923.17
8693	ENSG00000145113.22	ENSG00000272006.1	ENSG00000111247.14
8694	ENSG00000145147.20	ENSG00000272004.1	ENSG00000231140.3
8695	ENSG00000145191.14	ENSG00000272002.1	ENSG00000034152.18
8696	ENSG00000145192.13	ENSG00000271998.1	ENSG00000187682.2
8697	ENSG00000145194.18	ENSG00000271996.1	ENSG00000088881.20
8698	ENSG00000145198.14	ENSG00000271993.1	ENSG00000254827.5
8699	ENSG00000145214.14	ENSG00000271992.1	ENSG00000275995.1
8700	ENSG00000145216.16	ENSG00000271991.1	ENSG00000250012.1
8701	ENSG00000145217.14	ENSG00000271989.1	ENSG00000236358.1
8702	ENSG00000145220.14	ENSG00000271986.2	ENSG00000286234.1
8703	ENSG00000145241.11	ENSG00000271985.1	ENSG00000260882.1
8704	ENSG00000145242.13	ENSG00000271984.1	ENSG00000173376.14
8705	ENSG00000145244.12	ENSG00000271983.1	ENSG00000225636.1
8706	ENSG00000145246.13	ENSG00000271982.1	ENSG00000272196.2
8707	ENSG00000145247.11	ENSG00000271981.1	ENSG00000203414.2
8708	ENSG00000145248.7	ENSG00000271980.1	ENSG00000106302.9
8709	ENSG00000145283.7	ENSG00000271978.1	ENSG00000198538.11
8710	ENSG00000145284.12	ENSG00000271977.1	ENSG00000256518.3
8711	ENSG00000145287.10	ENSG00000271976.1	ENSG00000250869.2
8712	ENSG00000145293.16	ENSG00000271974.1	ENSG00000249664.1
8713	ENSG00000145309.6	ENSG00000271973.1	ENSG00000254185.1
8714	ENSG00000145321.13	ENSG00000271971.1	ENSG00000176435.6
8715	ENSG00000145331.14	ENSG00000271969.1	ENSG00000198300.14
8716	ENSG00000145332.14	ENSG00000271968.2	ENSG00000270076.1
8717	ENSG00000145335.15	ENSG00000271967.1	ENSG00000224316.1
8718	ENSG00000145337.5	ENSG00000271966.1	ENSG00000111877.17
8719	ENSG00000145348.16	ENSG00000271964.1	ENSG00000213509.4
8720	ENSG00000145349.17	ENSG00000271963.1	ENSG00000219039.2
8721	ENSG00000145354.12	ENSG00000271959.1	ENSG00000253965.1
8722	ENSG00000145358.6	ENSG00000271958.1	ENSG00000257146.1
8723	ENSG00000145362.18	ENSG00000271955.2	ENSG00000283752.1
8724	ENSG00000145365.11	ENSG00000271952.1	ENSG00000227421.1
8725	ENSG00000145375.8	ENSG00000271949.1	ENSG00000243900.3
8726	ENSG00000145384.3	ENSG00000271947.1	ENSG00000244331.1
8727	ENSG00000145386.10	ENSG00000271945.1	ENSG00000272459.1
8728	ENSG00000145388.15	ENSG00000271943.2	ENSG00000265746.1
8729	ENSG00000145390.11	ENSG00000271938.1	ENSG00000240392.1
8730	ENSG00000145391.13	ENSG00000271937.1	ENSG00000262223.7
8731	ENSG00000145414.9	ENSG00000271934.2	ENSG00000271468.1
8732	ENSG00000145416.13	ENSG00000271933.1	ENSG00000249487.6
8733	ENSG00000145423.5	ENSG00000271932.1	ENSG00000272203.1
8734	ENSG00000145425.10	ENSG00000271931.1	ENSG00000155729.13
8735	ENSG00000145428.14	ENSG00000271930.1	ENSG00000245213.6
8736	ENSG00000145431.11	ENSG00000271926.1	ENSG00000137675.4
8737	ENSG00000145439.12	ENSG00000271924.1	ENSG00000213041.4
8738	ENSG00000145451.13	ENSG00000271923.1	ENSG00000273897.1
8739	ENSG00000145476.16	ENSG00000271922.1	ENSG00000273079.5
8740	ENSG00000145491.12	ENSG00000271919.1	ENSG00000231965.4
8741	ENSG00000145494.11	ENSG00000271918.1	ENSG00000273891.1
8742	ENSG00000145495.16	ENSG00000271917.1	ENSG00000182583.12

8743	ENSG00000145506.13	ENSG00000271916.1	ENSG00000284012.1
8744	ENSG00000145526.12	ENSG00000271914.1	ENSG00000241587.3
8745	ENSG00000145536.15	ENSG00000271913.6	ENSG00000235974.1
8746	ENSG00000145545.12	ENSG00000271911.1	ENSG00000235559.1
8747	ENSG00000145555.15	ENSG00000271907.1	ENSG00000259627.1
8748	ENSG00000145569.6	ENSG00000271904.1	ENSG00000259883.1
8749	ENSG00000145592.14	ENSG00000271901.1	ENSG00000271198.1
8750	ENSG00000145604.15	ENSG00000271900.1	ENSG00000232803.1
8751	ENSG00000145623.13	ENSG00000271899.1	ENSG00000240791.1
8752	ENSG00000145626.11	ENSG00000271898.1	ENSG00000189433.6
8753	ENSG00000145632.15	ENSG00000271897.1	ENSG00000140386.13
8754	ENSG00000145642.12	ENSG00000271895.2	ENSG00000270437.1
8755	ENSG00000145649.8	ENSG00000271894.1	ENSG00000234389.1
8756	ENSG00000145675.15	ENSG00000271893.1	ENSG00000229671.2
8757	ENSG00000145681.10	ENSG00000271892.1	ENSG00000259109.1
8758	ENSG00000145685.14	ENSG00000271890.1	ENSG00000233651.2
8759	ENSG00000145687.16	ENSG00000271889.1	ENSG00000236155.6
8760	ENSG00000145692.15	ENSG00000271888.1	ENSG00000275339.1
8761	ENSG00000145700.9	ENSG00000271886.1	ENSG00000220237.1
8762	ENSG00000145703.16	ENSG00000271882.1	ENSG00000249077.1
8763	ENSG00000145708.11	ENSG00000271880.1	ENSG00000243444.8
8764	ENSG00000145715.14	ENSG00000271874.1	ENSG00000225192.1
8765	ENSG00000145721.12	ENSG00000271871.1	ENSG00000112977.15
8766	ENSG00000145723.17	ENSG00000271869.1	ENSG00000046889.19
8767	ENSG00000145725.19	ENSG00000271868.1	ENSG00000231332.2
8768	ENSG00000145730.20	ENSG00000271862.1	ENSG00000270711.1
8769	ENSG00000145734.19	ENSG00000271860.6	ENSG00000268696.2
8770	ENSG00000145736.14	ENSG00000271858.5	ENSG00000280070.1
8771	ENSG00000145740.19	ENSG00000271857.1	ENSG00000248696.1
8772	ENSG00000145741.15	ENSG00000271856.1	ENSG00000251819.1
8773	ENSG00000145743.15	ENSG00000271855.1	ENSG00000176716.5
8774	ENSG00000145757.16	ENSG00000271853.5	ENSG00000010932.17
8775	ENSG00000145777.15	ENSG00000271852.1	ENSG00000229955.1
8776	ENSG00000145779.8	ENSG00000271850.1	ENSG00000214366.4
8777	ENSG00000145780.8	ENSG00000271849.1	ENSG00000260455.1
8778	ENSG00000145781.9	ENSG00000271848.1	ENSG00000104901.6
8779	ENSG00000145782.13	ENSG00000271843.1	ENSG00000285804.1
8780	ENSG00000145794.17	ENSG00000271841.1	ENSG00000248654.1
8781	ENSG00000145808.10	ENSG00000271833.1	ENSG00000242516.1
8782	ENSG00000145817.17	ENSG00000271830.1	ENSG00000283108.1
8783	ENSG00000145819.17	ENSG00000271828.1	ENSG00000181929.12
8784	ENSG00000145824.12	ENSG00000271826.5	ENSG00000229956.10
8785	ENSG00000145826.9	ENSG00000271825.1	ENSG00000213753.11
8786	ENSG00000145832.14	ENSG00000271824.1	ENSG00000261217.2
8787	ENSG00000145833.16	ENSG00000271821.1	ENSG00000267052.2
8788	ENSG00000145835.6	ENSG00000271820.1	ENSG00000224794.2
8789	ENSG00000145839.1	ENSG00000271819.1	ENSG00000213793.5
8790	ENSG00000145850.9	ENSG00000271818.1	ENSG00000240828.1
8791	ENSG00000145860.11	ENSG00000271817.2	ENSG00000255959.1
8792	ENSG00000145861.8	ENSG00000271816.1	ENSG00000005007.12
8793	ENSG00000145863.11	ENSG00000271815.1	ENSG00000285749.1
8794	ENSG00000145864.12	ENSG00000271814.1	ENSG00000251226.1
8795	ENSG00000145868.16	ENSG00000271811.1	ENSG00000277950.1

8796	ENSG00000145879.11	ENSG00000271810.5	ENSG00000179447.2
8797	ENSG00000145882.11	ENSG00000271806.1	ENSG00000257194.2
8798	ENSG00000145888.10	ENSG00000271803.1	ENSG00000146216.13
8799	ENSG00000145901.15	ENSG00000271798.1	ENSG00000182568.17
8800	ENSG00000145907.15	ENSG00000271797.1	ENSG00000260213.6
8801	ENSG00000145908.12	ENSG00000271795.1	ENSG00000276393.1
8802	ENSG00000145911.6	ENSG00000271793.1	ENSG00000088926.13
8803	ENSG00000145912.8	ENSG00000271792.1	ENSG00000249007.1
8804	ENSG00000145916.19	ENSG00000271788.1	ENSG00000267298.1
8805	ENSG00000145919.10	ENSG00000271784.1	ENSG00000244009.1
8806	ENSG00000145920.15	ENSG00000271781.1	ENSG00000253490.5
8807	ENSG00000145934.16	ENSG00000271779.1	ENSG00000236745.2
8808	ENSG00000145936.9	ENSG00000271778.1	ENSG00000004838.14
8809	ENSG00000145945.6	ENSG00000271776.1	ENSG00000173542.8
8810	ENSG00000145949.10	ENSG00000271774.1	ENSG00000240927.3
8811	ENSG00000145975.14	ENSG00000271771.1	ENSG00000222035.3
8812	ENSG00000145979.18	ENSG00000271766.1	ENSG00000258729.2
8813	ENSG00000145982.12	ENSG00000271765.1	ENSG00000259394.2
8814	ENSG00000145990.11	ENSG00000271763.1	ENSG00000223484.7
8815	ENSG00000145996.11	ENSG00000271761.1	ENSG00000228339.1
8816	ENSG00000146001.5	ENSG00000271758.2	ENSG00000274841.1
8817	ENSG00000146005.4	ENSG00000271757.1	ENSG00000261616.1
8818	ENSG00000146006.8	ENSG00000271755.1	ENSG00000246774.1
8819	ENSG00000146007.10	ENSG00000271754.1	ENSG00000267125.2
8820	ENSG00000146013.11	ENSG00000271752.1	ENSG00000225108.1
8821	ENSG00000146021.15	ENSG00000271751.1	ENSG00000241295.1
8822	ENSG00000146038.12	ENSG00000271749.1	ENSG00000228843.2
8823	ENSG00000146039.10	ENSG00000271746.1	ENSG00000286257.1
8824	ENSG00000146047.6	ENSG00000271743.1	ENSG00000213406.3
8825	ENSG00000146049.1	ENSG00000271742.1	ENSG00000162779.22
8826	ENSG00000146054.18	ENSG00000271741.1	ENSG00000135838.13
8827	ENSG00000146063.20	ENSG00000271739.1	ENSG00000275944.1
8828	ENSG00000146066.3	ENSG00000271737.1	ENSG00000242882.1
8829	ENSG00000146067.16	ENSG00000271734.1	ENSG00000238172.2
8830	ENSG00000146070.16	ENSG00000271732.1	ENSG00000255477.1
8831	ENSG00000146072.6	ENSG00000271730.1	ENSG00000242894.3
8832	ENSG00000146083.12	ENSG00000271727.1	ENSG00000180245.5
8833	ENSG00000146085.8	ENSG00000271725.1	ENSG00000272033.1
8834	ENSG00000146090.16	ENSG00000271724.1	ENSG00000147454.14
8835	ENSG00000146094.14	ENSG00000271723.5	ENSG00000205212.4
8836	ENSG00000146109.5	ENSG00000271717.1	ENSG00000228754.1
8837	ENSG00000146112.12	ENSG00000271716.1	ENSG00000168152.13
8838	ENSG00000146122.17	ENSG00000271715.1	ENSG00000246250.2
8839	ENSG00000146143.18	ENSG00000271714.1	ENSG00000243883.3
8840	ENSG00000146147.15	ENSG00000271712.1	ENSG00000273523.1
8841	ENSG00000146151.13	ENSG00000271711.1	ENSG00000214872.8
8842	ENSG00000146166.16	ENSG00000271710.1	ENSG00000283566.2
8843	ENSG00000146192.15	ENSG00000271709.1	ENSG00000117505.13
8844	ENSG00000146197.9	ENSG00000271707.1	ENSG00000234361.1
8845	ENSG00000146205.13	ENSG00000271705.1	ENSG00000250739.1
8846	ENSG00000146215.13	ENSG00000271704.1	ENSG00000215065.3
8847	ENSG00000146216.13	ENSG00000271702.1	ENSG00000284594.1
8848	ENSG00000146221.10	ENSG00000271701.1	ENSG00000249753.2

8849	ENSG00000146223.14	ENSG00000271699.5	ENSG00000206913.1
8850	ENSG00000146232.16	ENSG00000271698.1	ENSG00000227986.1
8851	ENSG00000146233.8	ENSG00000271697.1	ENSG00000072756.17
8852	ENSG00000146242.8	ENSG00000271696.1	ENSG00000255726.1
8853	ENSG00000146243.14	ENSG00000271693.1	ENSG00000168131.4
8854	ENSG00000146247.14	ENSG00000271691.1	ENSG00000270052.1
8855	ENSG00000146250.7	ENSG00000271687.1	ENSG00000224645.1
8856	ENSG00000146263.11	ENSG00000271686.1	ENSG00000252667.1
8857	ENSG00000146267.12	ENSG00000271682.1	ENSG00000271680.1
8858	ENSG00000146276.11	ENSG00000271681.1	ENSG00000166159.11
8859	ENSG00000146278.10	ENSG00000271680.1	ENSG00000270135.1
8860	ENSG00000146281.6	ENSG00000271679.1	ENSG00000278351.1
8861	ENSG00000146282.18	ENSG00000271677.1	ENSG00000250156.3
8862	ENSG00000146285.14	ENSG00000271676.1	ENSG00000035681.9
8863	ENSG00000146350.14	ENSG00000271672.1	ENSG00000229543.1
8864	ENSG00000146352.13	ENSG00000271671.1	ENSG00000249614.1
8865	ENSG00000146360.8	ENSG00000271670.1	ENSG00000279600.1
8866	ENSG00000146373.16	ENSG00000271667.1	ENSG00000266944.1
8867	ENSG00000146374.14	ENSG00000271666.1	ENSG00000214022.11
8868	ENSG00000146376.11	ENSG00000271664.1	ENSG00000232429.3
8869	ENSG00000146378.6	ENSG00000271662.1	ENSG00000204380.4
8870	ENSG00000146383.7	ENSG00000271661.1	ENSG00000225787.1
8871	ENSG00000146385.1	ENSG00000271660.1	ENSG00000231066.3
8872	ENSG00000146386.8	ENSG00000271659.1	ENSG00000006210.7
8873	ENSG00000146399.1	ENSG00000271657.1	ENSG00000105851.11
8874	ENSG00000146409.12	ENSG00000271656.1	ENSG00000272088.1
8875	ENSG00000146410.12	ENSG00000271655.1	ENSG00000236411.1
8876	ENSG00000146411.5	ENSG00000271654.1	ENSG00000159189.12
8877	ENSG00000146414.16	ENSG00000271653.1	ENSG00000213470.4
8878	ENSG00000146416.18	ENSG00000271652.1	ENSG00000242398.3
8879	ENSG00000146425.11	ENSG00000271650.1	ENSG00000267477.1
8880	ENSG00000146426.18	ENSG00000271649.1	ENSG00000228380.1
8881	ENSG00000146433.8	ENSG00000271647.1	ENSG00000242276.2
8882	ENSG00000146453.13	ENSG00000271646.1	ENSG00000233246.1
8883	ENSG00000146457.16	ENSG00000271644.1	ENSG00000217231.2
8884	ENSG00000146463.11	ENSG00000271642.1	ENSG00000181013.3
8885	ENSG00000146469.13	ENSG00000271639.1	ENSG00000239809.1
8886	ENSG00000146476.11	ENSG00000271638.1	ENSG00000223324.1
8887	ENSG00000146477.6	ENSG00000271635.1	ENSG00000237471.1
8888	ENSG00000146521.9	ENSG00000271632.1	ENSG00000279791.1
8889	ENSG00000146530.14	ENSG00000271631.1	ENSG00000285446.1
8890	ENSG00000146535.14	ENSG00000271629.1	ENSG00000243422.2
8891	ENSG00000146540.15	ENSG00000271627.1	ENSG00000243455.3
8892	ENSG00000146555.19	ENSG00000271626.1	ENSG00000254905.1
8893	ENSG00000146556.14	ENSG00000271625.1	ENSG00000229858.2
8894	ENSG00000146574.15	ENSG00000271624.1	ENSG00000206560.11
8895	ENSG00000146576.13	ENSG00000271623.1	ENSG00000262974.1
8896	ENSG00000146587.18	ENSG00000271621.1	ENSG00000105392.16
8897	ENSG00000146592.17	ENSG00000271620.1	ENSG00000075975.16
8898	ENSG00000146618.3	ENSG00000271618.1	ENSG00000091428.18
8899	ENSG00000146648.18	ENSG00000271616.1	ENSG00000138642.14
8900	ENSG00000146666.5	ENSG00000271615.1	ENSG00000177679.16
8901	ENSG00000146670.10	ENSG00000271614.1	ENSG00000186777.11

8902	ENSG00000146674.15	ENSG00000271612.1	ENSG00000073598.6
8903	ENSG00000146676.9	ENSG00000271611.1	ENSG00000283486.2
8904	ENSG00000146677.7	ENSG00000271609.1	ENSG00000241035.2
8905	ENSG00000146678.10	ENSG00000271608.1	ENSG00000074181.9
8906	ENSG00000146700.9	ENSG00000271607.1	ENSG00000281883.1
8907	ENSG00000146701.12	ENSG00000271606.1	ENSG00000245281.7
8908	ENSG00000146707.14	ENSG00000271605.6	ENSG00000226436.1
8909	ENSG00000146722.11	ENSG00000271602.1	ENSG00000187796.14
8910	ENSG00000146729.10	ENSG00000271601.4	ENSG00000168124.2
8911	ENSG00000146731.11	ENSG00000271600.1	ENSG00000270883.1
8912	ENSG00000146733.14	ENSG00000271598.1	ENSG00000236663.1
8913	ENSG00000146755.10	ENSG00000271597.1	ENSG00000230171.1
8914	ENSG00000146757.13	ENSG00000271596.1	ENSG00000236299.7
8915	ENSG00000146776.14	ENSG00000271595.1	ENSG00000233560.2
8916	ENSG00000146802.12	ENSG00000271590.1	ENSG00000255394.4
8917	ENSG00000146809.13	ENSG00000271589.1	ENSG00000132376.20
8918	ENSG00000146826.17	ENSG00000271588.1	ENSG00000206147.5
8919	ENSG00000146828.18	ENSG00000271587.1	ENSG00000167461.12
8920	ENSG00000146830.10	ENSG00000271586.1	ENSG00000148450.13
8921	ENSG00000146833.15	ENSG00000271585.1	ENSG00000224818.1
8922	ENSG00000146834.14	ENSG00000271584.2	ENSG00000228797.2
8923	ENSG00000146839.18	ENSG00000271583.1	ENSG00000170967.4
8924	ENSG00000146842.17	ENSG00000271582.1	ENSG00000131002.12
8925	ENSG00000146856.14	ENSG00000271581.1	ENSG00000243053.2
8926	ENSG00000146857.3	ENSG00000271579.1	ENSG00000224986.2
8927	ENSG00000146858.8	ENSG00000271578.1	ENSG00000234099.1
8928	ENSG00000146859.6	ENSG00000271576.1	ENSG00000108176.15
8929	ENSG00000146872.17	ENSG00000271573.1	ENSG00000054356.14
8930	ENSG00000146904.9	ENSG00000271569.1	ENSG00000273474.1
8931	ENSG00000146909.8	ENSG00000271568.1	ENSG00000231563.1
8932	ENSG00000146910.12	ENSG00000271565.1	ENSG00000154102.11
8933	ENSG00000146918.19	ENSG00000271564.1	ENSG00000240405.6
8934	ENSG00000146926.11	ENSG00000271563.1	ENSG00000243771.1
8935	ENSG00000146938.16	ENSG00000271560.1	ENSG00000252301.1
8936	ENSG00000146950.13	ENSG00000271559.1	ENSG00000197077.13
8937	ENSG00000146955.10	ENSG00000271558.1	ENSG00000272967.1
8938	ENSG00000146963.18	ENSG00000271557.1	ENSG00000253309.6
8939	ENSG00000146966.13	ENSG00000271555.1	ENSG00000254699.1
8940	ENSG00000147003.7	ENSG00000271554.1	ENSG00000284689.1
8941	ENSG00000147010.18	ENSG00000271553.1	ENSG00000231849.1
8942	ENSG00000147027.4	ENSG00000271551.2	ENSG00000237359.1
8943	ENSG00000147036.11	ENSG00000271550.1	ENSG00000143319.16
8944	ENSG00000147041.12	ENSG00000271547.1	ENSG00000211597.2
8945	ENSG00000147044.21	ENSG00000271546.1	ENSG00000228716.7
8946	ENSG00000147050.14	ENSG00000271544.1	ENSG00000135677.11
8947	ENSG00000147059.8	ENSG00000271543.1	ENSG00000272862.1
8948	ENSG00000147065.17	ENSG00000271538.5	ENSG00000258857.1
8949	ENSG00000147081.15	ENSG00000271537.1	ENSG00000274997.1
8950	ENSG00000147082.17	ENSG00000271536.1	ENSG00000229979.1
8951	ENSG00000147099.21	ENSG00000271533.1	ENSG00000158158.12
8952	ENSG00000147100.11	ENSG00000271532.1	ENSG00000263639.6
8953	ENSG00000147113.17	ENSG00000271530.1	ENSG00000272079.2
8954	ENSG00000147117.7	ENSG00000271527.1	ENSG00000240554.1

8955	ENSG00000147118.11	ENSG00000271526.1	ENSG00000271911.1
8956	ENSG00000147119.3	ENSG00000271525.1	ENSG00000137955.16
8957	ENSG00000147121.16	ENSG00000271524.1	ENSG00000259707.1
8958	ENSG00000147123.10	ENSG00000271523.1	ENSG00000227183.3
8959	ENSG00000147124.12	ENSG00000271522.1	ENSG00000178096.9
8960	ENSG00000147127.8	ENSG00000271519.1	ENSG00000187504.6
8961	ENSG00000147130.14	ENSG00000271511.1	ENSG00000255378.1
8962	ENSG00000147133.15	ENSG00000271509.1	ENSG00000283886.2
8963	ENSG00000147138.2	ENSG00000271508.1	ENSG00000167964.12
8964	ENSG00000147140.16	ENSG00000271507.1	ENSG00000270975.1
8965	ENSG00000147144.13	ENSG00000271503.6	ENSG00000213526.3
8966	ENSG00000147145.12	ENSG00000271502.1	ENSG00000223429.1
8967	ENSG00000147155.11	ENSG00000271500.1	ENSG00000184698.5
8968	ENSG00000147160.9	ENSG00000271499.1	ENSG00000250432.5
8969	ENSG00000147162.14	ENSG00000271498.1	ENSG00000273609.1
8970	ENSG00000147164.12	ENSG00000271496.1	ENSG00000267750.5
8971	ENSG00000147166.11	ENSG00000271495.1	ENSG00000267476.1
8972	ENSG00000147168.12	ENSG00000271494.1	ENSG00000228328.2
8973	ENSG00000147174.11	ENSG00000271492.1	ENSG00000268015.1
8974	ENSG00000147180.16	ENSG00000271491.1	ENSG00000112062.10
8975	ENSG00000147183.9	ENSG00000271490.1	ENSG00000158161.16
8976	ENSG00000147202.18	ENSG00000271488.1	ENSG00000228334.1
8977	ENSG00000147206.17	ENSG00000271486.1	ENSG00000270174.1
8978	ENSG00000147223.6	ENSG00000271484.1	ENSG00000256417.1
8979	ENSG00000147224.11	ENSG00000271483.1	ENSG00000277550.1
8980	ENSG00000147231.14	ENSG00000271482.1	ENSG00000226545.1
8981	ENSG00000147234.10	ENSG00000271480.1	ENSG00000198723.11
8982	ENSG00000147246.10	ENSG00000271477.1	ENSG00000213153.3
8983	ENSG00000147251.15	ENSG00000271475.1	ENSG00000184224.3
8984	ENSG00000147255.19	ENSG00000271474.1	ENSG00000240766.1
8985	ENSG00000147256.12	ENSG00000271468.1	ENSG00000271350.1
8986	ENSG00000147257.13	ENSG00000271466.1	ENSG00000215612.8
8987	ENSG00000147262.4	ENSG00000271465.1	ENSG00000271952.1
8988	ENSG00000147274.14	ENSG00000271464.1	ENSG00000253497.1
8989	ENSG00000147316.13	ENSG00000271462.1	ENSG00000250105.1
8990	ENSG00000147324.11	ENSG00000271461.1	ENSG00000227220.1
8991	ENSG00000147364.16	ENSG00000271459.1	ENSG00000230988.3
8992	ENSG00000147378.11	ENSG00000271457.1	ENSG00000170323.8
8993	ENSG00000147381.11	ENSG00000271456.1	ENSG00000232362.1
8994	ENSG00000147383.11	ENSG00000271455.1	ENSG00000285254.1
8995	ENSG00000147394.18	ENSG00000271454.1	ENSG00000279526.1
8996	ENSG00000147400.8	ENSG00000271452.1	ENSG00000249849.1
8997	ENSG00000147403.16	ENSG00000271449.7	ENSG00000266929.1
8998	ENSG00000147408.14	ENSG00000271447.6	ENSG00000279724.1
8999	ENSG00000147416.11	ENSG00000271443.1	ENSG00000226493.1
9000	ENSG00000147419.18	ENSG00000271440.1	ENSG00000184900.15
9001	ENSG00000147421.18	ENSG00000271439.1	ENSG00000113719.16
9002	ENSG00000147432.6	ENSG00000271437.1	ENSG00000204463.12
9003	ENSG00000147434.8	ENSG00000271435.1	ENSG00000130988.13
9004	ENSG00000147437.10	ENSG00000271434.3	ENSG00000223390.1
9005	ENSG00000147439.12	ENSG00000271433.1	ENSG00000076003.5
9006	ENSG00000147443.13	ENSG00000271429.1	ENSG00000253598.1
9007	ENSG00000147454.14	ENSG00000271428.1	ENSG00000136918.7

9008	ENSG00000147457.14	ENSG00000271427.1	ENSG00000264448.5
9009	ENSG00000147459.18	ENSG00000271426.1	ENSG00000277050.1
9010	ENSG00000147465.11	ENSG00000271425.8	ENSG00000230711.2
9011	ENSG00000147471.12	ENSG00000271424.1	ENSG00000103942.13
9012	ENSG00000147475.15	ENSG00000271423.1	ENSG00000201772.1
9013	ENSG00000147481.16	ENSG00000271421.1	ENSG00000256811.1
9014	ENSG00000147485.13	ENSG00000271420.1	ENSG00000267082.1
9015	ENSG00000147488.11	ENSG00000271419.1	ENSG00000236083.2
9016	ENSG00000147509.14	ENSG00000271418.1	ENSG00000254653.1
9017	ENSG00000147526.20	ENSG00000271415.1	ENSG00000256299.1
9018	ENSG00000147533.16	ENSG00000271414.1	ENSG00000055044.11
9019	ENSG00000147535.17	ENSG00000271413.1	ENSG00000258962.2
9020	ENSG00000147536.12	ENSG00000271412.1	ENSG00000223417.8
9021	ENSG00000147548.17	ENSG00000271410.1	ENSG00000234017.1
9022	ENSG00000147570.9	ENSG00000271409.1	ENSG00000242880.1
9023	ENSG00000147571.5	ENSG00000271408.1	ENSG00000232398.1
9024	ENSG00000147573.17	ENSG00000271404.1	ENSG00000236555.1
9025	ENSG00000147576.17	ENSG00000271402.1	ENSG00000243480.7
9026	ENSG00000147586.10	ENSG00000271401.1	ENSG00000203506.5
9027	ENSG00000147588.7	ENSG00000271400.1	ENSG00000280037.1
9028	ENSG00000147592.9	ENSG00000271399.1	ENSG00000227890.1
9029	ENSG00000147596.3	ENSG00000271398.1	ENSG00000271821.1
9030	ENSG00000147601.14	ENSG00000271397.1	ENSG00000237390.1
9031	ENSG00000147604.14	ENSG00000271396.1	ENSG00000286148.1
9032	ENSG00000147606.9	ENSG00000271395.1	ENSG00000248781.1
9033	ENSG00000147613.7	ENSG00000271394.1	ENSG00000253227.1
9034	ENSG00000147614.4	ENSG00000271392.1	ENSG00000250563.1
9035	ENSG00000147642.17	ENSG00000271390.1	ENSG00000268949.1
9036	ENSG00000147647.13	ENSG00000271389.1	ENSG00000181333.11
9037	ENSG00000147649.10	ENSG00000271387.1	ENSG00000250687.6
9038	ENSG00000147650.11	ENSG00000271386.1	ENSG00000225393.1
9039	ENSG00000147654.15	ENSG00000271385.1	ENSG00000183733.6
9040	ENSG00000147655.11	ENSG00000271384.1	ENSG00000205628.3
9041	ENSG00000147669.11	ENSG00000271383.7	ENSG00000260943.1
9042	ENSG00000147676.14	ENSG00000271382.1	ENSG00000267561.2
9043	ENSG00000147677.11	ENSG00000271381.2	ENSG00000023171.18
9044	ENSG00000147679.12	ENSG00000271380.1	ENSG00000226699.1
9045	ENSG00000147684.9	ENSG00000271379.1	ENSG00000253687.1
9046	ENSG00000147687.19	ENSG00000271378.1	ENSG00000259354.5
9047	ENSG00000147689.16	ENSG00000271377.1	ENSG00000253435.1
9048	ENSG00000147697.9	ENSG00000271375.1	ENSG00000277035.1
9049	ENSG00000147724.12	ENSG00000271373.1	ENSG00000230484.2
9050	ENSG00000147753.9	ENSG00000271370.1	ENSG00000205086.7
9051	ENSG00000147761.8	ENSG00000271369.1	ENSG00000166340.17
9052	ENSG00000147789.15	ENSG00000271368.1	ENSG00000259307.1
9053	ENSG00000147799.11	ENSG00000271367.1	ENSG00000102271.14
9054	ENSG00000147804.9	ENSG00000271366.1	ENSG00000267138.1
9055	ENSG00000147813.16	ENSG00000271365.1	ENSG00000252634.1
9056	ENSG00000147852.16	ENSG00000271364.1	ENSG00000254254.5
9057	ENSG00000147853.17	ENSG00000271362.1	ENSG00000069702.11
9058	ENSG00000147854.17	ENSG00000271361.1	ENSG00000206695.1
9059	ENSG00000147862.17	ENSG00000271360.1	ENSG00000228485.1
9060	ENSG00000147869.4	ENSG00000271358.1	ENSG00000268129.1

9061	ENSG00000147872.10	ENSG00000271357.1	ENSG00000186790.6
9062	ENSG00000147873.5	ENSG00000271356.1	ENSG00000182625.3
9063	ENSG00000147874.11	ENSG00000271355.1	ENSG00000230651.7
9064	ENSG00000147883.11	ENSG00000271353.1	ENSG00000156239.12
9065	ENSG00000147885.4	ENSG00000271351.1	ENSG00000179314.14
9066	ENSG00000147889.17	ENSG00000271350.1	ENSG00000116885.18
9067	ENSG00000147894.15	ENSG00000271349.1	ENSG00000263155.6
9068	ENSG00000147896.3	ENSG00000271346.1	ENSG00000280164.1
9069	ENSG00000147905.17	ENSG00000271344.1	ENSG00000225342.2
9070	ENSG00000147912.13	ENSG00000271343.1	ENSG00000227270.1
9071	ENSG00000147955.17	ENSG00000271340.1	ENSG00000251250.3
9072	ENSG00000147996.16	ENSG00000271339.1	ENSG00000271127.1
9073	ENSG00000148019.13	ENSG00000271338.1	ENSG00000137821.11
9074	ENSG00000148053.16	ENSG00000271337.1	ENSG00000231183.3
9075	ENSG00000148057.16	ENSG00000271336.1	ENSG00000267005.1
9076	ENSG00000148082.10	ENSG00000271335.5	ENSG00000242651.3
9077	ENSG00000148090.11	ENSG00000271334.5	ENSG00000226777.7
9078	ENSG00000148110.15	ENSG00000271333.1	ENSG00000274512.5
9079	ENSG00000148120.16	ENSG00000271332.1	ENSG00000266754.2
9080	ENSG00000148123.15	ENSG00000271330.1	ENSG00000143337.18
9081	ENSG00000148136.5	ENSG00000271329.1	ENSG00000229255.1
9082	ENSG00000148143.13	ENSG00000271328.1	ENSG00000258676.4
9083	ENSG00000148153.14	ENSG00000271327.1	ENSG00000267651.1
9084	ENSG00000148154.10	ENSG00000271322.1	ENSG00000273384.1
9085	ENSG00000148156.7	ENSG00000271321.1	ENSG00000275034.2
9086	ENSG00000148158.17	ENSG00000271320.1	ENSG00000256358.1
9087	ENSG00000148175.12	ENSG00000271318.1	ENSG00000279976.1
9088	ENSG00000148180.19	ENSG00000271317.1	ENSG00000272804.3
9089	ENSG00000148187.18	ENSG00000271315.1	ENSG00000260796.1
9090	ENSG00000148200.17	ENSG00000271314.1	ENSG00000177151.4
9091	ENSG00000148204.12	ENSG00000271313.1	ENSG00000229169.1
9092	ENSG00000148215.4	ENSG00000271309.1	ENSG00000260909.1
9093	ENSG00000148218.16	ENSG00000271307.3	ENSG00000257281.1
9094	ENSG00000148219.16	ENSG00000271306.1	ENSG00000280002.1
9095	ENSG00000148225.16	ENSG00000271304.2	ENSG00000206069.6
9096	ENSG00000148229.13	ENSG00000271303.1	ENSG00000236542.1
9097	ENSG00000148248.14	ENSG00000271302.1	ENSG00000142748.12
9098	ENSG00000148288.13	ENSG00000271299.1	ENSG00000164045.11
9099	ENSG00000148290.10	ENSG00000271298.1	ENSG00000203650.8
9100	ENSG00000148291.10	ENSG00000271296.1	ENSG00000160218.13
9101	ENSG00000148296.7	ENSG00000271291.1	ENSG00000286000.1
9102	ENSG00000148297.15	ENSG00000271290.1	ENSG00000242534.2
9103	ENSG00000148300.12	ENSG00000271288.1	ENSG00000249069.7
9104	ENSG00000148303.17	ENSG00000271287.1	ENSG00000263427.1
9105	ENSG00000148308.17	ENSG00000271286.1	ENSG00000273232.1
9106	ENSG00000148331.12	ENSG00000271284.1	ENSG00000242142.1
9107	ENSG00000148334.15	ENSG00000271283.1	ENSG00000268818.2
9108	ENSG00000148335.14	ENSG00000271278.1	ENSG00000213997.3
9109	ENSG00000148337.21	ENSG00000271277.1	ENSG00000255422.3
9110	ENSG00000148339.12	ENSG00000271275.1	ENSG00000270038.1
9111	ENSG00000148341.17	ENSG00000271272.1	ENSG00000217041.1
9112	ENSG00000148343.18	ENSG00000271271.5	ENSG00000120158.12
9113	ENSG00000148344.11	ENSG00000271270.6	ENSG00000237682.2

9114	ENSG00000148346.12	ENSG00000271269.1	ENSG00000132965.9
9115	ENSG00000148356.13	ENSG00000271268.1	ENSG00000229795.2
9116	ENSG00000148357.16	ENSG00000271267.1	ENSG00000236968.1
9117	ENSG00000148358.19	ENSG00000271266.1	ENSG00000248578.1
9118	ENSG00000148362.11	ENSG00000271265.1	ENSG00000269058.5
9119	ENSG00000148377.6	ENSG00000271264.1	ENSG00000231754.2
9120	ENSG00000148384.13	ENSG00000271259.1	ENSG00000279067.1
9121	ENSG00000148386.9	ENSG00000271257.1	ENSG00000261054.1
9122	ENSG00000148396.18	ENSG00000271254.6	ENSG00000226889.3
9123	ENSG00000148399.13	ENSG00000271253.1	ENSG00000215480.4
9124	ENSG00000148400.11	ENSG00000271252.1	ENSG00000136492.8
9125	ENSG00000148408.13	ENSG00000271251.1	ENSG00000273598.1
9126	ENSG00000148411.8	ENSG00000271250.1	ENSG00000154914.17
9127	ENSG00000148426.13	ENSG00000271249.1	ENSG00000215160.3
9128	ENSG00000148429.14	ENSG00000271248.1	ENSG00000206052.11
9129	ENSG00000148444.16	ENSG00000271245.1	ENSG00000223864.1
9130	ENSG00000148450.13	ENSG00000271243.1	ENSG00000007402.11
9131	ENSG00000148459.16	ENSG00000271242.1	ENSG00000266933.2
9132	ENSG00000148468.17	ENSG00000271240.1	ENSG00000261780.2
9133	ENSG00000148481.14	ENSG00000271239.1	ENSG00000126856.14
9134	ENSG00000148482.12	ENSG00000271238.1	ENSG00000279766.1
9135	ENSG00000148483.9	ENSG00000271237.1	ENSG00000258045.2
9136	ENSG00000148484.18	ENSG00000271236.1	ENSG00000273145.1
9137	ENSG00000148488.17	ENSG00000271235.1	ENSG00000265121.1
9138	ENSG00000148498.16	ENSG00000271234.1	ENSG00000256103.2
9139	ENSG00000148513.17	ENSG00000271232.1	ENSG00000271383.7
9140	ENSG00000148516.21	ENSG00000271231.1	ENSG00000259257.1
9141	ENSG00000148541.12	ENSG00000271230.1	ENSG00000214321.3
9142	ENSG00000148572.16	ENSG00000271228.1	ENSG00000267581.1
9143	ENSG00000148584.15	ENSG00000271227.1	ENSG00000227213.1
9144	ENSG00000148600.15	ENSG00000271225.1	ENSG00000105388.15
9145	ENSG00000148602.5	ENSG00000271222.1	ENSG00000251441.3
9146	ENSG00000148604.14	ENSG00000271220.2	ENSG00000243627.4
9147	ENSG00000148606.13	ENSG00000271219.1	ENSG00000267191.1
9148	ENSG00000148634.15	ENSG00000271218.1	ENSG00000267304.1
9149	ENSG00000148655.15	ENSG00000271216.1	ENSG00000178081.12
9150	ENSG00000148660.20	ENSG00000271215.1	ENSG00000219149.4
9151	ENSG00000148671.13	ENSG00000271214.1	ENSG00000274536.6
9152	ENSG00000148672.9	ENSG00000271211.1	ENSG00000067048.17
9153	ENSG00000148677.6	ENSG00000271209.1	ENSG00000120438.12
9154	ENSG00000148680.16	ENSG00000271208.1	ENSG00000163915.7
9155	ENSG00000148688.13	ENSG00000271207.1	ENSG00000121774.18
9156	ENSG00000148690.12	ENSG00000271205.1	ENSG00000198502.6
9157	ENSG00000148700.14	ENSG00000271204.1	ENSG00000214558.4
9158	ENSG00000148702.15	ENSG00000271202.1	ENSG00000230916.2
9159	ENSG00000148704.12	ENSG00000271201.1	ENSG00000258858.1
9160	ENSG00000148719.15	ENSG00000271200.1	ENSG00000225138.7
9161	ENSG00000148730.7	ENSG00000271199.1	ENSG00000228336.2
9162	ENSG00000148734.7	ENSG00000271198.1	ENSG00000207145.1
9163	ENSG00000148735.15	ENSG00000271196.1	ENSG00000153815.16
9164	ENSG00000148737.17	ENSG00000271195.1	ENSG00000180697.9
9165	ENSG00000148773.14	ENSG00000271194.1	ENSG00000265093.2
9166	ENSG00000148795.7	ENSG00000271192.1	ENSG00000277156.1

9167	ENSG00000148798.11	ENSG00000271190.1	ENSG00000226455.1
9168	ENSG00000148803.12	ENSG00000271187.1	ENSG00000269289.5
9169	ENSG00000148814.18	ENSG00000271185.1	ENSG00000121207.12
9170	ENSG00000148824.19	ENSG00000271184.1	ENSG00000110436.13
9171	ENSG00000148826.9	ENSG00000271182.1	ENSG00000230081.2
9172	ENSG00000148832.16	ENSG00000271181.1	ENSG00000241991.1
9173	ENSG00000148834.13	ENSG00000271180.1	ENSG00000259308.3
9174	ENSG00000148835.11	ENSG00000271179.1	ENSG00000251235.3
9175	ENSG00000148840.11	ENSG00000271178.1	ENSG00000270585.1
9176	ENSG00000148841.17	ENSG00000271177.1	ENSG00000239794.3
9177	ENSG00000148842.18	ENSG00000271175.1	ENSG00000103502.14
9178	ENSG00000148843.15	ENSG00000271174.1	ENSG00000144583.5
9179	ENSG00000148848.14	ENSG00000271173.1	ENSG00000213013.4
9180	ENSG00000148908.15	ENSG00000271172.1	ENSG00000256633.1
9181	ENSG00000148925.10	ENSG00000271171.2	ENSG00000205176.3
9182	ENSG00000148926.10	ENSG00000271167.1	ENSG00000111339.12
9183	ENSG00000148935.11	ENSG00000271166.1	ENSG00000138964.17
9184	ENSG00000148942.15	ENSG00000271164.1	ENSG00000230184.1
9185	ENSG00000148943.12	ENSG00000271163.1	ENSG00000230650.1
9186	ENSG00000148948.8	ENSG00000271162.1	ENSG00000261669.1
9187	ENSG00000148950.11	ENSG00000271161.1	ENSG00000250328.5
9188	ENSG00000148965.10	ENSG00000271159.1	ENSG00000226883.1
9189	ENSG00000148985.19	ENSG00000271158.1	ENSG00000251111.2
9190	ENSG00000149016.15	ENSG00000271157.1	ENSG00000279074.1
9191	ENSG00000149021.7	ENSG00000271156.1	ENSG00000134817.10
9192	ENSG00000149043.16	ENSG00000271155.1	ENSG00000278153.1
9193	ENSG00000149050.10	ENSG00000271154.1	ENSG00000162144.9
9194	ENSG00000149054.16	ENSG00000271153.1	ENSG00000158014.14
9195	ENSG00000149084.13	ENSG00000271151.1	ENSG00000223828.1
9196	ENSG00000149089.13	ENSG00000271150.1	ENSG00000231193.1
9197	ENSG00000149090.11	ENSG00000271149.1	ENSG00000230612.3
9198	ENSG00000149091.15	ENSG00000271148.1	ENSG00000068971.14
9199	ENSG00000149100.13	ENSG00000271147.7	ENSG00000226143.1
9200	ENSG00000149115.14	ENSG00000271146.1	ENSG00000267349.1
9201	ENSG00000149124.11	ENSG00000271143.1	ENSG00000186409.16
9202	ENSG00000149131.15	ENSG00000271142.1	ENSG00000276462.1
9203	ENSG00000149133.1	ENSG00000271141.1	ENSG00000276542.1
9204	ENSG00000149136.9	ENSG00000271140.1	ENSG00000159023.21
9205	ENSG00000149150.9	ENSG00000271138.1	ENSG00000232054.1
9206	ENSG00000149177.13	ENSG00000271137.1	ENSG00000272391.6
9207	ENSG00000149179.13	ENSG00000271136.1	ENSG00000255993.1
9208	ENSG00000149182.15	ENSG00000271134.1	ENSG00000249987.1
9209	ENSG00000149187.18	ENSG00000271133.5	ENSG00000196104.11
9210	ENSG00000149196.16	ENSG00000271131.1	ENSG00000234758.1
9211	ENSG00000149201.10	ENSG00000271130.1	ENSG00000164123.6
9212	ENSG00000149212.11	ENSG00000271129.1	ENSG00000137033.11
9213	ENSG00000149218.5	ENSG00000271128.1	ENSG00000279304.1
9214	ENSG00000149231.14	ENSG00000271127.1	ENSG00000164695.5
9215	ENSG00000149243.15	ENSG00000271123.1	ENSG00000224354.2
9216	ENSG00000149256.15	ENSG00000271122.1	ENSG00000129566.13
9217	ENSG00000149257.14	ENSG00000271121.2	ENSG00000273198.1
9218	ENSG00000149260.17	ENSG00000271119.1	ENSG00000234456.7
9219	ENSG00000149262.17	ENSG00000271118.1	ENSG00000271727.1

9220	ENSG00000149269.9	ENSG00000271117.1	ENSG00000267646.1
9221	ENSG00000149273.15	ENSG00000271115.1	ENSG00000134042.13
9222	ENSG00000149289.11	ENSG00000271114.1	ENSG00000172057.10
9223	ENSG00000149292.16	ENSG00000271113.1	ENSG00000172086.8
9224	ENSG00000149294.16	ENSG00000271111.1	ENSG00000228251.1
9225	ENSG00000149295.14	ENSG00000271109.1	ENSG00000276178.1
9226	ENSG00000149300.10	ENSG00000271108.1	ENSG00000237211.2
9227	ENSG00000149305.7	ENSG00000271105.1	ENSG00000231047.1
9228	ENSG00000149308.17	ENSG00000271101.1	ENSG00000266648.1
9229	ENSG00000149311.18	ENSG00000271100.1	ENSG00000223476.1
9230	ENSG00000149313.11	ENSG00000271099.1	ENSG00000255362.1
9231	ENSG00000149328.14	ENSG00000271098.1	ENSG00000241956.9
9232	ENSG00000149346.15	ENSG00000271097.1	ENSG00000272236.1
9233	ENSG00000149357.10	ENSG00000271096.1	ENSG00000270472.2
9234	ENSG00000149380.12	ENSG00000271095.1	ENSG00000199677.1
9235	ENSG00000149403.12	ENSG00000271094.1	ENSG00000234136.1
9236	ENSG00000149418.11	ENSG00000271093.1	ENSG00000144619.15
9237	ENSG00000149428.19	ENSG00000271092.5	ENSG00000279595.1
9238	ENSG00000149435.12	ENSG00000271088.1	ENSG00000223353.2
9239	ENSG00000149443.7	ENSG00000271086.6	ENSG00000233044.1
9240	ENSG00000149451.17	ENSG00000271081.1	ENSG00000118557.15
9241	ENSG00000149452.16	ENSG00000271079.1	ENSG00000229703.6
9242	ENSG00000149474.13	ENSG00000271078.1	ENSG00000125285.5
9243	ENSG00000149476.15	ENSG00000271077.1	ENSG00000276096.1
9244	ENSG00000149480.7	ENSG00000271075.1	ENSG00000141582.15
9245	ENSG00000149483.12	ENSG00000271074.1	ENSG00000229349.2
9246	ENSG00000149485.18	ENSG00000271072.1	ENSG00000267498.1
9247	ENSG00000149488.13	ENSG00000271071.1	ENSG00000016402.13
9248	ENSG00000149489.8	ENSG00000271070.1	ENSG00000279397.1
9249	ENSG00000149499.11	ENSG00000271065.1	ENSG00000243256.1
9250	ENSG00000149503.13	ENSG00000271064.1	ENSG00000168679.18
9251	ENSG00000149506.11	ENSG00000271063.1	ENSG00000213484.2
9252	ENSG00000149507.7	ENSG00000271057.1	ENSG00000260510.1
9253	ENSG00000149516.14	ENSG00000271056.1	ENSG00000166676.16
9254	ENSG00000149527.18	ENSG00000271053.1	ENSG00000240280.7
9255	ENSG00000149531.15	ENSG00000271052.1	ENSG00000114416.18
9256	ENSG00000149532.15	ENSG00000271049.1	ENSG00000248827.1
9257	ENSG00000149534.9	ENSG00000271048.1	ENSG00000236548.1
9258	ENSG00000149541.10	ENSG00000271047.1	ENSG00000271172.1
9259	ENSG00000149547.15	ENSG00000271046.1	ENSG00000096264.14
9260	ENSG00000149548.15	ENSG00000271045.1	ENSG00000170385.10
9261	ENSG00000149554.12	ENSG00000271044.1	ENSG00000267939.1
9262	ENSG00000149557.13	ENSG00000271043.2	ENSG00000199325.1
9263	ENSG00000149564.12	ENSG00000271042.1	ENSG00000155957.17
9264	ENSG00000149571.12	ENSG00000271041.1	ENSG00000276980.1
9265	ENSG00000149573.9	ENSG00000271040.1	ENSG00000177614.11
9266	ENSG00000149575.5	ENSG00000271036.1	ENSG00000260986.1
9267	ENSG00000149577.15	ENSG00000271035.1	ENSG00000135144.7
9268	ENSG00000149582.16	ENSG00000271034.1	ENSG00000124882.4
9269	ENSG00000149591.16	ENSG00000271032.1	ENSG00000259682.1
9270	ENSG00000149596.6	ENSG00000271029.1	ENSG00000224773.2
9271	ENSG00000149599.15	ENSG00000271028.1	ENSG00000175841.8
9272	ENSG00000149600.11	ENSG00000271027.1	ENSG00000212663.2

9273	ENSG00000149609.6	ENSG00000271026.1	ENSG00000238917.1
9274	ENSG00000149633.12	ENSG00000271025.1	ENSG00000215049.3
9275	ENSG00000149634.4	ENSG00000271024.1	ENSG00000243188.1
9276	ENSG00000149635.2	ENSG00000271022.1	ENSG00000227698.1
9277	ENSG00000149636.15	ENSG00000271021.1	ENSG00000183831.6
9278	ENSG00000149639.15	ENSG00000271018.1	ENSG00000153237.17
9279	ENSG00000149646.12	ENSG00000271015.1	ENSG00000215270.3
9280	ENSG00000149651.3	ENSG00000271014.1	ENSG00000267088.1
9281	ENSG00000149654.9	ENSG00000271013.1	ENSG00000182545.6
9282	ENSG00000149656.8	ENSG00000271011.1	ENSG00000242686.4
9283	ENSG00000149657.20	ENSG00000271010.1	ENSG00000258244.1
9284	ENSG00000149658.18	ENSG00000271009.3	ENSG00000254578.1
9285	ENSG00000149679.11	ENSG00000271005.1	ENSG00000285663.1
9286	ENSG00000149716.12	ENSG00000271003.1	ENSG00000148677.6
9287	ENSG00000149735.7	ENSG00000271002.1	ENSG00000249256.2
9288	ENSG00000149742.9	ENSG00000270999.1	ENSG00000272542.1
9289	ENSG00000149743.14	ENSG00000270997.1	ENSG00000230657.6
9290	ENSG00000149761.8	ENSG00000270996.1	ENSG00000231128.5
9291	ENSG00000149781.12	ENSG00000270995.1	ENSG00000226985.6
9292	ENSG00000149782.11	ENSG00000270994.1	ENSG00000214097.5
9293	ENSG00000149792.8	ENSG00000270993.1	ENSG00000269807.1
9294	ENSG00000149798.5	ENSG00000270992.1	ENSG00000183621.15
9295	ENSG00000149806.11	ENSG00000270990.1	ENSG00000260161.1
9296	ENSG00000149809.14	ENSG00000270989.1	ENSG00000258531.2
9297	ENSG00000149823.9	ENSG00000270988.1	ENSG00000286156.1
9298	ENSG00000149922.10	ENSG00000270987.1	ENSG00000273682.1
9299	ENSG00000149923.14	ENSG00000270986.1	ENSG00000170054.14
9300	ENSG00000149925.19	ENSG00000270983.1	ENSG00000254744.3
9301	ENSG00000149926.13	ENSG00000270982.1	ENSG00000102241.12
9302	ENSG00000149927.18	ENSG00000270981.1	ENSG00000172746.6
9303	ENSG00000149929.15	ENSG00000270980.1	ENSG00000268555.1
9304	ENSG00000149930.18	ENSG00000270978.1	ENSG00000254114.1
9305	ENSG00000149932.17	ENSG00000270977.1	ENSG00000259475.1
9306	ENSG00000149948.13	ENSG00000270976.1	ENSG00000229427.1
9307	ENSG00000149968.12	ENSG00000270975.1	ENSG00000224049.1
9308	ENSG00000149970.16	ENSG00000270973.1	ENSG00000142515.15
9309	ENSG00000149972.11	ENSG00000270972.1	ENSG00000224530.1
9310	ENSG00000150045.12	ENSG00000270971.2	ENSG00000269190.6
9311	ENSG00000150048.10	ENSG00000270969.1	ENSG00000011426.11
9312	ENSG00000150051.14	ENSG00000270966.1	ENSG00000172650.13
9313	ENSG00000150054.18	ENSG00000270965.1	ENSG00000174529.7
9314	ENSG00000150093.19	ENSG00000270964.1	ENSG00000237357.2
9315	ENSG00000150175.13	ENSG00000270962.1	ENSG00000165152.9
9316	ENSG00000150201.15	ENSG00000270961.1	ENSG00000166803.13
9317	ENSG00000150244.11	ENSG00000270960.1	ENSG00000237008.2
9318	ENSG00000150261.4	ENSG00000270959.1	ENSG00000187017.16
9319	ENSG00000150269.1	ENSG00000270957.1	ENSG00000213891.3
9320	ENSG00000150275.18	ENSG00000270956.1	ENSG00000269802.1
9321	ENSG00000150276.8	ENSG00000270955.1	ENSG00000283047.1
9322	ENSG00000150281.6	ENSG00000270954.1	ENSG00000167302.10
9323	ENSG00000150316.12	ENSG00000270953.1	ENSG00000231690.2
9324	ENSG00000150337.13	ENSG00000270951.1	ENSG00000285584.1
9325	ENSG00000150347.16	ENSG00000270948.1	ENSG00000259585.2

9326	ENSG00000150361.12	ENSG00000270947.1	ENSG00000266106.1
9327	ENSG00000150394.14	ENSG00000270946.5	ENSG00000254122.2
9328	ENSG00000150401.15	ENSG00000270945.1	ENSG00000279153.1
9329	ENSG00000150403.18	ENSG00000270941.1	ENSG00000265337.1
9330	ENSG00000150433.9	ENSG00000270938.1	ENSG00000152430.17
9331	ENSG00000150455.13	ENSG00000270937.1	ENSG00000254712.1
9332	ENSG00000150456.10	ENSG00000270936.1	ENSG00000240342.3
9333	ENSG00000150457.9	ENSG00000270935.1	ENSG00000270531.1
9334	ENSG00000150459.12	ENSG00000270934.1	ENSG00000138792.10
9335	ENSG00000150471.16	ENSG00000270933.1	ENSG00000270165.1
9336	ENSG00000150477.15	ENSG00000270930.1	ENSG00000244414.6
9337	ENSG00000150510.16	ENSG00000270929.1	ENSG00000136888.7
9338	ENSG00000150527.17	ENSG00000270927.1	ENSG00000128322.7
9339	ENSG00000150540.14	ENSG00000270926.1	ENSG00000231358.3
9340	ENSG00000150551.10	ENSG00000270924.1	ENSG00000239413.1
9341	ENSG00000150556.17	ENSG00000270923.1	ENSG00000285424.1
9342	ENSG00000150593.18	ENSG00000270921.1	ENSG00000259370.2
9343	ENSG00000150594.6	ENSG00000270920.1	ENSG00000271710.1
9344	ENSG00000150625.16	ENSG00000270919.1	ENSG00000259907.1
9345	ENSG00000150627.15	ENSG00000270917.1	ENSG00000229339.1
9346	ENSG00000150628.6	ENSG00000270916.1	ENSG00000179342.4
9347	ENSG00000150630.4	ENSG00000270915.1	ENSG00000274898.1
9348	ENSG00000150636.17	ENSG00000270914.1	ENSG00000230751.1
9349	ENSG00000150637.9	ENSG00000270912.1	ENSG00000159495.7
9350	ENSG00000150656.15	ENSG00000270911.1	ENSG00000160908.15
9351	ENSG00000150667.8	ENSG00000270909.1	ENSG00000260743.1
9352	ENSG00000150672.17	ENSG00000270906.1	ENSG00000213673.3
9353	ENSG00000150676.12	ENSG00000270904.1	ENSG00000243709.1
9354	ENSG00000150681.10	ENSG00000270903.1	ENSG00000282870.1
9355	ENSG00000150687.12	ENSG00000270902.1	ENSG00000271150.1
9356	ENSG00000150712.11	ENSG00000270900.1	ENSG00000144810.16
9357	ENSG00000150722.10	ENSG00000270898.5	ENSG00000269119.1
9358	ENSG00000150732.8	ENSG00000270897.1	ENSG00000242683.1
9359	ENSG00000150750.7	ENSG00000270896.1	ENSG00000166557.13
9360	ENSG00000150753.12	ENSG00000270894.1	ENSG00000242318.1
9361	ENSG00000150756.14	ENSG00000270893.1	ENSG00000242236.2
9362	ENSG00000150760.12	ENSG00000270892.1	ENSG00000139637.14
9363	ENSG00000150764.13	ENSG00000270891.1	ENSG00000219565.2
9364	ENSG00000150768.15	ENSG00000270890.1	ENSG00000243352.3
9365	ENSG00000150773.10	ENSG00000270889.1	ENSG00000226179.6
9366	ENSG00000150776.18	ENSG00000270885.1	ENSG00000214892.4
9367	ENSG00000150779.11	ENSG00000270880.1	ENSG00000115507.10
9368	ENSG00000150782.12	ENSG00000270878.1	ENSG00000233017.2
9369	ENSG00000150783.9	ENSG00000270876.1	ENSG00000237109.1
9370	ENSG00000150787.8	ENSG00000270874.1	ENSG00000075945.13
9371	ENSG00000150867.14	ENSG00000270872.2	ENSG00000261089.1
9372	ENSG00000150873.11	ENSG00000270871.1	ENSG00000216624.3
9373	ENSG00000150893.11	ENSG00000270870.1	ENSG00000264072.1
9374	ENSG00000150907.8	ENSG00000270868.1	ENSG00000177600.9
9375	ENSG00000150938.10	ENSG00000270866.1	ENSG00000254310.1
9376	ENSG00000150961.15	ENSG00000270865.1	ENSG00000278739.1
9377	ENSG00000150967.18	ENSG00000270864.1	ENSG00000233765.1
9378	ENSG00000150977.10	ENSG00000270863.1	ENSG00000218896.1

9379	ENSG00000150990.8	ENSG00000270861.1	ENSG00000262623.1
9380	ENSG00000150991.15	ENSG00000270859.1	ENSG00000260209.1
9381	ENSG00000150995.19	ENSG00000270858.1	ENSG00000260859.1
9382	ENSG00000151005.4	ENSG00000270857.1	ENSG00000241621.1
9383	ENSG00000151006.7	ENSG00000270856.1	ENSG00000140481.15
9384	ENSG00000151012.13	ENSG00000270855.1	ENSG00000269858.6
9385	ENSG00000151014.6	ENSG00000270852.1	ENSG00000277425.1
9386	ENSG00000151023.17	ENSG00000270850.1	ENSG00000132906.18
9387	ENSG00000151025.11	ENSG00000270849.1	ENSG00000258449.1
9388	ENSG00000151033.9	ENSG00000270846.1	ENSG00000276988.1
9389	ENSG00000151062.15	ENSG00000270842.1	ENSG00000259299.1
9390	ENSG00000151065.14	ENSG00000270839.1	ENSG00000099260.11
9391	ENSG00000151067.21	ENSG00000270838.1	ENSG00000278419.1
9392	ENSG00000151079.7	ENSG00000270837.1	ENSG00000268615.1
9393	ENSG00000151090.18	ENSG00000270835.2	ENSG00000120664.10
9394	ENSG00000151092.17	ENSG00000270832.1	ENSG00000262171.1
9395	ENSG00000151093.8	ENSG00000270831.1	ENSG00000251279.1
9396	ENSG00000151116.17	ENSG00000270830.1	ENSG00000198837.10
9397	ENSG00000151117.9	ENSG00000270829.1	ENSG00000233080.3
9398	ENSG00000151131.11	ENSG00000270828.1	ENSG00000251209.8
9399	ENSG00000151135.10	ENSG00000270826.1	ENSG00000226726.1
9400	ENSG00000151136.15	ENSG00000270824.1	ENSG00000283057.1
9401	ENSG00000151148.14	ENSG00000270823.1	ENSG00000281772.1
9402	ENSG00000151150.22	ENSG00000270822.1	ENSG00000231878.1
9403	ENSG00000151151.6	ENSG00000270820.5	ENSG00000186976.15
9404	ENSG00000151164.18	ENSG00000270818.1	ENSG00000141979.4
9405	ENSG00000151176.8	ENSG00000270816.5	ENSG00000168135.4
9406	ENSG00000151208.17	ENSG00000270815.1	ENSG00000136274.9
9407	ENSG00000151224.13	ENSG00000270814.1	ENSG00000254462.1
9408	ENSG00000151229.13	ENSG00000270813.2	ENSG00000259125.1
9409	ENSG00000151233.11	ENSG00000270812.1	ENSG00000165799.5
9410	ENSG00000151239.13	ENSG00000270811.1	ENSG00000280117.1
9411	ENSG00000151240.17	ENSG00000270809.1	ENSG00000240718.3
9412	ENSG00000151247.12	ENSG00000270808.1	ENSG00000106462.10
9413	ENSG00000151276.23	ENSG00000270807.1	ENSG00000273756.4
9414	ENSG00000151287.17	ENSG00000270806.2	ENSG00000260808.1
9415	ENSG00000151292.17	ENSG00000270804.1	ENSG00000134318.14
9416	ENSG00000151303.11	ENSG00000270802.2	ENSG00000279148.1
9417	ENSG00000151304.5	ENSG00000270800.3	ENSG00000271626.1
9418	ENSG00000151320.11	ENSG00000270799.1	ENSG00000258966.1
9419	ENSG00000151322.18	ENSG00000270798.1	ENSG00000279181.1
9420	ENSG00000151327.12	ENSG00000270797.1	ENSG00000235089.1
9421	ENSG00000151332.19	ENSG00000270794.1	ENSG00000198535.5
9422	ENSG00000151338.18	ENSG00000270793.1	ENSG00000109472.14
9423	ENSG00000151348.13	ENSG00000270792.5	ENSG00000179869.15
9424	ENSG00000151353.15	ENSG00000270791.1	ENSG00000204655.12
9425	ENSG00000151360.10	ENSG00000270790.1	ENSG00000278472.1
9426	ENSG00000151364.17	ENSG00000270789.1	ENSG00000111796.3
9427	ENSG00000151365.2	ENSG00000270788.1	ENSG00000183914.14
9428	ENSG00000151366.13	ENSG00000270782.1	ENSG00000260685.1
9429	ENSG00000151376.16	ENSG00000270781.1	ENSG00000271360.1
9430	ENSG00000151379.3	ENSG00000270780.1	ENSG00000235700.1
9431	ENSG00000151388.11	ENSG00000270779.1	ENSG00000279954.1

9432	ENSG00000151413.17	ENSG00000270778.1	ENSG00000227484.1
9433	ENSG00000151414.15	ENSG00000270777.1	ENSG00000277247.1
9434	ENSG00000151418.11	ENSG00000270776.1	ENSG00000232951.1
9435	ENSG00000151422.13	ENSG00000270775.1	ENSG00000278703.1
9436	ENSG00000151445.16	ENSG00000270773.1	ENSG00000225185.3
9437	ENSG00000151458.12	ENSG00000270772.1	ENSG00000256151.1
9438	ENSG00000151461.20	ENSG00000270771.1	ENSG00000274342.1
9439	ENSG00000151465.14	ENSG00000270767.1	ENSG00000211715.1
9440	ENSG00000151466.12	ENSG00000270766.1	ENSG00000205856.4
9441	ENSG00000151468.11	ENSG00000270765.5	ENSG00000235837.1
9442	ENSG00000151470.13	ENSG00000270764.1	ENSG00000259314.1
9443	ENSG00000151474.23	ENSG00000270763.1	ENSG00000236550.1
9444	ENSG00000151475.6	ENSG00000270762.1	ENSG00000163810.11
9445	ENSG00000151490.14	ENSG00000270761.1	ENSG00000225755.2
9446	ENSG00000151491.14	ENSG00000270760.1	ENSG00000228005.1
9447	ENSG00000151498.11	ENSG00000270759.1	ENSG00000235673.1
9448	ENSG00000151500.15	ENSG00000270757.1	ENSG00000213901.10
9449	ENSG00000151502.11	ENSG00000270755.1	ENSG00000286024.1
9450	ENSG00000151503.12	ENSG00000270754.1	ENSG00000158258.16
9451	ENSG00000151532.13	ENSG00000270753.1	ENSG00000235059.5
9452	ENSG00000151552.12	ENSG00000270751.1	ENSG00000233464.2
9453	ENSG00000151553.15	ENSG00000270749.1	ENSG00000156787.17
9454	ENSG00000151572.18	ENSG00000270748.1	ENSG00000250240.5
9455	ENSG00000151575.14	ENSG00000270747.1	ENSG00000258653.3
9456	ENSG00000151576.10	ENSG00000270745.1	ENSG00000239820.3
9457	ENSG00000151577.12	ENSG00000270742.1	ENSG00000254839.1
9458	ENSG00000151611.15	ENSG00000270741.1	ENSG00000253405.1
9459	ENSG00000151612.16	ENSG00000270739.1	ENSG00000182704.8
9460	ENSG00000151615.3	ENSG00000270736.1	ENSG00000162896.6
9461	ENSG00000151617.17	ENSG00000270734.1	ENSG00000241652.3
9462	ENSG00000151623.15	ENSG00000270733.1	ENSG00000232034.1
9463	ENSG00000151631.8	ENSG00000270729.1	ENSG00000215454.6
9464	ENSG00000151632.17	ENSG00000270728.1	ENSG00000234784.2
9465	ENSG00000151640.13	ENSG00000270727.1	ENSG00000250659.2
9466	ENSG00000151650.8	ENSG00000270726.6_PAR_Y	ENSG00000253092.1
9467	ENSG00000151651.16	ENSG00000270726.6	ENSG00000250371.1
9468	ENSG00000151655.19	ENSG00000270725.1	ENSG00000266522.1
9469	ENSG00000151657.12	ENSG00000270723.1	ENSG00000120885.21
9470	ENSG00000151665.12	ENSG00000270722.1	ENSG00000137807.15
9471	ENSG00000151687.14	ENSG00000270719.1	ENSG00000271040.1
9472	ENSG00000151689.13	ENSG00000270718.1	ENSG00000270292.1
9473	ENSG00000151690.15	ENSG00000270716.1	ENSG00000197993.9
9474	ENSG00000151692.15	ENSG00000270714.1	ENSG00000163171.7
9475	ENSG00000151693.11	ENSG00000270713.1	ENSG00000255484.2
9476	ENSG00000151694.13	ENSG00000270712.1	ENSG00000177197.7
9477	ENSG00000151702.17	ENSG00000270711.1	ENSG00000272354.1
9478	ENSG00000151704.15	ENSG00000270710.1	ENSG00000261105.5
9479	ENSG00000151715.7	ENSG00000270709.1	ENSG00000224988.1
9480	ENSG00000151718.16	ENSG00000270708.1	ENSG00000241832.1
9481	ENSG00000151725.12	ENSG00000270706.1	ENSG00000177694.16
9482	ENSG00000151726.14	ENSG00000270705.1	ENSG00000266786.1
9483	ENSG00000151729.11	ENSG00000270702.1	ENSG00000073282.13
9484	ENSG00000151743.11	ENSG00000270701.1	ENSG00000273733.1

9485	ENSG00000151746.14	ENSG00000270699.1	ENSG00000253590.1
9486	ENSG00000151748.14	ENSG00000270698.1	ENSG00000187049.9
9487	ENSG00000151773.13	ENSG00000270697.1	ENSG00000277233.1
9488	ENSG00000151778.11	ENSG00000270696.1	ENSG00000258964.1
9489	ENSG00000151779.13	ENSG00000270695.1	ENSG00000273213.2
9490	ENSG00000151789.12	ENSG00000270694.1	ENSG00000264278.1
9491	ENSG00000151790.9	ENSG00000270691.1	ENSG00000240882.1
9492	ENSG00000151806.14	ENSG00000270690.1	ENSG00000249773.3
9493	ENSG00000151812.14	ENSG00000270689.1	ENSG00000226819.1
9494	ENSG00000151834.15	ENSG00000270688.1	ENSG00000265542.5
9495	ENSG00000151835.16	ENSG00000270685.1	ENSG00000268392.1
9496	ENSG00000151838.12	ENSG00000270683.1	ENSG00000204438.11
9497	ENSG00000151846.8	ENSG00000270682.1	ENSG00000203756.7
9498	ENSG00000151849.15	ENSG00000270681.1	ENSG00000229010.1
9499	ENSG00000151876.13	ENSG00000270680.1	ENSG00000253209.1
9500	ENSG00000151881.14	ENSG00000270679.1	ENSG00000244588.5
9501	ENSG00000151882.11	ENSG00000270678.1	ENSG00000128573.25
9502	ENSG00000151883.18	ENSG00000270677.1	ENSG00000248874.5
9503	ENSG00000151892.14	ENSG00000270673.1	ENSG00000164237.9
9504	ENSG00000151893.15	ENSG00000270672.1	ENSG00000229619.3
9505	ENSG00000151914.20	ENSG00000270670.1	ENSG00000106330.12
9506	ENSG00000151917.18	ENSG00000270669.1	ENSG00000230563.3
9507	ENSG00000151923.17	ENSG00000270666.1	ENSG00000282317.1
9508	ENSG00000151929.9	ENSG00000270665.1	ENSG00000069535.14
9509	ENSG00000151948.12	ENSG00000270664.1	ENSG00000261375.1
9510	ENSG00000151952.16	ENSG00000270661.1	ENSG00000261872.1
9511	ENSG00000151962.8	ENSG00000270659.1	ENSG00000124249.7
9512	ENSG00000151963.4	ENSG00000270655.1	ENSG00000127586.17
9513	ENSG00000151967.18	ENSG00000270654.1	ENSG00000236397.3
9514	ENSG00000152034.10	ENSG00000270652.1	ENSG00000261019.1
9515	ENSG00000152049.6	ENSG00000270648.1	ENSG00000169057.22
9516	ENSG00000152056.17	ENSG00000270647.6	ENSG00000224039.1
9517	ENSG00000152061.23	ENSG00000270646.1	ENSG00000272814.1
9518	ENSG00000152076.18	ENSG00000270641.1	ENSG00000257494.1
9519	ENSG00000152078.10	ENSG00000270640.1	ENSG00000236711.2
9520	ENSG00000152082.14	ENSG00000270639.1	ENSG00000263353.3
9521	ENSG00000152086.9	ENSG00000270634.1	ENSG00000249159.6
9522	ENSG00000152092.16	ENSG00000270632.1	ENSG00000110328.6
9523	ENSG00000152093.8	ENSG00000270631.1	ENSG00000221639.1
9524	ENSG00000152102.18	ENSG00000270629.6	ENSG00000271755.1
9525	ENSG00000152104.12	ENSG00000270627.1	ENSG00000230170.1
9526	ENSG00000152117.17	ENSG00000270625.1	ENSG00000274760.1
9527	ENSG00000152127.9	ENSG00000270624.1	ENSG00000251994.1
9528	ENSG00000152128.13	ENSG00000270623.1	ENSG00000261069.3
9529	ENSG00000152133.14	ENSG00000270620.1	ENSG00000280073.1
9530	ENSG00000152137.6	ENSG00000270619.1	ENSG00000280216.1
9531	ENSG00000152147.11	ENSG00000270618.1	ENSG00000215568.9
9532	ENSG00000152154.11	ENSG00000270617.1	ENSG00000277504.1
9533	ENSG00000152192.8	ENSG00000270615.1	ENSG00000205300.3
9534	ENSG00000152193.8	ENSG00000270614.1	ENSG00000272691.1
9535	ENSG00000152207.7	ENSG00000270612.1	ENSG00000105278.11
9536	ENSG00000152208.13	ENSG00000270611.1	ENSG00000225434.2
9537	ENSG00000152213.3	ENSG00000270610.1	ENSG00000050393.11

9538	ENSG00000152214.14	ENSG00000270607.1	ENSG00000227289.2
9539	ENSG00000152217.18	ENSG00000270606.1	ENSG00000263394.1
9540	ENSG00000152219.5	ENSG00000270605.1	ENSG00000260647.1
9541	ENSG00000152223.14	ENSG00000270604.5	ENSG00000225741.1
9542	ENSG00000152229.18	ENSG00000270601.4	ENSG00000265313.6
9543	ENSG00000152234.16	ENSG00000270598.1	ENSG00000260725.1
9544	ENSG00000152240.13	ENSG00000270593.1	ENSG00000229836.1
9545	ENSG00000152242.11	ENSG00000270591.1	ENSG00000229686.1
9546	ENSG00000152253.9	ENSG00000270589.1	ENSG00000234132.2
9547	ENSG00000152254.10	ENSG00000270588.1	ENSG00000223513.1
9548	ENSG00000152256.13	ENSG00000270587.1	ENSG00000222750.1
9549	ENSG00000152266.7	ENSG00000270585.1	ENSG00000261514.1
9550	ENSG00000152270.9	ENSG00000270584.1	ENSG00000165078.12
9551	ENSG00000152291.14	ENSG00000270583.1	ENSG00000179603.17
9552	ENSG00000152292.17	ENSG00000270580.5	ENSG00000145384.3
9553	ENSG00000152315.5	ENSG00000270578.1	ENSG00000216901.1
9554	ENSG00000152332.16	ENSG00000270577.1	ENSG00000167359.8
9555	ENSG00000152348.16	ENSG00000270576.1	ENSG00000136754.17
9556	ENSG00000152359.14	ENSG00000270575.1	ENSG00000004897.12
9557	ENSG00000152377.14	ENSG00000270574.1	ENSG00000260459.2
9558	ENSG00000152380.10	ENSG00000270571.2	ENSG00000110944.9
9559	ENSG00000152382.6	ENSG00000270570.1	ENSG00000174469.22
9560	ENSG00000152402.10	ENSG00000270569.1	ENSG00000279378.1
9561	ENSG00000152404.15	ENSG00000270562.1	ENSG00000224324.1
9562	ENSG00000152409.9	ENSG00000270560.1	ENSG00000213333.3
9563	ENSG00000152413.14	ENSG00000270558.1	ENSG00000167080.8
9564	ENSG00000152422.15	ENSG00000270557.1	ENSG00000242893.3
9565	ENSG00000152430.17	ENSG00000270555.1	ENSG00000239445.5
9566	ENSG00000152433.14	ENSG00000270554.1	ENSG00000224848.1
9567	ENSG00000152439.12	ENSG00000270553.1	ENSG00000186676.3
9568	ENSG00000152443.13	ENSG00000270552.1	ENSG00000212607.1
9569	ENSG00000152454.4	ENSG00000270550.1	ENSG00000237336.1
9570	ENSG00000152455.15	ENSG00000270549.1	ENSG00000162723.10
9571	ENSG00000152457.18	ENSG00000270547.5	ENSG00000260788.5
9572	ENSG00000152463.14	ENSG00000270542.1	ENSG00000249068.1
9573	ENSG00000152464.15	ENSG00000270541.1	ENSG00000171992.13
9574	ENSG00000152465.18	ENSG00000270540.1	ENSG00000283023.1
9575	ENSG00000152467.9	ENSG00000270538.1	ENSG00000236202.1
9576	ENSG00000152475.7	ENSG00000270535.1	ENSG00000227210.1
9577	ENSG00000152484.14	ENSG00000270533.2	ENSG00000270759.1
9578	ENSG00000152492.15	ENSG00000270531.1	ENSG00000270060.1
9579	ENSG00000152495.11	ENSG00000270528.1	ENSG00000255786.1
9580	ENSG00000152503.9	ENSG00000270526.1	ENSG00000198542.14
9581	ENSG00000152518.8	ENSG00000270524.1	ENSG00000253535.5
9582	ENSG00000152520.14	ENSG00000270522.1	ENSG00000254703.2
9583	ENSG00000152527.14	ENSG00000270521.1	ENSG00000179157.4
9584	ENSG00000152556.17	ENSG00000270518.2	ENSG00000231407.5
9585	ENSG00000152558.15	ENSG00000270516.1	ENSG00000234619.1
9586	ENSG00000152578.13	ENSG00000270513.1	ENSG00000188340.2
9587	ENSG00000152580.8	ENSG00000270512.1	ENSG00000277464.1
9588	ENSG00000152582.14	ENSG00000270510.1	ENSG00000234884.1
9589	ENSG00000152583.12	ENSG00000270509.1	ENSG00000198570.5
9590	ENSG00000152591.14	ENSG00000270507.1	ENSG00000285779.1

9591	ENSG00000152592.13	ENSG00000270506.1	ENSG00000166963.13
9592	ENSG00000152595.16	ENSG00000270505.1	ENSG00000069764.9
9593	ENSG00000152601.17	ENSG00000270504.1	ENSG00000249129.1
9594	ENSG00000152611.12	ENSG00000270503.1	ENSG00000147526.20
9595	ENSG00000152620.13	ENSG00000270500.1	ENSG00000224666.3
9596	ENSG00000152642.10	ENSG00000270499.1	ENSG00000276093.1
9597	ENSG00000152661.8	ENSG00000270497.1	ENSG00000175868.14
9598	ENSG00000152669.9	ENSG00000270496.1	ENSG00000137080.4
9599	ENSG00000152670.18	ENSG00000270495.1	ENSG00000179055.7
9600	ENSG00000152672.8	ENSG00000270494.1	ENSG00000228828.1
9601	ENSG00000152683.14	ENSG00000270493.1	ENSG00000118503.15
9602	ENSG00000152684.11	ENSG00000270492.1	ENSG00000224609.7
9603	ENSG00000152689.18	ENSG00000270491.1	ENSG00000263862.1
9604	ENSG00000152700.14	ENSG00000270490.1	ENSG00000125879.4
9605	ENSG00000152705.8	ENSG00000270488.1	ENSG00000237238.3
9606	ENSG00000152749.8	ENSG00000270487.1	ENSG00000256694.1
9607	ENSG00000152760.10	ENSG00000270484.1	ENSG00000120868.13
9608	ENSG00000152763.17	ENSG00000270482.1	ENSG00000073008.15
9609	ENSG00000152766.6	ENSG00000270480.1	ENSG00000273011.1
9610	ENSG00000152767.16	ENSG00000270479.1	ENSG00000197016.12
9611	ENSG00000152778.9	ENSG00000270477.1	ENSG00000175315.3
9612	ENSG00000152779.14	ENSG00000270475.1	ENSG00000271100.1
9613	ENSG00000152782.16	ENSG00000270474.2	ENSG00000223774.5
9614	ENSG00000152784.15	ENSG00000270472.2	ENSG00000275340.1
9615	ENSG00000152785.7	ENSG00000270470.1	ENSG00000225760.2
9616	ENSG00000152795.17	ENSG00000270469.1	ENSG00000228323.2
9617	ENSG00000152804.11	ENSG00000270467.1	ENSG00000228169.3
9618	ENSG00000152818.18	ENSG00000270462.1	ENSG00000121769.7
9619	ENSG00000152822.14	ENSG00000270460.1	ENSG00000109534.17
9620	ENSG00000152894.14	ENSG00000270458.1	ENSG00000158623.14
9621	ENSG00000152904.11	ENSG00000270457.1	ENSG00000268889.1
9622	ENSG00000152910.19	ENSG00000270456.1	ENSG00000184261.4
9623	ENSG00000152926.14	ENSG00000270455.1	ENSG00000269888.1
9624	ENSG00000152931.7	ENSG00000270453.1	ENSG00000204347.4
9625	ENSG00000152932.8	ENSG00000270451.1	ENSG00000197183.14
9626	ENSG00000152936.10	ENSG00000270450.1	ENSG00000243199.1
9627	ENSG00000152939.16	ENSG00000270449.1	ENSG00000254153.1
9628	ENSG00000152942.19	ENSG00000270447.1	ENSG00000231548.2
9629	ENSG00000152944.9	ENSG00000270446.1	ENSG00000232352.1
9630	ENSG00000152952.12	ENSG00000270445.1	ENSG00000256424.1
9631	ENSG00000152953.13	ENSG00000270444.1	ENSG00000232333.1
9632	ENSG00000152954.12	ENSG00000270443.1	ENSG00000230555.2
9633	ENSG00000152969.19	ENSG00000270442.1	ENSG00000228325.5
9634	ENSG00000152977.10	ENSG00000270441.1	ENSG00000207181.1
9635	ENSG00000152990.14	ENSG00000270440.1	ENSG00000279917.1
9636	ENSG00000153002.12	ENSG00000270437.1	ENSG00000237064.1
9637	ENSG00000153006.16	ENSG00000270435.1	ENSG00000253982.1
9638	ENSG00000153012.12	ENSG00000270434.1	ENSG00000185480.11
9639	ENSG00000153015.16	ENSG00000270433.1	ENSG00000160051.11
9640	ENSG00000153029.14	ENSG00000270432.1	ENSG00000181634.8
9641	ENSG00000153037.14	ENSG00000270431.1	ENSG00000214073.2
9642	ENSG00000153044.10	ENSG00000270430.1	ENSG00000231437.3
9643	ENSG00000153046.18	ENSG00000270429.1	ENSG00000267091.1

9644	ENSG00000153048.11	ENSG00000270427.1	ENSG00000253730.1
9645	ENSG00000153060.7	ENSG00000270426.1	ENSG00000131435.13
9646	ENSG00000153064.12	ENSG00000270425.1	ENSG00000136874.11
9647	ENSG00000153066.12	ENSG00000270424.1	ENSG00000149532.15
9648	ENSG00000153071.15	ENSG00000270423.1	ENSG00000270249.1
9649	ENSG00000153086.14	ENSG00000270422.1	ENSG00000273710.1
9650	ENSG00000153093.18	ENSG00000270421.1	ENSG00000285758.1
9651	ENSG00000153094.23	ENSG00000270419.1	ENSG00000246662.6
9652	ENSG00000153107.13	ENSG00000270416.2	ENSG00000211944.2
9653	ENSG00000153113.23	ENSG00000270415.1	ENSG00000172270.19
9654	ENSG00000153130.17	ENSG00000270413.1	ENSG00000221190.1
9655	ENSG00000153132.13	ENSG00000270412.1	ENSG00000200816.1
9656	ENSG00000153140.8	ENSG00000270411.1	ENSG00000234690.6
9657	ENSG00000153147.6	ENSG00000270409.1	ENSG00000198692.10
9658	ENSG00000153157.13	ENSG00000270405.1	ENSG00000213946.3
9659	ENSG00000153162.9	ENSG00000270403.1	ENSG00000254996.5
9660	ENSG00000153165.18	ENSG00000270402.1	ENSG00000271825.1
9661	ENSG00000153179.13	ENSG00000270401.1	ENSG00000142864.14
9662	ENSG00000153187.20	ENSG00000270400.1	ENSG00000236848.2
9663	ENSG00000153201.16	ENSG00000270397.1	ENSG00000186148.13
9664	ENSG00000153207.15	ENSG00000270395.1	ENSG00000204195.3
9665	ENSG00000153208.16	ENSG00000270394.4	ENSG00000253799.1
9666	ENSG00000153214.11	ENSG00000270393.1	ENSG00000236530.2
9667	ENSG00000153230.4	ENSG00000270392.2	ENSG00000223669.1
9668	ENSG00000153233.13	ENSG00000270390.1	ENSG00000259461.1
9669	ENSG00000153234.14	ENSG00000270388.1	ENSG00000166840.13
9670	ENSG00000153237.17	ENSG00000270387.1	ENSG00000173227.14
9671	ENSG00000153246.13	ENSG00000270385.1	ENSG00000214132.4
9672	ENSG00000153250.20	ENSG00000270384.1	ENSG00000233674.2
9673	ENSG00000153253.17	ENSG00000270382.1	ENSG00000229937.7
9674	ENSG00000153283.12	ENSG00000270381.1	ENSG00000271981.1
9675	ENSG00000153291.16	ENSG00000270380.1	ENSG00000228040.1
9676	ENSG00000153292.16	ENSG00000270379.5	ENSG00000259424.1
9677	ENSG00000153294.11	ENSG00000270378.1	ENSG00000257907.2
9678	ENSG00000153303.17	ENSG00000270377.1	ENSG00000249899.5
9679	ENSG00000153310.19	ENSG00000270372.1	ENSG00000285952.1
9680	ENSG00000153317.15	ENSG00000270371.1	ENSG00000131885.17
9681	ENSG00000153339.14	ENSG00000270369.1	ENSG00000286092.1
9682	ENSG00000153347.9	ENSG00000270367.1	ENSG00000223403.4
9683	ENSG00000153363.12	ENSG00000270362.1	ENSG00000252653.1
9684	ENSG00000153391.15	ENSG00000270361.1	ENSG00000254561.3
9685	ENSG00000153395.10	ENSG00000270359.1	ENSG00000280916.2
9686	ENSG00000153404.14	ENSG00000270356.1	ENSG00000142453.11
9687	ENSG00000153406.13	ENSG00000270354.1	ENSG00000184022.4
9688	ENSG00000153443.13	ENSG00000270352.1	ENSG00000260494.1
9689	ENSG00000153446.15	ENSG00000270350.1	ENSG00000238029.1
9690	ENSG00000153485.5	ENSG00000270347.1	ENSG00000267497.1
9691	ENSG00000153487.12	ENSG00000270344.2	ENSG00000134243.12
9692	ENSG00000153495.10	ENSG00000270343.1	ENSG00000196458.11
9693	ENSG00000153498.12	ENSG00000270342.1	ENSG00000124225.16
9694	ENSG00000153531.13	ENSG00000270336.1	ENSG00000225546.5
9695	ENSG00000153551.13	ENSG00000270335.1	ENSG00000165376.10
9696	ENSG00000153558.15	ENSG00000270333.1	ENSG00000261080.1

9697	ENSG00000153560.12	ENSG00000270332.1	ENSG00000224553.1
9698	ENSG00000153561.13	ENSG00000270330.1	ENSG00000139269.3
9699	ENSG00000153563.15	ENSG00000270328.1	ENSG00000267345.1
9700	ENSG00000153574.9	ENSG00000270326.1	ENSG00000199133.3
9701	ENSG00000153684.15	ENSG00000270325.1	ENSG00000259892.1
9702	ENSG00000153707.17	ENSG00000270324.1	ENSG00000254558.1
9703	ENSG00000153714.6	ENSG00000270323.1	ENSG00000240589.3
9704	ENSG00000153721.19	ENSG00000270322.1	ENSG00000249931.4
9705	ENSG00000153767.10	ENSG00000270321.1	ENSG00000271392.1
9706	ENSG00000153774.9	ENSG00000270318.1	ENSG00000113296.14
9707	ENSG00000153779.10	ENSG00000270317.1	ENSG00000069974.16
9708	ENSG00000153786.12	ENSG00000270316.1	ENSG00000251458.1
9709	ENSG00000153789.13	ENSG00000270314.1	ENSG00000214955.5
9710	ENSG00000153790.12	ENSG00000270313.1	ENSG00000274852.1
9711	ENSG00000153802.11	ENSG00000270308.1	ENSG00000237714.1
9712	ENSG00000153814.13	ENSG00000270307.1	ENSG00000233101.10
9713	ENSG00000153815.16	ENSG00000270306.1	ENSG00000255374.3
9714	ENSG00000153820.13	ENSG00000270304.1	ENSG00000219409.2
9715	ENSG00000153822.13	ENSG00000270302.1	ENSG00000249767.1
9716	ENSG00000153823.18	ENSG00000270301.1	ENSG00000232083.3
9717	ENSG00000153827.13	ENSG00000270300.2	ENSG00000231381.2
9718	ENSG00000153832.12	ENSG00000270299.1	ENSG00000268652.1
9719	ENSG00000153879.9	ENSG00000270296.1	ENSG00000230918.1
9720	ENSG00000153885.14	ENSG00000270294.1	ENSG00000219682.4
9721	ENSG00000153896.18	ENSG00000270293.1	ENSG00000235271.5
9722	ENSG00000153898.13	ENSG00000270292.1	ENSG00000230010.1
9723	ENSG00000153902.14	ENSG00000270289.1	ENSG00000204033.9
9724	ENSG00000153904.20	ENSG00000270287.1	ENSG00000284618.1
9725	ENSG00000153914.16	ENSG00000270285.1	ENSG00000166165.13
9726	ENSG00000153922.10	ENSG00000270282.1	ENSG00000079101.16
9727	ENSG00000153923.10	ENSG00000270281.1	ENSG00000267500.1
9728	ENSG00000153930.11	ENSG00000270280.1	ENSG00000238271.2
9729	ENSG00000153933.10	ENSG00000270279.1	ENSG00000282416.1
9730	ENSG00000153936.17	ENSG00000270277.1	ENSG00000242267.6
9731	ENSG00000153944.11	ENSG00000270276.2	ENSG00000274849.1
9732	ENSG00000153956.16	ENSG00000270275.1	ENSG00000235768.1
9733	ENSG00000153975.10	ENSG00000270273.1	ENSG00000116030.16
9734	ENSG00000153976.3	ENSG00000270270.1	ENSG00000213445.10
9735	ENSG00000153982.11	ENSG00000270269.1	ENSG00000258090.1
9736	ENSG00000153989.8	ENSG00000270268.1	ENSG00000185792.9
9737	ENSG00000153993.13	ENSG00000270265.1	ENSG00000107485.17
9738	ENSG00000154001.14	ENSG00000270264.1	ENSG00000249706.1
9739	ENSG00000154007.6	ENSG00000270259.2	ENSG00000249252.5
9740	ENSG00000154016.13	ENSG00000270258.1	ENSG00000246640.1
9741	ENSG00000154025.15	ENSG00000270257.1	ENSG00000229752.1
9742	ENSG00000154027.19	ENSG00000270255.1	ENSG00000231650.1
9743	ENSG00000154040.20	ENSG00000270252.1	ENSG00000171124.13
9744	ENSG00000154059.11	ENSG00000270251.1	ENSG00000227198.1
9745	ENSG00000154065.17	ENSG00000270249.1	ENSG00000229380.1
9746	ENSG00000154079.6	ENSG00000270248.1	ENSG00000256220.1
9747	ENSG00000154080.14	ENSG00000270244.1	ENSG00000254105.1
9748	ENSG00000154096.13	ENSG00000270243.1	ENSG00000051009.10
9749	ENSG00000154099.18	ENSG00000270242.1	ENSG00000251687.1

9750	ENSG00000154102.11	ENSG00000270241.1	ENSG00000244661.1
9751	ENSG00000154114.12	ENSG00000270240.2	ENSG00000124003.12
9752	ENSG00000154118.13	ENSG00000270236.1	ENSG00000228118.3
9753	ENSG00000154122.14	ENSG00000270234.1	ENSG00000161958.11
9754	ENSG00000154124.4	ENSG00000270232.1	ENSG00000243655.2
9755	ENSG00000154127.10	ENSG00000270231.4	ENSG00000239917.3
9756	ENSG00000154133.14	ENSG00000270230.1	ENSG00000244230.3
9757	ENSG00000154134.15	ENSG00000270228.1	ENSG00000196302.5
9758	ENSG00000154143.2	ENSG00000270226.1	ENSG00000206603.1
9759	ENSG00000154144.13	ENSG00000270225.1	ENSG00000259469.1
9760	ENSG00000154146.13	ENSG00000270222.1	ENSG00000165283.16
9761	ENSG00000154153.13	ENSG00000270218.1	ENSG00000265136.1
9762	ENSG00000154162.14	ENSG00000270212.1	ENSG00000265113.1
9763	ENSG00000154165.5	ENSG00000270210.1	ENSG00000151748.14
9764	ENSG00000154174.7	ENSG00000270209.1	ENSG00000259380.5
9765	ENSG00000154175.17	ENSG00000270207.2	ENSG00000275438.1
9766	ENSG00000154188.10	ENSG00000270204.1	ENSG00000254559.1
9767	ENSG00000154198.14	ENSG00000270202.1	ENSG00000259186.1
9768	ENSG00000154217.15	ENSG00000270200.1	ENSG00000213779.4
9769	ENSG00000154222.14	ENSG00000270196.1	ENSG00000260621.1
9770	ENSG00000154227.13	ENSG00000270195.2	ENSG00000132704.16
9771	ENSG00000154229.12	ENSG00000270194.1	ENSG00000134030.14
9772	ENSG00000154237.13	ENSG00000270193.1	ENSG00000174429.4
9773	ENSG00000154240.17	ENSG00000270192.1	ENSG00000231671.1
9774	ENSG00000154252.11	ENSG00000270191.1	ENSG00000233028.1
9775	ENSG00000154258.17	ENSG00000270190.1	ENSG00000205084.11
9776	ENSG00000154262.13	ENSG00000270188.1	ENSG00000225900.1
9777	ENSG00000154263.17	ENSG00000270187.1	ENSG00000266456.1
9778	ENSG00000154265.16	ENSG00000270185.1	ENSG00000250892.1
9779	ENSG00000154269.15	ENSG00000270184.1	ENSG00000253159.3
9780	ENSG00000154274.15	ENSG00000270182.1	ENSG00000285697.1
9781	ENSG00000154277.12	ENSG00000270181.3	ENSG00000232380.1
9782	ENSG00000154305.17	ENSG00000270179.1	ENSG00000260198.1
9783	ENSG00000154309.8	ENSG00000270178.1	ENSG00000249780.1
9784	ENSG00000154310.17	ENSG00000270177.1	ENSG00000230561.4
9785	ENSG00000154316.16	ENSG00000270175.1	ENSG00000022840.16
9786	ENSG00000154319.16	ENSG00000270174.1	ENSG00000232084.5
9787	ENSG00000154328.16	ENSG00000270171.1	ENSG00000275630.1
9788	ENSG00000154330.13	ENSG00000270170.2	ENSG00000221858.3
9789	ENSG00000154342.6	ENSG00000270168.2	ENSG00000168000.14
9790	ENSG00000154358.20	ENSG00000270166.1	ENSG00000262298.1
9791	ENSG00000154359.13	ENSG00000270165.1	ENSG00000126010.5
9792	ENSG00000154370.16	ENSG00000270164.1	ENSG00000264862.2
9793	ENSG00000154380.17	ENSG00000270159.1	ENSG00000158748.4
9794	ENSG00000154415.7	ENSG00000270154.1	ENSG00000112599.9
9795	ENSG00000154429.11	ENSG00000270149.5	ENSG00000222445.1
9796	ENSG00000154438.7	ENSG00000270141.3	ENSG00000218233.1
9797	ENSG00000154447.15	ENSG00000270140.1	ENSG00000244405.8
9798	ENSG00000154451.14	ENSG00000270139.1	ENSG00000269177.1
9799	ENSG00000154473.18	ENSG00000270137.1	ENSG00000189269.12
9800	ENSG00000154478.3	ENSG00000270136.6	ENSG00000268460.1
9801	ENSG00000154479.13	ENSG00000270135.1	ENSG00000139971.15
9802	ENSG00000154485.5	ENSG00000270133.1	ENSG00000180438.15

9803	ENSG00000154493.18	ENSG00000270131.1	ENSG00000253187.2
9804	ENSG00000154511.12	ENSG00000270130.1	ENSG00000165264.11
9805	ENSG00000154518.9	ENSG00000270127.2	ENSG00000275665.1
9806	ENSG00000154529.14	ENSG00000270124.1	ENSG00000269349.1
9807	ENSG00000154545.16	ENSG00000270123.4	ENSG00000204385.12
9808	ENSG00000154548.9	ENSG00000270120.1	ENSG00000237224.4
9809	ENSG00000154553.15	ENSG00000270118.1	ENSG00000184774.10
9810	ENSG00000154556.18	ENSG00000270117.1	ENSG00000232019.1
9811	ENSG00000154582.16	ENSG00000270116.1	ENSG00000112852.6
9812	ENSG00000154589.6	ENSG00000270115.1	ENSG00000243977.1
9813	ENSG00000154608.14	ENSG00000270114.1	ENSG00000070886.12
9814	ENSG00000154611.14	ENSG00000270112.3	ENSG00000231837.2
9815	ENSG00000154620.6	ENSG00000270111.2	ENSG00000251365.3
9816	ENSG00000154639.19	ENSG00000270110.1	ENSG00000136840.19
9817	ENSG00000154640.14	ENSG00000270108.1	ENSG00000222806.1
9818	ENSG00000154642.11	ENSG00000270106.6	ENSG00000226080.1
9819	ENSG00000154645.14	ENSG00000270105.1	ENSG00000279301.4
9820	ENSG00000154646.9	ENSG00000270104.1	ENSG00000161609.10
9821	ENSG00000154654.15	ENSG00000270103.3	ENSG00000257831.1
9822	ENSG00000154655.15	ENSG00000270100.1	ENSG00000204869.8
9823	ENSG00000154678.17	ENSG00000270099.1	ENSG00000180999.11
9824	ENSG00000154710.17	ENSG00000270098.1	ENSG00000158796.17
9825	ENSG00000154719.13	ENSG00000270096.1	ENSG00000229863.1
9826	ENSG00000154721.15	ENSG00000270095.1	ENSG00000243033.2
9827	ENSG00000154723.12	ENSG00000270094.1	ENSG00000221971.3
9828	ENSG00000154727.10	ENSG00000270091.1	ENSG00000234162.2
9829	ENSG00000154734.15	ENSG00000270090.5	ENSG00000285095.1
9830	ENSG00000154736.6	ENSG00000270087.5	ENSG00000223979.2
9831	ENSG00000154743.17	ENSG00000270084.2	ENSG00000237292.1
9832	ENSG00000154760.14	ENSG00000270083.1	ENSG00000242348.3
9833	ENSG00000154764.5	ENSG00000270077.1	ENSG00000169862.19
9834	ENSG00000154767.14	ENSG00000270076.1	ENSG00000250006.2
9835	ENSG00000154781.16	ENSG00000270075.1	ENSG00000273257.1
9836	ENSG00000154783.11	ENSG00000270074.1	ENSG00000176907.4
9837	ENSG00000154803.13	ENSG00000270073.1	ENSG00000132581.9
9838	ENSG00000154813.10	ENSG00000270072.1	ENSG00000123329.18
9839	ENSG00000154814.14	ENSG00000270071.2	ENSG00000260566.2
9840	ENSG00000154822.18	ENSG00000270069.1	ENSG00000168314.17
9841	ENSG00000154832.14	ENSG00000270068.1	ENSG00000236022.6
9842	ENSG00000154839.10	ENSG00000270066.3	ENSG00000233590.1
9843	ENSG00000154845.15	ENSG00000270062.1	ENSG00000284637.1
9844	ENSG00000154856.13	ENSG00000270061.1	ENSG00000183166.11
9845	ENSG00000154864.12	ENSG00000270060.1	ENSG00000255347.1
9846	ENSG00000154874.15	ENSG00000270059.1	ENSG00000260693.1
9847	ENSG00000154889.16	ENSG00000270055.1	ENSG00000255446.1
9848	ENSG00000154898.15	ENSG00000270052.1	ENSG00000266311.6
9849	ENSG00000154914.17	ENSG00000270050.1	ENSG00000214491.8
9850	ENSG00000154917.11	ENSG00000270049.2	ENSG00000277017.1
9851	ENSG00000154920.14	ENSG00000270048.1	ENSG00000076662.10
9852	ENSG00000154928.18	ENSG00000270040.1	ENSG00000124233.12
9853	ENSG00000154930.15	ENSG00000270039.1	ENSG00000251017.1
9854	ENSG00000154945.7	ENSG00000270038.1	ENSG00000285847.1
9855	ENSG00000154957.14	ENSG00000270036.1	ENSG00000117461.15

9856	ENSG00000154975.14	ENSG00000270035.1	ENSG00000214338.10
9857	ENSG00000154978.13	ENSG00000270031.1	ENSG00000278513.1
9858	ENSG00000154997.9	ENSG00000270030.1	ENSG00000248873.1
9859	ENSG00000155008.14	ENSG00000270025.2	ENSG00000278217.1
9860	ENSG00000155011.9	ENSG00000270022.3	ENSG00000255358.1
9861	ENSG00000155016.18	ENSG00000270021.1	ENSG00000218690.2
9862	ENSG00000155026.16	ENSG00000270019.1	ENSG00000223505.2
9863	ENSG00000155034.19	ENSG00000270017.1	ENSG00000253385.1
9864	ENSG00000155052.14	ENSG00000270016.1	ENSG00000161904.12
9865	ENSG00000155066.16	ENSG00000270015.1	ENSG00000224856.2
9866	ENSG00000155070.8	ENSG00000270012.1	ENSG00000103056.12
9867	ENSG00000155085.15	ENSG00000270011.7	ENSG00000067840.12
9868	ENSG00000155087.4	ENSG00000270008.1	ENSG00000235240.1
9869	ENSG00000155090.15	ENSG00000270006.2	ENSG00000258839.3
9870	ENSG00000155093.19	ENSG00000270002.1	ENSG00000214559.3
9871	ENSG00000155096.14	ENSG00000270001.1	ENSG00000250072.5
9872	ENSG00000155097.12	ENSG00000270000.1	ENSG00000258294.5
9873	ENSG00000155099.8	ENSG00000269997.1	ENSG00000272837.1
9874	ENSG00000155100.10	ENSG00000269994.2	ENSG00000005175.10
9875	ENSG00000155111.15	ENSG00000269993.1	ENSG00000233379.1
9876	ENSG00000155115.7	ENSG00000269989.1	ENSG00000196338.12
9877	ENSG00000155158.20	ENSG00000269987.1	ENSG00000183632.14
9878	ENSG00000155189.12	ENSG00000269986.1	ENSG00000248243.1
9879	ENSG00000155229.21	ENSG00000269984.1	ENSG00000270270.1
9880	ENSG00000155249.5	ENSG00000269983.1	ENSG00000254695.1
9881	ENSG00000155252.13	ENSG00000269982.1	ENSG00000248553.1
9882	ENSG00000155254.13	ENSG00000269981.1	ENSG00000186204.14
9883	ENSG00000155256.17	ENSG00000269980.1	ENSG00000219703.1
9884	ENSG00000155265.11	ENSG00000269978.1	ENSG00000258407.1
9885	ENSG00000155269.12	ENSG00000269976.1	ENSG00000269026.2
9886	ENSG00000155275.19	ENSG00000269973.1	ENSG00000180919.3
9887	ENSG00000155287.11	ENSG00000269972.1	ENSG00000203867.8
9888	ENSG00000155304.6	ENSG00000269971.1	ENSG00000255305.1
9889	ENSG00000155307.18	ENSG00000269970.1	ENSG00000188610.12
9890	ENSG00000155313.15	ENSG00000269968.1	ENSG00000227907.1
9891	ENSG00000155324.9	ENSG00000269967.1	ENSG00000237170.3
9892	ENSG00000155329.12	ENSG00000269966.1	ENSG00000274443.4
9893	ENSG00000155330.10	ENSG00000269964.3	ENSG00000253671.2
9894	ENSG00000155363.18	ENSG00000269961.1	ENSG00000259235.1
9895	ENSG00000155366.16	ENSG00000269959.1	ENSG00000167210.17
9896	ENSG00000155367.15	ENSG00000269958.1	ENSG00000048545.13
9897	ENSG00000155368.16	ENSG00000269957.6	ENSG00000280435.1
9898	ENSG00000155380.11	ENSG00000269956.1	ENSG00000231646.5
9899	ENSG00000155393.13	ENSG00000269955.2	ENSG00000220785.7
9900	ENSG00000155428.12	ENSG00000269954.2	ENSG00000128591.15
9901	ENSG00000155438.12	ENSG00000269952.1	ENSG00000280767.3
9902	ENSG00000155463.13	ENSG00000269951.1	ENSG00000223834.3
9903	ENSG00000155465.18	ENSG00000269950.1	ENSG00000124915.10
9904	ENSG00000155495.9	ENSG00000269949.1	ENSG00000258556.1
9905	ENSG00000155506.17	ENSG00000269947.1	ENSG00000134827.8
9906	ENSG00000155508.13	ENSG00000269945.1	ENSG00000207554.1
9907	ENSG00000155511.18	ENSG00000269944.1	ENSG00000156475.18
9908	ENSG00000155530.3	ENSG00000269940.1	ENSG00000267650.1

9909	ENSG00000155542.11	ENSG00000269939.1	ENSG00000265033.2
9910	ENSG00000155545.19	ENSG00000269938.1	ENSG00000237818.1
9911	ENSG00000155561.15	ENSG00000269937.1	ENSG00000261435.1
9912	ENSG00000155592.16	ENSG00000269935.1	ENSG00000160838.14
9913	ENSG00000155621.15	ENSG00000269934.2	ENSG00000171368.12
9914	ENSG00000155622.7	ENSG00000269933.1	ENSG00000249173.5
9915	ENSG00000155629.15	ENSG00000269931.2	ENSG00000236116.1
9916	ENSG00000155636.15	ENSG00000269930.1	ENSG00000247033.1
9917	ENSG00000155657.26	ENSG00000269929.2	ENSG00000176510.5
9918	ENSG00000155659.15	ENSG00000269927.1	ENSG00000236348.1
9919	ENSG00000155660.11	ENSG00000269926.1	ENSG00000157551.19
9920	ENSG00000155666.11	ENSG00000269925.1	ENSG00000264270.1
9921	ENSG00000155714.13	ENSG00000269924.1	ENSG00000228415.3
9922	ENSG00000155719.17	ENSG00000269921.1	ENSG00000102904.14
9923	ENSG00000155729.13	ENSG00000269919.1	ENSG00000172159.16
9924	ENSG00000155744.9	ENSG00000269918.1	ENSG00000122026.10
9925	ENSG00000155749.12	ENSG00000269915.1	ENSG00000213076.3
9926	ENSG00000155754.15	ENSG00000269911.1	ENSG00000167513.9
9927	ENSG00000155755.19	ENSG00000269907.1	ENSG00000285447.1
9928	ENSG00000155760.2	ENSG00000269906.1	ENSG00000275432.1
9929	ENSG00000155761.13	ENSG00000269903.1	ENSG00000213916.2
9930	ENSG00000155792.10	ENSG00000269902.1	ENSG00000179362.14
9931	ENSG00000155816.20	ENSG00000269901.1	ENSG00000262585.1
9932	ENSG00000155827.12	ENSG00000269900.3	ENSG00000270218.1
9933	ENSG00000155833.15	ENSG00000269899.1	ENSG00000103549.21
9934	ENSG00000155846.17	ENSG00000269898.1	ENSG00000230373.8
9935	ENSG00000155849.15	ENSG00000269897.5	ENSG00000256779.2
9936	ENSG00000155850.7	ENSG00000269896.2	ENSG00000162494.6
9937	ENSG00000155858.6	ENSG00000269895.1	ENSG00000224414.1
9938	ENSG00000155868.8	ENSG00000269894.1	ENSG00000228086.1
9939	ENSG00000155875.15	ENSG00000269893.7	ENSG00000277867.1
9940	ENSG00000155876.5	ENSG00000269892.1	ENSG00000260194.1
9941	ENSG00000155886.11	ENSG00000269890.1	ENSG00000257480.1
9942	ENSG00000155890.4	ENSG00000269889.1	ENSG00000244267.1
9943	ENSG00000155893.13	ENSG00000269888.1	ENSG00000183463.5
9944	ENSG00000155897.10	ENSG00000269887.1	ENSG00000242479.1
9945	ENSG00000155903.11	ENSG00000269885.1	ENSG00000285492.1
9946	ENSG00000155906.18	ENSG00000269883.1	ENSG00000228417.1
9947	ENSG00000155918.7	ENSG00000269881.1	ENSG00000251665.1
9948	ENSG00000155926.14	ENSG00000269877.3	ENSG00000233072.1
9949	ENSG00000155957.17	ENSG00000269873.1	ENSG00000200275.1
9950	ENSG00000155959.11	ENSG00000269867.1	ENSG00000108556.9
9951	ENSG00000155961.5	ENSG00000269859.1	ENSG00000214998.2
9952	ENSG00000155962.13	ENSG00000269855.2	ENSG00000265413.1
9953	ENSG00000155966.13	ENSG00000269848.1	ENSG00000130584.11
9954	ENSG00000155970.12	ENSG00000269846.1	ENSG00000180662.8
9955	ENSG00000155974.12	ENSG00000269845.1	ENSG00000235587.2
9956	ENSG00000155975.10	ENSG00000269843.1	ENSG00000257178.5
9957	ENSG00000155980.12	ENSG00000269842.5	ENSG00000111536.5
9958	ENSG00000156006.5	ENSG00000269839.1	ENSG00000256374.2
9959	ENSG00000156009.10	ENSG00000269837.1	ENSG00000267076.1
9960	ENSG00000156011.17	ENSG00000269836.1	ENSG00000145979.18
9961	ENSG00000156017.13	ENSG00000269834.5	ENSG00000189182.9

9962	ENSG00000156026.14	ENSG00000269833.2	ENSG00000254294.1
9963	ENSG00000156030.13	ENSG00000269826.1	ENSG00000225822.4
9964	ENSG00000156042.17	ENSG00000269825.1	ENSG00000168594.15
9965	ENSG00000156049.7	ENSG00000269821.1	ENSG00000259230.1
9966	ENSG00000156050.9	ENSG00000269815.1	ENSG00000219433.2
9967	ENSG00000156052.10	ENSG00000269814.1	ENSG00000155886.11
9968	ENSG00000156076.10	ENSG00000269813.1	ENSG00000131873.7
9969	ENSG00000156096.14	ENSG00000269811.2	ENSG00000277308.1
9970	ENSG00000156097.12	ENSG00000269807.1	ENSG00000237982.1
9971	ENSG00000156103.16	ENSG00000269806.1	ENSG00000168671.10
9972	ENSG00000156110.13	ENSG00000269802.1	ENSG00000184647.11
9973	ENSG00000156113.23	ENSG00000269800.1	ENSG00000221836.3
9974	ENSG00000156127.6	ENSG00000269799.1	ENSG00000156150.8
9975	ENSG00000156136.10	ENSG00000269796.1	ENSG00000213939.4
9976	ENSG00000156140.10	ENSG00000269794.1	ENSG00000268058.1
9977	ENSG00000156150.8	ENSG00000269793.7	ENSG00000278445.1
9978	ENSG00000156162.16	ENSG00000269792.1	ENSG00000227692.1
9979	ENSG00000156170.13	ENSG00000269791.5	ENSG00000279845.1
9980	ENSG00000156171.14	ENSG00000269787.2	ENSG00000156006.5
9981	ENSG00000156172.6	ENSG00000269786.1	ENSG00000218428.1
9982	ENSG00000156194.18	ENSG00000269782.1	ENSG00000232757.1
9983	ENSG00000156206.14	ENSG00000269779.1	ENSG00000240167.1
9984	ENSG00000156218.13	ENSG00000269776.1	ENSG00000264384.2
9985	ENSG00000156219.16	ENSG00000269765.2	ENSG00000258476.5
9986	ENSG00000156222.12	ENSG00000269763.1	ENSG00000243978.8
9987	ENSG00000156232.7	ENSG00000269761.1	ENSG00000154263.17
9988	ENSG00000156234.7	ENSG00000269758.1	ENSG00000150656.15
9989	ENSG00000156239.12	ENSG00000269755.1	ENSG00000187033.9
9990	ENSG00000156253.7	ENSG00000269752.1	ENSG00000231795.1
9991	ENSG00000156256.15	ENSG00000269749.1	ENSG00000171847.10
9992	ENSG00000156261.13	ENSG00000269745.1	ENSG00000250036.1
9993	ENSG00000156265.15	ENSG00000269743.3	ENSG00000253047.1
9994	ENSG00000156269.4	ENSG00000269742.1	ENSG00000117222.14
9995	ENSG00000156273.16	ENSG00000269741.5	ENSG00000118520.14
9996	ENSG00000156282.4	ENSG00000269737.2	ENSG00000250038.6
9997	ENSG00000156284.5	ENSG00000269736.1	ENSG00000223658.8
9998	ENSG00000156298.12	ENSG00000269732.1	ENSG00000237655.1
9999	ENSG00000156299.13	ENSG00000269729.1	ENSG00000268070.1
10000	ENSG00000156304.14	ENSG00000269720.2	ENSG00000197070.14
10001	ENSG00000156313.13	ENSG00000269713.7	ENSG00000243020.1
10002	ENSG00000156345.17	ENSG00000269711.1	ENSG00000253891.1
10003	ENSG00000156374.16	ENSG00000269707.1	ENSG00000225039.1
10004	ENSG00000156381.9	ENSG00000269706.1	ENSG00000184937.14
10005	ENSG00000156384.14	ENSG00000269699.6	ENSG00000282024.1
10006	ENSG00000156395.13	ENSG00000269696.1	ENSG00000274758.1
10007	ENSG00000156398.13	ENSG00000269694.1	ENSG00000237765.7
10008	ENSG00000156411.9	ENSG00000269693.1	ENSG00000255931.1
10009	ENSG00000156413.13	ENSG00000269692.1	ENSG00000178999.13
10010	ENSG00000156414.19	ENSG00000269688.1	ENSG00000101307.15
10011	ENSG00000156427.8	ENSG00000269680.1	ENSG00000224334.1
10012	ENSG00000156453.13	ENSG00000269678.1	ENSG00000242791.2
10013	ENSG00000156463.18	ENSG00000269667.2	ENSG00000228046.2
10014	ENSG00000156466.10	ENSG00000269662.1	ENSG00000143368.10

10015	ENSG00000156467.9	ENSG00000269656.1	ENSG00000217897.2
10016	ENSG00000156469.8	ENSG00000269653.1	ENSG00000271401.1
10017	ENSG00000156471.12	ENSG00000269652.1	ENSG00000267130.1
10018	ENSG00000156475.18	ENSG00000269651.1	ENSG00000226944.1
10019	ENSG00000156482.11	ENSG00000269646.1	ENSG00000264540.2
10020	ENSG00000156486.7	ENSG00000269637.1	ENSG00000271220.2
10021	ENSG00000156500.15	ENSG00000269635.1	ENSG00000249965.2
10022	ENSG00000156502.14	ENSG00000269622.1	ENSG00000249763.1
10023	ENSG00000156504.16	ENSG00000269621.1	ENSG00000206145.8
10024	ENSG00000156508.18	ENSG00000269615.1	ENSG00000108878.5
10025	ENSG00000156509.14	ENSG00000269609.5	ENSG00000087157.19
10026	ENSG00000156510.13	ENSG00000269608.1	ENSG00000285177.1
10027	ENSG00000156515.23	ENSG00000269604.1	ENSG00000264545.1
10028	ENSG00000156521.14	ENSG00000269600.1	ENSG00000227070.1
10029	ENSG00000156531.16	ENSG00000269599.1	ENSG00000237285.1
10030	ENSG00000156535.15	ENSG00000269590.1	ENSG00000252707.1
10031	ENSG00000156564.8	ENSG00000269588.1	ENSG00000051341.14
10032	ENSG00000156574.9	ENSG00000269586.7	ENSG00000156885.6
10033	ENSG00000156575.2	ENSG00000269584.1	ENSG00000234927.1
10034	ENSG00000156587.16	ENSG00000269583.1	ENSG00000216835.3
10035	ENSG00000156599.11	ENSG00000269580.2	ENSG00000167904.15
10036	ENSG00000156603.18	ENSG00000269578.1	ENSG00000258337.1
10037	ENSG00000156639.12	ENSG00000269576.1	ENSG00000202415.1
10038	ENSG00000156642.16	ENSG00000269575.1	ENSG00000213994.3
10039	ENSG00000156650.14	ENSG00000269570.2	ENSG00000133063.16
10040	ENSG00000156671.14	ENSG00000269565.1	ENSG00000229418.2
10041	ENSG00000156675.15	ENSG00000269564.1	ENSG00000149600.11
10042	ENSG00000156687.11	ENSG00000269560.1	ENSG00000188056.11
10043	ENSG00000156689.7	ENSG00000269559.2	ENSG00000258477.1
10044	ENSG00000156697.13	ENSG00000269556.8	ENSG00000245711.2
10045	ENSG00000156709.14	ENSG00000269553.1	ENSG00000198121.13
10046	ENSG00000156711.17	ENSG00000269552.1	ENSG00000232842.2
10047	ENSG00000156735.11	ENSG00000269547.1	ENSG00000283324.1
10048	ENSG00000156738.17	ENSG00000269546.1	ENSG00000251131.1
10049	ENSG00000156755.10	ENSG00000269543.5	ENSG00000187944.2
10050	ENSG00000156787.17	ENSG00000269540.1	ENSG00000255398.2
10051	ENSG00000156795.7	ENSG00000269535.1	ENSG00000284783.1
10052	ENSG00000156802.13	ENSG00000269534.5	ENSG00000115738.10
10053	ENSG00000156804.7	ENSG00000269533.5	ENSG00000259357.2
10054	ENSG00000156831.8	ENSG00000269526.1	ENSG00000102239.5
10055	ENSG00000156853.12	ENSG00000269524.1	ENSG00000188078.5
10056	ENSG00000156858.11	ENSG00000269519.1	ENSG00000239345.2
10057	ENSG00000156860.15	ENSG00000269516.6	ENSG00000185614.5
10058	ENSG00000156869.13	ENSG00000269514.2	ENSG00000223575.2
10059	ENSG00000156873.16	ENSG00000269509.1	ENSG00000203321.2
10060	ENSG00000156875.14	ENSG00000269506.2	ENSG00000232075.1
10061	ENSG00000156876.10	ENSG00000269505.2	ENSG00000277758.4
10062	ENSG00000156885.6	ENSG00000269504.2	ENSG00000139329.5
10063	ENSG00000156886.11	ENSG00000269502.5	ENSG00000149654.9
10064	ENSG00000156920.10	ENSG00000269495.1	ENSG00000225981.1
10065	ENSG00000156925.12	ENSG00000269489.1	ENSG00000172538.6
10066	ENSG00000156928.4	ENSG00000269487.1	ENSG00000241114.1
10067	ENSG00000156931.15	ENSG00000269486.2	ENSG00000256546.1

10068	ENSG00000156958.15	ENSG00000269483.2	ENSG00000270941.1
10069	ENSG00000156959.9	ENSG00000269482.1	ENSG00000237349.1
10070	ENSG00000156966.7	ENSG00000269481.1	ENSG00000053108.17
10071	ENSG00000156968.9	ENSG00000269480.1	ENSG00000216775.3
10072	ENSG00000156970.12	ENSG00000269476.1	ENSG00000179292.5
10073	ENSG00000156973.14	ENSG00000269475.2	ENSG00000279243.2
10074	ENSG00000156976.17	ENSG00000269473.1	ENSG00000185686.18
10075	ENSG00000156983.15	ENSG00000269471.1	ENSG00000272702.1
10076	ENSG00000156990.14	ENSG00000269469.1	ENSG00000238180.1
10077	ENSG00000157005.4	ENSG00000269466.3	ENSG00000206579.8
10078	ENSG00000157014.11	ENSG00000269463.1	ENSG00000228292.1
10079	ENSG00000157017.15	ENSG00000269460.1	ENSG00000254536.1
10080	ENSG00000157020.18	ENSG00000269458.1	ENSG00000164175.15
10081	ENSG00000157021.9	ENSG00000269446.2	ENSG00000267641.1
10082	ENSG00000157036.13	ENSG00000269445.1	ENSG00000280341.1
10083	ENSG00000157045.9	ENSG00000269444.1	ENSG00000230665.3
10084	ENSG00000157060.16	ENSG00000269439.5	ENSG00000233060.1
10085	ENSG00000157064.11	ENSG00000269437.7	ENSG00000270733.1
10086	ENSG00000157077.14	ENSG00000269433.3	ENSG00000152684.11
10087	ENSG00000157087.19	ENSG00000269431.1	ENSG00000176728.8
10088	ENSG00000157093.9	ENSG00000269427.1	ENSG00000277587.1
10089	ENSG00000157103.12	ENSG00000269425.1	ENSG00000240738.1
10090	ENSG00000157106.16	ENSG00000269421.1	ENSG00000147689.16
10091	ENSG00000157107.14	ENSG00000269420.5	ENSG00000198729.5
10092	ENSG00000157110.16	ENSG00000269419.1	ENSG00000170837.2
10093	ENSG00000157111.13	ENSG00000269416.5	ENSG00000235288.3
10094	ENSG00000157119.11	ENSG00000269405.6	ENSG00000260404.3
10095	ENSG00000157131.10	ENSG00000269404.7	ENSG00000136110.13
10096	ENSG00000157150.5	ENSG00000269403.1	ENSG00000200198.1
10097	ENSG00000157152.17	ENSG00000269400.1	ENSG00000140287.11
10098	ENSG00000157168.19	ENSG00000269399.2	ENSG00000214897.4
10099	ENSG00000157181.16	ENSG00000269397.1	ENSG00000214195.4
10100	ENSG00000157184.7	ENSG00000269392.1	ENSG00000254112.1
10101	ENSG00000157191.20	ENSG00000269391.1	ENSG00000228139.1
10102	ENSG00000157193.16	ENSG00000269388.1	ENSG00000266420.2
10103	ENSG00000157211.11	ENSG00000269387.1	ENSG00000261744.1
10104	ENSG00000157212.18	ENSG00000269386.5	ENSG00000006756.16
10105	ENSG00000157214.14	ENSG00000269385.1	ENSG00000235695.1
10106	ENSG00000157216.15	ENSG00000269383.2	ENSG00000166984.11
10107	ENSG00000157219.4	ENSG00000269378.1	ENSG00000140931.20
10108	ENSG00000157224.16	ENSG00000269376.1	ENSG00000197258.5
10109	ENSG00000157227.13	ENSG00000269374.1	ENSG00000227154.4
10110	ENSG00000157240.3	ENSG00000269373.1	ENSG00000274021.1
10111	ENSG00000157259.8	ENSG00000269371.1	ENSG00000259015.1
10112	ENSG00000157303.11	ENSG00000269365.2	ENSG00000229484.1
10113	ENSG00000157306.14	ENSG00000269364.1	ENSG00000266783.1
10114	ENSG00000157315.5	ENSG00000269356.1	ENSG00000253412.1
10115	ENSG00000157322.17	ENSG00000269354.1	ENSG00000213478.2
10116	ENSG00000157326.19	ENSG00000269353.1	ENSG00000106733.21
10117	ENSG00000157330.10	ENSG00000269352.1	ENSG00000229593.1
10118	ENSG00000157335.20	ENSG00000269350.1	ENSG00000011028.14
10119	ENSG00000157343.8	ENSG00000269349.1	ENSG00000241129.3
10120	ENSG00000157349.16	ENSG00000269345.1	ENSG00000133561.15

10121	ENSG00000157350.13	ENSG00000269343.7	ENSG00000243373.3
10122	ENSG00000157353.17	ENSG00000269335.5	ENSG00000254907.1
10123	ENSG00000157368.10	ENSG00000269332.5	ENSG00000146966.13
10124	ENSG00000157379.14	ENSG00000269321.1	ENSG00000270915.1
10125	ENSG00000157388.17	ENSG00000269320.1	ENSG00000278473.1
10126	ENSG00000157399.14	ENSG00000269318.1	ENSG00000284934.1
10127	ENSG00000157404.15	ENSG00000269316.1	ENSG00000225502.2
10128	ENSG00000157423.18	ENSG00000269313.5	ENSG00000283209.1
10129	ENSG00000157426.14	ENSG00000269307.1	ENSG00000151005.4
10130	ENSG00000157429.15	ENSG00000269304.1	ENSG00000278704.1
10131	ENSG00000157445.15	ENSG00000269303.1	ENSG00000257341.5
10132	ENSG00000157450.15	ENSG00000269300.1	ENSG00000261617.1
10133	ENSG00000157456.8	ENSG00000269296.1	ENSG00000171722.12
10134	ENSG00000157470.12	ENSG00000269293.2	ENSG00000244383.2
10135	ENSG00000157483.8	ENSG00000269292.1	ENSG00000076928.17
10136	ENSG00000157500.12	ENSG00000269289.5	ENSG00000260886.1
10137	ENSG00000157502.13	ENSG00000269288.1	ENSG00000092345.13
10138	ENSG00000157510.14	ENSG00000269281.2	ENSG00000230542.6
10139	ENSG00000157514.16	ENSG00000269275.1	ENSG00000277196.4
10140	ENSG00000157538.14	ENSG00000269274.1	ENSG00000140522.12
10141	ENSG00000157540.21	ENSG00000269271.1	ENSG00000282907.1
10142	ENSG00000157542.11	ENSG00000269266.1	ENSG00000120658.13
10143	ENSG00000157551.19	ENSG00000269256.1	ENSG00000275152.5
10144	ENSG00000157554.19	ENSG00000269253.1	ENSG00000118197.14
10145	ENSG00000157557.12	ENSG00000269246.1	ENSG00000133800.8
10146	ENSG00000157570.11	ENSG00000269244.1	ENSG00000224961.2
10147	ENSG00000157578.13	ENSG00000269243.1	ENSG00000233260.1
10148	ENSG00000157593.19	ENSG00000269242.1	ENSG00000162413.16
10149	ENSG00000157600.12	ENSG00000269237.1	ENSG00000253313.5
10150	ENSG00000157601.14	ENSG00000269236.1	ENSG00000182687.4
10151	ENSG00000157613.10	ENSG00000269235.1	ENSG00000134545.13
10152	ENSG00000157617.17	ENSG00000269228.1	ENSG00000198642.6
10153	ENSG00000157625.15	ENSG00000269226.7	ENSG00000242697.2
10154	ENSG00000157637.13	ENSG00000269220.1	ENSG00000257579.1
10155	ENSG00000157653.11	ENSG00000269210.2	ENSG00000280285.1
10156	ENSG00000157654.17	ENSG00000269191.1	ENSG00000125246.15
10157	ENSG00000157657.14	ENSG00000269190.6	ENSG00000267255.1
10158	ENSG00000157680.15	ENSG00000269189.1	ENSG00000218803.1
10159	ENSG00000157693.15	ENSG00000269188.1	ENSG00000275479.1
10160	ENSG00000157703.15	ENSG00000269186.2	ENSG00000129009.13
10161	ENSG00000157734.14	ENSG00000269181.1	ENSG00000273855.1
10162	ENSG00000157741.15	ENSG00000269179.1	ENSG00000166535.20
10163	ENSG00000157764.13	ENSG00000269177.1	ENSG00000233953.1
10164	ENSG00000157765.13	ENSG00000269176.2	ENSG00000204049.1
10165	ENSG00000157766.16	ENSG00000269172.1	ENSG00000224011.1
10166	ENSG00000157778.8	ENSG00000269161.1	ENSG00000135406.14
10167	ENSG00000157782.9	ENSG00000269155.2	ENSG00000235098.8
10168	ENSG00000157796.18	ENSG00000269154.1	ENSG00000090889.12
10169	ENSG00000157800.18	ENSG00000269153.1	ENSG00000228343.1
10170	ENSG00000157823.17	ENSG00000269151.1	ENSG00000241785.3
10171	ENSG00000157827.20	ENSG00000269148.1	ENSG00000257464.1
10172	ENSG00000157833.13	ENSG00000269145.2	ENSG00000268362.5
10173	ENSG00000157837.16	ENSG00000269139.2	ENSG00000276087.2

10174	ENSG00000157851.17	ENSG00000269138.1	ENSG00000286178.1
10175	ENSG00000157856.12	ENSG00000269136.1	ENSG00000273471.1
10176	ENSG00000157869.15	ENSG00000269130.1	ENSG00000233577.6
10177	ENSG00000157870.16	ENSG00000269125.1	ENSG00000233396.7
10178	ENSG00000157873.17	ENSG00000269124.1	ENSG00000164078.13
10179	ENSG00000157881.14	ENSG00000269119.1	ENSG00000227087.4
10180	ENSG00000157884.11	ENSG00000269118.1	ENSG00000232202.1
10181	ENSG00000157890.17	ENSG00000269113.4	ENSG00000252722.1
10182	ENSG00000157895.11	ENSG00000269110.1	ENSG00000243847.3
10183	ENSG00000157911.10	ENSG00000269107.1	ENSG00000258410.1
10184	ENSG00000157916.20	ENSG00000269106.1	ENSG00000225008.1
10185	ENSG00000157927.16	ENSG00000269103.2	ENSG00000135409.11
10186	ENSG00000157933.10	ENSG00000269102.1	ENSG00000173926.6
10187	ENSG00000157954.15	ENSG00000269099.2	ENSG00000235021.1
10188	ENSG00000157965.11	ENSG00000269097.1	ENSG00000183090.5
10189	ENSG00000157978.11	ENSG00000269096.6	ENSG00000228463.10
10190	ENSG00000157985.19	ENSG00000269095.1	ENSG00000111670.16
10191	ENSG00000157992.12	ENSG00000269091.5	ENSG00000239964.3
10192	ENSG00000157999.5	ENSG00000269086.2	ENSG00000215386.12
10193	ENSG00000158006.14	ENSG00000269085.1	ENSG00000286188.1
10194	ENSG00000158008.9	ENSG00000269082.1	ENSG00000166143.9
10195	ENSG00000158014.14	ENSG00000269072.1	ENSG00000239701.1
10196	ENSG00000158019.20	ENSG00000269069.1	ENSG00000267440.1
10197	ENSG00000158022.6	ENSG00000269068.1	ENSG00000198169.9
10198	ENSG00000158023.10	ENSG00000269067.1	ENSG00000262185.2
10199	ENSG00000158042.8	ENSG00000269066.1	ENSG00000214290.8
10200	ENSG00000158050.5	ENSG00000269058.5	ENSG00000203825.4
10201	ENSG00000158055.15	ENSG00000269055.1	ENSG00000168078.10
10202	ENSG00000158062.20	ENSG00000269054.1	ENSG00000145386.10
10203	ENSG00000158077.4	ENSG00000269053.1	ENSG00000235979.8
10204	ENSG00000158079.16	ENSG00000269050.1	ENSG00000101333.16
10205	ENSG00000158089.15	ENSG00000269044.2	ENSG00000241020.1
10206	ENSG00000158092.7	ENSG00000269043.1	ENSG00000206549.13
10207	ENSG00000158104.11	ENSG00000269040.1	ENSG00000260870.1
10208	ENSG00000158106.14	ENSG00000269038.1	ENSG00000108352.12
10209	ENSG00000158109.15	ENSG00000269037.1	ENSG00000228848.3
10210	ENSG00000158113.13	ENSG00000269035.1	ENSG00000188223.9
10211	ENSG00000158122.12	ENSG00000269032.1	ENSG00000265078.2
10212	ENSG00000158125.10	ENSG00000269028.3	ENSG00000067113.17
10213	ENSG00000158156.7	ENSG00000269026.2	ENSG00000235893.5
10214	ENSG00000158158.12	ENSG00000269025.2	ENSG00000285165.1
10215	ENSG00000158161.16	ENSG00000269021.1	ENSG00000217929.4
10216	ENSG00000158163.15	ENSG00000269019.1	ENSG00000261239.6
10217	ENSG00000158164.7	ENSG00000269014.1	ENSG00000231700.3
10218	ENSG00000158169.13	ENSG00000269012.2	ENSG00000110848.8
10219	ENSG00000158186.13	ENSG00000269009.1	ENSG00000258924.4
10220	ENSG00000158195.11	ENSG00000269001.2	ENSG00000167494.9
10221	ENSG00000158201.10	ENSG00000268997.1	ENSG00000254959.6
10222	ENSG00000158220.14	ENSG00000268996.3	ENSG00000119636.16
10223	ENSG00000158234.12	ENSG00000268995.1	ENSG00000225365.1
10224	ENSG00000158246.8	ENSG00000268994.3	ENSG00000242175.3
10225	ENSG00000158258.16	ENSG00000268993.1	ENSG00000213244.3
10226	ENSG00000158270.12	ENSG00000268992.1	ENSG00000220305.1

10227	ENSG00000158286.13	ENSG00000268988.1	ENSG00000276757.1
10228	ENSG00000158290.16	ENSG00000268987.1	ENSG00000180437.5
10229	ENSG00000158292.7	ENSG00000268985.1	ENSG00000221986.7
10230	ENSG00000158296.14	ENSG00000268983.1	ENSG00000259453.1
10231	ENSG00000158301.18	ENSG00000268981.5	ENSG00000230970.3
10232	ENSG00000158315.11	ENSG00000268975.2	ENSG00000233818.1
10233	ENSG00000158321.16	ENSG00000268970.1	ENSG00000235847.2
10234	ENSG00000158352.15	ENSG00000268967.1	ENSG00000199975.1
10235	ENSG00000158373.8	ENSG00000268964.2	ENSG00000211661.2
10236	ENSG00000158402.20	ENSG00000268957.1	ENSG00000284797.1
10237	ENSG00000158406.4	ENSG00000268955.2	ENSG00000131721.5
10238	ENSG00000158411.11	ENSG00000268951.1	ENSG00000253773.2
10239	ENSG00000158417.11	ENSG00000268949.1	ENSG00000267063.1
10240	ENSG00000158423.17	ENSG00000268947.1	ENSG00000197665.7
10241	ENSG00000158427.15	ENSG00000268945.1	ENSG00000242207.1
10242	ENSG00000158428.4	ENSG00000268942.2	ENSG00000122691.13
10243	ENSG00000158435.8	ENSG00000268941.2	ENSG00000242941.1
10244	ENSG00000158445.10	ENSG00000268940.5	ENSG00000271639.1
10245	ENSG00000158457.6	ENSG00000268938.2	ENSG00000211935.3
10246	ENSG00000158458.20	ENSG00000268931.1	ENSG00000235272.2
10247	ENSG00000158467.16	ENSG00000268926.3	ENSG00000266498.1
10248	ENSG00000158470.5	ENSG00000268922.1	ENSG00000241568.3
10249	ENSG00000158473.6	ENSG00000268916.6	ENSG00000136999.5
10250	ENSG00000158477.7	ENSG00000268912.1	ENSG00000213145.9
10251	ENSG00000158480.11	ENSG00000268908.1	ENSG00000220867.1
10252	ENSG00000158481.13	ENSG00000268906.1	ENSG00000215833.3
10253	ENSG00000158482.10	ENSG00000268903.1	ENSG00000232742.3
10254	ENSG00000158483.16	ENSG00000268902.3	ENSG00000100600.15
10255	ENSG00000158485.10	ENSG00000268896.1	ENSG00000277778.2
10256	ENSG00000158486.13	ENSG00000268895.5	ENSG00000258186.2
10257	ENSG00000158488.16	ENSG00000268894.6	ENSG00000274514.1
10258	ENSG00000158497.3	ENSG00000268892.1	ENSG00000223177.1
10259	ENSG00000158516.12	ENSG00000268889.1	ENSG00000257848.1
10260	ENSG00000158517.13	ENSG00000268886.1	ENSG00000177628.16
10261	ENSG00000158525.15	ENSG00000268884.1	ENSG00000166831.9
10262	ENSG00000158526.8	ENSG00000268879.1	ENSG00000156711.17
10263	ENSG00000158528.12	ENSG00000268873.1	ENSG00000145283.7
10264	ENSG00000158545.15	ENSG00000268870.1	ENSG00000254602.1
10265	ENSG00000158552.13	ENSG00000268869.5	ENSG00000197530.12
10266	ENSG00000158553.4	ENSG00000268866.1	ENSG00000127870.17
10267	ENSG00000158555.15	ENSG00000268864.4	ENSG00000275585.2
10268	ENSG00000158560.14	ENSG00000268861.6	ENSG00000150510.16
10269	ENSG00000158571.10	ENSG00000268858.2	ENSG00000223086.1
10270	ENSG00000158578.20	ENSG00000268854.1	ENSG00000229877.1
10271	ENSG00000158604.15	ENSG00000268849.5	ENSG00000265739.1
10272	ENSG00000158615.9	ENSG00000268847.1	ENSG00000259895.1
10273	ENSG00000158623.14	ENSG00000268845.1	ENSG00000251129.1
10274	ENSG00000158636.16	ENSG00000268842.1	ENSG00000249396.2
10275	ENSG00000158639.12	ENSG00000268839.1	ENSG00000164989.17
10276	ENSG00000158669.11	ENSG00000268836.1	ENSG00000169551.12
10277	ENSG00000158683.8	ENSG00000268834.2	ENSG00000269103.2
10278	ENSG00000158691.14	ENSG00000268833.1	ENSG00000076770.14
10279	ENSG00000158710.14	ENSG00000268830.1	ENSG00000224287.2

10280	ENSG00000158711.13	ENSG00000268823.2	ENSG00000254409.3
10281	ENSG00000158714.11	ENSG00000268818.2	ENSG00000238140.1
10282	ENSG00000158715.6	ENSG00000268812.3	ENSG00000265943.1
10283	ENSG00000158716.8	ENSG00000268810.1	ENSG00000121390.18
10284	ENSG00000158717.10	ENSG00000268804.1	ENSG00000187867.8
10285	ENSG00000158731.2	ENSG00000268803.1	ENSG00000226673.2
10286	ENSG00000158747.14	ENSG00000268799.3	ENSG00000226472.8
10287	ENSG00000158748.4	ENSG00000268798.1	ENSG00000221792.1
10288	ENSG00000158764.7	ENSG00000268797.1	ENSG00000218890.1
10289	ENSG00000158769.18	ENSG00000268794.1	ENSG00000248449.2
10290	ENSG00000158773.14	ENSG00000268790.5	ENSG00000214628.3
10291	ENSG00000158786.4	ENSG00000268789.1	ENSG00000275228.1
10292	ENSG00000158792.16	ENSG00000268785.1	ENSG00000224117.1
10293	ENSG00000158793.14	ENSG00000268777.1	ENSG00000235449.2
10294	ENSG00000158796.17	ENSG00000268764.1	ENSG00000281087.1
10295	ENSG00000158805.12	ENSG00000268758.7	ENSG00000236869.1
10296	ENSG00000158806.14	ENSG00000268756.1	ENSG00000243648.1
10297	ENSG00000158813.18	ENSG00000268754.3	ENSG00000286113.1
10298	ENSG00000158815.11	ENSG00000268751.1	ENSG00000008197.4
10299	ENSG00000158816.15	ENSG00000268750.6	ENSG00000164039.15
10300	ENSG00000158825.6	ENSG00000268747.1	ENSG00000269534.5
10301	ENSG00000158828.8	ENSG00000268746.1	ENSG00000130826.18
10302	ENSG00000158850.15	ENSG00000268745.1	ENSG00000258024.1
10303	ENSG00000158856.18	ENSG00000268744.1	ENSG00000101440.9
10304	ENSG00000158859.10	ENSG00000268743.1	ENSG00000068697.7
10305	ENSG00000158863.22	ENSG00000268742.1	ENSG00000234817.2
10306	ENSG00000158864.12	ENSG00000268739.1	ENSG00000268140.1
10307	ENSG00000158865.12	ENSG00000268736.1	ENSG00000018625.14
10308	ENSG00000158869.11	ENSG00000268734.1	ENSG00000258510.2
10309	ENSG00000158874.11	ENSG00000268731.1	ENSG00000006837.12
10310	ENSG00000158882.15	ENSG00000268729.1	ENSG00000225214.1
10311	ENSG00000158887.16	ENSG00000268723.1	ENSG00000251224.1
10312	ENSG00000158901.11	ENSG00000268717.1	ENSG00000229721.3
10313	ENSG00000158941.16	ENSG00000268713.1	ENSG00000188554.14
10314	ENSG00000158955.11	ENSG00000268711.1	ENSG00000263477.1
10315	ENSG00000158966.15	ENSG00000268707.1	ENSG00000233685.1
10316	ENSG00000158985.13	ENSG00000268705.1	ENSG00000101955.15
10317	ENSG00000158987.20	ENSG00000268696.2	ENSG00000064270.12
10318	ENSG00000159023.21	ENSG00000268686.1	ENSG00000105467.8
10319	ENSG00000159055.4	ENSG00000268685.1	ENSG00000246115.3
10320	ENSG00000159063.13	ENSG00000268683.1	ENSG00000119121.22
10321	ENSG00000159069.14	ENSG00000268681.1	ENSG00000269421.1
10322	ENSG00000159079.19	ENSG00000268678.1	ENSG00000235862.2
10323	ENSG00000159082.17	ENSG00000268677.1	ENSG00000274630.1
10324	ENSG00000159086.15	ENSG00000268674.2	ENSG00000168765.17
10325	ENSG00000159110.19	ENSG00000268673.3	ENSG00000227117.6
10326	ENSG00000159111.13	ENSG00000268670.1	ENSG00000142182.8
10327	ENSG00000159128.14	ENSG00000268669.1	ENSG00000103187.8
10328	ENSG00000159131.17	ENSG00000268663.1	ENSG00000170409.7
10329	ENSG00000159140.20	ENSG00000268660.1	ENSG00000203581.7
10330	ENSG00000159147.18	ENSG00000268659.2	ENSG00000277922.1
10331	ENSG00000159164.10	ENSG00000268658.5	ENSG00000249947.2
10332	ENSG00000159166.13	ENSG00000268655.2	ENSG00000218596.2

10333	ENSG00000159167.12	ENSG00000268654.2	ENSG00000229256.1
10334	ENSG00000159173.19	ENSG00000268652.1	ENSG00000259823.6
10335	ENSG00000159176.14	ENSG00000268651.3	ENSG00000197847.12
10336	ENSG00000159182.5	ENSG00000268650.3	ENSG00000229160.1
10337	ENSG00000159184.7	ENSG00000268649.5	ENSG00000282301.2
10338	ENSG00000159186.4	ENSG00000268643.1	ENSG00000261427.6
10339	ENSG00000159189.12	ENSG00000268636.1	ENSG00000136158.12
10340	ENSG00000159197.3	ENSG00000268635.2	ENSG00000105694.3
10341	ENSG00000159199.14	ENSG00000268629.6	ENSG00000236670.1
10342	ENSG00000159200.18	ENSG00000268628.2	ENSG00000253173.3
10343	ENSG00000159202.18	ENSG00000268623.2	ENSG00000226688.6
10344	ENSG00000159208.16	ENSG00000268621.5	ENSG00000266258.1
10345	ENSG00000159210.9	ENSG00000268620.1	ENSG00000285544.1
10346	ENSG00000159212.12	ENSG00000268618.1	ENSG00000234036.4
10347	ENSG00000159214.13	ENSG00000268615.1	ENSG00000277125.1
10348	ENSG00000159216.18	ENSG00000268614.1	ENSG00000224643.5
10349	ENSG00000159217.10	ENSG00000268606.5	ENSG00000100266.19
10350	ENSG00000159224.4	ENSG00000268605.1	ENSG00000204044.6
10351	ENSG00000159228.13	ENSG00000268603.1	ENSG00000242611.2
10352	ENSG00000159231.6	ENSG00000268601.1	ENSG00000267691.1
10353	ENSG00000159239.13	ENSG00000268598.1	ENSG00000264932.3
10354	ENSG00000159247.13	ENSG00000268597.1	ENSG00000276454.1
10355	ENSG00000159248.5	ENSG00000268595.1	ENSG00000089101.18
10356	ENSG00000159256.13	ENSG00000268593.3	ENSG00000226179.6
10357	ENSG00000159259.8	ENSG00000268592.3	ENSG00000278621.1
10358	ENSG00000159261.11	ENSG00000268589.1	ENSG00000225555.1
10359	ENSG00000159263.15	ENSG00000268584.1	ENSG00000215692.2
10360	ENSG00000159267.14	ENSG00000268583.1	ENSG00000230064.1
10361	ENSG00000159289.6	ENSG00000268582.1	ENSG00000263940.2
10362	ENSG00000159307.19	ENSG00000268581.1	ENSG00000234946.1
10363	ENSG00000159314.11	ENSG00000268580.1	ENSG00000182040.9
10364	ENSG00000159322.17	ENSG00000268575.1	ENSG00000259520.5
10365	ENSG00000159335.16	ENSG00000268573.1	ENSG00000270679.1
10366	ENSG00000159337.6	ENSG00000268566.5	ENSG00000222630.2
10367	ENSG00000159339.13	ENSG00000268565.1	ENSG00000136379.12
10368	ENSG00000159346.13	ENSG00000268564.1	ENSG00000253582.1
10369	ENSG00000159348.13	ENSG00000268560.1	ENSG00000280767.3
10370	ENSG00000159352.15	ENSG00000268555.1	ENSG00000253989.2
10371	ENSG00000159363.18	ENSG00000268545.1	ENSG00000213047.13
10372	ENSG00000159374.17	ENSG00000268543.1	ENSG00000224081.9
10373	ENSG00000159377.11	ENSG00000268541.1	ENSG00000183844.16
10374	ENSG00000159387.7	ENSG00000268536.1	ENSG00000229880.1
10375	ENSG00000159388.6	ENSG00000268535.1	ENSG00000197067.6
10376	ENSG00000159398.15	ENSG00000268533.1	ENSG00000248719.1
10377	ENSG00000159399.9	ENSG00000268532.1	ENSG00000196557.13
10378	ENSG00000159403.17	ENSG00000268531.3	ENSG00000256540.1
10379	ENSG00000159409.14	ENSG00000268530.5	ENSG00000271644.1
10380	ENSG00000159423.17	ENSG00000268529.1	ENSG00000286142.1
10381	ENSG00000159433.12	ENSG00000268521.1	ENSG00000226114.1
10382	ENSG00000159445.13	ENSG00000268520.1	ENSG00000071054.16
10383	ENSG00000159450.12	ENSG00000268518.1	ENSG00000268203.1
10384	ENSG00000159455.8	ENSG00000268516.2	ENSG00000138944.8
10385	ENSG00000159459.12	ENSG00000268510.1	ENSG00000222267.1

10386	ENSG00000159461.15	ENSG00000268509.2	ENSG00000207008.1
10387	ENSG00000159479.17	ENSG00000268505.1	ENSG00000132518.6
10388	ENSG00000159495.7	ENSG00000268500.5	ENSG00000279668.2
10389	ENSG00000159496.14	ENSG00000268499.1	ENSG00000230551.4
10390	ENSG00000159516.9	ENSG00000268496.1	ENSG00000102683.7
10391	ENSG00000159527.3	ENSG00000268486.5	ENSG00000233780.3
10392	ENSG00000159556.10	ENSG00000268483.1	ENSG00000113407.13
10393	ENSG00000159579.14	ENSG00000268480.1	ENSG00000174343.6
10394	ENSG00000159588.15	ENSG00000268475.1	ENSG00000263709.1
10395	ENSG00000159592.11	ENSG00000268473.1	ENSG00000280286.1
10396	ENSG00000159593.15	ENSG00000268472.2	ENSG00000179277.9
10397	ENSG00000159596.7	ENSG00000268471.6	ENSG00000224041.3
10398	ENSG00000159618.16	ENSG00000268469.1	ENSG00000230542.6
10399	ENSG00000159625.14	ENSG00000268465.1	ENSG00000272172.1
10400	ENSG00000159640.16	ENSG00000268461.2	ENSG00000176890.16
10401	ENSG00000159648.11	ENSG00000268460.1	ENSG00000198000.12
10402	ENSG00000159650.9	ENSG00000268458.1	ENSG00000258216.6
10403	ENSG00000159658.11	ENSG00000268455.1	ENSG00000117751.18
10404	ENSG00000159674.12	ENSG00000268447.5	ENSG00000132819.17
10405	ENSG00000159685.10	ENSG00000268442.1	ENSG00000182473.22
10406	ENSG00000159692.15	ENSG00000268438.1	ENSG00000112679.14
10407	ENSG00000159708.18	ENSG00000268434.5	ENSG00000187054.15
10408	ENSG00000159712.10	ENSG00000268433.1	ENSG00000267166.5
10409	ENSG00000159713.11	ENSG00000268423.4	ENSG00000250938.5
10410	ENSG00000159714.11	ENSG00000268416.1	ENSG00000253759.1
10411	ENSG00000159720.12	ENSG00000268412.2	ENSG00000266950.1
10412	ENSG00000159723.4	ENSG00000268407.1	ENSG00000273812.2
10413	ENSG00000159733.13	ENSG00000268403.2	ENSG00000273979.1
10414	ENSG00000159753.14	ENSG00000268401.1	ENSG00000100362.13
10415	ENSG00000159761.15	ENSG00000268400.5	ENSG00000272864.1
10416	ENSG00000159763.4	ENSG00000268392.1	ENSG00000188643.11
10417	ENSG00000159784.17	ENSG00000268391.1	ENSG00000223821.1
10418	ENSG00000159788.19	ENSG00000268390.1	ENSG00000226008.1
10419	ENSG00000159792.10	ENSG00000268388.5	ENSG00000235304.1
10420	ENSG00000159840.16	ENSG00000268379.1	ENSG00000111249.14
10421	ENSG00000159842.15	ENSG00000268375.1	ENSG00000124143.10
10422	ENSG00000159860.7	ENSG00000268366.1	ENSG00000270716.1
10423	ENSG00000159871.15	ENSG00000268364.1	ENSG00000259132.1
10424	ENSG00000159873.10	ENSG00000268362.5	ENSG00000145536.15
10425	ENSG00000159882.13	ENSG00000268361.1	ENSG00000105697.9
10426	ENSG00000159884.12	ENSG00000268357.1	ENSG00000181234.9
10427	ENSG00000159885.14	ENSG00000268355.1	ENSG00000258689.1
10428	ENSG00000159899.14	ENSG00000268350.7	ENSG00000250390.2
10429	ENSG00000159904.11	ENSG00000268336.1	ENSG00000156486.7
10430	ENSG00000159905.14	ENSG00000268335.1	ENSG00000201441.1
10431	ENSG00000159915.12	ENSG00000268333.1	ENSG00000268496.1
10432	ENSG00000159917.17	ENSG00000268326.1	ENSG00000249859.10
10433	ENSG00000159921.15	ENSG00000268324.2	ENSG00000282304.1
10434	ENSG00000159958.6	ENSG00000268322.1	ENSG00000282308.1
10435	ENSG00000159961.2	ENSG00000268320.3	ENSG00000158445.10
10436	ENSG00000160007.18	ENSG00000268318.5	ENSG00000247877.6
10437	ENSG00000160013.9	ENSG00000268316.1	ENSG00000214534.5
10438	ENSG00000160014.16	ENSG00000268309.1	ENSG00000233705.6

10439	ENSG00000160049.12	ENSG00000268307.1	ENSG00000226801.2
10440	ENSG00000160050.14	ENSG00000268297.1	ENSG00000236031.1
10441	ENSG00000160051.11	ENSG00000268296.2	ENSG00000250762.1
10442	ENSG00000160055.19	ENSG00000268295.1	ENSG00000260519.1
10443	ENSG00000160058.18	ENSG00000268293.1	ENSG00000200558.1
10444	ENSG00000160062.15	ENSG00000268292.1	ENSG00000206195.10
10445	ENSG00000160072.19	ENSG00000268289.1	ENSG00000276884.1
10446	ENSG00000160075.12	ENSG00000268288.1	ENSG00000251890.1
10447	ENSG00000160087.20	ENSG00000268287.1	ENSG00000106536.19
10448	ENSG00000160094.15	ENSG00000268282.1	ENSG00000282122.1
10449	ENSG00000160097.18	ENSG00000268279.4	ENSG00000241219.1
10450	ENSG00000160111.13	ENSG00000268278.1	ENSG00000111732.11
10451	ENSG00000160113.5	ENSG00000268272.1	ENSG00000228820.2
10452	ENSG00000160117.15	ENSG00000268267.1	ENSG00000253908.1
10453	ENSG00000160124.9	ENSG00000268266.1	ENSG00000237062.1
10454	ENSG00000160131.13	ENSG00000268262.1	ENSG00000196431.4
10455	ENSG00000160145.15	ENSG00000268257.2	ENSG00000243509.6
10456	ENSG00000160161.9	ENSG00000268243.1	ENSG00000280832.1
10457	ENSG00000160172.10	ENSG00000268240.1	ENSG00000180801.14
10458	ENSG00000160179.18	ENSG00000268238.1	ENSG00000105501.12
10459	ENSG00000160180.15	ENSG00000268235.7	ENSG00000265798.6
10460	ENSG00000160181.9	ENSG00000268234.1	ENSG00000198039.11
10461	ENSG00000160182.3	ENSG00000268231.1	ENSG00000259438.1
10462	ENSG00000160183.15	ENSG00000268230.5	ENSG00000275488.1
10463	ENSG00000160185.15	ENSG00000268225.2	ENSG00000130147.16
10464	ENSG00000160188.10	ENSG00000268223.5	ENSG00000233846.2
10465	ENSG00000160190.14	ENSG00000268222.1	ENSG00000265073.1
10466	ENSG00000160191.18	ENSG00000268221.5	ENSG00000049130.15
10467	ENSG00000160193.11	ENSG00000268218.1	ENSG00000166750.10
10468	ENSG00000160194.18	ENSG00000268209.1	ENSG00000213028.3
10469	ENSG00000160199.14	ENSG00000268205.1	ENSG00000225885.7
10470	ENSG00000160200.17	ENSG00000268204.1	ENSG00000242473.1
10471	ENSG00000160201.11	ENSG00000268201.1	ENSG00000226088.1
10472	ENSG00000160202.7	ENSG00000268199.2	ENSG00000076716.9
10473	ENSG00000160207.9	ENSG00000268193.5	ENSG00000286078.1
10474	ENSG00000160208.13	ENSG00000268191.1	ENSG00000118432.12
10475	ENSG00000160209.19	ENSG00000268189.2	ENSG00000264668.2
10476	ENSG00000160211.17	ENSG00000268186.1	ENSG00000224302.1
10477	ENSG00000160213.7	ENSG00000268184.1	ENSG00000232794.1
10478	ENSG00000160214.12	ENSG00000268182.6	ENSG00000229021.2
10479	ENSG00000160216.19	ENSG00000268181.3	ENSG00000162706.13
10480	ENSG00000160218.13	ENSG00000268174.1	ENSG00000260511.1
10481	ENSG00000160219.12	ENSG00000268173.3	ENSG00000244236.3
10482	ENSG00000160221.18	ENSG00000268170.2	ENSG00000196550.10
10483	ENSG00000160223.17	ENSG00000268163.1	ENSG00000223566.1
10484	ENSG00000160224.17	ENSG00000268157.1	ENSG00000214988.4
10485	ENSG00000160226.16	ENSG00000268154.2	ENSG00000275074.2
10486	ENSG00000160229.12	ENSG00000268153.5	ENSG00000100104.13
10487	ENSG00000160233.8	ENSG00000268149.1	ENSG00000279717.1
10488	ENSG00000160255.18	ENSG00000268148.1	ENSG00000232220.2
10489	ENSG00000160256.13	ENSG00000268144.1	ENSG00000242747.1
10490	ENSG00000160271.16	ENSG00000268140.1	ENSG00000258602.1
10491	ENSG00000160282.14	ENSG00000268133.1	ENSG00000075399.14

10492	ENSG00000160284.15	ENSG00000268129.1	ENSG00000286191.1
10493	ENSG00000160285.15	ENSG00000268120.1	ENSG00000279609.1
10494	ENSG00000160293.17	ENSG00000268119.5	ENSG00000276302.1
10495	ENSG00000160294.11	ENSG00000268117.1	ENSG00000283201.1
10496	ENSG00000160298.17	ENSG00000268116.1	ENSG00000280153.1
10497	ENSG00000160299.17	ENSG00000268112.1	ENSG00000258806.2
10498	ENSG00000160305.18	ENSG00000268108.1	ENSG00000265545.1
10499	ENSG00000160307.10	ENSG00000268107.6	ENSG00000100987.14
10500	ENSG00000160310.18	ENSG00000268105.1	ENSG00000223660.1
10501	ENSG00000160318.6	ENSG00000268104.3	ENSG00000253556.1
10502	ENSG00000160321.15	ENSG00000268101.2	ENSG00000137648.17
10503	ENSG00000160323.18	ENSG00000268100.1	ENSG00000230534.6
10504	ENSG00000160325.14	ENSG00000268095.1	ENSG00000242732.4
10505	ENSG00000160326.14	ENSG00000268093.1	ENSG00000229656.6
10506	ENSG00000160336.15	ENSG00000268089.3	ENSG00000185523.6
10507	ENSG00000160339.16	ENSG00000268088.1	ENSG00000267466.1
10508	ENSG00000160345.13	ENSG00000268087.1	ENSG00000004939.15
10509	ENSG00000160349.9	ENSG00000268083.5	ENSG00000153707.17
10510	ENSG00000160352.15	ENSG00000268081.1	ENSG00000264047.2
10511	ENSG00000160360.13	ENSG00000268080.2	ENSG00000240974.1
10512	ENSG00000160392.13	ENSG00000268079.1	ENSG00000161921.15
10513	ENSG00000160396.8	ENSG00000268078.1	ENSG00000164199.18
10514	ENSG00000160401.15	ENSG00000268074.1	ENSG00000252712.1
10515	ENSG00000160404.18	ENSG00000268070.1	ENSG00000237719.1
10516	ENSG00000160408.14	ENSG00000268069.2	ENSG00000101670.12
10517	ENSG00000160410.15	ENSG00000268067.3	ENSG00000278198.1
10518	ENSG00000160439.16	ENSG00000268066.5	ENSG00000276281.1
10519	ENSG00000160445.11	ENSG00000268062.1	ENSG00000228601.1
10520	ENSG00000160446.19	ENSG00000268061.5	ENSG00000233844.1
10521	ENSG00000160447.7	ENSG00000268058.1	ENSG00000245080.7
10522	ENSG00000160460.16	ENSG00000268056.5	ENSG00000233459.1
10523	ENSG00000160469.16	ENSG00000268055.1	ENSG00000269303.1
10524	ENSG00000160471.13	ENSG00000268053.1	ENSG00000213015.9
10525	ENSG00000160472.5	ENSG00000268051.1	ENSG00000167264.18
10526	ENSG00000160505.15	ENSG00000268050.2	ENSG00000236671.8
10527	ENSG00000160539.6	ENSG00000268049.1	ENSG00000230951.1
10528	ENSG00000160551.11	ENSG00000268043.7	ENSG00000233870.1
10529	ENSG00000160563.14	ENSG00000268041.2	ENSG00000186334.9
10530	ENSG00000160570.14	ENSG00000268038.2	ENSG00000071794.16
10531	ENSG00000160584.16	ENSG00000268034.1	ENSG00000243049.3
10532	ENSG00000160588.10	ENSG00000268030.1	ENSG00000148773.14
10533	ENSG00000160593.18	ENSG00000268027.5	ENSG00000221874.4
10534	ENSG00000160602.14	ENSG00000268020.3	ENSG00000256294.8
10535	ENSG00000160606.11	ENSG00000268015.1	ENSG00000259045.1
10536	ENSG00000160613.12	ENSG00000268009.5	ENSG00000267114.1
10537	ENSG00000160633.13	ENSG00000268006.1	ENSG00000273407.1
10538	ENSG00000160654.10	ENSG00000268001.1	ENSG00000254851.1
10539	ENSG00000160678.11	ENSG00000267992.1	ENSG00000260622.1
10540	ENSG00000160679.13	ENSG00000267990.1	ENSG00000241175.3
10541	ENSG00000160683.4	ENSG00000267986.1	ENSG00000227009.1
10542	ENSG00000160685.13	ENSG00000267980.1	ENSG00000235688.2
10543	ENSG00000160688.19	ENSG00000267978.5	ENSG00000227243.3
10544	ENSG00000160691.18	ENSG00000267968.1	ENSG00000160882.11

10545	ENSG00000160695.14	ENSG00000267961.2	ENSG00000140463.14
10546	ENSG00000160703.16	ENSG00000267959.3	ENSG00000258092.1
10547	ENSG00000160710.16	ENSG00000267952.1	ENSG00000264019.1
10548	ENSG00000160712.13	ENSG00000267943.1	ENSG00000175093.5
10549	ENSG00000160714.10	ENSG00000267940.1	ENSG00000159387.7
10550	ENSG00000160716.5	ENSG00000267939.1	ENSG00000259712.1
10551	ENSG00000160741.17	ENSG00000267938.1	ENSG00000240934.2
10552	ENSG00000160746.12	ENSG00000267934.1	ENSG00000116918.14
10553	ENSG00000160752.14	ENSG00000267927.1	ENSG00000226676.1
10554	ENSG00000160753.16	ENSG00000267924.1	ENSG00000273874.1
10555	ENSG00000160766.14	ENSG00000267922.1	ENSG00000226345.1
10556	ENSG00000160767.21	ENSG00000267920.1	ENSG00000255760.1
10557	ENSG00000160781.17	ENSG00000267919.1	ENSG00000280119.1
10558	ENSG00000160783.19	ENSG00000267909.3	ENSG00000164010.15
10559	ENSG00000160785.13	ENSG00000267908.2	ENSG00000241420.3
10560	ENSG00000160789.20	ENSG00000267905.5	ENSG00000259840.1
10561	ENSG00000160791.13	ENSG00000267904.1	ENSG00000253269.1
10562	ENSG00000160796.17	ENSG00000267898.1	ENSG00000277913.1
10563	ENSG00000160799.11	ENSG00000267895.1	ENSG00000177675.8
10564	ENSG00000160801.14	ENSG00000267892.1	ENSG00000257094.1
10565	ENSG00000160803.8	ENSG00000267890.1	ENSG00000214237.10
10566	ENSG00000160808.9	ENSG00000267886.1	ENSG00000228065.10
10567	ENSG00000160813.7	ENSG00000267882.2	ENSG00000124593.16
10568	ENSG00000160818.16	ENSG00000267881.1	ENSG00000221933.3
10569	ENSG00000160838.14	ENSG00000267879.1	ENSG00000120068.6
10570	ENSG00000160856.21	ENSG00000267872.1	ENSG00000185634.12
10571	ENSG00000160862.13	ENSG00000267871.5	ENSG00000122180.5
10572	ENSG00000160867.15	ENSG00000267858.5	ENSG00000271973.1
10573	ENSG00000160868.15	ENSG00000267857.2	ENSG00000273582.1
10574	ENSG00000160870.14	ENSG00000267855.5	ENSG00000228021.7
10575	ENSG00000160877.6	ENSG00000267852.1	ENSG00000271108.1
10576	ENSG00000160882.11	ENSG00000267838.2	ENSG00000136881.11
10577	ENSG00000160883.11	ENSG00000267834.1	ENSG00000223685.5
10578	ENSG00000160886.13	ENSG00000267828.1	ENSG00000205571.13
10579	ENSG00000160888.7	ENSG00000267827.5	ENSG00000260844.2
10580	ENSG00000160908.15	ENSG00000267815.1	ENSG00000250214.1
10581	ENSG00000160917.15	ENSG00000267811.1	ENSG00000176485.11
10582	ENSG00000160932.11	ENSG00000267809.1	ENSG00000257327.1
10583	ENSG00000160948.14	ENSG00000267808.1	ENSG00000255340.1
10584	ENSG00000160949.17	ENSG00000267801.1	ENSG00000273291.5
10585	ENSG00000160951.4	ENSG00000267800.1	ENSG00000133216.16
10586	ENSG00000160953.16	ENSG00000267799.1	ENSG00000128708.13
10587	ENSG00000160957.13	ENSG00000267797.1	ENSG00000260262.1
10588	ENSG00000160959.8	ENSG00000267796.8	ENSG00000254974.1
10589	ENSG00000160961.12	ENSG00000267795.5	ENSG00000226498.2
10590	ENSG00000160963.14	ENSG00000267794.1	ENSG00000161103.11
10591	ENSG00000160972.9	ENSG00000267793.1	ENSG00000188234.13
10592	ENSG00000160973.7	ENSG00000267792.1	ENSG00000270808.1
10593	ENSG00000160991.16	ENSG00000267791.1	ENSG00000254289.2
10594	ENSG00000160993.4	ENSG00000267790.1	ENSG00000164120.14
10595	ENSG00000160994.4	ENSG00000267789.1	ENSG00000226723.2
10596	ENSG00000160999.10	ENSG00000267788.1	ENSG00000279191.1
10597	ENSG00000161010.15	ENSG00000267787.6	ENSG00000154721.15

10598	ENSG00000161011.20	ENSG00000267786.1	ENSG00000280000.1
10599	ENSG00000161013.17	ENSG00000267784.1	ENSG00000273162.1
10600	ENSG00000161016.17	ENSG00000267783.1	ENSG00000255029.1
10601	ENSG00000161021.13	ENSG00000267782.1	ENSG00000222108.1
10602	ENSG00000161031.13	ENSG00000267781.1	ENSG00000215002.2
10603	ENSG00000161036.13	ENSG00000267780.2	ENSG00000251598.2
10604	ENSG00000161040.16	ENSG00000267779.6	ENSG00000223703.1
10605	ENSG00000161048.11	ENSG00000267778.1	ENSG00000198077.10
10606	ENSG00000161055.4	ENSG00000267777.1	ENSG00000272650.1
10607	ENSG00000161057.12	ENSG00000267776.1	ENSG00000183530.14
10608	ENSG00000161082.13	ENSG00000267775.1	ENSG00000204971.3
10609	ENSG00000161091.13	ENSG00000267774.2	ENSG00000133069.17
10610	ENSG00000161103.11	ENSG00000267773.1	ENSG00000237594.2
10611	ENSG00000161132.6	ENSG00000267772.1	ENSG00000225127.2
10612	ENSG00000161133.16	ENSG00000267771.1	ENSG00000277769.1
10613	ENSG00000161149.12	ENSG00000267770.1	ENSG00000213062.4
10614	ENSG00000161179.13	ENSG00000267769.1	ENSG00000006704.10
10615	ENSG00000161180.11	ENSG00000267768.1	ENSG00000233144.1
10616	ENSG00000161202.19	ENSG00000267766.1	ENSG00000155508.13
10617	ENSG00000161203.13	ENSG00000267765.1	ENSG00000257702.3
10618	ENSG00000161204.11	ENSG00000267764.1	ENSG00000277020.4
10619	ENSG00000161217.12	ENSG00000267762.1	ENSG00000186416.14
10620	ENSG00000161243.9	ENSG00000267761.3	ENSG00000268605.1
10621	ENSG00000161249.21	ENSG00000267760.1	ENSG00000227433.1
10622	ENSG00000161265.15	ENSG00000267758.1	ENSG00000215278.4
10623	ENSG00000161267.12	ENSG00000267757.4	ENSG00000072571.20
10624	ENSG00000161270.19	ENSG00000267756.1	ENSG00000259231.2
10625	ENSG00000161277.10	ENSG00000267755.1	ENSG00000249006.1
10626	ENSG00000161281.11	ENSG00000267752.1	ENSG00000257847.1
10627	ENSG00000161298.18	ENSG00000267750.5	ENSG00000261468.1
10628	ENSG00000161328.11	ENSG00000267749.1	ENSG00000213261.3
10629	ENSG00000161381.14	ENSG00000267748.4	ENSG00000128849.10
10630	ENSG00000161395.14	ENSG00000267746.1	ENSG00000207808.1
10631	ENSG00000161405.17	ENSG00000267745.1	ENSG00000185899.1
10632	ENSG00000161509.14	ENSG00000267744.1	ENSG00000234895.1
10633	ENSG00000161513.12	ENSG00000267743.5	ENSG00000275714.1
10634	ENSG00000161526.15	ENSG00000267742.1	ENSG00000267248.2
10635	ENSG00000161533.12	ENSG00000267741.1	ENSG00000259710.1
10636	ENSG00000161542.16	ENSG00000267740.5	ENSG00000220721.2
10637	ENSG00000161544.10	ENSG00000267737.1	ENSG00000226918.1
10638	ENSG00000161547.16	ENSG00000267736.2	ENSG00000273253.2
10639	ENSG00000161551.14	ENSG00000267735.1	ENSG00000239317.1
10640	ENSG00000161558.11	ENSG00000267734.1	ENSG00000231254.2
10641	ENSG00000161594.6	ENSG00000267732.1	ENSG00000223558.1
10642	ENSG00000161609.10	ENSG00000267731.1	ENSG00000280186.1
10643	ENSG00000161610.1	ENSG00000267729.1	ENSG00000073754.6
10644	ENSG00000161618.10	ENSG00000267727.1	ENSG00000274475.1
10645	ENSG00000161634.11	ENSG00000267726.1	ENSG00000178919.8
10646	ENSG00000161638.11	ENSG00000267725.1	ENSG00000277859.1
10647	ENSG00000161640.15	ENSG00000267724.1	ENSG00000201955.1
10648	ENSG00000161642.17	ENSG00000267723.1	ENSG00000178723.7
10649	ENSG00000161643.12	ENSG00000267722.1	ENSG00000229326.3
10650	ENSG00000161647.18	ENSG00000267717.1	ENSG00000271916.1

10651	ENSG00000161649.13	ENSG00000267716.1	ENSG00000282757.3
10652	ENSG00000161652.12	ENSG00000267714.1	ENSG00000101076.16
10653	ENSG00000161653.10	ENSG00000267713.1	ENSG00000229195.1
10654	ENSG00000161654.9	ENSG00000267712.5	ENSG00000229531.5
10655	ENSG00000161664.6	ENSG00000267711.1	ENSG00000251188.1
10656	ENSG00000161671.16	ENSG00000267710.9	ENSG00000225542.1
10657	ENSG00000161677.11	ENSG00000267709.1	ENSG00000182450.13
10658	ENSG00000161681.15	ENSG00000267708.1	ENSG00000270025.2
10659	ENSG00000161682.14	ENSG00000267707.2	ENSG00000157833.13
10660	ENSG00000161692.18	ENSG00000267706.3	ENSG00000259304.1
10661	ENSG00000161714.12	ENSG00000267705.1	ENSG00000261071.1
10662	ENSG00000161791.14	ENSG00000267704.1	ENSG00000151650.8
10663	ENSG00000161798.7	ENSG00000267703.1	ENSG00000253223.1
10664	ENSG00000161800.13	ENSG00000267702.1	ENSG00000159167.12
10665	ENSG00000161807.3	ENSG00000267699.2	ENSG00000011347.9
10666	ENSG00000161813.22	ENSG00000267698.1	ENSG00000252315.1
10667	ENSG00000161835.11	ENSG00000267697.1	ENSG00000205361.8
10668	ENSG00000161847.14	ENSG00000267696.6	ENSG00000278852.1
10669	ENSG00000161849.3	ENSG00000267695.1	ENSG00000249098.1
10670	ENSG00000161850.2	ENSG00000267694.1	ENSG00000278576.1
10671	ENSG00000161860.7	ENSG00000267693.1	ENSG00000099194.6
10672	ENSG00000161888.11	ENSG00000267692.1	ENSG00000254203.1
10673	ENSG00000161896.12	ENSG00000267691.1	ENSG00000264490.3
10674	ENSG00000161904.12	ENSG00000267690.1	ENSG00000165555.9
10675	ENSG00000161905.12	ENSG00000267689.1	ENSG00000103005.11
10676	ENSG00000161911.11	ENSG00000267688.1	ENSG00000162753.13
10677	ENSG00000161912.18	ENSG00000267687.1	ENSG00000120802.13
10678	ENSG00000161914.10	ENSG00000267686.1	ENSG00000213663.4
10679	ENSG00000161920.10	ENSG00000267685.2	ENSG00000232656.7
10680	ENSG00000161921.15	ENSG00000267683.1	ENSG00000229739.2
10681	ENSG00000161929.15	ENSG00000267682.1	ENSG00000173662.21
10682	ENSG00000161939.19	ENSG00000267681.1	ENSG00000213400.3
10683	ENSG00000161944.16	ENSG00000267680.5	ENSG00000171241.9
10684	ENSG00000161955.16	ENSG00000267679.1	ENSG00000160284.15
10685	ENSG00000161956.13	ENSG00000267678.1	ENSG00000271666.1
10686	ENSG00000161958.11	ENSG00000267677.1	ENSG00000267201.1
10687	ENSG00000161960.15	ENSG00000267676.1	ENSG00000264666.1
10688	ENSG00000161970.14	ENSG00000267675.1	ENSG00000111981.5
10689	ENSG00000161973.11	ENSG00000267674.1	ENSG00000177300.6
10690	ENSG00000161980.5	ENSG00000267673.6	ENSG00000238283.3
10691	ENSG00000161981.10	ENSG00000267672.1	ENSG00000230177.1
10692	ENSG00000161992.6	ENSG00000267670.1	ENSG00000162598.13
10693	ENSG00000161996.19	ENSG00000267669.1	ENSG00000185467.7
10694	ENSG00000161999.12	ENSG00000267667.1	ENSG00000266153.1
10695	ENSG00000162004.17	ENSG00000267665.1	ENSG00000281016.1
10696	ENSG00000162006.9	ENSG00000267664.1	ENSG00000279940.1
10697	ENSG00000162009.8	ENSG00000267663.1	ENSG00000217495.2
10698	ENSG00000162032.16	ENSG00000267662.1	ENSG00000156755.10
10699	ENSG00000162039.15	ENSG00000267661.1	ENSG00000269662.1
10700	ENSG00000162040.6	ENSG00000267659.5	ENSG00000182352.8
10701	ENSG00000162062.15	ENSG00000267658.1	ENSG00000154646.9
10702	ENSG00000162063.13	ENSG00000267656.1	ENSG00000183255.12
10703	ENSG00000162065.13	ENSG00000267655.1	ENSG00000255723.1

10704	ENSG00000162066.15	ENSG00000267653.1	ENSG00000223884.6
10705	ENSG00000162068.1	ENSG00000267652.1	ENSG00000159712.10
10706	ENSG00000162069.15	ENSG00000267651.1	ENSG00000225947.1
10707	ENSG00000162073.13	ENSG00000267649.1	ENSG00000102466.15
10708	ENSG00000162076.13	ENSG00000267648.1	ENSG00000217195.1
10709	ENSG00000162078.11	ENSG00000267647.1	ENSG00000280068.1
10710	ENSG00000162086.14	ENSG00000267646.1	ENSG00000278463.1
10711	ENSG00000162104.10	ENSG00000267645.5	ENSG00000235310.1
10712	ENSG00000162105.18	ENSG00000267644.1	ENSG00000258632.2
10713	ENSG00000162129.13	ENSG00000267643.1	ENSG00000215023.2
10714	ENSG00000162139.10	ENSG00000267642.1	ENSG00000083720.13
10715	ENSG00000162144.9	ENSG00000267641.1	ENSG00000245025.2
10716	ENSG00000162148.11	ENSG00000267638.1	ENSG00000187537.13
10717	ENSG00000162174.12	ENSG00000267637.1	ENSG00000064042.18
10718	ENSG00000162188.6	ENSG00000267636.1	ENSG00000233430.3
10719	ENSG00000162191.13	ENSG00000267634.1	ENSG00000229521.2
10720	ENSG00000162194.12	ENSG00000267633.1	ENSG00000112367.10
10721	ENSG00000162222.14	ENSG00000267632.1	ENSG00000260571.1
10722	ENSG00000162227.8	ENSG00000267631.4	ENSG00000254233.1
10723	ENSG00000162231.14	ENSG00000267630.1	ENSG00000151715.7
10724	ENSG00000162236.11	ENSG00000267629.3	ENSG00000279691.1
10725	ENSG00000162241.12	ENSG00000267628.1	ENSG00000236880.1
10726	ENSG00000162244.11	ENSG00000267627.5	ENSG00000185186.9
10727	ENSG00000162267.12	ENSG00000267626.1	ENSG00000269433.3
10728	ENSG00000162298.18	ENSG00000267625.1	ENSG00000259377.1
10729	ENSG00000162300.13	ENSG00000267624.1	ENSG00000015592.16
10730	ENSG00000162302.13	ENSG00000267623.3	ENSG00000227499.1
10731	ENSG00000162337.12	ENSG00000267620.1	ENSG00000230876.7
10732	ENSG00000162341.17	ENSG00000267618.5	ENSG00000165449.11
10733	ENSG00000162344.4	ENSG00000267617.1	ENSG00000276505.1
10734	ENSG00000162365.12	ENSG00000267615.1	ENSG00000204790.9
10735	ENSG00000162366.8	ENSG00000267614.1	ENSG00000158321.16
10736	ENSG00000162367.11	ENSG00000267612.1	ENSG00000248514.1
10737	ENSG00000162368.13	ENSG00000267611.1	ENSG00000116667.14
10738	ENSG00000162373.13	ENSG00000267610.2	ENSG00000219951.4
10739	ENSG00000162374.17	ENSG00000267609.2	ENSG00000260077.1
10740	ENSG00000162377.6	ENSG00000267607.1	ENSG00000161939.19
10741	ENSG00000162378.13	ENSG00000267606.1	ENSG00000286012.1
10742	ENSG00000162383.12	ENSG00000267605.6	ENSG00000131473.17
10743	ENSG00000162384.14	ENSG00000267604.1	ENSG00000258860.1
10744	ENSG00000162385.11	ENSG00000267603.1	ENSG00000183476.12
10745	ENSG00000162390.17	ENSG00000267601.1	ENSG00000271321.1
10746	ENSG00000162391.12	ENSG00000267599.1	ENSG00000274010.1
10747	ENSG00000162396.6	ENSG00000267597.1	ENSG00000136099.13
10748	ENSG00000162398.11	ENSG00000267595.1	ENSG00000254006.5
10749	ENSG00000162399.8	ENSG00000267594.5	ENSG00000286039.1
10750	ENSG00000162402.14	ENSG00000267593.1	ENSG00000267139.1
10751	ENSG00000162407.9	ENSG00000267592.1	ENSG00000285696.1
10752	ENSG00000162408.11	ENSG00000267591.1	ENSG00000284191.1
10753	ENSG00000162409.11	ENSG00000267590.1	ENSG00000136238.18
10754	ENSG00000162413.16	ENSG00000267589.1	ENSG00000215544.6
10755	ENSG00000162415.7	ENSG00000267587.1	ENSG00000237088.1
10756	ENSG00000162419.12	ENSG00000267586.6	ENSG00000132002.8

10757	ENSG00000162426.14	ENSG00000267585.1	ENSG00000276150.1
10758	ENSG00000162430.17	ENSG00000267583.5	ENSG00000100012.11
10759	ENSG00000162433.15	ENSG00000267582.1	ENSG00000223427.1
10760	ENSG00000162434.12	ENSG00000267581.1	ENSG00000274424.1
10761	ENSG00000162437.14	ENSG00000267580.1	ENSG00000254924.2
10762	ENSG00000162438.11	ENSG00000267579.1	ENSG00000163395.16
10763	ENSG00000162441.12	ENSG00000267577.1	ENSG00000224806.2
10764	ENSG00000162444.12	ENSG00000267576.1	ENSG00000240096.1
10765	ENSG00000162456.9	ENSG00000267575.6	ENSG00000093009.9
10766	ENSG00000162458.13	ENSG00000267574.1	ENSG00000267014.5
10767	ENSG00000162460.7	ENSG00000267573.1	ENSG00000214943.4
10768	ENSG00000162461.8	ENSG00000267571.1	ENSG00000237740.1
10769	ENSG00000162482.5	ENSG00000267570.1	ENSG00000196131.6
10770	ENSG00000162490.7	ENSG00000267568.6	ENSG00000226642.1
10771	ENSG00000162493.16	ENSG00000267567.1	ENSG00000273340.1
10772	ENSG00000162494.6	ENSG00000267565.1	ENSG00000273644.1
10773	ENSG00000162496.9	ENSG00000267564.1	ENSG00000219814.2
10774	ENSG00000162510.6	ENSG00000267563.1	ENSG00000242375.1
10775	ENSG00000162511.8	ENSG00000267561.2	ENSG00000228121.1
10776	ENSG00000162512.16	ENSG00000267560.1	ENSG00000140848.17
10777	ENSG00000162517.13	ENSG00000267559.5	ENSG00000150672.17
10778	ENSG00000162520.14	ENSG00000267558.2	ENSG00000163564.14
10779	ENSG00000162521.19	ENSG00000267557.1	ENSG00000250437.1
10780	ENSG00000162522.11	ENSG00000267555.1	ENSG00000136457.10
10781	ENSG00000162526.7	ENSG00000267554.1	ENSG00000262815.1
10782	ENSG00000162542.13	ENSG00000267552.6	ENSG00000248408.1
10783	ENSG00000162543.6	ENSG00000267550.1	ENSG00000231787.4
10784	ENSG00000162545.6	ENSG00000267549.6	ENSG00000055130.16
10785	ENSG00000162551.14	ENSG00000267547.1	ENSG00000236951.5
10786	ENSG00000162552.15	ENSG00000267546.2	ENSG00000135312.6
10787	ENSG00000162571.13	ENSG00000267545.1	ENSG00000285280.1
10788	ENSG00000162572.20	ENSG00000267544.1	ENSG00000278674.1
10789	ENSG00000162576.16	ENSG00000267543.1	ENSG00000107566.14
10790	ENSG00000162585.17	ENSG00000267541.1	ENSG00000250471.2
10791	ENSG00000162591.16	ENSG00000267537.1	ENSG00000285825.1
10792	ENSG00000162592.10	ENSG00000267535.1	ENSG00000251476.2
10793	ENSG00000162595.6	ENSG00000267534.4	ENSG00000284631.1
10794	ENSG00000162598.13	ENSG00000267533.1	ENSG00000249318.1
10795	ENSG00000162599.16	ENSG00000267532.5	ENSG00000261386.2
10796	ENSG00000162600.12	ENSG00000267530.2	ENSG00000171425.10
10797	ENSG00000162601.10	ENSG00000267529.3	ENSG00000213853.10
10798	ENSG00000162604.12	ENSG00000267526.1	ENSG00000104714.14
10799	ENSG00000162607.13	ENSG00000267524.1	ENSG00000237400.1
10800	ENSG00000162613.16	ENSG00000267523.1	ENSG00000270900.1
10801	ENSG00000162614.18	ENSG00000267522.1	ENSG00000164935.6
10802	ENSG00000162616.9	ENSG00000267521.1	ENSG00000278543.1
10803	ENSG00000162618.14	ENSG00000267520.2	ENSG00000228294.6
10804	ENSG00000162620.16	ENSG00000267518.1	ENSG00000213703.2
10805	ENSG00000162621.6	ENSG00000267517.1	ENSG00000141968.8
10806	ENSG00000162623.16	ENSG00000267515.1	ENSG00000260999.1
10807	ENSG00000162624.14	ENSG00000267513.3	ENSG00000278493.1
10808	ENSG00000162627.17	ENSG00000267512.1	ENSG00000197585.9
10809	ENSG00000162630.5	ENSG00000267511.1	ENSG00000254843.1

10810	ENSG00000162631.18	ENSG00000267510.1	ENSG00000205220.12
10811	ENSG00000162636.16	ENSG00000267509.5	ENSG00000255042.3
10812	ENSG00000162639.16	ENSG00000267508.5	ENSG00000228349.1
10813	ENSG00000162641.19	ENSG00000267507.1	ENSG00000116752.6
10814	ENSG00000162642.14	ENSG00000267506.5	ENSG00000125629.15
10815	ENSG00000162643.13	ENSG00000267505.1	ENSG00000146834.14
10816	ENSG00000162645.13	ENSG00000267504.1	ENSG00000206597.1
10817	ENSG00000162650.16	ENSG00000267503.1	ENSG00000164109.14
10818	ENSG00000162654.9	ENSG00000267501.1	ENSG00000232907.7
10819	ENSG00000162664.17	ENSG00000267500.1	ENSG00000234942.2
10820	ENSG00000162669.16	ENSG00000267498.1	ENSG00000285993.1
10821	ENSG00000162670.11	ENSG00000267497.1	ENSG00000257925.1
10822	ENSG00000162676.12	ENSG00000267496.4	ENSG00000276592.1
10823	ENSG00000162685.6	ENSG00000267493.3	ENSG00000240919.1
10824	ENSG00000162687.17	ENSG00000267492.1	ENSG00000243273.1
10825	ENSG00000162688.17	ENSG00000267491.1	ENSG00000279387.1
10826	ENSG00000162692.12	ENSG00000267490.1	ENSG00000235051.3
10827	ENSG00000162694.14	ENSG00000267489.4	ENSG00000235251.2
10828	ENSG00000162695.12	ENSG00000267487.1	ENSG00000148700.14
10829	ENSG00000162699.5	ENSG00000267486.1	ENSG00000276128.1
10830	ENSG00000162702.8	ENSG00000267484.1	ENSG00000273568.1
10831	ENSG00000162704.16	ENSG00000267482.1	ENSG00000135451.13
10832	ENSG00000162706.13	ENSG00000267481.1	ENSG00000259876.1
10833	ENSG00000162711.17	ENSG00000267480.1	ENSG00000092931.11
10834	ENSG00000162714.12	ENSG00000267478.1	ENSG00000186871.7
10835	ENSG00000162722.9	ENSG00000267477.1	ENSG00000100764.14
10836	ENSG00000162723.10	ENSG00000267476.1	ENSG00000202512.1
10837	ENSG00000162727.3	ENSG00000267474.1	ENSG00000092330.17
10838	ENSG00000162728.5	ENSG00000267472.1	ENSG00000214783.9
10839	ENSG00000162729.14	ENSG00000267471.1	ENSG00000154252.11
10840	ENSG00000162733.18	ENSG00000267470.5	ENSG00000180929.6
10841	ENSG00000162734.12	ENSG00000267469.1	ENSG00000132623.16
10842	ENSG00000162735.18	ENSG00000267467.4	ENSG00000160505.15
10843	ENSG00000162736.17	ENSG00000267466.1	ENSG00000254266.5
10844	ENSG00000162738.6	ENSG00000267465.6	ENSG00000141378.14
10845	ENSG00000162739.14	ENSG00000267463.1	ENSG00000173918.15
10846	ENSG00000162745.10	ENSG00000267462.1	ENSG00000254061.1
10847	ENSG00000162746.14	ENSG00000267461.1	ENSG00000237980.1
10848	ENSG00000162747.11	ENSG00000267459.1	ENSG00000146267.12
10849	ENSG00000162753.13	ENSG00000267458.1	ENSG00000253539.1
10850	ENSG00000162755.14	ENSG00000267457.1	ENSG00000167642.13
10851	ENSG00000162757.4	ENSG00000267456.2	ENSG00000256712.1
10852	ENSG00000162761.14	ENSG00000267455.1	ENSG00000234869.1
10853	ENSG00000162763.3	ENSG00000267454.5	ENSG00000280494.2
10854	ENSG00000162769.13	ENSG00000267452.2	ENSG00000257643.1
10855	ENSG00000162771.7	ENSG00000267450.1	ENSG00000259134.6
10856	ENSG00000162772.17	ENSG00000267449.1	ENSG00000255073.8
10857	ENSG00000162775.15	ENSG00000267448.5	ENSG00000183562.3
10858	ENSG00000162777.17	ENSG00000267446.1	ENSG00000151577.12
10859	ENSG00000162779.22	ENSG00000267444.1	ENSG00000150051.14
10860	ENSG00000162782.16	ENSG00000267443.2	ENSG00000222012.1
10861	ENSG00000162783.10	ENSG00000267441.1	ENSG00000184560.8
10862	ENSG00000162804.14	ENSG00000267440.1	ENSG00000236532.5

10863	ENSG00000162813.18	ENSG00000267439.1	ENSG00000274114.2
10864	ENSG00000162814.11	ENSG00000267437.1	ENSG00000125637.15
10865	ENSG00000162817.7	ENSG00000267436.1	ENSG00000223911.1
10866	ENSG00000162819.12	ENSG00000267433.1	ENSG00000122711.9
10867	ENSG00000162825.16	ENSG00000267432.5	ENSG00000257264.5
10868	ENSG00000162836.12	ENSG00000267430.1	ENSG00000069943.10
10869	ENSG00000162840.4	ENSG00000267429.1	ENSG00000244203.2
10870	ENSG00000162843.17	ENSG00000267428.1	ENSG00000254468.2
10871	ENSG00000162849.16	ENSG00000267426.5	ENSG00000214552.4
10872	ENSG00000162851.8	ENSG00000267425.1	ENSG00000122674.11
10873	ENSG00000162852.14	ENSG00000267424.1	ENSG00000206706.1
10874	ENSG00000162869.16	ENSG00000267422.1	ENSG00000249004.1
10875	ENSG00000162873.14	ENSG00000267421.6	ENSG00000253143.3
10876	ENSG00000162877.13	ENSG00000267420.1	ENSG00000175746.6
10877	ENSG00000162878.13	ENSG00000267419.2	ENSG00000236285.1
10878	ENSG00000162881.6	ENSG00000267418.1	ENSG00000167977.9
10879	ENSG00000162882.15	ENSG00000267417.1	ENSG00000274611.3
10880	ENSG00000162885.13	ENSG00000267416.1	ENSG00000250337.6
10881	ENSG00000162888.4	ENSG00000267415.1	ENSG00000180549.7
10882	ENSG00000162889.10	ENSG00000267414.1	ENSG00000229444.1
10883	ENSG00000162891.10	ENSG00000267413.1	ENSG00000264767.2
10884	ENSG00000162892.15	ENSG00000267412.1	ENSG00000188015.10
10885	ENSG00000162894.12	ENSG00000267409.1	ENSG00000140015.20
10886	ENSG00000162896.6	ENSG00000267408.1	ENSG00000123360.12
10887	ENSG00000162897.15	ENSG00000267407.1	ENSG00000100031.19
10888	ENSG00000162909.18	ENSG00000267406.1	ENSG00000173281.5
10889	ENSG00000162910.19	ENSG00000267405.1	ENSG00000257231.1
10890	ENSG00000162913.9	ENSG00000267404.1	ENSG00000251234.1
10891	ENSG00000162923.16	ENSG00000267402.1	ENSG00000160298.17
10892	ENSG00000162924.14	ENSG00000267401.1	ENSG00000254429.1
10893	ENSG00000162927.14	ENSG00000267400.1	ENSG00000140274.13
10894	ENSG00000162928.9	ENSG00000267399.1	ENSG00000225190.11
10895	ENSG00000162929.13	ENSG00000267398.1	ENSG00000274159.1
10896	ENSG00000162931.12	ENSG00000267397.1	ENSG00000256783.1
10897	ENSG00000162944.10	ENSG00000267396.1	ENSG00000231503.4
10898	ENSG00000162946.22	ENSG00000267395.5	ENSG00000279135.1
10899	ENSG00000162947.4	ENSG00000267394.1	ENSG00000280502.1
10900	ENSG00000162949.16	ENSG00000267393.1	ENSG00000169208.2
10901	ENSG00000162951.11	ENSG00000267392.3	ENSG00000159788.19
10902	ENSG00000162959.13	ENSG00000267391.4	ENSG00000267699.2
10903	ENSG00000162961.14	ENSG00000267389.2	ENSG00000206838.1
10904	ENSG00000162971.11	ENSG00000267388.1	ENSG00000204913.6
10905	ENSG00000162972.10	ENSG00000267387.1	ENSG00000266265.3
10906	ENSG00000162975.5	ENSG00000267385.1	ENSG00000229623.1
10907	ENSG00000162976.13	ENSG00000267384.3	ENSG00000256681.1
10908	ENSG00000162980.17	ENSG00000267383.6	ENSG00000279813.1
10909	ENSG00000162981.14	ENSG00000267382.1	ENSG00000234117.2
10910	ENSG00000162989.5	ENSG00000267379.1	ENSG00000239218.2
10911	ENSG00000162992.3	ENSG00000267378.1	ENSG00000272904.1
10912	ENSG00000162994.16	ENSG00000267376.1	ENSG00000263982.1
10913	ENSG00000162997.15	ENSG00000267375.1	ENSG00000124102.5
10914	ENSG00000162998.5	ENSG00000267374.1	ENSG00000223518.5
10915	ENSG00000162999.12	ENSG00000267373.1	ENSG00000173706.14

10916	ENSG00000163001.11	ENSG00000267372.2	ENSG00000268400.5
10917	ENSG00000163002.13	ENSG00000267371.1	ENSG00000144935.15
10918	ENSG00000163006.12	ENSG00000267370.1	ENSG00000262456.1
10919	ENSG00000163012.3	ENSG00000267369.1	ENSG00000268957.1
10920	ENSG00000163013.11	ENSG00000267368.1	ENSG00000254829.1
10921	ENSG00000163016.10	ENSG00000267366.1	ENSG00000272969.1
10922	ENSG00000163017.13	ENSG00000267365.1	ENSG00000215895.4
10923	ENSG00000163026.12	ENSG00000267364.1	ENSG00000140525.17
10924	ENSG00000163029.16	ENSG00000267363.1	ENSG00000270207.2
10925	ENSG00000163032.11	ENSG00000267361.1	ENSG00000282935.1
10926	ENSG00000163040.14	ENSG00000267360.6	ENSG00000275516.1
10927	ENSG00000163041.10	ENSG00000267356.1	ENSG00000244232.3
10928	ENSG00000163046.15	ENSG00000267355.2	ENSG00000226734.1
10929	ENSG00000163050.17	ENSG00000267354.1	ENSG00000250424.4
10930	ENSG00000163053.11	ENSG00000267353.1	ENSG00000248405.10
10931	ENSG00000163060.8	ENSG00000267352.1	ENSG00000146166.16
10932	ENSG00000163064.6	ENSG00000267349.1	ENSG00000115041.13
10933	ENSG00000163069.12	ENSG00000267348.2	ENSG00000263065.1
10934	ENSG00000163071.11	ENSG00000267346.1	ENSG00000255808.1
10935	ENSG00000163072.15	ENSG00000267345.1	ENSG00000280179.1
10936	ENSG00000163075.13	ENSG00000267343.2	ENSG00000139287.13
10937	ENSG00000163081.3	ENSG00000267342.1	ENSG00000286220.1
10938	ENSG00000163082.10	ENSG00000267341.1	ENSG00000131876.17
10939	ENSG00000163083.6	ENSG00000267340.1	ENSG00000280087.1
10940	ENSG00000163092.19	ENSG00000267339.6	ENSG00000107745.19
10941	ENSG00000163093.12	ENSG00000267338.1	ENSG00000199837.1
10942	ENSG00000163098.5	ENSG00000267337.1	ENSG00000105483.18
10943	ENSG00000163104.17	ENSG00000267335.2	ENSG00000261371.6
10944	ENSG00000163106.10	ENSG00000267334.1	ENSG00000276547.1
10945	ENSG00000163110.15	ENSG00000267333.1	ENSG00000267042.1
10946	ENSG00000163114.5	ENSG00000267332.1	ENSG00000276509.1
10947	ENSG00000163116.10	ENSG00000267328.1	ENSG00000278702.1
10948	ENSG00000163121.10	ENSG00000267327.1	ENSG00000233589.1
10949	ENSG00000163125.15	ENSG00000267325.1	ENSG00000239521.8
10950	ENSG00000163126.15	ENSG00000267324.1	ENSG00000270863.1
10951	ENSG00000163131.11	ENSG00000267323.1	ENSG00000264196.1
10952	ENSG00000163132.7	ENSG00000267322.2	ENSG00000259399.1
10953	ENSG00000163138.19	ENSG00000267321.2	ENSG00000248268.1
10954	ENSG00000163141.19	ENSG00000267320.5	ENSG00000221116.1
10955	ENSG00000163145.12	ENSG00000267319.1	ENSG00000271182.1
10956	ENSG00000163154.6	ENSG00000267318.1	ENSG00000251296.1
10957	ENSG00000163155.12	ENSG00000267317.2	ENSG00000271370.1
10958	ENSG00000163156.11	ENSG00000267316.5	ENSG00000157985.19
10959	ENSG00000163157.15	ENSG00000267315.1	ENSG00000254715.3
10960	ENSG00000163159.12	ENSG00000267314.1	ENSG00000245694.9
10961	ENSG00000163161.13	ENSG00000267313.7	ENSG00000267257.1
10962	ENSG00000163162.8	ENSG00000267312.1	ENSG00000249240.2
10963	ENSG00000163166.15	ENSG00000267311.1	ENSG00000264829.1
10964	ENSG00000163170.12	ENSG00000267310.2	ENSG00000224505.2
10965	ENSG00000163171.7	ENSG00000267309.1	ENSG00000240654.6
10966	ENSG00000163191.6	ENSG00000267308.1	ENSG00000254332.1
10967	ENSG00000163202.5	ENSG00000267305.2	ENSG00000230629.2
10968	ENSG00000163206.6	ENSG00000267304.1	ENSG00000267655.1

10969	ENSG00000163207.7	ENSG00000267303.1	ENSG00000154133.14
10970	ENSG00000163209.15	ENSG00000267302.5	ENSG00000225901.1
10971	ENSG00000163214.21	ENSG00000267301.1	ENSG00000220157.4
10972	ENSG00000163216.7	ENSG00000267299.1	ENSG00000254088.1
10973	ENSG00000163217.2	ENSG00000267298.1	ENSG00000217289.3
10974	ENSG00000163218.15	ENSG00000267296.2	ENSG00000200587.1
10975	ENSG00000163219.11	ENSG00000267295.1	ENSG00000162929.13
10976	ENSG00000163220.11	ENSG00000267293.1	ENSG00000258878.1
10977	ENSG00000163221.9	ENSG00000267292.1	ENSG00000268531.3
10978	ENSG00000163235.16	ENSG00000267291.1	ENSG00000233613.5
10979	ENSG00000163239.12	ENSG00000267289.1	ENSG00000178026.13
10980	ENSG00000163249.12	ENSG00000267288.2	ENSG00000260025.1
10981	ENSG00000163251.4	ENSG00000267287.1	ENSG00000255595.4
10982	ENSG00000163254.5	ENSG00000267286.1	ENSG00000151322.18
10983	ENSG00000163257.11	ENSG00000267285.1	ENSG00000242767.1
10984	ENSG00000163263.7	ENSG00000267284.1	ENSG00000165091.17
10985	ENSG00000163273.4	ENSG00000267283.1	ENSG00000285647.1
10986	ENSG00000163281.11	ENSG00000267282.1	ENSG00000128274.17
10987	ENSG00000163283.7	ENSG00000267281.2	ENSG00000111911.7
10988	ENSG00000163285.8	ENSG00000267280.5	ENSG00000188910.8
10989	ENSG00000163286.9	ENSG00000267279.1	ENSG00000244734.4
10990	ENSG00000163288.13	ENSG00000267278.5	ENSG00000282949.1
10991	ENSG00000163291.14	ENSG00000267277.1	ENSG00000258590.5
10992	ENSG00000163293.12	ENSG00000267274.1	ENSG00000231512.6
10993	ENSG00000163295.5	ENSG00000267273.1	ENSG00000197734.9
10994	ENSG00000163297.17	ENSG00000267272.5	ENSG00000254610.3
10995	ENSG00000163312.11	ENSG00000267271.1	ENSG00000235997.2
10996	ENSG00000163319.11	ENSG00000267270.5	ENSG00000165182.11
10997	ENSG00000163320.11	ENSG00000267269.1	ENSG00000260167.1
10998	ENSG00000163322.14	ENSG00000267265.5	ENSG00000248115.1
10999	ENSG00000163328.13	ENSG00000267264.1	ENSG00000184361.13
11000	ENSG00000163331.11	ENSG00000267263.1	ENSG00000213830.3
11001	ENSG00000163344.6	ENSG00000267262.1	ENSG00000233862.5
11002	ENSG00000163346.17	ENSG00000267261.5	ENSG00000278486.1
11003	ENSG00000163347.6	ENSG00000267260.1	ENSG00000213744.3
11004	ENSG00000163348.4	ENSG00000267259.1	ENSG00000285706.1
11005	ENSG00000163349.22	ENSG00000267258.1	ENSG00000187554.13
11006	ENSG00000163352.5	ENSG00000267257.1	ENSG00000243708.10
11007	ENSG00000163354.15	ENSG00000267255.1	ENSG00000181027.10
11008	ENSG00000163357.10	ENSG00000267254.6	ENSG00000247049.2
11009	ENSG00000163359.15	ENSG00000267253.1	ENSG00000274516.2
11010	ENSG00000163362.11	ENSG00000267252.5	ENSG00000168477.19
11011	ENSG00000163364.9	ENSG00000267251.2	ENSG00000122386.10
11012	ENSG00000163374.19	ENSG00000267250.1	ENSG00000136280.16
11013	ENSG00000163376.11	ENSG00000267249.1	ENSG00000164946.19
11014	ENSG00000163377.16	ENSG00000267248.2	ENSG00000227948.4
11015	ENSG00000163378.14	ENSG00000267247.1	ENSG00000232377.1
11016	ENSG00000163380.15	ENSG00000267246.1	ENSG00000262668.2
11017	ENSG00000163382.12	ENSG00000267245.1	ENSG00000165762.3
11018	ENSG00000163389.12	ENSG00000267244.5	ENSG00000188771.4
11019	ENSG00000163393.13	ENSG00000267243.6	ENSG00000278665.1
11020	ENSG00000163394.5	ENSG00000267241.2	ENSG00000276984.1
11021	ENSG00000163395.16	ENSG00000267240.1	ENSG00000215284.2

11022	ENSG00000163399.16	ENSG00000267239.1	ENSG00000249751.4
11023	ENSG00000163406.11	ENSG00000267238.1	ENSG00000277214.1
11024	ENSG00000163412.13	ENSG00000267235.2	ENSG00000255819.7
11025	ENSG00000163421.9	ENSG00000267234.1	ENSG00000272406.1
11026	ENSG00000163424.9	ENSG00000267233.1	ENSG00000263815.2
11027	ENSG00000163428.4	ENSG00000267232.1	ENSG00000236809.2
11028	ENSG00000163430.12	ENSG00000267231.1	ENSG00000226032.2
11029	ENSG00000163431.13	ENSG00000267228.7	ENSG00000039987.6
11030	ENSG00000163435.16	ENSG00000267227.1	ENSG00000258569.1
11031	ENSG00000163440.12	ENSG00000267225.1	ENSG00000125385.9
11032	ENSG00000163444.12	ENSG00000267224.1	ENSG00000284564.1
11033	ENSG00000163449.11	ENSG00000267223.1	ENSG00000236104.3
11034	ENSG00000163453.11	ENSG00000267222.1	ENSG00000100353.18
11035	ENSG00000163462.18	ENSG00000267221.2	ENSG00000265263.1
11036	ENSG00000163463.11	ENSG00000267220.1	ENSG00000255062.1
11037	ENSG00000163464.7	ENSG00000267219.1	ENSG00000175104.15
11038	ENSG00000163466.16	ENSG00000267218.2	ENSG00000263809.1
11039	ENSG00000163467.11	ENSG00000267216.1	ENSG00000122756.15
11040	ENSG00000163468.15	ENSG00000267215.1	ENSG00000278873.1
11041	ENSG00000163472.19	ENSG00000267214.1	ENSG00000278950.1
11042	ENSG00000163479.14	ENSG00000267213.4	ENSG00000281969.1
11043	ENSG00000163481.8	ENSG00000267212.1	ENSG00000267544.1
11044	ENSG00000163482.12	ENSG00000267211.1	ENSG00000265763.4
11045	ENSG00000163485.17	ENSG00000267209.5	ENSG00000134982.17
11046	ENSG00000163491.16	ENSG00000267207.1	ENSG00000227006.1
11047	ENSG00000163492.15	ENSG00000267206.6	ENSG00000075292.19
11048	ENSG00000163497.2	ENSG00000267205.1	ENSG00000254923.1
11049	ENSG00000163499.12	ENSG00000267204.1	ENSG00000233138.1
11050	ENSG00000163501.7	ENSG00000267203.1	ENSG00000138135.6
11051	ENSG00000163507.14	ENSG00000267202.5	ENSG00000285025.1
11052	ENSG00000163508.12	ENSG00000267201.1	ENSG00000224721.1
11053	ENSG00000163510.14	ENSG00000267200.1	ENSG00000175206.10
11054	ENSG00000163512.14	ENSG00000267199.1	ENSG00000279581.1
11055	ENSG00000163513.17	ENSG00000267198.1	ENSG00000166856.3
11056	ENSG00000163515.6	ENSG00000267197.1	ENSG00000257653.1
11057	ENSG00000163516.13	ENSG00000267196.1	ENSG00000258159.1
11058	ENSG00000163517.15	ENSG00000267195.1	ENSG00000108179.14
11059	ENSG00000163518.11	ENSG00000267193.5	ENSG00000232325.4
11060	ENSG00000163519.13	ENSG00000267192.1	ENSG00000129824.16
11061	ENSG00000163520.14	ENSG00000267191.1	ENSG00000200537.1
11062	ENSG00000163521.16	ENSG00000267190.1	ENSG00000253720.1
11063	ENSG00000163527.9	ENSG00000267189.1	ENSG00000227632.2
11064	ENSG00000163528.13	ENSG00000267188.1	ENSG00000242622.1
11065	ENSG00000163530.4	ENSG00000267187.1	ENSG00000233254.1
11066	ENSG00000163531.15	ENSG00000267185.1	ENSG00000260441.5
11067	ENSG00000163534.15	ENSG00000267184.1	ENSG00000270105.1
11068	ENSG00000163535.18	ENSG00000267182.1	ENSG00000232732.10
11069	ENSG00000163536.12	ENSG00000267180.1	ENSG00000232692.1
11070	ENSG00000163539.17	ENSG00000267179.1	ENSG00000212335.1
11071	ENSG00000163541.12	ENSG00000267178.3	ENSG00000110786.18
11072	ENSG00000163545.8	ENSG00000267177.1	ENSG00000256469.1
11073	ENSG00000163554.13	ENSG00000267175.5	ENSG00000226454.2
11074	ENSG00000163558.13	ENSG00000267174.5	ENSG00000285530.1

11075	ENSG00000163563.8	ENSG00000267173.1	ENSG00000238165.1
11076	ENSG00000163564.14	ENSG00000267172.1	ENSG00000232879.2
11077	ENSG00000163565.18	ENSG00000267170.1	ENSG00000274328.1
11078	ENSG00000163568.15	ENSG00000267169.1	ENSG00000227329.2
11079	ENSG00000163576.18	ENSG00000267168.1	ENSG00000163814.8
11080	ENSG00000163577.8	ENSG00000267166.5	ENSG00000235721.1
11081	ENSG00000163581.14	ENSG00000267165.1	ENSG00000222014.5
11082	ENSG00000163584.18	ENSG00000267162.1	ENSG00000229992.1
11083	ENSG00000163586.9	ENSG00000267160.1	ENSG00000236732.1
11084	ENSG00000163590.14	ENSG00000267159.2	ENSG00000117215.14
11085	ENSG00000163596.16	ENSG00000267157.1	ENSG00000225725.4
11086	ENSG00000163597.14	ENSG00000267156.1	ENSG00000132437.18
11087	ENSG00000163599.16	ENSG00000267154.1	ENSG00000254403.1
11088	ENSG00000163600.12	ENSG00000267153.1	ENSG00000189120.5
11089	ENSG00000163602.10	ENSG00000267152.1	ENSG00000272701.3
11090	ENSG00000163605.14	ENSG00000267151.5	ENSG00000264070.1
11091	ENSG00000163606.11	ENSG00000267150.1	ENSG00000076344.16
11092	ENSG00000163607.15	ENSG00000267149.2	ENSG00000187775.16
11093	ENSG00000163608.15	ENSG00000267148.1	ENSG00000132746.14
11094	ENSG00000163611.11	ENSG00000267147.2	ENSG00000232097.1
11095	ENSG00000163612.10	ENSG00000267146.1	ENSG00000248971.2
11096	ENSG00000163617.11	ENSG00000267145.1	ENSG00000227776.1
11097	ENSG00000163618.18	ENSG00000267144.1	ENSG00000254721.1
11098	ENSG00000163623.9	ENSG00000267143.1	ENSG00000199477.1
11099	ENSG00000163624.6	ENSG00000267142.1	ENSG00000154639.19
11100	ENSG00000163625.15	ENSG00000267141.1	ENSG00000213140.3
11101	ENSG00000163626.16	ENSG00000267140.1	ENSG00000220267.1
11102	ENSG00000163629.13	ENSG00000267139.1	ENSG00000169385.3
11103	ENSG00000163630.11	ENSG00000267138.1	ENSG00000277918.1
11104	ENSG00000163631.17	ENSG00000267137.1	ENSG00000116670.15
11105	ENSG00000163632.13	ENSG00000267136.1	ENSG00000223965.2
11106	ENSG00000163633.12	ENSG00000267135.2	ENSG00000269578.1
11107	ENSG00000163634.12	ENSG00000267134.2	ENSG00000115008.5
11108	ENSG00000163635.18	ENSG00000267133.1	ENSG00000229582.3
11109	ENSG00000163636.10	ENSG00000267132.1	ENSG00000144730.18
11110	ENSG00000163637.13	ENSG00000267131.1	ENSG00000205236.6
11111	ENSG00000163638.13	ENSG00000267130.1	ENSG00000172247.4
11112	ENSG00000163644.15	ENSG00000267129.1	ENSG00000280350.1
11113	ENSG00000163645.15	ENSG00000267128.1	ENSG00000234881.1
11114	ENSG00000163646.11	ENSG00000267127.7	ENSG00000213560.4
11115	ENSG00000163655.16	ENSG00000267124.2	ENSG00000113389.16
11116	ENSG00000163659.13	ENSG00000267123.6	ENSG00000232750.2
11117	ENSG00000163660.11	ENSG00000267122.1	ENSG00000270008.1
11118	ENSG00000163661.4	ENSG00000267121.5	ENSG00000223621.1
11119	ENSG00000163666.10	ENSG00000267120.3	ENSG00000233228.1
11120	ENSG00000163673.7	ENSG00000267119.1	ENSG00000224217.1
11121	ENSG00000163681.14	ENSG00000267117.1	ENSG00000237200.1
11122	ENSG00000163682.16	ENSG00000267116.1	ENSG00000187569.3
11123	ENSG00000163683.12	ENSG00000267115.1	ENSG00000243181.2
11124	ENSG00000163684.11	ENSG00000267114.1	ENSG00000237473.1
11125	ENSG00000163686.14	ENSG00000267113.1	ENSG00000271771.1
11126	ENSG00000163687.13	ENSG00000267112.1	ENSG00000282527.1
11127	ENSG00000163689.20	ENSG00000267110.1	ENSG00000264727.1

11128	ENSG00000163694.15	ENSG00000267109.1	ENSG00000271278.1
11129	ENSG00000163697.17	ENSG00000267108.1	ENSG00000180447.7
11130	ENSG00000163701.19	ENSG00000267107.7	ENSG00000121594.12
11131	ENSG00000163702.20	ENSG00000267106.6	ENSG00000252623.1
11132	ENSG00000163703.17	ENSG00000267105.1	ENSG00000262248.1
11133	ENSG00000163704.12	ENSG00000267104.2	ENSG00000255481.2
11134	ENSG00000163705.12	ENSG00000267102.1	ENSG00000178503.6
11135	ENSG00000163710.9	ENSG00000267101.6	ENSG00000270137.1
11136	ENSG00000163714.17	ENSG00000267100.1	ENSG00000213579.3
11137	ENSG00000163719.19	ENSG00000267099.1	ENSG00000259780.3
11138	ENSG00000163728.11	ENSG00000267098.1	ENSG00000183148.6
11139	ENSG00000163734.4	ENSG00000267097.1	ENSG00000256338.2
11140	ENSG00000163735.7	ENSG00000267096.1	ENSG00000162374.17
11141	ENSG00000163736.4	ENSG00000267095.1	ENSG00000213938.3
11142	ENSG00000163737.3	ENSG00000267092.2	ENSG00000183023.18
11143	ENSG00000163738.18	ENSG00000267091.1	ENSG00000254865.1
11144	ENSG00000163739.4	ENSG00000267090.1	ENSG00000265401.1
11145	ENSG00000163743.13	ENSG00000267089.1	ENSG00000233954.6
11146	ENSG00000163746.11	ENSG00000267088.1	ENSG00000241529.3
11147	ENSG00000163749.17	ENSG00000267085.1	ENSG00000101203.16
11148	ENSG00000163751.4	ENSG00000267083.1	ENSG00000201544.1
11149	ENSG00000163754.17	ENSG00000267081.1	ENSG00000117707.15
11150	ENSG00000163755.8	ENSG00000267080.5	ENSG00000137225.13
11151	ENSG00000163762.7	ENSG00000267079.1	ENSG00000258736.1
11152	ENSG00000163781.13	ENSG00000267078.1	ENSG00000123595.7
11153	ENSG00000163785.12	ENSG00000267077.1	ENSG00000074590.13
11154	ENSG00000163788.14	ENSG00000267076.1	ENSG00000213386.3
11155	ENSG00000163792.6	ENSG00000267075.1	ENSG00000148734.7
11156	ENSG00000163793.12	ENSG00000267074.1	ENSG00000273183.1
11157	ENSG00000163794.6	ENSG00000267073.1	ENSG00000275410.5
11158	ENSG00000163795.14	ENSG00000267072.2	ENSG00000269694.1
11159	ENSG00000163798.13	ENSG00000267070.1	ENSG00000163508.12
11160	ENSG00000163803.13	ENSG00000267069.1	ENSG00000205822.10
11161	ENSG00000163806.16	ENSG00000267066.1	ENSG00000259866.1
11162	ENSG00000163807.6	ENSG00000267065.2	ENSG00000279114.1
11163	ENSG00000163808.17	ENSG00000267064.1	ENSG00000285839.1
11164	ENSG00000163810.11	ENSG00000267063.1	ENSG00000230163.1
11165	ENSG00000163811.12	ENSG00000267062.1	ENSG00000248522.1
11166	ENSG00000163812.14	ENSG00000267061.1	ENSG00000285694.1
11167	ENSG00000163814.8	ENSG00000267060.5	ENSG00000284625.1
11168	ENSG00000163815.6	ENSG00000267059.2	ENSG00000108256.9
11169	ENSG00000163817.16	ENSG00000267058.1	ENSG00000273269.3
11170	ENSG00000163818.17	ENSG00000267057.5	ENSG00000049245.13
11171	ENSG00000163820.15	ENSG00000267056.2	ENSG00000234995.1
11172	ENSG00000163823.4	ENSG00000267055.1	ENSG00000278725.1
11173	ENSG00000163825.4	ENSG00000267054.1	ENSG00000284041.1
11174	ENSG00000163827.13	ENSG00000267053.6	ENSG00000268144.1
11175	ENSG00000163832.16	ENSG00000267052.2	ENSG00000198178.10
11176	ENSG00000163833.8	ENSG00000267051.1	ENSG00000215883.10
11177	ENSG00000163840.10	ENSG00000267049.1	ENSG00000282804.1
11178	ENSG00000163848.19	ENSG00000267048.1	ENSG00000243429.1
11179	ENSG00000163864.17	ENSG00000267047.1	ENSG00000273816.1
11180	ENSG00000163866.9	ENSG00000267046.1	ENSG00000243562.3

11181	ENSG00000163867.17	ENSG00000267044.1	ENSG00000263690.2
11182	ENSG00000163870.15	ENSG00000267042.1	ENSG00000224569.3
11183	ENSG00000163872.16	ENSG00000267041.6	ENSG00000280022.1
11184	ENSG00000163873.10	ENSG00000267040.6	ENSG00000131094.4
11185	ENSG00000163874.11	ENSG00000267039.1	ENSG00000273294.1
11186	ENSG00000163875.15	ENSG00000267038.1	ENSG00000274505.1
11187	ENSG00000163877.11	ENSG00000267036.2	ENSG00000244730.1
11188	ENSG00000163879.11	ENSG00000267035.1	ENSG00000210184.1
11189	ENSG00000163882.9	ENSG00000267034.1	ENSG00000212657.1
11190	ENSG00000163884.4	ENSG00000267033.1	ENSG00000129636.12
11191	ENSG00000163885.12	ENSG00000267032.1	ENSG00000180042.5
11192	ENSG00000163888.4	ENSG00000267030.1	ENSG00000239614.1
11193	ENSG00000163898.10	ENSG00000267028.1	ENSG00000117472.10
11194	ENSG00000163900.11	ENSG00000267027.1	ENSG00000259402.1
11195	ENSG00000163902.12	ENSG00000267026.5	ENSG00000133433.10
11196	ENSG00000163904.13	ENSG00000267024.1	ENSG00000236546.1
11197	ENSG00000163909.8	ENSG00000267023.5	ENSG00000116990.11
11198	ENSG00000163913.11	ENSG00000267022.1	ENSG00000263501.1
11199	ENSG00000163914.4	ENSG00000267019.1	ENSG00000233487.6
11200	ENSG00000163915.7	ENSG00000267016.1	ENSG00000276067.2
11201	ENSG00000163918.10	ENSG00000267015.1	ENSG00000229491.5
11202	ENSG00000163923.10	ENSG00000267014.5	ENSG00000089351.14
11203	ENSG00000163930.10	ENSG00000267013.5	ENSG00000168748.14
11204	ENSG00000163931.16	ENSG00000267011.5	ENSG00000213648.10
11205	ENSG00000163932.14	ENSG00000267007.1	ENSG00000279719.1
11206	ENSG00000163933.10	ENSG00000267006.1	ENSG00000261025.1
11207	ENSG00000163935.14	ENSG00000267005.1	ENSG00000275811.1
11208	ENSG00000163938.17	ENSG00000267004.1	ENSG00000215914.4
11209	ENSG00000163939.18	ENSG00000267002.3	ENSG00000164181.14
11210	ENSG00000163945.16	ENSG00000267001.1	ENSG00000264078.1
11211	ENSG00000163946.13	ENSG00000267000.1	ENSG00000088053.11
11212	ENSG00000163947.11	ENSG00000266998.1	ENSG00000139973.16
11213	ENSG00000163950.13	ENSG00000266997.2	ENSG00000241052.1
11214	ENSG00000163956.12	ENSG00000266995.1	ENSG00000234500.1
11215	ENSG00000163958.14	ENSG00000266994.1	ENSG00000198959.12
11216	ENSG00000163959.10	ENSG00000266993.3	ENSG00000283228.1
11217	ENSG00000163960.12	ENSG00000266992.1	ENSG00000145879.11
11218	ENSG00000163961.4	ENSG00000266990.1	ENSG00000268713.1
11219	ENSG00000163964.14	ENSG00000266989.1	ENSG00000164220.7
11220	ENSG00000163975.12	ENSG00000266988.5	ENSG00000170962.13
11221	ENSG00000163982.5	ENSG00000266987.1	ENSG00000280103.1
11222	ENSG00000163993.7	ENSG00000266985.1	ENSG00000229859.9
11223	ENSG00000163995.20	ENSG00000266984.1	ENSG00000286269.1
11224	ENSG00000164002.11	ENSG00000266983.1	ENSG00000279796.1
11225	ENSG00000164007.10	ENSG00000266981.1	ENSG00000189058.9
11226	ENSG00000164008.16	ENSG00000266980.1	ENSG00000164535.15
11227	ENSG00000164010.15	ENSG00000266979.1	ENSG00000230063.1
11228	ENSG00000164011.18	ENSG00000266978.1	ENSG00000239263.1
11229	ENSG00000164022.16	ENSG00000266977.1	ENSG00000254399.2
11230	ENSG00000164023.14	ENSG00000266976.2	ENSG00000280095.1
11231	ENSG00000164024.12	ENSG00000266975.1	ENSG00000233099.1
11232	ENSG00000164031.17	ENSG00000266973.1	ENSG00000270106.6
11233	ENSG00000164032.12	ENSG00000266971.1	ENSG00000136573.14

11234	ENSG00000164035.10	ENSG00000266970.1	ENSG00000230313.1
11235	ENSG00000164037.16	ENSG00000266969.1	ENSG00000184009.11
11236	ENSG00000164038.15	ENSG00000266968.2	ENSG00000112303.14
11237	ENSG00000164039.15	ENSG00000266967.7	ENSG00000261667.1
11238	ENSG00000164040.16	ENSG00000266965.1	ENSG00000267694.1
11239	ENSG00000164045.11	ENSG00000266964.5	ENSG00000266101.1
11240	ENSG00000164047.5	ENSG00000266963.1	ENSG00000254489.1
11241	ENSG00000164048.14	ENSG00000266962.2	ENSG00000259756.1
11242	ENSG00000164049.14	ENSG00000266961.1	ENSG00000243333.3
11243	ENSG00000164050.13	ENSG00000266959.1	ENSG00000251614.1
11244	ENSG00000164051.14	ENSG00000266958.1	ENSG00000286268.1
11245	ENSG00000164053.21	ENSG00000266957.1	ENSG00000103018.16
11246	ENSG00000164054.15	ENSG00000266955.1	ENSG00000227742.2
11247	ENSG00000164056.11	ENSG00000266954.1	ENSG00000242756.2
11248	ENSG00000164061.5	ENSG00000266953.6	ENSG00000233626.2
11249	ENSG00000164062.13	ENSG00000266952.2	ENSG00000141497.14
11250	ENSG00000164066.13	ENSG00000266951.2	ENSG00000124370.11
11251	ENSG00000164068.16	ENSG00000266950.1	ENSG00000180769.9
11252	ENSG00000164070.12	ENSG00000266947.1	ENSG00000227217.1
11253	ENSG00000164073.10	ENSG00000266946.1	ENSG00000263798.2
11254	ENSG00000164074.15	ENSG00000266944.1	ENSG00000271963.1
11255	ENSG00000164076.17	ENSG00000266943.1	ENSG00000241084.1
11256	ENSG00000164077.14	ENSG00000266941.1	ENSG00000100036.13
11257	ENSG00000164078.13	ENSG00000266938.1	ENSG00000276436.1
11258	ENSG00000164080.13	ENSG00000266936.1	ENSG00000283005.1
11259	ENSG00000164081.12	ENSG00000266934.1	ENSG00000275672.1
11260	ENSG00000164082.15	ENSG00000266932.1	ENSG00000238021.6
11261	ENSG00000164086.10	ENSG00000266931.2	ENSG00000207294.1
11262	ENSG00000164087.7	ENSG00000266929.1	ENSG00000255003.1
11263	ENSG00000164088.18	ENSG00000266928.1	ENSG00000281571.2
11264	ENSG00000164089.9	ENSG00000266925.1	ENSG00000273233.1
11265	ENSG00000164091.12	ENSG00000266924.1	ENSG00000176933.5
11266	ENSG00000164093.16	ENSG00000266923.1	ENSG00000267140.1
11267	ENSG00000164096.13	ENSG00000266922.1	ENSG00000282299.1
11268	ENSG00000164099.3	ENSG00000266921.1	ENSG00000277761.1
11269	ENSG00000164100.9	ENSG00000266920.1	ENSG00000284930.1
11270	ENSG00000164104.11	ENSG00000266919.3	ENSG00000256108.1
11271	ENSG00000164105.4	ENSG00000266918.1	ENSG00000113100.10
11272	ENSG00000164106.8	ENSG00000266916.6	ENSG00000158856.18
11273	ENSG00000164107.8	ENSG00000266915.1	ENSG00000243519.1
11274	ENSG00000164109.14	ENSG00000266913.1	ENSG00000204666.3
11275	ENSG00000164111.15	ENSG00000266910.2	ENSG00000272576.1
11276	ENSG00000164112.13	ENSG00000266909.1	ENSG00000278797.1
11277	ENSG00000164113.11	ENSG00000266908.1	ENSG00000213778.2
11278	ENSG00000164114.19	ENSG00000266907.1	ENSG00000163933.10
11279	ENSG00000164116.16	ENSG00000266906.1	ENSG00000233740.1
11280	ENSG00000164117.13	ENSG00000266905.1	ENSG00000250033.5
11281	ENSG00000164118.13	ENSG00000266904.5	ENSG00000237506.3
11282	ENSG00000164120.14	ENSG00000266903.1	ENSG00000269482.1
11283	ENSG00000164122.8	ENSG00000266901.1	ENSG00000277022.1
11284	ENSG00000164123.6	ENSG00000266900.1	ENSG00000036549.13
11285	ENSG00000164124.10	ENSG00000266899.2	ENSG00000258608.1
11286	ENSG00000164125.15	ENSG00000266897.1	ENSG00000264113.2

11287	ENSG00000164128.7	ENSG00000266896.1	ENSG00000105290.12
11288	ENSG00000164129.12	ENSG00000266893.1	ENSG00000107954.10
11289	ENSG00000164134.13	ENSG00000266891.1	ENSG00000100060.17
11290	ENSG00000164136.17	ENSG00000266890.1	ENSG00000254973.1
11291	ENSG00000164142.16	ENSG00000266887.1	ENSG00000255240.5
11292	ENSG00000164144.16	ENSG00000266885.2	ENSG00000259598.1
11293	ENSG00000164151.12	ENSG00000266877.1	ENSG00000171462.15
11294	ENSG00000164161.10	ENSG00000266876.1	ENSG00000273586.2
11295	ENSG00000164162.14	ENSG00000266875.2	ENSG00000225110.2
11296	ENSG00000164163.11	ENSG00000266872.1	ENSG00000172320.3
11297	ENSG00000164164.16	ENSG00000266869.1	ENSG00000259083.1
11298	ENSG00000164167.12	ENSG00000266865.6	ENSG00000039600.11
11299	ENSG00000164168.7	ENSG00000266863.2	ENSG00000149050.10
11300	ENSG00000164169.13	ENSG00000266858.1	ENSG00000248126.1
11301	ENSG00000164171.11	ENSG00000266855.1	ENSG00000223345.3
11302	ENSG00000164172.19	ENSG00000266853.1	ENSG00000277157.1
11303	ENSG00000164175.15	ENSG00000266852.2	ENSG00000220739.2
11304	ENSG00000164176.13	ENSG00000266850.1	ENSG00000139132.14
11305	ENSG00000164180.13	ENSG00000266846.1	ENSG00000279094.3
11306	ENSG00000164181.14	ENSG00000266844.1	ENSG00000103512.15
11307	ENSG00000164182.11	ENSG00000266840.1	ENSG00000283303.1
11308	ENSG00000164185.6	ENSG00000266839.3	ENSG00000170315.13
11309	ENSG00000164187.7	ENSG00000266835.5	ENSG00000138083.5
11310	ENSG00000164188.8	ENSG00000266830.1	ENSG00000240106.2
11311	ENSG00000164190.18	ENSG00000266828.2	ENSG00000197536.11
11312	ENSG00000164197.12	ENSG00000266827.1	ENSG00000171496.4
11313	ENSG00000164199.18	ENSG00000266826.2	ENSG00000238324.1
11314	ENSG00000164209.17	ENSG00000266824.1	ENSG00000225172.5
11315	ENSG00000164211.13	ENSG00000266820.1	ENSG00000219438.8
11316	ENSG00000164219.10	ENSG00000266818.1	ENSG00000137947.12
11317	ENSG00000164220.7	ENSG00000266809.1	ENSG00000244451.1
11318	ENSG00000164221.13	ENSG00000266808.1	ENSG00000276160.1
11319	ENSG00000164236.12	ENSG00000266807.1	ENSG00000234896.1
11320	ENSG00000164237.9	ENSG00000266806.1	ENSG00000074319.13
11321	ENSG00000164241.13	ENSG00000266805.1	ENSG00000119522.16
11322	ENSG00000164244.20	ENSG00000266803.1	ENSG00000179571.10
11323	ENSG00000164251.5	ENSG00000266802.1	ENSG00000270911.1
11324	ENSG00000164252.13	ENSG00000266801.1	ENSG00000175221.15
11325	ENSG00000164253.14	ENSG00000266794.2	ENSG00000231943.8
11326	ENSG00000164256.10	ENSG00000266786.1	ENSG00000219435.6
11327	ENSG00000164258.12	ENSG00000266783.1	ENSG00000206927.1
11328	ENSG00000164265.9	ENSG00000266782.1	ENSG00000104892.17
11329	ENSG00000164266.10	ENSG00000266780.1	ENSG00000164244.20
11330	ENSG00000164270.17	ENSG00000266777.1	ENSG00000205592.14
11331	ENSG00000164283.13	ENSG00000266776.1	ENSG00000168928.13
11332	ENSG00000164284.15	ENSG00000266775.1	ENSG00000266195.2
11333	ENSG00000164287.12	ENSG00000266774.1	ENSG00000226147.1
11334	ENSG00000164291.16	ENSG00000266771.1	ENSG00000221953.5
11335	ENSG00000164292.13	ENSG00000266770.2	ENSG00000222022.1
11336	ENSG00000164294.14	ENSG00000266767.1	ENSG00000227359.1
11337	ENSG00000164296.7	ENSG00000266765.1	ENSG00000224185.1
11338	ENSG00000164299.6	ENSG00000266761.1	ENSG00000229217.1
11339	ENSG00000164300.17	ENSG00000266760.1	ENSG00000223642.7

11340	ENSG00000164303.11	ENSG00000266758.1	ENSG00000270039.1
11341	ENSG00000164304.16	ENSG00000266756.1	ENSG00000230026.2
11342	ENSG00000164305.19	ENSG00000266754.2	ENSG00000196482.17
11343	ENSG00000164306.11	ENSG00000266751.1	ENSG00000255101.1
11344	ENSG00000164307.13	ENSG00000266750.1	ENSG00000213453.3
11345	ENSG00000164308.16	ENSG00000266745.1	ENSG00000274569.1
11346	ENSG00000164309.15	ENSG00000266744.1	ENSG00000196504.16
11347	ENSG00000164318.18	ENSG00000266743.1	ENSG00000256185.1
11348	ENSG00000164323.14	ENSG00000266740.1	ENSG00000265327.2
11349	ENSG00000164325.8	ENSG00000266738.1	ENSG00000125319.14
11350	ENSG00000164326.5	ENSG00000266736.1	ENSG00000250575.1
11351	ENSG00000164327.13	ENSG00000266733.7	ENSG00000261529.1
11352	ENSG00000164329.13	ENSG00000266729.5	ENSG00000149735.7
11353	ENSG00000164330.17	ENSG00000266728.5	ENSG00000168116.14
11354	ENSG00000164331.10	ENSG00000266721.1	ENSG00000223522.1
11355	ENSG00000164332.8	ENSG00000266720.2	ENSG00000236199.1
11356	ENSG00000164334.15	ENSG00000266719.1	ENSG00000240247.7
11357	ENSG00000164338.10	ENSG00000266718.2	ENSG00000188383.8
11358	ENSG00000164342.12	ENSG00000266717.1	ENSG00000235782.1
11359	ENSG00000164344.16	ENSG00000266714.9	ENSG00000157168.19
11360	ENSG00000164346.10	ENSG00000266712.3	ENSG00000224340.1
11361	ENSG00000164347.18	ENSG00000266711.1	ENSG00000259989.1
11362	ENSG00000164362.19	ENSG00000266710.2	ENSG00000236281.1
11363	ENSG00000164363.10	ENSG00000266707.1	ENSG00000242715.7
11364	ENSG00000164366.3	ENSG00000266705.1	ENSG00000224800.1
11365	ENSG00000164379.7	ENSG00000266704.1	ENSG00000261645.6
11366	ENSG00000164385.8	ENSG00000266703.1	ENSG00000270802.2
11367	ENSG00000164393.8	ENSG00000266698.1	ENSG00000242182.3
11368	ENSG00000164398.13	ENSG00000266696.1	ENSG00000278969.1
11369	ENSG00000164399.4	ENSG00000266693.1	ENSG00000230584.1
11370	ENSG00000164400.6	ENSG00000266692.1	ENSG00000227913.1
11371	ENSG00000164402.14	ENSG00000266691.1	ENSG00000226067.6
11372	ENSG00000164403.14	ENSG00000266690.1	ENSG00000271978.1
11373	ENSG00000164404.8	ENSG00000266680.1	ENSG00000226823.1
11374	ENSG00000164405.11	ENSG00000266677.1	ENSG00000254290.1
11375	ENSG00000164406.7	ENSG00000266676.1	ENSG00000180332.6
11376	ENSG00000164411.11	ENSG00000266673.1	ENSG00000258114.1
11377	ENSG00000164414.18	ENSG00000266668.1	ENSG00000254398.1
11378	ENSG00000164418.20	ENSG00000266667.1	ENSG00000182783.5
11379	ENSG00000164430.16	ENSG00000266666.1	ENSG00000243389.1
11380	ENSG00000164434.12	ENSG00000266665.1	ENSG00000240137.5
11381	ENSG00000164438.6	ENSG00000266664.1	ENSG00000283529.1
11382	ENSG00000164440.15	ENSG00000266663.1	ENSG00000280348.1
11383	ENSG00000164442.10	ENSG00000266655.1	ENSG00000149927.18
11384	ENSG00000164451.13	ENSG00000266654.1	ENSG00000255440.1
11385	ENSG00000164458.9	ENSG00000266651.1	ENSG00000214108.4
11386	ENSG00000164463.12	ENSG00000266649.1	ENSG00000244674.1
11387	ENSG00000164465.18	ENSG00000266648.1	ENSG00000233220.3
11388	ENSG00000164466.13	ENSG00000266647.1	ENSG00000225513.1
11389	ENSG00000164483.17	ENSG00000266645.1	ENSG00000138435.15
11390	ENSG00000164484.11	ENSG00000266644.1	ENSG00000180068.10
11391	ENSG00000164485.14	ENSG00000266643.1	ENSG00000223920.2
11392	ENSG00000164488.12	ENSG00000266642.2	ENSG00000249363.1

11393	ENSG00000164494.12	ENSG00000266640.1	ENSG00000077942.19
11394	ENSG00000164500.6	ENSG00000266634.1	ENSG00000261024.7
11395	ENSG00000164506.14	ENSG00000266627.2	ENSG00000230569.1
11396	ENSG00000164508.4	ENSG00000266619.1	ENSG00000267360.6
11397	ENSG00000164509.14	ENSG00000266618.1	ENSG00000267395.5
11398	ENSG00000164512.18	ENSG00000266617.1	ENSG00000285577.1
11399	ENSG00000164520.11	ENSG00000266614.1	ENSG00000163126.15
11400	ENSG00000164530.15	ENSG00000266613.1	ENSG00000277916.1
11401	ENSG00000164532.10	ENSG00000266611.1	ENSG00000170632.14
11402	ENSG00000164535.15	ENSG00000266610.2	ENSG00000249540.1
11403	ENSG00000164542.12	ENSG00000266605.1	ENSG00000120949.15
11404	ENSG00000164543.7	ENSG00000266602.1	ENSG00000177182.11
11405	ENSG00000164548.11	ENSG00000266601.1	ENSG00000238078.1
11406	ENSG00000164556.7	ENSG00000266599.1	ENSG00000211749.1
11407	ENSG00000164574.16	ENSG00000266598.1	ENSG00000223734.2
11408	ENSG00000164576.12	ENSG00000266594.1	ENSG00000158553.4
11409	ENSG00000164587.13	ENSG00000266593.1	ENSG00000236188.1
11410	ENSG00000164588.7	ENSG00000266589.1	ENSG00000222038.3
11411	ENSG00000164591.13	ENSG00000266588.1	ENSG00000224126.2
11412	ENSG00000164597.13	ENSG00000266586.1	ENSG00000221164.1
11413	ENSG00000164600.7	ENSG00000266583.1	ENSG00000187904.3
11414	ENSG00000164603.12	ENSG00000266582.1	ENSG00000258593.2
11415	ENSG00000164604.12	ENSG00000266581.1	ENSG00000236483.1
11416	ENSG00000164609.10	ENSG00000266580.1	ENSG00000269985.1
11417	ENSG00000164610.9	ENSG00000266579.1	ENSG00000181409.13
11418	ENSG00000164611.13	ENSG00000266578.1	ENSG00000279660.1
11419	ENSG00000164615.5	ENSG00000266575.1	ENSG00000227374.1
11420	ENSG00000164616.16	ENSG00000266573.5	ENSG00000234156.2
11421	ENSG00000164619.10	ENSG00000266570.1	ENSG00000181222.15
11422	ENSG00000164620.9	ENSG00000266569.2	ENSG00000224203.2
11423	ENSG00000164621.5	ENSG00000266564.1	ENSG00000285569.1
11424	ENSG00000164626.9	ENSG00000266563.1	ENSG00000259286.3
11425	ENSG00000164627.18	ENSG00000266561.2	ENSG00000082213.18
11426	ENSG00000164631.19	ENSG00000266560.5	ENSG00000258285.1
11427	ENSG00000164638.10	ENSG00000266559.1	ENSG00000248477.6
11428	ENSG00000164645.3	ENSG00000266555.1	ENSG00000183570.16
11429	ENSG00000164647.9	ENSG00000266554.2	ENSG00000184203.8
11430	ENSG00000164649.20	ENSG00000266553.2	ENSG00000205057.5
11431	ENSG00000164651.16	ENSG00000266549.1	ENSG00000107863.18
11432	ENSG00000164654.16	ENSG00000266545.1	ENSG00000155761.13
11433	ENSG00000164659.15	ENSG00000266541.1	ENSG00000248672.5
11434	ENSG00000164663.14	ENSG00000266538.1	ENSG00000274559.3
11435	ENSG00000164674.15	ENSG00000266537.1	ENSG00000226098.4
11436	ENSG00000164675.11	ENSG00000266535.1	ENSG00000248795.1
11437	ENSG00000164683.17	ENSG00000266533.1	ENSG00000268705.1
11438	ENSG00000164684.13	ENSG00000266531.1	ENSG00000188761.13
11439	ENSG00000164687.11	ENSG00000266530.1	ENSG00000269516.6
11440	ENSG00000164690.8	ENSG00000266529.1	ENSG00000267669.1
11441	ENSG00000164691.17	ENSG00000266527.1	ENSG00000141391.14
11442	ENSG00000164692.17	ENSG00000266525.1	ENSG00000118193.12
11443	ENSG00000164694.17	ENSG00000266524.3	ENSG00000260648.3
11444	ENSG00000164695.5	ENSG00000266522.1	ENSG00000255059.1
11445	ENSG00000164707.15	ENSG00000266521.1	ENSG00000135903.19

11446	ENSG00000164708.6	ENSG00000266520.1	ENSG00000275613.1
11447	ENSG00000164713.9	ENSG00000266518.1	ENSG00000279851.2
11448	ENSG00000164715.6	ENSG00000266517.1	ENSG00000224216.1
11449	ENSG00000164729.7	ENSG00000266515.1	ENSG00000199627.1
11450	ENSG00000164733.21	ENSG00000266514.1	ENSG00000240371.1
11451	ENSG00000164736.6	ENSG00000266513.1	ENSG00000244251.1
11452	ENSG00000164740.5	ENSG00000266509.1	ENSG00000175352.11
11453	ENSG00000164741.15	ENSG00000266508.1	ENSG00000230912.1
11454	ENSG00000164742.16	ENSG00000266507.1	ENSG00000232196.3
11455	ENSG00000164743.5	ENSG00000266505.1	ENSG00000204866.8
11456	ENSG00000164744.13	ENSG00000266504.1	ENSG00000173762.8
11457	ENSG00000164746.14	ENSG00000266503.2	ENSG00000276568.1
11458	ENSG00000164749.11	ENSG00000266502.1	ENSG00000259138.1
11459	ENSG00000164751.15	ENSG00000266501.1	ENSG00000206178.2
11460	ENSG00000164754.14	ENSG00000266498.1	ENSG00000167208.15
11461	ENSG00000164756.12	ENSG00000266497.1	ENSG00000162669.16
11462	ENSG00000164758.7	ENSG00000266495.1	ENSG00000285925.1
11463	ENSG00000164761.9	ENSG00000266494.1	ENSG00000275291.1
11464	ENSG00000164764.11	ENSG00000266490.1	ENSG00000268799.3
11465	ENSG00000164776.9	ENSG00000266489.2	ENSG00000267755.1
11466	ENSG00000164778.4	ENSG00000266479.1	ENSG00000248698.6
11467	ENSG00000164794.9	ENSG00000266478.1	ENSG00000280424.1
11468	ENSG00000164796.18	ENSG00000266477.2	ENSG00000272508.1
11469	ENSG00000164808.16	ENSG00000266473.1	ENSG00000232460.4
11470	ENSG00000164815.11	ENSG00000266472.5	ENSG00000273336.1
11471	ENSG00000164816.8	ENSG00000266470.1	ENSG00000247624.6
11472	ENSG00000164818.16	ENSG00000266469.1	ENSG00000215131.10
11473	ENSG00000164821.4	ENSG00000266467.2	ENSG00000256812.1
11474	ENSG00000164822.5	ENSG00000266466.1	ENSG00000250381.1
11475	ENSG00000164823.11	ENSG00000266463.1	ENSG00000250286.1
11476	ENSG00000164825.4	ENSG00000266461.1	ENSG00000265182.1
11477	ENSG00000164828.18	ENSG00000266460.1	ENSG00000266954.1
11478	ENSG00000164830.18	ENSG00000266458.1	ENSG00000131914.11
11479	ENSG00000164841.5	ENSG00000266456.1	ENSG00000258989.1
11480	ENSG00000164845.16	ENSG00000266454.1	ENSG00000202538.1
11481	ENSG00000164849.10	ENSG00000266450.1	ENSG00000134815.19
11482	ENSG00000164850.15	ENSG00000266449.1	ENSG00000229672.2
11483	ENSG00000164853.9	ENSG00000266448.1	ENSG00000261863.1
11484	ENSG00000164855.16	ENSG00000266446.1	ENSG00000251568.1
11485	ENSG00000164867.11	ENSG00000266445.1	ENSG00000248923.1
11486	ENSG00000164871.17	ENSG00000266441.1	ENSG00000227192.1
11487	ENSG00000164877.19	ENSG00000266439.2	ENSG00000227195.10
11488	ENSG00000164879.7	ENSG00000266437.1	ENSG00000230453.9
11489	ENSG00000164880.16	ENSG00000266436.1	ENSG00000270742.1
11490	ENSG00000164885.13	ENSG00000266433.6	ENSG00000220522.2
11491	ENSG00000164889.14	ENSG00000266431.1	ENSG00000236915.2
11492	ENSG00000164893.9	ENSG00000266426.1	ENSG00000261553.5
11493	ENSG00000164896.20	ENSG00000266423.1	ENSG00000282499.1
11494	ENSG00000164897.13	ENSG00000266421.3	ENSG00000276409.5
11495	ENSG00000164898.13	ENSG00000266420.2	ENSG00000230783.1
11496	ENSG00000164900.4	ENSG00000266417.1	ENSG00000259818.1
11497	ENSG00000164902.14	ENSG00000266416.1	ENSG00000127528.5
11498	ENSG00000164904.18	ENSG00000266415.1	ENSG00000268870.1

11499	ENSG00000164916.11	ENSG00000266412.5	ENSG00000196534.5
11500	ENSG00000164919.11	ENSG00000266407.1	ENSG00000115592.11
11501	ENSG00000164920.9	ENSG00000266405.3	ENSG00000226321.5
11502	ENSG00000164924.18	ENSG00000266402.3	ENSG00000138207.14
11503	ENSG00000164929.17	ENSG00000266401.1	ENSG00000240356.6
11504	ENSG00000164930.12	ENSG00000266397.1	ENSG00000275569.1
11505	ENSG00000164932.13	ENSG00000266396.1	ENSG00000110060.9
11506	ENSG00000164933.12	ENSG00000266392.1	ENSG00000234377.7
11507	ENSG00000164934.14	ENSG00000266389.1	ENSG00000240409.1
11508	ENSG00000164935.6	ENSG00000266385.1	ENSG00000231982.1
11509	ENSG00000164938.14	ENSG00000266383.1	ENSG00000005238.19
11510	ENSG00000164941.13	ENSG00000266379.6	ENSG00000068489.12
11511	ENSG00000164944.12	ENSG00000266378.1	ENSG00000184434.7
11512	ENSG00000164946.19	ENSG00000266373.1	ENSG00000240457.3
11513	ENSG00000164949.8	ENSG00000266371.1	ENSG00000244527.1
11514	ENSG00000164951.16	ENSG00000266370.1	ENSG00000185028.4
11515	ENSG00000164953.15	ENSG00000266369.1	ENSG00000271868.1
11516	ENSG00000164961.16	ENSG00000266368.1	ENSG00000125931.11
11517	ENSG00000164967.10	ENSG00000266365.1	ENSG00000065833.9
11518	ENSG00000164970.15	ENSG00000266364.1	ENSG00000103832.10
11519	ENSG00000164972.13	ENSG00000266357.5	ENSG00000170801.10
11520	ENSG00000164975.15	ENSG00000266356.1	ENSG00000268051.1
11521	ENSG00000164976.9	ENSG00000266354.1	ENSG00000243007.1
11522	ENSG00000164978.18	ENSG00000266341.1	ENSG00000231439.4
11523	ENSG00000164983.7	ENSG00000266340.1	ENSG00000255303.2
11524	ENSG00000164985.15	ENSG00000266338.6	ENSG00000165935.9
11525	ENSG00000164989.17	ENSG00000266335.1	ENSG00000256278.1
11526	ENSG00000165006.14	ENSG00000266329.1	ENSG00000225891.1
11527	ENSG00000165023.7	ENSG00000266328.3	ENSG00000256148.1
11528	ENSG00000165025.15	ENSG00000266327.1	ENSG00000237746.1
11529	ENSG00000165028.12	ENSG00000266325.1	ENSG00000049759.18
11530	ENSG00000165029.16	ENSG00000266324.1	ENSG00000234152.1
11531	ENSG00000165030.4	ENSG00000266321.1	ENSG00000146094.14
11532	ENSG00000165046.12	ENSG00000266320.1	ENSG00000143862.8
11533	ENSG00000165055.15	ENSG00000266318.1	ENSG00000250794.2
11534	ENSG00000165059.7	ENSG00000266317.2	ENSG00000156218.13
11535	ENSG00000165060.13	ENSG00000266315.1	ENSG00000197748.12
11536	ENSG00000165061.15	ENSG00000266313.1	ENSG00000276031.1
11537	ENSG00000165066.12	ENSG00000266312.1	ENSG00000130208.9
11538	ENSG00000165071.14	ENSG00000266311.6	ENSG00000177788.5
11539	ENSG00000165072.10	ENSG00000266308.2	ENSG00000259198.1
11540	ENSG00000165076.13	ENSG00000266307.1	ENSG00000277299.1
11541	ENSG00000165078.12	ENSG00000266306.1	ENSG00000182798.10
11542	ENSG00000165084.16	ENSG00000266305.1	ENSG00000264840.1
11543	ENSG00000165091.17	ENSG00000266304.1	ENSG00000224224.1
11544	ENSG00000165092.13	ENSG00000266302.6	ENSG00000114638.8
11545	ENSG00000165097.15	ENSG00000266299.1	ENSG00000250346.1
11546	ENSG00000165102.15	ENSG00000266297.1	ENSG00000135045.7
11547	ENSG00000165105.10	ENSG00000266296.1	ENSG00000270230.1
11548	ENSG00000165113.13	ENSG00000266291.1	ENSG00000199313.1
11549	ENSG00000165115.15	ENSG00000266290.1	ENSG00000249005.3
11550	ENSG00000165118.15	ENSG00000266289.1	ENSG00000253780.1
11551	ENSG00000165119.21	ENSG00000266288.1	ENSG00000017483.15

11552	ENSG00000165120.5	ENSG00000266287.1	ENSG00000270578.1
11553	ENSG00000165121.11	ENSG00000266283.1	ENSG00000277545.1
11554	ENSG00000165124.18	ENSG00000266282.1	ENSG00000263293.2
11555	ENSG00000165125.20	ENSG00000266279.1	ENSG00000217862.2
11556	ENSG00000165131.7	ENSG00000266278.1	ENSG00000198830.11
11557	ENSG00000165138.18	ENSG00000266276.3	ENSG00000227589.1
11558	ENSG00000165140.10	ENSG00000266274.2	ENSG00000147813.16
11559	ENSG00000165152.9	ENSG00000266273.1	ENSG00000252835.1
11560	ENSG00000165156.15	ENSG00000266270.1	ENSG00000285792.1
11561	ENSG00000165164.14	ENSG00000266268.5	ENSG00000251246.1
11562	ENSG00000165168.7	ENSG00000266265.3	ENSG00000234865.3
11563	ENSG00000165169.11	ENSG00000266262.1	ENSG00000275029.1
11564	ENSG00000165171.11	ENSG00000266261.1	ENSG00000105737.9
11565	ENSG00000165175.15	ENSG00000266258.1	ENSG00000258959.2
11566	ENSG00000165178.9	ENSG00000266256.2	ENSG00000130150.12
11567	ENSG00000165181.16	ENSG00000266255.1	ENSG00000243243.5
11568	ENSG00000165182.11	ENSG00000266251.1	ENSG00000277498.1
11569	ENSG00000165185.14	ENSG00000266248.1	ENSG00000104320.13
11570	ENSG00000165186.12	ENSG00000266245.1	ENSG00000226002.1
11571	ENSG00000165188.13	ENSG00000266243.1	ENSG00000259098.1
11572	ENSG00000165192.13	ENSG00000266242.1	ENSG00000264940.4
11573	ENSG00000165194.15	ENSG00000266240.1	ENSG00000267475.1
11574	ENSG00000165195.15	ENSG00000266237.1	ENSG00000240759.1
11575	ENSG00000165197.5	ENSG00000266236.1	ENSG00000255320.1
11576	ENSG00000165202.3	ENSG00000266235.1	ENSG00000284660.1
11577	ENSG00000165204.3	ENSG00000266232.1	ENSG00000141012.13
11578	ENSG00000165209.18	ENSG00000266228.1	ENSG00000237281.1
11579	ENSG00000165215.6	ENSG00000266227.1	ENSG00000261336.1
11580	ENSG00000165219.21	ENSG00000266226.1	ENSG00000211968.3
11581	ENSG00000165233.18	ENSG00000266222.1	ENSG00000258919.1
11582	ENSG00000165238.16	ENSG00000266217.2	ENSG00000260274.1
11583	ENSG00000165240.20	ENSG00000266215.1	ENSG00000204464.7
11584	ENSG00000165244.7	ENSG00000266213.1	ENSG00000261570.1
11585	ENSG00000165246.14	ENSG00000266211.1	ENSG00000203852.3
11586	ENSG00000165259.14	ENSG00000266210.2	ENSG00000271466.1
11587	ENSG00000165264.11	ENSG00000266208.1	ENSG00000266651.1
11588	ENSG00000165269.13	ENSG00000266206.1	ENSG00000214089.3
11589	ENSG00000165271.17	ENSG00000266204.1	ENSG00000248710.1
11590	ENSG00000165272.16	ENSG00000266203.1	ENSG00000251333.3
11591	ENSG00000165275.10	ENSG00000266202.1	ENSG00000117906.14
11592	ENSG00000165280.16	ENSG00000266201.1	ENSG00000284391.1
11593	ENSG00000165282.13	ENSG00000266200.6	ENSG00000230113.1
11594	ENSG00000165283.16	ENSG00000266196.1	ENSG00000280017.1
11595	ENSG00000165288.10	ENSG00000266195.2	ENSG00000257880.1
11596	ENSG00000165300.7	ENSG00000266194.1	ENSG00000243660.10
11597	ENSG00000165304.8	ENSG00000266192.3	ENSG00000261656.5
11598	ENSG00000165309.14	ENSG00000266190.1	ENSG00000253562.1
11599	ENSG00000165312.6	ENSG00000266189.1	ENSG00000251655.6
11600	ENSG00000165322.17	ENSG00000266188.1	ENSG00000255291.2
11601	ENSG00000165323.15	ENSG00000266187.2	ENSG00000198951.11
11602	ENSG00000165325.13	ENSG00000266185.2	ENSG00000210151.2
11603	ENSG00000165338.16	ENSG00000266184.1	ENSG00000114626.18
11604	ENSG00000165349.12	ENSG00000266181.1	ENSG00000279705.1

11605	ENSG00000165355.7	ENSG00000266180.1	ENSG00000248569.1
11606	ENSG00000165359.15	ENSG00000266179.2	ENSG00000214761.3
11607	ENSG00000165370.2	ENSG00000266174.1	ENSG00000168883.20
11608	ENSG00000165376.10	ENSG00000266173.7	ENSG00000260192.2
11609	ENSG00000165379.13	ENSG00000266171.1	ENSG00000255549.2
11610	ENSG00000165383.11	ENSG00000266168.1	ENSG00000060762.19
11611	ENSG00000165389.7	ENSG00000266166.2	ENSG00000273890.1
11612	ENSG00000165392.10	ENSG00000266162.1	ENSG00000170790.5
11613	ENSG00000165406.16	ENSG00000266160.2	ENSG00000257950.3
11614	ENSG00000165409.17	ENSG00000266158.2	ENSG00000258922.1
11615	ENSG00000165410.14	ENSG00000266155.2	ENSG00000284627.1
11616	ENSG00000165416.14	ENSG00000266153.1	ENSG00000211725.3
11617	ENSG00000165417.12	ENSG00000266151.1	ENSG00000179930.5
11618	ENSG00000165424.6	ENSG00000266150.1	ENSG00000284464.1
11619	ENSG00000165434.8	ENSG00000266149.1	ENSG00000223945.2
11620	ENSG00000165443.12	ENSG00000266148.1	ENSG00000177238.14
11621	ENSG00000165449.11	ENSG00000266146.1	ENSG00000234622.6
11622	ENSG00000165457.14	ENSG00000266145.1	ENSG00000067798.16
11623	ENSG00000165458.14	ENSG00000266144.1	ENSG00000269526.1
11624	ENSG00000165462.5	ENSG00000266140.1	ENSG00000166928.10
11625	ENSG00000165471.6	ENSG00000266139.2	ENSG00000108509.21
11626	ENSG00000165474.7	ENSG00000266133.1	ENSG00000107521.19
11627	ENSG00000165475.15	ENSG00000266129.1	ENSG00000235034.6
11628	ENSG00000165476.14	ENSG00000266128.2	ENSG00000234488.1
11629	ENSG00000165478.7	ENSG00000266127.1	ENSG00000223908.5
11630	ENSG00000165480.16	ENSG00000266126.1	ENSG00000136867.11
11631	ENSG00000165487.14	ENSG00000266124.1	ENSG00000260088.1
11632	ENSG00000165490.13	ENSG00000266120.1	ENSG00000236138.4
11633	ENSG00000165494.11	ENSG00000266117.1	ENSG00000239247.3
11634	ENSG00000165495.16	ENSG00000266114.1	ENSG00000237298.9
11635	ENSG00000165496.4	ENSG00000266111.2	ENSG00000152527.14
11636	ENSG00000165501.17	ENSG00000266110.1	ENSG00000228882.1
11637	ENSG00000165502.6	ENSG00000266109.1	ENSG00000222489.1
11638	ENSG00000165506.14	ENSG00000266107.1	ENSG00000261873.2
11639	ENSG00000165507.8	ENSG00000266106.1	ENSG00000242272.1
11640	ENSG00000165509.13	ENSG00000266105.1	ENSG00000244131.2
11641	ENSG00000165511.6	ENSG00000266104.1	ENSG00000223695.1
11642	ENSG00000165512.5	ENSG00000266102.1	ENSG00000257529.5
11643	ENSG00000165516.11	ENSG00000266101.1	ENSG00000242522.1
11644	ENSG00000165521.15	ENSG00000266100.1	ENSG00000259364.1
11645	ENSG00000165525.18	ENSG00000266099.1	ENSG00000232341.2
11646	ENSG00000165526.9	ENSG00000266097.1	ENSG00000237763.9
11647	ENSG00000165527.7	ENSG00000266094.7	ENSG00000183134.5
11648	ENSG00000165533.18	ENSG00000266088.5	ENSG00000278344.1
11649	ENSG00000165548.11	ENSG00000266086.2	ENSG00000012817.15
11650	ENSG00000165553.4	ENSG00000266079.4	ENSG00000105679.9
11651	ENSG00000165555.9	ENSG00000266078.1	ENSG00000225264.3
11652	ENSG00000165556.9	ENSG00000266076.1	ENSG00000211724.3
11653	ENSG00000165566.12	ENSG00000266074.8	ENSG00000240707.2
11654	ENSG00000165568.18	ENSG00000266072.1	ENSG00000234176.1
11655	ENSG00000165572.7	ENSG00000266071.1	ENSG00000197584.12
11656	ENSG00000165583.14	ENSG00000266066.1	ENSG00000253917.4
11657	ENSG00000165584.15	ENSG00000266065.1	ENSG00000279404.1

11658	ENSG00000165588.16	ENSG00000266063.1	ENSG00000181322.14
11659	ENSG00000165591.6	ENSG00000266059.2	ENSG00000125508.3
11660	ENSG00000165606.8	ENSG00000266053.2	ENSG00000184497.12
11661	ENSG00000165617.14	ENSG00000266052.1	ENSG00000274397.1
11662	ENSG00000165621.8	ENSG00000266050.3	ENSG00000214128.11
11663	ENSG00000165623.10	ENSG00000266049.1	ENSG00000273618.1
11664	ENSG00000165626.17	ENSG00000266048.1	ENSG00000225246.1
11665	ENSG00000165629.20	ENSG00000266043.1	ENSG00000186451.2
11666	ENSG00000165630.14	ENSG00000266042.1	ENSG00000184635.15
11667	ENSG00000165632.8	ENSG00000266041.1	ENSG00000285845.1
11668	ENSG00000165633.13	ENSG00000266039.1	ENSG00000188000.4
11669	ENSG00000165637.13	ENSG00000266038.1	ENSG00000251408.1
11670	ENSG00000165643.10	ENSG00000266036.1	ENSG00000211678.2
11671	ENSG00000165644.10	ENSG00000266028.7	ENSG00000225193.5
11672	ENSG00000165646.13	ENSG00000266019.1	ENSG00000272081.1
11673	ENSG00000165650.12	ENSG00000266017.1	ENSG00000117280.13
11674	ENSG00000165655.17	ENSG00000266016.3	ENSG00000233268.3
11675	ENSG00000165660.8	ENSG00000266015.1	ENSG00000064652.11
11676	ENSG00000165661.16	ENSG00000266014.1	ENSG00000233725.7
11677	ENSG00000165669.14	ENSG00000266013.1	ENSG00000238198.1
11678	ENSG00000165671.20	ENSG00000266012.1	ENSG00000280138.1
11679	ENSG00000165672.7	ENSG00000266010.2	ENSG00000267559.5
11680	ENSG00000165675.16	ENSG00000266009.1	ENSG00000269300.1
11681	ENSG00000165678.21	ENSG00000266006.1	ENSG00000137948.18
11682	ENSG00000165682.14	ENSG00000266003.1	ENSG00000169194.9
11683	ENSG00000165684.4	ENSG00000266002.1	ENSG00000021355.13
11684	ENSG00000165685.8	ENSG00000265996.1	ENSG00000234998.1
11685	ENSG00000165688.12	ENSG00000265995.1	ENSG00000187672.13
11686	ENSG00000165689.17	ENSG00000265994.2	ENSG00000282602.1
11687	ENSG00000165694.9	ENSG00000265993.1	ENSG00000226327.3
11688	ENSG00000165695.9	ENSG00000265992.1	ENSG00000242411.2
11689	ENSG00000165698.16	ENSG00000265991.1	ENSG00000159674.12
11690	ENSG00000165699.14	ENSG00000265987.1	ENSG00000161714.12
11691	ENSG00000165702.14	ENSG00000265986.1	ENSG00000236234.1
11692	ENSG00000165704.15	ENSG00000265984.1	ENSG00000235742.1
11693	ENSG00000165714.11	ENSG00000265982.1	ENSG00000283726.1
11694	ENSG00000165716.11	ENSG00000265981.1	ENSG00000229664.1
11695	ENSG00000165724.6	ENSG00000265980.1	ENSG00000211779.3
11696	ENSG00000165730.16	ENSG00000265976.1	ENSG00000276386.1
11697	ENSG00000165731.19	ENSG00000265975.1	ENSG00000204528.3
11698	ENSG00000165732.13	ENSG00000265973.1	ENSG00000225591.2
11699	ENSG00000165733.8	ENSG00000265972.5	ENSG00000253457.2
11700	ENSG00000165752.17	ENSG00000265971.1	ENSG00000175073.8
11701	ENSG00000165757.9	ENSG00000265967.1	ENSG00000189152.10
11702	ENSG00000165762.3	ENSG00000265965.1	ENSG00000088756.12
11703	ENSG00000165775.18	ENSG00000265964.1	ENSG00000242887.1
11704	ENSG00000165782.10	ENSG00000265962.1	ENSG00000204498.11
11705	ENSG00000165792.17	ENSG00000265961.1	ENSG00000279886.1
11706	ENSG00000165794.10	ENSG00000265957.1	ENSG00000214322.3
11707	ENSG00000165795.23	ENSG00000265956.1	ENSG00000251009.2
11708	ENSG00000165799.5	ENSG00000265954.1	ENSG00000249476.1
11709	ENSG00000165801.10	ENSG00000265948.1	ENSG00000125780.12
11710	ENSG00000165802.22	ENSG00000265946.1	ENSG00000176769.9

11711	ENSG00000165804.16	ENSG00000265944.1	ENSG00000122641.11
11712	ENSG00000165805.10	ENSG00000265943.1	ENSG00000203930.11
11713	ENSG00000165806.19	ENSG00000265942.2	ENSG00000165816.13
11714	ENSG00000165807.8	ENSG00000265936.1	ENSG00000256116.1
11715	ENSG00000165810.17	ENSG00000265935.1	ENSG00000200041.1
11716	ENSG00000165813.20	ENSG00000265934.1	ENSG00000227905.1
11717	ENSG00000165816.13	ENSG00000265933.5	ENSG00000252155.1
11718	ENSG00000165819.12	ENSG00000265932.1	ENSG00000226984.1
11719	ENSG00000165821.12	ENSG00000265929.1	ENSG00000231414.1
11720	ENSG00000165828.15	ENSG00000265927.1	ENSG00000237491.9
11721	ENSG00000165832.6	ENSG00000265924.1	ENSG00000197296.6
11722	ENSG00000165837.11	ENSG00000265919.1	ENSG00000258613.1
11723	ENSG00000165841.11	ENSG00000265918.2	ENSG00000257769.1
11724	ENSG00000165861.14	ENSG00000265917.1	ENSG00000261159.1
11725	ENSG00000165863.17	ENSG00000265916.1	ENSG00000177202.3
11726	ENSG00000165868.14	ENSG00000265912.1	ENSG00000140093.10
11727	ENSG00000165874.13	ENSG00000265908.1	ENSG00000258083.2
11728	ENSG00000165879.8	ENSG00000265907.1	ENSG00000028839.10
11729	ENSG00000165886.5	ENSG00000265905.2	ENSG00000213113.3
11730	ENSG00000165887.11	ENSG00000265902.1	ENSG00000259033.1
11731	ENSG00000165891.16	ENSG00000265897.2	ENSG00000278954.1
11732	ENSG00000165895.19	ENSG00000265894.2	ENSG00000285844.1
11733	ENSG00000165898.13	ENSG00000265892.2	ENSG00000260465.1
11734	ENSG00000165899.11	ENSG00000265889.1	ENSG00000162891.10
11735	ENSG00000165905.18	ENSG00000265888.1	ENSG00000284294.1
11736	ENSG00000165912.16	ENSG00000265885.2	ENSG00000235023.1
11737	ENSG00000165914.15	ENSG00000265883.1	ENSG00000214049.7
11738	ENSG00000165915.14	ENSG00000265882.2	ENSG00000239873.2
11739	ENSG00000165916.8	ENSG00000265881.1	ENSG00000283064.1
11740	ENSG00000165917.10	ENSG00000265879.1	ENSG00000164458.9
11741	ENSG00000165923.16	ENSG00000265878.2	ENSG00000014919.12
11742	ENSG00000165929.13	ENSG00000265874.1	ENSG00000223203.1
11743	ENSG00000165934.12	ENSG00000265873.1	ENSG00000272248.1
11744	ENSG00000165935.9	ENSG00000265872.1	ENSG00000182722.5
11745	ENSG00000165943.5	ENSG00000265871.1	ENSG00000131634.14
11746	ENSG00000165948.11	ENSG00000265867.1	ENSG00000271049.1
11747	ENSG00000165949.12	ENSG00000265863.1	ENSG00000229107.2
11748	ENSG00000165953.9	ENSG00000265861.1	ENSG00000253476.1
11749	ENSG00000165959.12	ENSG00000265859.1	ENSG00000258730.1
11750	ENSG00000165966.15	ENSG00000265855.1	ENSG00000153789.13
11751	ENSG00000165970.11	ENSG00000265853.1	ENSG00000255329.1
11752	ENSG00000165972.13	ENSG00000265850.1	ENSG00000225402.1
11753	ENSG00000165973.18	ENSG00000265848.1	ENSG00000236494.1
11754	ENSG00000165983.14	ENSG00000265847.1	ENSG00000128284.19
11755	ENSG00000165985.10	ENSG00000265846.1	ENSG00000283479.1
11756	ENSG00000165995.20	ENSG00000265845.2	ENSG00000224881.1
11757	ENSG00000165996.14	ENSG00000265844.1	ENSG00000272449.2
11758	ENSG00000165997.5	ENSG00000265843.2	ENSG00000176994.11
11759	ENSG00000166002.7	ENSG00000265841.1	ENSG00000257896.1
11760	ENSG00000166004.15	ENSG00000265840.1	ENSG00000244676.5
11761	ENSG00000166006.13	ENSG00000265836.1	ENSG00000254019.1
11762	ENSG00000166007.11	ENSG00000265833.1	ENSG00000254872.3
11763	ENSG00000166012.16	ENSG00000265831.1	ENSG00000238272.1

11764	ENSG00000166013.11	ENSG00000265829.1	ENSG00000043591.5
11765	ENSG00000166016.6	ENSG00000265828.1	ENSG00000229988.2
11766	ENSG00000166024.13	ENSG00000265822.1	ENSG00000235795.1
11767	ENSG00000166025.18	ENSG00000265820.1	ENSG00000277170.1
11768	ENSG00000166033.13	ENSG00000265818.1	ENSG00000163673.7
11769	ENSG00000166035.11	ENSG00000265817.3	ENSG00000233733.1
11770	ENSG00000166037.11	ENSG00000265815.1	ENSG00000231504.1
11771	ENSG00000166046.11	ENSG00000265814.2	ENSG00000260303.1
11772	ENSG00000166049.11	ENSG00000265813.2	ENSG00000244296.3
11773	ENSG00000166068.13	ENSG00000265810.1	ENSG00000137075.18
11774	ENSG00000166069.13	ENSG00000265808.3	ENSG00000163618.18
11775	ENSG00000166073.10	ENSG00000265806.1	ENSG00000227252.1
11776	ENSG00000166086.12	ENSG00000265802.2	ENSG00000268543.1
11777	ENSG00000166090.8	ENSG00000265801.1	ENSG00000258912.1
11778	ENSG00000166091.21	ENSG00000265800.1	ENSG00000226141.1
11779	ENSG00000166104.15	ENSG00000265799.1	ENSG00000184100.6
11780	ENSG00000166105.16	ENSG00000265798.6	ENSG00000279991.1
11781	ENSG00000166106.3	ENSG00000265794.5	ENSG00000226864.2
11782	ENSG00000166111.9	ENSG00000265793.1	ENSG00000260799.1
11783	ENSG00000166118.8	ENSG00000265791.1	ENSG00000267590.1
11784	ENSG00000166123.14	ENSG00000265790.1	ENSG00000049283.18
11785	ENSG00000166126.10	ENSG00000265789.1	ENSG00000185988.13
11786	ENSG00000166128.13	ENSG00000265788.2	ENSG00000113369.9
11787	ENSG00000166130.15	ENSG00000265787.2	ENSG00000118564.14
11788	ENSG00000166133.18	ENSG00000265786.1	ENSG00000171643.14
11789	ENSG00000166135.14	ENSG00000265784.1	ENSG00000228657.1
11790	ENSG00000166136.16	ENSG00000265781.1	ENSG00000226089.2
11791	ENSG00000166140.17	ENSG00000265778.2	ENSG00000279979.1
11792	ENSG00000166143.9	ENSG00000265777.2	ENSG00000241230.3
11793	ENSG00000166145.14	ENSG00000265776.1	ENSG00000235725.1
11794	ENSG00000166147.13	ENSG00000265775.1	ENSG00000154898.15
11795	ENSG00000166148.3	ENSG00000265768.1	ENSG00000204682.6
11796	ENSG00000166152.3	ENSG00000265766.3	ENSG00000173085.14
11797	ENSG00000166153.16	ENSG00000265763.4	ENSG00000160678.11
11798	ENSG00000166159.11	ENSG00000265758.1	ENSG00000266936.1
11799	ENSG00000166160.9	ENSG00000265753.2	ENSG00000231108.2
11800	ENSG00000166164.15	ENSG00000265752.2	ENSG00000157796.18
11801	ENSG00000166165.13	ENSG00000265751.1	ENSG00000197753.10
11802	ENSG00000166166.13	ENSG00000265750.1	ENSG00000259928.1
11803	ENSG00000166167.18	ENSG00000265749.5	ENSG00000256849.1
11804	ENSG00000166169.17	ENSG00000265746.1	ENSG00000273181.1
11805	ENSG00000166170.9	ENSG00000265745.2	ENSG00000274029.1
11806	ENSG00000166171.13	ENSG00000265744.1	ENSG00000246560.2
11807	ENSG00000166173.10	ENSG00000265743.1	ENSG00000257839.1
11808	ENSG00000166181.13	ENSG00000265740.2	ENSG00000249212.1
11809	ENSG00000166183.16	ENSG00000265739.1	ENSG00000265533.1
11810	ENSG00000166188.2	ENSG00000265737.1	ENSG00000260352.1
11811	ENSG00000166189.7	ENSG00000265735.2	ENSG00000254750.1
11812	ENSG00000166192.15	ENSG00000265734.1	ENSG00000223475.1
11813	ENSG00000166197.16	ENSG00000265733.1	ENSG00000205445.3
11814	ENSG00000166199.13	ENSG00000265728.1	ENSG00000005302.18
11815	ENSG00000166200.15	ENSG00000265727.2	ENSG00000272263.1
11816	ENSG00000166206.15	ENSG00000265725.1	ENSG00000238258.1

11817	ENSG00000166211.7	ENSG00000265724.1	ENSG00000261546.1
11818	ENSG00000166220.12	ENSG00000265719.1	ENSG00000238184.1
11819	ENSG00000166224.16	ENSG00000265717.1	ENSG00000235370.6
11820	ENSG00000166225.8	ENSG00000265713.1	ENSG00000260815.3
11821	ENSG00000166226.13	ENSG00000265712.1	ENSG00000182531.7
11822	ENSG00000166228.9	ENSG00000265706.1	ENSG00000172889.16
11823	ENSG00000166233.15	ENSG00000265702.1	ENSG00000205838.14
11824	ENSG00000166246.14	ENSG00000265699.1	ENSG00000186766.7
11825	ENSG00000166250.12	ENSG00000265698.1	ENSG00000269921.1
11826	ENSG00000166257.8	ENSG00000265697.1	ENSG00000270607.1
11827	ENSG00000166260.11	ENSG00000265694.1	ENSG00000214651.4
11828	ENSG00000166261.11	ENSG00000265693.1	ENSG00000163832.16
11829	ENSG00000166262.15	ENSG00000265692.1	ENSG00000227417.3
11830	ENSG00000166263.13	ENSG00000265691.1	ENSG00000233755.1
11831	ENSG00000166265.12	ENSG00000265690.7	ENSG00000152359.14
11832	ENSG00000166266.14	ENSG00000265689.1	ENSG00000250686.2
11833	ENSG00000166268.10	ENSG00000265688.2	ENSG00000252316.1
11834	ENSG00000166272.18	ENSG00000265684.2	ENSG00000106537.8
11835	ENSG00000166275.15	ENSG00000265683.1	ENSG00000256124.5
11836	ENSG00000166278.15	ENSG00000265682.1	ENSG00000186834.3
11837	ENSG00000166289.6	ENSG00000265681.7	ENSG00000253328.2
11838	ENSG00000166292.12	ENSG00000265678.1	ENSG00000260851.6
11839	ENSG00000166295.9	ENSG00000265675.2	ENSG00000119820.11
11840	ENSG00000166311.10	ENSG00000265673.1	ENSG00000204978.2
11841	ENSG00000166313.19	ENSG00000265671.1	ENSG00000267986.1
11842	ENSG00000166317.12	ENSG00000265670.1	ENSG00000280623.1
11843	ENSG00000166321.14	ENSG00000265669.1	ENSG00000224358.1
11844	ENSG00000166323.13	ENSG00000265666.1	ENSG00000232131.1
11845	ENSG00000166326.7	ENSG00000265664.1	ENSG00000258086.1
11846	ENSG00000166329.2	ENSG00000265660.1	ENSG00000258072.1
11847	ENSG00000166333.13	ENSG00000265658.6_PAR_Y	ENSG00000117318.9
11848	ENSG00000166337.10	ENSG00000265658.6	ENSG00000144057.15
11849	ENSG00000166340.17	ENSG00000265657.1	ENSG00000232875.1
11850	ENSG00000166341.8	ENSG00000265656.1	ENSG00000232864.1
11851	ENSG00000166342.19	ENSG00000265653.1	ENSG00000107537.14
11852	ENSG00000166343.10	ENSG00000265648.2	ENSG00000267040.6
11853	ENSG00000166347.19	ENSG00000265646.2	ENSG00000172497.9
11854	ENSG00000166348.18	ENSG00000265644.1	ENSG00000117069.15
11855	ENSG00000166349.9	ENSG00000265643.1	ENSG00000269040.1
11856	ENSG00000166351.10	ENSG00000265641.1	ENSG00000080839.11
11857	ENSG00000166352.16	ENSG00000265639.1	ENSG00000236689.1
11858	ENSG00000166359.10	ENSG00000265635.1	ENSG00000159289.6
11859	ENSG00000166363.5	ENSG00000265631.1	ENSG00000125977.7
11860	ENSG00000166368.2	ENSG00000265630.2	ENSG00000280311.1
11861	ENSG00000166377.20	ENSG00000265626.1	ENSG00000269404.7
11862	ENSG00000166387.13	ENSG00000265625.1	ENSG00000232027.2
11863	ENSG00000166391.15	ENSG00000265623.1	ENSG00000151445.16
11864	ENSG00000166394.15	ENSG00000265618.1	ENSG00000134057.15
11865	ENSG00000166396.13	ENSG00000265617.1	ENSG00000258408.1
11866	ENSG00000166398.12	ENSG00000265614.1	ENSG00000253794.1
11867	ENSG00000166401.14	ENSG00000265612.1	ENSG00000076258.10
11868	ENSG00000166402.8	ENSG00000265606.1	ENSG00000237352.3
11869	ENSG00000166405.15	ENSG00000265599.1	ENSG00000275586.1

11870	ENSG00000166407.14	ENSG00000265598.1	ENSG00000152558.15
11871	ENSG00000166408.4	ENSG00000265596.1	ENSG00000283980.1
11872	ENSG00000166411.14	ENSG00000265595.1	ENSG00000229835.2
11873	ENSG00000166415.15	ENSG00000265593.1	ENSG00000227968.1
11874	ENSG00000166426.8	ENSG00000265590.9	ENSG00000172955.17
11875	ENSG00000166428.13	ENSG00000265588.1	ENSG00000272420.1
11876	ENSG00000166432.15	ENSG00000265584.1	ENSG00000276136.1
11877	ENSG00000166435.15	ENSG00000265574.1	ENSG00000276550.4
11878	ENSG00000166436.16	ENSG00000265566.2	ENSG00000109424.4
11879	ENSG00000166439.6	ENSG00000265565.1	ENSG00000171481.4
11880	ENSG00000166441.13	ENSG00000265564.1	ENSG00000244153.1
11881	ENSG00000166444.19	ENSG00000265561.2	ENSG00000279995.1
11882	ENSG00000166446.15	ENSG00000265559.2	ENSG00000258345.1
11883	ENSG00000166448.14	ENSG00000265558.1	ENSG00000214548.17
11884	ENSG00000166451.13	ENSG00000265556.1	ENSG00000253015.1
11885	ENSG00000166452.11	ENSG00000265555.1	ENSG00000172006.11
11886	ENSG00000166454.10	ENSG00000265554.1	ENSG00000269125.1
11887	ENSG00000166455.13	ENSG00000265552.1	ENSG00000235298.1
11888	ENSG00000166471.11	ENSG00000265547.1	ENSG00000267313.7
11889	ENSG00000166473.17	ENSG00000265545.1	ENSG00000184304.15
11890	ENSG00000166477.13	ENSG00000265544.1	ENSG00000279340.1
11891	ENSG00000166478.10	ENSG00000265542.5	ENSG00000261168.1
11892	ENSG00000166479.10	ENSG00000265541.1	ENSG00000175697.10
11893	ENSG00000166482.11	ENSG00000265539.1	ENSG00000204644.9
11894	ENSG00000166483.11	ENSG00000265538.1	ENSG00000248926.1
11895	ENSG00000166484.20	ENSG00000265537.1	ENSG00000167658.16
11896	ENSG00000166492.9	ENSG00000265535.2	ENSG00000185594.5
11897	ENSG00000166501.13	ENSG00000265533.1	ENSG00000110881.11
11898	ENSG00000166503.9	ENSG00000265531.3	ENSG00000226155.1
11899	ENSG00000166507.17	ENSG00000265527.1	ENSG00000278743.1
11900	ENSG00000166508.17	ENSG00000265526.1	ENSG00000256538.1
11901	ENSG00000166509.11	ENSG00000265521.1	ENSG00000267560.1
11902	ENSG00000166510.14	ENSG00000265520.1	ENSG00000250410.1
11903	ENSG00000166523.7	ENSG00000265519.1	ENSG00000239899.3
11904	ENSG00000166526.17	ENSG00000265514.1	ENSG00000256799.1
11905	ENSG00000166527.8	ENSG00000265511.1	ENSG00000231395.1
11906	ENSG00000166529.14	ENSG00000265510.1	ENSG00000257403.2
11907	ENSG00000166530.8	ENSG00000265507.1	ENSG00000257252.5
11908	ENSG00000166532.16	ENSG00000265503.1	ENSG00000269889.1
11909	ENSG00000166535.20	ENSG00000265499.1	ENSG00000279143.1
11910	ENSG00000166546.14	ENSG00000265496.5	ENSG00000261459.1
11911	ENSG00000166548.15	ENSG00000265494.1	ENSG00000259900.5
11912	ENSG00000166557.13	ENSG00000265491.5	ENSG00000263015.1
11913	ENSG00000166558.10	ENSG00000265490.1	ENSG00000171509.16
11914	ENSG00000166562.9	ENSG00000265489.1	ENSG00000256566.1
11915	ENSG00000166569.8	ENSG00000265487.1	ENSG00000228437.5
11916	ENSG00000166573.5	ENSG00000265486.2	ENSG00000259367.1
11917	ENSG00000166575.17	ENSG00000265485.6	ENSG00000237310.1
11918	ENSG00000166578.10	ENSG00000265484.1	ENSG00000232218.1
11919	ENSG00000166579.15	ENSG00000265483.1	ENSG00000236305.1
11920	ENSG00000166582.10	ENSG00000265480.5	ENSG00000279605.1
11921	ENSG00000166589.13	ENSG00000265479.6	ENSG00000262402.1
11922	ENSG00000166592.12	ENSG00000265478.2	ENSG00000226644.5

11923	ENSG00000166595.12	ENSG00000265477.1	ENSG00000202260.1
11924	ENSG00000166596.15	ENSG00000265474.1	ENSG00000078487.17
11925	ENSG00000166598.15	ENSG00000265472.1	ENSG00000164379.7
11926	ENSG00000166603.5	ENSG00000265470.1	ENSG00000250295.6
11927	ENSG00000166619.14	ENSG00000265469.1	ENSG00000236430.1
11928	ENSG00000166634.6	ENSG00000265465.1	ENSG00000254933.1
11929	ENSG00000166664.13	ENSG00000265462.1	ENSG00000135828.11
11930	ENSG00000166669.13	ENSG00000265460.6	ENSG00000227269.1
11931	ENSG00000166670.10	ENSG00000265458.1	ENSG00000225506.2
11932	ENSG00000166676.16	ENSG00000265456.1	ENSG00000120800.5
11933	ENSG00000166681.13	ENSG00000265453.1	ENSG00000228280.1
11934	ENSG00000166682.12	ENSG00000265452.1	ENSG00000274492.1
11935	ENSG00000166685.12	ENSG00000265451.1	ENSG00000255126.1
11936	ENSG00000166689.16	ENSG00000265450.1	ENSG00000253133.1
11937	ENSG00000166693.7	ENSG00000265445.1	ENSG00000256940.1
11938	ENSG00000166704.11	ENSG00000265444.1	ENSG00000239269.1
11939	ENSG00000166707.11	ENSG00000265443.1	ENSG00000281808.1
11940	ENSG00000166710.19	ENSG00000265442.1	ENSG00000233380.1
11941	ENSG00000166716.10	ENSG00000265439.2	ENSG00000165072.10
11942	ENSG00000166734.20	ENSG00000265437.1	ENSG00000284914.1
11943	ENSG00000166736.11	ENSG00000265435.1	ENSG00000148175.12
11944	ENSG00000166741.7	ENSG00000265433.1	ENSG00000243954.3
11945	ENSG00000166743.9	ENSG00000265432.1	ENSG00000213109.4
11946	ENSG00000166747.12	ENSG00000265429.3	ENSG00000184185.10
11947	ENSG00000166750.10	ENSG00000265428.1	ENSG00000186910.4
11948	ENSG00000166762.18	ENSG00000265425.1	ENSG00000274833.1
11949	ENSG00000166763.7	ENSG00000265423.1	ENSG00000148358.19
11950	ENSG00000166770.10	ENSG00000265422.1	ENSG00000267764.1
11951	ENSG00000166780.11	ENSG00000265421.1	ENSG00000284640.1
11952	ENSG00000166783.22	ENSG00000265420.1	ENSG00000228198.3
11953	ENSG00000166787.3	ENSG00000265418.1	ENSG00000254675.1
11954	ENSG00000166788.10	ENSG00000265417.1	ENSG00000260075.1
11955	ENSG00000166793.11	ENSG00000265415.1	ENSG00000188393.8
11956	ENSG00000166794.4	ENSG00000265413.1	ENSG00000126790.12
11957	ENSG00000166796.12	ENSG00000265411.1	ENSG00000225037.1
11958	ENSG00000166797.11	ENSG00000265408.1	ENSG00000263300.1
11959	ENSG00000166800.9	ENSG00000265407.1	ENSG00000231970.1
11960	ENSG00000166801.15	ENSG00000265402.1	ENSG00000142178.8
11961	ENSG00000166803.13	ENSG00000265401.1	ENSG00000264217.2
11962	ENSG00000166813.15	ENSG00000265400.1	ENSG00000274769.1
11963	ENSG00000166816.14	ENSG00000265399.1	ENSG00000267463.1
11964	ENSG00000166819.12	ENSG00000265396.1	ENSG00000253631.1
11965	ENSG00000166821.9	ENSG00000265395.1	ENSG00000232001.2
11966	ENSG00000166822.13	ENSG00000265394.1	ENSG00000264570.1
11967	ENSG00000166823.5	ENSG00000265393.1	ENSG00000215159.3
11968	ENSG00000166825.14	ENSG00000265392.1	ENSG00000159377.11
11969	ENSG00000166828.3	ENSG00000265390.1	ENSG00000270726.6
11970	ENSG00000166831.9	ENSG00000265388.2	ENSG00000283633.1
11971	ENSG00000166833.21	ENSG00000265386.2	ENSG00000180090.5
11972	ENSG00000166839.17	ENSG00000265380.1	ENSG00000223797.5
11973	ENSG00000166840.13	ENSG00000265378.1	ENSG00000257622.1
11974	ENSG00000166845.15	ENSG00000265376.1	ENSG00000149231.14
11975	ENSG00000166847.10	ENSG00000265375.1	ENSG00000265142.8

11976	ENSG00000166848.6	ENSG00000265374.1	ENSG00000264653.1
11977	ENSG00000166851.15	ENSG00000265373.2	ENSG00000168137.15
11978	ENSG00000166855.9	ENSG00000265372.1	ENSG00000215003.4
11979	ENSG00000166856.3	ENSG00000265370.1	ENSG00000232946.1
11980	ENSG00000166860.3	ENSG00000265369.3	ENSG00000269904.2
11981	ENSG00000166862.6	ENSG00000265368.1	ENSG00000248898.1
11982	ENSG00000166863.12	ENSG00000265366.6	ENSG00000165828.15
11983	ENSG00000166866.13	ENSG00000265359.1	ENSG00000131771.14
11984	ENSG00000166869.3	ENSG00000265357.1	ENSG00000230305.2
11985	ENSG00000166881.10	ENSG00000265356.2	ENSG00000224220.1
11986	ENSG00000166884.2	ENSG00000265355.1	ENSG00000187288.10
11987	ENSG00000166886.13	ENSG00000265354.4	ENSG00000241411.1
11988	ENSG00000166887.15	ENSG00000265352.1	ENSG00000249055.1
11989	ENSG00000166888.12	ENSG00000265349.1	ENSG00000236105.1
11990	ENSG00000166889.14	ENSG00000265347.1	ENSG00000249649.1
11991	ENSG00000166896.8	ENSG00000265345.1	ENSG00000205426.10
11992	ENSG00000166897.15	ENSG00000265342.1	ENSG00000160188.10
11993	ENSG00000166900.17	ENSG00000265340.1	ENSG00000128298.17
11994	ENSG00000166902.5	ENSG00000265337.1	ENSG00000256720.1
11995	ENSG00000166908.18	ENSG00000265334.1	ENSG00000184208.10
11996	ENSG00000166912.17	ENSG00000265333.1	ENSG00000227473.1
11997	ENSG00000166913.13	ENSG00000265331.1	ENSG00000260704.1
11998	ENSG00000166917.10	ENSG00000265328.1	ENSG00000248703.2
11999	ENSG00000166920.12	ENSG00000265327.2	ENSG00000139233.7
12000	ENSG00000166922.8	ENSG00000265322.1	ENSG00000187601.4
12001	ENSG00000166923.11	ENSG00000265321.1	ENSG00000118217.6
12002	ENSG00000166924.9	ENSG00000265319.2	ENSG00000228288.6
12003	ENSG00000166925.9	ENSG00000265316.1	ENSG00000257718.1
12004	ENSG00000166926.8	ENSG00000265315.1	ENSG00000273258.1
12005	ENSG00000166927.13	ENSG00000265313.6	ENSG00000270402.1
12006	ENSG00000166928.10	ENSG00000265306.1	ENSG00000234358.1
12007	ENSG00000166930.7	ENSG00000265304.1	ENSG00000258571.1
12008	ENSG00000166938.13	ENSG00000265303.1	ENSG00000196611.5
12009	ENSG00000166946.14	ENSG00000265301.1	ENSG00000224001.2
12010	ENSG00000166947.13	ENSG00000265296.1	ENSG00000213925.3
12011	ENSG00000166948.9	ENSG00000265293.2	ENSG00000107957.16
12012	ENSG00000166949.16	ENSG00000265291.1	ENSG00000249685.1
12013	ENSG00000166959.8	ENSG00000265289.1	ENSG00000248216.1
12014	ENSG00000166960.16	ENSG00000265287.2	ENSG00000275395.5
12015	ENSG00000166961.14	ENSG00000265284.1	ENSG00000167183.3
12016	ENSG00000166963.13	ENSG00000265282.1	ENSG00000237243.1
12017	ENSG00000166965.12	ENSG00000265281.1	ENSG00000177558.3
12018	ENSG00000166971.17	ENSG00000265279.1	ENSG00000281909.1
12019	ENSG00000166974.12	ENSG00000265273.1	ENSG00000204514.10
12020	ENSG00000166979.13	ENSG00000265272.2	ENSG00000119782.14
12021	ENSG00000166984.11	ENSG00000265265.1	ENSG00000270236.1
12022	ENSG00000166986.15	ENSG00000265263.1	ENSG00000232386.9
12023	ENSG00000166987.15	ENSG00000265262.1	ENSG00000272128.1
12024	ENSG00000166997.8	ENSG00000265261.1	ENSG00000159714.11
12025	ENSG00000167004.13	ENSG00000265260.2	ENSG00000276538.1
12026	ENSG00000167005.14	ENSG00000265258.1	ENSG00000242719.3
12027	ENSG00000167011.9	ENSG00000265257.5	ENSG00000102468.10
12028	ENSG00000167014.11	ENSG00000265254.1	ENSG00000286237.1

12029	ENSG00000167034.10	ENSG00000265253.1	ENSG00000270989.1
12030	ENSG00000167037.18	ENSG00000265252.1	ENSG00000177535.8
12031	ENSG00000167046.4	ENSG00000265251.1	ENSG00000199990.1
12032	ENSG00000167065.13	ENSG00000265247.1	ENSG00000229983.1
12033	ENSG00000167074.15	ENSG00000265246.1	ENSG00000259216.2
12034	ENSG00000167077.13	ENSG00000265243.1	ENSG00000270487.1
12035	ENSG00000167080.8	ENSG00000265241.6	ENSG00000240370.6
12036	ENSG00000167081.17	ENSG00000265240.1	ENSG00000271803.1
12037	ENSG00000167083.7	ENSG00000265237.1	ENSG00000160870.14
12038	ENSG00000167085.11	ENSG00000265236.1	ENSG00000198715.13
12039	ENSG00000167088.11	ENSG00000265233.1	ENSG00000174417.2
12040	ENSG00000167094.16	ENSG00000265227.1	ENSG00000221857.7
12041	ENSG00000167098.12	ENSG00000265226.1	ENSG00000274419.6
12042	ENSG00000167100.14	ENSG00000265222.1	ENSG00000143493.13
12043	ENSG00000167103.12	ENSG00000265218.1	ENSG00000187498.16
12044	ENSG00000167104.11	ENSG00000265217.1	ENSG00000219392.1
12045	ENSG00000167105.8	ENSG00000265215.1	ENSG00000271818.1
12046	ENSG00000167106.12	ENSG00000265214.1	ENSG00000213593.10
12047	ENSG00000167107.12	ENSG00000265213.1	ENSG00000273623.1
12048	ENSG00000167110.17	ENSG00000265210.1	ENSG00000169554.20
12049	ENSG00000167112.10	ENSG00000265206.5	ENSG00000273449.1
12050	ENSG00000167113.11	ENSG00000265205.1	ENSG00000261203.1
12051	ENSG00000167114.13	ENSG00000265204.1	ENSG00000254481.1
12052	ENSG00000167117.9	ENSG00000265203.1	ENSG00000168634.5
12053	ENSG00000167118.10	ENSG00000265201.1	ENSG00000162804.14
12054	ENSG00000167123.19	ENSG00000265195.1	ENSG00000229093.1
12055	ENSG00000167130.18	ENSG00000265194.1	ENSG00000075673.11
12056	ENSG00000167131.17	ENSG00000265193.1	ENSG00000254665.1
12057	ENSG00000167136.7	ENSG00000265190.6	ENSG00000262006.1
12058	ENSG00000167139.9	ENSG00000265188.1	ENSG00000272175.1
12059	ENSG00000167157.11	ENSG00000265185.5	ENSG00000233668.1
12060	ENSG00000167165.19	ENSG00000265182.1	ENSG00000243104.1
12061	ENSG00000167173.19	ENSG00000265181.1	ENSG00000255052.4
12062	ENSG00000167178.16	ENSG00000265180.1	ENSG00000251379.1
12063	ENSG00000167182.15	ENSG00000265179.6	ENSG00000255292.8
12064	ENSG00000167183.3	ENSG00000265178.1	ENSG00000260733.1
12065	ENSG00000167186.11	ENSG00000265176.1	ENSG00000176834.14
12066	ENSG00000167191.12	ENSG00000265174.1	ENSG00000149380.12
12067	ENSG00000167193.8	ENSG00000265172.1	ENSG00000136603.14
12068	ENSG00000167194.7	ENSG00000265170.2	ENSG00000279095.1
12069	ENSG00000167195.7	ENSG00000265168.1	ENSG00000107897.19
12070	ENSG00000167196.14	ENSG00000265165.1	ENSG00000198818.10
12071	ENSG00000167202.11	ENSG00000265164.1	ENSG00000279599.1
12072	ENSG00000167207.13	ENSG00000265163.1	ENSG00000113262.16
12073	ENSG00000167208.15	ENSG00000265160.1	ENSG00000233485.1
12074	ENSG00000167210.17	ENSG00000265158.1	ENSG00000134899.20
12075	ENSG00000167216.16	ENSG00000265154.1	ENSG00000283036.2
12076	ENSG00000167220.12	ENSG00000265148.5	ENSG00000254186.2
12077	ENSG00000167232.14	ENSG00000265145.1	ENSG00000250896.1
12078	ENSG00000167244.20	ENSG00000265144.1	ENSG00000226995.8
12079	ENSG00000167257.11	ENSG00000265142.8	ENSG00000222017.1
12080	ENSG00000167258.13	ENSG00000265141.2	ENSG00000276755.1
12081	ENSG00000167261.14	ENSG00000265140.1	ENSG00000167771.6

12082	ENSG00000167264.18	ENSG00000265139.1	ENSG00000166477.13
12083	ENSG00000167272.10	ENSG00000265137.3	ENSG00000227685.1
12084	ENSG00000167280.16	ENSG00000265136.1	ENSG00000227714.1
12085	ENSG00000167281.19	ENSG00000265135.1	ENSG00000283154.2
12086	ENSG00000167283.8	ENSG00000265134.1	ENSG00000116141.15
12087	ENSG00000167286.9	ENSG00000265128.1	ENSG00000283088.1
12088	ENSG00000167291.16	ENSG00000265126.1	ENSG00000273450.1
12089	ENSG00000167302.10	ENSG00000265125.5	ENSG00000102172.16
12090	ENSG00000167306.20	ENSG00000265123.2	ENSG00000261266.2
12091	ENSG00000167311.13	ENSG00000265121.1	ENSG00000238035.8
12092	ENSG00000167315.18	ENSG00000265118.5	ENSG00000279500.1
12093	ENSG00000167323.11	ENSG00000265115.1	ENSG00000235785.1
12094	ENSG00000167325.15	ENSG00000265113.1	ENSG00000254192.1
12095	ENSG00000167332.8	ENSG00000265112.1	ENSG00000222047.8
12096	ENSG00000167333.13	ENSG00000265110.1	ENSG00000200534.1
12097	ENSG00000167346.7	ENSG00000265107.3	ENSG00000270356.1
12098	ENSG00000167355.8	ENSG00000265102.1	ENSG00000229878.1
12099	ENSG00000167359.8	ENSG00000265101.1	ENSG00000069399.15
12100	ENSG00000167360.6	ENSG00000265100.1	ENSG00000251520.1
12101	ENSG00000167363.14	ENSG00000265099.1	ENSG00000138660.11
12102	ENSG00000167371.20	ENSG00000265098.3	ENSG00000167447.12
12103	ENSG00000167377.18	ENSG00000265097.1	ENSG00000229999.6
12104	ENSG00000167378.8	ENSG00000265096.1	ENSG00000138018.18
12105	ENSG00000167380.16	ENSG00000265095.1	ENSG00000206120.11
12106	ENSG00000167384.10	ENSG00000265094.1	ENSG00000229847.8
12107	ENSG00000167390.8	ENSG00000265093.2	ENSG00000166394.15
12108	ENSG00000167393.17	ENSG00000265092.1	ENSG00000270482.1
12109	ENSG00000167393.17_PAR_Y	ENSG00000265091.5	ENSG00000249937.7
12110	ENSG00000167394.13	ENSG00000265089.1	ENSG00000160789.20
12111	ENSG00000167395.10	ENSG00000265083.1	ENSG00000259595.1
12112	ENSG00000167397.15	ENSG00000265078.2	ENSG00000066056.14
12113	ENSG00000167414.4	ENSG00000265075.1	ENSG00000145423.5
12114	ENSG00000167419.10	ENSG00000265073.1	ENSG00000129007.14
12115	ENSG00000167434.10	ENSG00000265069.1	ENSG00000283907.1
12116	ENSG00000167447.12	ENSG00000265064.1	ENSG00000277103.1
12117	ENSG00000167459.16	ENSG00000265061.1	ENSG00000238181.2
12118	ENSG00000167460.16	ENSG00000265060.1	ENSG00000285582.1
12119	ENSG00000167461.12	ENSG00000265057.1	ENSG00000001630.17
12120	ENSG00000167468.16	ENSG00000265056.1	ENSG00000226964.1
12121	ENSG00000167470.12	ENSG00000265055.1	ENSG00000235586.1
12122	ENSG00000167476.10	ENSG00000265052.2	ENSG00000138675.16
12123	ENSG00000167483.18	ENSG00000265046.1	ENSG00000250778.1
12124	ENSG00000167487.12	ENSG00000265043.1	ENSG00000108578.15
12125	ENSG00000167491.17	ENSG00000265041.1	ENSG00000239705.1
12126	ENSG00000167494.9	ENSG00000265038.1	ENSG00000139656.6
12127	ENSG00000167508.12	ENSG00000265033.2	ENSG00000260664.2
12128	ENSG00000167513.9	ENSG00000265031.1	ENSG00000228793.1
12129	ENSG00000167515.10	ENSG00000265028.1	ENSG00000224677.1
12130	ENSG00000167522.16	ENSG00000265019.1	ENSG00000256879.1
12131	ENSG00000167523.13	ENSG00000265015.1	ENSG00000234557.2
12132	ENSG00000167524.14	ENSG00000265014.1	ENSG00000254431.1
12133	ENSG00000167525.13	ENSG00000265010.1	ENSG00000144158.4
12134	ENSG00000167526.13	ENSG00000265008.1	ENSG00000248787.2

12135	ENSG00000167528.12	ENSG00000265007.1	ENSG00000163646.11
12136	ENSG00000167531.6	ENSG00000265005.1	ENSG00000256894.1
12137	ENSG00000167535.8	ENSG00000265003.1	ENSG00000180287.16
12138	ENSG00000167536.14	ENSG00000265000.1	ENSG00000156959.9
12139	ENSG00000167543.16	ENSG00000264999.1	ENSG00000231851.5
12140	ENSG00000167548.15	ENSG00000264997.1	ENSG00000264808.1
12141	ENSG00000167549.18	ENSG00000264994.1	ENSG00000236280.1
12142	ENSG00000167550.11	ENSG00000264990.1	ENSG00000260834.1
12143	ENSG00000167552.14	ENSG00000264986.1	ENSG00000251322.7
12144	ENSG00000167553.15	ENSG00000264985.3	ENSG00000225568.1
12145	ENSG00000167554.15	ENSG00000264984.1	ENSG00000273113.1
12146	ENSG00000167555.14	ENSG00000264982.1	ENSG00000257645.1
12147	ENSG00000167562.12	ENSG00000264981.1	ENSG00000284639.1
12148	ENSG00000167565.13	ENSG00000264979.1	ENSG00000264501.2
12149	ENSG00000167566.17	ENSG00000264978.2	ENSG00000283683.1
12150	ENSG00000167578.18	ENSG00000264975.1	ENSG00000153179.13
12151	ENSG00000167580.7	ENSG00000264974.1	ENSG00000257052.1
12152	ENSG00000167588.13	ENSG00000264971.1	ENSG00000186417.14
12153	ENSG00000167595.15	ENSG00000264970.2	ENSG00000160868.15
12154	ENSG00000167600.14	ENSG00000264968.1	ENSG00000241869.3
12155	ENSG00000167601.12	ENSG00000264966.1	ENSG00000259776.1
12156	ENSG00000167604.14	ENSG00000264964.1	ENSG00000279480.1
12157	ENSG00000167608.11	ENSG00000264963.2	ENSG00000228317.1
12158	ENSG00000167612.13	ENSG00000264961.1	ENSG00000276945.1
12159	ENSG00000167613.16	ENSG00000264958.2	ENSG00000235726.6
12160	ENSG00000167614.13	ENSG00000264956.1	ENSG00000200356.1
12161	ENSG00000167615.16	ENSG00000264954.1	ENSG00000225311.1
12162	ENSG00000167617.3	ENSG00000264947.1	ENSG00000280114.1
12163	ENSG00000167618.10	ENSG00000264943.1	ENSG00000285609.1
12164	ENSG00000167619.12	ENSG00000264941.1	ENSG00000218857.1
12165	ENSG00000167625.10	ENSG00000264940.4	ENSG00000243396.1
12166	ENSG00000167632.15	ENSG00000264937.1	ENSG00000268970.1
12167	ENSG00000167633.18	ENSG00000264934.1	ENSG00000267179.1
12168	ENSG00000167634.12	ENSG00000264933.2	ENSG00000265107.3
12169	ENSG00000167635.11	ENSG00000264932.3	ENSG00000225883.2
12170	ENSG00000167637.17	ENSG00000264931.1	ENSG00000100034.14
12171	ENSG00000167641.11	ENSG00000264930.1	ENSG00000249593.6
12172	ENSG00000167642.13	ENSG00000264926.1	ENSG00000274387.1
12173	ENSG00000167644.12	ENSG00000264924.1	ENSG00000169126.15
12174	ENSG00000167645.17	ENSG00000264922.1	ENSG00000206557.6
12175	ENSG00000167646.13	ENSG00000264920.1	ENSG00000140798.16
12176	ENSG00000167653.5	ENSG00000264916.2	ENSG00000253767.2
12177	ENSG00000167654.18	ENSG00000264914.1	ENSG00000254307.2
12178	ENSG00000167656.5	ENSG00000264911.1	ENSG00000149599.15
12179	ENSG00000167657.14	ENSG00000264910.2	ENSG00000272150.5
12180	ENSG00000167658.16	ENSG00000264907.1	ENSG00000160113.5
12181	ENSG00000167664.8	ENSG00000264906.1	ENSG00000137474.21
12182	ENSG00000167670.16	ENSG00000264902.1	ENSG00000166147.13
12183	ENSG00000167671.12	ENSG00000264901.1	ENSG00000101958.14
12184	ENSG00000167674.15	ENSG00000264897.1	ENSG00000256678.1
12185	ENSG00000167676.4	ENSG00000264895.1	ENSG00000271437.1
12186	ENSG00000167680.16	ENSG00000264892.1	ENSG00000284772.1
12187	ENSG00000167685.15	ENSG00000264885.1	ENSG00000277967.1

12188	ENSG00000167693.17	ENSG00000264881.1	ENSG00000136048.14
12189	ENSG00000167695.15	ENSG00000264880.1	ENSG00000265158.1
12190	ENSG00000167699.13	ENSG00000264879.2	ENSG00000253102.1
12191	ENSG00000167700.9	ENSG00000264876.1	ENSG00000258967.1
12192	ENSG00000167701.14	ENSG00000264874.1	ENSG00000265254.1
12193	ENSG00000167702.12	ENSG00000264869.1	ENSG00000152076.18
12194	ENSG00000167703.14	ENSG00000264864.1	ENSG00000255057.1
12195	ENSG00000167705.12	ENSG00000264862.2	ENSG00000198821.10
12196	ENSG00000167711.13	ENSG00000264860.1	ENSG00000140092.14
12197	ENSG00000167716.18	ENSG00000264859.5	ENSG00000125631.7
12198	ENSG00000167720.13	ENSG00000264857.1	ENSG00000243312.3
12199	ENSG00000167721.10	ENSG00000264853.1	ENSG00000239946.1
12200	ENSG00000167723.14	ENSG00000264850.1	ENSG00000238057.9
12201	ENSG00000167733.13	ENSG00000264845.2	ENSG00000221603.1
12202	ENSG00000167740.9	ENSG00000264843.1	ENSG00000203690.12
12203	ENSG00000167741.10	ENSG00000264840.1	ENSG00000271010.1
12204	ENSG00000167747.14	ENSG00000264837.1	ENSG00000178531.6
12205	ENSG00000167748.11	ENSG00000264834.1	ENSG00000261701.7
12206	ENSG00000167749.11	ENSG00000264833.1	ENSG00000285761.1
12207	ENSG00000167751.13	ENSG00000264829.1	ENSG00000124275.14
12208	ENSG00000167754.13	ENSG00000264825.1	ENSG00000244176.1
12209	ENSG00000167755.15	ENSG00000264824.1	ENSG00000198734.10
12210	ENSG00000167757.14	ENSG00000264823.3	ENSG00000259010.1
12211	ENSG00000167759.13	ENSG00000264814.1	ENSG00000172663.9
12212	ENSG00000167766.18	ENSG00000264813.6	ENSG00000228414.6
12213	ENSG00000167767.14	ENSG00000264812.1	ENSG00000284693.1
12214	ENSG00000167768.4	ENSG00000264810.1	ENSG00000266036.1
12215	ENSG00000167769.4	ENSG00000264809.2	ENSG00000157851.17
12216	ENSG00000167770.11	ENSG00000264808.1	ENSG00000198832.10
12217	ENSG00000167771.6	ENSG00000264803.1	ENSG00000215237.6
12218	ENSG00000167772.12	ENSG00000264802.1	ENSG00000214114.9
12219	ENSG00000167774.2	ENSG00000264801.1	ENSG00000255213.1
12220	ENSG00000167775.11	ENSG00000264800.1	ENSG00000256995.7
12221	ENSG00000167778.9	ENSG00000264796.1	ENSG00000278588.1
12222	ENSG00000167779.9	ENSG00000264793.1	ENSG00000230601.6
12223	ENSG00000167780.12	ENSG00000264792.1	ENSG00000270775.1
12224	ENSG00000167785.9	ENSG00000264791.1	ENSG00000277602.1
12225	ENSG00000167791.13	ENSG00000264790.1	ENSG00000180251.4
12226	ENSG00000167792.12	ENSG00000264788.1	ENSG00000276573.1
12227	ENSG00000167797.7	ENSG00000264785.1	ENSG00000256806.5
12228	ENSG00000167798.17	ENSG00000264781.1	ENSG00000250651.1
12229	ENSG00000167799.10	ENSG00000264775.1	ENSG00000214941.8
12230	ENSG00000167800.9	ENSG00000264773.1	ENSG00000260619.1
12231	ENSG00000167807.15	ENSG00000264772.6	ENSG00000104055.15
12232	ENSG00000167815.12	ENSG00000264769.1	ENSG00000178662.16
12233	ENSG00000167825.3	ENSG00000264767.2	ENSG00000136546.15
12234	ENSG00000167840.13	ENSG00000264765.1	ENSG00000235237.1
12235	ENSG00000167842.15	ENSG00000264764.1	ENSG00000267484.1
12236	ENSG00000167850.3	ENSG00000264763.1	ENSG00000142677.4
12237	ENSG00000167851.14	ENSG00000264760.1	ENSG00000095627.9
12238	ENSG00000167858.13	ENSG00000264757.1	ENSG00000285727.1
12239	ENSG00000167861.16	ENSG00000264755.1	ENSG00000200913.1
12240	ENSG00000167862.10	ENSG00000264754.1	ENSG00000257434.1

12241	ENSG00000167863.12	ENSG00000264750.1	ENSG00000181092.10
12242	ENSG00000167874.6	ENSG00000264747.1	ENSG00000007174.18
12243	ENSG00000167880.7	ENSG00000264745.1	ENSG00000232599.1
12244	ENSG00000167881.15	ENSG00000264744.1	ENSG00000249092.1
12245	ENSG00000167889.12	ENSG00000264743.2	ENSG00000236526.1
12246	ENSG00000167895.14	ENSG00000264741.1	ENSG00000267628.1
12247	ENSG00000167900.12	ENSG00000264739.1	ENSG00000164692.17
12248	ENSG00000167904.15	ENSG00000264737.1	ENSG00000223760.4
12249	ENSG00000167910.4	ENSG00000264736.1	ENSG00000188215.10
12250	ENSG00000167912.5	ENSG00000264735.1	ENSG00000096063.16
12251	ENSG00000167914.11	ENSG00000264734.1	ENSG00000230186.3
12252	ENSG00000167916.5	ENSG00000264733.1	ENSG00000213988.11
12253	ENSG00000167920.8	ENSG00000264732.1	ENSG00000259793.1
12254	ENSG00000167925.16	ENSG00000264729.1	ENSG00000131233.9
12255	ENSG00000167930.16	ENSG00000264727.1	ENSG00000090339.9
12256	ENSG00000167941.3	ENSG00000264725.1	ENSG00000138757.14
12257	ENSG00000167962.14	ENSG00000264722.1	ENSG00000074660.16
12258	ENSG00000167964.12	ENSG00000264720.3	ENSG00000197503.4
12259	ENSG00000167965.18	ENSG00000264717.5	ENSG00000269919.1
12260	ENSG00000167967.16	ENSG00000264714.1	ENSG00000145362.18
12261	ENSG00000167968.13	ENSG00000264712.3	ENSG00000240048.1
12262	ENSG00000167969.13	ENSG00000264707.1	ENSG00000167741.10
12263	ENSG00000167971.15	ENSG00000264706.2	ENSG00000234678.2
12264	ENSG00000167972.14	ENSG00000264705.1	ENSG00000237510.7
12265	ENSG00000167977.9	ENSG00000264703.1	ENSG00000230712.2
12266	ENSG00000167978.17	ENSG00000264701.1	ENSG00000279687.1
12267	ENSG00000167981.7	ENSG00000264699.1	ENSG00000185043.11
12268	ENSG00000167984.18	ENSG00000264698.1	ENSG00000217769.4
12269	ENSG00000167986.13	ENSG00000264697.1	ENSG00000041988.15
12270	ENSG00000167987.11	ENSG00000264695.1	ENSG00000267688.1
12271	ENSG00000167992.13	ENSG00000264693.1	ENSG00000175130.7
12272	ENSG00000167994.12	ENSG00000264689.1	ENSG00000273312.2
12273	ENSG00000167995.16	ENSG00000264685.1	ENSG00000188828.11
12274	ENSG00000167996.16	ENSG00000264684.1	ENSG00000230392.1
12275	ENSG00000168000.14	ENSG00000264678.1	ENSG00000259365.1
12276	ENSG00000168002.12	ENSG00000264676.2	ENSG00000278274.1
12277	ENSG00000168003.16	ENSG00000264675.1	ENSG00000165156.15
12278	ENSG00000168004.9	ENSG00000264673.1	ENSG00000169604.19
12279	ENSG00000168005.9	ENSG00000264672.5	ENSG00000196860.8
12280	ENSG00000168010.11	ENSG00000264668.2	ENSG00000265185.5
12281	ENSG00000168014.16	ENSG00000264666.1	ENSG00000204592.9
12282	ENSG00000168016.14	ENSG00000264663.1	ENSG00000248559.1
12283	ENSG00000168026.18	ENSG00000264662.1	ENSG00000263535.2
12284	ENSG00000168028.14	ENSG00000264661.3	ENSG00000277290.1
12285	ENSG00000168032.9	ENSG00000264660.2	ENSG00000249699.1
12286	ENSG00000168036.18	ENSG00000264659.1	ENSG00000256312.1
12287	ENSG00000168038.11	ENSG00000264658.1	ENSG00000174950.11
12288	ENSG00000168040.4	ENSG00000264657.1	ENSG00000149635.2
12289	ENSG00000168056.15	ENSG00000264655.1	ENSG00000233421.4
12290	ENSG00000168060.16	ENSG00000264653.1	ENSG00000168509.20
12291	ENSG00000168061.15	ENSG00000264647.1	ENSG00000226284.1
12292	ENSG00000168062.10	ENSG00000264644.1	ENSG00000166478.10
12293	ENSG00000168065.15	ENSG00000264643.1	ENSG00000224315.2

12294	ENSG00000168066.20	ENSG00000264638.1	ENSG00000110047.18
12295	ENSG00000168067.12	ENSG00000264635.1	ENSG00000173431.2
12296	ENSG00000168070.12	ENSG00000264634.1	ENSG00000096093.16
12297	ENSG00000168071.22	ENSG00000264633.1	ENSG00000279049.1
12298	ENSG00000168077.14	ENSG00000264630.5	ENSG00000186522.15
12299	ENSG00000168078.10	ENSG00000264623.3	ENSG00000253228.1
12300	ENSG00000168079.17	ENSG00000264622.1	ENSG00000275437.1
12301	ENSG00000168081.9	ENSG00000264621.1	ENSG00000147059.8
12302	ENSG00000168090.10	ENSG00000264618.2	ENSG00000254274.1
12303	ENSG00000168092.14	ENSG00000264617.1	ENSG00000139537.11
12304	ENSG00000168096.14	ENSG00000264616.1	ENSG00000231386.1
12305	ENSG00000168101.14	ENSG00000264615.2	ENSG00000254230.1
12306	ENSG00000168116.14	ENSG00000264614.1	ENSG00000167703.14
12307	ENSG00000168118.11	ENSG00000264613.2	ENSG00000275097.1
12308	ENSG00000168122.4	ENSG00000264610.1	ENSG00000249028.2
12309	ENSG00000168124.2	ENSG00000264608.1	ENSG00000070371.16
12310	ENSG00000168126.3	ENSG00000264607.1	ENSG00000272275.1
12311	ENSG00000168129.8	ENSG00000264603.1	ENSG00000279296.1
12312	ENSG00000168131.4	ENSG00000264598.1	ENSG00000112769.19
12313	ENSG00000168135.4	ENSG00000264596.1	ENSG00000282886.1
12314	ENSG00000168137.15	ENSG00000264595.1	ENSG00000214142.2
12315	ENSG00000168140.5	ENSG00000264592.2	ENSG00000285802.1
12316	ENSG00000168143.9	ENSG00000264589.3	ENSG00000268628.2
12317	ENSG00000168148.3	ENSG00000264587.5	ENSG00000238269.8
12318	ENSG00000168152.13	ENSG00000264585.1	ENSG00000175029.17
12319	ENSG00000168158.3	ENSG00000264583.1	ENSG00000115561.16
12320	ENSG00000168159.12	ENSG00000264582.2	ENSG00000174680.9
12321	ENSG00000168172.9	ENSG00000264580.1	ENSG00000171703.17
12322	ENSG00000168175.15	ENSG00000264578.1	ENSG00000213642.3
12323	ENSG00000168209.5	ENSG00000264577.1	ENSG00000252633.1
12324	ENSG00000168214.20	ENSG00000264575.1	ENSG00000203685.10
12325	ENSG00000168216.12	ENSG00000264574.1	ENSG00000197472.15
12326	ENSG00000168228.15	ENSG00000264573.2	ENSG00000279414.1
12327	ENSG00000168229.3	ENSG00000264572.1	ENSG00000233537.1
12328	ENSG00000168234.13	ENSG00000264571.1	ENSG00000241280.1
12329	ENSG00000168237.18	ENSG00000264570.1	ENSG00000180433.5
12330	ENSG00000168243.11	ENSG00000264569.1	ENSG00000215277.8
12331	ENSG00000168246.6	ENSG00000264566.3	ENSG00000146576.13
12332	ENSG00000168255.20	ENSG00000264564.1	ENSG00000219773.1
12333	ENSG00000168256.18	ENSG00000264563.1	ENSG00000188677.14
12334	ENSG00000168259.15	ENSG00000264559.1	ENSG00000279013.1
12335	ENSG00000168263.9	ENSG00000264558.1	ENSG00000170689.10
12336	ENSG00000168264.10	ENSG00000264554.2	ENSG00000214249.3
12337	ENSG00000168267.6	ENSG00000264553.1	ENSG00000175471.19
12338	ENSG00000168268.11	ENSG00000264549.1	ENSG00000274818.1
12339	ENSG00000168269.10	ENSG00000264548.1	ENSG00000173198.6
12340	ENSG00000168273.8	ENSG00000264546.1	ENSG00000213172.3
12341	ENSG00000168275.15	ENSG00000264545.1	ENSG00000151276.23
12342	ENSG00000168280.17	ENSG00000264540.2	ENSG00000238288.1
12343	ENSG00000168282.5	ENSG00000264539.1	ENSG00000214760.3
12344	ENSG00000168283.14	ENSG00000264538.6	ENSG00000277501.1
12345	ENSG00000168286.2	ENSG00000264536.1	ENSG00000177459.11
12346	ENSG00000168288.13	ENSG00000264534.3	ENSG00000017427.16

12347	ENSG00000168291.13	ENSG00000264530.2	ENSG00000237133.1
12348	ENSG00000168297.15	ENSG00000264529.1	ENSG00000260978.1
12349	ENSG00000168298.6	ENSG00000264525.1	ENSG00000205682.2
12350	ENSG00000168300.14	ENSG00000264522.6	ENSG00000259384.7
12351	ENSG00000168301.13	ENSG00000264520.1	ENSG00000226970.2
12352	ENSG00000168303.8	ENSG00000264519.2	ENSG00000261886.1
12353	ENSG00000168306.13	ENSG00000264515.5	ENSG00000257921.6
12354	ENSG00000168309.18	ENSG00000264514.1	ENSG00000228507.1
12355	ENSG00000168310.11	ENSG00000264513.1	ENSG00000198829.6
12356	ENSG00000168314.17	ENSG00000264512.2	ENSG00000226816.3
12357	ENSG00000168329.13	ENSG00000264511.1	ENSG00000279942.1
12358	ENSG00000168333.13	ENSG00000264503.1	ENSG00000230606.10
12359	ENSG00000168334.9	ENSG00000264501.2	ENSG00000121297.7
12360	ENSG00000168348.4	ENSG00000264500.1	ENSG00000226510.1
12361	ENSG00000168350.8	ENSG00000264497.1	ENSG00000241281.1
12362	ENSG00000168356.11	ENSG00000264494.1	ENSG00000231102.1
12363	ENSG00000168367.10	ENSG00000264493.1	ENSG00000283125.1
12364	ENSG00000168374.11	ENSG00000264491.1	ENSG00000236754.5
12365	ENSG00000168385.18	ENSG00000264490.3	ENSG00000279913.1
12366	ENSG00000168386.18	ENSG00000264488.1	ENSG00000204283.3
12367	ENSG00000168389.17	ENSG00000264486.1	ENSG00000273554.4
12368	ENSG00000168393.13	ENSG00000264484.2	ENSG00000274717.1
12369	ENSG00000168394.11	ENSG00000264483.1	ENSG00000179388.9
12370	ENSG00000168395.15	ENSG00000264482.1	ENSG00000163347.6
12371	ENSG00000168397.17	ENSG00000264480.1	ENSG00000270624.1
12372	ENSG00000168398.6	ENSG00000264477.1	ENSG00000251586.1
12373	ENSG00000168404.13	ENSG00000264475.1	ENSG00000257607.1
12374	ENSG00000168405.17	ENSG00000264474.1	ENSG00000067842.17
12375	ENSG00000168411.14	ENSG00000264472.4	ENSG00000103021.9
12376	ENSG00000168412.6	ENSG00000264470.1	ENSG00000229781.1
12377	ENSG00000168418.7	ENSG00000264468.1	ENSG00000285517.1
12378	ENSG00000168421.13	ENSG00000264464.1	ENSG00000263711.5
12379	ENSG00000168427.9	ENSG00000264462.1	ENSG00000258122.1
12380	ENSG00000168434.13	ENSG00000264458.1	ENSG00000164756.12
12381	ENSG00000168438.15	ENSG00000264456.1	ENSG00000273821.1
12382	ENSG00000168439.16	ENSG00000264452.1	ENSG00000159346.13
12383	ENSG00000168447.11	ENSG00000264451.1	ENSG00000188958.9
12384	ENSG00000168453.15	ENSG00000264449.5	ENSG00000167862.10
12385	ENSG00000168454.11	ENSG00000264448.5	ENSG00000198862.14
12386	ENSG00000168461.13	ENSG00000264444.1	ENSG00000286063.1
12387	ENSG00000168476.12	ENSG00000264443.1	ENSG00000107611.15
12388	ENSG00000168477.19	ENSG00000264438.2	ENSG00000189366.9
12389	ENSG00000168481.9	ENSG00000264435.1	ENSG00000189089.5
12390	ENSG00000168484.12	ENSG00000264434.2	ENSG00000170153.11
12391	ENSG00000168487.19	ENSG00000264431.1	ENSG00000135845.10
12392	ENSG00000168488.18	ENSG00000264429.1	ENSG00000236172.6
12393	ENSG00000168490.14	ENSG00000264426.1	ENSG00000228170.1
12394	ENSG00000168491.10	ENSG00000264425.1	ENSG00000229816.1
12395	ENSG00000168495.13	ENSG00000264424.1	ENSG00000233393.1
12396	ENSG00000168496.4	ENSG00000264423.2	ENSG00000140057.9
12397	ENSG00000168497.5	ENSG00000264422.1	ENSG00000164400.6
12398	ENSG00000168502.17	ENSG00000264421.1	ENSG00000232346.1
12399	ENSG00000168505.7	ENSG00000264419.1	ENSG00000165757.9

12400	ENSG00000168509.20	ENSG00000264408.1	ENSG00000255002.1
12401	ENSG00000168515.3	ENSG00000264406.1	ENSG00000241217.3
12402	ENSG00000168517.10	ENSG00000264405.1	ENSG00000106211.9
12403	ENSG00000168522.13	ENSG00000264404.2	ENSG00000269713.7
12404	ENSG00000168530.16	ENSG00000264402.1	ENSG00000256340.8
12405	ENSG00000168538.16	ENSG00000264400.2	ENSG00000197176.7
12406	ENSG00000168539.4	ENSG00000264399.1	ENSG00000164304.16
12407	ENSG00000168542.14	ENSG00000264397.1	ENSG00000085276.18
12408	ENSG00000168546.11	ENSG00000264395.1	ENSG00000258491.2
12409	ENSG00000168556.7	ENSG00000264391.2	ENSG00000285728.1
12410	ENSG00000168564.6	ENSG00000264390.1	ENSG00000204556.4
12411	ENSG00000168566.13	ENSG00000264388.1	ENSG00000257175.2
12412	ENSG00000168569.7	ENSG00000264387.1	ENSG00000225548.6
12413	ENSG00000168575.10	ENSG00000264386.1	ENSG00000272851.1
12414	ENSG00000168582.4	ENSG00000264384.2	ENSG00000260296.1
12415	ENSG00000168589.15	ENSG00000264379.1	ENSG00000278212.2
12416	ENSG00000168591.16	ENSG00000264377.1	ENSG00000240047.2
12417	ENSG00000168594.15	ENSG00000264373.1	ENSG00000279080.1
12418	ENSG00000168610.14	ENSG00000264371.1	ENSG00000243504.1
12419	ENSG00000168612.4	ENSG00000264370.1	ENSG00000274600.1
12420	ENSG00000168615.12	ENSG00000264365.1	ENSG00000131013.3
12421	ENSG00000168619.16	ENSG00000264364.3	ENSG00000178752.16
12422	ENSG00000168621.15	ENSG00000264359.1	ENSG00000227945.1
12423	ENSG00000168630.3	ENSG00000264358.1	ENSG00000138079.13
12424	ENSG00000168631.12	ENSG00000264357.1	ENSG00000243265.1
12425	ENSG00000168634.5	ENSG00000264354.1	ENSG00000275064.1
12426	ENSG00000168646.13	ENSG00000264352.2	ENSG00000166897.15
12427	ENSG00000168653.11	ENSG00000264350.1	ENSG00000284308.1
12428	ENSG00000168658.18	ENSG00000264349.1	ENSG00000262332.1
12429	ENSG00000168661.14	ENSG00000264346.1	ENSG00000118017.3
12430	ENSG00000168671.10	ENSG00000264345.1	ENSG00000206066.3
12431	ENSG00000168672.4	ENSG00000264342.3	ENSG00000229917.2
12432	ENSG00000168675.18	ENSG00000264341.1	ENSG00000251596.1
12433	ENSG00000168676.11	ENSG00000264340.1	ENSG00000183246.6
12434	ENSG00000168679.18	ENSG00000264339.1	ENSG00000130856.16
12435	ENSG00000168685.15	ENSG00000264334.1	ENSG00000253154.2
12436	ENSG00000168701.19	ENSG00000264330.1	ENSG00000129480.13
12437	ENSG00000168702.17	ENSG00000264324.1	ENSG00000271947.1
12438	ENSG00000168703.5	ENSG00000264322.2	ENSG00000213082.3
12439	ENSG00000168710.18	ENSG00000264319.1	ENSG00000259899.1
12440	ENSG00000168724.16	ENSG00000264317.2	ENSG00000125089.17
12441	ENSG00000168734.14	ENSG00000264316.1	ENSG00000157800.18
12442	ENSG00000168743.12	ENSG00000264315.1	ENSG00000102970.10
12443	ENSG00000168746.8	ENSG00000264314.1	ENSG00000275144.1
12444	ENSG00000168748.14	ENSG00000264313.2	ENSG00000108370.17
12445	ENSG00000168754.15	ENSG00000264311.1	ENSG00000223773.7
12446	ENSG00000168757.12	ENSG00000264309.1	ENSG00000240375.1
12447	ENSG00000168758.11	ENSG00000264304.1	ENSG00000270714.1
12448	ENSG00000168763.16	ENSG00000264301.1	ENSG00000161405.17
12449	ENSG00000168765.17	ENSG00000264300.1	ENSG00000229183.8
12450	ENSG00000168769.13	ENSG00000264297.2	ENSG00000219747.1
12451	ENSG00000168772.11	ENSG00000264296.1	ENSG00000273167.1
12452	ENSG00000168778.12	ENSG00000264295.1	ENSG00000115274.15

12453	ENSG00000168781.22	ENSG00000264294.1	ENSG00000255899.1
12454	ENSG00000168785.8	ENSG00000264293.2	ENSG00000174990.6
12455	ENSG00000168792.5	ENSG00000264292.1	ENSG00000103647.12
12456	ENSG00000168795.5	ENSG00000264290.1	ENSG00000233387.1
12457	ENSG00000168802.13	ENSG00000264289.1	ENSG00000284727.1
12458	ENSG00000168803.15	ENSG00000264281.3	ENSG00000154258.17
12459	ENSG00000168806.7	ENSG00000264279.1	ENSG00000237640.1
12460	ENSG00000168807.16	ENSG00000264278.1	ENSG00000278009.1
12461	ENSG00000168811.7	ENSG00000264275.1	ENSG00000149547.15
12462	ENSG00000168813.17	ENSG00000264274.1	ENSG00000268307.1
12463	ENSG00000168818.10	ENSG00000264273.1	ENSG00000159251.7
12464	ENSG00000168824.14	ENSG00000264272.1	ENSG00000269352.1
12465	ENSG00000168826.16	ENSG00000264271.2	ENSG00000207751.3
12466	ENSG00000168827.14	ENSG00000264270.1	ENSG00000263603.1
12467	ENSG00000168828.5	ENSG00000264269.1	ENSG00000258984.5
12468	ENSG00000168830.8	ENSG00000264268.1	ENSG00000275297.1
12469	ENSG00000168843.14	ENSG00000264266.1	ENSG00000147145.12
12470	ENSG00000168852.13	ENSG00000264265.1	ENSG00000234883.5
12471	ENSG00000168872.17	ENSG00000264262.1	ENSG00000261399.1
12472	ENSG00000168874.13	ENSG00000264260.1	ENSG00000188342.12
12473	ENSG00000168875.2	ENSG00000264257.1	ENSG00000155906.18
12474	ENSG00000168876.9	ENSG00000264254.1	ENSG00000102962.5
12475	ENSG00000168878.16	ENSG00000264251.2	ENSG00000164035.10
12476	ENSG00000168883.20	ENSG00000264250.2	ENSG00000231233.1
12477	ENSG00000168884.14	ENSG00000264249.1	ENSG00000177303.10
12478	ENSG00000168887.11	ENSG00000264247.1	ENSG00000260740.2
12479	ENSG00000168890.14	ENSG00000264246.1	ENSG00000101190.12
12480	ENSG00000168894.9	ENSG00000264245.1	ENSG00000213411.2
12481	ENSG00000168899.5	ENSG00000264243.1	ENSG00000105374.10
12482	ENSG00000168903.8	ENSG00000264240.1	ENSG00000116962.15
12483	ENSG00000168904.15	ENSG00000264236.1	ENSG00000155011.9
12484	ENSG00000168906.13	ENSG00000264235.5	ENSG00000184428.12
12485	ENSG00000168907.13	ENSG00000264233.1	ENSG00000206203.5
12486	ENSG00000168913.7	ENSG00000264232.1	ENSG00000221938.5
12487	ENSG00000168916.15	ENSG00000264230.9	ENSG00000249550.6
12488	ENSG00000168917.9	ENSG00000264229.1	ENSG00000163121.10
12489	ENSG00000168918.14	ENSG00000264226.1	ENSG00000256101.5
12490	ENSG00000168924.15	ENSG00000264217.2	ENSG00000251366.1
12491	ENSG00000168925.11	ENSG00000264215.1	ENSG00000189001.11
12492	ENSG00000168928.13	ENSG00000264212.1	ENSG00000178821.13
12493	ENSG00000168930.13	ENSG00000264211.1	ENSG00000152578.13
12494	ENSG00000168936.11	ENSG00000264210.1	ENSG00000235651.1
12495	ENSG00000168938.5	ENSG00000264201.1	ENSG00000245149.3
12496	ENSG00000168939.11	ENSG00000264200.1	ENSG00000229390.1
12497	ENSG00000168939.11_PAR_Y	ENSG00000264196.1	ENSG00000267124.2
12498	ENSG00000168944.15	ENSG00000264193.1	ENSG00000196090.12
12499	ENSG00000168952.15	ENSG00000264192.1	ENSG00000213337.9
12500	ENSG00000168955.3	ENSG00000264189.1	ENSG00000227929.4
12501	ENSG00000168958.19	ENSG00000264188.1	ENSG00000262202.4
12502	ENSG00000168959.14	ENSG00000264187.1	ENSG00000211655.3
12503	ENSG00000168961.17	ENSG00000264186.1	ENSG00000162520.14
12504	ENSG00000168967.14	ENSG00000264179.1	ENSG00000138829.12
12505	ENSG00000168970.22	ENSG00000264177.1	ENSG00000272418.1

12506	ENSG00000168992.4	ENSG00000264176.1	ENSG00000237917.1
12507	ENSG00000168993.15	ENSG00000264175.1	ENSG00000279504.1
12508	ENSG00000168994.13	ENSG00000264174.1	ENSG00000231636.1
12509	ENSG00000168995.13	ENSG00000264172.1	ENSG00000280266.1
12510	ENSG00000169006.7	ENSG00000264171.1	ENSG00000249620.1
12511	ENSG00000169016.17	ENSG00000264169.2	ENSG00000118276.11
12512	ENSG00000169018.6	ENSG00000264168.2	ENSG00000233543.1
12513	ENSG00000169019.11	ENSG00000264167.1	ENSG00000258895.1
12514	ENSG00000169020.10	ENSG00000264164.1	ENSG00000205424.1
12515	ENSG00000169021.6	ENSG00000264163.1	ENSG00000179335.18
12516	ENSG00000169026.12	ENSG00000264160.1	ENSG00000008128.23
12517	ENSG00000169031.19	ENSG00000264158.1	ENSG00000277382.1
12518	ENSG00000169032.9	ENSG00000264157.3	ENSG00000273989.1
12519	ENSG00000169035.12	ENSG00000264151.5	ENSG00000267328.1
12520	ENSG00000169040.14	ENSG00000264150.1	ENSG00000230837.1
12521	ENSG00000169045.17	ENSG00000264149.1	ENSG00000236290.1
12522	ENSG00000169047.5	ENSG00000264148.1	ENSG00000244157.1
12523	ENSG00000169057.22	ENSG00000264145.2	ENSG00000277825.1
12524	ENSG00000169059.12	ENSG00000264141.1	ENSG00000223930.6
12525	ENSG00000169062.15	ENSG00000264139.1	ENSG00000255552.7
12526	ENSG00000169064.12	ENSG00000264138.1	ENSG00000144285.19
12527	ENSG00000169067.4	ENSG00000264136.1	ENSG00000234319.1
12528	ENSG00000169071.15	ENSG00000264131.1	ENSG00000169575.4
12529	ENSG00000169075.7	ENSG00000264128.2	ENSG00000233321.1
12530	ENSG00000169083.16	ENSG00000264127.1	ENSG00000284776.1
12531	ENSG00000169084.13	ENSG00000264125.1	ENSG00000286001.1
12532	ENSG00000169084.13_PAR_Y	ENSG00000264119.1	ENSG00000220585.4
12533	ENSG00000169085.13	ENSG00000264116.5	ENSG00000185880.13
12534	ENSG00000169087.11	ENSG00000264115.1	ENSG00000137203.12
12535	ENSG00000169093.16	ENSG00000264113.2	ENSG00000228695.10
12536	ENSG00000169093.16_PAR_Y	ENSG00000264112.1	ENSG00000286019.1
12537	ENSG00000169100.14	ENSG00000264110.1	ENSG00000273473.1
12538	ENSG00000169100.14_PAR_Y	ENSG00000264108.1	ENSG00000272631.1
12539	ENSG00000169105.7	ENSG00000264107.1	ENSG00000222028.4
12540	ENSG00000169116.11	ENSG00000264105.1	ENSG00000259397.3
12541	ENSG00000169118.18	ENSG00000264104.1	ENSG00000285677.1
12542	ENSG00000169122.11	ENSG00000264102.1	ENSG00000217330.1
12543	ENSG00000169126.15	ENSG00000264101.1	ENSG00000218274.2
12544	ENSG00000169129.15	ENSG00000264099.1	ENSG00000223638.3
12545	ENSG00000169131.13	ENSG00000264093.1	ENSG00000269444.1
12546	ENSG00000169136.11	ENSG00000264092.2	ENSG00000268592.3
12547	ENSG00000169139.12	ENSG00000264090.1	ENSG00000177873.13
12548	ENSG00000169154.7	ENSG00000264089.1	ENSG00000251056.1
12549	ENSG00000169155.10	ENSG00000264084.1	ENSG00000186510.12
12550	ENSG00000169164.7	ENSG00000264083.1	ENSG00000241859.7
12551	ENSG00000169169.14	ENSG00000264080.1	ENSG00000231907.2
12552	ENSG00000169174.10	ENSG00000264078.1	ENSG00000258802.1
12553	ENSG00000169180.11	ENSG00000264075.1	ENSG00000279840.1
12554	ENSG00000169181.13	ENSG00000264073.1	ENSG00000221406.1
12555	ENSG00000169184.6	ENSG00000264072.1	ENSG00000214821.4
12556	ENSG00000169188.5	ENSG00000264071.2	ENSG00000136810.13
12557	ENSG00000169189.17	ENSG00000264070.1	ENSG00000211625.2
12558	ENSG00000169193.12	ENSG00000264069.3	ENSG00000256433.2

12559	ENSG00000169194.9	ENSG00000264067.1	ENSG00000205334.2
12560	ENSG00000169203.16	ENSG00000264066.6	ENSG00000167333.13
12561	ENSG00000169208.2	ENSG00000264063.1	ENSG00000286138.1
12562	ENSG00000169213.7	ENSG00000264061.1	ENSG00000260144.1
12563	ENSG00000169214.4	ENSG00000264060.1	ENSG00000225314.1
12564	ENSG00000169217.9	ENSG00000264058.2	ENSG00000236760.1
12565	ENSG00000169218.14	ENSG00000264057.1	ENSG00000163421.9
12566	ENSG00000169220.18	ENSG00000264056.1	ENSG00000205746.9
12567	ENSG00000169221.14	ENSG00000264054.1	ENSG00000167363.14
12568	ENSG00000169223.15	ENSG00000264050.1	ENSG00000230835.1
12569	ENSG00000169224.13	ENSG00000264049.1	ENSG00000223452.3
12570	ENSG00000169228.14	ENSG00000264047.2	ENSG00000207496.1
12571	ENSG00000169230.10	ENSG00000264044.1	ENSG00000176302.12
12572	ENSG00000169231.13	ENSG00000264041.2	ENSG00000005471.17
12573	ENSG00000169239.13	ENSG00000264040.1	ENSG00000100889.11
12574	ENSG00000169241.19	ENSG00000264037.1	ENSG00000248991.1
12575	ENSG00000169242.12	ENSG00000264036.2	ENSG00000242405.2
12576	ENSG00000169245.6	ENSG00000264032.1	ENSG00000214263.2
12577	ENSG00000169246.16	ENSG00000264031.1	ENSG00000258957.1
12578	ENSG00000169247.12	ENSG00000264030.2	ENSG00000220378.3
12579	ENSG00000169248.12	ENSG00000264029.1	ENSG00000230585.2
12580	ENSG00000169249.12	ENSG00000264028.2	ENSG00000261586.2
12581	ENSG00000169251.12	ENSG00000264026.1	ENSG00000285572.1
12582	ENSG00000169252.5	ENSG00000264024.2	ENSG00000232491.1
12583	ENSG00000169253.3	ENSG00000264023.1	ENSG00000275186.1
12584	ENSG00000169255.15	ENSG00000264019.1	ENSG00000237076.1
12585	ENSG00000169258.7	ENSG00000264017.2	ENSG00000171136.6
12586	ENSG00000169271.3	ENSG00000264016.2	ENSG00000250138.4
12587	ENSG00000169282.17	ENSG00000264015.1	ENSG00000264916.2
12588	ENSG00000169288.18	ENSG00000264014.1	ENSG00000285578.1
12589	ENSG00000169291.10	ENSG00000264013.1	ENSG00000222427.1
12590	ENSG00000169297.7	ENSG00000264012.1	ENSG00000234222.6
12591	ENSG00000169299.14	ENSG00000264010.1	ENSG00000103966.11
12592	ENSG00000169302.16	ENSG00000264007.1	ENSG00000279353.1
12593	ENSG00000169306.9	ENSG00000264006.8	ENSG00000176046.8
12594	ENSG00000169313.9	ENSG00000264005.1	ENSG00000140563.15
12595	ENSG00000169314.14	ENSG00000264004.1	ENSG00000119203.14
12596	ENSG00000169325.10	ENSG00000264002.2	ENSG00000272077.1
12597	ENSG00000169327.5	ENSG00000264000.1	ENSG00000267160.1
12598	ENSG00000169330.8	ENSG00000263999.2	ENSG00000274209.4
12599	ENSG00000169340.9	ENSG00000263993.2	ENSG00000164920.9
12600	ENSG00000169344.15	ENSG00000263990.1	ENSG00000243988.1
12601	ENSG00000169347.16	ENSG00000263989.2	ENSG00000240244.3
12602	ENSG00000169359.15	ENSG00000263988.2	ENSG00000179097.5
12603	ENSG00000169371.13	ENSG00000263987.1	ENSG00000206199.10
12604	ENSG00000169372.13	ENSG00000263986.1	ENSG00000165905.18
12605	ENSG00000169375.15	ENSG00000263982.1	ENSG00000259784.1
12606	ENSG00000169379.15	ENSG00000263981.1	ENSG00000273487.1
12607	ENSG00000169385.3	ENSG00000263979.1	ENSG00000226051.7
12608	ENSG00000169393.9	ENSG00000263978.1	ENSG00000213358.3
12609	ENSG00000169397.3	ENSG00000263976.1	ENSG00000283828.1
12610	ENSG00000169398.19	ENSG00000263974.2	ENSG00000143458.12
12611	ENSG00000169402.15	ENSG00000263973.1	ENSG00000224481.2

12612	ENSG00000169403.12	ENSG00000263972.1	ENSG00000176692.7
12613	ENSG00000169410.9	ENSG00000263970.1	ENSG00000158050.5
12614	ENSG00000169413.3	ENSG00000263969.2	ENSG00000213931.7
12615	ENSG00000169418.10	ENSG00000263968.2	ENSG00000131152.4
12616	ENSG00000169427.8	ENSG00000263967.1	ENSG00000101892.12
12617	ENSG00000169429.11	ENSG00000263964.1	ENSG00000157191.20
12618	ENSG00000169432.15	ENSG00000263963.1	ENSG00000125775.15
12619	ENSG00000169435.14	ENSG00000263961.8	ENSG00000261449.1
12620	ENSG00000169436.17	ENSG00000263958.1	ENSG00000243069.7
12621	ENSG00000169439.12	ENSG00000263956.6	ENSG00000211753.4
12622	ENSG00000169442.9	ENSG00000263955.2	ENSG00000276851.1
12623	ENSG00000169446.5	ENSG00000263952.5	ENSG00000275385.1
12624	ENSG00000169469.9	ENSG00000263946.1	ENSG00000233893.2
12625	ENSG00000169474.4	ENSG00000263945.1	ENSG00000188596.11
12626	ENSG00000169484.3	ENSG00000263944.2	ENSG00000180539.7
12627	ENSG00000169488.6	ENSG00000263941.2	ENSG00000230022.1
12628	ENSG00000169490.17	ENSG00000263940.2	ENSG00000198967.4
12629	ENSG00000169495.5	ENSG00000263938.1	ENSG00000228887.3
12630	ENSG00000169499.15	ENSG00000263935.1	ENSG00000228737.2
12631	ENSG00000169504.15	ENSG00000263934.4	ENSG00000139160.13
12632	ENSG00000169507.9	ENSG00000263932.1	ENSG00000261839.1
12633	ENSG00000169508.7	ENSG00000263926.1	ENSG00000198952.8
12634	ENSG00000169509.6	ENSG00000263924.1	ENSG00000224579.1
12635	ENSG00000169515.7	ENSG00000263923.1	ENSG00000226330.1
12636	ENSG00000169519.21	ENSG00000263918.1	ENSG00000267397.1
12637	ENSG00000169548.3	ENSG00000263917.1	ENSG00000270393.1
12638	ENSG00000169550.14	ENSG00000263916.1	ENSG00000259309.2
12639	ENSG00000169551.12	ENSG00000263914.1	ENSG00000224607.4
12640	ENSG00000169554.20	ENSG00000263909.1	ENSG00000264269.1
12641	ENSG00000169562.10	ENSG00000263908.1	ENSG00000119681.12
12642	ENSG00000169564.6	ENSG00000263905.2	ENSG00000284138.1
12643	ENSG00000169567.11	ENSG00000263904.1	ENSG00000175318.12
12644	ENSG00000169570.10	ENSG00000263897.1	ENSG00000267340.1
12645	ENSG00000169575.4	ENSG00000263895.1	ENSG00000131475.7
12646	ENSG00000169583.12	ENSG00000263894.1	ENSG00000177212.4
12647	ENSG00000169592.15	ENSG00000263893.2	ENSG00000220506.2
12648	ENSG00000169594.13	ENSG00000263892.3	ENSG00000081014.11
12649	ENSG00000169598.15	ENSG00000263890.1	ENSG00000274719.1
12650	ENSG00000169599.12	ENSG00000263887.7	ENSG00000240793.1
12651	ENSG00000169604.19	ENSG00000263885.1	ENSG00000213770.3
12652	ENSG00000169605.5	ENSG00000263884.1	ENSG00000229205.4
12653	ENSG00000169607.13	ENSG00000263883.1	ENSG00000269915.1
12654	ENSG00000169609.14	ENSG00000263882.2	ENSG00000228022.5
12655	ENSG00000169612.4	ENSG00000263881.1	ENSG00000144847.12
12656	ENSG00000169618.6	ENSG00000263878.1	ENSG00000253899.1
12657	ENSG00000169621.10	ENSG00000263874.2	ENSG00000229230.3
12658	ENSG00000169627.8	ENSG00000263873.1	ENSG00000230417.11
12659	ENSG00000169629.11	ENSG00000263872.1	ENSG00000107796.13
12660	ENSG00000169635.10	ENSG00000263870.1	ENSG00000168056.15
12661	ENSG00000169641.13	ENSG00000263863.1	ENSG00000149418.11
12662	ENSG00000169660.16	ENSG00000263862.1	ENSG00000239198.1
12663	ENSG00000169662.8	ENSG00000263861.1	ENSG00000229827.1
12664	ENSG00000169668.11	ENSG00000263860.1	ENSG00000230306.1

12665	ENSG00000169676.5	ENSG00000263859.1	ENSG00000090975.12
12666	ENSG00000169679.14	ENSG00000263858.1	ENSG00000163788.14
12667	ENSG00000169682.18	ENSG00000263857.1	ENSG00000213740.2
12668	ENSG00000169683.8	ENSG00000263849.1	ENSG00000266501.1
12669	ENSG00000169684.13	ENSG00000263847.1	ENSG00000225465.8
12670	ENSG00000169688.11	ENSG00000263846.1	ENSG00000171388.12
12671	ENSG00000169689.15	ENSG00000263843.1	ENSG00000135324.6
12672	ENSG00000169692.13	ENSG00000263841.2	ENSG00000269304.1
12673	ENSG00000169696.16	ENSG00000263838.1	ENSG00000168032.9
12674	ENSG00000169704.4	ENSG00000263834.1	ENSG00000264311.1
12675	ENSG00000169710.9	ENSG00000263831.1	ENSG00000132297.11
12676	ENSG00000169714.16	ENSG00000263829.1	ENSG00000213234.4
12677	ENSG00000169715.14	ENSG00000263828.1	ENSG00000284654.1
12678	ENSG00000169717.6	ENSG00000263826.1	ENSG00000267568.6
12679	ENSG00000169718.17	ENSG00000263823.1	ENSG00000267491.1
12680	ENSG00000169727.12	ENSG00000263821.1	ENSG00000231848.1
12681	ENSG00000169733.12	ENSG00000263818.5	ENSG00000185837.3
12682	ENSG00000169738.7	ENSG00000263816.1	ENSG00000137440.4
12683	ENSG00000169740.14	ENSG00000263815.2	ENSG00000177306.4
12684	ENSG00000169744.13	ENSG00000263813.1	ENSG00000246145.1
12685	ENSG00000169750.9	ENSG00000263811.1	ENSG00000272582.1
12686	ENSG00000169752.17	ENSG00000263809.1	ENSG00000255050.1
12687	ENSG00000169756.16	ENSG00000263800.1	ENSG00000245888.6
12688	ENSG00000169758.13	ENSG00000263798.2	ENSG00000261793.1
12689	ENSG00000169760.17	ENSG00000263797.1	ENSG00000176402.5
12690	ENSG00000169762.17	ENSG00000263795.1	ENSG00000228432.1
12691	ENSG00000169763.14	ENSG00000263794.2	ENSG00000258466.5
12692	ENSG00000169764.15	ENSG00000263793.1	ENSG00000212710.4
12693	ENSG00000169777.6	ENSG00000263790.1	ENSG00000248400.2
12694	ENSG00000169783.12	ENSG00000263788.1	ENSG00000256673.1
12695	ENSG00000169789.10	ENSG00000263787.1	ENSG00000260246.1
12696	ENSG00000169800.13	ENSG00000263786.1	ENSG00000284873.1
12697	ENSG00000169807.10	ENSG00000263785.1	ENSG00000121210.16
12698	ENSG00000169811.8	ENSG00000263783.1	ENSG00000268536.1
12699	ENSG00000169813.16	ENSG00000263781.3	ENSG00000169429.11
12700	ENSG00000169814.13	ENSG00000263776.1	ENSG00000227355.2
12701	ENSG00000169826.8	ENSG00000263772.1	ENSG00000198807.12
12702	ENSG00000169836.5	ENSG00000263766.5	ENSG00000124780.14
12703	ENSG00000169840.4	ENSG00000263765.5	ENSG00000155659.15
12704	ENSG00000169849.8	ENSG00000263764.1	ENSG00000243753.5
12705	ENSG00000169851.15	ENSG00000263763.1	ENSG00000177455.13
12706	ENSG00000169855.20	ENSG00000263762.1	ENSG00000265912.1
12707	ENSG00000169856.8	ENSG00000263761.2	ENSG00000131746.13
12708	ENSG00000169857.8	ENSG00000263756.1	ENSG00000124657.1
12709	ENSG00000169860.7	ENSG00000263755.2	ENSG00000249310.2
12710	ENSG00000169862.19	ENSG00000263752.1	ENSG00000280392.1
12711	ENSG00000169871.13	ENSG00000263750.1	ENSG00000235641.4
12712	ENSG00000169876.13	ENSG00000263749.2	ENSG00000272220.1
12713	ENSG00000169877.10	ENSG00000263748.1	ENSG00000228376.3
12714	ENSG00000169884.14	ENSG00000263746.1	ENSG00000272678.1
12715	ENSG00000169885.10	ENSG00000263745.6	ENSG00000260235.1
12716	ENSG00000169891.18	ENSG00000263744.1	ENSG00000009844.16
12717	ENSG00000169894.18	ENSG00000263742.1	ENSG00000233622.3

12718	ENSG00000169895.6	ENSG00000263741.1	ENSG00000215297.3
12719	ENSG00000169896.17	ENSG00000263740.2	ENSG00000124256.15
12720	ENSG00000169900.7	ENSG00000263735.1	ENSG00000174460.4
12721	ENSG00000169902.15	ENSG00000263734.1	ENSG00000064313.12
12722	ENSG00000169903.6	ENSG00000263733.1	ENSG00000165646.13
12723	ENSG00000169905.12	ENSG00000263730.1	ENSG00000187536.4
12724	ENSG00000169906.5	ENSG00000263729.1	ENSG00000242968.1
12725	ENSG00000169908.12	ENSG00000263727.1	ENSG00000164366.3
12726	ENSG00000169914.6	ENSG00000263725.1	ENSG00000231426.6
12727	ENSG00000169918.9	ENSG00000263724.1	ENSG00000284433.1
12728	ENSG00000169919.17	ENSG00000263723.1	ENSG00000204920.11
12729	ENSG00000169925.16	ENSG00000263720.1	ENSG00000181803.3
12730	ENSG00000169926.11	ENSG00000263717.1	ENSG00000282059.1
12731	ENSG00000169933.13	ENSG00000263716.1	ENSG00000264404.2
12732	ENSG00000169946.14	ENSG00000263715.7	ENSG00000237804.1
12733	ENSG00000169951.9	ENSG00000263712.1	ENSG00000275793.1
12734	ENSG00000169953.12	ENSG00000263711.5	ENSG00000237350.1
12735	ENSG00000169955.7	ENSG00000263709.1	ENSG00000215717.6
12736	ENSG00000169957.10	ENSG00000263708.2	ENSG00000011007.12
12737	ENSG00000169962.5	ENSG00000263707.1	ENSG00000214961.2
12738	ENSG00000169964.8	ENSG00000263705.1	ENSG00000125266.7
12739	ENSG00000169967.16	ENSG00000263698.1	ENSG00000276727.1
12740	ENSG00000169972.12	ENSG00000263697.1	ENSG00000186715.11
12741	ENSG00000169976.6	ENSG00000263693.1	ENSG00000258885.1
12742	ENSG00000169981.11	ENSG00000263690.2	ENSG00000143158.11
12743	ENSG00000169989.2	ENSG00000263688.1	ENSG00000172987.12
12744	ENSG00000169991.11	ENSG00000263684.1	ENSG00000253692.3
12745	ENSG00000169992.10	ENSG00000263683.1	ENSG00000286105.1
12746	ENSG00000169994.18	ENSG00000263682.1	ENSG00000115363.14
12747	ENSG00000170004.16	ENSG00000263681.1	ENSG00000010404.18
12748	ENSG00000170006.12	ENSG00000263680.2	ENSG00000230530.1
12749	ENSG00000170011.14	ENSG00000263677.1	ENSG00000253861.1
12750	ENSG00000170017.12	ENSG00000263676.1	ENSG00000107518.18
12751	ENSG00000170027.7	ENSG00000263675.1	ENSG00000179300.3
12752	ENSG00000170035.16	ENSG00000263674.1	ENSG00000285521.1
12753	ENSG00000170037.13	ENSG00000263672.2	ENSG00000240086.6
12754	ENSG00000170043.11	ENSG00000263670.1	ENSG00000236086.4
12755	ENSG00000170044.8	ENSG00000263669.2	ENSG00000238231.2
12756	ENSG00000170049.9	ENSG00000263667.2	ENSG00000204193.10
12757	ENSG00000170054.14	ENSG00000263666.1	ENSG00000059377.17
12758	ENSG00000170074.19	ENSG00000263657.1	ENSG00000227379.2
12759	ENSG00000170075.9	ENSG00000263655.1	ENSG00000243027.3
12760	ENSG00000170085.18	ENSG00000263652.1	ENSG00000160161.9
12761	ENSG00000170088.14	ENSG00000263649.1	ENSG00000255028.5
12762	ENSG00000170089.15	ENSG00000263648.1	ENSG00000182168.15
12763	ENSG00000170091.11	ENSG00000263647.1	ENSG00000275143.1
12764	ENSG00000170092.14	ENSG00000263644.1	ENSG00000089163.4
12765	ENSG00000170099.6	ENSG00000263643.1	ENSG00000204186.9
12766	ENSG00000170100.13	ENSG00000263642.1	ENSG00000256682.2
12767	ENSG00000170113.16	ENSG00000263641.1	ENSG00000197471.12
12768	ENSG00000170122.5	ENSG00000263639.6	ENSG00000116106.12
12769	ENSG00000170128.4	ENSG00000263637.1	ENSG00000248374.1
12770	ENSG00000170142.11	ENSG00000263635.1	ENSG00000235724.9

12771	ENSG00000170144.20	ENSG00000263634.1	ENSG00000230399.1
12772	ENSG00000170145.5	ENSG00000263631.1	ENSG00000114650.20
12773	ENSG00000170152.5	ENSG00000263629.1	ENSG00000177398.18
12774	ENSG00000170153.11	ENSG00000263628.1	ENSG00000279452.1
12775	ENSG00000170160.17	ENSG00000263627.1	ENSG00000236739.3
12776	ENSG00000170161.6	ENSG00000263624.1	ENSG00000259005.1
12777	ENSG00000170162.14	ENSG00000263622.1	ENSG00000249209.2
12778	ENSG00000170165.5	ENSG00000263620.1	ENSG00000234737.1
12779	ENSG00000170166.6	ENSG00000263618.5	ENSG00000161057.12
12780	ENSG00000170175.11	ENSG00000263616.2	ENSG00000000457.14
12781	ENSG00000170178.6	ENSG00000263615.1	ENSG00000250261.1
12782	ENSG00000170180.22	ENSG00000263613.1	ENSG00000144837.9
12783	ENSG00000170185.9	ENSG00000263612.1	ENSG00000254987.1
12784	ENSG00000170190.16	ENSG00000263611.1	ENSG00000174358.16
12785	ENSG00000170191.5	ENSG00000263609.1	ENSG00000234293.1
12786	ENSG00000170209.4	ENSG00000263608.2	ENSG00000239998.6
12787	ENSG00000170214.5	ENSG00000263606.1	ENSG00000137727.12
12788	ENSG00000170222.11	ENSG00000263604.1	ENSG00000102128.8
12789	ENSG00000170231.15	ENSG00000263603.1	ENSG00000232268.6
12790	ENSG00000170234.13	ENSG00000263602.1	ENSG00000180628.15
12791	ENSG00000170236.14	ENSG00000263600.1	ENSG00000260101.1
12792	ENSG00000170242.17	ENSG00000263597.1	ENSG00000152292.17
12793	ENSG00000170248.15	ENSG00000263595.2	ENSG00000124664.11
12794	ENSG00000170255.7	ENSG00000263594.1	ENSG00000215032.2
12795	ENSG00000170260.8	ENSG00000263593.1	ENSG00000222004.7
12796	ENSG00000170262.12	ENSG00000263588.1	ENSG00000238266.2
12797	ENSG00000170264.13	ENSG00000263586.1	ENSG00000276027.1
12798	ENSG00000170265.12	ENSG00000263585.1	ENSG00000119446.14
12799	ENSG00000170266.16	ENSG00000263584.1	ENSG00000164265.9
12800	ENSG00000170270.5	ENSG00000263583.1	ENSG00000236813.1
12801	ENSG00000170271.11	ENSG00000263581.1	ENSG00000178055.11
12802	ENSG00000170275.14	ENSG00000263575.1	ENSG00000171777.16
12803	ENSG00000170276.6	ENSG00000263574.1	ENSG00000095917.14
12804	ENSG00000170279.3	ENSG00000263573.1	ENSG00000226530.1
12805	ENSG00000170289.12	ENSG00000263572.2	ENSG00000233728.1
12806	ENSG00000170290.4	ENSG00000263571.2	ENSG00000265987.1
12807	ENSG00000170291.14	ENSG00000263567.1	ENSG00000228918.3
12808	ENSG00000170293.9	ENSG00000263563.6	ENSG00000254157.1
12809	ENSG00000170296.10	ENSG00000263561.1	ENSG00000055208.19
12810	ENSG00000170298.15	ENSG00000263558.2	ENSG00000255379.1
12811	ENSG00000170310.15	ENSG00000263556.2	ENSG00000227879.2
12812	ENSG00000170312.15	ENSG00000263551.5	ENSG00000230508.1
12813	ENSG00000170315.13	ENSG00000263547.1	ENSG00000008256.16
12814	ENSG00000170322.14	ENSG00000263540.1	ENSG00000117322.17
12815	ENSG00000170323.8	ENSG00000263537.2	ENSG00000177590.7
12816	ENSG00000170324.21	ENSG00000263535.2	ENSG00000275527.1
12817	ENSG00000170325.14	ENSG00000263533.1	ENSG00000258648.1
12818	ENSG00000170340.11	ENSG00000263531.1	ENSG00000120805.14
12819	ENSG00000170345.10	ENSG00000263529.2	ENSG00000271967.1
12820	ENSG00000170348.9	ENSG00000263528.8	ENSG00000220392.1
12821	ENSG00000170356.9	ENSG00000263527.1	ENSG00000232408.1
12822	ENSG00000170364.12	ENSG00000263526.1	ENSG00000260876.5
12823	ENSG00000170365.10	ENSG00000263520.1	ENSG00000111275.13

12824	ENSG00000170367.5	ENSG00000263515.1	ENSG00000107960.11
12825	ENSG00000170369.4	ENSG00000263514.1	ENSG00000284879.1
12826	ENSG00000170370.12	ENSG00000263513.5	ENSG00000175110.11
12827	ENSG00000170373.8	ENSG00000263512.1	ENSG00000095587.9
12828	ENSG00000170374.6	ENSG00000263511.1	ENSG00000065809.13
12829	ENSG00000170379.20	ENSG00000263510.1	ENSG00000254975.1
12830	ENSG00000170381.14	ENSG00000263508.6	ENSG00000237745.1
12831	ENSG00000170382.12	ENSG00000263505.1	ENSG00000168126.3
12832	ENSG00000170385.10	ENSG00000263503.1	ENSG00000272888.6
12833	ENSG00000170390.16	ENSG00000263501.1	ENSG00000253301.5
12834	ENSG00000170396.8	ENSG00000263499.1	ENSG00000264023.1
12835	ENSG00000170409.7	ENSG00000263494.1	ENSG00000258443.1
12836	ENSG00000170412.17	ENSG00000263490.2	ENSG00000259078.2
12837	ENSG00000170417.16	ENSG00000263489.1	ENSG00000283977.1
12838	ENSG00000170419.10	ENSG00000263485.1	ENSG00000212766.9
12839	ENSG00000170421.12	ENSG00000263483.1	ENSG00000242588.6
12840	ENSG00000170423.12	ENSG00000263482.3	ENSG00000181104.7
12841	ENSG00000170425.3	ENSG00000263479.2	ENSG00000280561.1
12842	ENSG00000170426.2	ENSG00000263477.1	ENSG00000231871.5
12843	ENSG00000170430.10	ENSG00000263476.1	ENSG00000283427.1
12844	ENSG00000170439.7	ENSG00000263468.1	ENSG00000252743.1
12845	ENSG00000170442.11	ENSG00000263466.1	ENSG00000232654.1
12846	ENSG00000170445.14	ENSG00000263465.4	ENSG00000235720.1
12847	ENSG00000170448.12	ENSG00000263464.2	ENSG00000254691.1
12848	ENSG00000170454.6	ENSG00000263460.1	ENSG00000267253.1
12849	ENSG00000170456.16	ENSG00000263458.1	ENSG00000279721.1
12850	ENSG00000170458.14	ENSG00000263456.1	ENSG00000232539.1
12851	ENSG00000170464.10	ENSG00000263453.1	ENSG00000225675.2
12852	ENSG00000170465.10	ENSG00000263450.1	ENSG00000070778.13
12853	ENSG00000170468.7	ENSG00000263445.1	ENSG00000261221.3
12854	ENSG00000170469.10	ENSG00000263443.1	ENSG00000271582.1
12855	ENSG00000170471.15	ENSG00000263439.1	ENSG00000230914.3
12856	ENSG00000170473.17	ENSG00000263438.5	ENSG00000186038.9
12857	ENSG00000170476.15	ENSG00000263436.1	ENSG00000270282.1
12858	ENSG00000170477.13	ENSG00000263435.1	ENSG00000184106.8
12859	ENSG00000170482.17	ENSG00000263433.1	ENSG00000282826.2
12860	ENSG00000170484.9	ENSG00000263432.2	ENSG00000106113.19
12861	ENSG00000170485.17	ENSG00000263429.3	ENSG00000091106.19
12862	ENSG00000170486.11	ENSG00000263427.1	ENSG00000272973.1
12863	ENSG00000170498.9	ENSG00000263426.2	ENSG00000244618.3
12864	ENSG00000170500.12	ENSG00000263424.1	ENSG00000277543.1
12865	ENSG00000170502.13	ENSG00000263417.4	ENSG00000212371.1
12866	ENSG00000170509.12	ENSG00000263414.1	ENSG00000166317.12
12867	ENSG00000170515.14	ENSG00000263413.2	ENSG00000233189.1
12868	ENSG00000170516.17	ENSG00000263412.1	ENSG00000260659.1
12869	ENSG00000170522.9	ENSG00000263410.2	ENSG00000267742.1
12870	ENSG00000170523.3	ENSG00000263409.1	ENSG00000234645.3
12871	ENSG00000170525.21	ENSG00000263407.1	ENSG00000135314.12
12872	ENSG00000170537.13	ENSG00000263405.1	ENSG00000225673.3
12873	ENSG00000170540.15	ENSG00000263403.1	ENSG00000268886.1
12874	ENSG00000170542.6	ENSG00000263400.7	ENSG00000157423.18
12875	ENSG00000170545.17	ENSG00000263399.1	ENSG00000228532.4
12876	ENSG00000170549.4	ENSG00000263396.1	ENSG00000243083.6

12877	ENSG00000170558.9	ENSG00000263394.1	ENSG00000169621.10
12878	ENSG00000170561.13	ENSG00000263393.1	ENSG00000258651.1
12879	ENSG00000170571.12	ENSG00000263390.1	ENSG00000238213.1
12880	ENSG00000170577.8	ENSG00000263389.1	ENSG00000211946.3
12881	ENSG00000170579.17	ENSG00000263388.1	ENSG00000213305.3
12882	ENSG00000170581.14	ENSG00000263382.1	ENSG00000188580.14
12883	ENSG00000170584.11	ENSG00000263381.1	ENSG00000105404.11
12884	ENSG00000170604.5	ENSG00000263375.1	ENSG00000116117.18
12885	ENSG00000170605.6	ENSG00000263372.1	ENSG00000234311.1
12886	ENSG00000170606.15	ENSG00000263370.1	ENSG00000264028.2
12887	ENSG00000170608.3	ENSG00000263369.6	ENSG00000263883.1
12888	ENSG00000170613.4	ENSG00000263368.1	ENSG00000230224.1
12889	ENSG00000170615.14	ENSG00000263366.2	ENSG00000115486.12
12890	ENSG00000170619.10	ENSG00000263363.1	ENSG00000276298.1
12891	ENSG00000170624.13	ENSG00000263361.1	ENSG00000179299.17
12892	ENSG00000170627.11	ENSG00000263360.2	ENSG00000226049.3
12893	ENSG00000170629.14	ENSG00000263355.1	ENSG00000133030.21
12894	ENSG00000170631.15	ENSG00000263354.1	ENSG00000051596.9
12895	ENSG00000170632.14	ENSG00000263353.3	ENSG00000058799.15
12896	ENSG00000170633.16	ENSG00000263345.1	ENSG00000101444.13
12897	ENSG00000170634.13	ENSG00000263343.1	ENSG00000196975.16
12898	ENSG00000170638.9	ENSG00000263342.1	ENSG00000226026.5
12899	ENSG00000170653.19	ENSG00000263338.1	ENSG00000275129.1
12900	ENSG00000170667.14	ENSG00000263335.1	ENSG00000268182.6
12901	ENSG00000170677.6	ENSG00000263331.1	ENSG00000273073.1
12902	ENSG00000170681.6	ENSG00000263327.6	ENSG00000270127.2
12903	ENSG00000170683.6	ENSG00000263326.1	ENSG00000278828.1
12904	ENSG00000170684.9	ENSG00000263325.1	ENSG00000237605.1
12905	ENSG00000170688.4	ENSG00000263317.1	ENSG00000232628.5
12906	ENSG00000170689.10	ENSG00000263316.1	ENSG00000241418.1
12907	ENSG00000170703.15	ENSG00000263312.1	ENSG00000185040.13
12908	ENSG00000170734.11	ENSG00000263311.1	ENSG00000274591.1
12909	ENSG00000170743.17	ENSG00000263307.1	ENSG00000189079.16
12910	ENSG00000170745.12	ENSG00000263305.1	ENSG00000171316.12
12911	ENSG00000170748.6	ENSG00000263301.1	ENSG00000280374.1
12912	ENSG00000170759.11	ENSG00000263300.1	ENSG00000267783.1
12913	ENSG00000170775.2	ENSG00000263293.2	ENSG00000255478.1
12914	ENSG00000170776.21	ENSG00000263280.1	ENSG00000137269.15
12915	ENSG00000170777.10	ENSG00000263279.1	ENSG00000273248.1
12916	ENSG00000170779.10	ENSG00000263278.2	ENSG00000164746.14
12917	ENSG00000170782.3	ENSG00000263276.1	ENSG00000236675.1
12918	ENSG00000170786.12	ENSG00000263272.1	ENSG00000249109.1
12919	ENSG00000170788.14	ENSG00000263271.1	ENSG00000276522.1
12920	ENSG00000170790.5	ENSG00000263266.2	ENSG00000225549.3
12921	ENSG00000170791.17	ENSG00000263264.1	ENSG00000167711.13
12922	ENSG00000170801.10	ENSG00000263257.2	ENSG00000263413.2
12923	ENSG00000170802.16	ENSG00000263253.1	ENSG00000118680.13
12924	ENSG00000170807.12	ENSG00000263252.1	ENSG00000275343.1
12925	ENSG00000170819.4	ENSG00000263244.2	ENSG00000279781.1
12926	ENSG00000170827.9	ENSG00000263241.1	ENSG00000105699.16
12927	ENSG00000170832.13	ENSG00000263237.1	ENSG00000241188.3
12928	ENSG00000170835.14	ENSG00000263235.1	ENSG00000187999.4
12929	ENSG00000170836.11	ENSG00000263234.1	ENSG00000131080.15

12930	ENSG00000170837.2	ENSG00000263232.2	ENSG00000235582.2
12931	ENSG00000170846.17	ENSG00000263220.1	ENSG00000138411.13
12932	ENSG00000170848.15	ENSG00000263219.1	ENSG00000241790.2
12933	ENSG00000170852.11	ENSG00000263218.2	ENSG00000285458.1
12934	ENSG00000170854.18	ENSG00000263212.2	ENSG00000255165.1
12935	ENSG00000170855.4	ENSG00000263207.2	ENSG00000147082.17
12936	ENSG00000170858.10	ENSG00000263206.1	ENSG00000161640.15
12937	ENSG00000170860.4	ENSG00000263201.1	ENSG00000236325.1
12938	ENSG00000170871.12	ENSG00000263199.1	ENSG00000269524.1
12939	ENSG00000170873.18	ENSG00000263196.1	ENSG00000099849.15
12940	ENSG00000170876.7	ENSG00000263189.1	ENSG00000100994.12
12941	ENSG00000170881.4	ENSG00000263179.1	ENSG00000261314.1
12942	ENSG00000170889.14	ENSG00000263177.1	ENSG00000104177.18
12943	ENSG00000170890.14	ENSG00000263176.1	ENSG00000229996.1
12944	ENSG00000170891.11	ENSG00000263171.1	ENSG00000242154.1
12945	ENSG00000170892.11	ENSG00000263167.1	ENSG00000269971.1
12946	ENSG00000170893.4	ENSG00000263165.1	ENSG00000259090.1
12947	ENSG00000170899.11	ENSG00000263164.1	ENSG00000258803.1
12948	ENSG00000170903.11	ENSG00000263159.1	ENSG00000137106.18
12949	ENSG00000170906.15	ENSG00000263155.6	ENSG00000266171.1
12950	ENSG00000170909.13	ENSG00000263154.1	ENSG00000198075.10
12951	ENSG00000170915.9	ENSG00000263146.2	ENSG00000271554.1
12952	ENSG00000170917.13	ENSG00000263142.5	ENSG00000280122.1
12953	ENSG00000170919.15	ENSG00000263126.1	ENSG00000214389.2
12954	ENSG00000170920.2	ENSG00000263125.1	ENSG00000227242.4
12955	ENSG00000170921.15	ENSG00000263120.1	ENSG00000267241.2
12956	ENSG00000170923.3	ENSG00000263110.1	ENSG00000267666.2
12957	ENSG00000170925.3	ENSG00000263107.1	ENSG00000239868.3
12958	ENSG00000170927.14	ENSG00000263105.1	ENSG00000196604.12
12959	ENSG00000170929.6	ENSG00000263098.1	ENSG00000089199.10
12960	ENSG00000170935.7	ENSG00000263096.1	ENSG00000234350.4
12961	ENSG00000170946.15	ENSG00000263089.1	ENSG00000133466.14
12962	ENSG00000170948.3	ENSG00000263081.1	ENSG00000113248.6
12963	ENSG00000170949.17	ENSG00000263080.1	ENSG00000237737.5
12964	ENSG00000170950.5	ENSG00000263072.6	ENSG00000285807.1
12965	ENSG00000170953.3	ENSG00000263070.1	ENSG00000243871.3
12966	ENSG00000170954.11	ENSG00000263069.5	ENSG00000163541.12
12967	ENSG00000170955.10	ENSG00000263065.1	ENSG00000166268.10
12968	ENSG00000170956.17	ENSG00000263063.1	ENSG00000067082.15
12969	ENSG00000170961.7	ENSG00000263051.1	ENSG00000179913.10
12970	ENSG00000170962.13	ENSG00000263050.1	ENSG00000188959.9
12971	ENSG00000170965.10	ENSG00000263045.1	ENSG00000136908.17
12972	ENSG00000170967.4	ENSG00000263041.1	ENSG00000261312.1
12973	ENSG00000170983.3	ENSG00000263033.2	ENSG00000173715.16
12974	ENSG00000170989.9	ENSG00000263029.1	ENSG00000163072.15
12975	ENSG00000171004.18	ENSG00000263017.1	ENSG00000272249.1
12976	ENSG00000171014.2	ENSG00000263015.1	ENSG00000284824.1
12977	ENSG00000171016.12	ENSG00000263011.1	ENSG00000228502.1
12978	ENSG00000171017.11	ENSG00000263006.6	ENSG00000230750.1
12979	ENSG00000171033.13	ENSG00000263004.1	ENSG00000249926.2
12980	ENSG00000171044.10	ENSG00000263002.8	ENSG00000273335.1
12981	ENSG00000171045.15	ENSG00000263001.6	ENSG00000128045.7
12982	ENSG00000171049.9	ENSG00000262999.1	ENSG00000266997.2

12983	ENSG00000171051.8	ENSG00000262995.1	ENSG00000285108.1
12984	ENSG00000171053.9	ENSG00000262990.1	ENSG00000230303.5
12985	ENSG00000171054.8	ENSG00000262983.1	ENSG00000269901.1
12986	ENSG00000171055.15	ENSG00000262980.1	ENSG00000276282.1
12987	ENSG00000171056.8	ENSG00000262979.1	ENSG00000075651.16
12988	ENSG00000171060.11	ENSG00000262974.1	ENSG00000138160.6
12989	ENSG00000171067.11	ENSG00000262967.1	ENSG00000132825.7
12990	ENSG00000171084.15	ENSG00000262966.2	ENSG00000204396.10
12991	ENSG00000171094.18	ENSG00000262962.1	ENSG00000279250.1
12992	ENSG00000171097.14	ENSG00000262961.1	ENSG00000111348.9
12993	ENSG00000171100.14	ENSG00000262959.2	ENSG00000146666.5
12994	ENSG00000171101.13	ENSG00000262953.1	ENSG00000220937.3
12995	ENSG00000171102.14	ENSG00000262950.1	ENSG00000220695.1
12996	ENSG00000171103.11	ENSG00000262943.7	ENSG00000212643.3
12997	ENSG00000171105.14	ENSG00000262921.1	ENSG00000279294.1
12998	ENSG00000171109.19	ENSG00000262920.5	ENSG00000263424.1
12999	ENSG00000171115.4	ENSG00000262919.8	ENSG00000233680.4
13000	ENSG00000171116.7	ENSG00000262905.1	ENSG00000244086.1
13001	ENSG00000171119.2	ENSG00000262904.1	ENSG00000144354.14
13002	ENSG00000171121.16	ENSG00000262903.1	ENSG00000196666.5
13003	ENSG00000171124.13	ENSG00000262902.1	ENSG00000233383.1
13004	ENSG00000171126.8	ENSG00000262899.1	ENSG00000226762.2
13005	ENSG00000171130.18	ENSG00000262898.2	ENSG00000165695.9
13006	ENSG00000171132.14	ENSG00000262890.1	ENSG00000127743.6
13007	ENSG00000171133.3	ENSG00000262885.1	ENSG00000104848.1
13008	ENSG00000171135.14	ENSG00000262884.1	ENSG00000117475.14
13009	ENSG00000171136.6	ENSG00000262881.1	ENSG00000241120.1
13010	ENSG00000171148.13	ENSG00000262880.1	ENSG00000197476.3
13011	ENSG00000171150.9	ENSG00000262879.5	ENSG00000237484.5
13012	ENSG00000171155.8	ENSG00000262877.4	ENSG00000286098.1
13013	ENSG00000171159.5	ENSG00000262874.2	ENSG00000175229.7
13014	ENSG00000171160.18	ENSG00000262870.1	ENSG00000109684.15
13015	ENSG00000171161.13	ENSG00000262869.1	ENSG00000163081.3
13016	ENSG00000171163.15	ENSG00000262855.1	ENSG00000168878.16
13017	ENSG00000171169.9	ENSG00000262848.1	ENSG00000260617.1
13018	ENSG00000171174.15	ENSG00000262837.1	ENSG00000170946.15
13019	ENSG00000171180.2	ENSG00000262833.1	ENSG00000201684.1
13020	ENSG00000171189.17	ENSG00000262831.1	ENSG00000151364.17
13021	ENSG00000171195.11	ENSG00000262818.1	ENSG00000255122.1
13022	ENSG00000171199.11	ENSG00000262815.1	ENSG00000223723.1
13023	ENSG00000171201.11	ENSG00000262814.8	ENSG00000167608.11
13024	ENSG00000171202.7	ENSG00000262810.1	ENSG00000198691.13
13025	ENSG00000171204.12	ENSG00000262803.1	ENSG00000280291.1
13026	ENSG00000171206.14	ENSG00000262801.5	ENSG00000214102.7
13027	ENSG00000171208.9	ENSG00000262791.1	ENSG00000198081.11
13028	ENSG00000171209.3	ENSG00000262786.1	ENSG00000218676.1
13029	ENSG00000171217.5	ENSG00000262777.1	ENSG00000161642.17
13030	ENSG00000171219.9	ENSG00000262772.1	ENSG00000153956.16
13031	ENSG00000171222.10	ENSG00000262769.1	ENSG00000178996.14
13032	ENSG00000171223.6	ENSG00000262768.2	ENSG00000235045.2
13033	ENSG00000171224.9	ENSG00000262766.1	ENSG00000223946.1
13034	ENSG00000171227.7	ENSG00000262759.1	ENSG00000257534.1
13035	ENSG00000171234.14	ENSG00000262732.1	ENSG00000184949.16

13036	ENSG00000171236.10	ENSG00000262730.1	ENSG00000274256.1
13037	ENSG00000171241.9	ENSG00000262728.5	ENSG00000223503.1
13038	ENSG00000171243.8	ENSG00000262714.1	ENSG00000184207.8
13039	ENSG00000171246.6	ENSG00000262712.1	ENSG00000268941.2
13040	ENSG00000171262.11	ENSG00000262708.1	ENSG00000248155.1
13041	ENSG00000171291.8	ENSG00000262703.1	ENSG00000007306.15
13042	ENSG00000171295.12	ENSG00000262700.1	ENSG00000230944.1
13043	ENSG00000171298.13	ENSG00000262693.1	ENSG00000246430.6
13044	ENSG00000171302.17	ENSG00000262692.1	ENSG00000227487.3
13045	ENSG00000171303.7	ENSG00000262691.1	ENSG00000238259.1
13046	ENSG00000171307.19	ENSG00000262686.1	ENSG00000260644.6
13047	ENSG00000171310.11	ENSG00000262681.2	ENSG00000101198.15
13048	ENSG00000171311.12	ENSG00000262678.1	ENSG00000011332.19
13049	ENSG00000171314.9	ENSG00000262668.2	ENSG00000239686.1
13050	ENSG00000171316.12	ENSG00000262664.3	ENSG00000270427.1
13051	ENSG00000171320.14	ENSG00000262663.1	ENSG00000125457.14
13052	ENSG00000171345.13	ENSG00000262662.1	ENSG00000144744.17
13053	ENSG00000171346.16	ENSG00000262660.1	ENSG00000164938.14
13054	ENSG00000171357.6	ENSG00000262655.4	ENSG00000236928.3
13055	ENSG00000171365.17	ENSG00000262652.1	ENSG00000084636.18
13056	ENSG00000171368.12	ENSG00000262651.1	ENSG00000260884.1
13057	ENSG00000171385.9	ENSG00000262648.1	ENSG00000129422.14
13058	ENSG00000171388.12	ENSG00000262636.1	ENSG00000184139.8
13059	ENSG00000171396.11	ENSG00000262633.2	ENSG00000271774.1
13060	ENSG00000171401.15	ENSG00000262628.1	ENSG00000216636.1
13061	ENSG00000171402.15	ENSG00000262624.1	ENSG00000182718.16
13062	ENSG00000171403.10	ENSG00000262623.1	ENSG00000260558.2
13063	ENSG00000171405.12	ENSG00000262621.5	ENSG00000279409.1
13064	ENSG00000171408.14	ENSG00000262619.1	ENSG00000248290.1
13065	ENSG00000171421.13	ENSG00000262609.1	ENSG00000261189.1
13066	ENSG00000171425.10	ENSG00000262587.2	ENSG00000187686.4
13067	ENSG00000171428.14	ENSG00000262585.1	ENSG00000198650.11
13068	ENSG00000171431.3	ENSG00000262583.1	ENSG00000276105.1
13069	ENSG00000171433.12	ENSG00000262580.5	ENSG00000213020.10
13070	ENSG00000171435.13	ENSG00000262576.3	ENSG00000121410.11
13071	ENSG00000171443.7	ENSG00000262566.1	ENSG00000213005.3
13072	ENSG00000171444.18	ENSG00000262561.1	ENSG00000279220.3
13073	ENSG00000171446.6	ENSG00000262560.1	ENSG00000243323.7
13074	ENSG00000171448.9	ENSG00000262558.1	ENSG00000224668.1
13075	ENSG00000171450.5	ENSG00000262554.1	ENSG00000256747.1
13076	ENSG00000171451.14	ENSG00000262543.1	ENSG00000255114.1
13077	ENSG00000171453.19	ENSG00000262539.1	ENSG00000283156.1
13078	ENSG00000171456.19	ENSG00000262533.1	ENSG00000261338.2
13079	ENSG00000171459.4	ENSG00000262529.1	ENSG00000226632.1
13080	ENSG00000171462.15	ENSG00000262528.2	ENSG00000279372.1
13081	ENSG00000171466.10	ENSG00000262526.2	ENSG00000213312.3
13082	ENSG00000171467.16	ENSG00000262521.1	ENSG00000267549.6
13083	ENSG00000171469.11	ENSG00000262519.1	ENSG00000240002.1
13084	ENSG00000171475.14	ENSG00000262518.1	ENSG00000144596.13
13085	ENSG00000171476.22	ENSG00000262516.1	ENSG00000214973.3
13086	ENSG00000171478.7	ENSG00000262514.1	ENSG00000236316.2
13087	ENSG00000171481.4	ENSG00000262503.1	ENSG00000164690.8
13088	ENSG00000171483.13	ENSG00000262497.1	ENSG00000260091.1

13089	ENSG00000171487.14	ENSG00000262495.1	ENSG00000109184.15
13090	ENSG00000171488.15	ENSG00000262492.1	ENSG00000107831.12
13091	ENSG00000171489.10	ENSG00000262488.1	ENSG00000234886.1
13092	ENSG00000171490.13	ENSG00000262484.1	ENSG00000160310.18
13093	ENSG00000171492.14	ENSG00000262482.1	ENSG00000271366.1
13094	ENSG00000171495.17	ENSG00000262481.5	ENSG00000254831.1
13095	ENSG00000171496.4	ENSG00000262480.2	ENSG00000258871.1
13096	ENSG00000171497.5	ENSG00000262477.1	ENSG00000261068.2
13097	ENSG00000171501.9	ENSG00000262471.1	ENSG00000184454.7
13098	ENSG00000171502.15	ENSG00000262470.2	ENSG00000148082.10
13099	ENSG00000171503.11	ENSG00000262468.6	ENSG00000213121.2
13100	ENSG00000171505.5	ENSG00000262461.6	ENSG00000129467.13
13101	ENSG00000171509.16	ENSG00000262456.1	ENSG00000173894.11
13102	ENSG00000171517.6	ENSG00000262454.3	ENSG00000168079.17
13103	ENSG00000171522.6	ENSG00000262445.3	ENSG00000232874.1
13104	ENSG00000171530.14	ENSG00000262434.1	ENSG00000181009.5
13105	ENSG00000171532.5	ENSG00000262429.1	ENSG00000274528.1
13106	ENSG00000171533.11	ENSG00000262420.3	ENSG00000099995.19
13107	ENSG00000171540.7	ENSG00000262413.1	ENSG00000204231.10
13108	ENSG00000171551.12	ENSG00000262412.1	ENSG00000121446.20
13109	ENSG00000171552.13	ENSG00000262410.1	ENSG00000279663.2
13110	ENSG00000171557.16	ENSG00000262408.1	ENSG00000276362.1
13111	ENSG00000171560.15	ENSG00000262406.2	ENSG00000279431.1
13112	ENSG00000171561.4	ENSG00000262402.1	ENSG00000232855.6
13113	ENSG00000171564.11	ENSG00000262400.1	ENSG00000109471.4
13114	ENSG00000171566.12	ENSG00000262384.1	ENSG00000271717.1
13115	ENSG00000171570.11	ENSG00000262381.1	ENSG00000236806.1
13116	ENSG00000171574.18	ENSG00000262380.1	ENSG00000233514.1
13117	ENSG00000171587.15	ENSG00000262372.1	ENSG00000162399.8
13118	ENSG00000171595.14	ENSG00000262370.5	ENSG00000120217.14
13119	ENSG00000171596.7	ENSG00000262366.1	ENSG00000259150.5
13120	ENSG00000171603.17	ENSG00000262362.1	ENSG00000273027.1
13121	ENSG00000171604.12	ENSG00000262358.1	ENSG00000257042.1
13122	ENSG00000171606.18	ENSG00000262352.1	ENSG00000236456.1
13123	ENSG00000171608.15	ENSG00000262343.1	ENSG00000279805.1
13124	ENSG00000171611.9	ENSG00000262339.5	ENSG00000125378.16
13125	ENSG00000171612.7	ENSG00000262333.1	ENSG00000271937.1
13126	ENSG00000171617.14	ENSG00000262332.1	ENSG00000225194.3
13127	ENSG00000171621.14	ENSG00000262322.1	ENSG00000185974.7
13128	ENSG00000171631.14	ENSG00000262316.1	ENSG00000267793.1
13129	ENSG00000171634.18	ENSG00000262313.1	ENSG00000126945.9
13130	ENSG00000171643.14	ENSG00000262312.2	ENSG00000198610.11
13131	ENSG00000171649.12	ENSG00000262304.2	ENSG00000278997.1
13132	ENSG00000171657.6	ENSG00000262302.1	ENSG00000241185.2
13133	ENSG00000171658.8	ENSG00000262298.1	ENSG00000255855.2
13134	ENSG00000171659.15	ENSG00000262296.1	ENSG00000237934.1
13135	ENSG00000171671.6	ENSG00000262294.1	ENSG00000201098.1
13136	ENSG00000171680.21	ENSG00000262292.2	ENSG00000005243.10
13137	ENSG00000171681.12	ENSG00000262271.1	ENSG00000166197.16
13138	ENSG00000171695.10	ENSG00000262267.5	ENSG00000136859.10
13139	ENSG00000171700.14	ENSG00000262265.2	ENSG00000213529.3
13140	ENSG00000171703.17	ENSG00000262262.1	ENSG00000215812.5
13141	ENSG00000171711.2	ENSG00000262259.1	ENSG00000148308.17

13142	ENSG00000171714.11	ENSG00000262248.1	ENSG00000131408.14
13143	ENSG00000171720.10	ENSG00000262246.6	ENSG00000234130.2
13144	ENSG00000171722.12	ENSG00000262235.2	ENSG00000231830.1
13145	ENSG00000171723.15	ENSG00000262231.1	ENSG00000187905.10
13146	ENSG00000171724.3	ENSG00000262228.2	ENSG00000229941.5
13147	ENSG00000171729.14	ENSG00000262227.1	ENSG00000120334.15
13148	ENSG00000171735.19	ENSG00000262223.7	ENSG00000172733.11
13149	ENSG00000171747.9	ENSG00000262213.1	ENSG00000108852.14
13150	ENSG00000171757.15	ENSG00000262211.1	ENSG00000211801.3
13151	ENSG00000171759.10	ENSG00000262209.3	ENSG00000273496.1
13152	ENSG00000171763.19	ENSG00000262202.4	ENSG00000102287.19
13153	ENSG00000171766.16	ENSG00000262198.1	ENSG00000221571.3
13154	ENSG00000171772.16	ENSG00000262188.1	ENSG00000236990.1
13155	ENSG00000171773.2	ENSG00000262187.2	ENSG00000099860.9
13156	ENSG00000171777.16	ENSG00000262185.2	ENSG00000166664.13
13157	ENSG00000171786.6	ENSG00000262181.2	ENSG00000230359.5
13158	ENSG00000171790.15	ENSG00000262180.1	ENSG00000174912.7
13159	ENSG00000171791.13	ENSG00000262179.2	ENSG00000228634.1
13160	ENSG00000171792.11	ENSG00000262172.1	ENSG00000196754.10
13161	ENSG00000171793.15	ENSG00000262171.1	ENSG00000284715.1
13162	ENSG00000171794.4	ENSG00000262165.2	ENSG00000116793.16
13163	ENSG00000171798.18	ENSG00000262160.1	ENSG00000229770.2
13164	ENSG00000171804.10	ENSG00000262158.1	ENSG00000286088.1
13165	ENSG00000171806.12	ENSG00000262155.1	ENSG00000270800.3
13166	ENSG00000171811.14	ENSG00000262154.1	ENSG00000225766.10
13167	ENSG00000171812.13	ENSG00000262152.6	ENSG00000277669.1
13168	ENSG00000171813.14	ENSG00000262151.1	ENSG00000228398.3
13169	ENSG00000171815.5	ENSG00000262147.1	ENSG00000131477.11
13170	ENSG00000171817.17	ENSG00000262141.1	ENSG00000242197.2
13171	ENSG00000171819.5	ENSG00000262140.1	ENSG00000164100.9
13172	ENSG00000171823.7	ENSG00000262136.1	ENSG00000187527.10
13173	ENSG00000171824.14	ENSG00000262135.1	ENSG00000230778.1
13174	ENSG00000171827.10	ENSG00000262133.1	ENSG00000235333.3
13175	ENSG00000171840.11	ENSG00000262120.1	ENSG00000281005.1
13176	ENSG00000171843.16	ENSG00000262119.1	ENSG00000268603.1
13177	ENSG00000171847.10	ENSG00000262118.1	ENSG00000232385.2
13178	ENSG00000171848.15	ENSG00000262117.5	ENSG00000249013.1
13179	ENSG00000171853.16	ENSG00000262116.1	ENSG00000131069.20
13180	ENSG00000171855.7	ENSG00000262115.1	ENSG00000231187.2
13181	ENSG00000171858.18	ENSG00000262112.1	ENSG00000119541.10
13182	ENSG00000171860.4	ENSG00000262107.1	ENSG00000266897.1
13183	ENSG00000171861.11	ENSG00000262106.1	ENSG00000110700.7
13184	ENSG00000171862.11	ENSG00000262099.1	ENSG00000185483.12
13185	ENSG00000171863.14	ENSG00000262098.1	ENSG00000227331.1
13186	ENSG00000171864.5	ENSG00000262097.1	ENSG00000261821.2
13187	ENSG00000171865.10	ENSG00000262096.2	ENSG00000136931.10
13188	ENSG00000171867.16	ENSG00000262095.1	ENSG00000253333.1
13189	ENSG00000171872.4	ENSG00000262094.1	ENSG00000263370.1
13190	ENSG00000171873.7	ENSG00000262090.1	ENSG00000198156.10
13191	ENSG00000171877.21	ENSG00000262089.1	ENSG00000257222.1
13192	ENSG00000171885.15	ENSG00000262085.2	ENSG00000154493.18
13193	ENSG00000171889.3	ENSG00000262081.2	ENSG00000280092.1
13194	ENSG00000171903.16	ENSG00000262079.1	ENSG00000280379.1

13195	ENSG00000171914.16	ENSG00000262075.3	ENSG00000224051.7
13196	ENSG00000171916.16	ENSG00000262074.6	ENSG00000250950.1
13197	ENSG00000171928.14	ENSG00000262067.1	ENSG00000230536.1
13198	ENSG00000171931.12	ENSG00000262061.5	ENSG00000253616.5
13199	ENSG00000171936.2	ENSG00000262052.1	ENSG00000227815.2
13200	ENSG00000171940.13	ENSG00000262050.1	ENSG00000212807.2
13201	ENSG00000171942.4	ENSG00000262048.1	ENSG00000230833.1
13202	ENSG00000171943.11	ENSG00000262039.1	ENSG00000242173.9
13203	ENSG00000171944.2	ENSG00000262038.1	ENSG00000123416.15
13204	ENSG00000171951.5	ENSG00000262031.1	ENSG00000196539.3
13205	ENSG00000171953.16	ENSG00000262020.1	ENSG00000110400.11
13206	ENSG00000171954.13	ENSG00000262011.1	ENSG00000148719.15
13207	ENSG00000171956.7	ENSG00000262008.1	ENSG00000188305.6
13208	ENSG00000171960.11	ENSG00000262006.1	ENSG00000099875.14
13209	ENSG00000171962.17	ENSG00000262003.1	ENSG00000270178.1
13210	ENSG00000171970.13	ENSG00000262001.1	ENSG00000232880.1
13211	ENSG00000171984.15	ENSG00000262000.1	ENSG00000230005.2
13212	ENSG00000171987.1	ENSG00000261997.1	ENSG00000263781.3
13213	ENSG00000171988.19	ENSG00000261996.1	ENSG00000259678.1
13214	ENSG00000171989.5	ENSG00000261987.2	ENSG00000255145.2
13215	ENSG00000171992.13	ENSG00000261978.1	ENSG00000234139.3
13216	ENSG00000171999.7	ENSG00000261976.2	ENSG00000172938.3
13217	ENSG00000172000.7	ENSG00000261971.7	ENSG00000166428.13
13218	ENSG00000172005.11	ENSG00000261970.1	ENSG00000249142.1
13219	ENSG00000172006.11	ENSG00000261965.1	ENSG00000121440.15
13220	ENSG00000172007.6	ENSG00000261963.3	ENSG00000227290.1
13221	ENSG00000172009.15	ENSG00000261959.1	ENSG00000233791.5
13222	ENSG00000172014.12	ENSG00000261949.5	ENSG00000240785.2
13223	ENSG00000172016.15	ENSG00000261939.1	ENSG00000276975.3
13224	ENSG00000172020.12	ENSG00000261938.1	ENSG00000254340.1
13225	ENSG00000172023.7	ENSG00000261934.2	ENSG00000213856.3
13226	ENSG00000172031.7	ENSG00000261924.1	ENSG00000204832.9
13227	ENSG00000172037.14	ENSG00000261916.1	ENSG00000143184.5
13228	ENSG00000172046.18	ENSG00000261915.6	ENSG00000160712.13
13229	ENSG00000172053.18	ENSG00000261914.2	ENSG00000232987.2
13230	ENSG00000172057.10	ENSG00000261904.1	ENSG00000269954.2
13231	ENSG00000172058.15	ENSG00000261889.1	ENSG00000232901.1
13232	ENSG00000172059.11	ENSG00000261888.1	ENSG00000207117.1
13233	ENSG00000172061.8	ENSG00000261886.1	ENSG00000170469.10
13234	ENSG00000172062.16	ENSG00000261884.2	ENSG00000225521.1
13235	ENSG00000172071.12	ENSG00000261882.1	ENSG00000197140.15
13236	ENSG00000172073.4	ENSG00000261879.6	ENSG00000166596.15
13237	ENSG00000172081.14	ENSG00000261873.2	ENSG00000258181.1
13238	ENSG00000172086.8	ENSG00000261872.1	ENSG00000238123.1
13239	ENSG00000172113.9	ENSG00000261868.1	ENSG00000156574.9
13240	ENSG00000172115.8	ENSG00000261866.2	ENSG00000144410.4
13241	ENSG00000172116.22	ENSG00000261864.1	ENSG00000172971.7
13242	ENSG00000172123.12	ENSG00000261863.1	ENSG00000279039.1
13243	ENSG00000172137.19	ENSG00000261857.7	ENSG00000232828.1
13244	ENSG00000172139.15	ENSG00000261856.1	ENSG00000120500.17
13245	ENSG00000172146.2	ENSG00000261848.5	ENSG00000272861.1
13246	ENSG00000172148.3	ENSG00000261845.2	ENSG00000118946.12
13247	ENSG00000172150.4	ENSG00000261842.1	ENSG00000271580.1

13248	ENSG00000172154.10	ENSG00000261840.2	ENSG00000128268.12
13249	ENSG00000172155.9	ENSG00000261839.1	ENSG00000206190.11
13250	ENSG00000172156.3	ENSG00000261838.5	ENSG00000243365.3
13251	ENSG00000172159.16	ENSG00000261837.1	ENSG00000136928.7
13252	ENSG00000172164.15	ENSG00000261835.1	ENSG00000248925.1
13253	ENSG00000172167.8	ENSG00000261834.2	ENSG00000213416.4
13254	ENSG00000172171.11	ENSG00000261833.2	ENSG00000234428.2
13255	ENSG00000172172.7	ENSG00000261832.6	ENSG00000183260.7
13256	ENSG00000172175.14	ENSG00000261831.2	ENSG00000272412.2
13257	ENSG00000172179.12	ENSG00000261829.1	ENSG00000254855.1
13258	ENSG00000172183.15	ENSG00000261826.1	ENSG00000100558.9
13259	ENSG00000172186.7	ENSG00000261824.6	ENSG00000230929.5
13260	ENSG00000172188.5	ENSG00000261823.1	ENSG00000179994.11
13261	ENSG00000172197.10	ENSG00000261822.1	ENSG00000233746.1
13262	ENSG00000172199.1	ENSG00000261821.2	ENSG00000260170.1
13263	ENSG00000172201.12	ENSG00000261820.1	ENSG00000159720.12
13264	ENSG00000172208.6	ENSG00000261819.1	ENSG00000253829.1
13265	ENSG00000172209.6	ENSG00000261818.1	ENSG00000233523.1
13266	ENSG00000172215.6	ENSG00000261817.1	ENSG00000234264.1
13267	ENSG00000172216.5	ENSG00000261816.1	ENSG00000165186.12
13268	ENSG00000172232.10	ENSG00000261815.1	ENSG00000184226.15
13269	ENSG00000172236.17	ENSG00000261813.2	ENSG00000272370.1
13270	ENSG00000172238.4	ENSG00000261812.6	ENSG00000274933.5
13271	ENSG00000172239.14	ENSG00000261811.1	ENSG00000227973.1
13272	ENSG00000172243.17	ENSG00000261810.1	ENSG00000253146.1
13273	ENSG00000172244.9	ENSG00000261809.1	ENSG00000250765.6
13274	ENSG00000172247.4	ENSG00000261807.2	ENSG00000234031.1
13275	ENSG00000172250.16	ENSG00000261804.1	ENSG00000236132.1
13276	ENSG00000172260.15	ENSG00000261803.1	ENSG00000131398.13
13277	ENSG00000172262.11	ENSG00000261802.1	ENSG00000258354.1
13278	ENSG00000172264.17	ENSG00000261801.5	ENSG00000268433.1
13279	ENSG00000172269.19	ENSG00000261800.1	ENSG00000241563.3
13280	ENSG00000172270.19	ENSG00000261799.1	ENSG00000279266.1
13281	ENSG00000172273.12	ENSG00000261798.1	ENSG00000233646.1
13282	ENSG00000172283.10	ENSG00000261797.1	ENSG00000240770.5
13283	ENSG00000172288.7	ENSG00000261796.1	ENSG00000124422.12
13284	ENSG00000172289.3	ENSG00000261795.1	ENSG00000130783.13
13285	ENSG00000172292.14	ENSG00000261794.1	ENSG00000102100.16
13286	ENSG00000172294.7	ENSG00000261793.1	ENSG00000119630.14
13287	ENSG00000172296.13	ENSG00000261792.1	ENSG00000160179.18
13288	ENSG00000172297.7	ENSG00000261790.1	ENSG00000168952.15
13289	ENSG00000172301.11	ENSG00000261789.1	ENSG00000232389.1
13290	ENSG00000172315.6	ENSG00000261787.2	ENSG00000283445.1
13291	ENSG00000172318.5	ENSG00000261786.1	ENSG00000174332.5
13292	ENSG00000172320.3	ENSG00000261783.1	ENSG00000256588.1
13293	ENSG00000172322.14	ENSG00000261782.1	ENSG00000092096.16
13294	ENSG00000172324.5	ENSG00000261781.1	ENSG00000203907.9
13295	ENSG00000172331.12	ENSG00000261780.2	ENSG00000164306.11
13296	ENSG00000172336.5	ENSG00000261779.1	ENSG00000230492.1
13297	ENSG00000172339.10	ENSG00000261778.1	ENSG00000132681.16
13298	ENSG00000172340.14	ENSG00000261776.1	ENSG00000184111.6
13299	ENSG00000172342.5	ENSG00000261775.1	ENSG00000146530.14
13300	ENSG00000172345.14	ENSG00000261774.1	ENSG00000110723.12

13301	ENSG00000172346.15	ENSG00000261773.1	ENSG00000236876.3
13302	ENSG00000172348.14	ENSG00000261771.5	ENSG00000167653.5
13303	ENSG00000172349.17	ENSG00000261770.1	ENSG00000143171.13
13304	ENSG00000172350.10	ENSG00000261766.1	ENSG00000102010.15
13305	ENSG00000172352.5	ENSG00000261765.1	ENSG00000255850.2
13306	ENSG00000172354.10	ENSG00000261764.1	ENSG00000228589.3
13307	ENSG00000172361.6	ENSG00000261763.1	ENSG00000100721.11
13308	ENSG00000172362.3	ENSG00000261762.1	ENSG00000249215.1
13309	ENSG00000172365.3	ENSG00000261761.4	ENSG00000236337.1
13310	ENSG00000172366.20	ENSG00000261760.8	ENSG00000270697.1
13311	ENSG00000172367.16	ENSG00000261759.1	ENSG00000149633.12
13312	ENSG00000172375.13	ENSG00000261758.1	ENSG00000133661.16
13313	ENSG00000172377.2	ENSG00000261757.1	ENSG00000230395.1
13314	ENSG00000172379.21	ENSG00000261754.2	ENSG00000205037.2
13315	ENSG00000172380.6	ENSG00000261752.1	ENSG00000284512.1
13316	ENSG00000172382.10	ENSG00000261751.1	ENSG00000159259.8
13317	ENSG00000172399.5	ENSG00000261749.2	ENSG00000166262.15
13318	ENSG00000172403.11	ENSG00000261748.1	ENSG00000112312.10
13319	ENSG00000172404.4	ENSG00000261747.3	ENSG00000161036.13
13320	ENSG00000172409.6	ENSG00000261745.2	ENSG00000279434.1
13321	ENSG00000172410.4	ENSG00000261744.1	ENSG00000279900.1
13322	ENSG00000172421.9	ENSG00000261743.1	ENSG00000278847.1
13323	ENSG00000172425.10	ENSG00000261742.5	ENSG00000261451.1
13324	ENSG00000172426.16	ENSG00000261741.5	ENSG00000259202.1
13325	ENSG00000172428.11	ENSG00000261739.2	ENSG00000268107.6
13326	ENSG00000172432.19	ENSG00000261738.6	ENSG00000235421.1
13327	ENSG00000172456.17	ENSG00000261736.1	ENSG00000227486.1
13328	ENSG00000172457.6	ENSG00000261734.1	ENSG00000213270.5
13329	ENSG00000172458.4	ENSG00000261732.1	ENSG00000204001.9
13330	ENSG00000172459.4	ENSG00000261731.2	ENSG00000177352.10
13331	ENSG00000172460.16	ENSG00000261730.1	ENSG00000237977.1
13332	ENSG00000172461.11	ENSG00000261729.1	ENSG00000164047.5
13333	ENSG00000172464.3	ENSG00000261728.1	ENSG00000243404.2
13334	ENSG00000172465.14	ENSG00000261727.2	ENSG00000224747.1
13335	ENSG00000172466.16	ENSG00000261725.1	ENSG00000253409.1
13336	ENSG00000172468.13	ENSG00000261723.1	ENSG00000251566.1
13337	ENSG00000172469.16	ENSG00000261722.1	ENSG00000105204.14
13338	ENSG00000172476.4	ENSG00000261720.1	ENSG00000168397.17
13339	ENSG00000172478.18	ENSG00000261719.1	ENSG00000223042.1
13340	ENSG00000172482.5	ENSG00000261717.5	ENSG00000277991.4
13341	ENSG00000172487.4	ENSG00000261716.2	ENSG00000224775.2
13342	ENSG00000172489.6	ENSG00000261715.1	ENSG00000199805.1
13343	ENSG00000172493.20	ENSG00000261714.2	ENSG00000259553.5
13344	ENSG00000172497.9	ENSG00000261713.6	ENSG00000186118.8
13345	ENSG00000172500.12	ENSG00000261711.6	ENSG00000267027.1
13346	ENSG00000172508.10	ENSG00000261710.1	ENSG00000277342.1
13347	ENSG00000172519.9	ENSG00000261709.3	ENSG00000182636.6
13348	ENSG00000172530.20	ENSG00000261708.1	ENSG00000267751.5
13349	ENSG00000172531.15	ENSG00000261707.1	ENSG00000235554.1
13350	ENSG00000172534.14	ENSG00000261706.1	ENSG00000168256.18
13351	ENSG00000172538.6	ENSG00000261704.2	ENSG00000269890.1
13352	ENSG00000172543.8	ENSG00000261703.1	ENSG00000177125.5
13353	ENSG00000172548.14	ENSG00000261702.2	ENSG00000011485.14

13354	ENSG00000172551.11	ENSG00000261701.7	ENSG00000255031.5
13355	ENSG00000172554.12	ENSG00000261697.5	ENSG00000261219.1
13356	ENSG00000172568.5	ENSG00000261696.1	ENSG00000277595.1
13357	ENSG00000172572.6	ENSG00000261695.1	ENSG00000230804.2
13358	ENSG00000172575.12	ENSG00000261692.1	ENSG00000255548.1
13359	ENSG00000172578.12	ENSG00000261691.1	ENSG00000236896.1
13360	ENSG00000172586.8	ENSG00000261689.1	ENSG00000147604.14
13361	ENSG00000172590.18	ENSG00000261687.1	ENSG00000233133.1
13362	ENSG00000172594.13	ENSG00000261684.2	ENSG00000278266.1
13363	ENSG00000172602.11	ENSG00000261683.1	ENSG00000230943.2
13364	ENSG00000172613.8	ENSG00000261682.1	ENSG00000144045.14
13365	ENSG00000172638.13	ENSG00000261680.1	ENSG00000260418.1
13366	ENSG00000172640.3	ENSG00000261679.2	ENSG00000235852.1
13367	ENSG00000172650.13	ENSG00000261678.3	ENSG00000133401.16
13368	ENSG00000172661.18	ENSG00000261675.1	ENSG00000255974.8
13369	ENSG00000172663.9	ENSG00000261673.1	ENSG00000232724.1
13370	ENSG00000172667.11	ENSG00000261671.1	ENSG00000226806.1
13371	ENSG00000172671.19	ENSG00000261670.1	ENSG00000101017.13
13372	ENSG00000172673.10	ENSG00000261669.1	ENSG00000271129.1
13373	ENSG00000172680.1	ENSG00000261668.1	ENSG00000138119.17
13374	ENSG00000172687.13	ENSG00000261667.1	ENSG00000260979.1
13375	ENSG00000172689.2	ENSG00000261666.1	ENSG00000273143.1
13376	ENSG00000172716.16	ENSG00000261665.2	ENSG00000213081.3
13377	ENSG00000172717.16	ENSG00000261664.5	ENSG00000144031.12
13378	ENSG00000172724.12	ENSG00000261663.1	ENSG00000249655.1
13379	ENSG00000172725.14	ENSG00000261659.2	ENSG00000249241.1
13380	ENSG00000172728.15	ENSG00000261656.5	ENSG00000254693.1
13381	ENSG00000172731.14	ENSG00000261655.1	ENSG00000280604.1
13382	ENSG00000172732.12	ENSG00000261653.1	ENSG00000266903.1
13383	ENSG00000172733.11	ENSG00000261652.2	ENSG00000234667.2
13384	ENSG00000172738.12	ENSG00000261651.1	ENSG00000260331.1
13385	ENSG00000172742.5	ENSG00000261650.1	ENSG00000258812.1
13386	ENSG00000172746.6	ENSG00000261649.6	ENSG00000234772.1
13387	ENSG00000172748.14	ENSG00000261648.1	ENSG00000128683.14
13388	ENSG00000172752.14	ENSG00000261647.1	ENSG00000251497.2
13389	ENSG00000172757.13	ENSG00000261646.1	ENSG00000241886.1
13390	ENSG00000172765.17	ENSG00000261645.6	ENSG00000236047.1
13391	ENSG00000172766.19	ENSG00000261644.2	ENSG00000163687.13
13392	ENSG00000172769.3	ENSG00000261642.1	ENSG00000143344.15
13393	ENSG00000172771.12	ENSG00000261641.2	ENSG00000232545.1
13394	ENSG00000172772.3	ENSG00000261638.1	ENSG00000181404.17
13395	ENSG00000172775.17	ENSG00000261637.2	ENSG00000213014.3
13396	ENSG00000172780.16	ENSG00000261636.1	ENSG00000278875.1
13397	ENSG00000172782.11	ENSG00000261635.2	ENSG00000230799.1
13398	ENSG00000172785.18	ENSG00000261634.3	ENSG00000239200.1
13399	ENSG00000172789.3	ENSG00000261633.1	ENSG00000275576.1
13400	ENSG00000172794.20	ENSG00000261632.1	ENSG00000242068.1
13401	ENSG00000172795.16	ENSG00000261630.1	ENSG00000163235.16
13402	ENSG00000172799.5	ENSG00000261629.1	ENSG00000101276.16
13403	ENSG00000172803.18	ENSG00000261628.1	ENSG00000147403.16
13404	ENSG00000172809.13	ENSG00000261627.1	ENSG00000279261.2
13405	ENSG00000172817.4	ENSG00000261625.1	ENSG00000169895.6
13406	ENSG00000172818.10	ENSG00000261624.1	ENSG00000229172.1

13407	ENSG00000172819.17	ENSG00000261623.1	ENSG00000085491.17
13408	ENSG00000172824.16	ENSG00000261620.1	ENSG00000173083.15
13409	ENSG00000172828.13	ENSG00000261618.2	ENSG00000271732.1
13410	ENSG00000172830.13	ENSG00000261617.1	ENSG00000227938.1
13411	ENSG00000172831.12	ENSG00000261616.1	ENSG00000238132.2
13412	ENSG00000172840.6	ENSG00000261615.6	ENSG00000242060.1
13413	ENSG00000172845.15	ENSG00000261614.1	ENSG00000273104.1
13414	ENSG00000172867.4	ENSG00000261613.2	ENSG00000274560.1
13415	ENSG00000172869.14	ENSG00000261612.1	ENSG00000273179.1
13416	ENSG00000172878.14	ENSG00000261611.6	ENSG00000214041.4
13417	ENSG00000172888.12	ENSG00000261610.1	ENSG00000136535.15
13418	ENSG00000172889.16	ENSG00000261609.6	ENSG00000168658.18
13419	ENSG00000172890.12	ENSG00000261608.1	ENSG00000197334.2
13420	ENSG00000172893.15	ENSG00000261607.1	ENSG00000197153.4
13421	ENSG00000172900.12	ENSG00000261606.5	ENSG00000188916.9
13422	ENSG00000172901.20	ENSG00000261604.1	ENSG00000170615.14
13423	ENSG00000172912.4	ENSG00000261603.2	ENSG00000249779.1
13424	ENSG00000172915.18	ENSG00000261602.1	ENSG00000178764.8
13425	ENSG00000172922.9	ENSG00000261600.1	ENSG00000127419.17
13426	ENSG00000172927.8	ENSG00000261599.6	ENSG00000225025.2
13427	ENSG00000172932.14	ENSG00000261596.2	ENSG00000005381.7
13428	ENSG00000172935.9	ENSG00000261595.1	ENSG00000263331.1
13429	ENSG00000172936.14	ENSG00000261594.3	ENSG00000108511.10
13430	ENSG00000172938.3	ENSG00000261593.1	ENSG00000222162.1
13431	ENSG00000172939.9	ENSG00000261592.1	ENSG00000225564.6
13432	ENSG00000172940.12	ENSG00000261590.1	ENSG00000254034.2
13433	ENSG00000172943.19	ENSG00000261588.1	ENSG00000277873.1
13434	ENSG00000172954.13	ENSG00000261587.2	ENSG00000201793.1
13435	ENSG00000172955.17	ENSG00000261586.2	ENSG00000185291.11
13436	ENSG00000172965.15	ENSG00000261584.1	ENSG00000170558.9
13437	ENSG00000172967.7	ENSG00000261583.1	ENSG00000132832.10
13438	ENSG00000172969.7	ENSG00000261582.1	ENSG00000236114.1
13439	ENSG00000172971.7	ENSG00000261581.1	ENSG00000260905.1
13440	ENSG00000172974.11	ENSG00000261580.1	ENSG00000235833.1
13441	ENSG00000172977.13	ENSG00000261578.1	ENSG00000226036.1
13442	ENSG00000172985.10	ENSG00000261575.2	ENSG00000186088.16
13443	ENSG00000172987.12	ENSG00000261573.1	ENSG00000213859.6
13444	ENSG00000172992.12	ENSG00000261570.1	ENSG00000175749.11
13445	ENSG00000172995.16	ENSG00000261569.5	ENSG00000263338.1
13446	ENSG00000173011.12	ENSG00000261566.1	ENSG00000272914.1
13447	ENSG00000173013.5	ENSG00000261564.1	ENSG00000267787.6
13448	ENSG00000173020.11	ENSG00000261561.1	ENSG00000271607.1
13449	ENSG00000173039.19	ENSG00000261560.1	ENSG00000115616.2
13450	ENSG00000173040.13	ENSG00000261559.1	ENSG00000212605.1
13451	ENSG00000173041.12	ENSG00000261558.1	ENSG00000264294.1
13452	ENSG00000173064.13	ENSG00000261557.1	ENSG00000231265.1
13453	ENSG00000173065.13	ENSG00000261556.9	ENSG00000261247.1
13454	ENSG00000173068.18	ENSG00000261555.1	ENSG00000276675.1
13455	ENSG00000173077.15	ENSG00000261554.1	ENSG00000253208.1
13456	ENSG00000173080.5	ENSG00000261553.5	ENSG00000242951.1
13457	ENSG00000173083.15	ENSG00000261552.1	ENSG00000101306.10
13458	ENSG00000173085.14	ENSG00000261549.2	ENSG00000273980.1
13459	ENSG00000173093.12	ENSG00000261548.1	ENSG00000229596.3

13460	ENSG00000173110.8	ENSG00000261546.1	ENSG00000255375.2
13461	ENSG00000173113.6	ENSG00000261544.1	ENSG00000225321.2
13462	ENSG00000173114.13	ENSG00000261543.1	ENSG00000159650.9
13463	ENSG00000173120.15	ENSG00000261542.1	ENSG00000122786.20
13464	ENSG00000173124.14	ENSG00000261541.1	ENSG00000237848.1
13465	ENSG00000173137.12	ENSG00000261540.1	ENSG00000131379.9
13466	ENSG00000173141.5	ENSG00000261538.1	ENSG00000254556.1
13467	ENSG00000173145.11	ENSG00000261537.1	ENSG00000241030.1
13468	ENSG00000173153.14	ENSG00000261536.2	ENSG00000136536.15
13469	ENSG00000173156.7	ENSG00000261535.1	ENSG00000229214.2
13470	ENSG00000173157.16	ENSG00000261534.1	ENSG00000122548.5
13471	ENSG00000173163.10	ENSG00000261532.1	ENSG00000237074.2
13472	ENSG00000173166.18	ENSG00000261529.1	ENSG00000042062.12
13473	ENSG00000173171.14	ENSG00000261528.2	ENSG00000126458.4
13474	ENSG00000173175.14	ENSG00000261527.1	ENSG00000271133.5
13475	ENSG00000173193.15	ENSG00000261526.2	ENSG00000275063.1
13476	ENSG00000173198.6	ENSG00000261524.1	ENSG00000257366.1
13477	ENSG00000173200.13	ENSG00000261523.1	ENSG00000280384.1
13478	ENSG00000173207.13	ENSG00000261522.5	ENSG00000233347.1
13479	ENSG00000173208.4	ENSG00000261520.5	ENSG00000271002.1
13480	ENSG00000173209.23	ENSG00000261519.3	ENSG00000281453.1
13481	ENSG00000173210.19	ENSG00000261517.1	ENSG00000137709.10
13482	ENSG00000173212.4	ENSG00000261515.1	ENSG00000211898.7
13483	ENSG00000173213.9	ENSG00000261514.1	ENSG00000254433.1
13484	ENSG00000173214.5	ENSG00000261513.1	ENSG00000251485.1
13485	ENSG00000173218.15	ENSG00000261512.2	ENSG00000213448.3
13486	ENSG00000173221.14	ENSG00000261511.1	ENSG00000259943.1
13487	ENSG00000173226.17	ENSG00000261509.6	ENSG00000230054.3
13488	ENSG00000173227.14	ENSG00000261507.1	ENSG00000269535.1
13489	ENSG00000173230.15	ENSG00000261505.1	ENSG00000168496.4
13490	ENSG00000173231.6	ENSG00000261504.1	ENSG00000268316.1
13491	ENSG00000173237.4	ENSG00000261502.3	ENSG00000227733.9
13492	ENSG00000173239.13	ENSG00000261501.1	ENSG00000002933.9
13493	ENSG00000173250.2	ENSG00000261499.2	ENSG00000011376.11
13494	ENSG00000173253.15	ENSG00000261498.1	ENSG00000250207.1
13495	ENSG00000173258.12	ENSG00000261497.1	ENSG00000240991.3
13496	ENSG00000173261.9	ENSG00000261491.1	ENSG00000272192.1
13497	ENSG00000173262.11	ENSG00000261490.1	ENSG00000244226.1
13498	ENSG00000173264.14	ENSG00000261489.1	ENSG00000101928.13
13499	ENSG00000173267.14	ENSG00000261488.1	ENSG00000128594.8
13500	ENSG00000173269.14	ENSG00000261487.1	ENSG00000275197.1
13501	ENSG00000173272.16	ENSG00000261486.1	ENSG00000266922.1
13502	ENSG00000173273.16	ENSG00000261485.1	ENSG00000145920.15
13503	ENSG00000173275.12	ENSG00000261482.1	ENSG00000249258.2
13504	ENSG00000173276.14	ENSG00000261481.1	ENSG00000251513.2
13505	ENSG00000173281.5	ENSG00000261480.1	ENSG00000248121.8
13506	ENSG00000173285.4	ENSG00000261478.1	ENSG00000229950.1
13507	ENSG00000173295.7	ENSG00000261476.1	ENSG00000237991.3
13508	ENSG00000173302.5	ENSG00000261475.2	ENSG00000165168.7
13509	ENSG00000173320.12	ENSG00000261474.1	ENSG00000132530.17
13510	ENSG00000173327.8	ENSG00000261472.1	ENSG00000277250.1
13511	ENSG00000173334.4	ENSG00000261471.1	ENSG00000279122.1
13512	ENSG00000173335.4	ENSG00000261470.1	ENSG00000259205.2

13513	ENSG00000173338.12	ENSG00000261469.1	ENSG00000242748.1
13514	ENSG00000173349.5	ENSG00000261468.1	ENSG00000237303.2
13515	ENSG00000173357.12	ENSG00000261467.2	ENSG00000156049.7
13516	ENSG00000173366.11	ENSG00000261466.2	ENSG00000235527.6
13517	ENSG00000173369.16	ENSG00000261465.6	ENSG00000149256.15
13518	ENSG00000173372.17	ENSG00000261462.1	ENSG00000233191.2
13519	ENSG00000173376.14	ENSG00000261460.1	ENSG00000224905.6
13520	ENSG00000173389.16	ENSG00000261459.1	ENSG00000270116.1
13521	ENSG00000173391.9	ENSG00000261458.1	ENSG00000214259.3
13522	ENSG00000173401.9	ENSG00000261457.3	ENSG00000234354.3
13523	ENSG00000173402.11	ENSG00000261456.5	ENSG00000256875.2
13524	ENSG00000173404.5	ENSG00000261455.1	ENSG00000227321.2
13525	ENSG00000173406.15	ENSG00000261453.1	ENSG00000214629.3
13526	ENSG00000173409.14	ENSG00000261451.1	ENSG00000222335.1
13527	ENSG00000173418.11	ENSG00000261449.1	ENSG00000215467.2
13528	ENSG00000173421.17	ENSG00000261448.1	ENSG00000159860.7
13529	ENSG00000173431.2	ENSG00000261447.1	ENSG00000223901.2
13530	ENSG00000173432.12	ENSG00000261446.3	ENSG00000268117.1
13531	ENSG00000173436.15	ENSG00000261445.1	ENSG00000255533.1
13532	ENSG00000173442.13	ENSG00000261444.1	ENSG00000237328.1
13533	ENSG00000173451.7	ENSG00000261442.1	ENSG00000230037.1
13534	ENSG00000173452.13	ENSG00000261441.1	ENSG00000082074.17
13535	ENSG00000173456.4	ENSG00000261440.1	ENSG00000081189.15
13536	ENSG00000173457.11	ENSG00000261439.1	ENSG00000228215.2
13537	ENSG00000173464.14	ENSG00000261438.1	ENSG00000236146.2
13538	ENSG00000173465.7	ENSG00000261437.1	ENSG00000260390.1
13539	ENSG00000173467.9	ENSG00000261436.5	ENSG00000275413.1
13540	ENSG00000173473.11	ENSG00000261435.1	ENSG00000176532.4
13541	ENSG00000173480.11	ENSG00000261434.1	ENSG00000271991.1
13542	ENSG00000173482.16	ENSG00000261433.1	ENSG00000267952.1
13543	ENSG00000173486.13	ENSG00000261432.1	ENSG00000275506.1
13544	ENSG00000173511.9	ENSG00000261431.1	ENSG00000255629.1
13545	ENSG00000173517.10	ENSG00000261430.1	ENSG00000235912.1
13546	ENSG00000173530.6	ENSG00000261429.2	ENSG00000231259.5
13547	ENSG00000173531.15	ENSG00000261427.6	ENSG00000278891.1
13548	ENSG00000173535.14	ENSG00000261426.1	ENSG00000257496.1
13549	ENSG00000173540.12	ENSG00000261424.1	ENSG00000275678.1
13550	ENSG00000173542.8	ENSG00000261423.1	ENSG00000104140.7
13551	ENSG00000173545.5	ENSG00000261421.1	ENSG00000186104.10
13552	ENSG00000173546.7	ENSG00000261420.1	ENSG00000259338.1
13553	ENSG00000173548.8	ENSG00000261419.1	ENSG00000115297.11
13554	ENSG00000173557.15	ENSG00000261418.1	ENSG00000136842.14
13555	ENSG00000173559.12	ENSG00000261416.1	ENSG00000234184.5
13556	ENSG00000173567.15	ENSG00000261411.1	ENSG00000229976.1
13557	ENSG00000173572.11	ENSG00000261410.1	ENSG00000275361.1
13558	ENSG00000173575.21	ENSG00000261409.1	ENSG00000231784.8
13559	ENSG00000173578.7	ENSG00000261408.7	ENSG00000240761.1
13560	ENSG00000173581.7	ENSG00000261407.1	ENSG00000255566.1
13561	ENSG00000173585.16	ENSG00000261405.2	ENSG00000255523.1
13562	ENSG00000173588.14	ENSG00000261404.7	ENSG00000276189.1
13563	ENSG00000173597.9	ENSG00000261403.1	ENSG00000266975.1
13564	ENSG00000173598.14	ENSG00000261402.1	ENSG00000167483.18
13565	ENSG00000173599.14	ENSG00000261401.1	ENSG00000244355.7

13566	ENSG00000173610.12	ENSG00000261400.1	ENSG00000198276.16
13567	ENSG00000173611.17	ENSG00000261399.1	ENSG00000105825.13
13568	ENSG00000173612.9	ENSG00000261398.1	ENSG00000279417.1
13569	ENSG00000173614.14	ENSG00000261397.1	ENSG00000256243.2
13570	ENSG00000173621.9	ENSG00000261396.1	ENSG00000263388.1
13571	ENSG00000173626.9	ENSG00000261395.3	ENSG00000172771.12
13572	ENSG00000173627.8	ENSG00000261394.1	ENSG00000197712.12
13573	ENSG00000173638.19	ENSG00000261393.1	ENSG00000257528.1
13574	ENSG00000173641.17	ENSG00000261392.1	ENSG00000248968.1
13575	ENSG00000173653.7	ENSG00000261391.1	ENSG00000185864.16
13576	ENSG00000173660.12	ENSG00000261390.5	ENSG00000251532.1
13577	ENSG00000173662.21	ENSG00000261385.1	ENSG00000244171.4
13578	ENSG00000173673.7	ENSG00000261384.1	ENSG00000243423.1
13579	ENSG00000173674.11	ENSG00000261382.1	ENSG00000264179.1
13580	ENSG00000173678.14	ENSG00000261379.1	ENSG00000102445.18
13581	ENSG00000173679.2	ENSG00000261377.5	ENSG00000220908.2
13582	ENSG00000173681.16	ENSG00000261376.1	ENSG00000010327.10
13583	ENSG00000173692.13	ENSG00000261375.1	ENSG00000114315.4
13584	ENSG00000173698.18	ENSG00000261373.1	ENSG00000140623.13
13585	ENSG00000173699.16	ENSG00000261371.6	ENSG00000162843.17
13586	ENSG00000173702.7	ENSG00000261369.1	ENSG00000259513.1
13587	ENSG00000173705.9	ENSG00000261368.1	ENSG00000263464.2
13588	ENSG00000173706.14	ENSG00000261367.1	ENSG00000258102.4
13589	ENSG00000173714.7	ENSG00000261366.1	ENSG00000268756.1
13590	ENSG00000173715.16	ENSG00000261365.1	ENSG00000183474.15
13591	ENSG00000173726.11	ENSG00000261364.1	ENSG00000234367.1
13592	ENSG00000173727.12	ENSG00000261363.2	ENSG00000260934.1
13593	ENSG00000173728.10	ENSG00000261362.1	ENSG00000229168.4
13594	ENSG00000173744.17	ENSG00000261360.1	ENSG00000257846.2
13595	ENSG00000173757.9	ENSG00000261359.2	ENSG00000166548.15
13596	ENSG00000173762.8	ENSG00000261357.1	ENSG00000144331.20
13597	ENSG00000173769.4	ENSG00000261356.2	ENSG00000268120.1
13598	ENSG00000173786.17	ENSG00000261350.1	ENSG00000120051.15
13599	ENSG00000173801.17	ENSG00000261349.1	ENSG00000197882.3
13600	ENSG00000173805.15	ENSG00000261348.1	ENSG00000202354.1
13601	ENSG00000173809.18	ENSG00000261347.1	ENSG00000277173.1
13602	ENSG00000173810.9	ENSG00000261346.1	ENSG00000254842.6
13603	ENSG00000173811.10	ENSG00000261342.1	ENSG00000254859.1
13604	ENSG00000173812.11	ENSG00000261341.6	ENSG00000160766.14
13605	ENSG00000173818.17	ENSG00000261340.1	ENSG00000204188.7
13606	ENSG00000173821.19	ENSG00000261338.2	ENSG00000259768.5
13607	ENSG00000173825.7	ENSG00000261336.1	ENSG00000096996.15
13608	ENSG00000173826.14	ENSG00000261335.1	ENSG00000226789.1
13609	ENSG00000173838.12	ENSG00000261334.1	ENSG00000142319.18
13610	ENSG00000173846.13	ENSG00000261333.2	ENSG00000229308.1
13611	ENSG00000173848.19	ENSG00000261332.1	ENSG00000211654.2
13612	ENSG00000173852.14	ENSG00000261330.1	ENSG00000151287.17
13613	ENSG00000173862.3	ENSG00000261329.5	ENSG00000263590.2
13614	ENSG00000173867.10	ENSG00000261327.4	ENSG00000070601.10
13615	ENSG00000173868.11	ENSG00000261326.3	ENSG00000257513.7
13616	ENSG00000173875.13	ENSG00000261325.1	ENSG00000235420.7
13617	ENSG00000173889.16	ENSG00000261324.2	ENSG00000225096.2
13618	ENSG00000173890.17	ENSG00000261320.1	ENSG00000218617.1

13619	ENSG00000173894.11	ENSG00000261319.1	ENSG00000118257.16
13620	ENSG00000173898.13	ENSG00000261318.1	ENSG00000259544.1
13621	ENSG00000173905.8	ENSG00000261316.1	ENSG00000261807.2
13622	ENSG00000173908.8	ENSG00000261315.2	ENSG00000136715.18
13623	ENSG00000173914.12	ENSG00000261314.1	ENSG00000279130.1
13624	ENSG00000173915.16	ENSG00000261313.1	ENSG00000182888.5
13625	ENSG00000173917.10	ENSG00000261312.1	ENSG00000104960.15
13626	ENSG00000173918.15	ENSG00000261310.1	ENSG00000268992.1
13627	ENSG00000173926.6	ENSG00000261308.2	ENSG00000150316.12
13628	ENSG00000173928.2	ENSG00000261307.1	ENSG00000127399.15
13629	ENSG00000173930.9	ENSG00000261305.2	ENSG00000258680.2
13630	ENSG00000173933.20	ENSG00000261304.1	ENSG00000260318.1
13631	ENSG00000173947.14	ENSG00000261303.6	ENSG00000252391.1
13632	ENSG00000173950.16	ENSG00000261302.5	ENSG00000185615.15
13633	ENSG00000173954.8	ENSG00000261299.1	ENSG00000275278.1
13634	ENSG00000173960.14	ENSG00000261298.1	ENSG00000270828.1
13635	ENSG00000173966.7	ENSG00000261296.1	ENSG00000117862.12
13636	ENSG00000173976.15	ENSG00000261294.1	ENSG00000223773.7
13637	ENSG00000173988.12	ENSG00000261293.1	ENSG00000197358.9
13638	ENSG00000173991.5	ENSG00000261292.2	ENSG00000164530.15
13639	ENSG00000173992.9	ENSG00000261291.1	ENSG00000262094.1
13640	ENSG00000174004.6	ENSG00000261290.1	ENSG00000278611.1
13641	ENSG00000174007.8	ENSG00000261289.2	ENSG00000276131.1
13642	ENSG00000174010.9	ENSG00000261288.1	ENSG00000177697.19
13643	ENSG00000174013.8	ENSG00000261286.1	ENSG00000118473.22
13644	ENSG00000174015.10	ENSG00000261285.6	ENSG00000233471.2
13645	ENSG00000174016.11	ENSG00000261284.2	ENSG00000278903.3
13646	ENSG00000174021.11	ENSG00000261282.1	ENSG00000251377.1
13647	ENSG00000174028.6	ENSG00000261281.1	ENSG00000272163.1
13648	ENSG00000174032.17	ENSG00000261278.1	ENSG00000256162.2
13649	ENSG00000174038.13	ENSG00000261276.1	ENSG00000232021.6
13650	ENSG00000174059.16	ENSG00000261275.1	ENSG00000282785.1
13651	ENSG00000174080.11	ENSG00000261274.1	ENSG00000275286.1
13652	ENSG00000174099.11	ENSG00000261273.1	ENSG00000114251.14
13653	ENSG00000174106.3	ENSG00000261272.1	ENSG00000213851.3
13654	ENSG00000174109.5	ENSG00000261270.1	ENSG00000254631.5
13655	ENSG00000174123.11	ENSG00000261269.1	ENSG00000255608.1
13656	ENSG00000174125.8	ENSG00000261268.1	ENSG00000238222.3
13657	ENSG00000174130.12	ENSG00000261267.1	ENSG00000273711.2
13658	ENSG00000174132.9	ENSG00000261266.2	ENSG00000177556.12
13659	ENSG00000174136.12	ENSG00000261265.1	ENSG00000134138.20
13660	ENSG00000174137.12	ENSG00000261263.2	ENSG00000236333.3
13661	ENSG00000174145.8	ENSG00000261261.2	ENSG00000227609.2
13662	ENSG00000174151.14	ENSG00000261260.1	ENSG00000240776.1
13663	ENSG00000174156.15	ENSG00000261259.1	ENSG00000268812.3
13664	ENSG00000174165.8	ENSG00000261257.1	ENSG00000235225.1
13665	ENSG00000174171.5	ENSG00000261251.1	ENSG00000239888.3
13666	ENSG00000174173.7	ENSG00000261250.1	ENSG00000230291.5
13667	ENSG00000174175.17	ENSG00000261249.1	ENSG00000256618.2
13668	ENSG00000174177.13	ENSG00000261248.1	ENSG00000250081.1
13669	ENSG00000174197.16	ENSG00000261247.1	ENSG00000270670.1
13670	ENSG00000174206.12	ENSG00000261245.2	ENSG00000272991.1
13671	ENSG00000174225.14	ENSG00000261244.1	ENSG00000228369.2

13672	ENSG00000174226.9	ENSG00000261243.1	ENSG00000160194.18
13673	ENSG00000174227.16	ENSG00000261242.1	ENSG00000280376.1
13674	ENSG00000174231.17	ENSG00000261241.6	ENSG00000279713.1
13675	ENSG00000174233.11	ENSG00000261240.1	ENSG00000196758.3
13676	ENSG00000174236.3	ENSG00000261239.6	ENSG00000284649.1
13677	ENSG00000174238.14	ENSG00000261238.1	ENSG00000129226.14
13678	ENSG00000174243.10	ENSG00000261236.8	ENSG00000234491.1
13679	ENSG00000174255.6	ENSG00000261235.1	ENSG00000215302.8
13680	ENSG00000174276.6	ENSG00000261234.1	ENSG00000272620.1
13681	ENSG00000174279.4	ENSG00000261233.2	ENSG00000134864.10
13682	ENSG00000174282.12	ENSG00000261232.2	ENSG00000220842.6
13683	ENSG00000174292.12	ENSG00000261231.6	ENSG00000224611.1
13684	ENSG00000174306.21	ENSG00000261227.2	ENSG00000274529.5
13685	ENSG00000174307.6	ENSG00000261226.1	ENSG00000152457.18
13686	ENSG00000174325.5	ENSG00000261222.2	ENSG00000254373.1
13687	ENSG00000174326.11	ENSG00000261221.3	ENSG00000172031.7
13688	ENSG00000174327.6	ENSG00000261220.2	ENSG00000259347.6
13689	ENSG00000174332.5	ENSG00000261219.1	ENSG00000213343.5
13690	ENSG00000174339.2	ENSG00000261218.5	ENSG00000269106.1
13691	ENSG00000174343.6	ENSG00000261217.2	ENSG00000165566.12
13692	ENSG00000174348.13	ENSG00000261216.1	ENSG00000232173.2
13693	ENSG00000174353.17	ENSG00000261215.1	ENSG00000234106.3
13694	ENSG00000174358.16	ENSG00000261213.1	ENSG00000237793.3
13695	ENSG00000174365.19	ENSG00000261212.1	ENSG00000120332.15
13696	ENSG00000174370.9	ENSG00000261211.1	ENSG00000187492.9
13697	ENSG00000174371.17	ENSG00000261210.7	ENSG00000213513.3
13698	ENSG00000174373.16	ENSG00000261209.1	ENSG00000261394.1
13699	ENSG00000174384.9	ENSG00000261208.1	ENSG00000261072.2
13700	ENSG00000174403.15	ENSG00000261207.1	ENSG00000172113.9
13701	ENSG00000174405.13	ENSG00000261206.1	ENSG00000270532.1
13702	ENSG00000174407.13	ENSG00000261205.2	ENSG00000237978.5
13703	ENSG00000174408.8	ENSG00000261204.1	ENSG00000223722.3
13704	ENSG00000174417.2	ENSG00000261203.1	ENSG00000123095.6
13705	ENSG00000174418.7	ENSG00000261202.1	ENSG00000280299.1
13706	ENSG00000174428.17	ENSG00000261200.1	ENSG00000279464.1
13707	ENSG00000174429.4	ENSG00000261199.1	ENSG00000231445.1
13708	ENSG00000174437.17	ENSG00000261198.1	ENSG00000243955.6
13709	ENSG00000174442.12	ENSG00000261197.2	ENSG00000159479.17
13710	ENSG00000174444.15	ENSG00000261196.1	ENSG00000122692.9
13711	ENSG00000174446.13	ENSG00000261195.1	ENSG00000095970.16
13712	ENSG00000174448.8	ENSG00000261194.1	ENSG00000151247.12
13713	ENSG00000174450.11	ENSG00000261193.1	ENSG00000184995.7
13714	ENSG00000174453.9	ENSG00000261192.1	ENSG00000221533.1
13715	ENSG00000174456.14	ENSG00000261191.1	ENSG00000260948.1
13716	ENSG00000174460.4	ENSG00000261190.5	ENSG00000170683.6
13717	ENSG00000174469.22	ENSG00000261189.1	ENSG00000242256.3
13718	ENSG00000174473.16	ENSG00000261188.1	ENSG00000101405.3
13719	ENSG00000174482.10	ENSG00000261187.1	ENSG00000217239.3
13720	ENSG00000174483.20	ENSG00000261186.2	ENSG00000229388.1
13721	ENSG00000174485.15	ENSG00000261184.2	ENSG00000271225.1
13722	ENSG00000174495.12	ENSG00000261183.5	ENSG00000281691.1
13723	ENSG00000174498.14	ENSG00000261182.1	ENSG00000267314.1
13724	ENSG00000174500.13	ENSG00000261181.1	ENSG00000120329.6

13725	ENSG00000174501.14	ENSG00000261178.2	ENSG00000261298.1
13726	ENSG00000174502.19	ENSG00000261177.1	ENSG00000267015.1
13727	ENSG00000174514.13	ENSG00000261175.6	ENSG00000109158.11
13728	ENSG00000174516.15	ENSG00000261174.2	ENSG00000236841.7
13729	ENSG00000174521.7	ENSG00000261173.1	ENSG00000042445.14
13730	ENSG00000174527.9	ENSG00000261172.1	ENSG00000271482.1
13731	ENSG00000174529.7	ENSG00000261170.1	ENSG00000204610.13
13732	ENSG00000174547.13	ENSG00000261168.1	ENSG00000270021.1
13733	ENSG00000174562.13	ENSG00000261167.1	ENSG00000204706.14
13734	ENSG00000174564.13	ENSG00000261166.1	ENSG00000214432.9
13735	ENSG00000174567.8	ENSG00000261161.1	ENSG00000255929.5
13736	ENSG00000174572.3	ENSG00000261159.1	ENSG00000075131.10
13737	ENSG00000174574.16	ENSG00000261158.1	ENSG00000167695.15
13738	ENSG00000174576.10	ENSG00000261156.6	ENSG00000260174.1
13739	ENSG00000174579.4	ENSG00000261154.1	ENSG00000272694.1
13740	ENSG00000174586.11	ENSG00000261153.2	ENSG00000153107.13
13741	ENSG00000174599.5	ENSG00000261151.1	ENSG00000172974.11
13742	ENSG00000174600.14	ENSG00000261150.2	ENSG00000268942.2
13743	ENSG00000174606.14	ENSG00000261146.1	ENSG00000231725.1
13744	ENSG00000174607.11	ENSG00000261145.1	ENSG00000272384.1
13745	ENSG00000174611.12	ENSG00000261144.1	ENSG00000230397.1
13746	ENSG00000174628.16	ENSG00000261143.1	ENSG00000125534.10
13747	ENSG00000174640.13	ENSG00000261141.1	ENSG00000116151.14
13748	ENSG00000174652.19	ENSG00000261140.1	ENSG00000123080.11
13749	ENSG00000174667.4	ENSG00000261136.1	ENSG00000214121.4
13750	ENSG00000174669.12	ENSG00000261135.1	ENSG00000225364.1
13751	ENSG00000174672.16	ENSG00000261131.1	ENSG00000179761.12
13752	ENSG00000174677.4	ENSG00000261130.5	ENSG00000265558.1
13753	ENSG00000174678.9	ENSG00000261129.1	ENSG00000106799.13
13754	ENSG00000174680.9	ENSG00000261127.2	ENSG00000279573.1
13755	ENSG00000174684.7	ENSG00000261126.7	ENSG00000120437.8
13756	ENSG00000174695.10	ENSG00000261124.1	ENSG00000144648.16
13757	ENSG00000174697.5	ENSG00000261123.1	ENSG00000225492.6
13758	ENSG00000174705.13	ENSG00000261122.6	ENSG00000242477.1
13759	ENSG00000174715.7	ENSG00000261121.1	ENSG00000229399.1
13760	ENSG00000174718.12	ENSG00000261120.1	ENSG00000278259.4
13761	ENSG00000174720.16	ENSG00000261117.1	ENSG00000267151.5
13762	ENSG00000174721.9	ENSG00000261116.1	ENSG00000252774.1
13763	ENSG00000174738.13	ENSG00000261115.6	ENSG00000117724.13
13764	ENSG00000174740.8	ENSG00000261114.1	ENSG00000258735.1
13765	ENSG00000174744.14	ENSG00000261113.1	ENSG00000233609.3
13766	ENSG00000174748.20	ENSG00000261111.1	ENSG00000100644.17
13767	ENSG00000174749.6	ENSG00000261108.2	ENSG00000262118.1
13768	ENSG00000174775.17	ENSG00000261105.5	ENSG00000155252.13
13769	ENSG00000174776.11	ENSG00000261104.1	ENSG00000236445.4
13770	ENSG00000174780.16	ENSG00000261103.1	ENSG00000279989.1
13771	ENSG00000174788.10	ENSG00000261102.2	ENSG00000278979.1
13772	ENSG00000174791.11	ENSG00000261101.2	ENSG00000174403.15
13773	ENSG00000174792.10	ENSG00000261098.1	ENSG00000225527.1
13774	ENSG00000174796.12	ENSG00000261097.1	ENSG00000248192.1
13775	ENSG00000174799.10	ENSG00000261095.1	ENSG00000238084.4
13776	ENSG00000174804.4	ENSG00000261094.2	ENSG00000244607.6
13777	ENSG00000174807.4	ENSG00000261093.1	ENSG00000253607.1

13778	ENSG00000174808.12	ENSG00000261092.1	ENSG00000124557.12
13779	ENSG00000174827.13	ENSG00000261090.1	ENSG00000177663.13
13780	ENSG00000174837.15	ENSG00000261089.1	ENSG00000249388.2
13781	ENSG00000174839.13	ENSG00000261087.1	ENSG00000144649.9
13782	ENSG00000174840.9	ENSG00000261084.1	ENSG00000176986.16
13783	ENSG00000174842.17	ENSG00000261083.2	ENSG00000239272.1
13784	ENSG00000174844.14	ENSG00000261082.1	ENSG00000229048.5
13785	ENSG00000174851.16	ENSG00000261081.1	ENSG00000217783.2
13786	ENSG00000174871.11	ENSG00000261080.1	ENSG00000179456.10
13787	ENSG00000174876.16	ENSG00000261079.1	ENSG00000271018.1
13788	ENSG00000174885.12	ENSG00000261078.1	ENSG00000139044.11
13789	ENSG00000174886.13	ENSG00000261077.1	ENSG00000276728.1
13790	ENSG00000174891.13	ENSG00000261076.1	ENSG00000234272.1
13791	ENSG00000174898.16	ENSG00000261075.1	ENSG00000268545.1
13792	ENSG00000174899.11	ENSG00000261072.2	ENSG00000275713.2
13793	ENSG00000174903.16	ENSG00000261071.1	ENSG00000232640.1
13794	ENSG00000174912.7	ENSG00000261070.1	ENSG00000262503.1
13795	ENSG00000174914.2	ENSG00000261069.3	ENSG00000262516.1
13796	ENSG00000174915.12	ENSG00000261068.2	ENSG00000197210.7
13797	ENSG00000174917.8	ENSG00000261067.7	ENSG00000255138.1
13798	ENSG00000174928.16	ENSG00000261066.1	ENSG00000185973.11
13799	ENSG00000174930.4	ENSG00000261065.1	ENSG00000255737.2
13800	ENSG00000174937.4	ENSG00000261063.1	ENSG00000267361.1
13801	ENSG00000174938.14	ENSG00000261061.1	ENSG00000272395.7
13802	ENSG00000174939.11	ENSG00000261060.1	ENSG00000277801.1
13803	ENSG00000174943.11	ENSG00000261058.1	ENSG00000158816.15
13804	ENSG00000174944.9	ENSG00000261057.1	ENSG00000213514.2
13805	ENSG00000174945.13	ENSG00000261056.2	ENSG00000259915.2
13806	ENSG00000174946.6	ENSG00000261055.1	ENSG00000275389.1
13807	ENSG00000174948.6	ENSG00000261054.1	ENSG00000175084.11
13808	ENSG00000174950.11	ENSG00000261053.1	ENSG00000267632.1
13809	ENSG00000174951.11	ENSG00000261052.5	ENSG00000200235.1
13810	ENSG00000174953.14	ENSG00000261051.1	ENSG00000267808.1
13811	ENSG00000174957.1	ENSG00000261049.2	ENSG00000253317.1
13812	ENSG00000174963.17	ENSG00000261048.1	ENSG00000242170.3
13813	ENSG00000174970.5	ENSG00000261047.1	ENSG00000203811.1
13814	ENSG00000174977.8	ENSG00000261046.1	ENSG00000197291.8
13815	ENSG00000174982.3	ENSG00000261045.2	ENSG00000269958.1
13816	ENSG00000174989.13	ENSG00000261044.1	ENSG00000171951.5
13817	ENSG00000174990.6	ENSG00000261043.5	ENSG00000203879.12
13818	ENSG00000174992.7	ENSG00000261041.1	ENSG00000229088.1
13819	ENSG00000174996.11	ENSG00000261040.7	ENSG00000159128.14
13820	ENSG00000175003.14	ENSG00000261039.2	ENSG00000240579.1
13821	ENSG00000175018.12	ENSG00000261038.1	ENSG00000255734.1
13822	ENSG00000175029.17	ENSG00000261037.1	ENSG00000237731.1
13823	ENSG00000175040.6	ENSG00000261036.1	ENSG00000125810.10
13824	ENSG00000175048.17	ENSG00000261035.1	ENSG00000227729.4
13825	ENSG00000175054.15	ENSG00000261033.1	ENSG00000233183.2
13826	ENSG00000175061.17	ENSG00000261030.1	ENSG00000130720.13
13827	ENSG00000175063.17	ENSG00000261029.1	ENSG00000176868.2
13828	ENSG00000175065.11	ENSG00000261028.1	ENSG00000255476.1
13829	ENSG00000175066.16	ENSG00000261026.1	ENSG00000249631.5
13830	ENSG00000175073.8	ENSG00000261025.1	ENSG00000235932.2

13831	ENSG00000175077.5	ENSG00000261024.7	ENSG00000226577.1
13832	ENSG00000175084.11	ENSG00000261020.1	ENSG00000233718.7
13833	ENSG00000175087.10	ENSG00000261019.1	ENSG00000253770.1
13834	ENSG00000175093.5	ENSG00000261018.1	ENSG00000248213.3
13835	ENSG00000175097.7	ENSG00000261017.1	ENSG00000227107.1
13836	ENSG00000175104.15	ENSG00000261014.1	ENSG00000277882.1
13837	ENSG00000175105.7	ENSG00000261012.2	ENSG00000204805.9
13838	ENSG00000175106.16	ENSG00000261011.1	ENSG00000271568.1
13839	ENSG00000175110.11	ENSG00000261010.1	ENSG00000124215.16
13840	ENSG00000175115.12	ENSG00000261009.1	ENSG00000283743.2
13841	ENSG00000175121.11	ENSG00000261008.6	ENSG00000278981.1
13842	ENSG00000175130.7	ENSG00000261007.2	ENSG00000215120.2
13843	ENSG00000175137.11	ENSG00000261003.1	ENSG00000261124.1
13844	ENSG00000175143.4	ENSG00000261002.5	ENSG00000238245.2
13845	ENSG00000175147.12	ENSG00000261000.1	ENSG00000130956.14
13846	ENSG00000175155.9	ENSG00000260999.1	ENSG00000229212.8
13847	ENSG00000175161.13	ENSG00000260997.1	ENSG00000111087.10
13848	ENSG00000175164.15	ENSG00000260996.1	ENSG00000230666.5
13849	ENSG00000175166.17	ENSG00000260995.2	ENSG00000124507.11
13850	ENSG00000175170.15	ENSG00000260994.1	ENSG00000223896.2
13851	ENSG00000175175.6	ENSG00000260992.1	ENSG00000230709.1
13852	ENSG00000175182.15	ENSG00000260991.2	ENSG00000229944.3
13853	ENSG00000175183.10	ENSG00000260990.1	ENSG00000267319.1
13854	ENSG00000175189.4	ENSG00000260989.1	ENSG00000281756.1
13855	ENSG00000175193.13	ENSG00000260988.1	ENSG00000235049.1
13856	ENSG00000175197.12	ENSG00000260986.1	ENSG00000196850.6
13857	ENSG00000175198.17	ENSG00000260984.1	ENSG00000280166.1
13858	ENSG00000175202.4	ENSG00000260983.1	ENSG00000065989.16
13859	ENSG00000175203.15	ENSG00000260981.1	ENSG00000168348.4
13860	ENSG00000175206.10	ENSG00000260978.1	ENSG00000108688.11
13861	ENSG00000175213.2	ENSG00000260976.1	ENSG00000247151.7
13862	ENSG00000175215.11	ENSG00000260975.1	ENSG00000254887.1
13863	ENSG00000175216.14	ENSG00000260974.1	ENSG00000225828.1
13864	ENSG00000175220.12	ENSG00000260973.1	ENSG00000271623.1
13865	ENSG00000175221.15	ENSG00000260972.1	ENSG00000278376.1
13866	ENSG00000175224.16	ENSG00000260971.4	ENSG00000268472.2
13867	ENSG00000175229.7	ENSG00000260970.1	ENSG00000259031.1
13868	ENSG00000175262.14	ENSG00000260969.1	ENSG00000062038.14
13869	ENSG00000175264.8	ENSG00000260967.1	ENSG00000224420.3
13870	ENSG00000175265.17	ENSG00000260966.1	ENSG00000196091.13
13871	ENSG00000175267.15	ENSG00000260965.1	ENSG00000224939.1
13872	ENSG00000175274.18	ENSG00000260963.1	ENSG00000253291.1
13873	ENSG00000175279.22	ENSG00000260962.1	ENSG00000244493.1
13874	ENSG00000175283.7	ENSG00000260959.1	ENSG00000278590.1
13875	ENSG00000175287.19	ENSG00000260958.2	ENSG00000275542.1
13876	ENSG00000175294.5	ENSG00000260957.1	ENSG00000215021.8
13877	ENSG00000175302.5	ENSG00000260955.2	ENSG00000135441.7
13878	ENSG00000175305.17	ENSG00000260954.1	ENSG00000129048.6
13879	ENSG00000175309.15	ENSG00000260953.1	ENSG00000206634.1
13880	ENSG00000175311.7	ENSG00000260951.1	ENSG00000165028.12
13881	ENSG00000175315.3	ENSG00000260949.1	ENSG00000156269.4
13882	ENSG00000175318.12	ENSG00000260948.1	ENSG00000134061.5
13883	ENSG00000175319.3	ENSG00000260947.1	ENSG00000274064.1

13884	ENSG00000175322.11	ENSG00000260945.1	ENSG00000169733.12
13885	ENSG00000175324.10	ENSG00000260944.1	ENSG00000285283.1
13886	ENSG00000175325.2	ENSG00000260943.1	ENSG00000264672.5
13887	ENSG00000175329.12	ENSG00000260942.1	ENSG00000280231.1
13888	ENSG00000175334.8	ENSG00000260941.1	ENSG00000268266.1
13889	ENSG00000175336.10	ENSG00000260939.1	ENSG00000262412.1
13890	ENSG00000175344.17	ENSG00000260937.5	ENSG00000151704.15
13891	ENSG00000175348.11	ENSG00000260934.1	ENSG00000249047.2
13892	ENSG00000175352.11	ENSG00000260933.1	ENSG00000116977.18
13893	ENSG00000175354.20	ENSG00000260932.1	ENSG00000196741.5
13894	ENSG00000175356.13	ENSG00000260930.1	ENSG00000132972.19
13895	ENSG00000175376.9	ENSG00000260928.2	ENSG00000106853.20
13896	ENSG00000175387.16	ENSG00000260927.1	ENSG00000248885.1
13897	ENSG00000175390.14	ENSG00000260926.2	ENSG00000008394.13
13898	ENSG00000175395.16	ENSG00000260924.2	ENSG00000187325.5
13899	ENSG00000175414.7	ENSG00000260923.6	ENSG00000218839.5
13900	ENSG00000175416.13	ENSG00000260922.2	ENSG00000272872.1
13901	ENSG00000175426.11	ENSG00000260921.1	ENSG00000274001.1
13902	ENSG00000175445.16	ENSG00000260920.2	ENSG00000184166.2
13903	ENSG00000175449.14	ENSG00000260919.1	ENSG00000214773.1
13904	ENSG00000175455.15	ENSG00000260918.1	ENSG00000235371.1
13905	ENSG00000175463.11	ENSG00000260917.1	ENSG00000197249.13
13906	ENSG00000175467.15	ENSG00000260916.7	ENSG00000173077.15
13907	ENSG00000175470.20	ENSG00000260914.3	ENSG00000280062.1
13908	ENSG00000175471.19	ENSG00000260913.1	ENSG00000273230.1
13909	ENSG00000175482.9	ENSG00000260912.1	ENSG00000248583.1
13910	ENSG00000175485.2	ENSG00000260911.2	ENSG00000257752.1
13911	ENSG00000175489.10	ENSG00000260910.1	ENSG00000235286.1
13912	ENSG00000175497.16	ENSG00000260909.1	ENSG00000151498.11
13913	ENSG00000175505.11	ENSG00000260908.1	ENSG00000219355.2
13914	ENSG00000175509.8	ENSG00000260907.1	ENSG00000278825.1
13915	ENSG00000175513.9	ENSG00000260905.1	ENSG00000278987.1
13916	ENSG00000175514.2	ENSG00000260903.3	ENSG00000250565.7
13917	ENSG00000175518.6	ENSG00000260902.1	ENSG00000177839.6
13918	ENSG00000175520.9	ENSG00000260900.1	ENSG00000237892.1
13919	ENSG00000175535.6	ENSG00000260898.5	ENSG00000269973.1
13920	ENSG00000175536.7	ENSG00000260897.1	ENSG00000279031.1
13921	ENSG00000175538.10	ENSG00000260896.6	ENSG00000204991.11
13922	ENSG00000175544.13	ENSG00000260895.1	ENSG00000152117.17
13923	ENSG00000175548.9	ENSG00000260894.1	ENSG00000275647.1
13924	ENSG00000175550.8	ENSG00000260892.1	ENSG00000286010.1
13925	ENSG00000175556.16	ENSG00000260891.1	ENSG00000153132.13
13926	ENSG00000175564.13	ENSG00000260889.1	ENSG00000142530.10
13927	ENSG00000175567.9	ENSG00000260887.2	ENSG00000156170.13
13928	ENSG00000175573.7	ENSG00000260886.1	ENSG00000273599.1
13929	ENSG00000175575.12	ENSG00000260884.1	ENSG00000225712.1
13930	ENSG00000175581.13	ENSG00000260883.1	ENSG00000127564.17
13931	ENSG00000175582.19	ENSG00000260882.1	ENSG00000176531.10
13932	ENSG00000175591.11	ENSG00000260880.4	ENSG00000150276.8
13933	ENSG00000175592.8	ENSG00000260879.1	ENSG00000241061.3
13934	ENSG00000175595.14	ENSG00000260878.1	ENSG00000274076.1
13935	ENSG00000175600.15	ENSG00000260877.2	ENSG00000139278.10
13936	ENSG00000175602.3	ENSG00000260876.5	ENSG00000142871.17

13937	ENSG00000175604.2	ENSG00000260874.5	ENSG00000271271.5
13938	ENSG00000175606.11	ENSG00000260871.1	ENSG00000226572.1
13939	ENSG00000175611.11	ENSG00000260870.1	ENSG00000285978.1
13940	ENSG00000175619.3	ENSG00000260869.1	ENSG00000136698.8
13941	ENSG00000175634.15	ENSG00000260868.1	ENSG00000279322.1
13942	ENSG00000175643.10	ENSG00000260867.1	ENSG00000265399.1
13943	ENSG00000175646.4	ENSG00000260866.2	ENSG00000264954.1
13944	ENSG00000175658.4	ENSG00000260865.1	ENSG00000261592.1
13945	ENSG00000175662.17	ENSG00000260864.1	ENSG00000175265.17
13946	ENSG00000175664.10	ENSG00000260863.1	ENSG00000175182.15
13947	ENSG00000175676.15	ENSG00000260862.1	ENSG00000273141.1
13948	ENSG00000175691.9	ENSG00000260861.6	ENSG00000250492.1
13949	ENSG00000175697.10	ENSG00000260860.2	ENSG00000117411.16
13950	ENSG00000175699.14	ENSG00000260859.1	ENSG00000265683.1
13951	ENSG00000175701.10	ENSG00000260858.1	ENSG00000105499.14
13952	ENSG00000175707.9	ENSG00000260857.2	ENSG00000211950.2
13953	ENSG00000175711.8	ENSG00000260855.1	ENSG00000183775.11
13954	ENSG00000175718.9	ENSG00000260854.1	ENSG00000279623.1
13955	ENSG00000175727.14	ENSG00000260853.2	ENSG00000260650.1
13956	ENSG00000175728.4	ENSG00000260852.1	ENSG00000100300.18
13957	ENSG00000175730.8	ENSG00000260851.6	ENSG00000128656.14
13958	ENSG00000175741.5	ENSG00000260850.2	ENSG00000243338.3
13959	ENSG00000175745.13	ENSG00000260848.1	ENSG00000253953.2
13960	ENSG00000175746.6	ENSG00000260847.1	ENSG00000008083.14
13961	ENSG00000175749.11	ENSG00000260846.2	ENSG00000111653.20
13962	ENSG00000175756.13	ENSG00000260845.1	ENSG00000264635.1
13963	ENSG00000175764.14	ENSG00000260844.2	ENSG00000273320.1
13964	ENSG00000175766.13	ENSG00000260840.2	ENSG00000163220.11
13965	ENSG00000175768.13	ENSG00000260838.3	ENSG00000211958.2
13966	ENSG00000175773.13	ENSG00000260836.2	ENSG00000285797.1
13967	ENSG00000175779.2	ENSG00000260835.1	ENSG00000255725.2
13968	ENSG00000175782.10	ENSG00000260834.1	ENSG00000271151.1
13969	ENSG00000175785.13	ENSG00000260832.1	ENSG00000187714.7
13970	ENSG00000175787.16	ENSG00000260830.1	ENSG00000279509.1
13971	ENSG00000175792.11	ENSG00000260828.1	ENSG00000164758.7
13972	ENSG00000175793.12	ENSG00000260827.1	ENSG00000285966.1
13973	ENSG00000175800.5	ENSG00000260823.1	ENSG00000186329.9
13974	ENSG00000175806.15	ENSG00000260818.2	ENSG00000248159.1
13975	ENSG00000175809.5	ENSG00000260817.2	ENSG00000273920.1
13976	ENSG00000175820.3	ENSG00000260816.2	ENSG00000158815.11
13977	ENSG00000175826.12	ENSG00000260815.3	ENSG00000283648.1
13978	ENSG00000175832.13	ENSG00000260814.2	ENSG00000234718.7
13979	ENSG00000175841.8	ENSG00000260812.1	ENSG00000124194.16
13980	ENSG00000175854.12	ENSG00000260811.1	ENSG00000281332.1
13981	ENSG00000175857.8	ENSG00000260810.1	ENSG00000033800.13
13982	ENSG00000175866.15	ENSG00000260809.1	ENSG00000280734.2
13983	ENSG00000175868.14	ENSG00000260808.1	ENSG00000143119.14
13984	ENSG00000175874.10	ENSG00000260807.6	ENSG00000231588.1
13985	ENSG00000175877.4	ENSG00000260806.1	ENSG00000159445.13
13986	ENSG00000175879.9	ENSG00000260805.2	ENSG00000269873.1
13987	ENSG00000175886.10	ENSG00000260804.3	ENSG00000219607.3
13988	ENSG00000175893.11	ENSG00000260803.1	ENSG00000196600.12
13989	ENSG00000175894.17	ENSG00000260802.1	ENSG00000204290.10

13990	ENSG00000175895.4	ENSG00000260799.1	ENSG00000260096.1
13991	ENSG00000175899.14	ENSG00000260798.1	ENSG00000237082.1
13992	ENSG00000175906.5	ENSG00000260797.1	ENSG00000277077.1
13993	ENSG00000175920.18	ENSG00000260796.1	ENSG00000232706.3
13994	ENSG00000175931.13	ENSG00000260795.1	ENSG00000269386.5
13995	ENSG00000175938.7	ENSG00000260793.2	ENSG00000273691.1
13996	ENSG00000175946.8	ENSG00000260792.1	ENSG00000131944.9
13997	ENSG00000175967.3	ENSG00000260790.1	ENSG00000265052.2
13998	ENSG00000175970.11	ENSG00000260788.5	ENSG00000235091.2
13999	ENSG00000175984.15	ENSG00000260786.1	ENSG00000233623.2
14000	ENSG00000175985.10	ENSG00000260785.1	ENSG00000259042.2
14001	ENSG00000176007.10	ENSG00000260784.1	ENSG00000252797.1
14002	ENSG00000176009.3	ENSG00000260782.1	ENSG00000230965.1
14003	ENSG00000176014.12	ENSG00000260781.1	ENSG00000223650.1
14004	ENSG00000176018.13	ENSG00000260780.1	ENSG00000185033.14
14005	ENSG00000176020.8	ENSG00000260779.1	ENSG00000169253.3
14006	ENSG00000176022.6	ENSG00000260778.5	ENSG00000283138.1
14007	ENSG00000176024.18	ENSG00000260777.1	ENSG00000117115.13
14008	ENSG00000176029.13	ENSG00000260776.5	ENSG00000213809.9
14009	ENSG00000176040.13	ENSG00000260774.1	ENSG00000213371.4
14010	ENSG00000176043.5	ENSG00000260773.1	ENSG00000183513.9
14011	ENSG00000176046.8	ENSG00000260771.1	ENSG00000237280.1
14012	ENSG00000176049.16	ENSG00000260765.2	ENSG00000282418.1
14013	ENSG00000176054.6	ENSG00000260764.1	ENSG00000260744.1
14014	ENSG00000176055.10	ENSG00000260763.1	ENSG00000138750.15
14015	ENSG00000176058.13	ENSG00000260762.1	ENSG00000267039.1
14016	ENSG00000176076.7	ENSG00000260761.1	ENSG00000233783.7
14017	ENSG00000176083.17	ENSG00000260759.1	ENSG00000075539.14
14018	ENSG00000176087.15	ENSG00000260757.1	ENSG00000105371.10
14019	ENSG00000176092.15	ENSG00000260756.1	ENSG00000177596.1
14020	ENSG00000176095.12	ENSG00000260755.1	ENSG00000168395.15
14021	ENSG00000176101.12	ENSG00000260751.2	ENSG00000006831.10
14022	ENSG00000176102.13	ENSG00000260750.5	ENSG00000284461.2
14023	ENSG00000176105.14	ENSG00000260747.1	ENSG00000275607.1
14024	ENSG00000176108.9	ENSG00000260746.2	ENSG00000170889.14
14025	ENSG00000176115.9	ENSG00000260744.1	ENSG00000169040.14
14026	ENSG00000176124.13	ENSG00000260743.1	ENSG00000160781.17
14027	ENSG00000176125.5	ENSG00000260742.1	ENSG00000211776.2
14028	ENSG00000176134.5	ENSG00000260741.1	ENSG00000236426.5
14029	ENSG00000176136.6	ENSG00000260740.2	ENSG00000274015.1
14030	ENSG00000176142.13	ENSG00000260739.1	ENSG00000251521.2
14031	ENSG00000176148.16	ENSG00000260738.1	ENSG00000261644.2
14032	ENSG00000176153.12	ENSG00000260737.5	ENSG00000268975.2
14033	ENSG00000176155.18	ENSG00000260735.2	ENSG00000272720.1
14034	ENSG00000176160.10	ENSG00000260734.5	ENSG00000165650.12
14035	ENSG00000176165.10	ENSG00000260733.1	ENSG00000204657.4
14036	ENSG00000176170.13	ENSG00000260731.1	ENSG00000249149.2
14037	ENSG00000176171.11	ENSG00000260729.1	ENSG00000205184.2
14038	ENSG00000176177.9	ENSG00000260727.1	ENSG00000240694.9
14039	ENSG00000176182.6	ENSG00000260726.2	ENSG00000228216.1
14040	ENSG00000176183.8	ENSG00000260725.1	ENSG00000122420.10
14041	ENSG00000176194.17	ENSG00000260724.1	ENSG00000237415.2
14042	ENSG00000176198.3	ENSG00000260723.1	ENSG00000164116.16

14043	ENSG00000176204.13	ENSG00000260722.1	ENSG00000286239.1
14044	ENSG00000176208.9	ENSG00000260721.1	ENSG00000254893.6
14045	ENSG00000176209.11	ENSG00000260720.1	ENSG00000008517.16
14046	ENSG00000176219.3	ENSG00000260719.1	ENSG00000271659.1
14047	ENSG00000176222.8	ENSG00000260715.1	ENSG00000196242.9
14048	ENSG00000176225.13	ENSG00000260714.1	ENSG00000262870.1
14049	ENSG00000176230.6	ENSG00000260711.2	ENSG00000250274.1
14050	ENSG00000176231.2	ENSG00000260710.1	ENSG00000189223.14
14051	ENSG00000176232.10	ENSG00000260708.1	ENSG00000271081.1
14052	ENSG00000176236.6	ENSG00000260706.5	ENSG00000241738.1
14053	ENSG00000176239.7	ENSG00000260704.1	ENSG00000182359.15
14054	ENSG00000176243.9	ENSG00000260702.3	ENSG00000179219.5
14055	ENSG00000176244.7	ENSG00000260701.1	ENSG00000258256.1
14056	ENSG00000176246.1	ENSG00000260698.1	ENSG00000092850.12
14057	ENSG00000176248.9	ENSG00000260695.1	ENSG00000272949.1
14058	ENSG00000176253.3	ENSG00000260694.1	ENSG00000230753.5
14059	ENSG00000176256.10	ENSG00000260691.6	ENSG00000261113.1
14060	ENSG00000176261.15	ENSG00000260690.1	ENSG00000230807.1
14061	ENSG00000176268.5	ENSG00000260689.2	ENSG00000149575.5
14062	ENSG00000176269.3	ENSG00000260688.1	ENSG00000274396.1
14063	ENSG00000176273.15	ENSG00000260686.1	ENSG00000235100.3
14064	ENSG00000176281.4	ENSG00000260685.1	ENSG00000134253.10
14065	ENSG00000176289.4	ENSG00000260683.1	ENSG00000188037.11
14066	ENSG00000176290.8	ENSG00000260682.3	ENSG00000168427.9
14067	ENSG00000176293.19	ENSG00000260681.1	ENSG00000197083.11
14068	ENSG00000176294.5	ENSG00000260680.1	ENSG00000229559.2
14069	ENSG00000176299.5	ENSG00000260679.1	ENSG00000176641.11
14070	ENSG00000176302.12	ENSG00000260678.1	ENSG00000277858.1
14071	ENSG00000176312.4	ENSG00000260677.1	ENSG00000254349.5
14072	ENSG00000176318.8	ENSG00000260676.5	ENSG00000178538.10
14073	ENSG00000176320.2	ENSG00000260675.1	ENSG00000229334.1
14074	ENSG00000176340.4	ENSG00000260674.2	ENSG00000070759.17
14075	ENSG00000176343.5	ENSG00000260673.1	ENSG00000124429.18
14076	ENSG00000176349.11	ENSG00000260672.1	ENSG00000167971.15
14077	ENSG00000176358.15	ENSG00000260671.2	ENSG00000259581.3
14078	ENSG00000176371.14	ENSG00000260670.1	ENSG00000104941.8
14079	ENSG00000176378.8	ENSG00000260669.2	ENSG00000211656.3
14080	ENSG00000176381.6	ENSG00000260668.1	ENSG00000260470.1
14081	ENSG00000176383.9	ENSG00000260664.2	ENSG00000129993.14
14082	ENSG00000176386.9	ENSG00000260662.2	ENSG00000241746.1
14083	ENSG00000176387.7	ENSG00000260661.1	ENSG00000081277.12
14084	ENSG00000176390.12	ENSG00000260660.4	ENSG00000201183.1
14085	ENSG00000176393.11	ENSG00000260659.1	ENSG00000158008.9
14086	ENSG00000176396.10	ENSG00000260658.5	ENSG00000265261.1
14087	ENSG00000176399.4	ENSG00000260657.2	ENSG00000231256.7
14088	ENSG00000176401.5	ENSG00000260653.5	ENSG00000241520.1
14089	ENSG00000176402.5	ENSG00000260650.1	ENSG00000144035.3
14090	ENSG00000176406.22	ENSG00000260649.1	ENSG00000268658.5
14091	ENSG00000176407.18	ENSG00000260648.3	ENSG00000181652.19
14092	ENSG00000176410.7	ENSG00000260647.1	ENSG00000230870.2
14093	ENSG00000176422.14	ENSG00000260646.1	ENSG00000236360.2
14094	ENSG00000176428.6	ENSG00000260645.2	ENSG00000267203.1
14095	ENSG00000176435.6	ENSG00000260644.6	ENSG00000228043.5

14096	ENSG00000176438.12	ENSG00000260643.2	ENSG00000115596.4
14097	ENSG00000176444.19	ENSG00000260642.1	ENSG00000143933.17
14098	ENSG00000176454.14	ENSG00000260641.1	ENSG00000228701.1
14099	ENSG00000176463.14	ENSG00000260640.1	ENSG00000198677.11
14100	ENSG00000176472.10	ENSG00000260639.1	ENSG00000214184.3
14101	ENSG00000176473.13	ENSG00000260635.1	ENSG00000174010.9
14102	ENSG00000176476.9	ENSG00000260633.1	ENSG00000277245.1
14103	ENSG00000176485.11	ENSG00000260631.1	ENSG00000286219.1
14104	ENSG00000176490.5	ENSG00000260630.6	ENSG00000201274.1
14105	ENSG00000176495.3	ENSG00000260629.2	ENSG00000011198.9
14106	ENSG00000176510.5	ENSG00000260628.5	ENSG00000267503.1
14107	ENSG00000176515.1	ENSG00000260626.1	ENSG00000204052.4
14108	ENSG00000176531.10	ENSG00000260625.3	ENSG00000091583.11
14109	ENSG00000176532.4	ENSG00000260624.1	ENSG00000158717.10
14110	ENSG00000176533.13	ENSG00000260622.1	ENSG00000140832.9
14111	ENSG00000176540.3	ENSG00000260621.1	ENSG00000167751.13
14112	ENSG00000176542.10	ENSG00000260620.1	ENSG00000223877.4
14113	ENSG00000176547.9	ENSG00000260619.1	ENSG00000259188.5
14114	ENSG00000176555.1	ENSG00000260618.1	ENSG00000235472.1
14115	ENSG00000176563.9	ENSG00000260617.1	ENSG00000203325.3
14116	ENSG00000176566.5	ENSG00000260616.6	ENSG00000169224.13
14117	ENSG00000176567.1	ENSG00000260615.1	ENSG00000261279.5
14118	ENSG00000176571.11	ENSG00000260612.1	ENSG00000267002.3
14119	ENSG00000176584.18	ENSG00000260611.1	ENSG00000005156.11
14120	ENSG00000176593.7	ENSG00000260610.1	ENSG00000228058.1
14121	ENSG00000176595.4	ENSG00000260608.1	ENSG00000261030.1
14122	ENSG00000176597.12	ENSG00000260605.1	ENSG00000259410.5
14123	ENSG00000176601.12	ENSG00000260604.2	ENSG00000279347.1
14124	ENSG00000176605.8	ENSG00000260602.2	ENSG00000262712.1
14125	ENSG00000176619.13	ENSG00000260601.1	ENSG00000146469.13
14126	ENSG00000176623.12	ENSG00000260600.1	ENSG00000183760.10
14127	ENSG00000176624.11	ENSG00000260599.1	ENSG00000179873.14
14128	ENSG00000176635.17	ENSG00000260598.1	ENSG00000248544.2
14129	ENSG00000176641.11	ENSG00000260597.1	ENSG00000100079.7
14130	ENSG00000176654.12	ENSG00000260596.5	ENSG00000154957.14
14131	ENSG00000176658.17	ENSG00000260595.1	ENSG00000105519.16
14132	ENSG00000176659.8	ENSG00000260594.1	ENSG00000231043.3
14133	ENSG00000176678.5	ENSG00000260593.1	ENSG00000135537.16
14134	ENSG00000176679.8	ENSG00000260590.1	ENSG00000133874.2
14135	ENSG00000176681.14	ENSG00000260589.1	ENSG00000176244.7
14136	ENSG00000176692.7	ENSG00000260588.1	ENSG00000067191.16
14137	ENSG00000176695.8	ENSG00000260586.2	ENSG00000224646.2
14138	ENSG00000176697.19	ENSG00000260585.1	ENSG00000251364.6
14139	ENSG00000176700.20	ENSG00000260584.1	ENSG00000164176.13
14140	ENSG00000176714.9	ENSG00000260583.1	ENSG00000182400.15
14141	ENSG00000176715.16	ENSG00000260582.1	ENSG00000184347.14
14142	ENSG00000176716.5	ENSG00000260581.1	ENSG00000090857.13
14143	ENSG00000176720.6	ENSG00000260580.1	ENSG00000226828.1
14144	ENSG00000176723.10	ENSG00000260579.1	ENSG00000102753.10
14145	ENSG00000176728.8	ENSG00000260578.1	ENSG00000275325.4
14146	ENSG00000176731.12	ENSG00000260577.2	ENSG00000214433.4
14147	ENSG00000176732.6	ENSG00000260576.1	ENSG00000099219.14
14148	ENSG00000176742.2	ENSG00000260575.1	ENSG00000184389.9

14149	ENSG00000176746.6	ENSG00000260574.1	ENSG00000152154.11
14150	ENSG00000176748.6	ENSG00000260573.3	ENSG00000125037.12
14151	ENSG00000176749.9	ENSG00000260572.1	ENSG00000122642.11
14152	ENSG00000176752.6	ENSG00000260571.1	ENSG00000272240.1
14153	ENSG00000176753.5	ENSG00000260570.1	ENSG00000277287.1
14154	ENSG00000176754.12	ENSG00000260569.2	ENSG00000213197.3
14155	ENSG00000176761.7	ENSG00000260568.1	ENSG00000204583.10
14156	ENSG00000176769.9	ENSG00000260566.2	ENSG00000104064.17
14157	ENSG00000176771.17	ENSG00000260565.6	ENSG00000267697.1
14158	ENSG00000176774.5	ENSG00000260564.1	ENSG00000105639.18
14159	ENSG00000176782.2	ENSG00000260563.3	ENSG00000272783.1
14160	ENSG00000176783.15	ENSG00000260558.2	ENSG00000185340.15
14161	ENSG00000176787.2	ENSG00000260555.1	ENSG00000267795.5
14162	ENSG00000176788.9	ENSG00000260552.1	ENSG00000229029.2
14163	ENSG00000176797.3	ENSG00000260551.1	ENSG00000204983.14
14164	ENSG00000176798.2	ENSG00000260550.2	ENSG00000236549.1
14165	ENSG00000176809.10	ENSG00000260549.1	ENSG00000258423.1
14166	ENSG00000176812.7	ENSG00000260548.1	ENSG00000188365.3
14167	ENSG00000176826.15	ENSG00000260545.1	ENSG00000105523.3
14168	ENSG00000176834.14	ENSG00000260542.1	ENSG00000271730.1
14169	ENSG00000176840.11	ENSG00000260541.1	ENSG00000262769.1
14170	ENSG00000176842.14	ENSG00000260540.2	ENSG00000180638.17
14171	ENSG00000176845.13	ENSG00000260537.2	ENSG00000227066.1
14172	ENSG00000176853.16	ENSG00000260536.1	ENSG00000263201.1
14173	ENSG00000176855.11	ENSG00000260532.1	ENSG00000228223.3
14174	ENSG00000176857.5	ENSG00000260530.1	ENSG00000167065.13
14175	ENSG00000176868.2	ENSG00000260528.4	ENSG00000179178.11
14176	ENSG00000176871.8	ENSG00000260526.1	ENSG00000259804.1
14177	ENSG00000176882.5	ENSG00000260525.1	ENSG00000225079.2
14178	ENSG00000176884.15	ENSG00000260524.1	ENSG00000248884.1
14179	ENSG00000176887.7	ENSG00000260522.1	ENSG00000230806.1
14180	ENSG00000176890.16	ENSG00000260520.1	ENSG00000259499.1
14181	ENSG00000176893.5	ENSG00000260519.1	ENSG00000224091.1
14182	ENSG00000176894.10	ENSG00000260518.2	ENSG00000267868.1
14183	ENSG00000176895.9	ENSG00000260517.3	ENSG00000035115.21
14184	ENSG00000176896.8	ENSG00000260516.2	ENSG00000285237.1
14185	ENSG00000176900.2	ENSG00000260515.1	ENSG00000165409.17
14186	ENSG00000176903.4	ENSG00000260514.1	ENSG00000233070.1
14187	ENSG00000176904.3	ENSG00000260511.1	ENSG00000271615.1
14188	ENSG00000176907.4	ENSG00000260510.1	ENSG00000231916.1
14189	ENSG00000176909.12	ENSG00000260509.2	ENSG00000239223.3
14190	ENSG00000176912.3	ENSG00000260507.1	ENSG00000200687.1
14191	ENSG00000176915.15	ENSG00000260505.1	ENSG00000207650.1
14192	ENSG00000176919.12	ENSG00000260504.1	ENSG00000272159.1
14193	ENSG00000176920.12	ENSG00000260500.1	ENSG00000255882.1
14194	ENSG00000176922.4	ENSG00000260498.5	ENSG00000256981.1
14195	ENSG00000176923.5	ENSG00000260497.1	ENSG00000229022.1
14196	ENSG00000176925.7	ENSG00000260496.3	ENSG00000184209.14
14197	ENSG00000176927.15	ENSG00000260495.1	ENSG00000236463.1
14198	ENSG00000176928.6	ENSG00000260494.1	ENSG00000231955.1
14199	ENSG00000176933.5	ENSG00000260492.2	ENSG00000133812.15
14200	ENSG00000176945.17	ENSG00000260490.2	ENSG00000108947.5
14201	ENSG00000176946.12	ENSG00000260488.1	ENSG00000185758.9

14202	ENSG00000176951.6	ENSG00000260487.1	ENSG00000036565.15
14203	ENSG00000176953.12	ENSG00000260484.1	ENSG00000243926.1
14204	ENSG00000176956.12	ENSG00000260483.2	ENSG00000234424.2
14205	ENSG00000176970.8	ENSG00000260482.3	ENSG00000272076.1
14206	ENSG00000176971.3	ENSG00000260480.1	ENSG00000125726.11
14207	ENSG00000176973.8	ENSG00000260479.1	ENSG00000240225.10
14208	ENSG00000176974.20	ENSG00000260478.1	ENSG00000177613.8
14209	ENSG00000176978.14	ENSG00000260477.7	ENSG00000259228.1
14210	ENSG00000176979.14	ENSG00000260476.1	ENSG00000250090.1
14211	ENSG00000176984.6	ENSG00000260473.2	ENSG00000123560.14
14212	ENSG00000176986.16	ENSG00000260472.1	ENSG00000249021.1
14213	ENSG00000176988.9	ENSG00000260471.1	ENSG00000268056.5
14214	ENSG00000176994.11	ENSG00000260470.1	ENSG00000251552.1
14215	ENSG00000176998.4	ENSG00000260469.2	ENSG00000136826.15
14216	ENSG00000177000.12	ENSG00000260468.1	ENSG00000286084.1
14217	ENSG00000177023.2	ENSG00000260467.1	ENSG00000115520.8
14218	ENSG00000177025.3	ENSG00000260466.1	ENSG00000270380.1
14219	ENSG00000177030.16	ENSG00000260465.1	ENSG00000100813.14
14220	ENSG00000177034.16	ENSG00000260464.1	ENSG00000213876.4
14221	ENSG00000177042.14	ENSG00000260461.1	ENSG00000270874.1
14222	ENSG00000177045.9	ENSG00000260460.1	ENSG00000248643.5
14223	ENSG00000177047.6	ENSG00000260459.2	ENSG00000132671.5
14224	ENSG00000177051.6	ENSG00000260458.3	ENSG00000258555.6
14225	ENSG00000177054.14	ENSG00000260457.2	ENSG00000278144.1
14226	ENSG00000177058.12	ENSG00000260456.6	ENSG00000267092.2
14227	ENSG00000177076.6	ENSG00000260455.1	ENSG00000257127.6
14228	ENSG00000177082.12	ENSG00000260454.1	ENSG00000230576.1
14229	ENSG00000177084.16	ENSG00000260452.1	ENSG00000138772.13
14230	ENSG00000177096.9	ENSG00000260451.1	ENSG00000203734.11
14231	ENSG00000177098.8	ENSG00000260450.1	ENSG00000267083.1
14232	ENSG00000177103.14	ENSG00000260447.1	ENSG00000186940.6
14233	ENSG00000177105.10	ENSG00000260445.1	ENSG00000240723.3
14234	ENSG00000177106.16	ENSG00000260444.1	ENSG00000116703.13
14235	ENSG00000177108.5	ENSG00000260443.1	ENSG00000082898.16
14236	ENSG00000177112.7	ENSG00000260442.5	ENSG00000130368.5
14237	ENSG00000177119.16	ENSG00000260441.5	ENSG00000265692.1
14238	ENSG00000177125.5	ENSG00000260440.2	ENSG00000117632.23
14239	ENSG00000177133.10	ENSG00000260438.1	ENSG00000125650.4
14240	ENSG00000177138.16	ENSG00000260436.1	ENSG00000239995.2
14241	ENSG00000177143.4	ENSG00000260433.2	ENSG00000234478.1
14242	ENSG00000177144.7	ENSG00000260431.2	ENSG00000104859.15
14243	ENSG00000177150.13	ENSG00000260430.1	ENSG00000133985.3
14244	ENSG00000177151.4	ENSG00000260428.3	ENSG00000197301.7
14245	ENSG00000177156.11	ENSG00000260427.1	ENSG00000174951.11
14246	ENSG00000177169.10	ENSG00000260426.1	ENSG00000176919.12
14247	ENSG00000177173.5	ENSG00000260425.1	ENSG00000267339.6
14248	ENSG00000177174.1	ENSG00000260423.1	ENSG00000276715.4
14249	ENSG00000177181.15	ENSG00000260422.1	ENSG00000273514.1
14250	ENSG00000177182.11	ENSG00000260420.2	ENSG00000284373.1
14251	ENSG00000177186.3	ENSG00000260419.1	ENSG00000105369.9
14252	ENSG00000177189.13	ENSG00000260418.1	ENSG00000163634.12
14253	ENSG00000177191.2	ENSG00000260417.1	ENSG00000149043.16
14254	ENSG00000177192.13	ENSG00000260416.1	ENSG00000227309.1

14255	ENSG00000177197.7	ENSG00000260414.1	ENSG00000135547.9
14256	ENSG00000177200.17	ENSG00000260413.1	ENSG00000229409.1
14257	ENSG00000177201.2	ENSG00000260412.1	ENSG00000250046.3
14258	ENSG00000177202.3	ENSG00000260410.2	ENSG00000259209.3
14259	ENSG00000177212.4	ENSG00000260409.1	ENSG00000247970.2
14260	ENSG00000177225.17	ENSG00000260406.1	ENSG00000240755.1
14261	ENSG00000177233.2	ENSG00000260405.1	ENSG00000183128.7
14262	ENSG00000177234.7	ENSG00000260404.3	ENSG00000164932.13
14263	ENSG00000177238.14	ENSG00000260403.1	ENSG00000100156.10
14264	ENSG00000177239.15	ENSG00000260402.1	ENSG00000215373.4
14265	ENSG00000177243.3	ENSG00000260401.1	ENSG00000266717.1
14266	ENSG00000177257.3	ENSG00000260400.1	ENSG00000243280.2
14267	ENSG00000177261.8	ENSG00000260399.1	ENSG00000180953.11
14268	ENSG00000177272.9	ENSG00000260398.1	ENSG00000132199.20
14269	ENSG00000177275.4	ENSG00000260395.1	ENSG00000250541.1
14270	ENSG00000177283.7	ENSG00000260394.2	ENSG00000257365.8
14271	ENSG00000177291.3	ENSG00000260393.1	ENSG00000181240.13
14272	ENSG00000177294.7	ENSG00000260392.5	ENSG00000129214.15
14273	ENSG00000177300.6	ENSG00000260391.2	ENSG00000255121.2
14274	ENSG00000177301.15	ENSG00000260390.1	ENSG00000273305.1
14275	ENSG00000177302.15	ENSG00000260389.1	ENSG00000251537.4
14276	ENSG00000177303.10	ENSG00000260388.2	ENSG00000278528.1
14277	ENSG00000177306.4	ENSG00000260387.1	ENSG00000131591.17
14278	ENSG00000177311.11	ENSG00000260386.6	ENSG00000285533.1
14279	ENSG00000177324.13	ENSG00000260385.1	ENSG00000285909.1
14280	ENSG00000177335.10	ENSG00000260382.1	ENSG00000184292.6
14281	ENSG00000177337.7	ENSG00000260381.2	ENSG00000267908.2
14282	ENSG00000177338.13	ENSG00000260379.1	ENSG00000153446.15
14283	ENSG00000177340.5	ENSG00000260378.1	ENSG00000131504.17
14284	ENSG00000177350.6	ENSG00000260377.1	ENSG00000267135.2
14285	ENSG00000177352.10	ENSG00000260375.1	ENSG00000258484.4
14286	ENSG00000177354.12	ENSG00000260372.6	ENSG00000215375.6
14287	ENSG00000177359.20	ENSG00000260371.1	ENSG00000188386.7
14288	ENSG00000177363.5	ENSG00000260370.1	ENSG00000257210.1
14289	ENSG00000177369.8	ENSG00000260369.2	ENSG00000089685.15
14290	ENSG00000177370.5	ENSG00000260368.1	ENSG00000275328.1
14291	ENSG00000177374.13	ENSG00000260367.2	ENSG00000204511.3
14292	ENSG00000177380.14	ENSG00000260366.1	ENSG00000183111.12
14293	ENSG00000177383.4	ENSG00000260364.1	ENSG00000234171.2
14294	ENSG00000177398.18	ENSG00000260362.1	ENSG00000277740.1
14295	ENSG00000177400.6	ENSG00000260360.1	ENSG00000232450.1
14296	ENSG00000177406.4	ENSG00000260357.1	ENSG00000242808.8
14297	ENSG00000177409.12	ENSG00000260352.1	ENSG00000259462.2
14298	ENSG00000177410.12	ENSG00000260351.1	ENSG00000224616.2
14299	ENSG00000177414.13	ENSG00000260350.1	ENSG00000226499.1
14300	ENSG00000177418.2	ENSG00000260349.1	ENSG00000274400.1
14301	ENSG00000177425.11	ENSG00000260348.1	ENSG00000232125.3
14302	ENSG00000177426.20	ENSG00000260347.1	ENSG00000256235.2
14303	ENSG00000177427.13	ENSG00000260345.1	ENSG00000227644.2
14304	ENSG00000177432.7	ENSG00000260344.2	ENSG00000260128.6
14305	ENSG00000177447.6	ENSG00000260343.1	ENSG00000263393.1
14306	ENSG00000177452.7	ENSG00000260342.2	ENSG00000254877.1
14307	ENSG00000177453.7	ENSG00000260341.1	ENSG00000250415.1

14308	ENSG00000177455.13	ENSG00000260340.1	ENSG00000228506.2
14309	ENSG00000177459.11	ENSG00000260339.1	ENSG00000276185.1
14310	ENSG00000177462.7	ENSG00000260338.1	ENSG00000176358.15
14311	ENSG00000177463.15	ENSG00000260337.3	ENSG00000205837.7
14312	ENSG00000177464.5	ENSG00000260335.1	ENSG00000276058.1
14313	ENSG00000177465.4	ENSG00000260332.1	ENSG00000272942.1
14314	ENSG00000177468.6	ENSG00000260331.1	ENSG00000196427.13
14315	ENSG00000177469.13	ENSG00000260329.1	ENSG00000277438.1
14316	ENSG00000177476.3	ENSG00000260328.1	ENSG00000078295.16
14317	ENSG00000177479.19	ENSG00000260327.2	ENSG00000249604.1
14318	ENSG00000177483.11	ENSG00000260326.1	ENSG00000228612.2
14319	ENSG00000177485.7	ENSG00000260325.1	ENSG00000184232.9
14320	ENSG00000177489.1	ENSG00000260322.1	ENSG00000109475.16
14321	ENSG00000177494.6	ENSG00000260318.1	ENSG00000236582.1
14322	ENSG00000177504.10	ENSG00000260317.1	ENSG00000283141.1
14323	ENSG00000177508.12	ENSG00000260316.2	ENSG00000119729.12
14324	ENSG00000177511.6	ENSG00000260314.3	ENSG00000242156.1
14325	ENSG00000177519.4	ENSG00000260312.1	ENSG00000258831.1
14326	ENSG00000177535.8	ENSG00000260311.1	ENSG00000258988.1
14327	ENSG00000177542.11	ENSG00000260310.1	ENSG00000217653.1
14328	ENSG00000177548.13	ENSG00000260308.1	ENSG00000262312.2
14329	ENSG00000177551.5	ENSG00000260307.2	ENSG00000227962.1
14330	ENSG00000177553.6	ENSG00000260306.1	ENSG00000257359.1
14331	ENSG00000177556.12	ENSG00000260305.1	ENSG00000276413.1
14332	ENSG00000177558.3	ENSG00000260304.1	ENSG00000153802.11
14333	ENSG00000177565.16	ENSG00000260303.1	ENSG00000240854.1
14334	ENSG00000177570.14	ENSG00000260302.2	ENSG00000256341.1
14335	ENSG00000177575.12	ENSG00000260300.5	ENSG00000162437.14
14336	ENSG00000177576.11	ENSG00000260298.2	ENSG00000182108.11
14337	ENSG00000177586.6	ENSG00000260296.1	ENSG00000179218.13
14338	ENSG00000177590.7	ENSG00000260293.2	ENSG00000224238.2
14339	ENSG00000177595.18	ENSG00000260291.1	ENSG00000232082.1
14340	ENSG00000177596.1	ENSG00000260290.2	ENSG00000142549.9
14341	ENSG00000177599.13	ENSG00000260289.1	ENSG00000000938.13
14342	ENSG00000177600.9	ENSG00000260288.3	ENSG00000116183.11
14343	ENSG00000177602.5	ENSG00000260287.4	ENSG00000232320.6
14344	ENSG00000177606.6	ENSG00000260286.3	ENSG00000279616.1
14345	ENSG00000177613.8	ENSG00000260285.1	ENSG00000241535.1
14346	ENSG00000177614.11	ENSG00000260284.1	ENSG00000168243.11
14347	ENSG00000177627.10	ENSG00000260282.1	ENSG00000266971.1
14348	ENSG00000177628.16	ENSG00000260281.5	ENSG00000159496.14
14349	ENSG00000177640.15	ENSG00000260280.5	ENSG00000235885.7
14350	ENSG00000177646.19	ENSG00000260279.3	ENSG00000249635.1
14351	ENSG00000177663.13	ENSG00000260278.1	ENSG00000260657.2
14352	ENSG00000177666.17	ENSG00000260277.1	ENSG00000243894.1
14353	ENSG00000177669.3	ENSG00000260276.2	ENSG00000105852.11
14354	ENSG00000177673.3	ENSG00000260275.1	ENSG00000279179.1
14355	ENSG00000177674.16	ENSG00000260274.1	ENSG00000237939.1
14356	ENSG00000177675.8	ENSG00000260273.1	ENSG00000231977.1
14357	ENSG00000177679.16	ENSG00000260272.1	ENSG00000115163.15
14358	ENSG00000177683.14	ENSG00000260271.2	ENSG00000171045.15
14359	ENSG00000177684.3	ENSG00000260269.5	ENSG00000276931.1
14360	ENSG00000177685.17	ENSG00000260268.5	ENSG00000261792.1

14361	ENSG00000177688.6	ENSG00000260267.1	ENSG00000229186.4
14362	ENSG00000177689.10	ENSG00000260266.1	ENSG00000166863.12
14363	ENSG00000177692.11	ENSG00000260265.1	ENSG00000223305.1
14364	ENSG00000177693.4	ENSG00000260264.1	ENSG00000249465.1
14365	ENSG00000177694.16	ENSG00000260262.1	ENSG00000215472.10
14366	ENSG00000177697.19	ENSG00000260261.2	ENSG00000226609.1
14367	ENSG00000177699.4	ENSG00000260260.1	ENSG00000064787.13
14368	ENSG00000177700.6	ENSG00000260259.1	ENSG00000231633.1
14369	ENSG00000177706.9	ENSG00000260258.2	ENSG00000180189.10
14370	ENSG00000177707.11	ENSG00000260257.2	ENSG00000187479.7
14371	ENSG00000177710.5	ENSG00000260256.1	ENSG00000236699.9
14372	ENSG00000177721.4	ENSG00000260255.1	ENSG00000255479.1
14373	ENSG00000177725.5	ENSG00000260254.1	ENSG00000242169.2
14374	ENSG00000177728.17	ENSG00000260253.1	ENSG00000100129.18
14375	ENSG00000177731.16	ENSG00000260252.1	ENSG00000258705.2
14376	ENSG00000177732.8	ENSG00000260251.1	ENSG00000232850.3
14377	ENSG00000177733.6	ENSG00000260249.2	ENSG00000258968.2
14378	ENSG00000177736.4	ENSG00000260247.1	ENSG00000228484.2
14379	ENSG00000177738.3	ENSG00000260246.1	ENSG00000271335.5
14380	ENSG00000177752.14	ENSG00000260244.1	ENSG00000183778.17
14381	ENSG00000177757.2	ENSG00000260242.1	ENSG00000279306.1
14382	ENSG00000177776.8	ENSG00000260240.4	ENSG00000096872.16
14383	ENSG00000177788.5	ENSG00000260239.1	ENSG00000265688.2
14384	ENSG00000177791.11	ENSG00000260238.6	ENSG00000283069.1
14385	ENSG00000177800.2	ENSG00000260237.1	ENSG00000256136.1
14386	ENSG00000177803.7	ENSG00000260236.1	ENSG00000161973.11
14387	ENSG00000177807.9	ENSG00000260235.1	ENSG00000248890.1
14388	ENSG00000177822.7	ENSG00000260234.5	ENSG00000231485.1
14389	ENSG00000177830.17	ENSG00000260233.3	ENSG00000108055.9
14390	ENSG00000177839.6	ENSG00000260232.2	ENSG00000268278.1
14391	ENSG00000177842.12	ENSG00000260230.3	ENSG00000259797.1
14392	ENSG00000177853.14	ENSG00000260229.1	ENSG00000196367.13
14393	ENSG00000177854.8	ENSG00000260228.5	ENSG00000269246.1
14394	ENSG00000177855.8	ENSG00000260224.1	ENSG00000183318.11
14395	ENSG00000177868.12	ENSG00000260223.1	ENSG00000237542.1
14396	ENSG00000177873.13	ENSG00000260220.6	ENSG00000157637.13
14397	ENSG00000177875.4	ENSG00000260219.2	ENSG00000261704.2
14398	ENSG00000177879.16	ENSG00000260218.1	ENSG00000278573.1
14399	ENSG00000177885.15	ENSG00000260217.1	ENSG00000151726.14
14400	ENSG00000177888.7	ENSG00000260213.6	ENSG00000249383.2
14401	ENSG00000177889.10	ENSG00000260212.1	ENSG00000253595.5
14402	ENSG00000177910.7	ENSG00000260211.2	ENSG00000282915.1
14403	ENSG00000177917.10	ENSG00000260209.1	ENSG00000168438.15
14404	ENSG00000177932.6	ENSG00000260207.1	ENSG00000196812.5
14405	ENSG00000177938.4	ENSG00000260206.1	ENSG00000144747.17
14406	ENSG00000177943.14	ENSG00000260205.1	ENSG00000285427.1
14407	ENSG00000177946.6	ENSG00000260202.2	ENSG00000235750.10
14408	ENSG00000177947.13	ENSG00000260201.2	ENSG00000255245.4
14409	ENSG00000177951.17	ENSG00000260198.1	ENSG00000256817.1
14410	ENSG00000177954.13	ENSG00000260197.1	ENSG00000197308.9
14411	ENSG00000177963.14	ENSG00000260196.1	ENSG00000183072.10
14412	ENSG00000177971.8	ENSG00000260194.1	ENSG00000271789.1
14413	ENSG00000177981.11	ENSG00000260193.1	ENSG00000165997.5

14414	ENSG00000177984.7	ENSG00000260192.2	ENSG00000261832.6
14415	ENSG00000177989.13	ENSG00000260190.1	ENSG00000167565.13
14416	ENSG00000177990.11	ENSG00000260188.1	ENSG00000268154.2
14417	ENSG00000177992.9	ENSG00000260187.2	ENSG00000164303.11
14418	ENSG00000177993.3	ENSG00000260186.5	ENSG00000229832.1
14419	ENSG00000177994.16	ENSG00000260185.1	ENSG00000229091.3
14420	ENSG00000178015.5	ENSG00000260184.1	ENSG00000004799.8
14421	ENSG00000178021.11	ENSG00000260183.1	ENSG00000187510.9
14422	ENSG00000178026.13	ENSG00000260182.1	ENSG00000133742.13
14423	ENSG00000178028.14	ENSG00000260179.1	ENSG00000169282.17
14424	ENSG00000178031.17	ENSG00000260177.1	ENSG00000266983.1
14425	ENSG00000178033.6	ENSG00000260176.1	ENSG00000254595.1
14426	ENSG00000178035.12	ENSG00000260174.1	ENSG00000214653.4
14427	ENSG00000178038.17	ENSG00000260173.1	ENSG00000197977.4
14428	ENSG00000178053.18	ENSG00000260172.1	ENSG00000255545.7
14429	ENSG00000178055.11	ENSG00000260171.1	ENSG00000163374.19
14430	ENSG00000178057.14	ENSG00000260170.1	ENSG00000109511.11
14431	ENSG00000178074.6	ENSG00000260167.1	ENSG00000161243.9
14432	ENSG00000178075.20	ENSG00000260166.1	ENSG00000242435.1
14433	ENSG00000178078.11	ENSG00000260165.1	ENSG00000268912.1
14434	ENSG00000178081.12	ENSG00000260163.1	ENSG00000266992.1
14435	ENSG00000178082.6	ENSG00000260162.2	ENSG00000172179.12
14436	ENSG00000178084.1	ENSG00000260161.1	ENSG00000101850.13
14437	ENSG00000178093.13	ENSG00000260160.1	ENSG00000179242.16
14438	ENSG00000178096.9	ENSG00000260159.1	ENSG00000271757.1
14439	ENSG00000178104.19	ENSG00000260158.1	ENSG00000150625.16
14440	ENSG00000178105.11	ENSG00000260157.3	ENSG00000260630.6
14441	ENSG00000178107.2	ENSG00000260156.1	ENSG00000224401.2
14442	ENSG00000178115.11	ENSG00000260153.1	ENSG00000256331.1
14443	ENSG00000178125.14	ENSG00000260152.2	ENSG00000236998.2
14444	ENSG00000178127.13	ENSG00000260151.1	ENSG00000265559.2
14445	ENSG00000178130.9	ENSG00000260148.1	ENSG00000226781.1
14446	ENSG00000178146.9	ENSG00000260147.1	ENSG00000250685.7
14447	ENSG00000178149.17	ENSG00000260146.1	ENSG00000272142.1
14448	ENSG00000178150.10	ENSG00000260145.1	ENSG00000274275.1
14449	ENSG00000178162.8	ENSG00000260144.1	ENSG00000123178.15
14450	ENSG00000178163.8	ENSG00000260142.1	ENSG00000253691.2
14451	ENSG00000178171.10	ENSG00000260141.1	ENSG00000270499.1
14452	ENSG00000178172.7	ENSG00000260139.6	ENSG00000211780.3
14453	ENSG00000178175.12	ENSG00000260137.1	ENSG00000259887.1
14454	ENSG00000178177.15	ENSG00000260136.5	ENSG00000276809.1
14455	ENSG00000178184.16	ENSG00000260134.1	ENSG00000211670.2
14456	ENSG00000178187.7	ENSG00000260133.1	ENSG00000218582.2
14457	ENSG00000178188.14	ENSG00000260132.1	ENSG00000249839.1
14458	ENSG00000178199.13	ENSG00000260131.1	ENSG00000261822.1
14459	ENSG00000178201.4	ENSG00000260128.6	ENSG00000167244.20
14460	ENSG00000178202.13	ENSG00000260126.1	ENSG00000273350.1
14461	ENSG00000178209.15	ENSG00000260125.1	ENSG00000235916.1
14462	ENSG00000178217.14	ENSG00000260123.1	ENSG00000148935.11
14463	ENSG00000178222.13	ENSG00000260122.1	ENSG00000144034.14
14464	ENSG00000178226.10	ENSG00000260121.1	ENSG00000265091.5
14465	ENSG00000178229.8	ENSG00000260120.1	ENSG00000120327.6
14466	ENSG00000178233.17	ENSG00000260118.1	ENSG00000196834.12

14467	ENSG00000178234.13	ENSG00000260115.1	ENSG00000187855.5
14468	ENSG00000178235.7	ENSG00000260113.3	ENSG00000258777.1
14469	ENSG00000178243.4	ENSG00000260112.1	ENSG00000226783.1
14470	ENSG00000178248.11	ENSG00000260111.1	ENSG00000261798.1
14471	ENSG00000178252.18	ENSG00000260109.1	ENSG00000181625.17
14472	ENSG00000178257.3	ENSG00000260108.1	ENSG00000198805.11
14473	ENSG00000178279.4	ENSG00000260107.1	ENSG00000111615.14
14474	ENSG00000178287.18	ENSG00000260105.6	ENSG00000279357.1
14475	ENSG00000178295.15	ENSG00000260104.1	ENSG00000187951.11
14476	ENSG00000178297.13	ENSG00000260103.2	ENSG00000203875.11
14477	ENSG00000178301.3	ENSG00000260102.1	ENSG00000101608.12
14478	ENSG00000178307.9	ENSG00000260101.1	ENSG00000257139.1
14479	ENSG00000178338.11	ENSG00000260100.1	ENSG00000231292.6
14480	ENSG00000178342.4	ENSG00000260097.2	ENSG00000128564.7
14481	ENSG00000178343.5	ENSG00000260096.1	ENSG00000228568.1
14482	ENSG00000178358.4	ENSG00000260095.1	ENSG00000258783.1
14483	ENSG00000178363.4	ENSG00000260094.1	ENSG00000228308.2
14484	ENSG00000178372.7	ENSG00000260093.1	ENSG00000233008.5
14485	ENSG00000178381.11	ENSG00000260091.1	ENSG00000125954.12
14486	ENSG00000178385.15	ENSG00000260090.1	ENSG00000257390.5
14487	ENSG00000178386.13	ENSG00000260089.2	ENSG00000240497.2
14488	ENSG00000178394.4	ENSG00000260088.1	ENSG00000256019.1
14489	ENSG00000178395.6	ENSG00000260087.1	ENSG00000273476.1
14490	ENSG00000178397.13	ENSG00000260086.2	ENSG00000247081.7
14491	ENSG00000178401.16	ENSG00000260084.1	ENSG00000176049.16
14492	ENSG00000178403.4	ENSG00000260083.1	ENSG00000087237.12
14493	ENSG00000178404.9	ENSG00000260082.1	ENSG00000224993.3
14494	ENSG00000178409.13	ENSG00000260081.2	ENSG00000258733.5
14495	ENSG00000178412.4	ENSG00000260077.1	ENSG00000154928.18
14496	ENSG00000178425.14	ENSG00000260075.1	ENSG00000100461.18
14497	ENSG00000178429.9	ENSG00000260073.2	ENSG00000227060.6
14498	ENSG00000178440.6	ENSG00000260072.1	ENSG00000072818.12
14499	ENSG00000178445.9	ENSG00000260071.1	ENSG00000258820.5
14500	ENSG00000178449.9	ENSG00000260070.1	ENSG00000170396.8
14501	ENSG00000178457.3	ENSG00000260068.1	ENSG00000262380.1
14502	ENSG00000178458.5	ENSG00000260067.1	ENSG00000134809.9
14503	ENSG00000178460.18	ENSG00000260066.1	ENSG00000230262.7
14504	ENSG00000178462.12	ENSG00000260064.1	ENSG00000112473.18
14505	ENSG00000178464.6	ENSG00000260063.1	ENSG00000230836.1
14506	ENSG00000178467.18	ENSG00000260062.3	ENSG00000163918.10
14507	ENSG00000178473.7	ENSG00000260060.1	ENSG00000260019.1
14508	ENSG00000178498.16	ENSG00000260059.1	ENSG00000212994.5
14509	ENSG00000178502.6	ENSG00000260058.1	ENSG00000267580.1
14510	ENSG00000178503.6	ENSG00000260057.5	ENSG00000186487.19
14511	ENSG00000178522.14	ENSG00000260053.2	ENSG00000229563.6
14512	ENSG00000178531.6	ENSG00000260052.1	ENSG00000283526.1
14513	ENSG00000178537.10	ENSG00000260051.1	ENSG00000141448.9
14514	ENSG00000178538.10	ENSG00000260048.2	ENSG00000006468.14
14515	ENSG00000178556.8	ENSG00000260047.2	ENSG00000262880.1
14516	ENSG00000178562.18	ENSG00000260046.1	ENSG00000227836.1
14517	ENSG00000178567.7	ENSG00000260042.1	ENSG00000277089.4
14518	ENSG00000178568.15	ENSG00000260041.5	ENSG00000158006.14
14519	ENSG00000178573.7	ENSG00000260038.1	ENSG00000226496.2

14520	ENSG00000178585.15	ENSG00000260037.5	ENSG00000229867.1
14521	ENSG00000178586.5	ENSG00000260036.1	ENSG00000180855.16
14522	ENSG00000178597.7	ENSG00000260035.1	ENSG00000233503.1
14523	ENSG00000178602.8	ENSG00000260034.1	ENSG00000138600.10
14524	ENSG00000178605.13	ENSG00000260033.1	ENSG00000245060.7
14525	ENSG00000178605.13_PAR_Y	ENSG00000260032.2	ENSG00000268845.1
14526	ENSG00000178607.16	ENSG00000260031.1	ENSG00000279885.1
14527	ENSG00000178623.12	ENSG00000260030.1	ENSG00000237984.3
14528	ENSG00000178631.7	ENSG00000260029.2	ENSG00000206448.3
14529	ENSG00000178636.8	ENSG00000260027.4	ENSG00000224430.2
14530	ENSG00000178645.12	ENSG00000260026.2	ENSG00000113532.13
14531	ENSG00000178654.11	ENSG00000260025.1	ENSG00000259528.1
14532	ENSG00000178660.6	ENSG00000260024.1	ENSG00000226948.2
14533	ENSG00000178662.16	ENSG00000260022.1	ENSG00000242255.1
14534	ENSG00000178665.16	ENSG00000260021.1	ENSG00000255843.1
14535	ENSG00000178685.14	ENSG00000260019.1	ENSG00000216490.4
14536	ENSG00000178690.3	ENSG00000260018.1	ENSG00000231680.1
14537	ENSG00000178691.11	ENSG00000260017.1	ENSG00000161912.18
14538	ENSG00000178694.10	ENSG00000260015.1	ENSG00000187135.7
14539	ENSG00000178695.5	ENSG00000260014.1	ENSG00000244675.2
14540	ENSG00000178700.7	ENSG00000260012.1	ENSG00000011105.14
14541	ENSG00000178715.8	ENSG00000260011.2	ENSG00000280778.1
14542	ENSG00000178718.6	ENSG00000260010.1	ENSG00000250321.1
14543	ENSG00000178719.17	ENSG00000260009.1	ENSG00000104972.15
14544	ENSG00000178722.12	ENSG00000260008.1	ENSG00000272536.2
14545	ENSG00000178723.7	ENSG00000260007.3	ENSG00000204256.13
14546	ENSG00000178726.6	ENSG00000260005.6	ENSG00000205436.7
14547	ENSG00000178732.5	ENSG00000260004.1	ENSG00000176343.5
14548	ENSG00000178734.5	ENSG00000260003.1	ENSG00000256222.2
14549	ENSG00000178741.12	ENSG00000260001.6	ENSG00000270429.1
14550	ENSG00000178750.3	ENSG00000260000.2	ENSG00000187474.5
14551	ENSG00000178752.16	ENSG00000259999.1	ENSG00000261141.1
14552	ENSG00000178761.15	ENSG00000259998.1	ENSG00000164683.17
14553	ENSG00000178762.4	ENSG00000259997.2	ENSG00000183307.3
14554	ENSG00000178764.8	ENSG00000259996.1	ENSG00000267421.6
14555	ENSG00000178772.7	ENSG00000259995.1	ENSG00000099284.14
14556	ENSG00000178773.15	ENSG00000259994.1	ENSG00000148204.12
14557	ENSG00000178776.5	ENSG00000259993.1	ENSG00000229054.1
14558	ENSG00000178789.9	ENSG00000259992.1	ENSG00000204352.3
14559	ENSG00000178795.9	ENSG00000259990.2	ENSG00000249884.8
14560	ENSG00000178796.12	ENSG00000259989.1	ENSG00000283312.1
14561	ENSG00000178802.18	ENSG00000259987.1	ENSG00000285920.1
14562	ENSG00000178803.11	ENSG00000259986.1	ENSG00000171433.12
14563	ENSG00000178804.7	ENSG00000259985.1	ENSG00000153922.10
14564	ENSG00000178809.11	ENSG00000259984.1	ENSG00000225416.1
14565	ENSG00000178814.17	ENSG00000259983.1	ENSG00000033627.16
14566	ENSG00000178821.13	ENSG00000259982.1	ENSG00000169490.17
14567	ENSG00000178826.11	ENSG00000259981.1	ENSG00000197558.11
14568	ENSG00000178828.7	ENSG00000259979.1	ENSG00000250115.2
14569	ENSG00000178836.6	ENSG00000259978.1	ENSG00000283867.1
14570	ENSG00000178852.16	ENSG00000259977.1	ENSG00000143248.13
14571	ENSG00000178878.12	ENSG00000259976.3	ENSG00000270917.1
14572	ENSG00000178882.14	ENSG00000259974.2	ENSG00000225940.6

14573	ENSG00000178896.9	ENSG00000259972.2	ENSG00000214335.3
14574	ENSG00000178904.19	ENSG00000259970.1	ENSG00000196114.3
14575	ENSG00000178913.7	ENSG00000259969.1	ENSG00000260271.2
14576	ENSG00000178917.16	ENSG00000259968.3	ENSG00000116690.12
14577	ENSG00000178919.8	ENSG00000259967.1	ENSG00000282034.1
14578	ENSG00000178921.14	ENSG00000259966.6	ENSG00000222179.1
14579	ENSG00000178922.16	ENSG00000259964.6	ENSG00000248648.1
14580	ENSG00000178927.18	ENSG00000259963.1	ENSG00000251380.3
14581	ENSG00000178928.8	ENSG00000259962.1	ENSG00000226526.1
14582	ENSG00000178934.4	ENSG00000259961.1	ENSG00000134183.11
14583	ENSG00000178935.5	ENSG00000259959.1	ENSG00000197696.10
14584	ENSG00000178947.9	ENSG00000259956.2	ENSG00000202392.1
14585	ENSG00000178950.17	ENSG00000259955.1	ENSG00000230826.1
14586	ENSG00000178951.9	ENSG00000259954.1	ENSG00000117407.17
14587	ENSG00000178952.11	ENSG00000259953.1	ENSG00000225216.6
14588	ENSG00000178965.14	ENSG00000259952.1	ENSG00000272368.2
14589	ENSG00000178966.16	ENSG00000259950.1	ENSG00000237154.2
14590	ENSG00000178971.15	ENSG00000259948.2	ENSG00000225536.2
14591	ENSG00000178974.10	ENSG00000259947.1	ENSG00000184270.5
14592	ENSG00000178977.3	ENSG00000259946.1	ENSG00000259103.2
14593	ENSG00000178980.15	ENSG00000259945.1	ENSG00000170515.14
14594	ENSG00000178982.9	ENSG00000259944.1	ENSG00000241877.1
14595	ENSG00000178988.11	ENSG00000259943.1	ENSG00000257563.1
14596	ENSG00000178996.14	ENSG00000259941.1	ENSG00000164236.12
14597	ENSG00000178997.11	ENSG00000259940.2	ENSG00000267834.1
14598	ENSG00000178999.13	ENSG00000259939.1	ENSG00000113594.10
14599	ENSG00000179002.5	ENSG00000259937.2	ENSG00000231217.1
14600	ENSG00000179008.9	ENSG00000259935.1	ENSG00000165805.10
14601	ENSG00000179010.14	ENSG00000259934.1	ENSG00000183379.9
14602	ENSG00000179021.10	ENSG00000259933.6	ENSG00000267369.1
14603	ENSG00000179023.8	ENSG00000259932.1	ENSG00000099958.14
14604	ENSG00000179028.4	ENSG00000259931.2	ENSG00000263740.2
14605	ENSG00000179029.14	ENSG00000259929.5	ENSG00000206192.7
14606	ENSG00000179031.8	ENSG00000259928.1	ENSG00000089177.18
14607	ENSG00000179038.8	ENSG00000259926.1	ENSG00000276368.1
14608	ENSG00000179041.4	ENSG00000259925.1	ENSG00000139173.10
14609	ENSG00000179044.16	ENSG00000259924.1	ENSG00000233040.2
14610	ENSG00000179046.8	ENSG00000259923.1	ENSG00000257109.3
14611	ENSG00000179051.14	ENSG00000259922.1	ENSG00000271511.1
14612	ENSG00000179055.7	ENSG00000259921.1	ENSG00000241959.3
14613	ENSG00000179057.13	ENSG00000259920.1	ENSG00000285188.1
14614	ENSG00000179058.7	ENSG00000259918.1	ENSG00000274383.1
14615	ENSG00000179059.10	ENSG00000259917.1	ENSG00000161610.1
14616	ENSG00000179066.8	ENSG00000259916.1	ENSG00000270061.1
14617	ENSG00000179071.5	ENSG00000259915.2	ENSG00000213937.4
14618	ENSG00000179073.6	ENSG00000259914.1	ENSG00000185198.11
14619	ENSG00000179082.3	ENSG00000259912.1	ENSG00000164167.12
14620	ENSG00000179083.6	ENSG00000259910.1	ENSG00000225111.1
14621	ENSG00000179085.7	ENSG00000259909.1	ENSG00000100003.18
14622	ENSG00000179088.14	ENSG00000259907.1	ENSG00000277349.1
14623	ENSG00000179091.5	ENSG00000259906.1	ENSG00000221963.6
14624	ENSG00000179094.16	ENSG00000259905.6	ENSG00000171786.6
14625	ENSG00000179097.5	ENSG00000259904.1	ENSG00000140675.13

14626	ENSG00000179101.5	ENSG00000259900.5	ENSG00000283288.1
14627	ENSG00000179104.9	ENSG00000259899.1	ENSG00000256269.9
14628	ENSG00000179111.9	ENSG00000259897.2	ENSG00000266973.1
14629	ENSG00000179115.11	ENSG00000259895.1	ENSG00000154134.15
14630	ENSG00000179119.15	ENSG00000259892.1	ENSG00000077684.16
14631	ENSG00000179131.7	ENSG00000259891.1	ENSG00000226070.1
14632	ENSG00000179133.13	ENSG00000259890.1	ENSG00000264016.2
14633	ENSG00000179134.15	ENSG00000259889.1	ENSG00000130544.12
14634	ENSG00000179136.6	ENSG00000259884.1	ENSG00000106868.16
14635	ENSG00000179141.9	ENSG00000259883.1	ENSG00000243063.1
14636	ENSG00000179142.2	ENSG00000259882.2	ENSG00000203397.2
14637	ENSG00000179144.5	ENSG00000259881.1	ENSG00000123575.9
14638	ENSG00000179148.9	ENSG00000259878.1	ENSG00000248664.1
14639	ENSG00000179151.13	ENSG00000259877.2	ENSG00000273209.1
14640	ENSG00000179152.20	ENSG00000259876.1	ENSG00000103184.12
14641	ENSG00000179157.4	ENSG00000259874.1	ENSG00000185271.8
14642	ENSG00000179163.11	ENSG00000259873.1	ENSG00000258529.5
14643	ENSG00000179165.11	ENSG00000259871.1	ENSG00000268854.1
14644	ENSG00000179168.14	ENSG00000259870.1	ENSG00000237226.1
14645	ENSG00000179170.11	ENSG00000259869.2	ENSG00000270577.1
14646	ENSG00000179172.9	ENSG00000259867.5	ENSG00000108960.9
14647	ENSG00000179178.11	ENSG00000259866.1	ENSG00000015413.9
14648	ENSG00000179195.16	ENSG00000259865.1	ENSG00000162949.16
14649	ENSG00000179213.13	ENSG00000259864.1	ENSG00000273139.1
14650	ENSG00000179218.13	ENSG00000259863.1	ENSG00000176055.10
14651	ENSG00000179219.5	ENSG00000259862.1	ENSG00000239437.3
14652	ENSG00000179222.17	ENSG00000259861.1	ENSG00000154553.15
14653	ENSG00000179240.9	ENSG00000259856.1	ENSG00000224903.1
14654	ENSG00000179241.13	ENSG00000259855.1	ENSG00000255342.1
14655	ENSG00000179242.16	ENSG00000259854.1	ENSG00000175203.15
14656	ENSG00000179253.3	ENSG00000259852.2	ENSG00000274427.1
14657	ENSG00000179256.2	ENSG00000259849.1	ENSG00000286146.1
14658	ENSG00000179262.10	ENSG00000259848.9	ENSG00000177576.11
14659	ENSG00000179270.7	ENSG00000259847.1	ENSG00000134779.15
14660	ENSG00000179271.3	ENSG00000259846.1	ENSG00000236312.3
14661	ENSG00000179277.9	ENSG00000259845.1	ENSG00000120833.14
14662	ENSG00000179284.5	ENSG00000259844.2	ENSG00000230487.8
14663	ENSG00000179292.5	ENSG00000259843.2	ENSG00000284957.1
14664	ENSG00000179295.17	ENSG00000259842.2	ENSG00000151006.7
14665	ENSG00000179299.17	ENSG00000259841.6	ENSG00000235145.2
14666	ENSG00000179300.3	ENSG00000259840.1	ENSG00000236226.1
14667	ENSG00000179304.16	ENSG00000259838.1	ENSG00000182866.17
14668	ENSG00000179314.14	ENSG00000259837.1	ENSG00000224208.1
14669	ENSG00000179331.3	ENSG00000259836.1	ENSG00000258658.1
14670	ENSG00000179335.18	ENSG00000259834.1	ENSG00000285231.1
14671	ENSG00000179342.4	ENSG00000259833.5	ENSG00000215878.3
14672	ENSG00000179344.16	ENSG00000259832.1	ENSG00000266953.6
14673	ENSG00000179348.11	ENSG00000259831.1	ENSG00000226872.1
14674	ENSG00000179361.17	ENSG00000259828.1	ENSG00000124782.20
14675	ENSG00000179362.14	ENSG00000259827.1	ENSG00000237828.1
14676	ENSG00000179363.7	ENSG00000259826.1	ENSG00000275427.1
14677	ENSG00000179364.13	ENSG00000259823.6	ENSG00000229587.2
14678	ENSG00000179387.10	ENSG00000259822.2	ENSG00000285244.1

14679	ENSG00000179388.9	ENSG00000259821.1	ENSG00000257923.11
14680	ENSG00000179397.18	ENSG00000259820.1	ENSG00000106415.13
14681	ENSG00000179399.15	ENSG00000259819.1	ENSG00000254985.1
14682	ENSG00000179403.12	ENSG00000259818.1	ENSG00000259788.1
14683	ENSG00000179406.7	ENSG00000259817.2	ENSG00000187954.12
14684	ENSG00000179407.3	ENSG00000259815.1	ENSG00000236867.1
14685	ENSG00000179409.11	ENSG00000259814.1	ENSG00000164144.16
14686	ENSG00000179412.10	ENSG00000259813.1	ENSG00000283706.2
14687	ENSG00000179420.11	ENSG00000259810.2	ENSG00000143457.11
14688	ENSG00000179428.2	ENSG00000259807.1	ENSG00000106125.14
14689	ENSG00000179431.6	ENSG00000259805.1	ENSG00000171794.4
14690	ENSG00000179443.5	ENSG00000259803.7	ENSG00000229905.1
14691	ENSG00000179447.2	ENSG00000259802.1	ENSG00000258724.1
14692	ENSG00000179452.2	ENSG00000259800.1	ENSG00000135211.6
14693	ENSG00000179454.13	ENSG00000259798.1	ENSG00000076826.9
14694	ENSG00000179455.8	ENSG00000259797.1	ENSG00000122687.17
14695	ENSG00000179456.10	ENSG00000259793.1	ENSG00000180015.12
14696	ENSG00000179460.5	ENSG00000259792.1	ENSG00000188672.18
14697	ENSG00000179468.5	ENSG00000259791.1	ENSG00000236242.1
14698	ENSG00000179476.8	ENSG00000259790.1	ENSG00000249188.1
14699	ENSG00000179477.10	ENSG00000259789.2	ENSG00000173163.10
14700	ENSG00000179520.10	ENSG00000259788.1	ENSG00000262484.1
14701	ENSG00000179523.4	ENSG00000259786.6	ENSG00000244677.3
14702	ENSG00000179526.17	ENSG00000259784.1	ENSG00000269792.1
14703	ENSG00000179528.16	ENSG00000259783.5	ENSG00000182021.10
14704	ENSG00000179532.12	ENSG00000259782.2	ENSG00000154642.11
14705	ENSG00000179542.16	ENSG00000259781.1	ENSG00000267107.7
14706	ENSG00000179546.4	ENSG00000259780.3	ENSG00000119943.13
14707	ENSG00000179562.3	ENSG00000259779.1	ENSG00000231976.8
14708	ENSG00000179564.4	ENSG00000259776.1	ENSG00000076356.7
14709	ENSG00000179571.10	ENSG00000259775.1	ENSG00000230658.1
14710	ENSG00000179577.3	ENSG00000259774.1	ENSG00000244371.2
14711	ENSG00000179580.10	ENSG00000259773.1	ENSG00000147485.13
14712	ENSG00000179583.19	ENSG00000259772.6	ENSG00000102387.15
14713	ENSG00000179588.9	ENSG00000259771.1	ENSG00000166482.11
14714	ENSG00000179593.16	ENSG00000259770.2	ENSG00000272936.1
14715	ENSG00000179598.6	ENSG00000259769.1	ENSG00000225449.3
14716	ENSG00000179600.3	ENSG00000259768.5	ENSG00000230528.7
14717	ENSG00000179603.17	ENSG00000259767.1	ENSG00000255992.1
14718	ENSG00000179604.10	ENSG00000259764.1	ENSG00000267293.1
14719	ENSG00000179611.3	ENSG00000259763.2	ENSG00000174606.14
14720	ENSG00000179615.2	ENSG00000259762.1	ENSG00000179262.10
14721	ENSG00000179626.4	ENSG00000259761.1	ENSG00000276916.1
14722	ENSG00000179627.10	ENSG00000259760.1	ENSG00000147010.18
14723	ENSG00000179630.11	ENSG00000259759.1	ENSG00000178722.12
14724	ENSG00000179632.10	ENSG00000259757.1	ENSG00000261527.1
14725	ENSG00000179636.15	ENSG00000259756.1	ENSG00000198258.10
14726	ENSG00000179639.10	ENSG00000259755.1	ENSG00000261076.1
14727	ENSG00000179673.5	ENSG00000259754.1	ENSG00000250338.1
14728	ENSG00000179674.3	ENSG00000259753.1	ENSG00000248996.1
14729	ENSG00000179676.6	ENSG00000259751.1	ENSG00000283360.1
14730	ENSG00000179695.2	ENSG00000259750.1	ENSG00000141434.11
14731	ENSG00000179698.13	ENSG00000259749.1	ENSG00000204792.2

14732	ENSG00000179709.8	ENSG00000259744.1	ENSG00000216412.4
14733	ENSG00000179715.13	ENSG00000259743.2	ENSG00000213519.2
14734	ENSG00000179743.4	ENSG00000259742.1	ENSG00000262803.1
14735	ENSG00000179750.16	ENSG00000259740.1	ENSG00000184933.5
14736	ENSG00000179751.6	ENSG00000259738.1	ENSG00000272656.1
14737	ENSG00000179755.3	ENSG00000259737.6	ENSG00000197930.13
14738	ENSG00000179761.12	ENSG00000259736.1	ENSG00000160563.14
14739	ENSG00000179766.19	ENSG00000259735.1	ENSG00000174567.8
14740	ENSG00000179774.8	ENSG00000259734.1	ENSG00000239374.1
14741	ENSG00000179776.19	ENSG00000259732.1	ENSG00000070367.16
14742	ENSG00000179796.12	ENSG00000259731.2	ENSG00000284484.1
14743	ENSG00000179799.9	ENSG00000259730.1	ENSG00000167202.11
14744	ENSG00000179813.7	ENSG00000259728.5	ENSG00000232518.3
14745	ENSG00000179817.5	ENSG00000259727.1	ENSG00000145425.10
14746	ENSG00000179818.13	ENSG00000259726.1	ENSG00000206228.4
14747	ENSG00000179820.16	ENSG00000259725.1	ENSG00000102760.13
14748	ENSG00000179826.6	ENSG00000259724.1	ENSG00000223972.5
14749	ENSG00000179832.17	ENSG00000259723.1	ENSG00000244705.1
14750	ENSG00000179833.4	ENSG00000259722.2	ENSG00000072657.8
14751	ENSG00000179840.5	ENSG00000259721.1	ENSG00000259807.1
14752	ENSG00000179841.8	ENSG00000259720.1	ENSG00000084710.14
14753	ENSG00000179846.9	ENSG00000259719.5	ENSG00000217027.1
14754	ENSG00000179855.7	ENSG00000259717.1	ENSG00000270538.1
14755	ENSG00000179859.9	ENSG00000259715.1	ENSG00000232778.1
14756	ENSG00000179862.6	ENSG00000259713.1	ENSG00000227550.2
14757	ENSG00000179869.15	ENSG00000259712.1	ENSG00000179344.16
14758	ENSG00000179873.14	ENSG00000259711.1	ENSG00000147488.11
14759	ENSG00000179886.5	ENSG00000259710.1	ENSG00000277977.1
14760	ENSG00000179889.18	ENSG00000259709.1	ENSG00000273983.1
14761	ENSG00000179899.8	ENSG00000259708.1	ENSG00000232591.1
14762	ENSG00000179902.12	ENSG00000259707.1	ENSG00000276810.1
14763	ENSG00000179909.15	ENSG00000259706.1	ENSG00000270659.1
14764	ENSG00000179912.20	ENSG00000259705.1	ENSG00000261649.6
14765	ENSG00000179913.10	ENSG00000259704.1	ENSG00000244151.1
14766	ENSG00000179914.5	ENSG00000259702.3	ENSG00000102804.15
14767	ENSG00000179915.23	ENSG00000259701.1	ENSG00000286035.1
14768	ENSG00000179918.18	ENSG00000259700.3	ENSG00000235204.1
14769	ENSG00000179919.3	ENSG00000259699.2	ENSG00000260518.2
14770	ENSG00000179921.15	ENSG00000259698.1	ENSG00000229853.1
14771	ENSG00000179922.5	ENSG00000259697.1	ENSG00000256982.1
14772	ENSG00000179930.5	ENSG00000259696.2	ENSG00000258012.1
14773	ENSG00000179933.6	ENSG00000259695.2	ENSG00000170356.9
14774	ENSG00000179934.6	ENSG00000259694.1	ENSG00000268112.1
14775	ENSG00000179935.9	ENSG00000259692.5	ENSG00000164087.7
14776	ENSG00000179938.12	ENSG00000259691.2	ENSG00000262497.1
14777	ENSG00000179941.8	ENSG00000259690.1	ENSG00000272465.1
14778	ENSG00000179943.8	ENSG00000259688.1	ENSG00000101464.10
14779	ENSG00000179950.13	ENSG00000259686.1	ENSG00000134014.17
14780	ENSG00000179954.16	ENSG00000259685.2	ENSG00000171517.6
14781	ENSG00000179958.8	ENSG00000259684.1	ENSG00000111727.12
14782	ENSG00000179965.11	ENSG00000259683.1	ENSG00000231645.2
14783	ENSG00000179967.11	ENSG00000259682.1	ENSG00000261302.5
14784	ENSG00000179978.11	ENSG00000259681.1	ENSG00000211460.12

14785	ENSG00000179981.10	ENSG00000259680.5	ENSG00000284048.2
14786	ENSG00000179988.14	ENSG00000259678.1	ENSG00000204525.16
14787	ENSG00000179994.11	ENSG00000259677.1	ENSG00000227301.1
14788	ENSG00000179997.8	ENSG00000259676.1	ENSG00000230311.2
14789	ENSG00000180008.9	ENSG00000259675.1	ENSG00000169877.10
14790	ENSG00000180011.7	ENSG00000259673.5	ENSG00000250994.1
14791	ENSG00000180015.12	ENSG00000259672.2	ENSG00000010278.14
14792	ENSG00000180016.2	ENSG00000259671.1	ENSG00000242798.1
14793	ENSG00000180019.5	ENSG00000259670.1	ENSG00000188483.7
14794	ENSG00000180035.12	ENSG00000259669.5	ENSG00000253190.3
14795	ENSG00000180042.5	ENSG00000259668.5	ENSG00000257803.2
14796	ENSG00000180043.11	ENSG00000259666.2	ENSG00000128805.14
14797	ENSG00000180044.5	ENSG00000259665.1	ENSG00000200310.1
14798	ENSG00000180053.7	ENSG00000259664.2	ENSG00000116017.11
14799	ENSG00000180061.9	ENSG00000259663.2	ENSG00000198408.14
14800	ENSG00000180066.9	ENSG00000259661.1	ENSG00000095752.7
14801	ENSG00000180068.10	ENSG00000259660.2	ENSG00000244627.5
14802	ENSG00000180071.20	ENSG00000259659.1	ENSG00000284060.1
14803	ENSG00000180083.10	ENSG00000259658.5	ENSG00000187994.14
14804	ENSG00000180089.5	ENSG00000259657.1	ENSG00000229994.1
14805	ENSG00000180090.5	ENSG00000259656.1	ENSG00000138587.6
14806	ENSG00000180096.12	ENSG00000259655.1	ENSG00000251669.5
14807	ENSG00000180098.9	ENSG00000259654.1	ENSG00000267662.1
14808	ENSG00000180105.11	ENSG00000259652.1	ENSG00000225979.1
14809	ENSG00000180113.16	ENSG00000259651.2	ENSG00000269102.1
14810	ENSG00000180116.15	ENSG00000259650.1	ENSG00000171385.9
14811	ENSG00000180138.7	ENSG00000259649.4	ENSG00000136824.19
14812	ENSG00000180139.11	ENSG00000259648.1	ENSG00000280213.1
14813	ENSG00000180150.5	ENSG00000259647.1	ENSG00000261789.1
14814	ENSG00000180152.3	ENSG00000259646.1	ENSG00000108702.3
14815	ENSG00000180155.20	ENSG00000259645.1	ENSG00000226245.1
14816	ENSG00000180172.7	ENSG00000259644.1	ENSG00000277502.1
14817	ENSG00000180176.14	ENSG00000259642.2	ENSG00000234969.1
14818	ENSG00000180178.11	ENSG00000259639.6	ENSG00000162040.6
14819	ENSG00000180182.11	ENSG00000259637.1	ENSG00000147509.14
14820	ENSG00000180185.11	ENSG00000259636.1	ENSG00000227039.6
14821	ENSG00000180189.10	ENSG00000259635.1	ENSG00000254046.1
14822	ENSG00000180190.11	ENSG00000259633.1	ENSG00000262772.1
14823	ENSG00000180198.16	ENSG00000259632.2	ENSG00000223724.1
14824	ENSG00000180205.3	ENSG00000259631.1	ENSG00000072954.7
14825	ENSG00000180209.11	ENSG00000259630.2	ENSG00000280058.1
14826	ENSG00000180210.14	ENSG00000259628.1	ENSG00000213412.3
14827	ENSG00000180211.5	ENSG00000259627.1	ENSG00000233837.1
14828	ENSG00000180219.2	ENSG00000259626.2	ENSG00000233087.7
14829	ENSG00000180221.6	ENSG00000259624.1	ENSG00000162592.10
14830	ENSG00000180228.13	ENSG00000259623.1	ENSG00000052749.13
14831	ENSG00000180229.12	ENSG00000259622.1	ENSG00000257954.1
14832	ENSG00000180230.6	ENSG00000259621.1	ENSG00000182325.11
14833	ENSG00000180233.11	ENSG00000259620.1	ENSG00000126861.5
14834	ENSG00000180245.5	ENSG00000259619.2	ENSG00000235268.2
14835	ENSG00000180251.4	ENSG00000259618.1	ENSG00000279423.1
14836	ENSG00000180257.13	ENSG00000259617.1	ENSG00000225798.1
14837	ENSG00000180259.9	ENSG00000259616.2	ENSG00000236484.2

14838	ENSG00000180263.14	ENSG00000259615.1	ENSG00000267756.1
14839	ENSG00000180264.11	ENSG00000259614.2	ENSG00000102802.10
14840	ENSG00000180269.7	ENSG00000259612.1	ENSG00000238150.1
14841	ENSG00000180279.7	ENSG00000259611.1	ENSG00000263938.1
14842	ENSG00000180284.5	ENSG00000259610.1	ENSG00000141086.18
14843	ENSG00000180287.16	ENSG00000259609.1	ENSG00000256436.1
14844	ENSG00000180304.14	ENSG00000259608.1	ENSG00000269600.1
14845	ENSG00000180305.5	ENSG00000259607.1	ENSG00000211445.12
14846	ENSG00000180316.12	ENSG00000259605.3	ENSG00000229727.6
14847	ENSG00000180318.4	ENSG00000259604.5	ENSG00000214248.2
14848	ENSG00000180329.14	ENSG00000259603.1	ENSG00000255441.1
14849	ENSG00000180332.6	ENSG00000259602.1	ENSG00000090487.11
14850	ENSG00000180336.18	ENSG00000259601.2	ENSG00000111837.11
14851	ENSG00000180340.6	ENSG00000259600.2	ENSG00000128250.5
14852	ENSG00000180346.3	ENSG00000259598.1	ENSG00000260304.1
14853	ENSG00000180347.13	ENSG00000259595.1	ENSG00000226360.5
14854	ENSG00000180353.11	ENSG00000259594.5	ENSG00000132207.17
14855	ENSG00000180354.16	ENSG00000259593.1	ENSG00000279884.1
14856	ENSG00000180357.9	ENSG00000259592.1	ENSG00000213239.3
14857	ENSG00000180370.10	ENSG00000259591.1	ENSG00000240912.1
14858	ENSG00000180376.16	ENSG00000259590.1	ENSG00000196177.12
14859	ENSG00000180383.3	ENSG00000259589.2	ENSG00000218069.2
14860	ENSG00000180385.8	ENSG00000259588.1	ENSG00000185559.15
14861	ENSG00000180386.7	ENSG00000259587.2	ENSG00000261610.1
14862	ENSG00000180389.7	ENSG00000259586.1	ENSG00000137198.9
14863	ENSG00000180398.12	ENSG00000259585.2	ENSG00000228509.5
14864	ENSG00000180409.3	ENSG00000259584.1	ENSG00000237993.1
14865	ENSG00000180422.3	ENSG00000259583.2	ENSG00000225406.1
14866	ENSG00000180423.4	ENSG00000259582.3	ENSG00000229891.1
14867	ENSG00000180424.7	ENSG00000259581.3	ENSG00000228060.2
14868	ENSG00000180425.11	ENSG00000259580.1	ENSG00000188559.15
14869	ENSG00000180432.5	ENSG00000259579.1	ENSG00000248896.2
14870	ENSG00000180433.5	ENSG00000259577.1	ENSG00000221855.1
14871	ENSG00000180437.5	ENSG00000259575.1	ENSG00000277705.1
14872	ENSG00000180438.15	ENSG00000259573.1	ENSG00000130307.11
14873	ENSG00000180440.4	ENSG00000259572.6	ENSG00000286032.1
14874	ENSG00000180447.7	ENSG00000259571.1	ENSG00000273356.1
14875	ENSG00000180448.10	ENSG00000259570.1	ENSG00000147592.9
14876	ENSG00000180458.2	ENSG00000259569.2	ENSG00000222044.1
14877	ENSG00000180475.4	ENSG00000259565.2	ENSG00000162105.18
14878	ENSG00000180479.13	ENSG00000259563.1	ENSG00000236562.4
14879	ENSG00000180481.11	ENSG00000259562.2	ENSG00000253263.1
14880	ENSG00000180483.7	ENSG00000259561.1	ENSG00000260903.3
14881	ENSG00000180488.15	ENSG00000259560.1	ENSG00000258120.1
14882	ENSG00000180509.12	ENSG00000259558.1	ENSG00000267289.1
14883	ENSG00000180525.12	ENSG00000259557.2	ENSG00000271079.1
14884	ENSG00000180530.11	ENSG00000259556.2	ENSG00000118491.9
14885	ENSG00000180532.10	ENSG00000259555.1	ENSG00000249693.2
14886	ENSG00000180535.3	ENSG00000259554.1	ENSG00000227536.1
14887	ENSG00000180537.13	ENSG00000259553.5	ENSG00000218208.1
14888	ENSG00000180539.7	ENSG00000259551.1	ENSG00000259632.2
14889	ENSG00000180543.4	ENSG00000259550.2	ENSG00000200312.1
14890	ENSG00000180549.7	ENSG00000259548.1	ENSG00000163501.7

14891	ENSG00000180573.9	ENSG00000259547.1	ENSG00000185245.8
14892	ENSG00000180574.3	ENSG00000259546.1	ENSG00000159173.19
14893	ENSG00000180581.7	ENSG00000259545.2	ENSG00000255404.1
14894	ENSG00000180592.16	ENSG00000259544.1	ENSG00000126822.17
14895	ENSG00000180596.7	ENSG00000259543.1	ENSG00000124900.12
14896	ENSG00000180610.10	ENSG00000259542.5	ENSG00000087269.16
14897	ENSG00000180611.7	ENSG00000259541.5	ENSG00000054793.13
14898	ENSG00000180613.11	ENSG00000259540.1	ENSG00000179284.5
14899	ENSG00000180616.9	ENSG00000259539.1	ENSG00000260391.2
14900	ENSG00000180626.10	ENSG00000259538.1	ENSG00000216316.1
14901	ENSG00000180628.15	ENSG00000259536.5	ENSG00000277182.1
14902	ENSG00000180638.17	ENSG00000259535.1	ENSG00000250075.6
14903	ENSG00000180644.8	ENSG00000259534.1	ENSG00000227295.2
14904	ENSG00000180658.4	ENSG00000259533.2	ENSG00000271410.1
14905	ENSG00000180660.7	ENSG00000259532.1	ENSG00000225802.3
14906	ENSG00000180662.8	ENSG00000259530.1	ENSG00000104412.8
14907	ENSG00000180663.5	ENSG00000259529.2	ENSG00000099715.14
14908	ENSG00000180667.10	ENSG00000259528.1	ENSG00000184349.13
14909	ENSG00000180673.9	ENSG00000259527.2	ENSG00000284610.1
14910	ENSG00000180694.14	ENSG00000259525.1	ENSG00000234789.1
14911	ENSG00000180697.9	ENSG00000259523.1	ENSG00000205100.2
14912	ENSG00000180708.5	ENSG00000259522.3	ENSG00000280207.1
14913	ENSG00000180712.3	ENSG00000259521.1	ENSG00000255639.3
14914	ENSG00000180714.3	ENSG00000259520.5	ENSG00000273084.1
14915	ENSG00000180720.7	ENSG00000259519.1	ENSG00000243679.1
14916	ENSG00000180723.6	ENSG00000259518.1	ENSG00000231857.1
14917	ENSG00000180730.4	ENSG00000259517.3	ENSG00000111907.21
14918	ENSG00000180739.14	ENSG00000259516.2	ENSG00000236301.5
14919	ENSG00000180745.5	ENSG00000259514.1	ENSG00000269050.1
14920	ENSG00000180747.15	ENSG00000259513.1	ENSG00000224637.1
14921	ENSG00000180758.12	ENSG00000259512.3	ENSG00000169193.12
14922	ENSG00000180764.13	ENSG00000259511.2	ENSG00000278075.1
14923	ENSG00000180767.9	ENSG00000259509.2	ENSG00000259445.1
14924	ENSG00000180769.9	ENSG00000259508.1	ENSG00000176383.9
14925	ENSG00000180770.3	ENSG00000259507.1	ENSG00000267426.5
14926	ENSG00000180773.15	ENSG00000259505.1	ENSG00000269696.1
14927	ENSG00000180776.15	ENSG00000259504.2	ENSG00000227725.3
14928	ENSG00000180777.13	ENSG00000259503.1	ENSG00000256092.2
14929	ENSG00000180785.10	ENSG00000259502.1	ENSG00000090238.11
14930	ENSG00000180787.6	ENSG00000259500.1	ENSG00000282828.1
14931	ENSG00000180801.14	ENSG00000259499.1	ENSG00000233845.1
14932	ENSG00000180803.3	ENSG00000259498.1	ENSG00000226268.3
14933	ENSG00000180806.5	ENSG00000259496.2	ENSG00000226838.1
14934	ENSG00000180815.14	ENSG00000259495.2	ENSG00000090621.14
14935	ENSG00000180817.12	ENSG00000259494.2	ENSG00000230699.2
14936	ENSG00000180818.5	ENSG00000259493.2	ENSG00000181847.12
14937	ENSG00000180822.11	ENSG00000259490.2	ENSG00000273056.1
14938	ENSG00000180828.2	ENSG00000259489.2	ENSG00000265496.5
14939	ENSG00000180834.7	ENSG00000259488.2	ENSG00000126803.9
14940	ENSG00000180846.8	ENSG00000259485.1	ENSG00000278224.6
14941	ENSG00000180855.16	ENSG00000259482.1	ENSG00000270846.1
14942	ENSG00000180861.9	ENSG00000259481.1	ENSG00000228812.7
14943	ENSG00000180867.11	ENSG00000259479.6	ENSG00000219932.6

14944	ENSG00000180869.4	ENSG00000259478.2	ENSG00000163154.6
14945	ENSG00000180871.8	ENSG00000259477.1	ENSG00000203326.11
14946	ENSG00000180872.5	ENSG00000259476.1	ENSG00000249319.2
14947	ENSG00000180875.5	ENSG00000259475.1	ENSG00000256196.1
14948	ENSG00000180878.2	ENSG00000259474.1	ENSG00000211819.3
14949	ENSG00000180879.13	ENSG00000259473.1	ENSG00000202411.1
14950	ENSG00000180881.19	ENSG00000259471.1	ENSG00000227751.1
14951	ENSG00000180884.10	ENSG00000259470.2	ENSG00000225518.2
14952	ENSG00000180891.13	ENSG00000259469.1	ENSG00000266680.1
14953	ENSG00000180900.19	ENSG00000259468.1	ENSG00000175063.17
14954	ENSG00000180901.11	ENSG00000259467.1	ENSG00000199691.1
14955	ENSG00000180902.18	ENSG00000259466.1	ENSG00000270977.1
14956	ENSG00000180909.2	ENSG00000259465.2	ENSG00000129465.16
14957	ENSG00000180910.8	ENSG00000259464.2	ENSG00000219395.2
14958	ENSG00000180913.3	ENSG00000259463.1	ENSG00000279058.2
14959	ENSG00000180914.10	ENSG00000259462.2	ENSG00000278158.1
14960	ENSG00000180917.17	ENSG00000259461.1	ENSG00000143107.9
14961	ENSG00000180919.3	ENSG00000259460.1	ENSG00000224490.5
14962	ENSG00000180921.7	ENSG00000259459.5	ENSG00000168070.12
14963	ENSG00000180926.6	ENSG00000259458.1	ENSG00000186086.18
14964	ENSG00000180929.6	ENSG00000259457.1	ENSG00000266111.2
14965	ENSG00000180934.6	ENSG00000259456.3	ENSG00000188002.10
14966	ENSG00000180938.6	ENSG00000259454.1	ENSG00000253690.1
14967	ENSG00000180953.11	ENSG00000259453.1	ENSG00000239280.1
14968	ENSG00000180957.17	ENSG00000259452.2	ENSG00000239377.1
14969	ENSG00000180964.17	ENSG00000259449.1	ENSG00000214243.3
14970	ENSG00000180974.4	ENSG00000259447.1	ENSG00000182791.5
14971	ENSG00000180979.10	ENSG00000259446.5	ENSG00000240771.7
14972	ENSG00000180988.2	ENSG00000259445.1	ENSG00000225971.1
14973	ENSG00000180992.7	ENSG00000259444.1	ENSG00000115935.17
14974	ENSG00000180998.12	ENSG00000259443.1	ENSG00000228076.2
14975	ENSG00000180999.11	ENSG00000259442.1	ENSG00000265574.1
14976	ENSG00000181001.2	ENSG00000259441.2	ENSG00000276396.1
14977	ENSG00000181004.10	ENSG00000259440.2	ENSG00000142700.12
14978	ENSG00000181007.8	ENSG00000259439.2	ENSG00000276855.1
14979	ENSG00000181009.5	ENSG00000259438.1	ENSG00000267030.1
14980	ENSG00000181013.3	ENSG00000259437.1	ENSG00000275400.1
14981	ENSG00000181016.9	ENSG00000259436.1	ENSG00000256211.1
14982	ENSG00000181017.5	ENSG00000259435.3	ENSG00000230207.1
14983	ENSG00000181019.13	ENSG00000259434.1	ENSG00000183833.16
14984	ENSG00000181023.7	ENSG00000259433.2	ENSG00000264503.1
14985	ENSG00000181026.15	ENSG00000259432.1	ENSG00000185291.11
14986	ENSG00000181027.10	ENSG00000259431.5	ENSG00000174137.12
14987	ENSG00000181029.9	ENSG00000259430.1	ENSG00000124449.7
14988	ENSG00000181031.15	ENSG00000259429.5	ENSG00000260515.1
14989	ENSG00000181035.13	ENSG00000259428.1	ENSG00000131848.9
14990	ENSG00000181036.14	ENSG00000259427.2	ENSG00000256955.2
14991	ENSG00000181038.13	ENSG00000259426.5	ENSG00000104549.12
14992	ENSG00000181045.15	ENSG00000259425.5	ENSG00000226004.1
14993	ENSG00000181061.13	ENSG00000259424.1	ENSG00000237387.1
14994	ENSG00000181072.11	ENSG00000259423.2	ENSG00000218418.2
14995	ENSG00000181074.4	ENSG00000259422.1	ENSG00000144040.12
14996	ENSG00000181085.15	ENSG00000259421.1	ENSG00000258561.1

14997	ENSG00000181090.21	ENSG00000259420.5	ENSG00000171155.8
14998	ENSG00000181092.10	ENSG00000259419.2	ENSG00000237550.5
14999	ENSG00000181097.5	ENSG00000259418.1	ENSG00000256192.1
15000	ENSG00000181101.7	ENSG00000259417.3	ENSG00000187416.12
15001	ENSG00000181104.7	ENSG00000259416.2	ENSG00000228981.3
15002	ENSG00000181109.3	ENSG00000259415.1	ENSG00000266932.1
15003	ENSG00000181123.8	ENSG00000259414.1	ENSG00000179918.18
15004	ENSG00000181126.13	ENSG00000259413.2	ENSG00000272477.1
15005	ENSG00000181135.16	ENSG00000259411.1	ENSG00000164949.8
15006	ENSG00000181143.15	ENSG00000259410.5	ENSG00000254726.3
15007	ENSG00000181163.13	ENSG00000259409.1	ENSG00000259143.1
15008	ENSG00000181171.5	ENSG00000259408.2	ENSG00000220418.1
15009	ENSG00000181191.11	ENSG00000259407.1	ENSG00000235141.1
15010	ENSG00000181192.12	ENSG00000259405.1	ENSG00000241889.1
15011	ENSG00000181195.11	ENSG00000259404.5	ENSG00000136052.9
15012	ENSG00000181201.3	ENSG00000259403.1	ENSG00000109118.14
15013	ENSG00000181211.1	ENSG00000259402.1	ENSG00000254236.1
15014	ENSG00000181214.7	ENSG00000259399.1	ENSG00000213640.3
15015	ENSG00000181215.15	ENSG00000259398.1	ENSG00000101280.8
15016	ENSG00000181218.5	ENSG00000259397.3	ENSG00000276188.1
15017	ENSG00000181220.17	ENSG00000259396.1	ENSG00000269959.1
15018	ENSG00000181222.15	ENSG00000259395.1	ENSG00000276674.1
15019	ENSG00000181227.3	ENSG00000259393.2	ENSG00000243103.3
15020	ENSG00000181234.9	ENSG00000259392.2	ENSG00000283698.1
15021	ENSG00000181240.13	ENSG00000259390.3	ENSG00000139549.4
15022	ENSG00000181260.8	ENSG00000259389.2	ENSG00000249087.6
15023	ENSG00000181264.8	ENSG00000259388.1	ENSG00000090932.10
15024	ENSG00000181273.2	ENSG00000259385.1	ENSG00000219626.9
15025	ENSG00000181274.6	ENSG00000259384.7	ENSG00000147364.16
15026	ENSG00000181282.4	ENSG00000259383.1	ENSG00000260880.4
15027	ENSG00000181284.2	ENSG00000259381.2	ENSG00000259912.1
15028	ENSG00000181291.7	ENSG00000259380.5	ENSG00000149646.12
15029	ENSG00000181296.3	ENSG00000259379.1	ENSG00000163354.15
15030	ENSG00000181315.11	ENSG00000259378.1	ENSG00000272333.5
15031	ENSG00000181322.14	ENSG00000259377.1	ENSG00000225705.2
15032	ENSG00000181323.8	ENSG00000259376.1	ENSG00000239763.2
15033	ENSG00000181325.7	ENSG00000259375.1	ENSG00000249986.1
15034	ENSG00000181333.11	ENSG00000259374.2	ENSG00000259378.1
15035	ENSG00000181350.11	ENSG00000259371.2	ENSG00000234785.1
15036	ENSG00000181355.21	ENSG00000259370.2	ENSG00000162104.10
15037	ENSG00000181358.3	ENSG00000259369.1	ENSG00000245680.10
15038	ENSG00000181359.5	ENSG00000259368.1	ENSG00000230789.1
15039	ENSG00000181371.4	ENSG00000259367.1	ENSG00000253854.1
15040	ENSG00000181374.8	ENSG00000259366.1	ENSG00000286193.1
15041	ENSG00000181378.14	ENSG00000259365.1	ENSG00000142544.7
15042	ENSG00000181381.13	ENSG00000259364.1	ENSG00000214719.12
15043	ENSG00000181392.16	ENSG00000259363.5	ENSG00000111786.9
15044	ENSG00000181396.13	ENSG00000259362.2	ENSG00000072201.13
15045	ENSG00000181404.17	ENSG00000259361.6	ENSG00000253676.1
15046	ENSG00000181408.3	ENSG00000259360.1	ENSG00000279009.1
15047	ENSG00000181409.13	ENSG00000259359.1	ENSG00000226877.8
15048	ENSG00000181418.8	ENSG00000259358.1	ENSG00000228007.1
15049	ENSG00000181433.9	ENSG00000259357.2	ENSG00000268191.1

15050	ENSG00000181444.13	ENSG00000259356.1	ENSG00000252800.1
15051	ENSG00000181449.4	ENSG00000259354.5	ENSG00000087085.15
15052	ENSG00000181450.17	ENSG00000259353.1	ENSG00000270988.1
15053	ENSG00000181458.10	ENSG00000259352.1	ENSG00000211957.2
15054	ENSG00000181467.4	ENSG00000259351.1	ENSG00000272979.1
15055	ENSG00000181472.5	ENSG00000259350.2	ENSG00000276950.5
15056	ENSG00000181481.14	ENSG00000259349.1	ENSG00000131669.10
15057	ENSG00000181499.2	ENSG00000259347.6	ENSG00000203411.3
15058	ENSG00000181511.5	ENSG00000259346.2	ENSG00000161594.6
15059	ENSG00000181513.14	ENSG00000259345.6	ENSG00000166946.14
15060	ENSG00000181514.6	ENSG00000259343.6	ENSG00000277013.1
15061	ENSG00000181518.4	ENSG00000259342.1	ENSG00000123307.4
15062	ENSG00000181523.13	ENSG00000259341.1	ENSG00000136383.6
15063	ENSG00000181524.6	ENSG00000259340.2	ENSG00000119979.18
15064	ENSG00000181541.5	ENSG00000259339.2	ENSG00000231310.3
15065	ENSG00000181544.15	ENSG00000259338.1	ENSG00000234118.1
15066	ENSG00000181552.3	ENSG00000259337.4	ENSG00000226160.1
15067	ENSG00000181555.20	ENSG00000259336.1	ENSG00000276597.1
15068	ENSG00000181562.4	ENSG00000259335.1	ENSG00000260228.5
15069	ENSG00000181577.16	ENSG00000259334.1	ENSG00000286155.1
15070	ENSG00000181585.5	ENSG00000259333.1	ENSG00000167588.13
15071	ENSG00000181588.16	ENSG00000259332.3	ENSG00000214562.14
15072	ENSG00000181609.5	ENSG00000259331.1	ENSG00000126952.16
15073	ENSG00000181610.13	ENSG00000259330.2	ENSG00000249474.1
15074	ENSG00000181616.9	ENSG00000259327.1	ENSG00000248988.1
15075	ENSG00000181617.5	ENSG00000259326.1	ENSG00000254701.3
15076	ENSG00000181619.11	ENSG00000259324.2	ENSG00000271387.1
15077	ENSG00000181625.17	ENSG00000259322.1	ENSG00000177119.16
15078	ENSG00000181626.11	ENSG00000259321.1	ENSG00000201207.1
15079	ENSG00000181631.7	ENSG00000259320.1	ENSG00000130021.13
15080	ENSG00000181634.8	ENSG00000259319.1	ENSG00000279594.1
15081	ENSG00000181638.18	ENSG00000259318.1	ENSG00000226928.1
15082	ENSG00000181649.7	ENSG00000259317.1	ENSG00000232702.3
15083	ENSG00000181652.19	ENSG00000259315.1	ENSG00000225152.1
15084	ENSG00000181656.7	ENSG00000259314.1	ENSG00000258048.1
15085	ENSG00000181666.18	ENSG00000259312.1	ENSG00000196946.10
15086	ENSG00000181690.8	ENSG00000259309.2	ENSG00000274068.1
15087	ENSG00000181693.8	ENSG00000259308.3	ENSG00000198885.9
15088	ENSG00000181698.4	ENSG00000259307.1	ENSG00000234622.6
15089	ENSG00000181704.12	ENSG00000259306.2	ENSG00000270072.1
15090	ENSG00000181705.2	ENSG00000259305.6	ENSG00000259658.5
15091	ENSG00000181718.6	ENSG00000259304.1	ENSG00000232445.1
15092	ENSG00000181722.16	ENSG00000259303.5	ENSG00000048342.16
15093	ENSG00000181733.3	ENSG00000259302.1	ENSG00000100312.10
15094	ENSG00000181741.7	ENSG00000259301.2	ENSG00000205581.11
15095	ENSG00000181744.8	ENSG00000259300.2	ENSG00000268163.1
15096	ENSG00000181751.10	ENSG00000259299.1	ENSG00000259468.1
15097	ENSG00000181752.3	ENSG00000259298.1	ENSG00000158406.4
15098	ENSG00000181754.7	ENSG00000259296.1	ENSG00000259531.2
15099	ENSG00000181761.5	ENSG00000259295.6	ENSG00000255847.5
15100	ENSG00000181767.3	ENSG00000259294.1	ENSG00000141449.14
15101	ENSG00000181773.7	ENSG00000259293.1	ENSG00000260690.1
15102	ENSG00000181778.4	ENSG00000259291.2	ENSG00000134940.13

15103	ENSG00000181780.4	ENSG00000259290.1	ENSG00000254682.1
15104	ENSG00000181781.9	ENSG00000259289.2	ENSG00000231728.3
15105	ENSG00000181785.2	ENSG00000259288.6	ENSG00000066032.18
15106	ENSG00000181786.4	ENSG00000259287.2	ENSG00000250806.2
15107	ENSG00000181788.4	ENSG00000259286.3	ENSG00000243064.8
15108	ENSG00000181789.14	ENSG00000259285.1	ENSG00000228238.1
15109	ENSG00000181790.11	ENSG00000259284.1	ENSG00000187952.9
15110	ENSG00000181798.2	ENSG00000259283.2	ENSG00000282358.1
15111	ENSG00000181800.5	ENSG00000259282.5	ENSG00000241112.1
15112	ENSG00000181803.3	ENSG00000259281.1	ENSG00000283638.2
15113	ENSG00000181804.15	ENSG00000259280.1	ENSG00000248112.1
15114	ENSG00000181817.6	ENSG00000259278.1	ENSG00000197870.12
15115	ENSG00000181819.14	ENSG00000259277.1	ENSG00000205572.9
15116	ENSG00000181826.10	ENSG00000259276.1	ENSG00000258535.1
15117	ENSG00000181827.14	ENSG00000259275.3	ENSG00000272381.1
15118	ENSG00000181830.8	ENSG00000259274.1	ENSG00000274253.4
15119	ENSG00000181837.7	ENSG00000259273.1	ENSG00000125851.10
15120	ENSG00000181847.12	ENSG00000259271.1	ENSG00000285756.1
15121	ENSG00000181852.17	ENSG00000259270.1	ENSG00000180739.14
15122	ENSG00000181856.14	ENSG00000259269.1	ENSG00000205060.11
15123	ENSG00000181867.3	ENSG00000259268.1	ENSG00000270325.1
15124	ENSG00000181873.13	ENSG00000259267.2	ENSG00000259583.2
15125	ENSG00000181885.18	ENSG00000259266.1	ENSG00000278861.1
15126	ENSG00000181894.15	ENSG00000259265.1	ENSG00000122367.19
15127	ENSG00000181896.12	ENSG00000259262.1	ENSG00000182013.18
15128	ENSG00000181903.5	ENSG00000259261.2	ENSG00000105767.3
15129	ENSG00000181904.9	ENSG00000259259.1	ENSG00000272945.1
15130	ENSG00000181908.5	ENSG00000259258.1	ENSG00000248810.1
15131	ENSG00000181915.4	ENSG00000259257.1	ENSG00000238225.1
15132	ENSG00000181924.7	ENSG00000259256.1	ENSG00000229862.5
15133	ENSG00000181929.12	ENSG00000259255.1	ENSG00000258615.1
15134	ENSG00000181938.13	ENSG00000259254.1	ENSG00000115361.8
15135	ENSG00000181939.3	ENSG00000259252.1	ENSG00000107643.16
15136	ENSG00000181943.5	ENSG00000259251.2	ENSG00000239819.2
15137	ENSG00000181950.3	ENSG00000259250.1	ENSG00000277142.1
15138	ENSG00000181958.4	ENSG00000259248.6	ENSG00000235605.1
15139	ENSG00000181961.4	ENSG00000259247.1	ENSG00000231164.1
15140	ENSG00000181963.5	ENSG00000259246.4	ENSG00000229798.1
15141	ENSG00000181965.6	ENSG00000259245.1	ENSG00000224251.6
15142	ENSG00000181977.3	ENSG00000259244.1	ENSG00000124207.17
15143	ENSG00000181982.18	ENSG00000259242.2	ENSG00000149451.17
15144	ENSG00000181984.11	ENSG00000259241.1	ENSG00000131044.17
15145	ENSG00000181991.16	ENSG00000259240.1	ENSG00000137463.5
15146	ENSG00000181995.12	ENSG00000259239.1	ENSG00000285639.1
15147	ENSG00000181997.8	ENSG00000259238.1	ENSG00000254739.1
15148	ENSG00000182000.9	ENSG00000259237.1	ENSG00000257038.1
15149	ENSG00000182004.13	ENSG00000259236.1	ENSG00000132424.16
15150	ENSG00000182010.11	ENSG00000259235.1	ENSG00000246263.2
15151	ENSG00000182013.18	ENSG00000259234.5	ENSG00000283045.1
15152	ENSG00000182021.10	ENSG00000259231.2	ENSG00000233558.1
15153	ENSG00000182022.18	ENSG00000259230.1	ENSG00000249713.1
15154	ENSG00000182035.12	ENSG00000259229.2	ENSG00000156136.10
15155	ENSG00000182040.9	ENSG00000259228.1	ENSG00000015520.14

15156	ENSG00000182048.11	ENSG00000259227.2	ENSG00000272426.1
15157	ENSG00000182050.13	ENSG00000259224.2	ENSG00000258130.8
15158	ENSG00000182053.12	ENSG00000259223.2	ENSG00000231500.7
15159	ENSG00000182054.9	ENSG00000259222.2	ENSG00000157014.11
15160	ENSG00000182057.5	ENSG00000259221.5	ENSG00000267023.5
15161	ENSG00000182070.5	ENSG00000259219.1	ENSG00000149294.16
15162	ENSG00000182077.11	ENSG00000259218.5	ENSG00000244089.1
15163	ENSG00000182083.7	ENSG00000259217.2	ENSG00000161914.10
15164	ENSG00000182087.14	ENSG00000259216.2	ENSG00000269054.1
15165	ENSG00000182093.15	ENSG00000259215.1	ENSG00000132155.11
15166	ENSG00000182095.14	ENSG00000259214.1	ENSG00000163218.15
15167	ENSG00000182103.5	ENSG00000259213.1	ENSG00000259639.6
15168	ENSG00000182107.6	ENSG00000259212.1	ENSG00000239405.1
15169	ENSG00000182108.11	ENSG00000259211.1	ENSG00000186326.3
15170	ENSG00000182111.8	ENSG00000259209.3	ENSG00000261329.5
15171	ENSG00000182117.5	ENSG00000259208.2	ENSG00000213927.3
15172	ENSG00000182118.8	ENSG00000259207.7	ENSG00000267676.1
15173	ENSG00000182132.13	ENSG00000259205.2	ENSG00000102871.16
15174	ENSG00000182134.16	ENSG00000259204.1	ENSG00000140527.15
15175	ENSG00000182141.10	ENSG00000259203.1	ENSG00000252481.1
15176	ENSG00000182149.20	ENSG00000259202.1	ENSG00000285708.1
15177	ENSG00000182150.15	ENSG00000259201.1	ENSG00000188626.6
15178	ENSG00000182154.8	ENSG00000259200.1	ENSG00000258344.1
15179	ENSG00000182156.10	ENSG00000259199.1	ENSG00000258985.1
15180	ENSG00000182158.15	ENSG00000259198.1	ENSG00000272823.1
15181	ENSG00000182162.11	ENSG00000259196.1	ENSG00000277558.1
15182	ENSG00000182162.11_PAR_Y	ENSG00000259195.1	ENSG00000245719.1
15183	ENSG00000182165.17	ENSG00000259194.1	ENSG00000244265.1
15184	ENSG00000182168.15	ENSG00000259192.1	ENSG00000150637.9
15185	ENSG00000182170.3	ENSG00000259191.2	ENSG00000175857.8
15186	ENSG00000182173.13	ENSG00000259188.5	ENSG00000233381.3
15187	ENSG00000182175.14	ENSG00000259187.1	ENSG00000172661.18
15188	ENSG00000182177.14	ENSG00000259186.1	ENSG00000237130.1
15189	ENSG00000182179.13	ENSG00000259185.1	ENSG00000144218.18
15190	ENSG00000182180.14	ENSG00000259183.1	ENSG00000090776.6
15191	ENSG00000182183.15	ENSG00000259182.5	ENSG00000241641.1
15192	ENSG00000182185.18	ENSG00000259181.1	ENSG00000227502.2
15193	ENSG00000182187.4	ENSG00000259180.1	ENSG00000267212.1
15194	ENSG00000182195.7	ENSG00000259179.1	ENSG00000279557.1
15195	ENSG00000182196.13	ENSG00000259178.1	ENSG00000224536.2
15196	ENSG00000182197.11	ENSG00000259177.1	ENSG00000165556.9
15197	ENSG00000182199.11	ENSG00000259175.1	ENSG00000112146.16
15198	ENSG00000182208.14	ENSG00000259173.2	ENSG00000237679.2
15199	ENSG00000182218.9	ENSG00000259172.1	ENSG00000254207.1
15200	ENSG00000182220.14	ENSG00000259171.1	ENSG00000279307.2
15201	ENSG00000182223.7	ENSG00000259170.1	ENSG00000221125.2
15202	ENSG00000182224.12	ENSG00000259169.1	ENSG00000228271.1
15203	ENSG00000182230.12	ENSG00000259168.1	ENSG00000090316.16
15204	ENSG00000182240.16	ENSG00000259167.2	ENSG00000232166.1
15205	ENSG00000182247.10	ENSG00000259166.1	ENSG00000249170.1
15206	ENSG00000182253.14	ENSG00000259165.1	ENSG00000106633.16
15207	ENSG00000182255.7	ENSG00000259164.1	ENSG00000130985.17
15208	ENSG00000182256.13	ENSG00000259163.1	ENSG00000223764.2

15209	ENSG00000182257.8	ENSG00000259162.1	ENSG00000174276.6
15210	ENSG00000182261.4	ENSG00000259161.2	ENSG00000225163.4
15211	ENSG00000182263.14	ENSG00000259160.1	ENSG00000180263.14
15212	ENSG00000182264.8	ENSG00000259158.4	ENSG00000179361.17
15213	ENSG00000182271.12	ENSG00000259157.1	ENSG00000270184.1
15214	ENSG00000182272.12	ENSG00000259156.7	ENSG00000280511.1
15215	ENSG00000182287.14	ENSG00000259155.2	ENSG00000224063.5
15216	ENSG00000182307.14	ENSG00000259154.1	ENSG00000279162.1
15217	ENSG00000182308.7	ENSG00000259153.1	ENSG00000255875.2
15218	ENSG00000182310.14	ENSG00000259152.1	ENSG00000248283.2
15219	ENSG00000182315.9	ENSG00000259151.2	ENSG00000281379.2
15220	ENSG00000182318.6	ENSG00000259150.5	ENSG00000279905.1
15221	ENSG00000182324.6	ENSG00000259149.1	ENSG00000102897.10
15222	ENSG00000182325.11	ENSG00000259148.1	ENSG00000111834.12
15223	ENSG00000182326.15	ENSG00000259146.3	ENSG00000204872.4
15224	ENSG00000182327.8	ENSG00000259144.2	ENSG00000126453.9
15225	ENSG00000182329.13	ENSG00000259143.1	ENSG00000259630.2
15226	ENSG00000182330.10	ENSG00000259142.1	ENSG00000251330.3
15227	ENSG00000182333.14	ENSG00000259140.2	ENSG00000254396.1
15228	ENSG00000182334.2	ENSG00000259137.1	ENSG00000268849.5
15229	ENSG00000182346.19	ENSG00000259136.1	ENSG00000224557.7
15230	ENSG00000182347.10	ENSG00000259135.1	ENSG00000229087.1
15231	ENSG00000182348.6	ENSG00000259134.6	ENSG00000234003.1
15232	ENSG00000182351.7	ENSG00000259133.5	ENSG00000140564.12
15233	ENSG00000182352.8	ENSG00000259132.1	ENSG00000241057.2
15234	ENSG00000182359.15	ENSG00000259130.1	ENSG00000237732.9
15235	ENSG00000182362.14	ENSG00000259129.5	ENSG00000147573.17
15236	ENSG00000182365.4	ENSG00000259126.1	ENSG00000169903.6
15237	ENSG00000182366.9	ENSG00000259125.1	ENSG00000145779.8
15238	ENSG00000182372.9	ENSG00000259124.1	ENSG00000150337.13
15239	ENSG00000182376.2	ENSG00000259123.1	ENSG00000254772.10
15240	ENSG00000182378.14	ENSG00000259122.2	ENSG00000188199.10
15241	ENSG00000182378.14_PAR_Y	ENSG00000259121.2	ENSG00000181788.4
15242	ENSG00000182379.10	ENSG00000259120.3	ENSG00000026103.22
15243	ENSG00000182383.8	ENSG00000259119.1	ENSG00000228847.1
15244	ENSG00000182389.19	ENSG00000259118.5	ENSG00000270190.1
15245	ENSG00000182393.3	ENSG00000259117.1	ENSG00000259656.1
15246	ENSG00000182397.14	ENSG00000259116.2	ENSG00000106809.11
15247	ENSG00000182400.15	ENSG00000259115.1	ENSG00000279879.1
15248	ENSG00000182405.6	ENSG00000259113.1	ENSG00000268678.1
15249	ENSG00000182415.9	ENSG00000259112.2	ENSG00000181097.5
15250	ENSG00000182446.14	ENSG00000259111.1	ENSG00000259169.1
15251	ENSG00000182447.4	ENSG00000259110.1	ENSG00000131236.17
15252	ENSG00000182450.13	ENSG00000259109.1	ENSG00000286050.1
15253	ENSG00000182459.5	ENSG00000259108.2	ENSG00000277265.1
15254	ENSG00000182463.16	ENSG00000259107.1	ENSG00000284906.1
15255	ENSG00000182472.8	ENSG00000259106.1	ENSG00000099960.13
15256	ENSG00000182473.22	ENSG00000259105.1	ENSG00000270019.1
15257	ENSG00000182477.5	ENSG00000259104.2	ENSG00000184307.15
15258	ENSG00000182481.9	ENSG00000259103.2	ENSG00000164512.18
15259	ENSG00000182484.15	ENSG00000259102.2	ENSG00000237749.4
15260	ENSG00000182484.15_PAR_Y	ENSG00000259100.1	ENSG00000213376.4
15261	ENSG00000182487.12	ENSG00000259099.2	ENSG00000156009.10

15262	ENSG00000182489.9	ENSG00000259098.1	ENSG00000254056.1
15263	ENSG00000182492.16	ENSG00000259097.1	ENSG00000216723.1
15264	ENSG00000182504.11	ENSG00000259096.1	ENSG00000186407.7
15265	ENSG00000182508.14	ENSG00000259095.1	ENSG00000262732.1
15266	ENSG00000182511.12	ENSG00000259094.1	ENSG00000251387.1
15267	ENSG00000182512.5	ENSG00000259093.1	ENSG00000166949.16
15268	ENSG00000182518.13	ENSG00000259092.1	ENSG00000232063.1
15269	ENSG00000182521.5	ENSG00000259091.1	ENSG00000237819.5
15270	ENSG00000182531.7	ENSG00000259090.1	ENSG00000224628.2
15271	ENSG00000182533.6	ENSG00000259089.2	ENSG00000081386.12
15272	ENSG00000182541.18	ENSG00000259088.1	ENSG00000229782.1
15273	ENSG00000182544.8	ENSG00000259087.5	ENSG00000234553.1
15274	ENSG00000182545.6	ENSG00000259086.3	ENSG00000260582.1
15275	ENSG00000182551.14	ENSG00000259084.6	ENSG00000278202.1
15276	ENSG00000182552.15	ENSG00000259083.1	ENSG00000255026.1
15277	ENSG00000182557.8	ENSG00000259082.1	ENSG00000135083.15
15278	ENSG00000182565.8	ENSG00000259081.1	ENSG00000255794.7
15279	ENSG00000182566.13	ENSG00000259080.1	ENSG00000225231.1
15280	ENSG00000182568.17	ENSG00000259079.1	ENSG00000201413.1
15281	ENSG00000182574.8	ENSG00000259078.2	ENSG00000281731.2
15282	ENSG00000182575.7	ENSG00000259077.1	ENSG00000271746.1
15283	ENSG00000182578.13	ENSG00000259076.1	ENSG00000134668.12
15284	ENSG00000182580.3	ENSG00000259075.6	ENSG00000253549.5
15285	ENSG00000182583.12	ENSG00000259074.1	ENSG00000273297.1
15286	ENSG00000182584.5	ENSG00000259073.1	ENSG00000120057.5
15287	ENSG00000182585.9	ENSG00000259072.1	ENSG00000146910.12
15288	ENSG00000182586.8	ENSG00000259071.1	ENSG00000244268.1
15289	ENSG00000182591.5	ENSG00000259070.6	ENSG00000180155.20
15290	ENSG00000182600.9	ENSG00000259069.1	ENSG00000229785.1
15291	ENSG00000182601.7	ENSG00000259068.1	ENSG00000235529.1
15292	ENSG00000182606.15	ENSG00000259067.1	ENSG00000175115.12
15293	ENSG00000182612.10	ENSG00000259066.5	ENSG00000234380.2
15294	ENSG00000182613.2	ENSG00000259065.1	ENSG00000253923.2
15295	ENSG00000182621.18	ENSG00000259064.2	ENSG00000259411.1
15296	ENSG00000182625.3	ENSG00000259062.2	ENSG00000106236.4
15297	ENSG00000182628.13	ENSG00000259061.1	ENSG00000146005.4
15298	ENSG00000182631.6	ENSG00000259060.7	ENSG00000231896.1
15299	ENSG00000182632.15	ENSG00000259059.1	ENSG00000186648.15
15300	ENSG00000182634.8	ENSG00000259058.1	ENSG00000235043.3
15301	ENSG00000182636.6	ENSG00000259057.1	ENSG00000164941.13
15302	ENSG00000182645.6	ENSG00000259056.1	ENSG00000086967.10
15303	ENSG00000182648.12	ENSG00000259055.1	ENSG00000226899.1
15304	ENSG00000182652.3	ENSG00000259054.1	ENSG00000242247.11
15305	ENSG00000182667.14	ENSG00000259053.1	ENSG00000274267.1
15306	ENSG00000182670.13	ENSG00000259052.1	ENSG00000278330.1
15307	ENSG00000182674.6	ENSG00000259051.1	ENSG00000213150.2
15308	ENSG00000182676.5	ENSG00000259050.2	ENSG00000230499.1
15309	ENSG00000182685.7	ENSG00000259049.1	ENSG00000243779.1
15310	ENSG00000182687.4	ENSG00000259048.1	ENSG00000242162.1
15311	ENSG00000182698.11	ENSG00000259047.1	ENSG00000268902.3
15312	ENSG00000182700.4	ENSG00000259046.1	ENSG00000169679.14
15313	ENSG00000182704.8	ENSG00000259045.1	ENSG00000258128.2
15314	ENSG00000182707.4	ENSG00000259044.1	ENSG00000215097.3

15315	ENSG00000182712.16	ENSG00000259043.1	ENSG00000187079.17
15316	ENSG00000182718.16	ENSG00000259042.2	ENSG00000196470.12
15317	ENSG00000182722.5	ENSG00000259041.1	ENSG00000120156.21
15318	ENSG00000182732.18	ENSG00000259040.5	ENSG00000185332.8
15319	ENSG00000182742.6	ENSG00000259039.2	ENSG00000229291.1
15320	ENSG00000182747.5	ENSG00000259038.1	ENSG00000265894.2
15321	ENSG00000182749.5	ENSG00000259037.1	ENSG00000230131.5
15322	ENSG00000182752.10	ENSG00000259036.1	ENSG00000174453.9
15323	ENSG00000182759.3	ENSG00000259035.1	ENSG00000274031.1
15324	ENSG00000182768.8	ENSG00000259033.1	ENSG00000068383.19
15325	ENSG00000182771.19	ENSG00000259032.2	ENSG00000277795.1
15326	ENSG00000182774.12	ENSG00000259031.1	ENSG00000278817.1
15327	ENSG00000182776.4	ENSG00000259030.7	ENSG00000257966.1
15328	ENSG00000182782.7	ENSG00000259029.1	ENSG00000264745.1
15329	ENSG00000182783.5	ENSG00000259028.1	ENSG00000102309.13
15330	ENSG00000182791.5	ENSG00000259026.1	ENSG00000262652.1
15331	ENSG00000182793.11	ENSG00000259025.1	ENSG00000165775.18
15332	ENSG00000182795.13	ENSG00000259024.6	ENSG00000232952.1
15333	ENSG00000182796.14	ENSG00000259023.2	ENSG00000229298.1
15334	ENSG00000182798.10	ENSG00000259022.2	ENSG00000231817.8
15335	ENSG00000182809.10	ENSG00000259021.2	ENSG00000126432.14
15336	ENSG00000182810.6	ENSG00000259020.3	ENSG00000161265.15
15337	ENSG00000182814.7	ENSG00000259019.1	ENSG00000269987.1
15338	ENSG00000182816.8	ENSG00000259018.1	ENSG00000229111.1
15339	ENSG00000182824.7	ENSG00000259017.1	ENSG00000266371.1
15340	ENSG00000182827.9	ENSG00000259016.1	ENSG00000164411.11
15341	ENSG00000182831.12	ENSG00000259015.1	ENSG00000261625.1
15342	ENSG00000182836.10	ENSG00000259013.2	ENSG00000284214.1
15343	ENSG00000182841.12	ENSG00000259012.2	ENSG00000188985.6
15344	ENSG00000182853.12	ENSG00000259011.1	ENSG00000260337.3
15345	ENSG00000182854.8	ENSG00000259010.1	ENSG00000148660.20
15346	ENSG00000182858.14	ENSG00000259009.4	ENSG00000251095.6
15347	ENSG00000182866.17	ENSG00000259008.1	ENSG00000274093.1
15348	ENSG00000182870.13	ENSG00000259007.1	ENSG00000178947.9
15349	ENSG00000182871.15	ENSG00000259006.1	ENSG00000232060.3
15350	ENSG00000182872.16	ENSG00000259005.1	ENSG00000206991.1
15351	ENSG00000182873.5	ENSG00000259003.1	ENSG00000240311.1
15352	ENSG00000182885.17	ENSG00000259002.2	ENSG00000130876.11
15353	ENSG00000182888.5	ENSG00000259001.3	ENSG00000175376.9
15354	ENSG00000182890.4	ENSG00000259000.1	ENSG00000229981.7
15355	ENSG00000182896.12	ENSG00000258998.1	ENSG00000244926.6
15356	ENSG00000182898.4	ENSG00000258997.1	ENSG00000285701.1
15357	ENSG00000182899.16	ENSG00000258996.2	ENSG00000283525.1
15358	ENSG00000182901.16	ENSG00000258995.1	ENSG00000136011.15
15359	ENSG00000182902.14	ENSG00000258994.1	ENSG00000257918.1
15360	ENSG00000182903.16	ENSG00000258993.1	ENSG00000238275.2
15361	ENSG00000182912.6	ENSG00000258992.6	ENSG00000163046.15
15362	ENSG00000182916.8	ENSG00000258991.1	ENSG00000228340.5
15363	ENSG00000182919.14	ENSG00000258990.2	ENSG00000102878.16
15364	ENSG00000182921.9	ENSG00000258989.1	ENSG00000279366.1
15365	ENSG00000182923.17	ENSG00000258988.1	ENSG00000176601.12
15366	ENSG00000182931.9	ENSG00000258987.1	ENSG00000264188.1
15367	ENSG00000182934.12	ENSG00000258986.7	ENSG00000185220.12

15368	ENSG00000182938.5	ENSG00000258985.1	ENSG00000140006.11
15369	ENSG00000182944.17	ENSG00000258984.5	ENSG00000168807.16
15370	ENSG00000182950.3	ENSG00000258983.2	ENSG00000237580.1
15371	ENSG00000182952.5	ENSG00000258982.1	ENSG00000071967.12
15372	ENSG00000182957.16	ENSG00000258981.1	ENSG00000265566.2
15373	ENSG00000182963.10	ENSG00000258980.1	ENSG00000231552.1
15374	ENSG00000182965.6	ENSG00000258979.2	ENSG00000111424.10
15375	ENSG00000182968.4	ENSG00000258978.1	ENSG00000213394.3
15376	ENSG00000182973.18	ENSG00000258977.1	ENSG00000257809.1
15377	ENSG00000182974.3	ENSG00000258976.1	ENSG00000229020.4
15378	ENSG00000182979.18	ENSG00000258975.1	ENSG00000140107.11
15379	ENSG00000182983.14	ENSG00000258973.1	ENSG00000286151.1
15380	ENSG00000182985.17	ENSG00000258972.1	ENSG00000155269.12
15381	ENSG00000182986.13	ENSG00000258971.1	ENSG00000134690.11
15382	ENSG00000182993.4	ENSG00000258970.1	ENSG00000272104.1
15383	ENSG00000183010.16	ENSG00000258969.1	ENSG00000135766.8
15384	ENSG00000183011.13	ENSG00000258968.2	ENSG00000160213.7
15385	ENSG00000183018.9	ENSG00000258967.1	ENSG00000100351.16
15386	ENSG00000183019.7	ENSG00000258966.1	ENSG00000161203.13
15387	ENSG00000183020.13	ENSG00000258965.2	ENSG00000108187.16
15388	ENSG00000183022.5	ENSG00000258964.1	ENSG00000223145.1
15389	ENSG00000183023.18	ENSG00000258963.1	ENSG00000130313.7
15390	ENSG00000183024.3	ENSG00000258962.2	ENSG00000225470.7
15391	ENSG00000183032.11	ENSG00000258960.1	ENSG00000259192.1
15392	ENSG00000183034.12	ENSG00000258959.2	ENSG00000285688.1
15393	ENSG00000183035.12	ENSG00000258958.1	ENSG00000279110.1
15394	ENSG00000183036.11	ENSG00000258957.1	ENSG00000166246.14
15395	ENSG00000183044.12	ENSG00000258956.2	ENSG00000118369.13
15396	ENSG00000183048.12	ENSG00000258955.1	ENSG00000274612.1
15397	ENSG00000183049.12	ENSG00000258954.1	ENSG00000268438.1
15398	ENSG00000183054.11	ENSG00000258952.2	ENSG00000175305.17
15399	ENSG00000183055.5	ENSG00000258951.1	ENSG00000244380.1
15400	ENSG00000183060.15	ENSG00000258949.1	ENSG00000115295.20
15401	ENSG00000183066.14	ENSG00000258948.2	ENSG00000263644.1
15402	ENSG00000183067.5	ENSG00000258947.7	ENSG00000130433.7
15403	ENSG00000183072.10	ENSG00000258945.1	ENSG00000062598.18
15404	ENSG00000183077.15	ENSG00000258944.1	ENSG00000085563.14
15405	ENSG00000183087.15	ENSG00000258943.1	ENSG00000101846.6
15406	ENSG00000183090.5	ENSG00000258942.1	ENSG00000270130.1
15407	ENSG00000183091.19	ENSG00000258940.2	ENSG00000153982.11
15408	ENSG00000183092.16	ENSG00000258938.1	ENSG00000260012.1
15409	ENSG00000183098.11	ENSG00000258935.1	ENSG00000275367.1
15410	ENSG00000183111.12	ENSG00000258934.1	ENSG00000126337.13
15411	ENSG00000183114.8	ENSG00000258933.1	ENSG00000154611.14
15412	ENSG00000183117.19	ENSG00000258932.5	ENSG00000279082.3
15413	ENSG00000183122.5	ENSG00000258931.1	ENSG00000260955.2
15414	ENSG00000183128.7	ENSG00000258930.1	ENSG00000259512.3
15415	ENSG00000183134.5	ENSG00000258928.1	ENSG00000124787.13
15416	ENSG00000183137.14	ENSG00000258927.1	ENSG00000132837.14
15417	ENSG00000183145.9	ENSG00000258925.2	ENSG00000272734.1
15418	ENSG00000183146.4	ENSG00000258924.4	ENSG00000211707.3
15419	ENSG00000183148.6	ENSG00000258923.2	ENSG00000177570.14
15420	ENSG00000183150.8	ENSG00000258922.1	ENSG00000166407.14

15421	ENSG00000183153.6	ENSG00000258921.1	ENSG00000162692.12
15422	ENSG00000183154.1	ENSG00000258920.1	ENSG00000260714.1
15423	ENSG00000183155.4	ENSG00000258919.1	ENSG00000242288.9
15424	ENSG00000183160.9	ENSG00000258918.1	ENSG00000187391.21
15425	ENSG00000183161.5	ENSG00000258917.1	ENSG00000266872.1
15426	ENSG00000183166.11	ENSG00000258916.2	ENSG00000087338.5
15427	ENSG00000183169.6	ENSG00000258915.1	ENSG00000157837.16
15428	ENSG00000183171.5	ENSG00000258914.1	ENSG00000286175.1
15429	ENSG00000183172.8	ENSG00000258913.1	ENSG00000269938.1
15430	ENSG00000183185.9	ENSG00000258912.1	ENSG00000246889.2
15431	ENSG00000183186.8	ENSG00000258910.3	ENSG00000285751.1
15432	ENSG00000183196.10	ENSG00000258909.1	ENSG00000236814.1
15433	ENSG00000183199.6	ENSG00000258908.1	ENSG00000283175.1
15434	ENSG00000183206.17	ENSG00000258907.2	ENSG00000138684.8
15435	ENSG00000183207.14	ENSG00000258906.2	ENSG00000179841.8
15436	ENSG00000183208.12	ENSG00000258905.1	ENSG00000257135.6
15437	ENSG00000183230.17	ENSG00000258904.1	ENSG00000231090.2
15438	ENSG00000183239.5	ENSG00000258903.1	ENSG00000285772.1
15439	ENSG00000183242.11	ENSG00000258902.1	ENSG00000185721.12
15440	ENSG00000183246.6	ENSG00000258901.2	ENSG00000286149.1
15441	ENSG00000183248.12	ENSG00000258900.1	ENSG00000280649.2
15442	ENSG00000183249.8	ENSG00000258899.1	ENSG00000254473.1
15443	ENSG00000183250.11	ENSG00000258897.1	ENSG00000279707.1
15444	ENSG00000183251.4	ENSG00000258896.1	ENSG00000251493.5
15445	ENSG00000183255.12	ENSG00000258895.1	ENSG00000135373.12
15446	ENSG00000183258.12	ENSG00000258894.1	ENSG00000239552.2
15447	ENSG00000183260.7	ENSG00000258893.1	ENSG00000280279.1
15448	ENSG00000183269.5	ENSG00000258892.1	ENSG00000235863.3
15449	ENSG00000183273.7	ENSG00000258891.1	ENSG00000270951.1
15450	ENSG00000183281.14	ENSG00000258890.7	ENSG00000106952.7
15451	ENSG00000183283.16	ENSG00000258888.1	ENSG00000237307.2
15452	ENSG00000183287.14	ENSG00000258887.3	ENSG00000111704.11
15453	ENSG00000183291.17	ENSG00000258886.2	ENSG00000204816.5
15454	ENSG00000183292.13	ENSG00000258885.1	ENSG00000197496.6
15455	ENSG00000183298.5	ENSG00000258884.1	ENSG00000171148.13
15456	ENSG00000183303.2	ENSG00000258883.1	ENSG00000268288.1
15457	ENSG00000183304.10	ENSG00000258881.6	ENSG00000280107.1
15458	ENSG00000183305.13	ENSG00000258878.1	ENSG00000169231.13
15459	ENSG00000183307.3	ENSG00000258877.2	ENSG00000225731.1
15460	ENSG00000183308.6	ENSG00000258876.1	ENSG00000173068.18
15461	ENSG00000183309.11	ENSG00000258875.5	ENSG00000224629.1
15462	ENSG00000183310.3	ENSG00000258874.1	ENSG00000171596.7
15463	ENSG00000183313.4	ENSG00000258873.2	ENSG00000259244.1
15464	ENSG00000183317.17	ENSG00000258872.2	ENSG00000214889.3
15465	ENSG00000183318.11	ENSG00000258871.1	ENSG00000219891.2
15466	ENSG00000183323.12	ENSG00000258870.1	ENSG00000169122.11
15467	ENSG00000183324.11	ENSG00000258869.1	ENSG00000231584.8
15468	ENSG00000183336.8	ENSG00000258868.1	ENSG00000245848.3
15469	ENSG00000183337.16	ENSG00000258867.5	ENSG00000082458.12
15470	ENSG00000183340.7	ENSG00000258866.1	ENSG00000167524.14
15471	ENSG00000183346.7	ENSG00000258864.1	ENSG00000203883.7
15472	ENSG00000183347.14	ENSG00000258863.1	ENSG00000265273.1
15473	ENSG00000183354.12	ENSG00000258861.1	ENSG00000273333.2

15474	ENSG00000183378.11	ENSG00000258860.1	ENSG00000260973.1
15475	ENSG00000183379.9	ENSG00000258859.1	ENSG00000199523.1
15476	ENSG00000183385.9	ENSG00000258858.1	ENSG00000242912.3
15477	ENSG00000183386.10	ENSG00000258857.1	ENSG00000234617.2
15478	ENSG00000183389.5	ENSG00000258856.2	ENSG00000224585.1
15479	ENSG00000183395.5	ENSG00000258855.1	ENSG00000236521.1
15480	ENSG00000183396.3	ENSG00000258854.1	ENSG00000281162.2
15481	ENSG00000183397.5	ENSG00000258853.1	ENSG00000273415.2
15482	ENSG00000183401.12	ENSG00000258851.1	ENSG00000155530.3
15483	ENSG00000183421.12	ENSG00000258850.1	ENSG00000250734.2
15484	ENSG00000183423.11	ENSG00000258849.1	ENSG00000180210.14
15485	ENSG00000183426.16	ENSG00000258848.1	ENSG00000121481.11
15486	ENSG00000183431.12	ENSG00000258847.1	ENSG00000232470.1
15487	ENSG00000183432.6	ENSG00000258846.1	ENSG00000253633.1
15488	ENSG00000183434.9	ENSG00000258845.1	ENSG00000224802.2
15489	ENSG00000183439.8	ENSG00000258844.1	ENSG00000063127.15
15490	ENSG00000183444.10	ENSG00000258843.1	ENSG00000236779.1
15491	ENSG00000183454.17	ENSG00000258842.1	ENSG00000142347.17
15492	ENSG00000183458.13	ENSG00000258841.1	ENSG00000166473.17
15493	ENSG00000183463.5	ENSG00000258839.3	ENSG00000250116.2
15494	ENSG00000183470.9	ENSG00000258837.1	ENSG00000233971.1
15495	ENSG00000183474.15	ENSG00000258836.2	ENSG00000275126.1
15496	ENSG00000183475.12	ENSG00000258835.1	ENSG00000284671.1
15497	ENSG00000183476.12	ENSG00000258834.4	ENSG00000204791.10
15498	ENSG00000183479.12	ENSG00000258831.1	ENSG00000183054.11
15499	ENSG00000183484.12	ENSG00000258830.1	ENSG00000262319.1
15500	ENSG00000183486.13	ENSG00000258829.1	ENSG00000255010.1
15501	ENSG00000183495.13	ENSG00000258828.2	ENSG00000256746.5
15502	ENSG00000183496.6	ENSG00000258827.2	ENSG00000182048.11
15503	ENSG00000183506.17	ENSG00000258826.5	ENSG00000244514.3
15504	ENSG00000183508.5	ENSG00000258824.2	ENSG00000260869.1
15505	ENSG00000183513.9	ENSG00000258823.1	ENSG00000257696.1
15506	ENSG00000183514.4	ENSG00000258822.2	ENSG00000134152.11
15507	ENSG00000183520.12	ENSG00000258820.5	ENSG00000172543.8
15508	ENSG00000183527.11	ENSG00000258818.4	ENSG00000172765.17
15509	ENSG00000183530.14	ENSG00000258817.1	ENSG00000285618.1
15510	ENSG00000183535.9	ENSG00000258816.1	ENSG00000185105.5
15511	ENSG00000183542.5	ENSG00000258815.1	ENSG00000145349.17
15512	ENSG00000183549.10	ENSG00000258814.1	ENSG00000266094.7
15513	ENSG00000183559.11	ENSG00000258813.2	ENSG00000204003.8
15514	ENSG00000183560.9	ENSG00000258812.1	ENSG00000129194.7
15515	ENSG00000183566.10	ENSG00000258811.1	ENSG00000124208.16
15516	ENSG00000183569.18	ENSG00000258808.1	ENSG00000241717.1
15517	ENSG00000183570.16	ENSG00000258807.5	ENSG00000123268.9
15518	ENSG00000183571.11	ENSG00000258806.2	ENSG00000226314.8
15519	ENSG00000183576.13	ENSG00000258805.1	ENSG00000231434.1
15520	ENSG00000183578.7	ENSG00000258804.1	ENSG00000227308.2
15521	ENSG00000183579.15	ENSG00000258803.1	ENSG00000266751.1
15522	ENSG00000183580.10	ENSG00000258802.1	ENSG00000130427.3
15523	ENSG00000183586.8	ENSG00000258800.1	ENSG00000001626.15
15524	ENSG00000183597.15	ENSG00000258799.1	ENSG00000124587.14
15525	ENSG00000183598.3	ENSG00000258798.1	ENSG00000148229.13
15526	ENSG00000183604.14	ENSG00000258797.2	ENSG00000119686.10

15527	ENSG00000183605.16	ENSG00000258796.1	ENSG00000011566.15
15528	ENSG00000183615.6	ENSG00000258795.1	ENSG00000272617.3
15529	ENSG00000183617.5	ENSG00000258794.3	ENSG00000262434.1
15530	ENSG00000183621.15	ENSG00000258793.1	ENSG00000179813.7
15531	ENSG00000183624.14	ENSG00000258792.4	ENSG00000175854.12
15532	ENSG00000183625.15	ENSG00000258791.8	ENSG00000157303.11
15533	ENSG00000183628.13	ENSG00000258790.1	ENSG00000265055.1
15534	ENSG00000183629.13	ENSG00000258789.1	ENSG00000256552.2
15535	ENSG00000183631.5	ENSG00000258788.3	ENSG00000280400.1
15536	ENSG00000183632.14	ENSG00000258787.1	ENSG00000237017.1
15537	ENSG00000183638.6	ENSG00000258785.5	ENSG00000142082.15
15538	ENSG00000183640.5	ENSG00000258784.1	ENSG00000243845.3
15539	ENSG00000183643.4	ENSG00000258783.1	ENSG00000203527.2
15540	ENSG00000183644.13	ENSG00000258782.3	ENSG00000207340.1
15541	ENSG00000183647.10	ENSG00000258781.3	ENSG00000277675.1
15542	ENSG00000183648.10	ENSG00000258780.1	ENSG00000227373.5
15543	ENSG00000183654.9	ENSG00000258779.7	ENSG00000271596.1
15544	ENSG00000183655.13	ENSG00000258778.2	ENSG00000265975.1
15545	ENSG00000183662.11	ENSG00000258777.1	ENSG00000214787.10
15546	ENSG00000183663.6	ENSG00000258776.1	ENSG00000269749.1
15547	ENSG00000183665.5	ENSG00000258774.1	ENSG00000116194.13
15548	ENSG00000183666.17	ENSG00000258773.1	ENSG00000162664.17
15549	ENSG00000183668.18	ENSG00000258772.1	ENSG00000258754.7
15550	ENSG00000183671.12	ENSG00000258771.1	ENSG00000108788.11
15551	ENSG00000183674.11	ENSG00000258770.1	ENSG00000112984.12
15552	ENSG00000183682.8	ENSG00000258769.1	ENSG00000184163.3
15553	ENSG00000183684.7	ENSG00000258768.2	ENSG00000213111.5
15554	ENSG00000183688.4	ENSG00000258767.1	ENSG00000092758.17
15555	ENSG00000183690.13	ENSG00000258766.1	ENSG00000127314.18
15556	ENSG00000183691.4	ENSG00000258765.1	ENSG00000276240.2
15557	ENSG00000183695.2	ENSG00000258764.1	ENSG00000198961.10
15558	ENSG00000183696.14	ENSG00000258763.5	ENSG00000229676.3
15559	ENSG00000183704.7	ENSG00000258762.1	ENSG00000140396.13
15560	ENSG00000183706.5	ENSG00000258761.1	ENSG00000112053.13
15561	ENSG00000183709.7	ENSG00000258760.1	ENSG00000237772.1
15562	ENSG00000183715.13	ENSG00000258759.1	ENSG00000115009.13
15563	ENSG00000183718.5	ENSG00000258758.1	ENSG00000196460.14
15564	ENSG00000183722.9	ENSG00000258757.1	ENSG00000125618.17
15565	ENSG00000183723.12	ENSG00000258754.7	ENSG00000286134.1
15566	ENSG00000183726.11	ENSG00000258753.1	ENSG00000225419.1
15567	ENSG00000183729.3	ENSG00000258752.1	ENSG00000162639.16
15568	ENSG00000183733.6	ENSG00000258751.1	ENSG00000284057.1
15569	ENSG00000183734.4	ENSG00000258750.2	ENSG00000254103.1
15570	ENSG00000183735.10	ENSG00000258749.1	ENSG00000123395.14
15571	ENSG00000183741.11	ENSG00000258748.1	ENSG00000214391.3
15572	ENSG00000183742.12	ENSG00000258747.1	ENSG00000065923.10
15573	ENSG00000183747.12	ENSG00000258746.2	ENSG00000166483.11
15574	ENSG00000183751.14	ENSG00000258745.1	ENSG00000263731.1
15575	ENSG00000183753.10	ENSG00000258744.1	ENSG00000262759.1
15576	ENSG00000183760.10	ENSG00000258743.5	ENSG00000237065.2
15577	ENSG00000183762.12	ENSG00000258742.5	ENSG00000281649.1
15578	ENSG00000183763.9	ENSG00000258741.3	ENSG00000240853.3
15579	ENSG00000183765.21	ENSG00000258740.1	ENSG00000232928.2

15580	ENSG00000183770.7	ENSG00000258739.1	ENSG00000091656.17
15581	ENSG00000183773.15	ENSG00000258738.1	ENSG00000270788.1
15582	ENSG00000183775.11	ENSG00000258737.2	ENSG00000260488.1
15583	ENSG00000183778.17	ENSG00000258736.1	ENSG00000235957.1
15584	ENSG00000183779.7	ENSG00000258735.1	ENSG00000217512.1
15585	ENSG00000183780.13	ENSG00000258734.2	ENSG00000257246.1
15586	ENSG00000183783.7	ENSG00000258733.5	ENSG00000272161.1
15587	ENSG00000183784.7	ENSG00000258732.1	ENSG00000121281.12
15588	ENSG00000183785.15	ENSG00000258731.1	ENSG00000102886.15
15589	ENSG00000183791.4	ENSG00000258730.1	ENSG00000205002.3
15590	ENSG00000183793.13	ENSG00000258729.2	ENSG00000248863.2
15591	ENSG00000183795.8	ENSG00000258728.1	ENSG00000270898.5
15592	ENSG00000183798.5	ENSG00000258727.1	ENSG00000261652.2
15593	ENSG00000183801.8	ENSG00000258726.1	ENSG00000170634.13
15594	ENSG00000183807.8	ENSG00000258725.1	ENSG00000126218.11
15595	ENSG00000183808.11	ENSG00000258724.1	ENSG00000259655.1
15596	ENSG00000183813.6	ENSG00000258723.1	ENSG00000236853.2
15597	ENSG00000183814.15	ENSG00000258722.2	ENSG00000162174.12
15598	ENSG00000183822.3	ENSG00000258721.3	ENSG00000224965.1
15599	ENSG00000183826.18	ENSG00000258719.1	ENSG00000280244.1
15600	ENSG00000183828.15	ENSG00000258718.2	ENSG00000234585.6
15601	ENSG00000183831.6	ENSG00000258717.1	ENSG00000137693.14
15602	ENSG00000183833.16	ENSG00000258716.1	ENSG00000267645.5
15603	ENSG00000183837.9	ENSG00000258713.2	ENSG00000285646.1
15604	ENSG00000183840.7	ENSG00000258712.2	ENSG00000197863.9
15605	ENSG00000183844.16	ENSG00000258711.2	ENSG00000254685.6
15606	ENSG00000183850.14	ENSG00000258710.7	ENSG00000241216.1
15607	ENSG00000183853.18	ENSG00000258708.1	ENSG00000198618.5
15608	ENSG00000183856.11	ENSG00000258707.2	ENSG00000269891.2
15609	ENSG00000183862.5	ENSG00000258706.1	ENSG00000247213.6
15610	ENSG00000183864.5	ENSG00000258705.2	ENSG00000138738.10
15611	ENSG00000183873.15	ENSG00000258703.1	ENSG00000161888.11
15612	ENSG00000183876.9	ENSG00000258702.2	ENSG00000234477.1
15613	ENSG00000183878.15	ENSG00000258701.1	ENSG00000249799.1
15614	ENSG00000183888.4	ENSG00000258700.5	ENSG00000236682.1
15615	ENSG00000183889.12	ENSG00000258699.1	ENSG00000267006.1
15616	ENSG00000183891.6	ENSG00000258698.1	ENSG00000196364.9
15617	ENSG00000183908.6	ENSG00000258696.1	ENSG00000229453.2
15618	ENSG00000183909.6	ENSG00000258695.2	ENSG00000261294.1
15619	ENSG00000183911.7	ENSG00000258694.1	ENSG00000239300.5
15620	ENSG00000183914.14	ENSG00000258693.1	ENSG00000126759.13
15621	ENSG00000183918.16	ENSG00000258692.2	ENSG00000253712.1
15622	ENSG00000183921.7	ENSG00000258691.1	ENSG00000226701.1
15623	ENSG00000183929.7	ENSG00000258690.1	ENSG00000233594.2
15624	ENSG00000183935.5	ENSG00000258689.1	ENSG00000135472.9
15625	ENSG00000183938.5	ENSG00000258687.1	ENSG00000146592.17
15626	ENSG00000183943.5	ENSG00000258685.2	ENSG00000258230.2
15627	ENSG00000183955.13	ENSG00000258684.2	ENSG00000181819.14
15628	ENSG00000183960.9	ENSG00000258683.1	ENSG00000234797.5
15629	ENSG00000183963.18	ENSG00000258682.1	ENSG00000233286.1
15630	ENSG00000183971.8	ENSG00000258681.1	ENSG00000119969.15
15631	ENSG00000183977.13	ENSG00000258680.2	ENSG00000271723.5
15632	ENSG00000183978.8	ENSG00000258679.1	ENSG00000255301.1

15633	ENSG00000183979.8	ENSG00000258678.1	ENSG00000147606.9
15634	ENSG00000183981.8	ENSG00000258677.2	ENSG00000255267.3
15635	ENSG00000184005.11	ENSG00000258676.4	ENSG00000121653.11
15636	ENSG00000184007.21	ENSG00000258675.1	ENSG00000184076.13
15637	ENSG00000184009.11	ENSG00000258674.5	ENSG00000159212.12
15638	ENSG00000184012.12	ENSG00000258673.1	ENSG00000286108.1
15639	ENSG00000184014.8	ENSG00000258672.1	ENSG00000132185.16
15640	ENSG00000184022.4	ENSG00000258671.2	ENSG00000135338.14
15641	ENSG00000184029.9	ENSG00000258670.1	ENSG00000224722.3
15642	ENSG00000184032.2	ENSG00000258668.1	ENSG00000003987.14
15643	ENSG00000184033.13	ENSG00000258667.1	ENSG00000227203.3
15644	ENSG00000184047.18	ENSG00000258666.1	ENSG00000169418.10
15645	ENSG00000184055.4	ENSG00000258665.1	ENSG00000228069.1
15646	ENSG00000184056.14	ENSG00000258663.1	ENSG00000254226.5
15647	ENSG00000184058.14	ENSG00000258662.1	ENSG00000175832.13
15648	ENSG00000184060.11	ENSG00000258661.1	ENSG00000279225.1
15649	ENSG00000184068.2	ENSG00000258660.1	ENSG00000205726.15
15650	ENSG00000184076.13	ENSG00000258659.6	ENSG00000172977.13
15651	ENSG00000184083.12	ENSG00000258658.1	ENSG00000101331.15
15652	ENSG00000184084.7	ENSG00000258657.5	ENSG00000257957.1
15653	ENSG00000184100.6	ENSG00000258656.1	ENSG00000223559.1
15654	ENSG00000184106.8	ENSG00000258655.2	ENSG00000270390.1
15655	ENSG00000184108.7	ENSG00000258654.1	ENSG00000137460.8
15656	ENSG00000184110.14	ENSG00000258653.3	ENSG00000278921.2
15657	ENSG00000184111.6	ENSG00000258650.1	ENSG00000144668.12
15658	ENSG00000184113.9	ENSG00000258649.1	ENSG00000249601.2
15659	ENSG00000184115.17	ENSG00000258648.1	ENSG00000187210.14
15660	ENSG00000184117.11	ENSG00000258647.5	ENSG00000111276.11
15661	ENSG00000184139.8	ENSG00000258646.1	ENSG00000277895.1
15662	ENSG00000184140.6	ENSG00000258645.2	ENSG00000148516.21
15663	ENSG00000184144.12	ENSG00000258644.5	ENSG00000232149.1
15664	ENSG00000184148.4	ENSG00000258643.5	ENSG00000126467.11
15665	ENSG00000184154.14	ENSG00000258642.1	ENSG00000103067.13
15666	ENSG00000184155.8	ENSG00000258641.1	ENSG00000104976.12
15667	ENSG00000184156.17	ENSG00000258640.3	ENSG00000285928.1
15668	ENSG00000184160.7	ENSG00000258639.1	ENSG00000278705.1
15669	ENSG00000184162.14	ENSG00000258638.2	ENSG00000230568.1
15670	ENSG00000184163.3	ENSG00000258637.1	ENSG00000254806.5
15671	ENSG00000184164.14	ENSG00000258636.1	ENSG00000274614.1
15672	ENSG00000184166.2	ENSG00000258634.3	ENSG00000250144.1
15673	ENSG00000184178.16	ENSG00000258633.1	ENSG00000237774.3
15674	ENSG00000184182.18	ENSG00000258632.2	ENSG00000239256.1
15675	ENSG00000184185.10	ENSG00000258631.2	ENSG00000099330.9
15676	ENSG00000184188.6	ENSG00000258630.1	ENSG00000115109.14
15677	ENSG00000184194.5	ENSG00000258629.1	ENSG00000128606.13
15678	ENSG00000184203.8	ENSG00000258628.1	ENSG00000235010.1
15679	ENSG00000184205.14	ENSG00000258627.1	ENSG00000168484.12
15680	ENSG00000184206.11	ENSG00000258626.2	ENSG00000260907.1
15681	ENSG00000184207.8	ENSG00000258625.2	ENSG00000171476.22
15682	ENSG00000184208.10	ENSG00000258624.1	ENSG00000141316.13
15683	ENSG00000184209.14	ENSG00000258623.1	ENSG00000128310.2
15684	ENSG00000184210.5	ENSG00000258622.2	ENSG00000183729.3
15685	ENSG00000184216.14	ENSG00000258620.1	ENSG00000231684.3

15686	ENSG00000184220.12	ENSG00000258619.2	ENSG00000214530.9
15687	ENSG00000184221.13	ENSG00000258618.2	ENSG00000283265.1
15688	ENSG00000184224.3	ENSG00000258616.5	ENSG00000124610.4
15689	ENSG00000184226.15	ENSG00000258615.1	ENSG00000266900.1
15690	ENSG00000184227.8	ENSG00000258613.1	ENSG00000229635.1
15691	ENSG00000184232.9	ENSG00000258611.2	ENSG00000235848.4
15692	ENSG00000184254.17	ENSG00000258610.1	ENSG00000262587.2
15693	ENSG00000184258.7	ENSG00000258609.3	ENSG00000267719.1
15694	ENSG00000184260.5	ENSG00000258608.1	ENSG00000112624.12
15695	ENSG00000184261.4	ENSG00000258605.1	ENSG00000158483.16
15696	ENSG00000184270.5	ENSG00000258604.1	ENSG00000228331.2
15697	ENSG00000184271.17	ENSG00000258603.3	ENSG00000105323.16
15698	ENSG00000184274.3	ENSG00000258602.1	ENSG00000260072.1
15699	ENSG00000184276.2	ENSG00000258601.1	ENSG00000255839.1
15700	ENSG00000184277.13	ENSG00000258600.1	ENSG00000285849.1
15701	ENSG00000184281.15	ENSG00000258599.2	ENSG00000230076.1
15702	ENSG00000184292.6	ENSG00000258598.1	ENSG00000225071.1
15703	ENSG00000184293.7	ENSG00000258597.3	ENSG00000224599.1
15704	ENSG00000184302.6	ENSG00000258595.2	ENSG00000273723.1
15705	ENSG00000184303.5	ENSG00000258594.1	ENSG00000110888.17
15706	ENSG00000184304.15	ENSG00000258593.2	ENSG00000213467.4
15707	ENSG00000184305.15	ENSG00000258592.1	ENSG00000281404.1
15708	ENSG00000184307.15	ENSG00000258591.2	ENSG00000235318.3
15709	ENSG00000184313.20	ENSG00000258590.5	ENSG00000259782.2
15710	ENSG00000184319.16	ENSG00000258588.3	ENSG00000164825.4
15711	ENSG00000184321.1	ENSG00000258587.1	ENSG00000244998.1
15712	ENSG00000184330.12	ENSG00000258586.1	ENSG00000178498.16
15713	ENSG00000184343.11	ENSG00000258585.2	ENSG00000152763.17
15714	ENSG00000184344.4	ENSG00000258584.2	ENSG00000278313.1
15715	ENSG00000184345.4	ENSG00000258583.6	ENSG00000237317.2
15716	ENSG00000184347.14	ENSG00000258581.2	ENSG00000184445.12
15717	ENSG00000184349.13	ENSG00000258580.1	ENSG00000223825.5
15718	ENSG00000184350.10	ENSG00000258578.2	ENSG00000143198.13
15719	ENSG00000184351.7	ENSG00000258577.1	ENSG00000072133.11
15720	ENSG00000184357.4	ENSG00000258576.1	ENSG00000225968.7
15721	ENSG00000184361.13	ENSG00000258573.5	ENSG00000266876.1
15722	ENSG00000184363.10	ENSG00000258571.1	ENSG00000244255.5
15723	ENSG00000184368.16	ENSG00000258570.1	ENSG00000283503.1
15724	ENSG00000184371.14	ENSG00000258569.1	ENSG00000271849.1
15725	ENSG00000184374.3	ENSG00000258567.1	ENSG00000099866.15
15726	ENSG00000184378.3	ENSG00000258566.2	ENSG00000253295.1
15727	ENSG00000184381.18	ENSG00000258565.1	ENSG00000230894.1
15728	ENSG00000184384.14	ENSG00000258564.2	ENSG00000249993.1
15729	ENSG00000184385.2	ENSG00000258563.2	ENSG00000283654.3
15730	ENSG00000184388.5	ENSG00000258562.2	ENSG00000234289.5
15731	ENSG00000184389.9	ENSG00000258561.1	ENSG00000184274.3
15732	ENSG00000184394.2	ENSG00000258560.1	ENSG00000114744.9
15733	ENSG00000184402.15	ENSG00000258559.2	ENSG00000266469.1
15734	ENSG00000184408.10	ENSG00000258558.1	ENSG00000261204.1
15735	ENSG00000184414.2	ENSG00000258557.1	ENSG00000286102.1
15736	ENSG00000184423.5	ENSG00000258556.1	ENSG00000225022.1
15737	ENSG00000184428.12	ENSG00000258555.6	ENSG00000217026.3
15738	ENSG00000184432.10	ENSG00000258554.1	ENSG00000254065.1

15739	ENSG00000184434.7	ENSG00000258553.5	ENSG00000223416.3
15740	ENSG00000184436.11	ENSG00000258552.1	ENSG00000258744.1
15741	ENSG00000184441.4	ENSG00000258551.5	ENSG00000181798.2
15742	ENSG00000184445.12	ENSG00000258550.1	ENSG00000284707.1
15743	ENSG00000184451.5	ENSG00000258549.1	ENSG00000264573.2
15744	ENSG00000184454.7	ENSG00000258548.5	ENSG00000257551.1
15745	ENSG00000184459.8	ENSG00000258546.1	ENSG00000242612.7
15746	ENSG00000184465.16	ENSG00000258545.5	ENSG00000228995.1
15747	ENSG00000184470.21	ENSG00000258544.1	ENSG00000166801.15
15748	ENSG00000184471.7	ENSG00000258542.1	ENSG00000125430.9
15749	ENSG00000184478.7	ENSG00000258541.1	ENSG00000241571.2
15750	ENSG00000184481.16	ENSG00000258540.1	ENSG00000243304.3
15751	ENSG00000184486.9	ENSG00000258538.5	ENSG00000263772.1
15752	ENSG00000184489.11	ENSG00000258537.5	ENSG00000169515.7
15753	ENSG00000184492.6	ENSG00000258536.1	ENSG00000117228.10
15754	ENSG00000184497.12	ENSG00000258535.1	ENSG00000026025.16
15755	ENSG00000184500.15	ENSG00000258534.1	ENSG00000285848.1
15756	ENSG00000184502.4	ENSG00000258532.1	ENSG00000125454.12
15757	ENSG00000184507.15	ENSG00000258531.2	ENSG00000170624.13
15758	ENSG00000184508.11	ENSG00000258529.5	ENSG00000247595.4
15759	ENSG00000184515.11	ENSG00000258527.1	ENSG00000179111.9
15760	ENSG00000184517.12	ENSG00000258526.6	ENSG00000224610.1
15761	ENSG00000184523.4	ENSG00000258525.1	ENSG00000103426.12
15762	ENSG00000184524.6	ENSG00000258524.1	ENSG00000168394.11
15763	ENSG00000184530.9	ENSG00000258521.1	ENSG00000279782.1
15764	ENSG00000184544.11	ENSG00000258520.1	ENSG00000069248.12
15765	ENSG00000184545.11	ENSG00000258519.1	ENSG00000197180.2
15766	ENSG00000184557.4	ENSG00000258517.1	ENSG00000249772.1
15767	ENSG00000184560.8	ENSG00000258516.1	ENSG00000258830.1
15768	ENSG00000184564.10	ENSG00000258515.1	ENSG00000253392.2
15769	ENSG00000184566.3	ENSG00000258513.1	ENSG00000276593.1
15770	ENSG00000184571.13	ENSG00000258512.1	ENSG00000285215.2
15771	ENSG00000184574.10	ENSG00000258511.1	ENSG00000258646.1
15772	ENSG00000184575.12	ENSG00000258510.2	ENSG00000250155.1
15773	ENSG00000184584.13	ENSG00000258509.1	ENSG00000286045.1
15774	ENSG00000184588.18	ENSG00000258507.1	ENSG00000272699.1
15775	ENSG00000184599.13	ENSG00000258506.2	ENSG00000100162.15
15776	ENSG00000184601.10	ENSG00000258505.1	ENSG00000162704.16
15777	ENSG00000184602.6	ENSG00000258504.2	ENSG00000224837.1
15778	ENSG00000184608.8	ENSG00000258503.1	ENSG00000226403.1
15779	ENSG00000184611.11	ENSG00000258502.5	ENSG00000231621.1
15780	ENSG00000184612.8	ENSG00000258501.1	ENSG00000182749.5
15781	ENSG00000184613.10	ENSG00000258500.2	ENSG00000250215.1
15782	ENSG00000184616.9	ENSG00000258498.8	ENSG00000271662.1
15783	ENSG00000184617.10	ENSG00000258497.1	ENSG00000223804.5
15784	ENSG00000184619.5	ENSG00000258496.1	ENSG00000262067.1
15785	ENSG00000184624.4	ENSG00000258494.1	ENSG00000230283.1
15786	ENSG00000184634.16	ENSG00000258493.2	ENSG00000243554.1
15787	ENSG00000184635.15	ENSG00000258491.2	ENSG00000254719.1
15788	ENSG00000184640.18	ENSG00000258490.1	ENSG00000163888.4
15789	ENSG00000184647.11	ENSG00000258489.3	ENSG00000280422.1
15790	ENSG00000184650.10	ENSG00000258488.2	ENSG00000119714.11
15791	ENSG00000184659.5	ENSG00000258487.1	ENSG00000257043.1

15792	ENSG00000184661.14	ENSG00000258485.2	ENSG00000237419.1
15793	ENSG00000184669.9	ENSG00000258484.4	ENSG00000260517.3
15794	ENSG00000184672.12	ENSG00000258483.3	ENSG00000227671.4
15795	ENSG00000184675.11	ENSG00000258482.1	ENSG00000286263.1
15796	ENSG00000184677.18	ENSG00000258481.1	ENSG00000081818.3
15797	ENSG00000184678.10	ENSG00000258479.5	ENSG00000169093.16
15798	ENSG00000184697.7	ENSG00000258478.1	ENSG00000181143.15
15799	ENSG00000184698.5	ENSG00000258477.1	ENSG00000283674.2
15800	ENSG00000184702.20	ENSG00000258476.5	ENSG00000143341.12
15801	ENSG00000184708.18	ENSG00000258474.1	ENSG00000200418.1
15802	ENSG00000184709.7	ENSG00000258473.1	ENSG00000253284.2
15803	ENSG00000184716.13	ENSG00000258472.8	ENSG00000105419.17
15804	ENSG00000184719.12	ENSG00000258471.2	ENSG00000230115.1
15805	ENSG00000184724.5	ENSG00000258469.1	ENSG00000169946.14
15806	ENSG00000184730.11	ENSG00000258468.1	ENSG00000200419.1
15807	ENSG00000184731.6	ENSG00000258467.1	ENSG00000274949.1
15808	ENSG00000184735.6	ENSG00000258466.5	ENSG00000130816.16
15809	ENSG00000184741.6	ENSG00000258465.7	ENSG00000100122.7
15810	ENSG00000184743.12	ENSG00000258464.1	ENSG00000259955.1
15811	ENSG00000184752.13	ENSG00000258463.1	ENSG00000235945.1
15812	ENSG00000184761.8	ENSG00000258462.2	ENSG00000239265.5
15813	ENSG00000184774.10	ENSG00000258461.5	ENSG00000258623.1
15814	ENSG00000184785.6	ENSG00000258460.1	ENSG00000253818.1
15815	ENSG00000184786.6	ENSG00000258459.1	ENSG00000214027.3
15816	ENSG00000184787.19	ENSG00000258458.6	ENSG00000250053.1
15817	ENSG00000184788.13	ENSG00000258457.5	ENSG00000065308.5
15818	ENSG00000184789.6	ENSG00000258456.2	ENSG00000255445.1
15819	ENSG00000184792.16	ENSG00000258455.1	ENSG00000180221.6
15820	ENSG00000184795.9	ENSG00000258454.1	ENSG00000266958.1
15821	ENSG00000184809.12	ENSG00000258453.3	ENSG00000149948.13
15822	ENSG00000184811.4	ENSG00000258452.1	ENSG00000114933.16
15823	ENSG00000184814.5	ENSG00000258451.1	ENSG00000142657.21
15824	ENSG00000184828.10	ENSG00000258450.1	ENSG00000256563.2
15825	ENSG00000184831.13	ENSG00000258448.1	ENSG00000272832.1
15826	ENSG00000184838.15	ENSG00000258446.1	ENSG00000196372.13
15827	ENSG00000184840.11	ENSG00000258445.3	ENSG00000241546.1
15828	ENSG00000184844.6	ENSG00000258444.1	ENSG00000259081.1
15829	ENSG00000184845.4	ENSG00000258443.1	ENSG00000128294.16
15830	ENSG00000184856.6	ENSG00000258441.1	ENSG00000165912.16
15831	ENSG00000184857.8	ENSG00000258440.1	ENSG00000276417.1
15832	ENSG00000184860.10	ENSG00000258439.1	ENSG00000179776.19
15833	ENSG00000184863.11	ENSG00000258438.3	ENSG00000264293.2
15834	ENSG00000184867.14	ENSG00000258437.1	ENSG00000260317.1
15835	ENSG00000184887.13	ENSG00000258436.1	ENSG00000228384.4
15836	ENSG00000184895.7	ENSG00000258435.1	ENSG00000111262.6
15837	ENSG00000184897.6	ENSG00000258433.1	ENSG00000214700.5
15838	ENSG00000184898.7	ENSG00000258431.1	ENSG00000277621.1
15839	ENSG00000184900.15	ENSG00000258430.1	ENSG00000233776.5
15840	ENSG00000184903.9	ENSG00000258429.2	ENSG00000273300.1
15841	ENSG00000184905.9	ENSG00000258428.5	ENSG00000241088.2
15842	ENSG00000184906.11	ENSG00000258427.3	ENSG00000173714.7
15843	ENSG00000184908.17	ENSG00000258426.1	ENSG00000103978.15
15844	ENSG00000184911.14	ENSG00000258425.1	ENSG00000175538.10

15845	ENSG00000184916.9	ENSG00000258424.1	ENSG00000169116.11
15846	ENSG00000184922.14	ENSG00000258423.1	ENSG00000224183.1
15847	ENSG00000184923.12	ENSG00000258422.5	ENSG00000235568.6
15848	ENSG00000184924.5	ENSG00000258421.2	ENSG00000198019.12
15849	ENSG00000184925.12	ENSG00000258420.1	ENSG00000234174.1
15850	ENSG00000184933.5	ENSG00000258419.1	ENSG00000106086.20
15851	ENSG00000184937.14	ENSG00000258417.3	ENSG00000235332.2
15852	ENSG00000184939.16	ENSG00000258416.1	ENSG00000268916.6
15853	ENSG00000184945.13	ENSG00000258415.2	ENSG00000279495.1
15854	ENSG00000184949.16	ENSG00000258414.1	ENSG00000200091.1
15855	ENSG00000184954.4	ENSG00000258413.1	ENSG00000250303.3
15856	ENSG00000184956.16	ENSG00000258412.1	ENSG00000253483.1
15857	ENSG00000184961.5	ENSG00000258411.2	ENSG00000072832.14
15858	ENSG00000184967.7	ENSG00000258410.1	ENSG00000225572.1
15859	ENSG00000184979.9	ENSG00000258408.1	ENSG00000274618.1
15860	ENSG00000184983.10	ENSG00000258407.1	ENSG00000172172.7
15861	ENSG00000184984.9	ENSG00000258406.1	ENSG00000263429.3
15862	ENSG00000184985.16	ENSG00000258405.10	ENSG00000253463.1
15863	ENSG00000184986.11	ENSG00000258404.1	ENSG00000132204.13
15864	ENSG00000184988.8	ENSG00000258402.1	ENSG00000233875.1
15865	ENSG00000184990.13	ENSG00000258401.1	ENSG00000039139.9
15866	ENSG00000184991.3	ENSG00000258400.1	ENSG00000166321.14
15867	ENSG00000184992.11	ENSG00000258399.7	ENSG00000164972.13
15868	ENSG00000184995.7	ENSG00000258397.1	ENSG00000280332.1
15869	ENSG00000184999.11	ENSG00000258394.1	ENSG00000275880.1
15870	ENSG00000185000.12	ENSG00000258393.1	ENSG00000170681.6
15871	ENSG00000185002.10	ENSG00000258392.1	ENSG00000143569.19
15872	ENSG00000185008.17	ENSG00000258390.1	ENSG00000126214.21
15873	ENSG00000185009.12	ENSG00000258388.7	ENSG00000253971.1
15874	ENSG00000185010.15	ENSG00000258387.1	ENSG00000142405.21
15875	ENSG00000185013.16	ENSG00000258386.1	ENSG00000177051.6
15876	ENSG00000185015.8	ENSG00000258385.1	ENSG00000153404.14
15877	ENSG00000185019.17	ENSG00000258384.1	ENSG00000182552.15
15878	ENSG00000185022.12	ENSG00000258383.1	ENSG00000235816.3
15879	ENSG00000185024.17	ENSG00000258381.1	ENSG00000225076.1
15880	ENSG00000185028.4	ENSG00000258380.1	ENSG00000285480.1
15881	ENSG00000185031.6	ENSG00000258379.1	ENSG00000271761.1
15882	ENSG00000185033.14	ENSG00000258378.1	ENSG00000250267.2
15883	ENSG00000185037.9	ENSG00000258377.1	ENSG00000020633.18
15884	ENSG00000185038.14	ENSG00000258376.2	ENSG00000284179.1
15885	ENSG00000185040.13	ENSG00000258375.2	ENSG00000241661.1
15886	ENSG00000185043.11	ENSG00000258373.1	ENSG00000250906.1
15887	ENSG00000185046.18	ENSG00000258369.1	ENSG00000244617.2
15888	ENSG00000185049.14	ENSG00000258368.2	ENSG00000271155.1
15889	ENSG00000185052.11	ENSG00000258367.2	ENSG00000231513.3
15890	ENSG00000185053.14	ENSG00000258365.1	ENSG00000248538.7
15891	ENSG00000185055.11	ENSG00000258364.1	ENSG00000265753.2
15892	ENSG00000185056.10	ENSG00000258360.1	ENSG00000244535.1
15893	ENSG00000185065.6	ENSG00000258359.1	ENSG00000266490.1
15894	ENSG00000185069.2	ENSG00000258358.2	ENSG00000260608.1
15895	ENSG00000185070.11	ENSG00000258357.1	ENSG00000246225.6
15896	ENSG00000185074.7	ENSG00000258355.1	ENSG00000260256.1
15897	ENSG00000185078.3	ENSG00000258354.1	ENSG00000154545.16

15898	ENSG00000185085.2	ENSG00000258352.1	ENSG00000284695.1
15899	ENSG00000185088.14	ENSG00000258350.1	ENSG00000268995.1
15900	ENSG00000185090.14	ENSG00000258346.1	ENSG00000259564.2
15901	ENSG00000185100.10	ENSG00000258345.1	ENSG00000272940.1
15902	ENSG00000185101.13	ENSG00000258344.1	ENSG00000152990.14
15903	ENSG00000185104.20	ENSG00000258343.1	ENSG00000257964.1
15904	ENSG00000185105.5	ENSG00000258342.1	ENSG00000250942.1
15905	ENSG00000185112.5	ENSG00000258338.1	ENSG00000226361.5
15906	ENSG00000185115.5	ENSG00000258337.1	ENSG00000253537.3
15907	ENSG00000185122.11	ENSG00000258336.2	ENSG00000225948.2
15908	ENSG00000185127.6	ENSG00000258334.1	ENSG00000250574.1
15909	ENSG00000185129.6	ENSG00000258332.2	ENSG00000229118.1
15910	ENSG00000185130.5	ENSG00000258331.1	ENSG00000124177.15
15911	ENSG00000185133.14	ENSG00000258325.2	ENSG00000231340.1
15912	ENSG00000185149.6	ENSG00000258324.2	ENSG00000233522.1
15913	ENSG00000185155.11	ENSG00000258323.1	ENSG00000241939.3
15914	ENSG00000185156.5	ENSG00000258320.1	ENSG00000010282.14
15915	ENSG00000185158.12	ENSG00000258317.1	ENSG00000259153.1
15916	ENSG00000185163.10	ENSG00000258316.1	ENSG00000206075.14
15917	ENSG00000185164.14	ENSG00000258315.5	ENSG00000206937.1
15918	ENSG00000185168.5	ENSG00000258314.3	ENSG00000082397.17
15919	ENSG00000185176.12	ENSG00000258313.1	ENSG00000234402.1
15920	ENSG00000185177.12	ENSG00000258312.1	ENSG00000241765.2
15921	ENSG00000185182.14	ENSG00000258311.5	ENSG00000235513.1
15922	ENSG00000185186.9	ENSG00000258308.5	ENSG00000260280.5
15923	ENSG00000185187.13	ENSG00000258304.1	ENSG00000257711.1
15924	ENSG00000185189.18	ENSG00000258303.1	ENSG00000224976.2
15925	ENSG00000185198.11	ENSG00000258302.2	ENSG00000213061.2
15926	ENSG00000185201.16	ENSG00000258301.3	ENSG00000273399.2
15927	ENSG00000185203.12	ENSG00000258300.2	ENSG00000249489.1
15928	ENSG00000185203.12_PAR_Y	ENSG00000258294.5	ENSG00000104419.14
15929	ENSG00000185215.9	ENSG00000258292.1	ENSG00000187840.5
15930	ENSG00000185219.17	ENSG00000258290.1	ENSG00000236535.1
15931	ENSG00000185220.12	ENSG00000258289.8	ENSG00000226705.1
15932	ENSG00000185221.6	ENSG00000258288.1	ENSG00000212443.1
15933	ENSG00000185222.10	ENSG00000258285.1	ENSG00000274507.1
15934	ENSG00000185231.4	ENSG00000258284.1	ENSG00000231123.1
15935	ENSG00000185236.12	ENSG00000258283.1	ENSG00000227615.1
15936	ENSG00000185238.13	ENSG00000258282.2	ENSG00000100479.13
15937	ENSG00000185245.8	ENSG00000258279.3	ENSG00000142192.21
15938	ENSG00000185246.17	ENSG00000258278.2	ENSG00000101084.18
15939	ENSG00000185247.15	ENSG00000258275.1	ENSG00000264772.6
15940	ENSG00000185250.15	ENSG00000258274.1	ENSG00000284994.1
15941	ENSG00000185252.19	ENSG00000258273.1	ENSG00000164543.7
15942	ENSG00000185261.14	ENSG00000258272.1	ENSG00000267127.7
15943	ENSG00000185262.9	ENSG00000258271.1	ENSG00000239316.3
15944	ENSG00000185264.12	ENSG00000258265.1	ENSG00000232374.5
15945	ENSG00000185267.10	ENSG00000258262.1	ENSG00000248375.1
15946	ENSG00000185269.12	ENSG00000258260.1	ENSG00000225479.1
15947	ENSG00000185271.8	ENSG00000258256.1	ENSG00000100425.18
15948	ENSG00000185272.14	ENSG00000258254.1	ENSG00000273796.1
15949	ENSG00000185274.12	ENSG00000258253.1	ENSG00000277151.1
15950	ENSG00000185275.6	ENSG00000258251.1	ENSG00000143776.18

15951	ENSG00000185278.15	ENSG00000258249.1	ENSG00000108830.10
15952	ENSG00000185290.4	ENSG00000258245.1	ENSG00000163755.8
15953	ENSG00000185291.11	ENSG00000258244.1	ENSG00000267026.5
15954	ENSG00000185291.11_PAR_Y	ENSG00000258240.5	ENSG00000166148.3
15955	ENSG00000185294.6	ENSG00000258239.1	ENSG00000251602.6
15956	ENSG00000185298.13	ENSG00000258235.1	ENSG00000249840.2
15957	ENSG00000185303.16	ENSG00000258234.1	ENSG00000254252.1
15958	ENSG00000185304.15	ENSG00000258233.1	ENSG00000203616.2
15959	ENSG00000185305.11	ENSG00000258232.2	ENSG00000198336.9
15960	ENSG00000185306.12	ENSG00000258231.5	ENSG00000234509.1
15961	ENSG00000185313.7	ENSG00000258230.2	ENSG00000226849.1
15962	ENSG00000185319.5	ENSG00000258227.7	ENSG00000234062.8
15963	ENSG00000185324.22	ENSG00000258225.1	ENSG00000262112.1
15964	ENSG00000185332.8	ENSG00000258224.1	ENSG00000267033.1
15965	ENSG00000185338.5	ENSG00000258223.6	ENSG00000198064.13
15966	ENSG00000185339.8	ENSG00000258220.1	ENSG00000166455.13
15967	ENSG00000185340.15	ENSG00000258216.6	ENSG00000180044.5
15968	ENSG00000185344.14	ENSG00000258215.1	ENSG00000197446.9
15969	ENSG00000185345.21	ENSG00000258214.1	ENSG00000183671.12
15970	ENSG00000185347.17	ENSG00000258212.1	ENSG00000004776.13
15971	ENSG00000185352.8	ENSG00000258210.1	ENSG00000232344.2
15972	ENSG00000185359.13	ENSG00000258206.1	ENSG00000141741.12
15973	ENSG00000185361.9	ENSG00000258205.1	ENSG00000204194.1
15974	ENSG00000185372.3	ENSG00000258204.1	ENSG00000234426.2
15975	ENSG00000185379.20	ENSG00000258203.5	ENSG00000106772.18
15976	ENSG00000185385.4	ENSG00000258202.1	ENSG00000254988.1
15977	ENSG00000185386.15	ENSG00000258199.1	ENSG00000279390.1
15978	ENSG00000185390.2	ENSG00000258197.1	ENSG00000272123.1
15979	ENSG00000185404.16	ENSG00000258196.1	ENSG00000134569.10
15980	ENSG00000185414.20	ENSG00000258193.1	ENSG00000242327.1
15981	ENSG00000185418.16	ENSG00000258186.2	ENSG00000103429.11
15982	ENSG00000185420.19	ENSG00000258185.2	ENSG00000226359.1
15983	ENSG00000185432.12	ENSG00000258184.1	ENSG00000230772.1
15984	ENSG00000185433.9	ENSG00000258183.5	ENSG00000235770.5
15985	ENSG00000185436.12	ENSG00000258181.1	ENSG00000024862.17
15986	ENSG00000185437.13	ENSG00000258179.1	ENSG00000150687.12
15987	ENSG00000185442.13	ENSG00000258178.1	ENSG00000270772.1
15988	ENSG00000185448.10	ENSG00000258177.1	ENSG00000227344.2
15989	ENSG00000185453.13	ENSG00000258175.1	ENSG00000070495.14
15990	ENSG00000185467.7	ENSG00000258173.1	ENSG00000230580.1
15991	ENSG00000185475.11	ENSG00000258171.1	ENSG00000233478.1
15992	ENSG00000185477.5	ENSG00000258170.1	ENSG00000248778.1
15993	ENSG00000185479.6	ENSG00000258169.1	ENSG00000139053.3
15994	ENSG00000185480.11	ENSG00000258168.5	ENSG00000181201.3
15995	ENSG00000185482.8	ENSG00000258167.1	ENSG00000226471.6
15996	ENSG00000185483.12	ENSG00000258162.3	ENSG00000212864.3
15997	ENSG00000185485.14	ENSG00000258159.1	ENSG00000271788.1
15998	ENSG00000185495.10	ENSG00000258154.2	ENSG00000232482.2
15999	ENSG00000185499.16	ENSG00000258153.1	ENSG00000138670.17
16000	ENSG00000185504.17	ENSG00000258150.6	ENSG00000235262.1
16001	ENSG00000185507.20	ENSG00000258148.2	ENSG00000252079.1
16002	ENSG00000185513.16	ENSG00000258144.1	ENSG00000198225.5
16003	ENSG00000185515.14	ENSG00000258142.2	ENSG00000172345.14

16004	ENSG00000185518.11	ENSG00000258140.2	ENSG00000143442.22
16005	ENSG00000185519.9	ENSG00000258137.5	ENSG00000196189.13
16006	ENSG00000185522.9	ENSG00000258136.1	ENSG00000258172.1
16007	ENSG00000185523.6	ENSG00000258135.5	ENSG00000229893.2
16008	ENSG00000185527.12	ENSG00000258134.1	ENSG00000219163.2
16009	ENSG00000185532.17	ENSG00000258133.1	ENSG00000284716.1
16010	ENSG00000185559.15	ENSG00000258131.1	ENSG00000083635.8
16011	ENSG00000185561.10	ENSG00000258130.8	ENSG00000103707.10
16012	ENSG00000185565.12	ENSG00000258128.2	ENSG00000212993.5
16013	ENSG00000185567.7	ENSG00000258125.1	ENSG00000242052.1
16014	ENSG00000185585.20	ENSG00000258123.1	ENSG00000173950.16
16015	ENSG00000185591.10	ENSG00000258122.1	ENSG00000112763.17
16016	ENSG00000185594.5	ENSG00000258121.1	ENSG00000231741.2
16017	ENSG00000185596.16	ENSG00000258120.1	ENSG00000275927.1
16018	ENSG00000185607.5	ENSG00000258119.1	ENSG00000258116.1
16019	ENSG00000185608.8	ENSG00000258118.1	ENSG00000267459.1
16020	ENSG00000185610.6	ENSG00000258117.2	ENSG00000243289.1
16021	ENSG00000185614.5	ENSG00000258116.1	ENSG00000178162.8
16022	ENSG00000185615.15	ENSG00000258115.1	ENSG00000182247.10
16023	ENSG00000185619.18	ENSG00000258114.1	ENSG00000233435.2
16024	ENSG00000185621.11	ENSG00000258112.1	ENSG00000171492.14
16025	ENSG00000185624.15	ENSG00000258111.1	ENSG00000182903.16
16026	ENSG00000185627.18	ENSG00000258108.2	ENSG00000135914.6
16027	ENSG00000185630.18	ENSG00000258107.2	ENSG00000258134.1
16028	ENSG00000185631.7	ENSG00000258104.1	ENSG00000243974.1
16029	ENSG00000185633.10	ENSG00000258102.4	ENSG00000251535.1
16030	ENSG00000185634.12	ENSG00000258101.2	ENSG00000241458.1
16031	ENSG00000185638.9	ENSG00000258100.2	ENSG00000262089.1
16032	ENSG00000185640.5	ENSG00000258099.1	ENSG00000177627.10
16033	ENSG00000185641.6	ENSG00000258098.1	ENSG00000255823.4
16034	ENSG00000185650.9	ENSG00000258096.1	ENSG00000164879.7
16035	ENSG00000185651.14	ENSG00000258092.1	ENSG00000270813.2
16036	ENSG00000185652.12	ENSG00000258091.1	ENSG00000156381.9
16037	ENSG00000185658.13	ENSG00000258090.1	ENSG00000267711.1
16038	ENSG00000185662.9	ENSG00000258088.1	ENSG00000251211.1
16039	ENSG00000185664.14	ENSG00000258086.1	ENSG00000152242.11
16040	ENSG00000185666.14	ENSG00000258084.5	ENSG00000154065.17
16041	ENSG00000185668.7	ENSG00000258083.2	ENSG00000271200.1
16042	ENSG00000185669.6	ENSG00000258082.1	ENSG00000177951.17
16043	ENSG00000185670.8	ENSG00000258081.3	ENSG00000188372.15
16044	ENSG00000185674.9	ENSG00000258080.1	ENSG00000211795.3
16045	ENSG00000185681.13	ENSG00000258077.2	ENSG00000160318.6
16046	ENSG00000185684.14	ENSG00000258076.1	ENSG00000249978.1
16047	ENSG00000185686.18	ENSG00000258074.1	ENSG00000130943.6
16048	ENSG00000185689.16	ENSG00000258073.1	ENSG00000157765.13
16049	ENSG00000185697.16	ENSG00000258072.1	ENSG00000163002.13
16050	ENSG00000185700.10	ENSG00000258071.1	ENSG00000204581.2
16051	ENSG00000185701.7	ENSG00000258068.1	ENSG00000270022.3
16052	ENSG00000185710.9	ENSG00000258066.1	ENSG00000075391.16
16053	ENSG00000185716.12	ENSG00000258065.1	ENSG00000105538.10
16054	ENSG00000185721.12	ENSG00000258064.1	ENSG00000163518.11
16055	ENSG00000185722.18	ENSG00000258057.5	ENSG00000261471.1
16056	ENSG00000185728.17	ENSG00000258056.2	ENSG00000223117.1

16057	ENSG00000185730.7	ENSG00000258053.1	ENSG00000168159.12
16058	ENSG00000185736.16	ENSG00000258052.1	ENSG00000163597.14
16059	ENSG00000185737.13	ENSG00000258051.1	ENSG00000244036.3
16060	ENSG00000185739.13	ENSG00000258050.2	ENSG00000237161.4
16061	ENSG00000185742.6	ENSG00000258048.1	ENSG00000169220.18
16062	ENSG00000185745.10	ENSG00000258045.2	ENSG00000139725.8
16063	ENSG00000185753.13	ENSG00000258044.1	ENSG00000234925.2
16064	ENSG00000185758.9	ENSG00000258039.2	ENSG00000207142.1
16065	ENSG00000185760.15	ENSG00000258038.5	ENSG00000268736.1
16066	ENSG00000185761.11	ENSG00000258036.2	ENSG00000116544.12
16067	ENSG00000185774.16	ENSG00000258035.1	ENSG00000182752.10
16068	ENSG00000185775.9	ENSG00000258034.1	ENSG00000202474.1
16069	ENSG00000185787.14	ENSG00000258033.1	ENSG00000259096.1
16070	ENSG00000185792.9	ENSG00000258028.2	ENSG00000148798.11
16071	ENSG00000185798.8	ENSG00000258027.2	ENSG00000221926.12
16072	ENSG00000185800.12	ENSG00000258026.1	ENSG00000132031.13
16073	ENSG00000185803.9	ENSG00000258024.1	ENSG00000188389.11
16074	ENSG00000185808.13	ENSG00000258021.1	ENSG00000272173.1
16075	ENSG00000185811.18	ENSG00000258018.1	ENSG00000229990.3
16076	ENSG00000185813.10	ENSG00000258017.1	ENSG00000244361.1
16077	ENSG00000185818.7	ENSG00000258016.1	ENSG00000075426.12
16078	ENSG00000185821.3	ENSG00000258013.2	ENSG00000236266.1
16079	ENSG00000185823.3	ENSG00000258012.1	ENSG00000260537.2
16080	ENSG00000185825.16	ENSG00000258011.2	ENSG00000173366.11
16081	ENSG00000185829.17	ENSG00000258010.4	ENSG00000092201.10
16082	ENSG00000185834.10	ENSG00000258007.1	ENSG00000259052.1
16083	ENSG00000185837.3	ENSG00000258001.1	ENSG00000184697.7
16084	ENSG00000185838.14	ENSG00000257998.1	ENSG00000233208.5
16085	ENSG00000185839.3	ENSG00000257997.5	ENSG00000277851.1
16086	ENSG00000185842.15	ENSG00000257995.1	ENSG00000163624.6
16087	ENSG00000185847.7	ENSG00000257994.1	ENSG00000117477.12
16088	ENSG00000185860.13	ENSG00000257991.1	ENSG00000229330.2
16089	ENSG00000185862.7	ENSG00000257989.1	ENSG00000226533.1
16090	ENSG00000185863.7	ENSG00000257987.5	ENSG00000219553.2
16091	ENSG00000185864.16	ENSG00000257986.2	ENSG00000163535.18
16092	ENSG00000185869.14	ENSG00000257985.1	ENSG00000282876.1
16093	ENSG00000185873.7	ENSG00000257979.1	ENSG00000177548.13
16094	ENSG00000185875.13	ENSG00000257976.1	ENSG00000240122.1
16095	ENSG00000185880.13	ENSG00000257966.1	ENSG00000186369.10
16096	ENSG00000185883.12	ENSG00000257964.1	ENSG00000171649.12
16097	ENSG00000185885.16	ENSG00000257962.1	ENSG00000198924.8
16098	ENSG00000185888.5	ENSG00000257959.1	ENSG00000255151.2
16099	ENSG00000185894.8	ENSG00000257958.1	ENSG00000274315.1
16100	ENSG00000185896.11	ENSG00000257957.1	ENSG00000213434.2
16101	ENSG00000185897.6	ENSG00000257956.1	ENSG00000236159.1
16102	ENSG00000185899.1	ENSG00000257955.1	ENSG00000214047.4
16103	ENSG00000185900.9	ENSG00000257954.1	ENSG00000150773.10
16104	ENSG00000185903.10	ENSG00000257953.1	ENSG00000249435.1
16105	ENSG00000185904.11	ENSG00000257951.2	ENSG00000152104.12
16106	ENSG00000185905.4	ENSG00000257950.3	ENSG00000271557.1
16107	ENSG00000185909.15	ENSG00000257949.7	ENSG00000286147.1
16108	ENSG00000185915.5	ENSG00000257948.1	ENSG00000231181.1
16109	ENSG00000185917.13	ENSG00000257947.1	ENSG00000267741.1

16110	ENSG00000185920.15	ENSG00000257943.2	ENSG00000103241.7
16111	ENSG00000185924.7	ENSG00000257941.1	ENSG00000227061.1
16112	ENSG00000185926.1	ENSG00000257940.1	ENSG00000131831.18
16113	ENSG00000185933.6	ENSG00000257935.2	ENSG00000163629.13
16114	ENSG00000185940.10	ENSG00000257932.1	ENSG00000252699.1
16115	ENSG00000185942.11	ENSG00000257927.1	ENSG00000137486.17
16116	ENSG00000185946.16	ENSG00000257925.1	ENSG00000211794.3
16117	ENSG00000185947.15	ENSG00000257924.1	ENSG00000170298.15
16118	ENSG00000185950.9	ENSG00000257923.11	ENSG00000164068.16
16119	ENSG00000185955.5	ENSG00000257921.6	ENSG00000168903.8
16120	ENSG00000185958.9	ENSG00000257920.1	ENSG00000184156.17
16121	ENSG00000185960.14	ENSG00000257918.1	ENSG00000167641.11
16122	ENSG00000185960.14_PAR_Y	ENSG00000257915.1	ENSG00000261552.1
16123	ENSG00000185962.1	ENSG00000257913.2	ENSG00000280113.2
16124	ENSG00000185963.14	ENSG00000257912.1	ENSG00000213903.9
16125	ENSG00000185966.3	ENSG00000257910.1	ENSG00000233877.2
16126	ENSG00000185972.5	ENSG00000257906.2	ENSG00000214788.3
16127	ENSG00000185973.11	ENSG00000257905.1	ENSG00000272911.1
16128	ENSG00000185974.7	ENSG00000257904.1	ENSG00000214626.2
16129	ENSG00000185982.6	ENSG00000257900.2	ENSG00000267727.1
16130	ENSG00000185985.9	ENSG00000257897.1	ENSG00000283809.1
16131	ENSG00000185986.12	ENSG00000257896.1	ENSG00000145431.11
16132	ENSG00000185988.13	ENSG00000257894.2	ENSG00000277406.2
16133	ENSG00000185989.11	ENSG00000257893.2	ENSG00000224713.4
16134	ENSG00000186001.13	ENSG00000257891.1	ENSG00000253273.2
16135	ENSG00000186007.10	ENSG00000257890.1	ENSG00000237852.1
16136	ENSG00000186009.4	ENSG00000257885.1	ENSG00000257616.1
16137	ENSG00000186010.19	ENSG00000257884.2	ENSG00000115317.11
16138	ENSG00000186017.14	ENSG00000257883.1	ENSG00000243836.5
16139	ENSG00000186019.10	ENSG00000257880.1	ENSG00000266744.1
16140	ENSG00000186020.13	ENSG00000257879.1	ENSG00000261864.1
16141	ENSG00000186026.7	ENSG00000257878.1	ENSG00000228599.1
16142	ENSG00000186038.9	ENSG00000257875.1	ENSG00000155026.16
16143	ENSG00000186047.10	ENSG00000257872.1	ENSG00000225778.5
16144	ENSG00000186049.8	ENSG00000257870.1	ENSG00000224781.1
16145	ENSG00000186051.6	ENSG00000257869.1	ENSG00000267110.1
16146	ENSG00000186056.10	ENSG00000257865.1	ENSG00000279399.1
16147	ENSG00000186063.13	ENSG00000257864.1	ENSG00000198934.4
16148	ENSG00000186073.13	ENSG00000257863.1	ENSG00000204632.11
16149	ENSG00000186074.19	ENSG00000257860.5	ENSG00000174938.14
16150	ENSG00000186075.12	ENSG00000257859.1	ENSG00000272855.1
16151	ENSG00000186076.5	ENSG00000257858.1	ENSG00000224116.6
16152	ENSG00000186081.12	ENSG00000257855.1	ENSG00000227076.1
16153	ENSG00000186082.9	ENSG00000257853.2	ENSG00000108468.15
16154	ENSG00000186086.18	ENSG00000257852.1	ENSG00000271128.1
16155	ENSG00000186088.16	ENSG00000257851.2	ENSG00000244610.3
16156	ENSG00000186090.10	ENSG00000257849.1	ENSG00000269533.5
16157	ENSG00000186092.6	ENSG00000257848.1	ENSG00000224886.2
16158	ENSG00000186094.17	ENSG00000257847.1	ENSG00000224550.1
16159	ENSG00000186103.4	ENSG00000257846.2	ENSG00000276486.1
16160	ENSG00000186104.10	ENSG00000257845.1	ENSG00000123106.10
16161	ENSG00000186105.8	ENSG00000257844.2	ENSG00000243051.3
16162	ENSG00000186106.11	ENSG00000257842.5	ENSG00000260914.3

16163	ENSG00000186111.9	ENSG00000257839.1	ENSG00000152463.14
16164	ENSG00000186113.1	ENSG00000257838.5	ENSG00000244192.1
16165	ENSG00000186115.13	ENSG00000257837.1	ENSG00000203799.13
16166	ENSG00000186118.8	ENSG00000257835.1	ENSG00000275350.1
16167	ENSG00000186119.8	ENSG00000257831.1	ENSG00000257391.1
16168	ENSG00000186124.4	ENSG00000257830.1	ENSG00000228778.2
16169	ENSG00000186130.5	ENSG00000257829.1	ENSG00000229161.2
16170	ENSG00000186132.15	ENSG00000257826.1	ENSG00000244021.4
16171	ENSG00000186136.1	ENSG00000257825.1	ENSG00000250786.1
16172	ENSG00000186141.9	ENSG00000257824.1	ENSG00000255443.1
16173	ENSG00000186143.11	ENSG00000257823.1	ENSG00000267521.1
16174	ENSG00000186146.1	ENSG00000257820.1	ENSG00000265282.1
16175	ENSG00000186148.13	ENSG00000257818.1	ENSG00000122574.10
16176	ENSG00000186150.4	ENSG00000257817.1	ENSG00000253307.1
16177	ENSG00000186152.6	ENSG00000257815.5	ENSG00000274985.1
16178	ENSG00000186153.17	ENSG00000257813.2	ENSG00000274024.1
16179	ENSG00000186160.5	ENSG00000257809.1	ENSG00000205449.11
16180	ENSG00000186162.10	ENSG00000257808.1	ENSG00000169021.6
16181	ENSG00000186163.9	ENSG00000257807.1	ENSG00000265818.1
16182	ENSG00000186166.9	ENSG00000257803.2	ENSG00000261996.1
16183	ENSG00000186174.12	ENSG00000257802.1	ENSG00000198848.12
16184	ENSG00000186184.17	ENSG00000257800.1	ENSG00000268833.1
16185	ENSG00000186185.13	ENSG00000257797.1	ENSG00000242583.1
16186	ENSG00000186187.11	ENSG00000257792.1	ENSG00000271259.1
16187	ENSG00000186188.10	ENSG00000257790.1	ENSG00000102710.20
16188	ENSG00000186190.7	ENSG00000257787.1	ENSG00000228286.3
16189	ENSG00000186191.7	ENSG00000257786.1	ENSG00000164821.4
16190	ENSG00000186193.9	ENSG00000257784.1	ENSG00000257953.1
16191	ENSG00000186197.14	ENSG00000257781.1	ENSG00000232311.1
16192	ENSG00000186198.4	ENSG00000257780.5	ENSG00000132749.11
16193	ENSG00000186204.14	ENSG00000257779.1	ENSG00000204217.13
16194	ENSG00000186205.13	ENSG00000257777.1	ENSG00000224497.1
16195	ENSG00000186207.5	ENSG00000257773.1	ENSG00000275597.1
16196	ENSG00000186212.3	ENSG00000257771.5	ENSG00000228753.1
16197	ENSG00000186222.4	ENSG00000257769.1	ENSG00000151458.12
16198	ENSG00000186226.9	ENSG00000257767.2	ENSG00000119318.13
16199	ENSG00000186230.7	ENSG00000257766.1	ENSG00000128253.14
16200	ENSG00000186231.17	ENSG00000257764.2	ENSG00000281530.1
16201	ENSG00000186234.7	ENSG00000257763.1	ENSG00000221838.9
16202	ENSG00000186235.10	ENSG00000257762.5	ENSG00000283667.1
16203	ENSG00000186244.7	ENSG00000257761.1	ENSG00000285873.1
16204	ENSG00000186260.16	ENSG00000257759.2	ENSG00000244756.1
16205	ENSG00000186265.10	ENSG00000257758.1	ENSG00000231527.6
16206	ENSG00000186268.3	ENSG00000257757.1	ENSG00000238273.3
16207	ENSG00000186272.12	ENSG00000257756.5	ENSG00000231686.1
16208	ENSG00000186280.7	ENSG00000257754.1	ENSG00000243317.8
16209	ENSG00000186281.12	ENSG00000257752.1	ENSG00000150630.4
16210	ENSG00000186283.14	ENSG00000257751.2	ENSG00000213087.3
16211	ENSG00000186288.5	ENSG00000257750.1	ENSG00000232457.4
16212	ENSG00000186297.12	ENSG00000257748.1	ENSG00000249930.1
16213	ENSG00000186298.11	ENSG00000257747.1	ENSG00000107021.16
16214	ENSG00000186300.12	ENSG00000257746.1	ENSG00000213420.8
16215	ENSG00000186301.8	ENSG00000257743.8	ENSG00000256723.1

16216	ENSG00000186306.1	ENSG00000257741.5	ENSG00000174527.9
16217	ENSG00000186310.9	ENSG00000257740.1	ENSG00000217566.2
16218	ENSG00000186312.10	ENSG00000257738.2	ENSG00000262833.1
16219	ENSG00000186314.11	ENSG00000257737.1	ENSG00000229839.6
16220	ENSG00000186318.16	ENSG00000257735.1	ENSG00000272031.3
16221	ENSG00000186326.3	ENSG00000257732.1	ENSG00000221947.7
16222	ENSG00000186328.4	ENSG00000257731.2	ENSG00000235238.1
16223	ENSG00000186329.9	ENSG00000257730.1	ENSG00000188191.15
16224	ENSG00000186334.9	ENSG00000257729.2	ENSG00000139780.7
16225	ENSG00000186335.9	ENSG00000257727.6	ENSG00000241506.1
16226	ENSG00000186340.15	ENSG00000257726.1	ENSG00000173262.11
16227	ENSG00000186350.11	ENSG00000257725.1	ENSG00000189403.15
16228	ENSG00000186352.9	ENSG00000257723.1	ENSG00000013441.16
16229	ENSG00000186364.12	ENSG00000257721.1	ENSG00000277446.1
16230	ENSG00000186367.6	ENSG00000257720.1	ENSG00000273369.1
16231	ENSG00000186369.10	ENSG00000257718.1	ENSG00000261231.6
16232	ENSG00000186376.15	ENSG00000257715.1	ENSG00000077063.11
16233	ENSG00000186377.8	ENSG00000257711.1	ENSG00000066468.22
16234	ENSG00000186393.5	ENSG00000257704.3	ENSG00000133193.12
16235	ENSG00000186395.8	ENSG00000257703.5	ENSG00000075785.12
16236	ENSG00000186399.10	ENSG00000257702.3	ENSG00000187193.9
16237	ENSG00000186407.7	ENSG00000257700.1	ENSG00000285907.1
16238	ENSG00000186409.16	ENSG00000257698.1	ENSG00000284661.1
16239	ENSG00000186416.14	ENSG00000257696.1	ENSG00000157617.17
16240	ENSG00000186417.14	ENSG00000257691.2	ENSG00000279182.1
16241	ENSG00000186431.19	ENSG00000257687.1	ENSG00000226756.1
16242	ENSG00000186432.9	ENSG00000257683.1	ENSG00000260159.1
16243	ENSG00000186439.13	ENSG00000257682.1	ENSG00000278467.1
16244	ENSG00000186440.2	ENSG00000257681.1	ENSG00000239382.10
16245	ENSG00000186442.7	ENSG00000257680.1	ENSG00000214222.2
16246	ENSG00000186446.12	ENSG00000257677.1	ENSG00000113273.17
16247	ENSG00000186448.15	ENSG00000257675.1	ENSG00000213790.2
16248	ENSG00000186451.2	ENSG00000257674.1	ENSG00000113070.8
16249	ENSG00000186452.11	ENSG00000257672.2	ENSG00000259985.1
16250	ENSG00000186453.13	ENSG00000257671.1	ENSG00000204956.5
16251	ENSG00000186458.5	ENSG00000257668.1	ENSG00000246477.3
16252	ENSG00000186462.8	ENSG00000257666.1	ENSG00000268790.5
16253	ENSG00000186466.5	ENSG00000257664.1	ENSG00000258289.8
16254	ENSG00000186468.13	ENSG00000257663.1	ENSG00000147164.12
16255	ENSG00000186469.8	ENSG00000257662.1	ENSG00000256314.1
16256	ENSG00000186470.14	ENSG00000257660.5	ENSG00000239282.7
16257	ENSG00000186471.12	ENSG00000257658.1	ENSG00000171450.5
16258	ENSG00000186472.20	ENSG00000257657.2	ENSG00000197142.10
16259	ENSG00000186474.15	ENSG00000257654.1	ENSG00000255467.1
16260	ENSG00000186479.4	ENSG00000257653.1	ENSG00000047634.15
16261	ENSG00000186480.13	ENSG00000257649.1	ENSG00000260816.2
16262	ENSG00000186481.16	ENSG00000257648.1	ENSG00000129757.13
16263	ENSG00000186487.19	ENSG00000257647.1	ENSG00000270276.2
16264	ENSG00000186493.12	ENSG00000257645.1	ENSG00000187144.11
16265	ENSG00000186496.12	ENSG00000257644.1	ENSG00000135702.14
16266	ENSG00000186501.14	ENSG00000257643.1	ENSG00000198342.10
16267	ENSG00000186508.4	ENSG00000257642.1	ENSG00000263859.1
16268	ENSG00000186509.4	ENSG00000257639.1	ENSG00000239593.1

16269	ENSG00000186510.12	ENSG00000257636.6	ENSG00000104133.15
16270	ENSG00000186513.3	ENSG00000257635.2	ENSG00000165462.5
16271	ENSG00000186517.14	ENSG00000257634.1	ENSG00000101665.9
16272	ENSG00000186522.15	ENSG00000257629.1	ENSG00000241358.1
16273	ENSG00000186523.14	ENSG00000257624.1	ENSG00000131368.8
16274	ENSG00000186526.12	ENSG00000257622.1	ENSG00000277561.5
16275	ENSG00000186529.16	ENSG00000257621.7	ENSG00000176731.12
16276	ENSG00000186532.11	ENSG00000257616.1	ENSG00000241409.1
16277	ENSG00000186543.7	ENSG00000257614.1	ENSG00000255535.2
16278	ENSG00000186562.7	ENSG00000257612.1	ENSG00000124067.17
16279	ENSG00000186564.5	ENSG00000257611.1	ENSG00000226302.1
16280	ENSG00000186566.13	ENSG00000257609.1	ENSG00000146215.13
16281	ENSG00000186567.12	ENSG00000257607.1	ENSG00000076242.14
16282	ENSG00000186572.2	ENSG00000257605.2	ENSG00000162714.12
16283	ENSG00000186575.17	ENSG00000257604.1	ENSG00000239396.3
16284	ENSG00000186577.14	ENSG00000257603.1	ENSG00000259319.1
16285	ENSG00000186579.2	ENSG00000257599.2	ENSG00000157110.16
16286	ENSG00000186583.11	ENSG00000257596.1	ENSG00000112276.14
16287	ENSG00000186591.12	ENSG00000257595.2	ENSG00000205056.8
16288	ENSG00000186594.14	ENSG00000257594.4	ENSG00000260452.1
16289	ENSG00000186599.7	ENSG00000257591.5	ENSG00000274944.4
16290	ENSG00000186603.5	ENSG00000257588.1	ENSG00000207445.1
16291	ENSG00000186615.10	ENSG00000257587.1	ENSG00000166153.16
16292	ENSG00000186625.14	ENSG00000257586.1	ENSG00000269653.1
16293	ENSG00000186628.12	ENSG00000257585.1	ENSG00000183549.10
16294	ENSG00000186635.14	ENSG00000257582.5	ENSG00000130529.16
16295	ENSG00000186638.16	ENSG00000257580.1	ENSG00000151116.17
16296	ENSG00000186642.16	ENSG00000257579.1	ENSG00000215199.3
16297	ENSG00000186645.7	ENSG00000257572.1	ENSG00000244528.1
16298	ENSG00000186648.15	ENSG00000257570.1	ENSG00000263089.1
16299	ENSG00000186652.10	ENSG00000257569.1	ENSG00000214717.12
16300	ENSG00000186654.21	ENSG00000257568.1	ENSG00000115844.11
16301	ENSG00000186660.15	ENSG00000257564.1	ENSG00000237798.1
16302	ENSG00000186665.9	ENSG00000257563.1	ENSG00000237003.1
16303	ENSG00000186666.6	ENSG00000257558.2	ENSG00000254248.1
16304	ENSG00000186675.6	ENSG00000257557.2	ENSG00000226125.1
16305	ENSG00000186676.3	ENSG00000257556.1	ENSG00000247765.2
16306	ENSG00000186678.7	ENSG00000257553.1	ENSG00000255672.1
16307	ENSG00000186684.12	ENSG00000257551.1	ENSG00000154608.14
16308	ENSG00000186687.16	ENSG00000257550.1	ENSG00000256377.5
16309	ENSG00000186704.9	ENSG00000257548.1	ENSG00000223525.1
16310	ENSG00000186710.11	ENSG00000257545.5	ENSG00000150540.14
16311	ENSG00000186714.12	ENSG00000257543.1	ENSG00000127362.2
16312	ENSG00000186715.11	ENSG00000257542.5	ENSG00000277159.1
16313	ENSG00000186716.20	ENSG00000257541.1	ENSG00000046651.15
16314	ENSG00000186723.4	ENSG00000257539.2	ENSG00000230659.3
16315	ENSG00000186732.14	ENSG00000257534.1	ENSG00000243663.1
16316	ENSG00000186743.2	ENSG00000257531.2	ENSG00000119326.15
16317	ENSG00000186765.11	ENSG00000257530.1	ENSG00000176788.9
16318	ENSG00000186766.7	ENSG00000257529.5	ENSG00000176697.19
16319	ENSG00000186767.6	ENSG00000257528.1	ENSG00000227825.4
16320	ENSG00000186777.11	ENSG00000257526.2	ENSG00000183779.7
16321	ENSG00000186787.8	ENSG00000257524.6	ENSG00000272744.1

16322	ENSG00000186788.13	ENSG00000257523.2	ENSG00000261438.1
16323	ENSG00000186790.6	ENSG00000257522.6	ENSG00000147896.3
16324	ENSG00000186792.17	ENSG00000257520.1	ENSG00000263220.1
16325	ENSG00000186795.1	ENSG00000257519.1	ENSG00000053501.13
16326	ENSG00000186803.3	ENSG00000257517.1	ENSG00000161980.5
16327	ENSG00000186806.5	ENSG00000257515.1	ENSG00000179774.8
16328	ENSG00000186810.8	ENSG00000257514.5	ENSG00000129925.11
16329	ENSG00000186812.13	ENSG00000257513.7	ENSG00000273365.1
16330	ENSG00000186814.14	ENSG00000257512.1	ENSG00000183793.13
16331	ENSG00000186815.12	ENSG00000257511.1	ENSG00000227544.9
16332	ENSG00000186818.12	ENSG00000257510.1	ENSG00000285085.1
16333	ENSG00000186825.5	ENSG00000257509.1	ENSG00000272953.1
16334	ENSG00000186827.11	ENSG00000257507.1	ENSG00000129295.9
16335	ENSG00000186831.11	ENSG00000257506.1	ENSG00000285051.1
16336	ENSG00000186832.9	ENSG00000257504.1	ENSG00000170374.6
16337	ENSG00000186834.3	ENSG00000257503.1	ENSG00000133275.16
16338	ENSG00000186838.13	ENSG00000257501.6	ENSG00000217227.1
16339	ENSG00000186842.4	ENSG00000257500.1	ENSG00000106004.5
16340	ENSG00000186844.5	ENSG00000257497.2	ENSG00000279891.1
16341	ENSG00000186847.6	ENSG00000257496.1	ENSG00000183153.6
16342	ENSG00000186854.11	ENSG00000257495.5	ENSG00000172554.12
16343	ENSG00000186860.4	ENSG00000257494.1	ENSG00000100410.8
16344	ENSG00000186862.19	ENSG00000257493.1	ENSG00000213066.13
16345	ENSG00000186866.16	ENSG00000257489.6	ENSG00000074706.13
16346	ENSG00000186867.10	ENSG00000257488.5	ENSG00000248677.1
16347	ENSG00000186868.15	ENSG00000257480.1	ENSG00000197721.16
16348	ENSG00000186871.7	ENSG00000257477.1	ENSG00000249876.1
16349	ENSG00000186881.3	ENSG00000257476.1	ENSG00000237705.1
16350	ENSG00000186886.8	ENSG00000257475.2	ENSG00000224452.1
16351	ENSG00000186889.10	ENSG00000257474.5	ENSG00000198406.7
16352	ENSG00000186891.14	ENSG00000257472.1	ENSG00000139899.10
16353	ENSG00000186895.4	ENSG00000257470.1	ENSG00000238072.1
16354	ENSG00000186897.5	ENSG00000257467.5	ENSG00000242330.3
16355	ENSG00000186907.8	ENSG00000257465.2	ENSG00000273096.1
16356	ENSG00000186908.14	ENSG00000257464.1	ENSG00000282772.1
16357	ENSG00000186910.4	ENSG00000257458.1	ENSG00000223350.2
16358	ENSG00000186912.6	ENSG00000257456.1	ENSG00000153234.14
16359	ENSG00000186918.14	ENSG00000257454.1	ENSG00000123454.11
16360	ENSG00000186919.12	ENSG00000257453.1	ENSG00000258445.3
16361	ENSG00000186924.3	ENSG00000257452.1	ENSG00000279192.1
16362	ENSG00000186925.6	ENSG00000257449.1	ENSG00000166986.15
16363	ENSG00000186930.4	ENSG00000257446.3	ENSG00000110811.20
16364	ENSG00000186940.6	ENSG00000257444.1	ENSG00000198454.2
16365	ENSG00000186943.1	ENSG00000257443.1	ENSG00000105497.8
16366	ENSG00000186951.16	ENSG00000257438.1	ENSG00000203362.2
16367	ENSG00000186952.15	ENSG00000257435.1	ENSG00000258584.2
16368	ENSG00000186960.11	ENSG00000257434.1	ENSG00000240869.3
16369	ENSG00000186965.5	ENSG00000257433.5	ENSG00000136895.19
16370	ENSG00000186967.6	ENSG00000257432.1	ENSG00000179935.9
16371	ENSG00000186970.4	ENSG00000257431.1	ENSG00000281010.1
16372	ENSG00000186971.3	ENSG00000257429.1	ENSG00000241413.3
16373	ENSG00000186973.11	ENSG00000257426.1	ENSG00000257613.1
16374	ENSG00000186976.15	ENSG00000257415.1	ENSG00000126500.3

16375	ENSG00000186977.2	ENSG00000257414.1	ENSG00000068323.17
16376	ENSG00000186980.6	ENSG00000257411.1	ENSG00000285879.1
16377	ENSG00000186994.11	ENSG00000257410.1	ENSG00000269044.2
16378	ENSG00000186998.16	ENSG00000257408.2	ENSG00000196634.3
16379	ENSG00000187003.6	ENSG00000257407.1	ENSG00000259736.1
16380	ENSG00000187005.5	ENSG00000257405.1	ENSG00000263050.1
16381	ENSG00000187010.21	ENSG00000257404.1	ENSG00000267690.1
16382	ENSG00000187012.10	ENSG00000257403.2	ENSG00000161544.10
16383	ENSG00000187013.4	ENSG00000257402.2	ENSG00000197302.10
16384	ENSG00000187017.16	ENSG00000257400.1	ENSG00000278673.1
16385	ENSG00000187021.15	ENSG00000257398.7	ENSG00000241095.1
16386	ENSG00000187024.14	ENSG00000257395.1	ENSG00000235121.1
16387	ENSG00000187026.2	ENSG00000257392.1	ENSG00000262874.2
16388	ENSG00000187033.9	ENSG00000257391.1	ENSG00000246763.6
16389	ENSG00000187037.8	ENSG00000257389.1	ENSG00000113593.12
16390	ENSG00000187048.13	ENSG00000257386.1	ENSG00000182814.7
16391	ENSG00000187049.9	ENSG00000257384.1	ENSG00000100284.21
16392	ENSG00000187051.9	ENSG00000257381.3	ENSG00000260583.1
16393	ENSG00000187054.15	ENSG00000257379.1	ENSG00000197483.10
16394	ENSG00000187066.8	ENSG00000257378.1	ENSG00000260550.2
16395	ENSG00000187068.3	ENSG00000257376.1	ENSG00000281856.1
16396	ENSG00000187079.17	ENSG00000257373.1	ENSG00000224598.1
16397	ENSG00000187080.9	ENSG00000257368.2	ENSG00000112335.15
16398	ENSG00000187082.2	ENSG00000257366.1	ENSG00000259848.9
16399	ENSG00000187091.13	ENSG00000257365.8	ENSG00000236653.2
16400	ENSG00000187094.11	ENSG00000257364.1	ENSG00000260689.2
16401	ENSG00000187097.12	ENSG00000257360.1	ENSG00000109519.13
16402	ENSG00000187098.15	ENSG00000257359.1	ENSG00000165066.12
16403	ENSG00000187103.3	ENSG00000257356.2	ENSG00000197790.1
16404	ENSG00000187105.8	ENSG00000257355.1	ENSG00000271567.1
16405	ENSG00000187109.14	ENSG00000257350.1	ENSG00000214076.3
16406	ENSG00000187116.13	ENSG00000257346.1	ENSG00000255234.5
16407	ENSG00000187118.13	ENSG00000257345.2	ENSG00000274430.1
16408	ENSG00000187122.17	ENSG00000257343.1	ENSG00000239671.1
16409	ENSG00000187123.15	ENSG00000257342.1	ENSG00000279985.1
16410	ENSG00000187134.14	ENSG00000257341.5	ENSG00000112486.16
16411	ENSG00000187135.7	ENSG00000257337.6	ENSG00000242667.1
16412	ENSG00000187140.5	ENSG00000257336.1	ENSG00000085449.15
16413	ENSG00000187144.11	ENSG00000257335.8	ENSG00000231940.1
16414	ENSG00000187147.18	ENSG00000257332.1	ENSG00000143653.10
16415	ENSG00000187151.7	ENSG00000257331.1	ENSG00000225361.3
16416	ENSG00000187164.20	ENSG00000257329.1	ENSG00000266970.1
16417	ENSG00000187166.1	ENSG00000257327.1	ENSG00000235579.1
16418	ENSG00000187170.4	ENSG00000257325.1	ENSG00000279235.1
16419	ENSG00000187172.15	ENSG00000257323.1	ENSG00000120899.18
16420	ENSG00000187173.4	ENSG00000257322.5	ENSG00000105146.12
16421	ENSG00000187175.5	ENSG00000257319.1	ENSG00000180279.7
16422	ENSG00000187180.3	ENSG00000257316.1	ENSG00000182372.9
16423	ENSG00000187185.4	ENSG00000257315.2	ENSG00000163092.19
16424	ENSG00000187186.14	ENSG00000257310.1	ENSG00000178397.13
16425	ENSG00000187187.14	ENSG00000257308.1	ENSG00000178057.14
16426	ENSG00000187189.10	ENSG00000257307.1	ENSG00000242971.3
16427	ENSG00000187191.14	ENSG00000257303.1	ENSG00000270689.1

16428	ENSG00000187193.9	ENSG00000257302.1	ENSG00000285133.1
16429	ENSG00000187210.14	ENSG00000257300.1	ENSG00000135525.18
16430	ENSG00000187223.3	ENSG00000257298.1	ENSG00000012124.17
16431	ENSG00000187229.3	ENSG00000257296.1	ENSG00000253626.3
16432	ENSG00000187231.14	ENSG00000257294.2	ENSG00000198513.11
16433	ENSG00000187238.5	ENSG00000257292.2	ENSG00000174238.14
16434	ENSG00000187239.17	ENSG00000257289.1	ENSG00000170786.12
16435	ENSG00000187240.15	ENSG00000257288.1	ENSG00000253119.1
16436	ENSG00000187242.5	ENSG00000257287.1	ENSG00000273214.1
16437	ENSG00000187243.16	ENSG00000257286.1	ENSG00000176749.9
16438	ENSG00000187244.11	ENSG00000257285.5	ENSG00000248161.5
16439	ENSG00000187257.16	ENSG00000257284.1	ENSG00000141696.13
16440	ENSG00000187258.13	ENSG00000257283.1	ENSG00000133962.7
16441	ENSG00000187260.15	ENSG00000257281.1	ENSG00000152904.11
16442	ENSG00000187266.14	ENSG00000257279.1	ENSG00000152348.16
16443	ENSG00000187268.12	ENSG00000257277.1	ENSG00000162594.15
16444	ENSG00000187272.6	ENSG00000257275.6	ENSG00000258682.1
16445	ENSG00000187288.10	ENSG00000257272.1	ENSG00000255437.1
16446	ENSG00000187323.12	ENSG00000257271.1	ENSG00000113140.11
16447	ENSG00000187325.5	ENSG00000257270.1	ENSG00000163705.12
16448	ENSG00000187372.11	ENSG00000257268.1	ENSG00000108387.14
16449	ENSG00000187391.21	ENSG00000257267.3	ENSG00000279837.1
16450	ENSG00000187398.12	ENSG00000257265.1	ENSG00000261790.1
16451	ENSG00000187416.12	ENSG00000257264.5	ENSG00000167900.12
16452	ENSG00000187446.12	ENSG00000257262.1	ENSG00000254244.1
16453	ENSG00000187472.4	ENSG00000257261.5	ENSG00000230928.1
16454	ENSG00000187474.5	ENSG00000257259.2	ENSG00000261349.1
16455	ENSG00000187475.5	ENSG00000257258.1	ENSG00000204428.12
16456	ENSG00000187479.7	ENSG00000257256.1	ENSG00000068985.5
16457	ENSG00000187481.2	ENSG00000257254.1	ENSG00000230162.1
16458	ENSG00000187483.9	ENSG00000257253.2	ENSG00000230777.1
16459	ENSG00000187486.5	ENSG00000257252.5	ENSG00000267682.1
16460	ENSG00000187492.9	ENSG00000257246.1	ENSG00000233838.5
16461	ENSG00000187498.16	ENSG00000257243.1	ENSG00000267178.3
16462	ENSG00000187504.6	ENSG00000257242.7	ENSG00000278879.1
16463	ENSG00000187510.9	ENSG00000257241.1	ENSG00000172209.6
16464	ENSG00000187513.9	ENSG00000257239.1	ENSG00000279887.1
16465	ENSG00000187514.16	ENSG00000257237.1	ENSG00000178927.18
16466	ENSG00000187516.6	ENSG00000257235.1	ENSG00000247516.7
16467	ENSG00000187522.16	ENSG00000257231.1	ENSG00000265845.2
16468	ENSG00000187527.10	ENSG00000257230.1	ENSG00000131470.14
16469	ENSG00000187531.14	ENSG00000257228.5	ENSG00000105696.9
16470	ENSG00000187533.13	ENSG00000257226.1	ENSG00000261641.2
16471	ENSG00000187534.6	ENSG00000257225.1	ENSG00000278582.1
16472	ENSG00000187535.14	ENSG00000257224.1	ENSG00000266707.1
16473	ENSG00000187536.4	ENSG00000257222.1	ENSG00000102755.12
16474	ENSG00000187537.13	ENSG00000257221.3	ENSG00000164898.13
16475	ENSG00000187545.5	ENSG00000257220.1	ENSG00000106686.16
16476	ENSG00000187546.14	ENSG00000257219.5	ENSG00000116922.14
16477	ENSG00000187550.8	ENSG00000257218.6	ENSG00000236438.7
16478	ENSG00000187553.9	ENSG00000257210.1	ENSG00000189423.12
16479	ENSG00000187554.13	ENSG00000257207.5	ENSG00000214917.3
16480	ENSG00000187555.15	ENSG00000257202.1	ENSG00000280136.2

16481	ENSG00000187556.7	ENSG00000257199.2	ENSG00000230067.3
16482	ENSG00000187559.5	ENSG00000257198.6	ENSG00000112818.10
16483	ENSG00000187566.5	ENSG00000257195.1	ENSG00000254873.1
16484	ENSG00000187569.3	ENSG00000257194.2	ENSG00000188582.8
16485	ENSG00000187581.2	ENSG00000257193.1	ENSG00000258940.2
16486	ENSG00000187583.10	ENSG00000257191.1	ENSG00000276045.3
16487	ENSG00000187589.8	ENSG00000257185.1	ENSG00000113742.13
16488	ENSG00000187595.16	ENSG00000257184.3	ENSG00000236601.2
16489	ENSG00000187600.14	ENSG00000257183.1	ENSG00000177000.12
16490	ENSG00000187601.4	ENSG00000257181.1	ENSG00000257715.1
16491	ENSG00000187605.15	ENSG00000257180.1	ENSG00000152580.8
16492	ENSG00000187607.16	ENSG00000257179.1	ENSG00000146670.10
16493	ENSG00000187608.9	ENSG00000257178.5	ENSG00000089169.15
16494	ENSG00000187609.16	ENSG00000257176.2	ENSG00000162576.16
16495	ENSG00000187612.1	ENSG00000257175.2	ENSG00000134884.15
16496	ENSG00000187616.4	ENSG00000257173.1	ENSG00000260252.1
16497	ENSG00000187621.14	ENSG00000257171.2	ENSG00000254131.1
16498	ENSG00000187624.9	ENSG00000257169.1	ENSG00000228548.1
16499	ENSG00000187626.9	ENSG00000257167.2	ENSG00000171533.11
16500	ENSG00000187627.15	ENSG00000257165.5	ENSG00000196873.15
16501	ENSG00000187630.17	ENSG00000257164.5	ENSG00000255559.1
16502	ENSG00000187634.12	ENSG00000257162.6	ENSG00000123009.4
16503	ENSG00000187642.9	ENSG00000257159.1	ENSG00000162643.13
16504	ENSG00000187650.3	ENSG00000257157.1	ENSG00000248445.5
16505	ENSG00000187653.11	ENSG00000257155.1	ENSG00000185499.16
16506	ENSG00000187657.6	ENSG00000257150.3	ENSG00000271653.1
16507	ENSG00000187658.7	ENSG00000257146.1	ENSG00000225159.1
16508	ENSG00000187664.9	ENSG00000257142.1	ENSG00000138069.18
16509	ENSG00000187672.13	ENSG00000257139.1	ENSG00000253297.1
16510	ENSG00000187676.8	ENSG00000257138.1	ENSG00000105048.17
16511	ENSG00000187678.9	ENSG00000257137.6	ENSG00000270986.1
16512	ENSG00000187682.2	ENSG00000257135.6	ENSG00000269967.1
16513	ENSG00000187686.4	ENSG00000257129.1	ENSG00000198055.11
16514	ENSG00000187688.15	ENSG00000257128.1	ENSG00000251555.1
16515	ENSG00000187689.10	ENSG00000257127.6	ENSG00000116455.14
16516	ENSG00000187690.4	ENSG00000257126.5	ENSG00000227992.1
16517	ENSG00000187699.10	ENSG00000257125.1	ENSG00000249741.2
16518	ENSG00000187701.4	ENSG00000257124.2	ENSG00000258377.1
16519	ENSG00000187713.7	ENSG00000257122.5	ENSG00000127129.10
16520	ENSG00000187714.7	ENSG00000257121.1	ENSG00000157087.19
16521	ENSG00000187715.13	ENSG00000257120.1	ENSG00000147601.14
16522	ENSG00000187720.14	ENSG00000257119.1	ENSG00000146067.16
16523	ENSG00000187721.8	ENSG00000257115.1	ENSG00000124496.12
16524	ENSG00000187726.8	ENSG00000257114.2	ENSG00000175147.12
16525	ENSG00000187730.9	ENSG00000257113.1	ENSG00000158764.7
16526	ENSG00000187733.6	ENSG00000257109.3	ENSG00000269091.5
16527	ENSG00000187735.14	ENSG00000257108.2	ENSG00000225578.1
16528	ENSG00000187736.12	ENSG00000257105.1	ENSG00000139505.11
16529	ENSG00000187741.15	ENSG00000257103.8	ENSG00000225415.2
16530	ENSG00000187742.14	ENSG00000257101.1	ENSG00000206585.1
16531	ENSG00000187747.2	ENSG00000257097.1	ENSG00000247240.7
16532	ENSG00000187753.13	ENSG00000257096.1	ENSG00000234513.1
16533	ENSG00000187754.8	ENSG00000257095.1	ENSG00000278291.1

16534	ENSG00000187758.8	ENSG00000257094.1	ENSG00000172803.18
16535	ENSG00000187762.5	ENSG00000257093.7	ENSG00000262728.5
16536	ENSG00000187763.3	ENSG00000257087.1	ENSG00000100258.18
16537	ENSG00000187764.11	ENSG00000257086.1	ENSG00000169327.5
16538	ENSG00000187766.1	ENSG00000257084.1	ENSG00000259490.2
16539	ENSG00000187772.8	ENSG00000257083.6	ENSG00000253438.3
16540	ENSG00000187773.8	ENSG00000257078.1	ENSG00000243775.2
16541	ENSG00000187775.16	ENSG00000257075.1	ENSG00000234568.3
16542	ENSG00000187778.14	ENSG00000257074.3	ENSG00000227258.5
16543	ENSG00000187783.12	ENSG00000257070.1	ENSG00000083454.22
16544	ENSG00000187790.11	ENSG00000257069.6	ENSG00000211672.2
16545	ENSG00000187791.13	ENSG00000257067.5	ENSG00000138741.11
16546	ENSG00000187792.5	ENSG00000257065.1	ENSG00000273541.1
16547	ENSG00000187796.14	ENSG00000257062.6	ENSG00000240296.1
16548	ENSG00000187800.13	ENSG00000257060.6	ENSG00000279121.1
16549	ENSG00000187801.15	ENSG00000257058.1	ENSG00000268201.1
16550	ENSG00000187806.8	ENSG00000257057.2	ENSG00000266610.2
16551	ENSG00000187808.5	ENSG00000257056.2	ENSG00000257885.1
16552	ENSG00000187812.12	ENSG00000257052.1	ENSG00000283755.2
16553	ENSG00000187815.10	ENSG00000257048.1	ENSG00000272351.1
16554	ENSG00000187821.8	ENSG00000257046.5	ENSG00000220920.1
16555	ENSG00000187823.3	ENSG00000257045.1	ENSG00000259751.1
16556	ENSG00000187824.8	ENSG00000257043.1	ENSG00000051128.19
16557	ENSG00000187833.7	ENSG00000257042.1	ENSG00000267731.1
16558	ENSG00000187837.3	ENSG00000257038.1	ENSG00000248466.1
16559	ENSG00000187838.17	ENSG00000257037.1	ENSG00000171522.6
16560	ENSG00000187840.5	ENSG00000257035.1	ENSG00000213777.5
16561	ENSG00000187847.4	ENSG00000257027.1	ENSG00000227835.8
16562	ENSG00000187848.13	ENSG00000257025.1	ENSG00000251593.1
16563	ENSG00000187855.5	ENSG00000257023.1	ENSG00000197859.10
16564	ENSG00000187857.4	ENSG00000257022.1	ENSG00000241333.3
16565	ENSG00000187860.10	ENSG00000257021.1	ENSG00000073067.14
16566	ENSG00000187862.11	ENSG00000257016.1	ENSG00000229133.1
16567	ENSG00000187866.8	ENSG00000257012.1	ENSG00000235880.1
16568	ENSG00000187867.8	ENSG00000257009.1	ENSG00000278700.1
16569	ENSG00000187870.7	ENSG00000257008.6	ENSG00000180988.2
16570	ENSG00000187871.2	ENSG00000257005.1	ENSG00000234232.7
16571	ENSG00000187889.13	ENSG00000257004.1	ENSG00000259666.2
16572	ENSG00000187893.10	ENSG00000257002.1	ENSG00000099139.13
16573	ENSG00000187900.4	ENSG00000257000.1	ENSG00000122870.11
16574	ENSG00000187902.11	ENSG00000256995.7	ENSG00000263432.2
16575	ENSG00000187904.3	ENSG00000256994.1	ENSG00000224165.5
16576	ENSG00000187905.10	ENSG00000256988.1	ENSG00000260084.1
16577	ENSG00000187908.18	ENSG00000256987.1	ENSG00000169760.17
16578	ENSG00000187912.11	ENSG00000256986.2	ENSG00000188676.13
16579	ENSG00000187918.5	ENSG00000256984.2	ENSG00000198900.6
16580	ENSG00000187922.13	ENSG00000256982.1	ENSG00000258908.1
16581	ENSG00000187942.11	ENSG00000256981.1	ENSG00000092036.18
16582	ENSG00000187944.2	ENSG00000256977.12	ENSG00000141854.9
16583	ENSG00000187950.8	ENSG00000256975.1	ENSG00000225137.1
16584	ENSG00000187951.11	ENSG00000256973.1	ENSG00000141232.5
16585	ENSG00000187952.9	ENSG00000256972.1	ENSG00000267284.1
16586	ENSG00000187953.10	ENSG00000256971.1	ENSG00000083857.14

16587	ENSG00000187954.12	ENSG00000256969.1	ENSG00000213104.3
16588	ENSG00000187955.12	ENSG00000256968.1	ENSG00000236905.2
16589	ENSG00000187957.8	ENSG00000256967.1	ENSG00000240069.1
16590	ENSG00000187959.9	ENSG00000256966.6	ENSG00000200494.1
16591	ENSG00000187961.14	ENSG00000256963.1	ENSG00000254287.1
16592	ENSG00000187969.5	ENSG00000256955.2	ENSG00000142733.16
16593	ENSG00000187979.4	ENSG00000256951.1	ENSG00000067141.17
16594	ENSG00000187980.6	ENSG00000256950.2	ENSG00000258451.1
16595	ENSG00000187984.12	ENSG00000256948.1	ENSG00000283164.1
16596	ENSG00000187987.9	ENSG00000256947.1	ENSG00000214717.12
16597	ENSG00000187988.5	ENSG00000256944.1	ENSG00000101220.17
16598	ENSG00000187994.14	ENSG00000256943.1	ENSG00000199804.1
16599	ENSG00000187997.11	ENSG00000256940.1	ENSG00000243264.2
16600	ENSG00000187999.4	ENSG00000256937.1	ENSG00000279144.1
16601	ENSG00000188000.4	ENSG00000256928.1	ENSG00000226669.2
16602	ENSG00000188001.10	ENSG00000256925.2	ENSG00000102125.16
16603	ENSG00000188002.10	ENSG00000256923.1	ENSG00000184863.11
16604	ENSG00000188004.10	ENSG00000256922.1	ENSG00000177875.4
16605	ENSG00000188010.14	ENSG00000256917.1	ENSG00000238286.1
16606	ENSG00000188011.5	ENSG00000256916.1	ENSG00000128254.13
16607	ENSG00000188013.6	ENSG00000256915.1	ENSG00000235196.3
16608	ENSG00000188015.10	ENSG00000256913.1	ENSG00000236857.3
16609	ENSG00000188021.8	ENSG00000256912.1	ENSG00000227999.1
16610	ENSG00000188026.13	ENSG00000256906.1	ENSG00000252139.1
16611	ENSG00000188032.9	ENSG00000256904.1	ENSG00000216285.5
16612	ENSG00000188033.10	ENSG00000256902.1	ENSG00000271452.1
16613	ENSG00000188038.8	ENSG00000256898.1	ENSG00000262831.1
16614	ENSG00000188039.14	ENSG00000256897.1	ENSG00000243307.2
16615	ENSG00000188042.8	ENSG00000256896.1	ENSG00000176204.13
16616	ENSG00000188050.2	ENSG00000256894.1	ENSG00000185339.8
16617	ENSG00000188051.7	ENSG00000256892.2	ENSG00000228816.1
16618	ENSG00000188056.11	ENSG00000256888.5	ENSG00000284708.1
16619	ENSG00000188060.7	ENSG00000256884.1	ENSG00000258227.7
16620	ENSG00000188064.10	ENSG00000256879.1	ENSG00000166902.5
16621	ENSG00000188070.9	ENSG00000256875.2	ENSG00000135973.2
16622	ENSG00000188076.2	ENSG00000256870.2	ENSG00000154262.13
16623	ENSG00000188078.5	ENSG00000256863.1	ENSG00000164185.6
16624	ENSG00000188086.13	ENSG00000256862.2	ENSG00000106546.14
16625	ENSG00000188089.13	ENSG00000256861.1	ENSG00000272988.1
16626	ENSG00000188092.14	ENSG00000256852.1	ENSG00000227885.2
16627	ENSG00000188095.6	ENSG00000256851.1	ENSG00000229870.1
16628	ENSG00000188100.9	ENSG00000256849.1	ENSG00000177693.4
16629	ENSG00000188101.5	ENSG00000256847.1	ENSG00000270445.1
16630	ENSG00000188107.14	ENSG00000256844.1	ENSG00000278287.1
16631	ENSG00000188112.9	ENSG00000256843.1	ENSG00000229897.2
16632	ENSG00000188120.14	ENSG00000256837.1	ENSG00000240966.3
16633	ENSG00000188124.3	ENSG00000256835.1	ENSG00000084234.17
16634	ENSG00000188130.14	ENSG00000256827.1	ENSG00000264575.1
16635	ENSG00000188133.6	ENSG00000256826.1	ENSG00000281903.2
16636	ENSG00000188152.12	ENSG00000256825.4	ENSG00000254741.1
16637	ENSG00000188153.13	ENSG00000256824.1	ENSG00000231461.1
16638	ENSG00000188155.11	ENSG00000256817.1	ENSG00000236391.3
16639	ENSG00000188157.15	ENSG00000256814.1	ENSG00000225840.2

16640	ENSG00000188158.15	ENSG00000256813.1	ENSG00000235453.10
16641	ENSG00000188162.10	ENSG00000256812.1	ENSG00000286018.1
16642	ENSG00000188163.8	ENSG00000256811.1	ENSG00000279852.1
16643	ENSG00000188167.9	ENSG00000256810.1	ENSG00000267344.1
16644	ENSG00000188171.16	ENSG00000256806.5	ENSG00000204444.11
16645	ENSG00000188175.9	ENSG00000256804.1	ENSG00000129991.12
16646	ENSG00000188176.12	ENSG00000256803.1	ENSG00000221539.1
16647	ENSG00000188177.14	ENSG00000256802.2	ENSG00000091436.17
16648	ENSG00000188185.11	ENSG00000256799.1	ENSG00000124731.13
16649	ENSG00000188186.10	ENSG00000256797.1	ENSG00000141738.14
16650	ENSG00000188191.15	ENSG00000256789.1	ENSG00000230604.2
16651	ENSG00000188199.10	ENSG00000256783.1	ENSG00000164430.16
16652	ENSG00000188211.8	ENSG00000256779.2	ENSG00000107959.16
16653	ENSG00000188215.10	ENSG00000256777.1	ENSG00000136169.16
16654	ENSG00000188219.14	ENSG00000256774.1	ENSG00000260896.6
16655	ENSG00000188223.9	ENSG00000256771.3	ENSG00000093010.13
16656	ENSG00000188227.13	ENSG00000256769.1	ENSG00000231665.1
16657	ENSG00000188229.6	ENSG00000256762.1	ENSG00000242282.6
16658	ENSG00000188234.13	ENSG00000256757.1	ENSG00000235777.1
16659	ENSG00000188242.4	ENSG00000256756.1	ENSG00000271550.1
16660	ENSG00000188243.12	ENSG00000256752.1	ENSG00000274751.1
16661	ENSG00000188257.11	ENSG00000256751.5	ENSG00000169738.7
16662	ENSG00000188263.10	ENSG00000256750.1	ENSG00000277319.1
16663	ENSG00000188266.14	ENSG00000256748.1	ENSG00000089123.16
16664	ENSG00000188269.9	ENSG00000256747.1	ENSG00000263531.1
16665	ENSG00000188277.9	ENSG00000256746.5	ENSG00000138449.10
16666	ENSG00000188280.11	ENSG00000256745.1	ENSG00000113600.11
16667	ENSG00000188282.12	ENSG00000256742.1	ENSG00000153086.14
16668	ENSG00000188283.11	ENSG00000256739.1	ENSG00000242113.3
16669	ENSG00000188290.10	ENSG00000256737.1	ENSG00000162006.9
16670	ENSG00000188293.6	ENSG00000256734.1	ENSG00000007944.15
16671	ENSG00000188295.14	ENSG00000256733.1	ENSG00000135404.11
16672	ENSG00000188305.6	ENSG00000256732.1	ENSG00000282408.1
16673	ENSG00000188306.6	ENSG00000256723.1	ENSG00000162728.5
16674	ENSG00000188312.14	ENSG00000256721.1	ENSG00000233426.3
16675	ENSG00000188313.13	ENSG00000256720.1	ENSG00000173818.17
16676	ENSG00000188314.3	ENSG00000256717.1	ENSG00000188859.6
16677	ENSG00000188315.8	ENSG00000256714.1	ENSG00000286017.1
16678	ENSG00000188316.14	ENSG00000256713.7	ENSG00000102230.13
16679	ENSG00000188321.13	ENSG00000256712.1	ENSG00000145782.13
16680	ENSG00000188322.5	ENSG00000256709.2	ENSG00000275431.1
16681	ENSG00000188324.4	ENSG00000256708.1	ENSG00000137210.13
16682	ENSG00000188334.3	ENSG00000256706.1	ENSG00000285815.1
16683	ENSG00000188338.15	ENSG00000256705.3	ENSG00000240898.1
16684	ENSG00000188340.2	ENSG00000256704.3	ENSG00000184828.10
16685	ENSG00000188342.12	ENSG00000256699.1	ENSG00000280238.1
16686	ENSG00000188343.12	ENSG00000256695.1	ENSG00000189077.11
16687	ENSG00000188352.12	ENSG00000256694.1	ENSG00000167460.16
16688	ENSG00000188365.3	ENSG00000256691.1	ENSG00000171055.15
16689	ENSG00000188368.9	ENSG00000256690.1	ENSG00000230273.1
16690	ENSG00000188372.15	ENSG00000256686.1	ENSG00000207406.1
16691	ENSG00000188373.5	ENSG00000256684.1	ENSG00000246339.5
16692	ENSG00000188379.6	ENSG00000256683.7	ENSG00000186517.14

16693	ENSG00000188383.8	ENSG00000256682.2	ENSG00000253330.1
16694	ENSG00000188385.11	ENSG00000256681.1	ENSG00000231345.3
16695	ENSG00000188386.7	ENSG00000256678.1	ENSG00000154175.17
16696	ENSG00000188388.10	ENSG00000256674.1	ENSG00000155465.18
16697	ENSG00000188389.11	ENSG00000256673.1	ENSG00000255857.5
16698	ENSG00000188393.8	ENSG00000256672.1	ENSG00000049247.13
16699	ENSG00000188394.6	ENSG00000256671.6	ENSG00000172350.10
16700	ENSG00000188396.3	ENSG00000256670.1	ENSG00000238260.1
16701	ENSG00000188399.5	ENSG00000256667.6	ENSG00000146221.10
16702	ENSG00000188403.7	ENSG00000256664.1	ENSG00000145721.12
16703	ENSG00000188404.9	ENSG00000256663.1	ENSG00000232412.1
16704	ENSG00000188408.5	ENSG00000256661.1	ENSG00000259219.1
16705	ENSG00000188419.14	ENSG00000256660.6	ENSG00000234571.1
16706	ENSG00000188425.3	ENSG00000256659.1	ENSG00000166589.13
16707	ENSG00000188428.20	ENSG00000256658.1	ENSG00000270906.1
16708	ENSG00000188438.4	ENSG00000256657.1	ENSG00000279819.1
16709	ENSG00000188439.3	ENSG00000256654.3	ENSG00000087510.7
16710	ENSG00000188451.8	ENSG00000256651.1	ENSG00000176387.7
16711	ENSG00000188452.14	ENSG00000256650.1	ENSG00000174672.16
16712	ENSG00000188459.4	ENSG00000256646.7	ENSG00000254585.4
16713	ENSG00000188460.4	ENSG00000256643.1	ENSG00000237481.1
16714	ENSG00000188467.10	ENSG00000256642.1	ENSG00000274508.1
16715	ENSG00000188483.7	ENSG00000256640.1	ENSG00000198053.11
16716	ENSG00000188486.3	ENSG00000256637.6	ENSG00000237927.1
16717	ENSG00000188487.11	ENSG00000256633.1	ENSG00000127241.16
16718	ENSG00000188488.14	ENSG00000256632.3	ENSG00000162944.10
16719	ENSG00000188493.15	ENSG00000256630.1	ENSG00000239470.3
16720	ENSG00000188501.11	ENSG00000256628.3	ENSG00000212930.3
16721	ENSG00000188505.5	ENSG00000256627.1	ENSG00000149489.8
16722	ENSG00000188508.11	ENSG00000256626.1	ENSG00000111237.18
16723	ENSG00000188511.12	ENSG00000256625.1	ENSG00000205863.10
16724	ENSG00000188512.6	ENSG00000256618.2	ENSG00000239650.4
16725	ENSG00000188517.16	ENSG00000256616.3	ENSG00000231535.6
16726	ENSG00000188522.15	ENSG00000256615.1	ENSG00000272732.1
16727	ENSG00000188523.8	ENSG00000256614.1	ENSG00000224464.2
16728	ENSG00000188525.3	ENSG00000256612.7	ENSG00000234261.3
16729	ENSG00000188529.14	ENSG00000256609.1	ENSG00000165118.15
16730	ENSG00000188536.13	ENSG00000256603.1	ENSG00000088833.17
16731	ENSG00000188542.9	ENSG00000256597.2	ENSG00000245156.1
16732	ENSG00000188549.12	ENSG00000256596.1	ENSG00000109758.8
16733	ENSG00000188554.14	ENSG00000256594.8	ENSG00000222037.5
16734	ENSG00000188558.6	ENSG00000256591.5	ENSG00000175938.7
16735	ENSG00000188559.15	ENSG00000256590.2	ENSG00000059122.16
16736	ENSG00000188566.13	ENSG00000256589.2	ENSG00000117868.16
16737	ENSG00000188573.7	ENSG00000256588.1	ENSG00000116748.21
16738	ENSG00000188580.14	ENSG00000256582.1	ENSG00000172568.5
16739	ENSG00000188581.9	ENSG00000256581.1	ENSG00000231948.2
16740	ENSG00000188582.8	ENSG00000256577.2	ENSG00000261242.1
16741	ENSG00000188585.9	ENSG00000256576.2	ENSG00000170017.12
16742	ENSG00000188596.11	ENSG00000256574.7	ENSG00000229000.1
16743	ENSG00000188599.17	ENSG00000256571.1	ENSG00000187984.12
16744	ENSG00000188603.19	ENSG00000256569.1	ENSG00000197410.13
16745	ENSG00000188610.12	ENSG00000256568.1	ENSG00000233980.1

16746	ENSG00000188611.14	ENSG00000256566.1	ENSG00000121905.10
16747	ENSG00000188612.12	ENSG00000256564.1	ENSG00000251192.7
16748	ENSG00000188613.7	ENSG00000256563.2	ENSG00000261342.1
16749	ENSG00000188620.10	ENSG00000256560.1	ENSG00000229196.3
16750	ENSG00000188624.3	ENSG00000256557.1	ENSG00000279129.1
16751	ENSG00000188626.6	ENSG00000256553.1	ENSG00000135776.5
16752	ENSG00000188629.12	ENSG00000256552.2	ENSG00000183856.11
16753	ENSG00000188636.4	ENSG00000256551.1	ENSG00000248356.1
16754	ENSG00000188641.13	ENSG00000256546.1	ENSG00000264229.1
16755	ENSG00000188643.11	ENSG00000256542.2	ENSG00000258457.5
16756	ENSG00000188646.6	ENSG00000256540.1	ENSG00000278066.1
16757	ENSG00000188647.13	ENSG00000256538.1	ENSG00000283703.2
16758	ENSG00000188649.14	ENSG00000256537.4	ENSG00000102007.11
16759	ENSG00000188655.10	ENSG00000256533.1	ENSG00000198106.8
16760	ENSG00000188656.8	ENSG00000256525.7	ENSG00000183292.13
16761	ENSG00000188659.9	ENSG00000256514.1	ENSG00000171877.21
16762	ENSG00000188660.4	ENSG00000256513.1	ENSG00000106009.16
16763	ENSG00000188662.6	ENSG00000256512.1	ENSG00000234515.1
16764	ENSG00000188668.4	ENSG00000256508.2	ENSG00000118997.14
16765	ENSG00000188672.18	ENSG00000256504.2	ENSG00000230679.1
16766	ENSG00000188674.11	ENSG00000256502.1	ENSG00000243230.1
16767	ENSG00000188676.13	ENSG00000256500.5	ENSG00000184277.13
16768	ENSG00000188677.14	ENSG00000256499.1	ENSG00000107018.8
16769	ENSG00000188681.11	ENSG00000256496.1	ENSG00000211637.2
16770	ENSG00000188687.17	ENSG00000256494.1	ENSG00000126106.14
16771	ENSG00000188690.14	ENSG00000256484.1	ENSG00000243023.2
16772	ENSG00000188691.5	ENSG00000256482.1	ENSG00000170500.12
16773	ENSG00000188693.7	ENSG00000256481.1	ENSG00000238280.2
16774	ENSG00000188694.5	ENSG00000256480.1	ENSG00000137070.17
16775	ENSG00000188706.13	ENSG00000256474.1	ENSG00000243141.2
16776	ENSG00000188707.6	ENSG00000256473.1	ENSG00000178982.9
16777	ENSG00000188710.2	ENSG00000256469.1	ENSG00000235297.3
16778	ENSG00000188712.4	ENSG00000256465.1	ENSG00000226652.3
16779	ENSG00000188716.5	ENSG00000256464.1	ENSG00000253172.1
16780	ENSG00000188725.8	ENSG00000256463.8	ENSG00000143257.11
16781	ENSG00000188729.6	ENSG00000256462.1	ENSG00000104332.12
16782	ENSG00000188730.5	ENSG00000256458.1	ENSG00000200693.2
16783	ENSG00000188732.11	ENSG00000256453.2	ENSG00000143409.15
16784	ENSG00000188735.13	ENSG00000256452.1	ENSG00000284268.1
16785	ENSG00000188738.15	ENSG00000256450.1	ENSG00000270681.1
16786	ENSG00000188739.15	ENSG00000256448.5	ENSG00000267234.1
16787	ENSG00000188747.8	ENSG00000256443.1	ENSG00000072163.19
16788	ENSG00000188755.10	ENSG00000256442.1	ENSG00000214593.3
16789	ENSG00000188760.10	ENSG00000256441.1	ENSG00000273894.1
16790	ENSG00000188761.13	ENSG00000256440.1	ENSG00000285625.1
16791	ENSG00000188763.4	ENSG00000256436.1	ENSG00000198868.3
16792	ENSG00000188765.7	ENSG00000256433.2	ENSG00000145868.16
16793	ENSG00000188766.12	ENSG00000256424.1	ENSG00000131015.5
16794	ENSG00000188770.9	ENSG00000256422.5	ENSG00000262495.1
16795	ENSG00000188771.4	ENSG00000256420.1	ENSG00000158352.15
16796	ENSG00000188778.6	ENSG00000256417.1	ENSG00000248590.2
16797	ENSG00000188779.11	ENSG00000256407.2	ENSG00000104518.11
16798	ENSG00000188782.8	ENSG00000256403.1	ENSG00000050130.18

16799	ENSG00000188783.6	ENSG00000256400.1	ENSG00000204764.14
16800	ENSG00000188784.4	ENSG00000256399.1	ENSG00000285081.1
16801	ENSG00000188785.12	ENSG00000256394.2	ENSG00000163239.12
16802	ENSG00000188786.10	ENSG00000256393.1	ENSG00000197299.12
16803	ENSG00000188800.6	ENSG00000256389.1	ENSG00000169248.12
16804	ENSG00000188801.9	ENSG00000256385.1	ENSG00000275332.1
16805	ENSG00000188803.14	ENSG00000256381.1	ENSG00000272183.1
16806	ENSG00000188807.13	ENSG00000256379.2	ENSG00000160282.14
16807	ENSG00000188811.14	ENSG00000256378.1	ENSG00000284505.1
16808	ENSG00000188816.3	ENSG00000256377.5	ENSG00000247934.4
16809	ENSG00000188817.7	ENSG00000256374.2	ENSG00000232648.3
16810	ENSG00000188818.12	ENSG00000256372.1	ENSG00000130270.16
16811	ENSG00000188820.13	ENSG00000256371.1	ENSG00000196739.15
16812	ENSG00000188822.8	ENSG00000256364.1	ENSG00000224020.1
16813	ENSG00000188825.13	ENSG00000256362.1	ENSG00000104497.14
16814	ENSG00000188827.11	ENSG00000256361.1	ENSG00000170448.12
16815	ENSG00000188828.11	ENSG00000256358.1	ENSG00000269032.1
16816	ENSG00000188831.4	ENSG00000256357.1	ENSG00000104112.9
16817	ENSG00000188833.9	ENSG00000256356.1	ENSG00000272148.1
16818	ENSG00000188846.13	ENSG00000256355.1	ENSG00000263321.1
16819	ENSG00000188848.16	ENSG00000256353.1	ENSG00000100307.13
16820	ENSG00000188850.9	ENSG00000256351.1	ENSG00000272430.1
16821	ENSG00000188856.6	ENSG00000256350.1	ENSG00000243167.1
16822	ENSG00000188859.6	ENSG00000256349.1	ENSG00000120675.6
16823	ENSG00000188868.13	ENSG00000256347.1	ENSG00000254941.1
16824	ENSG00000188869.13	ENSG00000256346.1	ENSG00000232259.1
16825	ENSG00000188873.4	ENSG00000256343.7	ENSG00000229314.5
16826	ENSG00000188877.12	ENSG00000256341.1	ENSG00000254231.2
16827	ENSG00000188878.19	ENSG00000256340.8	ENSG00000267054.1
16828	ENSG00000188883.4	ENSG00000256339.1	ENSG00000099203.7
16829	ENSG00000188886.3	ENSG00000256338.2	ENSG00000169908.12
16830	ENSG00000188895.11	ENSG00000256331.1	ENSG00000218980.2
16831	ENSG00000188897.9	ENSG00000256325.1	ENSG00000229268.1
16832	ENSG00000188906.16	ENSG00000256321.6	ENSG00000115756.13
16833	ENSG00000188909.4	ENSG00000256315.1	ENSG00000115318.12
16834	ENSG00000188910.8	ENSG00000256314.1	ENSG00000249679.1
16835	ENSG00000188916.9	ENSG00000256312.1	ENSG00000105379.9
16836	ENSG00000188917.15	ENSG00000256311.1	ENSG00000250990.1
16837	ENSG00000188921.14	ENSG00000256310.1	ENSG00000223881.1
16838	ENSG00000188931.3	ENSG00000256306.1	ENSG00000267282.1
16839	ENSG00000188933.15	ENSG00000256299.1	ENSG00000231081.1
16840	ENSG00000188937.5	ENSG00000256298.1	ENSG00000156413.13
16841	ENSG00000188938.17	ENSG00000256294.8	ENSG00000182771.19
16842	ENSG00000188958.9	ENSG00000256293.2	ENSG00000122952.17
16843	ENSG00000188959.9	ENSG00000256292.1	ENSG00000197380.11
16844	ENSG00000188976.11	ENSG00000256288.1	ENSG00000274011.1
16845	ENSG00000188981.11	ENSG00000256287.1	ENSG00000267265.5
16846	ENSG00000188984.12	ENSG00000256286.1	ENSG00000170819.4
16847	ENSG00000188985.6	ENSG00000256285.1	ENSG00000112658.8
16848	ENSG00000188986.7	ENSG00000256283.1	ENSG00000213694.5
16849	ENSG00000188991.3	ENSG00000256282.1	ENSG00000269486.2
16850	ENSG00000188992.11	ENSG00000256281.1	ENSG00000106105.14
16851	ENSG00000188993.3	ENSG00000256280.1	ENSG00000272158.1

16852	ENSG00000188994.13	ENSG00000256278.1	ENSG00000228008.1
16853	ENSG00000188996.4	ENSG00000256274.1	ENSG00000205268.10
16854	ENSG00000188997.8	ENSG00000256273.1	ENSG00000186529.16
16855	ENSG00000189001.11	ENSG00000256271.1	ENSG00000221887.6
16856	ENSG00000189002.10	ENSG00000256268.1	ENSG00000100575.14
16857	ENSG00000189007.16	ENSG00000256263.1	ENSG00000196683.10
16858	ENSG00000189013.14	ENSG00000256262.1	ENSG00000188013.6
16859	ENSG00000189014.7	ENSG00000256259.1	ENSG00000225849.2
16860	ENSG00000189023.10	ENSG00000256258.1	ENSG00000200354.1
16861	ENSG00000189030.9	ENSG00000256257.1	ENSG00000271576.1
16862	ENSG00000189037.7	ENSG00000256256.1	ENSG00000129353.15
16863	ENSG00000189042.13	ENSG00000256254.1	ENSG00000264663.1
16864	ENSG00000189043.10	ENSG00000256250.1	ENSG00000175874.10
16865	ENSG00000189045.13	ENSG00000256249.1	ENSG00000280655.1
16866	ENSG00000189046.11	ENSG00000256243.2	ENSG00000101222.12
16867	ENSG00000189050.16	ENSG00000256238.1	ENSG00000104856.14
16868	ENSG00000189051.5	ENSG00000256237.1	ENSG00000252010.1
16869	ENSG00000189052.7	ENSG00000256235.2	ENSG00000258947.7
16870	ENSG00000189056.14	ENSG00000256234.1	ENSG00000101104.12
16871	ENSG00000189057.11	ENSG00000256232.1	ENSG00000075043.18
16872	ENSG00000189058.9	ENSG00000256229.8	ENSG00000225665.4
16873	ENSG00000189060.5	ENSG00000256226.1	ENSG00000213033.4
16874	ENSG00000189064.8	ENSG00000256223.6	ENSG00000268149.1
16875	ENSG00000189067.12	ENSG00000256222.2	ENSG00000213137.3
16876	ENSG00000189068.11	ENSG00000256221.1	ENSG00000231544.3
16877	ENSG00000189077.11	ENSG00000256220.1	ENSG00000204086.4
16878	ENSG00000189079.16	ENSG00000256218.1	ENSG00000239948.2
16879	ENSG00000189089.5	ENSG00000256211.1	ENSG00000178343.5
16880	ENSG00000189090.7	ENSG00000256210.3	ENSG00000264275.1
16881	ENSG00000189091.13	ENSG00000256209.1	ENSG00000173011.12
16882	ENSG00000189099.11	ENSG00000256206.2	ENSG00000236054.1
16883	ENSG00000189108.12	ENSG00000256204.1	ENSG00000224565.1
16884	ENSG00000189114.6	ENSG00000256199.1	ENSG00000234782.3
16885	ENSG00000189120.5	ENSG00000256197.1	ENSG00000171049.9
16886	ENSG00000189127.8	ENSG00000256196.1	ENSG00000174804.4
16887	ENSG00000189129.13	ENSG00000256195.2	ENSG00000248503.2
16888	ENSG00000189132.6	ENSG00000256193.5	ENSG00000255968.1
16889	ENSG00000189134.3	ENSG00000256192.1	ENSG00000146411.5
16890	ENSG00000189136.9	ENSG00000256189.1	ENSG00000203395.2
16891	ENSG00000189139.5	ENSG00000256188.3	ENSG00000091181.19
16892	ENSG00000189143.9	ENSG00000256185.1	ENSG00000258711.2
16893	ENSG00000189144.13	ENSG00000256181.1	ENSG00000213169.3
16894	ENSG00000189145.7	ENSG00000256176.1	ENSG00000224566.2
16895	ENSG00000189149.12	ENSG00000256172.1	ENSG00000170893.4
16896	ENSG00000189152.10	ENSG00000256171.1	ENSG00000250219.2
16897	ENSG00000189157.14	ENSG00000256167.1	ENSG00000232093.1
16898	ENSG00000189159.16	ENSG00000256166.1	ENSG00000279123.1
16899	ENSG00000189164.15	ENSG00000256162.2	ENSG00000163517.15
16900	ENSG00000189166.6	ENSG00000256159.1	ENSG00000231767.4
16901	ENSG00000189167.11	ENSG00000256157.1	ENSG00000130158.14
16902	ENSG00000189169.7	ENSG00000256155.1	ENSG00000101773.19
16903	ENSG00000189171.14	ENSG00000256152.2	ENSG00000059145.18
16904	ENSG00000189180.15	ENSG00000256151.1	ENSG00000165807.8

16905	ENSG00000189181.5	ENSG00000256150.2	ENSG00000114993.16
16906	ENSG00000189182.9	ENSG00000256149.1	ENSG00000198001.13
16907	ENSG00000189186.10	ENSG00000256148.1	ENSG00000273226.1
16908	ENSG00000189190.10	ENSG00000256146.1	ENSG00000229666.1
16909	ENSG00000189195.13	ENSG00000256139.2	ENSG00000282651.2
16910	ENSG00000189196.4	ENSG00000256138.1	ENSG00000280399.1
16911	ENSG00000189212.12	ENSG00000256137.1	ENSG00000213060.4
16912	ENSG00000189221.9	ENSG00000256136.1	ENSG00000261324.2
16913	ENSG00000189223.14	ENSG00000256134.1	ENSG00000081041.9
16914	ENSG00000189227.6	ENSG00000256128.5	ENSG00000213222.3
16915	ENSG00000189229.10	ENSG00000256125.1	ENSG00000264324.1
16916	ENSG00000189233.12	ENSG00000256124.5	ENSG00000213045.3
16917	ENSG00000189238.5	ENSG00000256120.1	ENSG00000070526.15
16918	ENSG00000189241.7	ENSG00000256116.1	ENSG00000260526.1
16919	ENSG00000189252.4	ENSG00000256115.5	ENSG00000285664.1
16920	ENSG00000189253.7	ENSG00000256108.1	ENSG00000145945.6
16921	ENSG00000189266.13	ENSG00000256103.2	ENSG00000250820.1
16922	ENSG00000189269.12	ENSG00000256101.5	ENSG00000275183.1
16923	ENSG00000189275.3	ENSG00000256100.1	ENSG00000266553.2
16924	ENSG00000189280.3	ENSG00000256098.1	ENSG00000213149.3
16925	ENSG00000189283.10	ENSG00000256093.1	ENSG00000268355.1
16926	ENSG00000189292.16	ENSG00000256092.2	ENSG00000283239.1
16927	ENSG00000189295.13	ENSG00000256091.1	ENSG00000253439.1
16928	ENSG00000189298.14	ENSG00000256087.7	ENSG00000029639.11
16929	ENSG00000189299.6	ENSG00000256085.1	ENSG00000278989.1
16930	ENSG00000189306.11	ENSG00000256084.1	ENSG00000147394.18
16931	ENSG00000189308.11	ENSG00000256083.1	ENSG00000178660.6
16932	ENSG00000189316.3	ENSG00000256081.2	ENSG00000280594.1
16933	ENSG00000189319.14	ENSG00000256079.1	ENSG00000267379.1
16934	ENSG00000189320.9	ENSG00000256077.5	ENSG00000269153.1
16935	ENSG00000189325.7	ENSG00000256075.1	ENSG00000164675.11
16936	ENSG00000189326.4	ENSG00000256073.3	ENSG00000236300.2
16937	ENSG00000189332.5	ENSG00000256072.1	ENSG00000171217.5
16938	ENSG00000189334.9	ENSG00000256071.1	ENSG00000272733.1
16939	ENSG00000189337.17	ENSG00000256070.1	ENSG00000235481.2
16940	ENSG00000189339.11	ENSG00000256069.7	ENSG00000261245.2
16941	ENSG00000189343.7	ENSG00000256064.1	ENSG00000102390.10
16942	ENSG00000189348.6	ENSG00000256061.7	ENSG00000224729.5
16943	ENSG00000189350.12	ENSG00000256060.2	ENSG00000282936.1
16944	ENSG00000189357.8	ENSG00000256056.1	ENSG00000095787.22
16945	ENSG00000189362.12	ENSG00000256053.7	ENSG00000164161.10
16946	ENSG00000189366.9	ENSG00000256050.2	ENSG00000263585.1
16947	ENSG00000189367.15	ENSG00000256045.2	ENSG00000233974.3
16948	ENSG00000189369.8	ENSG00000256044.1	ENSG00000268565.1
16949	ENSG00000189372.4	ENSG00000256043.3	ENSG00000235760.4
16950	ENSG00000189375.10	ENSG00000256041.1	ENSG00000226479.4
16951	ENSG00000189376.12	ENSG00000256040.2	ENSG00000254788.7
16952	ENSG00000189377.8	ENSG00000256039.1	ENSG00000236480.1
16953	ENSG00000189398.5	ENSG00000256037.1	ENSG00000270504.1
16954	ENSG00000189401.2	ENSG00000256035.1	ENSG00000225255.6
16955	ENSG00000189403.15	ENSG00000256034.1	ENSG00000270228.1
16956	ENSG00000189409.13	ENSG00000256030.1	ENSG00000213036.3
16957	ENSG00000189410.12	ENSG00000256029.6	ENSG00000250917.1

16958	ENSG00000189419.7	ENSG00000256028.2	ENSG00000204450.8
16959	ENSG00000189420.8	ENSG00000256025.1	ENSG00000282143.1
16960	ENSG00000189423.12	ENSG00000256022.5	ENSG00000277726.4
16961	ENSG00000189430.12	ENSG00000256021.1	ENSG00000243433.1
16962	ENSG00000189431.7	ENSG00000256020.1	ENSG00000243659.1
16963	ENSG00000189433.6	ENSG00000256019.1	ENSG00000177291.3
16964	ENSG00000194297.2	ENSG00000256013.1	ENSG00000254485.5
16965	ENSG00000194717.4	ENSG00000256011.1	ENSG00000238186.1
16966	ENSG00000195024.2	ENSG00000256008.2	ENSG00000232916.1
16967	ENSG00000195401.2	ENSG00000256006.1	ENSG00000178222.13
16968	ENSG00000196071.5	ENSG00000256004.1	ENSG00000275417.1
16969	ENSG00000196072.12	ENSG00000256001.1	ENSG00000230107.1
16970	ENSG00000196074.13	ENSG00000255998.1	ENSG00000196562.14
16971	ENSG00000196081.9	ENSG00000255996.1	ENSG00000226272.5
16972	ENSG00000196083.10	ENSG00000255995.1	ENSG00000184154.14
16973	ENSG00000196085.5	ENSG00000255993.1	ENSG00000109927.10
16974	ENSG00000196090.12	ENSG00000255992.1	ENSG00000100376.12
16975	ENSG00000196091.13	ENSG00000255991.2	ENSG00000077238.14
16976	ENSG00000196092.13	ENSG00000255988.1	ENSG00000279812.1
16977	ENSG00000196096.3	ENSG00000255987.1	ENSG00000273204.1
16978	ENSG00000196098.2	ENSG00000255986.6	ENSG00000254718.6
16979	ENSG00000196099.4	ENSG00000255983.1	ENSG00000249210.1
16980	ENSG00000196104.11	ENSG00000255982.1	ENSG00000143401.15
16981	ENSG00000196109.8	ENSG00000255980.1	ENSG00000100065.15
16982	ENSG00000196110.7	ENSG00000255977.1	ENSG00000132740.8
16983	ENSG00000196114.3	ENSG00000255976.1	ENSG00000267679.1
16984	ENSG00000196115.13	ENSG00000255974.8	ENSG00000163352.5
16985	ENSG00000196116.8	ENSG00000255973.1	ENSG00000125997.5
16986	ENSG00000196118.12	ENSG00000255972.1	ENSG00000211649.3
16987	ENSG00000196119.7	ENSG00000255970.1	ENSG00000253870.1
16988	ENSG00000196123.13	ENSG00000255968.1	ENSG00000217648.1
16989	ENSG00000196126.11	ENSG00000255967.1	ENSG00000127824.14
16990	ENSG00000196131.6	ENSG00000255966.1	ENSG00000189377.8
16991	ENSG00000196132.13	ENSG00000255965.1	ENSG00000077327.16
16992	ENSG00000196136.17	ENSG00000255964.1	ENSG00000228976.1
16993	ENSG00000196139.14	ENSG00000255960.1	ENSG00000215110.7
16994	ENSG00000196141.14	ENSG00000255959.1	ENSG00000174225.14
16995	ENSG00000196143.4	ENSG00000255958.1	ENSG00000272983.1
16996	ENSG00000196150.13	ENSG00000255951.1	ENSG00000272219.1
16997	ENSG00000196151.10	ENSG00000255949.1	ENSG00000230899.1
16998	ENSG00000196152.10	ENSG00000255947.1	ENSG00000116729.14
16999	ENSG00000196154.12	ENSG00000255946.1	ENSG00000261251.1
17000	ENSG00000196155.13	ENSG00000255945.1	ENSG00000142224.15
17001	ENSG00000196156.4	ENSG00000255944.1	ENSG00000285171.1
17002	ENSG00000196159.11	ENSG00000255933.1	ENSG00000183785.15
17003	ENSG00000196166.4	ENSG00000255931.1	ENSG00000231234.2
17004	ENSG00000196167.9	ENSG00000255929.5	ENSG00000215910.7
17005	ENSG00000196169.15	ENSG00000255928.1	ENSG00000144214.9
17006	ENSG00000196171.3	ENSG00000255923.1	ENSG00000163297.17
17007	ENSG00000196172.9	ENSG00000255921.1	ENSG00000250989.1
17008	ENSG00000196177.12	ENSG00000255919.1	ENSG00000156463.18
17009	ENSG00000196182.10	ENSG00000255916.1	ENSG00000205847.6
17010	ENSG00000196183.5	ENSG00000255910.1	ENSG00000241770.1

17011	ENSG00000196184.9	ENSG00000255909.1	ENSG00000163430.12
17012	ENSG00000196187.12	ENSG00000255903.2	ENSG00000025156.12
17013	ENSG00000196188.11	ENSG00000255900.1	ENSG00000271581.1
17014	ENSG00000196189.13	ENSG00000255899.1	ENSG00000136478.7
17015	ENSG00000196196.3	ENSG00000255893.1	ENSG00000149656.8
17016	ENSG00000196199.14	ENSG00000255892.1	ENSG00000224397.6
17017	ENSG00000196204.11	ENSG00000255886.1	ENSG00000067601.8
17018	ENSG00000196205.8	ENSG00000255885.1	ENSG00000227688.2
17019	ENSG00000196208.14	ENSG00000255883.1	ENSG00000138379.4
17020	ENSG00000196209.12	ENSG00000255882.1	ENSG00000273338.1
17021	ENSG00000196214.11	ENSG00000255875.2	ENSG00000258325.2
17022	ENSG00000196218.12	ENSG00000255874.2	ENSG00000278909.1
17023	ENSG00000196220.16	ENSG00000255872.3	ENSG00000232040.3
17024	ENSG00000196224.7	ENSG00000255871.2	ENSG00000259086.3
17025	ENSG00000196227.11	ENSG00000255870.1	ENSG00000162341.17
17026	ENSG00000196228.3	ENSG00000255867.1	ENSG00000263934.4
17027	ENSG00000196230.13	ENSG00000255866.1	ENSG00000219712.1
17028	ENSG00000196233.13	ENSG00000255863.2	ENSG00000130045.16
17029	ENSG00000196235.14	ENSG00000255860.3	ENSG00000106538.10
17030	ENSG00000196236.13	ENSG00000255858.1	ENSG00000260509.2
17031	ENSG00000196240.4	ENSG00000255857.5	ENSG00000159208.16
17032	ENSG00000196242.9	ENSG00000255856.2	ENSG00000244045.13
17033	ENSG00000196243.5	ENSG00000255855.2	ENSG00000279491.1
17034	ENSG00000196247.11	ENSG00000255853.1	ENSG00000100554.12
17035	ENSG00000196248.5	ENSG00000255851.2	ENSG00000279690.1
17036	ENSG00000196260.5	ENSG00000255850.2	ENSG00000139289.13
17037	ENSG00000196262.14	ENSG00000255847.5	ENSG00000125409.13
17038	ENSG00000196263.8	ENSG00000255845.1	ENSG00000198598.6
17039	ENSG00000196266.5	ENSG00000255843.1	ENSG00000121931.16
17040	ENSG00000196267.12	ENSG00000255839.1	ENSG00000259810.2
17041	ENSG00000196268.11	ENSG00000255838.1	ENSG00000241721.1
17042	ENSG00000196273.7	ENSG00000255837.1	ENSG00000230154.1
17043	ENSG00000196274.5	ENSG00000255836.1	ENSG00000087250.8
17044	ENSG00000196275.14	ENSG00000255835.1	ENSG00000214946.14
17045	ENSG00000196277.16	ENSG00000255833.2	ENSG00000187733.6
17046	ENSG00000196284.16	ENSG00000255830.2	ENSG00000166886.13
17047	ENSG00000196289.7	ENSG00000255829.1	ENSG00000078061.13
17048	ENSG00000196290.15	ENSG00000255825.1	ENSG00000108797.12
17049	ENSG00000196295.11	ENSG00000255823.4	ENSG00000233719.4
17050	ENSG00000196296.13	ENSG00000255819.7	ENSG00000214265.11
17051	ENSG00000196301.3	ENSG00000255817.1	ENSG00000148834.13
17052	ENSG00000196302.5	ENSG00000255815.3	ENSG00000274156.1
17053	ENSG00000196305.17	ENSG00000255814.1	ENSG00000173546.7
17054	ENSG00000196312.14	ENSG00000255811.1	ENSG00000091136.14
17055	ENSG00000196313.11	ENSG00000255808.1	ENSG00000214194.9
17056	ENSG00000196323.14	ENSG00000255807.1	ENSG00000105993.15
17057	ENSG00000196329.11	ENSG00000255804.2	ENSG00000083814.13
17058	ENSG00000196335.13	ENSG00000255801.1	ENSG00000177383.4
17059	ENSG00000196337.11	ENSG00000255794.7	ENSG00000156831.8
17060	ENSG00000196338.12	ENSG00000255790.5	ENSG00000214694.11
17061	ENSG00000196341.3	ENSG00000255786.1	ENSG00000236345.1
17062	ENSG00000196344.11	ENSG00000255780.1	ENSG00000272361.2
17063	ENSG00000196345.13	ENSG00000255776.1	ENSG00000184319.16

17064	ENSG00000196350.8	ENSG00000255775.1	ENSG00000234801.3
17065	ENSG00000196352.15	ENSG00000255774.2	ENSG00000269961.1
17066	ENSG00000196353.11	ENSG00000255772.5	ENSG00000278970.2
17067	ENSG00000196357.11	ENSG00000255769.7	ENSG00000256950.2
17068	ENSG00000196358.11	ENSG00000255767.1	ENSG00000284427.1
17069	ENSG00000196361.10	ENSG00000255763.1	ENSG00000184144.12
17070	ENSG00000196363.9	ENSG00000255760.1	ENSG00000258465.7
17071	ENSG00000196364.9	ENSG00000255757.1	ENSG00000225715.1
17072	ENSG00000196365.11	ENSG00000255753.1	ENSG00000264707.1
17073	ENSG00000196366.3	ENSG00000255750.5	ENSG00000096746.17
17074	ENSG00000196367.13	ENSG00000255749.1	ENSG00000185666.14
17075	ENSG00000196368.5	ENSG00000255746.1	ENSG00000182180.14
17076	ENSG00000196369.11	ENSG00000255745.1	ENSG00000217181.1
17077	ENSG00000196371.3	ENSG00000255741.1	ENSG00000234450.1
17078	ENSG00000196372.13	ENSG00000255734.1	ENSG00000215559.8
17079	ENSG00000196376.11	ENSG00000255733.5	ENSG00000256427.2
17080	ENSG00000196378.11	ENSG00000255730.5	ENSG00000136872.19
17081	ENSG00000196381.11	ENSG00000255727.1	ENSG00000167644.12
17082	ENSG00000196383.6	ENSG00000255726.1	ENSG00000137310.12
17083	ENSG00000196387.9	ENSG00000255723.1	ENSG00000259926.1
17084	ENSG00000196388.8	ENSG00000255717.6	ENSG00000260293.2
17085	ENSG00000196390.6	ENSG00000255714.1	ENSG00000258760.1
17086	ENSG00000196391.10	ENSG00000255713.2	ENSG00000215126.10
17087	ENSG00000196395.7	ENSG00000255710.2	ENSG00000240184.7
17088	ENSG00000196396.10	ENSG00000255704.1	ENSG00000278918.1
17089	ENSG00000196403.4	ENSG00000255703.1	ENSG00000175509.8
17090	ENSG00000196405.13	ENSG00000255700.2	ENSG00000254858.9
17091	ENSG00000196406.4	ENSG00000255693.2	ENSG00000254612.2
17092	ENSG00000196407.12	ENSG00000255692.1	ENSG00000125434.11
17093	ENSG00000196408.11	ENSG00000255690.2	ENSG00000099822.3
17094	ENSG00000196411.10	ENSG00000255689.1	ENSG00000218265.1
17095	ENSG00000196415.10	ENSG00000255686.1	ENSG00000211891.6
17096	ENSG00000196417.13	ENSG00000255680.1	ENSG00000251301.6
17097	ENSG00000196418.12	ENSG00000255679.1	ENSG00000007047.15
17098	ENSG00000196419.12	ENSG00000255672.1	ENSG00000272921.1
17099	ENSG00000196420.7	ENSG00000255671.1	ENSG00000280033.1
17100	ENSG00000196421.8	ENSG00000255670.1	ENSG00000279275.1
17101	ENSG00000196422.11	ENSG00000255669.2	ENSG00000222460.1
17102	ENSG00000196427.13	ENSG00000255666.6	ENSG00000196433.13
17103	ENSG00000196428.12	ENSG00000255664.1	ENSG00000170439.7
17104	ENSG00000196431.4	ENSG00000255663.1	ENSG00000169203.16
17105	ENSG00000196433.13	ENSG00000255660.1	ENSG00000230105.1
17106	ENSG00000196433.13_PAR_Y	ENSG00000255655.1	ENSG00000237493.3
17107	ENSG00000196436.8	ENSG00000255653.1	ENSG00000105642.15
17108	ENSG00000196437.11	ENSG00000255652.3	ENSG00000231087.2
17109	ENSG00000196440.11	ENSG00000255650.5	ENSG00000106392.10
17110	ENSG00000196449.4	ENSG00000255649.1	ENSG00000267064.1
17111	ENSG00000196453.8	ENSG00000255648.1	ENSG00000259970.1
17112	ENSG00000196455.8	ENSG00000255647.3	ENSG00000229985.2
17113	ENSG00000196456.12	ENSG00000255644.1	ENSG00000280439.1
17114	ENSG00000196458.11	ENSG00000255642.1	ENSG00000260918.1
17115	ENSG00000196459.14	ENSG00000255641.1	ENSG00000182240.16
17116	ENSG00000196460.14	ENSG00000255639.3	ENSG00000179387.10

17117	ENSG00000196465.10	ENSG00000255633.5	ENSG00000266916.6
17118	ENSG00000196466.10	ENSG00000255629.1	ENSG00000267534.4
17119	ENSG00000196468.7	ENSG00000255628.1	ENSG00000271254.6
17120	ENSG00000196470.12	ENSG00000255627.1	ENSG00000146242.8
17121	ENSG00000196472.4	ENSG00000255624.3	ENSG00000156265.15
17122	ENSG00000196475.5	ENSG00000255622.3	ENSG00000273184.1
17123	ENSG00000196476.12	ENSG00000255621.1	ENSG00000166925.9
17124	ENSG00000196482.17	ENSG00000255618.1	ENSG00000075213.11
17125	ENSG00000196497.17	ENSG00000255608.1	ENSG00000275088.1
17126	ENSG00000196498.13	ENSG00000255606.1	ENSG00000182487.12
17127	ENSG00000196502.11	ENSG00000255605.1	ENSG00000126254.12
17128	ENSG00000196503.4	ENSG00000255599.1	ENSG00000099256.19
17129	ENSG00000196504.16	ENSG00000255595.4	ENSG00000242493.3
17130	ENSG00000196505.11	ENSG00000255587.9	ENSG00000188152.12
17131	ENSG00000196507.11	ENSG00000255585.3	ENSG00000274105.1
17132	ENSG00000196510.12	ENSG00000255583.2	ENSG00000251389.1
17133	ENSG00000196511.14	ENSG00000255582.1	ENSG00000272954.1
17134	ENSG00000196517.11	ENSG00000255581.1	ENSG00000237227.1
17135	ENSG00000196526.10	ENSG00000255580.1	ENSG00000122557.10
17136	ENSG00000196531.10	ENSG00000255575.1	ENSG00000170846.17
17137	ENSG00000196534.5	ENSG00000255572.1	ENSG00000260360.1
17138	ENSG00000196535.16	ENSG00000255571.8	ENSG00000139910.20
17139	ENSG00000196539.3	ENSG00000255569.1	ENSG00000250384.1
17140	ENSG00000196542.8	ENSG00000255568.3	ENSG00000264546.1
17141	ENSG00000196544.7	ENSG00000255566.1	ENSG00000179528.16
17142	ENSG00000196547.15	ENSG00000255565.1	ENSG00000234769.7
17143	ENSG00000196549.10	ENSG00000255563.1	ENSG00000114439.19
17144	ENSG00000196550.10	ENSG00000255562.2	ENSG00000266598.1
17145	ENSG00000196553.15	ENSG00000255561.7	ENSG00000273703.1
17146	ENSG00000196557.13	ENSG00000255560.1	ENSG00000162407.9
17147	ENSG00000196562.14	ENSG00000255559.1	ENSG00000241839.10
17148	ENSG00000196564.4	ENSG00000255558.1	ENSG00000241553.12
17149	ENSG00000196565.15	ENSG00000255557.1	ENSG00000259025.1
17150	ENSG00000196566.2	ENSG00000255556.2	ENSG00000226567.1
17151	ENSG00000196569.12	ENSG00000255555.1	ENSG00000274859.1
17152	ENSG00000196570.3	ENSG00000255554.1	ENSG00000228192.7
17153	ENSG00000196576.15	ENSG00000255553.1	ENSG00000186197.14
17154	ENSG00000196581.10	ENSG00000255552.7	ENSG00000254783.1
17155	ENSG00000196584.3	ENSG00000255551.1	ENSG00000226457.1
17156	ENSG00000196586.14	ENSG00000255550.1	ENSG00000227413.1
17157	ENSG00000196588.16	ENSG00000255549.2	ENSG00000269815.1
17158	ENSG00000196589.6	ENSG00000255548.1	ENSG00000223482.7
17159	ENSG00000196591.12	ENSG00000255547.1	ENSG00000060140.9
17160	ENSG00000196593.9	ENSG00000255545.7	ENSG00000229894.4
17161	ENSG00000196597.12	ENSG00000255544.2	ENSG00000173991.5
17162	ENSG00000196600.12	ENSG00000255543.1	ENSG00000228057.1
17163	ENSG00000196604.12	ENSG00000255542.1	ENSG00000105784.15
17164	ENSG00000196605.7	ENSG00000255541.1	ENSG00000234961.1
17165	ENSG00000196611.5	ENSG00000255540.1	ENSG00000136531.16
17166	ENSG00000196616.14	ENSG00000255539.1	ENSG00000132024.17
17167	ENSG00000196620.10	ENSG00000255538.1	ENSG00000271138.1
17168	ENSG00000196628.16	ENSG00000255537.1	ENSG00000132646.11
17169	ENSG00000196632.10	ENSG00000255536.1	ENSG00000225075.1

17170	ENSG00000196634.3	ENSG00000255535.2	ENSG00000174715.7
17171	ENSG00000196636.7	ENSG00000255534.1	ENSG00000143376.14
17172	ENSG00000196639.6	ENSG00000255533.1	ENSG00000278942.1
17173	ENSG00000196642.19	ENSG00000255532.1	ENSG00000079435.10
17174	ENSG00000196646.12	ENSG00000255531.1	ENSG00000284726.1
17175	ENSG00000196652.11	ENSG00000255530.1	ENSG00000279699.1
17176	ENSG00000196653.12	ENSG00000255529.9	ENSG00000162631.18
17177	ENSG00000196655.11	ENSG00000255528.1	ENSG00000260772.1
17178	ENSG00000196656.7	ENSG00000255527.1	ENSG00000169181.13
17179	ENSG00000196659.9	ENSG00000255526.6	ENSG00000163848.19
17180	ENSG00000196660.10	ENSG00000255525.1	ENSG00000050820.17
17181	ENSG00000196663.16	ENSG00000255524.7	ENSG00000235095.1
17182	ENSG00000196664.5	ENSG00000255523.1	ENSG00000278982.1
17183	ENSG00000196666.5	ENSG00000255522.1	ENSG00000143353.12
17184	ENSG00000196668.3	ENSG00000255521.1	ENSG00000197780.9
17185	ENSG00000196670.13	ENSG00000255520.1	ENSG00000100603.13
17186	ENSG00000196678.14	ENSG00000255519.1	ENSG00000272273.1
17187	ENSG00000196683.10	ENSG00000255517.6	ENSG00000265750.1
17188	ENSG00000196684.12	ENSG00000255516.1	ENSG00000148053.16
17189	ENSG00000196689.12	ENSG00000255515.1	ENSG00000175164.15
17190	ENSG00000196693.15	ENSG00000255514.1	ENSG00000280426.1
17191	ENSG00000196696.12	ENSG00000255513.1	ENSG00000185022.12
17192	ENSG00000196700.9	ENSG00000255512.1	ENSG00000247095.2
17193	ENSG00000196704.12	ENSG00000255511.1	ENSG00000254441.1
17194	ENSG00000196705.8	ENSG00000255510.1	ENSG00000175779.2
17195	ENSG00000196711.9	ENSG00000255507.5	ENSG00000181817.6
17196	ENSG00000196712.17	ENSG00000255506.1	ENSG00000247925.2
17197	ENSG00000196715.7	ENSG00000255505.1	ENSG00000227407.1
17198	ENSG00000196724.12	ENSG00000255504.2	ENSG00000261404.7
17199	ENSG00000196730.13	ENSG00000255503.1	ENSG00000187783.12
17200	ENSG00000196734.8	ENSG00000255502.5	ENSG00000214832.5
17201	ENSG00000196735.11	ENSG00000255501.2	ENSG00000162522.11
17202	ENSG00000196739.15	ENSG00000255500.2	ENSG00000250120.7
17203	ENSG00000196741.5	ENSG00000255499.1	ENSG00000117525.14
17204	ENSG00000196743.8	ENSG00000255498.1	ENSG00000099625.13
17205	ENSG00000196747.4	ENSG00000255497.1	ENSG00000167261.14
17206	ENSG00000196748.10	ENSG00000255496.1	ENSG00000280425.2
17207	ENSG00000196754.10	ENSG00000255495.1	ENSG00000222872.1
17208	ENSG00000196756.12	ENSG00000255494.1	ENSG00000147533.16
17209	ENSG00000196757.8	ENSG00000255493.2	ENSG00000265916.1
17210	ENSG00000196758.3	ENSG00000255492.1	ENSG00000250021.7
17211	ENSG00000196767.8	ENSG00000255491.2	ENSG00000269907.1
17212	ENSG00000196772.4	ENSG00000255490.1	ENSG00000262151.1
17213	ENSG00000196776.16	ENSG00000255489.1	ENSG00000280543.1
17214	ENSG00000196778.3	ENSG00000255487.1	ENSG00000274598.1
17215	ENSG00000196781.15	ENSG00000255486.1	ENSG00000231748.1
17216	ENSG00000196782.12	ENSG00000255485.1	ENSG00000103534.17
17217	ENSG00000196787.3	ENSG00000255484.2	ENSG00000124391.4
17218	ENSG00000196792.12	ENSG00000255483.1	ENSG00000250999.1
17219	ENSG00000196793.13	ENSG00000255482.1	ENSG00000225969.2
17220	ENSG00000196796.5	ENSG00000255481.2	ENSG00000110080.18
17221	ENSG00000196800.6	ENSG00000255480.1	ENSG00000108823.16
17222	ENSG00000196805.7	ENSG00000255479.1	ENSG00000172901.20

17223	ENSG00000196810.4	ENSG00000255478.1	ENSG00000270503.1
17224	ENSG00000196811.12	ENSG00000255477.1	ENSG00000165416.14
17225	ENSG00000196812.5	ENSG00000255476.1	ENSG00000225648.5
17226	ENSG00000196814.15	ENSG00000255475.1	ENSG00000279329.1
17227	ENSG00000196821.10	ENSG00000255474.1	ENSG00000218018.2
17228	ENSG00000196826.7	ENSG00000255472.1	ENSG00000266086.2
17229	ENSG00000196832.4	ENSG00000255471.1	ENSG00000275024.1
17230	ENSG00000196834.12	ENSG00000255470.1	ENSG00000234129.7
17231	ENSG00000196839.13	ENSG00000255469.1	ENSG00000261837.1
17232	ENSG00000196843.16	ENSG00000255468.7	ENSG00000225282.1
17233	ENSG00000196844.8	ENSG00000255467.1	ENSG00000204110.6
17234	ENSG00000196850.6	ENSG00000255466.1	ENSG00000103528.17
17235	ENSG00000196859.7	ENSG00000255464.1	ENSG00000247345.2
17236	ENSG00000196860.8	ENSG00000255463.1	ENSG00000282386.1
17237	ENSG00000196862.9	ENSG00000255462.1	ENSG00000249274.1
17238	ENSG00000196865.4	ENSG00000255461.1	ENSG00000254852.8
17239	ENSG00000196866.2	ENSG00000255460.1	ENSG00000179636.15
17240	ENSG00000196867.7	ENSG00000255459.1	ENSG00000172824.16
17241	ENSG00000196872.12	ENSG00000255458.6	ENSG00000203865.9
17242	ENSG00000196873.15	ENSG00000255457.1	ENSG00000280067.1
17243	ENSG00000196876.15	ENSG00000255456.1	ENSG00000101470.10
17244	ENSG00000196878.15	ENSG00000255455.2	ENSG00000156222.12
17245	ENSG00000196890.4	ENSG00000255454.1	ENSG00000073111.14
17246	ENSG00000196893.3	ENSG00000255452.1	ENSG00000184481.16
17247	ENSG00000196900.5	ENSG00000255451.1	ENSG00000269956.1
17248	ENSG00000196911.10	ENSG00000255450.1	ENSG00000271428.1
17249	ENSG00000196912.12	ENSG00000255449.1	ENSG00000237356.6
17250	ENSG00000196914.9	ENSG00000255448.1	ENSG00000264569.1
17251	ENSG00000196917.5	ENSG00000255447.1	ENSG00000260183.1
17252	ENSG00000196922.10	ENSG00000255446.1	ENSG00000214016.3
17253	ENSG00000196923.14	ENSG00000255445.1	ENSG00000245293.2
17254	ENSG00000196924.16	ENSG00000255444.1	ENSG00000264443.1
17255	ENSG00000196932.11	ENSG00000255443.1	ENSG00000274667.1
17256	ENSG00000196933.5	ENSG00000255442.1	ENSG00000265190.6
17257	ENSG00000196935.9	ENSG00000255441.1	ENSG00000261604.1
17258	ENSG00000196937.11	ENSG00000255440.1	ENSG00000204010.3
17259	ENSG00000196943.14	ENSG00000255439.6	ENSG00000170091.11
17260	ENSG00000196944.4	ENSG00000255438.2	ENSG00000143373.18
17261	ENSG00000196946.10	ENSG00000255437.1	ENSG00000253394.5
17262	ENSG00000196950.14	ENSG00000255434.1	ENSG00000227176.1
17263	ENSG00000196951.10	ENSG00000255433.5	ENSG00000284630.1
17264	ENSG00000196954.14	ENSG00000255432.1	ENSG00000259984.1
17265	ENSG00000196961.12	ENSG00000255431.1	ENSG00000280109.3
17266	ENSG00000196967.10	ENSG00000255430.1	ENSG00000204304.12
17267	ENSG00000196968.11	ENSG00000255429.1	ENSG00000153012.12
17268	ENSG00000196970.8	ENSG00000255428.1	ENSG00000232611.1
17269	ENSG00000196975.16	ENSG00000255427.1	ENSG00000107242.18
17270	ENSG00000196976.7	ENSG00000255426.1	ENSG00000267318.1
17271	ENSG00000196979.1	ENSG00000255425.5	ENSG00000224187.1
17272	ENSG00000196981.3	ENSG00000255424.1	ENSG00000225024.1
17273	ENSG00000196990.8	ENSG00000255423.1	ENSG00000031691.7
17274	ENSG00000196993.8	ENSG00000255422.3	ENSG00000114487.9
17275	ENSG00000196998.18	ENSG00000255421.1	ENSG00000265972.5

17276	ENSG00000197006.14	ENSG00000255420.1	ENSG00000145063.14
17277	ENSG00000197008.9	ENSG00000255418.5	ENSG00000164241.13
17278	ENSG00000197013.10	ENSG00000255417.1	ENSG00000246323.2
17279	ENSG00000197016.12	ENSG00000255416.1	ENSG00000103199.13
17280	ENSG00000197019.5	ENSG00000255415.1	ENSG00000213839.4
17281	ENSG00000197020.11	ENSG00000255413.1	ENSG00000170190.16
17282	ENSG00000197021.9	ENSG00000255411.2	ENSG00000147065.17
17283	ENSG00000197023.6	ENSG00000255410.1	ENSG00000269001.2
17284	ENSG00000197024.8	ENSG00000255409.1	ENSG00000170425.3
17285	ENSG00000197037.11	ENSG00000255408.4	ENSG00000260947.1
17286	ENSG00000197038.6	ENSG00000255406.2	ENSG00000159210.9
17287	ENSG00000197043.14	ENSG00000255404.1	ENSG00000278195.2
17288	ENSG00000197044.11	ENSG00000255403.1	ENSG00000071894.17
17289	ENSG00000197045.13	ENSG00000255402.1	ENSG00000182841.12
17290	ENSG00000197046.11	ENSG00000255401.1	ENSG00000242551.2
17291	ENSG00000197050.11	ENSG00000255400.1	ENSG00000130222.11
17292	ENSG00000197054.11	ENSG00000255399.3	ENSG00000220201.7
17293	ENSG00000197056.11	ENSG00000255398.2	ENSG00000260286.3
17294	ENSG00000197057.9	ENSG00000255397.1	ENSG00000080007.8
17295	ENSG00000197061.4	ENSG00000255396.2	ENSG00000119397.16
17296	ENSG00000197062.11	ENSG00000255395.1	ENSG00000237624.1
17297	ENSG00000197063.11	ENSG00000255394.4	ENSG00000244642.3
17298	ENSG00000197067.6	ENSG00000255393.2	ENSG00000266920.1
17299	ENSG00000197070.14	ENSG00000255391.1	ENSG00000261771.5
17300	ENSG00000197077.13	ENSG00000255390.1	ENSG00000249360.1
17301	ENSG00000197079.8	ENSG00000255389.1	ENSG00000134153.10
17302	ENSG00000197081.13	ENSG00000255388.5	ENSG00000264322.2
17303	ENSG00000197083.11	ENSG00000255387.1	ENSG00000239839.7
17304	ENSG00000197084.5	ENSG00000255386.1	ENSG00000230175.1
17305	ENSG00000197085.11	ENSG00000255385.1	ENSG00000169641.13
17306	ENSG00000197092.5	ENSG00000255384.1	ENSG00000234145.1
17307	ENSG00000197093.11	ENSG00000255382.1	ENSG00000264659.1
17308	ENSG00000197099.8	ENSG00000255381.1	ENSG00000270441.1
17309	ENSG00000197102.12	ENSG00000255380.1	ENSG00000182022.18
17310	ENSG00000197106.7	ENSG00000255379.1	ENSG00000262962.1
17311	ENSG00000197110.8	ENSG00000255378.1	ENSG00000260017.1
17312	ENSG00000197111.15	ENSG00000255377.1	ENSG00000248475.5
17313	ENSG00000197114.11	ENSG00000255376.1	ENSG00000228150.1
17314	ENSG00000197119.13	ENSG00000255375.2	ENSG00000100056.12
17315	ENSG00000197121.15	ENSG00000255374.3	ENSG00000167552.14
17316	ENSG00000197122.11	ENSG00000255372.1	ENSG00000198056.14
17317	ENSG00000197123.9	ENSG00000255371.1	ENSG00000113739.10
17318	ENSG00000197124.12	ENSG00000255370.1	ENSG00000174106.3
17319	ENSG00000197125.3	ENSG00000255369.1	ENSG00000234518.2
17320	ENSG00000197128.11	ENSG00000255368.1	ENSG00000168309.18
17321	ENSG00000197134.12	ENSG00000255367.3	ENSG00000213849.3
17322	ENSG00000197136.4	ENSG00000255366.1	ENSG00000176153.12
17323	ENSG00000197140.15	ENSG00000255365.1	ENSG00000127530.3
17324	ENSG00000197142.10	ENSG00000255364.1	ENSG00000180530.11
17325	ENSG00000197147.13	ENSG00000255363.2	ENSG00000168263.9
17326	ENSG00000197149.5	ENSG00000255362.1	ENSG00000256660.6
17327	ENSG00000197150.12	ENSG00000255361.1	ENSG00000236297.1
17328	ENSG00000197153.4	ENSG00000255360.1	ENSG00000257621.7

17329	ENSG00000197157.11	ENSG00000255359.2	ENSG00000284116.1
17330	ENSG00000197161.7	ENSG00000255358.1	ENSG00000167468.16
17331	ENSG00000197162.9	ENSG00000255357.1	ENSG00000167895.14
17332	ENSG00000197165.11	ENSG00000255356.2	ENSG00000204802.3
17333	ENSG00000197168.13	ENSG00000255355.1	ENSG00000225770.1
17334	ENSG00000197170.10	ENSG00000255353.1	ENSG00000284240.1
17335	ENSG00000197171.6	ENSG00000255352.1	ENSG00000137101.12
17336	ENSG00000197172.10	ENSG00000255351.1	ENSG00000231861.2
17337	ENSG00000197176.7	ENSG00000255350.1	ENSG00000188396.3
17338	ENSG00000197177.15	ENSG00000255349.1	ENSG00000232680.2
17339	ENSG00000197180.2	ENSG00000255348.1	ENSG00000196156.4
17340	ENSG00000197181.12	ENSG00000255347.1	ENSG00000230686.1
17341	ENSG00000197182.14	ENSG00000255346.10	ENSG00000189067.12
17342	ENSG00000197183.14	ENSG00000255345.1	ENSG00000277449.1
17343	ENSG00000197185.7	ENSG00000255344.1	ENSG00000162148.11
17344	ENSG00000197191.5	ENSG00000255343.1	ENSG00000273597.1
17345	ENSG00000197208.6	ENSG00000255342.1	ENSG00000231711.2
17346	ENSG00000197210.7	ENSG00000255341.1	ENSG00000154803.13
17347	ENSG00000197213.9	ENSG00000255340.1	ENSG00000237990.3
17348	ENSG00000197214.5	ENSG00000255339.6	ENSG00000197927.12
17349	ENSG00000197217.13	ENSG00000255338.1	ENSG00000283701.1
17350	ENSG00000197223.11	ENSG00000255337.1	ENSG00000229425.2
17351	ENSG00000197226.12	ENSG00000255336.1	ENSG00000166780.11
17352	ENSG00000197233.7	ENSG00000255335.1	ENSG00000233041.8
17353	ENSG00000197238.4	ENSG00000255334.1	ENSG00000129538.14
17354	ENSG00000197241.3	ENSG00000255333.1	ENSG00000115159.16
17355	ENSG00000197245.6	ENSG00000255332.7	ENSG00000258405.10
17356	ENSG00000197249.13	ENSG00000255331.1	ENSG00000023902.14
17357	ENSG00000197251.3	ENSG00000255330.9	ENSG00000253433.1
17358	ENSG00000197253.13	ENSG00000255329.1	ENSG00000108798.9
17359	ENSG00000197254.4	ENSG00000255328.1	ENSG00000258527.1
17360	ENSG00000197256.10	ENSG00000255327.1	ENSG00000159648.11
17361	ENSG00000197258.5	ENSG00000255326.1	ENSG00000089048.14
17362	ENSG00000197261.11	ENSG00000255325.2	ENSG00000270110.1
17363	ENSG00000197265.9	ENSG00000255323.5	ENSG00000149809.14
17364	ENSG00000197272.2	ENSG00000255321.1	ENSG00000273155.1
17365	ENSG00000197273.4	ENSG00000255320.1	ENSG00000087586.17
17366	ENSG00000197275.14	ENSG00000255319.5	ENSG00000260641.1
17367	ENSG00000197279.4	ENSG00000255317.1	ENSG00000260868.1
17368	ENSG00000197283.16	ENSG00000255316.1	ENSG00000224152.1
17369	ENSG00000197291.8	ENSG00000255315.1	ENSG00000155506.17
17370	ENSG00000197296.6	ENSG00000255314.3	ENSG00000177674.16
17371	ENSG00000197299.12	ENSG00000255313.1	ENSG00000250992.1
17372	ENSG00000197301.7	ENSG00000255312.1	ENSG00000224672.4
17373	ENSG00000197302.10	ENSG00000255311.5	ENSG00000235308.1
17374	ENSG00000197308.9	ENSG00000255310.2	ENSG00000133818.14
17375	ENSG00000197309.3	ENSG00000255309.1	ENSG00000235128.1
17376	ENSG00000197312.12	ENSG00000255308.1	ENSG00000202019.1
17377	ENSG00000197320.5	ENSG00000255307.1	ENSG00000162614.18
17378	ENSG00000197321.14	ENSG00000255306.1	ENSG00000204936.10
17379	ENSG00000197322.3	ENSG00000255305.1	ENSG00000257642.1
17380	ENSG00000197323.12	ENSG00000255304.1	ENSG00000146856.14
17381	ENSG00000197324.9	ENSG00000255303.2	ENSG00000168040.4

17382	ENSG00000197329.12	ENSG00000255302.4	ENSG00000197976.12
17383	ENSG00000197332.8	ENSG00000255301.1	ENSG00000254206.5
17384	ENSG00000197334.2	ENSG00000255300.5	ENSG00000166024.13
17385	ENSG00000197343.11	ENSG00000255299.5	ENSG00000160183.15
17386	ENSG00000197345.13	ENSG00000255298.3	ENSG00000167077.13
17387	ENSG00000197353.3	ENSG00000255297.1	ENSG00000271889.1
17388	ENSG00000197355.11	ENSG00000255296.1	ENSG00000213612.3
17389	ENSG00000197358.9	ENSG00000255295.1	ENSG00000234558.1
17390	ENSG00000197360.9	ENSG00000255294.2	ENSG00000274744.1
17391	ENSG00000197361.7	ENSG00000255293.1	ENSG00000230212.6
17392	ENSG00000197362.14	ENSG00000255292.8	ENSG00000273064.1
17393	ENSG00000197363.9	ENSG00000255291.2	ENSG00000243539.3
17394	ENSG00000197364.7	ENSG00000255289.1	ENSG00000141562.18
17395	ENSG00000197372.9	ENSG00000255288.1	ENSG00000213361.2
17396	ENSG00000197375.12	ENSG00000255287.1	ENSG00000182782.7
17397	ENSG00000197376.3	ENSG00000255286.2	ENSG00000226478.3
17398	ENSG00000197380.11	ENSG00000255285.1	ENSG00000251455.1
17399	ENSG00000197381.16	ENSG00000255284.1	ENSG00000273219.1
17400	ENSG00000197385.5	ENSG00000255283.3	ENSG00000155070.8
17401	ENSG00000197386.12	ENSG00000255282.6	ENSG00000214193.10
17402	ENSG00000197403.4	ENSG00000255281.1	ENSG00000053918.16
17403	ENSG00000197405.8	ENSG00000255280.1	ENSG00000205794.4
17404	ENSG00000197406.7	ENSG00000255279.1	ENSG00000185950.9
17405	ENSG00000197408.10	ENSG00000255277.3	ENSG00000257790.1
17406	ENSG00000197409.7	ENSG00000255276.1	ENSG00000112715.21
17407	ENSG00000197410.13	ENSG00000255275.3	ENSG00000069667.16
17408	ENSG00000197415.12	ENSG00000255274.9	ENSG00000096092.6
17409	ENSG00000197416.4	ENSG00000255273.1	ENSG00000225703.1
17410	ENSG00000197417.7	ENSG00000255272.1	ENSG00000258168.5
17411	ENSG00000197421.9	ENSG00000255271.1	ENSG00000231551.8
17412	ENSG00000197428.3	ENSG00000255270.1	ENSG00000123096.11
17413	ENSG00000197429.10	ENSG00000255269.2	ENSG00000285994.1
17414	ENSG00000197430.11	ENSG00000255268.2	ENSG00000236388.1
17415	ENSG00000197437.4	ENSG00000255267.3	ENSG00000163362.11
17416	ENSG00000197442.10	ENSG00000255266.1	ENSG00000125207.7
17417	ENSG00000197444.10	ENSG00000255265.1	ENSG00000205143.2
17418	ENSG00000197446.9	ENSG00000255262.3	ENSG00000265206.5
17419	ENSG00000197448.14	ENSG00000255261.1	ENSG00000213875.3
17420	ENSG00000197451.12	ENSG00000255260.1	ENSG00000229759.1
17421	ENSG00000197454.2	ENSG00000255259.2	ENSG00000171236.10
17422	ENSG00000197457.10	ENSG00000255258.2	ENSG00000173966.7
17423	ENSG00000197461.13	ENSG00000255256.1	ENSG00000261840.2
17424	ENSG00000197462.3	ENSG00000255255.1	ENSG00000285933.1
17425	ENSG00000197465.14	ENSG00000255254.1	ENSG00000011478.12
17426	ENSG00000197467.14	ENSG00000255253.1	ENSG00000164342.12
17427	ENSG00000197468.5	ENSG00000255252.3	ENSG00000116584.18
17428	ENSG00000197471.12	ENSG00000255251.1	ENSG00000184182.18
17429	ENSG00000197472.15	ENSG00000255250.1	ENSG00000130695.15
17430	ENSG00000197475.11	ENSG00000255248.8	ENSG00000130803.15
17431	ENSG00000197476.3	ENSG00000255247.1	ENSG00000179165.11
17432	ENSG00000197479.6	ENSG00000255246.1	ENSG00000183615.6
17433	ENSG00000197483.10	ENSG00000255245.4	ENSG00000232818.2
17434	ENSG00000197487.8	ENSG00000255244.1	ENSG00000268581.1

17435	ENSG00000197496.6	ENSG00000255243.1	ENSG00000013563.14
17436	ENSG00000197497.11	ENSG00000255241.1	ENSG00000133398.4
17437	ENSG00000197498.13	ENSG00000255240.5	ENSG00000253716.5
17438	ENSG00000197503.4	ENSG00000255239.2	ENSG00000257074.3
17439	ENSG00000197506.7	ENSG00000255238.1	ENSG00000168214.20
17440	ENSG00000197520.10	ENSG00000255236.2	ENSG00000267570.1
17441	ENSG00000197530.12	ENSG00000255235.1	ENSG00000073712.15
17442	ENSG00000197532.2	ENSG00000255234.5	ENSG00000268743.1
17443	ENSG00000197535.14	ENSG00000255233.1	ENSG00000146859.6
17444	ENSG00000197536.11	ENSG00000255232.1	ENSG00000274367.1
17445	ENSG00000197540.8	ENSG00000255231.1	ENSG00000148180.19
17446	ENSG00000197548.12	ENSG00000255230.1	ENSG00000279894.1
17447	ENSG00000197549.9	ENSG00000255229.1	ENSG00000228283.3
17448	ENSG00000197550.3	ENSG00000255227.1	ENSG00000236545.1
17449	ENSG00000197555.9	ENSG00000255226.1	ENSG00000267448.5
17450	ENSG00000197557.7	ENSG00000255225.1	ENSG00000197982.14
17451	ENSG00000197558.11	ENSG00000255224.1	ENSG00000168491.10
17452	ENSG00000197561.7	ENSG00000255223.4	ENSG00000267214.1
17453	ENSG00000197562.10	ENSG00000255222.1	ENSG00000167083.7
17454	ENSG00000197563.11	ENSG00000255221.3	ENSG00000200113.1
17455	ENSG00000197565.15	ENSG00000255220.1	ENSG00000254028.1
17456	ENSG00000197566.10	ENSG00000255219.1	ENSG00000271207.1
17457	ENSG00000197568.14	ENSG00000255218.1	ENSG00000222046.2
17458	ENSG00000197575.6	ENSG00000255217.1	ENSG00000231615.2
17459	ENSG00000197576.13	ENSG00000255216.1	ENSG00000230649.2
17460	ENSG00000197579.8	ENSG00000255215.1	ENSG00000279416.1
17461	ENSG00000197580.11	ENSG00000255214.1	ENSG00000233952.1
17462	ENSG00000197582.5	ENSG00000255213.1	ENSG00000211714.3
17463	ENSG00000197584.12	ENSG00000255211.1	ENSG00000233338.1
17464	ENSG00000197585.9	ENSG00000255210.1	ENSG00000259171.1
17465	ENSG00000197586.12	ENSG00000255209.1	ENSG00000187753.13
17466	ENSG00000197587.10	ENSG00000255207.1	ENSG00000198211.8
17467	ENSG00000197588.9	ENSG00000255206.1	ENSG00000112195.9
17468	ENSG00000197591.3	ENSG00000255205.1	ENSG00000248746.6
17469	ENSG00000197594.13	ENSG00000255204.1	ENSG00000252950.1
17470	ENSG00000197595.4	ENSG00000255203.1	ENSG00000100532.12
17471	ENSG00000197599.12	ENSG00000255202.1	ENSG00000258872.2
17472	ENSG00000197601.13	ENSG00000255201.1	ENSG00000232878.3
17473	ENSG00000197603.14	ENSG00000255200.1	ENSG00000239830.1
17474	ENSG00000197608.11	ENSG00000255199.2	ENSG00000267115.1
17475	ENSG00000197614.11	ENSG00000255198.4	ENSG00000249309.1
17476	ENSG00000197616.12	ENSG00000255197.5	ENSG00000080802.18
17477	ENSG00000197617.8	ENSG00000255196.1	ENSG00000186462.8
17478	ENSG00000197619.14	ENSG00000255193.1	ENSG00000224618.1
17479	ENSG00000197620.11	ENSG00000255192.5	ENSG00000148153.14
17480	ENSG00000197622.13	ENSG00000255191.1	ENSG00000270781.1
17481	ENSG00000197627.3	ENSG00000255190.2	ENSG00000100364.18
17482	ENSG00000197629.5	ENSG00000255189.1	ENSG00000286177.1
17483	ENSG00000197632.9	ENSG00000255188.1	ENSG00000085185.15
17484	ENSG00000197635.10	ENSG00000255187.1	ENSG00000104808.8
17485	ENSG00000197641.11	ENSG00000255186.1	ENSG00000138640.15
17486	ENSG00000197644.2	ENSG00000255185.5	ENSG00000151466.12
17487	ENSG00000197646.7	ENSG00000255184.1	ENSG00000164099.3

17488	ENSG00000197647.11	ENSG00000255183.1	ENSG00000258973.1
17489	ENSG00000197651.4	ENSG00000255182.2	ENSG00000250069.1
17490	ENSG00000197653.15	ENSG00000255181.3	ENSG00000227586.5
17491	ENSG00000197658.9	ENSG00000255179.1	ENSG00000273038.2
17492	ENSG00000197665.7	ENSG00000255178.1	ENSG00000203666.12
17493	ENSG00000197670.6	ENSG00000255177.2	ENSG00000279174.1
17494	ENSG00000197674.7	ENSG00000255176.1	ENSG00000242876.3
17495	ENSG00000197683.4	ENSG00000255175.5	ENSG00000163263.7
17496	ENSG00000197692.5	ENSG00000255174.1	ENSG00000173727.12
17497	ENSG00000197694.15	ENSG00000255173.1	ENSG00000271384.1
17498	ENSG00000197696.10	ENSG00000255172.2	ENSG00000125910.5
17499	ENSG00000197702.13	ENSG00000255171.6	ENSG00000111186.13
17500	ENSG00000197705.9	ENSG00000255170.2	ENSG00000165863.17
17501	ENSG00000197706.3	ENSG00000255169.1	ENSG00000108825.17
17502	ENSG00000197712.12	ENSG00000255167.1	ENSG00000196218.12
17503	ENSG00000197713.15	ENSG00000255166.1	ENSG00000270035.1
17504	ENSG00000197714.9	ENSG00000255165.1	ENSG00000247400.3
17505	ENSG00000197721.16	ENSG00000255164.1	ENSG00000255142.1
17506	ENSG00000197724.11	ENSG00000255163.1	ENSG00000268655.2
17507	ENSG00000197728.11	ENSG00000255162.1	ENSG00000155903.11
17508	ENSG00000197734.9	ENSG00000255161.1	ENSG00000275362.1
17509	ENSG00000197744.5	ENSG00000255160.5	ENSG00000245598.6
17510	ENSG00000197745.2	ENSG00000255159.1	ENSG00000184005.11
17511	ENSG00000197746.14	ENSG00000255158.1	ENSG00000140259.7
17512	ENSG00000197747.9	ENSG00000255157.1	ENSG00000074527.12
17513	ENSG00000197748.12	ENSG00000255156.1	ENSG00000257376.1
17514	ENSG00000197753.10	ENSG00000255155.1	ENSG00000169612.4
17515	ENSG00000197756.9	ENSG00000255154.7	ENSG00000232772.1
17516	ENSG00000197757.8	ENSG00000255153.1	ENSG00000047249.18
17517	ENSG00000197763.16	ENSG00000255152.8	ENSG00000229539.1
17518	ENSG00000197766.7	ENSG00000255151.2	ENSG00000091592.16
17519	ENSG00000197768.10	ENSG00000255150.2	ENSG00000120555.13
17520	ENSG00000197769.5	ENSG00000255149.1	ENSG00000158486.13
17521	ENSG00000197771.12	ENSG00000255148.2	ENSG00000163909.8
17522	ENSG00000197774.12	ENSG00000255147.1	ENSG00000259056.1
17523	ENSG00000197776.8	ENSG00000255146.1	ENSG00000067836.12
17524	ENSG00000197779.13	ENSG00000255145.2	ENSG00000141526.16
17525	ENSG00000197780.9	ENSG00000255144.1	ENSG00000182774.12
17526	ENSG00000197782.14	ENSG00000255143.1	ENSG00000223500.1
17527	ENSG00000197785.13	ENSG00000255142.1	ENSG00000137145.20
17528	ENSG00000197786.3	ENSG00000255141.1	ENSG00000285967.1
17529	ENSG00000197790.1	ENSG00000255140.1	ENSG00000284378.1
17530	ENSG00000197794.2	ENSG00000255139.1	ENSG00000162836.12
17531	ENSG00000197798.9	ENSG00000255138.1	ENSG00000241666.2
17532	ENSG00000197808.12	ENSG00000255136.2	ENSG00000110200.8
17533	ENSG00000197813.5	ENSG00000255135.3	ENSG00000100625.9
17534	ENSG00000197815.4	ENSG00000255134.1	ENSG00000163053.11
17535	ENSG00000197816.13	ENSG00000255133.1	ENSG00000236914.3
17536	ENSG00000197818.11	ENSG00000255132.1	ENSG00000223459.6
17537	ENSG00000197822.11	ENSG00000255131.2	ENSG00000108255.7
17538	ENSG00000197826.11	ENSG00000255130.1	ENSG00000231162.4
17539	ENSG00000197837.3	ENSG00000255129.5	ENSG00000234722.4
17540	ENSG00000197838.4	ENSG00000255128.1	ENSG00000263179.1

17541	ENSG00000197841.15	ENSG00000255127.3	ENSG00000272836.1
17542	ENSG00000197847.12	ENSG00000255126.1	ENSG00000071575.11
17543	ENSG00000197849.6	ENSG00000255123.1	ENSG00000023608.5
17544	ENSG00000197852.11	ENSG00000255122.1	ENSG00000204611.7
17545	ENSG00000197857.13	ENSG00000255121.2	ENSG00000180914.10
17546	ENSG00000197858.11	ENSG00000255120.5	ENSG00000241772.2
17547	ENSG00000197859.10	ENSG00000255119.1	ENSG00000273888.1
17548	ENSG00000197860.10	ENSG00000255118.1	ENSG00000258199.1
17549	ENSG00000197863.9	ENSG00000255117.5	ENSG00000242314.1
17550	ENSG00000197866.3	ENSG00000255116.2	ENSG00000228702.1
17551	ENSG00000197870.12	ENSG00000255115.2	ENSG00000265272.2
17552	ENSG00000197872.11	ENSG00000255114.1	ENSG00000240015.2
17553	ENSG00000197879.16	ENSG00000255113.1	ENSG00000077984.6
17554	ENSG00000197880.8	ENSG00000255112.2	ENSG00000054983.17
17555	ENSG00000197882.3	ENSG00000255111.1	ENSG00000230733.2
17556	ENSG00000197885.10	ENSG00000255110.1	ENSG00000225200.2
17557	ENSG00000197887.4	ENSG00000255109.5	ENSG00000199683.1
17558	ENSG00000197888.2	ENSG00000255108.1	ENSG00000228523.1
17559	ENSG00000197889.10	ENSG00000255107.1	ENSG00000278746.1
17560	ENSG00000197891.12	ENSG00000255106.1	ENSG00000174015.10
17561	ENSG00000197892.13	ENSG00000255105.1	ENSG00000257599.2
17562	ENSG00000197893.13	ENSG00000255104.8	ENSG00000100628.11
17563	ENSG00000197894.11	ENSG00000255102.1	ENSG00000140538.16
17564	ENSG00000197901.11	ENSG00000255101.1	ENSG00000216657.1
17565	ENSG00000197903.7	ENSG00000255100.1	ENSG00000236481.1
17566	ENSG00000197905.9	ENSG00000255099.1	ENSG00000180901.11
17567	ENSG00000197912.15	ENSG00000255097.1	ENSG00000277856.1
17568	ENSG00000197915.6	ENSG00000255096.2	ENSG00000011201.12
17569	ENSG00000197919.5	ENSG00000255095.2	ENSG00000256043.3
17570	ENSG00000197921.6	ENSG00000255094.1	ENSG00000239569.3
17571	ENSG00000197927.12	ENSG00000255093.2	ENSG00000214145.6
17572	ENSG00000197928.10	ENSG00000255092.1	ENSG00000259762.1
17573	ENSG00000197930.13	ENSG00000255091.1	ENSG00000279964.1
17574	ENSG00000197933.13	ENSG00000255089.1	ENSG00000213250.5
17575	ENSG00000197934.8	ENSG00000255088.1	ENSG00000069275.13
17576	ENSG00000197935.6	ENSG00000255087.3	ENSG00000226825.1
17577	ENSG00000197937.12	ENSG00000255086.1	ENSG00000234297.2
17578	ENSG00000197938.5	ENSG00000255085.8	ENSG00000214869.5
17579	ENSG00000197943.10	ENSG00000255084.1	ENSG00000229436.1
17580	ENSG00000197948.11	ENSG00000255083.2	ENSG00000203697.11
17581	ENSG00000197951.9	ENSG00000255082.1	ENSG00000196704.12
17582	ENSG00000197953.5	ENSG00000255081.1	ENSG00000259161.2
17583	ENSG00000197956.10	ENSG00000255079.1	ENSG00000274629.1
17584	ENSG00000197958.13	ENSG00000255078.1	ENSG00000186714.12
17585	ENSG00000197959.14	ENSG00000255077.1	ENSG00000144455.14
17586	ENSG00000197961.11	ENSG00000255076.2	ENSG00000139496.16
17587	ENSG00000197965.12	ENSG00000255075.1	ENSG00000122042.9
17588	ENSG00000197969.13	ENSG00000255074.1	ENSG00000276953.1
17589	ENSG00000197971.15	ENSG00000255073.8	ENSG00000163811.12
17590	ENSG00000197976.12	ENSG00000255072.1	ENSG00000211783.3
17591	ENSG00000197976.12_PAR_Y	ENSG00000255071.3	ENSG00000120685.20
17592	ENSG00000197977.4	ENSG00000255070.1	ENSG00000265992.1
17593	ENSG00000197978.9	ENSG00000255067.1	ENSG00000176495.3

17594	ENSG00000197980.12	ENSG00000255065.1	ENSG00000254548.1
17595	ENSG00000197982.14	ENSG00000255063.1	ENSG00000114646.10
17596	ENSG00000197984.4	ENSG00000255062.1	ENSG00000170802.16
17597	ENSG00000197989.14	ENSG00000255060.1	ENSG00000232931.5
17598	ENSG00000197990.6	ENSG00000255059.1	ENSG00000232634.1
17599	ENSG00000197991.11	ENSG00000255058.1	ENSG00000284713.1
17600	ENSG00000197992.7	ENSG00000255057.1	ENSG00000166046.11
17601	ENSG00000197993.9	ENSG00000255055.1	ENSG00000164610.9
17602	ENSG00000198000.12	ENSG00000255054.3	ENSG00000266978.1
17603	ENSG00000198001.13	ENSG00000255053.1	ENSG00000254634.4
17604	ENSG00000198003.12	ENSG00000255052.4	ENSG00000044012.4
17605	ENSG00000198010.12	ENSG00000255051.1	ENSG00000198908.12
17606	ENSG00000198015.13	ENSG00000255050.1	ENSG00000162630.5
17607	ENSG00000198018.7	ENSG00000255048.1	ENSG00000213598.4
17608	ENSG00000198019.12	ENSG00000255047.1	ENSG00000275496.4
17609	ENSG00000198021.7	ENSG00000255046.1	ENSG00000176136.6
17610	ENSG00000198022.6	ENSG00000255045.1	ENSG00000078140.14
17611	ENSG00000198026.8	ENSG00000255043.1	ENSG00000137817.17
17612	ENSG00000198028.3	ENSG00000255042.3	ENSG00000188690.14
17613	ENSG00000198033.12	ENSG00000255041.1	ENSG00000204622.11
17614	ENSG00000198034.11	ENSG00000255040.1	ENSG00000100968.14
17615	ENSG00000198039.11	ENSG00000255039.1	ENSG00000196433.13
17616	ENSG00000198040.10	ENSG00000255038.1	ENSG00000205659.10
17617	ENSG00000198042.11	ENSG00000255037.1	ENSG00000262810.1
17618	ENSG00000198046.12	ENSG00000255036.6	ENSG00000236165.1
17619	ENSG00000198049.7	ENSG00000255035.2	ENSG00000278239.1
17620	ENSG00000198053.11	ENSG00000255033.1	ENSG00000225333.6
17621	ENSG00000198054.12	ENSG00000255032.1	ENSG00000181741.7
17622	ENSG00000198055.11	ENSG00000255031.5	ENSG00000227467.3
17623	ENSG00000198056.14	ENSG00000255030.1	ENSG00000198265.12
17624	ENSG00000198060.10	ENSG00000255029.1	ENSG00000279200.1
17625	ENSG00000198062.14	ENSG00000255028.5	ENSG00000224008.1
17626	ENSG00000198064.13	ENSG00000255027.2	ENSG00000095397.14
17627	ENSG00000198074.10	ENSG00000255026.1	ENSG00000268649.5
17628	ENSG00000198075.10	ENSG00000255025.1	ENSG00000171206.14
17629	ENSG00000198077.10	ENSG00000255022.1	ENSG00000283393.1
17630	ENSG00000198081.11	ENSG00000255021.1	ENSG00000280693.2
17631	ENSG00000198083.9	ENSG00000255020.1	ENSG00000268499.1
17632	ENSG00000198087.7	ENSG00000255019.1	ENSG00000267288.2
17633	ENSG00000198088.10	ENSG00000255018.1	ENSG00000132016.11
17634	ENSG00000198089.15	ENSG00000255016.1	ENSG00000207205.1
17635	ENSG00000198090.3	ENSG00000255015.1	ENSG00000235897.1
17636	ENSG00000198092.5	ENSG00000255014.1	ENSG00000215271.7
17637	ENSG00000198093.11	ENSG00000255012.2	ENSG00000275180.1
17638	ENSG00000198099.8	ENSG00000255011.1	ENSG00000226243.1
17639	ENSG00000198104.3	ENSG00000255010.1	ENSG00000210049.1
17640	ENSG00000198105.14	ENSG00000255009.4	ENSG00000235018.1
17641	ENSG00000198106.8	ENSG00000255008.2	ENSG00000079257.8
17642	ENSG00000198108.4	ENSG00000255007.1	ENSG00000216921.8
17643	ENSG00000198113.3	ENSG00000255006.1	ENSG00000220925.2
17644	ENSG00000198121.13	ENSG00000255005.1	ENSG00000254275.6
17645	ENSG00000198125.13	ENSG00000255004.1	ENSG00000101278.6
17646	ENSG00000198128.4	ENSG00000255003.1	ENSG00000276968.1

17647	ENSG00000198129.2	ENSG00000255002.1	ENSG00000225210.10
17648	ENSG00000198130.16	ENSG00000255001.1	ENSG00000225678.2
17649	ENSG00000198131.14	ENSG00000255000.1	ENSG00000181481.14
17650	ENSG00000198133.8	ENSG00000254999.4	ENSG00000086544.3
17651	ENSG00000198134.3	ENSG00000254997.3	ENSG00000235119.1
17652	ENSG00000198142.5	ENSG00000254996.5	ENSG00000233739.1
17653	ENSG00000198146.4	ENSG00000254995.4	ENSG00000269604.1
17654	ENSG00000198153.8	ENSG00000254993.2	ENSG00000188822.8
17655	ENSG00000198155.5	ENSG00000254992.1	ENSG00000242412.1
17656	ENSG00000198156.10	ENSG00000254991.1	ENSG00000177669.3
17657	ENSG00000198157.10	ENSG00000254990.5	ENSG00000229534.1
17658	ENSG00000198160.14	ENSG00000254989.1	ENSG00000196378.11
17659	ENSG00000198162.12	ENSG00000254988.1	ENSG00000157240.3
17660	ENSG00000198168.9	ENSG00000254987.1	ENSG00000053328.8
17661	ENSG00000198169.9	ENSG00000254986.7	ENSG00000227189.2
17662	ENSG00000198171.13	ENSG00000254985.1	ENSG00000230319.1
17663	ENSG00000198173.4	ENSG00000254984.1	ENSG00000226435.10
17664	ENSG00000198176.13	ENSG00000254983.1	ENSG00000275966.1
17665	ENSG00000198178.10	ENSG00000254980.1	ENSG00000249086.1
17666	ENSG00000198182.13	ENSG00000254979.5	ENSG00000159842.15
17667	ENSG00000198183.12	ENSG00000254978.2	ENSG00000181350.11
17668	ENSG00000198185.11	ENSG00000254976.1	ENSG00000013810.19
17669	ENSG00000198189.11	ENSG00000254975.1	ENSG00000267119.1
17670	ENSG00000198198.16	ENSG00000254974.1	ENSG00000230626.4
17671	ENSG00000198203.10	ENSG00000254973.1	ENSG00000229311.1
17672	ENSG00000198205.6	ENSG00000254972.1	ENSG00000146839.18
17673	ENSG00000198208.11	ENSG00000254971.1	ENSG00000242766.1
17674	ENSG00000198211.8	ENSG00000254968.6	ENSG00000148290.10
17675	ENSG00000198216.12	ENSG00000254966.1	ENSG00000140284.11
17676	ENSG00000198217.4	ENSG00000254965.1	ENSG00000261208.1
17677	ENSG00000198218.11	ENSG00000254964.1	ENSG00000144579.7
17678	ENSG00000198221.8	ENSG00000254963.1	ENSG00000236377.1
17679	ENSG00000198223.16	ENSG00000254961.1	ENSG00000115085.13
17680	ENSG00000198223.16_PAR_Y	ENSG00000254960.2	ENSG00000234193.1
17681	ENSG00000198225.5	ENSG00000254959.6	ENSG00000258424.1
17682	ENSG00000198231.13	ENSG00000254957.1	ENSG00000275445.1
17683	ENSG00000198237.8	ENSG00000254954.1	ENSG00000249245.2
17684	ENSG00000198242.14	ENSG00000254953.1	ENSG00000257875.1
17685	ENSG00000198246.9	ENSG00000254952.1	ENSG00000198929.13
17686	ENSG00000198251.7	ENSG00000254951.7	ENSG00000267457.1
17687	ENSG00000198252.12	ENSG00000254949.1	ENSG00000286020.1
17688	ENSG00000198258.10	ENSG00000254948.1	ENSG00000280417.1
17689	ENSG00000198261.3	ENSG00000254947.1	ENSG00000206834.1
17690	ENSG00000198265.12	ENSG00000254946.1	ENSG00000152672.8
17691	ENSG00000198270.13	ENSG00000254944.1	ENSG00000271605.6
17692	ENSG00000198271.4	ENSG00000254943.1	ENSG00000213304.3
17693	ENSG00000198276.16	ENSG00000254942.1	ENSG00000259419.2
17694	ENSG00000198277.6	ENSG00000254941.1	ENSG00000283792.1
17695	ENSG00000198283.2	ENSG00000254940.1	ENSG00000163833.8
17696	ENSG00000198284.9	ENSG00000254939.1	ENSG00000249855.1
17697	ENSG00000198286.9	ENSG00000254938.1	ENSG00000278966.2
17698	ENSG00000198298.13	ENSG00000254937.1	ENSG00000121742.18
17699	ENSG00000198300.14	ENSG00000254936.4	ENSG00000219545.11

17700	ENSG00000198301.12	ENSG00000254935.1	ENSG00000245552.6
17701	ENSG00000198312.4	ENSG00000254934.5	ENSG00000115155.17
17702	ENSG00000198315.11	ENSG00000254933.1	ENSG00000267149.2
17703	ENSG00000198324.13	ENSG00000254932.2	ENSG00000268530.5
17704	ENSG00000198326.9	ENSG00000254931.2	ENSG00000101236.17
17705	ENSG00000198331.10	ENSG00000254930.1	ENSG00000205755.11
17706	ENSG00000198336.9	ENSG00000254929.6	ENSG00000226239.1
17707	ENSG00000198342.10	ENSG00000254928.1	ENSG00000119715.15
17708	ENSG00000198346.11	ENSG00000254927.1	ENSG00000257267.3
17709	ENSG00000198353.8	ENSG00000254926.1	ENSG00000225992.1
17710	ENSG00000198354.7	ENSG00000254925.1	ENSG00000262052.1
17711	ENSG00000198355.5	ENSG00000254924.2	ENSG00000211710.3
17712	ENSG00000198356.11	ENSG00000254923.1	ENSG00000247627.2
17713	ENSG00000198358.4	ENSG00000254921.1	ENSG00000279141.3
17714	ENSG00000198363.18	ENSG00000254920.1	ENSG00000231473.2
17715	ENSG00000198367.3	ENSG00000254919.1	ENSG00000168334.9
17716	ENSG00000198369.10	ENSG00000254917.1	ENSG00000261801.5
17717	ENSG00000198373.12	ENSG00000254916.1	ENSG00000214944.9
17718	ENSG00000198380.12	ENSG00000254915.1	ENSG00000226963.1
17719	ENSG00000198382.9	ENSG00000254914.1	ENSG00000254319.5
17720	ENSG00000198390.4	ENSG00000254913.1	ENSG00000088832.17
17721	ENSG00000198393.8	ENSG00000254912.2	ENSG00000136694.8
17722	ENSG00000198398.2	ENSG00000254911.3	ENSG00000278301.1
17723	ENSG00000198399.14	ENSG00000254910.1	ENSG00000223916.1
17724	ENSG00000198400.11	ENSG00000254909.1	ENSG00000187715.13
17725	ENSG00000198406.7	ENSG00000254907.1	ENSG00000220804.8
17726	ENSG00000198408.14	ENSG00000254906.1	ENSG00000140416.21
17727	ENSG00000198414.5	ENSG00000254905.1	ENSG00000285016.1
17728	ENSG00000198416.9	ENSG00000254903.1	ENSG00000286245.1
17729	ENSG00000198417.7	ENSG00000254902.1	ENSG00000138735.16
17730	ENSG00000198420.10	ENSG00000254901.8	ENSG00000168255.20
17731	ENSG00000198429.9	ENSG00000254900.1	ENSG00000162755.14
17732	ENSG00000198431.16	ENSG00000254898.1	ENSG00000188993.3
17733	ENSG00000198435.4	ENSG00000254897.1	ENSG00000283765.1
17734	ENSG00000198440.9	ENSG00000254896.1	ENSG00000130592.15
17735	ENSG00000198443.6	ENSG00000254895.2	ENSG00000272472.1
17736	ENSG00000198445.4	ENSG00000254894.1	ENSG00000251624.1
17737	ENSG00000198452.7	ENSG00000254893.6	ENSG00000167613.16
17738	ENSG00000198453.13	ENSG00000254892.1	ENSG00000182489.9
17739	ENSG00000198454.2	ENSG00000254891.1	ENSG00000215845.11
17740	ENSG00000198455.4	ENSG00000254890.1	ENSG00000283399.1
17741	ENSG00000198464.14	ENSG00000254889.1	ENSG00000231346.5
17742	ENSG00000198466.12	ENSG00000254888.2	ENSG00000261305.2
17743	ENSG00000198467.14	ENSG00000254887.1	ENSG00000277797.1
17744	ENSG00000198468.8	ENSG00000254885.1	ENSG00000280295.1
17745	ENSG00000198471.1	ENSG00000254884.1	ENSG00000186230.7
17746	ENSG00000198478.8	ENSG00000254883.1	ENSG00000230337.1
17747	ENSG00000198482.12	ENSG00000254880.1	ENSG00000268034.1
17748	ENSG00000198483.13	ENSG00000254879.1	ENSG00000131323.14
17749	ENSG00000198488.10	ENSG00000254878.1	ENSG00000271590.1
17750	ENSG00000198491.3	ENSG00000254877.1	ENSG00000273387.1
17751	ENSG00000198492.16	ENSG00000254876.5	ENSG00000187229.3
17752	ENSG00000198496.11	ENSG00000254874.1	ENSG00000244515.1

17753	ENSG00000198498.10	ENSG00000254873.1	ENSG00000263327.6
17754	ENSG00000198502.6	ENSG00000254872.3	ENSG00000181963.5
17755	ENSG00000198513.11	ENSG00000254871.1	ENSG00000254901.8
17756	ENSG00000198515.13	ENSG00000254870.5	ENSG00000127603.25
17757	ENSG00000198517.10	ENSG00000254867.1	ENSG00000254810.1
17758	ENSG00000198520.12	ENSG00000254866.2	ENSG00000230701.2
17759	ENSG00000198521.11	ENSG00000254865.1	ENSG00000246792.2
17760	ENSG00000198522.13	ENSG00000254864.1	ENSG00000272482.1
17761	ENSG00000198523.6	ENSG00000254863.1	ENSG00000010219.13
17762	ENSG00000198526.7	ENSG00000254862.5	ENSG00000076864.19
17763	ENSG00000198535.5	ENSG00000254861.1	ENSG00000255967.1
17764	ENSG00000198538.11	ENSG00000254860.5	ENSG00000188004.10
17765	ENSG00000198542.14	ENSG00000254859.1	ENSG00000068305.17
17766	ENSG00000198546.15	ENSG00000254858.9	ENSG00000241981.2
17767	ENSG00000198547.8	ENSG00000254857.1	ENSG00000284368.1
17768	ENSG00000198551.10	ENSG00000254856.1	ENSG00000118004.17
17769	ENSG00000198553.9	ENSG00000254855.1	ENSG00000279288.1
17770	ENSG00000198554.12	ENSG00000254854.1	ENSG00000270640.1
17771	ENSG00000198555.7	ENSG00000254853.1	ENSG00000244425.3
17772	ENSG00000198556.14	ENSG00000254852.8	ENSG00000272758.5
17773	ENSG00000198561.13	ENSG00000254851.1	ENSG00000145777.15
17774	ENSG00000198563.14	ENSG00000254850.2	ENSG00000249175.1
17775	ENSG00000198569.9	ENSG00000254848.1	ENSG00000134571.10
17776	ENSG00000198570.5	ENSG00000254847.1	ENSG00000143036.17
17777	ENSG00000198573.6	ENSG00000254846.1	ENSG00000273958.1
17778	ENSG00000198574.6	ENSG00000254844.4	ENSG00000009950.16
17779	ENSG00000198576.4	ENSG00000254843.1	ENSG00000231579.3
17780	ENSG00000198580.7	ENSG00000254842.6	ENSG00000235963.1
17781	ENSG00000198585.11	ENSG00000254841.1	ENSG00000239322.1
17782	ENSG00000198586.14	ENSG00000254840.1	ENSG00000138430.16
17783	ENSG00000198589.12	ENSG00000254839.1	ENSG00000226647.2
17784	ENSG00000198590.11	ENSG00000254838.5	ENSG00000229299.2
17785	ENSG00000198597.9	ENSG00000254837.2	ENSG00000255153.1
17786	ENSG00000198598.6	ENSG00000254836.1	ENSG00000213275.2
17787	ENSG00000198601.3	ENSG00000254835.1	ENSG00000137812.19
17788	ENSG00000198604.11	ENSG00000254834.4	ENSG00000197122.11
17789	ENSG00000198610.11	ENSG00000254833.1	ENSG00000243675.1
17790	ENSG00000198612.11	ENSG00000254832.1	ENSG00000183171.5
17791	ENSG00000198618.5	ENSG00000254831.1	ENSG00000257740.1
17792	ENSG00000198624.13	ENSG00000254830.1	ENSG00000220248.1
17793	ENSG00000198625.13	ENSG00000254829.1	ENSG00000166068.13
17794	ENSG00000198626.16	ENSG00000254828.1	ENSG00000136869.15
17795	ENSG00000198633.10	ENSG00000254827.5	ENSG00000123485.12
17796	ENSG00000198642.6	ENSG00000254826.1	ENSG00000166582.10
17797	ENSG00000198643.7	ENSG00000254825.1	ENSG00000203730.2
17798	ENSG00000198646.14	ENSG00000254824.1	ENSG00000250132.6
17799	ENSG00000198648.11	ENSG00000254823.1	ENSG00000285703.1
17800	ENSG00000198650.11	ENSG00000254822.1	ENSG00000142910.16
17801	ENSG00000198658.4	ENSG00000254821.1	ENSG00000251136.8
17802	ENSG00000198663.16	ENSG00000254820.1	ENSG00000258111.1
17803	ENSG00000198668.11	ENSG00000254819.1	ENSG00000109072.14
17804	ENSG00000198670.11	ENSG00000254818.1	ENSG00000197409.7
17805	ENSG00000198671.3	ENSG00000254817.1	ENSG00000125144.13

17806	ENSG00000198673.10	ENSG00000254816.1	ENSG00000226427.1
17807	ENSG00000198674.3	ENSG00000254815.5	ENSG00000282996.1
17808	ENSG00000198677.11	ENSG00000254813.5	ENSG00000173391.9
17809	ENSG00000198678.5	ENSG00000254812.1	ENSG00000281831.1
17810	ENSG00000198680.4	ENSG00000254811.5	ENSG00000187010.21
17811	ENSG00000198681.7	ENSG00000254807.1	ENSG00000269392.1
17812	ENSG00000198682.13	ENSG00000254806.5	ENSG00000261758.1
17813	ENSG00000198685.3	ENSG00000254805.1	ENSG00000154620.6
17814	ENSG00000198689.11	ENSG00000254804.1	ENSG00000224885.1
17815	ENSG00000198690.9	ENSG00000254803.1	ENSG00000175806.15
17816	ENSG00000198691.13	ENSG00000254802.1	ENSG00000163793.12
17817	ENSG00000198692.10	ENSG00000254801.1	ENSG00000119723.16
17818	ENSG00000198695.2	ENSG00000254800.2	ENSG00000285035.1
17819	ENSG00000198700.10	ENSG00000254799.1	ENSG00000152894.14
17820	ENSG00000198703.2	ENSG00000254798.1	ENSG00000272968.5
17821	ENSG00000198704.9	ENSG00000254796.1	ENSG00000145687.16
17822	ENSG00000198707.15	ENSG00000254795.1	ENSG00000258881.6
17823	ENSG00000198711.5	ENSG00000254794.1	ENSG00000257580.1
17824	ENSG00000198712.1	ENSG00000254793.1	ENSG00000147862.17
17825	ENSG00000198715.13	ENSG00000254792.1	ENSG00000227591.5
17826	ENSG00000198718.13	ENSG00000254791.1	ENSG00000273090.1
17827	ENSG00000198719.9	ENSG00000254790.1	ENSG00000231560.1
17828	ENSG00000198720.13	ENSG00000254789.2	ENSG00000135801.9
17829	ENSG00000198721.12	ENSG00000254788.7	ENSG00000122490.19
17830	ENSG00000198722.14	ENSG00000254787.1	ENSG00000272601.1
17831	ENSG00000198723.11	ENSG00000254786.1	ENSG00000092203.14
17832	ENSG00000198727.2	ENSG00000254785.1	ENSG00000229927.2
17833	ENSG00000198728.10	ENSG00000254784.1	ENSG00000259719.5
17834	ENSG00000198729.5	ENSG00000254783.1	ENSG00000230385.1
17835	ENSG00000198730.9	ENSG00000254781.1	ENSG00000255507.5
17836	ENSG00000198732.10	ENSG00000254780.1	ENSG00000231249.1
17837	ENSG00000198734.10	ENSG00000254779.4	ENSG00000224945.1
17838	ENSG00000198736.11	ENSG00000254777.5	ENSG00000258757.1
17839	ENSG00000198738.4	ENSG00000254776.1	ENSG00000104880.17
17840	ENSG00000198739.11	ENSG00000254775.1	ENSG00000152127.9
17841	ENSG00000198740.8	ENSG00000254772.10	ENSG00000149823.9
17842	ENSG00000198742.9	ENSG00000254771.1	ENSG00000166736.11
17843	ENSG00000198743.7	ENSG00000254770.1	ENSG00000254963.1
17844	ENSG00000198744.5	ENSG00000254769.3	ENSG00000219085.1
17845	ENSG00000198746.13	ENSG00000254768.5	ENSG00000068438.15
17846	ENSG00000198752.11	ENSG00000254767.1	ENSG00000230592.2
17847	ENSG00000198753.12	ENSG00000254765.1	ENSG00000198673.10
17848	ENSG00000198754.5	ENSG00000254764.2	ENSG00000167874.6
17849	ENSG00000198755.11	ENSG00000254762.1	ENSG00000262959.2
17850	ENSG00000198756.12	ENSG00000254760.1	ENSG00000277745.1
17851	ENSG00000198758.10	ENSG00000254759.1	ENSG00000213300.5
17852	ENSG00000198759.12	ENSG00000254758.1	ENSG00000285653.1
17853	ENSG00000198763.3	ENSG00000254757.1	ENSG00000228541.1
17854	ENSG00000198765.12	ENSG00000254756.1	ENSG00000240583.12
17855	ENSG00000198768.10	ENSG00000254755.1	ENSG00000070018.9
17856	ENSG00000198771.11	ENSG00000254754.1	ENSG00000133597.11
17857	ENSG00000198774.5	ENSG00000254753.1	ENSG00000280152.1
17858	ENSG00000198780.12	ENSG00000254752.2	ENSG00000048162.20

17859	ENSG00000198783.6	ENSG00000254751.3	ENSG00000188211.8
17860	ENSG00000198785.5	ENSG00000254750.1	ENSG00000167004.13
17861	ENSG00000198786.2	ENSG00000254749.1	ENSG00000286185.1
17862	ENSG00000198787.5	ENSG00000254748.1	ENSG00000258240.5
17863	ENSG00000198788.8	ENSG00000254747.1	ENSG00000226259.10
17864	ENSG00000198791.12	ENSG00000254746.5	ENSG00000164741.15
17865	ENSG00000198792.13	ENSG00000254744.3	ENSG00000166145.14
17866	ENSG00000198793.12	ENSG00000254743.1	ENSG00000055955.16
17867	ENSG00000198794.12	ENSG00000254741.1	ENSG00000189129.13
17868	ENSG00000198795.11	ENSG00000254740.2	ENSG00000249852.1
17869	ENSG00000198796.7	ENSG00000254739.1	ENSG00000100075.10
17870	ENSG00000198797.7	ENSG00000254738.1	ENSG00000233998.1
17871	ENSG00000198798.5	ENSG00000254737.2	ENSG00000232586.1
17872	ENSG00000198799.12	ENSG00000254736.1	ENSG00000256646.7
17873	ENSG00000198804.2	ENSG00000254735.1	ENSG00000232032.1
17874	ENSG00000198805.11	ENSG00000254734.1	ENSG00000267283.1
17875	ENSG00000198807.12	ENSG00000254733.1	ENSG00000202395.1
17876	ENSG00000198812.4	ENSG00000254732.1	ENSG00000242527.1
17877	ENSG00000198814.12	ENSG00000254731.1	ENSG00000179673.5
17878	ENSG00000198815.9	ENSG00000254730.1	ENSG00000237015.1
17879	ENSG00000198816.7	ENSG00000254728.1	ENSG00000136630.13
17880	ENSG00000198818.10	ENSG00000254727.1	ENSG00000211972.2
17881	ENSG00000198821.10	ENSG00000254726.3	ENSG00000280411.1
17882	ENSG00000198822.10	ENSG00000254725.1	ENSG00000135094.11
17883	ENSG00000198824.7	ENSG00000254724.1	ENSG00000269397.1
17884	ENSG00000198825.13	ENSG00000254723.1	ENSG00000126583.11
17885	ENSG00000198826.11	ENSG00000254722.1	ENSG00000277150.1
17886	ENSG00000198829.6	ENSG00000254721.1	ENSG00000172361.6
17887	ENSG00000198830.11	ENSG00000254720.1	ENSG00000229648.1
17888	ENSG00000198832.10	ENSG00000254719.1	ENSG00000203747.11
17889	ENSG00000198833.7	ENSG00000254717.2	ENSG00000061676.15
17890	ENSG00000198835.4	ENSG00000254715.3	ENSG00000137216.19
17891	ENSG00000198836.9	ENSG00000254714.1	ENSG00000166510.14
17892	ENSG00000198837.10	ENSG00000254713.2	ENSG00000163686.14
17893	ENSG00000198838.13	ENSG00000254712.1	ENSG00000259330.2
17894	ENSG00000198839.9	ENSG00000254710.1	ENSG00000167281.19
17895	ENSG00000198840.2	ENSG00000254709.7	ENSG00000103653.16
17896	ENSG00000198841.3	ENSG00000254708.1	ENSG00000115271.11
17897	ENSG00000198842.9	ENSG00000254707.1	ENSG00000253250.3
17898	ENSG00000198843.13	ENSG00000254706.2	ENSG00000256084.1
17899	ENSG00000198844.12	ENSG00000254705.1	ENSG00000167914.11
17900	ENSG00000198846.6	ENSG00000254704.3	ENSG00000160111.13
17901	ENSG00000198848.12	ENSG00000254702.1	ENSG00000260911.2
17902	ENSG00000198851.9	ENSG00000254701.3	ENSG00000271474.1
17903	ENSG00000198853.12	ENSG00000254700.1	ENSG00000284738.1
17904	ENSG00000198854.5	ENSG00000254699.1	ENSG00000125170.11
17905	ENSG00000198855.7	ENSG00000254698.1	ENSG00000226337.3
17906	ENSG00000198856.13	ENSG00000254697.1	ENSG00000225733.5
17907	ENSG00000198857.3	ENSG00000254695.1	ENSG00000265808.3
17908	ENSG00000198858.10	ENSG00000254694.1	ENSG00000213842.2
17909	ENSG00000198860.12	ENSG00000254693.1	ENSG00000254459.1
17910	ENSG00000198862.14	ENSG00000254692.1	ENSG00000206028.1
17911	ENSG00000198863.7	ENSG00000254691.1	ENSG00000284744.1

17912	ENSG00000198865.10	ENSG00000254689.2	ENSG00000238227.8
17913	ENSG00000198868.3	ENSG00000254688.1	ENSG00000069493.15
17914	ENSG00000198870.7	ENSG00000254687.1	ENSG00000239821.3
17915	ENSG00000198873.11	ENSG00000254686.2	ENSG00000116096.6
17916	ENSG00000198874.13	ENSG00000254685.6	ENSG00000152137.6
17917	ENSG00000198876.13	ENSG00000254684.1	ENSG00000259648.1
17918	ENSG00000198879.11	ENSG00000254683.1	ENSG00000229663.1
17919	ENSG00000198881.10	ENSG00000254682.1	ENSG00000256210.3
17920	ENSG00000198883.12	ENSG00000254681.6	ENSG00000248684.1
17921	ENSG00000198885.9	ENSG00000254680.1	ENSG00000089693.10
17922	ENSG00000198886.2	ENSG00000254678.1	ENSG00000242952.1
17923	ENSG00000198887.9	ENSG00000254677.1	ENSG00000086712.13
17924	ENSG00000198888.2	ENSG00000254676.1	ENSG00000250698.1
17925	ENSG00000198889.5	ENSG00000254675.1	ENSG00000162194.12
17926	ENSG00000198890.8	ENSG00000254674.1	ENSG00000143028.9
17927	ENSG00000198892.6	ENSG00000254673.1	ENSG00000253302.1
17928	ENSG00000198894.8	ENSG00000254672.1	ENSG00000162972.10
17929	ENSG00000198898.14	ENSG00000254670.1	ENSG00000153487.12
17930	ENSG00000198899.2	ENSG00000254669.1	ENSG00000135439.11
17931	ENSG00000198900.6	ENSG00000254668.1	ENSG00000273240.1
17932	ENSG00000198908.12	ENSG00000254665.1	ENSG00000146540.15
17933	ENSG00000198909.7	ENSG00000254664.1	ENSG00000239799.1
17934	ENSG00000198910.13	ENSG00000254663.1	ENSG00000215096.3
17935	ENSG00000198911.12	ENSG00000254662.1	ENSG00000104213.12
17936	ENSG00000198912.11	ENSG00000254661.2	ENSG00000103855.18
17937	ENSG00000198914.3	ENSG00000254660.1	ENSG00000140955.10
17938	ENSG00000198915.11	ENSG00000254659.2	ENSG00000284876.1
17939	ENSG00000198917.13	ENSG00000254658.3	ENSG00000187559.5
17940	ENSG00000198918.8	ENSG00000254656.2	ENSG00000196358.11
17941	ENSG00000198919.13	ENSG00000254655.1	ENSG00000279494.1
17942	ENSG00000198920.10	ENSG00000254654.1	ENSG00000127481.15
17943	ENSG00000198923.4	ENSG00000254653.1	ENSG00000120279.6
17944	ENSG00000198924.8	ENSG00000254651.1	ENSG00000160211.17
17945	ENSG00000198925.12	ENSG00000254650.1	ENSG00000275355.1
17946	ENSG00000198929.13	ENSG00000254649.1	ENSG00000187837.3
17947	ENSG00000198931.10	ENSG00000254648.1	ENSG00000183638.6
17948	ENSG00000198932.13	ENSG00000254647.6	ENSG00000049768.15
17949	ENSG00000198933.9	ENSG00000254646.1	ENSG00000231398.1
17950	ENSG00000198934.4	ENSG00000254645.1	ENSG00000170242.17
17951	ENSG00000198937.9	ENSG00000254644.1	ENSG00000257477.1
17952	ENSG00000198938.2	ENSG00000254642.1	ENSG00000226721.2
17953	ENSG00000198939.8	ENSG00000254641.1	ENSG00000164307.13
17954	ENSG00000198944.5	ENSG00000254639.1	ENSG00000159423.17
17955	ENSG00000198945.7	ENSG00000254638.1	ENSG00000236617.2
17956	ENSG00000198947.15	ENSG00000254637.1	ENSG00000174611.12
17957	ENSG00000198948.12	ENSG00000254636.1	ENSG00000110841.14
17958	ENSG00000198951.11	ENSG00000254635.5	ENSG00000196262.14
17959	ENSG00000198952.8	ENSG00000254634.4	ENSG00000279198.1
17960	ENSG00000198954.8	ENSG00000254633.1	ENSG00000111644.8
17961	ENSG00000198959.12	ENSG00000254632.1	ENSG00000214888.3
17962	ENSG00000198960.11	ENSG00000254631.5	ENSG00000154734.15
17963	ENSG00000198961.10	ENSG00000254630.1	ENSG00000122566.21
17964	ENSG00000198964.14	ENSG00000254629.1	ENSG00000188095.6

17965	ENSG00000198965.4	ENSG00000254627.1	ENSG00000272689.1
17966	ENSG00000198967.4	ENSG00000254626.1	ENSG00000122728.6
17967	ENSG00000198972.3	ENSG00000254625.1	ENSG00000272109.1
17968	ENSG00000198973.4	ENSG00000254624.1	ENSG00000180340.6
17969	ENSG00000198974.3	ENSG00000254623.1	ENSG00000173621.9
17970	ENSG00000198975.2	ENSG00000254622.1	ENSG00000263311.1
17971	ENSG00000198976.1	ENSG00000254621.1	ENSG00000142188.17
17972	ENSG00000198982.4	ENSG00000254620.1	ENSG00000231770.5
17973	ENSG00000198983.1	ENSG00000254619.1	ENSG00000235590.7
17974	ENSG00000198984.1	ENSG00000254618.1	ENSG00000277791.5
17975	ENSG00000198987.1	ENSG00000254617.1	ENSG00000258570.1
17976	ENSG00000198995.3	ENSG00000254616.1	ENSG00000277617.1
17977	ENSG00000198997.2	ENSG00000254615.2	ENSG00000246203.2
17978	ENSG00000199001.1	ENSG00000254614.2	ENSG00000269667.2
17979	ENSG00000199005.4	ENSG00000254613.2	ENSG00000259069.1
17980	ENSG00000199012.2	ENSG00000254612.2	ENSG00000204778.4
17981	ENSG00000199015.4	ENSG00000254610.3	ENSG00000228157.4
17982	ENSG00000199017.2	ENSG00000254609.1	ENSG00000264017.2
17983	ENSG00000199020.3	ENSG00000254607.2	ENSG00000243403.1
17984	ENSG00000199023.3	ENSG00000254606.1	ENSG00000234322.1
17985	ENSG00000199024.1	ENSG00000254605.1	ENSG00000105281.12
17986	ENSG00000199025.4	ENSG00000254604.1	ENSG00000127507.18
17987	ENSG00000199030.2	ENSG00000254603.1	ENSG00000271824.1
17988	ENSG00000199031.1	ENSG00000254601.1	ENSG00000111728.10
17989	ENSG00000199032.1	ENSG00000254599.1	ENSG00000187486.5
17990	ENSG00000199035.2	ENSG00000254598.2	ENSG00000242067.1
17991	ENSG00000199036.1	ENSG00000254596.1	ENSG00000135476.11
17992	ENSG00000199038.1	ENSG00000254595.1	ENSG00000169427.8
17993	ENSG00000199043.1	ENSG00000254594.5	ENSG00000260490.2
17994	ENSG00000199047.3	ENSG00000254593.1	ENSG00000267150.1
17995	ENSG00000199051.3	ENSG00000254592.1	ENSG00000223466.1
17996	ENSG00000199053.3	ENSG00000254591.1	ENSG00000145358.6
17997	ENSG00000199059.3	ENSG00000254590.1	ENSG00000150048.10
17998	ENSG00000199065.3	ENSG00000254589.1	ENSG00000117020.17
17999	ENSG00000199069.3	ENSG00000254588.1	ENSG00000243661.1
18000	ENSG00000199072.3	ENSG00000254587.1	ENSG00000262097.1
18001	ENSG00000199075.1	ENSG00000254586.1	ENSG00000229519.2
18002	ENSG00000199077.3	ENSG00000254585.4	ENSG00000284747.1
18003	ENSG00000199080.1	ENSG00000254584.1	ENSG00000285704.1
18004	ENSG00000199082.1	ENSG00000254583.1	ENSG00000267213.4
18005	ENSG00000199085.3	ENSG00000254582.1	ENSG00000164305.19
18006	ENSG00000199088.5	ENSG00000254581.1	ENSG00000171953.16
18007	ENSG00000199090.1	ENSG00000254580.1	ENSG00000228487.2
18008	ENSG00000199092.4	ENSG00000254579.2	ENSG00000272871.1
18009	ENSG00000199094.3	ENSG00000254578.1	ENSG00000279201.1
18010	ENSG00000199095.1	ENSG00000254577.1	ENSG00000179477.10
18011	ENSG00000199102.1	ENSG00000254576.2	ENSG00000167536.14
18012	ENSG00000199104.2	ENSG00000254575.1	ENSG00000279853.1
18013	ENSG00000199107.3	ENSG00000254574.1	ENSG00000143207.21
18014	ENSG00000199109.4	ENSG00000254573.1	ENSG00000246528.3
18015	ENSG00000199121.4	ENSG00000254572.2	ENSG00000253729.7
18016	ENSG00000199122.3	ENSG00000254571.1	ENSG00000239494.2
18017	ENSG00000199127.1	ENSG00000254569.1	ENSG00000265579.1

18018	ENSG00000199130.3	ENSG00000254568.1	ENSG00000228808.1
18019	ENSG00000199132.1	ENSG00000254567.2	ENSG00000165209.18
18020	ENSG00000199133.3	ENSG00000254566.1	ENSG00000229227.8
18021	ENSG00000199135.1	ENSG00000254565.1	ENSG00000251152.1
18022	ENSG00000199143.1	ENSG00000254564.1	ENSG00000255503.1
18023	ENSG00000199145.1	ENSG00000254563.1	ENSG00000216809.1
18024	ENSG00000199150.3	ENSG00000254562.6	ENSG00000198467.14
18025	ENSG00000199151.3	ENSG00000254561.3	ENSG00000259600.2
18026	ENSG00000199153.1	ENSG00000254560.5	ENSG00000166922.8
18027	ENSG00000199156.1	ENSG00000254559.1	ENSG00000273212.1
18028	ENSG00000199157.2	ENSG00000254558.1	ENSG00000173917.10
18029	ENSG00000199158.1	ENSG00000254557.1	ENSG00000162627.17
18030	ENSG00000199161.1	ENSG00000254556.1	ENSG00000126878.13
18031	ENSG00000199165.3	ENSG00000254555.1	ENSG00000169668.11
18032	ENSG00000199168.3	ENSG00000254554.1	ENSG00000102144.15
18033	ENSG00000199169.1	ENSG00000254553.1	ENSG00000129244.9
18034	ENSG00000199172.3	ENSG00000254552.1	ENSG00000148824.19
18035	ENSG00000199177.1	ENSG00000254551.1	ENSG00000214578.5
18036	ENSG00000199179.3	ENSG00000254550.1	ENSG00000073756.12
18037	ENSG00000199196.1	ENSG00000254548.1	ENSG00000271959.1
18038	ENSG00000199197.1	ENSG00000254547.1	ENSG00000284826.1
18039	ENSG00000199200.2	ENSG00000254546.1	ENSG00000203280.4
18040	ENSG00000199201.1	ENSG00000254545.1	ENSG00000138744.15
18041	ENSG00000199202.1	ENSG00000254544.1	ENSG00000258869.1
18042	ENSG00000199203.1	ENSG00000254543.1	ENSG00000213889.10
18043	ENSG00000199204.1	ENSG00000254542.1	ENSG00000112110.10
18044	ENSG00000199212.1	ENSG00000254541.1	ENSG00000115350.11
18045	ENSG00000199217.1	ENSG00000254540.1	ENSG00000273381.1
18046	ENSG00000199218.1	ENSG00000254539.1	ENSG00000112343.11
18047	ENSG00000199219.1	ENSG00000254538.1	ENSG00000249242.8
18048	ENSG00000199220.1	ENSG00000254537.1	ENSG00000237433.1
18049	ENSG00000199222.1	ENSG00000254536.1	ENSG00000214999.3
18050	ENSG00000199223.1	ENSG00000254535.4	ENSG00000204103.4
18051	ENSG00000199224.1	ENSG00000254534.1	ENSG00000270082.1
18052	ENSG00000199226.1	ENSG00000254533.1	ENSG00000170653.19
18053	ENSG00000199231.1	ENSG00000254532.1	ENSG00000231864.2
18054	ENSG00000199237.1	ENSG00000254531.1	ENSG00000107593.17
18055	ENSG00000199240.1	ENSG00000254530.1	ENSG00000274549.1
18056	ENSG00000199241.1	ENSG00000254529.1	ENSG00000146574.15
18057	ENSG00000199245.1	ENSG00000254527.1	ENSG00000145113.22
18058	ENSG00000199246.1	ENSG00000254526.1	ENSG00000178623.12
18059	ENSG00000199248.1	ENSG00000254524.1	ENSG00000273293.1
18060	ENSG00000199251.1	ENSG00000254522.1	ENSG00000261101.2
18061	ENSG00000199260.1	ENSG00000254521.6	ENSG00000280385.1
18062	ENSG00000199263.1	ENSG00000254519.4	ENSG00000141096.5
18063	ENSG00000199266.1	ENSG00000254518.1	ENSG00000137261.14
18064	ENSG00000199270.1	ENSG00000254517.1	ENSG00000213471.10
18065	ENSG00000199272.1	ENSG00000254516.1	ENSG00000130595.19
18066	ENSG00000199273.1	ENSG00000254515.1	ENSG00000228329.2
18067	ENSG00000199276.1	ENSG00000254514.1	ENSG00000248406.1
18068	ENSG00000199279.1	ENSG00000254512.1	ENSG00000276026.1
18069	ENSG00000199282.1	ENSG00000254511.1	ENSG00000226356.2
18070	ENSG00000199283.1	ENSG00000254510.1	ENSG00000278643.1

18071	ENSG00000199285.1	ENSG00000254509.1	ENSG00000127463.15
18072	ENSG00000199286.1	ENSG00000254508.5	ENSG00000244620.1
18073	ENSG00000199289.1	ENSG00000254507.2	ENSG00000206910.1
18074	ENSG00000199290.1	ENSG00000254506.1	ENSG00000172954.13
18075	ENSG00000199291.1	ENSG00000254505.10	ENSG00000224769.1
18076	ENSG00000199293.1	ENSG00000254503.1	ENSG00000092010.15
18077	ENSG00000199295.1	ENSG00000254502.1	ENSG00000279597.1
18078	ENSG00000199299.1	ENSG00000254501.1	ENSG00000248318.1
18079	ENSG00000199301.1	ENSG00000254500.1	ENSG00000183196.10
18080	ENSG00000199303.1	ENSG00000254499.1	ENSG00000196357.11
18081	ENSG00000199306.1	ENSG00000254498.1	ENSG00000127325.19
18082	ENSG00000199308.1	ENSG00000254497.1	ENSG00000213539.4
18083	ENSG00000199311.1	ENSG00000254496.1	ENSG00000272768.1
18084	ENSG00000199313.1	ENSG00000254495.1	ENSG00000279232.2
18085	ENSG00000199315.1	ENSG00000254492.1	ENSG00000164736.6
18086	ENSG00000199318.1	ENSG00000254491.1	ENSG00000236773.1
18087	ENSG00000199319.1	ENSG00000254490.1	ENSG00000229211.3
18088	ENSG00000199321.1	ENSG00000254489.1	ENSG00000137819.13
18089	ENSG00000199322.1	ENSG00000254488.1	ENSG00000160352.15
18090	ENSG00000199325.1	ENSG00000254487.2	ENSG00000144283.21
18091	ENSG00000199326.1	ENSG00000254486.1	ENSG00000255321.1
18092	ENSG00000199327.1	ENSG00000254485.5	ENSG00000223318.1
18093	ENSG00000199331.1	ENSG00000254484.1	ENSG00000253846.2
18094	ENSG00000199332.1	ENSG00000254483.1	ENSG00000234546.3
18095	ENSG00000199334.1	ENSG00000254482.1	ENSG00000173210.19
18096	ENSG00000199335.1	ENSG00000254481.1	ENSG00000279684.1
18097	ENSG00000199337.1	ENSG00000254480.1	ENSG00000237950.1
18098	ENSG00000199347.1	ENSG00000254479.1	ENSG00000236409.2
18099	ENSG00000199348.1	ENSG00000254478.1	ENSG00000141084.11
18100	ENSG00000199349.1	ENSG00000254477.2	ENSG00000273328.5
18101	ENSG00000199350.1	ENSG00000254475.1	ENSG00000109445.11
18102	ENSG00000199352.1	ENSG00000254473.1	ENSG00000186075.12
18103	ENSG00000199354.1	ENSG00000254472.1	ENSG00000257527.1
18104	ENSG00000199357.1	ENSG00000254471.1	ENSG00000087299.12
18105	ENSG00000199360.1	ENSG00000254470.2	ENSG00000214439.4
18106	ENSG00000199361.1	ENSG00000254469.7	ENSG00000285314.1
18107	ENSG00000199362.1	ENSG00000254468.2	ENSG00000148926.10
18108	ENSG00000199363.1	ENSG00000254467.1	ENSG00000224825.2
18109	ENSG00000199364.1	ENSG00000254466.2	ENSG00000004142.12
18110	ENSG00000199366.1	ENSG00000254465.1	ENSG00000179088.14
18111	ENSG00000199368.1	ENSG00000254464.1	ENSG00000279319.1
18112	ENSG00000199370.2	ENSG00000254463.1	ENSG00000187240.15
18113	ENSG00000199373.1	ENSG00000254462.1	ENSG00000187678.9
18114	ENSG00000199377.1	ENSG00000254461.1	ENSG00000228212.1
18115	ENSG00000199378.1	ENSG00000254460.1	ENSG00000250658.1
18116	ENSG00000199381.1	ENSG00000254459.1	ENSG00000284522.1
18117	ENSG00000199385.1	ENSG00000254458.1	ENSG00000266145.1
18118	ENSG00000199390.1	ENSG00000254457.3	ENSG00000202343.1
18119	ENSG00000199392.1	ENSG00000254456.5	ENSG00000122194.18
18120	ENSG00000199394.1	ENSG00000254455.1	ENSG00000273076.1
18121	ENSG00000199395.1	ENSG00000254454.2	ENSG00000251039.2
18122	ENSG00000199396.1	ENSG00000254453.1	ENSG00000100234.11
18123	ENSG00000199398.1	ENSG00000254452.1	ENSG00000255221.3

18124	ENSG00000199400.1	ENSG00000254451.2	ENSG00000214526.3
18125	ENSG00000199402.1	ENSG00000254450.1	ENSG00000245975.2
18126	ENSG00000199404.1	ENSG00000254449.1	ENSG00000086200.16
18127	ENSG00000199405.1	ENSG00000254447.3	ENSG00000236980.9
18128	ENSG00000199407.1	ENSG00000254445.1	ENSG00000225292.2
18129	ENSG00000199409.1	ENSG00000254444.1	ENSG00000196188.11
18130	ENSG00000199410.1	ENSG00000254443.1	ENSG00000258504.2
18131	ENSG00000199411.1	ENSG00000254442.1	ENSG00000233261.3
18132	ENSG00000199415.1	ENSG00000254441.1	ENSG00000254531.1
18133	ENSG00000199420.1	ENSG00000254440.3	ENSG00000196531.10
18134	ENSG00000199422.1	ENSG00000254438.1	ENSG00000262480.2
18135	ENSG00000199424.1	ENSG00000254437.1	ENSG00000020181.17
18136	ENSG00000199426.1	ENSG00000254436.1	ENSG00000147465.11
18137	ENSG00000199436.1	ENSG00000254434.1	ENSG00000224717.1
18138	ENSG00000199440.1	ENSG00000254433.1	ENSG00000163840.10
18139	ENSG00000199442.1	ENSG00000254432.1	ENSG00000284808.1
18140	ENSG00000199444.1	ENSG00000254431.1	ENSG00000085998.14
18141	ENSG00000199446.1	ENSG00000254430.1	ENSG00000200488.1
18142	ENSG00000199448.1	ENSG00000254429.1	ENSG00000135052.16
18143	ENSG00000199450.1	ENSG00000254428.1	ENSG00000109265.14
18144	ENSG00000199453.1	ENSG00000254427.1	ENSG00000272316.1
18145	ENSG00000199454.1	ENSG00000254425.1	ENSG00000099977.14
18146	ENSG00000199455.1	ENSG00000254424.1	ENSG00000268087.1
18147	ENSG00000199458.1	ENSG00000254423.1	ENSG00000219870.2
18148	ENSG00000199459.1	ENSG00000254422.1	ENSG00000188032.9
18149	ENSG00000199460.2	ENSG00000254420.1	ENSG00000283597.2
18150	ENSG00000199461.1	ENSG00000254419.1	ENSG00000157570.11
18151	ENSG00000199466.1	ENSG00000254418.1	ENSG00000127412.6
18152	ENSG00000199468.1	ENSG00000254417.1	ENSG00000230896.1
18153	ENSG00000199469.1	ENSG00000254416.5	ENSG00000276017.1
18154	ENSG00000199471.1	ENSG00000254415.3	ENSG00000263893.2
18155	ENSG00000199472.1	ENSG00000254413.8	ENSG00000214456.8
18156	ENSG00000199473.1	ENSG00000254412.1	ENSG00000114541.15
18157	ENSG00000199474.1	ENSG00000254411.1	ENSG00000261687.1
18158	ENSG00000199475.1	ENSG00000254409.3	ENSG00000259548.1
18159	ENSG00000199476.1	ENSG00000254408.1	ENSG00000160325.14
18160	ENSG00000199477.1	ENSG00000254407.1	ENSG00000212308.1
18161	ENSG00000199480.1	ENSG00000254406.1	ENSG00000040531.14
18162	ENSG00000199482.1	ENSG00000254404.1	ENSG00000229156.1
18163	ENSG00000199483.1	ENSG00000254403.1	ENSG00000239862.1
18164	ENSG00000199487.1	ENSG00000254402.7	ENSG00000149100.13
18165	ENSG00000199488.1	ENSG00000254401.2	ENSG00000166181.13
18166	ENSG00000199489.1	ENSG00000254399.2	ENSG00000233527.8
18167	ENSG00000199490.1	ENSG00000254398.1	ENSG00000230358.4
18168	ENSG00000199492.1	ENSG00000254397.1	ENSG00000121898.13
18169	ENSG00000199497.1	ENSG00000254396.1	ENSG00000205929.11
18170	ENSG00000199506.1	ENSG00000254394.1	ENSG00000089682.16
18171	ENSG00000199508.1	ENSG00000254392.1	ENSG00000243544.3
18172	ENSG00000199509.1	ENSG00000254391.1	ENSG00000242083.2
18173	ENSG00000199512.1	ENSG00000254389.3	ENSG00000222018.1
18174	ENSG00000199514.1	ENSG00000254388.1	ENSG00000248751.6
18175	ENSG00000199515.1	ENSG00000254387.1	ENSG00000277117.4
18176	ENSG00000199516.1	ENSG00000254384.1	ENSG00000224307.1

18177	ENSG00000199520.1	ENSG00000254383.1	ENSG00000226086.5
18178	ENSG00000199523.1	ENSG00000254381.3	ENSG00000217889.3
18179	ENSG00000199525.1	ENSG00000254380.1	ENSG00000225092.2
18180	ENSG00000199529.1	ENSG00000254377.5	ENSG00000268049.1
18181	ENSG00000199530.1	ENSG00000254376.2	ENSG00000276442.1
18182	ENSG00000199535.1	ENSG00000254373.1	ENSG00000125898.13
18183	ENSG00000199536.1	ENSG00000254372.1	ENSG00000165175.15
18184	ENSG00000199540.1	ENSG00000254370.1	ENSG00000257818.1
18185	ENSG00000199545.1	ENSG00000254369.6	ENSG00000079931.15
18186	ENSG00000199546.1	ENSG00000254367.5	ENSG00000281732.1
18187	ENSG00000199550.1	ENSG00000254366.6	ENSG00000270554.1
18188	ENSG00000199551.1	ENSG00000254365.1	ENSG00000119703.14
18189	ENSG00000199552.1	ENSG00000254364.1	ENSG00000258684.2
18190	ENSG00000199556.1	ENSG00000254363.6	ENSG00000283709.1
18191	ENSG00000199562.1	ENSG00000254362.1	ENSG00000143624.14
18192	ENSG00000199564.1	ENSG00000254361.1	ENSG00000101439.9
18193	ENSG00000199565.1	ENSG00000254358.1	ENSG00000204099.11
18194	ENSG00000199566.1	ENSG00000254357.1	ENSG00000183022.5
18195	ENSG00000199567.1	ENSG00000254355.1	ENSG00000175003.14
18196	ENSG00000199568.1	ENSG00000254352.1	ENSG00000227440.1
18197	ENSG00000199570.1	ENSG00000254351.1	ENSG00000068400.13
18198	ENSG00000199571.1	ENSG00000254350.1	ENSG00000112530.11
18199	ENSG00000199572.1	ENSG00000254349.5	ENSG00000285329.1
18200	ENSG00000199574.1	ENSG00000254348.1	ENSG00000174326.11
18201	ENSG00000199575.1	ENSG00000254347.1	ENSG00000128585.18
18202	ENSG00000199577.1	ENSG00000254346.2	ENSG00000249222.1
18203	ENSG00000199580.1	ENSG00000254345.1	ENSG00000088876.11
18204	ENSG00000199584.1	ENSG00000254344.2	ENSG00000223387.6
18205	ENSG00000199585.1	ENSG00000254343.2	ENSG00000188107.14
18206	ENSG00000199591.1	ENSG00000254342.1	ENSG00000145390.11
18207	ENSG00000199592.1	ENSG00000254341.2	ENSG00000228686.2
18208	ENSG00000199593.1	ENSG00000254340.1	ENSG00000261188.1
18209	ENSG00000199594.1	ENSG00000254339.5	ENSG00000257497.2
18210	ENSG00000199595.1	ENSG00000254338.1	ENSG00000183715.13
18211	ENSG00000199598.1	ENSG00000254337.1	ENSG00000232934.7
18212	ENSG00000199601.1	ENSG00000254336.1	ENSG00000279718.1
18213	ENSG00000199603.1	ENSG00000254335.1	ENSG00000277440.1
18214	ENSG00000199605.1	ENSG00000254334.1	ENSG00000205810.8
18215	ENSG00000199609.1	ENSG00000254333.1	ENSG00000254362.1
18216	ENSG00000199620.1	ENSG00000254332.1	ENSG00000213683.4
18217	ENSG00000199622.1	ENSG00000254331.1	ENSG00000283929.1
18218	ENSG00000199626.1	ENSG00000254330.1	ENSG00000258640.3
18219	ENSG00000199627.1	ENSG00000254329.1	ENSG00000234997.1
18220	ENSG00000199629.1	ENSG00000254328.1	ENSG00000255933.1
18221	ENSG00000199630.1	ENSG00000254326.1	ENSG00000225920.2
18222	ENSG00000199631.1	ENSG00000254325.2	ENSG00000272462.2
18223	ENSG00000199633.1	ENSG00000254324.3	ENSG00000168955.3
18224	ENSG00000199634.1	ENSG00000254321.1	ENSG00000211645.2
18225	ENSG00000199635.1	ENSG00000254320.1	ENSG00000284614.1
18226	ENSG00000199636.1	ENSG00000254319.5	ENSG00000223547.10
18227	ENSG00000199638.1	ENSG00000254317.1	ENSG00000213493.3
18228	ENSG00000199640.1	ENSG00000254316.1	ENSG00000155016.18
18229	ENSG00000199643.1	ENSG00000254315.1	ENSG00000135709.12

18230	ENSG00000199645.1	ENSG00000254314.1	ENSG00000223823.1
18231	ENSG00000199646.1	ENSG00000254313.1	ENSG00000223656.1
18232	ENSG00000199652.2	ENSG00000254312.1	ENSG00000232487.1
18233	ENSG00000199664.1	ENSG00000254311.1	ENSG00000278740.1
18234	ENSG00000199666.2	ENSG00000254310.1	ENSG00000153339.14
18235	ENSG00000199667.1	ENSG00000254309.1	ENSG00000273483.1
18236	ENSG00000199668.1	ENSG00000254308.1	ENSG00000236570.1
18237	ENSG00000199672.1	ENSG00000254307.2	ENSG00000182308.7
18238	ENSG00000199673.1	ENSG00000254306.1	ENSG00000166971.17
18239	ENSG00000199674.1	ENSG00000254305.1	ENSG00000105376.5
18240	ENSG00000199676.1	ENSG00000254303.1	ENSG00000116701.14
18241	ENSG00000199677.1	ENSG00000254302.1	ENSG00000077782.20
18242	ENSG00000199683.1	ENSG00000254300.1	ENSG00000116815.16
18243	ENSG00000199687.1	ENSG00000254299.2	ENSG00000254765.1
18244	ENSG00000199691.1	ENSG00000254298.1	ENSG00000196778.3
18245	ENSG00000199695.1	ENSG00000254297.1	ENSG00000119508.18
18246	ENSG00000199697.1	ENSG00000254295.1	ENSG00000237931.1
18247	ENSG00000199698.1	ENSG00000254294.1	ENSG00000235001.3
18248	ENSG00000199700.1	ENSG00000254293.1	ENSG00000256167.1
18249	ENSG00000199701.1	ENSG00000254292.1	ENSG00000211899.10
18250	ENSG00000199702.1	ENSG00000254291.1	ENSG00000246627.6
18251	ENSG00000199704.1	ENSG00000254290.1	ENSG00000276089.1
18252	ENSG00000199705.1	ENSG00000254289.2	ENSG00000100276.10
18253	ENSG00000199709.1	ENSG00000254288.1	ENSG00000243649.8
18254	ENSG00000199710.1	ENSG00000254287.1	ENSG00000196993.8
18255	ENSG00000199711.1	ENSG00000254285.3	ENSG00000162971.11
18256	ENSG00000199712.1	ENSG00000254283.1	ENSG00000228474.6
18257	ENSG00000199713.1	ENSG00000254279.1	ENSG00000258099.1
18258	ENSG00000199715.1	ENSG00000254278.1	ENSG00000105971.15
18259	ENSG00000199716.1	ENSG00000254277.1	ENSG00000234068.6
18260	ENSG00000199728.1	ENSG00000254275.6	ENSG00000144711.16
18261	ENSG00000199730.1	ENSG00000254274.1	ENSG00000185650.9
18262	ENSG00000199731.1	ENSG00000254273.1	ENSG00000272815.1
18263	ENSG00000199732.1	ENSG00000254272.1	ENSG00000214077.4
18264	ENSG00000199733.1	ENSG00000254271.1	ENSG00000257681.1
18265	ENSG00000199735.1	ENSG00000254270.1	ENSG00000272343.1
18266	ENSG00000199739.1	ENSG00000254268.1	ENSG00000132677.13
18267	ENSG00000199740.1	ENSG00000254266.5	ENSG00000221996.6
18268	ENSG00000199744.1	ENSG00000254265.1	ENSG00000168010.11
18269	ENSG00000199751.1	ENSG00000254264.2	ENSG00000187556.7
18270	ENSG00000199753.1	ENSG00000254263.1	ENSG00000260895.1
18271	ENSG00000199756.1	ENSG00000254261.1	ENSG00000111057.11
18272	ENSG00000199762.1	ENSG00000254260.1	ENSG00000270300.2
18273	ENSG00000199764.1	ENSG00000254258.1	ENSG00000237425.1
18274	ENSG00000199765.1	ENSG00000254256.1	ENSG00000262481.5
18275	ENSG00000199767.1	ENSG00000254255.1	ENSG00000160180.15
18276	ENSG00000199771.1	ENSG00000254254.5	ENSG00000231822.1
18277	ENSG00000199773.1	ENSG00000254253.1	ENSG00000240809.1
18278	ENSG00000199780.1	ENSG00000254252.1	ENSG00000129518.9
18279	ENSG00000199781.1	ENSG00000254251.1	ENSG00000212238.1
18280	ENSG00000199782.1	ENSG00000254249.1	ENSG00000165813.20
18281	ENSG00000199783.1	ENSG00000254248.1	ENSG00000231655.1
18282	ENSG00000199784.1	ENSG00000254247.1	ENSG00000112183.15

18283	ENSG00000199785.1	ENSG00000254246.1	ENSG00000251141.5
18284	ENSG00000199786.1	ENSG00000254245.2	ENSG00000280094.2
18285	ENSG00000199787.1	ENSG00000254244.1	ENSG00000272478.1
18286	ENSG00000199788.1	ENSG00000254242.1	ENSG00000267644.1
18287	ENSG00000199790.1	ENSG00000254241.1	ENSG00000184432.10
18288	ENSG00000199791.1	ENSG00000254240.1	ENSG00000271119.1
18289	ENSG00000199792.1	ENSG00000254239.1	ENSG00000135747.11
18290	ENSG00000199796.1	ENSG00000254238.1	ENSG00000264235.5
18291	ENSG00000199797.1	ENSG00000254237.5	ENSG00000227684.2
18292	ENSG00000199798.1	ENSG00000254236.1	ENSG00000269711.1
18293	ENSG00000199801.1	ENSG00000254233.1	ENSG00000258551.5
18294	ENSG00000199803.1	ENSG00000254231.2	ENSG00000219200.11
18295	ENSG00000199804.1	ENSG00000254230.1	ENSG00000167306.20
18296	ENSG00000199805.1	ENSG00000254229.2	ENSG00000088298.13
18297	ENSG00000199806.1	ENSG00000254228.2	ENSG00000197550.3
18298	ENSG00000199809.1	ENSG00000254227.1	ENSG00000272010.1
18299	ENSG00000199812.1	ENSG00000254226.5	ENSG00000267670.1
18300	ENSG00000199814.1	ENSG00000254225.1	ENSG00000154099.18
18301	ENSG00000199815.2	ENSG00000254224.1	ENSG00000272308.1
18302	ENSG00000199824.1	ENSG00000254222.1	ENSG00000116761.11
18303	ENSG00000199827.1	ENSG00000254221.2	ENSG00000185385.4
18304	ENSG00000199831.1	ENSG00000254220.1	ENSG00000228863.8
18305	ENSG00000199832.1	ENSG00000254219.1	ENSG00000280233.1
18306	ENSG00000199833.1	ENSG00000254216.1	ENSG00000101974.14
18307	ENSG00000199836.1	ENSG00000254215.1	ENSG00000227700.1
18308	ENSG00000199837.1	ENSG00000254213.2	ENSG00000211787.1
18309	ENSG00000199839.1	ENSG00000254212.1	ENSG00000106070.19
18310	ENSG00000199840.1	ENSG00000254211.5	ENSG00000163217.2
18311	ENSG00000199843.1	ENSG00000254209.1	ENSG00000276742.1
18312	ENSG00000199845.1	ENSG00000254208.1	ENSG00000132702.13
18313	ENSG00000199846.1	ENSG00000254207.1	ENSG00000285791.1
18314	ENSG00000199849.1	ENSG00000254206.5	ENSG00000012223.12
18315	ENSG00000199851.2	ENSG00000254205.1	ENSG00000235908.1
18316	ENSG00000199855.1	ENSG00000254204.1	ENSG00000228763.1
18317	ENSG00000199856.2	ENSG00000254203.1	ENSG00000273963.1
18318	ENSG00000199857.1	ENSG00000254202.1	ENSG00000101871.14
18319	ENSG00000199858.1	ENSG00000254201.1	ENSG00000217801.10
18320	ENSG00000199859.1	ENSG00000254200.1	ENSG00000115850.10
18321	ENSG00000199862.1	ENSG00000254198.1	ENSG00000120093.11
18322	ENSG00000199865.1	ENSG00000254197.1	ENSG00000181408.3
18323	ENSG00000199866.1	ENSG00000254195.1	ENSG00000282939.1
18324	ENSG00000199867.1	ENSG00000254194.5	ENSG00000182901.16
18325	ENSG00000199870.1	ENSG00000254193.1	ENSG00000258811.1
18326	ENSG00000199872.1	ENSG00000254192.1	ENSG00000237945.7
18327	ENSG00000199874.1	ENSG00000254190.1	ENSG00000229948.2
18328	ENSG00000199875.1	ENSG00000254189.1	ENSG00000188186.10
18329	ENSG00000199878.1	ENSG00000254187.1	ENSG00000285053.1
18330	ENSG00000199879.1	ENSG00000254186.2	ENSG00000163746.11
18331	ENSG00000199880.1	ENSG00000254185.1	ENSG00000227920.2
18332	ENSG00000199881.1	ENSG00000254183.1	ENSG00000163467.11
18333	ENSG00000199883.1	ENSG00000254182.1	ENSG00000279530.2
18334	ENSG00000199884.1	ENSG00000254181.1	ENSG00000261783.1
18335	ENSG00000199885.1	ENSG00000254180.1	ENSG00000189166.6

18336	ENSG00000199886.1	ENSG00000254178.1	ENSG00000179915.23
18337	ENSG00000199890.1	ENSG00000254177.1	ENSG00000274213.1
18338	ENSG00000199892.2	ENSG00000254176.1	ENSG00000279924.1
18339	ENSG00000199894.1	ENSG00000254175.1	ENSG00000164548.11
18340	ENSG00000199895.1	ENSG00000254174.1	ENSG00000125965.9
18341	ENSG00000199899.2	ENSG00000254172.1	ENSG00000272030.1
18342	ENSG00000199900.1	ENSG00000254171.1	ENSG00000225690.1
18343	ENSG00000199901.1	ENSG00000254170.1	ENSG00000205309.14
18344	ENSG00000199903.1	ENSG00000254167.1	ENSG00000234699.1
18345	ENSG00000199905.1	ENSG00000254166.2	ENSG00000272662.1
18346	ENSG00000199906.1	ENSG00000254165.1	ENSG00000151093.8
18347	ENSG00000199910.1	ENSG00000254164.1	ENSG00000228078.1
18348	ENSG00000199911.1	ENSG00000254163.1	ENSG00000162551.14
18349	ENSG00000199912.1	ENSG00000254162.1	ENSG00000240663.3
18350	ENSG00000199913.1	ENSG00000254161.1	ENSG00000136527.18
18351	ENSG00000199914.1	ENSG00000254160.1	ENSG00000251484.3
18352	ENSG00000199920.1	ENSG00000254158.1	ENSG00000131142.13
18353	ENSG00000199921.1	ENSG00000254157.1	ENSG00000272002.1
18354	ENSG00000199924.1	ENSG00000254156.1	ENSG00000123143.12
18355	ENSG00000199927.2	ENSG00000254154.8	ENSG00000138621.12
18356	ENSG00000199929.1	ENSG00000254153.1	ENSG00000060566.14
18357	ENSG00000199932.1	ENSG00000254152.1	ENSG00000268861.6
18358	ENSG00000199933.1	ENSG00000254151.1	ENSG00000249516.1
18359	ENSG00000199934.1	ENSG00000254150.1	ENSG00000177335.10
18360	ENSG00000199936.1	ENSG00000254146.1	ENSG00000273456.1
18361	ENSG00000199938.1	ENSG00000254145.1	ENSG00000286205.1
18362	ENSG00000199940.1	ENSG00000254143.1	ENSG00000269896.2
18363	ENSG00000199942.1	ENSG00000254141.1	ENSG00000244625.6
18364	ENSG00000199944.1	ENSG00000254139.1	ENSG00000161681.15
18365	ENSG00000199949.2	ENSG00000254138.1	ENSG00000179071.5
18366	ENSG00000199953.1	ENSG00000254136.1	ENSG00000236516.1
18367	ENSG00000199959.1	ENSG00000254135.2	ENSG00000102854.16
18368	ENSG00000199960.1	ENSG00000254134.1	ENSG00000213452.4
18369	ENSG00000199961.1	ENSG00000254132.1	ENSG00000224086.5
18370	ENSG00000199962.1	ENSG00000254131.1	ENSG00000187627.15
18371	ENSG00000199963.1	ENSG00000254130.1	ENSG00000232203.3
18372	ENSG00000199964.1	ENSG00000254129.1	ENSG00000265531.3
18373	ENSG00000199968.1	ENSG00000254127.1	ENSG00000035928.16
18374	ENSG00000199970.1	ENSG00000254126.7	ENSG00000277258.5
18375	ENSG00000199971.1	ENSG00000254124.1	ENSG00000269837.1
18376	ENSG00000199975.1	ENSG00000254123.1	ENSG00000267249.1
18377	ENSG00000199979.1	ENSG00000254122.2	ENSG00000196696.12
18378	ENSG00000199985.1	ENSG00000254120.1	ENSG00000149531.15
18379	ENSG00000199986.1	ENSG00000254119.5	ENSG00000270112.3
18380	ENSG00000199990.1	ENSG00000254118.1	ENSG00000228830.1
18381	ENSG00000199994.1	ENSG00000254115.1	ENSG00000120837.8
18382	ENSG00000200003.1	ENSG00000254114.1	ENSG00000273284.1
18383	ENSG00000200008.1	ENSG00000254113.1	ENSG00000162746.14
18384	ENSG00000200011.1	ENSG00000254112.1	ENSG00000100450.13
18385	ENSG00000200013.1	ENSG00000254111.2	ENSG00000213438.2
18386	ENSG00000200021.1	ENSG00000254109.5	ENSG00000205302.7
18387	ENSG00000200024.1	ENSG00000254106.1	ENSG00000158485.10
18388	ENSG00000200026.1	ENSG00000254105.1	ENSG00000230320.1

18389	ENSG00000200028.1	ENSG00000254104.2	ENSG00000102794.9
18390	ENSG00000200029.1	ENSG00000254103.1	ENSG00000120549.18
18391	ENSG00000200033.1	ENSG00000254102.1	ENSG00000181444.13
18392	ENSG00000200034.1	ENSG00000254101.6	ENSG00000250746.1
18393	ENSG00000200036.1	ENSG00000254099.1	ENSG00000153551.13
18394	ENSG00000200040.1	ENSG00000254098.1	ENSG00000100116.16
18395	ENSG00000200041.1	ENSG00000254097.1	ENSG00000279957.1
18396	ENSG00000200047.1	ENSG00000254095.1	ENSG00000122644.13
18397	ENSG00000200048.1	ENSG00000254094.1	ENSG00000271793.1
18398	ENSG00000200049.1	ENSG00000254093.9	ENSG00000164855.16
18399	ENSG00000200051.1	ENSG00000254092.1	ENSG00000268320.3
18400	ENSG00000200052.1	ENSG00000254091.2	ENSG00000243629.1
18401	ENSG00000200057.1	ENSG00000254090.1	ENSG00000264538.6
18402	ENSG00000200058.1	ENSG00000254089.1	ENSG00000172893.15
18403	ENSG00000200059.1	ENSG00000254088.1	ENSG00000234776.5
18404	ENSG00000200060.1	ENSG00000254087.8	ENSG00000228800.1
18405	ENSG00000200062.1	ENSG00000254086.1	ENSG00000213731.2
18406	ENSG00000200063.1	ENSG00000254084.1	ENSG00000181649.7
18407	ENSG00000200064.1	ENSG00000254083.5	ENSG00000155265.11
18408	ENSG00000200065.1	ENSG00000254081.1	ENSG00000283415.1
18409	ENSG00000200070.1	ENSG00000254080.1	ENSG00000259224.2
18410	ENSG00000200072.1	ENSG00000254079.1	ENSG00000284882.1
18411	ENSG00000200075.1	ENSG00000254077.1	ENSG00000144426.18
18412	ENSG00000200079.1	ENSG00000254076.1	ENSG00000279858.1
18413	ENSG00000200084.1	ENSG00000254075.1	ENSG00000177646.19
18414	ENSG00000200085.1	ENSG00000254073.1	ENSG00000163877.11
18415	ENSG00000200086.1	ENSG00000254070.1	ENSG00000286167.1
18416	ENSG00000200087.1	ENSG00000254069.2	ENSG00000103066.13
18417	ENSG00000200089.1	ENSG00000254067.2	ENSG00000234902.6
18418	ENSG00000200090.1	ENSG00000254066.1	ENSG00000230069.3
18419	ENSG00000200091.1	ENSG00000254065.1	ENSG00000168158.3
18420	ENSG00000200095.1	ENSG00000254064.1	ENSG00000267672.1
18421	ENSG00000200097.1	ENSG00000254063.2	ENSG00000157766.16
18422	ENSG00000200101.1	ENSG00000254061.1	ENSG00000163631.17
18423	ENSG00000200102.1	ENSG00000254060.1	ENSG00000249934.2
18424	ENSG00000200105.1	ENSG00000254057.1	ENSG00000255517.6
18425	ENSG00000200106.1	ENSG00000254056.1	ENSG00000240490.3
18426	ENSG00000200107.1	ENSG00000254055.1	ENSG00000270240.2
18427	ENSG00000200108.1	ENSG00000254054.2	ENSG00000177508.12
18428	ENSG00000200112.1	ENSG00000254053.1	ENSG00000251050.1
18429	ENSG00000200113.1	ENSG00000254052.1	ENSG00000230074.1
18430	ENSG00000200114.1	ENSG00000254051.1	ENSG00000155974.12
18431	ENSG00000200118.1	ENSG00000254050.1	ENSG00000100504.17
18432	ENSG00000200120.1	ENSG00000254049.1	ENSG00000282535.1
18433	ENSG00000200121.2	ENSG00000254048.1	ENSG00000275092.1
18434	ENSG00000200131.1	ENSG00000254047.2	ENSG00000131097.7
18435	ENSG00000200132.1	ENSG00000254046.1	ENSG00000215210.3
18436	ENSG00000200135.1	ENSG00000254045.1	ENSG00000253722.1
18437	ENSG00000200138.1	ENSG00000254044.5	ENSG00000188549.12
18438	ENSG00000200139.1	ENSG00000254043.1	ENSG00000263063.1
18439	ENSG00000200142.1	ENSG00000254042.1	ENSG00000262902.1
18440	ENSG00000200146.1	ENSG00000254041.1	ENSG00000213886.3
18441	ENSG00000200150.1	ENSG00000254040.1	ENSG00000260924.2

18442	ENSG00000200151.1	ENSG00000254039.1	ENSG00000225978.3
18443	ENSG00000200152.1	ENSG00000254038.1	ENSG00000278434.1
18444	ENSG00000200153.1	ENSG00000254037.2	ENSG00000224892.6
18445	ENSG00000200156.1	ENSG00000254036.1	ENSG00000213003.3
18446	ENSG00000200161.1	ENSG00000254035.1	ENSG00000259465.2
18447	ENSG00000200162.1	ENSG00000254034.2	ENSG00000271833.1
18448	ENSG00000200163.1	ENSG00000254033.1	ENSG00000115138.11
18449	ENSG00000200164.1	ENSG00000254031.5	ENSG00000151079.7
18450	ENSG00000200168.1	ENSG00000254030.1	ENSG00000125084.11
18451	ENSG00000200169.1	ENSG00000254029.1	ENSG00000244060.2
18452	ENSG00000200170.1	ENSG00000254028.1	ENSG00000225080.1
18453	ENSG00000200171.1	ENSG00000254027.1	ENSG00000272049.1
18454	ENSG00000200174.1	ENSG00000254026.1	ENSG00000182508.14
18455	ENSG00000200175.1	ENSG00000254025.1	ENSG00000172578.12
18456	ENSG00000200176.1	ENSG00000254024.1	ENSG00000175764.14
18457	ENSG00000200179.1	ENSG00000254023.1	ENSG00000215241.3
18458	ENSG00000200183.1	ENSG00000254021.1	ENSG00000092969.12
18459	ENSG00000200184.1	ENSG00000254020.1	ENSG00000257354.2
18460	ENSG00000200189.1	ENSG00000254019.1	ENSG00000248416.1
18461	ENSG00000200191.1	ENSG00000254017.1	ENSG00000132549.18
18462	ENSG00000200197.1	ENSG00000254016.3	ENSG00000107819.13
18463	ENSG00000200198.1	ENSG00000254015.1	ENSG00000171094.18
18464	ENSG00000200201.1	ENSG00000254014.1	ENSG00000265474.1
18465	ENSG00000200204.1	ENSG00000254013.1	ENSG00000113966.10
18466	ENSG00000200206.1	ENSG00000254012.1	ENSG00000177283.7
18467	ENSG00000200208.1	ENSG00000254011.1	ENSG00000161021.13
18468	ENSG00000200209.1	ENSG00000254009.1	ENSG00000282951.1
18469	ENSG00000200211.1	ENSG00000254008.1	ENSG00000168589.15
18470	ENSG00000200213.1	ENSG00000254007.1	ENSG00000274276.4
18471	ENSG00000200215.3	ENSG00000254006.5	ENSG00000145107.15
18472	ENSG00000200216.2	ENSG00000254004.7	ENSG00000075461.6
18473	ENSG00000200217.1	ENSG00000254003.1	ENSG00000157500.12
18474	ENSG00000200218.1	ENSG00000254002.1	ENSG00000242609.1
18475	ENSG00000200220.1	ENSG00000254001.5	ENSG00000261762.1
18476	ENSG00000200222.2	ENSG00000254000.1	ENSG00000168303.8
18477	ENSG00000200224.2	ENSG00000253999.1	ENSG00000143947.13
18478	ENSG00000200225.1	ENSG00000253998.3	ENSG00000285467.1
18479	ENSG00000200227.1	ENSG00000253997.1	ENSG00000232712.6
18480	ENSG00000200231.1	ENSG00000253995.1	ENSG00000280276.1
18481	ENSG00000200235.1	ENSG00000253994.1	ENSG00000260278.1
18482	ENSG00000200237.1	ENSG00000253993.1	ENSG00000109572.13
18483	ENSG00000200238.1	ENSG00000253992.1	ENSG00000196167.9
18484	ENSG00000200241.1	ENSG00000253991.1	ENSG00000226660.2
18485	ENSG00000200243.1	ENSG00000253989.2	ENSG00000133424.20
18486	ENSG00000200246.1	ENSG00000253988.1	ENSG00000247473.2
18487	ENSG00000200247.1	ENSG00000253986.1	ENSG00000095015.6
18488	ENSG00000200248.1	ENSG00000253985.1	ENSG00000261916.1
18489	ENSG00000200250.1	ENSG00000253983.2	ENSG00000180139.11
18490	ENSG00000200252.1	ENSG00000253981.5	ENSG00000279825.1
18491	ENSG00000200253.1	ENSG00000253980.1	ENSG00000255089.1
18492	ENSG00000200254.1	ENSG00000253979.1	ENSG00000284699.1
18493	ENSG00000200256.1	ENSG00000253978.1	ENSG00000132423.12
18494	ENSG00000200257.1	ENSG00000253977.1	ENSG00000156968.9

18495	ENSG00000200259.1	ENSG00000253976.1	ENSG00000226645.1
18496	ENSG00000200261.1	ENSG00000253975.1	ENSG00000156076.10
18497	ENSG00000200262.1	ENSG00000253974.5	ENSG00000232811.1
18498	ENSG00000200267.1	ENSG00000253973.2	ENSG00000229989.3
18499	ENSG00000200269.1	ENSG00000253972.5	ENSG00000223496.3
18500	ENSG00000200274.1	ENSG00000253971.1	ENSG00000186340.15
18501	ENSG00000200275.1	ENSG00000253970.1	ENSG00000182393.3
18502	ENSG00000200278.1	ENSG00000253968.1	ENSG00000235772.1
18503	ENSG00000200279.1	ENSG00000253966.1	ENSG00000266934.1
18504	ENSG00000200281.1	ENSG00000253965.1	ENSG00000254452.1
18505	ENSG00000200283.1	ENSG00000253964.1	ENSG00000148346.12
18506	ENSG00000200287.1	ENSG00000253963.1	ENSG00000212128.2
18507	ENSG00000200288.1	ENSG00000253961.1	ENSG00000260874.5
18508	ENSG00000200291.1	ENSG00000253960.1	ENSG00000135929.9
18509	ENSG00000200293.1	ENSG00000253959.1	ENSG00000151136.15
18510	ENSG00000200294.1	ENSG00000253958.1	ENSG00000163728.11
18511	ENSG00000200295.1	ENSG00000253957.1	ENSG00000169075.7
18512	ENSG00000200296.1	ENSG00000253956.1	ENSG00000275106.1
18513	ENSG00000200298.1	ENSG00000253955.1	ENSG00000285303.1
18514	ENSG00000200301.1	ENSG00000253954.3	ENSG00000260774.1
18515	ENSG00000200303.1	ENSG00000253953.2	ENSG00000260037.5
18516	ENSG00000200304.1	ENSG00000253952.1	ENSG00000250900.6
18517	ENSG00000200305.1	ENSG00000253951.1	ENSG00000243081.2
18518	ENSG00000200309.1	ENSG00000253949.1	ENSG00000214846.4
18519	ENSG00000200310.1	ENSG00000253948.1	ENSG00000170209.4
18520	ENSG00000200312.1	ENSG00000253947.1	ENSG00000249236.2
18521	ENSG00000200313.1	ENSG00000253946.1	ENSG00000169397.3
18522	ENSG00000200314.1	ENSG00000253945.1	ENSG00000278834.1
18523	ENSG00000200318.2	ENSG00000253944.1	ENSG00000214797.3
18524	ENSG00000200320.1	ENSG00000253943.1	ENSG00000136206.4
18525	ENSG00000200325.1	ENSG00000253942.1	ENSG00000253372.5
18526	ENSG00000200326.1	ENSG00000253941.1	ENSG00000262558.1
18527	ENSG00000200327.1	ENSG00000253940.1	ENSG00000254826.1
18528	ENSG00000200331.1	ENSG00000253939.1	ENSG00000100055.21
18529	ENSG00000200332.1	ENSG00000253937.1	ENSG00000283528.2
18530	ENSG00000200334.1	ENSG00000253936.1	ENSG00000160593.18
18531	ENSG00000200336.1	ENSG00000253935.1	ENSG00000078399.18
18532	ENSG00000200338.1	ENSG00000253934.2	ENSG00000176087.15
18533	ENSG00000200340.1	ENSG00000253932.1	ENSG00000128262.8
18534	ENSG00000200343.1	ENSG00000253931.1	ENSG00000127074.14
18535	ENSG00000200344.1	ENSG00000253930.1	ENSG00000270424.1
18536	ENSG00000200345.2	ENSG00000253927.1	ENSG00000151023.17
18537	ENSG00000200350.1	ENSG00000253926.1	ENSG00000115828.17
18538	ENSG00000200351.1	ENSG00000253925.1	ENSG00000231793.5
18539	ENSG00000200354.1	ENSG00000253924.1	ENSG00000259238.1
18540	ENSG00000200355.1	ENSG00000253923.2	ENSG00000248714.6
18541	ENSG00000200356.1	ENSG00000253921.1	ENSG00000276345.1
18542	ENSG00000200360.1	ENSG00000253920.1	ENSG00000182256.13
18543	ENSG00000200361.1	ENSG00000253919.1	ENSG00000203363.2
18544	ENSG00000200366.1	ENSG00000253917.4	ENSG00000231086.1
18545	ENSG00000200367.1	ENSG00000253916.1	ENSG00000255390.1
18546	ENSG00000200369.1	ENSG00000253915.1	ENSG00000232618.1
18547	ENSG00000200370.1	ENSG00000253913.1	ENSG00000280014.1

18548	ENSG00000200372.1	ENSG00000253912.1	ENSG00000232896.1
18549	ENSG00000200376.1	ENSG00000253911.1	ENSG00000186446.12
18550	ENSG00000200377.1	ENSG00000253910.2	ENSG00000162069.15
18551	ENSG00000200378.1	ENSG00000253908.1	ENSG00000201581.1
18552	ENSG00000200379.1	ENSG00000253907.1	ENSG00000199454.1
18553	ENSG00000200381.1	ENSG00000253906.1	ENSG00000105808.17
18554	ENSG00000200385.1	ENSG00000253903.2	ENSG00000228986.1
18555	ENSG00000200388.1	ENSG00000253901.1	ENSG00000283378.1
18556	ENSG00000200389.1	ENSG00000253900.1	ENSG00000110042.8
18557	ENSG00000200390.1	ENSG00000253899.1	ENSG00000250656.2
18558	ENSG00000200391.1	ENSG00000253898.1	ENSG00000255835.1
18559	ENSG00000200393.1	ENSG00000253897.2	ENSG00000162063.13
18560	ENSG00000200394.1	ENSG00000253896.3	ENSG00000152785.7
18561	ENSG00000200397.1	ENSG00000253895.1	ENSG00000243607.3
18562	ENSG00000200398.1	ENSG00000253894.1	ENSG00000029534.20
18563	ENSG00000200403.1	ENSG00000253893.2	ENSG00000243738.3
18564	ENSG00000200406.1	ENSG00000253892.1	ENSG00000196967.10
18565	ENSG00000200407.1	ENSG00000253891.1	ENSG00000126246.10
18566	ENSG00000200408.1	ENSG00000253889.1	ENSG00000237797.1
18567	ENSG00000200411.1	ENSG00000253888.1	ENSG00000227683.1
18568	ENSG00000200413.1	ENSG00000253887.1	ENSG00000174521.7
18569	ENSG00000200418.1	ENSG00000253886.1	ENSG00000271810.5
18570	ENSG00000200419.1	ENSG00000253885.1	ENSG00000274272.1
18571	ENSG00000200421.1	ENSG00000253884.1	ENSG00000259146.3
18572	ENSG00000200422.1	ENSG00000253883.1	ENSG00000270330.1
18573	ENSG00000200424.1	ENSG00000253882.6	ENSG00000244723.3
18574	ENSG00000200427.1	ENSG00000253881.1	ENSG00000113838.13
18575	ENSG00000200428.1	ENSG00000253880.1	ENSG00000232117.1
18576	ENSG00000200431.1	ENSG00000253879.1	ENSG00000240449.1
18577	ENSG00000200432.1	ENSG00000253878.5	ENSG00000204257.15
18578	ENSG00000200434.1	ENSG00000253877.5	ENSG00000279649.1
18579	ENSG00000200436.1	ENSG00000253875.1	ENSG00000211664.3
18580	ENSG00000200437.1	ENSG00000253874.1	ENSG00000236308.1
18581	ENSG00000200443.1	ENSG00000253873.6	ENSG00000143590.14
18582	ENSG00000200444.1	ENSG00000253872.1	ENSG00000275022.1
18583	ENSG00000200446.1	ENSG00000253871.1	ENSG00000165246.14
18584	ENSG00000200448.1	ENSG00000253870.1	ENSG00000229707.1
18585	ENSG00000200455.1	ENSG00000253869.1	ENSG00000172828.13
18586	ENSG00000200456.1	ENSG00000253868.3	ENSG00000212695.5
18587	ENSG00000200462.1	ENSG00000253866.1	ENSG00000147536.12
18588	ENSG00000200463.1	ENSG00000253865.1	ENSG00000113504.21
18589	ENSG00000200468.1	ENSG00000253862.1	ENSG00000273687.1
18590	ENSG00000200469.1	ENSG00000253861.1	ENSG00000004864.13
18591	ENSG00000200471.1	ENSG00000253860.1	ENSG00000243004.5
18592	ENSG00000200472.1	ENSG00000253859.2	ENSG00000196586.14
18593	ENSG00000200473.1	ENSG00000253858.1	ENSG00000283761.1
18594	ENSG00000200475.1	ENSG00000253857.1	ENSG00000231104.8
18595	ENSG00000200478.1	ENSG00000253855.1	ENSG00000198105.14
18596	ENSG00000200480.1	ENSG00000253853.1	ENSG00000165837.11
18597	ENSG00000200483.1	ENSG00000253852.1	ENSG00000108848.16
18598	ENSG00000200484.1	ENSG00000253851.1	ENSG00000182132.13
18599	ENSG00000200485.1	ENSG00000253849.1	ENSG00000134077.16
18600	ENSG00000200486.1	ENSG00000253848.1	ENSG00000238099.2

18601	ENSG00000200487.1	ENSG00000253846.2	ENSG00000277200.1
18602	ENSG00000200488.1	ENSG00000253845.1	ENSG00000047578.13
18603	ENSG00000200492.2	ENSG00000253844.1	ENSG00000224596.7
18604	ENSG00000200494.1	ENSG00000253843.1	ENSG00000188010.14
18605	ENSG00000200495.1	ENSG00000253842.1	ENSG00000267430.1
18606	ENSG00000200496.1	ENSG00000253841.1	ENSG00000251215.1
18607	ENSG00000200502.1	ENSG00000253840.1	ENSG00000230578.3
18608	ENSG00000200503.1	ENSG00000253838.1	ENSG00000251298.1
18609	ENSG00000200506.1	ENSG00000253837.1	ENSG00000211638.2
18610	ENSG00000200508.1	ENSG00000253836.1	ENSG00000103888.17
18611	ENSG00000200516.1	ENSG00000253834.1	ENSG00000271670.1
18612	ENSG00000200520.1	ENSG00000253833.1	ENSG00000187942.11
18613	ENSG00000200521.1	ENSG00000253832.1	ENSG00000232284.7
18614	ENSG00000200522.1	ENSG00000253831.2	ENSG00000156206.14
18615	ENSG00000200525.1	ENSG00000253829.1	ENSG00000196132.13
18616	ENSG00000200526.1	ENSG00000253828.3	ENSG00000272501.1
18617	ENSG00000200527.1	ENSG00000253826.1	ENSG00000229605.5
18618	ENSG00000200528.1	ENSG00000253825.1	ENSG00000283475.1
18619	ENSG00000200530.1	ENSG00000253824.1	ENSG00000249835.2
18620	ENSG00000200534.1	ENSG00000253823.1	ENSG00000272456.1
18621	ENSG00000200536.1	ENSG00000253822.1	ENSG00000163534.15
18622	ENSG00000200537.1	ENSG00000253821.1	ENSG00000242082.2
18623	ENSG00000200538.2	ENSG00000253820.1	ENSG00000097096.9
18624	ENSG00000200544.1	ENSG00000253818.1	ENSG00000132563.16
18625	ENSG00000200545.2	ENSG00000253817.1	ENSG00000141028.6
18626	ENSG00000200547.1	ENSG00000253816.3	ENSG00000240692.3
18627	ENSG00000200550.1	ENSG00000253814.1	ENSG00000237748.1
18628	ENSG00000200552.1	ENSG00000253813.1	ENSG00000226068.1
18629	ENSG00000200553.1	ENSG00000253811.1	ENSG00000261549.2
18630	ENSG00000200554.1	ENSG00000253810.1	ENSG00000214062.5
18631	ENSG00000200555.1	ENSG00000253809.1	ENSG00000143553.10
18632	ENSG00000200556.1	ENSG00000253808.1	ENSG00000270893.1
18633	ENSG00000200558.1	ENSG00000253807.5	ENSG00000247982.6
18634	ENSG00000200560.1	ENSG00000253806.1	ENSG00000271304.2
18635	ENSG00000200563.1	ENSG00000253805.1	ENSG00000267258.1
18636	ENSG00000200564.1	ENSG00000253803.1	ENSG00000171747.9
18637	ENSG00000200566.1	ENSG00000253802.1	ENSG00000260592.1
18638	ENSG00000200570.1	ENSG00000253801.2	ENSG00000115128.7
18639	ENSG00000200571.1	ENSG00000253800.1	ENSG00000259120.3
18640	ENSG00000200572.1	ENSG00000253799.1	ENSG00000250318.1
18641	ENSG00000200575.1	ENSG00000253798.2	ENSG00000123453.18
18642	ENSG00000200579.1	ENSG00000253797.2	ENSG00000228071.4
18643	ENSG00000200587.1	ENSG00000253796.1	ENSG00000228956.8
18644	ENSG00000200591.1	ENSG00000253795.1	ENSG00000163737.3
18645	ENSG00000200593.1	ENSG00000253794.1	ENSG00000254870.5
18646	ENSG00000200594.1	ENSG00000253793.1	ENSG00000255867.1
18647	ENSG00000200597.1	ENSG00000253792.1	ENSG00000162840.4
18648	ENSG00000200600.1	ENSG00000253790.1	ENSG00000158113.13
18649	ENSG00000200601.1	ENSG00000253789.1	ENSG00000142669.15
18650	ENSG00000200605.1	ENSG00000253787.1	ENSG00000229336.1
18651	ENSG00000200608.1	ENSG00000253786.1	ENSG00000107099.15
18652	ENSG00000200610.1	ENSG00000253785.1	ENSG00000241318.3
18653	ENSG00000200612.1	ENSG00000253784.1	ENSG00000229628.1

18654	ENSG00000200613.1	ENSG00000253783.1	ENSG00000269559.2
18655	ENSG00000200615.1	ENSG00000253782.1	ENSG00000258512.1
18656	ENSG00000200616.1	ENSG00000253781.1	ENSG00000241735.2
18657	ENSG00000200619.1	ENSG00000253780.1	ENSG00000218189.4
18658	ENSG00000200620.1	ENSG00000253779.1	ENSG00000279075.1
18659	ENSG00000200622.1	ENSG00000253778.1	ENSG00000251791.1
18660	ENSG00000200623.1	ENSG00000253777.1	ENSG00000266312.1
18661	ENSG00000200624.1	ENSG00000253776.1	ENSG00000133687.16
18662	ENSG00000200626.1	ENSG00000253775.2	ENSG00000146457.16
18663	ENSG00000200629.1	ENSG00000253774.1	ENSG00000138814.17
18664	ENSG00000200630.1	ENSG00000253773.2	ENSG00000268322.1
18665	ENSG00000200632.1	ENSG00000253772.1	ENSG00000224790.2
18666	ENSG00000200635.1	ENSG00000253771.6	ENSG00000246308.1
18667	ENSG00000200636.1	ENSG00000253770.1	ENSG00000188573.7
18668	ENSG00000200637.1	ENSG00000253768.1	ENSG00000120318.16
18669	ENSG00000200638.1	ENSG00000253767.2	ENSG00000257169.1
18670	ENSG00000200645.1	ENSG00000253766.1	ENSG00000128791.12
18671	ENSG00000200646.1	ENSG00000253765.1	ENSG00000264254.1
18672	ENSG00000200648.1	ENSG00000253764.2	ENSG00000165934.12
18673	ENSG00000200650.1	ENSG00000253763.1	ENSG00000070915.9
18674	ENSG00000200651.1	ENSG00000253762.1	ENSG00000219451.3
18675	ENSG00000200652.1	ENSG00000253760.1	ENSG00000092199.17
18676	ENSG00000200653.1	ENSG00000253759.1	ENSG00000235919.4
18677	ENSG00000200656.1	ENSG00000253756.1	ENSG00000220583.1
18678	ENSG00000200661.1	ENSG00000253755.1	ENSG00000228661.1
18679	ENSG00000200664.1	ENSG00000253754.1	ENSG00000069011.16
18680	ENSG00000200665.1	ENSG00000253752.1	ENSG00000254477.2
18681	ENSG00000200670.1	ENSG00000253750.1	ENSG00000244002.1
18682	ENSG00000200673.1	ENSG00000253749.1	ENSG00000162869.16
18683	ENSG00000200674.1	ENSG00000253748.2	ENSG00000120656.11
18684	ENSG00000200677.1	ENSG00000253747.1	ENSG00000179031.8
18685	ENSG00000200680.1	ENSG00000253746.1	ENSG00000155876.5
18686	ENSG00000200681.1	ENSG00000253745.1	ENSG00000233196.2
18687	ENSG00000200683.1	ENSG00000253744.1	ENSG00000126067.12
18688	ENSG00000200685.1	ENSG00000253743.1	ENSG00000101977.21
18689	ENSG00000200686.1	ENSG00000253742.1	ENSG00000119402.17
18690	ENSG00000200687.1	ENSG00000253741.1	ENSG00000213069.3
18691	ENSG00000200688.1	ENSG00000253740.5	ENSG00000173838.12
18692	ENSG00000200693.2	ENSG00000253739.1	ENSG00000174007.8
18693	ENSG00000200701.1	ENSG00000253738.1	ENSG00000231831.1
18694	ENSG00000200702.1	ENSG00000253737.1	ENSG00000138399.18
18695	ENSG00000200703.1	ENSG00000253736.2	ENSG00000158874.11
18696	ENSG00000200706.1	ENSG00000253735.1	ENSG00000279474.1
18697	ENSG00000200708.1	ENSG00000253734.1	ENSG00000160199.14
18698	ENSG00000200709.1	ENSG00000253733.3	ENSG00000089916.17
18699	ENSG00000200711.1	ENSG00000253732.1	ENSG00000271382.1
18700	ENSG00000200713.1	ENSG00000253731.2	ENSG00000168356.11
18701	ENSG00000200714.1	ENSG00000253730.1	ENSG00000275302.2
18702	ENSG00000200718.1	ENSG00000253729.7	ENSG00000132326.12
18703	ENSG00000200719.1	ENSG00000253728.5	ENSG00000264049.1
18704	ENSG00000200720.1	ENSG00000253726.1	ENSG00000258044.1
18705	ENSG00000200726.1	ENSG00000253725.1	ENSG00000137266.14
18706	ENSG00000200728.1	ENSG00000253723.1	ENSG00000125462.17

18707	ENSG00000200731.1	ENSG00000253722.1	ENSG00000218027.2
18708	ENSG00000200732.1	ENSG00000253721.1	ENSG00000213569.4
18709	ENSG00000200733.1	ENSG00000253720.1	ENSG00000262074.6
18710	ENSG00000200737.1	ENSG00000253719.3	ENSG00000231920.1
18711	ENSG00000200738.1	ENSG00000253717.1	ENSG00000150594.6
18712	ENSG00000200741.1	ENSG00000253716.5	ENSG00000273428.3
18713	ENSG00000200742.1	ENSG00000253715.1	ENSG00000269487.1
18714	ENSG00000200745.1	ENSG00000253714.1	ENSG00000228839.5
18715	ENSG00000200750.1	ENSG00000253713.1	ENSG00000286192.1
18716	ENSG00000200752.1	ENSG00000253712.1	ENSG00000228897.1
18717	ENSG00000200753.1	ENSG00000253711.1	ENSG00000261226.1
18718	ENSG00000200754.1	ENSG00000253710.4	ENSG00000073905.8
18719	ENSG00000200755.1	ENSG00000253709.1	ENSG00000089723.10
18720	ENSG00000200756.1	ENSG00000253708.1	ENSG00000125355.15
18721	ENSG00000200757.1	ENSG00000253707.1	ENSG00000237887.1
18722	ENSG00000200759.1	ENSG00000253706.5	ENSG00000229358.3
18723	ENSG00000200761.1	ENSG00000253704.1	ENSG00000244457.2
18724	ENSG00000200763.1	ENSG00000253703.2	ENSG00000076984.17
18725	ENSG00000200764.1	ENSG00000253702.1	ENSG00000175544.13
18726	ENSG00000200769.1	ENSG00000253699.1	ENSG00000272558.1
18727	ENSG00000200774.1	ENSG00000253698.1	ENSG00000144369.13
18728	ENSG00000200779.1	ENSG00000253697.1	ENSG00000155561.15
18729	ENSG00000200783.1	ENSG00000253696.2	ENSG00000273516.1
18730	ENSG00000200785.1	ENSG00000253695.1	ENSG00000249790.2
18731	ENSG00000200786.1	ENSG00000253693.1	ENSG00000232520.1
18732	ENSG00000200788.1	ENSG00000253692.3	ENSG00000186453.13
18733	ENSG00000200789.1	ENSG00000253691.2	ENSG00000231528.2
18734	ENSG00000200790.1	ENSG00000253690.1	ENSG00000266524.3
18735	ENSG00000200792.1	ENSG00000253688.2	ENSG00000272636.4
18736	ENSG00000200794.1	ENSG00000253687.1	ENSG00000248019.2
18737	ENSG00000200795.1	ENSG00000253686.1	ENSG00000144848.10
18738	ENSG00000200796.1	ENSG00000253685.1	ENSG00000240837.3
18739	ENSG00000200799.1	ENSG00000253684.1	ENSG00000239881.1
18740	ENSG00000200800.1	ENSG00000253683.1	ENSG00000213209.2
18741	ENSG00000200801.1	ENSG00000253682.1	ENSG00000149548.15
18742	ENSG00000200806.1	ENSG00000253681.1	ENSG00000180316.12
18743	ENSG00000200807.1	ENSG00000253680.1	ENSG00000230869.1
18744	ENSG00000200812.1	ENSG00000253679.1	ENSG00000101152.11
18745	ENSG00000200814.1	ENSG00000253678.2	ENSG00000254791.1
18746	ENSG00000200815.1	ENSG00000253677.1	ENSG00000232884.7
18747	ENSG00000200816.1	ENSG00000253675.1	ENSG00000205352.11
18748	ENSG00000200817.1	ENSG00000253674.1	ENSG00000144306.14
18749	ENSG00000200818.1	ENSG00000253673.1	ENSG00000261535.1
18750	ENSG00000200822.1	ENSG00000253672.1	ENSG00000161016.17
18751	ENSG00000200823.1	ENSG00000253671.2	ENSG00000188763.4
18752	ENSG00000200827.1	ENSG00000253670.1	ENSG00000261599.6
18753	ENSG00000200829.1	ENSG00000253669.3	ENSG00000119862.13
18754	ENSG00000200830.1	ENSG00000253668.1	ENSG00000243911.3
18755	ENSG00000200831.1	ENSG00000253667.2	ENSG00000235674.2
18756	ENSG00000200832.1	ENSG00000253666.1	ENSG00000231852.8
18757	ENSG00000200834.1	ENSG00000253665.1	ENSG00000173960.14
18758	ENSG00000200839.1	ENSG00000253664.1	ENSG00000188038.8
18759	ENSG00000200840.1	ENSG00000253663.1	ENSG00000250405.2

18760	ENSG00000200842.1	ENSG00000253661.1	ENSG00000214331.8
18761	ENSG00000200847.1	ENSG00000253660.1	ENSG00000209702.1
18762	ENSG00000200849.1	ENSG00000253659.1	ENSG00000087365.15
18763	ENSG00000200852.1	ENSG00000253658.5	ENSG00000279693.1
18764	ENSG00000200855.1	ENSG00000253657.1	ENSG00000130948.10
18765	ENSG00000200857.1	ENSG00000253656.1	ENSG00000180815.14
18766	ENSG00000200860.1	ENSG00000253655.2	ENSG00000139985.6
18767	ENSG00000200867.1	ENSG00000253654.2	ENSG00000269560.1
18768	ENSG00000200869.1	ENSG00000253653.1	ENSG00000233175.2
18769	ENSG00000200871.1	ENSG00000253652.1	ENSG00000250612.2
18770	ENSG00000200872.1	ENSG00000253651.1	ENSG00000258741.3
18771	ENSG00000200873.1	ENSG00000253650.1	ENSG00000203593.3
18772	ENSG00000200874.1	ENSG00000253649.5	ENSG00000226758.1
18773	ENSG00000200875.1	ENSG00000253648.1	ENSG00000238197.5
18774	ENSG00000200877.1	ENSG00000253647.1	ENSG00000005961.18
18775	ENSG00000200879.1	ENSG00000253646.1	ENSG00000286154.1
18776	ENSG00000200882.1	ENSG00000253645.1	ENSG00000225224.1
18777	ENSG00000200883.1	ENSG00000253644.1	ENSG00000284634.1
18778	ENSG00000200884.1	ENSG00000253643.5	ENSG00000266962.2
18779	ENSG00000200885.1	ENSG00000253642.5	ENSG00000251323.2
18780	ENSG00000200887.1	ENSG00000253641.5	ENSG00000130475.14
18781	ENSG00000200888.1	ENSG00000253639.1	ENSG00000170049.9
18782	ENSG00000200889.1	ENSG00000253638.1	ENSG00000218521.1
18783	ENSG00000200890.1	ENSG00000253637.1	ENSG00000285599.1
18784	ENSG00000200891.1	ENSG00000253636.1	ENSG00000005513.10
18785	ENSG00000200893.1	ENSG00000253635.1	ENSG00000285091.1
18786	ENSG00000200895.1	ENSG00000253634.2	ENSG00000126391.14
18787	ENSG00000200897.1	ENSG00000253633.1	ENSG00000242208.1
18788	ENSG00000200898.1	ENSG00000253632.1	ENSG00000135617.4
18789	ENSG00000200902.1	ENSG00000253631.1	ENSG00000223987.1
18790	ENSG00000200903.1	ENSG00000253630.1	ENSG00000235101.1
18791	ENSG00000200906.1	ENSG00000253629.1	ENSG00000272195.1
18792	ENSG00000200913.1	ENSG00000253628.1	ENSG00000262636.1
18793	ENSG00000200914.1	ENSG00000253627.1	ENSG00000237476.1
18794	ENSG00000200917.1	ENSG00000253626.3	ENSG00000230795.3
18795	ENSG00000200922.1	ENSG00000253625.1	ENSG00000256040.2
18796	ENSG00000200924.1	ENSG00000253623.1	ENSG00000275494.1
18797	ENSG00000200926.1	ENSG00000253622.1	ENSG00000260456.6
18798	ENSG00000200935.1	ENSG00000253621.1	ENSG00000280388.1
18799	ENSG00000200941.1	ENSG00000253620.2	ENSG00000040933.15
18800	ENSG00000200942.1	ENSG00000253619.1	ENSG00000184363.10
18801	ENSG00000200949.1	ENSG00000253618.1	ENSG00000172331.12
18802	ENSG00000200953.1	ENSG00000253617.2	ENSG00000159593.15
18803	ENSG00000200957.1	ENSG00000253616.5	ENSG00000227477.1
18804	ENSG00000200959.1	ENSG00000253615.1	ENSG00000125869.10
18805	ENSG00000200963.1	ENSG00000253614.1	ENSG00000166228.9
18806	ENSG00000200966.1	ENSG00000253613.2	ENSG00000226571.2
18807	ENSG00000200969.1	ENSG00000253612.1	ENSG00000264644.1
18808	ENSG00000200972.1	ENSG00000253611.1	ENSG00000166889.14
18809	ENSG00000200974.1	ENSG00000253610.1	ENSG00000224891.1
18810	ENSG00000200975.1	ENSG00000253608.1	ENSG00000166569.8
18811	ENSG00000200976.1	ENSG00000253607.1	ENSG00000254054.2
18812	ENSG00000200982.1	ENSG00000253606.2	ENSG00000145040.4

18813	ENSG00000200983.1	ENSG00000253605.1	ENSG00000060303.5
18814	ENSG00000200985.1	ENSG00000253604.1	ENSG00000123191.14
18815	ENSG00000200986.1	ENSG00000253603.1	ENSG00000140009.18
18816	ENSG00000200987.1	ENSG00000253602.2	ENSG00000229372.1
18817	ENSG00000200991.1	ENSG00000253600.1	ENSG00000270832.1
18818	ENSG00000200997.1	ENSG00000253598.1	ENSG00000258904.1
18819	ENSG00000200998.1	ENSG00000253596.1	ENSG00000283900.1
18820	ENSG00000200999.1	ENSG00000253595.5	ENSG00000239365.2
18821	ENSG00000201000.1	ENSG00000253593.2	ENSG00000204822.7
18822	ENSG00000201001.1	ENSG00000253592.1	ENSG00000224839.1
18823	ENSG00000201003.1	ENSG00000253591.1	ENSG00000112118.19
18824	ENSG00000201006.1	ENSG00000253590.1	ENSG00000253981.5
18825	ENSG00000201009.1	ENSG00000253587.2	ENSG00000259589.2
18826	ENSG00000201010.1	ENSG00000253586.1	ENSG00000075643.6
18827	ENSG00000201012.1	ENSG00000253585.1	ENSG00000277767.1
18828	ENSG00000201013.1	ENSG00000253584.1	ENSG00000163590.14
18829	ENSG00000201014.1	ENSG00000253583.1	ENSG00000226982.4
18830	ENSG00000201015.1	ENSG00000253582.1	ENSG00000108839.12
18831	ENSG00000201016.1	ENSG00000253581.1	ENSG00000206816.1
18832	ENSG00000201021.1	ENSG00000253580.1	ENSG00000275882.1
18833	ENSG00000201023.1	ENSG00000253579.1	ENSG00000237989.1
18834	ENSG00000201025.1	ENSG00000253578.1	ENSG00000182366.9
18835	ENSG00000201026.1	ENSG00000253577.1	ENSG00000259580.1
18836	ENSG00000201027.1	ENSG00000253576.1	ENSG00000225931.3
18837	ENSG00000201028.1	ENSG00000253574.5	ENSG00000212658.1
18838	ENSG00000201031.1	ENSG00000253573.2	ENSG00000251629.6
18839	ENSG00000201032.1	ENSG00000253572.2	ENSG00000224786.2
18840	ENSG00000201033.1	ENSG00000253571.1	ENSG00000265100.1
18841	ENSG00000201034.1	ENSG00000253570.1	ENSG00000163645.15
18842	ENSG00000201035.1	ENSG00000253569.1	ENSG00000148498.16
18843	ENSG00000201036.1	ENSG00000253568.1	ENSG00000231942.3
18844	ENSG00000201041.1	ENSG00000253567.1	ENSG00000260257.2
18845	ENSG00000201042.1	ENSG00000253564.1	ENSG00000261478.1
18846	ENSG00000201044.1	ENSG00000253563.2	ENSG00000260302.2
18847	ENSG00000201047.1	ENSG00000253562.1	ENSG00000138376.11
18848	ENSG00000201048.1	ENSG00000253561.1	ENSG00000279212.1
18849	ENSG00000201050.1	ENSG00000253560.1	ENSG00000250130.1
18850	ENSG00000201059.1	ENSG00000253559.1	ENSG00000163875.15
18851	ENSG00000201065.1	ENSG00000253558.1	ENSG00000257086.1
18852	ENSG00000201066.1	ENSG00000253557.5	ENSG00000107290.14
18853	ENSG00000201070.1	ENSG00000253556.1	ENSG00000218823.2
18854	ENSG00000201071.1	ENSG00000253555.1	ENSG00000223878.1
18855	ENSG00000201074.1	ENSG00000253554.6	ENSG00000227777.1
18856	ENSG00000201075.2	ENSG00000253553.6	ENSG00000113597.18
18857	ENSG00000201076.1	ENSG00000253552.7	ENSG00000006652.14
18858	ENSG00000201077.1	ENSG00000253551.1	ENSG00000170270.5
18859	ENSG00000201078.1	ENSG00000253550.1	ENSG00000118620.13
18860	ENSG00000201084.1	ENSG00000253549.5	ENSG00000206341.7
18861	ENSG00000201085.1	ENSG00000253548.1	ENSG00000249483.1
18862	ENSG00000201086.1	ENSG00000253547.2	ENSG00000183837.9
18863	ENSG00000201088.1	ENSG00000253546.1	ENSG00000214279.13
18864	ENSG00000201095.1	ENSG00000253545.1	ENSG00000175895.4
18865	ENSG00000201096.1	ENSG00000253544.1	ENSG00000164291.16

18866	ENSG00000201097.1	ENSG00000253543.1	ENSG00000283881.1
18867	ENSG00000201098.1	ENSG00000253542.7	ENSG00000227259.2
18868	ENSG00000201102.1	ENSG00000253541.1	ENSG00000066557.6
18869	ENSG00000201104.1	ENSG00000253539.1	ENSG00000175137.11
18870	ENSG00000201109.1	ENSG00000253538.1	ENSG00000185897.6
18871	ENSG00000201113.1	ENSG00000253537.3	ENSG00000116031.9
18872	ENSG00000201114.1	ENSG00000253536.1	ENSG00000243566.6
18873	ENSG00000201118.1	ENSG00000253535.5	ENSG00000254912.2
18874	ENSG00000201119.1	ENSG00000253534.1	ENSG00000235224.1
18875	ENSG00000201121.1	ENSG00000253532.1	ENSG00000229700.1
18876	ENSG00000201129.1	ENSG00000253530.1	ENSG00000109586.12
18877	ENSG00000201133.1	ENSG00000253528.2	ENSG00000228126.1
18878	ENSG00000201134.1	ENSG00000253527.1	ENSG00000173674.11
18879	ENSG00000201135.1	ENSG00000253526.1	ENSG00000202444.1
18880	ENSG00000201136.1	ENSG00000253525.1	ENSG00000242990.2
18881	ENSG00000201140.1	ENSG00000253524.1	ENSG00000233236.1
18882	ENSG00000201142.1	ENSG00000253523.1	ENSG00000285819.1
18883	ENSG00000201143.1	ENSG00000253522.6	ENSG00000112685.14
18884	ENSG00000201145.1	ENSG00000253521.2	ENSG00000155367.15
18885	ENSG00000201148.1	ENSG00000253520.1	ENSG00000214447.4
18886	ENSG00000201151.1	ENSG00000253519.1	ENSG00000285889.1
18887	ENSG00000201153.1	ENSG00000253516.1	ENSG00000183055.5
18888	ENSG00000201155.1	ENSG00000253515.1	ENSG00000146197.9
18889	ENSG00000201157.1	ENSG00000253513.1	ENSG00000272764.1
18890	ENSG00000201160.1	ENSG00000253512.1	ENSG00000205133.11
18891	ENSG00000201161.1	ENSG00000253510.1	ENSG00000267243.6
18892	ENSG00000201162.1	ENSG00000253509.1	ENSG00000231908.1
18893	ENSG00000201164.1	ENSG00000253508.1	ENSG00000137509.11
18894	ENSG00000201165.1	ENSG00000253507.5	ENSG00000224479.6
18895	ENSG00000201168.1	ENSG00000253506.2	ENSG00000167646.13
18896	ENSG00000201170.1	ENSG00000253505.2	ENSG00000266981.1
18897	ENSG00000201176.1	ENSG00000253504.1	ENSG00000255521.1
18898	ENSG00000201178.1	ENSG00000253503.5	ENSG00000271778.1
18899	ENSG00000201179.1	ENSG00000253502.1	ENSG00000132846.6
18900	ENSG00000201180.1	ENSG00000253501.1	ENSG00000270816.5
18901	ENSG00000201182.1	ENSG00000253500.5	ENSG00000213684.4
18902	ENSG00000201183.1	ENSG00000253499.2	ENSG00000180776.15
18903	ENSG00000201184.1	ENSG00000253497.1	ENSG00000259856.1
18904	ENSG00000201185.1	ENSG00000253496.3	ENSG00000213279.2
18905	ENSG00000201186.1	ENSG00000253495.1	ENSG00000281538.1
18906	ENSG00000201196.1	ENSG00000253493.1	ENSG00000124243.17
18907	ENSG00000201198.1	ENSG00000253492.1	ENSG00000272966.1
18908	ENSG00000201201.1	ENSG00000253491.2	ENSG00000278985.1
18909	ENSG00000201207.1	ENSG00000253490.5	ENSG00000269925.1
18910	ENSG00000201208.1	ENSG00000253489.1	ENSG00000267882.2
18911	ENSG00000201209.1	ENSG00000253488.1	ENSG00000121270.15
18912	ENSG00000201210.1	ENSG00000253487.1	ENSG00000236762.1
18913	ENSG00000201216.1	ENSG00000253485.2	ENSG00000103160.12
18914	ENSG00000201217.1	ENSG00000253484.1	ENSG00000176289.4
18915	ENSG00000201218.1	ENSG00000253483.1	ENSG00000137764.20
18916	ENSG00000201221.1	ENSG00000253482.1	ENSG00000225603.3
18917	ENSG00000201223.1	ENSG00000253481.2	ENSG00000250568.1
18918	ENSG00000201228.1	ENSG00000253480.1	ENSG00000269439.5

18919	ENSG00000201229.1	ENSG00000253479.5	ENSG00000230408.3
18920	ENSG00000201231.1	ENSG00000253477.5	ENSG00000279331.1
18921	ENSG00000201239.1	ENSG00000253476.1	ENSG00000224712.12
18922	ENSG00000201240.1	ENSG00000253475.1	ENSG00000218175.2
18923	ENSG00000201241.1	ENSG00000253474.1	ENSG00000260757.1
18924	ENSG00000201242.1	ENSG00000253472.1	ENSG00000134882.15
18925	ENSG00000201243.1	ENSG00000253471.1	ENSG00000091073.19
18926	ENSG00000201245.1	ENSG00000253470.1	ENSG00000142875.19
18927	ENSG00000201247.1	ENSG00000253469.1	ENSG00000240801.1
18928	ENSG00000201253.1	ENSG00000253468.1	ENSG00000198918.8
18929	ENSG00000201255.1	ENSG00000253467.2	ENSG00000241810.1
18930	ENSG00000201260.1	ENSG00000253465.1	ENSG00000173267.14
18931	ENSG00000201263.1	ENSG00000253463.1	ENSG00000279814.1
18932	ENSG00000201264.1	ENSG00000253462.1	ENSG00000165061.15
18933	ENSG00000201270.1	ENSG00000253461.1	ENSG00000250731.1
18934	ENSG00000201271.1	ENSG00000253460.2	ENSG00000254013.1
18935	ENSG00000201273.1	ENSG00000253458.1	ENSG00000225313.5
18936	ENSG00000201274.1	ENSG00000253457.2	ENSG00000174177.13
18937	ENSG00000201277.1	ENSG00000253456.1	ENSG00000168476.12
18938	ENSG00000201279.1	ENSG00000253455.1	ENSG00000227525.4
18939	ENSG00000201282.1	ENSG00000253454.1	ENSG00000234065.2
18940	ENSG00000201285.1	ENSG00000253452.5	ENSG00000010671.15
18941	ENSG00000201287.1	ENSG00000253451.1	ENSG00000179523.4
18942	ENSG00000201288.1	ENSG00000253449.1	ENSG00000249690.1
18943	ENSG00000201289.1	ENSG00000253448.1	ENSG00000230082.1
18944	ENSG00000201291.1	ENSG00000253447.6	ENSG00000168411.14
18945	ENSG00000201292.1	ENSG00000253445.1	ENSG00000273275.1
18946	ENSG00000201294.1	ENSG00000253444.1	ENSG00000275638.1
18947	ENSG00000201296.1	ENSG00000253441.1	ENSG00000205913.6
18948	ENSG00000201297.1	ENSG00000253440.1	ENSG00000259381.2
18949	ENSG00000201298.1	ENSG00000253439.1	ENSG00000128482.16
18950	ENSG00000201300.1	ENSG00000253438.3	ENSG00000131018.23
18951	ENSG00000201301.1	ENSG00000253437.1	ENSG00000125257.15
18952	ENSG00000201302.1	ENSG00000253435.1	ENSG00000250220.1
18953	ENSG00000201308.1	ENSG00000253434.7	ENSG00000070718.12
18954	ENSG00000201309.1	ENSG00000253433.1	ENSG00000275132.1
18955	ENSG00000201311.1	ENSG00000253432.1	ENSG00000156234.7
18956	ENSG00000201312.1	ENSG00000253431.1	ENSG00000270948.1
18957	ENSG00000201314.1	ENSG00000253430.1	ENSG00000185920.15
18958	ENSG00000201315.1	ENSG00000253429.1	ENSG00000250334.5
18959	ENSG00000201316.1	ENSG00000253428.1	ENSG00000244167.1
18960	ENSG00000201317.1	ENSG00000253427.1	ENSG00000186350.11
18961	ENSG00000201318.1	ENSG00000253425.2	ENSG00000131969.15
18962	ENSG00000201321.1	ENSG00000253424.1	ENSG00000258101.2
18963	ENSG00000201324.1	ENSG00000253423.1	ENSG00000205456.11
18964	ENSG00000201325.1	ENSG00000253422.1	ENSG00000241431.1
18965	ENSG00000201326.1	ENSG00000253421.1	ENSG00000265415.1
18966	ENSG00000201329.2	ENSG00000253420.1	ENSG00000103642.12
18967	ENSG00000201330.1	ENSG00000253418.1	ENSG00000235321.1
18968	ENSG00000201331.1	ENSG00000253417.5	ENSG00000224451.2
18969	ENSG00000201339.1	ENSG00000253416.1	ENSG00000137364.5
18970	ENSG00000201340.1	ENSG00000253415.1	ENSG00000172336.5
18971	ENSG00000201341.1	ENSG00000253414.2	ENSG00000088179.9

18972	ENSG00000201342.1	ENSG00000253413.1	ENSG00000278022.1
18973	ENSG00000201343.1	ENSG00000253412.1	ENSG00000123201.14
18974	ENSG00000201346.2	ENSG00000253410.1	ENSG00000018408.14
18975	ENSG00000201347.1	ENSG00000253408.5	ENSG00000251354.3
18976	ENSG00000201348.1	ENSG00000253407.1	ENSG00000256628.3
18977	ENSG00000201351.1	ENSG00000253406.1	ENSG00000278214.1
18978	ENSG00000201354.1	ENSG00000253405.1	ENSG00000237604.1
18979	ENSG00000201355.1	ENSG00000253404.1	ENSG00000186625.14
18980	ENSG00000201356.1	ENSG00000253403.1	ENSG00000285854.1
18981	ENSG00000201358.1	ENSG00000253401.1	ENSG00000106052.13
18982	ENSG00000201361.1	ENSG00000253400.1	ENSG00000105829.13
18983	ENSG00000201363.2	ENSG00000253399.1	ENSG00000251580.2
18984	ENSG00000201364.1	ENSG00000253398.1	ENSG00000243107.1
18985	ENSG00000201365.1	ENSG00000253397.1	ENSG00000268686.1
18986	ENSG00000201367.1	ENSG00000253396.1	ENSG00000180881.19
18987	ENSG00000201368.2	ENSG00000253395.1	ENSG00000234608.7
18988	ENSG00000201370.1	ENSG00000253394.5	ENSG00000233292.1
18989	ENSG00000201371.1	ENSG00000253393.1	ENSG00000258677.2
18990	ENSG00000201372.1	ENSG00000253392.2	ENSG00000114978.18
18991	ENSG00000201376.1	ENSG00000253391.1	ENSG00000184702.20
18992	ENSG00000201377.1	ENSG00000253390.1	ENSG00000230847.4
18993	ENSG00000201379.1	ENSG00000253389.2	ENSG00000230368.2
18994	ENSG00000201382.1	ENSG00000253388.1	ENSG00000164251.5
18995	ENSG00000201384.1	ENSG00000253387.1	ENSG00000164466.13
18996	ENSG00000201386.1	ENSG00000253386.1	ENSG00000003400.14
18997	ENSG00000201388.1	ENSG00000253385.1	ENSG00000197099.8
18998	ENSG00000201390.1	ENSG00000253384.1	ENSG00000256347.1
18999	ENSG00000201392.1	ENSG00000253383.1	ENSG00000273106.1
19000	ENSG00000201393.1	ENSG00000253382.3	ENSG00000095464.9
19001	ENSG00000201394.1	ENSG00000253381.1	ENSG00000228679.1
19002	ENSG00000201395.1	ENSG00000253380.1	ENSG00000100319.12
19003	ENSG00000201398.1	ENSG00000253377.1	ENSG00000072364.13
19004	ENSG00000201403.1	ENSG00000253376.1	ENSG00000235079.1
19005	ENSG00000201405.1	ENSG00000253374.5	ENSG00000036672.16
19006	ENSG00000201407.1	ENSG00000253373.1	ENSG00000183780.13
19007	ENSG00000201410.1	ENSG00000253372.5	ENSG00000258758.1
19008	ENSG00000201412.1	ENSG00000253370.2	ENSG00000197746.14
19009	ENSG00000201413.1	ENSG00000253369.1	ENSG00000270442.1
19010	ENSG00000201415.1	ENSG00000253368.4	ENSG00000170955.10
19011	ENSG00000201420.1	ENSG00000253367.1	ENSG00000261613.2
19012	ENSG00000201421.1	ENSG00000253366.3	ENSG00000251102.1
19013	ENSG00000201423.1	ENSG00000253365.1	ENSG00000253144.1
19014	ENSG00000201426.1	ENSG00000253363.6	ENSG00000146858.8
19015	ENSG00000201428.1	ENSG00000253362.1	ENSG00000259962.1
19016	ENSG00000201431.1	ENSG00000253361.2	ENSG00000248335.1
19017	ENSG00000201432.1	ENSG00000253359.1	ENSG00000102781.14
19018	ENSG00000201433.1	ENSG00000253358.1	ENSG00000250669.1
19019	ENSG00000201435.1	ENSG00000253357.1	ENSG00000236829.9
19020	ENSG00000201436.1	ENSG00000253356.1	ENSG00000187699.10
19021	ENSG00000201439.1	ENSG00000253355.1	ENSG00000286077.1
19022	ENSG00000201440.1	ENSG00000253354.1	ENSG00000229644.6
19023	ENSG00000201441.1	ENSG00000253352.9	ENSG00000240233.3
19024	ENSG00000201442.2	ENSG00000253351.1	ENSG00000235378.2

19025	ENSG00000201443.1	ENSG00000253349.1	ENSG00000062725.10
19026	ENSG00000201444.1	ENSG00000253348.1	ENSG00000232827.2
19027	ENSG00000201447.1	ENSG00000253347.1	ENSG00000151327.12
19028	ENSG00000201448.1	ENSG00000253346.1	ENSG00000121690.11
19029	ENSG00000201451.1	ENSG00000253345.1	ENSG00000272630.1
19030	ENSG00000201452.1	ENSG00000253344.1	ENSG00000232629.9
19031	ENSG00000201457.1	ENSG00000253343.1	ENSG00000144736.14
19032	ENSG00000201458.1	ENSG00000253342.1	ENSG00000160963.14
19033	ENSG00000201464.1	ENSG00000253341.1	ENSG00000275896.5
19034	ENSG00000201465.1	ENSG00000253340.1	ENSG00000214203.4
19035	ENSG00000201466.1	ENSG00000253339.1	ENSG00000196826.7
19036	ENSG00000201467.1	ENSG00000253338.1	ENSG00000125871.14
19037	ENSG00000201469.1	ENSG00000253336.1	ENSG00000267368.1
19038	ENSG00000201470.1	ENSG00000253335.1	ENSG00000272555.1
19039	ENSG00000201474.1	ENSG00000253334.1	ENSG00000196199.14
19040	ENSG00000201476.1	ENSG00000253333.1	ENSG00000242337.5
19041	ENSG00000201482.1	ENSG00000253332.1	ENSG00000171116.7
19042	ENSG00000201483.1	ENSG00000253331.1	ENSG00000241361.8
19043	ENSG00000201487.1	ENSG00000253330.1	ENSG00000236095.1
19044	ENSG00000201489.1	ENSG00000253329.1	ENSG00000230489.1
19045	ENSG00000201491.1	ENSG00000253328.2	ENSG00000280587.1
19046	ENSG00000201492.1	ENSG00000253327.2	ENSG00000164283.13
19047	ENSG00000201493.1	ENSG00000253326.2	ENSG00000214725.9
19048	ENSG00000201496.1	ENSG00000253325.1	ENSG00000276520.1
19049	ENSG00000201498.1	ENSG00000253322.1	ENSG00000176654.12
19050	ENSG00000201499.1	ENSG00000253321.1	ENSG00000211934.3
19051	ENSG00000201500.1	ENSG00000253320.6	ENSG00000218073.1
19052	ENSG00000201501.1	ENSG00000253319.1	ENSG00000156976.17
19053	ENSG00000201502.1	ENSG00000253318.1	ENSG00000227036.7
19054	ENSG00000201510.1	ENSG00000253317.1	ENSG00000197275.14
19055	ENSG00000201511.1	ENSG00000253315.1	ENSG00000214354.3
19056	ENSG00000201512.1	ENSG00000253314.6	ENSG00000099785.10
19057	ENSG00000201516.1	ENSG00000253313.5	ENSG00000234469.4
19058	ENSG00000201517.1	ENSG00000253312.1	ENSG00000102145.14
19059	ENSG00000201518.1	ENSG00000253311.2	ENSG00000131187.9
19060	ENSG00000201519.1	ENSG00000253310.1	ENSG00000258034.1
19061	ENSG00000201523.1	ENSG00000253309.6	ENSG00000262147.1
19062	ENSG00000201524.1	ENSG00000253308.2	ENSG00000215218.4
19063	ENSG00000201527.1	ENSG00000253307.1	ENSG00000239264.8
19064	ENSG00000201529.1	ENSG00000253305.2	ENSG00000159239.13
19065	ENSG00000201532.1	ENSG00000253304.2	ENSG00000149564.12
19066	ENSG00000201533.1	ENSG00000253303.1	ENSG00000138029.14
19067	ENSG00000201535.2	ENSG00000253302.1	ENSG00000231412.2
19068	ENSG00000201541.1	ENSG00000253301.5	ENSG00000232037.3
19069	ENSG00000201542.1	ENSG00000253300.1	ENSG00000042493.16
19070	ENSG00000201544.1	ENSG00000253299.1	ENSG00000105610.5
19071	ENSG00000201545.1	ENSG00000253298.1	ENSG00000186205.13
19072	ENSG00000201547.1	ENSG00000253297.1	ENSG00000239246.3
19073	ENSG00000201548.1	ENSG00000253295.1	ENSG00000271862.1
19074	ENSG00000201549.1	ENSG00000253294.1	ENSG00000201957.1
19075	ENSG00000201550.1	ENSG00000253293.5	ENSG00000131016.17
19076	ENSG00000201554.1	ENSG00000253292.1	ENSG00000172062.16
19077	ENSG00000201555.1	ENSG00000253291.1	ENSG00000179094.16

19078	ENSG00000201557.1	ENSG00000253290.1	ENSG00000285731.1
19079	ENSG00000201558.1	ENSG00000253288.1	ENSG00000239254.1
19080	ENSG00000201560.1	ENSG00000253287.1	ENSG00000249160.4
19081	ENSG00000201563.1	ENSG00000253286.5	ENSG00000248360.7
19082	ENSG00000201564.1	ENSG00000253284.2	ENSG00000272523.1
19083	ENSG00000201565.1	ENSG00000253283.1	ENSG00000196209.12
19084	ENSG00000201566.1	ENSG00000253282.1	ENSG00000101052.12
19085	ENSG00000201567.1	ENSG00000253281.6	ENSG00000225924.2
19086	ENSG00000201569.1	ENSG00000253280.1	ENSG00000233586.1
19087	ENSG00000201570.1	ENSG00000253279.5	ENSG00000111344.11
19088	ENSG00000201573.1	ENSG00000253278.1	ENSG00000230539.1
19089	ENSG00000201574.1	ENSG00000253276.3	ENSG00000156738.17
19090	ENSG00000201579.1	ENSG00000253275.1	ENSG00000211662.2
19091	ENSG00000201581.1	ENSG00000253274.1	ENSG00000203546.7
19092	ENSG00000201583.1	ENSG00000253273.2	ENSG00000117139.17
19093	ENSG00000201584.1	ENSG00000253271.2	ENSG00000233975.1
19094	ENSG00000201586.1	ENSG00000253270.1	ENSG00000127720.8
19095	ENSG00000201588.1	ENSG00000253269.1	ENSG00000122705.17
19096	ENSG00000201591.1	ENSG00000253267.5	ENSG00000228906.1
19097	ENSG00000201592.1	ENSG00000253266.1	ENSG00000267291.1
19098	ENSG00000201594.1	ENSG00000253265.1	ENSG00000265139.1
19099	ENSG00000201595.1	ENSG00000253263.1	ENSG00000162188.6
19100	ENSG00000201596.1	ENSG00000253262.1	ENSG00000274602.5
19101	ENSG00000201598.1	ENSG00000253261.1	ENSG00000158164.7
19102	ENSG00000201600.1	ENSG00000253260.1	ENSG00000006757.12
19103	ENSG00000201602.1	ENSG00000253259.1	ENSG00000164611.13
19104	ENSG00000201604.1	ENSG00000253258.1	ENSG00000144445.17
19105	ENSG00000201607.1	ENSG00000253257.1	ENSG00000273107.1
19106	ENSG00000201608.1	ENSG00000253256.1	ENSG00000157036.13
19107	ENSG00000201609.1	ENSG00000253252.2	ENSG00000241123.1
19108	ENSG00000201610.1	ENSG00000253251.2	ENSG00000154736.6
19109	ENSG00000201612.1	ENSG00000253250.3	ENSG00000118849.10
19110	ENSG00000201613.1	ENSG00000253247.1	ENSG00000161055.4
19111	ENSG00000201616.1	ENSG00000253245.2	ENSG00000240859.2
19112	ENSG00000201618.1	ENSG00000253244.1	ENSG00000235887.2
19113	ENSG00000201619.1	ENSG00000253242.1	ENSG00000141279.16
19114	ENSG00000201620.1	ENSG00000253241.1	ENSG00000132958.17
19115	ENSG00000201622.1	ENSG00000253240.1	ENSG00000101367.9
19116	ENSG00000201623.1	ENSG00000253239.1	ENSG00000241059.2
19117	ENSG00000201624.1	ENSG00000253238.2	ENSG00000285877.1
19118	ENSG00000201627.1	ENSG00000253237.2	ENSG00000076043.10
19119	ENSG00000201628.1	ENSG00000253236.1	ENSG00000176912.3
19120	ENSG00000201633.1	ENSG00000253235.1	ENSG00000225986.1
19121	ENSG00000201634.1	ENSG00000253234.1	ENSG00000269794.1
19122	ENSG00000201635.1	ENSG00000253233.1	ENSG00000164484.11
19123	ENSG00000201638.2	ENSG00000253232.1	ENSG00000008226.19
19124	ENSG00000201640.2	ENSG00000253231.1	ENSG00000205054.7
19125	ENSG00000201641.1	ENSG00000253230.8	ENSG00000135541.21
19126	ENSG00000201642.1	ENSG00000253229.1	ENSG00000285881.1
19127	ENSG00000201643.1	ENSG00000253228.1	ENSG00000225094.3
19128	ENSG00000201644.2	ENSG00000253227.1	ENSG00000118160.14
19129	ENSG00000201648.1	ENSG00000253226.1	ENSG00000100503.23
19130	ENSG00000201649.1	ENSG00000253225.1	ENSG00000198840.2

19131	ENSG00000201658.1	ENSG00000253224.1	ENSG00000114204.14
19132	ENSG00000201659.1	ENSG00000253223.1	ENSG00000179213.13
19133	ENSG00000201660.1	ENSG00000253220.2	ENSG00000254271.1
19134	ENSG00000201662.1	ENSG00000253219.1	ENSG00000137767.14
19135	ENSG00000201663.1	ENSG00000253218.1	ENSG00000232043.1
19136	ENSG00000201665.1	ENSG00000253217.1	ENSG00000185442.13
19137	ENSG00000201666.1	ENSG00000253216.1	ENSG00000174903.16
19138	ENSG00000201668.1	ENSG00000253215.1	ENSG00000236986.6
19139	ENSG00000201671.1	ENSG00000253213.1	ENSG00000179148.9
19140	ENSG00000201672.1	ENSG00000253210.1	ENSG00000146755.10
19141	ENSG00000201675.1	ENSG00000253209.1	ENSG00000063660.9
19142	ENSG00000201676.1	ENSG00000253208.1	ENSG00000115183.15
19143	ENSG00000201679.1	ENSG00000253207.3	ENSG00000075407.18
19144	ENSG00000201680.1	ENSG00000253206.1	ENSG00000101447.15
19145	ENSG00000201683.1	ENSG00000253205.5	ENSG00000171695.10
19146	ENSG00000201684.1	ENSG00000253203.6	ENSG00000182179.13
19147	ENSG00000201687.1	ENSG00000253202.1	ENSG00000222985.1
19148	ENSG00000201689.1	ENSG00000253200.1	ENSG00000152969.19
19149	ENSG00000201690.1	ENSG00000253199.1	ENSG00000180172.7
19150	ENSG00000201695.1	ENSG00000253198.1	ENSG00000100243.21
19151	ENSG00000201699.1	ENSG00000253197.5	ENSG00000149150.9
19152	ENSG00000201700.3	ENSG00000253196.1	ENSG00000273802.2
19153	ENSG00000201701.1	ENSG00000253195.2	ENSG00000175197.12
19154	ENSG00000201704.1	ENSG00000253194.2	ENSG00000171611.9
19155	ENSG00000201707.1	ENSG00000253191.1	ENSG00000168273.8
19156	ENSG00000201708.1	ENSG00000253190.3	ENSG00000258752.1
19157	ENSG00000201709.1	ENSG00000253189.1	ENSG00000237522.1
19158	ENSG00000201710.1	ENSG00000253188.1	ENSG00000247950.6
19159	ENSG00000201711.1	ENSG00000253187.2	ENSG00000187902.11
19160	ENSG00000201713.1	ENSG00000253186.2	ENSG00000269821.1
19161	ENSG00000201715.1	ENSG00000253184.1	ENSG00000263142.5
19162	ENSG00000201723.2	ENSG00000253183.1	ENSG00000270020.1
19163	ENSG00000201724.1	ENSG00000253182.1	ENSG00000255111.1
19164	ENSG00000201725.1	ENSG00000253181.1	ENSG00000179021.10
19165	ENSG00000201727.1	ENSG00000253180.1	ENSG00000138678.11
19166	ENSG00000201728.1	ENSG00000253179.1	ENSG00000250982.2
19167	ENSG00000201733.1	ENSG00000253178.1	ENSG00000261327.4
19168	ENSG00000201736.1	ENSG00000253177.1	ENSG00000259049.1
19169	ENSG00000201737.1	ENSG00000253176.3	ENSG00000134255.14
19170	ENSG00000201741.1	ENSG00000253175.1	ENSG00000246223.8
19171	ENSG00000201742.1	ENSG00000253174.2	ENSG00000261528.2
19172	ENSG00000201744.1	ENSG00000253173.3	ENSG00000273001.1
19173	ENSG00000201746.1	ENSG00000253172.1	ENSG00000228915.3
19174	ENSG00000201747.1	ENSG00000253171.1	ENSG00000235319.1
19175	ENSG00000201749.1	ENSG00000253170.1	ENSG00000135951.15
19176	ENSG00000201752.1	ENSG00000253169.2	ENSG00000162512.16
19177	ENSG00000201754.1	ENSG00000253168.1	ENSG00000270316.1
19178	ENSG00000201756.1	ENSG00000253167.1	ENSG00000211966.2
19179	ENSG00000201758.1	ENSG00000253166.2	ENSG00000149591.16
19180	ENSG00000201761.1	ENSG00000253165.1	ENSG00000166439.6
19181	ENSG00000201763.1	ENSG00000253164.5	ENSG00000146373.16
19182	ENSG00000201766.1	ENSG00000253163.1	ENSG00000224342.1
19183	ENSG00000201770.1	ENSG00000253162.1	ENSG00000216365.2

19184	ENSG00000201772.1	ENSG00000253161.5	ENSG00000279016.1
19185	ENSG00000201774.1	ENSG00000253160.1	ENSG00000255224.1
19186	ENSG00000201775.1	ENSG00000253159.3	ENSG00000261363.2
19187	ENSG00000201778.1	ENSG00000253158.1	ENSG00000171659.15
19188	ENSG00000201780.1	ENSG00000253156.1	ENSG00000267744.1
19189	ENSG00000201782.1	ENSG00000253155.1	ENSG00000240821.1
19190	ENSG00000201785.1	ENSG00000253154.2	ENSG00000271743.1
19191	ENSG00000201786.1	ENSG00000253153.1	ENSG00000274270.1
19192	ENSG00000201788.1	ENSG00000253152.1	ENSG00000276533.1
19193	ENSG00000201789.1	ENSG00000253149.3	ENSG00000260793.2
19194	ENSG00000201790.1	ENSG00000253148.1	ENSG00000168229.3
19195	ENSG00000201791.1	ENSG00000253147.5	ENSG00000269226.7
19196	ENSG00000201793.1	ENSG00000253146.1	ENSG00000141469.18
19197	ENSG00000201794.1	ENSG00000253143.3	ENSG00000211647.1
19198	ENSG00000201796.1	ENSG00000253142.1	ENSG00000236583.1
19199	ENSG00000201800.1	ENSG00000253141.1	ENSG00000057704.13
19200	ENSG00000201801.1	ENSG00000253140.1	ENSG00000109805.10
19201	ENSG00000201805.1	ENSG00000253139.1	ENSG00000148832.16
19202	ENSG00000201806.1	ENSG00000253138.5	ENSG00000258656.1
19203	ENSG00000201807.1	ENSG00000253137.1	ENSG00000211695.2
19204	ENSG00000201809.1	ENSG00000253135.1	ENSG00000205923.3
19205	ENSG00000201810.1	ENSG00000253134.1	ENSG00000204455.7
19206	ENSG00000201811.1	ENSG00000253133.1	ENSG00000162976.13
19207	ENSG00000201812.1	ENSG00000253132.1	ENSG00000261008.6
19208	ENSG00000201813.1	ENSG00000253131.1	ENSG00000236432.7
19209	ENSG00000201815.1	ENSG00000253130.1	ENSG00000100330.15
19210	ENSG00000201816.1	ENSG00000253127.1	ENSG00000280168.1
19211	ENSG00000201818.1	ENSG00000253126.1	ENSG00000171444.18
19212	ENSG00000201820.1	ENSG00000253125.1	ENSG00000210127.1
19213	ENSG00000201821.1	ENSG00000253124.1	ENSG00000116652.6
19214	ENSG00000201822.1	ENSG00000253123.3	ENSG00000095139.14
19215	ENSG00000201823.1	ENSG00000253122.1	ENSG00000233330.1
19216	ENSG00000201825.1	ENSG00000253121.1	ENSG00000168676.11
19217	ENSG00000201826.1	ENSG00000253120.1	ENSG00000162076.13
19218	ENSG00000201827.1	ENSG00000253119.1	ENSG00000260475.1
19219	ENSG00000201830.1	ENSG00000253118.1	ENSG00000280080.2
19220	ENSG00000201831.1	ENSG00000253117.5	ENSG00000157259.8
19221	ENSG00000201839.1	ENSG00000253116.1	ENSG00000254944.1
19222	ENSG00000201843.1	ENSG00000253115.1	ENSG00000226746.2
19223	ENSG00000201846.1	ENSG00000253114.2	ENSG00000226310.1
19224	ENSG00000201847.1	ENSG00000253112.1	ENSG00000154589.6
19225	ENSG00000201850.1	ENSG00000253111.2	ENSG00000116852.14
19226	ENSG00000201852.1	ENSG00000253110.1	ENSG00000131400.8
19227	ENSG00000201856.1	ENSG00000253109.1	ENSG00000181220.17
19228	ENSG00000201860.1	ENSG00000253108.1	ENSG00000169989.2
19229	ENSG00000201861.1	ENSG00000253107.1	ENSG00000233029.3
19230	ENSG00000201863.1	ENSG00000253106.1	ENSG00000286230.1
19231	ENSG00000201867.1	ENSG00000253105.5	ENSG00000142937.12
19232	ENSG00000201868.1	ENSG00000253104.1	ENSG00000286055.1
19233	ENSG00000201869.1	ENSG00000253103.1	ENSG00000283429.1
19234	ENSG00000201875.1	ENSG00000253102.1	ENSG00000269899.1
19235	ENSG00000201876.1	ENSG00000253100.1	ENSG00000007312.12
19236	ENSG00000201881.1	ENSG00000253099.1	ENSG00000043093.14

19237	ENSG00000201882.1	ENSG00000253098.1	ENSG00000135226.17
19238	ENSG00000201884.1	ENSG00000253097.1	ENSG00000108001.13
19239	ENSG00000201885.1	ENSG00000253096.1	ENSG00000270281.1
19240	ENSG00000201892.1	ENSG00000253094.1	ENSG00000259116.2
19241	ENSG00000201894.1	ENSG00000253093.1	ENSG00000136279.20
19242	ENSG00000201896.1	ENSG00000253092.1	ENSG00000176927.15
19243	ENSG00000201898.1	ENSG00000253090.2	ENSG00000270339.3
19244	ENSG00000201899.1	ENSG00000253089.1	ENSG00000138172.11
19245	ENSG00000201900.1	ENSG00000253088.1	ENSG00000174173.7
19246	ENSG00000201901.1	ENSG00000253087.1	ENSG00000277283.1
19247	ENSG00000201907.1	ENSG00000253086.1	ENSG00000151948.12
19248	ENSG00000201909.1	ENSG00000253085.1	ENSG00000162733.18
19249	ENSG00000201910.1	ENSG00000253084.3	ENSG00000198795.11
19250	ENSG00000201912.1	ENSG00000253083.1	ENSG00000103375.11
19251	ENSG00000201913.1	ENSG00000253081.1	ENSG00000244256.3
19252	ENSG00000201916.1	ENSG00000253080.1	ENSG00000149506.11
19253	ENSG00000201919.1	ENSG00000253079.1	ENSG00000149932.17
19254	ENSG00000201920.1	ENSG00000253078.1	ENSG00000123965.13
19255	ENSG00000201922.1	ENSG00000253077.1	ENSG00000273010.1
19256	ENSG00000201923.1	ENSG00000253075.1	ENSG00000157890.17
19257	ENSG00000201925.1	ENSG00000253074.1	ENSG00000199631.1
19258	ENSG00000201931.1	ENSG00000253073.1	ENSG00000100865.15
19259	ENSG00000201933.1	ENSG00000253072.1	ENSG00000223711.1
19260	ENSG00000201938.1	ENSG00000253070.1	ENSG00000166888.12
19261	ENSG00000201939.1	ENSG00000253067.1	ENSG00000137801.10
19262	ENSG00000201942.1	ENSG00000253066.1	ENSG00000236255.1
19263	ENSG00000201943.1	ENSG00000253065.1	ENSG00000285676.1
19264	ENSG00000201944.1	ENSG00000253064.1	ENSG00000132300.19
19265	ENSG00000201945.1	ENSG00000253063.1	ENSG00000230002.3
19266	ENSG00000201950.1	ENSG00000253060.1	ENSG00000260643.2
19267	ENSG00000201954.1	ENSG00000253059.1	ENSG00000244668.1
19268	ENSG00000201955.1	ENSG00000253058.1	ENSG00000000971.15
19269	ENSG00000201957.1	ENSG00000253057.1	ENSG00000261739.2
19270	ENSG00000201959.1	ENSG00000253056.1	ENSG00000213772.3
19271	ENSG00000201962.1	ENSG00000253055.1	ENSG00000258274.1
19272	ENSG00000201965.1	ENSG00000253054.1	ENSG00000143369.15
19273	ENSG00000201966.1	ENSG00000253053.1	ENSG00000204930.9
19274	ENSG00000201967.1	ENSG00000253051.1	ENSG00000213500.3
19275	ENSG00000201968.1	ENSG00000253049.1	ENSG00000065526.11
19276	ENSG00000201969.1	ENSG00000253048.1	ENSG00000235328.1
19277	ENSG00000201980.1	ENSG00000253047.1	ENSG00000158805.12
19278	ENSG00000201984.1	ENSG00000253043.1	ENSG00000227001.3
19279	ENSG00000201987.1	ENSG00000253042.1	ENSG00000142039.4
19280	ENSG00000201988.2	ENSG00000253041.1	ENSG00000279410.1
19281	ENSG00000201990.1	ENSG00000253040.1	ENSG00000185630.18
19282	ENSG00000201992.1	ENSG00000253039.1	ENSG00000218891.5
19283	ENSG00000201998.1	ENSG00000253038.1	ENSG00000106688.12
19284	ENSG00000201999.1	ENSG00000253035.1	ENSG00000177575.12
19285	ENSG00000202000.1	ENSG00000253032.1	ENSG00000274677.1
19286	ENSG00000202001.2	ENSG00000253031.1	ENSG00000228314.1
19287	ENSG00000202008.1	ENSG00000253030.1	ENSG00000239642.6
19288	ENSG00000202014.1	ENSG00000253028.1	ENSG00000274386.5
19289	ENSG00000202016.1	ENSG00000253027.1	ENSG00000165171.11

19290	ENSG00000202017.1	ENSG00000253026.1	ENSG00000260646.1
19291	ENSG00000202019.1	ENSG00000253025.1	ENSG00000279314.1
19292	ENSG00000202021.1	ENSG00000253024.1	ENSG00000185252.19
19293	ENSG00000202023.1	ENSG00000253023.1	ENSG00000012983.11
19294	ENSG00000202024.1	ENSG00000253022.1	ENSG00000235138.1
19295	ENSG00000202025.1	ENSG00000253021.1	ENSG00000205791.3
19296	ENSG00000202026.1	ENSG00000253020.2	ENSG00000274561.1
19297	ENSG00000202027.1	ENSG00000253019.1	ENSG00000176732.6
19298	ENSG00000202029.1	ENSG00000253016.1	ENSG00000187091.13
19299	ENSG00000202031.1	ENSG00000253015.1	ENSG00000279482.1
19300	ENSG00000202034.1	ENSG00000253013.1	ENSG00000211812.1
19301	ENSG00000202035.1	ENSG00000253010.1	ENSG00000170370.12
19302	ENSG00000202039.1	ENSG00000253008.2	ENSG00000285730.1
19303	ENSG00000202041.1	ENSG00000253007.2	ENSG00000196865.4
19304	ENSG00000202044.1	ENSG00000253006.1	ENSG00000143924.19
19305	ENSG00000202046.1	ENSG00000253005.1	ENSG00000239607.3
19306	ENSG00000202047.1	ENSG00000253003.1	ENSG00000258384.1
19307	ENSG00000202048.1	ENSG00000253001.1	ENSG00000272934.1
19308	ENSG00000202050.1	ENSG00000253000.1	ENSG00000058335.15
19309	ENSG00000202051.1	ENSG00000252999.1	ENSG00000279800.2
19310	ENSG00000202054.1	ENSG00000252998.1	ENSG00000078369.18
19311	ENSG00000202056.1	ENSG00000252996.1	ENSG00000254614.2
19312	ENSG00000202058.1	ENSG00000252995.1	ENSG00000141480.18
19313	ENSG00000202059.1	ENSG00000252994.1	ENSG00000204564.12
19314	ENSG00000202060.1	ENSG00000252993.1	ENSG00000170935.7
19315	ENSG00000202063.1	ENSG00000252992.1	ENSG00000238062.5
19316	ENSG00000202069.1	ENSG00000252991.1	ENSG00000126773.13
19317	ENSG00000202070.1	ENSG00000252990.1	ENSG00000278922.1
19318	ENSG00000202071.1	ENSG00000252989.1	ENSG00000237976.1
19319	ENSG00000202074.1	ENSG00000252988.1	ENSG00000109047.7
19320	ENSG00000202078.1	ENSG00000252987.1	ENSG00000138378.19
19321	ENSG00000202079.1	ENSG00000252985.1	ENSG00000204529.4
19322	ENSG00000202081.1	ENSG00000252984.1	ENSG00000243695.1
19323	ENSG00000202082.2	ENSG00000252983.1	ENSG00000204314.12
19324	ENSG00000202089.1	ENSG00000252982.1	ENSG00000223914.2
19325	ENSG00000202092.1	ENSG00000252980.1	ENSG00000014138.9
19326	ENSG00000202093.1	ENSG00000252979.1	ENSG00000129028.9
19327	ENSG00000202095.1	ENSG00000252978.1	ENSG00000179922.5
19328	ENSG00000202099.1	ENSG00000252977.1	ENSG00000275379.1
19329	ENSG00000202100.1	ENSG00000252975.1	ENSG00000275846.1
19330	ENSG00000202103.1	ENSG00000252973.1	ENSG00000181004.10
19331	ENSG00000202111.1	ENSG00000252971.1	ENSG00000222448.1
19332	ENSG00000202112.1	ENSG00000252970.1	ENSG00000226377.1
19333	ENSG00000202119.1	ENSG00000252969.1	ENSG00000230655.3
19334	ENSG00000202124.1	ENSG00000252965.1	ENSG00000259371.2
19335	ENSG00000202125.1	ENSG00000252964.1	ENSG00000211701.2
19336	ENSG00000202137.1	ENSG00000252963.1	ENSG00000256537.4
19337	ENSG00000202141.1	ENSG00000252962.1	ENSG00000073861.3
19338	ENSG00000202142.1	ENSG00000252960.1	ENSG00000105205.7
19339	ENSG00000202144.1	ENSG00000252959.1	ENSG00000106526.10
19340	ENSG00000202146.1	ENSG00000252957.1	ENSG00000163293.12
19341	ENSG00000202147.1	ENSG00000252956.1	ENSG00000183542.5
19342	ENSG00000202150.1	ENSG00000252955.1	ENSG00000235672.1

19343	ENSG00000202151.1	ENSG00000252953.1	ENSG00000284687.1
19344	ENSG00000202157.1	ENSG00000252952.1	ENSG00000214803.3
19345	ENSG00000202160.1	ENSG00000252951.1	ENSG00000229939.1
19346	ENSG00000202164.1	ENSG00000252950.1	ENSG00000137824.16
19347	ENSG00000202167.1	ENSG00000252948.1	ENSG00000233868.1
19348	ENSG00000202169.1	ENSG00000252947.1	ENSG00000056972.19
19349	ENSG00000202172.1	ENSG00000252945.1	ENSG00000163683.12
19350	ENSG00000202174.1	ENSG00000252944.1	ENSG00000228519.3
19351	ENSG00000202175.1	ENSG00000252943.1	ENSG00000277450.1
19352	ENSG00000202177.1	ENSG00000252942.1	ENSG00000257103.8
19353	ENSG00000202182.1	ENSG00000252941.1	ENSG00000006459.11
19354	ENSG00000202183.1	ENSG00000252937.1	ENSG00000233308.1
19355	ENSG00000202184.1	ENSG00000252936.1	ENSG00000256282.1
19356	ENSG00000202186.1	ENSG00000252935.1	ENSG00000286145.1
19357	ENSG00000202187.1	ENSG00000252934.1	ENSG00000237972.1
19358	ENSG00000202188.1	ENSG00000252933.1	ENSG00000242829.1
19359	ENSG00000202189.1	ENSG00000252931.1	ENSG00000254979.5
19360	ENSG00000202190.1	ENSG00000252930.1	ENSG00000237672.1
19361	ENSG00000202191.1	ENSG00000252929.1	ENSG00000152492.15
19362	ENSG00000202193.1	ENSG00000252928.1	ENSG00000259518.1
19363	ENSG00000202195.1	ENSG00000252927.1	ENSG00000283787.1
19364	ENSG00000202198.1	ENSG00000252923.1	ENSG00000257743.8
19365	ENSG00000202199.1	ENSG00000252922.1	ENSG00000213080.3
19366	ENSG00000202200.1	ENSG00000252920.1	ENSG00000204520.13
19367	ENSG00000202205.1	ENSG00000252919.1	ENSG00000224363.2
19368	ENSG00000202206.1	ENSG00000252917.1	ENSG00000224402.2
19369	ENSG00000202211.1	ENSG00000252916.1	ENSG00000257008.6
19370	ENSG00000202215.1	ENSG00000252915.1	ENSG00000105877.18
19371	ENSG00000202216.2	ENSG00000252914.1	ENSG00000160883.11
19372	ENSG00000202217.1	ENSG00000252913.1	ENSG00000260458.3
19373	ENSG00000202222.1	ENSG00000252909.1	ENSG00000187741.15
19374	ENSG00000202224.1	ENSG00000252908.1	ENSG00000278527.1
19375	ENSG00000202225.1	ENSG00000252906.1	ENSG00000183891.6
19376	ENSG00000202227.1	ENSG00000252905.1	ENSG00000172339.10
19377	ENSG00000202229.1	ENSG00000252904.1	ENSG00000279108.1
19378	ENSG00000202231.1	ENSG00000252903.2	ENSG00000285160.1
19379	ENSG00000202233.2	ENSG00000252902.1	ENSG00000274554.1
19380	ENSG00000202237.1	ENSG00000252900.1	ENSG00000239152.1
19381	ENSG00000202239.1	ENSG00000252898.1	ENSG00000198218.11
19382	ENSG00000202240.1	ENSG00000252897.1	ENSG00000184613.10
19383	ENSG00000202241.1	ENSG00000252894.2	ENSG00000234476.2
19384	ENSG00000202242.1	ENSG00000252892.1	ENSG00000278972.1
19385	ENSG00000202245.1	ENSG00000252891.1	ENSG00000273287.2
19386	ENSG00000202248.1	ENSG00000252890.1	ENSG00000248607.1
19387	ENSG00000202249.1	ENSG00000252889.1	ENSG00000170571.12
19388	ENSG00000202251.1	ENSG00000252888.1	ENSG00000200152.1
19389	ENSG00000202252.1	ENSG00000252887.1	ENSG00000196096.3
19390	ENSG00000202254.1	ENSG00000252886.1	ENSG00000261754.2
19391	ENSG00000202255.1	ENSG00000252884.1	ENSG00000108479.11
19392	ENSG00000202257.1	ENSG00000252882.1	ENSG00000123349.14
19393	ENSG00000202259.1	ENSG00000252881.1	ENSG00000286265.1
19394	ENSG00000202260.1	ENSG00000252879.1	ENSG00000101057.16
19395	ENSG00000202261.1	ENSG00000252877.1	ENSG00000112761.20

19396	ENSG00000202263.1	ENSG00000252874.2	ENSG00000262648.1
19397	ENSG00000202264.1	ENSG00000252872.1	ENSG00000105695.15
19398	ENSG00000202265.1	ENSG00000252870.1	ENSG00000251349.3
19399	ENSG00000202268.2	ENSG00000252868.1	ENSG00000139055.7
19400	ENSG00000202269.1	ENSG00000252867.1	ENSG00000233056.2
19401	ENSG00000202270.1	ENSG00000252866.1	ENSG00000234484.1
19402	ENSG00000202272.1	ENSG00000252864.1	ENSG00000267121.5
19403	ENSG00000202273.1	ENSG00000252863.1	ENSG00000174059.16
19404	ENSG00000202275.1	ENSG00000252861.1	ENSG00000240322.3
19405	ENSG00000202276.1	ENSG00000252860.1	ENSG00000158806.14
19406	ENSG00000202279.1	ENSG00000252859.1	ENSG00000230749.4
19407	ENSG00000202281.1	ENSG00000252858.1	ENSG00000275202.1
19408	ENSG00000202283.1	ENSG00000252857.1	ENSG00000121064.12
19409	ENSG00000202285.1	ENSG00000252856.1	ENSG00000223583.1
19410	ENSG00000202290.1	ENSG00000252854.1	ENSG00000275121.1
19411	ENSG00000202293.1	ENSG00000252852.1	ENSG00000229106.1
19412	ENSG00000202296.1	ENSG00000252850.1	ENSG00000226253.1
19413	ENSG00000202297.1	ENSG00000252848.1	ENSG00000225792.1
19414	ENSG00000202300.1	ENSG00000252847.1	ENSG00000183248.12
19415	ENSG00000202304.1	ENSG00000252845.1	ENSG00000119138.4
19416	ENSG00000202306.1	ENSG00000252840.1	ENSG00000162645.13
19417	ENSG00000202308.1	ENSG00000252839.1	ENSG00000204628.11
19418	ENSG00000202309.1	ENSG00000252837.1	ENSG00000285776.1
19419	ENSG00000202310.1	ENSG00000252835.1	ENSG00000166250.12
19420	ENSG00000202313.1	ENSG00000252833.1	ENSG00000243038.1
19421	ENSG00000202314.1	ENSG00000252832.1	ENSG00000248980.1
19422	ENSG00000202317.1	ENSG00000252830.2	ENSG00000077264.15
19423	ENSG00000202318.1	ENSG00000252828.1	ENSG00000236330.1
19424	ENSG00000202322.1	ENSG00000252827.1	ENSG00000244115.1
19425	ENSG00000202324.1	ENSG00000252826.1	ENSG00000112212.12
19426	ENSG00000202329.1	ENSG00000252824.1	ENSG00000163536.12
19427	ENSG00000202331.1	ENSG00000252823.1	ENSG00000152705.8
19428	ENSG00000202332.1	ENSG00000252822.1	ENSG00000235033.7
19429	ENSG00000202334.1	ENSG00000252821.1	ENSG00000279633.1
19430	ENSG00000202335.1	ENSG00000252820.1	ENSG00000150756.14
19431	ENSG00000202336.1	ENSG00000252816.1	ENSG00000232740.1
19432	ENSG00000202339.1	ENSG00000252815.1	ENSG00000237836.5
19433	ENSG00000202341.1	ENSG00000252814.1	ENSG00000198643.7
19434	ENSG00000202343.1	ENSG00000252812.1	ENSG00000214021.16
19435	ENSG00000202344.1	ENSG00000252810.1	ENSG00000250264.1
19436	ENSG00000202345.1	ENSG00000252807.1	ENSG00000211956.2
19437	ENSG00000202347.1	ENSG00000252806.1	ENSG00000170231.15
19438	ENSG00000202350.1	ENSG00000252804.1	ENSG00000196396.10
19439	ENSG00000202351.1	ENSG00000252802.1	ENSG00000105514.8
19440	ENSG00000202354.1	ENSG00000252800.1	ENSG00000161905.12
19441	ENSG00000202356.1	ENSG00000252798.1	ENSG00000143919.15
19442	ENSG00000202357.1	ENSG00000252797.1	ENSG00000276116.2
19443	ENSG00000202358.1	ENSG00000252796.1	ENSG00000120210.8
19444	ENSG00000202360.1	ENSG00000252795.1	ENSG00000204136.10
19445	ENSG00000202361.1	ENSG00000252794.1	ENSG00000279765.3
19446	ENSG00000202363.1	ENSG00000252787.2	ENSG00000249176.1
19447	ENSG00000202368.1	ENSG00000252784.1	ENSG00000225077.3
19448	ENSG00000202373.1	ENSG00000252783.1	ENSG00000269235.1

19449	ENSG00000202374.1	ENSG00000252782.1	ENSG00000163734.4
19450	ENSG00000202377.1	ENSG00000252780.1	ENSG00000260790.1
19451	ENSG00000202379.1	ENSG00000252779.1	ENSG00000254198.1
19452	ENSG00000202380.1	ENSG00000252778.1	ENSG00000228818.1
19453	ENSG00000202382.1	ENSG00000252777.1	ENSG00000151470.13
19454	ENSG00000202383.1	ENSG00000252776.1	ENSG00000107147.13
19455	ENSG00000202385.1	ENSG00000252774.1	ENSG00000103254.10
19456	ENSG00000202386.1	ENSG00000252772.1	ENSG00000118804.8
19457	ENSG00000202388.1	ENSG00000252770.1	ENSG00000264578.1
19458	ENSG00000202389.1	ENSG00000252769.1	ENSG00000226986.4
19459	ENSG00000202392.1	ENSG00000252768.1	ENSG00000167759.13
19460	ENSG00000202395.1	ENSG00000252767.1	ENSG00000136378.15
19461	ENSG00000202398.1	ENSG00000252766.1	ENSG00000280208.1
19462	ENSG00000202399.1	ENSG00000252765.1	ENSG00000270728.1
19463	ENSG00000202400.1	ENSG00000252764.1	ENSG00000179954.16
19464	ENSG00000202402.1	ENSG00000252763.1	ENSG00000147127.8
19465	ENSG00000202406.1	ENSG00000252762.1	ENSG00000205790.1
19466	ENSG00000202407.1	ENSG00000252761.1	ENSG00000277998.1
19467	ENSG00000202408.2	ENSG00000252760.1	ENSG00000184986.11
19468	ENSG00000202410.1	ENSG00000252759.1	ENSG00000140265.12
19469	ENSG00000202411.1	ENSG00000252758.1	ENSG00000143612.20
19470	ENSG00000202412.1	ENSG00000252757.1	ENSG00000124490.14
19471	ENSG00000202414.1	ENSG00000252756.1	ENSG00000178568.15
19472	ENSG00000202415.1	ENSG00000252755.1	ENSG00000169026.12
19473	ENSG00000202417.1	ENSG00000252752.1	ENSG00000276161.1
19474	ENSG00000202422.1	ENSG00000252751.1	ENSG00000284606.1
19475	ENSG00000202423.1	ENSG00000252750.1	ENSG00000246273.7
19476	ENSG00000202427.1	ENSG00000252749.1	ENSG00000257226.1
19477	ENSG00000202428.1	ENSG00000252747.1	ENSG00000164037.16
19478	ENSG00000202429.1	ENSG00000252746.1	ENSG00000130520.10
19479	ENSG00000202430.1	ENSG00000252745.1	ENSG00000112038.18
19480	ENSG00000202431.1	ENSG00000252744.1	ENSG00000176318.8
19481	ENSG00000202433.1	ENSG00000252743.1	ENSG00000261130.5
19482	ENSG00000202434.1	ENSG00000252742.1	ENSG00000198874.13
19483	ENSG00000202438.1	ENSG00000252739.1	ENSG00000227082.2
19484	ENSG00000202440.1	ENSG00000252735.1	ENSG00000232254.1
19485	ENSG00000202441.2	ENSG00000252734.1	ENSG00000253980.1
19486	ENSG00000202444.1	ENSG00000252729.1	ENSG00000177106.16
19487	ENSG00000202445.1	ENSG00000252727.1	ENSG00000138036.18
19488	ENSG00000202449.1	ENSG00000252726.1	ENSG00000227917.2
19489	ENSG00000202459.1	ENSG00000252725.1	ENSG00000165025.15
19490	ENSG00000202461.1	ENSG00000252724.1	ENSG00000213592.4
19491	ENSG00000202468.1	ENSG00000252723.2	ENSG00000249464.5
19492	ENSG00000202469.1	ENSG00000252722.1	ENSG00000154710.17
19493	ENSG00000202470.1	ENSG00000252721.1	ENSG00000254509.1
19494	ENSG00000202471.1	ENSG00000252720.1	ENSG00000135750.14
19495	ENSG00000202472.1	ENSG00000252719.1	ENSG00000205903.7
19496	ENSG00000202473.1	ENSG00000252718.1	ENSG00000197815.4
19497	ENSG00000202474.1	ENSG00000252717.1	ENSG00000254560.5
19498	ENSG00000202476.1	ENSG00000252716.1	ENSG00000215533.8
19499	ENSG00000202478.1	ENSG00000252714.1	ENSG00000272824.1
19500	ENSG00000202479.1	ENSG00000252713.1	ENSG00000238083.7
19501	ENSG00000202485.1	ENSG00000252712.1	ENSG00000167920.8

19502	ENSG00000202490.1	ENSG00000252711.1	ENSG00000233494.1
19503	ENSG00000202491.1	ENSG00000252710.1	ENSG00000254793.1
19504	ENSG00000202495.1	ENSG00000252707.1	ENSG00000189253.7
19505	ENSG00000202497.1	ENSG00000252705.1	ENSG00000101596.15
19506	ENSG00000202498.1	ENSG00000252704.1	ENSG00000237575.4
19507	ENSG00000202499.1	ENSG00000252700.1	ENSG00000256706.1
19508	ENSG00000202502.1	ENSG00000252699.1	ENSG00000102225.16
19509	ENSG00000202503.1	ENSG00000252697.1	ENSG00000160305.18
19510	ENSG00000202508.1	ENSG00000252696.1	ENSG00000115904.12
19511	ENSG00000202512.1	ENSG00000252695.1	ENSG00000197054.11
19512	ENSG00000202514.1	ENSG00000252693.1	ENSG00000143921.8
19513	ENSG00000202515.1	ENSG00000252692.1	ENSG00000101421.4
19514	ENSG00000202517.1	ENSG00000252691.1	ENSG00000235440.1
19515	ENSG00000202521.1	ENSG00000252690.3	ENSG00000006638.11
19516	ENSG00000202522.1	ENSG00000252689.1	ENSG00000173153.14
19517	ENSG00000202523.1	ENSG00000252688.1	ENSG00000269374.1
19518	ENSG00000202526.1	ENSG00000252686.1	ENSG00000129055.12
19519	ENSG00000202528.1	ENSG00000252685.1	ENSG00000103671.9
19520	ENSG00000202529.1	ENSG00000252684.1	ENSG00000273165.1
19521	ENSG00000202532.1	ENSG00000252682.1	ENSG00000167705.12
19522	ENSG00000202533.1	ENSG00000252681.1	ENSG00000258560.1
19523	ENSG00000202534.1	ENSG00000252680.1	ENSG00000156875.14
19524	ENSG00000202536.2	ENSG00000252677.1	ENSG00000259709.1
19525	ENSG00000202537.1	ENSG00000252674.1	ENSG00000263326.1
19526	ENSG00000202538.1	ENSG00000252673.1	ENSG00000104325.7
19527	ENSG00000202542.1	ENSG00000252671.1	ENSG00000273765.1
19528	ENSG00000202560.4	ENSG00000252667.1	ENSG00000242999.3
19529	ENSG00000202566.4	ENSG00000252661.1	ENSG00000141577.14
19530	ENSG00000202569.4	ENSG00000252660.1	ENSG00000257350.1
19531	ENSG00000202601.4	ENSG00000252659.1	ENSG00000101335.10
19532	ENSG00000202609.4	ENSG00000252658.1	ENSG00000204650.14
19533	ENSG00000203258.3	ENSG00000252657.1	ENSG00000227436.1
19534	ENSG00000203262.3	ENSG00000252656.1	ENSG00000279549.1
19535	ENSG00000203266.3	ENSG00000252655.1	ENSG00000171180.2
19536	ENSG00000203279.3	ENSG00000252654.1	ENSG00000271377.1
19537	ENSG00000203280.4	ENSG00000252653.1	ENSG00000108958.4
19538	ENSG00000203286.5	ENSG00000252652.1	ENSG00000234282.1
19539	ENSG00000203288.3	ENSG00000252651.1	ENSG00000111229.16
19540	ENSG00000203307.2	ENSG00000252650.1	ENSG00000232909.1
19541	ENSG00000203321.2	ENSG00000252649.1	ENSG00000229124.6
19542	ENSG00000203325.3	ENSG00000252647.1	ENSG00000100226.16
19543	ENSG00000203326.11	ENSG00000252645.1	ENSG00000260773.1
19544	ENSG00000203327.2	ENSG00000252644.1	ENSG00000231652.2
19545	ENSG00000203334.4	ENSG00000252643.1	ENSG00000252355.1
19546	ENSG00000203335.4	ENSG00000252642.1	ENSG00000262410.1
19547	ENSG00000203356.2	ENSG00000252641.2	ENSG00000228692.2
19548	ENSG00000203362.2	ENSG00000252640.1	ENSG00000169218.14
19549	ENSG00000203363.2	ENSG00000252639.1	ENSG00000233913.7
19550	ENSG00000203364.2	ENSG00000252637.1	ENSG00000276471.1
19551	ENSG00000203387.2	ENSG00000252636.1	ENSG00000161082.13
19552	ENSG00000203392.3	ENSG00000252635.1	ENSG00000117791.16
19553	ENSG00000203395.2	ENSG00000252634.1	ENSG00000150636.17
19554	ENSG00000203396.3	ENSG00000252633.1	ENSG00000239523.5

19555	ENSG00000203397.2	ENSG00000252628.2	ENSG00000242992.3
19556	ENSG00000203402.2	ENSG00000252627.1	ENSG00000259105.1
19557	ENSG00000203408.4	ENSG00000252626.1	ENSG00000274979.1
19558	ENSG00000203411.3	ENSG00000252625.1	ENSG00000214784.4
19559	ENSG00000203413.3	ENSG00000252624.1	ENSG00000142173.15
19560	ENSG00000203414.2	ENSG00000252623.1	ENSG00000075073.14
19561	ENSG00000203416.3	ENSG00000252622.1	ENSG00000214185.3
19562	ENSG00000203434.2	ENSG00000252621.1	ENSG00000269656.1
19563	ENSG00000203435.2	ENSG00000252620.1	ENSG00000285554.1
19564	ENSG00000203436.2	ENSG00000252619.1	ENSG00000205955.4
19565	ENSG00000203437.3	ENSG00000252618.1	ENSG00000272885.1
19566	ENSG00000203441.2	ENSG00000252615.1	ENSG00000236946.2
19567	ENSG00000203446.2	ENSG00000252614.1	ENSG00000038210.13
19568	ENSG00000203462.2	ENSG00000252612.1	ENSG00000168101.14
19569	ENSG00000203469.2	ENSG00000252611.1	ENSG00000205593.12
19570	ENSG00000203472.3	ENSG00000252608.1	ENSG00000232626.3
19571	ENSG00000203485.13	ENSG00000252607.1	ENSG00000167632.15
19572	ENSG00000203489.3	ENSG00000252606.1	ENSG00000285560.1
19573	ENSG00000203492.4	ENSG00000252604.1	ENSG00000258645.2
19574	ENSG00000203496.9	ENSG00000252603.1	ENSG00000108785.7
19575	ENSG00000203497.2	ENSG00000252601.1	ENSG00000204420.9
19576	ENSG00000203498.2	ENSG00000252599.1	ENSG00000183479.12
19577	ENSG00000203499.11	ENSG00000252598.1	ENSG00000067715.14
19578	ENSG00000203506.5	ENSG00000252597.1	ENSG00000105650.22
19579	ENSG00000203520.3	ENSG00000252595.1	ENSG00000147044.21
19580	ENSG00000203523.3	ENSG00000252594.1	ENSG00000233247.3
19581	ENSG00000203527.2	ENSG00000252593.1	ENSG00000225171.2
19582	ENSG00000203531.3	ENSG00000252591.1	ENSG00000206535.8
19583	ENSG00000203546.7	ENSG00000252590.1	ENSG00000151491.14
19584	ENSG00000203560.3	ENSG00000252587.1	ENSG00000284931.1
19585	ENSG00000203565.2	ENSG00000252585.1	ENSG00000180869.4
19586	ENSG00000203573.4	ENSG00000252583.2	ENSG00000151552.12
19587	ENSG00000203585.4	ENSG00000252582.1	ENSG00000263917.1
19588	ENSG00000203588.3	ENSG00000252581.1	ENSG00000267152.1
19589	ENSG00000203593.3	ENSG00000252580.1	ENSG00000087995.15
19590	ENSG00000203601.3	ENSG00000252578.1	ENSG00000284471.1
19591	ENSG00000203605.3	ENSG00000252577.1	ENSG00000138308.5
19592	ENSG00000203616.2	ENSG00000252574.2	ENSG00000225938.1
19593	ENSG00000203618.5	ENSG00000252569.1	ENSG00000176177.9
19594	ENSG00000203620.2	ENSG00000252568.1	ENSG00000164663.14
19595	ENSG00000203635.2	ENSG00000252563.1	ENSG00000006007.12
19596	ENSG00000203643.3	ENSG00000252562.1	ENSG00000040275.17
19597	ENSG00000203644.3	ENSG00000252561.1	ENSG00000260891.1
19598	ENSG00000203645.2	ENSG00000252560.1	ENSG00000197320.5
19599	ENSG00000203647.2	ENSG00000252558.1	ENSG00000174939.11
19600	ENSG00000203648.3	ENSG00000252556.1	ENSG00000013561.18
19601	ENSG00000203650.8	ENSG00000252555.1	ENSG00000277130.1
19602	ENSG00000203661.4	ENSG00000252554.1	ENSG00000168461.13
19603	ENSG00000203663.4	ENSG00000252553.1	ENSG00000259605.3
19604	ENSG00000203664.7	ENSG00000252552.1	ENSG00000226839.1
19605	ENSG00000203666.12	ENSG00000252550.1	ENSG00000214359.3
19606	ENSG00000203667.10	ENSG00000252549.1	ENSG00000174099.11
19607	ENSG00000203668.2	ENSG00000252548.1	ENSG00000226715.3

19608	ENSG00000203684.5	ENSG00000252546.1	ENSG00000224635.1
19609	ENSG00000203685.10	ENSG00000252545.1	ENSG00000272902.2
19610	ENSG00000203688.5	ENSG00000252544.1	ENSG00000213918.10
19611	ENSG00000203690.12	ENSG00000252542.1	ENSG00000072415.8
19612	ENSG00000203697.11	ENSG00000252540.1	ENSG00000269906.1
19613	ENSG00000203705.11	ENSG00000252539.1	ENSG00000251409.1
19614	ENSG00000203706.8	ENSG00000252537.1	ENSG00000274964.1
19615	ENSG00000203709.11	ENSG00000252535.1	ENSG00000258565.1
19616	ENSG00000203710.11	ENSG00000252534.1	ENSG00000276900.1
19617	ENSG00000203711.13	ENSG00000252533.1	ENSG00000197566.10
19618	ENSG00000203721.6	ENSG00000252532.1	ENSG00000133328.4
19619	ENSG00000203722.8	ENSG00000252530.1	ENSG00000180979.10
19620	ENSG00000203724.11	ENSG00000252526.1	ENSG00000281392.1
19621	ENSG00000203727.4	ENSG00000252524.1	ENSG00000272510.1
19622	ENSG00000203729.8	ENSG00000252523.1	ENSG00000271614.1
19623	ENSG00000203730.2	ENSG00000252521.1	ENSG00000273017.1
19624	ENSG00000203733.5	ENSG00000252519.1	ENSG00000116120.10
19625	ENSG00000203734.11	ENSG00000252517.1	ENSG00000278763.1
19626	ENSG00000203737.3	ENSG00000252516.1	ENSG00000124831.19
19627	ENSG00000203739.3	ENSG00000252515.2	ENSG00000175161.13
19628	ENSG00000203740.3	ENSG00000252514.1	ENSG00000150281.6
19629	ENSG00000203747.11	ENSG00000252513.1	ENSG00000122824.11
19630	ENSG00000203756.7	ENSG00000252512.1	ENSG00000171223.6
19631	ENSG00000203757.2	ENSG00000252510.1	ENSG00000203472.3
19632	ENSG00000203758.4	ENSG00000252509.1	ENSG00000230537.1
19633	ENSG00000203760.8	ENSG00000252508.1	ENSG00000107968.10
19634	ENSG00000203761.5	ENSG00000252507.1	ENSG00000239388.8
19635	ENSG00000203772.7	ENSG00000252506.1	ENSG00000160767.21
19636	ENSG00000203778.8	ENSG00000252505.1	ENSG00000196981.3
19637	ENSG00000203780.10	ENSG00000252503.1	ENSG00000272277.1
19638	ENSG00000203781.4	ENSG00000252501.1	ENSG00000100296.13
19639	ENSG00000203782.6	ENSG00000252499.1	ENSG00000271344.1
19640	ENSG00000203783.5	ENSG00000252498.1	ENSG00000223922.1
19641	ENSG00000203784.3	ENSG00000252497.1	ENSG00000286044.1
19642	ENSG00000203785.9	ENSG00000252496.1	ENSG00000265752.2
19643	ENSG00000203786.6	ENSG00000252494.1	ENSG00000170619.10
19644	ENSG00000203791.15	ENSG00000252491.1	ENSG00000127483.18
19645	ENSG00000203795.2	ENSG00000252490.1	ENSG00000275895.7
19646	ENSG00000203797.10	ENSG00000252489.1	ENSG00000227678.7
19647	ENSG00000203799.13	ENSG00000252487.1	ENSG00000273454.1
19648	ENSG00000203801.8	ENSG00000252486.1	ENSG00000169504.15
19649	ENSG00000203804.4	ENSG00000252485.1	ENSG00000182648.12
19650	ENSG00000203805.10	ENSG00000252484.1	ENSG00000170891.11
19651	ENSG00000203808.11	ENSG00000252483.1	ENSG00000233448.2
19652	ENSG00000203809.6	ENSG00000252482.1	ENSG00000267838.2
19653	ENSG00000203811.1	ENSG00000252481.1	ENSG00000272595.2
19654	ENSG00000203812.2	ENSG00000252480.1	ENSG00000100614.18
19655	ENSG00000203814.6	ENSG00000252479.1	ENSG00000151883.18
19656	ENSG00000203818.7	ENSG00000252475.1	ENSG00000139631.18
19657	ENSG00000203825.4	ENSG00000252474.1	ENSG00000173442.13
19658	ENSG00000203837.5	ENSG00000252473.1	ENSG00000271268.1
19659	ENSG00000203852.3	ENSG00000252472.1	ENSG00000272146.5
19660	ENSG00000203855.7	ENSG00000252470.1	ENSG00000106993.12

19661	ENSG00000203857.10	ENSG00000252469.1	ENSG00000284648.1
19662	ENSG00000203858.3	ENSG00000252468.1	ENSG00000212293.1
19663	ENSG00000203859.10	ENSG00000252467.1	ENSG00000236085.1
19664	ENSG00000203864.3	ENSG00000252466.1	ENSG00000241983.3
19665	ENSG00000203865.9	ENSG00000252464.1	ENSG00000241438.1
19666	ENSG00000203867.8	ENSG00000252462.1	ENSG00000188263.10
19667	ENSG00000203870.5	ENSG00000252461.1	ENSG00000139112.11
19668	ENSG00000203872.7	ENSG00000252460.1	ENSG00000167414.4
19669	ENSG00000203875.11	ENSG00000252459.1	ENSG00000285732.1
19670	ENSG00000203876.9	ENSG00000252458.1	ENSG00000162711.17
19671	ENSG00000203877.8	ENSG00000252457.1	ENSG00000137563.12
19672	ENSG00000203878.11	ENSG00000252456.1	ENSG00000275229.1
19673	ENSG00000203879.12	ENSG00000252454.3	ENSG00000274825.1
19674	ENSG00000203880.12	ENSG00000252452.1	ENSG00000270696.1
19675	ENSG00000203883.7	ENSG00000252451.1	ENSG00000225125.2
19676	ENSG00000203896.10	ENSG00000252450.1	ENSG00000241360.2
19677	ENSG00000203897.3	ENSG00000252449.1	ENSG00000232687.1
19678	ENSG00000203900.2	ENSG00000252448.1	ENSG00000124615.20
19679	ENSG00000203907.9	ENSG00000252446.1	ENSG00000229320.3
19680	ENSG00000203908.4	ENSG00000252444.1	ENSG00000109667.12
19681	ENSG00000203909.4	ENSG00000252443.1	ENSG00000272008.1
19682	ENSG00000203910.9	ENSG00000252441.1	ENSG00000267607.1
19683	ENSG00000203914.4	ENSG00000252438.1	ENSG00000284837.1
19684	ENSG00000203923.4	ENSG00000252437.1	ENSG00000141485.16
19685	ENSG00000203926.5	ENSG00000252436.1	ENSG00000214273.4
19686	ENSG00000203930.11	ENSG00000252433.1	ENSG00000204851.6
19687	ENSG00000203933.2	ENSG00000252431.1	ENSG00000267543.1
19688	ENSG00000203942.4	ENSG00000252429.2	ENSG00000139620.12
19689	ENSG00000203943.8	ENSG00000252428.1	ENSG00000233223.2
19690	ENSG00000203950.6	ENSG00000252427.1	ENSG00000278263.2
19691	ENSG00000203952.9	ENSG00000252426.1	ENSG00000176148.16
19692	ENSG00000203963.11	ENSG00000252425.1	ENSG00000203999.8
19693	ENSG00000203965.13	ENSG00000252424.1	ENSG00000272719.1
19694	ENSG00000203970.3	ENSG00000252423.1	ENSG00000239532.1
19695	ENSG00000203971.2	ENSG00000252422.1	ENSG00000270962.1
19696	ENSG00000203972.10	ENSG00000252421.1	ENSG00000225195.2
19697	ENSG00000203985.11	ENSG00000252420.1	ENSG00000255148.2
19698	ENSG00000203987.2	ENSG00000252417.1	ENSG00000141258.13
19699	ENSG00000203989.4	ENSG00000252416.1	ENSG00000108100.18
19700	ENSG00000203993.4	ENSG00000252415.1	ENSG00000134186.12
19701	ENSG00000203995.9	ENSG00000252414.1	ENSG00000219928.2
19702	ENSG00000203999.8	ENSG00000252413.1	ENSG00000140044.13
19703	ENSG00000204001.9	ENSG00000252412.1	ENSG00000276289.4
19704	ENSG00000204003.8	ENSG00000252411.1	ENSG00000198838.13
19705	ENSG00000204006.9	ENSG00000252410.1	ENSG00000248697.1
19706	ENSG00000204007.6	ENSG00000252409.1	ENSG00000181894.15
19707	ENSG00000204010.3	ENSG00000252408.1	ENSG00000111554.14
19708	ENSG00000204011.4	ENSG00000252407.1	ENSG00000233122.2
19709	ENSG00000204019.5	ENSG00000252404.1	ENSG00000204671.1
19710	ENSG00000204020.5	ENSG00000252401.1	ENSG00000235706.7
19711	ENSG00000204021.4	ENSG00000252400.1	ENSG00000135046.14
19712	ENSG00000204022.9	ENSG00000252398.1	ENSG00000196366.3
19713	ENSG00000204025.7	ENSG00000252397.1	ENSG00000010438.16

19714	ENSG00000204031.3	ENSG00000252396.1	ENSG00000125968.9
19715	ENSG00000204033.9	ENSG00000252395.2	ENSG00000204613.11
19716	ENSG00000204044.6	ENSG00000252393.1	ENSG00000123353.10
19717	ENSG00000204049.1	ENSG00000252391.1	ENSG00000279119.1
19718	ENSG00000204052.4	ENSG00000252390.1	ENSG00000272338.2
19719	ENSG00000204053.6	ENSG00000252386.1	ENSG00000230979.3
19720	ENSG00000204054.13	ENSG00000252385.1	ENSG00000241693.2
19721	ENSG00000204055.4	ENSG00000252383.1	ENSG00000278932.3
19722	ENSG00000204060.7	ENSG00000252377.1	ENSG00000284685.1
19723	ENSG00000204065.3	ENSG00000252376.1	ENSG00000232104.3
19724	ENSG00000204070.10	ENSG00000252374.1	ENSG00000137975.8
19725	ENSG00000204071.10	ENSG00000252373.1	ENSG00000197622.13
19726	ENSG00000204072.3	ENSG00000252371.1	ENSG00000213613.2
19727	ENSG00000204084.13	ENSG00000252370.1	ENSG00000066379.15
19728	ENSG00000204086.4	ENSG00000252369.1	ENSG00000137747.16
19729	ENSG00000204091.7	ENSG00000252368.1	ENSG00000241233.3
19730	ENSG00000204092.2	ENSG00000252367.1	ENSG00000205771.6
19731	ENSG00000204099.11	ENSG00000252366.1	ENSG00000178913.7
19732	ENSG00000204103.4	ENSG00000252364.1	ENSG00000236901.6
19733	ENSG00000204104.12	ENSG00000252363.1	ENSG00000223735.1
19734	ENSG00000204110.6	ENSG00000252362.1	ENSG00000233912.1
19735	ENSG00000204113.6	ENSG00000252361.1	ENSG00000285781.1
19736	ENSG00000204116.11	ENSG00000252358.1	ENSG00000111669.15
19737	ENSG00000204117.1	ENSG00000252357.1	ENSG00000256271.1
19738	ENSG00000204118.2	ENSG00000252355.1	ENSG00000213432.2
19739	ENSG00000204120.14	ENSG00000252353.1	ENSG00000254952.1
19740	ENSG00000204121.2	ENSG00000252352.1	ENSG00000283317.1
19741	ENSG00000204128.6	ENSG00000252351.1	ENSG00000279865.1
19742	ENSG00000204130.13	ENSG00000252350.1	ENSG00000245275.7
19743	ENSG00000204131.9	ENSG00000252349.1	ENSG00000200737.1
19744	ENSG00000204136.10	ENSG00000252348.1	ENSG00000275139.1
19745	ENSG00000204138.13	ENSG00000252347.1	ENSG00000227191.8
19746	ENSG00000204140.10	ENSG00000252346.1	ENSG00000215063.3
19747	ENSG00000204147.10	ENSG00000252343.1	ENSG00000229776.1
19748	ENSG00000204148.3	ENSG00000252342.1	ENSG00000132357.14
19749	ENSG00000204149.11	ENSG00000252341.1	ENSG00000213296.4
19750	ENSG00000204152.11	ENSG00000252339.1	ENSG00000179583.19
19751	ENSG00000204160.12	ENSG00000252338.1	ENSG00000186638.16
19752	ENSG00000204161.14	ENSG00000252337.1	ENSG00000100897.17
19753	ENSG00000204165.5	ENSG00000252336.1	ENSG00000250614.1
19754	ENSG00000204172.12	ENSG00000252335.1	ENSG00000129514.6
19755	ENSG00000204173.11	ENSG00000252334.1	ENSG00000004455.16
19756	ENSG00000204174.7	ENSG00000252333.1	ENSG00000183060.15
19757	ENSG00000204175.5	ENSG00000252332.1	ENSG00000184659.5
19758	ENSG00000204176.13	ENSG00000252329.1	ENSG00000205560.12
19759	ENSG00000204177.10	ENSG00000252328.1	ENSG00000225017.1
19760	ENSG00000204178.11	ENSG00000252326.1	ENSG00000239628.1
19761	ENSG00000204179.10	ENSG00000252325.1	ENSG00000186115.13
19762	ENSG00000204183.2	ENSG00000252323.1	ENSG00000171595.14
19763	ENSG00000204186.9	ENSG00000252322.1	ENSG00000211799.3
19764	ENSG00000204188.7	ENSG00000252321.1	ENSG00000245748.1
19765	ENSG00000204193.10	ENSG00000252320.1	ENSG00000224195.1
19766	ENSG00000204194.1	ENSG00000252319.1	ENSG00000233230.1

19767	ENSG00000204195.3	ENSG00000252317.1	ENSG00000104883.8
19768	ENSG00000204196.5	ENSG00000252316.1	ENSG00000188313.13
19769	ENSG00000204209.13	ENSG00000252315.1	ENSG00000227034.1
19770	ENSG00000204217.13	ENSG00000252313.1	ENSG00000162068.1
19771	ENSG00000204219.10	ENSG00000252312.1	ENSG00000111860.14
19772	ENSG00000204220.11	ENSG00000252311.1	ENSG00000272414.6
19773	ENSG00000204227.5	ENSG00000252307.1	ENSG00000121900.19
19774	ENSG00000204228.4	ENSG00000252305.1	ENSG00000273123.1
19775	ENSG00000204231.10	ENSG00000252302.1	ENSG00000272644.1
19776	ENSG00000204237.5	ENSG00000252301.1	ENSG00000232010.1
19777	ENSG00000204241.7	ENSG00000252297.1	ENSG00000254317.1
19778	ENSG00000204246.3	ENSG00000252296.2	ENSG00000279267.1
19779	ENSG00000204248.10	ENSG00000252294.1	ENSG00000131183.11
19780	ENSG00000204250.3	ENSG00000252292.1	ENSG00000273055.1
19781	ENSG00000204252.14	ENSG00000252291.1	ENSG00000172940.12
19782	ENSG00000204253.4	ENSG00000252290.1	ENSG00000123500.10
19783	ENSG00000204256.13	ENSG00000252289.1	ENSG00000205755.11
19784	ENSG00000204257.15	ENSG00000252288.1	ENSG00000248196.1
19785	ENSG00000204261.8	ENSG00000252287.1	ENSG00000230459.3
19786	ENSG00000204262.13	ENSG00000252284.1	ENSG00000167772.12
19787	ENSG00000204264.10	ENSG00000252283.1	ENSG00000237442.3
19788	ENSG00000204267.14	ENSG00000252282.1	ENSG00000259024.6
19789	ENSG00000204271.13	ENSG00000252279.1	ENSG00000134757.5
19790	ENSG00000204272.12	ENSG00000252277.1	ENSG00000271500.1
19791	ENSG00000204277.1	ENSG00000252275.1	ENSG00000224429.7
19792	ENSG00000204278.12	ENSG00000252274.1	ENSG00000272754.1
19793	ENSG00000204279.7	ENSG00000252273.1	ENSG00000124459.12
19794	ENSG00000204282.4	ENSG00000252272.1	ENSG00000104331.9
19795	ENSG00000204283.3	ENSG00000252271.1	ENSG00000259135.1
19796	ENSG00000204287.14	ENSG00000252269.1	ENSG00000188996.4
19797	ENSG00000204290.10	ENSG00000252268.1	ENSG00000279057.1
19798	ENSG00000204291.11	ENSG00000252267.1	ENSG00000121552.4
19799	ENSG00000204296.11	ENSG00000252266.1	ENSG00000232938.2
19800	ENSG00000204300.7	ENSG00000252264.1	ENSG00000273129.1
19801	ENSG00000204301.6	ENSG00000252263.1	ENSG00000211967.3
19802	ENSG00000204304.12	ENSG00000252262.1	ENSG00000257906.2
19803	ENSG00000204305.14	ENSG00000252261.1	ENSG00000137802.14
19804	ENSG00000204308.8	ENSG00000252260.1	ENSG00000211596.3
19805	ENSG00000204310.13	ENSG00000252259.1	ENSG00000082684.15
19806	ENSG00000204311.13	ENSG00000252258.1	ENSG00000185798.8
19807	ENSG00000204314.12	ENSG00000252257.1	ENSG00000011590.14
19808	ENSG00000204315.4	ENSG00000252255.1	ENSG00000242689.3
19809	ENSG00000204316.12	ENSG00000252254.1	ENSG00000142327.13
19810	ENSG00000204323.5	ENSG00000252252.1	ENSG00000241984.2
19811	ENSG00000204334.7	ENSG00000252250.1	ENSG00000264044.1
19812	ENSG00000204335.3	ENSG00000252249.1	ENSG00000010626.15
19813	ENSG00000204338.8	ENSG00000252247.2	ENSG00000070610.14
19814	ENSG00000204344.14	ENSG00000252246.1	ENSG00000260651.1
19815	ENSG00000204345.1	ENSG00000252244.1	ENSG00000233355.7
19816	ENSG00000204347.4	ENSG00000252243.1	ENSG00000189157.14
19817	ENSG00000204348.10	ENSG00000252242.1	ENSG00000265188.1
19818	ENSG00000204351.12	ENSG00000252238.1	ENSG00000280607.1
19819	ENSG00000204352.3	ENSG00000252237.1	ENSG00000285018.1

19820	ENSG00000204356.14	ENSG00000252236.1	ENSG00000272696.1
19821	ENSG00000204361.9	ENSG00000252233.1	ENSG00000271754.1
19822	ENSG00000204362.6	ENSG00000252231.1	ENSG00000122126.17
19823	ENSG00000204363.4	ENSG00000252230.1	ENSG00000229932.3
19824	ENSG00000204366.3	ENSG00000252228.1	ENSG00000246877.1
19825	ENSG00000204368.6	ENSG00000252225.1	ENSG00000226581.1
19826	ENSG00000204370.11	ENSG00000252224.1	ENSG00000236785.1
19827	ENSG00000204371.11	ENSG00000252223.1	ENSG00000154640.14
19828	ENSG00000204379.10	ENSG00000252222.1	ENSG00000271420.1
19829	ENSG00000204380.4	ENSG00000252220.1	ENSG00000253347.1
19830	ENSG00000204381.11	ENSG00000252219.2	ENSG00000153064.12
19831	ENSG00000204382.11	ENSG00000252218.1	ENSG00000275756.1
19832	ENSG00000204385.12	ENSG00000252217.1	ENSG00000100664.11
19833	ENSG00000204386.10	ENSG00000252214.1	ENSG00000157388.17
19834	ENSG00000204387.13	ENSG00000252213.1	ENSG00000228638.1
19835	ENSG00000204388.7	ENSG00000252212.1	ENSG00000100583.4
19836	ENSG00000204389.9	ENSG00000252211.1	ENSG00000251682.1
19837	ENSG00000204390.10	ENSG00000252210.1	ENSG00000213888.3
19838	ENSG00000204392.11	ENSG00000252209.1	ENSG00000189339.11
19839	ENSG00000204393.7	ENSG00000252207.1	ENSG00000161813.22
19840	ENSG00000204394.13	ENSG00000252206.1	ENSG00000230325.1
19841	ENSG00000204396.10	ENSG00000252205.1	ENSG00000059691.12
19842	ENSG00000204397.7	ENSG00000252204.1	ENSG00000197253.13
19843	ENSG00000204398.5	ENSG00000252202.1	ENSG00000198130.16
19844	ENSG00000204399.3	ENSG00000252200.1	ENSG00000171119.2
19845	ENSG00000204403.9	ENSG00000252199.1	ENSG00000254003.1
19846	ENSG00000204406.13	ENSG00000252198.1	ENSG00000257275.6
19847	ENSG00000204410.15	ENSG00000252193.1	ENSG00000269072.1
19848	ENSG00000204414.13	ENSG00000252192.1	ENSG00000243207.6
19849	ENSG00000204420.9	ENSG00000252191.1	ENSG00000100373.10
19850	ENSG00000204421.3	ENSG00000252190.1	ENSG00000278965.1
19851	ENSG00000204422.7	ENSG00000252188.1	ENSG00000102910.13
19852	ENSG00000204424.9	ENSG00000252186.1	ENSG00000258425.1
19853	ENSG00000204427.12	ENSG00000252185.1	ENSG00000182327.8
19854	ENSG00000204428.12	ENSG00000252184.1	ENSG00000275765.5
19855	ENSG00000204429.4	ENSG00000252183.1	ENSG00000223609.10
19856	ENSG00000204434.5	ENSG00000252182.1	ENSG00000276603.1
19857	ENSG00000204435.13	ENSG00000252179.1	ENSG00000143507.18
19858	ENSG00000204437.7	ENSG00000252178.1	ENSG00000263874.2
19859	ENSG00000204438.11	ENSG00000252174.1	ENSG00000277694.1
19860	ENSG00000204439.3	ENSG00000252173.1	ENSG00000256274.1
19861	ENSG00000204442.3	ENSG00000252172.1	ENSG00000219986.2
19862	ENSG00000204444.11	ENSG00000252171.1	ENSG00000271871.1
19863	ENSG00000204446.4	ENSG00000252170.2	ENSG00000100311.16
19864	ENSG00000204449.3	ENSG00000252169.1	ENSG00000180834.7
19865	ENSG00000204450.8	ENSG00000252167.1	ENSG00000165684.4
19866	ENSG00000204455.7	ENSG00000252166.1	ENSG00000127415.13
19867	ENSG00000204456.4	ENSG00000252164.1	ENSG00000166265.12
19868	ENSG00000204460.3	ENSG00000252163.1	ENSG00000167216.16
19869	ENSG00000204463.12	ENSG00000252162.1	ENSG00000259100.1
19870	ENSG00000204464.7	ENSG00000252161.1	ENSG00000238251.2
19871	ENSG00000204469.12	ENSG00000252158.1	ENSG00000179038.8
19872	ENSG00000204471.3	ENSG00000252157.1	ENSG00000097046.13

19873	ENSG00000204472.13	ENSG00000252156.1	ENSG00000166337.10
19874	ENSG00000204475.10	ENSG00000252155.1	ENSG00000053372.5
19875	ENSG00000204478.9	ENSG00000252153.1	ENSG00000006634.8
19876	ENSG00000204479.4	ENSG00000252151.1	ENSG00000184350.10
19877	ENSG00000204480.8	ENSG00000252149.1	ENSG00000236200.5
19878	ENSG00000204481.7	ENSG00000252148.1	ENSG00000187792.5
19879	ENSG00000204482.10	ENSG00000252147.1	ENSG00000112208.11
19880	ENSG00000204498.11	ENSG00000252145.1	ENSG00000070985.13
19881	ENSG00000204501.7	ENSG00000252143.1	ENSG00000278627.1
19882	ENSG00000204505.4	ENSG00000252141.1	ENSG00000169933.13
19883	ENSG00000204510.5	ENSG00000252139.1	ENSG00000252021.1
19884	ENSG00000204511.3	ENSG00000252138.1	ENSG00000276493.1
19885	ENSG00000204514.10	ENSG00000252137.1	ENSG00000101901.11
19886	ENSG00000204516.10	ENSG00000252136.1	ENSG00000131153.9
19887	ENSG00000204518.2	ENSG00000252135.1	ENSG00000255838.1
19888	ENSG00000204519.11	ENSG00000252133.1	ENSG00000188739.15
19889	ENSG00000204520.13	ENSG00000252132.1	ENSG00000240849.11
19890	ENSG00000204524.7	ENSG00000252130.1	ENSG00000108684.14
19891	ENSG00000204525.16	ENSG00000252129.1	ENSG00000126838.10
19892	ENSG00000204528.3	ENSG00000252128.1	ENSG00000144559.10
19893	ENSG00000204529.4	ENSG00000252126.2	ENSG00000226029.1
19894	ENSG00000204531.18	ENSG00000252125.1	ENSG00000110318.13
19895	ENSG00000204532.6	ENSG00000252122.1	ENSG00000122592.8
19896	ENSG00000204536.14	ENSG00000252121.1	ENSG00000230663.1
19897	ENSG00000204538.4	ENSG00000252119.1	ENSG00000225956.1
19898	ENSG00000204539.4	ENSG00000252118.1	ENSG00000183323.12
19899	ENSG00000204540.10	ENSG00000252117.1	ENSG00000132470.14
19900	ENSG00000204542.2	ENSG00000252116.1	ENSG00000271303.1
19901	ENSG00000204544.5	ENSG00000252115.1	ENSG00000241755.1
19902	ENSG00000204547.7	ENSG00000252113.2	ENSG00000248821.1
19903	ENSG00000204548.3	ENSG00000252112.1	ENSG00000260329.1
19904	ENSG00000204555.3	ENSG00000252108.1	ENSG00000216802.1
19905	ENSG00000204556.4	ENSG00000252107.1	ENSG00000160224.17
19906	ENSG00000204560.10	ENSG00000252106.2	ENSG00000266990.1
19907	ENSG00000204564.12	ENSG00000252105.1	ENSG00000147535.17
19908	ENSG00000204568.12	ENSG00000252104.1	ENSG00000099246.16
19909	ENSG00000204569.10	ENSG00000252103.1	ENSG00000231006.1
19910	ENSG00000204571.6	ENSG00000252101.2	ENSG00000119632.4
19911	ENSG00000204572.9	ENSG00000252098.1	ENSG00000260423.1
19912	ENSG00000204574.13	ENSG00000252097.1	ENSG00000254187.1
19913	ENSG00000204576.11	ENSG00000252096.1	ENSG00000113721.14
19914	ENSG00000204577.11	ENSG00000252094.1	ENSG00000269028.3
19915	ENSG00000204580.13	ENSG00000252091.1	ENSG00000241322.10
19916	ENSG00000204581.2	ENSG00000252089.1	ENSG00000219881.1
19917	ENSG00000204583.10	ENSG00000252087.1	ENSG00000125531.7
19918	ENSG00000204584.1	ENSG00000252086.1	ENSG00000286267.1
19919	ENSG00000204588.5	ENSG00000252084.1	ENSG00000112210.12
19920	ENSG00000204590.12	ENSG00000252083.1	ENSG00000259212.1
19921	ENSG00000204592.9	ENSG00000252082.1	ENSG00000185728.17
19922	ENSG00000204595.1	ENSG00000252081.1	ENSG00000280057.1
19923	ENSG00000204599.14	ENSG00000252080.1	ENSG00000246575.2
19924	ENSG00000204603.6	ENSG00000252079.1	ENSG00000213904.8
19925	ENSG00000204604.11	ENSG00000252076.1	ENSG00000237520.1

19926	ENSG00000204610.13	ENSG00000252074.1	ENSG00000273972.1
19927	ENSG00000204611.7	ENSG00000252073.1	ENSG00000277386.1
19928	ENSG00000204612.1	ENSG00000252072.1	ENSG00000251636.1
19929	ENSG00000204613.11	ENSG00000252070.1	ENSG00000211717.3
19930	ENSG00000204614.9	ENSG00000252069.1	ENSG00000237232.7
19931	ENSG00000204616.11	ENSG00000252068.1	ENSG00000111206.12
19932	ENSG00000204618.8	ENSG00000252067.1	ENSG00000215861.5
19933	ENSG00000204619.8	ENSG00000252066.1	ENSG00000163884.4
19934	ENSG00000204620.3	ENSG00000252065.1	ENSG00000281179.1
19935	ENSG00000204622.11	ENSG00000252064.1	ENSG00000264673.1
19936	ENSG00000204623.9	ENSG00000252063.1	ENSG00000162572.20
19937	ENSG00000204624.8	ENSG00000252062.1	ENSG00000112902.12
19938	ENSG00000204625.10	ENSG00000252061.1	ENSG00000260828.1
19939	ENSG00000204628.11	ENSG00000252060.1	ENSG00000170293.9
19940	ENSG00000204632.11	ENSG00000252057.2	ENSG00000150667.8
19941	ENSG00000204634.12	ENSG00000252053.1	ENSG00000141013.17
19942	ENSG00000204637.4	ENSG00000252051.1	ENSG00000257475.2
19943	ENSG00000204640.1	ENSG00000252050.1	ENSG00000228158.2
19944	ENSG00000204642.14	ENSG00000252049.1	ENSG00000255801.1
19945	ENSG00000204644.9	ENSG00000252047.1	ENSG00000126934.13
19946	ENSG00000204648.11	ENSG00000252046.1	ENSG00000261114.1
19947	ENSG00000204650.14	ENSG00000252045.1	ENSG00000235748.1
19948	ENSG00000204652.6	ENSG00000252042.1	ENSG00000260439.1
19949	ENSG00000204653.10	ENSG00000252041.1	ENSG00000259904.1
19950	ENSG00000204655.12	ENSG00000252040.1	ENSG00000148842.18
19951	ENSG00000204657.4	ENSG00000252039.1	ENSG00000115998.7
19952	ENSG00000204658.5	ENSG00000252037.1	ENSG00000259316.11
19953	ENSG00000204659.5	ENSG00000252036.1	ENSG00000249180.1
19954	ENSG00000204661.9	ENSG00000252035.1	ENSG00000234814.8
19955	ENSG00000204662.2	ENSG00000252034.1	ENSG00000279953.1
19956	ENSG00000204663.9	ENSG00000252033.1	ENSG00000204060.7
19957	ENSG00000204666.3	ENSG00000252032.1	ENSG00000285793.1
19958	ENSG00000204669.10	ENSG00000252031.1	ENSG00000078098.14
19959	ENSG00000204670.7	ENSG00000252030.1	ENSG00000225507.1
19960	ENSG00000204671.1	ENSG00000252029.1	ENSG00000109436.8
19961	ENSG00000204673.10	ENSG00000252028.1	ENSG00000174744.14
19962	ENSG00000204677.11	ENSG00000252027.1	ENSG00000213714.1
19963	ENSG00000204681.11	ENSG00000252026.1	ENSG00000134575.10
19964	ENSG00000204682.6	ENSG00000252025.1	ENSG00000266896.1
19965	ENSG00000204683.11	ENSG00000252023.1	ENSG00000130558.19
19966	ENSG00000204684.3	ENSG00000252022.1	ENSG00000142279.12
19967	ENSG00000204685.6	ENSG00000252021.1	ENSG00000273080.1
19968	ENSG00000204687.4	ENSG00000252020.1	ENSG00000259418.1
19969	ENSG00000204688.9	ENSG00000252019.1	ENSG00000121067.18
19970	ENSG00000204694.11	ENSG00000252018.1	ENSG00000177706.9
19971	ENSG00000204695.4	ENSG00000252017.1	ENSG00000248671.7
19972	ENSG00000204697.3	ENSG00000252016.1	ENSG00000228106.5
19973	ENSG00000204699.4	ENSG00000252015.1	ENSG00000167941.3
19974	ENSG00000204700.5	ENSG00000252014.1	ENSG00000224159.1
19975	ENSG00000204701.2	ENSG00000252013.1	ENSG00000230267.7
19976	ENSG00000204702.5	ENSG00000252012.1	ENSG00000267302.5
19977	ENSG00000204703.5	ENSG00000252011.1	ENSG00000231393.1
19978	ENSG00000204704.2	ENSG00000252010.1	ENSG00000126947.12

19979	ENSG00000204705.3	ENSG00000252008.1	ENSG00000236576.1
19980	ENSG00000204706.14	ENSG00000252005.2	ENSG00000158793.14
19981	ENSG00000204709.4	ENSG00000252003.1	ENSG00000124103.9
19982	ENSG00000204710.2	ENSG00000252002.1	ENSG00000011143.16
19983	ENSG00000204711.9	ENSG00000252001.1	ENSG00000103316.11
19984	ENSG00000204713.11	ENSG00000251999.1	ENSG00000215388.3
19985	ENSG00000204718.3	ENSG00000251998.1	ENSG00000260290.2
19986	ENSG00000204740.11	ENSG00000251997.1	ENSG00000166734.20
19987	ENSG00000204745.4	ENSG00000251996.1	ENSG00000113312.11
19988	ENSG00000204754.3	ENSG00000251994.1	ENSG00000285744.1
19989	ENSG00000204758.7	ENSG00000251993.1	ENSG00000275393.1
19990	ENSG00000204764.14	ENSG00000251992.1	ENSG00000114942.14
19991	ENSG00000204767.4	ENSG00000251991.1	ENSG00000182156.10
19992	ENSG00000204776.6	ENSG00000251990.1	ENSG00000109819.9
19993	ENSG00000204778.4	ENSG00000251988.1	ENSG00000126003.7
19994	ENSG00000204779.2	ENSG00000251987.1	ENSG00000153253.17
19995	ENSG00000204780.5	ENSG00000251986.1	ENSG00000214772.2
19996	ENSG00000204787.8	ENSG00000251985.1	ENSG00000169992.10
19997	ENSG00000204789.4	ENSG00000251983.1	ENSG00000213025.2
19998	ENSG00000204790.9	ENSG00000251982.1	ENSG00000187870.7
19999	ENSG00000204791.10	ENSG00000251981.1	ENSG00000267978.5
20000	ENSG00000204792.2	ENSG00000251980.1	ENSG00000273590.4
20001	ENSG00000204801.7	ENSG00000251978.1	ENSG00000189316.3
20002	ENSG00000204802.3	ENSG00000251977.1	ENSG00000264438.2
20003	ENSG00000204805.9	ENSG00000251976.1	ENSG00000223969.5
20004	ENSG00000204814.5	ENSG00000251975.1	ENSG00000140368.12
20005	ENSG00000204815.10	ENSG00000251974.1	ENSG00000236824.2
20006	ENSG00000204816.5	ENSG00000251973.1	ENSG00000206589.1
20007	ENSG00000204818.4	ENSG00000251972.1	ENSG00000112379.9
20008	ENSG00000204822.7	ENSG00000251971.1	ENSG00000267316.5
20009	ENSG00000204832.9	ENSG00000251970.1	ENSG00000201457.1
20010	ENSG00000204837.4	ENSG00000251967.1	ENSG00000231367.5
20011	ENSG00000204839.9	ENSG00000251965.1	ENSG00000180423.4
20012	ENSG00000204842.16	ENSG00000251961.1	ENSG00000210112.1
20013	ENSG00000204843.12	ENSG00000251960.1	ENSG00000126251.6
20014	ENSG00000204849.8	ENSG00000251958.1	ENSG00000147206.17
20015	ENSG00000204850.4	ENSG00000251957.1	ENSG00000184923.12
20016	ENSG00000204851.6	ENSG00000251956.1	ENSG00000241749.4
20017	ENSG00000204852.15	ENSG00000251955.1	ENSG00000144821.9
20018	ENSG00000204856.12	ENSG00000251954.1	ENSG00000124140.13
20019	ENSG00000204859.13	ENSG00000251953.1	ENSG00000196151.10
20020	ENSG00000204860.4	ENSG00000251952.1	ENSG00000213171.2
20021	ENSG00000204866.8	ENSG00000251951.1	ENSG00000248633.1
20022	ENSG00000204869.8	ENSG00000251947.1	ENSG00000084734.9
20023	ENSG00000204872.4	ENSG00000251946.1	ENSG00000230615.6
20024	ENSG00000204873.4	ENSG00000251943.1	ENSG00000234429.1
20025	ENSG00000204876.4	ENSG00000251942.1	ENSG00000228570.7
20026	ENSG00000204880.7	ENSG00000251941.1	ENSG00000179598.6
20027	ENSG00000204882.3	ENSG00000251940.1	ENSG00000148795.7
20028	ENSG00000204887.4	ENSG00000251939.1	ENSG00000160401.15
20029	ENSG00000204889.10	ENSG00000251937.1	ENSG00000282501.1
20030	ENSG00000204894.4	ENSG00000251936.1	ENSG00000174944.9
20031	ENSG00000204897.6	ENSG00000251935.1	ENSG00000229417.1

20032	ENSG00000204899.6	ENSG00000251934.1	ENSG00000253772.1
20033	ENSG00000204904.7	ENSG00000251931.1	ENSG00000160307.10
20034	ENSG00000204909.8	ENSG00000251929.1	ENSG00000170122.5
20035	ENSG00000204913.6	ENSG00000251925.1	ENSG00000103091.15
20036	ENSG00000204915.3	ENSG00000251924.1	ENSG00000196092.13
20037	ENSG00000204918.3	ENSG00000251923.1	ENSG00000278002.1
20038	ENSG00000204919.1	ENSG00000251922.1	ENSG00000233621.1
20039	ENSG00000204920.11	ENSG00000251920.1	ENSG00000187860.10
20040	ENSG00000204922.5	ENSG00000251919.1	ENSG00000211669.3
20041	ENSG00000204923.4	ENSG00000251917.1	ENSG00000212769.5
20042	ENSG00000204928.2	ENSG00000251916.1	ENSG00000258732.1
20043	ENSG00000204929.12	ENSG00000251915.1	ENSG00000109920.12
20044	ENSG00000204930.9	ENSG00000251914.1	ENSG00000260281.5
20045	ENSG00000204933.3	ENSG00000251913.1	ENSG00000118523.6
20046	ENSG00000204934.10	ENSG00000251908.1	ENSG00000196273.7
20047	ENSG00000204936.10	ENSG00000251907.1	ENSG00000273442.1
20048	ENSG00000204941.14	ENSG00000251906.2	ENSG00000037241.7
20049	ENSG00000204946.10	ENSG00000251905.1	ENSG00000151789.12
20050	ENSG00000204947.9	ENSG00000251904.1	ENSG00000170684.9
20051	ENSG00000204949.8	ENSG00000251900.1	ENSG00000116001.16
20052	ENSG00000204950.4	ENSG00000251898.1	ENSG00000211955.2
20053	ENSG00000204952.2	ENSG00000251897.1	ENSG00000132436.11
20054	ENSG00000204954.10	ENSG00000251896.1	ENSG00000248429.5
20055	ENSG00000204956.5	ENSG00000251895.1	ENSG00000279744.1
20056	ENSG00000204959.4	ENSG00000251893.2	ENSG00000226576.1
20057	ENSG00000204960.6	ENSG00000251892.1	ENSG00000285269.2
20058	ENSG00000204961.6	ENSG00000251891.1	ENSG00000259048.1
20059	ENSG00000204962.6	ENSG00000251890.1	ENSG00000163006.12
20060	ENSG00000204963.6	ENSG00000251889.1	ENSG00000135218.19
20061	ENSG00000204965.9	ENSG00000251888.1	ENSG00000186594.14
20062	ENSG00000204967.11	ENSG00000251887.2	ENSG00000229961.3
20063	ENSG00000204969.7	ENSG00000251886.1	ENSG00000215414.4
20064	ENSG00000204970.9	ENSG00000251884.1	ENSG00000240877.3
20065	ENSG00000204971.3	ENSG00000251883.1	ENSG00000226107.1
20066	ENSG00000204977.10	ENSG00000251882.1	ENSG00000230798.5
20067	ENSG00000204978.2	ENSG00000251880.1	ENSG00000231682.1
20068	ENSG00000204979.7	ENSG00000251878.1	ENSG00000100522.10
20069	ENSG00000204982.3	ENSG00000251877.1	ENSG00000243710.7
20070	ENSG00000204983.14	ENSG00000251875.1	ENSG00000034677.13
20071	ENSG00000204989.3	ENSG00000251874.1	ENSG00000205307.12
20072	ENSG00000204990.3	ENSG00000251873.1	ENSG00000277209.1
20073	ENSG00000204991.11	ENSG00000251870.1	ENSG00000163357.10
20074	ENSG00000204993.3	ENSG00000251869.1	ENSG00000229241.1
20075	ENSG00000205002.3	ENSG00000251868.1	ENSG00000144820.8
20076	ENSG00000205015.1	ENSG00000251867.4	ENSG00000129932.8
20077	ENSG00000205018.2	ENSG00000251866.1	ENSG00000169925.16
20078	ENSG00000205022.9	ENSG00000251865.1	ENSG00000243469.1
20079	ENSG00000205025.8	ENSG00000251864.1	ENSG00000270147.1
20080	ENSG00000205029.1	ENSG00000251862.2	ENSG00000161526.15
20081	ENSG00000205030.1	ENSG00000251861.1	ENSG00000182541.18
20082	ENSG00000205035.8	ENSG00000251859.1	ENSG00000119866.21
20083	ENSG00000205037.2	ENSG00000251858.1	ENSG00000166006.13
20084	ENSG00000205038.12	ENSG00000251857.1	ENSG00000285159.1

20085	ENSG00000205041.1	ENSG00000251856.1	ENSG00000164180.13
20086	ENSG00000205044.5	ENSG00000251854.1	ENSG00000112877.8
20087	ENSG00000205045.9	ENSG00000251852.1	ENSG00000167014.11
20088	ENSG00000205054.7	ENSG00000251851.1	ENSG00000232309.2
20089	ENSG00000205056.8	ENSG00000251850.1	ENSG00000218358.2
20090	ENSG00000205057.5	ENSG00000251844.1	ENSG00000248205.1
20091	ENSG00000205060.11	ENSG00000251843.1	ENSG00000173372.17
20092	ENSG00000205076.4	ENSG00000251842.1	ENSG00000269947.1
20093	ENSG00000205078.5	ENSG00000251841.1	ENSG00000142632.17
20094	ENSG00000205084.11	ENSG00000251840.1	ENSG00000207501.1
20095	ENSG00000205085.11	ENSG00000251839.1	ENSG00000237704.1
20096	ENSG00000205086.7	ENSG00000251838.1	ENSG00000235381.1
20097	ENSG00000205089.7	ENSG00000251837.1	ENSG00000275409.1
20098	ENSG00000205090.9	ENSG00000251835.1	ENSG00000273327.1
20099	ENSG00000205097.6	ENSG00000251834.1	ENSG00000229251.3
20100	ENSG00000205100.2	ENSG00000251831.1	ENSG00000272092.1
20101	ENSG00000205105.6	ENSG00000251830.1	ENSG00000162073.13
20102	ENSG00000205106.4	ENSG00000251829.1	ENSG00000267053.6
20103	ENSG00000205108.5	ENSG00000251828.1	ENSG00000272588.1
20104	ENSG00000205111.8	ENSG00000251825.1	ENSG00000135862.6
20105	ENSG00000205116.3	ENSG00000251823.2	ENSG00000260052.1
20106	ENSG00000205126.2	ENSG00000251822.1	ENSG00000104321.11
20107	ENSG00000205129.8	ENSG00000251821.1	ENSG00000232719.1
20108	ENSG00000205133.11	ENSG00000251819.1	ENSG00000177807.9
20109	ENSG00000205138.3	ENSG00000251818.1	ENSG00000002587.10
20110	ENSG00000205143.2	ENSG00000251816.1	ENSG00000169696.16
20111	ENSG00000205155.8	ENSG00000251815.1	ENSG00000279696.1
20112	ENSG00000205174.4	ENSG00000251814.1	ENSG00000112294.12
20113	ENSG00000205176.3	ENSG00000251813.1	ENSG00000244063.1
20114	ENSG00000205177.6	ENSG00000251812.1	ENSG00000227262.3
20115	ENSG00000205181.6	ENSG00000251811.1	ENSG00000279981.1
20116	ENSG00000205184.2	ENSG00000251810.1	ENSG00000232600.3
20117	ENSG00000205186.3	ENSG00000251809.1	ENSG00000188394.6
20118	ENSG00000205189.12	ENSG00000251807.1	ENSG00000273008.1
20119	ENSG00000205208.5	ENSG00000251805.1	ENSG00000267346.1
20120	ENSG00000205209.7	ENSG00000251804.1	ENSG00000186912.6
20121	ENSG00000205212.4	ENSG00000251803.1	ENSG00000261433.1
20122	ENSG00000205213.14	ENSG00000251802.1	ENSG00000261609.6
20123	ENSG00000205215.8	ENSG00000251799.1	ENSG00000262140.1
20124	ENSG00000205220.12	ENSG00000251798.1	ENSG00000145216.16
20125	ENSG00000205221.12	ENSG00000251796.1	ENSG00000226057.7
20126	ENSG00000205231.1	ENSG00000251794.1	ENSG00000156384.14
20127	ENSG00000205236.6	ENSG00000251792.1	ENSG00000157782.9
20128	ENSG00000205238.9	ENSG00000251791.1	ENSG00000213309.3
20129	ENSG00000205240.4	ENSG00000251789.2	ENSG00000278249.1
20130	ENSG00000205250.9	ENSG00000251788.1	ENSG00000217442.4
20131	ENSG00000205266.10	ENSG00000251787.1	ENSG00000259422.1
20132	ENSG00000205267.5	ENSG00000251785.1	ENSG00000211699.2
20133	ENSG00000205268.10	ENSG00000251783.1	ENSG00000185591.10
20134	ENSG00000205269.6	ENSG00000251781.1	ENSG00000247315.3
20135	ENSG00000205274.3	ENSG00000251779.1	ENSG00000197702.13
20136	ENSG00000205277.9	ENSG00000251778.1	ENSG00000120784.16
20137	ENSG00000205279.8	ENSG00000251776.1	ENSG00000087502.18

20138	ENSG00000205293.4	ENSG00000251775.1	ENSG00000075223.14
20139	ENSG00000205300.3	ENSG00000251774.1	ENSG00000214401.4
20140	ENSG00000205301.11	ENSG00000251773.1	ENSG00000166707.11
20141	ENSG00000205302.7	ENSG00000251770.1	ENSG00000259448.2
20142	ENSG00000205307.12	ENSG00000251768.1	ENSG00000229391.7
20143	ENSG00000205309.14	ENSG00000251767.1	ENSG00000117394.21
20144	ENSG00000205312.8	ENSG00000251766.1	ENSG00000257515.1
20145	ENSG00000205318.5	ENSG00000251764.1	ENSG00000233672.6
20146	ENSG00000205323.9	ENSG00000251763.1	ENSG00000118939.17
20147	ENSG00000205325.1	ENSG00000251761.1	ENSG00000241923.2
20148	ENSG00000205327.3	ENSG00000251760.1	ENSG00000117601.13
20149	ENSG00000205328.2	ENSG00000251759.1	ENSG00000099338.23
20150	ENSG00000205329.2	ENSG00000251757.1	ENSG00000019169.10
20151	ENSG00000205330.4	ENSG00000251756.1	ENSG00000159899.14
20152	ENSG00000205331.5	ENSG00000251754.1	ENSG00000271998.1
20153	ENSG00000205333.5	ENSG00000251753.1	ENSG00000266980.1
20154	ENSG00000205334.2	ENSG00000251752.1	ENSG00000255432.1
20155	ENSG00000205336.11	ENSG00000251751.1	ENSG00000114770.17
20156	ENSG00000205339.10	ENSG00000251750.1	ENSG00000226853.2
20157	ENSG00000205352.11	ENSG00000251748.1	ENSG00000239959.1
20158	ENSG00000205356.9	ENSG00000251747.1	ENSG00000218739.10
20159	ENSG00000205358.3	ENSG00000251746.1	ENSG00000168306.13
20160	ENSG00000205359.9	ENSG00000251745.1	ENSG00000232160.6
20161	ENSG00000205360.6	ENSG00000251744.1	ENSG00000120889.13
20162	ENSG00000205361.8	ENSG00000251742.1	ENSG00000269972.1
20163	ENSG00000205362.11	ENSG00000251741.1	ENSG00000279333.1
20164	ENSG00000205363.5	ENSG00000251739.1	ENSG00000177369.8
20165	ENSG00000205364.4	ENSG00000251735.1	ENSG00000148344.11
20166	ENSG00000205396.11	ENSG00000251733.1	ENSG00000226477.1
20167	ENSG00000205403.13	ENSG00000251732.1	ENSG00000127946.17
20168	ENSG00000205409.3	ENSG00000251730.1	ENSG00000271046.1
20169	ENSG00000205412.5	ENSG00000251729.1	ENSG00000067533.6
20170	ENSG00000205413.8	ENSG00000251728.1	ENSG00000227811.2
20171	ENSG00000205414.1	ENSG00000251727.1	ENSG00000235659.1
20172	ENSG00000205420.11	ENSG00000251726.1	ENSG00000021776.11
20173	ENSG00000205423.11	ENSG00000251724.1	ENSG00000214867.3
20174	ENSG00000205424.1	ENSG00000251722.1	ENSG00000163939.18
20175	ENSG00000205426.10	ENSG00000251720.1	ENSG00000188175.9
20176	ENSG00000205433.4	ENSG00000251719.1	ENSG00000005020.13
20177	ENSG00000205436.7	ENSG00000251718.1	ENSG00000254709.7
20178	ENSG00000205439.10	ENSG00000251717.1	ENSG00000175536.7
20179	ENSG00000205442.12	ENSG00000251715.1	ENSG00000130449.6
20180	ENSG00000205444.3	ENSG00000251714.1	ENSG00000253831.2
20181	ENSG00000205445.3	ENSG00000251712.3	ENSG00000000460.17
20182	ENSG00000205449.11	ENSG00000251711.1	ENSG00000070731.10
20183	ENSG00000205452.5	ENSG00000251707.2	ENSG00000254965.1
20184	ENSG00000205456.11	ENSG00000251706.1	ENSG00000250326.1
20185	ENSG00000205457.11	ENSG00000251705.1	ENSG00000273313.1
20186	ENSG00000205464.12	ENSG00000251704.1	ENSG00000143147.14
20187	ENSG00000205476.9	ENSG00000251703.1	ENSG00000090686.15
20188	ENSG00000205482.9	ENSG00000251702.1	ENSG00000253629.1
20189	ENSG00000205485.13	ENSG00000251698.1	ENSG00000259363.5
20190	ENSG00000205488.9	ENSG00000251697.1	ENSG00000283118.1

20191	ENSG00000205494.9	ENSG00000251694.1	ENSG00000227382.1
20192	ENSG00000205495.1	ENSG00000251692.7	ENSG00000279391.1
20193	ENSG00000205496.1	ENSG00000251691.2	ENSG00000171811.14
20194	ENSG00000205497.4	ENSG00000251689.1	ENSG00000132967.9
20195	ENSG00000205500.8	ENSG00000251688.1	ENSG00000253955.1
20196	ENSG00000205502.4	ENSG00000251687.1	ENSG00000181924.7
20197	ENSG00000205517.12	ENSG00000251686.1	ENSG00000137571.11
20198	ENSG00000205531.13	ENSG00000251685.3	ENSG00000106780.9
20199	ENSG00000205534.6	ENSG00000251682.1	ENSG00000110048.12
20200	ENSG00000205537.2	ENSG00000251680.5	ENSG00000213608.5
20201	ENSG00000205542.11	ENSG00000251679.1	ENSG00000231049.2
20202	ENSG00000205544.4	ENSG00000251678.1	ENSG00000277782.1
20203	ENSG00000205549.9	ENSG00000251676.1	ENSG00000179988.14
20204	ENSG00000205559.4	ENSG00000251675.1	ENSG00000278535.5
20205	ENSG00000205560.12	ENSG00000251670.1	ENSG00000145916.19
20206	ENSG00000205562.2	ENSG00000251669.5	ENSG00000267736.2
20207	ENSG00000205571.13	ENSG00000251668.2	ENSG00000270558.1
20208	ENSG00000205572.9	ENSG00000251667.1	ENSG00000186009.4
20209	ENSG00000205578.6	ENSG00000251666.1	ENSG00000116791.14
20210	ENSG00000205579.3	ENSG00000251665.1	ENSG00000246465.1
20211	ENSG00000205581.11	ENSG00000251664.4	ENSG00000111640.15
20212	ENSG00000205583.13	ENSG00000251663.1	ENSG00000285865.1
20213	ENSG00000205584.6	ENSG00000251661.3	ENSG00000251359.4
20214	ENSG00000205592.14	ENSG00000251660.1	ENSG00000285557.1
20215	ENSG00000205593.12	ENSG00000251656.1	ENSG00000236953.1
20216	ENSG00000205596.4	ENSG00000251655.6	ENSG00000171503.11
20217	ENSG00000205609.12	ENSG00000251654.2	ENSG00000229719.5
20218	ENSG00000205611.4	ENSG00000251652.1	ENSG00000151303.11
20219	ENSG00000205622.10	ENSG00000251649.1	ENSG00000232373.2
20220	ENSG00000205625.2	ENSG00000251648.2	ENSG00000272663.1
20221	ENSG00000205628.3	ENSG00000251647.2	ENSG00000236859.6
20222	ENSG00000205629.12	ENSG00000251644.1	ENSG00000265204.1
20223	ENSG00000205632.3	ENSG00000251643.1	ENSG00000189127.8
20224	ENSG00000205634.6	ENSG00000251642.1	ENSG00000283515.1
20225	ENSG00000205636.3	ENSG00000251639.2	ENSG00000207688.2
20226	ENSG00000205639.10	ENSG00000251638.1	ENSG00000138071.13
20227	ENSG00000205642.10	ENSG00000251637.6	ENSG00000132604.11
20228	ENSG00000205643.11	ENSG00000251636.1	ENSG00000264057.1
20229	ENSG00000205644.5	ENSG00000251635.1	ENSG00000255384.1
20230	ENSG00000205649.7	ENSG00000251634.2	ENSG00000197779.13
20231	ENSG00000205653.2	ENSG00000251633.3	ENSG00000277582.1
20232	ENSG00000205659.10	ENSG00000251632.1	ENSG00000274026.2
20233	ENSG00000205667.2	ENSG00000251630.1	ENSG00000180113.16
20234	ENSG00000205669.3	ENSG00000251629.6	ENSG00000178386.13
20235	ENSG00000205670.11	ENSG00000251627.1	ENSG00000239840.1
20236	ENSG00000205678.7	ENSG00000251624.1	ENSG00000175309.15
20237	ENSG00000205682.2	ENSG00000251623.2	ENSG00000274173.1
20238	ENSG00000205683.11	ENSG00000251621.1	ENSG00000271828.1
20239	ENSG00000205693.3	ENSG00000251620.2	ENSG00000167434.10
20240	ENSG00000205695.3	ENSG00000251619.1	ENSG00000162817.7
20241	ENSG00000205696.4	ENSG00000251618.1	ENSG00000169682.18
20242	ENSG00000205702.11	ENSG00000251616.1	ENSG00000154380.17
20243	ENSG00000205704.6	ENSG00000251615.3	ENSG00000176273.15

20244	ENSG00000205707.10	ENSG00000251614.1	ENSG00000231443.2
20245	ENSG00000205710.4	ENSG00000251613.3	ENSG00000244052.1
20246	ENSG00000205716.4	ENSG00000251611.1	ENSG00000234332.1
20247	ENSG00000205718.9	ENSG00000251610.2	ENSG00000278050.1
20248	ENSG00000205726.15	ENSG00000251609.2	ENSG00000176903.4
20249	ENSG00000205730.6	ENSG00000251608.2	ENSG00000135334.9
20250	ENSG00000205740.2	ENSG00000251605.1	ENSG00000256751.5
20251	ENSG00000205744.10	ENSG00000251604.1	ENSG00000153147.6
20252	ENSG00000205745.2	ENSG00000251602.6	ENSG00000101384.12
20253	ENSG00000205746.9	ENSG00000251601.1	ENSG00000259556.2
20254	ENSG00000205754.11	ENSG00000251599.1	ENSG00000099337.5
20255	ENSG00000205755.11	ENSG00000251598.2	ENSG00000283559.1
20256	ENSG00000205755.11_PAR_Y	ENSG00000251597.1	ENSG00000235109.7
20257	ENSG00000205758.12	ENSG00000251596.1	ENSG00000161040.16
20258	ENSG00000205763.13	ENSG00000251595.7	ENSG00000267058.1
20259	ENSG00000205765.9	ENSG00000251593.1	ENSG00000255666.6
20260	ENSG00000205767.4	ENSG00000251591.2	ENSG00000133321.11
20261	ENSG00000205771.6	ENSG00000251590.1	ENSG00000282339.1
20262	ENSG00000205777.17	ENSG00000251588.2	ENSG00000139540.12
20263	ENSG00000205784.3	ENSG00000251587.1	ENSG00000261278.1
20264	ENSG00000205786.8	ENSG00000251586.1	ENSG00000176945.17
20265	ENSG00000205790.1	ENSG00000251585.1	ENSG00000280162.1
20266	ENSG00000205791.3	ENSG00000251584.1	ENSG00000132510.10
20267	ENSG00000205794.4	ENSG00000251583.1	ENSG00000145703.16
20268	ENSG00000205795.4	ENSG00000251580.2	ENSG00000284237.1
20269	ENSG00000205808.5	ENSG00000251579.1	ENSG00000095321.17
20270	ENSG00000205809.9	ENSG00000251578.1	ENSG00000144339.12
20271	ENSG00000205810.8	ENSG00000251577.5	ENSG00000240143.1
20272	ENSG00000205822.10	ENSG00000251575.2	ENSG00000197561.7
20273	ENSG00000205830.1	ENSG00000251574.7	ENSG00000261373.1
20274	ENSG00000205832.7	ENSG00000251573.2	ENSG00000240230.6
20275	ENSG00000205835.8	ENSG00000251572.2	ENSG00000221843.4
20276	ENSG00000205837.7	ENSG00000251571.1	ENSG00000269997.1
20277	ENSG00000205838.14	ENSG00000251569.1	ENSG00000232701.1
20278	ENSG00000205846.3	ENSG00000251568.1	ENSG00000259318.1
20279	ENSG00000205847.6	ENSG00000251567.5	ENSG00000206344.7
20280	ENSG00000205853.10	ENSG00000251566.1	ENSG00000101230.6
20281	ENSG00000205856.4	ENSG00000251563.1	ENSG00000280149.1
20282	ENSG00000205857.2	ENSG00000251562.8	ENSG00000137818.12
20283	ENSG00000205858.9	ENSG00000251557.2	ENSG00000235008.1
20284	ENSG00000205861.11	ENSG00000251556.1	ENSG00000268379.1
20285	ENSG00000205863.10	ENSG00000251555.1	ENSG00000184838.15
20286	ENSG00000205864.1	ENSG00000251553.1	ENSG00000136205.17
20287	ENSG00000205865.4	ENSG00000251552.1	ENSG00000270521.1
20288	ENSG00000205866.3	ENSG00000251550.1	ENSG00000249786.7
20289	ENSG00000205867.3	ENSG00000251549.2	ENSG00000206013.2
20290	ENSG00000205869.2	ENSG00000251548.1	ENSG00000145332.14
20291	ENSG00000205871.5	ENSG00000251546.1	ENSG00000255274.9
20292	ENSG00000205879.5	ENSG00000251545.2	ENSG00000262370.5
20293	ENSG00000205882.8	ENSG00000251544.1	ENSG00000170545.17
20294	ENSG00000205883.2	ENSG00000251543.1	ENSG00000223799.1
20295	ENSG00000205884.2	ENSG00000251542.1	ENSG00000203772.7
20296	ENSG00000205885.7	ENSG00000251539.1	ENSG00000014164.7

20297	ENSG00000205890.3	ENSG00000251538.6	ENSG00000102786.15
20298	ENSG00000205897.5	ENSG00000251537.4	ENSG00000253320.6
20299	ENSG00000205898.3	ENSG00000251536.2	ENSG00000090661.12
20300	ENSG00000205899.3	ENSG00000251535.1	ENSG00000162458.13
20301	ENSG00000205903.7	ENSG00000251533.2	ENSG00000179101.5
20302	ENSG00000205913.6	ENSG00000251532.1	ENSG00000256171.1
20303	ENSG00000205916.11	ENSG00000251529.1	ENSG00000237037.9
20304	ENSG00000205918.9	ENSG00000251527.1	ENSG00000243910.7
20305	ENSG00000205922.4	ENSG00000251526.1	ENSG00000175267.15
20306	ENSG00000205923.3	ENSG00000251525.6	ENSG00000211813.2
20307	ENSG00000205927.5	ENSG00000251523.1	ENSG00000271714.1
20308	ENSG00000205929.11	ENSG00000251521.2	ENSG00000124134.9
20309	ENSG00000205930.9	ENSG00000251520.1	ENSG00000268583.1
20310	ENSG00000205936.3	ENSG00000251518.1	ENSG00000163681.14
20311	ENSG00000205937.12	ENSG00000251517.1	ENSG00000217488.2
20312	ENSG00000205940.8	ENSG00000251516.1	ENSG00000176390.12
20313	ENSG00000205944.11	ENSG00000251515.1	ENSG00000182600.9
20314	ENSG00000205946.1	ENSG00000251513.2	ENSG00000198624.13
20315	ENSG00000205955.4	ENSG00000251511.1	ENSG00000175643.10
20316	ENSG00000205959.3	ENSG00000251510.1	ENSG00000245614.3
20317	ENSG00000205971.3	ENSG00000251508.1	ENSG00000265800.1
20318	ENSG00000205976.4	ENSG00000251506.1	ENSG00000241489.8
20319	ENSG00000205978.6	ENSG00000251504.1	ENSG00000241544.1
20320	ENSG00000205981.8	ENSG00000251503.8	ENSG00000121022.14
20321	ENSG00000205989.1	ENSG00000251501.1	ENSG00000277198.1
20322	ENSG00000206013.2	ENSG00000251498.1	ENSG00000196735.11
20323	ENSG00000206014.7	ENSG00000251497.2	ENSG00000262692.1
20324	ENSG00000206026.7	ENSG00000251495.1	ENSG00000233802.8
20325	ENSG00000206028.1	ENSG00000251493.5	ENSG00000002549.12
20326	ENSG00000206034.1	ENSG00000251492.1	ENSG00000224367.6
20327	ENSG00000206042.1	ENSG00000251491.2	ENSG00000240498.8
20328	ENSG00000206043.6	ENSG00000251490.1	ENSG00000003402.20
20329	ENSG00000206047.2	ENSG00000251489.2	ENSG00000197238.4
20330	ENSG00000206052.11	ENSG00000251488.1	ENSG00000232815.1
20331	ENSG00000206053.13	ENSG00000251487.1	ENSG00000233707.3
20332	ENSG00000206062.4	ENSG00000251485.1	ENSG00000213999.16
20333	ENSG00000206066.3	ENSG00000251484.3	ENSG00000152944.9
20334	ENSG00000206069.6	ENSG00000251483.2	ENSG00000267737.1
20335	ENSG00000206072.12	ENSG00000251482.1	ENSG00000163162.8
20336	ENSG00000206073.10	ENSG00000251478.1	ENSG00000258526.6
20337	ENSG00000206075.14	ENSG00000251477.1	ENSG00000059728.11
20338	ENSG00000206077.11	ENSG00000251476.2	ENSG00000250909.1
20339	ENSG00000206090.4	ENSG00000251474.6	ENSG00000197837.3
20340	ENSG00000206102.2	ENSG00000251473.2	ENSG00000106078.19
20341	ENSG00000206104.2	ENSG00000251471.1	ENSG00000134460.17
20342	ENSG00000206105.2	ENSG00000251470.1	ENSG00000167554.15
20343	ENSG00000206106.2	ENSG00000251468.2	ENSG00000234937.1
20344	ENSG00000206107.2	ENSG00000251467.1	ENSG00000058668.14
20345	ENSG00000206113.10	ENSG00000251464.2	ENSG00000239571.1
20346	ENSG00000206120.11	ENSG00000251463.2	ENSG00000272070.1
20347	ENSG00000206127.10	ENSG00000251461.3	ENSG00000124789.11
20348	ENSG00000206129.4	ENSG00000251460.1	ENSG00000259540.1
20349	ENSG00000206140.11	ENSG00000251459.1	ENSG00000149634.4

20350	ENSG00000206142.9	ENSG00000251458.1	ENSG00000268518.1
20351	ENSG00000206144.5	ENSG00000251456.1	ENSG00000182223.7
20352	ENSG00000206145.8	ENSG00000251455.1	ENSG00000171262.11
20353	ENSG00000206147.5	ENSG00000251454.1	ENSG00000164818.16
20354	ENSG00000206149.10	ENSG00000251453.1	ENSG00000216480.2
20355	ENSG00000206150.4	ENSG00000251452.2	ENSG00000163751.4
20356	ENSG00000206159.11	ENSG00000251451.1	ENSG00000197540.8
20357	ENSG00000206168.1	ENSG00000251450.1	ENSG00000269489.1
20358	ENSG00000206172.8	ENSG00000251449.2	ENSG00000251467.1
20359	ENSG00000206177.7	ENSG00000251448.1	ENSG00000204569.10
20360	ENSG00000206178.2	ENSG00000251447.1	ENSG00000204305.14
20361	ENSG00000206181.5	ENSG00000251446.1	ENSG00000171853.16
20362	ENSG00000206187.3	ENSG00000251445.1	ENSG00000198176.13
20363	ENSG00000206190.11	ENSG00000251443.1	ENSG00000101197.13
20364	ENSG00000206192.7	ENSG00000251441.3	ENSG00000084112.15
20365	ENSG00000206195.10	ENSG00000251440.2	ENSG00000232759.1
20366	ENSG00000206199.10	ENSG00000251439.1	ENSG00000255569.1
20367	ENSG00000206203.5	ENSG00000251438.1	ENSG00000102174.9
20368	ENSG00000206228.4	ENSG00000251437.1	ENSG00000234432.4
20369	ENSG00000206249.3	ENSG00000251436.2	ENSG00000268186.1
20370	ENSG00000206260.3	ENSG00000251435.1	ENSG00000198380.12
20371	ENSG00000206262.9	ENSG00000251434.1	ENSG00000102981.9
20372	ENSG00000206337.10	ENSG00000251433.1	ENSG00000007264.15
20373	ENSG00000206341.7	ENSG00000251432.6	ENSG00000212664.5
20374	ENSG00000206344.7	ENSG00000251431.1	ENSG00000119844.15
20375	ENSG00000206356.5	ENSG00000251430.1	ENSG00000119650.12
20376	ENSG00000206384.10	ENSG00000251429.1	ENSG00000104611.12
20377	ENSG00000206417.8	ENSG00000251427.1	ENSG00000274588.2
20378	ENSG00000206418.4	ENSG00000251426.1	ENSG00000138798.12
20379	ENSG00000206422.2	ENSG00000251424.1	ENSG00000173545.5
20380	ENSG00000206432.4	ENSG00000251423.2	ENSG00000249806.1
20381	ENSG00000206448.3	ENSG00000251421.2	ENSG00000163219.11
20382	ENSG00000206474.8	ENSG00000251419.1	ENSG00000135436.8
20383	ENSG00000206503.13	ENSG00000251418.2	ENSG00000140105.18
20384	ENSG00000206527.10	ENSG00000251417.2	ENSG00000255435.6
20385	ENSG00000206530.11	ENSG00000251416.1	ENSG00000125952.19
20386	ENSG00000206531.10	ENSG00000251414.1	ENSG00000156966.7
20387	ENSG00000206532.2	ENSG00000251413.1	ENSG00000197591.3
20388	ENSG00000206535.8	ENSG00000251412.1	ENSG00000273361.1
20389	ENSG00000206536.1	ENSG00000251411.1	ENSG00000230124.8
20390	ENSG00000206538.9	ENSG00000251410.1	ENSG00000233452.6
20391	ENSG00000206549.13	ENSG00000251409.1	ENSG00000108469.15
20392	ENSG00000206552.4	ENSG00000251408.1	ENSG00000214541.3
20393	ENSG00000206557.6	ENSG00000251407.1	ENSG00000109832.14
20394	ENSG00000206559.8	ENSG00000251402.3	ENSG00000284968.1
20395	ENSG00000206560.11	ENSG00000251401.2	ENSG00000111110.12
20396	ENSG00000206561.13	ENSG00000251400.1	ENSG00000274847.1
20397	ENSG00000206562.11	ENSG00000251399.1	ENSG00000104529.17
20398	ENSG00000206567.9	ENSG00000251398.1	ENSG00000189362.12
20399	ENSG00000206573.8	ENSG00000251396.6	ENSG00000154305.17
20400	ENSG00000206579.8	ENSG00000251395.1	ENSG00000171914.16
20401	ENSG00000206582.1	ENSG00000251393.3	ENSG00000278600.1
20402	ENSG00000206583.1	ENSG00000251391.4	ENSG00000121879.5

20403	ENSG00000206585.1	ENSG00000251389.1	ENSG00000235962.5
20404	ENSG00000206587.1	ENSG00000251388.1	ENSG00000247570.2
20405	ENSG00000206588.1	ENSG00000251387.1	ENSG00000185164.14
20406	ENSG00000206589.1	ENSG00000251385.2	ENSG00000267364.1
20407	ENSG00000206590.1	ENSG00000251383.1	ENSG00000130751.9
20408	ENSG00000206592.1	ENSG00000251381.7	ENSG00000271949.1
20409	ENSG00000206593.1	ENSG00000251380.3	ENSG00000130762.15
20410	ENSG00000206595.1	ENSG00000251378.1	ENSG00000231050.1
20411	ENSG00000206596.1	ENSG00000251377.1	ENSG00000214050.8
20412	ENSG00000206597.1	ENSG00000251376.1	ENSG00000183624.14
20413	ENSG00000206598.1	ENSG00000251374.1	ENSG00000119684.15
20414	ENSG00000206599.1	ENSG00000251373.1	ENSG00000164574.16
20415	ENSG00000206600.1	ENSG00000251372.5	ENSG00000017797.13
20416	ENSG00000206601.1	ENSG00000251371.1	ENSG00000143194.13
20417	ENSG00000206602.1	ENSG00000251370.1	ENSG00000164542.12
20418	ENSG00000206603.1	ENSG00000251369.8	ENSG00000235363.1
20419	ENSG00000206604.1	ENSG00000251368.1	ENSG00000275557.1
20420	ENSG00000206605.1	ENSG00000251367.1	ENSG00000124795.16
20421	ENSG00000206606.1	ENSG00000251366.1	ENSG00000278864.1
20422	ENSG00000206609.1	ENSG00000251365.3	ENSG00000268412.2
20423	ENSG00000206611.1	ENSG00000251364.6	ENSG00000103510.20
20424	ENSG00000206612.1	ENSG00000251363.3	ENSG00000167815.12
20425	ENSG00000206613.1	ENSG00000251361.1	ENSG00000283632.2
20426	ENSG00000206614.1	ENSG00000251360.2	ENSG00000277632.2
20427	ENSG00000206615.1	ENSG00000251359.4	ENSG00000265713.1
20428	ENSG00000206616.1	ENSG00000251357.4	ENSG00000240005.5
20429	ENSG00000206617.1	ENSG00000251356.1	ENSG00000163013.11
20430	ENSG00000206618.1	ENSG00000251354.3	ENSG00000149179.13
20431	ENSG00000206620.1	ENSG00000251353.1	ENSG00000235090.1
20432	ENSG00000206621.1	ENSG00000251352.1	ENSG00000253349.1
20433	ENSG00000206622.1	ENSG00000251350.1	ENSG00000115461.5
20434	ENSG00000206623.1	ENSG00000251349.3	ENSG00000100902.10
20435	ENSG00000206624.1	ENSG00000251348.1	ENSG00000168003.16
20436	ENSG00000206625.1	ENSG00000251347.2	ENSG00000243339.3
20437	ENSG00000206627.1	ENSG00000251345.2	ENSG00000243402.1
20438	ENSG00000206629.1	ENSG00000251342.1	ENSG00000237525.6
20439	ENSG00000206630.1	ENSG00000251340.1	ENSG00000207457.1
20440	ENSG00000206631.1	ENSG00000251339.5	ENSG00000276422.1
20441	ENSG00000206633.1	ENSG00000251338.1	ENSG00000224281.4
20442	ENSG00000206634.1	ENSG00000251336.1	ENSG00000237429.1
20443	ENSG00000206635.1	ENSG00000251334.2	ENSG00000036054.13
20444	ENSG00000206636.1	ENSG00000251333.3	ENSG00000136026.14
20445	ENSG00000206637.1	ENSG00000251332.1	ENSG00000211623.2
20446	ENSG00000206638.1	ENSG00000251330.3	ENSG00000166257.8
20447	ENSG00000206639.1	ENSG00000251329.1	ENSG00000218757.1
20448	ENSG00000206640.1	ENSG00000251326.1	ENSG00000232940.5
20449	ENSG00000206641.1	ENSG00000251325.1	ENSG00000276524.1
20450	ENSG00000206644.1	ENSG00000251324.1	ENSG00000267626.1
20451	ENSG00000206645.1	ENSG00000251323.2	ENSG00000068354.16
20452	ENSG00000206646.1	ENSG00000251322.7	ENSG00000280077.1
20453	ENSG00000206647.1	ENSG00000251321.1	ENSG00000106328.9
20454	ENSG00000206649.1	ENSG00000251320.1	ENSG00000218283.2
20455	ENSG00000206650.1	ENSG00000251314.2	ENSG00000244459.2

20456	ENSG00000206651.1	ENSG00000251313.1	ENSG00000198945.7
20457	ENSG00000206652.1	ENSG00000251312.2	ENSG00000108774.14
20458	ENSG00000206654.1	ENSG00000251311.1	ENSG00000152760.10
20459	ENSG00000206656.1	ENSG00000251310.1	ENSG00000134531.10
20460	ENSG00000206658.1	ENSG00000251309.1	ENSG00000280486.1
20461	ENSG00000206659.1	ENSG00000251308.1	ENSG00000275091.1
20462	ENSG00000206660.1	ENSG00000251307.1	ENSG00000285850.1
20463	ENSG00000206661.1	ENSG00000251306.1	ENSG00000274441.1
20464	ENSG00000206662.1	ENSG00000251303.1	ENSG00000277775.1
20465	ENSG00000206663.1	ENSG00000251301.6	ENSG00000267022.1
20466	ENSG00000206665.1	ENSG00000251300.1	ENSG00000280273.2
20467	ENSG00000206669.1	ENSG00000251299.1	ENSG00000217527.1
20468	ENSG00000206671.1	ENSG00000251298.1	ENSG00000148634.15
20469	ENSG00000206672.1	ENSG00000251297.1	ENSG00000164082.15
20470	ENSG00000206674.1	ENSG00000251296.1	ENSG00000175793.12
20471	ENSG00000206675.1	ENSG00000251294.1	ENSG00000164587.13
20472	ENSG00000206676.1	ENSG00000251293.1	ENSG00000262209.3
20473	ENSG00000206677.2	ENSG00000251292.1	ENSG00000175485.2
20474	ENSG00000206678.1	ENSG00000251291.1	ENSG00000274987.1
20475	ENSG00000206679.1	ENSG00000251288.2	ENSG00000213885.3
20476	ENSG00000206680.1	ENSG00000251287.8	ENSG00000255177.2
20477	ENSG00000206681.1	ENSG00000251286.2	ENSG00000115170.13
20478	ENSG00000206682.1	ENSG00000251285.2	ENSG00000109787.13
20479	ENSG00000206684.1	ENSG00000251284.2	ENSG00000251348.1
20480	ENSG00000206685.1	ENSG00000251283.1	ENSG00000178464.6
20481	ENSG00000206686.1	ENSG00000251281.1	ENSG00000130768.15
20482	ENSG00000206687.1	ENSG00000251279.1	ENSG00000185101.13
20483	ENSG00000206688.1	ENSG00000251278.1	ENSG00000253669.3
20484	ENSG00000206690.1	ENSG00000251276.1	ENSG00000180878.2
20485	ENSG00000206692.1	ENSG00000251273.3	ENSG00000230724.9
20486	ENSG00000206693.1	ENSG00000251271.3	ENSG00000179862.6
20487	ENSG00000206695.1	ENSG00000251270.1	ENSG00000271716.1
20488	ENSG00000206697.1	ENSG00000251266.1	ENSG00000258591.2
20489	ENSG00000206698.1	ENSG00000251264.1	ENSG00000134986.13
20490	ENSG00000206699.1	ENSG00000251261.4	ENSG00000151838.12
20491	ENSG00000206700.1	ENSG00000251260.2	ENSG00000248275.1
20492	ENSG00000206701.1	ENSG00000251259.1	ENSG00000165752.17
20493	ENSG00000206702.1	ENSG00000251258.2	ENSG00000163528.13
20494	ENSG00000206703.1	ENSG00000251257.2	ENSG00000230310.1
20495	ENSG00000206704.1	ENSG00000251256.1	ENSG00000228053.1
20496	ENSG00000206705.1	ENSG00000251254.1	ENSG00000246228.6
20497	ENSG00000206706.1	ENSG00000251253.1	ENSG00000185958.9
20498	ENSG00000206708.1	ENSG00000251252.1	ENSG00000197057.9
20499	ENSG00000206709.1	ENSG00000251250.3	ENSG00000173805.15
20500	ENSG00000206710.1	ENSG00000251249.1	ENSG00000272923.1
20501	ENSG00000206711.1	ENSG00000251248.1	ENSG00000112578.10
20502	ENSG00000206712.1	ENSG00000251247.11	ENSG00000237331.1
20503	ENSG00000206713.1	ENSG00000251246.1	ENSG00000262766.1
20504	ENSG00000206714.1	ENSG00000251244.1	ENSG00000272834.1
20505	ENSG00000206715.1	ENSG00000251243.1	ENSG00000229915.1
20506	ENSG00000206716.1	ENSG00000251239.1	ENSG00000130203.10
20507	ENSG00000206717.1	ENSG00000251237.1	ENSG00000230042.1
20508	ENSG00000206718.1	ENSG00000251236.1	ENSG00000243849.1

20509	ENSG00000206719.1	ENSG00000251235.3	ENSG00000233559.1
20510	ENSG00000206721.1	ENSG00000251234.1	ENSG00000104687.14
20511	ENSG00000206722.1	ENSG00000251230.5	ENSG00000218357.3
20512	ENSG00000206723.1	ENSG00000251229.1	ENSG00000266714.9
20513	ENSG00000206724.1	ENSG00000251228.1	ENSG00000187621.14
20514	ENSG00000206725.1	ENSG00000251226.1	ENSG00000196890.4
20515	ENSG00000206726.1	ENSG00000251224.1	ENSG00000243508.1
20516	ENSG00000206727.1	ENSG00000251223.1	ENSG00000227719.1
20517	ENSG00000206728.1	ENSG00000251221.1	ENSG00000252830.2
20518	ENSG00000206729.1	ENSG00000251220.2	ENSG00000183690.13
20519	ENSG00000206730.1	ENSG00000251219.1	ENSG00000173614.14
20520	ENSG00000206731.1	ENSG00000251218.1	ENSG00000237188.5
20521	ENSG00000206732.1	ENSG00000251216.1	ENSG00000113269.14
20522	ENSG00000206733.1	ENSG00000251215.1	ENSG00000249119.1
20523	ENSG00000206734.1	ENSG00000251214.1	ENSG00000138035.15
20524	ENSG00000206737.1	ENSG00000251213.1	ENSG00000214820.3
20525	ENSG00000206738.1	ENSG00000251212.1	ENSG00000215838.4
20526	ENSG00000206739.1	ENSG00000251211.1	ENSG00000231760.5
20527	ENSG00000206741.1	ENSG00000251210.5	ENSG00000254812.1
20528	ENSG00000206743.1	ENSG00000251209.8	ENSG00000011405.13
20529	ENSG00000206744.1	ENSG00000251206.1	ENSG00000167525.13
20530	ENSG00000206745.1	ENSG00000251205.2	ENSG00000279945.1
20531	ENSG00000206746.1	ENSG00000251204.1	ENSG00000226332.2
20532	ENSG00000206747.1	ENSG00000251203.1	ENSG00000276846.1
20533	ENSG00000206749.1	ENSG00000251201.8	ENSG00000203739.3
20534	ENSG00000206751.1	ENSG00000251200.1	ENSG00000169372.13
20535	ENSG00000206752.1	ENSG00000251199.5	ENSG00000256221.1
20536	ENSG00000206754.1	ENSG00000251196.1	ENSG00000135932.11
20537	ENSG00000206755.1	ENSG00000251195.1	ENSG00000205336.11
20538	ENSG00000206756.1	ENSG00000251194.2	ENSG00000227337.1
20539	ENSG00000206758.1	ENSG00000251193.1	ENSG00000255498.1
20540	ENSG00000206759.1	ENSG00000251192.7	ENSG00000273628.1
20541	ENSG00000206760.1	ENSG00000251191.7	ENSG00000118873.16
20542	ENSG00000206762.1	ENSG00000251189.1	ENSG00000279207.1
20543	ENSG00000206763.1	ENSG00000251188.1	ENSG00000285710.1
20544	ENSG00000206764.1	ENSG00000251187.5	ENSG00000226383.6
20545	ENSG00000206765.1	ENSG00000251186.1	ENSG00000278017.1
20546	ENSG00000206766.1	ENSG00000251185.1	ENSG00000239407.5
20547	ENSG00000206767.1	ENSG00000251184.1	ENSG00000101473.17
20548	ENSG00000206768.1	ENSG00000251183.1	ENSG00000109689.16
20549	ENSG00000206769.1	ENSG00000251182.1	ENSG00000228201.1
20550	ENSG00000206770.1	ENSG00000251179.1	ENSG00000105193.9
20551	ENSG00000206772.1	ENSG00000251178.1	ENSG00000185619.18
20552	ENSG00000206774.1	ENSG00000251177.2	ENSG00000113073.15
20553	ENSG00000206775.1	ENSG00000251176.1	ENSG00000197324.9
20554	ENSG00000206776.1	ENSG00000251175.5	ENSG00000197705.9
20555	ENSG00000206777.1	ENSG00000251174.1	ENSG00000155875.15
20556	ENSG00000206778.1	ENSG00000251173.1	ENSG00000104918.8
20557	ENSG00000206779.1	ENSG00000251172.1	ENSG00000206077.11
20558	ENSG00000206780.1	ENSG00000251171.1	ENSG00000169230.10
20559	ENSG00000206781.1	ENSG00000251170.5	ENSG00000254258.1
20560	ENSG00000206782.1	ENSG00000251169.2	ENSG00000250290.1
20561	ENSG00000206784.1	ENSG00000251168.1	ENSG00000146918.19

20562	ENSG00000206785.1	ENSG00000251166.1	ENSG00000171017.11
20563	ENSG00000206786.1	ENSG00000251165.5	ENSG00000199053.3
20564	ENSG00000206787.1	ENSG00000251163.1	ENSG00000228835.1
20565	ENSG00000206788.1	ENSG00000251162.1	ENSG00000155542.11
20566	ENSG00000206790.1	ENSG00000251161.3	ENSG00000151835.16
20567	ENSG00000206791.1	ENSG00000251159.1	ENSG00000263345.1
20568	ENSG00000206792.1	ENSG00000251158.1	ENSG00000285868.1
20569	ENSG00000206795.1	ENSG00000251155.2	ENSG00000278607.1
20570	ENSG00000206796.1	ENSG00000251154.1	ENSG00000279024.1
20571	ENSG00000206797.1	ENSG00000251152.1	ENSG00000215035.2
20572	ENSG00000206799.1	ENSG00000251151.2	ENSG00000128815.19
20573	ENSG00000206800.1	ENSG00000251149.1	ENSG00000133872.13
20574	ENSG00000206801.1	ENSG00000251148.1	ENSG00000260007.3
20575	ENSG00000206802.1	ENSG00000251147.1	ENSG00000196208.14
20576	ENSG00000206803.1	ENSG00000251144.1	ENSG00000250608.1
20577	ENSG00000206804.1	ENSG00000251143.1	ENSG00000136848.17
20578	ENSG00000206805.1	ENSG00000251142.1	ENSG00000118855.19
20579	ENSG00000206806.1	ENSG00000251141.5	ENSG00000177721.4
20580	ENSG00000206807.1	ENSG00000251139.2	ENSG00000127184.13
20581	ENSG00000206808.1	ENSG00000251138.6	ENSG00000168566.13
20582	ENSG00000206811.1	ENSG00000251137.1	ENSG00000171621.14
20583	ENSG00000206812.1	ENSG00000251136.8	ENSG00000203757.2
20584	ENSG00000206813.1	ENSG00000251135.2	ENSG00000235489.4
20585	ENSG00000206814.1	ENSG00000251132.1	ENSG00000235781.1
20586	ENSG00000206815.1	ENSG00000251131.1	ENSG00000136451.9
20587	ENSG00000206816.1	ENSG00000251129.1	ENSG00000179902.12
20588	ENSG00000206817.1	ENSG00000251128.1	ENSG00000059758.8
20589	ENSG00000206818.1	ENSG00000251127.2	ENSG00000272692.1
20590	ENSG00000206819.1	ENSG00000251126.2	ENSG00000120616.15
20591	ENSG00000206820.1	ENSG00000251125.1	ENSG00000218313.1
20592	ENSG00000206822.1	ENSG00000251123.1	ENSG00000261268.1
20593	ENSG00000206824.1	ENSG00000251122.1	ENSG00000198420.10
20594	ENSG00000206826.1	ENSG00000251118.1	ENSG00000099834.18
20595	ENSG00000206827.1	ENSG00000251113.2	ENSG00000137074.18
20596	ENSG00000206828.1	ENSG00000251112.2	ENSG00000187118.13
20597	ENSG00000206832.1	ENSG00000251111.2	ENSG00000231697.3
20598	ENSG00000206833.1	ENSG00000251108.1	ENSG00000240674.1
20599	ENSG00000206834.1	ENSG00000251107.1	ENSG00000286136.1
20600	ENSG00000206835.1	ENSG00000251106.1	ENSG00000129657.16
20601	ENSG00000206836.1	ENSG00000251105.1	ENSG00000124784.9
20602	ENSG00000206838.1	ENSG00000251101.1	ENSG00000250526.1
20603	ENSG00000206839.1	ENSG00000251099.2	ENSG00000180673.9
20604	ENSG00000206840.1	ENSG00000251095.6	ENSG00000213023.11
20605	ENSG00000206841.1	ENSG00000251093.1	ENSG00000211800.3
20606	ENSG00000206842.1	ENSG00000251090.2	ENSG00000243697.1
20607	ENSG00000206843.1	ENSG00000251088.1	ENSG00000236678.7
20608	ENSG00000206844.1	ENSG00000251087.3	ENSG00000225447.1
20609	ENSG00000206845.1	ENSG00000251081.1	ENSG00000175395.16
20610	ENSG00000206846.1	ENSG00000251080.1	ENSG00000178425.14
20611	ENSG00000206847.1	ENSG00000251079.6	ENSG00000165185.14
20612	ENSG00000206848.1	ENSG00000251078.1	ENSG00000259869.2
20613	ENSG00000206849.1	ENSG00000251076.1	ENSG00000244563.1
20614	ENSG00000206850.1	ENSG00000251075.1	ENSG00000081479.14

20615	ENSG00000206852.1	ENSG00000251074.1	ENSG00000204177.10
20616	ENSG00000206853.1	ENSG00000251073.1	ENSG00000276754.1
20617	ENSG00000206854.1	ENSG00000251072.2	ENSG00000163823.4
20618	ENSG00000206855.1	ENSG00000251066.1	ENSG00000136213.10
20619	ENSG00000206857.1	ENSG00000251061.2	ENSG00000258458.6
20620	ENSG00000206858.1	ENSG00000251059.5	ENSG00000175920.18
20621	ENSG00000206859.1	ENSG00000251058.1	ENSG00000173681.16
20622	ENSG00000206862.1	ENSG00000251056.1	ENSG00000163281.11
20623	ENSG00000206863.1	ENSG00000251055.2	ENSG00000229463.2
20624	ENSG00000206864.1	ENSG00000251054.1	ENSG00000269481.1
20625	ENSG00000206865.1	ENSG00000251051.1	ENSG00000075035.10
20626	ENSG00000206866.1	ENSG00000251049.2	ENSG00000253667.2
20627	ENSG00000206867.1	ENSG00000251048.1	ENSG00000250764.1
20628	ENSG00000206869.1	ENSG00000251046.2	ENSG00000268062.1
20629	ENSG00000206870.1	ENSG00000251045.1	ENSG00000184611.11
20630	ENSG00000206871.1	ENSG00000251044.2	ENSG00000163993.7
20631	ENSG00000206875.1	ENSG00000251040.1	ENSG00000261671.1
20632	ENSG00000206877.1	ENSG00000251039.2	ENSG00000228120.2
20633	ENSG00000206878.1	ENSG00000251038.1	ENSG00000259366.1
20634	ENSG00000206880.1	ENSG00000251035.1	ENSG00000136237.18
20635	ENSG00000206881.1	ENSG00000251034.1	ENSG00000163093.12
20636	ENSG00000206882.1	ENSG00000251033.1	ENSG00000236449.1
20637	ENSG00000206885.1	ENSG00000251032.1	ENSG00000259917.1
20638	ENSG00000206886.1	ENSG00000251031.1	ENSG00000100448.4
20639	ENSG00000206887.1	ENSG00000251027.1	ENSG00000173862.3
20640	ENSG00000206888.1	ENSG00000251026.1	ENSG00000261332.1
20641	ENSG00000206889.1	ENSG00000251025.1	ENSG00000232233.1
20642	ENSG00000206891.1	ENSG00000251023.1	ENSG00000257345.2
20643	ENSG00000206892.1	ENSG00000251022.6	ENSG00000236756.4
20644	ENSG00000206895.1	ENSG00000251019.1	ENSG00000215481.9
20645	ENSG00000206896.1	ENSG00000251018.2	ENSG00000238107.1
20646	ENSG00000206897.1	ENSG00000251017.1	ENSG00000160209.19
20647	ENSG00000206898.1	ENSG00000251015.1	ENSG00000256020.1
20648	ENSG00000206899.1	ENSG00000251014.1	ENSG00000224046.1
20649	ENSG00000206900.1	ENSG00000251013.1	ENSG00000184612.8
20650	ENSG00000206901.1	ENSG00000251012.2	ENSG00000223756.6
20651	ENSG00000206903.1	ENSG00000251011.5	ENSG00000248472.8
20652	ENSG00000206905.1	ENSG00000251010.1	ENSG00000267106.6
20653	ENSG00000206906.1	ENSG00000251009.2	ENSG00000135365.15
20654	ENSG00000206907.1	ENSG00000251008.1	ENSG00000239213.5
20655	ENSG00000206908.1	ENSG00000251005.1	ENSG00000132406.12
20656	ENSG00000206909.1	ENSG00000251003.8	ENSG00000260276.2
20657	ENSG00000206910.1	ENSG00000251002.7	ENSG00000142089.16
20658	ENSG00000206911.1	ENSG00000251001.1	ENSG00000259715.1
20659	ENSG00000206912.1	ENSG00000251000.1	ENSG00000212961.4
20660	ENSG00000206913.1	ENSG00000250997.1	ENSG00000271265.1
20661	ENSG00000206914.1	ENSG00000250995.1	ENSG00000188451.8
20662	ENSG00000206915.1	ENSG00000250994.1	ENSG00000270096.1
20663	ENSG00000206917.1	ENSG00000250993.1	ENSG00000230304.1
20664	ENSG00000206918.1	ENSG00000250992.1	ENSG00000156030.13
20665	ENSG00000206920.1	ENSG00000250990.1	ENSG00000213315.5
20666	ENSG00000206921.1	ENSG00000250989.1	ENSG00000174799.10
20667	ENSG00000206922.1	ENSG00000250988.7	ENSG00000256293.2

20668	ENSG00000206923.1	ENSG00000250986.1	ENSG00000167395.10
20669	ENSG00000206924.1	ENSG00000250984.1	ENSG00000278514.1
20670	ENSG00000206925.1	ENSG00000250983.1	ENSG00000124702.18
20671	ENSG00000206926.1	ENSG00000250982.2	ENSG00000227401.1
20672	ENSG00000206927.1	ENSG00000250981.1	ENSG00000196214.11
20673	ENSG00000206929.1	ENSG00000250980.1	ENSG00000248780.1
20674	ENSG00000206931.1	ENSG00000250979.1	ENSG00000232682.2
20675	ENSG00000206932.1	ENSG00000250978.5	ENSG00000135503.13
20676	ENSG00000206935.1	ENSG00000250977.1	ENSG00000234882.1
20677	ENSG00000206936.1	ENSG00000250976.1	ENSG00000147400.8
20678	ENSG00000206937.1	ENSG00000250974.3	ENSG00000203356.2
20679	ENSG00000206938.1	ENSG00000250973.1	ENSG00000070501.12
20680	ENSG00000206939.1	ENSG00000250972.1	ENSG00000142046.15
20681	ENSG00000206941.1	ENSG00000250971.2	ENSG00000220563.1
20682	ENSG00000206944.1	ENSG00000250969.1	ENSG00000106327.13
20683	ENSG00000206947.1	ENSG00000250968.1	ENSG00000074416.14
20684	ENSG00000206948.1	ENSG00000250966.2	ENSG00000100403.12
20685	ENSG00000206949.1	ENSG00000250962.1	ENSG00000102385.12
20686	ENSG00000206950.1	ENSG00000250961.1	ENSG00000268614.1
20687	ENSG00000206951.1	ENSG00000250959.2	ENSG00000135378.4
20688	ENSG00000206952.3	ENSG00000250958.1	ENSG00000184007.21
20689	ENSG00000206954.1	ENSG00000250957.1	ENSG00000022267.17
20690	ENSG00000206957.2	ENSG00000250956.1	ENSG00000230266.1
20691	ENSG00000206958.1	ENSG00000250955.1	ENSG00000153037.14
20692	ENSG00000206959.1	ENSG00000250954.5	ENSG00000223591.5
20693	ENSG00000206960.1	ENSG00000250951.1	ENSG00000243680.1
20694	ENSG00000206961.1	ENSG00000250950.1	ENSG00000284419.1
20695	ENSG00000206962.1	ENSG00000250949.1	ENSG00000177302.15
20696	ENSG00000206963.1	ENSG00000250948.1	ENSG00000164112.13
20697	ENSG00000206964.1	ENSG00000250947.1	ENSG00000137959.16
20698	ENSG00000206965.1	ENSG00000250946.2	ENSG00000274294.1
20699	ENSG00000206967.1	ENSG00000250945.1	ENSG00000148671.13
20700	ENSG00000206969.1	ENSG00000250942.1	ENSG00000167740.9
20701	ENSG00000206970.1	ENSG00000250940.1	ENSG00000284184.1
20702	ENSG00000206972.1	ENSG00000250939.2	ENSG00000275601.1
20703	ENSG00000206973.1	ENSG00000250938.5	ENSG00000114209.15
20704	ENSG00000206974.1	ENSG00000250934.1	ENSG00000122545.19
20705	ENSG00000206975.1	ENSG00000250933.1	ENSG00000270379.5
20706	ENSG00000206976.1	ENSG00000250930.5	ENSG00000145757.16
20707	ENSG00000206977.1	ENSG00000250929.2	ENSG00000275897.1
20708	ENSG00000206978.1	ENSG00000250928.1	ENSG00000239827.8
20709	ENSG00000206979.1	ENSG00000250927.1	ENSG00000226686.7
20710	ENSG00000206980.1	ENSG00000250923.1	ENSG00000182580.3
20711	ENSG00000206981.1	ENSG00000250922.1	ENSG00000268520.1
20712	ENSG00000206982.1	ENSG00000250921.1	ENSG00000239920.2
20713	ENSG00000206983.1	ENSG00000250920.2	ENSG00000158792.16
20714	ENSG00000206985.1	ENSG00000250919.1	ENSG00000115977.19
20715	ENSG00000206987.1	ENSG00000250917.1	ENSG00000223274.6
20716	ENSG00000206989.1	ENSG00000250915.1	ENSG00000147166.11
20717	ENSG00000206990.1	ENSG00000250914.1	ENSG00000270726.6
20718	ENSG00000206991.1	ENSG00000250913.2	ENSG00000260236.1
20719	ENSG00000206992.1	ENSG00000250910.8	ENSG00000259182.5
20720	ENSG00000206995.1	ENSG00000250909.1	ENSG00000277031.1

20721	ENSG00000206996.1	ENSG00000250908.1	ENSG00000138615.6
20722	ENSG00000206997.1	ENSG00000250906.1	ENSG00000204652.6
20723	ENSG00000206998.1	ENSG00000250905.1	ENSG00000277203.1
20724	ENSG00000206999.1	ENSG00000250903.8	ENSG00000143443.9
20725	ENSG00000207000.1	ENSG00000250902.1	ENSG00000279912.1
20726	ENSG00000207001.1	ENSG00000250900.6	ENSG00000175556.16
20727	ENSG00000207002.1	ENSG00000250899.3	ENSG00000047579.19
20728	ENSG00000207003.1	ENSG00000250897.2	ENSG00000091010.6
20729	ENSG00000207004.1	ENSG00000250896.1	ENSG00000204604.11
20730	ENSG00000207005.1	ENSG00000250895.1	ENSG00000268401.1
20731	ENSG00000207007.1	ENSG00000250894.1	ENSG00000196449.4
20732	ENSG00000207008.1	ENSG00000250893.1	ENSG00000167258.13
20733	ENSG00000207009.1	ENSG00000250892.1	ENSG00000110697.13
20734	ENSG00000207010.1	ENSG00000250891.1	ENSG00000255038.1
20735	ENSG00000207011.1	ENSG00000250890.1	ENSG00000099940.12
20736	ENSG00000207012.2	ENSG00000250888.1	ENSG00000244378.1
20737	ENSG00000207013.1	ENSG00000250887.1	ENSG00000260428.3
20738	ENSG00000207014.1	ENSG00000250886.1	ENSG00000269881.1
20739	ENSG00000207016.1	ENSG00000250885.1	ENSG00000232926.1
20740	ENSG00000207019.1	ENSG00000250884.1	ENSG00000162415.7
20741	ENSG00000207020.1	ENSG00000250882.1	ENSG00000266921.1
20742	ENSG00000207021.1	ENSG00000250878.3	ENSG00000189171.14
20743	ENSG00000207022.1	ENSG00000250877.1	ENSG00000230201.5
20744	ENSG00000207023.1	ENSG00000250874.1	ENSG00000244213.1
20745	ENSG00000207024.1	ENSG00000250869.2	ENSG00000273018.6
20746	ENSG00000207025.1	ENSG00000250868.4	ENSG00000225177.5
20747	ENSG00000207026.1	ENSG00000250866.1	ENSG00000131725.14
20748	ENSG00000207027.1	ENSG00000250865.1	ENSG00000205542.11
20749	ENSG00000207029.1	ENSG00000250863.1	ENSG00000163322.14
20750	ENSG00000207031.1	ENSG00000250862.1	ENSG00000257322.5
20751	ENSG00000207032.1	ENSG00000250860.1	ENSG00000242607.1
20752	ENSG00000207033.1	ENSG00000250859.1	ENSG00000279617.1
20753	ENSG00000207034.1	ENSG00000250858.1	ENSG00000267365.1
20754	ENSG00000207036.1	ENSG00000250857.3	ENSG00000213599.10
20755	ENSG00000207037.1	ENSG00000250855.1	ENSG00000166183.16
20756	ENSG00000207039.1	ENSG00000250853.1	ENSG00000284540.1
20757	ENSG00000207041.1	ENSG00000250850.2	ENSG00000185633.10
20758	ENSG00000207042.1	ENSG00000250848.1	ENSG00000138801.9
20759	ENSG00000207044.1	ENSG00000250847.1	ENSG00000270124.1
20760	ENSG00000207045.1	ENSG00000250846.6	ENSG00000253521.2
20761	ENSG00000207046.1	ENSG00000250844.2	ENSG00000260618.1
20762	ENSG00000207047.2	ENSG00000250842.1	ENSG00000099937.11
20763	ENSG00000207049.1	ENSG00000250839.1	ENSG00000244720.1
20764	ENSG00000207051.1	ENSG00000250838.1	ENSG00000261889.1
20765	ENSG00000207052.1	ENSG00000250835.1	ENSG00000230426.3
20766	ENSG00000207053.1	ENSG00000250834.1	ENSG00000233058.1
20767	ENSG00000207056.1	ENSG00000250833.1	ENSG00000186376.15
20768	ENSG00000207058.1	ENSG00000250831.1	ENSG00000183921.7
20769	ENSG00000207060.1	ENSG00000250830.1	ENSG00000258716.1
20770	ENSG00000207061.1	ENSG00000250829.2	ENSG00000274767.1
20771	ENSG00000207062.1	ENSG00000250828.2	ENSG00000265168.1
20772	ENSG00000207063.1	ENSG00000250827.1	ENSG00000250956.1
20773	ENSG00000207065.1	ENSG00000250826.2	ENSG00000240666.2

20774	ENSG00000207067.1	ENSG00000250825.1	ENSG00000273520.5
20775	ENSG00000207068.1	ENSG00000250822.1	ENSG00000077943.8
20776	ENSG00000207069.1	ENSG00000250821.2	ENSG00000197976.12
20777	ENSG00000207071.1	ENSG00000250820.1	ENSG00000178796.12
20778	ENSG00000207072.1	ENSG00000250819.1	ENSG00000243896.4
20779	ENSG00000207073.1	ENSG00000250816.1	ENSG00000187243.16
20780	ENSG00000207075.1	ENSG00000250815.1	ENSG00000197165.11
20781	ENSG00000207076.1	ENSG00000250814.2	ENSG00000259172.1
20782	ENSG00000207077.1	ENSG00000250813.1	ENSG00000260000.2
20783	ENSG00000207080.2	ENSG00000250812.2	ENSG00000160014.16
20784	ENSG00000207081.1	ENSG00000250808.1	ENSG00000125787.11
20785	ENSG00000207082.1	ENSG00000250806.2	ENSG00000173914.12
20786	ENSG00000207083.1	ENSG00000250804.1	ENSG00000221937.4
20787	ENSG00000207084.1	ENSG00000250803.6	ENSG00000044459.15
20788	ENSG00000207086.1	ENSG00000250802.7	ENSG00000205707.10
20789	ENSG00000207087.1	ENSG00000250801.1	ENSG00000179639.10
20790	ENSG00000207088.1	ENSG00000250799.9	ENSG00000116237.16
20791	ENSG00000207089.1	ENSG00000250796.1	ENSG00000204116.11
20792	ENSG00000207090.1	ENSG00000250794.2	ENSG00000228168.1
20793	ENSG00000207091.1	ENSG00000250791.1	ENSG00000175931.13
20794	ENSG00000207092.1	ENSG00000250790.4	ENSG00000254449.1
20795	ENSG00000207093.1	ENSG00000250788.1	ENSG00000280038.1
20796	ENSG00000207094.1	ENSG00000250787.1	ENSG00000139187.10
20797	ENSG00000207095.1	ENSG00000250786.1	ENSG00000175455.15
20798	ENSG00000207097.1	ENSG00000250782.1	ENSG00000147251.15
20799	ENSG00000207098.1	ENSG00000250781.1	ENSG00000159685.10
20800	ENSG00000207099.1	ENSG00000250778.1	ENSG00000267141.1
20801	ENSG00000207100.1	ENSG00000250777.1	ENSG00000233038.6
20802	ENSG00000207101.1	ENSG00000250775.1	ENSG00000228352.2
20803	ENSG00000207104.1	ENSG00000250772.5	ENSG00000147434.8
20804	ENSG00000207105.1	ENSG00000250771.2	ENSG00000131759.18
20805	ENSG00000207108.1	ENSG00000250770.3	ENSG00000083307.11
20806	ENSG00000207109.1	ENSG00000250769.2	ENSG00000264230.9
20807	ENSG00000207110.1	ENSG00000250768.1	ENSG00000253837.1
20808	ENSG00000207112.1	ENSG00000250767.1	ENSG00000267157.1
20809	ENSG00000207113.1	ENSG00000250765.6	ENSG00000216863.9
20810	ENSG00000207114.1	ENSG00000250762.1	ENSG00000285627.1
20811	ENSG00000207115.1	ENSG00000250761.2	ENSG00000228623.6
20812	ENSG00000207116.1	ENSG00000250756.1	ENSG00000159588.15
20813	ENSG00000207117.1	ENSG00000250754.5	ENSG00000101413.11
20814	ENSG00000207118.1	ENSG00000250753.2	ENSG00000196664.5
20815	ENSG00000207119.2	ENSG00000250752.2	ENSG00000206113.10
20816	ENSG00000207121.1	ENSG00000250751.1	ENSG00000242960.1
20817	ENSG00000207122.1	ENSG00000250750.1	ENSG00000269446.2
20818	ENSG00000207123.1	ENSG00000250749.1	ENSG00000035403.17
20819	ENSG00000207124.1	ENSG00000250748.6	ENSG00000180957.17
20820	ENSG00000207127.1	ENSG00000250747.1	ENSG00000211452.10
20821	ENSG00000207128.1	ENSG00000250746.1	ENSG00000269292.1
20822	ENSG00000207129.1	ENSG00000250745.2	ENSG00000259062.2
20823	ENSG00000207130.1	ENSG00000250742.3	ENSG00000187862.11
20824	ENSG00000207131.1	ENSG00000250741.6	ENSG00000164327.13
20825	ENSG00000207132.1	ENSG00000250740.1	ENSG00000167625.10
20826	ENSG00000207133.1	ENSG00000250739.1	ENSG00000272564.1

20827	ENSG00000207134.1	ENSG00000250735.5	ENSG00000257298.1
20828	ENSG00000207135.1	ENSG00000250734.2	ENSG00000115750.17
20829	ENSG00000207136.1	ENSG00000250733.5	ENSG00000178295.15
20830	ENSG00000207137.1	ENSG00000250732.1	ENSG00000166199.13
20831	ENSG00000207138.1	ENSG00000250731.1	ENSG00000186049.8
20832	ENSG00000207139.1	ENSG00000250730.2	ENSG00000160685.13
20833	ENSG00000207142.1	ENSG00000250728.1	ENSG00000153066.12
20834	ENSG00000207144.1	ENSG00000250727.1	ENSG00000114013.16
20835	ENSG00000207145.1	ENSG00000250726.1	ENSG00000178021.11
20836	ENSG00000207146.1	ENSG00000250725.2	ENSG00000103226.18
20837	ENSG00000207147.1	ENSG00000250723.6	ENSG00000149922.10
20838	ENSG00000207148.1	ENSG00000250722.6	ENSG00000226439.3
20839	ENSG00000207149.1	ENSG00000250721.1	ENSG00000266010.2
20840	ENSG00000207150.1	ENSG00000250719.1	ENSG00000272763.1
20841	ENSG00000207151.1	ENSG00000250716.1	ENSG00000250644.3
20842	ENSG00000207153.1	ENSG00000250715.1	ENSG00000261557.1
20843	ENSG00000207154.1	ENSG00000250714.3	ENSG00000232295.7
20844	ENSG00000207155.1	ENSG00000250712.1	ENSG00000225213.2
20845	ENSG00000207156.1	ENSG00000250711.1	ENSG00000085721.12
20846	ENSG00000207157.1	ENSG00000250710.3	ENSG00000273443.1
20847	ENSG00000207158.1	ENSG00000250709.1	ENSG00000279672.1
20848	ENSG00000207160.1	ENSG00000250708.1	ENSG00000233627.2
20849	ENSG00000207161.1	ENSG00000250706.1	ENSG00000279330.1
20850	ENSG00000207162.1	ENSG00000250705.1	ENSG00000277925.1
20851	ENSG00000207163.1	ENSG00000250704.1	ENSG00000226625.1
20852	ENSG00000207164.1	ENSG00000250703.1	ENSG00000256745.1
20853	ENSG00000207165.1	ENSG00000250699.1	ENSG00000158296.14
20854	ENSG00000207166.1	ENSG00000250698.1	ENSG00000144589.22
20855	ENSG00000207167.1	ENSG00000250697.1	ENSG00000143570.18
20856	ENSG00000207168.1	ENSG00000250696.5	ENSG00000188585.9
20857	ENSG00000207169.1	ENSG00000250694.1	ENSG00000060069.16
20858	ENSG00000207170.1	ENSG00000250693.2	ENSG00000223973.2
20859	ENSG00000207171.1	ENSG00000250692.1	ENSG00000176624.11
20860	ENSG00000207172.1	ENSG00000250688.1	ENSG00000233554.5
20861	ENSG00000207173.1	ENSG00000250687.6	ENSG00000100888.13
20862	ENSG00000207174.1	ENSG00000250686.2	ENSG00000271228.1
20863	ENSG00000207175.1	ENSG00000250685.7	ENSG00000232970.1
20864	ENSG00000207176.1	ENSG00000250684.4	ENSG00000279803.1
20865	ENSG00000207177.1	ENSG00000250682.5	ENSG00000268529.1
20866	ENSG00000207178.1	ENSG00000250681.1	ENSG00000204560.10
20867	ENSG00000207180.1	ENSG00000250678.1	ENSG00000128829.12
20868	ENSG00000207181.1	ENSG00000250677.1	ENSG00000253298.1
20869	ENSG00000207182.1	ENSG00000250674.1	ENSG00000269400.1
20870	ENSG00000207183.1	ENSG00000250673.2	ENSG00000108405.4
20871	ENSG00000207185.1	ENSG00000250672.1	ENSG00000236383.8
20872	ENSG00000207186.1	ENSG00000250670.1	ENSG00000238042.5
20873	ENSG00000207187.1	ENSG00000250669.1	ENSG00000259242.2
20874	ENSG00000207189.1	ENSG00000250668.1	ENSG00000182197.11
20875	ENSG00000207190.1	ENSG00000250667.1	ENSG00000139719.10
20876	ENSG00000207191.1	ENSG00000250666.1	ENSG00000257595.2
20877	ENSG00000207192.1	ENSG00000250665.1	ENSG00000256597.2
20878	ENSG00000207193.1	ENSG00000250662.1	ENSG00000165678.21
20879	ENSG00000207194.1	ENSG00000250659.2	ENSG00000196593.9

20880	ENSG00000207195.1	ENSG00000250658.1	ENSG00000143537.13
20881	ENSG00000207196.1	ENSG00000250657.1	ENSG00000214144.3
20882	ENSG00000207197.1	ENSG00000250656.2	ENSG00000163221.9
20883	ENSG00000207198.1	ENSG00000250655.1	ENSG00000261582.1
20884	ENSG00000207199.1	ENSG00000250654.7	ENSG00000270903.1
20885	ENSG00000207200.1	ENSG00000250650.1	ENSG00000272799.1
20886	ENSG00000207201.1	ENSG00000250646.1	ENSG00000036257.13
20887	ENSG00000207202.1	ENSG00000250645.1	ENSG00000237301.1
20888	ENSG00000207203.1	ENSG00000250644.3	ENSG00000269066.1
20889	ENSG00000207204.1	ENSG00000250643.1	ENSG00000188641.13
20890	ENSG00000207205.1	ENSG00000250642.3	ENSG00000156395.13
20891	ENSG00000207207.1	ENSG00000250641.1	ENSG00000138639.18
20892	ENSG00000207208.1	ENSG00000250640.2	ENSG00000174652.19
20893	ENSG00000207209.1	ENSG00000250637.2	ENSG00000233999.3
20894	ENSG00000207210.1	ENSG00000250636.1	ENSG00000159409.14
20895	ENSG00000207214.1	ENSG00000250635.1	ENSG00000270553.1
20896	ENSG00000207215.2	ENSG00000250634.5	ENSG00000243678.11
20897	ENSG00000207217.1	ENSG00000250632.1	ENSG00000107263.18
20898	ENSG00000207218.1	ENSG00000250630.1	ENSG00000247853.2
20899	ENSG00000207220.1	ENSG00000250629.1	ENSG00000247708.7
20900	ENSG00000207221.1	ENSG00000250627.2	ENSG00000102057.10
20901	ENSG00000207222.1	ENSG00000250626.2	ENSG00000236279.7
20902	ENSG00000207223.1	ENSG00000250625.1	ENSG00000105997.22
20903	ENSG00000207225.1	ENSG00000250624.1	ENSG00000177990.11
20904	ENSG00000207227.1	ENSG00000250623.1	ENSG00000267501.1
20905	ENSG00000207229.1	ENSG00000250622.1	ENSG00000036448.10
20906	ENSG00000207231.1	ENSG00000250620.1	ENSG00000146830.10
20907	ENSG00000207233.1	ENSG00000250619.1	ENSG00000259483.1
20908	ENSG00000207234.1	ENSG00000250618.1	ENSG00000162398.11
20909	ENSG00000207235.1	ENSG00000250615.1	ENSG00000111785.20
20910	ENSG00000207237.1	ENSG00000250614.1	ENSG00000226220.1
20911	ENSG00000207240.1	ENSG00000250613.1	ENSG00000105988.6
20912	ENSG00000207241.1	ENSG00000250612.2	ENSG00000237025.1
20913	ENSG00000207242.1	ENSG00000250611.1	ENSG00000286194.1
20914	ENSG00000207243.1	ENSG00000250609.1	ENSG00000111816.8
20915	ENSG00000207244.1	ENSG00000250608.1	ENSG00000078549.14
20916	ENSG00000207245.1	ENSG00000250604.1	ENSG00000163141.19
20917	ENSG00000207247.1	ENSG00000250603.1	ENSG00000071909.18
20918	ENSG00000207248.1	ENSG00000250602.6	ENSG00000237324.3
20919	ENSG00000207249.1	ENSG00000250600.1	ENSG00000265479.6
20920	ENSG00000207251.1	ENSG00000250597.5	ENSG00000222835.1
20921	ENSG00000207252.1	ENSG00000250596.2	ENSG00000283773.1
20922	ENSG00000207255.1	ENSG00000250594.2	ENSG00000283361.2
20923	ENSG00000207256.1	ENSG00000250592.1	ENSG00000185475.11
20924	ENSG00000207257.1	ENSG00000250591.3	ENSG00000260682.3
20925	ENSG00000207258.1	ENSG00000250590.5	ENSG00000135063.19
20926	ENSG00000207260.1	ENSG00000250587.2	ENSG00000140090.17
20927	ENSG00000207261.1	ENSG00000250585.2	ENSG00000177469.13
20928	ENSG00000207263.1	ENSG00000250584.2	ENSG00000267633.1
20929	ENSG00000207264.1	ENSG00000250583.1	ENSG00000243468.5
20930	ENSG00000207265.1	ENSG00000250582.1	ENSG00000109971.14
20931	ENSG00000207266.1	ENSG00000250580.1	ENSG00000204084.13
20932	ENSG00000207267.1	ENSG00000250579.1	ENSG00000223619.1

20933	ENSG00000207268.1	ENSG00000250577.1	ENSG00000165996.14
20934	ENSG00000207269.1	ENSG00000250576.1	ENSG00000121851.13
20935	ENSG00000207270.1	ENSG00000250575.1	ENSG00000241525.4
20936	ENSG00000207271.1	ENSG00000250574.1	ENSG00000157510.14
20937	ENSG00000207274.1	ENSG00000250573.1	ENSG00000234241.1
20938	ENSG00000207275.1	ENSG00000250572.1	ENSG00000229052.2
20939	ENSG00000207276.1	ENSG00000250571.7	ENSG00000224186.8
20940	ENSG00000207277.1	ENSG00000250569.1	ENSG00000126603.8
20941	ENSG00000207278.1	ENSG00000250568.1	ENSG00000250903.8
20942	ENSG00000207279.1	ENSG00000250567.1	ENSG00000057757.10
20943	ENSG00000207280.1	ENSG00000250566.1	ENSG00000101150.17
20944	ENSG00000207281.1	ENSG00000250565.7	ENSG00000137834.15
20945	ENSG00000207282.1	ENSG00000250564.1	ENSG00000237217.1
20946	ENSG00000207283.1	ENSG00000250563.1	ENSG00000213542.3
20947	ENSG00000207286.1	ENSG00000250562.1	ENSG00000189014.7
20948	ENSG00000207287.1	ENSG00000250561.2	ENSG00000236498.1
20949	ENSG00000207289.1	ENSG00000250560.2	ENSG00000183960.9
20950	ENSG00000207290.1	ENSG00000250556.1	ENSG00000134917.10
20951	ENSG00000207291.1	ENSG00000250555.2	ENSG00000213872.3
20952	ENSG00000207292.1	ENSG00000250551.1	ENSG00000115353.11
20953	ENSG00000207293.1	ENSG00000250550.3	ENSG00000233111.1
20954	ENSG00000207294.1	ENSG00000250548.6	ENSG00000230172.1
20955	ENSG00000207295.1	ENSG00000250547.1	ENSG00000153823.18
20956	ENSG00000207296.1	ENSG00000250546.5	ENSG00000135074.15
20957	ENSG00000207297.1	ENSG00000250544.1	ENSG00000270159.1
20958	ENSG00000207298.1	ENSG00000250543.1	ENSG00000215595.1
20959	ENSG00000207299.1	ENSG00000250541.1	ENSG00000129951.18
20960	ENSG00000207300.1	ENSG00000250540.1	ENSG00000233231.1
20961	ENSG00000207302.1	ENSG00000250539.1	ENSG00000215196.4
20962	ENSG00000207303.1	ENSG00000250538.5	ENSG00000285932.1
20963	ENSG00000207304.1	ENSG00000250536.1	ENSG00000134291.12
20964	ENSG00000207305.1	ENSG00000250532.1	ENSG00000118960.13
20965	ENSG00000207306.1	ENSG00000250529.1	ENSG00000134684.10
20966	ENSG00000207307.1	ENSG00000250526.1	ENSG00000216331.2
20967	ENSG00000207308.1	ENSG00000250524.1	ENSG00000277840.1
20968	ENSG00000207309.1	ENSG00000250523.1	ENSG00000129204.16
20969	ENSG00000207310.1	ENSG00000250522.1	ENSG00000184110.14
20970	ENSG00000207312.1	ENSG00000250519.6	ENSG00000248540.2
20971	ENSG00000207313.1	ENSG00000250517.2	ENSG00000124145.6
20972	ENSG00000207316.1	ENSG00000250516.1	ENSG00000164867.11
20973	ENSG00000207317.1	ENSG00000250515.1	ENSG00000157578.13
20974	ENSG00000207318.1	ENSG00000250514.1	ENSG00000275558.1
20975	ENSG00000207319.1	ENSG00000250511.1	ENSG00000188522.15
20976	ENSG00000207320.1	ENSG00000250510.8	ENSG00000211815.3
20977	ENSG00000207321.1	ENSG00000250509.1	ENSG00000077721.16
20978	ENSG00000207322.1	ENSG00000250508.1	ENSG00000241838.3
20979	ENSG00000207323.1	ENSG00000250507.1	ENSG00000114867.20
20980	ENSG00000207325.1	ENSG00000250506.7	ENSG00000253327.2
20981	ENSG00000207326.1	ENSG00000250505.1	ENSG00000160233.8
20982	ENSG00000207327.1	ENSG00000250504.2	ENSG00000257941.1
20983	ENSG00000207328.1	ENSG00000250503.1	ENSG00000267695.1
20984	ENSG00000207329.1	ENSG00000250501.2	ENSG00000260388.2
20985	ENSG00000207330.1	ENSG00000250500.1	ENSG00000185133.14

20986	ENSG00000207331.1	ENSG00000250497.1	ENSG00000274742.1
20987	ENSG00000207332.1	ENSG00000250496.2	ENSG00000140543.14
20988	ENSG00000207333.1	ENSG00000250494.1	ENSG00000261884.2
20989	ENSG00000207334.1	ENSG00000250493.2	ENSG00000151348.13
20990	ENSG00000207336.1	ENSG00000250492.1	ENSG00000162227.8
20991	ENSG00000207338.1	ENSG00000250490.1	ENSG00000273348.1
20992	ENSG00000207340.1	ENSG00000250488.1	ENSG00000231827.3
20993	ENSG00000207341.1	ENSG00000250486.4	ENSG00000276566.1
20994	ENSG00000207342.1	ENSG00000250485.1	ENSG00000260803.1
20995	ENSG00000207343.1	ENSG00000250484.2	ENSG00000254479.1
20996	ENSG00000207344.1	ENSG00000250483.1	ENSG00000105996.7
20997	ENSG00000207345.1	ENSG00000250482.3	ENSG00000230013.1
20998	ENSG00000207347.1	ENSG00000250481.1	ENSG00000188042.8
20999	ENSG00000207349.1	ENSG00000250480.3	ENSG00000123091.5
21000	ENSG00000207351.1	ENSG00000250479.8	ENSG00000130612.15
21001	ENSG00000207352.1	ENSG00000250476.1	ENSG00000132688.11
21002	ENSG00000207356.1	ENSG00000250475.1	ENSG00000144134.18
21003	ENSG00000207357.1	ENSG00000250474.2	ENSG00000111058.8
21004	ENSG00000207359.1	ENSG00000250473.1	ENSG00000156127.6
21005	ENSG00000207360.1	ENSG00000250472.1	ENSG00000230470.1
21006	ENSG00000207361.1	ENSG00000250471.2	ENSG00000279276.1
21007	ENSG00000207362.1	ENSG00000250470.1	ENSG00000236065.2
21008	ENSG00000207363.1	ENSG00000250467.1	ENSG00000189419.7
21009	ENSG00000207364.1	ENSG00000250464.1	ENSG00000258891.1
21010	ENSG00000207365.1	ENSG00000250462.8	ENSG00000167614.13
21011	ENSG00000207366.1	ENSG00000250461.1	ENSG00000134262.13
21012	ENSG00000207367.1	ENSG00000250458.2	ENSG00000275055.1
21013	ENSG00000207368.1	ENSG00000250456.1	ENSG00000259137.1
21014	ENSG00000207369.1	ENSG00000250455.1	ENSG00000232871.8
21015	ENSG00000207370.1	ENSG00000250453.1	ENSG00000213228.5
21016	ENSG00000207371.1	ENSG00000250451.5	ENSG00000286118.1
21017	ENSG00000207375.1	ENSG00000250448.1	ENSG00000272760.1
21018	ENSG00000207378.1	ENSG00000250447.5	ENSG00000102178.13
21019	ENSG00000207379.1	ENSG00000250446.1	ENSG00000260368.1
21020	ENSG00000207380.1	ENSG00000250444.1	ENSG00000171714.11
21021	ENSG00000207381.1	ENSG00000250442.1	ENSG00000055070.17
21022	ENSG00000207382.1	ENSG00000250441.1	ENSG00000251203.1
21023	ENSG00000207383.1	ENSG00000250438.1	ENSG00000172014.12
21024	ENSG00000207384.1	ENSG00000250437.1	ENSG00000259107.1
21025	ENSG00000207385.1	ENSG00000250436.1	ENSG00000145817.17
21026	ENSG00000207386.1	ENSG00000250433.1	ENSG00000119725.18
21027	ENSG00000207387.1	ENSG00000250432.5	ENSG00000064601.18
21028	ENSG00000207389.1	ENSG00000250431.1	ENSG00000261556.9
21029	ENSG00000207391.1	ENSG00000250430.1	ENSG00000203416.3
21030	ENSG00000207392.1	ENSG00000250428.1	ENSG00000036828.16
21031	ENSG00000207393.1	ENSG00000250427.1	ENSG00000198780.12
21032	ENSG00000207394.1	ENSG00000250426.2	ENSG00000222385.1
21033	ENSG00000207395.1	ENSG00000250425.1	ENSG00000170468.7
21034	ENSG00000207397.1	ENSG00000250424.4	ENSG00000167693.17
21035	ENSG00000207399.1	ENSG00000250423.2	ENSG00000269693.1
21036	ENSG00000207401.1	ENSG00000250422.1	ENSG00000108448.21
21037	ENSG00000207402.1	ENSG00000250421.5	ENSG00000111679.17
21038	ENSG00000207403.1	ENSG00000250420.8	ENSG00000230946.3

21039	ENSG00000207404.1	ENSG00000250418.1	ENSG00000135999.12
21040	ENSG00000207405.1	ENSG00000250417.1	ENSG00000286127.1
21041	ENSG00000207406.1	ENSG00000250416.2	ENSG00000224072.1
21042	ENSG00000207407.1	ENSG00000250415.1	ENSG00000268222.1
21043	ENSG00000207408.1	ENSG00000250413.1	ENSG00000099917.17
21044	ENSG00000207410.1	ENSG00000250412.1	ENSG00000161132.6
21045	ENSG00000207411.1	ENSG00000250410.1	ENSG00000239419.3
21046	ENSG00000207412.1	ENSG00000250409.1	ENSG00000239793.1
21047	ENSG00000207414.1	ENSG00000250407.1	ENSG00000278974.1
21048	ENSG00000207415.1	ENSG00000250406.1	ENSG00000283345.1
21049	ENSG00000207416.1	ENSG00000250405.2	ENSG00000257596.1
21050	ENSG00000207417.1	ENSG00000250403.1	ENSG00000283863.1
21051	ENSG00000207419.1	ENSG00000250402.2	ENSG00000124541.7
21052	ENSG00000207420.1	ENSG00000250398.1	ENSG00000212391.1
21053	ENSG00000207421.1	ENSG00000250397.2	ENSG00000099954.18
21054	ENSG00000207422.1	ENSG00000250393.1	ENSG00000272831.1
21055	ENSG00000207425.1	ENSG00000250392.2	ENSG00000164332.8
21056	ENSG00000207426.1	ENSG00000250391.1	ENSG00000109321.11
21057	ENSG00000207428.1	ENSG00000250390.2	ENSG00000269924.1
21058	ENSG00000207430.1	ENSG00000250389.1	ENSG00000272068.1
21059	ENSG00000207431.2	ENSG00000250387.2	ENSG00000255185.5
21060	ENSG00000207432.1	ENSG00000250386.1	ENSG00000130005.12
21061	ENSG00000207433.1	ENSG00000250385.2	ENSG00000126860.11
21062	ENSG00000207434.1	ENSG00000250384.1	ENSG00000169495.5
21063	ENSG00000207435.1	ENSG00000250383.1	ENSG00000279659.1
21064	ENSG00000207438.1	ENSG00000250381.1	ENSG00000203644.3
21065	ENSG00000207439.1	ENSG00000250379.1	ENSG00000251175.5
21066	ENSG00000207440.1	ENSG00000250378.3	ENSG00000141404.16
21067	ENSG00000207441.1	ENSG00000250377.1	ENSG00000273188.1
21068	ENSG00000207443.1	ENSG00000250376.1	ENSG00000151090.18
21069	ENSG00000207444.1	ENSG00000250375.2	ENSG00000236469.1
21070	ENSG00000207445.1	ENSG00000250372.1	ENSG00000278887.2
21071	ENSG00000207448.1	ENSG00000250371.1	ENSG00000205763.13
21072	ENSG00000207449.1	ENSG00000250366.2	ENSG00000139223.2
21073	ENSG00000207450.1	ENSG00000250365.6	ENSG00000167535.8
21074	ENSG00000207451.1	ENSG00000250363.1	ENSG00000069956.12
21075	ENSG00000207452.1	ENSG00000250362.1	ENSG00000125804.13
21076	ENSG00000207453.1	ENSG00000250361.8	ENSG00000227155.7
21077	ENSG00000207454.1	ENSG00000250360.1	ENSG00000177885.15
21078	ENSG00000207455.1	ENSG00000250359.1	ENSG00000078319.9
21079	ENSG00000207456.1	ENSG00000250358.5	ENSG00000265408.1
21080	ENSG00000207457.1	ENSG00000250357.1	ENSG00000132915.11
21081	ENSG00000207458.1	ENSG00000250356.1	ENSG00000228278.4
21082	ENSG00000207459.1	ENSG00000250354.1	ENSG00000224387.1
21083	ENSG00000207460.1	ENSG00000250351.1	ENSG00000260549.1
21084	ENSG00000207461.1	ENSG00000250350.1	ENSG00000137338.5
21085	ENSG00000207462.1	ENSG00000250349.3	ENSG00000270820.5
21086	ENSG00000207466.1	ENSG00000250348.1	ENSG00000282639.1
21087	ENSG00000207467.1	ENSG00000250347.1	ENSG00000182253.14
21088	ENSG00000207468.1	ENSG00000250346.1	ENSG00000261043.5
21089	ENSG00000207472.1	ENSG00000250345.2	ENSG00000227073.1
21090	ENSG00000207473.1	ENSG00000250344.1	ENSG00000188493.15
21091	ENSG00000207474.1	ENSG00000250343.1	ENSG00000267143.1

21092	ENSG00000207475.1	ENSG00000250342.1	ENSG00000162032.16
21093	ENSG00000207476.1	ENSG00000250341.1	ENSG00000230873.8
21094	ENSG00000207480.1	ENSG00000250340.1	ENSG00000235012.1
21095	ENSG00000207481.1	ENSG00000250339.2	ENSG00000133740.11
21096	ENSG00000207483.1	ENSG00000250338.1	ENSG00000106714.17
21097	ENSG00000207484.1	ENSG00000250337.6	ENSG00000174564.13
21098	ENSG00000207486.1	ENSG00000250334.5	ENSG00000143155.13
21099	ENSG00000207488.1	ENSG00000250333.2	ENSG00000115540.15
21100	ENSG00000207490.1	ENSG00000250332.1	ENSG00000137776.17
21101	ENSG00000207492.1	ENSG00000250331.1	ENSG00000212124.2
21102	ENSG00000207493.1	ENSG00000250330.1	ENSG00000225408.1
21103	ENSG00000207495.1	ENSG00000250329.1	ENSG00000105963.15
21104	ENSG00000207496.1	ENSG00000250328.5	ENSG00000226252.1
21105	ENSG00000207497.1	ENSG00000250327.1	ENSG00000170089.15
21106	ENSG00000207499.1	ENSG00000250326.1	ENSG00000265478.2
21107	ENSG00000207500.1	ENSG00000250325.1	ENSG00000284194.1
21108	ENSG00000207501.1	ENSG00000250324.2	ENSG00000136367.14
21109	ENSG00000207502.1	ENSG00000250322.2	ENSG00000225294.1
21110	ENSG00000207503.1	ENSG00000250321.1	ENSG00000280007.1
21111	ENSG00000207504.1	ENSG00000250320.5	ENSG00000231993.1
21112	ENSG00000207505.1	ENSG00000250319.1	ENSG00000258796.1
21113	ENSG00000207507.1	ENSG00000250318.1	ENSG00000105617.3
21114	ENSG00000207508.1	ENSG00000250317.8	ENSG00000277411.1
21115	ENSG00000207511.1	ENSG00000250316.1	ENSG00000116489.13
21116	ENSG00000207512.1	ENSG00000250315.2	ENSG00000267323.1
21117	ENSG00000207513.1	ENSG00000250313.2	ENSG00000244538.1
21118	ENSG00000207515.1	ENSG00000250310.2	ENSG00000131389.17
21119	ENSG00000207516.1	ENSG00000250309.2	ENSG00000241357.1
21120	ENSG00000207518.1	ENSG00000250308.2	ENSG00000277324.1
21121	ENSG00000207523.1	ENSG00000250307.2	ENSG00000221994.10
21122	ENSG00000207524.1	ENSG00000250306.1	ENSG00000278899.1
21123	ENSG00000207525.1	ENSG00000250305.9	ENSG00000227033.1
21124	ENSG00000207546.1	ENSG00000250304.2	ENSG00000183762.12
21125	ENSG00000207547.1	ENSG00000250303.3	ENSG00000261587.2
21126	ENSG00000207548.3	ENSG00000250302.2	ENSG00000117713.20
21127	ENSG00000207549.1	ENSG00000250300.1	ENSG00000158042.8
21128	ENSG00000207550.1	ENSG00000250299.6	ENSG00000234684.6
21129	ENSG00000207551.1	ENSG00000250298.4	ENSG00000244273.1
21130	ENSG00000207552.1	ENSG00000250295.6	ENSG00000236264.5
21131	ENSG00000207554.1	ENSG00000250293.1	ENSG00000144868.13
21132	ENSG00000207559.1	ENSG00000250292.1	ENSG00000228543.1
21133	ENSG00000207561.1	ENSG00000250290.1	ENSG00000101474.12
21134	ENSG00000207562.3	ENSG00000250289.1	ENSG00000271553.1
21135	ENSG00000207563.1	ENSG00000250286.1	ENSG00000169251.12
21136	ENSG00000207568.1	ENSG00000250284.2	ENSG00000196353.11
21137	ENSG00000207569.2	ENSG00000250282.1	ENSG00000080608.10
21138	ENSG00000207571.1	ENSG00000250280.2	ENSG00000231859.1
21139	ENSG00000207573.1	ENSG00000250277.2	ENSG00000263080.1
21140	ENSG00000207574.1	ENSG00000250273.1	ENSG00000270103.3
21141	ENSG00000207575.1	ENSG00000250272.1	ENSG00000166343.10
21142	ENSG00000207577.1	ENSG00000250271.2	ENSG00000173678.14
21143	ENSG00000207578.1	ENSG00000250268.3	ENSG00000273149.1
21144	ENSG00000207579.1	ENSG00000250267.2	ENSG00000073605.18

21145	ENSG00000207580.1	ENSG00000250266.1	ENSG00000223855.1
21146	ENSG00000207581.1	ENSG00000250264.1	ENSG00000163584.18
21147	ENSG00000207582.1	ENSG00000250263.1	ENSG00000279569.1
21148	ENSG00000207583.1	ENSG00000250261.1	ENSG00000230953.2
21149	ENSG00000207585.1	ENSG00000250260.1	ENSG00000234825.3
21150	ENSG00000207586.1	ENSG00000250259.1	ENSG00000270921.1
21151	ENSG00000207587.1	ENSG00000250258.1	ENSG00000260442.5
21152	ENSG00000207588.1	ENSG00000250257.1	ENSG00000124491.15
21153	ENSG00000207589.1	ENSG00000250256.1	ENSG00000265784.1
21154	ENSG00000207590.1	ENSG00000250254.2	ENSG00000250222.1
21155	ENSG00000207594.1	ENSG00000250253.3	ENSG00000285991.1
21156	ENSG00000207595.1	ENSG00000250252.1	ENSG00000232479.2
21157	ENSG00000207597.1	ENSG00000250251.6	ENSG00000279489.1
21158	ENSG00000207598.1	ENSG00000250250.1	ENSG00000205578.6
21159	ENSG00000207599.1	ENSG00000250249.2	ENSG00000114757.19
21160	ENSG00000207600.3	ENSG00000250247.1	ENSG00000277654.6
21161	ENSG00000207604.3	ENSG00000250244.5	ENSG00000155090.15
21162	ENSG00000207605.3	ENSG00000250243.2	ENSG00000255097.1
21163	ENSG00000207606.1	ENSG00000250242.1	ENSG00000228079.2
21164	ENSG00000207607.3	ENSG00000250241.5	ENSG00000284167.1
21165	ENSG00000207608.3	ENSG00000250238.1	ENSG00000087053.18
21166	ENSG00000207609.3	ENSG00000250237.1	ENSG00000183604.14
21167	ENSG00000207611.1	ENSG00000250234.1	ENSG00000230882.1
21168	ENSG00000207612.1	ENSG00000250231.1	ENSG00000266385.1
21169	ENSG00000207613.1	ENSG00000250230.2	ENSG00000233695.2
21170	ENSG00000207614.1	ENSG00000250229.1	ENSG00000256229.8
21171	ENSG00000207615.3	ENSG00000250227.1	ENSG00000139266.6
21172	ENSG00000207616.1	ENSG00000250223.1	ENSG00000233536.2
21173	ENSG00000207617.4	ENSG00000250222.1	ENSG00000279785.1
21174	ENSG00000207619.1	ENSG00000250221.1	ENSG00000179750.16
21175	ENSG00000207620.1	ENSG00000250220.1	ENSG00000077463.15
21176	ENSG00000207622.1	ENSG00000250219.2	ENSG00000123119.12
21177	ENSG00000207624.1	ENSG00000250218.1	ENSG00000270885.1
21178	ENSG00000207625.1	ENSG00000250215.1	ENSG00000112081.17
21179	ENSG00000207626.1	ENSG00000250214.1	ENSG00000259162.1
21180	ENSG00000207627.1	ENSG00000250213.2	ENSG00000270964.1
21181	ENSG00000207628.3	ENSG00000250210.5	ENSG00000266445.1
21182	ENSG00000207629.1	ENSG00000250208.6	ENSG00000258365.1
21183	ENSG00000207630.1	ENSG00000250207.1	ENSG00000230793.1
21184	ENSG00000207631.1	ENSG00000250205.1	ENSG00000179818.13
21185	ENSG00000207632.1	ENSG00000250204.2	ENSG00000172900.12
21186	ENSG00000207633.3	ENSG00000250202.2	ENSG00000197448.14
21187	ENSG00000207634.1	ENSG00000250200.1	ENSG00000027644.5
21188	ENSG00000207635.1	ENSG00000250198.2	ENSG00000248333.8
21189	ENSG00000207637.1	ENSG00000250197.1	ENSG00000153560.12
21190	ENSG00000207638.1	ENSG00000250195.1	ENSG00000278175.3
21191	ENSG00000207639.1	ENSG00000250194.1	ENSG00000170956.17
21192	ENSG00000207641.1	ENSG00000250193.1	ENSG00000259291.2
21193	ENSG00000207642.1	ENSG00000250192.1	ENSG00000104970.11
21194	ENSG00000207644.1	ENSG00000250191.1	ENSG00000186198.4
21195	ENSG00000207645.2	ENSG00000250190.1	ENSG00000263206.1
21196	ENSG00000207646.1	ENSG00000250189.2	ENSG00000279268.1
21197	ENSG00000207647.1	ENSG00000250186.3	ENSG00000143363.17

21198	ENSG00000207649.3	ENSG00000250185.2	ENSG00000096080.11
21199	ENSG00000207650.1	ENSG00000250183.2	ENSG00000102738.7
21200	ENSG00000207651.1	ENSG00000250182.3	ENSG00000051825.14
21201	ENSG00000207652.1	ENSG00000250180.1	ENSG00000248905.8
21202	ENSG00000207653.1	ENSG00000250174.5	ENSG00000154645.14
21203	ENSG00000207654.4	ENSG00000250173.1	ENSG00000118508.5
21204	ENSG00000207656.1	ENSG00000250170.1	ENSG00000240489.1
21205	ENSG00000207688.2	ENSG00000250169.1	ENSG00000274227.1
21206	ENSG00000207689.1	ENSG00000250167.1	ENSG00000241945.8
21207	ENSG00000207691.1	ENSG00000250166.2	ENSG00000128918.15
21208	ENSG00000207692.3	ENSG00000250164.1	ENSG00000285558.1
21209	ENSG00000207693.1	ENSG00000250162.1	ENSG00000131126.18
21210	ENSG00000207695.1	ENSG00000250161.1	ENSG00000274080.1
21211	ENSG00000207696.1	ENSG00000250158.1	ENSG00000180581.7
21212	ENSG00000207697.1	ENSG00000250156.3	ENSG00000233538.1
21213	ENSG00000207698.1	ENSG00000250155.1	ENSG00000130254.12
21214	ENSG00000207699.1	ENSG00000250151.9	ENSG00000233690.1
21215	ENSG00000207701.1	ENSG00000250150.1	ENSG00000272808.4
21216	ENSG00000207702.1	ENSG00000250149.1	ENSG00000183077.15
21217	ENSG00000207703.1	ENSG00000250148.1	ENSG00000226329.2
21218	ENSG00000207704.2	ENSG00000250147.1	ENSG00000267383.6
21219	ENSG00000207705.2	ENSG00000250145.1	ENSG00000072135.13
21220	ENSG00000207706.1	ENSG00000250141.1	ENSG00000234637.1
21221	ENSG00000207708.1	ENSG00000250140.1	ENSG00000143061.17
21222	ENSG00000207711.1	ENSG00000250138.4	ENSG00000237877.6
21223	ENSG00000207712.1	ENSG00000250137.1	ENSG00000243491.1
21224	ENSG00000207713.3	ENSG00000250135.1	ENSG00000275719.1
21225	ENSG00000207714.3	ENSG00000250133.2	ENSG00000175575.12
21226	ENSG00000207716.1	ENSG00000250132.6	ENSG00000081087.15
21227	ENSG00000207717.1	ENSG00000250131.1	ENSG00000276030.1
21228	ENSG00000207719.1	ENSG00000250130.1	ENSG00000248476.1
21229	ENSG00000207721.1	ENSG00000250129.5	ENSG00000248485.2
21230	ENSG00000207722.1	ENSG00000250127.1	ENSG00000157350.13
21231	ENSG00000207725.3	ENSG00000250126.1	ENSG00000254997.3
21232	ENSG00000207726.3	ENSG00000250125.3	ENSG00000253456.1
21233	ENSG00000207728.1	ENSG00000250124.1	ENSG00000180383.3
21234	ENSG00000207729.3	ENSG00000250122.1	ENSG00000135925.9
21235	ENSG00000207730.3	ENSG00000250120.7	ENSG00000189325.7
21236	ENSG00000207731.1	ENSG00000250118.1	ENSG00000139410.15
21237	ENSG00000207732.1	ENSG00000250116.2	ENSG00000203392.3
21238	ENSG00000207734.1	ENSG00000250115.2	ENSG00000176014.12
21239	ENSG00000207735.1	ENSG00000250114.1	ENSG00000256713.7
21240	ENSG00000207736.1	ENSG00000250111.3	ENSG00000206384.10
21241	ENSG00000207737.1	ENSG00000250107.1	ENSG00000279176.1
21242	ENSG00000207738.1	ENSG00000250106.1	ENSG00000273153.1
21243	ENSG00000207739.1	ENSG00000250105.1	ENSG00000224606.1
21244	ENSG00000207741.1	ENSG00000250103.2	ENSG00000111665.11
21245	ENSG00000207742.3	ENSG00000250102.5	ENSG00000279286.2
21246	ENSG00000207743.3	ENSG00000250101.1	ENSG00000201315.1
21247	ENSG00000207744.1	ENSG00000250100.2	ENSG00000254884.1
21248	ENSG00000207746.1	ENSG00000250099.1	ENSG00000274297.1
21249	ENSG00000207749.4	ENSG00000250098.1	ENSG00000255108.1
21250	ENSG00000207750.1	ENSG00000250095.5	ENSG00000255474.1

21251	ENSG00000207751.3	ENSG00000250092.2	ENSG00000110218.9
21252	ENSG00000207752.1	ENSG00000250090.1	ENSG00000285162.1
21253	ENSG00000207754.3	ENSG00000250088.1	ENSG00000121380.12
21254	ENSG00000207755.3	ENSG00000250084.1	ENSG00000082641.16
21255	ENSG00000207756.1	ENSG00000250082.1	ENSG00000163082.10
21256	ENSG00000207757.1	ENSG00000250081.1	ENSG00000232188.1
21257	ENSG00000207758.1	ENSG00000250080.1	ENSG00000178342.4
21258	ENSG00000207759.1	ENSG00000250079.1	ENSG00000145743.15
21259	ENSG00000207761.3	ENSG00000250078.1	ENSG00000170827.9
21260	ENSG00000207762.1	ENSG00000250076.1	ENSG00000243056.2
21261	ENSG00000207763.1	ENSG00000250075.6	ENSG00000260382.1
21262	ENSG00000207766.1	ENSG00000250074.1	ENSG00000249680.2
21263	ENSG00000207767.3	ENSG00000250073.2	ENSG00000123908.12
21264	ENSG00000207768.3	ENSG00000250072.5	ENSG00000237541.3
21265	ENSG00000207769.1	ENSG00000250071.1	ENSG00000101542.10
21266	ENSG00000207771.1	ENSG00000250069.1	ENSG00000103037.11
21267	ENSG00000207773.1	ENSG00000250068.1	ENSG00000274139.1
21268	ENSG00000207775.1	ENSG00000250066.1	ENSG00000261766.1
21269	ENSG00000207776.1	ENSG00000250064.1	ENSG00000261786.1
21270	ENSG00000207778.3	ENSG00000250062.5	ENSG00000278532.1
21271	ENSG00000207779.1	ENSG00000250060.1	ENSG00000105866.15
21272	ENSG00000207780.1	ENSG00000250057.1	ENSG00000183971.8
21273	ENSG00000207781.1	ENSG00000250056.6	ENSG00000205885.7
21274	ENSG00000207782.3	ENSG00000250053.1	ENSG00000172236.17
21275	ENSG00000207784.3	ENSG00000250050.1	ENSG00000180730.4
21276	ENSG00000207785.1	ENSG00000250049.6	ENSG00000171311.12
21277	ENSG00000207788.1	ENSG00000250048.2	ENSG00000204315.4
21278	ENSG00000207789.1	ENSG00000250046.3	ENSG00000206150.4
21279	ENSG00000207797.1	ENSG00000250043.1	ENSG00000105373.19
21280	ENSG00000207798.1	ENSG00000250042.1	ENSG00000271147.7
21281	ENSG00000207799.1	ENSG00000250041.2	ENSG00000039560.14
21282	ENSG00000207800.3	ENSG00000250040.2	ENSG00000238009.6
21283	ENSG00000207802.1	ENSG00000250039.3	ENSG00000282798.1
21284	ENSG00000207803.1	ENSG00000250038.6	ENSG00000236908.2
21285	ENSG00000207804.1	ENSG00000250037.1	ENSG00000134962.7
21286	ENSG00000207805.3	ENSG00000250036.1	ENSG00000222005.9
21287	ENSG00000207807.1	ENSG00000250034.1	ENSG00000182944.17
21288	ENSG00000207808.1	ENSG00000250033.5	ENSG00000253558.1
21289	ENSG00000207810.1	ENSG00000250032.1	ENSG00000232973.12
21290	ENSG00000207811.3	ENSG00000250031.1	ENSG00000127954.12
21291	ENSG00000207813.1	ENSG00000250030.2	ENSG00000151655.19
21292	ENSG00000207814.1	ENSG00000250027.1	ENSG00000169169.14
21293	ENSG00000207815.1	ENSG00000250026.5	ENSG00000132912.12
21294	ENSG00000207816.1	ENSG00000250025.2	ENSG00000264456.1
21295	ENSG00000207818.1	ENSG00000250024.1	ENSG00000205078.5
21296	ENSG00000207820.1	ENSG00000250021.7	ENSG00000157703.15
21297	ENSG00000207821.1	ENSG00000250020.1	ENSG00000223725.6
21298	ENSG00000207825.1	ENSG00000250017.1	ENSG00000138030.13
21299	ENSG00000207826.1	ENSG00000250016.2	ENSG00000203724.11
21300	ENSG00000207827.1	ENSG00000250015.1	ENSG00000278816.1
21301	ENSG00000207837.1	ENSG00000250013.1	ENSG00000178935.5
21302	ENSG00000207838.1	ENSG00000250012.1	ENSG00000104432.14
21303	ENSG00000207839.1	ENSG00000250011.1	ENSG00000249140.1

21304	ENSG00000207861.1	ENSG00000250007.6	ENSG00000114383.10
21305	ENSG00000207862.1	ENSG00000250006.2	ENSG00000268105.1
21306	ENSG00000207863.1	ENSG00000250001.1	ENSG00000185272.14
21307	ENSG00000207864.3	ENSG00000249998.1	ENSG00000227676.3
21308	ENSG00000207866.1	ENSG00000249997.1	ENSG00000228285.2
21309	ENSG00000207867.1	ENSG00000249996.1	ENSG00000101194.18
21310	ENSG00000207868.1	ENSG00000249995.1	ENSG00000223396.4
21311	ENSG00000207869.1	ENSG00000249994.1	ENSG00000271390.1
21312	ENSG00000207870.1	ENSG00000249993.1	ENSG00000134851.13
21313	ENSG00000207871.2	ENSG00000249992.1	ENSG00000211640.4
21314	ENSG00000207873.1	ENSG00000249990.1	ENSG00000237214.3
21315	ENSG00000207874.1	ENSG00000249988.1	ENSG00000177138.16
21316	ENSG00000207922.1	ENSG00000249987.1	ENSG00000273702.1
21317	ENSG00000207923.1	ENSG00000249986.1	ENSG00000260953.1
21318	ENSG00000207924.3	ENSG00000249985.1	ENSG00000021826.15
21319	ENSG00000207925.1	ENSG00000249984.1	ENSG00000278867.1
21320	ENSG00000207926.1	ENSG00000249982.1	ENSG00000171798.18
21321	ENSG00000207927.1	ENSG00000249981.1	ENSG00000285672.1
21322	ENSG00000207930.1	ENSG00000249978.1	ENSG00000145990.11
21323	ENSG00000207931.1	ENSG00000249977.1	ENSG00000274124.1
21324	ENSG00000207932.1	ENSG00000249976.2	ENSG00000109016.17
21325	ENSG00000207933.3	ENSG00000249975.1	ENSG00000144407.9
21326	ENSG00000207934.1	ENSG00000249973.2	ENSG00000232630.1
21327	ENSG00000207935.3	ENSG00000249971.1	ENSG00000164556.7
21328	ENSG00000207938.1	ENSG00000249970.1	ENSG00000261441.1
21329	ENSG00000207940.1	ENSG00000249967.1	ENSG00000163812.14
21330	ENSG00000207941.1	ENSG00000249966.1	ENSG00000211942.3
21331	ENSG00000207942.2	ENSG00000249965.2	ENSG00000022976.15
21332	ENSG00000207943.1	ENSG00000249963.1	ENSG00000274461.1
21333	ENSG00000207944.1	ENSG00000249962.1	ENSG00000197971.15
21334	ENSG00000207946.3	ENSG00000249961.9	ENSG00000089335.21
21335	ENSG00000207947.3	ENSG00000249960.2	ENSG00000099840.13
21336	ENSG00000207948.3	ENSG00000249959.5	ENSG00000123609.10
21337	ENSG00000207951.1	ENSG00000249958.1	ENSG00000182551.14
21338	ENSG00000207952.1	ENSG00000249956.4	ENSG00000279738.1
21339	ENSG00000207954.1	ENSG00000249955.1	ENSG00000095370.20
21340	ENSG00000207955.5	ENSG00000249951.1	ENSG00000101412.13
21341	ENSG00000207956.1	ENSG00000249950.1	ENSG00000267895.1
21342	ENSG00000207957.1	ENSG00000249948.6	ENSG00000279481.1
21343	ENSG00000207959.1	ENSG00000249947.2	ENSG00000131730.16
21344	ENSG00000207960.1	ENSG00000249945.1	ENSG00000236782.7
21345	ENSG00000207961.3	ENSG00000249944.1	ENSG00000249084.1
21346	ENSG00000207962.1	ENSG00000249943.1	ENSG00000091879.13
21347	ENSG00000207963.1	ENSG00000249942.1	ENSG00000230146.1
21348	ENSG00000207965.1	ENSG00000249941.1	ENSG00000164904.18
21349	ENSG00000207967.1	ENSG00000249937.7	ENSG00000267080.5
21350	ENSG00000207969.1	ENSG00000249936.3	ENSG00000139531.13
21351	ENSG00000207970.3	ENSG00000249934.2	ENSG00000134001.13
21352	ENSG00000207971.1	ENSG00000249930.1	ENSG00000229456.1
21353	ENSG00000207972.1	ENSG00000249928.2	ENSG00000143742.13
21354	ENSG00000207973.1	ENSG00000249927.1	ENSG00000269918.1
21355	ENSG00000207974.1	ENSG00000249926.2	ENSG00000130303.13
21356	ENSG00000207975.1	ENSG00000249924.2	ENSG00000253736.2

21357	ENSG00000207976.1	ENSG00000249921.2	ENSG00000285571.1
21358	ENSG00000207978.1	ENSG00000249920.2	ENSG00000171401.15
21359	ENSG00000207979.1	ENSG00000249919.1	ENSG00000136807.13
21360	ENSG00000207980.1	ENSG00000249917.2	ENSG00000251562.8
21361	ENSG00000207981.1	ENSG00000249916.1	ENSG00000178146.9
21362	ENSG00000207982.1	ENSG00000249915.8	ENSG00000188512.6
21363	ENSG00000207983.1	ENSG00000249912.1	ENSG00000181061.13
21364	ENSG00000207984.1	ENSG00000249910.3	ENSG00000225057.2
21365	ENSG00000207987.1	ENSG00000249909.1	ENSG00000211663.2
21366	ENSG00000207988.1	ENSG00000249908.2	ENSG00000246082.2
21367	ENSG00000207989.3	ENSG00000249906.1	ENSG00000250129.5
21368	ENSG00000207990.3	ENSG00000249904.1	ENSG00000197168.13
21369	ENSG00000207991.1	ENSG00000249901.1	ENSG00000103351.13
21370	ENSG00000207992.1	ENSG00000249899.5	ENSG00000256407.2
21371	ENSG00000207993.3	ENSG00000249898.7	ENSG00000151553.15
21372	ENSG00000207994.1	ENSG00000249896.2	ENSG00000231305.3
21373	ENSG00000207995.1	ENSG00000249894.1	ENSG00000230872.1
21374	ENSG00000207996.1	ENSG00000249893.1	ENSG00000258446.1
21375	ENSG00000207997.1	ENSG00000249892.1	ENSG00000180964.17
21376	ENSG00000208000.1	ENSG00000249891.2	ENSG00000197915.6
21377	ENSG00000208001.1	ENSG00000249890.1	ENSG00000236753.6
21378	ENSG00000208002.1	ENSG00000249889.1	ENSG00000232124.2
21379	ENSG00000208003.1	ENSG00000249888.1	ENSG00000162777.17
21380	ENSG00000208004.2	ENSG00000249887.1	ENSG00000213068.3
21381	ENSG00000208005.1	ENSG00000249885.1	ENSG00000261136.1
21382	ENSG00000208006.1	ENSG00000249884.8	ENSG00000106266.11
21383	ENSG00000208008.1	ENSG00000249883.1	ENSG00000269940.1
21384	ENSG00000208009.1	ENSG00000249882.1	ENSG00000156886.11
21385	ENSG00000208012.1	ENSG00000249881.1	ENSG00000272509.1
21386	ENSG00000208013.3	ENSG00000249878.1	ENSG00000179593.16
21387	ENSG00000208014.1	ENSG00000249877.1	ENSG00000282393.1
21388	ENSG00000208015.1	ENSG00000249876.1	ENSG00000139180.11
21389	ENSG00000208017.3	ENSG00000249875.1	ENSG00000133246.12
21390	ENSG00000208018.1	ENSG00000249873.6	ENSG00000164050.13
21391	ENSG00000208022.1	ENSG00000249870.1	ENSG00000259508.1
21392	ENSG00000208023.3	ENSG00000249869.1	ENSG00000185689.16
21393	ENSG00000208024.1	ENSG00000249868.5	ENSG00000144362.12
21394	ENSG00000208025.1	ENSG00000249867.5	ENSG00000279465.1
21395	ENSG00000208027.4	ENSG00000249866.1	ENSG00000224034.1
21396	ENSG00000208028.1	ENSG00000249865.1	ENSG00000166974.12
21397	ENSG00000208032.1	ENSG00000249863.2	ENSG00000273059.1
21398	ENSG00000208033.1	ENSG00000249861.4	ENSG00000278766.2
21399	ENSG00000208036.1	ENSG00000249860.3	ENSG00000224533.4
21400	ENSG00000208037.1	ENSG00000249859.10	ENSG00000283117.1
21401	ENSG00000208308.1	ENSG00000249858.2	ENSG00000182759.3
21402	ENSG00000208772.1	ENSG00000249857.1	ENSG00000184058.14
21403	ENSG00000208839.1	ENSG00000249856.1	ENSG00000255337.1
21404	ENSG00000208883.1	ENSG00000249855.1	ENSG00000270806.2
21405	ENSG00000208892.1	ENSG00000249854.2	ENSG00000224992.2
21406	ENSG00000209042.1	ENSG00000249853.7	ENSG00000008323.15
21407	ENSG00000209082.1	ENSG00000249852.1	ENSG00000072182.12
21408	ENSG00000209480.1	ENSG00000249851.1	ENSG00000105676.14
21409	ENSG00000209482.1	ENSG00000249850.1	ENSG00000269037.1

21410	ENSG00000209582.1	ENSG00000249849.1	ENSG00000144290.17
21411	ENSG00000209645.1	ENSG00000249848.1	ENSG00000228594.4
21412	ENSG00000209702.1	ENSG00000249847.1	ENSG00000278638.1
21413	ENSG00000210049.1	ENSG00000249846.6	ENSG00000145354.12
21414	ENSG00000210077.1	ENSG00000249844.1	ENSG00000198355.5
21415	ENSG00000210082.2	ENSG00000249843.1	ENSG00000108039.18
21416	ENSG00000210100.1	ENSG00000249842.1	ENSG00000256888.5
21417	ENSG00000210107.1	ENSG00000249840.2	ENSG00000107317.13
21418	ENSG00000210112.1	ENSG00000249839.1	ENSG00000115112.8
21419	ENSG00000210117.1	ENSG00000249838.1	ENSG00000180998.12
21420	ENSG00000210127.1	ENSG00000249837.1	ENSG00000177699.4
21421	ENSG00000210135.1	ENSG00000249835.2	ENSG00000134769.21
21422	ENSG00000210140.1	ENSG00000249834.3	ENSG00000009954.11
21423	ENSG00000210144.1	ENSG00000249833.6	ENSG00000254777.5
21424	ENSG00000210151.2	ENSG00000249831.2	ENSG00000234431.2
21425	ENSG00000210154.1	ENSG00000249830.1	ENSG00000271964.1
21426	ENSG00000210156.1	ENSG00000249829.2	ENSG00000185052.11
21427	ENSG00000210164.1	ENSG00000249828.2	ENSG00000182986.13
21428	ENSG00000210174.1	ENSG00000249825.5	ENSG00000064763.11
21429	ENSG00000210176.1	ENSG00000249820.1	ENSG00000232891.1
21430	ENSG00000210181.1	ENSG00000249819.1	ENSG00000228175.3
21431	ENSG00000210184.1	ENSG00000249818.1	ENSG00000188687.17
21432	ENSG00000210194.1	ENSG00000249816.6	ENSG00000258917.1
21433	ENSG00000210195.2	ENSG00000249815.1	ENSG00000213549.3
21434	ENSG00000210196.2	ENSG00000249811.2	ENSG00000231494.1
21435	ENSG00000210678.1	ENSG00000249808.2	ENSG00000267197.1
21436	ENSG00000210709.1	ENSG00000249807.1	ENSG00000229717.2
21437	ENSG00000210741.1	ENSG00000249806.1	ENSG00000164296.7
21438	ENSG00000210825.1	ENSG00000249803.5	ENSG00000188295.14
21439	ENSG00000210839.1	ENSG00000249799.1	ENSG00000231884.1
21440	ENSG00000210841.1	ENSG00000249798.1	ENSG00000249459.8
21441	ENSG00000211137.1	ENSG00000249797.1	ENSG00000223863.1
21442	ENSG00000211445.12	ENSG00000249795.1	ENSG00000280429.1
21443	ENSG00000211448.11	ENSG00000249792.1	ENSG00000196576.15
21444	ENSG00000211450.10	ENSG00000249791.1	ENSG00000259781.1
21445	ENSG00000211451.12	ENSG00000249790.2	ENSG00000212464.1
21446	ENSG00000211452.10	ENSG00000249787.1	ENSG00000249471.8
21447	ENSG00000211454.14	ENSG00000249786.7	ENSG00000046653.15
21448	ENSG00000211455.8	ENSG00000249785.2	ENSG00000260220.6
21449	ENSG00000211456.11	ENSG00000249784.1	ENSG00000227051.6
21450	ENSG00000211459.2	ENSG00000249782.1	ENSG00000100983.11
21451	ENSG00000211460.12	ENSG00000249781.6	ENSG00000229851.1
21452	ENSG00000211491.2	ENSG00000249780.1	ENSG00000103472.10
21453	ENSG00000211513.5	ENSG00000249779.1	ENSG00000258568.1
21454	ENSG00000211514.1	ENSG00000249778.1	ENSG00000153443.13
21455	ENSG00000211520.2	ENSG00000249777.1	ENSG00000146842.17
21456	ENSG00000211532.1	ENSG00000249776.5	ENSG00000163017.13
21457	ENSG00000211538.1	ENSG00000249774.1	ENSG00000227543.4
21458	ENSG00000211543.2	ENSG00000249773.3	ENSG00000283122.1
21459	ENSG00000211563.4	ENSG00000249772.1	ENSG00000145555.15
21460	ENSG00000211568.1	ENSG00000249771.1	ENSG00000154556.18
21461	ENSG00000211574.1	ENSG00000249770.1	ENSG00000225684.3
21462	ENSG00000211575.3	ENSG00000249768.2	ENSG00000243517.1

21463	ENSG00000211578.3	ENSG00000249767.1	ENSG00000230581.2
21464	ENSG00000211579.2	ENSG00000249766.1	ENSG00000276412.2
21465	ENSG00000211580.1	ENSG00000249764.1	ENSG00000110693.17
21466	ENSG00000211581.1	ENSG00000249763.1	ENSG00000228399.1
21467	ENSG00000211582.1	ENSG00000249761.1	ENSG00000213291.3
21468	ENSG00000211583.3	ENSG00000249755.2	ENSG00000065675.14
21469	ENSG00000211584.14	ENSG00000249754.2	ENSG00000285774.1
21470	ENSG00000211590.1	ENSG00000249753.2	ENSG00000232499.2
21471	ENSG00000211591.1	ENSG00000249752.1	ENSG00000260105.6
21472	ENSG00000211592.8	ENSG00000249751.4	ENSG00000105518.14
21473	ENSG00000211593.2	ENSG00000249748.1	ENSG00000229723.1
21474	ENSG00000211594.2	ENSG00000249747.1	ENSG00000272647.3
21475	ENSG00000211595.2	ENSG00000249746.1	ENSG00000130638.17
21476	ENSG00000211596.3	ENSG00000249745.1	ENSG00000147118.11
21477	ENSG00000211597.2	ENSG00000249744.1	ENSG00000164124.10
21478	ENSG00000211598.2	ENSG00000249743.5	ENSG00000230183.4
21479	ENSG00000211599.2	ENSG00000249742.1	ENSG00000250413.1
21480	ENSG00000211611.2	ENSG00000249741.2	ENSG00000228236.2
21481	ENSG00000211623.2	ENSG00000249740.2	ENSG00000115457.10
21482	ENSG00000211625.2	ENSG00000249738.10	ENSG00000273218.1
21483	ENSG00000211626.2	ENSG00000249737.1	ENSG00000176236.6
21484	ENSG00000211632.4	ENSG00000249736.1	ENSG00000257660.5
21485	ENSG00000211633.3	ENSG00000249735.1	ENSG00000283234.1
21486	ENSG00000211637.2	ENSG00000249731.1	ENSG00000243953.1
21487	ENSG00000211638.2	ENSG00000249730.1	ENSG00000273962.1
21488	ENSG00000211639.2	ENSG00000249729.2	ENSG00000214413.8
21489	ENSG00000211640.4	ENSG00000249727.1	ENSG00000223523.1
21490	ENSG00000211641.3	ENSG00000249726.1	ENSG00000218631.1
21491	ENSG00000211642.3	ENSG00000249725.1	ENSG00000185168.5
21492	ENSG00000211643.2	ENSG00000249722.1	ENSG00000068137.15
21493	ENSG00000211644.3	ENSG00000249721.1	ENSG00000234630.1
21494	ENSG00000211645.2	ENSG00000249717.1	ENSG00000272661.1
21495	ENSG00000211647.1	ENSG00000249715.12	ENSG00000184293.7
21496	ENSG00000211648.2	ENSG00000249713.1	ENSG00000225640.1
21497	ENSG00000211649.3	ENSG00000249710.1	ENSG00000186787.8
21498	ENSG00000211650.2	ENSG00000249709.8	ENSG00000124701.5
21499	ENSG00000211651.3	ENSG00000249708.1	ENSG00000247121.6
21500	ENSG00000211652.2	ENSG00000249706.1	ENSG00000145022.4
21501	ENSG00000211653.2	ENSG00000249700.8	ENSG00000148943.12
21502	ENSG00000211654.2	ENSG00000249699.1	ENSG00000276057.1
21503	ENSG00000211655.3	ENSG00000249698.1	ENSG00000141579.7
21504	ENSG00000211656.3	ENSG00000249697.1	ENSG00000250696.5
21505	ENSG00000211657.3	ENSG00000249695.6	ENSG00000228960.6
21506	ENSG00000211658.2	ENSG00000249693.2	ENSG00000237718.2
21507	ENSG00000211659.2	ENSG00000249692.1	ENSG00000120509.10
21508	ENSG00000211660.3	ENSG00000249691.1	ENSG00000227827.3
21509	ENSG00000211661.2	ENSG00000249690.1	ENSG00000173200.13
21510	ENSG00000211662.2	ENSG00000249689.1	ENSG00000272858.1
21511	ENSG00000211663.2	ENSG00000249688.1	ENSG00000064489.22
21512	ENSG00000211664.3	ENSG00000249686.1	ENSG00000269019.1
21513	ENSG00000211665.3	ENSG00000249685.1	ENSG00000138459.9
21514	ENSG00000211666.2	ENSG00000249684.5	ENSG00000275532.1
21515	ENSG00000211667.3	ENSG00000249681.2	ENSG00000266473.1

21516	ENSG00000211668.2	ENSG00000249680.2	ENSG00000185651.14
21517	ENSG00000211669.3	ENSG00000249679.1	ENSG00000232915.1
21518	ENSG00000211670.2	ENSG00000249678.1	ENSG00000231898.8
21519	ENSG00000211672.2	ENSG00000249675.1	ENSG00000240435.2
21520	ENSG00000211673.2	ENSG00000249673.6	ENSG00000105479.15
21521	ENSG00000211674.2	ENSG00000249669.9	ENSG00000284987.1
21522	ENSG00000211675.2	ENSG00000249668.1	ENSG00000121316.11
21523	ENSG00000211676.2	ENSG00000249667.1	ENSG00000161643.12
21524	ENSG00000211677.2	ENSG00000249664.1	ENSG00000196436.8
21525	ENSG00000211678.2	ENSG00000249662.6	ENSG00000177156.11
21526	ENSG00000211679.2	ENSG00000249661.1	ENSG00000108312.15
21527	ENSG00000211680.2	ENSG00000249658.1	ENSG00000112799.9
21528	ENSG00000211681.2	ENSG00000249655.1	ENSG00000226754.1
21529	ENSG00000211682.2	ENSG00000249654.1	ENSG00000148426.13
21530	ENSG00000211683.3	ENSG00000249650.1	ENSG00000119147.10
21531	ENSG00000211684.2	ENSG00000249649.1	ENSG00000235802.1
21532	ENSG00000211685.3	ENSG00000249647.2	ENSG00000254093.9
21533	ENSG00000211687.1	ENSG00000249646.2	ENSG00000105426.16
21534	ENSG00000211688.1	ENSG00000249642.1	ENSG00000255587.9
21535	ENSG00000211689.7	ENSG00000249641.2	ENSG00000197858.11
21536	ENSG00000211690.1	ENSG00000249639.1	ENSG00000285565.1
21537	ENSG00000211691.2	ENSG00000249638.1	ENSG00000127955.17
21538	ENSG00000211692.1	ENSG00000249637.1	ENSG00000155868.8
21539	ENSG00000211693.2	ENSG00000249635.1	ENSG00000138780.14
21540	ENSG00000211694.2	ENSG00000249634.1	ENSG00000225062.1
21541	ENSG00000211695.2	ENSG00000249633.1	ENSG00000278396.1
21542	ENSG00000211696.2	ENSG00000249631.5	ENSG00000082212.13
21543	ENSG00000211697.4	ENSG00000249628.3	ENSG00000286086.1
21544	ENSG00000211698.2	ENSG00000249627.1	ENSG00000179934.6
21545	ENSG00000211699.2	ENSG00000249626.1	ENSG00000260001.6
21546	ENSG00000211701.2	ENSG00000249624.9	ENSG00000228495.1
21547	ENSG00000211706.2	ENSG00000249623.1	ENSG00000069696.6
21548	ENSG00000211707.3	ENSG00000249621.1	ENSG00000132432.14
21549	ENSG00000211710.3	ENSG00000249620.1	ENSG00000242574.9
21550	ENSG00000211713.3	ENSG00000249619.1	ENSG00000174136.12
21551	ENSG00000211714.3	ENSG00000249618.5	ENSG00000074657.13
21552	ENSG00000211715.1	ENSG00000249617.1	ENSG00000110090.13
21553	ENSG00000211716.2	ENSG00000249616.1	ENSG00000026559.14
21554	ENSG00000211717.3	ENSG00000249614.1	ENSG00000105793.15
21555	ENSG00000211720.3	ENSG00000249613.1	ENSG00000224207.3
21556	ENSG00000211721.2	ENSG00000249610.1	ENSG00000164684.13
21557	ENSG00000211724.3	ENSG00000249609.1	ENSG00000277400.1
21558	ENSG00000211725.3	ENSG00000249607.1	ENSG00000146205.13
21559	ENSG00000211727.3	ENSG00000249606.1	ENSG00000197935.6
21560	ENSG00000211728.2	ENSG00000249605.1	ENSG00000152102.18
21561	ENSG00000211731.1	ENSG00000249604.1	ENSG00000126602.11
21562	ENSG00000211734.3	ENSG00000249602.1	ENSG00000196126.11
21563	ENSG00000211739.4	ENSG00000249601.2	ENSG00000113749.7
21564	ENSG00000211745.3	ENSG00000249600.1	ENSG00000248831.2
21565	ENSG00000211746.3	ENSG00000249599.1	ENSG00000133104.14
21566	ENSG00000211747.3	ENSG00000249593.6	ENSG00000278367.1
21567	ENSG00000211749.1	ENSG00000249592.5	ENSG00000279281.1
21568	ENSG00000211750.2	ENSG00000249590.7	ENSG00000280828.1

21569	ENSG00000211751.9	ENSG00000249588.1	ENSG00000203499.11
21570	ENSG00000211752.3	ENSG00000249584.1	ENSG00000175604.2
21571	ENSG00000211753.4	ENSG00000249582.1	ENSG00000005486.17
21572	ENSG00000211764.1	ENSG00000249581.2	ENSG00000284829.1
21573	ENSG00000211765.1	ENSG00000249580.1	ENSG00000226396.1
21574	ENSG00000211766.1	ENSG00000249579.2	ENSG00000261000.1
21575	ENSG00000211767.1	ENSG00000249577.1	ENSG00000059588.10
21576	ENSG00000211768.1	ENSG00000249574.1	ENSG00000251186.1
21577	ENSG00000211769.1	ENSG00000249568.1	ENSG00000154874.15
21578	ENSG00000211770.1	ENSG00000249564.1	ENSG00000250506.7
21579	ENSG00000211771.1	ENSG00000249563.1	ENSG00000187730.9
21580	ENSG00000211772.11	ENSG00000249562.1	ENSG00000267065.2
21581	ENSG00000211776.2	ENSG00000249558.1	ENSG00000230736.2
21582	ENSG00000211777.2	ENSG00000249557.2	ENSG00000225783.7
21583	ENSG00000211778.2	ENSG00000249555.2	ENSG00000013573.17
21584	ENSG00000211779.3	ENSG00000249553.1	ENSG00000265936.1
21585	ENSG00000211780.3	ENSG00000249551.1	ENSG00000272447.1
21586	ENSG00000211781.3	ENSG00000249550.6	ENSG00000139890.10
21587	ENSG00000211782.2	ENSG00000249547.1	ENSG00000263968.2
21588	ENSG00000211783.3	ENSG00000249545.1	ENSG00000279138.1
21589	ENSG00000211784.2	ENSG00000249542.2	ENSG00000185839.3
21590	ENSG00000211785.1	ENSG00000249541.1	ENSG00000272301.1
21591	ENSG00000211786.3	ENSG00000249540.1	ENSG00000237886.1
21592	ENSG00000211787.1	ENSG00000249539.1	ENSG00000171314.9
21593	ENSG00000211788.2	ENSG00000249534.1	ENSG00000230185.4
21594	ENSG00000211789.2	ENSG00000249532.5	ENSG00000188691.5
21595	ENSG00000211790.2	ENSG00000249531.2	ENSG00000211666.2
21596	ENSG00000211791.2	ENSG00000249526.1	ENSG00000225369.1
21597	ENSG00000211792.2	ENSG00000249525.1	ENSG00000261335.1
21598	ENSG00000211793.2	ENSG00000249522.1	ENSG00000234040.3
21599	ENSG00000211794.3	ENSG00000249521.1	ENSG00000279456.1
21600	ENSG00000211795.3	ENSG00000249520.1	ENSG00000130748.7
21601	ENSG00000211796.1	ENSG00000249519.1	ENSG00000140279.12
21602	ENSG00000211797.2	ENSG00000249518.1	ENSG00000164902.14
21603	ENSG00000211798.3	ENSG00000249516.1	ENSG00000260082.1
21604	ENSG00000211799.3	ENSG00000249515.1	ENSG00000143514.17
21605	ENSG00000211800.3	ENSG00000249514.1	ENSG00000134202.10
21606	ENSG00000211801.3	ENSG00000249513.2	ENSG00000158525.15
21607	ENSG00000211802.3	ENSG00000249510.1	ENSG00000135722.9
21608	ENSG00000211803.2	ENSG00000249509.1	ENSG00000111224.14
21609	ENSG00000211804.3	ENSG00000249506.3	ENSG00000199455.1
21610	ENSG00000211805.1	ENSG00000249505.1	ENSG00000267175.5
21611	ENSG00000211806.2	ENSG00000249504.3	ENSG00000243538.1
21612	ENSG00000211807.3	ENSG00000249503.1	ENSG00000282021.1
21613	ENSG00000211808.3	ENSG00000249502.2	ENSG00000261476.1
21614	ENSG00000211809.2	ENSG00000249501.1	ENSG00000105711.12
21615	ENSG00000211810.3	ENSG00000249500.1	ENSG00000198468.8
21616	ENSG00000211812.1	ENSG00000249497.1	ENSG00000142025.16
21617	ENSG00000211813.2	ENSG00000249495.1	ENSG00000201321.1
21618	ENSG00000211814.1	ENSG00000249494.5	ENSG00000167807.15
21619	ENSG00000211815.3	ENSG00000249493.1	ENSG00000162526.7
21620	ENSG00000211816.2	ENSG00000249492.1	ENSG00000136631.14
21621	ENSG00000211817.2	ENSG00000249491.1	ENSG00000224259.6

21622	ENSG00000211818.1	ENSG00000249490.1	ENSG00000234127.9
21623	ENSG00000211819.3	ENSG00000249489.1	ENSG00000267049.1
21624	ENSG00000211820.1	ENSG00000249488.1	ENSG00000127511.9
21625	ENSG00000211821.2	ENSG00000249487.6	ENSG00000266389.1
21626	ENSG00000211825.1	ENSG00000249486.1	ENSG00000230756.1
21627	ENSG00000211826.1	ENSG00000249485.1	ENSG00000166446.15
21628	ENSG00000211827.1	ENSG00000249484.8	ENSG00000189190.10
21629	ENSG00000211828.1	ENSG00000249483.1	ENSG00000087842.11
21630	ENSG00000211829.9	ENSG00000249482.1	ENSG00000229931.1
21631	ENSG00000211831.2	ENSG00000249481.6	ENSG00000270031.1
21632	ENSG00000211832.2	ENSG00000249479.1	ENSG00000166619.14
21633	ENSG00000211833.1	ENSG00000249478.1	ENSG00000224289.1
21634	ENSG00000211834.1	ENSG00000249476.1	ENSG00000137841.12
21635	ENSG00000211835.1	ENSG00000249474.1	ENSG00000174151.14
21636	ENSG00000211836.1	ENSG00000249472.1	ENSG00000197506.7
21637	ENSG00000211837.1	ENSG00000249471.8	ENSG00000198881.10
21638	ENSG00000211838.1	ENSG00000249467.1	ENSG00000158560.14
21639	ENSG00000211840.1	ENSG00000249465.1	ENSG00000237842.2
21640	ENSG00000211841.1	ENSG00000249464.5	ENSG00000275371.1
21641	ENSG00000211842.1	ENSG00000249463.1	ENSG00000073803.14
21642	ENSG00000211843.1	ENSG00000249462.1	ENSG00000272189.1
21643	ENSG00000211844.1	ENSG00000249460.1	ENSG00000115290.9
21644	ENSG00000211845.1	ENSG00000249459.8	ENSG00000139746.15
21645	ENSG00000211846.1	ENSG00000249458.1	ENSG00000166987.15
21646	ENSG00000211847.1	ENSG00000249456.1	ENSG00000243156.8
21647	ENSG00000211848.1	ENSG00000249454.1	ENSG00000188825.13
21648	ENSG00000211849.1	ENSG00000249453.1	ENSG00000187988.5
21649	ENSG00000211850.1	ENSG00000249452.2	ENSG00000124406.16
21650	ENSG00000211851.1	ENSG00000249451.1	ENSG00000278876.1
21651	ENSG00000211854.1	ENSG00000249449.1	ENSG00000264462.1
21652	ENSG00000211855.1	ENSG00000249448.1	ENSG00000228409.6
21653	ENSG00000211856.1	ENSG00000249446.2	ENSG00000137831.15
21654	ENSG00000211857.1	ENSG00000249444.2	ENSG00000027001.10
21655	ENSG00000211858.1	ENSG00000249443.1	ENSG00000162396.6
21656	ENSG00000211859.1	ENSG00000249441.1	ENSG00000105568.18
21657	ENSG00000211860.1	ENSG00000249439.1	ENSG00000221932.6
21658	ENSG00000211861.1	ENSG00000249438.2	ENSG00000244491.1
21659	ENSG00000211862.1	ENSG00000249436.1	ENSG00000255247.1
21660	ENSG00000211863.1	ENSG00000249435.1	ENSG00000269949.1
21661	ENSG00000211864.2	ENSG00000249433.1	ENSG00000182518.13
21662	ENSG00000211865.1	ENSG00000249429.1	ENSG00000273747.1
21663	ENSG00000211866.1	ENSG00000249426.1	ENSG00000269945.1
21664	ENSG00000211867.1	ENSG00000249425.1	ENSG00000209582.1
21665	ENSG00000211868.1	ENSG00000249421.1	ENSG00000183287.14
21666	ENSG00000211869.1	ENSG00000249419.1	ENSG00000125877.13
21667	ENSG00000211870.1	ENSG00000249418.2	ENSG00000137942.16
21668	ENSG00000211871.1	ENSG00000249417.1	ENSG00000282989.1
21669	ENSG00000211872.1	ENSG00000249416.2	ENSG00000256596.1
21670	ENSG00000211873.1	ENSG00000249413.2	ENSG00000273576.1
21671	ENSG00000211875.1	ENSG00000249411.1	ENSG00000235698.1
21672	ENSG00000211876.1	ENSG00000249410.1	ENSG00000260122.1
21673	ENSG00000211877.1	ENSG00000249409.1	ENSG00000130382.9
21674	ENSG00000211878.1	ENSG00000249407.1	ENSG00000173221.14

21675	ENSG00000211879.1	ENSG00000249406.2	ENSG00000148572.16
21676	ENSG00000211880.1	ENSG00000249405.1	ENSG00000197822.11
21677	ENSG00000211881.1	ENSG00000249404.1	ENSG00000176761.7
21678	ENSG00000211882.1	ENSG00000249403.1	ENSG00000157326.19
21679	ENSG00000211883.1	ENSG00000249400.1	ENSG00000140968.11
21680	ENSG00000211884.1	ENSG00000249396.2	ENSG00000243824.1
21681	ENSG00000211886.1	ENSG00000249395.2	ENSG00000196565.15
21682	ENSG00000211887.2	ENSG00000249392.1	ENSG00000263513.5
21683	ENSG00000211888.2	ENSG00000249388.2	ENSG00000136935.13
21684	ENSG00000211890.4	ENSG00000249387.1	ENSG00000228939.1
21685	ENSG00000211891.6	ENSG00000249386.1	ENSG00000204178.11
21686	ENSG00000211892.4	ENSG00000249383.2	ENSG00000256582.1
21687	ENSG00000211893.4	ENSG00000249382.2	ENSG00000285612.1
21688	ENSG00000211895.5	ENSG00000249381.1	ENSG00000260361.1
21689	ENSG00000211896.7	ENSG00000249380.1	ENSG00000130037.4
21690	ENSG00000211897.9	ENSG00000249379.1	ENSG00000243686.2
21691	ENSG00000211898.7	ENSG00000249378.5	ENSG00000250751.1
21692	ENSG00000211899.10	ENSG00000249375.7	ENSG00000178175.12
21693	ENSG00000211900.2	ENSG00000249373.2	ENSG00000213189.5
21694	ENSG00000211904.2	ENSG00000249372.1	ENSG00000170100.13
21695	ENSG00000211905.1	ENSG00000249368.1	ENSG00000166965.12
21696	ENSG00000211907.1	ENSG00000249367.1	ENSG00000171956.7
21697	ENSG00000211909.1	ENSG00000249364.5	ENSG00000251332.1
21698	ENSG00000211911.1	ENSG00000249363.1	ENSG00000237126.8
21699	ENSG00000211912.1	ENSG00000249362.1	ENSG00000081248.11
21700	ENSG00000211914.1	ENSG00000249360.1	ENSG00000100299.18
21701	ENSG00000211915.1	ENSG00000249359.2	ENSG00000282997.1
21702	ENSG00000211917.1	ENSG00000249353.2	ENSG00000146250.7
21703	ENSG00000211918.1	ENSG00000249352.4	ENSG00000177791.11
21704	ENSG00000211920.1	ENSG00000249351.2	ENSG00000270571.2
21705	ENSG00000211921.1	ENSG00000249349.1	ENSG00000105717.14
21706	ENSG00000211923.1	ENSG00000249348.1	ENSG00000282995.1
21707	ENSG00000211924.1	ENSG00000249347.1	ENSG00000109919.10
21708	ENSG00000211925.1	ENSG00000249346.6	ENSG00000205517.12
21709	ENSG00000211928.1	ENSG00000249345.7	ENSG00000266261.1
21710	ENSG00000211930.1	ENSG00000249343.2	ENSG00000156535.15
21711	ENSG00000211931.1	ENSG00000249341.1	ENSG00000155754.15
21712	ENSG00000211933.2	ENSG00000249338.1	ENSG00000229512.1
21713	ENSG00000211934.3	ENSG00000249337.2	ENSG00000006695.11
21714	ENSG00000211935.3	ENSG00000249335.1	ENSG00000237263.1
21715	ENSG00000211937.3	ENSG00000249334.1	ENSG00000205089.7
21716	ENSG00000211938.2	ENSG00000249333.1	ENSG00000148680.16
21717	ENSG00000211941.3	ENSG00000249332.2	ENSG00000249992.1
21718	ENSG00000211942.3	ENSG00000249330.2	ENSG00000237693.4
21719	ENSG00000211943.2	ENSG00000249328.2	ENSG00000060491.16
21720	ENSG00000211944.2	ENSG00000249326.1	ENSG00000163666.10
21721	ENSG00000211945.2	ENSG00000249321.1	ENSG00000224425.2
21722	ENSG00000211946.3	ENSG00000249320.1	ENSG00000234337.4
21723	ENSG00000211947.2	ENSG00000249319.2	ENSG00000273148.1
21724	ENSG00000211949.3	ENSG00000249318.1	ENSG00000171453.19
21725	ENSG00000211950.2	ENSG00000249317.1	ENSG00000172965.15
21726	ENSG00000211951.2	ENSG00000249316.2	ENSG00000122958.15
21727	ENSG00000211952.3	ENSG00000249312.1	ENSG00000259657.1

21728	ENSG00000211955.2	ENSG00000249311.4	ENSG00000240388.2
21729	ENSG00000211956.2	ENSG00000249310.2	ENSG00000163636.10
21730	ENSG00000211957.2	ENSG00000249309.1	ENSG00000259345.6
21731	ENSG00000211958.2	ENSG00000249307.6	ENSG00000229122.1
21732	ENSG00000211959.2	ENSG00000249306.5	ENSG00000272933.1
21733	ENSG00000211961.3	ENSG00000249305.1	ENSG00000061918.13
21734	ENSG00000211962.2	ENSG00000249304.1	ENSG00000267287.1
21735	ENSG00000211964.3	ENSG00000249302.2	ENSG00000234141.1
21736	ENSG00000211965.4	ENSG00000249301.1	ENSG00000160062.15
21737	ENSG00000211966.2	ENSG00000249297.2	ENSG00000160075.12
21738	ENSG00000211967.3	ENSG00000249295.1	ENSG00000275476.1
21739	ENSG00000211968.3	ENSG00000249293.1	ENSG00000225864.1
21740	ENSG00000211970.3	ENSG00000249290.5	ENSG00000128692.8
21741	ENSG00000211972.2	ENSG00000249289.1	ENSG00000111300.10
21742	ENSG00000211973.2	ENSG00000249288.1	ENSG00000082293.13
21743	ENSG00000211974.3	ENSG00000249287.1	ENSG00000262528.2
21744	ENSG00000211976.2	ENSG00000249286.1	ENSG00000230138.1
21745	ENSG00000211978.2	ENSG00000249285.1	ENSG00000259298.1
21746	ENSG00000211979.2	ENSG00000249284.1	ENSG00000189149.12
21747	ENSG00000211991.4	ENSG00000249283.3	ENSG00000227649.1
21748	ENSG00000211997.1	ENSG00000249282.1	ENSG00000266964.5
21749	ENSG00000212013.1	ENSG00000249279.5	ENSG00000259327.1
21750	ENSG00000212014.1	ENSG00000249278.1	ENSG00000110076.18
21751	ENSG00000212017.2	ENSG00000249277.1	ENSG00000163964.14
21752	ENSG00000212024.2	ENSG00000249276.1	ENSG00000125744.12
21753	ENSG00000212027.3	ENSG00000249275.1	ENSG00000269800.1
21754	ENSG00000212036.3	ENSG00000249274.1	ENSG00000165702.14
21755	ENSG00000212040.3	ENSG00000249272.1	ENSG00000170161.6
21756	ENSG00000212051.1	ENSG00000249271.2	ENSG00000172123.12
21757	ENSG00000212100.2	ENSG00000249270.1	ENSG00000249602.1
21758	ENSG00000212102.1	ENSG00000249269.1	ENSG00000281741.2
21759	ENSG00000212122.3	ENSG00000249267.6	ENSG00000239039.1
21760	ENSG00000212123.4	ENSG00000249266.1	ENSG00000086619.13
21761	ENSG00000212124.2	ENSG00000249264.1	ENSG00000118961.15
21762	ENSG00000212125.2	ENSG00000249263.2	ENSG00000160867.15
21763	ENSG00000212126.3	ENSG00000249262.2	ENSG00000261618.2
21764	ENSG00000212127.5	ENSG00000249261.1	ENSG00000152056.17
21765	ENSG00000212128.2	ENSG00000249259.3	ENSG00000237451.3
21766	ENSG00000212133.1	ENSG00000249258.2	ENSG00000177710.5
21767	ENSG00000212134.1	ENSG00000249257.1	ENSG00000253096.1
21768	ENSG00000212135.1	ENSG00000249256.2	ENSG00000198912.11
21769	ENSG00000212136.1	ENSG00000249255.1	ENSG00000182566.13
21770	ENSG00000212138.1	ENSG00000249253.1	ENSG00000127951.7
21771	ENSG00000212140.1	ENSG00000249252.5	ENSG00000272386.1
21772	ENSG00000212144.1	ENSG00000249249.1	ENSG00000163870.15
21773	ENSG00000212145.2	ENSG00000249247.1	ENSG00000183091.19
21774	ENSG00000212146.1	ENSG00000249245.2	ENSG00000201794.1
21775	ENSG00000212147.1	ENSG00000249244.1	ENSG00000243797.6
21776	ENSG00000212149.1	ENSG00000249242.8	ENSG00000259659.1
21777	ENSG00000212153.1	ENSG00000249241.1	ENSG00000254454.2
21778	ENSG00000212154.1	ENSG00000249239.2	ENSG00000226553.1
21779	ENSG00000212156.1	ENSG00000249238.1	ENSG00000110799.13
21780	ENSG00000212157.1	ENSG00000249237.5	ENSG00000234390.4

21781	ENSG00000212158.1	ENSG00000249236.2	ENSG00000257815.5
21782	ENSG00000212160.1	ENSG00000249235.1	ENSG00000213225.7
21783	ENSG00000212161.1	ENSG00000249234.1	ENSG00000167912.5
21784	ENSG00000212163.5	ENSG00000249231.7	ENSG00000238122.1
21785	ENSG00000212165.2	ENSG00000249230.1	ENSG00000281383.1
21786	ENSG00000212167.1	ENSG00000249229.2	ENSG00000141034.9
21787	ENSG00000212168.1	ENSG00000249228.1	ENSG00000261732.1
21788	ENSG00000212171.1	ENSG00000249226.1	ENSG00000154783.11
21789	ENSG00000212172.1	ENSG00000249225.1	ENSG00000163959.10
21790	ENSG00000212175.1	ENSG00000249222.1	ENSG00000124635.8
21791	ENSG00000212176.1	ENSG00000249219.1	ENSG00000166689.16
21792	ENSG00000212181.1	ENSG00000249216.1	ENSG00000215007.3
21793	ENSG00000212182.2	ENSG00000249215.1	ENSG00000181085.15
21794	ENSG00000212184.1	ENSG00000249213.1	ENSG00000155719.17
21795	ENSG00000212186.1	ENSG00000249212.1	ENSG00000169084.13
21796	ENSG00000212187.1	ENSG00000249210.1	ENSG00000254568.1
21797	ENSG00000212189.1	ENSG00000249209.2	ENSG00000204625.10
21798	ENSG00000212190.1	ENSG00000249207.1	ENSG00000086062.12
21799	ENSG00000212191.1	ENSG00000249206.2	ENSG00000280135.1
21800	ENSG00000212195.2	ENSG00000249203.1	ENSG00000242485.6
21801	ENSG00000212199.1	ENSG00000249201.2	ENSG00000273363.1
21802	ENSG00000212204.1	ENSG00000249200.1	ENSG00000104356.11
21803	ENSG00000212205.1	ENSG00000249199.1	ENSG00000276775.1
21804	ENSG00000212206.1	ENSG00000249198.1	ENSG00000259177.1
21805	ENSG00000212207.1	ENSG00000249197.1	ENSG00000183484.12
21806	ENSG00000212211.2	ENSG00000249196.6	ENSG00000251381.7
21807	ENSG00000212214.1	ENSG00000249193.1	ENSG00000116273.6
21808	ENSG00000212215.1	ENSG00000249192.1	ENSG00000225964.5
21809	ENSG00000212216.1	ENSG00000249191.1	ENSG00000276644.5
21810	ENSG00000212219.1	ENSG00000249189.1	ENSG00000197958.13
21811	ENSG00000212221.1	ENSG00000249188.1	ENSG00000228727.9
21812	ENSG00000212224.1	ENSG00000249186.1	ENSG00000157322.17
21813	ENSG00000212226.1	ENSG00000249184.1	ENSG00000181789.14
21814	ENSG00000212228.1	ENSG00000249183.1	ENSG00000104899.7
21815	ENSG00000212229.1	ENSG00000249180.1	ENSG00000213362.3
21816	ENSG00000212230.1	ENSG00000249177.1	ENSG00000276571.1
21817	ENSG00000212232.1	ENSG00000249176.1	ENSG00000172757.13
21818	ENSG00000212237.1	ENSG00000249175.1	ENSG00000239672.7
21819	ENSG00000212238.1	ENSG00000249174.1	ENSG00000213433.5
21820	ENSG00000212240.1	ENSG00000249173.5	ENSG00000268510.1
21821	ENSG00000212241.1	ENSG00000249171.1	ENSG00000172830.13
21822	ENSG00000212242.1	ENSG00000249170.1	ENSG00000238058.2
21823	ENSG00000212246.1	ENSG00000249169.1	ENSG00000267707.2
21824	ENSG00000212247.1	ENSG00000249167.1	ENSG00000240669.1
21825	ENSG00000212248.1	ENSG00000249166.1	ENSG00000087116.16
21826	ENSG00000212249.2	ENSG00000249163.3	ENSG00000167578.18
21827	ENSG00000212251.1	ENSG00000249162.1	ENSG00000160886.13
21828	ENSG00000212257.1	ENSG00000249160.4	ENSG00000203721.6
21829	ENSG00000212258.1	ENSG00000249159.6	ENSG00000261611.6
21830	ENSG00000212259.1	ENSG00000249158.6	ENSG00000261055.1
21831	ENSG00000212260.1	ENSG00000249157.1	ENSG00000196498.13
21832	ENSG00000212264.1	ENSG00000249156.2	ENSG00000176007.10
21833	ENSG00000212265.1	ENSG00000249153.1	ENSG00000236283.4

21834	ENSG00000212266.1	ENSG00000249152.1	ENSG00000184113.9
21835	ENSG00000212269.1	ENSG00000249150.1	ENSG00000243155.1
21836	ENSG00000212270.1	ENSG00000249149.2	ENSG00000277941.1
21837	ENSG00000212273.1	ENSG00000249148.2	ENSG00000169981.11
21838	ENSG00000212276.1	ENSG00000249145.2	ENSG00000257279.1
21839	ENSG00000212278.1	ENSG00000249142.1	ENSG00000105402.8
21840	ENSG00000212279.2	ENSG00000249141.1	ENSG00000176407.18
21841	ENSG00000212280.1	ENSG00000249140.1	ENSG00000283155.1
21842	ENSG00000212282.1	ENSG00000249139.2	ENSG00000261200.1
21843	ENSG00000212283.1	ENSG00000249138.1	ENSG00000185437.13
21844	ENSG00000212289.1	ENSG00000249135.1	ENSG00000188542.9
21845	ENSG00000212292.1	ENSG00000249131.1	ENSG00000236021.1
21846	ENSG00000212293.1	ENSG00000249129.1	ENSG00000180066.9
21847	ENSG00000212295.1	ENSG00000249128.1	ENSG00000232788.1
21848	ENSG00000212296.1	ENSG00000249127.1	ENSG00000147155.11
21849	ENSG00000212297.1	ENSG00000249125.1	ENSG00000231969.1
21850	ENSG00000212298.1	ENSG00000249122.1	ENSG00000241560.6
21851	ENSG00000212302.1	ENSG00000249119.1	ENSG00000213152.5
21852	ENSG00000212303.1	ENSG00000249116.1	ENSG00000272986.1
21853	ENSG00000212304.1	ENSG00000249115.9	ENSG00000269343.7
21854	ENSG00000212305.1	ENSG00000249114.1	ENSG00000173811.10
21855	ENSG00000212308.1	ENSG00000249112.1	ENSG00000185013.16
21856	ENSG00000212309.1	ENSG00000249111.1	ENSG00000205085.11
21857	ENSG00000212312.1	ENSG00000249109.1	ENSG00000279555.1
21858	ENSG00000212314.2	ENSG00000249106.1	ENSG00000182899.16
21859	ENSG00000212316.1	ENSG00000249105.2	ENSG00000272917.1
21860	ENSG00000212319.1	ENSG00000249104.2	ENSG00000227630.3
21861	ENSG00000212321.2	ENSG00000249102.2	ENSG00000135913.11
21862	ENSG00000212324.1	ENSG00000249101.1	ENSG00000137575.12
21863	ENSG00000212325.1	ENSG00000249100.1	ENSG00000230366.9
21864	ENSG00000212327.1	ENSG00000249099.1	ENSG00000279364.1
21865	ENSG00000212329.1	ENSG00000249098.1	ENSG00000198515.13
21866	ENSG00000212330.1	ENSG00000249096.6	ENSG00000242352.2
21867	ENSG00000212331.1	ENSG00000249094.2	ENSG00000248340.2
21868	ENSG00000212332.1	ENSG00000249092.1	ENSG00000070444.15
21869	ENSG00000212333.1	ENSG00000249091.1	ENSG00000100906.10
21870	ENSG00000212335.1	ENSG00000249089.1	ENSG00000243368.2
21871	ENSG00000212336.1	ENSG00000249087.6	ENSG00000170629.14
21872	ENSG00000212338.1	ENSG00000249086.1	ENSG00000188659.9
21873	ENSG00000212340.1	ENSG00000249085.1	ENSG00000275708.1
21874	ENSG00000212342.1	ENSG00000249084.1	ENSG00000156427.8
21875	ENSG00000212344.1	ENSG00000249082.2	ENSG00000284858.1
21876	ENSG00000212345.1	ENSG00000249081.1	ENSG00000213235.3
21877	ENSG00000212347.2	ENSG00000249079.1	ENSG00000251450.1
21878	ENSG00000212354.1	ENSG00000249077.1	ENSG00000280367.1
21879	ENSG00000212358.1	ENSG00000249074.1	ENSG00000213590.2
21880	ENSG00000212359.1	ENSG00000249073.1	ENSG00000276166.1
21881	ENSG00000212360.1	ENSG00000249072.1	ENSG00000249565.2
21882	ENSG00000212363.1	ENSG00000249071.2	ENSG00000128039.11
21883	ENSG00000212366.1	ENSG00000249069.7	ENSG00000115594.12
21884	ENSG00000212368.1	ENSG00000249068.1	ENSG00000159339.13
21885	ENSG00000212370.1	ENSG00000249066.1	ENSG00000163902.12
21886	ENSG00000212371.1	ENSG00000249065.2	ENSG00000205861.11

21887	ENSG00000212373.1	ENSG00000249064.1	ENSG00000230521.1
21888	ENSG00000212374.1	ENSG00000249061.1	ENSG00000066382.16
21889	ENSG00000212377.1	ENSG00000249057.1	ENSG00000144857.14
21890	ENSG00000212378.1	ENSG00000249056.1	ENSG00000223599.1
21891	ENSG00000212379.1	ENSG00000249055.1	ENSG00000226482.1
21892	ENSG00000212380.1	ENSG00000249054.2	ENSG00000224975.1
21893	ENSG00000212382.1	ENSG00000249053.1	ENSG00000280193.1
21894	ENSG00000212383.1	ENSG00000249052.1	ENSG00000166166.13
21895	ENSG00000212384.1	ENSG00000249051.2	ENSG00000101188.5
21896	ENSG00000212385.1	ENSG00000249050.1	ENSG00000249307.6
21897	ENSG00000212387.1	ENSG00000249049.1	ENSG00000198835.4
21898	ENSG00000212388.1	ENSG00000249048.2	ENSG00000271734.1
21899	ENSG00000212389.1	ENSG00000249047.2	ENSG00000221968.9
21900	ENSG00000212391.1	ENSG00000249045.1	ENSG00000151917.18
21901	ENSG00000212392.1	ENSG00000249041.1	ENSG00000274695.1
21902	ENSG00000212395.1	ENSG00000249038.2	ENSG00000176020.8
21903	ENSG00000212396.1	ENSG00000249036.1	ENSG00000250101.1
21904	ENSG00000212397.1	ENSG00000249035.6	ENSG00000124224.17
21905	ENSG00000212398.1	ENSG00000249031.1	ENSG00000286100.1
21906	ENSG00000212402.1	ENSG00000249028.2	ENSG00000249936.3
21907	ENSG00000212404.1	ENSG00000249026.2	ENSG00000283662.1
21908	ENSG00000212407.1	ENSG00000249025.1	ENSG00000237422.1
21909	ENSG00000212409.1	ENSG00000249023.1	ENSG00000274918.1
21910	ENSG00000212410.1	ENSG00000249022.1	ENSG00000272597.1
21911	ENSG00000212411.1	ENSG00000249021.1	ENSG00000168216.12
21912	ENSG00000212413.1	ENSG00000249020.1	ENSG00000248494.1
21913	ENSG00000212414.1	ENSG00000249019.2	ENSG00000104888.10
21914	ENSG00000212415.1	ENSG00000249018.2	ENSG00000280205.1
21915	ENSG00000212418.1	ENSG00000249017.1	ENSG00000217643.1
21916	ENSG00000212420.1	ENSG00000249016.1	ENSG00000006062.16
21917	ENSG00000212421.1	ENSG00000249014.2	ENSG00000176845.13
21918	ENSG00000212422.2	ENSG00000249013.1	ENSG00000221823.11
21919	ENSG00000212425.1	ENSG00000249012.2	ENSG00000207513.1
21920	ENSG00000212428.1	ENSG00000249008.1	ENSG00000204020.5
21921	ENSG00000212429.1	ENSG00000249007.1	ENSG00000268061.5
21922	ENSG00000212432.1	ENSG00000249006.1	ENSG00000285763.1
21923	ENSG00000212433.1	ENSG00000249005.3	ENSG00000124493.13
21924	ENSG00000212434.2	ENSG00000249004.1	ENSG00000211893.4
21925	ENSG00000212440.1	ENSG00000249003.1	ENSG00000187105.8
21926	ENSG00000212441.1	ENSG00000249002.2	ENSG00000253470.1
21927	ENSG00000212442.1	ENSG00000249001.5	ENSG00000170486.11
21928	ENSG00000212443.1	ENSG00000249000.1	ENSG00000176393.11
21929	ENSG00000212445.1	ENSG00000248998.2	ENSG00000134419.15
21930	ENSG00000212446.1	ENSG00000248996.1	ENSG00000157823.17
21931	ENSG00000212447.1	ENSG00000248995.2	ENSG00000147160.9
21932	ENSG00000212448.1	ENSG00000248994.1	ENSG00000251637.6
21933	ENSG00000212450.1	ENSG00000248993.1	ENSG00000232237.3
21934	ENSG00000212451.1	ENSG00000248991.1	ENSG00000224914.3
21935	ENSG00000212452.1	ENSG00000248990.1	ENSG00000124588.20
21936	ENSG00000212454.1	ENSG00000248988.1	ENSG00000049323.16
21937	ENSG00000212455.1	ENSG00000248987.2	ENSG00000261572.1
21938	ENSG00000212457.1	ENSG00000248984.1	ENSG00000262664.3
21939	ENSG00000212458.1	ENSG00000248980.1	ENSG00000276075.1

21940	ENSG00000212459.1	ENSG00000248979.2	ENSG00000250045.2
21941	ENSG00000212460.1	ENSG00000248978.2	ENSG00000125841.13
21942	ENSG00000212461.1	ENSG00000248977.1	ENSG00000186301.8
21943	ENSG00000212464.1	ENSG00000248975.2	ENSG00000159784.17
21944	ENSG00000212466.1	ENSG00000248973.2	ENSG00000239392.2
21945	ENSG00000212468.1	ENSG00000248971.2	ENSG00000087303.18
21946	ENSG00000212469.1	ENSG00000248969.1	ENSG00000138942.16
21947	ENSG00000212473.1	ENSG00000248968.1	ENSG00000277688.1
21948	ENSG00000212475.1	ENSG00000248967.1	ENSG00000286065.1
21949	ENSG00000212479.2	ENSG00000248966.1	ENSG00000129596.5
21950	ENSG00000212482.1	ENSG00000248965.1	ENSG00000168140.5
21951	ENSG00000212485.1	ENSG00000248964.6	ENSG00000051108.15
21952	ENSG00000212489.1	ENSG00000248962.1	ENSG00000235522.6
21953	ENSG00000212490.1	ENSG00000248958.2	ENSG00000275401.1
21954	ENSG00000212493.1	ENSG00000248956.1	ENSG00000235669.1
21955	ENSG00000212495.1	ENSG00000248955.1	ENSG00000224097.5
21956	ENSG00000212496.1	ENSG00000248954.1	ENSG00000007038.11
21957	ENSG00000212497.1	ENSG00000248951.2	ENSG00000239557.1
21958	ENSG00000212498.1	ENSG00000248950.1	ENSG00000240036.4
21959	ENSG00000212499.1	ENSG00000248949.1	ENSG00000198205.6
21960	ENSG00000212505.1	ENSG00000248946.2	ENSG00000258455.1
21961	ENSG00000212510.1	ENSG00000248944.1	ENSG00000100228.12
21962	ENSG00000212511.2	ENSG00000248943.1	ENSG00000179526.17
21963	ENSG00000212512.2	ENSG00000248942.2	ENSG00000127863.15
21964	ENSG00000212516.1	ENSG00000248939.1	ENSG00000003096.14
21965	ENSG00000212518.1	ENSG00000248936.1	ENSG00000156097.12
21966	ENSG00000212520.1	ENSG00000248935.1	ENSG00000215093.3
21967	ENSG00000212521.1	ENSG00000248933.2	ENSG00000255455.2
21968	ENSG00000212525.1	ENSG00000248932.5	ENSG00000232080.5
21969	ENSG00000212526.1	ENSG00000248931.1	ENSG00000234362.5
21970	ENSG00000212527.1	ENSG00000248930.1	ENSG00000259274.1
21971	ENSG00000212528.1	ENSG00000248929.1	ENSG00000137700.18
21972	ENSG00000212529.1	ENSG00000248928.1	ENSG00000278416.1
21973	ENSG00000212532.1	ENSG00000248927.1	ENSG00000176473.13
21974	ENSG00000212533.1	ENSG00000248926.1	ENSG00000251432.6
21975	ENSG00000212534.1	ENSG00000248925.1	ENSG00000281348.1
21976	ENSG00000212535.1	ENSG00000248924.2	ENSG00000203989.4
21977	ENSG00000212536.1	ENSG00000248923.1	ENSG00000176454.14
21978	ENSG00000212538.2	ENSG00000248921.1	ENSG00000196421.8
21979	ENSG00000212539.2	ENSG00000248920.2	ENSG00000198010.12
21980	ENSG00000212541.1	ENSG00000248919.7	ENSG00000240682.9
21981	ENSG00000212542.1	ENSG00000248918.2	ENSG00000165633.13
21982	ENSG00000212545.1	ENSG00000248916.1	ENSG00000178082.6
21983	ENSG00000212546.1	ENSG00000248915.2	ENSG00000099864.18
21984	ENSG00000212549.1	ENSG00000248913.1	ENSG00000178718.6
21985	ENSG00000212550.1	ENSG00000248912.1	ENSG00000184507.15
21986	ENSG00000212551.2	ENSG00000248911.2	ENSG00000183431.12
21987	ENSG00000212553.1	ENSG00000248909.1	ENSG00000060642.10
21988	ENSG00000212555.1	ENSG00000248908.1	ENSG00000260136.5
21989	ENSG00000212556.1	ENSG00000248907.1	ENSG00000081177.18
21990	ENSG00000212558.1	ENSG00000248905.8	ENSG00000140932.10
21991	ENSG00000212559.1	ENSG00000248903.1	ENSG00000211951.2
21992	ENSG00000212560.1	ENSG00000248901.1	ENSG00000241269.1

21993	ENSG00000212561.1	ENSG00000248898.1	ENSG00000232615.4
21994	ENSG00000212564.1	ENSG00000248896.2	ENSG00000129235.10
21995	ENSG00000212565.1	ENSG00000248895.1	ENSG00000265907.1
21996	ENSG00000212567.1	ENSG00000248893.3	ENSG00000223842.1
21997	ENSG00000212568.1	ENSG00000248891.2	ENSG00000224831.3
21998	ENSG00000212569.1	ENSG00000248890.1	ENSG00000280969.1
21999	ENSG00000212571.1	ENSG00000248886.2	ENSG00000185065.6
22000	ENSG00000212572.1	ENSG00000248885.1	ENSG00000209082.1
22001	ENSG00000212576.1	ENSG00000248884.1	ENSG00000272690.5
22002	ENSG00000212579.1	ENSG00000248883.2	ENSG00000162892.15
22003	ENSG00000212580.1	ENSG00000248881.1	ENSG00000183018.9
22004	ENSG00000212581.1	ENSG00000248880.1	ENSG00000163328.13
22005	ENSG00000212584.1	ENSG00000248878.1	ENSG00000272977.1
22006	ENSG00000212586.1	ENSG00000248877.2	ENSG00000234965.2
22007	ENSG00000212587.1	ENSG00000248876.1	ENSG00000160957.13
22008	ENSG00000212588.1	ENSG00000248874.5	ENSG00000126231.14
22009	ENSG00000212589.1	ENSG00000248872.1	ENSG00000024526.17
22010	ENSG00000212590.1	ENSG00000248871.1	ENSG00000285205.2
22011	ENSG00000212593.1	ENSG00000248870.1	ENSG00000266173.7
22012	ENSG00000212594.1	ENSG00000248869.6	ENSG00000183066.14
22013	ENSG00000212595.1	ENSG00000248867.1	ENSG00000271851.1
22014	ENSG00000212597.1	ENSG00000248866.1	ENSG00000179855.7
22015	ENSG00000212598.2	ENSG00000248863.2	ENSG00000188981.11
22016	ENSG00000212599.1	ENSG00000248859.2	ENSG00000247324.2
22017	ENSG00000212601.1	ENSG00000248858.7	ENSG00000164323.14
22018	ENSG00000212604.1	ENSG00000248854.1	ENSG00000120725.13
22019	ENSG00000212605.1	ENSG00000248853.1	ENSG00000187742.14
22020	ENSG00000212607.1	ENSG00000248851.1	ENSG00000278765.1
22021	ENSG00000212608.1	ENSG00000248850.1	ENSG00000102245.7
22022	ENSG00000212609.1	ENSG00000248848.1	ENSG00000237576.1
22023	ENSG00000212610.2	ENSG00000248847.2	ENSG00000232433.2
22024	ENSG00000212611.1	ENSG00000248846.2	ENSG00000075624.15
22025	ENSG00000212612.1	ENSG00000248844.6	ENSG00000084073.9
22026	ENSG00000212615.1	ENSG00000248843.1	ENSG00000211685.3
22027	ENSG00000212618.1	ENSG00000248842.1	ENSG00000270344.2
22028	ENSG00000212620.1	ENSG00000248840.2	ENSG00000171772.16
22029	ENSG00000212623.1	ENSG00000248839.1	ENSG00000234006.1
22030	ENSG00000212624.1	ENSG00000248838.2	ENSG00000152932.8
22031	ENSG00000212625.1	ENSG00000248837.6	ENSG00000213088.11
22032	ENSG00000212626.1	ENSG00000248834.2	ENSG00000103269.13
22033	ENSG00000212628.1	ENSG00000248831.2	ENSG00000179967.11
22034	ENSG00000212643.3	ENSG00000248830.1	ENSG00000157557.12
22035	ENSG00000212657.1	ENSG00000248827.1	ENSG00000120697.9
22036	ENSG00000212658.1	ENSG00000248826.2	ENSG00000236017.8
22037	ENSG00000212659.1	ENSG00000248824.2	ENSG00000227933.1
22038	ENSG00000212663.2	ENSG00000248822.1	ENSG00000286228.1
22039	ENSG00000212664.5	ENSG00000248821.1	ENSG00000254272.1
22040	ENSG00000212694.8	ENSG00000248820.2	ENSG00000198198.16
22041	ENSG00000212695.5	ENSG00000248817.1	ENSG00000134480.15
22042	ENSG00000212710.4	ENSG00000248816.1	ENSG00000273451.1
22043	ENSG00000212712.2	ENSG00000248813.1	ENSG00000152782.16
22044	ENSG00000212717.3	ENSG00000248810.1	ENSG00000158955.11
22045	ENSG00000212719.11	ENSG00000248809.6	ENSG00000153015.16

22046	ENSG00000212721.3	ENSG00000248807.1	ENSG00000250361.8
22047	ENSG00000212722.7	ENSG00000248803.1	ENSG00000267649.1
22048	ENSG00000212724.3	ENSG00000248802.1	ENSG00000228697.3
22049	ENSG00000212725.3	ENSG00000248801.6	ENSG00000175782.10
22050	ENSG00000212743.2	ENSG00000248799.1	ENSG00000226885.1
22051	ENSG00000212747.4	ENSG00000248796.1	ENSG00000285542.1
22052	ENSG00000212766.9	ENSG00000248795.1	ENSG00000119707.14
22053	ENSG00000212769.5	ENSG00000248794.1	ENSG00000259268.1
22054	ENSG00000212789.4	ENSG00000248792.1	ENSG00000140443.14
22055	ENSG00000212802.4	ENSG00000248791.1	ENSG00000223553.6
22056	ENSG00000212807.2	ENSG00000248790.1	ENSG00000266236.1
22057	ENSG00000212829.8	ENSG00000248789.5	ENSG00000233369.7
22058	ENSG00000212855.5	ENSG00000248787.2	ENSG00000255872.3
22059	ENSG00000212856.6	ENSG00000248785.1	ENSG00000270177.1
22060	ENSG00000212864.3	ENSG00000248783.1	ENSG00000100884.9
22061	ENSG00000212899.2	ENSG00000248781.1	ENSG00000041357.16
22062	ENSG00000212900.2	ENSG00000248780.1	ENSG00000153976.3
22063	ENSG00000212901.4	ENSG00000248779.1	ENSG00000158467.16
22064	ENSG00000212907.2	ENSG00000248778.1	ENSG00000163930.10
22065	ENSG00000212916.4	ENSG00000248777.1	ENSG00000129535.12
22066	ENSG00000212930.3	ENSG00000248775.1	ENSG00000228590.2
22067	ENSG00000212932.3	ENSG00000248774.1	ENSG00000025039.14
22068	ENSG00000212933.1	ENSG00000248773.1	ENSG00000260244.1
22069	ENSG00000212935.1	ENSG00000248772.1	ENSG00000268225.2
22070	ENSG00000212938.3	ENSG00000248771.5	ENSG00000272529.1
22071	ENSG00000212939.2	ENSG00000248770.1	ENSG00000189164.15
22072	ENSG00000212951.5	ENSG00000248769.1	ENSG00000273082.1
22073	ENSG00000212952.6	ENSG00000248767.2	ENSG00000205090.9
22074	ENSG00000212961.4	ENSG00000248766.2	ENSG00000240731.1
22075	ENSG00000212978.6	ENSG00000248765.1	ENSG00000185010.15
22076	ENSG00000212989.3	ENSG00000248764.1	ENSG00000241511.1
22077	ENSG00000212993.5	ENSG00000248763.2	ENSG00000240382.3
22078	ENSG00000212994.5	ENSG00000248762.2	ENSG00000123562.17
22079	ENSG00000213003.3	ENSG00000248761.1	ENSG00000166192.15
22080	ENSG00000213005.3	ENSG00000248758.1	ENSG00000240522.1
22081	ENSG00000213013.4	ENSG00000248757.2	ENSG00000153310.19
22082	ENSG00000213014.3	ENSG00000248755.1	ENSG00000090530.10
22083	ENSG00000213015.9	ENSG00000248753.1	ENSG00000171631.14
22084	ENSG00000213016.3	ENSG00000248752.2	ENSG00000223935.2
22085	ENSG00000213018.2	ENSG00000248751.6	ENSG00000183935.5
22086	ENSG00000213020.10	ENSG00000248750.2	ENSG00000127364.3
22087	ENSG00000213022.6	ENSG00000248749.1	ENSG00000211879.1
22088	ENSG00000213023.11	ENSG00000248748.1	ENSG00000267858.5
22089	ENSG00000213024.11	ENSG00000248747.1	ENSG00000272896.1
22090	ENSG00000213025.2	ENSG00000248746.6	ENSG00000141977.9
22091	ENSG00000213026.4	ENSG00000248745.2	ENSG00000151092.17
22092	ENSG00000213028.3	ENSG00000248744.1	ENSG00000267693.1
22093	ENSG00000213029.3	ENSG00000248740.5	ENSG00000129170.9
22094	ENSG00000213030.5	ENSG00000248739.1	ENSG00000211785.1
22095	ENSG00000213032.3	ENSG00000248738.6	ENSG00000254521.6
22096	ENSG00000213033.4	ENSG00000248736.2	ENSG00000234409.6
22097	ENSG00000213035.4	ENSG00000248735.2	ENSG00000240494.2
22098	ENSG00000213036.3	ENSG00000248734.2	ENSG00000008438.5

22099	ENSG00000213041.4	ENSG00000248733.1	ENSG00000225191.1
22100	ENSG00000213045.3	ENSG00000248730.1	ENSG00000247077.7
22101	ENSG00000213046.4	ENSG00000248729.1	ENSG00000124019.10
22102	ENSG00000213047.13	ENSG00000248727.5	ENSG00000150556.17
22103	ENSG00000213048.3	ENSG00000248725.3	ENSG00000103227.18
22104	ENSG00000213049.3	ENSG00000248724.6	ENSG00000229899.1
22105	ENSG00000213050.5	ENSG00000248722.1	ENSG00000206734.1
22106	ENSG00000213051.3	ENSG00000248720.1	ENSG00000279077.1
22107	ENSG00000213055.3	ENSG00000248719.1	ENSG00000108061.11
22108	ENSG00000213057.5	ENSG00000248717.1	ENSG00000183873.15
22109	ENSG00000213058.3	ENSG00000248716.2	ENSG00000253954.3
22110	ENSG00000213060.4	ENSG00000248715.2	ENSG00000109943.9
22111	ENSG00000213061.2	ENSG00000248714.6	ENSG00000274020.3
22112	ENSG00000213062.4	ENSG00000248713.1	ENSG00000188076.2
22113	ENSG00000213063.3	ENSG00000248712.7	ENSG00000213463.5
22114	ENSG00000213064.10	ENSG00000248711.1	ENSG00000163704.12
22115	ENSG00000213065.2	ENSG00000248710.1	ENSG00000064726.9
22116	ENSG00000213066.13	ENSG00000248709.2	ENSG00000132330.17
22117	ENSG00000213067.2	ENSG00000248708.1	ENSG00000179331.3
22118	ENSG00000213068.3	ENSG00000248705.1	ENSG00000196968.11
22119	ENSG00000213069.3	ENSG00000248704.1	ENSG00000282994.1
22120	ENSG00000213070.3	ENSG00000248703.2	ENSG00000226824.6
22121	ENSG00000213071.11	ENSG00000248702.1	ENSG00000068097.14
22122	ENSG00000213073.4	ENSG00000248701.1	ENSG00000284491.2
22123	ENSG00000213075.7	ENSG00000248699.1	ENSG00000274776.1
22124	ENSG00000213076.3	ENSG00000248698.6	ENSG00000013016.16
22125	ENSG00000213078.3	ENSG00000248697.1	ENSG00000263787.1
22126	ENSG00000213079.9	ENSG00000248696.1	ENSG00000121964.14
22127	ENSG00000213080.3	ENSG00000248694.1	ENSG00000101972.18
22128	ENSG00000213081.3	ENSG00000248693.1	ENSG00000242615.1
22129	ENSG00000213082.3	ENSG00000248692.5	ENSG00000155085.15
22130	ENSG00000213083.3	ENSG00000248690.7	ENSG00000113657.13
22131	ENSG00000213085.10	ENSG00000248687.2	ENSG00000125384.7
22132	ENSG00000213087.3	ENSG00000248685.5	ENSG00000279846.1
22133	ENSG00000213088.11	ENSG00000248684.1	ENSG00000157600.12
22134	ENSG00000213089.4	ENSG00000248682.1	ENSG00000233968.6
22135	ENSG00000213090.2	ENSG00000248677.1	ENSG00000165092.13
22136	ENSG00000213091.2	ENSG00000248676.1	ENSG00000261794.1
22137	ENSG00000213096.10	ENSG00000248674.1	ENSG00000267510.1
22138	ENSG00000213100.2	ENSG00000248673.1	ENSG00000162994.16
22139	ENSG00000213104.3	ENSG00000248672.5	ENSG00000261959.1
22140	ENSG00000213107.2	ENSG00000248671.7	ENSG00000160588.10
22141	ENSG00000213108.3	ENSG00000248669.1	ENSG00000161202.19
22142	ENSG00000213109.4	ENSG00000248668.2	ENSG00000125845.7
22143	ENSG00000213110.2	ENSG00000248667.1	ENSG00000169246.16
22144	ENSG00000213111.5	ENSG00000248664.1	ENSG00000104626.14
22145	ENSG00000213113.3	ENSG00000248663.6	ENSG00000047617.15
22146	ENSG00000213115.2	ENSG00000248660.1	ENSG00000105671.12
22147	ENSG00000213117.4	ENSG00000248659.1	ENSG00000212939.2
22148	ENSG00000213118.4	ENSG00000248656.1	ENSG00000068308.13
22149	ENSG00000213120.3	ENSG00000248654.1	ENSG00000165874.13
22150	ENSG00000213121.2	ENSG00000248652.2	ENSG00000240087.3
22151	ENSG00000213122.4	ENSG00000248651.1	ENSG00000235947.1

22152	ENSG00000213123.11	ENSG00000248648.1	ENSG00000124107.5
22153	ENSG00000213126.2	ENSG00000248647.2	ENSG00000261596.2
22154	ENSG00000213128.3	ENSG00000248646.1	ENSG00000281358.1
22155	ENSG00000213130.3	ENSG00000248645.1	ENSG00000135740.17
22156	ENSG00000213131.3	ENSG00000248643.5	ENSG00000101255.11
22157	ENSG00000213133.4	ENSG00000248642.1	ENSG00000205209.7
22158	ENSG00000213137.3	ENSG00000248641.1	ENSG00000110931.19
22159	ENSG00000213139.8	ENSG00000248640.1	ENSG00000224138.1
22160	ENSG00000213140.3	ENSG00000248639.2	ENSG00000203711.13
22161	ENSG00000213144.2	ENSG00000248637.1	ENSG00000259671.1
22162	ENSG00000213145.9	ENSG00000248636.6	ENSG00000119699.7
22163	ENSG00000213147.3	ENSG00000248635.1	ENSG00000146872.17
22164	ENSG00000213148.3	ENSG00000248634.1	ENSG00000226582.1
22165	ENSG00000213149.3	ENSG00000248633.1	ENSG00000186652.10
22166	ENSG00000213150.2	ENSG00000248629.1	ENSG00000228430.9
22167	ENSG00000213152.5	ENSG00000248627.1	ENSG00000278384.1
22168	ENSG00000213153.3	ENSG00000248626.1	ENSG00000234009.1
22169	ENSG00000213155.4	ENSG00000248625.1	ENSG00000111664.10
22170	ENSG00000213157.3	ENSG00000248624.1	ENSG00000274943.1
22171	ENSG00000213158.6	ENSG00000248621.2	ENSG00000258702.2
22172	ENSG00000213159.4	ENSG00000248618.1	ENSG00000120705.13
22173	ENSG00000213160.10	ENSG00000248616.1	ENSG00000243759.1
22174	ENSG00000213167.3	ENSG00000248613.1	ENSG00000158470.5
22175	ENSG00000213169.3	ENSG00000248611.1	ENSG00000174718.12
22176	ENSG00000213170.3	ENSG00000248610.1	ENSG00000273748.1
22177	ENSG00000213171.2	ENSG00000248608.2	ENSG00000255389.1
22178	ENSG00000213172.3	ENSG00000248607.1	ENSG00000136305.11
22179	ENSG00000213174.3	ENSG00000248605.5	ENSG00000280132.1
22180	ENSG00000213176.4	ENSG00000248601.1	ENSG00000272638.1
22181	ENSG00000213177.3	ENSG00000248600.1	ENSG00000104826.13
22182	ENSG00000213178.3	ENSG00000248599.1	ENSG00000112592.14
22183	ENSG00000213179.4	ENSG00000248597.1	ENSG00000138867.16
22184	ENSG00000213180.4	ENSG00000248596.7	ENSG00000213252.3
22185	ENSG00000213181.3	ENSG00000248593.3	ENSG00000171044.10
22186	ENSG00000213182.4	ENSG00000248592.7	ENSG00000214465.3
22187	ENSG00000213183.3	ENSG00000248591.1	ENSG00000264739.1
22188	ENSG00000213184.3	ENSG00000248588.2	ENSG00000170128.4
22189	ENSG00000213185.7	ENSG00000248587.7	ENSG00000148925.10
22190	ENSG00000213186.8	ENSG00000248586.2	ENSG00000146085.8
22191	ENSG00000213187.3	ENSG00000248585.2	ENSG00000267493.3
22192	ENSG00000213188.3	ENSG00000248583.1	ENSG00000182965.6
22193	ENSG00000213189.5	ENSG00000248578.1	ENSG00000286104.1
22194	ENSG00000213190.3	ENSG00000248577.1	ENSG00000113231.13
22195	ENSG00000213194.3	ENSG00000248576.1	ENSG00000092529.24
22196	ENSG00000213197.3	ENSG00000248574.1	ENSG00000198157.10
22197	ENSG00000213199.8	ENSG00000248573.1	ENSG00000251675.1
22198	ENSG00000213201.3	ENSG00000248572.5	ENSG00000261346.1
22199	ENSG00000213203.3	ENSG00000248571.1	ENSG00000273343.1
22200	ENSG00000213204.8	ENSG00000248569.1	ENSG00000143702.16
22201	ENSG00000213205.3	ENSG00000248568.1	ENSG00000198682.13
22202	ENSG00000213209.2	ENSG00000248567.1	ENSG00000256671.6
22203	ENSG00000213210.4	ENSG00000248565.2	ENSG00000267321.2
22204	ENSG00000213211.2	ENSG00000248564.1	ENSG00000225411.3

22205	ENSG00000213212.3	ENSG00000248560.1	ENSG00000254662.1
22206	ENSG00000213213.13	ENSG00000248559.1	ENSG00000260366.1
22207	ENSG00000213214.4	ENSG00000248557.1	ENSG00000269952.1
22208	ENSG00000213215.5	ENSG00000248555.6	ENSG00000233154.5
22209	ENSG00000213216.2	ENSG00000248554.1	ENSG00000231039.2
22210	ENSG00000213218.10	ENSG00000248553.1	ENSG00000250602.6
22211	ENSG00000213221.5	ENSG00000248552.1	ENSG00000196597.12
22212	ENSG00000213222.3	ENSG00000248551.1	ENSG00000220749.4
22213	ENSG00000213225.7	ENSG00000248550.3	ENSG00000273289.1
22214	ENSG00000213226.4	ENSG00000248548.1	ENSG00000261799.1
22215	ENSG00000213228.5	ENSG00000248547.2	ENSG00000232134.1
22216	ENSG00000213231.13	ENSG00000248546.3	ENSG00000159335.16
22217	ENSG00000213232.2	ENSG00000248545.2	ENSG00000130940.15
22218	ENSG00000213233.5	ENSG00000248544.2	ENSG00000134375.11
22219	ENSG00000213234.4	ENSG00000248543.2	ENSG00000143552.9
22220	ENSG00000213235.3	ENSG00000248542.2	ENSG00000272717.1
22221	ENSG00000213236.3	ENSG00000248540.2	ENSG00000229431.1
22222	ENSG00000213237.4	ENSG00000248539.1	ENSG00000215045.8
22223	ENSG00000213238.6	ENSG00000248538.7	ENSG00000147231.14
22224	ENSG00000213239.3	ENSG00000248537.1	ENSG00000082153.18
22225	ENSG00000213244.3	ENSG00000248533.1	ENSG00000273308.1
22226	ENSG00000213246.7	ENSG00000248532.2	ENSG00000227237.1
22227	ENSG00000213247.3	ENSG00000248531.3	ENSG00000165572.7
22228	ENSG00000213250.5	ENSG00000248530.1	ENSG00000131781.13
22229	ENSG00000213252.3	ENSG00000248529.5	ENSG00000031081.10
22230	ENSG00000213253.5	ENSG00000248528.1	ENSG00000244184.1
22231	ENSG00000213260.3	ENSG00000248527.1	ENSG00000215304.3
22232	ENSG00000213261.3	ENSG00000248525.2	ENSG00000204681.11
22233	ENSG00000213262.3	ENSG00000248522.1	ENSG00000104879.5
22234	ENSG00000213264.3	ENSG00000248521.1	ENSG00000171604.12
22235	ENSG00000213265.8	ENSG00000248518.1	ENSG00000141744.3
22236	ENSG00000213269.2	ENSG00000248517.1	ENSG00000285330.1
22237	ENSG00000213270.5	ENSG00000248516.1	ENSG00000226445.1
22238	ENSG00000213272.5	ENSG00000248515.1	ENSG00000273797.1
22239	ENSG00000213275.2	ENSG00000248514.1	ENSG00000272693.4
22240	ENSG00000213277.3	ENSG00000248511.1	ENSG00000145331.14
22241	ENSG00000213279.2	ENSG00000248510.2	ENSG00000166833.21
22242	ENSG00000213280.2	ENSG00000248508.7	ENSG00000213073.4
22243	ENSG00000213281.5	ENSG00000248507.1	ENSG00000234562.1
22244	ENSG00000213285.4	ENSG00000248506.1	ENSG00000111962.8
22245	ENSG00000213287.3	ENSG00000248505.1	ENSG00000115840.14
22246	ENSG00000213290.4	ENSG00000248503.2	ENSG00000142611.17
22247	ENSG00000213291.3	ENSG00000248498.4	ENSG00000248367.1
22248	ENSG00000213293.4	ENSG00000248494.1	ENSG00000264630.5
22249	ENSG00000213295.3	ENSG00000248493.1	ENSG00000232000.3
22250	ENSG00000213296.4	ENSG00000248491.5	ENSG00000139722.7
22251	ENSG00000213297.8	ENSG00000248490.1	ENSG00000170027.7
22252	ENSG00000213300.5	ENSG00000248489.1	ENSG00000168813.17
22253	ENSG00000213301.2	ENSG00000248488.2	ENSG00000188681.11
22254	ENSG00000213302.3	ENSG00000248487.9	ENSG00000198804.2
22255	ENSG00000213303.3	ENSG00000248485.2	ENSG00000101181.17
22256	ENSG00000213304.3	ENSG00000248484.1	ENSG00000072694.20
22257	ENSG00000213305.3	ENSG00000248483.6	ENSG00000083828.16

22258	ENSG00000213307.4	ENSG00000248482.1	ENSG00000078401.7
22259	ENSG00000213309.3	ENSG00000248480.1	ENSG00000260409.1
22260	ENSG00000213310.3	ENSG00000248479.1	ENSG00000232150.3
22261	ENSG00000213312.3	ENSG00000248477.6	ENSG00000164885.13
22262	ENSG00000213315.5	ENSG00000248476.1	ENSG00000276840.1
22263	ENSG00000213316.9	ENSG00000248475.5	ENSG00000226185.2
22264	ENSG00000213318.4	ENSG00000248474.1	ENSG00000267370.1
22265	ENSG00000213326.4	ENSG00000248473.1	ENSG00000103034.14
22266	ENSG00000213328.3	ENSG00000248472.8	ENSG00000105325.14
22267	ENSG00000213331.4	ENSG00000248471.2	ENSG00000140030.6
22268	ENSG00000213332.4	ENSG00000248469.1	ENSG00000086570.12
22269	ENSG00000213333.3	ENSG00000248468.1	ENSG00000197461.13
22270	ENSG00000213335.4	ENSG00000248466.1	ENSG00000244649.4
22271	ENSG00000213337.9	ENSG00000248464.1	ENSG00000185668.7
22272	ENSG00000213338.3	ENSG00000248462.1	ENSG00000138668.19
22273	ENSG00000213339.9	ENSG00000248461.2	ENSG00000223648.4
22274	ENSG00000213341.11	ENSG00000248459.1	ENSG00000262039.1
22275	ENSG00000213343.5	ENSG00000248458.2	ENSG00000119698.12
22276	ENSG00000213344.2	ENSG00000248457.1	ENSG00000203995.9
22277	ENSG00000213347.10	ENSG00000248456.1	ENSG00000106028.11
22278	ENSG00000213352.3	ENSG00000248455.5	ENSG00000115825.10
22279	ENSG00000213355.3	ENSG00000248452.2	ENSG00000236976.1
22280	ENSG00000213358.3	ENSG00000248449.2	ENSG00000068724.16
22281	ENSG00000213361.2	ENSG00000248448.2	ENSG00000279254.1
22282	ENSG00000213362.3	ENSG00000248447.2	ENSG00000141576.16
22283	ENSG00000213363.4	ENSG00000248445.5	ENSG00000225963.7
22284	ENSG00000213365.3	ENSG00000248444.1	ENSG00000224431.1
22285	ENSG00000213366.13	ENSG00000248443.1	ENSG00000267680.5
22286	ENSG00000213368.3	ENSG00000248442.2	ENSG00000277978.1
22287	ENSG00000213370.3	ENSG00000248441.6	ENSG00000227115.7
22288	ENSG00000213371.4	ENSG00000248440.1	ENSG00000163491.16
22289	ENSG00000213373.7	ENSG00000248439.2	ENSG00000240040.6
22290	ENSG00000213376.4	ENSG00000248434.1	ENSG00000239474.7
22291	ENSG00000213380.15	ENSG00000248432.1	ENSG00000161847.14
22292	ENSG00000213383.2	ENSG00000248431.1	ENSG00000211667.3
22293	ENSG00000213384.2	ENSG00000248430.1	ENSG00000256973.1
22294	ENSG00000213385.3	ENSG00000248429.5	ENSG00000260625.3
22295	ENSG00000213386.3	ENSG00000248428.1	ENSG00000231856.2
22296	ENSG00000213390.11	ENSG00000248426.1	ENSG00000223839.7
22297	ENSG00000213393.5	ENSG00000248425.1	ENSG00000272205.1
22298	ENSG00000213394.3	ENSG00000248424.1	ENSG00000254428.1
22299	ENSG00000213397.10	ENSG00000248423.1	ENSG00000214274.9
22300	ENSG00000213398.7	ENSG00000248422.1	ENSG00000251093.1
22301	ENSG00000213399.3	ENSG00000248420.1	ENSG00000234665.8
22302	ENSG00000213400.3	ENSG00000248419.2	ENSG00000076944.16
22303	ENSG00000213401.10	ENSG00000248418.2	ENSG00000266079.4
22304	ENSG00000213402.3	ENSG00000248417.1	ENSG00000204959.4
22305	ENSG00000213403.2	ENSG00000248416.1	ENSG00000272839.1
22306	ENSG00000213406.3	ENSG00000248415.1	ENSG00000279923.1
22307	ENSG00000213409.4	ENSG00000248409.2	ENSG00000233665.8
22308	ENSG00000213411.2	ENSG00000248408.1	ENSG00000188112.9
22309	ENSG00000213412.3	ENSG00000248406.1	ENSG00000253686.1
22310	ENSG00000213413.2	ENSG00000248405.10	ENSG00000267096.1

22311	ENSG00000213414.3	ENSG00000248403.1	ENSG00000228264.1
22312	ENSG00000213416.4	ENSG00000248401.2	ENSG00000146054.18
22313	ENSG00000213417.3	ENSG00000248400.2	ENSG00000280069.1
22314	ENSG00000213420.8	ENSG00000248399.1	ENSG00000262953.1
22315	ENSG00000213421.4	ENSG00000248397.1	ENSG00000226624.1
22316	ENSG00000213423.4	ENSG00000248396.1	ENSG00000273658.1
22317	ENSG00000213424.9	ENSG00000248394.1	ENSG00000237515.9
22318	ENSG00000213430.6	ENSG00000248393.1	ENSG00000168826.16
22319	ENSG00000213431.4	ENSG00000248391.5	ENSG00000213970.4
22320	ENSG00000213432.2	ENSG00000248388.5	ENSG00000106346.11
22321	ENSG00000213433.5	ENSG00000248387.1	ENSG00000212952.6
22322	ENSG00000213434.2	ENSG00000248385.7	ENSG00000114395.10
22323	ENSG00000213435.3	ENSG00000248383.4	ENSG00000228549.3
22324	ENSG00000213438.2	ENSG00000248378.1	ENSG00000275485.1
22325	ENSG00000213439.3	ENSG00000248377.1	ENSG00000111077.17
22326	ENSG00000213440.2	ENSG00000248376.2	ENSG00000231643.4
22327	ENSG00000213442.5	ENSG00000248375.1	ENSG00000124570.19
22328	ENSG00000213443.2	ENSG00000248374.1	ENSG00000146192.15
22329	ENSG00000213445.10	ENSG00000248373.5	ENSG00000160097.18
22330	ENSG00000213448.3	ENSG00000248371.5	ENSG00000272913.1
22331	ENSG00000213449.2	ENSG00000248370.1	ENSG00000038427.16
22332	ENSG00000213450.4	ENSG00000248369.1	ENSG00000140403.12
22333	ENSG00000213451.2	ENSG00000248367.1	ENSG00000175800.5
22334	ENSG00000213452.4	ENSG00000248366.1	ENSG00000256250.1
22335	ENSG00000213453.3	ENSG00000248365.3	ENSG00000060982.15
22336	ENSG00000213455.3	ENSG00000248364.1	ENSG00000234899.9
22337	ENSG00000213461.3	ENSG00000248363.5	ENSG00000182183.15
22338	ENSG00000213462.5	ENSG00000248362.1	ENSG00000211970.3
22339	ENSG00000213463.5	ENSG00000248360.7	ENSG00000149516.14
22340	ENSG00000213465.8	ENSG00000248359.1	ENSG00000214652.6
22341	ENSG00000213467.4	ENSG00000248358.2	ENSG00000174446.13
22342	ENSG00000213468.6	ENSG00000248356.1	ENSG00000091844.8
22343	ENSG00000213470.4	ENSG00000248355.1	ENSG00000189367.15
22344	ENSG00000213471.10	ENSG00000248351.1	ENSG00000269814.1
22345	ENSG00000213478.2	ENSG00000248350.1	ENSG00000211869.1
22346	ENSG00000213480.3	ENSG00000248349.1	ENSG00000112414.14
22347	ENSG00000213483.4	ENSG00000248347.3	ENSG00000019186.10
22348	ENSG00000213484.2	ENSG00000248346.1	ENSG00000110330.8
22349	ENSG00000213486.3	ENSG00000248343.1	ENSG00000174407.13
22350	ENSG00000213487.3	ENSG00000248340.2	ENSG00000075891.21
22351	ENSG00000213488.4	ENSG00000248339.1	ENSG00000285901.1
22352	ENSG00000213489.3	ENSG00000248338.1	ENSG00000224424.7
22353	ENSG00000213492.2	ENSG00000248337.1	ENSG00000166896.8
22354	ENSG00000213493.3	ENSG00000248336.1	ENSG00000266677.1
22355	ENSG00000213495.3	ENSG00000248335.1	ENSG00000122122.10
22356	ENSG00000213498.3	ENSG00000248334.6	ENSG00000204390.10
22357	ENSG00000213500.3	ENSG00000248333.8	ENSG00000105011.9
22358	ENSG00000213509.4	ENSG00000248332.6	ENSG00000261288.1
22359	ENSG00000213512.3	ENSG00000248330.5	ENSG00000277368.1
22360	ENSG00000213513.3	ENSG00000248329.6	ENSG00000224927.2
22361	ENSG00000213514.2	ENSG00000248328.1	ENSG00000251667.1
22362	ENSG00000213516.10	ENSG00000248327.1	ENSG00000141447.18
22363	ENSG00000213519.2	ENSG00000248323.6	ENSG00000234219.1

22364	ENSG00000213522.4	ENSG00000248322.1	ENSG00000141252.20
22365	ENSG00000213523.10	ENSG00000248320.1	ENSG00000087589.16
22366	ENSG00000213525.3	ENSG00000248319.1	ENSG00000101098.12
22367	ENSG00000213526.3	ENSG00000248318.1	ENSG00000100271.17
22368	ENSG00000213527.5	ENSG00000248317.1	ENSG00000169570.10
22369	ENSG00000213529.3	ENSG00000248315.1	ENSG00000280326.1
22370	ENSG00000213530.3	ENSG00000248313.1	ENSG00000219102.3
22371	ENSG00000213533.12	ENSG00000248311.1	ENSG00000269834.5
22372	ENSG00000213536.2	ENSG00000248309.7	ENSG00000122679.8
22373	ENSG00000213538.5	ENSG00000248308.1	ENSG00000226742.4
22374	ENSG00000213539.4	ENSG00000248307.5	ENSG00000110665.11
22375	ENSG00000213540.3	ENSG00000248305.1	ENSG00000112406.5
22376	ENSG00000213542.3	ENSG00000248302.3	ENSG00000143199.17
22377	ENSG00000213543.2	ENSG00000248300.1	ENSG00000103742.12
22378	ENSG00000213547.3	ENSG00000248296.1	ENSG00000260238.6
22379	ENSG00000213548.3	ENSG00000248295.2	ENSG00000255182.2
22380	ENSG00000213549.3	ENSG00000248294.1	ENSG00000211728.2
22381	ENSG00000213551.5	ENSG00000248293.1	ENSG00000012171.19
22382	ENSG00000213553.4	ENSG00000248290.1	ENSG00000106829.18
22383	ENSG00000213556.3	ENSG00000248288.1	ENSG00000138107.13
22384	ENSG00000213557.4	ENSG00000248287.1	ENSG00000111012.10
22385	ENSG00000213558.3	ENSG00000248286.1	ENSG00000166342.19
22386	ENSG00000213559.4	ENSG00000248285.1	ENSG00000090554.13
22387	ENSG00000213560.4	ENSG00000248283.2	ENSG00000231799.2
22388	ENSG00000213561.4	ENSG00000248282.1	ENSG00000245317.2
22389	ENSG00000213563.6	ENSG00000248281.1	ENSG00000144749.13
22390	ENSG00000213568.4	ENSG00000248279.5	ENSG00000279733.1
22391	ENSG00000213569.4	ENSG00000248278.1	ENSG00000152128.13
22392	ENSG00000213574.2	ENSG00000248275.1	ENSG00000172572.6
22393	ENSG00000213578.6	ENSG00000248271.1	ENSG00000234913.1
22394	ENSG00000213579.3	ENSG00000248268.1	ENSG00000285629.1
22395	ENSG00000213585.11	ENSG00000248266.1	ENSG00000148288.13
22396	ENSG00000213587.3	ENSG00000248265.2	ENSG00000224321.1
22397	ENSG00000213588.6	ENSG00000248262.1	ENSG00000230697.1
22398	ENSG00000213590.2	ENSG00000248261.1	ENSG00000088970.16
22399	ENSG00000213592.4	ENSG00000248259.1	ENSG00000116285.13
22400	ENSG00000213593.10	ENSG00000248257.7	ENSG00000146722.11
22401	ENSG00000213594.4	ENSG00000248256.1	ENSG00000241685.10
22402	ENSG00000213598.4	ENSG00000248254.1	ENSG00000232810.4
22403	ENSG00000213599.10	ENSG00000248249.1	ENSG00000232713.2
22404	ENSG00000213600.3	ENSG00000248245.1	ENSG00000180409.3
22405	ENSG00000213601.3	ENSG00000248243.1	ENSG00000152061.23
22406	ENSG00000213604.4	ENSG00000248242.1	ENSG00000113361.13
22407	ENSG00000213605.2	ENSG00000248240.1	ENSG00000227388.2
22408	ENSG00000213606.3	ENSG00000248238.1	ENSG00000232458.1
22409	ENSG00000213607.4	ENSG00000248237.2	ENSG00000171873.7
22410	ENSG00000213608.5	ENSG00000248236.1	ENSG00000215022.7
22411	ENSG00000213609.3	ENSG00000248235.6	ENSG00000032742.17
22412	ENSG00000213612.3	ENSG00000248234.1	ENSG00000130962.17
22413	ENSG00000213613.2	ENSG00000248231.1	ENSG00000152503.9
22414	ENSG00000213614.9	ENSG00000248229.1	ENSG00000276747.1
22415	ENSG00000213619.10	ENSG00000248228.1	ENSG00000266913.1
22416	ENSG00000213620.3	ENSG00000248227.1	ENSG00000110987.8

22417	ENSG00000213621.3	ENSG00000248223.1	ENSG00000167173.19
22418	ENSG00000213625.9	ENSG00000248222.5	ENSG00000229918.1
22419	ENSG00000213626.13	ENSG00000248221.1	ENSG00000273951.1
22420	ENSG00000213630.3	ENSG00000248216.1	ENSG00000187980.6
22421	ENSG00000213638.6	ENSG00000248215.5	ENSG00000228719.1
22422	ENSG00000213639.10	ENSG00000248213.3	ENSG00000179799.9
22423	ENSG00000213640.3	ENSG00000248211.1	ENSG00000176092.15
22424	ENSG00000213641.4	ENSG00000248210.1	ENSG00000239415.1
22425	ENSG00000213642.3	ENSG00000248209.1	ENSG00000211789.2
22426	ENSG00000213643.3	ENSG00000248208.1	ENSG00000267453.7
22427	ENSG00000213644.2	ENSG00000248206.1	ENSG00000101353.14
22428	ENSG00000213645.2	ENSG00000248205.1	ENSG00000270000.1
22429	ENSG00000213648.10	ENSG00000248203.1	ENSG00000225632.1
22430	ENSG00000213650.3	ENSG00000248202.1	ENSG00000261614.1
22431	ENSG00000213652.3	ENSG00000248200.2	ENSG00000129625.13
22432	ENSG00000213653.4	ENSG00000248199.1	ENSG00000243015.2
22433	ENSG00000213654.10	ENSG00000248197.1	ENSG00000213237.4
22434	ENSG00000213655.4	ENSG00000248196.1	ENSG00000140323.6
22435	ENSG00000213657.3	ENSG00000248195.1	ENSG00000238152.2
22436	ENSG00000213658.11	ENSG00000248192.1	ENSG00000183963.18
22437	ENSG00000213659.4	ENSG00000248191.1	ENSG00000275236.1
22438	ENSG00000213661.3	ENSG00000248188.2	ENSG00000235459.5
22439	ENSG00000213663.4	ENSG00000248187.1	ENSG00000244733.5
22440	ENSG00000213664.4	ENSG00000248185.1	ENSG00000283689.1
22441	ENSG00000213667.3	ENSG00000248184.1	ENSG00000151468.11
22442	ENSG00000213669.2	ENSG00000248180.1	ENSG00000233836.7
22443	ENSG00000213671.3	ENSG00000248176.1	ENSG00000261534.1
22444	ENSG00000213672.8	ENSG00000248174.5	ENSG00000240288.7
22445	ENSG00000213673.3	ENSG00000248172.1	ENSG00000155962.13
22446	ENSG00000213676.13	ENSG00000248170.1	ENSG00000164331.10
22447	ENSG00000213683.4	ENSG00000248167.7	ENSG00000172348.14
22448	ENSG00000213684.4	ENSG00000248165.1	ENSG00000272115.1
22449	ENSG00000213689.14	ENSG00000248162.1	ENSG00000221705.1
22450	ENSG00000213690.3	ENSG00000248161.5	ENSG00000235374.2
22451	ENSG00000213693.4	ENSG00000248160.1	ENSG00000268869.5
22452	ENSG00000213694.5	ENSG00000248159.1	ENSG00000265148.5
22453	ENSG00000213695.3	ENSG00000248156.2	ENSG00000286264.1
22454	ENSG00000213697.3	ENSG00000248155.1	ENSG00000283052.1
22455	ENSG00000213698.2	ENSG00000248152.1	ENSG00000186314.11
22456	ENSG00000213699.9	ENSG00000248150.1	ENSG00000215154.6
22457	ENSG00000213700.3	ENSG00000248148.1	ENSG00000239002.3
22458	ENSG00000213701.4	ENSG00000248145.1	ENSG00000213147.3
22459	ENSG00000213703.2	ENSG00000248144.6	ENSG00000260005.6
22460	ENSG00000213704.3	ENSG00000248143.1	ENSG00000279407.1
22461	ENSG00000213706.2	ENSG00000248139.2	ENSG00000153914.16
22462	ENSG00000213707.2	ENSG00000248138.5	ENSG00000169093.16
22463	ENSG00000213708.6	ENSG00000248137.1	ENSG00000079156.17
22464	ENSG00000213711.3	ENSG00000248136.1	ENSG00000184925.12
22465	ENSG00000213713.3	ENSG00000248134.1	ENSG00000204262.13
22466	ENSG00000213714.1	ENSG00000248133.1	ENSG00000103479.16
22467	ENSG00000213716.3	ENSG00000248132.2	ENSG00000172409.6
22468	ENSG00000213717.3	ENSG00000248131.5	ENSG00000213605.2
22469	ENSG00000213719.8	ENSG00000248128.1	ENSG00000052802.13

22470	ENSG00000213721.3	ENSG00000248127.1	ENSG00000251623.2
22471	ENSG00000213722.9	ENSG00000248126.1	ENSG00000261158.1
22472	ENSG00000213724.4	ENSG00000248125.1	ENSG00000273837.1
22473	ENSG00000213726.5	ENSG00000248124.7	ENSG00000184500.15
22474	ENSG00000213727.3	ENSG00000248122.1	ENSG00000285545.1
22475	ENSG00000213729.3	ENSG00000248121.8	ENSG00000037280.16
22476	ENSG00000213730.3	ENSG00000248120.1	ENSG00000108244.16
22477	ENSG00000213731.2	ENSG00000248118.1	ENSG00000114529.12
22478	ENSG00000213735.2	ENSG00000248117.1	ENSG00000240418.1
22479	ENSG00000213736.2	ENSG00000248115.1	ENSG00000196730.13
22480	ENSG00000213739.3	ENSG00000248114.1	ENSG00000258546.1
22481	ENSG00000213740.2	ENSG00000248113.2	ENSG00000261532.1
22482	ENSG00000213741.10	ENSG00000248112.1	ENSG00000175445.16
22483	ENSG00000213742.6	ENSG00000248109.2	ENSG00000139687.15
22484	ENSG00000213744.3	ENSG00000248107.1	ENSG00000243646.10
22485	ENSG00000213747.2	ENSG00000248106.2	ENSG00000229715.5
22486	ENSG00000213750.4	ENSG00000248105.1	ENSG00000106443.16
22487	ENSG00000213752.3	ENSG00000248104.1	ENSG00000182912.6
22488	ENSG00000213753.11	ENSG00000248103.1	ENSG00000187595.16
22489	ENSG00000213754.2	ENSG00000248101.2	ENSG00000152784.15
22490	ENSG00000213755.3	ENSG00000248100.2	ENSG00000266049.1
22491	ENSG00000213757.3	ENSG00000248099.4	ENSG00000224578.5
22492	ENSG00000213759.9	ENSG00000248098.12	ENSG00000229590.3
22493	ENSG00000213760.10	ENSG00000248092.7	ENSG00000279041.1
22494	ENSG00000213761.4	ENSG00000248079.2	ENSG00000225880.5
22495	ENSG00000213762.12	ENSG00000248050.1	ENSG00000241484.9
22496	ENSG00000213763.4	ENSG00000248049.6	ENSG00000128245.15
22497	ENSG00000213770.3	ENSG00000248029.2	ENSG00000259080.1
22498	ENSG00000213771.3	ENSG00000248027.1	ENSG00000185262.9
22499	ENSG00000213772.3	ENSG00000248019.2	ENSG00000228335.1
22500	ENSG00000213774.3	ENSG00000248015.7	ENSG00000260196.1
22501	ENSG00000213777.5	ENSG00000248008.2	ENSG00000139618.14
22502	ENSG00000213778.2	ENSG00000247993.2	ENSG00000197467.14
22503	ENSG00000213779.4	ENSG00000247982.6	ENSG00000286256.1
22504	ENSG00000213780.11	ENSG00000247970.2	ENSG00000226254.1
22505	ENSG00000213781.3	ENSG00000247950.6	ENSG00000246375.2
22506	ENSG00000213782.7	ENSG00000247925.2	ENSG00000170275.14
22507	ENSG00000213783.4	ENSG00000247911.3	ENSG00000085231.14
22508	ENSG00000213785.3	ENSG00000247903.1	ENSG00000276390.1
22509	ENSG00000213786.3	ENSG00000247877.6	ENSG00000125900.13
22510	ENSG00000213787.3	ENSG00000247872.2	ENSG00000273604.1
22511	ENSG00000213790.2	ENSG00000247867.2	ENSG00000272410.5
22512	ENSG00000213791.4	ENSG00000247828.7	ENSG00000112077.16
22513	ENSG00000213793.5	ENSG00000247810.7	ENSG00000100321.15
22514	ENSG00000213798.3	ENSG00000247809.7	ENSG00000254501.1
22515	ENSG00000213799.12	ENSG00000247796.2	ENSG00000280325.1
22516	ENSG00000213801.4	ENSG00000247775.2	ENSG00000168062.10
22517	ENSG00000213809.9	ENSG00000247774.6	ENSG00000285671.1
22518	ENSG00000213816.3	ENSG00000247765.2	ENSG00000088930.8
22519	ENSG00000213820.3	ENSG00000247763.2	ENSG00000267767.2
22520	ENSG00000213822.6	ENSG00000247746.4	ENSG00000116754.13
22521	ENSG00000213830.3	ENSG00000247735.2	ENSG00000169692.13
22522	ENSG00000213839.4	ENSG00000247728.2	ENSG00000233820.2

22523	ENSG00000213842.2	ENSG00000247708.7	ENSG00000254263.1
22524	ENSG00000213849.3	ENSG00000247699.2	ENSG00000183668.18
22525	ENSG00000213851.3	ENSG00000247679.2	ENSG00000157445.15
22526	ENSG00000213853.10	ENSG00000247675.6	ENSG00000259700.3
22527	ENSG00000213854.3	ENSG00000247627.2	ENSG00000126870.16
22528	ENSG00000213856.3	ENSG00000247626.4	ENSG00000177946.6
22529	ENSG00000213857.3	ENSG00000247624.6	ENSG00000186642.16
22530	ENSG00000213859.6	ENSG00000247596.9	ENSG00000268785.1
22531	ENSG00000213860.4	ENSG00000247595.4	ENSG00000259654.1
22532	ENSG00000213862.4	ENSG00000247572.7	ENSG00000272434.1
22533	ENSG00000213863.2	ENSG00000247570.2	ENSG00000104695.13
22534	ENSG00000213864.3	ENSG00000247556.6	ENSG00000247679.2
22535	ENSG00000213865.7	ENSG00000247516.7	ENSG00000239945.1
22536	ENSG00000213866.3	ENSG00000247498.9	ENSG00000260908.1
22537	ENSG00000213867.4	ENSG00000247473.2	ENSG00000259118.5
22538	ENSG00000213871.3	ENSG00000247416.3	ENSG00000228606.2
22539	ENSG00000213872.3	ENSG00000247402.2	ENSG00000259884.1
22540	ENSG00000213873.4	ENSG00000247400.3	ENSG00000049860.14
22541	ENSG00000213875.3	ENSG00000247381.3	ENSG00000103051.19
22542	ENSG00000213876.4	ENSG00000247373.3	ENSG00000250305.9
22543	ENSG00000213877.3	ENSG00000247372.2	ENSG00000268500.5
22544	ENSG00000213880.3	ENSG00000247363.2	ENSG00000141519.15
22545	ENSG00000213881.3	ENSG00000247345.2	ENSG00000177981.11
22546	ENSG00000213882.2	ENSG00000247324.2	ENSG00000148826.9
22547	ENSG00000213885.3	ENSG00000247317.3	ENSG00000267735.1
22548	ENSG00000213886.3	ENSG00000247311.2	ENSG00000119900.9
22549	ENSG00000213888.3	ENSG00000247287.2	ENSG00000234624.2
22550	ENSG00000213889.10	ENSG00000247271.6	ENSG00000173080.5
22551	ENSG00000213891.3	ENSG00000247240.7	ENSG00000260193.1
22552	ENSG00000213892.12	ENSG00000247228.2	ENSG00000232133.1
22553	ENSG00000213896.4	ENSG00000247213.6	ENSG00000237651.6
22554	ENSG00000213900.2	ENSG00000247199.4	ENSG00000250654.7
22555	ENSG00000213901.10	ENSG00000247193.2	ENSG00000236349.1
22556	ENSG00000213903.9	ENSG00000247157.6	ENSG00000075420.13
22557	ENSG00000213904.8	ENSG00000247151.7	ENSG00000162889.10
22558	ENSG00000213906.10	ENSG00000247137.8	ENSG00000134463.15
22559	ENSG00000213908.4	ENSG00000247134.6	ENSG00000010030.14
22560	ENSG00000213911.2	ENSG00000247131.5	ENSG00000065618.20
22561	ENSG00000213916.2	ENSG00000247130.2	ENSG00000174607.11
22562	ENSG00000213917.2	ENSG00000247121.6	ENSG00000100604.13
22563	ENSG00000213918.10	ENSG00000247095.2	ENSG00000137275.14
22564	ENSG00000213920.9	ENSG00000247092.6	ENSG00000276633.1
22565	ENSG00000213921.7	ENSG00000247081.7	ENSG00000116871.15
22566	ENSG00000213923.12	ENSG00000247077.7	ENSG00000285581.1
22567	ENSG00000213924.3	ENSG00000247049.2	ENSG00000205106.4
22568	ENSG00000213925.3	ENSG00000247033.1	ENSG00000196420.7
22569	ENSG00000213926.3	ENSG00000247011.2	ENSG00000049192.15
22570	ENSG00000213927.3	ENSG00000246985.7	ENSG00000271380.1
22571	ENSG00000213928.8	ENSG00000246982.6	ENSG00000178297.13
22572	ENSG00000213930.11	ENSG00000246922.8	ENSG00000231999.6
22573	ENSG00000213931.7	ENSG00000246898.1	ENSG00000275450.1
22574	ENSG00000213934.8	ENSG00000246877.1	ENSG00000196917.5
22575	ENSG00000213935.3	ENSG00000246876.6	ENSG00000259562.2

22576	ENSG00000213937.4	ENSG00000246863.2	ENSG00000160539.6
22577	ENSG00000213938.3	ENSG00000246859.2	ENSG00000130347.12
22578	ENSG00000213939.4	ENSG00000246851.1	ENSG00000111602.12
22579	ENSG00000213940.4	ENSG00000246820.2	ENSG00000137860.12
22580	ENSG00000213942.3	ENSG00000246792.2	ENSG00000231369.1
22581	ENSG00000213943.3	ENSG00000246790.2	ENSG00000104388.15
22582	ENSG00000213946.3	ENSG00000246777.1	ENSG00000198744.5
22583	ENSG00000213949.9	ENSG00000246774.1	ENSG00000120708.17
22584	ENSG00000213950.2	ENSG00000246763.6	ENSG00000157680.15
22585	ENSG00000213954.3	ENSG00000246740.2	ENSG00000151657.12
22586	ENSG00000213956.4	ENSG00000246731.2	ENSG00000108828.16
22587	ENSG00000213958.2	ENSG00000246705.4	ENSG00000237505.7
22588	ENSG00000213959.2	ENSG00000246695.7	ENSG00000184984.9
22589	ENSG00000213962.2	ENSG00000246662.6	ENSG00000126460.11
22590	ENSG00000213963.6	ENSG00000246640.1	ENSG00000215769.8
22591	ENSG00000213964.3	ENSG00000246627.6	ENSG00000225087.1
22592	ENSG00000213965.3	ENSG00000246596.7	ENSG00000140459.18
22593	ENSG00000213967.10	ENSG00000246582.2	ENSG00000134996.11
22594	ENSG00000213970.4	ENSG00000246575.2	ENSG00000213672.8
22595	ENSG00000213972.3	ENSG00000246560.2	ENSG00000102870.6
22596	ENSG00000213973.9	ENSG00000246548.3	ENSG00000256898.1
22597	ENSG00000213976.4	ENSG00000246541.2	ENSG00000143603.19
22598	ENSG00000213977.8	ENSG00000246528.3	ENSG00000236254.1
22599	ENSG00000213979.3	ENSG00000246526.2	ENSG00000285723.1
22600	ENSG00000213981.8	ENSG00000246523.7	ENSG00000143149.12
22601	ENSG00000213983.11	ENSG00000246477.3	ENSG00000138381.10
22602	ENSG00000213985.4	ENSG00000246465.1	ENSG00000121680.16
22603	ENSG00000213987.4	ENSG00000246451.2	ENSG00000164828.18
22604	ENSG00000213988.11	ENSG00000246430.6	ENSG00000152484.14
22605	ENSG00000213994.3	ENSG00000246422.2	ENSG00000261044.1
22606	ENSG00000213995.11	ENSG00000246394.7	ENSG00000279528.1
22607	ENSG00000213996.13	ENSG00000246379.7	ENSG00000077514.8
22608	ENSG00000213997.3	ENSG00000246375.2	ENSG00000043514.16
22609	ENSG00000213999.16	ENSG00000246366.6	ENSG00000284862.2
22610	ENSG00000214003.2	ENSG00000246363.3	ENSG00000184206.11
22611	ENSG00000214009.2	ENSG00000246350.1	ENSG00000168066.20
22612	ENSG00000214012.4	ENSG00000246339.5	ENSG00000268509.2
22613	ENSG00000214013.9	ENSG00000246334.2	ENSG00000275383.1
22614	ENSG00000214015.3	ENSG00000246331.2	ENSG00000120690.16
22615	ENSG00000214016.3	ENSG00000246323.2	ENSG00000211895.5
22616	ENSG00000214018.3	ENSG00000246316.7	ENSG00000279227.1
22617	ENSG00000214019.2	ENSG00000246308.1	ENSG00000206034.1
22618	ENSG00000214020.3	ENSG00000246283.2	ENSG00000211781.3
22619	ENSG00000214021.16	ENSG00000246273.7	ENSG00000162607.13
22620	ENSG00000214022.11	ENSG00000246263.2	ENSG00000250254.2
22621	ENSG00000214024.2	ENSG00000246250.2	ENSG00000230147.2
22622	ENSG00000214025.2	ENSG00000246228.6	ENSG00000163961.4
22623	ENSG00000214026.11	ENSG00000246225.6	ENSG00000004468.13
22624	ENSG00000214027.3	ENSG00000246223.8	ENSG00000136273.13
22625	ENSG00000214029.5	ENSG00000246214.1	ENSG00000262050.1
22626	ENSG00000214031.2	ENSG00000246211.2	ENSG00000269911.1
22627	ENSG00000214035.3	ENSG00000246203.2	ENSG00000168026.18
22628	ENSG00000214039.8	ENSG00000246174.7	ENSG00000172458.4

22629	ENSG00000214041.4	ENSG00000246145.1	ENSG00000254416.5
22630	ENSG00000214042.1	ENSG00000246130.1	ENSG00000240889.1
22631	ENSG00000214043.7	ENSG00000246115.3	ENSG00000234857.2
22632	ENSG00000214045.3	ENSG00000246100.3	ENSG00000173327.8
22633	ENSG00000214046.8	ENSG00000246095.2	ENSG00000159596.7
22634	ENSG00000214047.4	ENSG00000246090.6	ENSG00000177917.10
22635	ENSG00000214049.7	ENSG00000246089.3	ENSG00000125895.5
22636	ENSG00000214050.8	ENSG00000246084.2	ENSG00000163697.17
22637	ENSG00000214051.3	ENSG00000246082.2	ENSG00000228113.7
22638	ENSG00000214062.5	ENSG00000246067.7	ENSG00000102053.12
22639	ENSG00000214063.11	ENSG00000246022.2	ENSG00000261717.5
22640	ENSG00000214064.3	ENSG00000246016.2	ENSG00000253106.1
22641	ENSG00000214067.2	ENSG00000245975.2	ENSG00000274423.1
22642	ENSG00000214070.3	ENSG00000245970.2	ENSG00000141750.7
22643	ENSG00000214071.4	ENSG00000245958.6	ENSG00000103148.15
22644	ENSG00000214073.2	ENSG00000245954.7	ENSG00000158578.20
22645	ENSG00000214074.3	ENSG00000245937.7	ENSG00000237311.2
22646	ENSG00000214076.3	ENSG00000245928.2	ENSG00000263466.1
22647	ENSG00000214077.4	ENSG00000245910.8	ENSG00000267264.1
22648	ENSG00000214078.12	ENSG00000245904.3	ENSG00000224438.3
22649	ENSG00000214081.8	ENSG00000245888.6	ENSG00000204590.12
22650	ENSG00000214087.8	ENSG00000245870.3	ENSG00000236393.1
22651	ENSG00000214089.3	ENSG00000245869.2	ENSG00000201492.1
22652	ENSG00000214093.3	ENSG00000245864.2	ENSG00000240459.2
22653	ENSG00000214097.5	ENSG00000245857.2	ENSG00000186710.11
22654	ENSG00000214100.8	ENSG00000245849.7	ENSG00000248527.1
22655	ENSG00000214102.7	ENSG00000245848.3	ENSG00000241352.3
22656	ENSG00000214106.8	ENSG00000245832.6	ENSG00000100462.16
22657	ENSG00000214107.8	ENSG00000245812.2	ENSG00000260063.1
22658	ENSG00000214108.4	ENSG00000245768.6	ENSG00000246526.2
22659	ENSG00000214110.3	ENSG00000245750.8	ENSG00000228624.7
22660	ENSG00000214111.3	ENSG00000245748.1	ENSG00000145191.14
22661	ENSG00000214113.10	ENSG00000245729.2	ENSG00000225791.6
22662	ENSG00000214114.9	ENSG00000245719.1	ENSG00000240231.1
22663	ENSG00000214121.4	ENSG00000245711.2	ENSG00000112079.9
22664	ENSG00000214124.3	ENSG00000245688.1	ENSG00000198003.12
22665	ENSG00000214125.2	ENSG00000245685.6	ENSG00000255189.1
22666	ENSG00000214128.11	ENSG00000245680.10	ENSG00000247903.1
22667	ENSG00000214132.4	ENSG00000245667.2	ENSG00000285258.1
22668	ENSG00000214135.8	ENSG00000245662.3	ENSG00000223891.5
22669	ENSG00000214140.11	ENSG00000245651.2	ENSG00000136866.13
22670	ENSG00000214141.4	ENSG00000245648.1	ENSG00000151743.11
22671	ENSG00000214142.2	ENSG00000245614.3	ENSG00000166743.9
22672	ENSG00000214144.3	ENSG00000245598.6	ENSG00000230482.1
22673	ENSG00000214145.6	ENSG00000245573.8	ENSG00000188613.7
22674	ENSG00000214146.2	ENSG00000245571.6	ENSG00000248713.1
22675	ENSG00000214147.2	ENSG00000245556.2	ENSG00000245146.6
22676	ENSG00000214160.9	ENSG00000245552.6	ENSG00000198719.9
22677	ENSG00000214161.3	ENSG00000245532.8	ENSG00000122483.17
22678	ENSG00000214174.8	ENSG00000245526.10	ENSG00000086288.11
22679	ENSG00000214176.9	ENSG00000245522.2	ENSG00000141452.9
22680	ENSG00000214182.5	ENSG00000245498.6	ENSG00000176563.9
22681	ENSG00000214184.3	ENSG00000245482.2	ENSG00000177181.15

22682	ENSG00000214185.3	ENSG00000245479.2	ENSG00000285813.1
22683	ENSG00000214188.9	ENSG00000245468.3	ENSG00000123124.13
22684	ENSG00000214189.9	ENSG00000245385.2	ENSG00000259079.1
22685	ENSG00000214190.2	ENSG00000245384.1	ENSG00000265118.5
22686	ENSG00000214192.3	ENSG00000245330.4	ENSG00000169402.15
22687	ENSG00000214193.10	ENSG00000245322.6	ENSG00000083845.9
22688	ENSG00000214194.9	ENSG00000245311.2	ENSG00000065882.16
22689	ENSG00000214195.4	ENSG00000245293.2	ENSG00000271121.2
22690	ENSG00000214198.8	ENSG00000245281.7	ENSG00000198728.10
22691	ENSG00000214199.3	ENSG00000245275.7	ENSG00000141627.13
22692	ENSG00000214200.2	ENSG00000245261.1	ENSG00000122861.16
22693	ENSG00000214203.4	ENSG00000245248.7	ENSG00000102699.6
22694	ENSG00000214204.4	ENSG00000245213.6	ENSG00000227383.1
22695	ENSG00000214207.2	ENSG00000245205.3	ENSG00000245017.2
22696	ENSG00000214210.4	ENSG00000245164.7	ENSG00000267681.1
22697	ENSG00000214211.2	ENSG00000245156.1	ENSG00000092051.17
22698	ENSG00000214212.9	ENSG00000245149.3	ENSG00000141293.16
22699	ENSG00000214216.10	ENSG00000245148.2	ENSG00000184916.9
22700	ENSG00000214222.2	ENSG00000245146.6	ENSG00000250850.2
22701	ENSG00000214223.4	ENSG00000245112.2	ENSG00000263624.1
22702	ENSG00000214226.9	ENSG00000245105.3	ENSG00000115145.10
22703	ENSG00000214237.10	ENSG00000245080.7	ENSG00000139182.14
22704	ENSG00000214243.3	ENSG00000245067.6	ENSG00000166682.12
22705	ENSG00000214244.4	ENSG00000245060.7	ENSG00000123570.4
22706	ENSG00000214245.3	ENSG00000245059.2	ENSG00000217416.4
22707	ENSG00000214248.2	ENSG00000245025.2	ENSG00000157916.20
22708	ENSG00000214249.3	ENSG00000245017.2	ENSG00000068831.19
22709	ENSG00000214252.4	ENSG00000245008.4	ENSG00000177426.20
22710	ENSG00000214253.8	ENSG00000244998.1	ENSG00000160460.16
22711	ENSG00000214254.3	ENSG00000244968.6	ENSG00000254094.1
22712	ENSG00000214255.5	ENSG00000244953.1	ENSG00000077152.10
22713	ENSG00000214259.3	ENSG00000244952.2	ENSG00000230216.1
22714	ENSG00000214262.4	ENSG00000244945.1	ENSG00000129250.12
22715	ENSG00000214263.2	ENSG00000244932.2	ENSG00000149573.9
22716	ENSG00000214264.4	ENSG00000244926.6	ENSG00000273355.1
22717	ENSG00000214265.11	ENSG00000244921.2	ENSG00000236948.2
22718	ENSG00000214266.2	ENSG00000244879.7	ENSG00000065320.9
22719	ENSG00000214268.2	ENSG00000244791.2	ENSG00000225165.3
22720	ENSG00000214269.3	ENSG00000244757.1	ENSG00000104671.8
22721	ENSG00000214273.4	ENSG00000244756.1	ENSG00000248636.6
22722	ENSG00000214274.9	ENSG00000244754.8	ENSG00000165475.15
22723	ENSG00000214278.4	ENSG00000244753.2	ENSG00000265241.6
22724	ENSG00000214279.13	ENSG00000244752.3	ENSG00000240180.1
22725	ENSG00000214280.3	ENSG00000244748.3	ENSG00000105619.13
22726	ENSG00000214281.3	ENSG00000244743.1	ENSG00000125347.14
22727	ENSG00000214282.3	ENSG00000244740.1	ENSG00000211611.2
22728	ENSG00000214283.4	ENSG00000244738.1	ENSG00000004961.15
22729	ENSG00000214285.2	ENSG00000244732.3	ENSG00000225569.1
22730	ENSG00000214286.2	ENSG00000244731.8	ENSG00000061337.15
22731	ENSG00000214288.4	ENSG00000244730.1	ENSG00000157827.20
22732	ENSG00000214289.2	ENSG00000244723.3	ENSG00000230260.1
22733	ENSG00000214290.8	ENSG00000244722.1	ENSG00000023572.9
22734	ENSG00000214293.8	ENSG00000244720.1	ENSG00000163071.11

22735	ENSG00000214295.4	ENSG00000244717.1	ENSG00000243313.2
22736	ENSG00000214297.3	ENSG00000244716.3	ENSG00000273669.1
22737	ENSG00000214298.3	ENSG00000244712.1	ENSG00000273302.1
22738	ENSG00000214300.7	ENSG00000244710.3	ENSG00000232860.7
22739	ENSG00000214301.4	ENSG00000244708.1	ENSG00000146410.12
22740	ENSG00000214305.4	ENSG00000244706.3	ENSG00000063015.19
22741	ENSG00000214309.5	ENSG00000244705.1	ENSG00000198553.9
22742	ENSG00000214313.8	ENSG00000244703.3	ENSG00000131115.16
22743	ENSG00000214318.3	ENSG00000244701.1	ENSG00000279278.1
22744	ENSG00000214319.2	ENSG00000244699.1	ENSG00000228782.7
22745	ENSG00000214321.3	ENSG00000244694.7	ENSG00000273382.1
22746	ENSG00000214322.3	ENSG00000244693.1	ENSG00000273271.1
22747	ENSG00000214324.5	ENSG00000244692.3	ENSG00000175470.20
22748	ENSG00000214326.2	ENSG00000244691.1	ENSG00000147872.10
22749	ENSG00000214329.7	ENSG00000244687.11	ENSG00000065361.15
22750	ENSG00000214330.4	ENSG00000244682.7	ENSG00000168564.6
22751	ENSG00000214331.8	ENSG00000244681.1	ENSG00000083937.9
22752	ENSG00000214335.3	ENSG00000244677.3	ENSG00000227348.1
22753	ENSG00000214336.4	ENSG00000244676.5	ENSG00000213923.12
22754	ENSG00000214338.10	ENSG00000244675.2	ENSG00000256128.5
22755	ENSG00000214342.2	ENSG00000244674.1	ENSG00000242797.3
22756	ENSG00000214344.4	ENSG00000244671.3	ENSG00000231794.5
22757	ENSG00000214347.8	ENSG00000244669.1	ENSG00000144824.20
22758	ENSG00000214351.5	ENSG00000244668.1	ENSG00000261678.3
22759	ENSG00000214353.7	ENSG00000244662.1	ENSG00000104722.14
22760	ENSG00000214354.3	ENSG00000244661.1	ENSG00000214914.3
22761	ENSG00000214357.8	ENSG00000244657.1	ENSG00000180846.8
22762	ENSG00000214359.3	ENSG00000244653.1	ENSG00000037897.17
22763	ENSG00000214360.4	ENSG00000244652.1	ENSG00000226266.6
22764	ENSG00000214362.2	ENSG00000244650.2	ENSG00000228218.1
22765	ENSG00000214366.4	ENSG00000244646.4	ENSG00000120159.13
22766	ENSG00000214367.7	ENSG00000244642.3	ENSG00000105251.10
22767	ENSG00000214369.2	ENSG00000244641.2	ENSG00000232176.1
22768	ENSG00000214374.2	ENSG00000244640.1	ENSG00000274363.1
22769	ENSG00000214376.5	ENSG00000244632.3	ENSG00000100181.22
22770	ENSG00000214380.4	ENSG00000244630.2	ENSG00000260855.1
22771	ENSG00000214381.5	ENSG00000244627.5	ENSG00000197582.5
22772	ENSG00000214389.2	ENSG00000244625.6	ENSG00000273447.1
22773	ENSG00000214391.3	ENSG00000244624.3	ENSG00000171428.14
22774	ENSG00000214401.4	ENSG00000244623.1	ENSG00000112139.16
22775	ENSG00000214402.6	ENSG00000244621.1	ENSG00000261286.1
22776	ENSG00000214405.3	ENSG00000244620.1	ENSG00000124875.10
22777	ENSG00000214407.3	ENSG00000244619.2	ENSG00000273466.1
22778	ENSG00000214413.8	ENSG00000244618.3	ENSG00000255201.1
22779	ENSG00000214414.9	ENSG00000244617.2	ENSG00000261773.1
22780	ENSG00000214415.3	ENSG00000244615.1	ENSG00000242736.1
22781	ENSG00000214417.4	ENSG00000244610.3	ENSG00000242071.3
22782	ENSG00000214424.5	ENSG00000244607.6	ENSG00000107447.8
22783	ENSG00000214425.7	ENSG00000244604.1	ENSG00000228061.6
22784	ENSG00000214428.3	ENSG00000244593.1	ENSG00000144028.15
22785	ENSG00000214429.3	ENSG00000244588.5	ENSG00000150401.15
22786	ENSG00000214432.9	ENSG00000244586.1	ENSG00000221978.12
22787	ENSG00000214433.4	ENSG00000244585.1	ENSG00000262905.1

22788	ENSG00000214434.2	ENSG00000244582.2	ENSG00000273907.1
22789	ENSG00000214435.8	ENSG00000244578.1	ENSG00000248554.1
22790	ENSG00000214439.4	ENSG00000244575.3	ENSG00000108417.3
22791	ENSG00000214447.4	ENSG00000244573.3	ENSG00000204323.5
22792	ENSG00000214455.4	ENSG00000244571.1	ENSG00000108518.7
22793	ENSG00000214456.8	ENSG00000244568.3	ENSG00000274421.1
22794	ENSG00000214457.3	ENSG00000244565.1	ENSG00000260661.1
22795	ENSG00000214460.3	ENSG00000244564.1	ENSG00000064115.11
22796	ENSG00000214465.3	ENSG00000244563.1	ENSG00000124383.9
22797	ENSG00000214484.3	ENSG00000244561.1	ENSG00000138185.20
22798	ENSG00000214485.6	ENSG00000244560.7	ENSG00000172794.20
22799	ENSG00000214487.3	ENSG00000244559.1	ENSG00000282875.1
22800	ENSG00000214491.8	ENSG00000244558.5	ENSG00000154188.10
22801	ENSG00000214510.10	ENSG00000244556.1	ENSG00000223534.1
22802	ENSG00000214511.3	ENSG00000244551.2	ENSG00000025434.19
22803	ENSG00000214513.3	ENSG00000244550.1	ENSG00000277232.2
22804	ENSG00000214514.8	ENSG00000244545.1	ENSG00000143502.15
22805	ENSG00000214517.10	ENSG00000244544.2	ENSG00000186187.11
22806	ENSG00000214518.3	ENSG00000244541.5	ENSG00000230825.1
22807	ENSG00000214525.4	ENSG00000244540.2	ENSG00000134323.12
22808	ENSG00000214526.3	ENSG00000244538.1	ENSG00000237633.3
22809	ENSG00000214530.9	ENSG00000244537.2	ENSG00000143768.13
22810	ENSG00000214533.3	ENSG00000244535.1	ENSG00000135318.12
22811	ENSG00000214534.5	ENSG00000244534.3	ENSG00000229325.1
22812	ENSG00000214535.3	ENSG00000244532.1	ENSG00000248508.7
22813	ENSG00000214541.3	ENSG00000244528.1	ENSG00000183011.13
22814	ENSG00000214544.7	ENSG00000244527.1	ENSG00000270755.1
22815	ENSG00000214546.3	ENSG00000244521.3	ENSG00000011132.12
22816	ENSG00000214548.17	ENSG00000244515.1	ENSG00000046604.13
22817	ENSG00000214549.2	ENSG00000244514.3	ENSG00000275441.1
22818	ENSG00000214552.4	ENSG00000244513.6	ENSG00000254929.6
22819	ENSG00000214553.10	ENSG00000244512.3	ENSG00000196381.11
22820	ENSG00000214558.4	ENSG00000244510.2	ENSG00000159640.16
22821	ENSG00000214559.3	ENSG00000244509.4	ENSG00000123643.13
22822	ENSG00000214560.4	ENSG00000244503.1	ENSG00000130813.18
22823	ENSG00000214561.3	ENSG00000244502.2	ENSG00000226751.2
22824	ENSG00000214562.14	ENSG00000244501.3	ENSG00000273319.1
22825	ENSG00000214563.2	ENSG00000244493.1	ENSG00000140199.11
22826	ENSG00000214575.9	ENSG00000244491.1	ENSG00000171570.11
22827	ENSG00000214578.5	ENSG00000244490.1	ENSG00000181555.20
22828	ENSG00000214581.6	ENSG00000244486.8	ENSG00000263412.1
22829	ENSG00000214584.3	ENSG00000244485.1	ENSG00000258499.1
22830	ENSG00000214593.3	ENSG00000244482.10	ENSG00000280129.1
22831	ENSG00000214595.11	ENSG00000244480.1	ENSG00000140650.12
22832	ENSG00000214602.3	ENSG00000244479.7	ENSG00000268189.2
22833	ENSG00000214604.3	ENSG00000244476.3	ENSG00000279706.1
22834	ENSG00000214607.2	ENSG00000244474.5	ENSG00000262477.1
22835	ENSG00000214612.3	ENSG00000244471.6	ENSG00000115421.13
22836	ENSG00000214614.2	ENSG00000244470.2	ENSG00000171246.6
22837	ENSG00000214617.9	ENSG00000244468.1	ENSG00000198498.10
22838	ENSG00000214626.2	ENSG00000244464.1	ENSG00000234636.2
22839	ENSG00000214628.3	ENSG00000244462.8	ENSG00000120278.16
22840	ENSG00000214629.3	ENSG00000244461.1	ENSG00000164506.14

22841	ENSG00000214641.3	ENSG00000244459.2	ENSG00000243742.5
22842	ENSG00000214642.1	ENSG00000244457.2	ENSG00000235488.1
22843	ENSG00000214643.6	ENSG00000244456.2	ENSG00000010610.10
22844	ENSG00000214646.8	ENSG00000244451.1	ENSG00000213430.6
22845	ENSG00000214650.2	ENSG00000244441.1	ENSG00000272170.1
22846	ENSG00000214651.4	ENSG00000244437.1	ENSG00000196123.13
22847	ENSG00000214652.6	ENSG00000244436.2	ENSG00000235169.8
22848	ENSG00000214653.4	ENSG00000244432.2	ENSG00000197191.5
22849	ENSG00000214654.8	ENSG00000244429.1	ENSG00000200291.1
22850	ENSG00000214655.10	ENSG00000244427.1	ENSG00000148835.11
22851	ENSG00000214657.4	ENSG00000244425.3	ENSG00000268584.1
22852	ENSG00000214659.4	ENSG00000244422.3	ENSG00000112245.11
22853	ENSG00000214660.6	ENSG00000244414.6	ENSG00000166578.10
22854	ENSG00000214668.4	ENSG00000244413.1	ENSG00000279809.1
22855	ENSG00000214669.3	ENSG00000244411.3	ENSG00000115523.16
22856	ENSG00000214671.4	ENSG00000244405.8	ENSG00000005187.12
22857	ENSG00000214676.4	ENSG00000244404.3	ENSG00000245648.1
22858	ENSG00000214681.4	ENSG00000244402.3	ENSG00000162873.14
22859	ENSG00000214684.2	ENSG00000244400.2	ENSG00000225544.1
22860	ENSG00000214686.5	ENSG00000244399.3	ENSG00000285999.1
22861	ENSG00000214688.6	ENSG00000244398.1	ENSG00000109107.14
22862	ENSG00000214691.8	ENSG00000244395.6	ENSG00000178115.11
22863	ENSG00000214694.11	ENSG00000244392.3	ENSG00000151320.11
22864	ENSG00000214695.3	ENSG00000244391.3	ENSG00000198740.8
22865	ENSG00000214700.5	ENSG00000244390.2	ENSG00000262848.1
22866	ENSG00000214702.6	ENSG00000244389.3	ENSG00000250635.1
22867	ENSG00000214706.10	ENSG00000244384.3	ENSG00000234523.1
22868	ENSG00000214708.4	ENSG00000244383.2	ENSG00000232334.1
22869	ENSG00000214711.10	ENSG00000244381.1	ENSG00000180592.16
22870	ENSG00000214717.12	ENSG00000244380.1	ENSG00000159708.18
22871	ENSG00000214717.12_PAR_Y	ENSG00000244378.1	ENSG00000203801.8
22872	ENSG00000214719.12	ENSG00000244376.3	ENSG00000112164.6
22873	ENSG00000214720.4	ENSG00000244372.3	ENSG00000196502.11
22874	ENSG00000214725.9	ENSG00000244371.2	ENSG00000267311.1
22875	ENSG00000214727.2	ENSG00000244363.3	ENSG00000205500.8
22876	ENSG00000214732.2	ENSG00000244362.3	ENSG00000104915.15
22877	ENSG00000214736.7	ENSG00000244361.1	ENSG00000107372.13
22878	ENSG00000214743.4	ENSG00000244358.1	ENSG00000270006.2
22879	ENSG00000214745.2	ENSG00000244357.3	ENSG00000280614.1
22880	ENSG00000214748.2	ENSG00000244356.3	ENSG00000158163.15
22881	ENSG00000214753.3	ENSG00000244355.7	ENSG00000139517.9
22882	ENSG00000214754.3	ENSG00000244349.1	ENSG00000232187.1
22883	ENSG00000214756.8	ENSG00000244346.1	ENSG00000233937.6
22884	ENSG00000214759.3	ENSG00000244345.1	ENSG00000197381.16
22885	ENSG00000214760.3	ENSG00000244342.5	ENSG00000197403.4
22886	ENSG00000214761.3	ENSG00000244337.2	ENSG00000248724.6
22887	ENSG00000214765.8	ENSG00000244335.3	ENSG00000178150.10
22888	ENSG00000214770.3	ENSG00000244332.1	ENSG00000236810.5
22889	ENSG00000214772.2	ENSG00000244331.1	ENSG00000259070.6
22890	ENSG00000214773.1	ENSG00000244329.1	ENSG00000204624.8
22891	ENSG00000214776.12	ENSG00000244328.3	ENSG00000262227.1
22892	ENSG00000214782.7	ENSG00000244327.1	ENSG00000114021.12
22893	ENSG00000214783.9	ENSG00000244326.3	ENSG00000226942.2

22894	ENSG00000214784.4	ENSG00000244321.1	ENSG00000259790.1
22895	ENSG00000214787.10	ENSG00000244318.3	ENSG00000243704.3
22896	ENSG00000214788.3	ENSG00000244314.3	ENSG00000171202.7
22897	ENSG00000214794.4	ENSG00000244313.3	ENSG00000230280.2
22898	ENSG00000214796.8	ENSG00000244310.1	ENSG00000229692.3
22899	ENSG00000214797.3	ENSG00000244308.2	ENSG00000248774.1
22900	ENSG00000214803.3	ENSG00000244307.3	ENSG00000177340.5
22901	ENSG00000214807.2	ENSG00000244306.11	ENSG00000218459.1
22902	ENSG00000214810.4	ENSG00000244302.1	ENSG00000275070.1
22903	ENSG00000214812.3	ENSG00000244301.6	ENSG00000260708.1
22904	ENSG00000214814.7	ENSG00000244300.2	ENSG00000130402.12
22905	ENSG00000214815.2	ENSG00000244297.3	ENSG00000156273.16
22906	ENSG00000214819.1	ENSG00000244296.3	ENSG00000163655.16
22907	ENSG00000214820.3	ENSG00000244295.2	ENSG00000275158.1
22908	ENSG00000214821.4	ENSG00000244294.3	ENSG00000138078.15
22909	ENSG00000214822.8	ENSG00000244292.1	ENSG00000165959.12
22910	ENSG00000214823.3	ENSG00000244289.1	ENSG00000117984.14
22911	ENSG00000214825.2	ENSG00000244286.1	ENSG00000182397.14
22912	ENSG00000214826.5	ENSG00000244283.1	ENSG00000164414.18
22913	ENSG00000214827.10	ENSG00000244281.1	ENSG00000162772.17
22914	ENSG00000214832.5	ENSG00000244280.1	ENSG00000212125.2
22915	ENSG00000214835.3	ENSG00000244278.1	ENSG00000253930.1
22916	ENSG00000214837.8	ENSG00000244274.7	ENSG00000112290.13
22917	ENSG00000214842.5	ENSG00000244273.1	ENSG00000267412.1
22918	ENSG00000214843.3	ENSG00000244270.1	ENSG00000273102.1
22919	ENSG00000214846.4	ENSG00000244268.1	ENSG00000114854.7
22920	ENSG00000214851.4	ENSG00000244267.1	ENSG00000269110.1
22921	ENSG00000214853.5	ENSG00000244266.1	ENSG00000286112.1
22922	ENSG00000214855.9	ENSG00000244265.1	ENSG00000114988.11
22923	ENSG00000214856.11	ENSG00000244264.3	ENSG00000165732.13
22924	ENSG00000214857.4	ENSG00000244260.1	ENSG00000077800.13
22925	ENSG00000214860.5	ENSG00000244259.1	ENSG00000119457.8
22926	ENSG00000214866.8	ENSG00000244257.5	ENSG00000229056.2
22927	ENSG00000214867.3	ENSG00000244256.3	ENSG00000213075.7
22928	ENSG00000214869.5	ENSG00000244255.5	ENSG00000201766.1
22929	ENSG00000214870.8	ENSG00000244253.1	ENSG00000099622.14
22930	ENSG00000214872.8	ENSG00000244251.1	ENSG00000133101.10
22931	ENSG00000214875.2	ENSG00000244249.1	ENSG00000113758.13
22932	ENSG00000214878.2	ENSG00000244247.1	ENSG00000182993.4
22933	ENSG00000214880.3	ENSG00000244246.1	ENSG00000175220.12
22934	ENSG00000214881.4	ENSG00000244245.1	ENSG00000171163.15
22935	ENSG00000214883.4	ENSG00000244244.2	ENSG00000146833.15
22936	ENSG00000214886.4	ENSG00000244242.2	ENSG00000178201.4
22937	ENSG00000214888.3	ENSG00000244239.1	ENSG00000255112.2
22938	ENSG00000214889.3	ENSG00000244237.1	ENSG00000129521.13
22939	ENSG00000214890.3	ENSG00000244236.3	ENSG00000235703.5
22940	ENSG00000214891.9	ENSG00000244235.2	ENSG00000267827.5
22941	ENSG00000214892.4	ENSG00000244234.2	ENSG00000278999.1
22942	ENSG00000214894.6	ENSG00000244232.3	ENSG00000254909.1
22943	ENSG00000214896.4	ENSG00000244231.1	ENSG00000236559.1
22944	ENSG00000214897.4	ENSG00000244230.3	ENSG00000270467.1
22945	ENSG00000214900.9	ENSG00000244229.1	ENSG00000260288.3
22946	ENSG00000214903.4	ENSG00000244227.7	ENSG00000170011.14

22947	ENSG00000214904.5	ENSG00000244226.1	ENSG00000278196.3
22948	ENSG00000214908.3	ENSG00000244222.2	ENSG00000260464.1
22949	ENSG00000214914.3	ENSG00000244218.3	ENSG00000127957.18
22950	ENSG00000214915.3	ENSG00000244217.1	ENSG00000221916.4
22951	ENSG00000214917.3	ENSG00000244215.1	ENSG00000212533.1
22952	ENSG00000214919.3	ENSG00000244213.1	ENSG00000049246.14
22953	ENSG00000214922.9	ENSG00000244203.2	ENSG00000104731.14
22954	ENSG00000214925.3	ENSG00000244199.1	ENSG00000278558.4
22955	ENSG00000214930.2	ENSG00000244198.6	ENSG00000280212.1
22956	ENSG00000214940.8	ENSG00000244197.3	ENSG00000172260.15
22957	ENSG00000214941.8	ENSG00000244196.1	ENSG00000227159.8
22958	ENSG00000214942.5	ENSG00000244194.3	ENSG00000140464.19
22959	ENSG00000214943.4	ENSG00000244193.1	ENSG00000225526.4
22960	ENSG00000214944.9	ENSG00000244192.1	ENSG00000126746.17
22961	ENSG00000214946.14	ENSG00000244187.8	ENSG00000135093.13
22962	ENSG00000214954.8	ENSG00000244184.1	ENSG00000111321.11
22963	ENSG00000214955.5	ENSG00000244183.1	ENSG00000170852.11
22964	ENSG00000214960.10	ENSG00000244176.1	ENSG00000105656.13
22965	ENSG00000214961.2	ENSG00000244171.4	ENSG00000137204.14
22966	ENSG00000214967.5	ENSG00000244169.3	ENSG00000169715.14
22967	ENSG00000214970.8	ENSG00000244167.1	ENSG00000113272.14
22968	ENSG00000214973.3	ENSG00000244165.2	ENSG00000267102.1
22969	ENSG00000214975.4	ENSG00000244159.1	ENSG00000257195.1
22970	ENSG00000214976.2	ENSG00000244158.1	ENSG00000123684.13
22971	ENSG00000214978.7	ENSG00000244157.1	ENSG00000240652.1
22972	ENSG00000214980.4	ENSG00000244155.1	ENSG00000240445.3
22973	ENSG00000214988.4	ENSG00000244153.1	ENSG00000285693.1
22974	ENSG00000214992.6	ENSG00000244151.1	ENSG00000171813.14
22975	ENSG00000214998.2	ENSG00000244146.1	ENSG00000276256.1
22976	ENSG00000214999.3	ENSG00000244144.1	ENSG00000286025.1
22977	ENSG00000215000.4	ENSG00000244142.1	ENSG00000269937.1
22978	ENSG00000215002.2	ENSG00000244139.3	ENSG00000111674.9
22979	ENSG00000215003.4	ENSG00000244137.1	ENSG00000014257.16
22980	ENSG00000215004.3	ENSG00000244134.1	ENSG00000165113.13
22981	ENSG00000215005.2	ENSG00000244131.2	ENSG00000211658.2
22982	ENSG00000215006.4	ENSG00000244130.1	ENSG00000127561.15
22983	ENSG00000215007.3	ENSG00000244128.6	ENSG00000206262.9
22984	ENSG00000215009.5	ENSG00000244125.1	ENSG00000232485.2
22985	ENSG00000215012.9	ENSG00000244124.1	ENSG00000152240.13
22986	ENSG00000215014.5	ENSG00000244122.2	ENSG00000284719.1
22987	ENSG00000215016.2	ENSG00000244119.1	ENSG00000181381.13
22988	ENSG00000215018.9	ENSG00000244116.3	ENSG00000227145.1
22989	ENSG00000215021.8	ENSG00000244115.1	ENSG00000157540.21
22990	ENSG00000215022.7	ENSG00000244113.1	ENSG00000145088.9
22991	ENSG00000215023.2	ENSG00000244112.3	ENSG00000078403.17
22992	ENSG00000215029.9	ENSG00000244107.3	ENSG00000276861.1
22993	ENSG00000215030.5	ENSG00000244104.3	ENSG00000270638.1
22994	ENSG00000215032.2	ENSG00000244101.1	ENSG00000224698.1
22995	ENSG00000215034.4	ENSG00000244099.1	ENSG00000203878.11
22996	ENSG00000215035.2	ENSG00000244097.1	ENSG00000244540.2
22997	ENSG00000215037.2	ENSG00000244094.2	ENSG00000100077.15
22998	ENSG00000215039.6	ENSG00000244091.1	ENSG00000144063.3
22999	ENSG00000215041.10	ENSG00000244089.1	ENSG00000161013.17

23000	ENSG00000215043.2	ENSG00000244088.1	ENSG00000211706.2
23001	ENSG00000215045.8	ENSG00000244086.1	ENSG00000124772.12
23002	ENSG00000215049.3	ENSG00000244083.1	ENSG00000272572.1
23003	ENSG00000215054.3	ENSG00000244081.1	ENSG00000070785.16
23004	ENSG00000215057.4	ENSG00000244080.3	ENSG00000125730.16
23005	ENSG00000215063.3	ENSG00000244076.1	ENSG00000261147.1
23006	ENSG00000215065.3	ENSG00000244073.1	ENSG00000278668.1
23007	ENSG00000215067.9	ENSG00000244071.1	ENSG00000139438.5
23008	ENSG00000215068.7	ENSG00000244067.3	ENSG00000180658.4
23009	ENSG00000215070.4	ENSG00000244066.3	ENSG00000233716.1
23010	ENSG00000215085.4	ENSG00000244065.1	ENSG00000211820.1
23011	ENSG00000215086.2	ENSG00000244063.1	ENSG00000251503.8
23012	ENSG00000215088.3	ENSG00000244062.1	ENSG00000271984.1
23013	ENSG00000215089.3	ENSG00000244061.1	ENSG00000284052.1
23014	ENSG00000215093.3	ENSG00000244060.2	ENSG00000269763.1
23015	ENSG00000215094.3	ENSG00000244057.4	ENSG00000011052.21
23016	ENSG00000215096.3	ENSG00000244056.3	ENSG00000186073.13
23017	ENSG00000215097.3	ENSG00000244055.1	ENSG00000235300.4
23018	ENSG00000215102.2	ENSG00000244053.1	ENSG00000142330.20
23019	ENSG00000215105.4	ENSG00000244052.1	ENSG00000135486.17
23020	ENSG00000215110.7	ENSG00000244050.2	ENSG00000132464.11
23021	ENSG00000215112.6	ENSG00000244048.1	ENSG00000160410.15
23022	ENSG00000215113.6	ENSG00000244045.13	ENSG00000277739.1
23023	ENSG00000215114.9	ENSG00000244044.3	ENSG00000078902.16
23024	ENSG00000215115.6	ENSG00000244043.1	ENSG00000105492.16
23025	ENSG00000215117.5	ENSG00000244041.7	ENSG00000248794.1
23026	ENSG00000215120.2	ENSG00000244040.6	ENSG00000185924.7
23027	ENSG00000215124.2	ENSG00000244039.1	ENSG00000267452.2
23028	ENSG00000215126.10	ENSG00000244038.9	ENSG00000272583.1
23029	ENSG00000215127.6	ENSG00000244036.3	ENSG00000273216.1
23030	ENSG00000215131.10	ENSG00000244034.3	ENSG00000271699.5
23031	ENSG00000215146.5	ENSG00000244033.3	ENSG00000175820.3
23032	ENSG00000215148.8	ENSG00000244031.3	ENSG00000234345.2
23033	ENSG00000215149.3	ENSG00000244026.6	ENSG00000170909.13
23034	ENSG00000215151.4	ENSG00000244025.4	ENSG00000164105.4
23035	ENSG00000215154.6	ENSG00000244024.1	ENSG00000210082.2
23036	ENSG00000215156.5	ENSG00000244021.4	ENSG00000156345.17
23037	ENSG00000215158.9	ENSG00000244020.2	ENSG00000175899.14
23038	ENSG00000215159.3	ENSG00000244019.1	ENSG00000138769.11
23039	ENSG00000215160.3	ENSG00000244018.1	ENSG00000130413.15
23040	ENSG00000215162.1	ENSG00000244009.1	ENSG00000221970.2
23041	ENSG00000215165.3	ENSG00000244006.3	ENSG00000197128.11
23042	ENSG00000215168.2	ENSG00000244005.13	ENSG00000128886.12
23043	ENSG00000215174.2	ENSG00000244004.1	ENSG00000277883.1
23044	ENSG00000215177.3	ENSG00000244003.3	ENSG00000047644.18
23045	ENSG00000215179.5	ENSG00000244002.1	ENSG00000213671.3
23046	ENSG00000215182.8	ENSG00000244000.1	ENSG00000211798.3
23047	ENSG00000215183.5	ENSG00000243995.3	ENSG00000169084.13
23048	ENSG00000215184.2	ENSG00000243991.3	ENSG00000179066.8
23049	ENSG00000215186.6	ENSG00000243989.9	ENSG00000248592.7
23050	ENSG00000215187.11	ENSG00000243988.1	ENSG00000284428.1
23051	ENSG00000215190.9	ENSG00000243986.2	ENSG00000248188.2
23052	ENSG00000215193.12	ENSG00000243981.4	ENSG00000249141.1

23053	ENSG00000215196.4	ENSG00000243980.3	ENSG00000261542.1
23054	ENSG00000215197.4	ENSG00000243979.2	ENSG00000270720.1
23055	ENSG00000215198.3	ENSG00000243978.8	ENSG00000102218.6
23056	ENSG00000215199.3	ENSG00000243977.1	ENSG00000117133.11
23057	ENSG00000215203.2	ENSG00000243976.2	ENSG00000278991.1
23058	ENSG00000215206.5	ENSG00000243974.1	ENSG00000102001.12
23059	ENSG00000215208.3	ENSG00000243970.3	ENSG00000135185.12
23060	ENSG00000215210.3	ENSG00000243969.1	ENSG00000240823.3
23061	ENSG00000215217.7	ENSG00000243968.2	ENSG00000110696.10
23062	ENSG00000215218.4	ENSG00000243967.4	ENSG00000186654.21
23063	ENSG00000215221.2	ENSG00000243964.1	ENSG00000100979.15
23064	ENSG00000215223.3	ENSG00000243961.2	ENSG00000120075.5
23065	ENSG00000215227.4	ENSG00000243960.1	ENSG00000186666.6
23066	ENSG00000215231.7	ENSG00000243959.3	ENSG00000184523.4
23067	ENSG00000215236.3	ENSG00000243957.3	ENSG00000255495.1
23068	ENSG00000215237.6	ENSG00000243955.6	ENSG00000213089.4
23069	ENSG00000215241.3	ENSG00000243954.3	ENSG00000118260.15
23070	ENSG00000215244.2	ENSG00000243953.1	ENSG00000116663.11
23071	ENSG00000215246.5	ENSG00000243951.1	ENSG00000273784.4
23072	ENSG00000215251.4	ENSG00000243945.1	ENSG00000144554.10
23073	ENSG00000215252.11	ENSG00000243944.5	ENSG00000124205.17
23074	ENSG00000215256.3	ENSG00000243943.10	ENSG00000251402.3
23075	ENSG00000215262.8	ENSG00000243939.1	ENSG00000224875.2
23076	ENSG00000215263.2	ENSG00000243930.1	ENSG00000103355.13
23077	ENSG00000215264.4	ENSG00000243929.1	ENSG00000172799.5
23078	ENSG00000215267.8	ENSG00000243927.6	ENSG00000244509.4
23079	ENSG00000215268.3	ENSG00000243926.1	ENSG00000155657.26
23080	ENSG00000215269.5	ENSG00000243925.1	ENSG00000151693.11
23081	ENSG00000215270.3	ENSG00000243920.2	ENSG00000230734.1
23082	ENSG00000215271.7	ENSG00000243918.1	ENSG00000074276.10
23083	ENSG00000215274.5	ENSG00000243916.1	ENSG00000274828.1
23084	ENSG00000215277.8	ENSG00000243915.1	ENSG00000162461.8
23085	ENSG00000215278.4	ENSG00000243914.1	ENSG00000131469.14
23086	ENSG00000215283.3	ENSG00000243911.3	ENSG00000269646.1
23087	ENSG00000215284.2	ENSG00000243910.7	ENSG00000126215.14
23088	ENSG00000215286.3	ENSG00000243905.3	ENSG00000285628.1
23089	ENSG00000215288.4	ENSG00000243904.1	ENSG00000136897.8
23090	ENSG00000215296.10	ENSG00000243903.1	ENSG00000180535.3
23091	ENSG00000215297.3	ENSG00000243902.6	ENSG00000258357.1
23092	ENSG00000215301.10	ENSG00000243900.3	ENSG00000198015.13
23093	ENSG00000215302.8	ENSG00000243894.1	ENSG00000185485.14
23094	ENSG00000215304.3	ENSG00000243889.1	ENSG00000137965.11
23095	ENSG00000215305.10	ENSG00000243888.1	ENSG00000205238.9
23096	ENSG00000215310.2	ENSG00000243886.1	ENSG00000070388.11
23097	ENSG00000215311.3	ENSG00000243885.1	ENSG00000259661.1
23098	ENSG00000215313.3	ENSG00000243883.3	ENSG00000085831.15
23099	ENSG00000215317.2	ENSG00000243877.1	ENSG00000262903.1
23100	ENSG00000215319.2	ENSG00000243873.1	ENSG00000231905.2
23101	ENSG00000215325.4	ENSG00000243872.3	ENSG00000241007.1
23102	ENSG00000215326.3	ENSG00000243871.3	ENSG00000279145.2
23103	ENSG00000215333.3	ENSG00000243870.3	ENSG00000273455.1
23104	ENSG00000215339.3	ENSG00000243864.1	ENSG00000124006.15
23105	ENSG00000215343.7	ENSG00000243861.1	ENSG00000111328.7

23106	ENSG00000215347.4	ENSG00000243856.3	ENSG00000285204.1
23107	ENSG00000215349.2	ENSG00000243855.1	ENSG00000262943.7
23108	ENSG00000215351.3	ENSG00000243854.3	ENSG00000236554.1
23109	ENSG00000215353.2	ENSG00000243853.1	ENSG00000159625.14
23110	ENSG00000215354.9	ENSG00000243849.1	ENSG00000164309.15
23111	ENSG00000215356.4	ENSG00000243847.3	ENSG00000235191.1
23112	ENSG00000215357.2	ENSG00000243845.3	ENSG00000281398.4
23113	ENSG00000215367.10	ENSG00000243844.1	ENSG00000128833.13
23114	ENSG00000215368.5	ENSG00000243838.1	ENSG00000163472.19
23115	ENSG00000215369.3	ENSG00000243836.5	ENSG00000144741.17
23116	ENSG00000215371.3	ENSG00000243832.1	ENSG00000257225.1
23117	ENSG00000215372.6	ENSG00000243831.1	ENSG00000148200.17
23118	ENSG00000215373.4	ENSG00000243829.1	ENSG00000270149.5
23119	ENSG00000215374.5	ENSG00000243828.2	ENSG00000102078.16
23120	ENSG00000215375.6	ENSG00000243824.1	ENSG00000233121.1
23121	ENSG00000215378.3	ENSG00000243822.1	ENSG00000072062.13
23122	ENSG00000215380.2	ENSG00000243819.4	ENSG00000258471.2
23123	ENSG00000215381.3	ENSG00000243818.4	ENSG00000115539.14
23124	ENSG00000215386.12	ENSG00000243817.3	ENSG00000232260.2
23125	ENSG00000215388.3	ENSG00000243813.1	ENSG00000161653.10
23126	ENSG00000215397.4	ENSG00000243811.10	ENSG00000180616.9
23127	ENSG00000215398.11	ENSG00000243810.1	ENSG00000233319.1
23128	ENSG00000215399.2	ENSG00000243806.1	ENSG00000236156.2
23129	ENSG00000215403.1	ENSG00000243802.2	ENSG00000225259.4
23130	ENSG00000215409.4	ENSG00000243801.3	ENSG00000175906.5
23131	ENSG00000215414.4	ENSG00000243799.1	ENSG00000280011.1
23132	ENSG00000215417.12	ENSG00000243797.6	ENSG00000159200.18
23133	ENSG00000215418.2	ENSG00000243795.1	ENSG00000135968.20
23134	ENSG00000215421.9	ENSG00000243794.1	ENSG00000198585.11
23135	ENSG00000215424.9	ENSG00000243792.1	ENSG00000258581.2
23136	ENSG00000215440.12	ENSG00000243789.11	ENSG00000106034.18
23137	ENSG00000215441.3	ENSG00000243782.3	ENSG00000260941.1
23138	ENSG00000215444.2	ENSG00000243781.1	ENSG00000260691.6
23139	ENSG00000215448.3	ENSG00000243780.1	ENSG00000229132.2
23140	ENSG00000215450.2	ENSG00000243779.1	ENSG00000140479.17
23141	ENSG00000215452.5	ENSG00000243777.1	ENSG00000266290.1
23142	ENSG00000215454.6	ENSG00000243775.2	ENSG00000120659.15
23143	ENSG00000215455.4	ENSG00000243772.7	ENSG00000242457.1
23144	ENSG00000215456.6	ENSG00000243771.1	ENSG00000204406.13
23145	ENSG00000215457.5	ENSG00000243770.3	ENSG00000154930.15
23146	ENSG00000215458.8	ENSG00000243766.8	ENSG00000183773.15
23147	ENSG00000215464.4	ENSG00000243762.1	ENSG00000229191.1
23148	ENSG00000215467.2	ENSG00000243761.1	ENSG00000204677.11
23149	ENSG00000215472.10	ENSG00000243759.1	ENSG00000269877.3
23150	ENSG00000215474.7	ENSG00000243758.1	ENSG00000248455.5
23151	ENSG00000215475.5	ENSG00000243753.5	ENSG00000279103.1
23152	ENSG00000215477.2	ENSG00000243749.1	ENSG00000086696.11
23153	ENSG00000215478.8	ENSG00000243746.1	ENSG00000285518.1
23154	ENSG00000215480.4	ENSG00000243744.2	ENSG00000277578.1
23155	ENSG00000215481.9	ENSG00000243742.5	ENSG00000196670.13
23156	ENSG00000215482.3	ENSG00000243738.3	ENSG00000198932.13
23157	ENSG00000215483.10	ENSG00000243733.1	ENSG00000257243.1
23158	ENSG00000215486.5	ENSG00000243730.2	ENSG00000258643.5

23159	ENSG00000215492.6	ENSG00000243729.4	ENSG00000227028.6
23160	ENSG00000215493.3	ENSG00000243725.7	ENSG00000106565.18
23161	ENSG00000215498.9	ENSG00000243723.3	ENSG00000266127.1
23162	ENSG00000215506.5	ENSG00000243721.1	ENSG00000081019.13
23163	ENSG00000215507.10	ENSG00000243720.1	ENSG00000279692.1
23164	ENSG00000215512.9	ENSG00000243716.10	ENSG00000095066.11
23165	ENSG00000215515.2	ENSG00000243715.1	ENSG00000168772.11
23166	ENSG00000215520.3	ENSG00000243711.1	ENSG00000278677.1
23167	ENSG00000215529.12	ENSG00000243710.7	ENSG00000214812.3
23168	ENSG00000215533.8	ENSG00000243709.1	ENSG00000146453.13
23169	ENSG00000215537.3	ENSG00000243705.1	ENSG00000254162.1
23170	ENSG00000215540.10	ENSG00000243704.3	ENSG00000103266.11
23171	ENSG00000215544.6	ENSG00000243702.3	ENSG00000248682.1
23172	ENSG00000215545.1	ENSG00000243701.6	ENSG00000156564.8
23173	ENSG00000215546.2	ENSG00000243700.3	ENSG00000036530.8
23174	ENSG00000215547.1	ENSG00000243697.1	ENSG00000145365.11
23175	ENSG00000215548.2	ENSG00000243696.5	ENSG00000198488.10
23176	ENSG00000215553.3	ENSG00000243695.1	ENSG00000169629.11
23177	ENSG00000215559.8	ENSG00000243694.2	ENSG00000171497.5
23178	ENSG00000215560.2	ENSG00000243687.1	ENSG00000283463.1
23179	ENSG00000215562.2	ENSG00000243686.2	ENSG00000188611.14
23180	ENSG00000215567.5	ENSG00000243680.1	ENSG00000251013.1
23181	ENSG00000215568.9	ENSG00000243679.1	ENSG00000228757.1
23182	ENSG00000215571.5	ENSG00000243678.11	ENSG00000173369.16
23183	ENSG00000215572.2	ENSG00000243675.1	ENSG00000250299.6
23184	ENSG00000215580.11	ENSG00000243674.1	ENSG00000272791.1
23185	ENSG00000215583.3	ENSG00000243672.1	ENSG00000136960.12
23186	ENSG00000215586.2	ENSG00000243671.3	ENSG00000267422.1
23187	ENSG00000215589.3	ENSG00000243669.2	ENSG00000179564.4
23188	ENSG00000215595.1	ENSG00000243667.7	ENSG00000272525.1
23189	ENSG00000215601.3	ENSG00000243664.1	ENSG00000130309.11
23190	ENSG00000215603.4	ENSG00000243663.1	ENSG00000138468.16
23191	ENSG00000215604.3	ENSG00000243661.1	ENSG00000142233.11
23192	ENSG00000215606.4	ENSG00000243660.10	ENSG00000269463.1
23193	ENSG00000215612.8	ENSG00000243659.1	ENSG00000267322.2
23194	ENSG00000215630.6	ENSG00000243658.1	ENSG00000280194.1
23195	ENSG00000215644.10	ENSG00000243655.2	ENSG00000226091.7
23196	ENSG00000215692.2	ENSG00000243650.3	ENSG00000100429.18
23197	ENSG00000215695.1	ENSG00000243649.8	ENSG00000255874.2
23198	ENSG00000215704.9	ENSG00000243648.1	ENSG00000238121.5
23199	ENSG00000215712.10	ENSG00000243646.10	ENSG00000231341.1
23200	ENSG00000215717.6	ENSG00000243643.1	ENSG00000254618.1
23201	ENSG00000215720.4	ENSG00000243642.3	ENSG00000182154.8
23202	ENSG00000215734.3	ENSG00000243641.3	ENSG00000122477.12
23203	ENSG00000215760.2	ENSG00000243636.1	ENSG00000107651.13
23204	ENSG00000215765.3	ENSG00000243635.1	ENSG00000253819.1
23205	ENSG00000215769.8	ENSG00000243633.3	ENSG00000167470.12
23206	ENSG00000215771.2	ENSG00000243629.1	ENSG00000125843.11
23207	ENSG00000215784.6	ENSG00000243627.4	ENSG00000236008.2
23208	ENSG00000215785.2	ENSG00000243621.1	ENSG00000198814.12
23209	ENSG00000215788.10	ENSG00000243620.1	ENSG00000267469.1
23210	ENSG00000215790.7	ENSG00000243613.1	ENSG00000222375.1
23211	ENSG00000215795.2	ENSG00000243609.1	ENSG00000160471.13

23212	ENSG00000215796.3	ENSG00000243607.3	ENSG00000234369.1
23213	ENSG00000215800.2	ENSG00000243601.1	ENSG00000225399.4
23214	ENSG00000215802.3	ENSG00000243596.1	ENSG00000122432.17
23215	ENSG00000215805.3	ENSG00000243593.1	ENSG00000108379.10
23216	ENSG00000215807.4	ENSG00000243592.1	ENSG00000143512.13
23217	ENSG00000215808.3	ENSG00000243591.3	ENSG00000165898.13
23218	ENSG00000215811.6	ENSG00000243584.1	ENSG00000007520.4
23219	ENSG00000215812.5	ENSG00000243581.1	ENSG00000213442.5
23220	ENSG00000215817.7	ENSG00000243574.1	ENSG00000286053.1
23221	ENSG00000215819.3	ENSG00000243572.1	ENSG00000279653.1
23222	ENSG00000215833.3	ENSG00000243568.2	ENSG00000237351.1
23223	ENSG00000215834.10	ENSG00000243566.6	ENSG00000064199.6
23224	ENSG00000215835.2	ENSG00000243562.3	ENSG00000151338.18
23225	ENSG00000215837.7	ENSG00000243560.3	ENSG00000177042.14
23226	ENSG00000215838.4	ENSG00000243554.1	ENSG00000038274.17
23227	ENSG00000215840.3	ENSG00000243550.2	ENSG00000227053.1
23228	ENSG00000215841.3	ENSG00000243549.3	ENSG00000188760.10
23229	ENSG00000215845.11	ENSG00000243548.3	ENSG00000108821.13
23230	ENSG00000215846.6	ENSG00000243547.1	ENSG00000142765.18
23231	ENSG00000215853.3	ENSG00000243546.3	ENSG00000140905.10
23232	ENSG00000215859.9	ENSG00000243544.3	ENSG00000113761.12
23233	ENSG00000215861.5	ENSG00000243543.8	ENSG00000240890.2
23234	ENSG00000215864.6	ENSG00000243541.3	ENSG00000249691.1
23235	ENSG00000215866.7	ENSG00000243539.3	ENSG00000132010.16
23236	ENSG00000215867.4	ENSG00000243538.1	ENSG00000279897.2
23237	ENSG00000215869.4	ENSG00000243537.1	ENSG00000166167.18
23238	ENSG00000215871.2	ENSG00000243532.3	ENSG00000228013.1
23239	ENSG00000215873.2	ENSG00000243531.1	ENSG00000130227.17
23240	ENSG00000215874.3	ENSG00000243521.2	ENSG00000283294.1
23241	ENSG00000215875.4	ENSG00000243519.1	ENSG00000267541.1
23242	ENSG00000215878.3	ENSG00000243518.1	ENSG00000197794.2
23243	ENSG00000215881.3	ENSG00000243517.1	ENSG00000204659.5
23244	ENSG00000215883.10	ENSG00000243516.1	ENSG00000203737.3
23245	ENSG00000215887.5	ENSG00000243514.2	ENSG00000226763.4
23246	ENSG00000215893.3	ENSG00000243510.3	ENSG00000237702.2
23247	ENSG00000215895.4	ENSG00000243509.6	ENSG00000060971.18
23248	ENSG00000215899.3	ENSG00000243508.1	ENSG00000224680.4
23249	ENSG00000215905.4	ENSG00000243507.1	ENSG00000217702.3
23250	ENSG00000215906.8	ENSG00000243505.3	ENSG00000243746.1
23251	ENSG00000215908.10	ENSG00000243504.1	ENSG00000271820.1
23252	ENSG00000215909.3	ENSG00000243503.2	ENSG00000184661.14
23253	ENSG00000215910.7	ENSG00000243501.5	ENSG00000160207.9
23254	ENSG00000215912.12	ENSG00000243499.1	ENSG00000162426.14
23255	ENSG00000215914.4	ENSG00000243498.2	ENSG00000177034.16
23256	ENSG00000215915.9	ENSG00000243495.1	ENSG00000242371.1
23257	ENSG00000215930.1	ENSG00000243494.1	ENSG00000236489.1
23258	ENSG00000215938.3	ENSG00000243491.1	ENSG00000257283.1
23259	ENSG00000215939.3	ENSG00000243489.4	ENSG00000235253.1
23260	ENSG00000215943.1	ENSG00000243488.3	ENSG00000179240.9
23261	ENSG00000215952.1	ENSG00000243486.1	ENSG00000280375.1
23262	ENSG00000215957.1	ENSG00000243485.5	ENSG00000211727.3
23263	ENSG00000215961.1	ENSG00000243483.1	ENSG00000262482.1
23264	ENSG00000215966.1	ENSG00000243480.7	ENSG00000174600.14

23265	ENSG00000215973.1	ENSG00000243479.3	ENSG00000274265.4
23266	ENSG00000215991.1	ENSG00000243478.9	ENSG00000100650.15
23267	ENSG00000216001.3	ENSG00000243477.5	ENSG00000263961.8
23268	ENSG00000216005.1	ENSG00000243469.1	ENSG00000204381.11
23269	ENSG00000216009.3	ENSG00000243468.5	ENSG00000197635.10
23270	ENSG00000216031.1	ENSG00000243466.1	ENSG00000227516.1
23271	ENSG00000216035.1	ENSG00000243455.3	ENSG00000236519.1
23272	ENSG00000216056.1	ENSG00000243449.6	ENSG00000259001.3
23273	ENSG00000216058.1	ENSG00000243446.3	ENSG00000198131.14
23274	ENSG00000216060.1	ENSG00000243445.1	ENSG00000088899.15
23275	ENSG00000216064.1	ENSG00000243444.8	ENSG00000146904.9
23276	ENSG00000216069.1	ENSG00000243439.3	ENSG00000231063.1
23277	ENSG00000216075.1	ENSG00000243438.1	ENSG00000226306.6
23278	ENSG00000216077.3	ENSG00000243437.3	ENSG00000234004.4
23279	ENSG00000216098.1	ENSG00000243433.1	ENSG00000021574.12
23280	ENSG00000216099.3	ENSG00000243431.1	ENSG00000213071.11
23281	ENSG00000216101.3	ENSG00000243429.1	ENSG00000152207.7
23282	ENSG00000216135.3	ENSG00000243426.3	ENSG00000171208.9
23283	ENSG00000216141.3	ENSG00000243424.2	ENSG00000205339.10
23284	ENSG00000216171.1	ENSG00000243423.1	ENSG00000237757.2
23285	ENSG00000216179.1	ENSG00000243422.2	ENSG00000075568.17
23286	ENSG00000216191.1	ENSG00000243420.3	ENSG00000266402.3
23287	ENSG00000216192.1	ENSG00000243417.1	ENSG00000235917.1
23288	ENSG00000216195.3	ENSG00000243415.2	ENSG00000137815.14
23289	ENSG00000216265.1	ENSG00000243414.5	ENSG00000134955.11
23290	ENSG00000216285.5	ENSG00000243410.1	ENSG00000159348.13
23291	ENSG00000216306.3	ENSG00000243406.6	ENSG00000265666.1
23292	ENSG00000216307.2	ENSG00000243404.2	ENSG00000265491.5
23293	ENSG00000216316.1	ENSG00000243403.1	ENSG00000186152.6
23294	ENSG00000216324.1	ENSG00000243402.1	ENSG00000248858.7
23295	ENSG00000216331.2	ENSG00000243398.3	ENSG00000251575.2
23296	ENSG00000216347.1	ENSG00000243396.1	ENSG00000273111.6
23297	ENSG00000216352.1	ENSG00000243389.1	ENSG00000249667.1
23298	ENSG00000216359.1	ENSG00000243388.1	ENSG00000172840.6
23299	ENSG00000216360.1	ENSG00000243385.2	ENSG00000083544.14
23300	ENSG00000216364.1	ENSG00000243384.1	ENSG00000134812.8
23301	ENSG00000216365.2	ENSG00000243383.3	ENSG00000151690.15
23302	ENSG00000216368.2	ENSG00000243378.1	ENSG00000265315.1
23303	ENSG00000216378.1	ENSG00000243374.3	ENSG00000232676.1
23304	ENSG00000216412.4	ENSG00000243373.3	ENSG00000236525.1
23305	ENSG00000216425.4	ENSG00000243370.3	ENSG00000101986.12
23306	ENSG00000216436.2	ENSG00000243368.2	ENSG00000258413.1
23307	ENSG00000216439.1	ENSG00000243366.3	ENSG00000214093.3
23308	ENSG00000216444.1	ENSG00000243365.3	ENSG00000286094.1
23309	ENSG00000216471.3	ENSG00000243364.8	ENSG00000117360.13
23310	ENSG00000216475.1	ENSG00000243359.3	ENSG00000215319.2
23311	ENSG00000216480.2	ENSG00000243355.1	ENSG00000171657.6
23312	ENSG00000216490.4	ENSG00000243353.1	ENSG00000223361.5
23313	ENSG00000216516.1	ENSG00000243352.3	ENSG00000142185.16
23314	ENSG00000216518.1	ENSG00000243349.2	ENSG00000250980.1
23315	ENSG00000216519.2	ENSG00000243347.1	ENSG00000236478.2
23316	ENSG00000216523.1	ENSG00000243346.3	ENSG00000278616.1
23317	ENSG00000216548.2	ENSG00000243345.1	ENSG00000213326.4

23318	ENSG00000216560.4	ENSG00000243339.3	ENSG00000262180.1
23319	ENSG00000216588.9	ENSG00000243338.3	ENSG00000101460.13
23320	ENSG00000216613.2	ENSG00000243335.9	ENSG00000134317.18
23321	ENSG00000216616.1	ENSG00000243333.3	ENSG00000257285.5
23322	ENSG00000216621.8	ENSG00000243328.1	ENSG00000160993.4
23323	ENSG00000216624.3	ENSG00000243323.7	ENSG00000131089.15
23324	ENSG00000216629.1	ENSG00000243321.3	ENSG00000258938.1
23325	ENSG00000216636.1	ENSG00000243319.7	ENSG00000104218.14
23326	ENSG00000216639.1	ENSG00000243317.8	ENSG00000260059.1
23327	ENSG00000216642.1	ENSG00000243316.7	ENSG00000229151.1
23328	ENSG00000216649.3	ENSG00000243314.1	ENSG00000266117.1
23329	ENSG00000216657.1	ENSG00000243313.2	ENSG00000240317.3
23330	ENSG00000216663.3	ENSG00000243312.3	ENSG00000055957.11
23331	ENSG00000216671.6	ENSG00000243307.2	ENSG00000163872.16
23332	ENSG00000216676.2	ENSG00000243305.1	ENSG00000276384.1
23333	ENSG00000216687.2	ENSG00000243304.3	ENSG00000142945.13
23334	ENSG00000216708.1	ENSG00000243303.1	ENSG00000272848.2
23335	ENSG00000216710.1	ENSG00000243302.3	ENSG00000176261.15
23336	ENSG00000216713.1	ENSG00000243297.2	ENSG00000263786.1
23337	ENSG00000216718.7	ENSG00000243296.1	ENSG00000016391.11
23338	ENSG00000216721.3	ENSG00000243295.1	ENSG00000243284.1
23339	ENSG00000216723.1	ENSG00000243290.3	ENSG00000279476.1
23340	ENSG00000216740.2	ENSG00000243289.1	ENSG00000270953.1
23341	ENSG00000216753.3	ENSG00000243287.2	ENSG00000177025.3
23342	ENSG00000216754.2	ENSG00000243284.1	ENSG00000137869.14
23343	ENSG00000216762.1	ENSG00000243280.2	ENSG00000175463.11
23344	ENSG00000216775.3	ENSG00000243279.4	ENSG00000267776.1
23345	ENSG00000216777.1	ENSG00000243276.5	ENSG00000108561.8
23346	ENSG00000216781.2	ENSG00000243274.3	ENSG00000258430.1
23347	ENSG00000216802.1	ENSG00000243273.1	ENSG00000159228.13
23348	ENSG00000216809.1	ENSG00000243267.3	ENSG00000261487.1
23349	ENSG00000216811.2	ENSG00000243265.1	ENSG00000274191.1
23350	ENSG00000216813.1	ENSG00000243264.2	ENSG00000283041.1
23351	ENSG00000216817.1	ENSG00000243260.3	ENSG00000253174.2
23352	ENSG00000216819.1	ENSG00000243257.1	ENSG00000285382.1
23353	ENSG00000216824.2	ENSG00000243256.1	ENSG00000170345.10
23354	ENSG00000216829.6	ENSG00000243254.3	ENSG00000196724.12
23355	ENSG00000216835.3	ENSG00000243244.6	ENSG00000065243.20
23356	ENSG00000216844.1	ENSG00000243243.5	ENSG00000228143.2
23357	ENSG00000216853.1	ENSG00000243238.1	ENSG00000246422.2
23358	ENSG00000216854.2	ENSG00000243236.6	ENSG00000151474.23
23359	ENSG00000216859.1	ENSG00000243234.1	ENSG00000236493.2
23360	ENSG00000216863.9	ENSG00000243232.5	ENSG00000285888.1
23361	ENSG00000216866.5	ENSG00000243230.1	ENSG00000165568.18
23362	ENSG00000216867.2	ENSG00000243227.3	ENSG00000279069.1
23363	ENSG00000216895.9	ENSG00000243225.2	ENSG00000225338.1
23364	ENSG00000216901.1	ENSG00000243224.1	ENSG00000092421.16
23365	ENSG00000216902.3	ENSG00000243220.1	ENSG00000201364.1
23366	ENSG00000216904.2	ENSG00000243207.6	ENSG00000284874.1
23367	ENSG00000216906.2	ENSG00000243199.1	ENSG00000168899.5
23368	ENSG00000216913.2	ENSG00000243197.7	ENSG00000228615.1
23369	ENSG00000216915.2	ENSG00000243195.1	ENSG00000250073.2
23370	ENSG00000216917.2	ENSG00000243188.1	ENSG00000157404.15

23371	ENSG00000216921.8	ENSG00000243187.1	ENSG00000154122.14
23372	ENSG00000216937.13	ENSG00000243181.2	ENSG00000254389.3
23373	ENSG00000216938.3	ENSG00000243179.1	ENSG00000256188.3
23374	ENSG00000216966.1	ENSG00000243176.5	ENSG00000235082.2
23375	ENSG00000216977.1	ENSG00000243175.1	ENSG00000260988.1
23376	ENSG00000216990.2	ENSG00000243173.3	ENSG00000228252.9
23377	ENSG00000216998.1	ENSG00000243171.1	ENSG00000002822.15
23378	ENSG00000217004.1	ENSG00000243167.1	ENSG00000248278.1
23379	ENSG00000217026.3	ENSG00000243165.1	ENSG00000007384.15
23380	ENSG00000217027.1	ENSG00000243164.1	ENSG00000116741.8
23381	ENSG00000217030.1	ENSG00000243160.1	ENSG00000206177.7
23382	ENSG00000217041.1	ENSG00000243156.8	ENSG00000231475.3
23383	ENSG00000217044.1	ENSG00000243155.1	ENSG00000102034.16
23384	ENSG00000217060.1	ENSG00000243154.1	ENSG00000133318.13
23385	ENSG00000217067.2	ENSG00000243150.5	ENSG00000067221.14
23386	ENSG00000217078.1	ENSG00000243149.1	ENSG00000266644.1
23387	ENSG00000217083.1	ENSG00000243147.8	ENSG00000158195.11
23388	ENSG00000217085.2	ENSG00000243144.6	ENSG00000211962.2
23389	ENSG00000217089.1	ENSG00000243141.2	ENSG00000137100.16
23390	ENSG00000217094.2	ENSG00000243137.8	ENSG00000225793.2
23391	ENSG00000217120.1	ENSG00000243136.3	ENSG00000285942.1
23392	ENSG00000217128.12	ENSG00000243135.6	ENSG00000231989.4
23393	ENSG00000217130.1	ENSG00000243130.8	ENSG00000231609.6
23394	ENSG00000217135.2	ENSG00000243129.2	ENSG00000261868.1
23395	ENSG00000217139.2	ENSG00000243125.3	ENSG00000034239.11
23396	ENSG00000217159.2	ENSG00000243124.3	ENSG00000279529.1
23397	ENSG00000217160.2	ENSG00000243122.1	ENSG00000004534.15
23398	ENSG00000217165.1	ENSG00000243116.1	ENSG00000135205.15
23399	ENSG00000217169.2	ENSG00000243115.3	ENSG00000279565.1
23400	ENSG00000217178.1	ENSG00000243107.1	ENSG00000233411.1
23401	ENSG00000217179.1	ENSG00000243103.3	ENSG00000276334.1
23402	ENSG00000217181.1	ENSG00000243101.1	ENSG00000258317.1
23403	ENSG00000217195.1	ENSG00000243099.1	ENSG00000163817.16
23404	ENSG00000217227.1	ENSG00000243095.1	ENSG00000242094.1
23405	ENSG00000217228.1	ENSG00000243094.1	ENSG00000204072.3
23406	ENSG00000217231.2	ENSG00000243089.1	ENSG00000249695.6
23407	ENSG00000217236.2	ENSG00000243085.1	ENSG00000133247.14
23408	ENSG00000217239.3	ENSG00000243083.6	ENSG00000236375.3
23409	ENSG00000217241.1	ENSG00000243081.2	ENSG00000261824.6
23410	ENSG00000217261.4	ENSG00000243075.3	ENSG00000267757.4
23411	ENSG00000217268.1	ENSG00000243073.3	ENSG00000227782.2
23412	ENSG00000217272.1	ENSG00000243072.1	ENSG00000266965.1
23413	ENSG00000217275.2	ENSG00000243071.1	ENSG00000177731.16
23414	ENSG00000217289.3	ENSG00000243069.7	ENSG00000092098.17
23415	ENSG00000217314.2	ENSG00000243066.3	ENSG00000119946.11
23416	ENSG00000217315.1	ENSG00000243064.8	ENSG00000132000.13
23417	ENSG00000217325.2	ENSG00000243063.1	ENSG00000111199.10
23418	ENSG00000217327.3	ENSG00000243062.5	ENSG00000217624.2
23419	ENSG00000217330.1	ENSG00000243059.3	ENSG00000125386.15
23420	ENSG00000217331.1	ENSG00000243058.1	ENSG00000107014.9
23421	ENSG00000217334.1	ENSG00000243056.2	ENSG00000222020.2
23422	ENSG00000217372.2	ENSG00000243055.1	ENSG00000154269.15
23423	ENSG00000217377.1	ENSG00000243053.2	ENSG00000085433.15

23424	ENSG00000217379.2	ENSG00000243051.3	ENSG00000265451.1
23425	ENSG00000217385.1	ENSG00000243050.1	ENSG00000144228.8
23426	ENSG00000217404.2	ENSG00000243049.3	ENSG00000159111.13
23427	ENSG00000217408.2	ENSG00000243048.3	ENSG00000262877.4
23428	ENSG00000217414.2	ENSG00000243044.1	ENSG00000173928.2
23429	ENSG00000217416.4	ENSG00000243040.6	ENSG00000249624.9
23430	ENSG00000217442.4	ENSG00000243038.1	ENSG00000272356.1
23431	ENSG00000217447.1	ENSG00000243035.3	ENSG00000162441.12
23432	ENSG00000217455.8	ENSG00000243033.2	ENSG00000196368.5
23433	ENSG00000217477.2	ENSG00000243029.3	ENSG00000163435.16
23434	ENSG00000217482.2	ENSG00000243027.3	ENSG00000205584.6
23435	ENSG00000217483.1	ENSG00000243025.1	ENSG00000105613.10
23436	ENSG00000217488.2	ENSG00000243024.6	ENSG00000268119.5
23437	ENSG00000217495.2	ENSG00000243023.2	ENSG00000246334.2
23438	ENSG00000217512.1	ENSG00000243022.1	ENSG00000179151.13
23439	ENSG00000217514.1	ENSG00000243020.1	ENSG00000180336.18
23440	ENSG00000217527.1	ENSG00000243018.1	ENSG00000168916.15
23441	ENSG00000217539.2	ENSG00000243016.1	ENSG00000224858.5
23442	ENSG00000217555.12	ENSG00000243015.2	ENSG00000104774.13
23443	ENSG00000217557.1	ENSG00000243014.1	ENSG00000123815.12
23444	ENSG00000217566.2	ENSG00000243011.3	ENSG00000260920.2
23445	ENSG00000217576.7	ENSG00000243008.2	ENSG00000081913.14
23446	ENSG00000217585.1	ENSG00000243007.1	ENSG00000277586.2
23447	ENSG00000217612.2	ENSG00000243005.3	ENSG00000114316.12
23448	ENSG00000217624.2	ENSG00000242999.3	ENSG00000116991.10
23449	ENSG00000217631.1	ENSG00000242998.3	ENSG00000170365.10
23450	ENSG00000217643.1	ENSG00000242995.1	ENSG00000160087.20
23451	ENSG00000217644.5	ENSG00000242993.1	ENSG00000257852.1
23452	ENSG00000217646.1	ENSG00000242992.3	ENSG00000175170.15
23453	ENSG00000217648.1	ENSG00000242991.2	ENSG00000137171.15
23454	ENSG00000217653.1	ENSG00000242990.2	ENSG00000285338.1
23455	ENSG00000217680.1	ENSG00000242989.3	ENSG00000261526.2
23456	ENSG00000217684.2	ENSG00000242986.2	ENSG00000141867.17
23457	ENSG00000217686.2	ENSG00000242985.3	ENSG00000100027.15
23458	ENSG00000217702.3	ENSG00000242983.1	ENSG00000127220.6
23459	ENSG00000217707.2	ENSG00000242979.1	ENSG00000185621.11
23460	ENSG00000217716.3	ENSG00000242978.1	ENSG00000238168.5
23461	ENSG00000217718.1	ENSG00000242976.3	ENSG00000275549.1
23462	ENSG00000217733.2	ENSG00000242973.6	ENSG00000279360.1
23463	ENSG00000217746.1	ENSG00000242971.3	ENSG00000118292.9
23464	ENSG00000217767.2	ENSG00000242970.2	ENSG00000211782.2
23465	ENSG00000217769.4	ENSG00000242968.1	ENSG00000062370.16
23466	ENSG00000217770.1	ENSG00000242963.1	ENSG00000173402.11
23467	ENSG00000217776.1	ENSG00000242960.1	ENSG00000129315.11
23468	ENSG00000217783.2	ENSG00000242958.1	ENSG00000228446.2
23469	ENSG00000217786.1	ENSG00000242953.1	ENSG00000262621.5
23470	ENSG00000217791.4	ENSG00000242952.1	ENSG00000166920.12
23471	ENSG00000217801.10	ENSG00000242951.1	ENSG00000275645.1
23472	ENSG00000217805.2	ENSG00000242950.7	ENSG00000172232.10
23473	ENSG00000217809.1	ENSG00000242948.1	ENSG00000265802.2
23474	ENSG00000217811.1	ENSG00000242945.2	ENSG00000180481.11
23475	ENSG00000217824.1	ENSG00000242943.1	ENSG00000139168.8
23476	ENSG00000217825.2	ENSG00000242941.1	ENSG00000197956.10

23477	ENSG00000217835.3	ENSG00000242936.1	ENSG00000067900.8
23478	ENSG00000217862.2	ENSG00000242931.1	ENSG00000169902.15
23479	ENSG00000217878.2	ENSG00000242928.3	ENSG00000138032.21
23480	ENSG00000217889.3	ENSG00000242925.1	ENSG00000259004.1
23481	ENSG00000217896.2	ENSG00000242922.2	ENSG00000277491.1
23482	ENSG00000217897.2	ENSG00000242915.1	ENSG00000123342.16
23483	ENSG00000217929.4	ENSG00000242912.3	ENSG00000267990.1
23484	ENSG00000217930.8	ENSG00000242911.1	ENSG00000213585.11
23485	ENSG00000217950.4	ENSG00000242908.6	ENSG00000245468.3
23486	ENSG00000218014.1	ENSG00000242902.1	ENSG00000225936.1
23487	ENSG00000218016.2	ENSG00000242899.1	ENSG00000100422.14
23488	ENSG00000218018.2	ENSG00000242894.3	ENSG00000227500.10
23489	ENSG00000218020.3	ENSG00000242893.3	ENSG00000251920.1
23490	ENSG00000218027.2	ENSG00000242889.3	ENSG00000125388.20
23491	ENSG00000218029.3	ENSG00000242888.1	ENSG00000272438.1
23492	ENSG00000218048.2	ENSG00000242887.1	ENSG00000170390.16
23493	ENSG00000218049.1	ENSG00000242882.1	ENSG00000143322.20
23494	ENSG00000218052.5	ENSG00000242880.1	ENSG00000248641.1
23495	ENSG00000218069.2	ENSG00000242876.3	ENSG00000174749.6
23496	ENSG00000218073.1	ENSG00000242875.6	ENSG00000150873.11
23497	ENSG00000218089.1	ENSG00000242866.10	ENSG00000204472.13
23498	ENSG00000218107.1	ENSG00000242865.3	ENSG00000081665.14
23499	ENSG00000218109.5	ENSG00000242863.3	ENSG00000158966.15
23500	ENSG00000218125.1	ENSG00000242861.1	ENSG00000213816.3
23501	ENSG00000218143.2	ENSG00000242860.3	ENSG00000130590.14
23502	ENSG00000218153.2	ENSG00000242858.1	ENSG00000250573.1
23503	ENSG00000218173.1	ENSG00000242856.3	ENSG00000205871.5
23504	ENSG00000218175.2	ENSG00000242855.3	ENSG00000183691.4
23505	ENSG00000218180.2	ENSG00000242854.1	ENSG00000102678.6
23506	ENSG00000218186.2	ENSG00000242853.3	ENSG00000138669.9
23507	ENSG00000218187.2	ENSG00000242852.7	ENSG00000110046.13
23508	ENSG00000218189.4	ENSG00000242850.3	ENSG00000240535.8
23509	ENSG00000218194.1	ENSG00000242849.2	ENSG00000205740.2
23510	ENSG00000218198.3	ENSG00000242841.1	ENSG00000103353.16
23511	ENSG00000218208.1	ENSG00000242837.1	ENSG00000246100.3
23512	ENSG00000218213.1	ENSG00000242836.1	ENSG00000147677.11
23513	ENSG00000218226.1	ENSG00000242834.1	ENSG00000139192.12
23514	ENSG00000218227.3	ENSG00000242829.1	ENSG00000248932.5
23515	ENSG00000218233.1	ENSG00000242828.1	ENSG00000092067.5
23516	ENSG00000218261.1	ENSG00000242818.3	ENSG00000261240.1
23517	ENSG00000218265.1	ENSG00000242816.1	ENSG00000150760.12
23518	ENSG00000218274.2	ENSG00000242814.2	ENSG00000172936.14
23519	ENSG00000218281.1	ENSG00000242811.3	ENSG00000162819.12
23520	ENSG00000218283.2	ENSG00000242810.1	ENSG00000175711.8
23521	ENSG00000218300.1	ENSG00000242808.8	ENSG00000116032.5
23522	ENSG00000218305.4	ENSG00000242807.1	ENSG00000149577.15
23523	ENSG00000218313.1	ENSG00000242802.8	ENSG00000122778.9
23524	ENSG00000218336.9	ENSG00000242798.1	ENSG00000157483.8
23525	ENSG00000218337.1	ENSG00000242797.3	ENSG00000095585.16
23526	ENSG00000218347.2	ENSG00000242795.2	ENSG00000259241.1
23527	ENSG00000218350.1	ENSG00000242794.2	ENSG00000236936.1
23528	ENSG00000218351.2	ENSG00000242793.1	ENSG00000146955.10
23529	ENSG00000218357.3	ENSG00000242791.2	ENSG00000254704.3

23530	ENSG00000218358.2	ENSG00000242790.1	ENSG00000107882.11
23531	ENSG00000218359.1	ENSG00000242781.1	ENSG00000189295.13
23532	ENSG00000218363.1	ENSG00000242779.7	ENSG00000273265.1
23533	ENSG00000218410.1	ENSG00000242775.1	ENSG00000138756.17
23534	ENSG00000218416.4	ENSG00000242771.1	ENSG00000177350.6
23535	ENSG00000218418.2	ENSG00000242770.2	ENSG00000272950.1
23536	ENSG00000218424.2	ENSG00000242769.3	ENSG00000230999.1
23537	ENSG00000218426.5	ENSG00000242768.2	ENSG00000271752.1
23538	ENSG00000218428.1	ENSG00000242767.1	ENSG00000183199.6
23539	ENSG00000218454.2	ENSG00000242766.1	ENSG00000280303.2
23540	ENSG00000218459.1	ENSG00000242764.3	ENSG00000184669.9
23541	ENSG00000218472.2	ENSG00000242759.6	ENSG00000140104.14
23542	ENSG00000218475.2	ENSG00000242757.1	ENSG00000134802.17
23543	ENSG00000218476.2	ENSG00000242756.2	ENSG00000246130.1
23544	ENSG00000218483.1	ENSG00000242753.1	ENSG00000121957.14
23545	ENSG00000218490.1	ENSG00000242752.3	ENSG00000257773.1
23546	ENSG00000218499.1	ENSG00000242748.1	ENSG00000123836.15
23547	ENSG00000218502.3	ENSG00000242747.1	ENSG00000158089.15
23548	ENSG00000218510.8	ENSG00000242741.1	ENSG00000272746.1
23549	ENSG00000218512.2	ENSG00000242737.1	ENSG00000283914.1
23550	ENSG00000218520.5	ENSG00000242735.1	ENSG00000267745.1
23551	ENSG00000218521.1	ENSG00000242732.4	ENSG00000143318.13
23552	ENSG00000218536.1	ENSG00000242731.3	ENSG00000154479.13
23553	ENSG00000218537.1	ENSG00000242729.1	ENSG00000163633.12
23554	ENSG00000218549.2	ENSG00000242728.1	ENSG00000166816.14
23555	ENSG00000218561.1	ENSG00000242727.2	ENSG00000225370.1
23556	ENSG00000218565.2	ENSG00000242719.3	ENSG00000198879.11
23557	ENSG00000218574.1	ENSG00000242715.7	ENSG00000272181.1
23558	ENSG00000218577.1	ENSG00000242709.1	ENSG00000197024.8
23559	ENSG00000218582.2	ENSG00000242707.3	ENSG00000215840.3
23560	ENSG00000218586.3	ENSG00000242706.2	ENSG00000187513.9
23561	ENSG00000218596.2	ENSG00000242705.1	ENSG00000238125.1
23562	ENSG00000218617.1	ENSG00000242703.1	ENSG00000136542.9
23563	ENSG00000218631.1	ENSG00000242699.3	ENSG00000127328.21
23564	ENSG00000218632.3	ENSG00000242697.2	ENSG00000226084.5
23565	ENSG00000218643.2	ENSG00000242696.3	ENSG00000229515.1
23566	ENSG00000218672.1	ENSG00000242692.1	ENSG00000167748.11
23567	ENSG00000218676.1	ENSG00000242689.3	ENSG00000197813.5
23568	ENSG00000218682.1	ENSG00000242688.3	ENSG00000107443.16
23569	ENSG00000218689.2	ENSG00000242686.4	ENSG00000165424.6
23570	ENSG00000218690.2	ENSG00000242683.1	ENSG00000269570.2
23571	ENSG00000218698.1	ENSG00000242675.1	ENSG00000116774.12
23572	ENSG00000218713.1	ENSG00000242673.3	ENSG00000203761.5
23573	ENSG00000218716.1	ENSG00000242671.1	ENSG00000279637.1
23574	ENSG00000218725.2	ENSG00000242670.1	ENSG00000214655.10
23575	ENSG00000218728.3	ENSG00000242668.3	ENSG00000167315.18
23576	ENSG00000218730.1	ENSG00000242667.1	ENSG00000138175.9
23577	ENSG00000218732.1	ENSG00000242661.1	ENSG00000157064.11
23578	ENSG00000218739.10	ENSG00000242660.1	ENSG00000161835.11
23579	ENSG00000218748.1	ENSG00000242659.1	ENSG00000213468.6
23580	ENSG00000218749.1	ENSG00000242657.3	ENSG00000169905.12
23581	ENSG00000218754.4	ENSG00000242654.1	ENSG00000005108.16
23582	ENSG00000218757.1	ENSG00000242653.3	ENSG00000156515.23

23583	ENSG00000218766.1	ENSG00000242651.3	ENSG00000279066.1
23584	ENSG00000218772.2	ENSG00000242650.3	ENSG00000172602.11
23585	ENSG00000218776.4	ENSG00000242641.5	ENSG00000269350.1
23586	ENSG00000218792.2	ENSG00000242640.1	ENSG00000145248.7
23587	ENSG00000218793.1	ENSG00000242638.3	ENSG00000130561.17
23588	ENSG00000218803.1	ENSG00000242636.1	ENSG00000232699.3
23589	ENSG00000218806.2	ENSG00000242635.2	ENSG00000270115.1
23590	ENSG00000218809.1	ENSG00000242634.1	ENSG00000072682.18
23591	ENSG00000218813.1	ENSG00000242628.5	ENSG00000137726.17
23592	ENSG00000218819.4	ENSG00000242622.1	ENSG00000261448.1
23593	ENSG00000218823.2	ENSG00000242618.1	ENSG00000267277.1
23594	ENSG00000218834.1	ENSG00000242616.4	ENSG00000269843.1
23595	ENSG00000218839.5	ENSG00000242615.1	ENSG00000285830.1
23596	ENSG00000218857.1	ENSG00000242614.3	ENSG00000143815.15
23597	ENSG00000218868.1	ENSG00000242613.1	ENSG00000141098.13
23598	ENSG00000218870.1	ENSG00000242612.7	ENSG00000253986.1
23599	ENSG00000218872.1	ENSG00000242610.2	ENSG00000253678.2
23600	ENSG00000218890.1	ENSG00000242609.1	ENSG00000165115.15
23601	ENSG00000218891.5	ENSG00000242608.1	ENSG00000213621.3
23602	ENSG00000218893.1	ENSG00000242607.1	ENSG00000035499.13
23603	ENSG00000218896.1	ENSG00000242602.1	ENSG00000174080.11
23604	ENSG00000218902.3	ENSG00000242600.8	ENSG00000109320.12
23605	ENSG00000218965.1	ENSG00000242599.7	ENSG00000146285.14
23606	ENSG00000218976.2	ENSG00000242598.1	ENSG00000173692.13
23607	ENSG00000218980.2	ENSG00000242595.1	ENSG00000114805.17
23608	ENSG00000218986.1	ENSG00000242593.6	ENSG00000085382.12
23609	ENSG00000218991.1	ENSG00000242590.1	ENSG00000182224.12
23610	ENSG00000218996.1	ENSG00000242588.6	ENSG00000270433.1
23611	ENSG00000219023.1	ENSG00000242586.1	ENSG00000286261.1
23612	ENSG00000219027.2	ENSG00000242583.1	ENSG00000176915.15
23613	ENSG00000219039.2	ENSG00000242580.1	ENSG00000224852.1
23614	ENSG00000219061.4	ENSG00000242578.1	ENSG00000106524.9
23615	ENSG00000219073.7	ENSG00000242575.1	ENSG00000268521.1
23616	ENSG00000219074.1	ENSG00000242573.1	ENSG00000188647.13
23617	ENSG00000219085.1	ENSG00000242568.1	ENSG00000166033.13
23618	ENSG00000219087.2	ENSG00000242565.3	ENSG00000260314.3
23619	ENSG00000219088.1	ENSG00000242562.1	ENSG00000198939.8
23620	ENSG00000219095.1	ENSG00000242561.1	ENSG00000227081.5
23621	ENSG00000219102.3	ENSG00000242560.3	ENSG00000226855.1
23622	ENSG00000219133.2	ENSG00000242559.3	ENSG00000262777.1
23623	ENSG00000219135.1	ENSG00000242553.1	ENSG00000271993.1
23624	ENSG00000219139.1	ENSG00000242552.1	ENSG00000001084.12
23625	ENSG00000219146.1	ENSG00000242551.2	ENSG00000100629.17
23626	ENSG00000219149.4	ENSG00000242547.3	ENSG00000115590.14
23627	ENSG00000219150.2	ENSG00000242545.1	ENSG00000184227.8
23628	ENSG00000219159.4	ENSG00000242540.2	ENSG00000198933.9
23629	ENSG00000219163.2	ENSG00000242539.2	ENSG00000255339.6
23630	ENSG00000219186.2	ENSG00000242537.1	ENSG00000261220.2
23631	ENSG00000219188.1	ENSG00000242536.2	ENSG00000174501.14
23632	ENSG00000219190.1	ENSG00000242534.2	ENSG00000142751.15
23633	ENSG00000219200.11	ENSG00000242531.1	ENSG00000116874.11
23634	ENSG00000219201.4	ENSG00000242529.1	ENSG00000073711.11
23635	ENSG00000219222.1	ENSG00000242527.1	ENSG00000079974.17

23636	ENSG00000219240.2	ENSG00000242525.1	ENSG00000273295.1
23637	ENSG00000219249.2	ENSG00000242524.1	ENSG00000226519.1
23638	ENSG00000219253.1	ENSG00000242522.1	ENSG00000261126.7
23639	ENSG00000219257.2	ENSG00000242520.5	ENSG00000249492.1
23640	ENSG00000219262.1	ENSG00000242516.1	ENSG00000167123.19
23641	ENSG00000219273.1	ENSG00000242515.5	ENSG00000225616.2
23642	ENSG00000219274.1	ENSG00000242512.8	ENSG00000198431.16
23643	ENSG00000219280.1	ENSG00000242510.1	ENSG00000274486.1
23644	ENSG00000219284.2	ENSG00000242509.3	ENSG00000263244.2
23645	ENSG00000219294.6	ENSG00000242507.6	ENSG00000180537.13
23646	ENSG00000219297.1	ENSG00000242498.8	ENSG00000140961.13
23647	ENSG00000219298.1	ENSG00000242493.3	ENSG00000134376.16
23648	ENSG00000219302.2	ENSG00000242488.2	ENSG00000174628.16
23649	ENSG00000219314.1	ENSG00000242485.6	ENSG00000165490.13
23650	ENSG00000219329.1	ENSG00000242483.3	ENSG00000225535.6
23651	ENSG00000219355.2	ENSG00000242482.3	ENSG00000279641.1
23652	ENSG00000219361.1	ENSG00000242479.1	ENSG00000266325.1
23653	ENSG00000219368.3	ENSG00000242477.1	ENSG00000113583.8
23654	ENSG00000219375.1	ENSG00000242474.2	ENSG00000144681.10
23655	ENSG00000219384.1	ENSG00000242473.1	ENSG00000272114.1
23656	ENSG00000219387.2	ENSG00000242472.1	ENSG00000251000.1
23657	ENSG00000219391.1	ENSG00000242461.1	ENSG00000219681.2
23658	ENSG00000219392.1	ENSG00000242457.1	ENSG00000270550.1
23659	ENSG00000219395.2	ENSG00000242456.1	ENSG00000275778.2
23660	ENSG00000219404.2	ENSG00000242445.1	ENSG00000187987.9
23661	ENSG00000219409.2	ENSG00000242444.3	ENSG00000023839.11
23662	ENSG00000219410.6	ENSG00000242441.8	ENSG00000278601.1
23663	ENSG00000219430.2	ENSG00000242440.1	ENSG00000284981.1
23664	ENSG00000219433.2	ENSG00000242439.1	ENSG00000255328.1
23665	ENSG00000219435.6	ENSG00000242436.3	ENSG00000176920.12
23666	ENSG00000219438.8	ENSG00000242435.1	ENSG00000171365.17
23667	ENSG00000219445.2	ENSG00000242431.2	ENSG00000257838.5
23668	ENSG00000219451.3	ENSG00000242430.3	ENSG00000113303.11
23669	ENSG00000219453.1	ENSG00000242428.5	ENSG00000272144.1
23670	ENSG00000219463.1	ENSG00000242423.2	ENSG00000237264.4
23671	ENSG00000219470.1	ENSG00000242419.5	ENSG00000112306.8
23672	ENSG00000219481.10	ENSG00000242417.1	ENSG00000255987.1
23673	ENSG00000219487.2	ENSG00000242412.1	ENSG00000069869.16
23674	ENSG00000219491.6	ENSG00000242411.2	ENSG00000142687.18
23675	ENSG00000219492.4	ENSG00000242407.1	ENSG00000213742.6
23676	ENSG00000219500.1	ENSG00000242405.2	ENSG00000272750.1
23677	ENSG00000219507.4	ENSG00000242399.1	ENSG00000198464.14
23678	ENSG00000219529.2	ENSG00000242398.3	ENSG00000171105.14
23679	ENSG00000219532.2	ENSG00000242396.1	ENSG00000104957.14
23680	ENSG00000219545.11	ENSG00000242393.1	ENSG00000138778.11
23681	ENSG00000219547.1	ENSG00000242391.2	ENSG00000115364.14
23682	ENSG00000219549.1	ENSG00000242390.1	ENSG00000136436.14
23683	ENSG00000219553.2	ENSG00000242389.8	ENSG00000180667.10
23684	ENSG00000219559.1	ENSG00000242387.1	ENSG00000216866.5
23685	ENSG00000219565.2	ENSG00000242385.1	ENSG00000177868.12
23686	ENSG00000219575.1	ENSG00000242381.3	ENSG00000122417.15
23687	ENSG00000219582.3	ENSG00000242375.1	ENSG00000133983.15
23688	ENSG00000219592.2	ENSG00000242372.8	ENSG00000228347.1

23689	ENSG00000219604.1	ENSG00000242371.1	ENSG00000170382.12
23690	ENSG00000219607.3	ENSG00000242370.1	ENSG00000167207.13
23691	ENSG00000219608.3	ENSG00000242366.3	ENSG00000258056.2
23692	ENSG00000219619.2	ENSG00000242365.1	ENSG00000265194.1
23693	ENSG00000219622.1	ENSG00000242364.1	ENSG00000172927.8
23694	ENSG00000219626.9	ENSG00000242362.2	ENSG00000183283.16
23695	ENSG00000219627.1	ENSG00000242360.3	ENSG00000238098.9
23696	ENSG00000219642.2	ENSG00000242358.1	ENSG00000201822.1
23697	ENSG00000219653.1	ENSG00000242353.1	ENSG00000162739.14
23698	ENSG00000219665.8	ENSG00000242352.2	ENSG00000173867.10
23699	ENSG00000219666.2	ENSG00000242348.3	ENSG00000244490.1
23700	ENSG00000219669.1	ENSG00000242341.3	ENSG00000160961.12
23701	ENSG00000219681.2	ENSG00000242339.1	ENSG00000198400.11
23702	ENSG00000219682.4	ENSG00000242338.6	ENSG00000198246.9
23703	ENSG00000219693.3	ENSG00000242337.5	ENSG00000103343.13
23704	ENSG00000219699.2	ENSG00000242330.3	ENSG00000117614.10
23705	ENSG00000219700.1	ENSG00000242329.1	ENSG00000178233.17
23706	ENSG00000219702.1	ENSG00000242327.1	ENSG00000182141.10
23707	ENSG00000219703.1	ENSG00000242326.1	ENSG00000111291.8
23708	ENSG00000219712.1	ENSG00000242325.1	ENSG00000105668.7
23709	ENSG00000219722.1	ENSG00000242324.1	ENSG00000179348.11
23710	ENSG00000219736.1	ENSG00000242321.1	ENSG00000284740.1
23711	ENSG00000219738.2	ENSG00000242320.2	ENSG00000261423.1
23712	ENSG00000219747.1	ENSG00000242318.1	ENSG00000124196.5
23713	ENSG00000219755.1	ENSG00000242317.1	ENSG00000198836.9
23714	ENSG00000219757.1	ENSG00000242315.3	ENSG00000230968.1
23715	ENSG00000219758.6	ENSG00000242314.1	ENSG00000228141.7
23716	ENSG00000219770.1	ENSG00000242308.2	ENSG00000178105.11
23717	ENSG00000219773.1	ENSG00000242307.1	ENSG00000183569.18
23718	ENSG00000219776.2	ENSG00000242299.1	ENSG00000280181.1
23719	ENSG00000219784.2	ENSG00000242296.2	ENSG00000184371.14
23720	ENSG00000219790.3	ENSG00000242295.1	ENSG00000174640.13
23721	ENSG00000219797.2	ENSG00000242294.6	ENSG00000226148.1
23722	ENSG00000219806.1	ENSG00000242293.1	ENSG00000262879.5
23723	ENSG00000219807.2	ENSG00000242292.2	ENSG00000278404.1
23724	ENSG00000219814.2	ENSG00000242291.1	ENSG00000259522.3
23725	ENSG00000219863.5	ENSG00000242290.2	ENSG00000232995.7
23726	ENSG00000219867.1	ENSG00000242288.9	ENSG00000134470.21
23727	ENSG00000219870.2	ENSG00000242285.1	ENSG00000280020.1
23728	ENSG00000219881.1	ENSG00000242282.6	ENSG00000134020.8
23729	ENSG00000219891.2	ENSG00000242281.3	ENSG00000176083.17
23730	ENSG00000219902.1	ENSG00000242280.1	ENSG00000273424.1
23731	ENSG00000219926.11	ENSG00000242279.1	ENSG00000260277.1
23732	ENSG00000219928.2	ENSG00000242278.1	ENSG00000248727.5
23733	ENSG00000219930.4	ENSG00000242276.2	ENSG00000167074.15
23734	ENSG00000219932.6	ENSG00000242272.1	ENSG00000271711.1
23735	ENSG00000219940.2	ENSG00000242268.2	ENSG00000198586.14
23736	ENSG00000219941.1	ENSG00000242267.6	ENSG00000149136.9
23737	ENSG00000219951.4	ENSG00000242266.3	ENSG00000256569.1
23738	ENSG00000219986.2	ENSG00000242265.5	ENSG00000253224.1
23739	ENSG00000219992.2	ENSG00000242262.1	ENSG00000186020.13
23740	ENSG00000219993.1	ENSG00000242261.1	ENSG00000267355.2
23741	ENSG00000220008.3	ENSG00000242259.8	ENSG00000223802.7

23742	ENSG00000220030.1	ENSG00000242258.1	ENSG00000207231.1
23743	ENSG00000220069.1	ENSG00000242256.3	ENSG00000258976.1
23744	ENSG00000220076.1	ENSG00000242255.1	ENSG00000270587.1
23745	ENSG00000220091.2	ENSG00000242252.2	ENSG00000259918.1
23746	ENSG00000220105.2	ENSG00000242251.3	ENSG00000101082.14
23747	ENSG00000220110.1	ENSG00000242248.2	ENSG00000225855.6
23748	ENSG00000220113.2	ENSG00000242247.11	ENSG00000272767.1
23749	ENSG00000220125.1	ENSG00000242246.2	ENSG00000131446.16
23750	ENSG00000220130.1	ENSG00000242244.1	ENSG00000174697.5
23751	ENSG00000220131.1	ENSG00000242242.5	ENSG00000135482.7
23752	ENSG00000220132.1	ENSG00000242241.3	ENSG00000263847.1
23753	ENSG00000220139.1	ENSG00000242236.2	ENSG00000158023.10
23754	ENSG00000220154.2	ENSG00000242229.1	ENSG00000233264.2
23755	ENSG00000220157.4	ENSG00000242222.1	ENSG00000121577.13
23756	ENSG00000220161.4	ENSG00000242221.9	ENSG00000219507.4
23757	ENSG00000220181.1	ENSG00000242216.3	ENSG00000108679.13
23758	ENSG00000220184.2	ENSG00000242214.1	ENSG00000227165.8
23759	ENSG00000220201.7	ENSG00000242209.1	ENSG00000128654.14
23760	ENSG00000220204.1	ENSG00000242208.1	ENSG00000126247.10
23761	ENSG00000220205.9	ENSG00000242207.1	ENSG00000285737.1
23762	ENSG00000220212.1	ENSG00000242206.2	ENSG00000245970.2
23763	ENSG00000220237.1	ENSG00000242201.3	ENSG00000272563.1
23764	ENSG00000220240.1	ENSG00000242199.1	ENSG00000166452.11
23765	ENSG00000220248.1	ENSG00000242198.1	ENSG00000198707.15
23766	ENSG00000220256.4	ENSG00000242197.2	ENSG00000179023.8
23767	ENSG00000220267.1	ENSG00000242195.1	ENSG00000277692.1
23768	ENSG00000220291.1	ENSG00000242193.11	ENSG00000275223.1
23769	ENSG00000220305.1	ENSG00000242190.1	ENSG00000231875.1
23770	ENSG00000220311.1	ENSG00000242186.1	ENSG00000109794.13
23771	ENSG00000220323.4	ENSG00000242182.3	ENSG00000277959.1
23772	ENSG00000220326.2	ENSG00000242178.1	ENSG00000172939.9
23773	ENSG00000220340.1	ENSG00000242176.1	ENSG00000169413.3
23774	ENSG00000220343.5	ENSG00000242175.3	ENSG00000272182.1
23775	ENSG00000220347.1	ENSG00000242173.9	ENSG00000214313.8
23776	ENSG00000220349.4	ENSG00000242170.3	ENSG00000204856.12
23777	ENSG00000220370.1	ENSG00000242169.2	ENSG00000103599.20
23778	ENSG00000220377.1	ENSG00000242165.3	ENSG00000270673.1
23779	ENSG00000220378.3	ENSG00000242163.1	ENSG00000227187.1
23780	ENSG00000220392.1	ENSG00000242162.1	ENSG00000275559.1
23781	ENSG00000220412.1	ENSG00000242159.1	ENSG00000180921.7
23782	ENSG00000220418.1	ENSG00000242158.3	ENSG00000179627.10
23783	ENSG00000220446.2	ENSG00000242156.1	ENSG00000276768.1
23784	ENSG00000220447.1	ENSG00000242154.1	ENSG00000189060.5
23785	ENSG00000220472.1	ENSG00000242153.7	ENSG00000184378.3
23786	ENSG00000220483.4	ENSG00000242152.1	ENSG00000173653.7
23787	ENSG00000220494.4	ENSG00000242151.1	ENSG00000231595.1
23788	ENSG00000220505.2	ENSG00000242147.1	ENSG00000229017.6
23789	ENSG00000220506.2	ENSG00000242145.1	ENSG00000153902.14
23790	ENSG00000220514.1	ENSG00000242142.1	ENSG00000148219.16
23791	ENSG00000220515.2	ENSG00000242140.1	ENSG00000243609.1
23792	ENSG00000220517.2	ENSG00000242137.1	ENSG00000167548.15
23793	ENSG00000220522.2	ENSG00000242136.1	ENSG00000101489.20
23794	ENSG00000220537.1	ENSG00000242135.2	ENSG00000171617.14

23795	ENSG00000220540.1	ENSG00000242134.2	ENSG00000143595.13
23796	ENSG00000220541.4	ENSG00000242125.3	ENSG00000163145.12
23797	ENSG00000220553.1	ENSG00000242123.1	ENSG00000100991.12
23798	ENSG00000220556.4	ENSG00000242121.3	ENSG00000213967.10
23799	ENSG00000220557.1	ENSG00000242120.3	ENSG00000248008.2
23800	ENSG00000220563.1	ENSG00000242119.1	ENSG00000085788.13
23801	ENSG00000220575.7	ENSG00000242118.3	ENSG00000276088.1
23802	ENSG00000220581.1	ENSG00000242114.6	ENSG00000273004.1
23803	ENSG00000220583.1	ENSG00000242113.3	ENSG00000198189.11
23804	ENSG00000220585.4	ENSG00000242111.1	ENSG00000139163.15
23805	ENSG00000220586.2	ENSG00000242110.8	ENSG00000269236.1
23806	ENSG00000220598.1	ENSG00000242109.1	ENSG00000204620.3
23807	ENSG00000220600.2	ENSG00000242107.2	ENSG00000113916.18
23808	ENSG00000220614.1	ENSG00000242104.5	ENSG00000211818.1
23809	ENSG00000220635.2	ENSG00000242103.1	ENSG00000173597.9
23810	ENSG00000220643.1	ENSG00000242102.3	ENSG00000136197.12
23811	ENSG00000220660.2	ENSG00000242101.3	ENSG00000203943.8
23812	ENSG00000220666.2	ENSG00000242100.2	ENSG00000162735.18
23813	ENSG00000220685.3	ENSG00000242097.1	ENSG00000260923.6
23814	ENSG00000220694.2	ENSG00000242094.1	ENSG00000123700.4
23815	ENSG00000220695.1	ENSG00000242088.1	ENSG00000180346.3
23816	ENSG00000220702.1	ENSG00000242087.2	ENSG00000135549.15
23817	ENSG00000220721.2	ENSG00000242086.8	ENSG00000155066.16
23818	ENSG00000220725.1	ENSG00000242085.1	ENSG00000160201.11
23819	ENSG00000220730.2	ENSG00000242083.2	ENSG00000143632.14
23820	ENSG00000220733.1	ENSG00000242082.2	ENSG00000274963.1
23821	ENSG00000220734.1	ENSG00000242080.1	ENSG00000259950.1
23822	ENSG00000220739.2	ENSG00000242079.1	ENSG00000267074.1
23823	ENSG00000220744.1	ENSG00000242078.1	ENSG00000233337.1
23824	ENSG00000220745.2	ENSG00000242076.2	ENSG00000270580.5
23825	ENSG00000220748.2	ENSG00000242073.2	ENSG00000118242.16
23826	ENSG00000220749.4	ENSG00000242072.1	ENSG00000115129.14
23827	ENSG00000220771.2	ENSG00000242071.3	ENSG00000070159.14
23828	ENSG00000220773.1	ENSG00000242070.1	ENSG00000157349.16
23829	ENSG00000220785.7	ENSG00000242068.1	ENSG00000077420.16
23830	ENSG00000220793.5	ENSG00000242067.1	ENSG00000109944.10
23831	ENSG00000220804.8	ENSG00000242066.3	ENSG00000279086.1
23832	ENSG00000220831.4	ENSG00000242065.3	ENSG00000269845.1
23833	ENSG00000220842.6	ENSG00000242062.1	ENSG00000268336.1
23834	ENSG00000220848.5	ENSG00000242061.1	ENSG00000214046.8
23835	ENSG00000220867.1	ENSG00000242060.1	ENSG00000273674.4
23836	ENSG00000220868.2	ENSG00000242058.2	ENSG00000091972.18
23837	ENSG00000220871.1	ENSG00000242052.1	ENSG00000226205.1
23838	ENSG00000220875.1	ENSG00000242049.1	ENSG00000088038.19
23839	ENSG00000220884.2	ENSG00000242048.3	ENSG00000162129.13
23840	ENSG00000220891.1	ENSG00000242042.1	ENSG00000187166.1
23841	ENSG00000220908.2	ENSG00000242041.1	ENSG00000118482.11
23842	ENSG00000220913.1	ENSG00000242036.1	ENSG00000177483.11
23843	ENSG00000220918.1	ENSG00000242034.1	ENSG00000250771.2
23844	ENSG00000220920.1	ENSG00000242029.1	ENSG00000259112.2
23845	ENSG00000220924.4	ENSG00000242028.6	ENSG00000211949.3
23846	ENSG00000220925.2	ENSG00000242021.2	ENSG00000197415.12
23847	ENSG00000220937.3	ENSG00000242020.3	ENSG00000262429.1

23848	ENSG00000220948.5	ENSG00000242019.1	ENSG00000183864.5
23849	ENSG00000220949.2	ENSG00000242017.1	ENSG00000239835.1
23850	ENSG00000220960.1	ENSG00000242014.3	ENSG00000078795.16
23851	ENSG00000220986.1	ENSG00000242012.1	ENSG00000163568.15
23852	ENSG00000220988.1	ENSG00000242009.1	ENSG00000254527.1
23853	ENSG00000221015.1	ENSG00000242001.1	ENSG00000186081.12
23854	ENSG00000221017.1	ENSG00000241997.3	ENSG00000253731.2
23855	ENSG00000221023.1	ENSG00000241993.1	ENSG00000211665.3
23856	ENSG00000221025.1	ENSG00000241992.3	ENSG00000188404.9
23857	ENSG00000221028.1	ENSG00000241991.1	ENSG00000150681.10
23858	ENSG00000221031.1	ENSG00000241990.5	ENSG00000270091.1
23859	ENSG00000221033.1	ENSG00000241985.1	ENSG00000255084.1
23860	ENSG00000221036.5	ENSG00000241984.2	ENSG00000279356.1
23861	ENSG00000221038.1	ENSG00000241983.3	ENSG00000108622.11
23862	ENSG00000221039.3	ENSG00000241981.2	ENSG00000266368.1
23863	ENSG00000221040.2	ENSG00000241978.9	ENSG00000274092.1
23864	ENSG00000221042.1	ENSG00000241975.1	ENSG00000089289.16
23865	ENSG00000221043.2	ENSG00000241973.10	ENSG00000171680.21
23866	ENSG00000221044.2	ENSG00000241965.2	ENSG00000073614.12
23867	ENSG00000221046.1	ENSG00000241964.3	ENSG00000225643.1
23868	ENSG00000221052.3	ENSG00000241963.3	ENSG00000280670.3
23869	ENSG00000221055.1	ENSG00000241962.9	ENSG00000108861.9
23870	ENSG00000221059.1	ENSG00000241961.1	ENSG00000276991.1
23871	ENSG00000221060.1	ENSG00000241959.3	ENSG00000233892.1
23872	ENSG00000221063.3	ENSG00000241956.9	ENSG00000229331.1
23873	ENSG00000221065.1	ENSG00000241950.1	ENSG00000164096.13
23874	ENSG00000221066.1	ENSG00000241947.1	ENSG00000198825.13
23875	ENSG00000221081.1	ENSG00000241946.1	ENSG00000237803.5
23876	ENSG00000221083.1	ENSG00000241945.8	ENSG00000002726.20
23877	ENSG00000221091.1	ENSG00000241943.2	ENSG00000101115.13
23878	ENSG00000221093.1	ENSG00000241942.2	ENSG00000164978.18
23879	ENSG00000221102.1	ENSG00000241941.2	ENSG00000267263.1
23880	ENSG00000221114.1	ENSG00000241939.3	ENSG00000275963.1
23881	ENSG00000221116.1	ENSG00000241935.9	ENSG00000181800.5
23882	ENSG00000221120.1	ENSG00000241933.1	ENSG00000227304.3
23883	ENSG00000221125.2	ENSG00000241932.1	ENSG00000204131.9
23884	ENSG00000221139.1	ENSG00000241929.1	ENSG00000115310.18
23885	ENSG00000221148.1	ENSG00000241926.1	ENSG00000213799.12
23886	ENSG00000221164.1	ENSG00000241923.2	ENSG00000172289.3
23887	ENSG00000221176.1	ENSG00000241921.1	ENSG00000144460.12
23888	ENSG00000221184.1	ENSG00000241917.1	ENSG00000163545.8
23889	ENSG00000221187.1	ENSG00000241913.1	ENSG00000102780.16
23890	ENSG00000221190.1	ENSG00000241912.1	ENSG00000234163.1
23891	ENSG00000221200.1	ENSG00000241911.1	ENSG00000240563.2
23892	ENSG00000221203.1	ENSG00000241907.1	ENSG00000064012.21
23893	ENSG00000221206.1	ENSG00000241905.1	ENSG00000245750.8
23894	ENSG00000221214.1	ENSG00000241899.1	ENSG00000162383.12
23895	ENSG00000221216.1	ENSG00000241891.1	ENSG00000070540.13
23896	ENSG00000221227.1	ENSG00000241890.1	ENSG00000048740.18
23897	ENSG00000221230.1	ENSG00000241888.2	ENSG00000205702.11
23898	ENSG00000221238.1	ENSG00000241886.1	ENSG00000269145.2
23899	ENSG00000221240.1	ENSG00000241884.2	ENSG00000225131.2
23900	ENSG00000221241.1	ENSG00000241882.1	ENSG00000272255.1

23901	ENSG00000221245.1	ENSG00000241881.1	ENSG00000128311.14
23902	ENSG00000221251.1	ENSG00000241879.1	ENSG00000241631.3
23903	ENSG00000221255.1	ENSG00000241878.11	ENSG00000212123.4
23904	ENSG00000221261.1	ENSG00000241877.1	ENSG00000005001.10
23905	ENSG00000221263.1	ENSG00000241874.1	ENSG00000115268.9
23906	ENSG00000221264.1	ENSG00000241870.1	ENSG00000220091.2
23907	ENSG00000221265.1	ENSG00000241869.3	ENSG00000112851.14
23908	ENSG00000221269.1	ENSG00000241868.3	ENSG00000233332.1
23909	ENSG00000221273.1	ENSG00000241866.3	ENSG00000159904.11
23910	ENSG00000221275.1	ENSG00000241861.2	ENSG00000007129.18
23911	ENSG00000221287.1	ENSG00000241860.6	ENSG00000089195.15
23912	ENSG00000221288.1	ENSG00000241859.7	ENSG00000226085.3
23913	ENSG00000221296.2	ENSG00000241853.1	ENSG00000260949.1
23914	ENSG00000221300.1	ENSG00000241852.10	ENSG00000166444.19
23915	ENSG00000221303.1	ENSG00000241846.1	ENSG00000256937.1
23916	ENSG00000221305.1	ENSG00000241839.10	ENSG00000245534.6
23917	ENSG00000221325.1	ENSG00000241838.3	ENSG00000155749.12
23918	ENSG00000221331.1	ENSG00000241837.7	ENSG00000230725.5
23919	ENSG00000221332.2	ENSG00000241834.3	ENSG00000286172.1
23920	ENSG00000221333.1	ENSG00000241832.1	ENSG00000231889.7
23921	ENSG00000221340.1	ENSG00000241829.1	ENSG00000174171.5
23922	ENSG00000221345.2	ENSG00000241828.2	ENSG00000260917.1
23923	ENSG00000221348.1	ENSG00000241825.2	ENSG00000264339.1
23924	ENSG00000221355.1	ENSG00000241821.3	ENSG00000147003.7
23925	ENSG00000221363.1	ENSG00000241818.1	ENSG00000204843.12
23926	ENSG00000221365.1	ENSG00000241815.1	ENSG00000176024.18
23927	ENSG00000221369.1	ENSG00000241810.1	ENSG00000242261.1
23928	ENSG00000221371.1	ENSG00000241809.1	ENSG00000134504.13
23929	ENSG00000221375.1	ENSG00000241808.1	ENSG00000285331.2
23930	ENSG00000221381.1	ENSG00000241807.3	ENSG00000070950.10
23931	ENSG00000221387.1	ENSG00000241804.1	ENSG00000143393.16
23932	ENSG00000221390.1	ENSG00000241794.2	ENSG00000235159.1
23933	ENSG00000221393.1	ENSG00000241792.1	ENSG00000196407.12
23934	ENSG00000221394.1	ENSG00000241791.3	ENSG00000264066.6
23935	ENSG00000221398.1	ENSG00000241790.2	ENSG00000153046.18
23936	ENSG00000221400.2	ENSG00000241789.3	ENSG00000168454.11
23937	ENSG00000221406.1	ENSG00000241788.2	ENSG00000182333.14
23938	ENSG00000221410.1	ENSG00000241787.1	ENSG00000237883.1
23939	ENSG00000221411.1	ENSG00000241785.3	ENSG00000200087.1
23940	ENSG00000221420.2	ENSG00000241782.1	ENSG00000168096.14
23941	ENSG00000221421.1	ENSG00000241777.1	ENSG00000251259.1
23942	ENSG00000221430.1	ENSG00000241776.1	ENSG00000197037.11
23943	ENSG00000221436.1	ENSG00000241770.1	ENSG00000277027.1
23944	ENSG00000221439.1	ENSG00000241769.7	ENSG00000271537.1
23945	ENSG00000221440.1	ENSG00000241767.2	ENSG00000266946.1
23946	ENSG00000221442.1	ENSG00000241765.2	ENSG00000285399.2
23947	ENSG00000221445.3	ENSG00000241764.3	ENSG00000116883.8
23948	ENSG00000221455.2	ENSG00000241757.3	ENSG00000229140.9
23949	ENSG00000221456.1	ENSG00000241756.3	ENSG00000273261.1
23950	ENSG00000221459.1	ENSG00000241755.1	ENSG00000234983.1
23951	ENSG00000221461.2	ENSG00000241754.1	ENSG00000251194.2
23952	ENSG00000221463.3	ENSG00000241749.4	ENSG00000116016.14
23953	ENSG00000221464.1	ENSG00000241746.1	ENSG00000175602.3

23954	ENSG00000221466.2	ENSG00000241745.3	ENSG00000162959.13
23955	ENSG00000221468.1	ENSG00000241744.1	ENSG00000174175.17
23956	ENSG00000221475.1	ENSG00000241743.3	ENSG00000086504.16
23957	ENSG00000221476.2	ENSG00000241741.1	ENSG00000171812.13
23958	ENSG00000221479.3	ENSG00000241739.1	ENSG00000166451.13
23959	ENSG00000221491.2	ENSG00000241738.1	ENSG00000178226.10
23960	ENSG00000221493.1	ENSG00000241735.2	ENSG00000262420.3
23961	ENSG00000221494.1	ENSG00000241728.5	ENSG00000135069.14
23962	ENSG00000221496.2	ENSG00000241723.1	ENSG00000285420.1
23963	ENSG00000221498.1	ENSG00000241722.2	ENSG00000222043.2
23964	ENSG00000221500.1	ENSG00000241721.1	ENSG00000271888.1
23965	ENSG00000221502.1	ENSG00000241720.2	ENSG00000255837.1
23966	ENSG00000221507.1	ENSG00000241717.1	ENSG00000138755.5
23967	ENSG00000221510.1	ENSG00000241709.3	ENSG00000149311.18
23968	ENSG00000221514.1	ENSG00000241697.5	ENSG00000277007.1
23969	ENSG00000221518.1	ENSG00000241696.1	ENSG00000244357.3
23970	ENSG00000221520.1	ENSG00000241695.1	ENSG00000147799.11
23971	ENSG00000221525.1	ENSG00000241693.2	ENSG00000065534.18
23972	ENSG00000221527.1	ENSG00000241690.3	ENSG00000213860.4
23973	ENSG00000221533.1	ENSG00000241685.10	ENSG00000153347.9
23974	ENSG00000221537.1	ENSG00000241684.6	ENSG00000103995.14
23975	ENSG00000221539.1	ENSG00000241680.1	ENSG00000242258.1
23976	ENSG00000221540.1	ENSG00000241679.2	ENSG00000249738.10
23977	ENSG00000221545.1	ENSG00000241678.1	ENSG00000282885.1
23978	ENSG00000221548.1	ENSG00000241673.1	ENSG00000147117.7
23979	ENSG00000221552.1	ENSG00000241671.1	ENSG00000279228.2
23980	ENSG00000221562.1	ENSG00000241669.1	ENSG00000223837.2
23981	ENSG00000221563.1	ENSG00000241668.2	ENSG00000259000.1
23982	ENSG00000221564.1	ENSG00000241667.1	ENSG00000228107.1
23983	ENSG00000221571.3	ENSG00000241666.2	ENSG00000145901.15
23984	ENSG00000221574.1	ENSG00000241665.3	ENSG00000130741.11
23985	ENSG00000221583.1	ENSG00000241661.1	ENSG00000182220.14
23986	ENSG00000221585.1	ENSG00000241657.1	ENSG00000107833.10
23987	ENSG00000221586.1	ENSG00000241656.1	ENSG00000279161.1
23988	ENSG00000221594.1	ENSG00000241654.1	ENSG00000106400.12
23989	ENSG00000221598.3	ENSG00000241652.3	ENSG00000188026.13
23990	ENSG00000221601.1	ENSG00000241651.4	ENSG00000226415.1
23991	ENSG00000221603.1	ENSG00000241648.1	ENSG00000158234.12
23992	ENSG00000221604.1	ENSG00000241644.2	ENSG00000121073.14
23993	ENSG00000221611.1	ENSG00000241641.1	ENSG00000173698.18
23994	ENSG00000221614.1	ENSG00000241640.2	ENSG00000228149.1
23995	ENSG00000221616.1	ENSG00000241636.1	ENSG00000050344.9
23996	ENSG00000221628.1	ENSG00000241635.7	ENSG00000111885.7
23997	ENSG00000221630.3	ENSG00000241634.1	ENSG00000125538.11
23998	ENSG00000221633.2	ENSG00000241631.3	ENSG00000164742.16
23999	ENSG00000221634.1	ENSG00000241627.3	ENSG00000159905.14
24000	ENSG00000221638.2	ENSG00000241625.3	ENSG00000136144.12
24001	ENSG00000221639.1	ENSG00000241622.1	ENSG00000244754.8
24002	ENSG00000221641.1	ENSG00000241621.1	ENSG00000170734.11
24003	ENSG00000221643.1	ENSG00000241613.3	ENSG00000257335.8
24004	ENSG00000221649.1	ENSG00000241612.1	ENSG00000174444.15
24005	ENSG00000221650.1	ENSG00000241607.1	ENSG00000132466.18
24006	ENSG00000221656.1	ENSG00000241604.3	ENSG00000179304.16

24007	ENSG00000221662.1	ENSG00000241598.5	ENSG00000105171.10
24008	ENSG00000221669.1	ENSG00000241597.2	ENSG00000233406.5
24009	ENSG00000221673.2	ENSG00000241596.1	ENSG00000238249.2
24010	ENSG00000221676.1	ENSG00000241595.2	ENSG00000169885.10
24011	ENSG00000221680.1	ENSG00000241593.5	ENSG00000226976.3
24012	ENSG00000221697.1	ENSG00000241592.1	ENSG00000113068.9
24013	ENSG00000221698.1	ENSG00000241590.1	ENSG00000104823.9
24014	ENSG00000221703.1	ENSG00000241588.3	ENSG00000272906.1
24015	ENSG00000221705.1	ENSG00000241587.3	ENSG00000269743.3
24016	ENSG00000221710.1	ENSG00000241582.1	ENSG00000271635.1
24017	ENSG00000221711.1	ENSG00000241577.1	ENSG00000286223.1
24018	ENSG00000221716.1	ENSG00000241573.1	ENSG00000173757.9
24019	ENSG00000221719.1	ENSG00000241572.1	ENSG00000242265.5
24020	ENSG00000221725.1	ENSG00000241571.2	ENSG00000101639.18
24021	ENSG00000221737.1	ENSG00000241570.8	ENSG00000100316.16
24022	ENSG00000221739.1	ENSG00000241568.3	ENSG00000261064.1
24023	ENSG00000221740.1	ENSG00000241566.1	ENSG00000147471.12
24024	ENSG00000221745.3	ENSG00000241563.3	ENSG00000082996.19
24025	ENSG00000221750.1	ENSG00000241562.2	ENSG00000183688.4
24026	ENSG00000221753.1	ENSG00000241560.6	ENSG00000267419.2
24027	ENSG00000221754.1	ENSG00000241556.1	ENSG00000261971.7
24028	ENSG00000221760.1	ENSG00000241553.12	ENSG00000137628.17
24029	ENSG00000221763.1	ENSG00000241552.3	ENSG00000285591.1
24030	ENSG00000221768.2	ENSG00000241550.3	ENSG00000273749.5
24031	ENSG00000221771.1	ENSG00000241549.8	ENSG00000172264.17
24032	ENSG00000221782.1	ENSG00000241547.1	ENSG00000254461.1
24033	ENSG00000221783.1	ENSG00000241546.1	ENSG00000108474.16
24034	ENSG00000221788.1	ENSG00000241544.1	ENSG00000263647.1
24035	ENSG00000221792.1	ENSG00000241542.3	ENSG00000198768.10
24036	ENSG00000221801.1	ENSG00000241539.1	ENSG00000163739.4
24037	ENSG00000221803.1	ENSG00000241537.1	ENSG00000231909.7
24038	ENSG00000221806.1	ENSG00000241535.1	ENSG00000164403.14
24039	ENSG00000221808.1	ENSG00000241532.1	ENSG00000161031.13
24040	ENSG00000221813.4	ENSG00000241529.3	ENSG00000067445.20
24041	ENSG00000221817.9	ENSG00000241527.1	ENSG00000107020.10
24042	ENSG00000221818.9	ENSG00000241526.1	ENSG00000245685.6
24043	ENSG00000221819.6	ENSG00000241525.4	ENSG00000251357.4
24044	ENSG00000221821.4	ENSG00000241524.3	ENSG00000213213.13
24045	ENSG00000221823.11	ENSG00000241520.1	ENSG00000269243.1
24046	ENSG00000221826.10	ENSG00000241511.1	ENSG00000243488.3
24047	ENSG00000221829.9	ENSG00000241506.1	ENSG00000100196.11
24048	ENSG00000221836.3	ENSG00000241505.1	ENSG00000225400.1
24049	ENSG00000221837.5	ENSG00000241499.1	ENSG00000168517.10
24050	ENSG00000221838.9	ENSG00000241494.1	ENSG00000211791.2
24051	ENSG00000221840.4	ENSG00000241493.1	ENSG00000075702.18
24052	ENSG00000221843.4	ENSG00000241490.1	ENSG00000283128.1
24053	ENSG00000221844.2	ENSG00000241489.8	ENSG00000247735.2
24054	ENSG00000221845.4	ENSG00000241487.3	ENSG00000233262.1
24055	ENSG00000221849.2	ENSG00000241484.9	ENSG00000156482.11
24056	ENSG00000221852.5	ENSG00000241479.1	ENSG00000163932.14
24057	ENSG00000221855.1	ENSG00000241478.1	ENSG00000204634.12
24058	ENSG00000221857.7	ENSG00000241476.8	ENSG00000272840.1
24059	ENSG00000221858.3	ENSG00000241475.1	ENSG00000274210.1

24060	ENSG00000221859.2	ENSG00000241473.1	ENSG00000227492.1
24061	ENSG00000221864.4	ENSG00000241472.6	ENSG00000186280.7
24062	ENSG00000221866.9	ENSG00000241469.8	ENSG00000172638.13
24063	ENSG00000221867.9	ENSG00000241468.7	ENSG00000233170.4
24064	ENSG00000221869.4	ENSG00000241464.3	ENSG00000277662.1
24065	ENSG00000221874.4	ENSG00000241462.1	ENSG00000198727.2
24066	ENSG00000221878.12	ENSG00000241461.3	ENSG00000259416.2
24067	ENSG00000221879.3	ENSG00000241458.1	ENSG00000214018.3
24068	ENSG00000221880.3	ENSG00000241457.1	ENSG00000255670.1
24069	ENSG00000221882.3	ENSG00000241456.1	ENSG00000243055.1
24070	ENSG00000221883.3	ENSG00000241451.2	ENSG00000231826.5
24071	ENSG00000221886.4	ENSG00000241449.5	ENSG00000150551.10
24072	ENSG00000221887.6	ENSG00000241439.1	ENSG00000165832.6
24073	ENSG00000221888.4	ENSG00000241438.1	ENSG00000002016.17
24074	ENSG00000221890.4	ENSG00000241434.1	ENSG00000132963.8
24075	ENSG00000221891.2	ENSG00000241431.1	ENSG00000163701.19
24076	ENSG00000221900.5	ENSG00000241429.1	ENSG00000167889.12
24077	ENSG00000221909.3	ENSG00000241423.2	ENSG00000108950.12
24078	ENSG00000221910.2	ENSG00000241420.3	ENSG00000178988.11
24079	ENSG00000221914.10	ENSG00000241418.1	ENSG00000263657.1
24080	ENSG00000221916.4	ENSG00000241416.1	ENSG00000240219.1
24081	ENSG00000221923.9	ENSG00000241413.3	ENSG00000006047.13
24082	ENSG00000221926.12	ENSG00000241411.1	ENSG00000131931.8
24083	ENSG00000221930.6	ENSG00000241409.1	ENSG00000229589.1
24084	ENSG00000221931.2	ENSG00000241406.3	ENSG00000271320.1
24085	ENSG00000221932.6	ENSG00000241404.7	ENSG00000174705.13
24086	ENSG00000221933.3	ENSG00000241400.1	ENSG00000162636.16
24087	ENSG00000221937.4	ENSG00000241399.7	ENSG00000215695.1
24088	ENSG00000221938.5	ENSG00000241397.1	ENSG00000267547.1
24089	ENSG00000221944.7	ENSG00000241395.3	ENSG00000213965.3
24090	ENSG00000221946.7	ENSG00000241392.3	ENSG00000185100.10
24091	ENSG00000221947.7	ENSG00000241391.3	ENSG00000213871.3
24092	ENSG00000221949.5	ENSG00000241388.5	ENSG00000198865.10
24093	ENSG00000221953.5	ENSG00000241385.1	ENSG00000259408.2
24094	ENSG00000221954.2	ENSG00000241383.2	ENSG00000130779.20
24095	ENSG00000221955.10	ENSG00000241370.5	ENSG00000228084.1
24096	ENSG00000221957.8	ENSG00000241369.5	ENSG00000213204.8
24097	ENSG00000221962.5	ENSG00000241367.1	ENSG00000204536.14
24098	ENSG00000221963.6	ENSG00000241362.2	ENSG00000279907.1
24099	ENSG00000221968.9	ENSG00000241361.8	ENSG00000227097.5
24100	ENSG00000221970.2	ENSG00000241360.2	ENSG00000241599.1
24101	ENSG00000221971.3	ENSG00000241359.1	ENSG00000021300.14
24102	ENSG00000221977.2	ENSG00000241358.1	ENSG00000061656.9
24103	ENSG00000221978.12	ENSG00000241357.1	ENSG00000215795.2
24104	ENSG00000221983.7	ENSG00000241356.2	ENSG00000236514.1
24105	ENSG00000221986.7	ENSG00000241354.2	ENSG00000108349.17
24106	ENSG00000221988.13	ENSG00000241353.3	ENSG00000165181.16
24107	ENSG00000221989.2	ENSG00000241352.3	ENSG00000157741.15
24108	ENSG00000221990.4	ENSG00000241351.3	ENSG00000107771.17
24109	ENSG00000221994.10	ENSG00000241350.1	ENSG00000257539.2
24110	ENSG00000221995.5	ENSG00000241347.3	ENSG00000284922.1
24111	ENSG00000221996.6	ENSG00000241346.1	ENSG00000253744.1
24112	ENSG00000222000.7	ENSG00000241345.1	ENSG00000272666.1

24113	ENSG00000222001.2	ENSG00000241344.1	ENSG00000177076.6
24114	ENSG00000222004.7	ENSG00000241343.9	ENSG00000267007.1
24115	ENSG00000222005.9	ENSG00000241336.1	ENSG00000260729.1
24116	ENSG00000222007.6	ENSG00000241334.2	ENSG00000088826.18
24117	ENSG00000222009.8	ENSG00000241333.3	ENSG00000263366.2
24118	ENSG00000222011.9	ENSG00000241328.1	ENSG00000069345.12
24119	ENSG00000222012.1	ENSG00000241326.1	ENSG00000249054.2
24120	ENSG00000222014.5	ENSG00000241324.1	ENSG00000100342.20
24121	ENSG00000222017.1	ENSG00000241322.10	ENSG00000176783.15
24122	ENSG00000222018.1	ENSG00000241319.1	ENSG00000173275.12
24123	ENSG00000222019.7	ENSG00000241318.3	ENSG00000172845.15
24124	ENSG00000222020.2	ENSG00000241317.1	ENSG00000285976.1
24125	ENSG00000222022.1	ENSG00000241316.7	ENSG00000174348.13
24126	ENSG00000222024.2	ENSG00000241313.2	ENSG00000267216.1
24127	ENSG00000222028.4	ENSG00000241307.1	ENSG00000164023.14
24128	ENSG00000222030.1	ENSG00000241305.1	ENSG00000244041.7
24129	ENSG00000222031.1	ENSG00000241295.1	ENSG00000100106.21
24130	ENSG00000222032.1	ENSG00000241294.1	ENSG00000276077.4
24131	ENSG00000222033.1	ENSG00000241293.1	ENSG00000196876.15
24132	ENSG00000222035.3	ENSG00000241291.3	ENSG00000113083.14
24133	ENSG00000222036.7	ENSG00000241288.7	ENSG00000233093.5
24134	ENSG00000222037.5	ENSG00000241286.2	ENSG00000234511.10
24135	ENSG00000222038.3	ENSG00000241282.1	ENSG00000280387.1
24136	ENSG00000222041.11	ENSG00000241281.1	ENSG00000175189.4
24137	ENSG00000222042.1	ENSG00000241280.1	ENSG00000240634.1
24138	ENSG00000222043.2	ENSG00000241278.1	ENSG00000076706.17
24139	ENSG00000222044.1	ENSG00000241269.1	ENSG00000116128.11
24140	ENSG00000222046.2	ENSG00000241261.1	ENSG00000175514.2
24141	ENSG00000222047.8	ENSG00000241258.7	ENSG00000249138.1
24142	ENSG00000222051.1	ENSG00000241257.1	ENSG00000158427.15
24143	ENSG00000222054.1	ENSG00000241255.1	ENSG00000178445.9
24144	ENSG00000222057.1	ENSG00000241251.2	ENSG00000134871.18
24145	ENSG00000222067.1	ENSG00000241250.1	ENSG00000198680.4
24146	ENSG00000222068.1	ENSG00000241248.3	ENSG00000159307.19
24147	ENSG00000222069.1	ENSG00000241246.3	ENSG00000137936.18
24148	ENSG00000222071.1	ENSG00000241244.1	ENSG00000279762.3
24149	ENSG00000222072.1	ENSG00000241243.3	ENSG00000108107.14
24150	ENSG00000222076.1	ENSG00000241241.1	ENSG00000083444.17
24151	ENSG00000222078.1	ENSG00000241233.3	ENSG00000278422.1
24152	ENSG00000222087.1	ENSG00000241231.1	ENSG00000217950.4
24153	ENSG00000222092.1	ENSG00000241230.3	ENSG00000234534.1
24154	ENSG00000222094.1	ENSG00000241229.3	ENSG00000230715.3
24155	ENSG00000222095.1	ENSG00000241228.1	ENSG00000112242.15
24156	ENSG00000222099.1	ENSG00000241227.3	ENSG00000134056.12
24157	ENSG00000222102.1	ENSG00000241226.3	ENSG00000214013.9
24158	ENSG00000222107.1	ENSG00000241225.2	ENSG00000276071.1
24159	ENSG00000222108.1	ENSG00000241224.7	ENSG00000236852.1
24160	ENSG00000222111.1	ENSG00000241223.3	ENSG00000008382.15
24161	ENSG00000222112.1	ENSG00000241221.2	ENSG00000269970.1
24162	ENSG00000222114.1	ENSG00000241220.1	ENSG00000155961.5
24163	ENSG00000222118.1	ENSG00000241219.1	ENSG00000280232.1
24164	ENSG00000222122.1	ENSG00000241218.1	ENSG00000175193.13
24165	ENSG00000222123.1	ENSG00000241217.3	ENSG00000273375.1

24166	ENSG00000222126.1	ENSG00000241216.1	ENSG00000255150.2
24167	ENSG00000222129.1	ENSG00000241213.1	ENSG00000240350.2
24168	ENSG00000222139.1	ENSG00000241211.1	ENSG00000250966.2
24169	ENSG00000222145.1	ENSG00000241207.1	ENSG00000001460.18
24170	ENSG00000222146.1	ENSG00000241204.1	ENSG00000235530.6
24171	ENSG00000222148.1	ENSG00000241203.1	ENSG00000272668.2
24172	ENSG00000222150.1	ENSG00000241202.1	ENSG00000275484.1
24173	ENSG00000222154.1	ENSG00000241200.1	ENSG00000213049.3
24174	ENSG00000222160.1	ENSG00000241198.3	ENSG00000174028.6
24175	ENSG00000222162.1	ENSG00000241188.3	ENSG00000270765.5
24176	ENSG00000222164.1	ENSG00000241187.1	ENSG00000083223.18
24177	ENSG00000222170.1	ENSG00000241186.10	ENSG00000174740.8
24178	ENSG00000222174.1	ENSG00000241185.2	ENSG00000279106.1
24179	ENSG00000222177.1	ENSG00000241183.1	ENSG00000243819.4
24180	ENSG00000222178.1	ENSG00000241180.1	ENSG00000163617.11
24181	ENSG00000222179.1	ENSG00000241179.1	ENSG00000141026.6
24182	ENSG00000222182.1	ENSG00000241175.3	ENSG00000197670.6
24183	ENSG00000222185.1	ENSG00000241174.3	ENSG00000215313.3
24184	ENSG00000222202.1	ENSG00000241172.3	ENSG00000106948.16
24185	ENSG00000222205.1	ENSG00000241170.3	ENSG00000233328.3
24186	ENSG00000222206.1	ENSG00000241169.1	ENSG00000128271.22
24187	ENSG00000222207.1	ENSG00000241168.1	ENSG00000273311.1
24188	ENSG00000222208.1	ENSG00000241163.7	ENSG00000270706.1
24189	ENSG00000222209.1	ENSG00000241162.3	ENSG00000253305.2
24190	ENSG00000222210.1	ENSG00000241159.3	ENSG00000265681.7
24191	ENSG00000222213.1	ENSG00000241158.6	ENSG00000100418.8
24192	ENSG00000222220.1	ENSG00000241157.1	ENSG00000241680.1
24193	ENSG00000222222.1	ENSG00000241156.3	ENSG00000166402.8
24194	ENSG00000222224.1	ENSG00000241155.1	ENSG00000242294.6
24195	ENSG00000222225.1	ENSG00000241152.3	ENSG00000271105.1
24196	ENSG00000222230.1	ENSG00000241151.1	ENSG00000178127.13
24197	ENSG00000222231.1	ENSG00000241149.3	ENSG00000241634.1
24198	ENSG00000222232.1	ENSG00000241146.1	ENSG00000131409.13
24199	ENSG00000222236.1	ENSG00000241144.3	ENSG00000205534.6
24200	ENSG00000222238.1	ENSG00000241143.1	ENSG00000276115.1
24201	ENSG00000222240.1	ENSG00000241136.1	ENSG00000230084.5
24202	ENSG00000222244.1	ENSG00000241135.5	ENSG00000261594.3
24203	ENSG00000222246.1	ENSG00000241134.3	ENSG00000226318.1
24204	ENSG00000222248.1	ENSG00000241131.1	ENSG00000269892.1
24205	ENSG00000222249.1	ENSG00000241130.1	ENSG00000124920.13
24206	ENSG00000222251.1	ENSG00000241129.3	ENSG00000214650.2
24207	ENSG00000222255.1	ENSG00000241128.2	ENSG00000170484.9
24208	ENSG00000222257.1	ENSG00000241127.8	ENSG00000169896.17
24209	ENSG00000222259.1	ENSG00000241123.1	ENSG00000188811.14
24210	ENSG00000222266.1	ENSG00000241119.2	ENSG00000269584.1
24211	ENSG00000222267.1	ENSG00000241114.1	ENSG00000132570.14
24212	ENSG00000222268.1	ENSG00000241112.1	ENSG00000273521.1
24213	ENSG00000222276.1	ENSG00000241111.1	ENSG00000198018.7
24214	ENSG00000222281.1	ENSG00000241106.8	ENSG00000183439.8
24215	ENSG00000222282.1	ENSG00000241105.1	ENSG00000177410.12
24216	ENSG00000222285.1	ENSG00000241104.5	ENSG00000213942.3
24217	ENSG00000222287.1	ENSG00000241103.1	ENSG00000264735.1
24218	ENSG00000222293.1	ENSG00000241102.1	ENSG00000259442.1

24219	ENSG00000222297.1	ENSG00000241101.1	ENSG00000108576.10
24220	ENSG00000222300.1	ENSG00000241098.1	ENSG00000167779.9
24221	ENSG00000222302.1	ENSG00000241097.2	ENSG00000241015.2
24222	ENSG00000222303.1	ENSG00000241095.1	ENSG00000182175.14
24223	ENSG00000222305.1	ENSG00000241088.2	ENSG00000187676.8
24224	ENSG00000222308.1	ENSG00000241084.1	ENSG00000260487.1
24225	ENSG00000222312.1	ENSG00000241082.3	ENSG00000213184.3
24226	ENSG00000222313.1	ENSG00000241081.1	ENSG00000223551.1
24227	ENSG00000222314.1	ENSG00000241074.3	ENSG00000251139.2
24228	ENSG00000222317.1	ENSG00000241073.1	ENSG00000159658.11
24229	ENSG00000222320.1	ENSG00000241069.1	ENSG00000104450.12
24230	ENSG00000222321.1	ENSG00000241067.2	ENSG00000244242.2
24231	ENSG00000222327.1	ENSG00000241064.3	ENSG00000280378.1
24232	ENSG00000222328.1	ENSG00000241061.3	ENSG00000166401.14
24233	ENSG00000222329.1	ENSG00000241059.2	ENSG00000163520.14
24234	ENSG00000222335.1	ENSG00000241058.4	ENSG00000002586.20
24235	ENSG00000222337.1	ENSG00000241057.2	ENSG00000108091.11
24236	ENSG00000222338.1	ENSG00000241054.1	ENSG00000238005.3
24237	ENSG00000222343.1	ENSG00000241052.1	ENSG00000226935.6
24238	ENSG00000222344.1	ENSG00000241048.1	ENSG00000183770.7
24239	ENSG00000222345.1	ENSG00000241045.1	ENSG00000236911.6
24240	ENSG00000222346.1	ENSG00000241042.1	ENSG00000279794.1
24241	ENSG00000222351.1	ENSG00000241037.3	ENSG00000271938.1
24242	ENSG00000222352.1	ENSG00000241035.2	ENSG00000081377.16
24243	ENSG00000222355.1	ENSG00000241032.3	ENSG00000130939.19
24244	ENSG00000222356.1	ENSG00000241030.1	ENSG00000224963.3
24245	ENSG00000222357.1	ENSG00000241026.1	ENSG00000231064.7
24246	ENSG00000222359.1	ENSG00000241022.1	ENSG00000137845.15
24247	ENSG00000222361.1	ENSG00000241020.1	ENSG00000163116.10
24248	ENSG00000222363.1	ENSG00000241018.1	ENSG00000236364.3
24249	ENSG00000222365.1	ENSG00000241014.2	ENSG00000183734.4
24250	ENSG00000222370.1	ENSG00000241011.1	ENSG00000047648.22
24251	ENSG00000222371.1	ENSG00000241008.1	ENSG00000185585.20
24252	ENSG00000222375.1	ENSG00000241007.1	ENSG00000274917.1
24253	ENSG00000222376.1	ENSG00000241003.1	ENSG00000196526.10
24254	ENSG00000222378.1	ENSG00000241002.1	ENSG00000262160.1
24255	ENSG00000222383.1	ENSG00000240997.3	ENSG00000141294.10
24256	ENSG00000222385.1	ENSG00000240996.1	ENSG00000165914.15
24257	ENSG00000222386.1	ENSG00000240995.2	ENSG00000167996.16
24258	ENSG00000222389.1	ENSG00000240993.3	ENSG00000280372.1
24259	ENSG00000222394.2	ENSG00000240992.1	ENSG00000073464.12
24260	ENSG00000222395.2	ENSG00000240991.3	ENSG00000254681.6
24261	ENSG00000222397.1	ENSG00000240990.10	ENSG00000282850.2
24262	ENSG00000222398.1	ENSG00000240983.1	ENSG00000279996.1
24263	ENSG00000222399.1	ENSG00000240980.1	ENSG00000141505.12
24264	ENSG00000222404.1	ENSG00000240979.1	ENSG00000231731.7
24265	ENSG00000222405.1	ENSG00000240977.3	ENSG00000204618.8
24266	ENSG00000222407.1	ENSG00000240975.1	ENSG00000280274.1
24267	ENSG00000222412.1	ENSG00000240974.1	ENSG00000175294.5
24268	ENSG00000222413.1	ENSG00000240973.1	ENSG00000229184.2
24269	ENSG00000222414.1	ENSG00000240972.2	ENSG00000235978.6
24270	ENSG00000222416.1	ENSG00000240970.1	ENSG00000168556.7
24271	ENSG00000222418.1	ENSG00000240966.3	ENSG00000143882.12

24272	ENSG00000222419.1	ENSG00000240964.3	ENSG00000132622.11
24273	ENSG00000222421.1	ENSG00000240963.1	ENSG00000227694.1
24274	ENSG00000222426.1	ENSG00000240961.3	ENSG00000186566.13
24275	ENSG00000222427.1	ENSG00000240960.1	ENSG00000260095.1
24276	ENSG00000222428.1	ENSG00000240959.1	ENSG00000206908.1
24277	ENSG00000222429.1	ENSG00000240954.1	ENSG00000214199.3
24278	ENSG00000222430.1	ENSG00000240951.1	ENSG00000213553.4
24279	ENSG00000222431.1	ENSG00000240940.3	ENSG00000128567.17
24280	ENSG00000222432.1	ENSG00000240936.3	ENSG00000165300.7
24281	ENSG00000222436.1	ENSG00000240935.6	ENSG00000256912.1
24282	ENSG00000222438.1	ENSG00000240934.2	ENSG00000213609.3
24283	ENSG00000222439.1	ENSG00000240929.2	ENSG00000270017.1
24284	ENSG00000222440.1	ENSG00000240927.3	ENSG00000115107.20
24285	ENSG00000222445.1	ENSG00000240925.1	ENSG00000174428.17
24286	ENSG00000222448.1	ENSG00000240922.1	ENSG00000235501.5
24287	ENSG00000222449.1	ENSG00000240919.1	ENSG00000232454.2
24288	ENSG00000222451.1	ENSG00000240915.2	ENSG00000183977.13
24289	ENSG00000222452.1	ENSG00000240914.1	ENSG00000231362.1
24290	ENSG00000222455.1	ENSG00000240912.1	ENSG00000233922.2
24291	ENSG00000222457.1	ENSG00000240905.3	ENSG00000256803.1
24292	ENSG00000222459.1	ENSG00000240902.1	ENSG00000177732.8
24293	ENSG00000222460.1	ENSG00000240898.1	ENSG00000233757.6
24294	ENSG00000222465.1	ENSG00000240895.1	ENSG00000180425.11
24295	ENSG00000222467.1	ENSG00000240891.7	ENSG00000252464.1
24296	ENSG00000222468.1	ENSG00000240890.2	ENSG00000198087.7
24297	ENSG00000222472.1	ENSG00000240889.1	ENSG00000275464.4
24298	ENSG00000222477.1	ENSG00000240888.1	ENSG00000129450.8
24299	ENSG00000222486.1	ENSG00000240882.1	ENSG00000161647.18
24300	ENSG00000222488.1	ENSG00000240881.1	ENSG00000183784.7
24301	ENSG00000222489.1	ENSG00000240877.3	ENSG00000196371.3
24302	ENSG00000222490.1	ENSG00000240875.5	ENSG00000244952.2
24303	ENSG00000222496.1	ENSG00000240874.1	ENSG00000125447.17
24304	ENSG00000222499.1	ENSG00000240873.1	ENSG00000235245.1
24305	ENSG00000222500.1	ENSG00000240871.5	ENSG00000269335.5
24306	ENSG00000222501.1	ENSG00000240870.2	ENSG00000267260.1
24307	ENSG00000222503.1	ENSG00000240869.3	ENSG00000228196.1
24308	ENSG00000222506.1	ENSG00000240868.1	ENSG00000237868.1
24309	ENSG00000222509.1	ENSG00000240864.3	ENSG00000243560.3
24310	ENSG00000222511.2	ENSG00000240863.3	ENSG00000169609.14
24311	ENSG00000222514.1	ENSG00000240861.1	ENSG00000170180.22
24312	ENSG00000222515.1	ENSG00000240859.2	ENSG00000264577.1
24313	ENSG00000222520.1	ENSG00000240857.2	ENSG00000261504.1
24314	ENSG00000222522.1	ENSG00000240854.1	ENSG00000199645.1
24315	ENSG00000222524.1	ENSG00000240853.3	ENSG00000143545.8
24316	ENSG00000222529.1	ENSG00000240849.11	ENSG00000086232.13
24317	ENSG00000222532.1	ENSG00000240847.3	ENSG00000226416.1
24318	ENSG00000222533.1	ENSG00000240846.2	ENSG00000169955.7
24319	ENSG00000222536.1	ENSG00000240842.1	ENSG00000250031.1
24320	ENSG00000222543.1	ENSG00000240837.3	ENSG00000111252.10
24321	ENSG00000222544.1	ENSG00000240828.1	ENSG00000165887.11
24322	ENSG00000222546.1	ENSG00000240827.1	ENSG00000146350.14
24323	ENSG00000222552.1	ENSG00000240824.1	ENSG00000239559.2
24324	ENSG00000222558.1	ENSG00000240823.3	ENSG00000169704.4

24325	ENSG00000222560.1	ENSG00000240821.1	ENSG00000165383.11
24326	ENSG00000222561.1	ENSG00000240813.2	ENSG00000167985.6
24327	ENSG00000222574.1	ENSG00000240808.1	ENSG00000198794.12
24328	ENSG00000222578.1	ENSG00000240804.1	ENSG00000239670.1
24329	ENSG00000222579.1	ENSG00000240803.3	ENSG00000275329.1
24330	ENSG00000222581.1	ENSG00000240801.1	ENSG00000102934.10
24331	ENSG00000222582.1	ENSG00000240796.1	ENSG00000070404.10
24332	ENSG00000222583.1	ENSG00000240793.1	ENSG00000259649.4
24333	ENSG00000222585.1	ENSG00000240792.1	ENSG00000255220.1
24334	ENSG00000222588.1	ENSG00000240791.1	ENSG00000133065.11
24335	ENSG00000222589.1	ENSG00000240790.1	ENSG00000267342.1
24336	ENSG00000222592.1	ENSG00000240787.1	ENSG00000229547.4
24337	ENSG00000222594.1	ENSG00000240785.2	ENSG00000259932.1
24338	ENSG00000222598.1	ENSG00000240777.2	ENSG00000173809.18
24339	ENSG00000222601.1	ENSG00000240776.1	ENSG00000150457.9
24340	ENSG00000222604.2	ENSG00000240775.1	ENSG00000280015.1
24341	ENSG00000222607.1	ENSG00000240774.1	ENSG00000111405.9
24342	ENSG00000222608.1	ENSG00000240772.3	ENSG00000253919.1
24343	ENSG00000222609.1	ENSG00000240770.5	ENSG00000124333.16
24344	ENSG00000222610.1	ENSG00000240767.3	ENSG00000181544.15
24345	ENSG00000222612.1	ENSG00000240766.1	ENSG00000272681.2
24346	ENSG00000222613.1	ENSG00000240764.3	ENSG00000106246.17
24347	ENSG00000222614.1	ENSG00000240761.1	ENSG00000152078.10
24348	ENSG00000222616.1	ENSG00000240760.1	ENSG00000204758.7
24349	ENSG00000222617.1	ENSG00000240759.1	ENSG00000260806.1
24350	ENSG00000222623.1	ENSG00000240758.2	ENSG00000188033.10
24351	ENSG00000222624.1	ENSG00000240755.1	ENSG00000169032.9
24352	ENSG00000222626.1	ENSG00000240752.1	ENSG00000197576.13
24353	ENSG00000222627.1	ENSG00000240751.1	ENSG00000233296.1
24354	ENSG00000222629.1	ENSG00000240750.3	ENSG00000230641.1
24355	ENSG00000222630.2	ENSG00000240747.7	ENSG00000178537.10
24356	ENSG00000222635.1	ENSG00000240739.1	ENSG00000086061.16
24357	ENSG00000222636.1	ENSG00000240738.1	ENSG00000204386.10
24358	ENSG00000222640.1	ENSG00000240733.3	ENSG00000260495.1
24359	ENSG00000222644.1	ENSG00000240731.1	ENSG00000279352.1
24360	ENSG00000222649.1	ENSG00000240729.1	ENSG00000126351.12
24361	ENSG00000222650.1	ENSG00000240724.2	ENSG00000233064.2
24362	ENSG00000222652.1	ENSG00000240723.3	ENSG00000205531.13
24363	ENSG00000222658.1	ENSG00000240721.2	ENSG00000269514.2
24364	ENSG00000222659.1	ENSG00000240720.8	ENSG00000145217.14
24365	ENSG00000222663.1	ENSG00000240718.3	ENSG00000198099.8
24366	ENSG00000222664.1	ENSG00000240713.3	ENSG00000128408.8
24367	ENSG00000222666.2	ENSG00000240710.1	ENSG00000145649.8
24368	ENSG00000222667.1	ENSG00000240708.1	ENSG00000110025.13
24369	ENSG00000222675.1	ENSG00000240707.2	ENSG00000252105.1
24370	ENSG00000222678.1	ENSG00000240704.1	ENSG00000122481.17
24371	ENSG00000222679.1	ENSG00000240698.1	ENSG00000246363.3
24372	ENSG00000222682.1	ENSG00000240695.1	ENSG00000065518.8
24373	ENSG00000222685.1	ENSG00000240694.9	ENSG00000276672.1
24374	ENSG00000222686.1	ENSG00000240692.3	ENSG00000278058.1
24375	ENSG00000222691.1	ENSG00000240687.1	ENSG00000145284.12
24376	ENSG00000222693.1	ENSG00000240682.9	ENSG00000180611.7
24377	ENSG00000222698.1	ENSG00000240680.1	ENSG00000161944.16

24378	ENSG00000222701.1	ENSG00000240677.1	ENSG00000105649.9
24379	ENSG00000222704.1	ENSG00000240673.1	ENSG00000013364.19
24380	ENSG00000222705.1	ENSG00000240671.4	ENSG00000181291.7
24381	ENSG00000222706.1	ENSG00000240669.1	ENSG00000239221.3
24382	ENSG00000222713.1	ENSG00000240668.1	ENSG00000155463.13
24383	ENSG00000222714.1	ENSG00000240667.1	ENSG00000140836.17
24384	ENSG00000222715.1	ENSG00000240666.2	ENSG00000272980.4
24385	ENSG00000222721.1	ENSG00000240665.1	ENSG00000152256.13
24386	ENSG00000222724.1	ENSG00000240663.3	ENSG00000234709.2
24387	ENSG00000222726.1	ENSG00000240661.3	ENSG00000280434.1
24388	ENSG00000222727.1	ENSG00000240654.6	ENSG00000241429.1
24389	ENSG00000222733.1	ENSG00000240652.1	ENSG00000266844.1
24390	ENSG00000222736.1	ENSG00000240651.1	ENSG00000272157.1
24391	ENSG00000222740.1	ENSG00000240647.3	ENSG00000102580.15
24392	ENSG00000222741.1	ENSG00000240641.3	ENSG00000213492.2
24393	ENSG00000222743.1	ENSG00000240639.3	ENSG00000110851.12
24394	ENSG00000222744.1	ENSG00000240637.3	ENSG00000182732.18
24395	ENSG00000222747.1	ENSG00000240634.1	ENSG00000103121.8
24396	ENSG00000222750.1	ENSG00000240632.6	ENSG00000257167.2
24397	ENSG00000222755.1	ENSG00000240631.1	ENSG00000236723.2
24398	ENSG00000222760.1	ENSG00000240627.1	ENSG00000264895.1
24399	ENSG00000222761.1	ENSG00000240626.3	ENSG00000224531.6
24400	ENSG00000222764.1	ENSG00000240624.1	ENSG00000233785.1
24401	ENSG00000222765.1	ENSG00000240622.1	ENSG00000163736.4
24402	ENSG00000222767.1	ENSG00000240621.1	ENSG00000211793.2
24403	ENSG00000222774.1	ENSG00000240616.1	ENSG00000136247.14
24404	ENSG00000222777.1	ENSG00000240613.3	ENSG00000177098.8
24405	ENSG00000222778.1	ENSG00000240611.1	ENSG00000235910.1
24406	ENSG00000222783.1	ENSG00000240606.3	ENSG00000276003.1
24407	ENSG00000222784.1	ENSG00000240602.7	ENSG00000279443.1
24408	ENSG00000222788.1	ENSG00000240601.1	ENSG00000259514.1
24409	ENSG00000222790.1	ENSG00000240596.1	ENSG00000224356.5
24410	ENSG00000222791.1	ENSG00000240591.1	ENSG00000272100.1
24411	ENSG00000222792.1	ENSG00000240590.1	ENSG00000167657.14
24412	ENSG00000222795.1	ENSG00000240589.3	ENSG00000237668.1
24413	ENSG00000222796.1	ENSG00000240584.3	ENSG00000243701.6
24414	ENSG00000222800.1	ENSG00000240579.1	ENSG00000271122.1
24415	ENSG00000222806.1	ENSG00000240578.1	ENSG00000100138.15
24416	ENSG00000222808.1	ENSG00000240577.3	ENSG00000172809.13
24417	ENSG00000222810.1	ENSG00000240573.1	ENSG00000230673.3
24418	ENSG00000222821.1	ENSG00000240572.1	ENSG00000006453.14
24419	ENSG00000222826.1	ENSG00000240571.1	ENSG00000227312.2
24420	ENSG00000222831.1	ENSG00000240568.1	ENSG00000250474.2
24421	ENSG00000222832.1	ENSG00000240567.1	ENSG00000143387.13
24422	ENSG00000222835.1	ENSG00000240566.1	ENSG00000273314.1
24423	ENSG00000222838.1	ENSG00000240563.2	ENSG00000218510.8
24424	ENSG00000222842.1	ENSG00000240562.1	ENSG00000138134.12
24425	ENSG00000222844.1	ENSG00000240554.1	ENSG00000227896.2
24426	ENSG00000222845.1	ENSG00000240553.1	ENSG00000259803.7
24427	ENSG00000222849.1	ENSG00000240549.2	ENSG00000232774.7
24428	ENSG00000222852.1	ENSG00000240545.3	ENSG00000137177.20
24429	ENSG00000222854.1	ENSG00000240542.4	ENSG00000078618.21
24430	ENSG00000222858.1	ENSG00000240541.2	ENSG00000178809.11

24431	ENSG00000222859.1	ENSG00000240540.2	ENSG00000227508.6
24432	ENSG00000222862.2	ENSG00000240535.8	ENSG00000138709.19
24433	ENSG00000222869.1	ENSG00000240534.1	ENSG00000099308.10
24434	ENSG00000222870.1	ENSG00000240533.3	ENSG00000277701.4
24435	ENSG00000222872.1	ENSG00000240531.1	ENSG00000152404.15
24436	ENSG00000222874.1	ENSG00000240527.1	ENSG00000246985.7
24437	ENSG00000222880.1	ENSG00000240522.1	ENSG00000226904.1
24438	ENSG00000222881.1	ENSG00000240521.1	ENSG00000227766.1
24439	ENSG00000222883.1	ENSG00000240520.6	ENSG00000276698.1
24440	ENSG00000222889.1	ENSG00000240519.1	ENSG00000073146.16
24441	ENSG00000222890.1	ENSG00000240518.2	ENSG00000101290.14
24442	ENSG00000222895.1	ENSG00000240513.1	ENSG00000269680.1
24443	ENSG00000222898.1	ENSG00000240511.1	ENSG00000066739.12
24444	ENSG00000222909.1	ENSG00000240505.8	ENSG00000187642.9
24445	ENSG00000222915.1	ENSG00000240502.2	ENSG00000279373.1
24446	ENSG00000222920.1	ENSG00000240499.7	ENSG00000262691.1
24447	ENSG00000222921.1	ENSG00000240498.8	ENSG00000113790.11
24448	ENSG00000222922.1	ENSG00000240497.2	ENSG00000158715.6
24449	ENSG00000222923.1	ENSG00000240494.2	ENSG00000175061.17
24450	ENSG00000222924.1	ENSG00000240490.3	ENSG00000231245.2
24451	ENSG00000222931.1	ENSG00000240489.1	ENSG00000245261.1
24452	ENSG00000222932.1	ENSG00000240487.1	ENSG00000198920.10
24453	ENSG00000222934.1	ENSG00000240486.2	ENSG00000231019.1
24454	ENSG00000222937.1	ENSG00000240484.1	ENSG00000217644.5
24455	ENSG00000222940.1	ENSG00000240481.3	ENSG00000227057.10
24456	ENSG00000222941.2	ENSG00000240480.1	ENSG00000230510.6
24457	ENSG00000222942.1	ENSG00000240478.1	ENSG00000168280.17
24458	ENSG00000222950.1	ENSG00000240477.1	ENSG00000127527.14
24459	ENSG00000222952.1	ENSG00000240476.1	ENSG00000214226.9
24460	ENSG00000222955.1	ENSG00000240474.3	ENSG00000162604.12
24461	ENSG00000222958.1	ENSG00000240471.1	ENSG00000134371.12
24462	ENSG00000222960.1	ENSG00000240470.3	ENSG00000251600.7
24463	ENSG00000222966.1	ENSG00000240463.1	ENSG00000160117.15
24464	ENSG00000222969.1	ENSG00000240459.2	ENSG00000170836.11
24465	ENSG00000222971.1	ENSG00000240458.1	ENSG00000163576.18
24466	ENSG00000222972.1	ENSG00000240457.3	ENSG00000169085.13
24467	ENSG00000222973.1	ENSG00000240454.1	ENSG00000188933.15
24468	ENSG00000222974.1	ENSG00000240452.1	ENSG00000279792.1
24469	ENSG00000222976.1	ENSG00000240450.1	ENSG00000276724.1
24470	ENSG00000222979.1	ENSG00000240449.1	ENSG00000268006.1
24471	ENSG00000222982.1	ENSG00000240445.3	ENSG00000140990.15
24472	ENSG00000222983.1	ENSG00000240443.1	ENSG00000274251.1
24473	ENSG00000222985.1	ENSG00000240441.1	ENSG00000211947.2
24474	ENSG00000222986.1	ENSG00000240439.3	ENSG00000226616.1
24475	ENSG00000222987.1	ENSG00000240438.2	ENSG00000110934.11
24476	ENSG00000222990.1	ENSG00000240436.1	ENSG00000140470.14
24477	ENSG00000222997.1	ENSG00000240435.2	ENSG00000284543.1
24478	ENSG00000222998.1	ENSG00000240432.3	ENSG00000103253.18
24479	ENSG00000223001.1	ENSG00000240429.1	ENSG00000214851.4
24480	ENSG00000223003.1	ENSG00000240427.1	ENSG00000155893.13
24481	ENSG00000223004.1	ENSG00000240426.1	ENSG00000225831.1
24482	ENSG00000223006.1	ENSG00000240424.2	ENSG00000279348.1
24483	ENSG00000223007.1	ENSG00000240423.2	ENSG00000186854.11

24484	ENSG00000223012.1	ENSG00000240419.1	ENSG00000129197.14
24485	ENSG00000223013.1	ENSG00000240418.1	ENSG00000258376.2
24486	ENSG00000223015.1	ENSG00000240416.1	ENSG00000034533.11
24487	ENSG00000223019.1	ENSG00000240412.1	ENSG00000136261.15
24488	ENSG00000223023.1	ENSG00000240411.2	ENSG00000229689.3
24489	ENSG00000223024.1	ENSG00000240409.1	ENSG00000201388.1
24490	ENSG00000223027.1	ENSG00000240405.6	ENSG00000073910.21
24491	ENSG00000223037.1	ENSG00000240404.2	ENSG00000227799.1
24492	ENSG00000223039.1	ENSG00000240401.8	ENSG00000214300.7
24493	ENSG00000223040.1	ENSG00000240399.1	ENSG00000221990.4
24494	ENSG00000223042.1	ENSG00000240393.1	ENSG00000248668.2
24495	ENSG00000223044.1	ENSG00000240392.1	ENSG00000235172.7
24496	ENSG00000223046.1	ENSG00000240388.2	ENSG00000233902.1
24497	ENSG00000223047.1	ENSG00000240386.3	ENSG00000213316.9
24498	ENSG00000223056.1	ENSG00000240385.1	ENSG00000181023.7
24499	ENSG00000223060.1	ENSG00000240382.3	ENSG00000229422.2
24500	ENSG00000223062.1	ENSG00000240376.1	ENSG00000285486.1
24501	ENSG00000223064.1	ENSG00000240375.1	ENSG00000283208.2
24502	ENSG00000223075.1	ENSG00000240374.3	ENSG00000256280.1
24503	ENSG00000223076.1	ENSG00000240373.1	ENSG00000257808.1
24504	ENSG00000223078.1	ENSG00000240371.1	ENSG00000172748.14
24505	ENSG00000223080.1	ENSG00000240366.1	ENSG00000115525.18
24506	ENSG00000223086.1	ENSG00000240364.1	ENSG00000006016.11
24507	ENSG00000223087.2	ENSG00000240361.2	ENSG00000272282.1
24508	ENSG00000223088.1	ENSG00000240359.1	ENSG00000237399.7
24509	ENSG00000223092.1	ENSG00000240356.6	ENSG00000198954.8
24510	ENSG00000223096.1	ENSG00000240355.1	ENSG00000176209.11
24511	ENSG00000223107.1	ENSG00000240354.1	ENSG00000137270.11
24512	ENSG00000223109.1	ENSG00000240350.2	ENSG00000263528.8
24513	ENSG00000223113.1	ENSG00000240347.3	ENSG00000166352.16
24514	ENSG00000223117.1	ENSG00000240344.9	ENSG00000285864.1
24515	ENSG00000223118.1	ENSG00000240342.3	ENSG00000237135.1
24516	ENSG00000223120.1	ENSG00000240338.6	ENSG00000261436.5
24517	ENSG00000223125.1	ENSG00000240328.1	ENSG00000279873.2
24518	ENSG00000223126.1	ENSG00000240327.3	ENSG00000102931.8
24519	ENSG00000223128.1	ENSG00000240322.3	ENSG00000151240.17
24520	ENSG00000223131.1	ENSG00000240320.1	ENSG00000168917.9
24521	ENSG00000223136.1	ENSG00000240317.3	ENSG00000250733.5
24522	ENSG00000223138.1	ENSG00000240309.1	ENSG00000236148.5
24523	ENSG00000223142.1	ENSG00000240306.2	ENSG00000251079.6
24524	ENSG00000223145.1	ENSG00000240305.1	ENSG00000240531.1
24525	ENSG00000223152.1	ENSG00000240303.8	ENSG00000188649.14
24526	ENSG00000223156.1	ENSG00000240302.3	ENSG00000277246.1
24527	ENSG00000223158.1	ENSG00000240299.3	ENSG00000131697.18
24528	ENSG00000223162.1	ENSG00000240298.3	ENSG00000181982.18
24529	ENSG00000223168.1	ENSG00000240296.1	ENSG00000213949.9
24530	ENSG00000223169.1	ENSG00000240294.2	ENSG00000253506.2
24531	ENSG00000223174.1	ENSG00000240288.7	ENSG00000212743.2
24532	ENSG00000223175.1	ENSG00000240286.1	ENSG00000151012.13
24533	ENSG00000223177.1	ENSG00000240281.2	ENSG00000143450.16
24534	ENSG00000223179.3	ENSG00000240280.7	ENSG00000241852.10
24535	ENSG00000223181.1	ENSG00000240271.1	ENSG00000196914.9
24536	ENSG00000223188.1	ENSG00000240270.1	ENSG00000243175.1

24537	ENSG00000223189.1	ENSG00000240268.6	ENSG00000124399.4
24538	ENSG00000223190.1	ENSG00000240265.1	ENSG00000183598.3
24539	ENSG00000223191.1	ENSG00000240255.1	ENSG00000164880.16
24540	ENSG00000223197.1	ENSG00000240254.1	ENSG00000240366.1
24541	ENSG00000223198.1	ENSG00000240253.6	ENSG00000166359.10
24542	ENSG00000223202.1	ENSG00000240250.3	ENSG00000254503.1
24543	ENSG00000223203.1	ENSG00000240247.7	ENSG00000089009.15
24544	ENSG00000223208.1	ENSG00000240246.1	ENSG00000186364.12
24545	ENSG00000223212.1	ENSG00000240244.3	ENSG00000258810.1
24546	ENSG00000223215.1	ENSG00000240241.5	ENSG00000170160.17
24547	ENSG00000223217.1	ENSG00000240240.8	ENSG00000228137.1
24548	ENSG00000223220.1	ENSG00000240238.1	ENSG00000174720.16
24549	ENSG00000223223.1	ENSG00000240237.1	ENSG00000231290.5
24550	ENSG00000223224.1	ENSG00000240236.1	ENSG00000272129.1
24551	ENSG00000223225.1	ENSG00000240235.3	ENSG00000181541.5
24552	ENSG00000223229.1	ENSG00000240233.3	ENSG00000169564.6
24553	ENSG00000223238.1	ENSG00000240231.1	ENSG00000275939.1
24554	ENSG00000223245.1	ENSG00000240230.6	ENSG00000167131.17
24555	ENSG00000223247.1	ENSG00000240225.10	ENSG00000249264.1
24556	ENSG00000223254.1	ENSG00000240224.1	ENSG00000101856.10
24557	ENSG00000223256.1	ENSG00000240219.1	ENSG00000165269.13
24558	ENSG00000223258.1	ENSG00000240216.7	ENSG00000228979.4
24559	ENSG00000223259.1	ENSG00000240215.4	ENSG00000253868.3
24560	ENSG00000223260.1	ENSG00000240211.1	ENSG00000255746.1
24561	ENSG00000223262.1	ENSG00000240210.3	ENSG00000132205.11
24562	ENSG00000223263.1	ENSG00000240207.6	ENSG00000253234.1
24563	ENSG00000223265.1	ENSG00000240205.3	ENSG00000179886.5
24564	ENSG00000223269.1	ENSG00000240204.3	ENSG00000267414.1
24565	ENSG00000223271.1	ENSG00000240203.3	ENSG00000105427.10
24566	ENSG00000223273.1	ENSG00000240202.3	ENSG00000002834.18
24567	ENSG00000223274.6	ENSG00000240201.1	ENSG00000109339.22
24568	ENSG00000223274.6_PAR_Y	ENSG00000240199.3	ENSG00000161513.12
24569	ENSG00000223280.1	ENSG00000240198.5	ENSG00000275632.1
24570	ENSG00000223281.1	ENSG00000240197.1	ENSG00000185905.4
24571	ENSG00000223282.1	ENSG00000240194.7	ENSG00000249014.2
24572	ENSG00000223284.1	ENSG00000240189.3	ENSG00000229598.1
24573	ENSG00000223287.1	ENSG00000240186.3	ENSG00000259933.6
24574	ENSG00000223290.1	ENSG00000240184.7	ENSG00000174928.16
24575	ENSG00000223293.1	ENSG00000240183.3	ENSG00000237080.3
24576	ENSG00000223294.1	ENSG00000240180.1	ENSG00000111716.12
24577	ENSG00000223298.1	ENSG00000240179.1	ENSG00000240751.1
24578	ENSG00000223299.1	ENSG00000240175.1	ENSG00000130772.14
24579	ENSG00000223300.1	ENSG00000240174.1	ENSG00000120314.18
24580	ENSG00000223302.1	ENSG00000240173.3	ENSG00000138785.15
24581	ENSG00000223305.1	ENSG00000240167.1	ENSG00000250040.2
24582	ENSG00000223306.2	ENSG00000240163.1	ENSG00000100336.17
24583	ENSG00000223308.1	ENSG00000240160.3	ENSG00000263826.1
24584	ENSG00000223309.1	ENSG00000240159.1	ENSG00000177406.4
24585	ENSG00000223313.1	ENSG00000240156.1	ENSG00000110011.13
24586	ENSG00000223315.1	ENSG00000240152.2	ENSG00000251495.1
24587	ENSG00000223318.1	ENSG00000240151.3	ENSG00000169957.10
24588	ENSG00000223321.1	ENSG00000240143.1	ENSG00000149269.9
24589	ENSG00000223324.1	ENSG00000240138.1	ENSG00000197063.11

24590	ENSG00000223327.1	ENSG00000240135.1	ENSG00000167601.12
24591	ENSG00000223330.1	ENSG00000240132.1	ENSG00000112218.9
24592	ENSG00000223335.1	ENSG00000240131.1	ENSG00000273987.1
24593	ENSG00000223336.1	ENSG00000240128.1	ENSG00000270108.1
24594	ENSG00000223341.1	ENSG00000240125.1	ENSG00000272009.1
24595	ENSG00000223342.2	ENSG00000240122.1	ENSG00000115257.15
24596	ENSG00000223343.1	ENSG00000240121.1	ENSG00000214837.8
24597	ENSG00000223344.1	ENSG00000240116.3	ENSG00000211668.2
24598	ENSG00000223345.3	ENSG00000240108.1	ENSG00000120733.14
24599	ENSG00000223349.1	ENSG00000240107.1	ENSG00000255439.6
24600	ENSG00000223350.2	ENSG00000240106.2	ENSG00000283031.1
24601	ENSG00000223351.5	ENSG00000240103.2	ENSG00000235686.1
24602	ENSG00000223353.2	ENSG00000240100.1	ENSG00000245498.6
24603	ENSG00000223356.1	ENSG00000240098.3	ENSG00000148225.16
24604	ENSG00000223358.5	ENSG00000240097.1	ENSG00000106003.13
24605	ENSG00000223360.1	ENSG00000240096.1	ENSG00000128915.12
24606	ENSG00000223361.5	ENSG00000240095.1	ENSG00000136114.17
24607	ENSG00000223362.1	ENSG00000240093.1	ENSG00000171867.16
24608	ENSG00000223368.2	ENSG00000240089.2	ENSG00000174227.16
24609	ENSG00000223373.1	ENSG00000240087.3	ENSG00000109790.16
24610	ENSG00000223374.1	ENSG00000240086.6	ENSG00000253738.1
24611	ENSG00000223375.1	ENSG00000240084.1	ENSG00000038945.15
24612	ENSG00000223377.1	ENSG00000240083.2	ENSG00000259326.1
24613	ENSG00000223381.1	ENSG00000240074.1	ENSG00000265260.2
24614	ENSG00000223382.4	ENSG00000240069.1	ENSG00000064999.15
24615	ENSG00000223383.1	ENSG00000240068.1	ENSG00000168876.9
24616	ENSG00000223387.6	ENSG00000240065.8	ENSG00000081138.14
24617	ENSG00000223389.1	ENSG00000240063.1	ENSG00000175287.19
24618	ENSG00000223390.1	ENSG00000240058.3	ENSG00000174307.6
24619	ENSG00000223391.2	ENSG00000240057.5	ENSG00000251062.1
24620	ENSG00000223392.1	ENSG00000240056.2	ENSG00000178607.16
24621	ENSG00000223393.1	ENSG00000240053.8	ENSG00000243417.1
24622	ENSG00000223394.2	ENSG00000240052.1	ENSG00000110395.6
24623	ENSG00000223395.2	ENSG00000240051.1	ENSG00000279035.1
24624	ENSG00000223396.4	ENSG00000240048.1	ENSG00000225148.1
24625	ENSG00000223398.1	ENSG00000240047.2	ENSG00000259295.6
24626	ENSG00000223400.1	ENSG00000240045.2	ENSG00000285996.1
24627	ENSG00000223402.1	ENSG00000240043.1	ENSG00000229638.1
24628	ENSG00000223403.4	ENSG00000240041.1	ENSG00000263941.2
24629	ENSG00000223404.3	ENSG00000240040.6	ENSG00000137842.7
24630	ENSG00000223406.1	ENSG00000240038.6	ENSG00000163735.7
24631	ENSG00000223407.1	ENSG00000240036.4	ENSG00000172367.16
24632	ENSG00000223409.1	ENSG00000240034.1	ENSG00000160877.6
24633	ENSG00000223410.1	ENSG00000240033.1	ENSG00000164713.9
24634	ENSG00000223416.3	ENSG00000240032.1	ENSG00000015568.13
24635	ENSG00000223417.8	ENSG00000240031.1	ENSG00000213516.10
24636	ENSG00000223418.1	ENSG00000240027.2	ENSG00000227598.1
24637	ENSG00000223419.1	ENSG00000240024.5	ENSG00000260467.1
24638	ENSG00000223421.1	ENSG00000240023.1	ENSG00000124343.13
24639	ENSG00000223422.1	ENSG00000240021.10	ENSG00000197959.14
24640	ENSG00000223427.1	ENSG00000240015.2	ENSG00000246851.1
24641	ENSG00000223428.1	ENSG00000240014.3	ENSG00000257379.1
24642	ENSG00000223429.1	ENSG00000240012.1	ENSG00000169583.12

24643	ENSG00000223430.1	ENSG00000240006.1	ENSG00000185222.10
24644	ENSG00000223431.1	ENSG00000240005.5	ENSG00000172594.13
24645	ENSG00000223432.1	ENSG00000240003.2	ENSG00000117009.12
24646	ENSG00000223433.1	ENSG00000240002.1	ENSG00000248322.1
24647	ENSG00000223436.1	ENSG00000239998.6	ENSG00000234383.1
24648	ENSG00000223437.1	ENSG00000239997.1	ENSG00000146677.7
24649	ENSG00000223438.1	ENSG00000239995.2	ENSG00000174977.8
24650	ENSG00000223440.1	ENSG00000239994.2	ENSG00000258472.8
24651	ENSG00000223442.1	ENSG00000239992.1	ENSG00000249846.6
24652	ENSG00000223443.2	ENSG00000239991.1	ENSG00000101336.14
24653	ENSG00000223445.1	ENSG00000239989.1	ENSG00000261369.1
24654	ENSG00000223446.1	ENSG00000239988.1	ENSG00000173020.11
24655	ENSG00000223450.1	ENSG00000239986.2	ENSG00000167113.11
24656	ENSG00000223452.3	ENSG00000239985.2	ENSG00000182983.14
24657	ENSG00000223455.1	ENSG00000239984.3	ENSG00000274414.1
24658	ENSG00000223457.1	ENSG00000239983.1	ENSG00000128011.4
24659	ENSG00000223458.2	ENSG00000239981.1	ENSG00000140263.14
24660	ENSG00000223459.6	ENSG00000239978.1	ENSG00000256826.1
24661	ENSG00000223460.1	ENSG00000239969.5	ENSG00000115216.14
24662	ENSG00000223461.1	ENSG00000239967.1	ENSG00000188060.7
24663	ENSG00000223462.2	ENSG00000239964.3	ENSG00000269955.2
24664	ENSG00000223466.1	ENSG00000239961.3	ENSG00000240291.1
24665	ENSG00000223467.2	ENSG00000239959.1	ENSG00000113360.16
24666	ENSG00000223469.1	ENSG00000239958.3	ENSG00000269983.1
24667	ENSG00000223470.3	ENSG00000239953.3	ENSG00000128604.20
24668	ENSG00000223475.1	ENSG00000239951.1	ENSG00000182173.13
24669	ENSG00000223476.1	ENSG00000239948.2	ENSG00000112855.16
24670	ENSG00000223478.1	ENSG00000239946.1	ENSG00000275936.1
24671	ENSG00000223479.3	ENSG00000239945.1	ENSG00000196150.13
24672	ENSG00000223482.7	ENSG00000239944.1	ENSG00000233670.7
24673	ENSG00000223484.7	ENSG00000239942.2	ENSG00000253570.1
24674	ENSG00000223484.7_PAR_Y	ENSG00000239941.1	ENSG00000153989.8
24675	ENSG00000223485.3	ENSG00000239939.1	ENSG00000264937.1
24676	ENSG00000223486.1	ENSG00000239932.3	ENSG00000228097.1
24677	ENSG00000223487.1	ENSG00000239930.2	ENSG00000176022.6
24678	ENSG00000223488.2	ENSG00000239926.1	ENSG00000123975.5
24679	ENSG00000223489.1	ENSG00000239924.1	ENSG00000233654.1
24680	ENSG00000223490.2	ENSG00000239923.3	ENSG00000007866.21
24681	ENSG00000223492.1	ENSG00000239922.1	ENSG00000160200.17
24682	ENSG00000223495.2	ENSG00000239921.3	ENSG00000267545.1
24683	ENSG00000223496.3	ENSG00000239920.2	ENSG00000185298.13
24684	ENSG00000223497.1	ENSG00000239919.1	ENSG00000259488.2
24685	ENSG00000223498.1	ENSG00000239917.3	ENSG00000226708.1
24686	ENSG00000223500.1	ENSG00000239912.1	ENSG00000262655.4
24687	ENSG00000223501.9	ENSG00000239911.2	ENSG00000259664.2
24688	ENSG00000223502.1	ENSG00000239910.3	ENSG00000252147.1
24689	ENSG00000223503.1	ENSG00000239908.3	ENSG00000135480.16
24690	ENSG00000223504.1	ENSG00000239906.1	ENSG00000089876.11
24691	ENSG00000223505.2	ENSG00000239900.12	ENSG00000283398.1
24692	ENSG00000223506.2	ENSG00000239899.3	ENSG00000163161.13
24693	ENSG00000223508.5	ENSG00000239893.2	ENSG00000263004.1
24694	ENSG00000223509.8	ENSG00000239888.3	ENSG00000176658.17
24695	ENSG00000223510.6	ENSG00000239887.5	ENSG00000110925.6

24696	ENSG00000223511.7	ENSG00000239886.5	ENSG00000279433.1
24697	ENSG00000223511.7_PAR_Y	ENSG00000239884.3	ENSG00000078124.12
24698	ENSG00000223513.1	ENSG00000239883.8	ENSG00000206567.9
24699	ENSG00000223514.1	ENSG00000239881.1	ENSG00000101945.16
24700	ENSG00000223516.1	ENSG00000239880.1	ENSG00000232306.2
24701	ENSG00000223517.1	ENSG00000239877.2	ENSG00000283549.1
24702	ENSG00000223518.5	ENSG00000239873.2	ENSG00000225630.1
24703	ENSG00000223519.8	ENSG00000239872.1	ENSG00000263843.1
24704	ENSG00000223522.1	ENSG00000239870.1	ENSG00000215483.10
24705	ENSG00000223523.1	ENSG00000239862.1	ENSG00000272688.1
24706	ENSG00000223525.1	ENSG00000239861.1	ENSG00000258210.1
24707	ENSG00000223528.7	ENSG00000239857.7	ENSG00000180257.13
24708	ENSG00000223529.1	ENSG00000239856.3	ENSG00000182272.12
24709	ENSG00000223530.1	ENSG00000239855.1	ENSG00000254413.8
24710	ENSG00000223534.1	ENSG00000239840.1	ENSG00000266289.1
24711	ENSG00000223536.5	ENSG00000239839.7	ENSG00000220793.5
24712	ENSG00000223537.2	ENSG00000239835.1	ENSG00000234211.2
24713	ENSG00000223538.2	ENSG00000239831.1	ENSG00000113569.16
24714	ENSG00000223539.4	ENSG00000239830.1	ENSG00000235413.3
24715	ENSG00000223540.1	ENSG00000239829.1	ENSG00000260447.1
24716	ENSG00000223542.1	ENSG00000239828.6	ENSG00000277363.5
24717	ENSG00000223543.1	ENSG00000239827.8	ENSG00000117983.17
24718	ENSG00000223544.1	ENSG00000239825.3	ENSG00000277194.1
24719	ENSG00000223546.6	ENSG00000239823.2	ENSG00000196418.12
24720	ENSG00000223547.10	ENSG00000239822.3	ENSG00000285090.1
24721	ENSG00000223548.1	ENSG00000239821.3	ENSG00000258515.1
24722	ENSG00000223549.1	ENSG00000239820.3	ENSG00000274836.1
24723	ENSG00000223550.1	ENSG00000239819.2	ENSG00000231010.1
24724	ENSG00000223551.1	ENSG00000239810.3	ENSG00000198855.7
24725	ENSG00000223552.1	ENSG00000239809.1	ENSG00000236018.2
24726	ENSG00000223553.6	ENSG00000239808.3	ENSG00000170464.10
24727	ENSG00000223554.1	ENSG00000239805.1	ENSG00000279619.1
24728	ENSG00000223555.1	ENSG00000239804.1	ENSG00000026036.22
24729	ENSG00000223558.1	ENSG00000239801.1	ENSG00000256581.1
24730	ENSG00000223559.1	ENSG00000239799.1	ENSG00000279202.1
24731	ENSG00000223561.6	ENSG00000239797.1	ENSG00000277072.5
24732	ENSG00000223563.1	ENSG00000239794.3	ENSG00000236991.6
24733	ENSG00000223564.1	ENSG00000239793.1	ENSG00000049541.11
24734	ENSG00000223565.1	ENSG00000239792.2	ENSG00000100811.13
24735	ENSG00000223566.1	ENSG00000239791.1	ENSG00000225217.1
24736	ENSG00000223568.1	ENSG00000239789.6	ENSG00000276445.1
24737	ENSG00000223569.6	ENSG00000239783.1	ENSG00000203668.2
24738	ENSG00000223570.2	ENSG00000239780.1	ENSG00000228327.3
24739	ENSG00000223571.6	ENSG00000239779.7	ENSG00000273110.1
24740	ENSG00000223571.6_PAR_Y	ENSG00000239775.1	ENSG00000171763.19
24741	ENSG00000223572.9	ENSG00000239774.1	ENSG00000266728.5
24742	ENSG00000223573.7	ENSG00000239767.1	ENSG00000232869.2
24743	ENSG00000223574.1	ENSG00000239763.2	ENSG00000102401.20
24744	ENSG00000223575.2	ENSG00000239748.3	ENSG00000134444.14
24745	ENSG00000223576.2	ENSG00000239745.3	ENSG00000127589.4
24746	ENSG00000223581.1	ENSG00000239744.3	ENSG00000169131.13
24747	ENSG00000223583.1	ENSG00000239742.3	ENSG00000215712.10
24748	ENSG00000223584.1	ENSG00000239739.1	ENSG00000250317.8

24749	ENSG00000223586.5	ENSG00000239736.2	ENSG00000213563.6
24750	ENSG00000223587.1	ENSG00000239732.3	ENSG00000165355.7
24751	ENSG00000223589.2	ENSG00000239731.3	ENSG00000254470.2
24752	ENSG00000223591.5	ENSG00000239726.3	ENSG00000259158.4
24753	ENSG00000223592.2	ENSG00000239722.1	ENSG00000257386.1
24754	ENSG00000223593.1	ENSG00000239719.1	ENSG00000281181.1
24755	ENSG00000223595.1	ENSG00000239718.1	ENSG00000140465.14
24756	ENSG00000223597.2	ENSG00000239716.1	ENSG00000249858.2
24757	ENSG00000223598.1	ENSG00000239715.1	ENSG00000253636.1
24758	ENSG00000223599.1	ENSG00000239710.3	ENSG00000268069.2
24759	ENSG00000223600.1	ENSG00000239708.3	ENSG00000257277.1
24760	ENSG00000223601.2	ENSG00000239706.1	ENSG00000186951.16
24761	ENSG00000223602.2	ENSG00000239705.1	ENSG00000236540.7
24762	ENSG00000223603.1	ENSG00000239704.10	ENSG00000166272.18
24763	ENSG00000223604.1	ENSG00000239703.3	ENSG00000163106.10
24764	ENSG00000223605.1	ENSG00000239702.3	ENSG00000265664.1
24765	ENSG00000223608.1	ENSG00000239701.1	ENSG00000260179.1
24766	ENSG00000223609.10	ENSG00000239699.1	ENSG00000116641.17
24767	ENSG00000223611.5	ENSG00000239697.11	ENSG00000231062.1
24768	ENSG00000223612.3	ENSG00000239696.3	ENSG00000239775.1
24769	ENSG00000223614.5	ENSG00000239694.1	ENSG00000184584.13
24770	ENSG00000223615.2	ENSG00000239690.3	ENSG00000231703.2
24771	ENSG00000223617.2	ENSG00000239689.1	ENSG00000103769.10
24772	ENSG00000223619.1	ENSG00000239686.1	ENSG00000180138.7
24773	ENSG00000223620.3	ENSG00000239684.2	ENSG00000132329.11
24774	ENSG00000223621.1	ENSG00000239683.1	ENSG00000155254.13
24775	ENSG00000223622.2	ENSG00000239679.3	ENSG00000226784.2
24776	ENSG00000223623.1	ENSG00000239677.6	ENSG00000280852.2
24777	ENSG00000223624.1	ENSG00000239674.2	ENSG00000109771.15
24778	ENSG00000223625.1	ENSG00000239672.7	ENSG00000154822.18
24779	ENSG00000223626.1	ENSG00000239671.1	ENSG00000279149.1
24780	ENSG00000223628.2	ENSG00000239670.1	ENSG00000269737.2
24781	ENSG00000223629.1	ENSG00000239665.8	ENSG00000111266.8
24782	ENSG00000223631.1	ENSG00000239661.1	ENSG00000236035.2
24783	ENSG00000223634.1	ENSG00000239659.2	ENSG00000227740.1
24784	ENSG00000223635.1	ENSG00000239653.1	ENSG00000100599.16
24785	ENSG00000223636.1	ENSG00000239650.4	ENSG00000187824.8
24786	ENSG00000223637.7	ENSG00000239649.3	ENSG00000270011.7
24787	ENSG00000223638.3	ENSG00000239648.1	ENSG00000204308.8
24788	ENSG00000223640.1	ENSG00000239642.6	ENSG00000248586.2
24789	ENSG00000223641.1	ENSG00000239641.1	ENSG00000255562.2
24790	ENSG00000223642.7	ENSG00000239640.3	ENSG00000082014.16
24791	ENSG00000223643.6	ENSG00000239636.1	ENSG00000237846.1
24792	ENSG00000223646.1	ENSG00000239632.1	ENSG00000143816.8
24793	ENSG00000223647.1	ENSG00000239628.1	ENSG00000127580.17
24794	ENSG00000223648.4	ENSG00000239627.3	ENSG00000267272.5
24795	ENSG00000223649.1	ENSG00000239626.1	ENSG00000221944.7
24796	ENSG00000223650.1	ENSG00000239625.3	ENSG00000162543.6
24797	ENSG00000223651.1	ENSG00000239622.1	ENSG00000108094.14
24798	ENSG00000223652.2	ENSG00000239620.2	ENSG00000103126.15
24799	ENSG00000223653.5	ENSG00000239617.1	ENSG00000279673.1
24800	ENSG00000223655.1	ENSG00000239615.1	ENSG00000108700.5
24801	ENSG00000223657.2	ENSG00000239614.1	ENSG00000120832.10

24802	ENSG00000223658.8	ENSG00000239608.1	ENSG00000152377.14
24803	ENSG00000223660.1	ENSG00000239607.3	ENSG00000196405.13
24804	ENSG00000223662.1	ENSG00000239602.1	ENSG00000197345.13
24805	ENSG00000223663.2	ENSG00000239600.1	ENSG00000207110.1
24806	ENSG00000223665.1	ENSG00000239595.3	ENSG00000168487.19
24807	ENSG00000223668.1	ENSG00000239593.1	ENSG00000266100.1
24808	ENSG00000223669.1	ENSG00000239590.1	ENSG00000124191.18
24809	ENSG00000223671.2	ENSG00000239589.6	ENSG00000083168.11
24810	ENSG00000223672.2	ENSG00000239586.1	ENSG00000224796.1
24811	ENSG00000223675.1	ENSG00000239580.3	ENSG00000236088.9
24812	ENSG00000223676.1	ENSG00000239579.3	ENSG00000123405.14
24813	ENSG00000223677.1	ENSG00000239577.3	ENSG00000256007.1
24814	ENSG00000223678.1	ENSG00000239576.1	ENSG00000259652.1
24815	ENSG00000223679.1	ENSG00000239572.2	ENSG00000248221.1
24816	ENSG00000223683.1	ENSG00000239571.1	ENSG00000107104.18
24817	ENSG00000223684.1	ENSG00000239570.1	ENSG00000103174.13
24818	ENSG00000223685.5	ENSG00000239569.3	ENSG00000258674.5
24819	ENSG00000223688.2	ENSG00000239568.1	ENSG00000275342.4
24820	ENSG00000223691.1	ENSG00000239560.3	ENSG00000144642.21
24821	ENSG00000223692.1	ENSG00000239559.2	ENSG00000106683.15
24822	ENSG00000223694.1	ENSG00000239557.1	ENSG00000232555.1
24823	ENSG00000223695.1	ENSG00000239556.4	ENSG00000246084.2
24824	ENSG00000223697.3	ENSG00000239555.3	ENSG00000166507.17
24825	ENSG00000223698.3	ENSG00000239553.3	ENSG00000136240.10
24826	ENSG00000223701.3	ENSG00000239552.2	ENSG00000279882.1
24827	ENSG00000223702.1	ENSG00000239547.3	ENSG00000273341.1
24828	ENSG00000223703.1	ENSG00000239545.3	ENSG00000133111.3
24829	ENSG00000223704.1	ENSG00000239544.1	ENSG00000123892.12
24830	ENSG00000223705.9	ENSG00000239542.3	ENSG00000285665.1
24831	ENSG00000223707.2	ENSG00000239539.1	ENSG00000173295.7
24832	ENSG00000223709.6	ENSG00000239533.6	ENSG00000196636.7
24833	ENSG00000223710.1	ENSG00000239532.1	ENSG00000261693.1
24834	ENSG00000223711.1	ENSG00000239527.1	ENSG00000244161.1
24835	ENSG00000223714.1	ENSG00000239525.1	ENSG00000223947.1
24836	ENSG00000223715.2	ENSG00000239524.2	ENSG00000139926.15
24837	ENSG00000223716.2	ENSG00000239523.5	ENSG00000237903.1
24838	ENSG00000223717.1	ENSG00000239519.1	ENSG00000154548.9
24839	ENSG00000223718.3	ENSG00000239517.1	ENSG00000255363.2
24840	ENSG00000223719.1	ENSG00000239516.1	ENSG00000272223.1
24841	ENSG00000223721.3	ENSG00000239513.5	ENSG00000228801.5
24842	ENSG00000223722.3	ENSG00000239511.2	ENSG00000188089.13
24843	ENSG00000223723.1	ENSG00000239510.2	ENSG00000063515.2
24844	ENSG00000223724.1	ENSG00000239508.1	ENSG00000188338.15
24845	ENSG00000223725.6	ENSG00000239504.3	ENSG00000176909.12
24846	ENSG00000223726.1	ENSG00000239503.1	ENSG00000102901.13
24847	ENSG00000223727.6	ENSG00000239498.1	ENSG00000259349.1
24848	ENSG00000223728.3	ENSG00000239494.2	ENSG00000185960.14
24849	ENSG00000223729.1	ENSG00000239490.1	ENSG00000110906.13
24850	ENSG00000223730.1	ENSG00000239486.1	ENSG00000145220.14
24851	ENSG00000223731.2	ENSG00000239483.1	ENSG00000244682.7
24852	ENSG00000223732.2	ENSG00000239482.6	ENSG00000171469.11
24853	ENSG00000223733.1	ENSG00000239481.1	ENSG00000268744.1
24854	ENSG00000223734.2	ENSG00000239480.1	ENSG00000169184.6

24855	ENSG00000223735.1	ENSG00000239474.7	ENSG00000217325.2
24856	ENSG00000223738.3	ENSG00000239473.1	ENSG00000246451.2
24857	ENSG00000223739.1	ENSG00000239472.3	ENSG00000068001.14
24858	ENSG00000223740.1	ENSG00000239471.3	ENSG00000231131.7
24859	ENSG00000223741.1	ENSG00000239468.3	ENSG00000271631.1
24860	ENSG00000223742.1	ENSG00000239467.5	ENSG00000280054.1
24861	ENSG00000223744.1	ENSG00000239466.3	ENSG00000271551.2
24862	ENSG00000223745.7	ENSG00000239465.1	ENSG00000250189.2
24863	ENSG00000223746.1	ENSG00000239464.3	ENSG00000227934.1
24864	ENSG00000223749.9	ENSG00000239462.1	ENSG00000055332.18
24865	ENSG00000223750.1	ENSG00000239455.1	ENSG00000272267.2
24866	ENSG00000223751.1	ENSG00000239454.1	ENSG00000241566.1
24867	ENSG00000223753.2	ENSG00000239453.1	ENSG00000285667.1
24868	ENSG00000223754.1	ENSG00000239446.1	ENSG00000243547.1
24869	ENSG00000223756.6	ENSG00000239445.5	ENSG00000247372.2
24870	ENSG00000223760.4	ENSG00000239443.1	ENSG00000162738.6
24871	ENSG00000223761.1	ENSG00000239440.5	ENSG00000109220.11
24872	ENSG00000223764.2	ENSG00000239439.1	ENSG00000170899.11
24873	ENSG00000223765.2	ENSG00000239438.1	ENSG00000262165.2
24874	ENSG00000223768.2	ENSG00000239437.3	ENSG00000160055.19
24875	ENSG00000223770.5	ENSG00000239435.2	ENSG00000260097.2
24876	ENSG00000223772.1	ENSG00000239432.1	ENSG00000235236.1
24877	ENSG00000223773.7	ENSG00000239428.2	ENSG00000250536.1
24878	ENSG00000223773.7_PAR_Y	ENSG00000239426.4	ENSG00000089220.5
24879	ENSG00000223774.5	ENSG00000239419.3	ENSG00000221930.6
24880	ENSG00000223776.5	ENSG00000239415.1	ENSG00000163694.15
24881	ENSG00000223777.1	ENSG00000239413.1	ENSG00000062194.16
24882	ENSG00000223779.6	ENSG00000239412.1	ENSG00000273724.1
24883	ENSG00000223782.1	ENSG00000239408.1	ENSG00000145700.9
24884	ENSG00000223783.1	ENSG00000239407.5	ENSG00000114631.11
24885	ENSG00000223784.2	ENSG00000239405.1	ENSG00000259922.1
24886	ENSG00000223786.1	ENSG00000239402.3	ENSG00000134287.10
24887	ENSG00000223787.2	ENSG00000239398.3	ENSG00000126787.13
24888	ENSG00000223788.4	ENSG00000239397.1	ENSG00000082269.16
24889	ENSG00000223791.1	ENSG00000239396.3	ENSG00000273243.1
24890	ENSG00000223794.1	ENSG00000239395.2	ENSG00000012174.12
24891	ENSG00000223795.2	ENSG00000239393.1	ENSG00000173705.9
24892	ENSG00000223797.5	ENSG00000239392.2	ENSG00000185787.14
24893	ENSG00000223799.1	ENSG00000239390.3	ENSG00000182795.13
24894	ENSG00000223800.1	ENSG00000239389.8	ENSG00000283020.1
24895	ENSG00000223802.7	ENSG00000239388.8	ENSG00000250182.3
24896	ENSG00000223803.1	ENSG00000239383.1	ENSG00000033100.16
24897	ENSG00000223804.5	ENSG00000239382.10	ENSG00000232366.1
24898	ENSG00000223806.7	ENSG00000239381.6	ENSG00000102978.13
24899	ENSG00000223807.1	ENSG00000239377.1	ENSG00000259683.1
24900	ENSG00000223808.1	ENSG00000239374.1	ENSG00000279166.1
24901	ENSG00000223809.1	ENSG00000239367.2	ENSG00000223274.6
24902	ENSG00000223810.2	ENSG00000239365.2	ENSG00000130703.16
24903	ENSG00000223811.1	ENSG00000239356.3	ENSG00000164056.11
24904	ENSG00000223812.7	ENSG00000239354.1	ENSG00000153060.7
24905	ENSG00000223813.2	ENSG00000239351.1	ENSG00000275198.1
24906	ENSG00000223814.1	ENSG00000239350.2	ENSG00000235869.1
24907	ENSG00000223815.1	ENSG00000239345.2	ENSG00000153391.15

24908	ENSG00000223816.5	ENSG00000239344.1	ENSG00000241627.3
24909	ENSG00000223817.1	ENSG00000239333.3	ENSG00000172403.11
24910	ENSG00000223820.5	ENSG00000239332.5	ENSG00000254402.7
24911	ENSG00000223821.1	ENSG00000239327.1	ENSG00000106927.12
24912	ENSG00000223822.2	ENSG00000239323.1	ENSG00000214176.9
24913	ENSG00000223823.1	ENSG00000239322.1	ENSG00000198754.5
24914	ENSG00000223824.1	ENSG00000239320.1	ENSG00000105983.21
24915	ENSG00000223825.5	ENSG00000239319.3	ENSG00000092871.16
24916	ENSG00000223826.1	ENSG00000239317.1	ENSG00000135638.13
24917	ENSG00000223828.1	ENSG00000239316.3	ENSG00000258818.4
24918	ENSG00000223829.5	ENSG00000239315.4	ENSG00000159086.15
24919	ENSG00000223834.3	ENSG00000239314.1	ENSG00000105507.2
24920	ENSG00000223836.1	ENSG00000239311.1	ENSG00000148143.13
24921	ENSG00000223837.2	ENSG00000239306.4	ENSG00000014914.21
24922	ENSG00000223838.1	ENSG00000239305.7	ENSG00000278993.1
24923	ENSG00000223839.7	ENSG00000239304.1	ENSG00000237372.3
24924	ENSG00000223841.1	ENSG00000239300.5	ENSG00000114904.13
24925	ENSG00000223842.1	ENSG00000239293.1	ENSG00000276101.1
24926	ENSG00000223843.4	ENSG00000239291.1	ENSG00000249863.2
24927	ENSG00000223845.1	ENSG00000239288.1	ENSG00000268945.1
24928	ENSG00000223847.1	ENSG00000239281.2	ENSG00000172508.10
24929	ENSG00000223849.1	ENSG00000239280.1	ENSG00000196639.6
24930	ENSG00000223850.1	ENSG00000239279.3	ENSG00000110077.14
24931	ENSG00000223855.1	ENSG00000239272.1	ENSG00000271032.1
24932	ENSG00000223856.1	ENSG00000239269.1	ENSG00000275560.1
24933	ENSG00000223859.1	ENSG00000239268.2	ENSG00000229503.1
24934	ENSG00000223861.1	ENSG00000239265.5	ENSG00000128891.15
24935	ENSG00000223863.1	ENSG00000239264.8	ENSG00000164764.11
24936	ENSG00000223864.1	ENSG00000239263.1	ENSG00000283580.3
24937	ENSG00000223865.11	ENSG00000239261.1	ENSG00000206737.1
24938	ENSG00000223866.1	ENSG00000239257.1	ENSG00000244245.1
24939	ENSG00000223867.1	ENSG00000239256.1	ENSG00000169714.16
24940	ENSG00000223869.1	ENSG00000239255.1	ENSG00000227463.2
24941	ENSG00000223870.1	ENSG00000239254.1	ENSG00000197885.10
24942	ENSG00000223872.1	ENSG00000239253.1	ENSG00000133477.17
24943	ENSG00000223873.1	ENSG00000239250.3	ENSG00000228929.1
24944	ENSG00000223874.1	ENSG00000239249.3	ENSG00000139436.21
24945	ENSG00000223875.2	ENSG00000239247.3	ENSG00000278916.1
24946	ENSG00000223876.1	ENSG00000239246.3	ENSG00000279430.1
24947	ENSG00000223877.4	ENSG00000239238.1	ENSG00000273254.1
24948	ENSG00000223878.1	ENSG00000239228.3	ENSG00000271361.1
24949	ENSG00000223880.1	ENSG00000239227.1	ENSG00000254577.1
24950	ENSG00000223881.1	ENSG00000239226.1	ENSG00000230091.6
24951	ENSG00000223882.1	ENSG00000239225.1	ENSG00000250835.1
24952	ENSG00000223883.1	ENSG00000239224.3	ENSG00000110492.15
24953	ENSG00000223884.6	ENSG00000239223.3	ENSG00000182118.8
24954	ENSG00000223885.4	ENSG00000239221.3	ENSG00000231307.1
24955	ENSG00000223886.3	ENSG00000239219.2	ENSG00000214174.8
24956	ENSG00000223889.1	ENSG00000239218.2	ENSG00000119559.16
24957	ENSG00000223890.1	ENSG00000239216.1	ENSG00000267612.1
24958	ENSG00000223891.5	ENSG00000239215.1	ENSG00000270923.1
24959	ENSG00000223893.1	ENSG00000239213.5	ENSG00000156876.10
24960	ENSG00000223896.2	ENSG00000239212.2	ENSG00000263069.5

24961	ENSG00000223897.1	ENSG00000239211.3	ENSG00000176884.15
24962	ENSG00000223899.1	ENSG00000239210.2	ENSG00000260879.1
24963	ENSG00000223901.2	ENSG00000239207.2	ENSG00000188419.14
24964	ENSG00000223903.1	ENSG00000239205.1	ENSG00000282278.1
24965	ENSG00000223904.2	ENSG00000239202.3	ENSG00000231841.1
24966	ENSG00000223905.1	ENSG00000239201.1	ENSG00000178202.13
24967	ENSG00000223906.1	ENSG00000239200.1	ENSG00000235150.1
24968	ENSG00000223908.5	ENSG00000239199.1	ENSG00000090382.6
24969	ENSG00000223910.1	ENSG00000239198.1	ENSG00000204574.13
24970	ENSG00000223911.1	ENSG00000239197.1	ENSG00000225215.1
24971	ENSG00000223912.1	ENSG00000239195.1	ENSG00000234335.1
24972	ENSG00000223914.2	ENSG00000239194.1	ENSG00000167371.20
24973	ENSG00000223915.1	ENSG00000239190.1	ENSG00000284830.1
24974	ENSG00000223916.1	ENSG00000239189.1	ENSG00000141161.11
24975	ENSG00000223917.1	ENSG00000239185.1	ENSG00000163510.14
24976	ENSG00000223918.1	ENSG00000239184.1	ENSG00000285651.1
24977	ENSG00000223920.2	ENSG00000239183.1	ENSG00000256966.6
24978	ENSG00000223921.1	ENSG00000239182.1	ENSG00000277678.1
24979	ENSG00000223922.1	ENSG00000239180.1	ENSG00000250444.1
24980	ENSG00000223923.1	ENSG00000239175.1	ENSG00000174943.11
24981	ENSG00000223928.1	ENSG00000239169.1	ENSG00000093217.10
24982	ENSG00000223930.6	ENSG00000239168.1	ENSG00000109814.12
24983	ENSG00000223931.2	ENSG00000239153.1	ENSG00000267512.1
24984	ENSG00000223935.2	ENSG00000239152.1	ENSG00000196810.4
24985	ENSG00000223940.1	ENSG00000239151.1	ENSG00000153904.20
24986	ENSG00000223941.5	ENSG00000239149.1	ENSG00000113494.17
24987	ENSG00000223942.1	ENSG00000239148.1	ENSG00000135253.13
24988	ENSG00000223944.1	ENSG00000239143.1	ENSG00000158859.10
24989	ENSG00000223945.2	ENSG00000239142.1	ENSG00000125676.20
24990	ENSG00000223946.1	ENSG00000239137.1	ENSG00000260176.1
24991	ENSG00000223947.1	ENSG00000239129.1	ENSG00000138622.4
24992	ENSG00000223948.1	ENSG00000239128.1	ENSG00000272398.6
24993	ENSG00000223949.6	ENSG00000239127.1	ENSG00000233457.1
24994	ENSG00000223951.1	ENSG00000239122.1	ENSG00000255397.1
24995	ENSG00000223953.6	ENSG00000239119.1	ENSG00000146072.6
24996	ENSG00000223955.1	ENSG00000239118.1	ENSG00000247287.2
24997	ENSG00000223956.2	ENSG00000239115.1	ENSG00000088538.13
24998	ENSG00000223958.1	ENSG00000239112.1	ENSG00000225693.1
24999	ENSG00000223959.8	ENSG00000239108.1	ENSG00000230638.4
25000	ENSG00000223960.6	ENSG00000239106.1	ENSG00000154174.7
25001	ENSG00000223962.1	ENSG00000239105.1	ENSG00000220614.1
25002	ENSG00000223965.2	ENSG00000239102.1	ENSG00000272645.3
25003	ENSG00000223966.1	ENSG00000239099.1	ENSG00000111684.11
25004	ENSG00000223967.1	ENSG00000239096.1	ENSG00000138138.13
25005	ENSG00000223968.1	ENSG00000239093.1	ENSG00000205642.10
25006	ENSG00000223969.5	ENSG00000239082.1	ENSG00000174586.11
25007	ENSG00000223970.1	ENSG00000239081.1	ENSG00000285583.1
25008	ENSG00000223972.5	ENSG00000239080.1	ENSG00000279422.1
25009	ENSG00000223973.2	ENSG00000239079.1	ENSG00000100416.14
25010	ENSG00000223974.1	ENSG00000239078.1	ENSG00000253998.3
25011	ENSG00000223975.1	ENSG00000239075.1	ENSG00000249572.1
25012	ENSG00000223976.1	ENSG00000239069.1	ENSG00000185838.14
25013	ENSG00000223977.1	ENSG00000239057.2	ENSG00000233966.1

25014	ENSG00000223978.1	ENSG00000239053.1	ENSG00000234292.3
25015	ENSG00000223979.2	ENSG00000239043.1	ENSG00000159873.10
25016	ENSG00000223982.3	ENSG00000239041.1	ENSG00000272097.1
25017	ENSG00000223984.1	ENSG00000239040.1	ENSG00000149636.15
25018	ENSG00000223985.1	ENSG00000239039.1	ENSG00000282933.2
25019	ENSG00000223986.1	ENSG00000239035.1	ENSG00000235241.1
25020	ENSG00000223987.1	ENSG00000239030.1	ENSG00000250848.1
25021	ENSG00000223989.1	ENSG00000239023.1	ENSG00000198363.18
25022	ENSG00000223991.1	ENSG00000239021.1	ENSG00000204388.7
25023	ENSG00000223993.1	ENSG00000239014.1	ENSG00000246067.7
25024	ENSG00000223995.2	ENSG00000239010.1	ENSG00000200847.1
25025	ENSG00000223997.1	ENSG00000239008.1	ENSG00000151151.6
25026	ENSG00000223999.1	ENSG00000239007.1	ENSG00000160683.4
25027	ENSG00000224000.1	ENSG00000239005.1	ENSG00000211937.3
25028	ENSG00000224001.2	ENSG00000239003.1	ENSG00000233614.6
25029	ENSG00000224003.1	ENSG00000239002.3	ENSG00000254505.10
25030	ENSG00000224004.2	ENSG00000239001.1	ENSG00000250893.1
25031	ENSG00000224007.1	ENSG00000238998.1	ENSG00000127452.8
25032	ENSG00000224008.1	ENSG00000238987.1	ENSG00000268635.2
25033	ENSG00000224011.1	ENSG00000238966.1	ENSG00000242100.2
25034	ENSG00000224012.3	ENSG00000238965.1	ENSG00000226200.6
25035	ENSG00000224014.1	ENSG00000238964.1	ENSG00000121053.6
25036	ENSG00000224015.1	ENSG00000238963.2	ENSG00000189308.11
25037	ENSG00000224016.2	ENSG00000238962.1	ENSG00000258731.1
25038	ENSG00000224017.1	ENSG00000238961.1	ENSG00000230092.7
25039	ENSG00000224018.1	ENSG00000238959.1	ENSG00000221869.4
25040	ENSG00000224019.1	ENSG00000238950.1	ENSG00000104375.17
25041	ENSG00000224020.1	ENSG00000238949.1	ENSG00000180867.11
25042	ENSG00000224023.10	ENSG00000238943.1	ENSG00000157653.11
25043	ENSG00000224025.2	ENSG00000238942.1	ENSG00000255200.1
25044	ENSG00000224027.1	ENSG00000238941.1	ENSG00000178904.19
25045	ENSG00000224028.1	ENSG00000238936.1	ENSG00000241547.1
25046	ENSG00000224029.1	ENSG00000238934.1	ENSG00000281376.1
25047	ENSG00000224031.1	ENSG00000238933.1	ENSG00000083750.12
25048	ENSG00000224032.6	ENSG00000238926.1	ENSG00000254760.1
25049	ENSG00000224033.1	ENSG00000238924.2	ENSG00000162552.15
25050	ENSG00000224034.1	ENSG00000238923.1	ENSG00000185305.11
25051	ENSG00000224035.1	ENSG00000238917.1	ENSG00000233382.7
25052	ENSG00000224038.1	ENSG00000238913.1	ENSG00000253878.5
25053	ENSG00000224039.1	ENSG00000238912.1	ENSG00000234290.2
25054	ENSG00000224040.1	ENSG00000238908.1	ENSG00000154316.16
25055	ENSG00000224041.3	ENSG00000238906.1	ENSG00000171766.16
25056	ENSG00000224042.2	ENSG00000238904.1	ENSG00000184857.8
25057	ENSG00000224043.7	ENSG00000238902.1	ENSG00000215190.9
25058	ENSG00000224045.1	ENSG00000238901.1	ENSG00000228878.7
25059	ENSG00000224046.1	ENSG00000238898.1	ENSG00000137692.12
25060	ENSG00000224048.5	ENSG00000238886.1	ENSG00000110013.12
25061	ENSG00000224049.1	ENSG00000238884.1	ENSG00000115919.15
25062	ENSG00000224051.7	ENSG00000238882.1	ENSG00000100519.12
25063	ENSG00000224054.1	ENSG00000238880.1	ENSG00000267733.5
25064	ENSG00000224055.1	ENSG00000238875.1	ENSG00000224411.3
25065	ENSG00000224057.1	ENSG00000238862.1	ENSG00000198237.8
25066	ENSG00000224058.2	ENSG00000238854.1	ENSG00000148362.11

25067	ENSG00000224059.1	ENSG00000238845.1	ENSG00000131484.4
25068	ENSG00000224060.3	ENSG00000238842.1	ENSG00000101363.12
25069	ENSG00000224061.1	ENSG00000238840.1	ENSG00000090989.18
25070	ENSG00000224062.4	ENSG00000238837.3	ENSG00000254776.1
25071	ENSG00000224063.5	ENSG00000238835.1	ENSG00000244073.1
25072	ENSG00000224064.1	ENSG00000238833.1	ENSG00000124216.4
25073	ENSG00000224065.2	ENSG00000238832.1	ENSG00000286159.1
25074	ENSG00000224066.1	ENSG00000238830.1	ENSG00000205155.8
25075	ENSG00000224067.2	ENSG00000238829.1	ENSG00000095380.11
25076	ENSG00000224069.1	ENSG00000238825.1	ENSG00000211809.2
25077	ENSG00000224070.1	ENSG00000238819.1	ENSG00000041353.10
25078	ENSG00000224071.1	ENSG00000238813.1	ENSG00000164117.13
25079	ENSG00000224072.1	ENSG00000238812.1	ENSG00000277836.1
25080	ENSG00000224074.3	ENSG00000238808.1	ENSG00000197043.14
25081	ENSG00000224075.2	ENSG00000238804.1	ENSG00000170876.7
25082	ENSG00000224076.5	ENSG00000238797.1	ENSG00000230068.2
25083	ENSG00000224077.1	ENSG00000238795.1	ENSG00000165131.7
25084	ENSG00000224078.14	ENSG00000238793.1	ENSG00000093167.18
25085	ENSG00000224079.1	ENSG00000238789.1	ENSG00000230992.3
25086	ENSG00000224080.1	ENSG00000238788.1	ENSG00000151846.8
25087	ENSG00000224081.9	ENSG00000238785.1	ENSG00000080845.17
25088	ENSG00000224082.1	ENSG00000238783.1	ENSG00000167419.10
25089	ENSG00000224083.1	ENSG00000238782.1	ENSG00000253368.4
25090	ENSG00000224085.3	ENSG00000238778.1	ENSG00000275178.1
25091	ENSG00000224086.5	ENSG00000238777.1	ENSG00000187268.12
25092	ENSG00000224087.1	ENSG00000238765.1	ENSG00000224376.1
25093	ENSG00000224089.3	ENSG00000238764.1	ENSG00000225345.3
25094	ENSG00000224090.1	ENSG00000238761.1	ENSG00000162438.11
25095	ENSG00000224091.1	ENSG00000238759.1	ENSG00000212232.1
25096	ENSG00000224092.1	ENSG00000238755.3	ENSG00000267724.1
25097	ENSG00000224093.5	ENSG00000238754.1	ENSG00000196159.11
25098	ENSG00000224094.1	ENSG00000238750.1	ENSG00000225391.5
25099	ENSG00000224095.1	ENSG00000238749.1	ENSG00000272884.1
25100	ENSG00000224097.5	ENSG00000238745.1	ENSG00000258634.3
25101	ENSG00000224099.1	ENSG00000238741.1	ENSG00000282855.1
25102	ENSG00000224100.1	ENSG00000238735.1	ENSG00000234473.1
25103	ENSG00000224101.1	ENSG00000238731.1	ENSG00000277855.1
25104	ENSG00000224104.1	ENSG00000238730.1	ENSG00000079482.13
25105	ENSG00000224106.1	ENSG00000238728.1	ENSG00000175756.13
25106	ENSG00000224107.5	ENSG00000238724.1	ENSG00000254030.1
25107	ENSG00000224109.3	ENSG00000238723.1	ENSG00000239855.1
25108	ENSG00000224110.3	ENSG00000238721.1	ENSG00000224546.2
25109	ENSG00000224114.1	ENSG00000238719.1	ENSG00000028137.19
25110	ENSG00000224116.6	ENSG00000238713.1	ENSG00000166405.15
25111	ENSG00000224117.1	ENSG00000238711.1	ENSG00000162623.16
25112	ENSG00000224121.1	ENSG00000238709.1	ENSG00000251595.7
25113	ENSG00000224122.1	ENSG00000238707.1	ENSG00000120903.13
25114	ENSG00000224124.3	ENSG00000238705.1	ENSG00000105662.16
25115	ENSG00000224126.2	ENSG00000238704.1	ENSG00000101425.13
25116	ENSG00000224127.1	ENSG00000238698.1	ENSG00000228499.1
25117	ENSG00000224128.1	ENSG00000238697.1	ENSG00000278949.1
25118	ENSG00000224129.1	ENSG00000238694.1	ENSG00000148411.8
25119	ENSG00000224132.2	ENSG00000238685.1	ENSG00000101745.17

25120	ENSG00000224134.1	ENSG00000238680.1	ENSG00000167005.14
25121	ENSG00000224136.1	ENSG00000238669.1	ENSG00000155189.12
25122	ENSG00000224137.1	ENSG00000238658.1	ENSG00000281849.3
25123	ENSG00000224138.1	ENSG00000238653.1	ENSG00000166823.5
25124	ENSG00000224141.5	ENSG00000238650.1	ENSG00000141380.14
25125	ENSG00000224142.1	ENSG00000238649.1	ENSG00000189292.16
25126	ENSG00000224144.1	ENSG00000238645.1	ENSG00000132768.14
25127	ENSG00000224149.1	ENSG00000238632.1	ENSG00000276070.5
25128	ENSG00000224151.1	ENSG00000238628.1	ENSG00000168421.13
25129	ENSG00000224152.1	ENSG00000238627.1	ENSG00000236618.2
25130	ENSG00000224153.3	ENSG00000238622.1	ENSG00000266709.1
25131	ENSG00000224155.2	ENSG00000238619.1	ENSG00000168803.15
25132	ENSG00000224157.1	ENSG00000238616.1	ENSG00000213398.7
25133	ENSG00000224159.1	ENSG00000238610.1	ENSG00000159216.18
25134	ENSG00000224160.1	ENSG00000238609.1	ENSG00000233084.2
25135	ENSG00000224161.3	ENSG00000238606.1	ENSG00000236140.1
25136	ENSG00000224162.1	ENSG00000238597.1	ENSG00000198483.13
25137	ENSG00000224163.4	ENSG00000238594.1	ENSG00000258572.1
25138	ENSG00000224164.1	ENSG00000238590.1	ENSG00000151773.13
25139	ENSG00000224165.5	ENSG00000238585.1	ENSG00000006432.15
25140	ENSG00000224166.1	ENSG00000238584.1	ENSG00000131095.13
25141	ENSG00000224167.1	ENSG00000238578.1	ENSG00000170445.14
25142	ENSG00000224169.1	ENSG00000238575.1	ENSG00000123094.15
25143	ENSG00000224172.1	ENSG00000238570.1	ENSG00000142102.15
25144	ENSG00000224173.1	ENSG00000238562.1	ENSG00000204899.6
25145	ENSG00000224174.2	ENSG00000238561.1	ENSG00000158773.14
25146	ENSG00000224177.6	ENSG00000238560.1	ENSG00000204271.13
25147	ENSG00000224183.1	ENSG00000238558.1	ENSG00000272817.1
25148	ENSG00000224184.5	ENSG00000238554.1	ENSG00000141441.16
25149	ENSG00000224185.1	ENSG00000238551.1	ENSG00000283994.1
25150	ENSG00000224186.8	ENSG00000238542.1	ENSG00000258704.6
25151	ENSG00000224187.1	ENSG00000238540.1	ENSG00000013293.6
25152	ENSG00000224188.1	ENSG00000238531.1	ENSG00000273824.1
25153	ENSG00000224189.7	ENSG00000238529.1	ENSG00000110422.12
25154	ENSG00000224190.1	ENSG00000238523.1	ENSG00000142867.13
25155	ENSG00000224192.2	ENSG00000238519.1	ENSG00000258559.2
25156	ENSG00000224194.1	ENSG00000238517.1	ENSG00000280163.1
25157	ENSG00000224195.1	ENSG00000238516.1	ENSG00000283093.1
25158	ENSG00000224203.2	ENSG00000238509.1	ENSG00000196466.10
25159	ENSG00000224204.1	ENSG00000238503.1	ENSG00000185834.10
25160	ENSG00000224205.2	ENSG00000238500.1	ENSG00000286271.1
25161	ENSG00000224207.3	ENSG00000238498.1	ENSG00000260966.1
25162	ENSG00000224208.1	ENSG00000238493.1	ENSG00000260273.1
25163	ENSG00000224209.7	ENSG00000238490.1	ENSG00000147234.10
25164	ENSG00000224210.1	ENSG00000238489.1	ENSG00000235105.1
25165	ENSG00000224215.1	ENSG00000238485.1	ENSG00000230445.4
25166	ENSG00000224216.1	ENSG00000238482.1	ENSG00000185933.6
25167	ENSG00000224217.1	ENSG00000238478.1	ENSG00000274810.4
25168	ENSG00000224218.1	ENSG00000238468.1	ENSG00000130176.8
25169	ENSG00000224219.1	ENSG00000238457.1	ENSG00000233214.1
25170	ENSG00000224220.1	ENSG00000238456.1	ENSG00000187260.15
25171	ENSG00000224221.1	ENSG00000238452.1	ENSG00000229754.1
25172	ENSG00000224222.1	ENSG00000238447.1	ENSG00000245573.8

25173	ENSG00000224223.1	ENSG00000238446.1	ENSG00000211696.2
25174	ENSG00000224224.1	ENSG00000238444.1	ENSG00000163743.13
25175	ENSG00000224227.3	ENSG00000238443.1	ENSG00000182810.6
25176	ENSG00000224228.2	ENSG00000238441.1	ENSG00000131791.8
25177	ENSG00000224231.1	ENSG00000238440.1	ENSG00000171606.18
25178	ENSG00000224232.1	ENSG00000238431.1	ENSG00000256705.3
25179	ENSG00000224233.1	ENSG00000238427.1	ENSG00000204653.10
25180	ENSG00000224236.3	ENSG00000238426.1	ENSG00000244691.1
25181	ENSG00000224237.1	ENSG00000238423.1	ENSG00000284954.1
25182	ENSG00000224238.2	ENSG00000238420.1	ENSG00000226833.5
25183	ENSG00000224239.1	ENSG00000238419.1	ENSG00000173013.5
25184	ENSG00000224240.1	ENSG00000238417.1	ENSG00000162878.13
25185	ENSG00000224243.1	ENSG00000238410.1	ENSG00000256948.1
25186	ENSG00000224244.1	ENSG00000238406.1	ENSG00000134461.16
25187	ENSG00000224245.1	ENSG00000238405.1	ENSG00000273203.1
25188	ENSG00000224247.1	ENSG00000238399.1	ENSG00000159840.16
25189	ENSG00000224250.1	ENSG00000238391.1	ENSG00000197520.10
25190	ENSG00000224251.6	ENSG00000238390.1	ENSG00000105464.3
25191	ENSG00000224252.1	ENSG00000238387.1	ENSG00000283913.1
25192	ENSG00000224254.2	ENSG00000238386.1	ENSG00000228492.2
25193	ENSG00000224255.2	ENSG00000238382.1	ENSG00000168350.8
25194	ENSG00000224256.1	ENSG00000238380.1	ENSG00000274615.1
25195	ENSG00000224257.1	ENSG00000238379.1	ENSG00000160888.7
25196	ENSG00000224259.6	ENSG00000238374.1	ENSG00000272870.2
25197	ENSG00000224260.6	ENSG00000238372.1	ENSG00000235683.1
25198	ENSG00000224261.2	ENSG00000238371.1	ENSG00000162482.5
25199	ENSG00000224263.1	ENSG00000238370.1	ENSG00000217083.1
25200	ENSG00000224265.1	ENSG00000238367.1	ENSG00000203667.10
25201	ENSG00000224267.1	ENSG00000238366.1	ENSG00000259591.1
25202	ENSG00000224269.1	ENSG00000238365.3	ENSG00000261654.1
25203	ENSG00000224271.6	ENSG00000238364.1	ENSG00000178690.3
25204	ENSG00000224272.2	ENSG00000238363.1	ENSG00000272562.1
25205	ENSG00000224273.2	ENSG00000238358.2	ENSG00000269688.1
25206	ENSG00000224274.1	ENSG00000238357.1	ENSG00000135636.14
25207	ENSG00000224276.1	ENSG00000238344.1	ENSG00000279518.1
25208	ENSG00000224277.5	ENSG00000238326.1	ENSG00000111271.14
25209	ENSG00000224278.1	ENSG00000238324.1	ENSG00000240065.8
25210	ENSG00000224279.2	ENSG00000238317.2	ENSG00000120688.9
25211	ENSG00000224280.1	ENSG00000238316.1	ENSG00000023734.11
25212	ENSG00000224281.4	ENSG00000238311.1	ENSG00000174373.16
25213	ENSG00000224282.3	ENSG00000238304.1	ENSG00000205611.4
25214	ENSG00000224286.5	ENSG00000238302.1	ENSG00000144671.10
25215	ENSG00000224287.2	ENSG00000238300.1	ENSG00000263264.1
25216	ENSG00000224288.2	ENSG00000238297.2	ENSG00000227518.5
25217	ENSG00000224289.1	ENSG00000238295.1	ENSG00000170502.13
25218	ENSG00000224291.1	ENSG00000238291.1	ENSG00000164591.13
25219	ENSG00000224292.1	ENSG00000238290.1	ENSG00000245904.3
25220	ENSG00000224294.1	ENSG00000238288.1	ENSG00000279204.1
25221	ENSG00000224295.3	ENSG00000238287.1	ENSG00000091527.15
25222	ENSG00000224296.3	ENSG00000238286.1	ENSG00000245248.7
25223	ENSG00000224297.2	ENSG00000238285.1	ENSG00000163001.11
25224	ENSG00000224299.1	ENSG00000238284.1	ENSG00000164002.11
25225	ENSG00000224300.1	ENSG00000238283.3	ENSG00000260035.1

25226	ENSG00000224301.1	ENSG00000238282.1	ENSG00000145391.13
25227	ENSG00000224302.1	ENSG00000238280.2	ENSG00000091651.9
25228	ENSG00000224307.1	ENSG00000238279.1	ENSG00000254620.1
25229	ENSG00000224308.1	ENSG00000238278.3	ENSG00000229951.5
25230	ENSG00000224309.7	ENSG00000238277.1	ENSG00000091137.12
25231	ENSG00000224310.1	ENSG00000238276.5	ENSG00000236882.7
25232	ENSG00000224311.1	ENSG00000238275.2	ENSG00000188807.13
25233	ENSG00000224312.1	ENSG00000238273.3	ENSG00000260507.1
25234	ENSG00000224314.1	ENSG00000238272.1	ENSG00000137076.21
25235	ENSG00000224315.2	ENSG00000238271.2	ENSG00000141551.14
25236	ENSG00000224316.1	ENSG00000238270.1	ENSG00000237941.2
25237	ENSG00000224318.5	ENSG00000238269.8	ENSG00000197724.11
25238	ENSG00000224321.1	ENSG00000238267.1	ENSG00000197646.7
25239	ENSG00000224322.1	ENSG00000238266.2	ENSG00000232852.1
25240	ENSG00000224323.2	ENSG00000238265.1	ENSG00000278540.5
25241	ENSG00000224324.1	ENSG00000238263.1	ENSG00000245870.3
25242	ENSG00000224326.1	ENSG00000238262.1	ENSG00000234996.4
25243	ENSG00000224329.2	ENSG00000238260.1	ENSG00000166532.16
25244	ENSG00000224330.1	ENSG00000238259.1	ENSG00000137656.12
25245	ENSG00000224331.2	ENSG00000238258.1	ENSG00000102996.5
25246	ENSG00000224333.1	ENSG00000238257.1	ENSG00000277143.1
25247	ENSG00000224334.1	ENSG00000238256.1	ENSG00000243355.1
25248	ENSG00000224335.2	ENSG00000238254.3	ENSG00000274460.1
25249	ENSG00000224336.2	ENSG00000238251.2	ENSG00000167863.12
25250	ENSG00000224337.1	ENSG00000238250.1	ENSG00000240591.1
25251	ENSG00000224338.2	ENSG00000238249.2	ENSG00000145715.14
25252	ENSG00000224339.1	ENSG00000238247.2	ENSG00000226334.1
25253	ENSG00000224340.1	ENSG00000238246.1	ENSG00000172476.4
25254	ENSG00000224341.1	ENSG00000238245.2	ENSG00000118402.6
25255	ENSG00000224342.1	ENSG00000238244.3	ENSG00000071127.17
25256	ENSG00000224344.1	ENSG00000238242.1	ENSG00000243636.1
25257	ENSG00000224346.1	ENSG00000238241.2	ENSG00000154429.11
25258	ENSG00000224347.6	ENSG00000238236.1	ENSG00000187514.16
25259	ENSG00000224348.2	ENSG00000238235.1	ENSG00000167566.17
25260	ENSG00000224349.2	ENSG00000238232.1	ENSG00000187066.8
25261	ENSG00000224351.2	ENSG00000238231.2	ENSG00000173511.9
25262	ENSG00000224352.1	ENSG00000238230.1	ENSG00000235290.1
25263	ENSG00000224353.2	ENSG00000238228.1	ENSG00000256139.2
25264	ENSG00000224354.2	ENSG00000238227.8	ENSG00000225808.1
25265	ENSG00000224356.5	ENSG00000238225.1	ENSG00000270367.1
25266	ENSG00000224357.1	ENSG00000238224.1	ENSG00000242198.1
25267	ENSG00000224358.1	ENSG00000238223.1	ENSG00000102543.14
25268	ENSG00000224359.2	ENSG00000238222.3	ENSG00000206588.1
25269	ENSG00000224361.1	ENSG00000238220.1	ENSG00000270890.1
25270	ENSG00000224363.2	ENSG00000238217.5	ENSG00000213713.3
25271	ENSG00000224365.1	ENSG00000238215.1	ENSG00000176076.7
25272	ENSG00000224366.1	ENSG00000238213.1	ENSG00000164576.12
25273	ENSG00000224367.6	ENSG00000238212.2	ENSG00000204860.4
25274	ENSG00000224368.1	ENSG00000238211.1	ENSG00000257337.6
25275	ENSG00000224370.1	ENSG00000238210.3	ENSG00000129158.11
25276	ENSG00000224371.6	ENSG00000238207.1	ENSG00000147180.16
25277	ENSG00000224372.1	ENSG00000238205.3	ENSG00000225460.1
25278	ENSG00000224373.3	ENSG00000238202.1	ENSG00000228110.4

25279	ENSG00000224374.1	ENSG00000238201.1	ENSG00000018280.17
25280	ENSG00000224375.1	ENSG00000238200.1	ENSG00000236081.1
25281	ENSG00000224376.1	ENSG00000238199.1	ENSG00000258232.2
25282	ENSG00000224382.1	ENSG00000238198.1	ENSG00000274307.1
25283	ENSG00000224383.7	ENSG00000238197.5	ENSG00000152253.9
25284	ENSG00000224384.1	ENSG00000238195.1	ENSG00000196267.12
25285	ENSG00000224387.1	ENSG00000238194.1	ENSG00000239919.1
25286	ENSG00000224388.1	ENSG00000238193.1	ENSG00000235927.4
25287	ENSG00000224389.9	ENSG00000238192.1	ENSG00000178922.16
25288	ENSG00000224391.1	ENSG00000238191.3	ENSG00000255181.3
25289	ENSG00000224394.1	ENSG00000238190.1	ENSG00000111196.10
25290	ENSG00000224396.1	ENSG00000238189.2	ENSG00000106803.10
25291	ENSG00000224397.6	ENSG00000238188.1	ENSG00000152818.18
25292	ENSG00000224400.5	ENSG00000238186.1	ENSG00000080200.10
25293	ENSG00000224401.2	ENSG00000238185.1	ENSG00000270518.2
25294	ENSG00000224402.2	ENSG00000238184.1	ENSG00000234268.1
25295	ENSG00000224403.1	ENSG00000238183.2	ENSG00000247011.2
25296	ENSG00000224404.1	ENSG00000238181.2	ENSG00000106976.20
25297	ENSG00000224405.1	ENSG00000238180.1	ENSG00000102882.12
25298	ENSG00000224406.5	ENSG00000238178.6	ENSG00000139370.12
25299	ENSG00000224407.1	ENSG00000238176.1	ENSG00000183605.16
25300	ENSG00000224408.1	ENSG00000238173.1	ENSG00000006118.14
25301	ENSG00000224409.1	ENSG00000238172.2	ENSG00000099331.13
25302	ENSG00000224410.1	ENSG00000238171.1	ENSG00000110171.20
25303	ENSG00000224411.3	ENSG00000238169.1	ENSG00000184602.6
25304	ENSG00000224412.1	ENSG00000238168.5	ENSG00000122482.21
25305	ENSG00000224413.1	ENSG00000238166.2	ENSG00000213018.2
25306	ENSG00000224414.1	ENSG00000238165.1	ENSG00000110958.16
25307	ENSG00000224415.1	ENSG00000238164.6	ENSG00000221949.5
25308	ENSG00000224416.2	ENSG00000238162.1	ENSG00000225101.6
25309	ENSG00000224417.2	ENSG00000238161.1	ENSG00000162390.17
25310	ENSG00000224418.1	ENSG00000238160.1	ENSG00000143156.14
25311	ENSG00000224419.1	ENSG00000238158.6	ENSG00000260141.1
25312	ENSG00000224420.3	ENSG00000238156.1	ENSG00000053702.15
25313	ENSG00000224421.1	ENSG00000238154.1	ENSG00000252051.1
25314	ENSG00000224424.7	ENSG00000238153.1	ENSG00000281406.2
25315	ENSG00000224425.2	ENSG00000238152.2	ENSG00000211933.2
25316	ENSG00000224426.1	ENSG00000238151.1	ENSG00000175216.14
25317	ENSG00000224427.1	ENSG00000238150.1	ENSG00000262714.1
25318	ENSG00000224429.7	ENSG00000238149.1	ENSG00000164494.12
25319	ENSG00000224430.2	ENSG00000238145.2	ENSG00000112425.15
25320	ENSG00000224431.1	ENSG00000238143.1	ENSG00000243445.1
25321	ENSG00000224435.2	ENSG00000238142.1	ENSG00000140067.6
25322	ENSG00000224436.1	ENSG00000238141.2	ENSG00000240710.1
25323	ENSG00000224437.2	ENSG00000238140.1	ENSG00000184357.4
25324	ENSG00000224438.3	ENSG00000238139.2	ENSG00000106638.16
25325	ENSG00000224439.2	ENSG00000238138.1	ENSG00000121988.18
25326	ENSG00000224440.1	ENSG00000238137.2	ENSG00000162877.13
25327	ENSG00000224442.1	ENSG00000238135.1	ENSG00000224870.6
25328	ENSG00000224443.1	ENSG00000238132.2	ENSG00000285257.1
25329	ENSG00000224445.3	ENSG00000238131.1	ENSG00000271856.1
25330	ENSG00000224447.1	ENSG00000238129.5	ENSG00000280798.1
25331	ENSG00000224448.1	ENSG00000238125.1	ENSG00000260461.1

25332	ENSG00000224451.2	ENSG00000238124.1	ENSG00000115687.13
25333	ENSG00000224452.1	ENSG00000238123.1	ENSG00000178773.15
25334	ENSG00000224458.3	ENSG00000238122.1	ENSG00000279118.1
25335	ENSG00000224459.1	ENSG00000238121.5	ENSG00000152689.18
25336	ENSG00000224462.2	ENSG00000238120.1	ENSG00000095261.14
25337	ENSG00000224463.1	ENSG00000238118.1	ENSG00000231327.1
25338	ENSG00000224464.2	ENSG00000238117.1	ENSG00000165637.13
25339	ENSG00000224465.1	ENSG00000238116.2	ENSG00000225234.1
25340	ENSG00000224466.1	ENSG00000238113.6	ENSG00000185359.13
25341	ENSG00000224467.1	ENSG00000238111.1	ENSG00000132196.14
25342	ENSG00000224468.3	ENSG00000238110.1	ENSG00000228696.8
25343	ENSG00000224469.1	ENSG00000238109.1	ENSG00000196715.7
25344	ENSG00000224470.8	ENSG00000238108.1	ENSG00000140945.17
25345	ENSG00000224471.2	ENSG00000238107.1	ENSG00000250815.1
25346	ENSG00000224473.1	ENSG00000238105.7	ENSG00000178573.7
25347	ENSG00000224475.1	ENSG00000238103.4	ENSG00000225932.3
25348	ENSG00000224476.3	ENSG00000238102.1	ENSG00000215835.2
25349	ENSG00000224477.5	ENSG00000238099.2	ENSG00000279152.1
25350	ENSG00000224478.2	ENSG00000238098.9	ENSG00000206948.1
25351	ENSG00000224479.6	ENSG00000238097.1	ENSG00000130024.15
25352	ENSG00000224481.2	ENSG00000238094.1	ENSG00000149781.12
25353	ENSG00000224482.1	ENSG00000238092.1	ENSG00000204779.2
25354	ENSG00000224484.1	ENSG00000238090.1	ENSG00000118513.19
25355	ENSG00000224485.1	ENSG00000238088.1	ENSG00000248459.1
25356	ENSG00000224486.1	ENSG00000238087.3	ENSG00000155008.14
25357	ENSG00000224488.1	ENSG00000238086.5	ENSG00000184414.2
25358	ENSG00000224490.5	ENSG00000238085.1	ENSG00000162783.10
25359	ENSG00000224493.1	ENSG00000238084.4	ENSG00000116863.11
25360	ENSG00000224494.1	ENSG00000238083.7	ENSG00000205978.6
25361	ENSG00000224497.1	ENSG00000238082.1	ENSG00000227788.1
25362	ENSG00000224498.1	ENSG00000238081.1	ENSG00000213699.9
25363	ENSG00000224500.1	ENSG00000238079.1	ENSG00000250050.1
25364	ENSG00000224504.2	ENSG00000238078.1	ENSG00000276863.1
25365	ENSG00000224505.2	ENSG00000238077.1	ENSG00000134590.13
25366	ENSG00000224506.2	ENSG00000238076.1	ENSG00000166278.15
25367	ENSG00000224509.2	ENSG00000238075.1	ENSG00000169884.14
25368	ENSG00000224510.2	ENSG00000238074.5	ENSG00000125122.15
25369	ENSG00000224511.1	ENSG00000238073.1	ENSG00000233427.1
25370	ENSG00000224513.2	ENSG00000238072.1	ENSG00000164221.13
25371	ENSG00000224514.2	ENSG00000238069.1	ENSG00000125245.12
25372	ENSG00000224515.1	ENSG00000238067.1	ENSG00000226950.6
25373	ENSG00000224516.5	ENSG00000238066.1	ENSG00000189068.11
25374	ENSG00000224517.6	ENSG00000238065.1	ENSG00000239257.1
25375	ENSG00000224518.2	ENSG00000238063.2	ENSG00000229769.2
25376	ENSG00000224519.1	ENSG00000238062.5	ENSG00000114859.16
25377	ENSG00000224520.2	ENSG00000238061.2	ENSG00000166763.7
25378	ENSG00000224521.1	ENSG00000238059.1	ENSG00000203286.5
25379	ENSG00000224523.1	ENSG00000238058.2	ENSG00000274266.1
25380	ENSG00000224524.1	ENSG00000238057.9	ENSG00000095906.17
25381	ENSG00000224525.2	ENSG00000238055.1	ENSG00000280351.2
25382	ENSG00000224529.1	ENSG00000238054.2	ENSG00000230393.1
25383	ENSG00000224530.1	ENSG00000238051.1	ENSG00000235660.2
25384	ENSG00000224531.6	ENSG00000238049.2	ENSG00000108641.15

25385	ENSG00000224532.2	ENSG00000238048.1	ENSG00000171161.13
25386	ENSG00000224533.4	ENSG00000238047.1	ENSG00000030110.13
25387	ENSG00000224535.1	ENSG00000238046.1	ENSG00000151914.20
25388	ENSG00000224536.2	ENSG00000238045.9	ENSG00000118640.11
25389	ENSG00000224537.1	ENSG00000238043.1	ENSG00000135930.14
25390	ENSG00000224539.1	ENSG00000238042.5	ENSG00000269403.1
25391	ENSG00000224540.1	ENSG00000238041.3	ENSG00000269043.1
25392	ENSG00000224541.1	ENSG00000238040.1	ENSG00000163444.12
25393	ENSG00000224543.4	ENSG00000238039.1	ENSG00000111231.9
25394	ENSG00000224545.1	ENSG00000238038.1	ENSG00000205221.12
25395	ENSG00000224546.2	ENSG00000238037.1	ENSG00000158869.11
25396	ENSG00000224547.1	ENSG00000238035.8	ENSG00000244116.3
25397	ENSG00000224548.1	ENSG00000238034.1	ENSG00000070214.16
25398	ENSG00000224549.1	ENSG00000238032.1	ENSG00000152213.3
25399	ENSG00000224550.1	ENSG00000238031.1	ENSG00000235257.8
25400	ENSG00000224551.1	ENSG00000238029.1	ENSG00000047662.4
25401	ENSG00000224553.1	ENSG00000238026.1	ENSG00000268442.1
25402	ENSG00000224555.3	ENSG00000238025.1	ENSG00000213639.10
25403	ENSG00000224556.2	ENSG00000238024.1	ENSG00000135148.12
25404	ENSG00000224557.7	ENSG00000238022.1	ENSG00000104889.6
25405	ENSG00000224559.2	ENSG00000238021.6	ENSG00000228884.2
25406	ENSG00000224560.3	ENSG00000238020.1	ENSG00000100142.15
25407	ENSG00000224563.1	ENSG00000238019.1	ENSG00000267352.1
25408	ENSG00000224565.1	ENSG00000238018.2	ENSG00000143621.17
25409	ENSG00000224566.2	ENSG00000238015.2	ENSG00000254288.1
25410	ENSG00000224567.2	ENSG00000238013.1	ENSG00000230797.2
25411	ENSG00000224568.1	ENSG00000238012.1	ENSG00000161133.16
25412	ENSG00000224569.3	ENSG00000238010.1	ENSG00000270945.1
25413	ENSG00000224570.1	ENSG00000238009.6	ENSG00000140839.11
25414	ENSG00000224571.1	ENSG00000238008.1	ENSG00000285884.1
25415	ENSG00000224573.1	ENSG00000238007.1	ENSG00000244879.7
25416	ENSG00000224574.1	ENSG00000238005.3	ENSG00000125864.14
25417	ENSG00000224577.2	ENSG00000238004.1	ENSG00000279019.1
25418	ENSG00000224578.5	ENSG00000238003.1	ENSG00000182885.17
25419	ENSG00000224579.1	ENSG00000238002.3	ENSG00000106665.15
25420	ENSG00000224582.6	ENSG00000238001.2	ENSG00000187231.14
25421	ENSG00000224583.1	ENSG00000238000.1	ENSG00000072786.13
25422	ENSG00000224584.2	ENSG00000237999.2	ENSG00000188766.12
25423	ENSG00000224585.1	ENSG00000237997.1	ENSG00000280227.1
25424	ENSG00000224586.6	ENSG00000237994.1	ENSG00000177683.14
25425	ENSG00000224589.1	ENSG00000237993.1	ENSG00000135736.6
25426	ENSG00000224590.1	ENSG00000237992.2	ENSG00000003393.15
25427	ENSG00000224592.5	ENSG00000237991.3	ENSG00000242134.2
25428	ENSG00000224593.1	ENSG00000237990.3	ENSG00000279361.1
25429	ENSG00000224594.2	ENSG00000237989.1	ENSG00000149054.16
25430	ENSG00000224595.1	ENSG00000237987.1	ENSG00000275763.3
25431	ENSG00000224596.7	ENSG00000237986.3	ENSG00000211650.2
25432	ENSG00000224597.10	ENSG00000237984.3	ENSG00000198722.14
25433	ENSG00000224598.1	ENSG00000237982.1	ENSG00000165233.18
25434	ENSG00000224599.1	ENSG00000237980.1	ENSG00000121895.8
25435	ENSG00000224600.5	ENSG00000237979.1	ENSG00000266604.1
25436	ENSG00000224602.1	ENSG00000237978.5	ENSG00000258469.1
25437	ENSG00000224603.1	ENSG00000237977.1	ENSG00000163900.11

25438	ENSG00000224604.1	ENSG00000237976.1	ENSG00000161149.12
25439	ENSG00000224605.1	ENSG00000237975.6	ENSG00000101187.16
25440	ENSG00000224606.1	ENSG00000237974.1	ENSG00000241549.8
25441	ENSG00000224607.4	ENSG00000237973.1	ENSG00000267270.5
25442	ENSG00000224609.7	ENSG00000237972.1	ENSG00000124783.14
25443	ENSG00000224610.1	ENSG00000237971.1	ENSG00000214198.8
25444	ENSG00000224611.1	ENSG00000237970.1	ENSG00000160223.17
25445	ENSG00000224612.2	ENSG00000237968.1	ENSG00000275160.1
25446	ENSG00000224613.6	ENSG00000237964.3	ENSG00000230423.1
25447	ENSG00000224614.1	ENSG00000237963.1	ENSG00000229953.1
25448	ENSG00000224616.2	ENSG00000237961.4	ENSG00000146700.9
25449	ENSG00000224617.1	ENSG00000237959.1	ENSG00000136738.15
25450	ENSG00000224618.1	ENSG00000237957.6	ENSG00000184117.11
25451	ENSG00000224620.2	ENSG00000237956.1	ENSG00000272153.1
25452	ENSG00000224621.1	ENSG00000237955.1	ENSG00000143858.12
25453	ENSG00000224622.1	ENSG00000237954.7	ENSG00000253167.1
25454	ENSG00000224625.2	ENSG00000237953.1	ENSG00000132481.7
25455	ENSG00000224626.1	ENSG00000237952.1	ENSG00000259433.2
25456	ENSG00000224627.1	ENSG00000237951.1	ENSG00000009790.15
25457	ENSG00000224628.2	ENSG00000237950.1	ENSG00000133943.20
25458	ENSG00000224629.1	ENSG00000237949.1	ENSG00000169919.17
25459	ENSG00000224630.2	ENSG00000237948.1	ENSG00000232801.1
25460	ENSG00000224631.4	ENSG00000237947.1	ENSG00000105321.14
25461	ENSG00000224632.1	ENSG00000237945.7	ENSG00000163975.12
25462	ENSG00000224634.1	ENSG00000237943.6	ENSG00000255293.1
25463	ENSG00000224635.1	ENSG00000237941.2	ENSG00000120860.10
25464	ENSG00000224637.1	ENSG00000237940.3	ENSG00000243537.1
25465	ENSG00000224640.1	ENSG00000237939.1	ENSG00000175606.11
25466	ENSG00000224643.5	ENSG00000237938.5	ENSG00000171532.5
25467	ENSG00000224644.1	ENSG00000237937.5	ENSG00000248401.2
25468	ENSG00000224645.1	ENSG00000237936.1	ENSG00000163714.17
25469	ENSG00000224646.2	ENSG00000237934.1	ENSG00000233198.3
25470	ENSG00000224647.2	ENSG00000237931.1	ENSG00000279073.3
25471	ENSG00000224648.1	ENSG00000237930.1	ENSG00000097007.18
25472	ENSG00000224649.1	ENSG00000237929.1	ENSG00000169136.11
25473	ENSG00000224650.2	ENSG00000237928.5	ENSG00000171658.8
25474	ENSG00000224652.1	ENSG00000237927.1	ENSG00000184305.15
25475	ENSG00000224653.1	ENSG00000237926.1	ENSG00000227124.10
25476	ENSG00000224655.6	ENSG00000237924.1	ENSG00000213689.14
25477	ENSG00000224656.1	ENSG00000237923.1	ENSG00000156194.18
25478	ENSG00000224657.9	ENSG00000237922.1	ENSG00000205890.3
25479	ENSG00000224658.1	ENSG00000237921.2	ENSG00000163041.10
25480	ENSG00000224659.2	ENSG00000237920.2	ENSG00000253797.2
25481	ENSG00000224660.1	ENSG00000237919.5	ENSG00000147576.17
25482	ENSG00000224661.1	ENSG00000237917.1	ENSG00000155545.19
25483	ENSG00000224662.2	ENSG00000237916.1	ENSG00000174748.20
25484	ENSG00000224664.2	ENSG00000237914.5	ENSG00000111145.8
25485	ENSG00000224666.3	ENSG00000237913.1	ENSG00000279601.1
25486	ENSG00000224667.1	ENSG00000237911.2	ENSG00000253593.2
25487	ENSG00000224668.1	ENSG00000237910.1	ENSG00000124721.17
25488	ENSG00000224669.1	ENSG00000237906.1	ENSG00000140986.8
25489	ENSG00000224670.1	ENSG00000237904.2	ENSG00000260479.1
25490	ENSG00000224671.2	ENSG00000237903.1	ENSG00000254528.7

25491	ENSG00000224672.4	ENSG00000237902.1	ENSG00000079819.19
25492	ENSG00000224674.1	ENSG00000237901.1	ENSG00000147162.14
25493	ENSG00000224675.1	ENSG00000237899.1	ENSG00000242193.11
25494	ENSG00000224677.1	ENSG00000237897.2	ENSG00000183019.7
25495	ENSG00000224678.3	ENSG00000237896.6	ENSG00000182586.8
25496	ENSG00000224679.1	ENSG00000237892.1	ENSG00000267980.1
25497	ENSG00000224680.4	ENSG00000237891.1	ENSG00000260810.1
25498	ENSG00000224681.1	ENSG00000237888.1	ENSG00000065427.14
25499	ENSG00000224682.1	ENSG00000237887.1	ENSG00000132475.10
25500	ENSG00000224683.1	ENSG00000237886.1	ENSG00000101193.8
25501	ENSG00000224685.1	ENSG00000237885.1	ENSG00000254098.1
25502	ENSG00000224686.1	ENSG00000237883.1	ENSG00000102743.15
25503	ENSG00000224687.1	ENSG00000237882.1	ENSG00000144535.19
25504	ENSG00000224688.1	ENSG00000237880.1	ENSG00000261096.1
25505	ENSG00000224689.9	ENSG00000237879.1	ENSG00000155966.13
25506	ENSG00000224691.1	ENSG00000237877.6	ENSG00000116903.7
25507	ENSG00000224692.1	ENSG00000237876.1	ENSG00000188282.12
25508	ENSG00000224695.1	ENSG00000237875.1	ENSG00000264304.1
25509	ENSG00000224697.1	ENSG00000237874.2	ENSG00000105327.17
25510	ENSG00000224698.1	ENSG00000237873.1	ENSG00000213185.7
25511	ENSG00000224699.8	ENSG00000237872.4	ENSG00000226981.2
25512	ENSG00000224700.2	ENSG00000237870.6	ENSG00000273015.2
25513	ENSG00000224701.1	ENSG00000237868.1	ENSG00000174996.11
25514	ENSG00000224702.1	ENSG00000237864.1	ENSG00000142459.8
25515	ENSG00000224706.1	ENSG00000237863.2	ENSG00000236184.1
25516	ENSG00000224707.1	ENSG00000237862.1	ENSG00000112167.10
25517	ENSG00000224709.2	ENSG00000237861.1	ENSG00000185627.18
25518	ENSG00000224711.1	ENSG00000237860.1	ENSG00000257101.1
25519	ENSG00000224712.12	ENSG00000237859.2	ENSG00000198732.10
25520	ENSG00000224713.4	ENSG00000237856.1	ENSG00000064547.14
25521	ENSG00000224714.2	ENSG00000237854.3	ENSG00000172819.17
25522	ENSG00000224715.1	ENSG00000237853.6	ENSG00000274576.2
25523	ENSG00000224717.1	ENSG00000237852.1	ENSG00000267221.2
25524	ENSG00000224718.1	ENSG00000237851.1	ENSG00000224288.2
25525	ENSG00000224719.1	ENSG00000237850.7	ENSG00000095932.6
25526	ENSG00000224721.1	ENSG00000237849.1	ENSG00000237840.6
25527	ENSG00000224722.3	ENSG00000237848.1	ENSG00000211778.2
25528	ENSG00000224723.1	ENSG00000237846.1	ENSG00000227627.2
25529	ENSG00000224725.3	ENSG00000237845.1	ENSG00000143851.15
25530	ENSG00000224727.1	ENSG00000237844.1	ENSG00000132801.7
25531	ENSG00000224728.1	ENSG00000237843.1	ENSG00000225014.1
25532	ENSG00000224729.5	ENSG00000237842.2	ENSG00000100220.12
25533	ENSG00000224730.1	ENSG00000237841.1	ENSG00000153044.10
25534	ENSG00000224731.1	ENSG00000237840.6	ENSG00000176723.10
25535	ENSG00000224732.1	ENSG00000237838.1	ENSG00000168612.4
25536	ENSG00000224733.1	ENSG00000237837.1	ENSG00000091262.15
25537	ENSG00000224735.1	ENSG00000237836.5	ENSG00000197102.12
25538	ENSG00000224738.1	ENSG00000237835.1	ENSG00000070413.20
25539	ENSG00000224739.2	ENSG00000237833.1	ENSG00000274765.1
25540	ENSG00000224743.6	ENSG00000237832.1	ENSG00000271933.1
25541	ENSG00000224745.1	ENSG00000237828.1	ENSG00000182557.8
25542	ENSG00000224746.1	ENSG00000237827.1	ENSG00000106100.11
25543	ENSG00000224747.1	ENSG00000237824.2	ENSG00000251692.7

25544	ENSG00000224750.6	ENSG00000237823.1	ENSG00000220517.2
25545	ENSG00000224751.2	ENSG00000237821.1	ENSG00000009694.13
25546	ENSG00000224752.1	ENSG00000237819.5	ENSG00000136868.11
25547	ENSG00000224755.1	ENSG00000237818.1	ENSG00000231747.1
25548	ENSG00000224758.1	ENSG00000237816.1	ENSG00000105672.14
25549	ENSG00000224760.4	ENSG00000237813.3	ENSG00000169891.18
25550	ENSG00000224761.4	ENSG00000237810.3	ENSG00000141956.13
25551	ENSG00000224762.1	ENSG00000237807.3	ENSG00000232671.5
25552	ENSG00000224763.1	ENSG00000237806.1	ENSG00000278737.1
25553	ENSG00000224764.1	ENSG00000237804.1	ENSG00000143630.10
25554	ENSG00000224765.1	ENSG00000237803.5	ENSG00000260188.1
25555	ENSG00000224769.1	ENSG00000237802.7	ENSG00000248642.1
25556	ENSG00000224771.1	ENSG00000237801.6_PAR_Y	ENSG00000119328.12
25557	ENSG00000224773.2	ENSG00000237801.6	ENSG00000163521.16
25558	ENSG00000224775.2	ENSG00000237799.2	ENSG00000256500.5
25559	ENSG00000224776.3	ENSG00000237798.1	ENSG00000177337.7
25560	ENSG00000224777.3	ENSG00000237797.1	ENSG00000214485.6
25561	ENSG00000224778.1	ENSG00000237793.3	ENSG00000167130.18
25562	ENSG00000224781.1	ENSG00000237790.1	ENSG00000279602.1
25563	ENSG00000224783.1	ENSG00000237788.1	ENSG00000186300.12
25564	ENSG00000224784.2	ENSG00000237787.5	ENSG00000185386.15
25565	ENSG00000224785.1	ENSG00000237786.1	ENSG00000166845.15
25566	ENSG00000224786.2	ENSG00000237784.1	ENSG00000164051.14
25567	ENSG00000224788.1	ENSG00000237783.2	ENSG00000160447.7
25568	ENSG00000224789.1	ENSG00000237782.3	ENSG00000260060.1
25569	ENSG00000224790.2	ENSG00000237781.3	ENSG00000184939.16
25570	ENSG00000224791.1	ENSG00000237775.1	ENSG00000245112.2
25571	ENSG00000224792.6	ENSG00000237774.3	ENSG00000103194.15
25572	ENSG00000224794.2	ENSG00000237773.6	ENSG00000280046.1
25573	ENSG00000224795.1	ENSG00000237772.1	ENSG00000153283.12
25574	ENSG00000224796.1	ENSG00000237770.2	ENSG00000165312.6
25575	ENSG00000224797.1	ENSG00000237768.2	ENSG00000224383.7
25576	ENSG00000224799.1	ENSG00000237767.1	ENSG00000272677.1
25577	ENSG00000224800.1	ENSG00000237766.2	ENSG00000135900.4
25578	ENSG00000224802.2	ENSG00000237765.7	ENSG00000130489.14
25579	ENSG00000224805.2	ENSG00000237763.9	ENSG00000178921.14
25580	ENSG00000224806.2	ENSG00000237761.2	ENSG00000170310.15
25581	ENSG00000224807.5	ENSG00000237760.1	ENSG00000256987.1
25582	ENSG00000224808.1	ENSG00000237759.1	ENSG00000086730.17
25583	ENSG00000224809.2	ENSG00000237758.1	ENSG00000183889.12
25584	ENSG00000224810.1	ENSG00000237757.2	ENSG00000118194.19
25585	ENSG00000224812.2	ENSG00000237756.1	ENSG00000271966.1
25586	ENSG00000224814.1	ENSG00000237754.1	ENSG00000270959.1
25587	ENSG00000224815.3	ENSG00000237753.1	ENSG00000265791.1
25588	ENSG00000224816.1	ENSG00000237752.2	ENSG00000272755.1
25589	ENSG00000224817.1	ENSG00000237751.2	ENSG00000284702.1
25590	ENSG00000224818.1	ENSG00000237750.2	ENSG00000239951.1
25591	ENSG00000224819.1	ENSG00000237749.4	ENSG00000179407.3
25592	ENSG00000224820.2	ENSG00000237748.1	ENSG00000272566.1
25593	ENSG00000224821.5	ENSG00000237747.1	ENSG00000117400.17
25594	ENSG00000224822.1	ENSG00000237746.1	ENSG00000223026.1
25595	ENSG00000224825.2	ENSG00000237745.1	ENSG00000286129.1
25596	ENSG00000224826.1	ENSG00000237743.3	ENSG00000151532.13

25597	ENSG00000224827.1	ENSG00000237742.6	ENSG00000277715.1
25598	ENSG00000224829.3	ENSG00000237741.1	ENSG00000164114.19
25599	ENSG00000224830.2	ENSG00000237740.1	ENSG00000185960.14
25600	ENSG00000224831.3	ENSG00000237738.1	ENSG00000272468.1
25601	ENSG00000224832.2	ENSG00000237737.5	ENSG00000225580.2
25602	ENSG00000224834.1	ENSG00000237735.2	ENSG00000196118.12
25603	ENSG00000224836.1	ENSG00000237734.1	ENSG00000148296.7
25604	ENSG00000224837.1	ENSG00000237732.9	ENSG00000115541.11
25605	ENSG00000224839.1	ENSG00000237731.1	ENSG00000164362.19
25606	ENSG00000224842.2	ENSG00000237730.1	ENSG00000009413.15
25607	ENSG00000224843.7	ENSG00000237729.2	ENSG00000223750.1
25608	ENSG00000224844.1	ENSG00000237728.2	ENSG00000232024.2
25609	ENSG00000224846.2	ENSG00000237722.2	ENSG00000186130.5
25610	ENSG00000224848.1	ENSG00000237720.1	ENSG00000212719.11
25611	ENSG00000224851.2	ENSG00000237719.1	ENSG00000178385.15
25612	ENSG00000224852.1	ENSG00000237718.2	ENSG00000163393.13
25613	ENSG00000224853.2	ENSG00000237717.1	ENSG00000137409.19
25614	ENSG00000224854.3	ENSG00000237716.1	ENSG00000276966.2
25615	ENSG00000224855.5	ENSG00000237714.1	ENSG00000285812.1
25616	ENSG00000224856.2	ENSG00000237713.1	ENSG00000285151.1
25617	ENSG00000224857.1	ENSG00000237711.1	ENSG00000275759.1
25618	ENSG00000224858.5	ENSG00000237709.1	ENSG00000204410.15
25619	ENSG00000224860.1	ENSG00000237708.1	ENSG00000170667.14
25620	ENSG00000224861.1	ENSG00000237707.1	ENSG00000286231.1
25621	ENSG00000224863.2	ENSG00000237706.4	ENSG00000101868.10
25622	ENSG00000224864.4	ENSG00000237705.1	ENSG00000226608.3
25623	ENSG00000224865.7	ENSG00000237704.1	ENSG00000235602.5
25624	ENSG00000224866.1	ENSG00000237702.2	ENSG00000256453.2
25625	ENSG00000224869.1	ENSG00000237701.1	ENSG00000280401.1
25626	ENSG00000224870.6	ENSG00000237700.2	ENSG00000136878.13
25627	ENSG00000224873.1	ENSG00000237699.2	ENSG00000269473.1
25628	ENSG00000224875.2	ENSG00000237693.4	ENSG00000161911.11
25629	ENSG00000224876.1	ENSG00000237691.1	ENSG00000219532.2
25630	ENSG00000224877.4	ENSG00000237689.1	ENSG00000101574.15
25631	ENSG00000224879.1	ENSG00000237687.2	ENSG00000175213.2
25632	ENSG00000224880.1	ENSG00000237686.6	ENSG00000259335.1
25633	ENSG00000224881.1	ENSG00000237685.1	ENSG00000105137.13
25634	ENSG00000224883.1	ENSG00000237684.1	ENSG00000048028.11
25635	ENSG00000224884.1	ENSG00000237682.2	ENSG00000272037.1
25636	ENSG00000224885.1	ENSG00000237679.2	ENSG00000081148.12
25637	ENSG00000224886.2	ENSG00000237676.1	ENSG00000215182.8
25638	ENSG00000224887.1	ENSG00000237675.5	ENSG00000101443.18
25639	ENSG00000224888.4	ENSG00000237674.1	ENSG00000085063.16
25640	ENSG00000224891.1	ENSG00000237672.1	ENSG00000273199.1
25641	ENSG00000224892.6	ENSG00000237671.3	ENSG00000197579.8
25642	ENSG00000224893.6	ENSG00000237670.1	ENSG00000166295.9
25643	ENSG00000224895.1	ENSG00000237669.1	ENSG00000260417.1
25644	ENSG00000224896.2	ENSG00000237668.1	ENSG00000197980.12
25645	ENSG00000224897.7	ENSG00000237667.6	ENSG00000182057.5
25646	ENSG00000224899.1	ENSG00000237666.2	ENSG00000236698.1
25647	ENSG00000224901.1	ENSG00000237665.1	ENSG00000130559.19
25648	ENSG00000224902.5	ENSG00000237664.1	ENSG00000279320.1
25649	ENSG00000224903.1	ENSG00000237663.2	ENSG00000231079.7

25650	ENSG00000224904.1	ENSG00000237662.1	ENSG00000215529.12
25651	ENSG00000224905.6	ENSG00000237661.1	ENSG00000136250.11
25652	ENSG00000224906.3	ENSG00000237659.1	ENSG00000244476.3
25653	ENSG00000224907.1	ENSG00000237655.1	ENSG00000279620.1
25654	ENSG00000224908.1	ENSG00000237654.5	ENSG00000116711.10
25655	ENSG00000224910.1	ENSG00000237653.2	ENSG00000254635.5
25656	ENSG00000224914.3	ENSG00000237651.6	ENSG00000175701.10
25657	ENSG00000224916.9	ENSG00000237650.2	ENSG00000154227.13
25658	ENSG00000224917.1	ENSG00000237649.8	ENSG00000185674.9
25659	ENSG00000224918.1	ENSG00000237646.1	ENSG00000132478.10
25660	ENSG00000224919.1	ENSG00000237645.1	ENSG00000279970.1
25661	ENSG00000224920.1	ENSG00000237643.1	ENSG00000268173.3
25662	ENSG00000224922.1	ENSG00000237642.1	ENSG00000279365.1
25663	ENSG00000224924.6	ENSG00000237641.1	ENSG00000224985.1
25664	ENSG00000224927.2	ENSG00000237640.1	ENSG00000203327.2
25665	ENSG00000224928.2	ENSG00000237639.2	ENSG00000146063.20
25666	ENSG00000224931.4	ENSG00000237638.1	ENSG00000135447.17
25667	ENSG00000224932.1	ENSG00000237637.1	ENSG00000143546.10
25668	ENSG00000224933.2	ENSG00000237636.2	ENSG00000198034.11
25669	ENSG00000224934.4	ENSG00000237635.2	ENSG00000261377.5
25670	ENSG00000224935.1	ENSG00000237633.3	ENSG00000268621.5
25671	ENSG00000224936.1	ENSG00000237632.3	ENSG00000277508.1
25672	ENSG00000224939.1	ENSG00000237631.2	ENSG00000044446.11
25673	ENSG00000224940.8	ENSG00000237630.1	ENSG00000253180.1
25674	ENSG00000224942.1	ENSG00000237629.1	ENSG00000033050.9
25675	ENSG00000224943.1	ENSG00000237628.2	ENSG00000106560.11
25676	ENSG00000224944.1	ENSG00000237626.1	ENSG00000153495.10
25677	ENSG00000224945.1	ENSG00000237624.1	ENSG00000050405.13
25678	ENSG00000224946.1	ENSG00000237623.1	ENSG00000273654.1
25679	ENSG00000224947.1	ENSG00000237622.1	ENSG00000162599.16
25680	ENSG00000224948.1	ENSG00000237621.3	ENSG00000286207.1
25681	ENSG00000224949.2	ENSG00000237620.1	ENSG00000214351.5
25682	ENSG00000224950.2	ENSG00000237619.2	ENSG00000065548.18
25683	ENSG00000224953.1	ENSG00000237618.1	ENSG00000256972.1
25684	ENSG00000224955.1	ENSG00000237617.1	ENSG00000072803.17
25685	ENSG00000224957.5	ENSG00000237616.1	ENSG00000270170.2
25686	ENSG00000224958.5	ENSG00000237614.2	ENSG00000248571.1
25687	ENSG00000224959.1	ENSG00000237613.2	ENSG00000136371.10
25688	ENSG00000224960.4	ENSG00000237612.1	ENSG00000227507.2
25689	ENSG00000224961.2	ENSG00000237611.2	ENSG00000267758.1
25690	ENSG00000224962.4	ENSG00000237610.1	ENSG00000153714.6
25691	ENSG00000224963.3	ENSG00000237609.1	ENSG00000279467.1
25692	ENSG00000224964.1	ENSG00000237606.2	ENSG00000230257.3
25693	ENSG00000224965.1	ENSG00000237605.1	ENSG00000272087.1
25694	ENSG00000224966.4	ENSG00000237604.1	ENSG00000128309.16
25695	ENSG00000224967.1	ENSG00000237603.1	ENSG00000214212.9
25696	ENSG00000224968.1	ENSG00000237601.1	ENSG00000174946.6
25697	ENSG00000224969.1	ENSG00000237596.6	ENSG00000133997.11
25698	ENSG00000224970.2	ENSG00000237595.4	ENSG00000105835.12
25699	ENSG00000224971.1	ENSG00000237594.2	ENSG00000266527.1
25700	ENSG00000224972.1	ENSG00000237593.1	ENSG00000213260.3
25701	ENSG00000224973.5	ENSG00000237592.2	ENSG00000106261.17
25702	ENSG00000224975.1	ENSG00000237590.1	ENSG00000166501.13

25703	ENSG00000224976.2	ENSG00000237589.1	ENSG00000135835.12
25704	ENSG00000224977.1	ENSG00000237588.2	ENSG00000140451.13
25705	ENSG00000224978.1	ENSG00000237586.3	ENSG00000147383.11
25706	ENSG00000224980.1	ENSG00000237585.1	ENSG00000108591.10
25707	ENSG00000224981.1	ENSG00000237584.1	ENSG00000279977.1
25708	ENSG00000224982.4	ENSG00000237583.1	ENSG00000124713.6
25709	ENSG00000224984.1	ENSG00000237581.1	ENSG00000172716.16
25710	ENSG00000224985.1	ENSG00000237580.1	ENSG00000235351.1
25711	ENSG00000224986.2	ENSG00000237579.2	ENSG00000259167.2
25712	ENSG00000224987.1	ENSG00000237576.1	ENSG00000257108.2
25713	ENSG00000224988.1	ENSG00000237575.4	ENSG00000255141.1
25714	ENSG00000224989.2	ENSG00000237574.1	ENSG00000241081.1
25715	ENSG00000224992.2	ENSG00000237572.1	ENSG00000105229.7
25716	ENSG00000224993.3	ENSG00000237571.1	ENSG00000240053.8
25717	ENSG00000224995.1	ENSG00000237569.1	ENSG00000213865.7
25718	ENSG00000224999.1	ENSG00000237568.1	ENSG00000284691.1
25719	ENSG00000225000.1	ENSG00000237567.1	ENSG00000143633.13
25720	ENSG00000225002.2	ENSG00000237566.1	ENSG00000275740.1
25721	ENSG00000225003.1	ENSG00000237563.2	ENSG00000166086.12
25722	ENSG00000225005.1	ENSG00000237560.6	ENSG00000145414.9
25723	ENSG00000225006.1	ENSG00000237558.1	ENSG00000147257.13
25724	ENSG00000225007.1	ENSG00000237557.1	ENSG00000101265.16
25725	ENSG00000225008.1	ENSG00000237556.1	ENSG00000269220.1
25726	ENSG00000225011.4	ENSG00000237555.1	ENSG00000279262.1
25727	ENSG00000225012.5	ENSG00000237553.1	ENSG00000206892.1
25728	ENSG00000225014.1	ENSG00000237552.1	ENSG00000204160.12
25729	ENSG00000225016.1	ENSG00000237551.1	ENSG00000164930.12
25730	ENSG00000225017.1	ENSG00000237550.5	ENSG00000275791.1
25731	ENSG00000225018.1	ENSG00000237549.1	ENSG00000170954.11
25732	ENSG00000225022.1	ENSG00000237548.1	ENSG00000243829.1
25733	ENSG00000225024.1	ENSG00000237547.1	ENSG00000196074.13
25734	ENSG00000225025.2	ENSG00000237546.1	ENSG00000224738.1
25735	ENSG00000225026.1	ENSG00000237542.1	ENSG00000275854.1
25736	ENSG00000225027.2	ENSG00000237541.3	ENSG00000166710.19
25737	ENSG00000225028.1	ENSG00000237540.1	ENSG00000259539.1
25738	ENSG00000225030.1	ENSG00000237539.1	ENSG00000231119.2
25739	ENSG00000225031.1	ENSG00000237532.1	ENSG00000226054.2
25740	ENSG00000225032.5	ENSG00000237531.6_PAR_Y	ENSG00000124160.12
25741	ENSG00000225036.1	ENSG00000237531.6	ENSG00000271853.5
25742	ENSG00000225037.1	ENSG00000237530.1	ENSG00000232725.1
25743	ENSG00000225039.1	ENSG00000237528.1	ENSG00000095303.16
25744	ENSG00000225043.1	ENSG00000237527.1	ENSG00000177943.14
25745	ENSG00000225044.1	ENSG00000237525.6	ENSG00000140280.14
25746	ENSG00000225045.1	ENSG00000237524.10	ENSG00000167528.12
25747	ENSG00000225046.2	ENSG00000237523.1	ENSG00000147804.9
25748	ENSG00000225050.1	ENSG00000237522.1	ENSG00000237799.2
25749	ENSG00000225051.5	ENSG00000237521.3	ENSG00000146232.16
25750	ENSG00000225053.1	ENSG00000237520.1	ENSG00000183337.16
25751	ENSG00000225055.1	ENSG00000237517.9	ENSG00000055211.14
25752	ENSG00000225056.1	ENSG00000237515.9	ENSG00000117586.11
25753	ENSG00000225057.2	ENSG00000237514.3	ENSG00000250486.4
25754	ENSG00000225058.1	ENSG00000237513.1	ENSG00000267105.1
25755	ENSG00000225062.1	ENSG00000237512.6	ENSG00000272844.1

25756	ENSG00000225063.1	ENSG00000237510.7	ENSG00000281706.2
25757	ENSG00000225064.1	ENSG00000237506.3	ENSG00000026652.14
25758	ENSG00000225066.1	ENSG00000237505.7	ENSG00000187800.13
25759	ENSG00000225067.4	ENSG00000237503.2	ENSG00000163319.11
25760	ENSG00000225069.3	ENSG00000237501.1	ENSG00000204248.10
25761	ENSG00000225071.1	ENSG00000237500.1	ENSG00000232993.1
25762	ENSG00000225072.1	ENSG00000237499.6	ENSG00000253948.1
25763	ENSG00000225075.1	ENSG00000237498.3	ENSG00000162676.12
25764	ENSG00000225076.1	ENSG00000237494.1	ENSG00000171101.13
25765	ENSG00000225077.3	ENSG00000237493.3	ENSG00000225614.2
25766	ENSG00000225078.2	ENSG00000237492.1	ENSG00000282246.1
25767	ENSG00000225079.2	ENSG00000237491.9	ENSG00000179981.10
25768	ENSG00000225080.1	ENSG00000237490.3	ENSG00000178694.10
25769	ENSG00000225082.2	ENSG00000237489.5	ENSG00000151576.10
25770	ENSG00000225083.1	ENSG00000237487.1	ENSG00000187607.16
25771	ENSG00000225084.1	ENSG00000237484.5	ENSG00000179630.11
25772	ENSG00000225085.1	ENSG00000237483.1	ENSG00000074803.18
25773	ENSG00000225087.1	ENSG00000237481.1	ENSG00000260038.1
25774	ENSG00000225091.3	ENSG00000237480.2	ENSG00000266969.1
25775	ENSG00000225092.2	ENSG00000237479.1	ENSG00000130052.13
25776	ENSG00000225093.1	ENSG00000237478.2	ENSG00000117242.7
25777	ENSG00000225094.3	ENSG00000237477.1	ENSG00000203760.8
25778	ENSG00000225096.2	ENSG00000237476.1	ENSG00000230732.4
25779	ENSG00000225098.1	ENSG00000237475.2	ENSG00000198756.12
25780	ENSG00000225099.1	ENSG00000237473.1	ENSG00000147324.11
25781	ENSG00000225100.2	ENSG00000237472.1	ENSG00000236194.3
25782	ENSG00000225101.6	ENSG00000237471.1	ENSG00000175416.13
25783	ENSG00000225102.1	ENSG00000237470.3	ENSG00000105520.10
25784	ENSG00000225105.2	ENSG00000237469.2	ENSG00000184271.17
25785	ENSG00000225106.1	ENSG00000237468.2	ENSG00000220370.1
25786	ENSG00000225107.1	ENSG00000237467.1	ENSG00000240240.8
25787	ENSG00000225108.1	ENSG00000237466.1	ENSG00000240303.8
25788	ENSG00000225110.2	ENSG00000237464.1	ENSG00000259379.1
25789	ENSG00000225111.1	ENSG00000237463.5	ENSG00000101911.12
25790	ENSG00000225112.1	ENSG00000237461.1	ENSG00000277463.1
25791	ENSG00000225113.1	ENSG00000237460.2	ENSG00000175611.11
25792	ENSG00000225116.1	ENSG00000237458.1	ENSG00000261499.2
25793	ENSG00000225117.2	ENSG00000237457.6	ENSG00000136122.16
25794	ENSG00000225118.1	ENSG00000237456.3	ENSG00000227345.8
25795	ENSG00000225121.2	ENSG00000237453.1	ENSG00000185055.11
25796	ENSG00000225122.1	ENSG00000237452.2	ENSG00000079805.16
25797	ENSG00000225123.2	ENSG00000237451.3	ENSG00000272654.1
25798	ENSG00000225124.1	ENSG00000237450.5	ENSG00000134146.12
25799	ENSG00000225125.2	ENSG00000237449.1	ENSG00000147138.2
25800	ENSG00000225126.1	ENSG00000237447.1	ENSG00000179082.3
25801	ENSG00000225127.2	ENSG00000237446.1	ENSG00000202058.1
25802	ENSG00000225128.1	ENSG00000237445.2	ENSG00000118496.5
25803	ENSG00000225129.1	ENSG00000237444.1	ENSG00000255325.2
25804	ENSG00000225131.2	ENSG00000237443.1	ENSG00000173559.12
25805	ENSG00000225133.1	ENSG00000237442.3	ENSG00000272072.1
25806	ENSG00000225136.1	ENSG00000237441.10	ENSG00000105707.13
25807	ENSG00000225137.1	ENSG00000237440.9	ENSG00000002586.20
25808	ENSG00000225138.7	ENSG00000237439.1	ENSG00000107338.10

25809	ENSG00000225140.1	ENSG00000237438.7	ENSG00000260572.1
25810	ENSG00000225142.2	ENSG00000237437.1	ENSG00000260300.5
25811	ENSG00000225144.2	ENSG00000237436.1	ENSG00000179846.9
25812	ENSG00000225146.1	ENSG00000237435.9	ENSG00000141068.14
25813	ENSG00000225147.1	ENSG00000237434.1	ENSG00000237758.1
25814	ENSG00000225148.1	ENSG00000237433.1	ENSG00000280047.1
25815	ENSG00000225151.10	ENSG00000237432.2	ENSG00000148158.17
25816	ENSG00000225152.1	ENSG00000237429.1	ENSG00000251340.1
25817	ENSG00000225154.2	ENSG00000237428.1	ENSG00000278896.1
25818	ENSG00000225155.4	ENSG00000237427.1	ENSG00000279838.1
25819	ENSG00000225156.2	ENSG00000237426.1	ENSG00000259536.5
25820	ENSG00000225158.2	ENSG00000237425.1	ENSG00000285650.1
25821	ENSG00000225159.1	ENSG00000237424.1	ENSG00000251247.11
25822	ENSG00000225163.4	ENSG00000237423.1	ENSG00000262703.1
25823	ENSG00000225165.3	ENSG00000237422.1	ENSG00000204642.14
25824	ENSG00000225166.1	ENSG00000237419.1	ENSG00000263072.6
25825	ENSG00000225167.1	ENSG00000237418.1	ENSG00000227857.2
25826	ENSG00000225169.1	ENSG00000237417.1	ENSG00000234917.2
25827	ENSG00000225170.2	ENSG00000237415.2	ENSG00000177380.14
25828	ENSG00000225171.2	ENSG00000237414.2	ENSG00000225142.2
25829	ENSG00000225172.5	ENSG00000237413.5	ENSG00000261186.2
25830	ENSG00000225173.1	ENSG00000237412.6	ENSG00000254598.2
25831	ENSG00000225174.1	ENSG00000237410.1	ENSG00000160185.15
25832	ENSG00000225175.1	ENSG00000237409.2	ENSG00000168288.13
25833	ENSG00000225176.2	ENSG00000237408.1	ENSG00000181610.13
25834	ENSG00000225177.5	ENSG00000237407.1	ENSG00000004809.14
25835	ENSG00000225178.5	ENSG00000237406.1	ENSG00000118976.5
25836	ENSG00000225179.1	ENSG00000237402.1	ENSG00000183495.13
25837	ENSG00000225180.7	ENSG00000237401.7	ENSG00000271936.1
25838	ENSG00000225181.1	ENSG00000237400.1	ENSG00000259726.1
25839	ENSG00000225182.1	ENSG00000237399.7	ENSG00000224652.1
25840	ENSG00000225183.1	ENSG00000237398.1	ENSG00000285535.1
25841	ENSG00000225185.3	ENSG00000237396.1	ENSG00000268047.1
25842	ENSG00000225187.1	ENSG00000237390.1	ENSG00000134709.11
25843	ENSG00000225189.1	ENSG00000237389.1	ENSG00000156414.19
25844	ENSG00000225190.11	ENSG00000237388.2	ENSG00000267372.2
25845	ENSG00000225191.1	ENSG00000237387.1	ENSG00000260267.1
25846	ENSG00000225192.1	ENSG00000237385.1	ENSG00000257122.5
25847	ENSG00000225193.5	ENSG00000237383.1	ENSG00000253210.1
25848	ENSG00000225194.3	ENSG00000237382.2	ENSG00000136783.10
25849	ENSG00000225195.2	ENSG00000237381.2	ENSG00000135763.10
25850	ENSG00000225196.2	ENSG00000237380.7	ENSG00000187690.4
25851	ENSG00000225198.1	ENSG00000237379.1	ENSG00000266794.2
25852	ENSG00000225200.2	ENSG00000237378.3	ENSG00000229695.1
25853	ENSG00000225203.2	ENSG00000237377.2	ENSG00000267247.1
25854	ENSG00000225205.5	ENSG00000237373.1	ENSG00000285509.1
25855	ENSG00000225206.9	ENSG00000237371.1	ENSG00000073350.13
25856	ENSG00000225208.1	ENSG00000237370.1	ENSG00000185338.5
25857	ENSG00000225209.1	ENSG00000237365.1	ENSG00000158292.7
25858	ENSG00000225210.10	ENSG00000237363.1	ENSG00000283103.2
25859	ENSG00000225212.4	ENSG00000237361.2	ENSG00000126953.7
25860	ENSG00000225213.2	ENSG00000237360.2	ENSG00000255464.1
25861	ENSG00000225214.1	ENSG00000237359.1	ENSG00000279583.1

25862	ENSG00000225215.1	ENSG00000237358.1	ENSG00000203618.5
25863	ENSG00000225216.6	ENSG00000237357.2	ENSG00000100097.12
25864	ENSG00000225217.1	ENSG00000237356.6	ENSG00000078142.13
25865	ENSG00000225218.1	ENSG00000237354.1	ENSG00000196329.11
25866	ENSG00000225221.1	ENSG00000237353.6	ENSG00000286158.1
25867	ENSG00000225222.2	ENSG00000237352.3	ENSG00000188906.16
25868	ENSG00000225224.1	ENSG00000237351.1	ENSG00000100379.17
25869	ENSG00000225225.1	ENSG00000237350.1	ENSG00000185404.16
25870	ENSG00000225226.1	ENSG00000237349.1	ENSG00000071282.12
25871	ENSG00000225230.1	ENSG00000237347.2	ENSG00000202306.1
25872	ENSG00000225231.1	ENSG00000237346.1	ENSG00000277452.1
25873	ENSG00000225233.1	ENSG00000237345.1	ENSG00000206503.13
25874	ENSG00000225234.1	ENSG00000237343.1	ENSG00000182533.6
25875	ENSG00000225235.1	ENSG00000237342.1	ENSG00000078589.13
25876	ENSG00000225236.1	ENSG00000237341.1	ENSG00000236051.7
25877	ENSG00000225238.1	ENSG00000237339.5	ENSG00000163554.13
25878	ENSG00000225239.1	ENSG00000237338.1	ENSG00000130958.13
25879	ENSG00000225240.1	ENSG00000237337.1	ENSG00000118046.15
25880	ENSG00000225242.2	ENSG00000237336.1	ENSG00000079337.16
25881	ENSG00000225243.5	ENSG00000237331.1	ENSG00000270647.6
25882	ENSG00000225244.3	ENSG00000237330.3	ENSG00000128394.17
25883	ENSG00000225246.1	ENSG00000237329.2	ENSG00000067225.18
25884	ENSG00000225249.5	ENSG00000237328.1	ENSG00000173846.13
25885	ENSG00000225251.1	ENSG00000237327.1	ENSG00000162620.16
25886	ENSG00000225253.1	ENSG00000237326.1	ENSG00000175344.17
25887	ENSG00000225254.2	ENSG00000237325.3	ENSG00000054282.16
25888	ENSG00000225255.6	ENSG00000237324.3	ENSG00000234338.1
25889	ENSG00000225256.1	ENSG00000237323.1	ENSG00000144120.13
25890	ENSG00000225258.1	ENSG00000237322.1	ENSG00000175970.11
25891	ENSG00000225259.4	ENSG00000237321.1	ENSG00000162980.17
25892	ENSG00000225263.1	ENSG00000237320.2	ENSG00000188452.14
25893	ENSG00000225264.3	ENSG00000237319.1	ENSG00000182676.5
25894	ENSG00000225265.1	ENSG00000237317.2	ENSG00000196782.12
25895	ENSG00000225267.1	ENSG00000237316.1	ENSG00000279203.1
25896	ENSG00000225269.2	ENSG00000237314.2	ENSG00000132141.14
25897	ENSG00000225270.1	ENSG00000237312.1	ENSG00000183281.14
25898	ENSG00000225271.1	ENSG00000237311.2	ENSG00000160783.19
25899	ENSG00000225272.2	ENSG00000237310.1	ENSG00000227939.1
25900	ENSG00000225275.4	ENSG00000237309.1	ENSG00000268750.6
25901	ENSG00000225276.1	ENSG00000237308.1	ENSG00000164187.7
25902	ENSG00000225279.1	ENSG00000237307.2	ENSG00000258644.5
25903	ENSG00000225280.6	ENSG00000237306.1	ENSG00000104472.10
25904	ENSG00000225282.1	ENSG00000237303.2	ENSG00000283389.1
25905	ENSG00000225284.1	ENSG00000237302.1	ENSG00000133105.8
25906	ENSG00000225285.1	ENSG00000237301.1	ENSG00000138080.13
25907	ENSG00000225286.1	ENSG00000237300.2	ENSG00000182742.6
25908	ENSG00000225287.1	ENSG00000237299.1	ENSG00000225806.8
25909	ENSG00000225289.1	ENSG00000237298.9	ENSG00000286011.1
25910	ENSG00000225292.2	ENSG00000237297.1	ENSG00000118418.14
25911	ENSG00000225293.1	ENSG00000237296.9	ENSG00000128596.17
25912	ENSG00000225294.1	ENSG00000237294.1	ENSG00000160255.18
25913	ENSG00000225295.1	ENSG00000237293.1	ENSG00000154027.19
25914	ENSG00000225297.1	ENSG00000237292.1	ENSG00000142408.4

25915	ENSG00000225298.5	ENSG00000237290.2	ENSG00000241990.5
25916	ENSG00000225299.1	ENSG00000237289.9	ENSG00000224023.10
25917	ENSG00000225300.1	ENSG00000237286.1	ENSG00000267605.6
25918	ENSG00000225303.2	ENSG00000237285.1	ENSG00000154240.17
25919	ENSG00000225304.1	ENSG00000237283.1	ENSG00000181019.13
25920	ENSG00000225308.2	ENSG00000237282.3	ENSG00000271976.1
25921	ENSG00000225310.1	ENSG00000237281.1	ENSG00000197323.12
25922	ENSG00000225311.1	ENSG00000237280.1	ENSG00000105053.11
25923	ENSG00000225313.5	ENSG00000237279.2	ENSG00000255158.1
25924	ENSG00000225314.1	ENSG00000237278.2	ENSG00000115758.13
25925	ENSG00000225315.2	ENSG00000237276.8	ENSG00000243964.1
25926	ENSG00000225316.2	ENSG00000237275.1	ENSG00000066923.17
25927	ENSG00000225321.2	ENSG00000237274.2	ENSG00000215251.4
25928	ENSG00000225323.1	ENSG00000237273.1	ENSG00000158246.8
25929	ENSG00000225325.1	ENSG00000237272.2	ENSG00000234073.1
25930	ENSG00000225326.1	ENSG00000237271.1	ENSG00000042980.12
25931	ENSG00000225327.3	ENSG00000237269.1	ENSG00000125498.20
25932	ENSG00000225328.1	ENSG00000237268.2	ENSG00000175707.9
25933	ENSG00000225329.3	ENSG00000237267.1	ENSG00000267077.1
25934	ENSG00000225330.1	ENSG00000237266.1	ENSG00000151376.16
25935	ENSG00000225331.1	ENSG00000237265.6	ENSG00000063177.13
25936	ENSG00000225333.6	ENSG00000237264.4	ENSG00000122299.12
25937	ENSG00000225334.2	ENSG00000237263.1	ENSG00000228274.3
25938	ENSG00000225335.3	ENSG00000237262.1	ENSG00000197181.12
25939	ENSG00000225336.2	ENSG00000237261.2	ENSG00000102032.13
25940	ENSG00000225337.1	ENSG00000237260.1	ENSG00000260784.1
25941	ENSG00000225338.1	ENSG00000237259.1	ENSG00000105982.16
25942	ENSG00000225339.3	ENSG00000237257.1	ENSG00000257509.1
25943	ENSG00000225341.1	ENSG00000237256.1	ENSG00000166503.9
25944	ENSG00000225342.2	ENSG00000237254.2	ENSG00000170627.11
25945	ENSG00000225343.1	ENSG00000237252.1	ENSG00000172183.15
25946	ENSG00000225344.1	ENSG00000237251.2	ENSG00000197747.9
25947	ENSG00000225345.3	ENSG00000237250.3	ENSG00000170835.14
25948	ENSG00000225347.1	ENSG00000237249.1	ENSG00000272632.1
25949	ENSG00000225349.2	ENSG00000237248.5	ENSG00000156928.4
25950	ENSG00000225350.1	ENSG00000237247.6	ENSG00000273013.1
25951	ENSG00000225352.1	ENSG00000237243.1	ENSG00000231806.3
25952	ENSG00000225354.1	ENSG00000237242.1	ENSG00000122068.13
25953	ENSG00000225355.1	ENSG00000237238.3	ENSG00000100426.7
25954	ENSG00000225356.3	ENSG00000237236.1	ENSG00000179253.3
25955	ENSG00000225357.3	ENSG00000237235.2	ENSG00000145244.12
25956	ENSG00000225358.1	ENSG00000237234.7	ENSG00000204228.4
25957	ENSG00000225359.1	ENSG00000237233.2	ENSG00000127418.15
25958	ENSG00000225360.1	ENSG00000237232.7	ENSG00000272396.1
25959	ENSG00000225361.3	ENSG00000237230.1	ENSG00000237343.1
25960	ENSG00000225362.9	ENSG00000237227.1	ENSG00000136521.13
25961	ENSG00000225364.1	ENSG00000237226.1	ENSG00000171451.14
25962	ENSG00000225365.1	ENSG00000237225.2	ENSG00000100354.21
25963	ENSG00000225366.4	ENSG00000237224.4	ENSG00000165626.17
25964	ENSG00000225369.1	ENSG00000237223.6	ENSG00000274180.1
25965	ENSG00000225370.1	ENSG00000237222.3	ENSG00000196663.16
25966	ENSG00000225371.1	ENSG00000237221.1	ENSG00000198162.12
25967	ENSG00000225376.5	ENSG00000237220.1	ENSG00000120265.17

25968	ENSG00000225377.5	ENSG00000237217.1	ENSG00000173418.11
25969	ENSG00000225378.1	ENSG00000237215.3	ENSG00000261135.1
25970	ENSG00000225380.4	ENSG00000237213.2	ENSG00000254910.1
25971	ENSG00000225381.2	ENSG00000237212.1	ENSG00000165125.20
25972	ENSG00000225383.7	ENSG00000237211.2	ENSG00000234618.1
25973	ENSG00000225384.1	ENSG00000237210.1	ENSG00000196588.16
25974	ENSG00000225385.3	ENSG00000237208.1	ENSG00000180229.12
25975	ENSG00000225386.1	ENSG00000237207.2	ENSG00000122034.15
25976	ENSG00000225387.1	ENSG00000237206.1	ENSG00000276965.1
25977	ENSG00000225391.5	ENSG00000237205.1	ENSG00000174788.10
25978	ENSG00000225392.1	ENSG00000237202.1	ENSG00000123444.14
25979	ENSG00000225393.1	ENSG00000237200.1	ENSG00000279381.1
25980	ENSG00000225394.1	ENSG00000237197.1	ENSG00000226937.9
25981	ENSG00000225396.5	ENSG00000237195.1	ENSG00000254539.1
25982	ENSG00000225397.1	ENSG00000237194.1	ENSG00000184221.13
25983	ENSG00000225398.3	ENSG00000237193.1	ENSG00000178217.14
25984	ENSG00000225399.4	ENSG00000237190.4	ENSG00000186076.5
25985	ENSG00000225400.1	ENSG00000237188.5	ENSG00000158747.14
25986	ENSG00000225401.2	ENSG00000237187.8	ENSG00000244731.8
25987	ENSG00000225402.1	ENSG00000237186.1	ENSG00000159352.15
25988	ENSG00000225404.2	ENSG00000237185.1	ENSG00000228572.7
25989	ENSG00000225405.3	ENSG00000237183.1	ENSG00000263307.1
25990	ENSG00000225406.1	ENSG00000237182.1	ENSG00000160959.8
25991	ENSG00000225407.3	ENSG00000237181.1	ENSG00000224829.3
25992	ENSG00000225408.1	ENSG00000237180.1	ENSG00000261366.1
25993	ENSG00000225411.3	ENSG00000237179.5	ENSG00000272323.1
25994	ENSG00000225413.2	ENSG00000237178.2	ENSG00000242692.1
25995	ENSG00000225415.2	ENSG00000237176.4	ENSG00000266907.1
25996	ENSG00000225416.1	ENSG00000237175.1	ENSG00000279539.1
25997	ENSG00000225417.1	ENSG00000237174.7	ENSG00000218809.1
25998	ENSG00000225418.1	ENSG00000237173.1	ENSG00000154146.13
25999	ENSG00000225419.1	ENSG00000237172.4	ENSG00000213854.3
26000	ENSG00000225420.1	ENSG00000237171.1	ENSG00000248309.7
26001	ENSG00000225421.2	ENSG00000237170.3	ENSG00000122694.16
26002	ENSG00000225422.4	ENSG00000237169.1	ENSG00000054148.17
26003	ENSG00000225423.1	ENSG00000237167.1	ENSG00000174004.6
26004	ENSG00000225424.1	ENSG00000237166.1	ENSG00000276903.1
26005	ENSG00000225427.1	ENSG00000237164.1	ENSG00000268366.1
26006	ENSG00000225428.1	ENSG00000237163.1	ENSG00000164509.14
26007	ENSG00000225431.1	ENSG00000237162.1	ENSG00000179057.13
26008	ENSG00000225433.3	ENSG00000237161.4	ENSG00000163950.13
26009	ENSG00000225434.2	ENSG00000237160.2	ENSG00000141905.19
26010	ENSG00000225437.5	ENSG00000237159.5	ENSG00000140950.16
26011	ENSG00000225438.1	ENSG00000237158.1	ENSG00000285531.1
26012	ENSG00000225439.2	ENSG00000237154.2	ENSG00000179611.3
26013	ENSG00000225442.2	ENSG00000237153.3	ENSG00000178715.8
26014	ENSG00000225443.1	ENSG00000237149.5	ENSG00000256852.1
26015	ENSG00000225444.1	ENSG00000237148.1	ENSG00000270175.1
26016	ENSG00000225446.2	ENSG00000237141.1	ENSG00000228998.4
26017	ENSG00000225447.1	ENSG00000237140.2	ENSG00000163125.15
26018	ENSG00000225448.1	ENSG00000237139.1	ENSG00000277972.1
26019	ENSG00000225449.3	ENSG00000237138.1	ENSG00000178695.5
26020	ENSG00000225450.1	ENSG00000237137.1	ENSG00000285936.1

26021	ENSG00000225451.1	ENSG00000237136.7	ENSG00000235663.1
26022	ENSG00000225455.3	ENSG00000237135.1	ENSG00000165671.20
26023	ENSG00000225457.1	ENSG00000237133.1	ENSG00000184515.11
26024	ENSG00000225458.1	ENSG00000237131.1	ENSG00000064961.19
26025	ENSG00000225460.1	ENSG00000237130.1	ENSG00000134780.10
26026	ENSG00000225462.1	ENSG00000237129.1	ENSG00000279394.1
26027	ENSG00000225463.1	ENSG00000237128.1	ENSG00000136381.13
26028	ENSG00000225465.8	ENSG00000237127.1	ENSG00000276119.1
26029	ENSG00000225466.1	ENSG00000237126.8	ENSG00000124762.13
26030	ENSG00000225469.3	ENSG00000237125.9	ENSG00000283444.1
26031	ENSG00000225470.7	ENSG00000237124.1	ENSG00000121274.12
26032	ENSG00000225471.5	ENSG00000237121.1	ENSG00000205853.10
26033	ENSG00000225472.1	ENSG00000237119.1	ENSG00000286128.1
26034	ENSG00000225473.1	ENSG00000237118.3	ENSG00000237827.1
26035	ENSG00000225475.1	ENSG00000237115.2	ENSG00000275956.1
26036	ENSG00000225476.1	ENSG00000237111.1	ENSG00000183682.8
26037	ENSG00000225477.3	ENSG00000237110.2	ENSG00000261207.1
26038	ENSG00000225478.2	ENSG00000237109.1	ENSG00000277149.5
26039	ENSG00000225479.1	ENSG00000237107.3	ENSG00000132640.15
26040	ENSG00000225480.1	ENSG00000237106.2	ENSG00000212802.4
26041	ENSG00000225482.1	ENSG00000237101.1	ENSG00000278996.1
26042	ENSG00000225483.1	ENSG00000237099.1	ENSG00000128040.11
26043	ENSG00000225484.6	ENSG00000237094.12	ENSG00000108666.10
26044	ENSG00000225486.1	ENSG00000237092.1	ENSG00000278881.2
26045	ENSG00000225487.2	ENSG00000237090.1	ENSG00000251230.5
26046	ENSG00000225488.2	ENSG00000237089.2	ENSG00000248489.1
26047	ENSG00000225489.6	ENSG00000237088.1	ENSG00000223508.5
26048	ENSG00000225491.1	ENSG00000237087.1	ENSG00000141198.16
26049	ENSG00000225492.6	ENSG00000237085.2	ENSG00000280320.1
26050	ENSG00000225493.1	ENSG00000237083.1	ENSG00000238201.1
26051	ENSG00000225496.1	ENSG00000237082.1	ENSG00000033122.19
26052	ENSG00000225497.5	ENSG00000237080.3	ENSG00000135469.13
26053	ENSG00000225498.1	ENSG00000237077.1	ENSG00000135070.15
26054	ENSG00000225499.1	ENSG00000237076.1	ENSG00000158019.20
26055	ENSG00000225501.1	ENSG00000237074.2	ENSG00000180448.10
26056	ENSG00000225502.2	ENSG00000237073.1	ENSG00000277443.3
26057	ENSG00000225505.1	ENSG00000237072.1	ENSG00000153558.15
26058	ENSG00000225506.2	ENSG00000237070.1	ENSG00000227946.1
26059	ENSG00000225507.1	ENSG00000237068.1	ENSG00000116688.16
26060	ENSG00000225508.1	ENSG00000237065.2	ENSG00000162300.13
26061	ENSG00000225509.1	ENSG00000237064.1	ENSG00000125740.14
26062	ENSG00000225510.2	ENSG00000237063.1	ENSG00000172000.7
26063	ENSG00000225511.7	ENSG00000237062.1	ENSG00000158545.15
26064	ENSG00000225513.1	ENSG00000237061.1	ENSG00000157111.13
26065	ENSG00000225514.1	ENSG00000237058.1	ENSG00000118096.7
26066	ENSG00000225516.7	ENSG00000237057.2	ENSG00000256463.8
26067	ENSG00000225518.2	ENSG00000237055.1	ENSG00000143799.13
26068	ENSG00000225519.1	ENSG00000237054.9	ENSG00000185130.5
26069	ENSG00000225521.1	ENSG00000237053.1	ENSG00000271461.1
26070	ENSG00000225522.2	ENSG00000237049.1	ENSG00000283959.1
26071	ENSG00000225523.2	ENSG00000237048.3	ENSG00000225303.2
26072	ENSG00000225526.4	ENSG00000237042.1	ENSG00000196659.9
26073	ENSG00000225527.1	ENSG00000237041.1	ENSG00000172738.12

26074	ENSG00000225528.3	ENSG00000237040.6_PAR_Y	ENSG00000050628.20
26075	ENSG00000225530.1	ENSG00000237040.6	ENSG00000278811.4
26076	ENSG00000225531.1	ENSG00000237039.1	ENSG00000267198.1
26077	ENSG00000225532.1	ENSG00000237038.5	ENSG00000272226.1
26078	ENSG00000225533.1	ENSG00000237037.9	ENSG00000099251.14
26079	ENSG00000225535.6	ENSG00000237035.1	ENSG00000284196.2
26080	ENSG00000225536.2	ENSG00000237033.2	ENSG00000131437.15
26081	ENSG00000225537.1	ENSG00000237032.1	ENSG00000185813.10
26082	ENSG00000225538.2	ENSG00000237031.7	ENSG00000203797.10
26083	ENSG00000225539.6	ENSG00000237027.1	ENSG00000131558.15
26084	ENSG00000225542.1	ENSG00000237026.1	ENSG00000100908.14
26085	ENSG00000225544.1	ENSG00000237025.1	ENSG00000236137.1
26086	ENSG00000225545.1	ENSG00000237024.1	ENSG00000215343.7
26087	ENSG00000225546.5	ENSG00000237023.1	ENSG00000172197.10
26088	ENSG00000225548.6	ENSG00000237020.1	ENSG00000183662.11
26089	ENSG00000225549.3	ENSG00000237019.1	ENSG00000181045.15
26090	ENSG00000225551.1	ENSG00000237017.1	ENSG00000265690.7
26091	ENSG00000225552.1	ENSG00000237016.1	ENSG00000225698.3
26092	ENSG00000225554.1	ENSG00000237015.1	ENSG00000153561.13
26093	ENSG00000225555.1	ENSG00000237014.1	ENSG00000257261.5
26094	ENSG00000225556.1	ENSG00000237013.1	ENSG00000260910.1
26095	ENSG00000225557.1	ENSG00000237011.1	ENSG00000230155.6
26096	ENSG00000225558.1	ENSG00000237009.2	ENSG00000182923.17
26097	ENSG00000225559.1	ENSG00000237008.2	ENSG00000213366.13
26098	ENSG00000225560.6	ENSG00000237007.4	ENSG00000172985.10
26099	ENSG00000225561.1	ENSG00000237005.1	ENSG00000276957.1
26100	ENSG00000225563.1	ENSG00000237004.4	ENSG00000228665.2
26101	ENSG00000225564.6	ENSG00000237003.1	ENSG00000135272.10
26102	ENSG00000225568.1	ENSG00000237002.6	ENSG00000251015.1
26103	ENSG00000225569.1	ENSG00000237001.6	ENSG00000103852.13
26104	ENSG00000225570.1	ENSG00000237000.1	ENSG00000133121.21
26105	ENSG00000225572.1	ENSG00000236999.1	ENSG00000251345.2
26106	ENSG00000225573.4	ENSG00000236998.2	ENSG00000204149.11
26107	ENSG00000225574.1	ENSG00000236996.1	ENSG00000136286.16
26108	ENSG00000225578.1	ENSG00000236994.1	ENSG00000275221.1
26109	ENSG00000225579.1	ENSG00000236993.2	ENSG00000267424.1
26110	ENSG00000225580.2	ENSG00000236992.2	ENSG00000124104.19
26111	ENSG00000225581.3	ENSG00000236991.6	ENSG00000079313.15
26112	ENSG00000225582.1	ENSG00000236990.1	ENSG00000225422.4
26113	ENSG00000225583.1	ENSG00000236989.5	ENSG00000276651.1
26114	ENSG00000225585.1	ENSG00000236988.2	ENSG00000280035.1
26115	ENSG00000225588.2	ENSG00000236987.2	ENSG00000260160.1
26116	ENSG00000225591.2	ENSG00000236986.6	ENSG00000186577.14
26117	ENSG00000225594.1	ENSG00000236985.1	ENSG00000100439.10
26118	ENSG00000225595.2	ENSG00000236982.1	ENSG00000013503.10
26119	ENSG00000225598.1	ENSG00000236981.1	ENSG00000210135.1
26120	ENSG00000225602.5	ENSG00000236980.9	ENSG00000279088.1
26121	ENSG00000225603.3	ENSG00000236978.1	ENSG00000005469.11
26122	ENSG00000225605.2	ENSG00000236977.1	ENSG00000183722.9
26123	ENSG00000225606.1	ENSG00000236976.1	ENSG00000248712.7
26124	ENSG00000225609.1	ENSG00000236975.1	ENSG00000117425.14
26125	ENSG00000225610.1	ENSG00000236973.2	ENSG00000104969.10
26126	ENSG00000225611.1	ENSG00000236972.2	ENSG00000229233.1

26127	ENSG00000225612.1	ENSG00000236969.2	ENSG00000147100.11
26128	ENSG00000225613.2	ENSG00000236968.1	ENSG00000166924.9
26129	ENSG00000225614.2	ENSG00000236966.1	ENSG00000166979.13
26130	ENSG00000225615.1	ENSG00000236965.4	ENSG00000234589.4
26131	ENSG00000225616.2	ENSG00000236963.6	ENSG00000100151.16
26132	ENSG00000225619.1	ENSG00000236961.1	ENSG00000127191.18
26133	ENSG00000225620.1	ENSG00000236960.1	ENSG00000050438.17
26134	ENSG00000225622.1	ENSG00000236959.1	ENSG00000215482.3
26135	ENSG00000225623.1	ENSG00000236958.1	ENSG00000280088.1
26136	ENSG00000225624.1	ENSG00000236957.7	ENSG00000107130.10
26137	ENSG00000225625.4	ENSG00000236956.2	ENSG00000235072.2
26138	ENSG00000225626.3	ENSG00000236953.1	ENSG00000260597.1
26139	ENSG00000225630.1	ENSG00000236951.5	ENSG00000150456.10
26140	ENSG00000225632.1	ENSG00000236950.1	ENSG00000269984.1
26141	ENSG00000225636.1	ENSG00000236948.2	ENSG00000212127.5
26142	ENSG00000225637.1	ENSG00000236947.5	ENSG00000141759.15
26143	ENSG00000225638.1	ENSG00000236946.2	ENSG00000232407.3
26144	ENSG00000225639.1	ENSG00000236944.1	ENSG00000124766.6
26145	ENSG00000225640.1	ENSG00000236941.6	ENSG00000125149.11
26146	ENSG00000225642.1	ENSG00000236940.1	ENSG00000198785.5
26147	ENSG00000225643.1	ENSG00000236939.2	ENSG00000222421.1
26148	ENSG00000225644.1	ENSG00000236938.1	ENSG00000274897.3
26149	ENSG00000225647.1	ENSG00000236937.2	ENSG00000167105.8
26150	ENSG00000225648.5	ENSG00000236936.1	ENSG00000262663.1
26151	ENSG00000225649.5	ENSG00000236935.1	ENSG00000280327.1
26152	ENSG00000225650.2	ENSG00000236932.2	ENSG00000214894.6
26153	ENSG00000225652.1	ENSG00000236930.1	ENSG00000147174.11
26154	ENSG00000225653.1	ENSG00000236929.2	ENSG00000164209.17
26155	ENSG00000225655.5	ENSG00000236928.3	ENSG00000222041.11
26156	ENSG00000225656.1	ENSG00000236924.1	ENSG00000164440.15
26157	ENSG00000225657.2	ENSG00000236922.9	ENSG00000133243.8
26158	ENSG00000225658.1	ENSG00000236921.1	ENSG00000229036.7
26159	ENSG00000225660.1	ENSG00000236920.2	ENSG00000173156.7
26160	ENSG00000225661.7	ENSG00000236919.3	ENSG00000272682.1
26161	ENSG00000225661.7_PAR_Y	ENSG00000236917.1	ENSG00000089818.18
26162	ENSG00000225662.2	ENSG00000236915.2	ENSG00000103313.12
26163	ENSG00000225663.7	ENSG00000236914.3	ENSG00000135250.17
26164	ENSG00000225664.3	ENSG00000236913.1	ENSG00000096968.13
26165	ENSG00000225665.4	ENSG00000236911.6	ENSG00000148297.15
26166	ENSG00000225669.1	ENSG00000236909.1	ENSG00000275832.5
26167	ENSG00000225670.4	ENSG00000236908.2	ENSG00000136643.12
26168	ENSG00000225671.2	ENSG00000236907.1	ENSG00000124164.15
26169	ENSG00000225672.1	ENSG00000236905.2	ENSG00000246174.7
26170	ENSG00000225673.3	ENSG00000236901.6	ENSG00000189376.12
26171	ENSG00000225674.1	ENSG00000236900.1	ENSG00000266075.2
26172	ENSG00000225675.2	ENSG00000236897.1	ENSG00000261003.1
26173	ENSG00000225676.1	ENSG00000236896.1	ENSG00000168591.16
26174	ENSG00000225680.1	ENSG00000236894.3	ENSG00000114120.12
26175	ENSG00000225681.2	ENSG00000236893.1	ENSG00000099889.14
26176	ENSG00000225683.5	ENSG00000236892.1	ENSG00000203972.10
26177	ENSG00000225684.3	ENSG00000236890.2	ENSG00000237438.7
26178	ENSG00000225685.1	ENSG00000236889.1	ENSG00000272341.1
26179	ENSG00000225689.1	ENSG00000236888.1	ENSG00000119041.11

26180	ENSG00000225690.1	ENSG00000236887.1	ENSG00000144152.13
26181	ENSG00000225693.1	ENSG00000236886.2	ENSG00000154764.5
26182	ENSG00000225695.1	ENSG00000236885.2	ENSG00000155980.12
26183	ENSG00000225697.13	ENSG00000236883.1	ENSG00000166347.19
26184	ENSG00000225698.3	ENSG00000236882.7	ENSG00000034053.14
26185	ENSG00000225701.1	ENSG00000236880.1	ENSG00000232858.1
26186	ENSG00000225703.1	ENSG00000236878.1	ENSG00000151117.9
26187	ENSG00000225705.2	ENSG00000236877.2	ENSG00000170271.11
26188	ENSG00000225706.1	ENSG00000236876.3	ENSG00000272112.1
26189	ENSG00000225708.6	ENSG00000236875.3	ENSG00000280183.1
26190	ENSG00000225710.1	ENSG00000236874.1	ENSG00000233967.6
26191	ENSG00000225711.1	ENSG00000236872.1	ENSG00000237489.5
26192	ENSG00000225712.1	ENSG00000236871.7_PAR_Y	ENSG00000242220.7
26193	ENSG00000225713.5	ENSG00000236871.7	ENSG00000083817.9
26194	ENSG00000225715.1	ENSG00000236870.2	ENSG00000105246.6
26195	ENSG00000225716.1	ENSG00000236869.1	ENSG00000222009.8
26196	ENSG00000225718.1	ENSG00000236867.1	ENSG00000104953.20
26197	ENSG00000225719.2	ENSG00000236866.5	ENSG00000280173.1
26198	ENSG00000225720.6	ENSG00000236864.1	ENSG00000253214.1
26199	ENSG00000225721.5	ENSG00000236863.2	ENSG00000160396.8
26200	ENSG00000225722.1	ENSG00000236862.1	ENSG00000112541.14
26201	ENSG00000225723.1	ENSG00000236861.6	ENSG00000235299.2
26202	ENSG00000225724.1	ENSG00000236860.1	ENSG00000270264.1
26203	ENSG00000225725.4	ENSG00000236859.6	ENSG00000179943.8
26204	ENSG00000225726.1	ENSG00000236858.1	ENSG00000109674.4
26205	ENSG00000225727.3	ENSG00000236857.3	ENSG00000225401.2
26206	ENSG00000225728.2	ENSG00000236856.1	ENSG00000187953.10
26207	ENSG00000225730.1	ENSG00000236854.1	ENSG00000109332.20
26208	ENSG00000225731.1	ENSG00000236853.2	ENSG00000237943.6
26209	ENSG00000225733.5	ENSG00000236852.1	ENSG00000204850.4
26210	ENSG00000225735.1	ENSG00000236849.5	ENSG00000273472.1
26211	ENSG00000225738.2	ENSG00000236848.2	ENSG00000130656.5
26212	ENSG00000225739.1	ENSG00000236847.1	ENSG00000185909.15
26213	ENSG00000225740.1	ENSG00000236846.1	ENSG00000102837.7
26214	ENSG00000225741.1	ENSG00000236844.1	ENSG00000230805.6
26215	ENSG00000225742.5	ENSG00000236842.1	ENSG00000171843.16
26216	ENSG00000225744.1	ENSG00000236841.7	ENSG00000279631.1
26217	ENSG00000225746.11	ENSG00000236839.1	ENSG00000187764.11
26218	ENSG00000225750.1	ENSG00000236838.2	ENSG00000225377.5
26219	ENSG00000225751.2	ENSG00000236837.1	ENSG00000224877.4
26220	ENSG00000225752.1	ENSG00000236836.1	ENSG00000130383.7
26221	ENSG00000225755.2	ENSG00000236834.1	ENSG00000160326.14
26222	ENSG00000225756.1	ENSG00000236833.1	ENSG00000114784.4
26223	ENSG00000225758.1	ENSG00000236832.1	ENSG00000147548.17
26224	ENSG00000225759.1	ENSG00000236831.1	ENSG00000255970.1
26225	ENSG00000225760.2	ENSG00000236830.6	ENSG00000235002.1
26226	ENSG00000225761.1	ENSG00000236829.9	ENSG00000259211.1
26227	ENSG00000225762.1	ENSG00000236828.1	ENSG00000262061.5
26228	ENSG00000225764.1	ENSG00000236827.1	ENSG00000261606.5
26229	ENSG00000225765.1	ENSG00000236825.2	ENSG00000168286.2
26230	ENSG00000225766.10	ENSG00000236824.2	ENSG00000100239.16
26231	ENSG00000225767.1	ENSG00000236823.1	ENSG00000213079.9
26232	ENSG00000225768.1	ENSG00000236822.1	ENSG00000279048.1

26233	ENSG00000225769.1	ENSG00000236819.1	ENSG00000136830.12
26234	ENSG00000225770.1	ENSG00000236818.1	ENSG00000232748.3
26235	ENSG00000225774.2	ENSG00000236817.5	ENSG00000285077.2
26236	ENSG00000225775.1	ENSG00000236816.2	ENSG00000140511.11
26237	ENSG00000225777.1	ENSG00000236814.1	ENSG00000108175.17
26238	ENSG00000225778.5	ENSG00000236813.1	ENSG00000277383.1
26239	ENSG00000225779.1	ENSG00000236811.1	ENSG00000127920.5
26240	ENSG00000225781.1	ENSG00000236810.5	ENSG00000279349.1
26241	ENSG00000225783.7	ENSG00000236809.2	ENSG00000177103.14
26242	ENSG00000225785.1	ENSG00000236807.1	ENSG00000277075.2
26243	ENSG00000225787.1	ENSG00000236806.1	ENSG00000186591.12
26244	ENSG00000225790.1	ENSG00000236804.1	ENSG00000221883.3
26245	ENSG00000225791.6	ENSG00000236803.1	ENSG00000080031.10
26246	ENSG00000225792.1	ENSG00000236801.1	ENSG00000176105.14
26247	ENSG00000225793.2	ENSG00000236800.1	ENSG00000064666.15
26248	ENSG00000225794.2	ENSG00000236799.1	ENSG00000256664.1
26249	ENSG00000225795.1	ENSG00000236797.1	ENSG00000117036.12
26250	ENSG00000225796.2	ENSG00000236796.1	ENSG00000211713.3
26251	ENSG00000225797.2	ENSG00000236795.1	ENSG00000148841.17
26252	ENSG00000225798.1	ENSG00000236794.5	ENSG00000182150.15
26253	ENSG00000225801.1	ENSG00000236792.1	ENSG00000262873.1
26254	ENSG00000225802.3	ENSG00000236791.2	ENSG00000122877.16
26255	ENSG00000225803.1	ENSG00000236790.5	ENSG00000143434.15
26256	ENSG00000225805.4	ENSG00000236786.1	ENSG00000182257.8
26257	ENSG00000225806.8	ENSG00000236785.1	ENSG00000151176.8
26258	ENSG00000225807.1	ENSG00000236783.1	ENSG00000187778.14
26259	ENSG00000225808.1	ENSG00000236782.7	ENSG00000261118.1
26260	ENSG00000225809.1	ENSG00000236780.6	ENSG00000105991.8
26261	ENSG00000225811.1	ENSG00000236779.1	ENSG00000158169.13
26262	ENSG00000225813.1	ENSG00000236778.7	ENSG00000278831.1
26263	ENSG00000225814.1	ENSG00000236777.1	ENSG00000236256.9
26264	ENSG00000225815.2	ENSG00000236776.1	ENSG00000225695.1
26265	ENSG00000225816.1	ENSG00000236775.1	ENSG00000170191.5
26266	ENSG00000225818.1	ENSG00000236773.1	ENSG00000230438.6
26267	ENSG00000225819.1	ENSG00000236772.1	ENSG00000269755.1
26268	ENSG00000225822.4	ENSG00000236770.1	ENSG00000100916.14
26269	ENSG00000225823.1	ENSG00000236769.2	ENSG00000164106.8
26270	ENSG00000225825.1	ENSG00000236768.1	ENSG00000215424.9
26271	ENSG00000225826.1	ENSG00000236764.4	ENSG00000227527.2
26272	ENSG00000225827.1	ENSG00000236763.2	ENSG00000139624.12
26273	ENSG00000225828.1	ENSG00000236762.1	ENSG00000284690.2
26274	ENSG00000225830.13	ENSG00000236761.5	ENSG00000106636.8
26275	ENSG00000225831.1	ENSG00000236760.1	ENSG00000283537.2
26276	ENSG00000225832.1	ENSG00000236758.5	ENSG00000087253.13
26277	ENSG00000225833.1	ENSG00000236756.4	ENSG00000198721.12
26278	ENSG00000225836.1	ENSG00000236754.5	ENSG00000272716.1
26279	ENSG00000225839.2	ENSG00000236751.1	ENSG00000050030.15
26280	ENSG00000225840.2	ENSG00000236750.1	ENSG00000285824.1
26281	ENSG00000225842.1	ENSG00000236748.1	ENSG00000196220.16
26282	ENSG00000225843.1	ENSG00000236747.2	ENSG00000163827.13
26283	ENSG00000225846.4	ENSG00000236745.2	ENSG00000142784.16
26284	ENSG00000225849.2	ENSG00000236744.2	ENSG00000085365.18
26285	ENSG00000225850.3	ENSG00000236741.4	ENSG00000275709.1

26286	ENSG00000225851.1	ENSG00000236740.6	ENSG00000155363.18
26287	ENSG00000225854.2	ENSG00000236739.3	ENSG00000276141.4
26288	ENSG00000225855.6	ENSG00000236737.1	ENSG00000275757.1
26289	ENSG00000225856.2	ENSG00000236736.1	ENSG00000170412.17
26290	ENSG00000225857.5	ENSG00000236733.1	ENSG00000177359.20
26291	ENSG00000225858.1	ENSG00000236732.1	ENSG00000106404.13
26292	ENSG00000225864.1	ENSG00000236731.1	ENSG00000079950.14
26293	ENSG00000225867.1	ENSG00000236724.1	ENSG00000267046.1
26294	ENSG00000225868.7	ENSG00000236723.2	ENSG00000158517.13
26295	ENSG00000225869.4	ENSG00000236722.1	ENSG00000253742.1
26296	ENSG00000225870.1	ENSG00000236720.1	ENSG00000279586.1
26297	ENSG00000225871.2	ENSG00000236719.3	ENSG00000254415.3
26298	ENSG00000225872.3	ENSG00000236718.4	ENSG00000239617.1
26299	ENSG00000225873.4	ENSG00000236717.2	ENSG00000172932.14
26300	ENSG00000225876.1	ENSG00000236716.1	ENSG00000235066.7
26301	ENSG00000225877.1	ENSG00000236714.2	ENSG00000269506.2
26302	ENSG00000225878.1	ENSG00000236713.1	ENSG00000254811.5
26303	ENSG00000225879.1	ENSG00000236712.1	ENSG00000143753.13
26304	ENSG00000225880.5	ENSG00000236711.2	ENSG00000101298.15
26305	ENSG00000225881.1	ENSG00000236710.1	ENSG00000267060.5
26306	ENSG00000225882.1	ENSG00000236709.1	ENSG00000206562.11
26307	ENSG00000225883.2	ENSG00000236708.1	ENSG00000263006.6
26308	ENSG00000225884.2	ENSG00000236704.1	ENSG00000137672.13
26309	ENSG00000225885.7	ENSG00000236703.1	ENSG00000125691.13
26310	ENSG00000225886.3	ENSG00000236701.3	ENSG00000118407.15
26311	ENSG00000225889.7	ENSG00000236700.5	ENSG00000204947.9
26312	ENSG00000225891.1	ENSG00000236699.9	ENSG00000204351.12
26313	ENSG00000225893.1	ENSG00000236698.1	ENSG00000116747.12
26314	ENSG00000225895.1	ENSG00000236695.1	ENSG00000259673.5
26315	ENSG00000225896.1	ENSG00000236692.2	ENSG00000272518.1
26316	ENSG00000225898.1	ENSG00000236691.3	ENSG00000088367.22
26317	ENSG00000225899.7	ENSG00000236690.2	ENSG00000162378.13
26318	ENSG00000225900.1	ENSG00000236689.1	ENSG00000244327.1
26319	ENSG00000225901.1	ENSG00000236687.1	ENSG00000280063.1
26320	ENSG00000225903.1	ENSG00000236686.1	ENSG00000100207.18
26321	ENSG00000225904.1	ENSG00000236683.3	ENSG00000135127.11
26322	ENSG00000225905.1	ENSG00000236682.1	ENSG00000138246.17
26323	ENSG00000225906.1	ENSG00000236681.1	ENSG00000148356.13
26324	ENSG00000225907.1	ENSG00000236680.1	ENSG00000225329.3
26325	ENSG00000225911.1	ENSG00000236679.2	ENSG00000274214.1
26326	ENSG00000225912.1	ENSG00000236678.7	ENSG00000077150.19
26327	ENSG00000225913.2	ENSG00000236677.1	ENSG00000163605.14
26328	ENSG00000225914.2	ENSG00000236676.1	ENSG00000166349.9
26329	ENSG00000225916.1	ENSG00000236675.1	ENSG00000144852.17
26330	ENSG00000225918.1	ENSG00000236674.1	ENSG00000168439.16
26331	ENSG00000225919.1	ENSG00000236673.5	ENSG00000228427.2
26332	ENSG00000225920.2	ENSG00000236671.8	ENSG00000279259.1
26333	ENSG00000225921.7	ENSG00000236670.1	ENSG00000172728.15
26334	ENSG00000225922.1	ENSG00000236668.2	ENSG00000142655.13
26335	ENSG00000225923.1	ENSG00000236667.1	ENSG00000188177.14
26336	ENSG00000225924.2	ENSG00000236666.1	ENSG00000257221.3
26337	ENSG00000225925.1	ENSG00000236665.1	ENSG00000264247.1
26338	ENSG00000225928.2	ENSG00000236664.1	ENSG00000104205.14

26339	ENSG00000225929.1	ENSG00000236663.1	ENSG00000085415.16
26340	ENSG00000225930.4	ENSG00000236662.1	ENSG00000143761.16
26341	ENSG00000225931.3	ENSG00000236658.1	ENSG00000095564.14
26342	ENSG00000225932.3	ENSG00000236656.1	ENSG00000137880.6
26343	ENSG00000225933.1	ENSG00000236655.1	ENSG00000279328.1
26344	ENSG00000225934.2	ENSG00000236654.2	ENSG00000277785.1
26345	ENSG00000225935.1	ENSG00000236651.1	ENSG00000259165.1
26346	ENSG00000225936.1	ENSG00000236648.1	ENSG00000137414.6
26347	ENSG00000225937.2	ENSG00000236647.1	ENSG00000166813.15
26348	ENSG00000225938.1	ENSG00000236646.1	ENSG00000253764.2
26349	ENSG00000225940.6	ENSG00000236643.1	ENSG00000282608.1
26350	ENSG00000225942.1	ENSG00000236641.2	ENSG00000259498.1
26351	ENSG00000225943.1	ENSG00000236638.1	ENSG00000112419.14
26352	ENSG00000225944.1	ENSG00000236637.4	ENSG00000157693.15
26353	ENSG00000225945.1	ENSG00000236636.2	ENSG00000254064.1
26354	ENSG00000225947.1	ENSG00000236635.2	ENSG00000196547.15
26355	ENSG00000225948.2	ENSG00000236634.1	ENSG00000137331.12
26356	ENSG00000225949.1	ENSG00000236627.1	ENSG00000101191.16
26357	ENSG00000225950.8	ENSG00000236626.1	ENSG00000222276.1
26358	ENSG00000225951.1	ENSG00000236620.1	ENSG00000277566.1
26359	ENSG00000225952.1	ENSG00000236618.2	ENSG00000201967.1
26360	ENSG00000225953.2	ENSG00000236617.2	ENSG00000188039.14
26361	ENSG00000225956.1	ENSG00000236616.3	ENSG00000140406.3
26362	ENSG00000225957.1	ENSG00000236615.1	ENSG00000235408.5
26363	ENSG00000225959.1	ENSG00000236612.1	ENSG00000197548.12
26364	ENSG00000225960.1	ENSG00000236611.1	ENSG00000149930.18
26365	ENSG00000225963.7	ENSG00000236610.1	ENSG00000110455.13
26366	ENSG00000225964.5	ENSG00000236609.4	ENSG00000008283.16
26367	ENSG00000225965.1	ENSG00000236608.1	ENSG00000004478.8
26368	ENSG00000225968.7	ENSG00000236607.3	ENSG00000158315.11
26369	ENSG00000225969.2	ENSG00000236606.1	ENSG00000155304.6
26370	ENSG00000225970.1	ENSG00000236605.1	ENSG00000215267.8
26371	ENSG00000225971.1	ENSG00000236604.1	ENSG00000196757.8
26372	ENSG00000225972.1	ENSG00000236603.2	ENSG00000249502.2
26373	ENSG00000225973.4	ENSG00000236601.2	ENSG00000187122.17
26374	ENSG00000225974.1	ENSG00000236599.1	ENSG00000138448.12
26375	ENSG00000225975.6	ENSG00000236597.1	ENSG00000211752.3
26376	ENSG00000225976.5	ENSG00000236596.1	ENSG00000261655.1
26377	ENSG00000225978.3	ENSG00000236595.1	ENSG00000261684.2
26378	ENSG00000225979.1	ENSG00000236594.2	ENSG00000102349.18
26379	ENSG00000225980.4	ENSG00000236592.5	ENSG00000274372.4
26380	ENSG00000225981.1	ENSG00000236591.1	ENSG00000167775.11
26381	ENSG00000225982.1	ENSG00000236590.1	ENSG00000179428.2
26382	ENSG00000225984.1	ENSG00000236583.1	ENSG00000104131.13
26383	ENSG00000225986.1	ENSG00000236582.1	ENSG00000104833.12
26384	ENSG00000225988.1	ENSG00000236581.8	ENSG00000274523.5
26385	ENSG00000225990.2	ENSG00000236580.1	ENSG00000233013.10
26386	ENSG00000225991.1	ENSG00000236576.1	ENSG00000284673.1
26387	ENSG00000225992.1	ENSG00000236574.1	ENSG00000248529.5
26388	ENSG00000225997.1	ENSG00000236572.1	ENSG00000279554.1
26389	ENSG00000225999.1	ENSG00000236571.1	ENSG00000111581.10
26390	ENSG00000226002.1	ENSG00000236570.1	ENSG00000101898.6
26391	ENSG00000226003.2	ENSG00000236569.3	ENSG00000167615.16

26392	ENSG00000226004.1	ENSG00000236567.2	ENSG00000277548.1
26393	ENSG00000226005.4	ENSG00000236565.3	ENSG00000264695.1
26394	ENSG00000226007.2	ENSG00000236564.1	ENSG00000108592.17
26395	ENSG00000226008.1	ENSG00000236562.4	ENSG00000158555.15
26396	ENSG00000226009.1	ENSG00000236559.1	ENSG00000253651.1
26397	ENSG00000226010.1	ENSG00000236556.1	ENSG00000165672.7
26398	ENSG00000226011.1	ENSG00000236555.1	ENSG00000198496.11
26399	ENSG00000226012.1	ENSG00000236554.1	ENSG00000272810.1
26400	ENSG00000226013.1	ENSG00000236552.2	ENSG00000228172.5
26401	ENSG00000226014.1	ENSG00000236550.1	ENSG00000177453.7
26402	ENSG00000226015.2	ENSG00000236549.1	ENSG00000134184.12
26403	ENSG00000226016.1	ENSG00000236548.1	ENSG00000263234.1
26404	ENSG00000226017.2	ENSG00000236546.1	ENSG00000286116.1
26405	ENSG00000226020.4	ENSG00000236545.1	ENSG00000178404.9
26406	ENSG00000226022.1	ENSG00000236544.1	ENSG00000067057.17
26407	ENSG00000226023.6	ENSG00000236543.2	ENSG00000118363.12
26408	ENSG00000226024.1	ENSG00000236542.1	ENSG00000182578.13
26409	ENSG00000226025.9	ENSG00000236541.1	ENSG00000271725.1
26410	ENSG00000226026.5	ENSG00000236540.7	ENSG00000184313.20
26411	ENSG00000226028.2	ENSG00000236539.3	ENSG00000146701.12
26412	ENSG00000226029.1	ENSG00000236538.1	ENSG00000251032.1
26413	ENSG00000226030.1	ENSG00000236536.1	ENSG00000233927.5
26414	ENSG00000226031.5	ENSG00000236535.1	ENSG00000235859.5
26415	ENSG00000226032.2	ENSG00000236534.1	ENSG00000214283.4
26416	ENSG00000226034.2	ENSG00000236533.1	ENSG00000264364.3
26417	ENSG00000226036.1	ENSG00000236532.5	ENSG00000254717.2
26418	ENSG00000226037.1	ENSG00000236531.2	ENSG00000107902.14
26419	ENSG00000226038.5	ENSG00000236530.2	ENSG00000279968.2
26420	ENSG00000226040.3	ENSG00000236529.1	ENSG00000240695.1
26421	ENSG00000226041.1	ENSG00000236528.1	ENSG00000148339.12
26422	ENSG00000226042.1	ENSG00000236527.1	ENSG00000236278.2
26423	ENSG00000226043.1	ENSG00000236526.1	ENSG00000276291.5
26424	ENSG00000226045.1	ENSG00000236525.1	ENSG00000178631.7
26425	ENSG00000226046.1	ENSG00000236523.2	ENSG00000196388.8
26426	ENSG00000226047.1	ENSG00000236521.1	ENSG00000105088.8
26427	ENSG00000226048.1	ENSG00000236520.2	ENSG00000118507.17
26428	ENSG00000226049.3	ENSG00000236519.1	ENSG00000196081.9
26429	ENSG00000226051.7	ENSG00000236516.1	ENSG00000272335.1
26430	ENSG00000226053.1	ENSG00000236514.1	ENSG00000279245.1
26431	ENSG00000226054.2	ENSG00000236513.1	ENSG00000273154.3
26432	ENSG00000226055.2	ENSG00000236512.1	ENSG00000245466.1
26433	ENSG00000226056.1	ENSG00000236511.1	ENSG00000273768.1
26434	ENSG00000226057.7	ENSG00000236510.1	ENSG00000229376.3
26435	ENSG00000226058.1	ENSG00000236509.2	ENSG00000175283.7
26436	ENSG00000226059.2	ENSG00000236508.1	ENSG00000276700.1
26437	ENSG00000226061.1	ENSG00000236507.1	ENSG00000080573.7
26438	ENSG00000226063.1	ENSG00000236505.1	ENSG00000166333.13
26439	ENSG00000226064.1	ENSG00000236504.2	ENSG00000132554.20
26440	ENSG00000226065.1	ENSG00000236503.1	ENSG00000178896.9
26441	ENSG00000226066.1	ENSG00000236502.1	ENSG00000204152.11
26442	ENSG00000226067.6	ENSG00000236501.5	ENSG00000276148.1
26443	ENSG00000226068.1	ENSG00000236500.1	ENSG00000265411.1
26444	ENSG00000226070.1	ENSG00000236499.2	ENSG00000174125.8

26445	ENSG00000226072.1	ENSG00000236498.1	ENSG00000231672.6
26446	ENSG00000226074.4	ENSG00000236497.1	ENSG00000198547.8
26447	ENSG00000226075.2	ENSG00000236495.2	ENSG00000239884.3
26448	ENSG00000226078.1	ENSG00000236494.1	ENSG00000175279.22
26449	ENSG00000226079.1	ENSG00000236493.2	ENSG00000264290.1
26450	ENSG00000226080.1	ENSG00000236491.1	ENSG00000226564.1
26451	ENSG00000226081.2	ENSG00000236489.1	ENSG00000259516.2
26452	ENSG00000226082.2	ENSG00000236487.2	ENSG00000103569.9
26453	ENSG00000226083.5	ENSG00000236485.1	ENSG00000199977.1
26454	ENSG00000226084.5	ENSG00000236484.2	ENSG00000168267.6
26455	ENSG00000226085.3	ENSG00000236483.1	ENSG00000263417.4
26456	ENSG00000226086.5	ENSG00000236481.1	ENSG00000235838.1
26457	ENSG00000226087.1	ENSG00000236480.1	ENSG00000130598.16
26458	ENSG00000226088.1	ENSG00000236478.2	ENSG00000079246.16
26459	ENSG00000226089.2	ENSG00000236477.1	ENSG00000169217.9
26460	ENSG00000226091.7	ENSG00000236476.2	ENSG00000135424.16
26461	ENSG00000226092.4	ENSG00000236475.1	ENSG00000279700.1
26462	ENSG00000226093.1	ENSG00000236474.1	ENSG00000215187.11
26463	ENSG00000226094.1	ENSG00000236473.1	ENSG00000252393.1
26464	ENSG00000226096.1	ENSG00000236472.1	ENSG00000160145.15
26465	ENSG00000226097.5	ENSG00000236471.1	ENSG00000272800.2
26466	ENSG00000226098.4	ENSG00000236469.1	ENSG00000153140.8
26467	ENSG00000226101.1	ENSG00000236468.1	ENSG00000243406.6
26468	ENSG00000226102.3	ENSG00000236467.8	ENSG00000242970.2
26469	ENSG00000226107.1	ENSG00000236466.1	ENSG00000174500.13
26470	ENSG00000226108.2	ENSG00000236464.1	ENSG00000170860.4
26471	ENSG00000226110.2	ENSG00000236463.1	ENSG00000259628.1
26472	ENSG00000226112.1	ENSG00000236461.1	ENSG00000205767.4
26473	ENSG00000226113.1	ENSG00000236460.1	ENSG00000075884.14
26474	ENSG00000226114.1	ENSG00000236459.1	ENSG00000149257.14
26475	ENSG00000226115.1	ENSG00000236457.1	ENSG00000100109.17
26476	ENSG00000226116.1	ENSG00000236456.1	ENSG00000197429.10
26477	ENSG00000226117.1	ENSG00000236453.5	ENSG00000183092.16
26478	ENSG00000226118.1	ENSG00000236452.1	ENSG00000175792.11
26479	ENSG00000226119.1	ENSG00000236451.2	ENSG00000103245.14
26480	ENSG00000226121.2	ENSG00000236450.1	ENSG00000215455.4
26481	ENSG00000226122.1	ENSG00000236449.1	ENSG00000251620.2
26482	ENSG00000226124.7	ENSG00000236447.2	ENSG00000142208.16
26483	ENSG00000226125.1	ENSG00000236446.3	ENSG00000169314.14
26484	ENSG00000226126.2	ENSG00000236445.4	ENSG00000162511.8
26485	ENSG00000226128.1	ENSG00000236444.4	ENSG00000268804.1
26486	ENSG00000226130.1	ENSG00000236442.1	ENSG00000237669.1
26487	ENSG00000226132.1	ENSG00000236440.1	ENSG00000207955.5
26488	ENSG00000226133.6	ENSG00000236439.4	ENSG00000261416.1
26489	ENSG00000226134.1	ENSG00000236438.7	ENSG00000101782.15
26490	ENSG00000226137.5	ENSG00000236437.1	ENSG00000105202.9
26491	ENSG00000226138.4	ENSG00000236436.1	ENSG00000234807.6
26492	ENSG00000226140.5	ENSG00000236435.1	ENSG00000198440.9
26493	ENSG00000226141.1	ENSG00000236434.2	ENSG00000264924.1
26494	ENSG00000226142.2	ENSG00000236433.1	ENSG00000100711.13
26495	ENSG00000226143.1	ENSG00000236432.7	ENSG00000158220.14
26496	ENSG00000226144.2	ENSG00000236431.1	ENSG00000057657.17
26497	ENSG00000226145.7	ENSG00000236430.1	ENSG00000129071.9

26498	ENSG00000226147.1	ENSG00000236429.1	ENSG00000152413.14
26499	ENSG00000226148.1	ENSG00000236427.1	ENSG00000225051.5
26500	ENSG00000226149.5	ENSG00000236426.5	ENSG00000145839.1
26501	ENSG00000226153.1	ENSG00000236425.1	ENSG00000174255.6
26502	ENSG00000226155.1	ENSG00000236424.7	ENSG00000178952.11
26503	ENSG00000226156.1	ENSG00000236423.5	ENSG00000143842.15
26504	ENSG00000226157.1	ENSG00000236420.1	ENSG00000234323.6
26505	ENSG00000226158.1	ENSG00000236417.2	ENSG00000177201.2
26506	ENSG00000226159.1	ENSG00000236414.1	ENSG00000225093.1
26507	ENSG00000226160.1	ENSG00000236413.2	ENSG00000238039.1
26508	ENSG00000226161.1	ENSG00000236412.1	ENSG00000232729.7
26509	ENSG00000226163.1	ENSG00000236411.1	ENSG00000276600.5
26510	ENSG00000226164.2	ENSG00000236409.2	ENSG00000107862.4
26511	ENSG00000226166.2	ENSG00000236408.1	ENSG00000171916.16
26512	ENSG00000226167.1	ENSG00000236407.1	ENSG00000226174.7
26513	ENSG00000226168.1	ENSG00000236405.1	ENSG00000256464.1
26514	ENSG00000226169.1	ENSG00000236404.9	ENSG00000226677.3
26515	ENSG00000226172.2	ENSG00000236403.1	ENSG00000258538.5
26516	ENSG00000226174.7	ENSG00000236401.1	ENSG00000164649.20
26517	ENSG00000226179.6	ENSG00000236400.1	ENSG00000149403.12
26518	ENSG00000226179.6_PAR_Y	ENSG00000236398.2	ENSG00000230424.1
26519	ENSG00000226180.3	ENSG00000236397.3	ENSG00000267959.3
26520	ENSG00000226181.1	ENSG00000236396.8	ENSG00000117640.18
26521	ENSG00000226183.1	ENSG00000236395.1	ENSG00000120896.13
26522	ENSG00000226185.2	ENSG00000236394.2	ENSG00000166135.14
26523	ENSG00000226186.1	ENSG00000236393.1	ENSG00000151746.14
26524	ENSG00000226188.2	ENSG00000236391.3	ENSG00000277369.1
26525	ENSG00000226191.3	ENSG00000236390.1	ENSG00000204291.11
26526	ENSG00000226193.1	ENSG00000236389.1	ENSG00000099999.14
26527	ENSG00000226194.5	ENSG00000236388.1	ENSG00000242076.2
26528	ENSG00000226196.1	ENSG00000236387.1	ENSG00000206559.8
26529	ENSG00000226197.2	ENSG00000236386.1	ENSG00000225871.2
26530	ENSG00000226199.1	ENSG00000236385.1	ENSG00000067334.14
26531	ENSG00000226200.6	ENSG00000236384.7	ENSG00000143850.15
26532	ENSG00000226203.1	ENSG00000236383.8	ENSG00000125812.16
26533	ENSG00000226204.1	ENSG00000236382.1	ENSG00000159063.13
26534	ENSG00000226205.1	ENSG00000236380.5	ENSG00000279164.1
26535	ENSG00000226206.1	ENSG00000236379.2	ENSG00000277511.1
26536	ENSG00000226207.1	ENSG00000236378.1	ENSG00000196549.10
26537	ENSG00000226208.1	ENSG00000236377.1	ENSG00000101282.9
26538	ENSG00000226209.1	ENSG00000236376.1	ENSG00000172404.4
26539	ENSG00000226210.3	ENSG00000236375.3	ENSG00000185664.14
26540	ENSG00000226211.1	ENSG00000236373.1	ENSG00000184811.4
26541	ENSG00000226212.2	ENSG00000236372.1	ENSG00000259706.1
26542	ENSG00000226213.1	ENSG00000236371.5	ENSG00000186767.6
26543	ENSG00000226216.1	ENSG00000236366.2	ENSG00000239906.1
26544	ENSG00000226217.1	ENSG00000236365.1	ENSG00000114302.16
26545	ENSG00000226218.1	ENSG00000236364.3	ENSG00000279786.1
26546	ENSG00000226220.1	ENSG00000236362.8	ENSG00000205018.2
26547	ENSG00000226221.1	ENSG00000236360.2	ENSG00000255920.2
26548	ENSG00000226222.3	ENSG00000236359.1	ENSG00000240376.1
26549	ENSG00000226223.1	ENSG00000236358.1	ENSG00000008513.16
26550	ENSG00000226226.1	ENSG00000236357.1	ENSG00000237101.1

26551	ENSG00000226229.2	ENSG00000236356.1	ENSG00000270084.2
26552	ENSG00000226230.1	ENSG00000236355.2	ENSG00000114124.2
26553	ENSG00000226232.8	ENSG00000236354.1	ENSG00000260996.1
26554	ENSG00000226233.1	ENSG00000236352.1	ENSG00000100304.13
26555	ENSG00000226234.2	ENSG00000236349.1	ENSG00000273585.1
26556	ENSG00000226235.1	ENSG00000236348.1	ENSG00000255438.2
26557	ENSG00000226237.1	ENSG00000236347.1	ENSG00000214706.10
26558	ENSG00000226238.1	ENSG00000236343.1	ENSG00000255529.9
26559	ENSG00000226239.1	ENSG00000236341.1	ENSG00000254648.1
26560	ENSG00000226240.2	ENSG00000236340.5	ENSG00000196456.12
26561	ENSG00000226241.2	ENSG00000236339.1	ENSG00000224660.1
26562	ENSG00000226242.1	ENSG00000236337.1	ENSG00000019102.12
26563	ENSG00000226243.1	ENSG00000236336.1	ENSG00000211716.2
26564	ENSG00000226245.1	ENSG00000236335.1	ENSG00000275888.1
26565	ENSG00000226246.1	ENSG00000236334.2	ENSG00000277496.1
26566	ENSG00000226247.1	ENSG00000236333.3	ENSG00000214612.3
26567	ENSG00000226249.1	ENSG00000236332.1	ENSG00000181656.7
26568	ENSG00000226250.1	ENSG00000236330.1	ENSG00000204196.5
26569	ENSG00000226251.5	ENSG00000236326.1	ENSG00000254835.1
26570	ENSG00000226252.1	ENSG00000236325.1	ENSG00000167526.13
26571	ENSG00000226253.1	ENSG00000236324.1	ENSG00000146757.13
26572	ENSG00000226254.1	ENSG00000236323.1	ENSG00000262049.1
26573	ENSG00000226255.1	ENSG00000236320.3	ENSG00000233124.1
26574	ENSG00000226258.6	ENSG00000236319.2	ENSG00000079263.19
26575	ENSG00000226259.10	ENSG00000236318.2	ENSG00000243960.1
26576	ENSG00000226261.1	ENSG00000236317.1	ENSG00000204370.11
26577	ENSG00000226262.1	ENSG00000236316.2	ENSG00000211892.4
26578	ENSG00000226263.1	ENSG00000236313.1	ENSG00000242110.8
26579	ENSG00000226266.6	ENSG00000236312.3	ENSG00000125356.7
26580	ENSG00000226268.3	ENSG00000236311.6	ENSG00000278000.1
26581	ENSG00000226270.3	ENSG00000236308.1	ENSG00000251393.3
26582	ENSG00000226272.5	ENSG00000236307.2	ENSG00000286161.1
26583	ENSG00000226273.2	ENSG00000236306.2	ENSG00000148120.16
26584	ENSG00000226276.1	ENSG00000236305.1	ENSG00000228341.1
26585	ENSG00000226277.1	ENSG00000236304.1	ENSG00000104147.9
26586	ENSG00000226278.1	ENSG00000236303.3	ENSG00000272787.1
26587	ENSG00000226279.2	ENSG00000236301.5	ENSG00000137462.7
26588	ENSG00000226280.2	ENSG00000236300.2	ENSG00000280449.1
26589	ENSG00000226281.3	ENSG00000236299.7	ENSG00000118579.13
26590	ENSG00000226284.1	ENSG00000236297.1	ENSG00000274808.5
26591	ENSG00000226285.1	ENSG00000236295.1	ENSG00000233057.1
26592	ENSG00000226286.1	ENSG00000236292.1	ENSG00000237254.2
26593	ENSG00000226287.8	ENSG00000236290.1	ENSG00000272444.1
26594	ENSG00000226288.2	ENSG00000236289.3	ENSG00000072609.17
26595	ENSG00000226289.1	ENSG00000236287.8	ENSG00000065268.10
26596	ENSG00000226291.1	ENSG00000236285.1	ENSG00000235665.5
26597	ENSG00000226292.1	ENSG00000236284.1	ENSG00000141655.17
26598	ENSG00000226296.1	ENSG00000236281.1	ENSG00000227268.3
26599	ENSG00000226297.1	ENSG00000236280.1	ENSG00000245812.2
26600	ENSG00000226298.1	ENSG00000236279.7	ENSG00000153291.16
26601	ENSG00000226299.2	ENSG00000236278.2	ENSG00000135297.15
26602	ENSG00000226301.3	ENSG00000236277.1	ENSG00000188277.9
26603	ENSG00000226302.1	ENSG00000236276.1	ENSG00000279640.1

26604	ENSG00000226304.1	ENSG00000236274.1	ENSG00000162241.12
26605	ENSG00000226306.6	ENSG00000236272.1	ENSG00000231169.4
26606	ENSG00000226307.1	ENSG00000236268.5	ENSG00000265141.2
26607	ENSG00000226308.1	ENSG00000236267.1	ENSG00000147437.10
26608	ENSG00000226309.1	ENSG00000236264.5	ENSG00000240929.2
26609	ENSG00000226310.1	ENSG00000236263.1	ENSG00000153207.15
26610	ENSG00000226312.7	ENSG00000236262.1	ENSG00000129038.16
26611	ENSG00000226313.1	ENSG00000236261.1	ENSG00000146707.14
26612	ENSG00000226314.8	ENSG00000236259.1	ENSG00000100714.16
26613	ENSG00000226317.2	ENSG00000236258.1	ENSG00000253865.1
26614	ENSG00000226318.1	ENSG00000236257.1	ENSG00000159069.14
26615	ENSG00000226320.5	ENSG00000236256.9	ENSG00000230021.10
26616	ENSG00000226321.5	ENSG00000236255.1	ENSG00000260078.3
26617	ENSG00000226323.1	ENSG00000236254.1	ENSG00000186583.11
26618	ENSG00000226324.1	ENSG00000236253.7	ENSG00000163207.7
26619	ENSG00000226327.3	ENSG00000236252.2	ENSG00000112078.14
26620	ENSG00000226328.6	ENSG00000236248.1	ENSG00000104524.14
26621	ENSG00000226329.2	ENSG00000236246.2	ENSG00000140575.13
26622	ENSG00000226330.1	ENSG00000236244.1	ENSG00000237286.1
26623	ENSG00000226331.1	ENSG00000236243.2	ENSG00000180185.11
26624	ENSG00000226332.2	ENSG00000236242.1	ENSG00000197149.5
26625	ENSG00000226334.1	ENSG00000236241.1	ENSG00000124596.17
26626	ENSG00000226336.2	ENSG00000236240.1	ENSG00000240024.5
26627	ENSG00000226337.3	ENSG00000236238.2	ENSG00000283384.1
26628	ENSG00000226338.1	ENSG00000236235.1	ENSG00000138443.16
26629	ENSG00000226339.1	ENSG00000236234.1	ENSG00000271267.1
26630	ENSG00000226340.1	ENSG00000236232.1	ENSG00000275418.1
26631	ENSG00000226341.1	ENSG00000236231.1	ENSG00000145476.16
26632	ENSG00000226342.1	ENSG00000236230.2	ENSG00000087152.15
26633	ENSG00000226344.1	ENSG00000236229.1	ENSG00000285210.1
26634	ENSG00000226345.1	ENSG00000236226.1	ENSG00000129696.12
26635	ENSG00000226348.1	ENSG00000236217.4	ENSG00000197006.14
26636	ENSG00000226349.1	ENSG00000236216.5	ENSG00000112837.17
26637	ENSG00000226352.2	ENSG00000236213.1	ENSG00000058056.9
26638	ENSG00000226353.1	ENSG00000236212.1	ENSG00000255458.6
26639	ENSG00000226355.1	ENSG00000236211.1	ENSG00000230469.1
26640	ENSG00000226356.2	ENSG00000236209.1	ENSG00000244575.3
26641	ENSG00000226358.1	ENSG00000236208.1	ENSG00000225187.1
26642	ENSG00000226359.1	ENSG00000236206.1	ENSG00000211804.3
26643	ENSG00000226360.5	ENSG00000236205.1	ENSG00000088827.12
26644	ENSG00000226361.5	ENSG00000236202.1	ENSG00000114030.13
26645	ENSG00000226362.2	ENSG00000236200.5	ENSG00000166091.21
26646	ENSG00000226363.3	ENSG00000236199.1	ENSG00000106344.8
26647	ENSG00000226364.1	ENSG00000236197.3	ENSG00000226410.1
26648	ENSG00000226366.1	ENSG00000236195.1	ENSG00000197969.13
26649	ENSG00000226367.5	ENSG00000236194.3	ENSG00000272735.1
26650	ENSG00000226369.1	ENSG00000236193.3	ENSG00000107829.14
26651	ENSG00000226370.1	ENSG00000236191.3	ENSG00000223478.1
26652	ENSG00000226372.3	ENSG00000236190.1	ENSG00000261172.1
26653	ENSG00000226374.2	ENSG00000236189.2	ENSG00000255730.5
26654	ENSG00000226375.1	ENSG00000236188.1	ENSG00000106610.15
26655	ENSG00000226376.1	ENSG00000236187.1	ENSG00000108733.10
26656	ENSG00000226377.1	ENSG00000236185.1	ENSG00000124614.15

26657	ENSG00000226380.9	ENSG00000236184.1	ENSG00000134905.17
26658	ENSG00000226383.6	ENSG00000236182.1	ENSG00000136950.13
26659	ENSG00000226386.1	ENSG00000236180.2	ENSG00000264943.1
26660	ENSG00000226387.2	ENSG00000236179.1	ENSG00000233775.1
26661	ENSG00000226388.1	ENSG00000236176.2	ENSG00000275056.1
26662	ENSG00000226389.3	ENSG00000236175.3	ENSG00000124733.4
26663	ENSG00000226390.1	ENSG00000236173.1	ENSG00000178852.16
26664	ENSG00000226392.1	ENSG00000236172.6	ENSG00000166851.15
26665	ENSG00000226393.1	ENSG00000236171.1	ENSG00000259623.1
26666	ENSG00000226394.2	ENSG00000236170.1	ENSG00000228436.2
26667	ENSG00000226395.1	ENSG00000236168.1	ENSG00000178596.10
26668	ENSG00000226396.1	ENSG00000236167.1	ENSG00000162695.12
26669	ENSG00000226397.8	ENSG00000236166.1	ENSG00000161955.16
26670	ENSG00000226398.1	ENSG00000236165.1	ENSG00000112305.14
26671	ENSG00000226400.2	ENSG00000236164.1	ENSG00000260448.5
26672	ENSG00000226401.3	ENSG00000236162.1	ENSG00000132321.17
26673	ENSG00000226403.1	ENSG00000236160.1	ENSG00000179397.18
26674	ENSG00000226405.1	ENSG00000236159.1	ENSG00000253356.1
26675	ENSG00000226406.1	ENSG00000236158.1	ENSG00000198947.15
26676	ENSG00000226407.1	ENSG00000236155.6	ENSG00000185267.10
26677	ENSG00000226409.2	ENSG00000236154.1	ENSG00000013619.14
26678	ENSG00000226410.1	ENSG00000236153.1	ENSG00000259294.1
26679	ENSG00000226411.1	ENSG00000236152.1	ENSG00000256861.1
26680	ENSG00000226412.1	ENSG00000236151.1	ENSG00000157379.14
26681	ENSG00000226413.2	ENSG00000236148.5	ENSG00000159433.12
26682	ENSG00000226414.1	ENSG00000236146.2	ENSG00000230214.1
26683	ENSG00000226415.1	ENSG00000236145.1	ENSG00000203780.10
26684	ENSG00000226416.1	ENSG00000236144.7	ENSG00000050165.17
26685	ENSG00000226419.7	ENSG00000236141.1	ENSG00000091622.16
26686	ENSG00000226420.1	ENSG00000236140.1	ENSG00000279483.2
26687	ENSG00000226421.1	ENSG00000236138.4	ENSG00000277763.1
26688	ENSG00000226423.1	ENSG00000236137.1	ENSG00000113645.14
26689	ENSG00000226425.1	ENSG00000236136.1	ENSG00000163995.20
26690	ENSG00000226426.1	ENSG00000236133.1	ENSG00000183150.8
26691	ENSG00000226427.1	ENSG00000236132.1	ENSG00000229119.3
26692	ENSG00000226428.1	ENSG00000236131.1	ENSG00000232531.3
26693	ENSG00000226429.2	ENSG00000236130.2	ENSG00000218868.1
26694	ENSG00000226430.6	ENSG00000236129.1	ENSG00000257761.1
26695	ENSG00000226431.1	ENSG00000236126.3	ENSG00000184545.11
26696	ENSG00000226432.3	ENSG00000236125.3	ENSG00000223697.3
26697	ENSG00000226433.1	ENSG00000236124.1	ENSG00000175893.11
26698	ENSG00000226435.10	ENSG00000236123.1	ENSG00000104219.13
26699	ENSG00000226436.1	ENSG00000236121.1	ENSG00000144485.11
26700	ENSG00000226438.1	ENSG00000236120.6	ENSG00000158109.15
26701	ENSG00000226439.3	ENSG00000236119.1	ENSG00000166839.17
26702	ENSG00000226440.7	ENSG00000236118.1	ENSG00000139354.11
26703	ENSG00000226441.2	ENSG00000236117.1	ENSG00000281849.3
26704	ENSG00000226442.2	ENSG00000236116.1	ENSG00000145936.9
26705	ENSG00000226443.3	ENSG00000236115.1	ENSG00000259207.7
26706	ENSG00000226444.2	ENSG00000236114.1	ENSG00000227557.1
26707	ENSG00000226445.1	ENSG00000236111.5	ENSG00000112561.18
26708	ENSG00000226446.1	ENSG00000236110.1	ENSG00000184451.5
26709	ENSG00000226447.1	ENSG00000236109.1	ENSG00000134717.18

26710	ENSG00000226448.1	ENSG00000236108.1	ENSG00000274922.1
26711	ENSG00000226449.1	ENSG00000236107.9	ENSG00000277218.1
26712	ENSG00000226450.2	ENSG00000236106.1	ENSG00000170260.8
26713	ENSG00000226453.1	ENSG00000236105.1	ENSG00000219470.1
26714	ENSG00000226454.2	ENSG00000236104.3	ENSG00000259717.1
26715	ENSG00000226455.1	ENSG00000236102.2	ENSG00000023445.14
26716	ENSG00000226457.1	ENSG00000236101.1	ENSG00000171130.18
26717	ENSG00000226461.3	ENSG00000236099.1	ENSG00000174574.16
26718	ENSG00000226465.2	ENSG00000236098.1	ENSG00000100365.15
26719	ENSG00000226466.2	ENSG00000236097.1	ENSG00000230071.2
26720	ENSG00000226468.2	ENSG00000236095.1	ENSG00000148396.18
26721	ENSG00000226469.1	ENSG00000236094.1	ENSG00000088256.9
26722	ENSG00000226470.1	ENSG00000236091.1	ENSG00000100528.12
26723	ENSG00000226471.6	ENSG00000236090.2	ENSG00000156232.7
26724	ENSG00000226472.8	ENSG00000236088.9	ENSG00000154274.15
26725	ENSG00000226473.2	ENSG00000236086.4	ENSG00000258695.2
26726	ENSG00000226474.1	ENSG00000236083.2	ENSG00000135407.10
26727	ENSG00000226476.3	ENSG00000236081.1	ENSG00000226393.1
26728	ENSG00000226477.1	ENSG00000236080.2	ENSG00000184897.6
26729	ENSG00000226478.3	ENSG00000236079.1	ENSG00000116560.11
26730	ENSG00000226479.4	ENSG00000236078.1	ENSG00000176124.13
26731	ENSG00000226480.1	ENSG00000236077.2	ENSG00000196505.11
26732	ENSG00000226481.3	ENSG00000236076.1	ENSG00000271199.1
26733	ENSG00000226482.1	ENSG00000236075.1	ENSG00000210140.1
26734	ENSG00000226483.2	ENSG00000236073.2	ENSG00000259075.6
26735	ENSG00000226484.2	ENSG00000236072.1	ENSG00000280079.1
26736	ENSG00000226485.1	ENSG00000236069.2	ENSG00000156973.14
26737	ENSG00000226486.1	ENSG00000236068.2	ENSG00000172456.17
26738	ENSG00000226487.1	ENSG00000236065.2	ENSG00000258359.1
26739	ENSG00000226488.1	ENSG00000236064.1	ENSG00000273267.1
26740	ENSG00000226489.1	ENSG00000236062.1	ENSG00000227456.8
26741	ENSG00000226490.2	ENSG00000236060.2	ENSG00000270083.1
26742	ENSG00000226491.1	ENSG00000236058.3	ENSG00000275294.4
26743	ENSG00000226493.1	ENSG00000236056.1	ENSG00000140350.15
26744	ENSG00000226496.2	ENSG00000236054.1	ENSG00000214544.7
26745	ENSG00000226497.1	ENSG00000236053.1	ENSG00000230061.2
26746	ENSG00000226498.2	ENSG00000236052.1	ENSG00000196743.8
26747	ENSG00000226499.1	ENSG00000236051.7	ENSG00000157107.14
26748	ENSG00000226500.2	ENSG00000236049.1	ENSG00000163251.4
26749	ENSG00000226501.2	ENSG00000236048.2	ENSG00000251307.1
26750	ENSG00000226502.2	ENSG00000236047.1	ENSG00000204361.9
26751	ENSG00000226504.1	ENSG00000236046.1	ENSG00000228261.1
26752	ENSG00000226506.5	ENSG00000236045.1	ENSG00000107077.18
26753	ENSG00000226507.1	ENSG00000236044.1	ENSG00000173261.9
26754	ENSG00000226508.1	ENSG00000236042.2	ENSG00000272789.1
26755	ENSG00000226509.1	ENSG00000236041.1	ENSG00000211745.3
26756	ENSG00000226510.1	ENSG00000236040.1	ENSG00000167110.17
26757	ENSG00000226515.1	ENSG00000236039.2	ENSG00000248415.1
26758	ENSG00000226516.7	ENSG00000236036.1	ENSG00000148848.14
26759	ENSG00000226519.1	ENSG00000236035.2	ENSG00000115602.16
26760	ENSG00000226520.1	ENSG00000236032.3	ENSG00000170509.12
26761	ENSG00000226521.7	ENSG00000236031.1	ENSG00000058085.14
26762	ENSG00000226522.1	ENSG00000236028.1	ENSG00000126581.13

26763	ENSG00000226523.1	ENSG00000236027.2	ENSG00000188848.16
26764	ENSG00000226525.5	ENSG00000236026.1	ENSG00000092140.16
26765	ENSG00000226526.1	ENSG00000236025.1	ENSG00000254395.1
26766	ENSG00000226527.1	ENSG00000236024.1	ENSG00000154814.14
26767	ENSG00000226529.1	ENSG00000236022.6	ENSG00000246731.2
26768	ENSG00000226530.1	ENSG00000236021.1	ENSG00000157335.20
26769	ENSG00000226532.1	ENSG00000236018.2	ENSG00000141698.16
26770	ENSG00000226533.1	ENSG00000236017.8_PAR_Y	ENSG00000261218.5
26771	ENSG00000226534.1	ENSG00000236017.8	ENSG00000254732.1
26772	ENSG00000226535.1	ENSG00000236015.1	ENSG00000234353.2
26773	ENSG00000226536.1	ENSG00000236013.6	ENSG00000174456.14
26774	ENSG00000226537.2	ENSG00000236012.2	ENSG00000070423.18
26775	ENSG00000226539.1	ENSG00000236009.1	ENSG00000133028.12
26776	ENSG00000226540.2	ENSG00000236008.2	ENSG00000196954.14
26777	ENSG00000226541.1	ENSG00000236007.1	ENSG00000185482.8
26778	ENSG00000226542.1	ENSG00000236005.1	ENSG00000185250.15
26779	ENSG00000226543.3	ENSG00000236004.2	ENSG00000163157.15
26780	ENSG00000226544.4	ENSG00000236003.1	ENSG00000267470.5
26781	ENSG00000226545.1	ENSG00000235998.1	ENSG00000112299.8
26782	ENSG00000226547.2	ENSG00000235997.2	ENSG00000124251.11
26783	ENSG00000226548.1	ENSG00000235996.1	ENSG00000140332.15
26784	ENSG00000226549.3	ENSG00000235995.1	ENSG00000273973.1
26785	ENSG00000226552.2	ENSG00000235994.4	ENSG00000128641.19
26786	ENSG00000226553.1	ENSG00000235993.1	ENSG00000105355.9
26787	ENSG00000226554.1	ENSG00000235992.1	ENSG00000177599.13
26788	ENSG00000226555.1	ENSG00000235991.4	ENSG00000106617.14
26789	ENSG00000226556.2	ENSG00000235990.2	ENSG00000270829.1
26790	ENSG00000226557.1	ENSG00000235989.3	ENSG00000236935.1
26791	ENSG00000226558.1	ENSG00000235988.1	ENSG00000223803.1
26792	ENSG00000226562.3	ENSG00000235984.5	ENSG00000101452.15
26793	ENSG00000226564.1	ENSG00000235981.2	ENSG00000131467.10
26794	ENSG00000226565.2	ENSG00000235979.8	ENSG00000257802.1
26795	ENSG00000226566.2	ENSG00000235978.6	ENSG00000181135.16
26796	ENSG00000226567.1	ENSG00000235976.2	ENSG00000134256.12
26797	ENSG00000226570.2	ENSG00000235975.1	ENSG00000172262.11
26798	ENSG00000226571.2	ENSG00000235974.1	ENSG00000230572.5
26799	ENSG00000226572.1	ENSG00000235972.1	ENSG00000276085.1
26800	ENSG00000226573.1	ENSG00000235969.1	ENSG00000092020.10
26801	ENSG00000226576.1	ENSG00000235968.1	ENSG00000284964.1
26802	ENSG00000226577.1	ENSG00000235967.1	ENSG00000100068.12
26803	ENSG00000226578.1	ENSG00000235965.2	ENSG00000198730.9
26804	ENSG00000226579.1	ENSG00000235964.1	ENSG00000235111.1
26805	ENSG00000226580.1	ENSG00000235963.1	ENSG00000215148.8
26806	ENSG00000226581.1	ENSG00000235962.5	ENSG00000113580.15
26807	ENSG00000226582.1	ENSG00000235961.5	ENSG00000278769.1
26808	ENSG00000226587.1	ENSG00000235959.1	ENSG00000176593.7
26809	ENSG00000226590.1	ENSG00000235958.5	ENSG00000181856.14
26810	ENSG00000226592.1	ENSG00000235957.1	ENSG00000047597.7
26811	ENSG00000226594.1	ENSG00000235955.1	ENSG00000168028.14
26812	ENSG00000226595.1	ENSG00000235954.6	ENSG00000219249.2
26813	ENSG00000226597.1	ENSG00000235951.1	ENSG00000269951.1
26814	ENSG00000226599.1	ENSG00000235949.1	ENSG00000277301.1
26815	ENSG00000226600.3	ENSG00000235947.1	ENSG00000151014.6

26816	ENSG00000226601.1	ENSG00000235946.1	ENSG00000171302.17
26817	ENSG00000226604.2	ENSG00000235945.1	ENSG00000233266.1
26818	ENSG00000226605.1	ENSG00000235944.8	ENSG00000127081.14
26819	ENSG00000226608.3	ENSG00000235943.1	ENSG00000175600.15
26820	ENSG00000226609.1	ENSG00000235942.2	ENSG00000174844.14
26821	ENSG00000226611.2	ENSG00000235940.1	ENSG00000250548.6
26822	ENSG00000226616.1	ENSG00000235939.1	ENSG00000134072.11
26823	ENSG00000226617.1	ENSG00000235937.1	ENSG00000266579.1
26824	ENSG00000226619.1	ENSG00000235934.1	ENSG00000156017.13
26825	ENSG00000226620.7	ENSG00000235933.1	ENSG00000136104.20
26826	ENSG00000226621.1	ENSG00000235932.2	ENSG00000004779.10
26827	ENSG00000226622.5	ENSG00000235931.2	ENSG00000187097.12
26828	ENSG00000226624.1	ENSG00000235930.3	ENSG00000279236.1
26829	ENSG00000226625.1	ENSG00000235929.1	ENSG00000177947.13
26830	ENSG00000226626.2	ENSG00000235927.4	ENSG00000158079.16
26831	ENSG00000226627.1	ENSG00000235926.1	ENSG00000196455.8
26832	ENSG00000226629.1	ENSG00000235924.1	ENSG00000256553.1
26833	ENSG00000226631.1	ENSG00000235922.1	ENSG00000132356.11
26834	ENSG00000226632.1	ENSG00000235920.1	ENSG00000205918.9
26835	ENSG00000226633.2	ENSG00000235919.4	ENSG00000156508.18
26836	ENSG00000226636.1	ENSG00000235917.1	ENSG00000114648.12
26837	ENSG00000226640.2	ENSG00000235916.1	ENSG00000250569.1
26838	ENSG00000226641.1	ENSG00000235914.1	ENSG00000237593.1
26839	ENSG00000226642.1	ENSG00000235912.1	ENSG00000225335.3
26840	ENSG00000226643.2	ENSG00000235911.1	ENSG00000237773.6
26841	ENSG00000226644.5	ENSG00000235910.1	ENSG00000197498.13
26842	ENSG00000226645.1	ENSG00000235908.1	ENSG00000173947.14
26843	ENSG00000226646.1	ENSG00000235907.2	ENSG00000162302.13
26844	ENSG00000226647.2	ENSG00000235904.2	ENSG00000121903.14
26845	ENSG00000226648.1	ENSG00000235903.8	ENSG00000180917.17
26846	ENSG00000226649.1	ENSG00000235902.1	ENSG00000137962.13
26847	ENSG00000226652.3	ENSG00000235901.2	ENSG00000229273.1
26848	ENSG00000226653.3	ENSG00000235899.1	ENSG00000236830.6
26849	ENSG00000226655.1	ENSG00000235897.1	ENSG00000197013.10
26850	ENSG00000226658.1	ENSG00000235895.1	ENSG00000204179.10
26851	ENSG00000226659.1	ENSG00000235893.5	ENSG00000186889.10
26852	ENSG00000226660.2	ENSG00000235892.1	ENSG00000165349.12
26853	ENSG00000226661.1	ENSG00000235890.2	ENSG00000149679.11
26854	ENSG00000226662.2	ENSG00000235888.2	ENSG00000224822.1
26855	ENSG00000226663.2	ENSG00000235887.2	ENSG00000229806.1
26856	ENSG00000226664.1	ENSG00000235886.1	ENSG00000189045.13
26857	ENSG00000226665.1	ENSG00000235885.7	ENSG00000132881.12
26858	ENSG00000226666.1	ENSG00000235884.4	ENSG00000177045.9
26859	ENSG00000226668.5	ENSG00000235881.2	ENSG00000169962.5
26860	ENSG00000226669.2	ENSG00000235880.1	ENSG00000254228.2
26861	ENSG00000226670.1	ENSG00000235879.1	ENSG00000175730.8
26862	ENSG00000226671.1	ENSG00000235876.2	ENSG00000180509.12
26863	ENSG00000226673.2	ENSG00000235875.3	ENSG00000279962.1
26864	ENSG00000226674.10	ENSG00000235872.2	ENSG00000143845.15
26865	ENSG00000226676.1	ENSG00000235871.1	ENSG00000226491.1
26866	ENSG00000226677.3	ENSG00000235869.1	ENSG00000133422.13
26867	ENSG00000226679.1	ENSG00000235868.3	ENSG00000265749.5
26868	ENSG00000226680.2	ENSG00000235865.2	ENSG00000197056.11

26869	ENSG00000226681.1	ENSG00000235864.1	ENSG00000197251.3
26870	ENSG00000226683.2	ENSG00000235863.3	ENSG00000088726.16
26871	ENSG00000226685.4	ENSG00000235862.2	ENSG00000165917.10
26872	ENSG00000226686.7	ENSG00000235861.1	ENSG00000228063.1
26873	ENSG00000226688.6	ENSG00000235859.5	ENSG00000213857.3
26874	ENSG00000226690.8	ENSG00000235858.1	ENSG00000273036.3
26875	ENSG00000226693.1	ENSG00000235857.1	ENSG00000181274.6
26876	ENSG00000226694.2	ENSG00000235855.3	ENSG00000133119.13
26877	ENSG00000226695.1	ENSG00000235852.1	ENSG00000165046.12
26878	ENSG00000226696.6	ENSG00000235849.1	ENSG00000136490.9
26879	ENSG00000226698.1	ENSG00000235848.4	ENSG00000127249.15
26880	ENSG00000226699.1	ENSG00000235847.2	ENSG00000095209.12
26881	ENSG00000226700.1	ENSG00000235845.1	ENSG00000229659.1
26882	ENSG00000226701.1	ENSG00000235843.1	ENSG00000136235.16
26883	ENSG00000226702.1	ENSG00000235840.1	ENSG00000101343.14
26884	ENSG00000226703.1	ENSG00000235838.1	ENSG00000162385.11
26885	ENSG00000226705.1	ENSG00000235837.1	ENSG00000271404.1
26886	ENSG00000226706.1	ENSG00000235836.1	ENSG00000175224.16
26887	ENSG00000226707.1	ENSG00000235834.1	ENSG00000227953.6
26888	ENSG00000226708.1	ENSG00000235833.1	ENSG00000245522.2
26889	ENSG00000226709.1	ENSG00000235832.2	ENSG00000234751.1
26890	ENSG00000226711.6	ENSG00000235831.6	ENSG00000271943.2
26891	ENSG00000226715.3	ENSG00000235830.1	ENSG00000156804.7
26892	ENSG00000226716.1	ENSG00000235829.1	ENSG00000262633.2
26893	ENSG00000226717.2	ENSG00000235828.5	ENSG00000272686.1
26894	ENSG00000226718.1	ENSG00000235827.2	ENSG00000214960.10
26895	ENSG00000226721.2	ENSG00000235824.4	ENSG00000277965.1
26896	ENSG00000226722.3	ENSG00000235823.2	ENSG00000268947.1
26897	ENSG00000226723.2	ENSG00000235821.1	ENSG00000174013.8
26898	ENSG00000226724.1	ENSG00000235820.1	ENSG00000137441.8
26899	ENSG00000226725.2	ENSG00000235819.1	ENSG00000184719.12
26900	ENSG00000226726.1	ENSG00000235818.3	ENSG00000149499.11
26901	ENSG00000226729.3	ENSG00000235817.2	ENSG00000249825.5
26902	ENSG00000226733.3	ENSG00000235816.3	ENSG00000151640.13
26903	ENSG00000226734.1	ENSG00000235815.1	ENSG00000276523.1
26904	ENSG00000226739.1	ENSG00000235813.1	ENSG00000117091.10
26905	ENSG00000226741.1	ENSG00000235812.4	ENSG00000147202.18
26906	ENSG00000226742.4	ENSG00000235811.1	ENSG00000258303.1
26907	ENSG00000226744.1	ENSG00000235810.1	ENSG00000019991.17
26908	ENSG00000226745.2	ENSG00000235808.1	ENSG00000211451.12
26909	ENSG00000226746.2	ENSG00000235806.1	ENSG00000235613.2
26910	ENSG00000226747.6	ENSG00000235805.1	ENSG00000104660.18
26911	ENSG00000226750.1	ENSG00000235804.2	ENSG00000141568.21
26912	ENSG00000226751.2	ENSG00000235803.1	ENSG00000239483.1
26913	ENSG00000226752.9	ENSG00000235802.1	ENSG00000153885.14
26914	ENSG00000226753.1	ENSG00000235797.2	ENSG00000141552.17
26915	ENSG00000226754.1	ENSG00000235795.1	ENSG00000010319.6
26916	ENSG00000226755.2	ENSG00000235794.1	ENSG00000100433.15
26917	ENSG00000226756.1	ENSG00000235790.7	ENSG00000134644.15
26918	ENSG00000226757.2	ENSG00000235787.1	ENSG00000071539.14
26919	ENSG00000226758.1	ENSG00000235786.1	ENSG00000163257.11
26920	ENSG00000226759.7	ENSG00000235785.1	ENSG00000139218.18
26921	ENSG00000226761.3	ENSG00000235784.2	ENSG00000005448.16

26922	ENSG00000226762.2	ENSG00000235781.1	ENSG00000129472.15
26923	ENSG00000226763.4	ENSG00000235780.3	ENSG00000225796.2
26924	ENSG00000226764.2	ENSG00000235779.7	ENSG00000284431.1
26925	ENSG00000226766.1	ENSG00000235778.1	ENSG00000241985.1
26926	ENSG00000226767.1	ENSG00000235777.1	ENSG00000119888.10
26927	ENSG00000226769.1	ENSG00000235776.2	ENSG00000136986.10
26928	ENSG00000226770.1	ENSG00000235775.1	ENSG00000084463.8
26929	ENSG00000226771.1	ENSG00000235774.1	ENSG00000175155.9
26930	ENSG00000226772.1	ENSG00000235772.1	ENSG00000170921.15
26931	ENSG00000226773.1	ENSG00000235770.5	ENSG00000261215.1
26932	ENSG00000226774.1	ENSG00000235768.1	ENSG00000228300.14
26933	ENSG00000226776.1	ENSG00000235763.1	ENSG00000286242.1
26934	ENSG00000226777.7	ENSG00000235761.2	ENSG00000198910.13
26935	ENSG00000226779.1	ENSG00000235760.4	ENSG00000263164.1
26936	ENSG00000226780.1	ENSG00000235759.1	ENSG00000270757.1
26937	ENSG00000226781.1	ENSG00000235756.2	ENSG00000285476.1
26938	ENSG00000226782.2	ENSG00000235749.2	ENSG00000116459.11
26939	ENSG00000226783.1	ENSG00000235748.1	ENSG00000112293.15
26940	ENSG00000226784.2	ENSG00000235746.1	ENSG00000273062.1
26941	ENSG00000226789.1	ENSG00000235743.1	ENSG00000198026.8
26942	ENSG00000226790.3	ENSG00000235742.1	ENSG00000120341.18
26943	ENSG00000226791.6	ENSG00000235741.1	ENSG00000123607.15
26944	ENSG00000226792.7	ENSG00000235740.1	ENSG00000257000.1
26945	ENSG00000226794.1	ENSG00000235738.1	ENSG00000271538.5
26946	ENSG00000226797.1	ENSG00000235737.1	ENSG00000038532.16
26947	ENSG00000226798.1	ENSG00000235736.1	ENSG00000198925.12
26948	ENSG00000226800.5	ENSG00000235735.1	ENSG00000280248.1
26949	ENSG00000226801.2	ENSG00000235734.4	ENSG00000248208.1
26950	ENSG00000226802.1	ENSG00000235731.2	ENSG00000215018.9
26951	ENSG00000226803.8	ENSG00000235730.2	ENSG00000222112.1
26952	ENSG00000226804.4	ENSG00000235729.1	ENSG00000232434.2
26953	ENSG00000226806.1	ENSG00000235728.1	ENSG00000125107.18
26954	ENSG00000226807.6	ENSG00000235726.6	ENSG00000266126.1
26955	ENSG00000226808.2	ENSG00000235725.1	ENSG00000182379.10
26956	ENSG00000226810.3	ENSG00000235724.9	ENSG00000123838.11
26957	ENSG00000226812.2	ENSG00000235721.1	ENSG00000185641.6
26958	ENSG00000226813.2	ENSG00000235720.1	ENSG00000189283.10
26959	ENSG00000226814.2	ENSG00000235718.9	ENSG00000145428.14
26960	ENSG00000226816.3	ENSG00000235717.1	ENSG00000111879.19
26961	ENSG00000226818.1	ENSG00000235716.1	ENSG00000120709.11
26962	ENSG00000226819.1	ENSG00000235713.1	ENSG00000170006.12
26963	ENSG00000226820.2	ENSG00000235711.4	ENSG00000160856.21
26964	ENSG00000226822.2	ENSG00000235710.5	ENSG00000263916.1
26965	ENSG00000226823.1	ENSG00000235707.2	ENSG00000213409.4
26966	ENSG00000226824.6	ENSG00000235706.7	ENSG00000005893.15
26967	ENSG00000226827.1	ENSG00000235705.6	ENSG00000149806.11
26968	ENSG00000226828.1	ENSG00000235703.5	ENSG00000089248.7
26969	ENSG00000226829.1	ENSG00000235701.1	ENSG00000284526.1
26970	ENSG00000226831.1	ENSG00000235700.1	ENSG00000139679.15
26971	ENSG00000226833.5	ENSG00000235699.2	ENSG00000121060.18
26972	ENSG00000226835.2	ENSG00000235698.1	ENSG00000163879.11
26973	ENSG00000226836.5	ENSG00000235695.1	ENSG00000182472.8
26974	ENSG00000226837.2	ENSG00000235690.2	ENSG00000001631.15

26975	ENSG00000226838.1	ENSG00000235689.1	ENSG00000212536.1
26976	ENSG00000226839.1	ENSG00000235688.2	ENSG00000260496.3
26977	ENSG00000226840.1	ENSG00000235687.9	ENSG00000143033.18
26978	ENSG00000226842.1	ENSG00000235686.1	ENSG00000110536.14
26979	ENSG00000226843.1	ENSG00000235685.2	ENSG00000164338.10
26980	ENSG00000226845.1	ENSG00000235683.1	ENSG00000285600.1
26981	ENSG00000226846.1	ENSG00000235681.1	ENSG00000180422.3
26982	ENSG00000226847.1	ENSG00000235679.1	ENSG00000170092.14
26983	ENSG00000226849.1	ENSG00000235678.3	ENSG00000283375.1
26984	ENSG00000226851.1	ENSG00000235677.1	ENSG00000144843.11
26985	ENSG00000226853.2	ENSG00000235674.2	ENSG00000203722.8
26986	ENSG00000226854.1	ENSG00000235673.1	ENSG00000211721.2
26987	ENSG00000226855.1	ENSG00000235672.1	ENSG00000162367.11
26988	ENSG00000226856.6	ENSG00000235670.1	ENSG00000264176.1
26989	ENSG00000226857.1	ENSG00000235669.1	ENSG00000054219.11
26990	ENSG00000226859.1	ENSG00000235665.5	ENSG00000275115.1
26991	ENSG00000226860.1	ENSG00000235661.3	ENSG00000106348.18
26992	ENSG00000226861.1	ENSG00000235660.2	ENSG00000091986.15
26993	ENSG00000226862.1	ENSG00000235659.1	ENSG00000140987.20
26994	ENSG00000226863.1	ENSG00000235656.1	ENSG00000110880.10
26995	ENSG00000226864.2	ENSG00000235655.3	ENSG00000230381.2
26996	ENSG00000226867.1	ENSG00000235653.1	ENSG00000231466.2
26997	ENSG00000226868.1	ENSG00000235652.7	ENSG00000211592.8
26998	ENSG00000226869.6	ENSG00000235651.1	ENSG00000243066.3
26999	ENSG00000226870.2	ENSG00000235649.2	ENSG00000047932.14
27000	ENSG00000226871.1	ENSG00000235647.1	ENSG00000122970.16
27001	ENSG00000226872.1	ENSG00000235646.1	ENSG00000248099.4
27002	ENSG00000226873.1	ENSG00000235645.2	ENSG00000226661.1
27003	ENSG00000226874.1	ENSG00000235644.1	ENSG00000071889.16
27004	ENSG00000226875.1	ENSG00000235643.1	ENSG00000244630.2
27005	ENSG00000226876.3	ENSG00000235642.1	ENSG00000128989.10
27006	ENSG00000226877.8	ENSG00000235641.4	ENSG00000197134.12
27007	ENSG00000226878.2	ENSG00000235640.1	ENSG00000117533.15
27008	ENSG00000226881.5	ENSG00000235639.1	ENSG00000184047.18
27009	ENSG00000226883.1	ENSG00000235638.1	ENSG00000143622.11
27010	ENSG00000226885.1	ENSG00000235637.1	ENSG00000185507.20
27011	ENSG00000226886.1	ENSG00000235636.1	ENSG00000121716.20
27012	ENSG00000226887.8	ENSG00000235635.1	ENSG00000082781.12
27013	ENSG00000226888.2	ENSG00000235631.2	ENSG00000276231.4
27014	ENSG00000226889.3	ENSG00000235629.1	ENSG00000165548.11
27015	ENSG00000226890.1	ENSG00000235628.1	ENSG00000278356.1
27016	ENSG00000226891.8	ENSG00000235627.1	ENSG00000256977.12
27017	ENSG00000226899.1	ENSG00000235626.2	ENSG00000176974.20
27018	ENSG00000226900.1	ENSG00000235625.1	ENSG00000131686.15
27019	ENSG00000226902.1	ENSG00000235623.2	ENSG00000167508.12
27020	ENSG00000226903.1	ENSG00000235621.8	ENSG00000285410.1
27021	ENSG00000226904.1	ENSG00000235620.1	ENSG00000164066.13
27022	ENSG00000226905.1	ENSG00000235619.1	ENSG00000181804.15
27023	ENSG00000226906.1	ENSG00000235618.8	ENSG00000110002.15
27024	ENSG00000226908.1	ENSG00000235617.1	ENSG00000274653.1
27025	ENSG00000226912.1	ENSG00000235616.1	ENSG00000123213.23
27026	ENSG00000226913.1	ENSG00000235615.2	ENSG00000259651.2
27027	ENSG00000226915.1	ENSG00000235613.2	ENSG00000241168.1

27028	ENSG00000226917.5	ENSG00000235612.1	ENSG00000272604.1
27029	ENSG00000226918.1	ENSG00000235610.1	ENSG00000132541.11
27030	ENSG00000226919.3	ENSG00000235609.7	ENSG00000242866.10
27031	ENSG00000226920.1	ENSG00000235608.1	ENSG00000156298.12
27032	ENSG00000226921.6	ENSG00000235606.3	ENSG00000137309.19
27033	ENSG00000226925.1	ENSG00000235605.1	ENSG00000005700.15
27034	ENSG00000226926.7	ENSG00000235602.5	ENSG00000226952.1
27035	ENSG00000226927.1	ENSG00000235601.1	ENSG00000127084.19
27036	ENSG00000226928.1	ENSG00000235598.1	ENSG00000228382.1
27037	ENSG00000226929.3	ENSG00000235597.1	ENSG00000279413.1
27038	ENSG00000226930.1	ENSG00000235595.1	ENSG00000259065.1
27039	ENSG00000226933.1	ENSG00000235594.1	ENSG00000081026.19
27040	ENSG00000226935.6	ENSG00000235593.1	ENSG00000173258.12
27041	ENSG00000226937.9	ENSG00000235592.1	ENSG00000240204.3
27042	ENSG00000226938.1	ENSG00000235590.7	ENSG00000181991.16
27043	ENSG00000226939.1	ENSG00000235587.2	ENSG00000257242.7
27044	ENSG00000226941.8	ENSG00000235586.1	ENSG00000241316.7
27045	ENSG00000226942.2	ENSG00000235584.2	ENSG00000135976.19
27046	ENSG00000226943.3	ENSG00000235583.2	ENSG00000224067.2
27047	ENSG00000226944.1	ENSG00000235582.2	ENSG00000111215.12
27048	ENSG00000226945.1	ENSG00000235581.1	ENSG00000281469.1
27049	ENSG00000226946.1	ENSG00000235579.1	ENSG00000184922.14
27050	ENSG00000226947.1	ENSG00000235578.1	ENSG00000116898.12
27051	ENSG00000226948.2	ENSG00000235576.1	ENSG00000213339.9
27052	ENSG00000226949.1	ENSG00000235575.1	ENSG00000274370.1
27053	ENSG00000226950.6	ENSG00000235574.1	ENSG00000236056.1
27054	ENSG00000226952.1	ENSG00000235573.2	ENSG00000141428.17
27055	ENSG00000226953.7	ENSG00000235572.2	ENSG00000108506.12
27056	ENSG00000226954.1	ENSG00000235571.1	ENSG00000273137.1
27057	ENSG00000226955.1	ENSG00000235570.1	ENSG00000185808.13
27058	ENSG00000226956.1	ENSG00000235568.6	ENSG00000279958.1
27059	ENSG00000226957.1	ENSG00000235564.1	ENSG00000231113.2
27060	ENSG00000226960.1	ENSG00000235563.1	ENSG00000244097.1
27061	ENSG00000226961.3	ENSG00000235560.3	ENSG00000264343.6
27062	ENSG00000226962.1	ENSG00000235559.1	ENSG00000233024.7
27063	ENSG00000226963.1	ENSG00000235558.3	ENSG00000108433.16
27064	ENSG00000226964.1	ENSG00000235555.1	ENSG00000097033.14
27065	ENSG00000226965.1	ENSG00000235552.4	ENSG00000234705.1
27066	ENSG00000226967.1	ENSG00000235550.1	ENSG00000276182.1
27067	ENSG00000226968.1	ENSG00000235549.1	ENSG00000223486.1
27068	ENSG00000226969.1	ENSG00000235548.1	ENSG00000119915.5
27069	ENSG00000226970.2	ENSG00000235547.3	ENSG00000236564.1
27070	ENSG00000226971.1	ENSG00000235546.2	ENSG00000221882.3
27071	ENSG00000226972.2	ENSG00000235545.1	ENSG00000267244.5
27072	ENSG00000226973.1	ENSG00000235544.1	ENSG00000129911.9
27073	ENSG00000226976.3	ENSG00000235539.1	ENSG00000241326.1
27074	ENSG00000226977.1	ENSG00000235538.1	ENSG00000184983.10
27075	ENSG00000226978.1	ENSG00000235537.2	ENSG00000142634.13
27076	ENSG00000226979.8	ENSG00000235535.7	ENSG00000204923.4
27077	ENSG00000226981.2	ENSG00000235534.1	ENSG00000269161.1
27078	ENSG00000226982.4	ENSG00000235533.1	ENSG00000271643.2
27079	ENSG00000226983.2	ENSG00000235532.1	ENSG00000267769.1
27080	ENSG00000226984.1	ENSG00000235531.9	ENSG00000236801.1

27081	ENSG00000226985.6	ENSG00000235530.6	ENSG00000167637.17
27082	ENSG00000226986.4	ENSG00000235529.1	ENSG00000152939.16
27083	ENSG00000226987.1	ENSG00000235527.6	ENSG00000236017.8
27084	ENSG00000226989.1	ENSG00000235526.1	ENSG00000249626.1
27085	ENSG00000226990.1	ENSG00000235525.1	ENSG00000279026.1
27086	ENSG00000226991.2	ENSG00000235524.1	ENSG00000165810.17
27087	ENSG00000226992.1	ENSG00000235523.1	ENSG00000285669.1
27088	ENSG00000226994.7	ENSG00000235522.6	ENSG00000089558.9
27089	ENSG00000226995.8	ENSG00000235521.1	ENSG00000141503.16
27090	ENSG00000226996.5	ENSG00000235519.2	ENSG00000115657.14
27091	ENSG00000226998.1	ENSG00000235518.2	ENSG00000165966.15
27092	ENSG00000226999.1	ENSG00000235514.1	ENSG00000181751.10
27093	ENSG00000227000.2	ENSG00000235513.1	ENSG00000141458.13
27094	ENSG00000227001.3	ENSG00000235512.1	ENSG00000162642.14
27095	ENSG00000227002.2	ENSG00000235511.1	ENSG00000161533.12
27096	ENSG00000227004.2	ENSG00000235510.1	ENSG00000198763.3
27097	ENSG00000227006.1	ENSG00000235508.3	ENSG00000162373.13
27098	ENSG00000227007.1	ENSG00000235503.2	ENSG00000186481.16
27099	ENSG00000227008.2	ENSG00000235501.5	ENSG00000169299.14
27100	ENSG00000227009.1	ENSG00000235500.2	ENSG00000133169.6
27101	ENSG00000227011.4	ENSG00000235499.1	ENSG00000117262.19
27102	ENSG00000227012.2	ENSG00000235497.5	ENSG00000151952.16
27103	ENSG00000227013.3	ENSG00000235495.1	ENSG00000134013.15
27104	ENSG00000227014.6	ENSG00000235494.1	ENSG00000008516.17
27105	ENSG00000227015.1	ENSG00000235493.2	ENSG00000277577.1
27106	ENSG00000227016.3	ENSG00000235492.1	ENSG00000239219.2
27107	ENSG00000227017.1	ENSG00000235491.1	ENSG00000173269.14
27108	ENSG00000227018.1	ENSG00000235489.4	ENSG00000196781.15
27109	ENSG00000227019.2	ENSG00000235488.1	ENSG00000140632.17
27110	ENSG00000227021.1	ENSG00000235486.1	ENSG00000214563.2
27111	ENSG00000227023.1	ENSG00000235485.1	ENSG00000103489.11
27112	ENSG00000227028.6	ENSG00000235483.1	ENSG00000169710.9
27113	ENSG00000227029.1	ENSG00000235482.1	ENSG00000168994.13
27114	ENSG00000227032.1	ENSG00000235481.2	ENSG00000063322.14
27115	ENSG00000227033.1	ENSG00000235480.1	ENSG00000160606.11
27116	ENSG00000227034.1	ENSG00000235479.1	ENSG00000143942.5
27117	ENSG00000227035.1	ENSG00000235478.4	ENSG00000267222.1
27118	ENSG00000227036.7	ENSG00000235472.1	ENSG00000119535.17
27119	ENSG00000227038.3	ENSG00000235470.5	ENSG00000121039.10
27120	ENSG00000227039.6	ENSG00000235469.1	ENSG00000272416.1
27121	ENSG00000227040.4	ENSG00000235467.1	ENSG00000225951.1
27122	ENSG00000227042.1	ENSG00000235464.1	ENSG00000025423.11
27123	ENSG00000227043.2	ENSG00000235463.1	ENSG00000179743.4
27124	ENSG00000227045.1	ENSG00000235462.1	ENSG00000137878.17
27125	ENSG00000227047.1	ENSG00000235461.1	ENSG00000243498.2
27126	ENSG00000227048.2	ENSG00000235459.5	ENSG00000263235.1
27127	ENSG00000227050.1	ENSG00000235455.1	ENSG00000205940.8
27128	ENSG00000227051.6	ENSG00000235454.1	ENSG00000163155.12
27129	ENSG00000227053.1	ENSG00000235451.1	ENSG00000109536.12
27130	ENSG00000227054.1	ENSG00000235450.1	ENSG00000147570.9
27131	ENSG00000227055.1	ENSG00000235449.2	ENSG00000123104.12
27132	ENSG00000227056.2	ENSG00000235448.2	ENSG00000222019.7
27133	ENSG00000227057.10	ENSG00000235447.2	ENSG00000183828.15

27134	ENSG00000227058.1	ENSG00000235446.2	ENSG00000078053.17
27135	ENSG00000227059.6	ENSG00000235445.1	ENSG00000211652.2
27136	ENSG00000227060.6	ENSG00000235444.2	ENSG00000197872.11
27137	ENSG00000227061.1	ENSG00000235440.1	ENSG00000227094.1
27138	ENSG00000227062.2	ENSG00000235438.7	ENSG00000101161.8
27139	ENSG00000227063.5	ENSG00000235437.7	ENSG00000113638.14
27140	ENSG00000227064.1	ENSG00000235436.10	ENSG00000283839.1
27141	ENSG00000227066.1	ENSG00000235435.1	ENSG00000159692.15
27142	ENSG00000227067.3	ENSG00000235434.1	ENSG00000278341.1
27143	ENSG00000227068.1	ENSG00000235433.1	ENSG00000187531.14
27144	ENSG00000227069.1	ENSG00000235432.1	ENSG00000230817.5
27145	ENSG00000227070.1	ENSG00000235431.1	ENSG00000091129.20
27146	ENSG00000227071.1	ENSG00000235430.1	ENSG00000189350.12
27147	ENSG00000227072.1	ENSG00000235429.1	ENSG00000132170.21
27148	ENSG00000227073.1	ENSG00000235427.1	ENSG00000276317.1
27149	ENSG00000227075.1	ENSG00000235426.2	ENSG00000187185.4
27150	ENSG00000227076.1	ENSG00000235425.2	ENSG00000163803.13
27151	ENSG00000227077.3	ENSG00000235424.1	ENSG00000231105.1
27152	ENSG00000227078.1	ENSG00000235422.1	ENSG00000198743.7
27153	ENSG00000227080.2	ENSG00000235421.1	ENSG00000256262.1
27154	ENSG00000227081.5	ENSG00000235420.7	ENSG00000255769.7
27155	ENSG00000227082.2	ENSG00000235419.5	ENSG00000224228.2
27156	ENSG00000227083.1	ENSG00000235416.1	ENSG00000143740.14
27157	ENSG00000227087.4	ENSG00000235415.1	ENSG00000269976.1
27158	ENSG00000227088.1	ENSG00000235414.3	ENSG00000163249.12
27159	ENSG00000227089.2	ENSG00000235413.3	ENSG00000274929.1
27160	ENSG00000227090.1	ENSG00000235412.1	ENSG00000172071.12
27161	ENSG00000227091.1	ENSG00000235411.1	ENSG00000172967.7
27162	ENSG00000227094.1	ENSG00000235410.1	ENSG00000162747.11
27163	ENSG00000227096.1	ENSG00000235408.5	ENSG00000215067.9
27164	ENSG00000227097.5	ENSG00000235407.1	ENSG00000139579.13
27165	ENSG00000227098.1	ENSG00000235403.1	ENSG00000164292.13
27166	ENSG00000227101.1	ENSG00000235400.1	ENSG00000278785.1
27167	ENSG00000227102.2	ENSG00000235399.1	ENSG00000119899.13
27168	ENSG00000227104.3	ENSG00000235397.1	ENSG00000151461.20
27169	ENSG00000227105.1	ENSG00000235390.4	ENSG00000259092.1
27170	ENSG00000227107.1	ENSG00000235389.2	ENSG00000004866.20
27171	ENSG00000227108.1	ENSG00000235387.4	ENSG00000272432.1
27172	ENSG00000227109.1	ENSG00000235386.1	ENSG00000121749.15
27173	ENSG00000227110.6	ENSG00000235385.1	ENSG00000244080.3
27174	ENSG00000227113.2	ENSG00000235382.1	ENSG00000227574.2
27175	ENSG00000227115.7	ENSG00000235381.1	ENSG00000112149.10
27176	ENSG00000227117.6	ENSG00000235379.1	ENSG00000250159.6
27177	ENSG00000227118.3	ENSG00000235378.2	ENSG00000025708.14
27178	ENSG00000227120.1	ENSG00000235377.1	ENSG00000226658.1
27179	ENSG00000227121.1	ENSG00000235376.5	ENSG00000234072.1
27180	ENSG00000227123.1	ENSG00000235374.2	ENSG00000264530.2
27181	ENSG00000227124.10	ENSG00000235371.1	ENSG00000197782.14
27182	ENSG00000227125.1	ENSG00000235370.6	ENSG00000225489.6
27183	ENSG00000227128.4	ENSG00000235369.1	ENSG00000257594.4
27184	ENSG00000227131.1	ENSG00000235368.1	ENSG00000168502.17
27185	ENSG00000227133.1	ENSG00000235366.2	ENSG00000270419.1
27186	ENSG00000227134.1	ENSG00000235363.1	ENSG00000120820.12

27187	ENSG00000227138.1	ENSG00000235361.1	ENSG00000130653.16
27188	ENSG00000227139.1	ENSG00000235358.1	ENSG00000053438.10
27189	ENSG00000227140.3	ENSG00000235357.1	ENSG00000113732.9
27190	ENSG00000227141.2	ENSG00000235356.2	ENSG00000138777.20
27191	ENSG00000227143.1	ENSG00000235354.1	ENSG00000142606.16
27192	ENSG00000227145.1	ENSG00000235352.1	ENSG00000184787.19
27193	ENSG00000227148.1	ENSG00000235351.1	ENSG00000212978.6
27194	ENSG00000227149.1	ENSG00000235350.1	ENSG00000169118.18
27195	ENSG00000227150.1	ENSG00000235349.1	ENSG00000228748.2
27196	ENSG00000227151.4	ENSG00000235347.1	ENSG00000175348.11
27197	ENSG00000227152.6	ENSG00000235343.1	ENSG00000229120.2
27198	ENSG00000227154.4	ENSG00000235339.1	ENSG00000233483.3
27199	ENSG00000227155.7	ENSG00000235338.1	ENSG00000273420.1
27200	ENSG00000227157.1	ENSG00000235337.1	ENSG00000205683.11
27201	ENSG00000227158.1	ENSG00000235335.2	ENSG00000259417.3
27202	ENSG00000227159.8	ENSG00000235334.1	ENSG00000134853.12
27203	ENSG00000227159.8_PAR_Y	ENSG00000235333.3	ENSG00000165030.4
27204	ENSG00000227160.3	ENSG00000235332.2	ENSG00000113520.11
27205	ENSG00000227161.1	ENSG00000235330.2	ENSG00000228653.2
27206	ENSG00000227163.2	ENSG00000235329.2	ENSG00000233901.5
27207	ENSG00000227165.8	ENSG00000235328.1	ENSG00000160972.9
27208	ENSG00000227166.1	ENSG00000235325.1	ENSG00000284669.1
27209	ENSG00000227167.1	ENSG00000235323.2	ENSG00000226342.1
27210	ENSG00000227169.2	ENSG00000235321.1	ENSG00000228477.1
27211	ENSG00000227170.1	ENSG00000235319.1	ENSG00000116198.13
27212	ENSG00000227172.2	ENSG00000235318.3	ENSG00000197465.14
27213	ENSG00000227173.1	ENSG00000235316.1	ENSG00000173230.15
27214	ENSG00000227175.1	ENSG00000235315.2	ENSG00000285632.1
27215	ENSG00000227176.1	ENSG00000235314.1	ENSG00000183048.12
27216	ENSG00000227177.1	ENSG00000235312.1	ENSG00000234743.1
27217	ENSG00000227179.6	ENSG00000235310.1	ENSG00000205464.12
27218	ENSG00000227180.1	ENSG00000235308.1	ENSG00000264400.2
27219	ENSG00000227181.1	ENSG00000235306.2	ENSG00000213977.8
27220	ENSG00000227182.1	ENSG00000235304.1	ENSG00000277229.1
27221	ENSG00000227183.3	ENSG00000235303.1	ENSG00000047457.14
27222	ENSG00000227186.1	ENSG00000235301.1	ENSG00000277462.2
27223	ENSG00000227187.1	ENSG00000235300.4	ENSG00000254463.1
27224	ENSG00000227188.1	ENSG00000235299.2	ENSG00000233205.1
27225	ENSG00000227189.2	ENSG00000235298.1	ENSG00000273160.1
27226	ENSG00000227191.8	ENSG00000235297.3	ENSG00000122884.12
27227	ENSG00000227192.1	ENSG00000235296.1	ENSG00000243179.1
27228	ENSG00000227193.2	ENSG00000235295.1	ENSG00000275743.1
27229	ENSG00000227195.10	ENSG00000235294.1	ENSG00000044090.8
27230	ENSG00000227196.1	ENSG00000235293.1	ENSG00000273174.1
27231	ENSG00000227197.1	ENSG00000235292.1	ENSG00000163960.12
27232	ENSG00000227198.1	ENSG00000235290.1	ENSG00000171566.12
27233	ENSG00000227199.1	ENSG00000235289.1	ENSG00000241678.1
27234	ENSG00000227200.1	ENSG00000235288.3	ENSG00000099769.5
27235	ENSG00000227201.2	ENSG00000235286.1	ENSG00000115339.13
27236	ENSG00000227203.3	ENSG00000235285.1	ENSG00000168958.19
27237	ENSG00000227204.1	ENSG00000235284.1	ENSG00000285702.1
27238	ENSG00000227205.4	ENSG00000235282.3	ENSG00000180209.11
27239	ENSG00000227206.1	ENSG00000235281.1	ENSG00000183763.9

27240	ENSG00000227207.2	ENSG00000235280.2	ENSG00000102317.18
27241	ENSG00000227209.1	ENSG00000235279.1	ENSG00000100346.17
27242	ENSG00000227210.1	ENSG00000235278.2	ENSG00000166855.9
27243	ENSG00000227211.2	ENSG00000235277.1	ENSG00000184731.6
27244	ENSG00000227212.3	ENSG00000235275.3	ENSG00000250151.9
27245	ENSG00000227213.1	ENSG00000235274.1	ENSG00000077458.13
27246	ENSG00000227214.2	ENSG00000235271.5	ENSG00000134352.20
27247	ENSG00000227215.1	ENSG00000235269.1	ENSG00000223571.6
27248	ENSG00000227217.1	ENSG00000235268.2	ENSG00000140398.13
27249	ENSG00000227218.7	ENSG00000235267.1	ENSG00000130038.9
27250	ENSG00000227220.1	ENSG00000235266.1	ENSG00000211720.3
27251	ENSG00000227224.1	ENSG00000235264.1	ENSG00000278233.1
27252	ENSG00000227225.1	ENSG00000235263.1	ENSG00000167851.14
27253	ENSG00000227227.1	ENSG00000235262.1	ENSG00000070669.17
27254	ENSG00000227230.1	ENSG00000235258.1	ENSG00000223776.5
27255	ENSG00000227232.5	ENSG00000235257.8	ENSG00000105968.18
27256	ENSG00000227233.1	ENSG00000235256.1	ENSG00000216906.2
27257	ENSG00000227234.2	ENSG00000235254.3	ENSG00000126267.10
27258	ENSG00000227236.2	ENSG00000235253.1	ENSG00000100290.3
27259	ENSG00000227237.1	ENSG00000235251.2	ENSG00000284095.1
27260	ENSG00000227238.1	ENSG00000235248.3	ENSG00000108239.8
27261	ENSG00000227240.1	ENSG00000235246.1	ENSG00000227201.2
27262	ENSG00000227241.1	ENSG00000235245.1	ENSG00000128342.5
27263	ENSG00000227242.4	ENSG00000235244.3	ENSG00000116161.17
27264	ENSG00000227243.3	ENSG00000235243.1	ENSG00000104783.13
27265	ENSG00000227244.2	ENSG00000235242.1	ENSG00000170631.15
27266	ENSG00000227245.1	ENSG00000235241.1	ENSG00000280381.1
27267	ENSG00000227247.1	ENSG00000235240.1	ENSG00000066185.13
27268	ENSG00000227248.1	ENSG00000235239.1	ENSG00000162066.15
27269	ENSG00000227249.1	ENSG00000235238.1	ENSG00000198870.7
27270	ENSG00000227251.1	ENSG00000235237.1	ENSG00000171222.10
27271	ENSG00000227252.1	ENSG00000235236.1	ENSG00000187624.9
27272	ENSG00000227253.3	ENSG00000235235.6	ENSG00000236567.2
27273	ENSG00000227254.1	ENSG00000235232.1	ENSG00000261367.1
27274	ENSG00000227255.1	ENSG00000235227.1	ENSG00000279613.1
27275	ENSG00000227256.1	ENSG00000235226.1	ENSG00000115423.18
27276	ENSG00000227257.1	ENSG00000235225.1	ENSG00000095539.15
27277	ENSG00000227258.5	ENSG00000235224.1	ENSG00000238164.6
27278	ENSG00000227259.2	ENSG00000235221.2	ENSG00000003509.16
27279	ENSG00000227260.1	ENSG00000235219.1	ENSG00000261040.7
27280	ENSG00000227261.1	ENSG00000235218.2	ENSG00000102572.14
27281	ENSG00000227262.3	ENSG00000235217.6	ENSG00000204619.8
27282	ENSG00000227264.3	ENSG00000235215.2	ENSG00000111361.12
27283	ENSG00000227265.2	ENSG00000235214.1	ENSG00000077585.14
27284	ENSG00000227267.3	ENSG00000235213.1	ENSG00000136295.15
27285	ENSG00000227268.3	ENSG00000235211.4	ENSG00000133313.15
27286	ENSG00000227269.1	ENSG00000235209.1	ENSG00000272948.2
27287	ENSG00000227270.1	ENSG00000235208.2	ENSG00000137073.21
27288	ENSG00000227271.1	ENSG00000235207.1	ENSG00000162078.11
27289	ENSG00000227274.1	ENSG00000235205.1	ENSG00000232186.1
27290	ENSG00000227275.1	ENSG00000235204.1	ENSG00000185885.16
27291	ENSG00000227278.1	ENSG00000235203.2	ENSG00000126353.3
27292	ENSG00000227279.1	ENSG00000235202.1	ENSG00000227466.1

27293	ENSG00000227282.1	ENSG00000235201.1	ENSG00000234160.1
27294	ENSG00000227283.1	ENSG00000235200.2	ENSG00000228049.7
27295	ENSG00000227284.2	ENSG00000235199.1	ENSG00000185246.17
27296	ENSG00000227288.3	ENSG00000235198.1	ENSG00000175175.6
27297	ENSG00000227289.2	ENSG00000235197.1	ENSG00000160339.16
27298	ENSG00000227290.1	ENSG00000235196.3	ENSG00000243795.1
27299	ENSG00000227291.1	ENSG00000235194.8	ENSG00000274642.1
27300	ENSG00000227292.1	ENSG00000235192.1	ENSG00000261976.2
27301	ENSG00000227293.1	ENSG00000235191.1	ENSG00000237926.1
27302	ENSG00000227294.1	ENSG00000235190.1	ENSG00000156411.9
27303	ENSG00000227295.2	ENSG00000235189.1	ENSG00000162510.6
27304	ENSG00000227297.1	ENSG00000235186.1	ENSG00000188021.8
27305	ENSG00000227298.1	ENSG00000235185.1	ENSG00000196268.11
27306	ENSG00000227300.12	ENSG00000235183.3	ENSG00000163104.17
27307	ENSG00000227301.1	ENSG00000235182.1	ENSG00000104812.15
27308	ENSG00000227302.1	ENSG00000235181.1	ENSG00000204209.13
27309	ENSG00000227303.1	ENSG00000235180.1	ENSG00000088305.18
27310	ENSG00000227304.3	ENSG00000235175.1	ENSG00000144476.6
27311	ENSG00000227305.1	ENSG00000235174.1	ENSG00000165506.14
27312	ENSG00000227306.1	ENSG00000235173.7	ENSG00000236439.4
27313	ENSG00000227307.1	ENSG00000235172.7	ENSG00000115468.12
27314	ENSG00000227308.2	ENSG00000235170.1	ENSG00000139998.15
27315	ENSG00000227309.1	ENSG00000235169.8	ENSG00000227855.3
27316	ENSG00000227311.3	ENSG00000235168.1	ENSG00000279415.1
27317	ENSG00000227312.2	ENSG00000235166.7	ENSG00000237753.1
27318	ENSG00000227313.1	ENSG00000235165.2	ENSG00000243959.3
27319	ENSG00000227321.2	ENSG00000235163.2	ENSG00000151876.13
27320	ENSG00000227329.2	ENSG00000235162.8	ENSG00000214293.8
27321	ENSG00000227330.1	ENSG00000235160.1	ENSG00000286031.1
27322	ENSG00000227331.1	ENSG00000235159.1	ENSG00000103723.13
27323	ENSG00000227335.1	ENSG00000235158.1	ENSG00000090565.16
27324	ENSG00000227336.1	ENSG00000235156.5	ENSG00000157856.12
27325	ENSG00000227337.1	ENSG00000235154.1	ENSG00000142684.9
27326	ENSG00000227338.1	ENSG00000235152.1	ENSG00000148341.17
27327	ENSG00000227339.1	ENSG00000235151.1	ENSG00000204301.6
27328	ENSG00000227342.1	ENSG00000235150.1	ENSG00000164088.18
27329	ENSG00000227343.1	ENSG00000235149.1	ENSG00000173611.17
27330	ENSG00000227344.2	ENSG00000235148.2	ENSG00000268469.1
27331	ENSG00000227345.8	ENSG00000235147.1	ENSG00000010292.13
27332	ENSG00000227347.1	ENSG00000235146.2	ENSG00000226987.1
27333	ENSG00000227348.1	ENSG00000235145.2	ENSG00000272402.1
27334	ENSG00000227349.1	ENSG00000235143.1	ENSG00000171790.15
27335	ENSG00000227350.1	ENSG00000235142.9	ENSG00000170579.17
27336	ENSG00000227351.2	ENSG00000235141.1	ENSG00000265400.1
27337	ENSG00000227352.1	ENSG00000235140.2	ENSG00000150867.14
27338	ENSG00000227353.1	ENSG00000235139.2	ENSG00000203804.4
27339	ENSG00000227354.6	ENSG00000235138.1	ENSG00000117013.16
27340	ENSG00000227355.2	ENSG00000235137.2	ENSG00000275243.1
27341	ENSG00000227356.2	ENSG00000235136.1	ENSG00000184220.12
27342	ENSG00000227358.1	ENSG00000235133.1	ENSG00000278961.2
27343	ENSG00000227359.1	ENSG00000235131.1	ENSG00000231170.6
27344	ENSG00000227360.1	ENSG00000235129.1	ENSG00000249412.1
27345	ENSG00000227361.1	ENSG00000235128.1	ENSG00000231120.3

27346	ENSG00000227364.1	ENSG00000235127.1	ENSG00000251034.1
27347	ENSG00000227365.1	ENSG00000235126.1	ENSG00000146066.3
27348	ENSG00000227367.1	ENSG00000235124.1	ENSG00000265735.2
27349	ENSG00000227368.1	ENSG00000235123.5	ENSG00000219665.8
27350	ENSG00000227370.1	ENSG00000235122.3	ENSG00000129646.14
27351	ENSG00000227371.1	ENSG00000235121.1	ENSG00000259772.6
27352	ENSG00000227372.12	ENSG00000235120.1	ENSG00000064932.16
27353	ENSG00000227373.5	ENSG00000235118.8	ENSG00000178053.18
27354	ENSG00000227374.1	ENSG00000235115.3	ENSG00000188873.4
27355	ENSG00000227375.5	ENSG00000235113.1	ENSG00000162736.17
27356	ENSG00000227376.1	ENSG00000235112.1	ENSG00000230897.1
27357	ENSG00000227379.2	ENSG00000235111.1	ENSG00000276997.4
27358	ENSG00000227382.1	ENSG00000235110.5	ENSG00000237513.1
27359	ENSG00000227383.1	ENSG00000235109.7	ENSG00000231177.4
27360	ENSG00000227386.1	ENSG00000235108.1	ENSG00000223343.1
27361	ENSG00000227388.2	ENSG00000235106.10	ENSG00000102096.9
27362	ENSG00000227391.1	ENSG00000235105.1	ENSG00000281357.2
27363	ENSG00000227392.1	ENSG00000235102.1	ENSG00000283196.2
27364	ENSG00000227393.1	ENSG00000235101.1	ENSG00000230956.2
27365	ENSG00000227394.1	ENSG00000235100.3	ENSG00000081791.9
27366	ENSG00000227395.1	ENSG00000235099.1	ENSG00000226822.2
27367	ENSG00000227397.1	ENSG00000235098.8	ENSG00000141665.12
27368	ENSG00000227398.3	ENSG00000235097.1	ENSG00000278023.6
27369	ENSG00000227399.1	ENSG00000235096.2	ENSG00000185046.18
27370	ENSG00000227400.2	ENSG00000235095.1	ENSG00000136819.15
27371	ENSG00000227401.1	ENSG00000235094.3	ENSG00000204252.14
27372	ENSG00000227403.1	ENSG00000235092.5	ENSG00000223612.3
27373	ENSG00000227404.1	ENSG00000235091.2	ENSG00000196497.17
27374	ENSG00000227406.1	ENSG00000235090.1	ENSG00000163795.14
27375	ENSG00000227407.1	ENSG00000235089.1	ENSG00000182621.18
27376	ENSG00000227408.1	ENSG00000235088.1	ENSG00000166887.15
27377	ENSG00000227409.1	ENSG00000235086.1	ENSG00000165617.14
27378	ENSG00000227411.1	ENSG00000235085.3	ENSG00000086065.14
27379	ENSG00000227412.1	ENSG00000235084.3	ENSG00000178149.17
27380	ENSG00000227413.1	ENSG00000235083.1	ENSG00000237711.1
27381	ENSG00000227416.2	ENSG00000235082.2	ENSG00000054523.17
27382	ENSG00000227417.3	ENSG00000235081.1	ENSG00000279608.1
27383	ENSG00000227418.6	ENSG00000235080.2	ENSG00000250067.12
27384	ENSG00000227421.1	ENSG00000235078.1	ENSG00000249347.1
27385	ENSG00000227423.2	ENSG00000235077.1	ENSG00000215256.3
27386	ENSG00000227425.1	ENSG00000235076.2	ENSG00000137054.16
27387	ENSG00000227426.1	ENSG00000235072.2	ENSG00000159915.12
27388	ENSG00000227427.1	ENSG00000235071.1	ENSG00000256223.6
27389	ENSG00000227430.1	ENSG00000235070.3	ENSG00000284657.1
27390	ENSG00000227431.5	ENSG00000235069.2	ENSG00000091164.13
27391	ENSG00000227432.1	ENSG00000235066.7	ENSG00000162267.12
27392	ENSG00000227433.1	ENSG00000235065.1	ENSG00000087884.14
27393	ENSG00000227434.2	ENSG00000235064.1	ENSG00000212747.4
27394	ENSG00000227436.1	ENSG00000235062.4	ENSG00000143420.18
27395	ENSG00000227437.1	ENSG00000235061.3	ENSG00000050767.17
27396	ENSG00000227438.1	ENSG00000235060.1	ENSG00000151692.15
27397	ENSG00000227439.1	ENSG00000235059.5	ENSG00000101654.17
27398	ENSG00000227440.1	ENSG00000235058.1	ENSG00000258933.1

27399	ENSG00000227443.2	ENSG00000235056.1	ENSG00000267336.1
27400	ENSG00000227444.1	ENSG00000235055.3	ENSG00000124743.6
27401	ENSG00000227445.1	ENSG00000235054.3	ENSG00000238141.2
27402	ENSG00000227447.1	ENSG00000235051.3	ENSG00000140022.12
27403	ENSG00000227449.8	ENSG00000235050.6	ENSG00000269903.1
27404	ENSG00000227453.1	ENSG00000235049.1	ENSG00000092964.18
27405	ENSG00000227454.2	ENSG00000235047.1	ENSG00000214460.3
27406	ENSG00000227455.2	ENSG00000235045.2	ENSG00000108826.16
27407	ENSG00000227456.8	ENSG00000235044.1	ENSG00000227014.6
27408	ENSG00000227459.1	ENSG00000235043.3	ENSG00000240875.5
27409	ENSG00000227462.1	ENSG00000235042.1	ENSG00000271780.1
27410	ENSG00000227463.2	ENSG00000235040.1	ENSG00000137809.17
27411	ENSG00000227465.1	ENSG00000235039.1	ENSG00000171291.8
27412	ENSG00000227466.1	ENSG00000235038.1	ENSG00000151778.11
27413	ENSG00000227467.3	ENSG00000235036.4	ENSG00000225899.7
27414	ENSG00000227470.1	ENSG00000235035.1	ENSG00000128159.12
27415	ENSG00000227471.9	ENSG00000235034.6	ENSG00000236287.8
27416	ENSG00000227473.1	ENSG00000235033.7	ENSG00000183386.10
27417	ENSG00000227474.1	ENSG00000235032.1	ENSG00000168283.14
27418	ENSG00000227475.1	ENSG00000235029.1	ENSG00000126883.17
27419	ENSG00000227477.1	ENSG00000235028.3	ENSG00000151694.13
27420	ENSG00000227479.1	ENSG00000235027.1	ENSG00000125912.11
27421	ENSG00000227480.2	ENSG00000235026.5	ENSG00000271609.1
27422	ENSG00000227481.1	ENSG00000235024.1	ENSG00000109083.13
27423	ENSG00000227482.1	ENSG00000235023.1	ENSG00000263990.1
27424	ENSG00000227484.1	ENSG00000235021.1	ENSG00000138439.12
27425	ENSG00000227485.1	ENSG00000235020.4	ENSG00000146007.10
27426	ENSG00000227486.1	ENSG00000235018.1	ENSG00000054277.14
27427	ENSG00000227487.3	ENSG00000235016.1	ENSG00000261804.1
27428	ENSG00000227488.2	ENSG00000235015.1	ENSG00000116750.13
27429	ENSG00000227489.1	ENSG00000235014.1	ENSG00000136696.10
27430	ENSG00000227491.1	ENSG00000235013.1	ENSG00000242616.4
27431	ENSG00000227492.1	ENSG00000235012.1	ENSG00000165322.17
27432	ENSG00000227493.1	ENSG00000235011.2	ENSG00000130997.16
27433	ENSG00000227494.2	ENSG00000235010.1	ENSG00000145632.15
27434	ENSG00000227495.1	ENSG00000235009.1	ENSG00000273729.1
27435	ENSG00000227496.2	ENSG00000235008.1	ENSG00000198712.1
27436	ENSG00000227497.1	ENSG00000235007.2	ENSG00000230409.3
27437	ENSG00000227498.2	ENSG00000235005.1	ENSG00000172239.14
27438	ENSG00000227499.1	ENSG00000235004.1	ENSG00000185515.14
27439	ENSG00000227500.10	ENSG00000235002.1	ENSG00000226020.4
27440	ENSG00000227502.2	ENSG00000235001.3	ENSG00000175741.5
27441	ENSG00000227505.1	ENSG00000234999.1	ENSG00000140320.12
27442	ENSG00000227507.2	ENSG00000234998.1	ENSG00000105383.15
27443	ENSG00000227508.6	ENSG00000234997.1	ENSG00000261584.1
27444	ENSG00000227509.1	ENSG00000234996.4	ENSG00000224699.8
27445	ENSG00000227510.3	ENSG00000234995.1	ENSG00000185090.14
27446	ENSG00000227511.1	ENSG00000234993.1	ENSG00000155744.9
27447	ENSG00000227512.1	ENSG00000234988.1	ENSG00000177879.16
27448	ENSG00000227513.1	ENSG00000234986.1	ENSG00000210144.1
27449	ENSG00000227514.3	ENSG00000234985.2	ENSG00000152291.14
27450	ENSG00000227515.3	ENSG00000234984.1	ENSG00000131149.19
27451	ENSG00000227516.1	ENSG00000234983.1	ENSG00000280184.2

27452	ENSG00000227517.6	ENSG00000234982.1	ENSG00000109861.16
27453	ENSG00000227518.5	ENSG00000234981.1	ENSG00000198369.10
27454	ENSG00000227519.1	ENSG00000234979.1	ENSG00000226010.1
27455	ENSG00000227521.2	ENSG00000234977.1	ENSG00000118514.14
27456	ENSG00000227523.1	ENSG00000234975.6	ENSG00000112759.18
27457	ENSG00000227525.4	ENSG00000234973.5	ENSG00000139194.8
27458	ENSG00000227527.2	ENSG00000234969.1	ENSG00000285292.1
27459	ENSG00000227528.5	ENSG00000234967.1	ENSG00000183308.6
27460	ENSG00000227531.1	ENSG00000234965.2	ENSG00000280018.3
27461	ENSG00000227532.1	ENSG00000234962.5	ENSG00000109685.18
27462	ENSG00000227533.5	ENSG00000234961.1	ENSG00000040633.13
27463	ENSG00000227534.1	ENSG00000234958.6_PAR_Y	ENSG00000280161.1
27464	ENSG00000227535.2	ENSG00000234958.6	ENSG00000109063.15
27465	ENSG00000227536.1	ENSG00000234956.6	ENSG00000273730.1
27466	ENSG00000227537.1	ENSG00000234953.2	ENSG00000273345.5
27467	ENSG00000227538.1	ENSG00000234952.1	ENSG00000122335.16
27468	ENSG00000227540.1	ENSG00000234950.1	ENSG00000132773.12
27469	ENSG00000227541.1	ENSG00000234949.2	ENSG00000066027.12
27470	ENSG00000227542.1	ENSG00000234948.1	ENSG00000163638.13
27471	ENSG00000227543.4	ENSG00000234946.1	ENSG00000075415.12
27472	ENSG00000227544.9	ENSG00000234944.1	ENSG00000136244.12
27473	ENSG00000227545.1	ENSG00000234943.2	ENSG00000229454.1
27474	ENSG00000227547.3	ENSG00000234942.2	ENSG00000120254.15
27475	ENSG00000227549.1	ENSG00000234941.1	ENSG00000250365.6
27476	ENSG00000227550.2	ENSG00000234940.1	ENSG00000184709.7
27477	ENSG00000227551.1	ENSG00000234939.1	ENSG00000129437.10
27478	ENSG00000227554.1	ENSG00000234938.2	ENSG00000149639.15
27479	ENSG00000227555.1	ENSG00000234937.1	ENSG00000248791.1
27480	ENSG00000227556.1	ENSG00000234936.1	ENSG00000255231.1
27481	ENSG00000227557.1	ENSG00000234934.1	ENSG00000140506.16
27482	ENSG00000227560.1	ENSG00000234933.1	ENSG00000108384.15
27483	ENSG00000227562.1	ENSG00000234932.2	ENSG00000172116.22
27484	ENSG00000227563.1	ENSG00000234931.1	ENSG00000260139.6
27485	ENSG00000227564.2	ENSG00000234929.1	ENSG00000256663.1
27486	ENSG00000227568.1	ENSG00000234928.1	ENSG00000284395.1
27487	ENSG00000227573.2	ENSG00000234927.1	ENSG00000176349.11
27488	ENSG00000227574.2	ENSG00000234925.2	ENSG00000153214.11
27489	ENSG00000227578.1	ENSG00000234922.1	ENSG00000206596.1
27490	ENSG00000227579.5	ENSG00000234921.1	ENSG00000258951.1
27491	ENSG00000227581.2	ENSG00000234919.1	ENSG00000270694.1
27492	ENSG00000227582.2	ENSG00000234918.1	ENSG00000145708.11
27493	ENSG00000227583.1	ENSG00000234917.2	ENSG00000072858.10
27494	ENSG00000227584.2	ENSG00000234915.1	ENSG00000124575.6
27495	ENSG00000227585.2	ENSG00000234913.1	ENSG00000078967.13
27496	ENSG00000227586.5	ENSG00000234912.12	ENSG00000170364.12
27497	ENSG00000227588.2	ENSG00000234910.2	ENSG00000171502.15
27498	ENSG00000227589.1	ENSG00000234907.1	ENSG00000179840.5
27499	ENSG00000227590.2	ENSG00000234906.10	ENSG00000143543.15
27500	ENSG00000227591.5	ENSG00000234903.1	ENSG00000102043.16
27501	ENSG00000227592.1	ENSG00000234901.1	ENSG00000122547.11
27502	ENSG00000227593.2	ENSG00000234900.1	ENSG00000279943.1
27503	ENSG00000227597.1	ENSG00000234899.9	ENSG00000179958.8
27504	ENSG00000227598.1	ENSG00000234898.1	ENSG00000108551.5

27505	ENSG00000227599.1	ENSG00000234896.1	ENSG00000163539.17
27506	ENSG00000227602.1	ENSG00000234895.1	ENSG00000130758.8
27507	ENSG00000227604.3	ENSG00000234894.1	ENSG00000177432.7
27508	ENSG00000227606.2	ENSG00000234892.1	ENSG00000133863.8
27509	ENSG00000227607.2	ENSG00000234891.1	ENSG00000269887.1
27510	ENSG00000227609.2	ENSG00000234890.1	ENSG00000260259.1
27511	ENSG00000227610.1	ENSG00000234889.2	ENSG00000152192.8
27512	ENSG00000227611.1	ENSG00000234888.1	ENSG00000154655.15
27513	ENSG00000227613.1	ENSG00000234886.1	ENSG00000008294.21
27514	ENSG00000227615.1	ENSG00000234884.1	ENSG00000006534.16
27515	ENSG00000227616.1	ENSG00000234883.5	ENSG00000234192.1
27516	ENSG00000227617.8	ENSG00000234882.1	ENSG00000172081.14
27517	ENSG00000227619.2	ENSG00000234881.1	ENSG00000198890.8
27518	ENSG00000227620.4	ENSG00000234880.1	ENSG00000249532.5
27519	ENSG00000227621.1	ENSG00000234878.1	ENSG00000237276.8
27520	ENSG00000227623.1	ENSG00000234877.2	ENSG00000189238.5
27521	ENSG00000227624.2	ENSG00000234872.2	ENSG00000168175.15
27522	ENSG00000227627.2	ENSG00000234869.1	ENSG00000113456.19
27523	ENSG00000227629.1	ENSG00000234865.3	ENSG00000142156.14
27524	ENSG00000227630.3	ENSG00000234864.1	ENSG00000255449.1
27525	ENSG00000227632.2	ENSG00000234863.1	ENSG00000159176.14
27526	ENSG00000227633.1	ENSG00000234862.1	ENSG00000248944.1
27527	ENSG00000227635.1	ENSG00000234861.2	ENSG00000198561.13
27528	ENSG00000227638.2	ENSG00000234860.1	ENSG00000254707.1
27529	ENSG00000227640.2	ENSG00000234859.5	ENSG00000214736.7
27530	ENSG00000227644.2	ENSG00000234857.2	ENSG00000183117.19
27531	ENSG00000227645.2	ENSG00000234855.1	ENSG00000259802.1
27532	ENSG00000227646.7	ENSG00000234854.1	ENSG00000271751.1
27533	ENSG00000227649.1	ENSG00000234853.2	ENSG00000270055.1
27534	ENSG00000227653.1	ENSG00000234851.4	ENSG00000284644.1
27535	ENSG00000227657.1	ENSG00000234850.1	ENSG00000250461.1
27536	ENSG00000227658.1	ENSG00000234848.1	ENSG00000092200.12
27537	ENSG00000227659.1	ENSG00000234844.1	ENSG00000247199.4
27538	ENSG00000227660.1	ENSG00000234842.1	ENSG00000263716.1
27539	ENSG00000227661.1	ENSG00000234841.4	ENSG00000155714.13
27540	ENSG00000227663.1	ENSG00000234840.1	ENSG00000141873.11
27541	ENSG00000227666.1	ENSG00000234839.1	ENSG00000279716.1
27542	ENSG00000227667.1	ENSG00000234838.3	ENSG00000175866.15
27543	ENSG00000227671.4	ENSG00000234837.1	ENSG00000100298.15
27544	ENSG00000227673.1	ENSG00000234835.1	ENSG00000169087.11
27545	ENSG00000227674.2	ENSG00000234832.1	ENSG00000181908.5
27546	ENSG00000227676.3	ENSG00000234830.8	ENSG00000181830.8
27547	ENSG00000227678.7	ENSG00000234829.4	ENSG00000275454.1
27548	ENSG00000227679.1	ENSG00000234828.8	ENSG00000108963.17
27549	ENSG00000227680.1	ENSG00000234826.1	ENSG00000279107.1
27550	ENSG00000227681.5	ENSG00000234825.3	ENSG00000104365.15
27551	ENSG00000227682.2	ENSG00000234821.1	ENSG00000198663.16
27552	ENSG00000227683.1	ENSG00000234819.1	ENSG00000146676.9
27553	ENSG00000227684.2	ENSG00000234818.1	ENSG00000090612.21
27554	ENSG00000227685.1	ENSG00000234817.2	ENSG00000156642.16
27555	ENSG00000227687.1	ENSG00000234816.2	ENSG00000166800.9
27556	ENSG00000227688.2	ENSG00000234814.8	ENSG00000198382.9
27557	ENSG00000227689.1	ENSG00000234813.1	ENSG00000079432.7

27558	ENSG00000227692.1	ENSG00000234812.1	ENSG00000115718.17
27559	ENSG00000227693.1	ENSG00000234811.1	ENSG00000214402.6
27560	ENSG00000227694.1	ENSG00000234810.3	ENSG00000260145.1
27561	ENSG00000227695.5	ENSG00000234807.6	ENSG00000181163.13
27562	ENSG00000227698.1	ENSG00000234806.1	ENSG00000223861.1
27563	ENSG00000227700.1	ENSG00000234805.1	ENSG00000204424.9
27564	ENSG00000227702.1	ENSG00000234803.8	ENSG00000273061.1
27565	ENSG00000227704.1	ENSG00000234801.3	ENSG00000186501.14
27566	ENSG00000227705.1	ENSG00000234800.2	ENSG00000211797.2
27567	ENSG00000227706.3	ENSG00000234797.5	ENSG00000224940.8
27568	ENSG00000227707.1	ENSG00000234796.1	ENSG00000107864.15
27569	ENSG00000227708.1	ENSG00000234795.10	ENSG00000118298.12
27570	ENSG00000227709.4	ENSG00000234793.1	ENSG00000068615.18
27571	ENSG00000227710.1	ENSG00000234792.1	ENSG00000118655.6
27572	ENSG00000227711.3	ENSG00000234791.1	ENSG00000170917.13
27573	ENSG00000227712.1	ENSG00000234790.1	ENSG00000155621.15
27574	ENSG00000227713.1	ENSG00000234789.1	ENSG00000121797.10
27575	ENSG00000227714.1	ENSG00000234788.1	ENSG00000242553.1
27576	ENSG00000227716.1	ENSG00000234787.7	ENSG00000139323.14
27577	ENSG00000227717.4	ENSG00000234785.1	ENSG00000105287.12
27578	ENSG00000227718.1	ENSG00000234784.2	ENSG00000155827.12
27579	ENSG00000227719.1	ENSG00000234782.3	ENSG00000142539.9
27580	ENSG00000227721.1	ENSG00000234781.2	ENSG00000100058.12
27581	ENSG00000227722.2	ENSG00000234780.1	ENSG00000109270.13
27582	ENSG00000227723.1	ENSG00000234779.1	ENSG00000027847.14
27583	ENSG00000227725.3	ENSG00000234777.2	ENSG00000263970.1
27584	ENSG00000227726.1	ENSG00000234776.5	ENSG00000255883.1
27585	ENSG00000227728.2	ENSG00000234775.1	ENSG00000154079.6
27586	ENSG00000227729.4	ENSG00000234773.7	ENSG00000248546.3
27587	ENSG00000227730.2	ENSG00000234772.1	ENSG00000134109.11
27588	ENSG00000227733.9	ENSG00000234771.3	ENSG00000279458.2
27589	ENSG00000227734.1	ENSG00000234770.1	ENSG00000061273.18
27590	ENSG00000227735.1	ENSG00000234769.7	ENSG00000276405.1
27591	ENSG00000227736.1	ENSG00000234768.1	ENSG00000130818.12
27592	ENSG00000227737.3	ENSG00000234767.1	ENSG00000272599.2
27593	ENSG00000227738.1	ENSG00000234766.1	ENSG00000152767.16
27594	ENSG00000227740.1	ENSG00000234765.1	ENSG00000182612.10
27595	ENSG00000227741.1	ENSG00000234764.2	ENSG00000099942.13
27596	ENSG00000227742.2	ENSG00000234763.2	ENSG00000215114.9
27597	ENSG00000227743.2	ENSG00000234761.1	ENSG00000220323.4
27598	ENSG00000227744.4	ENSG00000234758.1	ENSG00000132004.13
27599	ENSG00000227745.1	ENSG00000234756.1	ENSG00000265218.1
27600	ENSG00000227747.2	ENSG00000234754.1	ENSG00000122025.14
27601	ENSG00000227748.1	ENSG00000234753.5	ENSG00000165494.11
27602	ENSG00000227751.1	ENSG00000234752.1	ENSG00000169758.13
27603	ENSG00000227753.3	ENSG00000234751.1	ENSG00000166716.10
27604	ENSG00000227755.1	ENSG00000234750.1	ENSG00000049089.15
27605	ENSG00000227757.3	ENSG00000234749.5	ENSG00000108306.13
27606	ENSG00000227758.1	ENSG00000234748.1	ENSG00000271857.1
27607	ENSG00000227759.2	ENSG00000234745.11	ENSG00000274501.1
27608	ENSG00000227760.2	ENSG00000234744.1	ENSG00000203279.3
27609	ENSG00000227762.4	ENSG00000234743.1	ENSG00000211803.2
27610	ENSG00000227764.1	ENSG00000234742.1	ENSG00000251666.1

27611	ENSG00000227765.4	ENSG00000234741.8	ENSG00000258388.7
27612	ENSG00000227766.1	ENSG00000234740.1	ENSG00000217835.3
27613	ENSG00000227769.7	ENSG00000234737.1	ENSG00000122862.5
27614	ENSG00000227770.1	ENSG00000234736.5	ENSG00000204685.6
27615	ENSG00000227773.1	ENSG00000234735.1	ENSG00000248839.1
27616	ENSG00000227775.3	ENSG00000234732.1	ENSG00000272606.1
27617	ENSG00000227776.1	ENSG00000234731.2	ENSG00000110801.13
27618	ENSG00000227777.1	ENSG00000234730.1	ENSG00000164951.16
27619	ENSG00000227779.1	ENSG00000234726.1	ENSG00000226423.1
27620	ENSG00000227782.2	ENSG00000234724.1	ENSG00000165121.11
27621	ENSG00000227784.1	ENSG00000234723.1	ENSG00000166140.17
27622	ENSG00000227785.1	ENSG00000234722.4	ENSG00000179029.14
27623	ENSG00000227788.1	ENSG00000234721.2	ENSG00000270194.1
27624	ENSG00000227789.1	ENSG00000234720.3	ENSG00000235354.1
27625	ENSG00000227790.7	ENSG00000234719.8	ENSG00000251229.1
27626	ENSG00000227791.3	ENSG00000234718.7	ENSG00000197265.9
27627	ENSG00000227795.1	ENSG00000234717.1	ENSG00000280789.2
27628	ENSG00000227799.1	ENSG00000234716.1	ENSG00000153165.18
27629	ENSG00000227800.1	ENSG00000234715.1	ENSG00000269621.1
27630	ENSG00000227802.1	ENSG00000234714.1	ENSG00000138311.17
27631	ENSG00000227803.2	ENSG00000234713.3	ENSG00000125846.15
27632	ENSG00000227805.1	ENSG00000234712.1	ENSG00000239653.1
27633	ENSG00000227806.1	ENSG00000234710.1	ENSG00000145949.10
27634	ENSG00000227809.1	ENSG00000234709.2	ENSG00000167094.16
27635	ENSG00000227811.2	ENSG00000234707.2	ENSG00000241255.1
27636	ENSG00000227813.1	ENSG00000234706.1	ENSG00000266373.1
27637	ENSG00000227814.3	ENSG00000234705.1	ENSG00000221914.10
27638	ENSG00000227815.2	ENSG00000234703.1	ENSG00000228526.7
27639	ENSG00000227817.4	ENSG00000234702.1	ENSG00000137804.13
27640	ENSG00000227818.1	ENSG00000234701.1	ENSG00000265293.2
27641	ENSG00000227824.1	ENSG00000234700.1	ENSG00000154277.12
27642	ENSG00000227825.4	ENSG00000234699.1	ENSG00000283696.1
27643	ENSG00000227827.3	ENSG00000234698.1	ENSG00000205041.1
27644	ENSG00000227830.1	ENSG00000234696.1	ENSG00000275881.1
27645	ENSG00000227832.1	ENSG00000234695.1	ENSG00000213763.4
27646	ENSG00000227834.1	ENSG00000234694.1	ENSG00000111846.17
27647	ENSG00000227835.8	ENSG00000234693.1	ENSG00000155256.17
27648	ENSG00000227836.1	ENSG00000234692.1	ENSG00000135596.18
27649	ENSG00000227837.1	ENSG00000234690.6	ENSG00000142541.17
27650	ENSG00000227838.1	ENSG00000234688.1	ENSG00000157954.15
27651	ENSG00000227840.1	ENSG00000234686.1	ENSG00000240979.1
27652	ENSG00000227841.1	ENSG00000234685.1	ENSG00000101310.16
27653	ENSG00000227842.1	ENSG00000234684.6	ENSG00000160345.13
27654	ENSG00000227845.2	ENSG00000234683.1	ENSG00000121089.4
27655	ENSG00000227846.1	ENSG00000234682.1	ENSG00000096654.15
27656	ENSG00000227847.1	ENSG00000234680.1	ENSG00000259626.2
27657	ENSG00000227848.1	ENSG00000234678.2	ENSG00000090905.19
27658	ENSG00000227850.1	ENSG00000234677.1	ENSG00000102048.15
27659	ENSG00000227851.1	ENSG00000234676.1	ENSG00000273432.1
27660	ENSG00000227852.1	ENSG00000234675.1	ENSG00000275465.6
27661	ENSG00000227854.1	ENSG00000234671.1	ENSG00000241612.1
27662	ENSG00000227855.3	ENSG00000234670.1	ENSG00000239556.4
27663	ENSG00000227857.2	ENSG00000234667.2	ENSG00000229622.1

27664	ENSG00000227860.1	ENSG00000234665.8	ENSG00000137055.15
27665	ENSG00000227862.1	ENSG00000234664.1	ENSG00000273210.1
27666	ENSG00000227863.2	ENSG00000234663.6	ENSG00000204882.3
27667	ENSG00000227864.1	ENSG00000234661.2	ENSG00000243094.1
27668	ENSG00000227867.1	ENSG00000234660.1	ENSG00000071655.18
27669	ENSG00000227868.6	ENSG00000234658.1	ENSG00000170471.15
27670	ENSG00000227871.1	ENSG00000234655.3	ENSG00000177191.2
27671	ENSG00000227873.1	ENSG00000234653.1	ENSG00000187134.14
27672	ENSG00000227874.1	ENSG00000234652.1	ENSG00000272078.1
27673	ENSG00000227875.2	ENSG00000234650.5	ENSG00000267278.5
27674	ENSG00000227877.6	ENSG00000234647.2	ENSG00000262700.1
27675	ENSG00000227878.2	ENSG00000234646.6	ENSG00000127952.17
27676	ENSG00000227879.2	ENSG00000234645.3	ENSG00000260645.2
27677	ENSG00000227880.1	ENSG00000234644.2	ENSG00000267565.1
27678	ENSG00000227881.1	ENSG00000234640.1	ENSG00000176155.18
27679	ENSG00000227882.1	ENSG00000234639.1	ENSG00000139239.7
27680	ENSG00000227883.1	ENSG00000234638.1	ENSG00000243830.2
27681	ENSG00000227885.2	ENSG00000234637.1	ENSG00000204277.1
27682	ENSG00000227887.1	ENSG00000234636.2	ENSG00000237499.6
27683	ENSG00000227888.4	ENSG00000234634.2	ENSG00000226744.1
27684	ENSG00000227890.1	ENSG00000234632.1	ENSG00000236197.3
27685	ENSG00000227892.1	ENSG00000234630.1	ENSG00000117298.16
27686	ENSG00000227893.1	ENSG00000234629.1	ENSG00000217716.3
27687	ENSG00000227895.5	ENSG00000234627.3	ENSG00000133678.14
27688	ENSG00000227896.2	ENSG00000234626.1	ENSG00000133393.13
27689	ENSG00000227902.2	ENSG00000234625.1	ENSG00000240401.8
27690	ENSG00000227905.1	ENSG00000234624.2	ENSG00000231255.1
27691	ENSG00000227906.7	ENSG00000234622.6_PAR_Y	ENSG00000229585.4
27692	ENSG00000227907.1	ENSG00000234622.6	ENSG00000286071.1
27693	ENSG00000227908.3	ENSG00000234620.1	ENSG00000249661.1
27694	ENSG00000227910.1	ENSG00000234619.1	ENSG00000109854.13
27695	ENSG00000227911.3	ENSG00000234618.1	ENSG00000108813.11
27696	ENSG00000227912.1	ENSG00000234617.2	ENSG00000178971.15
27697	ENSG00000227913.1	ENSG00000234616.8	ENSG00000100483.14
27698	ENSG00000227914.3	ENSG00000234614.1	ENSG00000184260.5
27699	ENSG00000227915.1	ENSG00000234613.1	ENSG00000176720.6
27700	ENSG00000227917.2	ENSG00000234612.1	ENSG00000100314.4
27701	ENSG00000227919.1	ENSG00000234611.1	ENSG00000265656.1
27702	ENSG00000227920.2	ENSG00000234608.7	ENSG00000206652.1
27703	ENSG00000227922.1	ENSG00000234604.2	ENSG00000088035.17
27704	ENSG00000227923.1	ENSG00000234602.7	ENSG00000124313.15
27705	ENSG00000227924.2	ENSG00000234601.1	ENSG00000273783.1
27706	ENSG00000227925.1	ENSG00000234597.1	ENSG00000165309.14
27707	ENSG00000227927.2	ENSG00000234595.1	ENSG00000226137.5
27708	ENSG00000227929.4	ENSG00000234594.1	ENSG00000256234.1
27709	ENSG00000227930.1	ENSG00000234593.2	ENSG00000258539.1
27710	ENSG00000227932.2	ENSG00000234592.1	ENSG00000080371.6
27711	ENSG00000227933.1	ENSG00000234590.1	ENSG00000165502.6
27712	ENSG00000227934.1	ENSG00000234589.4	ENSG00000102984.15
27713	ENSG00000227935.1	ENSG00000234588.1	ENSG00000136514.3
27714	ENSG00000227937.1	ENSG00000234587.1	ENSG00000279632.1
27715	ENSG00000227938.1	ENSG00000234586.2	ENSG00000072274.13
27716	ENSG00000227939.1	ENSG00000234585.6	ENSG00000275023.4

27717	ENSG00000227940.1	ENSG00000234584.1	ENSG00000205266.10
27718	ENSG00000227941.1	ENSG00000234583.1	ENSG00000119285.11
27719	ENSG00000227942.1	ENSG00000234580.1	ENSG00000231125.2
27720	ENSG00000227945.1	ENSG00000234579.1	ENSG00000122406.13
27721	ENSG00000227946.1	ENSG00000234578.1	ENSG00000164620.9
27722	ENSG00000227947.1	ENSG00000234577.1	ENSG00000168234.13
27723	ENSG00000227948.4	ENSG00000234576.2	ENSG00000240057.5
27724	ENSG00000227949.1	ENSG00000234575.2	ENSG00000176476.9
27725	ENSG00000227950.1	ENSG00000234573.1	ENSG00000228162.1
27726	ENSG00000227952.2	ENSG00000234572.1	ENSG00000269069.1
27727	ENSG00000227953.6	ENSG00000234571.1	ENSG00000237248.5
27728	ENSG00000227954.6	ENSG00000234570.1	ENSG00000168228.15
27729	ENSG00000227958.1	ENSG00000234569.1	ENSG00000177688.6
27730	ENSG00000227959.1	ENSG00000234568.3	ENSG00000273306.1
27731	ENSG00000227960.1	ENSG00000234567.1	ENSG00000133627.18
27732	ENSG00000227962.1	ENSG00000234566.1	ENSG00000196183.5
27733	ENSG00000227963.1	ENSG00000234565.1	ENSG00000273374.1
27734	ENSG00000227964.1	ENSG00000234564.1	ENSG00000119778.15
27735	ENSG00000227965.1	ENSG00000234562.1	ENSG00000271013.1
27736	ENSG00000227968.1	ENSG00000234560.4	ENSG00000184014.8
27737	ENSG00000227969.1	ENSG00000234559.1	ENSG00000267710.9
27738	ENSG00000227970.1	ENSG00000234558.1	ENSG00000215347.4
27739	ENSG00000227971.1	ENSG00000234557.2	ENSG00000182484.15
27740	ENSG00000227972.1	ENSG00000234556.1	ENSG00000105928.15
27741	ENSG00000227973.1	ENSG00000234553.1	ENSG00000273559.5
27742	ENSG00000227979.1	ENSG00000234551.1	ENSG00000115255.11
27743	ENSG00000227981.2	ENSG00000234548.6	ENSG00000242372.8
27744	ENSG00000227982.1	ENSG00000234546.3	ENSG00000166592.12
27745	ENSG00000227983.1	ENSG00000234545.7	ENSG00000231766.1
27746	ENSG00000227986.1	ENSG00000234544.2	ENSG00000138115.14
27747	ENSG00000227987.1	ENSG00000234542.1	ENSG00000241644.2
27748	ENSG00000227988.1	ENSG00000234541.2	ENSG00000273445.1
27749	ENSG00000227992.1	ENSG00000234540.1	ENSG00000152804.11
27750	ENSG00000227994.1	ENSG00000234538.1	ENSG00000198046.12
27751	ENSG00000227995.1	ENSG00000234537.1	ENSG00000108669.16
27752	ENSG00000227999.1	ENSG00000234535.1	ENSG00000260083.1
27753	ENSG00000228000.1	ENSG00000234534.1	ENSG00000222222.1
27754	ENSG00000228002.2	ENSG00000234529.2	ENSG00000100167.20
27755	ENSG00000228004.1	ENSG00000234527.1	ENSG00000165512.5
27756	ENSG00000228005.1	ENSG00000234524.1	ENSG00000279730.2
27757	ENSG00000228007.1	ENSG00000234523.1	ENSG00000260672.1
27758	ENSG00000228008.1	ENSG00000234522.1	ENSG00000258654.1
27759	ENSG00000228010.5	ENSG00000234521.2	ENSG00000258745.1
27760	ENSG00000228013.1	ENSG00000234520.5	ENSG00000230454.1
27761	ENSG00000228014.1	ENSG00000234519.1	ENSG00000236833.1
27762	ENSG00000228015.1	ENSG00000234515.1	ENSG00000254154.8
27763	ENSG00000228016.1	ENSG00000234513.1	ENSG00000276203.4
27764	ENSG00000228019.1	ENSG00000234512.1	ENSG00000175567.9
27765	ENSG00000228020.2	ENSG00000234511.10	ENSG00000169047.5
27766	ENSG00000228021.7	ENSG00000234509.1	ENSG00000236064.1
27767	ENSG00000228022.5	ENSG00000234506.5	ENSG00000272494.1
27768	ENSG00000228024.1	ENSG00000234504.2	ENSG00000088448.14
27769	ENSG00000228027.1	ENSG00000234503.1	ENSG00000084092.7

27770	ENSG00000228028.2	ENSG00000234502.2	ENSG00000164808.16
27771	ENSG00000228030.2	ENSG00000234500.1	ENSG00000232684.1
27772	ENSG00000228031.3	ENSG00000234498.3	ENSG00000161692.18
27773	ENSG00000228033.5	ENSG00000234497.5	ENSG00000230364.1
27774	ENSG00000228034.1	ENSG00000234496.2	ENSG00000258525.1
27775	ENSG00000228035.1	ENSG00000234494.7	ENSG00000124257.6
27776	ENSG00000228036.1	ENSG00000234493.4	ENSG00000231389.7
27777	ENSG00000228037.1	ENSG00000234492.4	ENSG00000264968.1
27778	ENSG00000228038.1	ENSG00000234491.1	ENSG00000027075.15
27779	ENSG00000228039.3	ENSG00000234489.1	ENSG00000275964.1
27780	ENSG00000228040.1	ENSG00000234488.1	ENSG00000156869.13
27781	ENSG00000228041.2	ENSG00000234485.5	ENSG00000149084.13
27782	ENSG00000228042.2	ENSG00000234484.1	ENSG00000168275.15
27783	ENSG00000228043.5	ENSG00000234481.1	ENSG00000109272.4
27784	ENSG00000228044.2	ENSG00000234480.1	ENSG00000213588.6
27785	ENSG00000228045.1	ENSG00000234479.2	ENSG00000165071.14
27786	ENSG00000228046.2	ENSG00000234478.1	ENSG00000227617.8
27787	ENSG00000228048.1	ENSG00000234477.1	ENSG00000118162.14
27788	ENSG00000228049.7	ENSG00000234476.2	ENSG00000257181.1
27789	ENSG00000228050.1	ENSG00000234474.2	ENSG00000238105.7
27790	ENSG00000228051.1	ENSG00000234473.1	ENSG00000239883.8
27791	ENSG00000228053.1	ENSG00000234471.1	ENSG00000136147.18
27792	ENSG00000228054.2	ENSG00000234469.4	ENSG00000172243.17
27793	ENSG00000228055.2	ENSG00000234467.1	ENSG00000252906.1
27794	ENSG00000228056.3	ENSG00000234466.1	ENSG00000139644.13
27795	ENSG00000228057.1	ENSG00000234465.11	ENSG00000187747.2
27796	ENSG00000228058.1	ENSG00000234464.1	ENSG00000013374.16
27797	ENSG00000228060.2	ENSG00000234463.1	ENSG00000269191.1
27798	ENSG00000228061.6	ENSG00000234460.2	ENSG00000127578.7
27799	ENSG00000228063.1	ENSG00000234459.1	ENSG00000256897.1
27800	ENSG00000228064.1	ENSG00000234458.4	ENSG00000260898.5
27801	ENSG00000228065.10	ENSG00000234457.1	ENSG00000148218.16
27802	ENSG00000228067.1	ENSG00000234456.7	ENSG00000223923.1
27803	ENSG00000228069.1	ENSG00000234455.1	ENSG00000101966.12
27804	ENSG00000228071.4	ENSG00000234452.5	ENSG00000261455.1
27805	ENSG00000228072.1	ENSG00000234451.1	ENSG00000240132.1
27806	ENSG00000228073.1	ENSG00000234450.1	ENSG00000269399.2
27807	ENSG00000228074.1	ENSG00000234448.1	ENSG00000137185.12
27808	ENSG00000228075.5	ENSG00000234446.2	ENSG00000249096.6
27809	ENSG00000228076.2	ENSG00000234445.1	ENSG00000281896.1
27810	ENSG00000228078.1	ENSG00000234444.10	ENSG00000272941.1
27811	ENSG00000228079.2	ENSG00000234442.4	ENSG00000274225.1
27812	ENSG00000228081.2	ENSG00000234441.1	ENSG00000162384.14
27813	ENSG00000228082.1	ENSG00000234439.1	ENSG00000091536.18
27814	ENSG00000228083.2	ENSG00000234438.4	ENSG00000197948.11
27815	ENSG00000228084.1	ENSG00000234436.1	ENSG00000171056.8
27816	ENSG00000228085.1	ENSG00000234435.2	ENSG00000158528.12
27817	ENSG00000228086.1	ENSG00000234429.1	ENSG00000212549.1
27818	ENSG00000228089.1	ENSG00000234428.2	ENSG00000231167.3
27819	ENSG00000228092.1	ENSG00000234427.1	ENSG00000115526.11
27820	ENSG00000228095.3	ENSG00000234426.2	ENSG00000163462.18
27821	ENSG00000228097.1	ENSG00000234425.1	ENSG00000267546.2
27822	ENSG00000228098.1	ENSG00000234424.2	ENSG00000279265.1

27823	ENSG00000228100.1	ENSG00000234423.1	ENSG00000111737.12
27824	ENSG00000228105.1	ENSG00000234422.1	ENSG00000173040.13
27825	ENSG00000228106.5	ENSG00000234421.2	ENSG00000138663.9
27826	ENSG00000228107.1	ENSG00000234420.7	ENSG00000138395.15
27827	ENSG00000228108.1	ENSG00000234419.1	ENSG00000276710.4
27828	ENSG00000228109.1	ENSG00000234418.1	ENSG00000075618.18
27829	ENSG00000228110.4	ENSG00000234415.1	ENSG00000224373.3
27830	ENSG00000228111.1	ENSG00000234414.7	ENSG00000136731.12
27831	ENSG00000228112.1	ENSG00000234413.1	ENSG00000143494.15
27832	ENSG00000228113.7	ENSG00000234409.6	ENSG00000138685.15
27833	ENSG00000228115.1	ENSG00000234406.1	ENSG00000163867.17
27834	ENSG00000228118.3	ENSG00000234405.1	ENSG00000256967.1
27835	ENSG00000228120.2	ENSG00000234404.2	ENSG00000239975.2
27836	ENSG00000228121.1	ENSG00000234402.1	ENSG00000236120.6
27837	ENSG00000228122.1	ENSG00000234399.1	ENSG00000197535.14
27838	ENSG00000228123.4	ENSG00000234398.1	ENSG00000168918.14
27839	ENSG00000228124.1	ENSG00000234397.3	ENSG00000163866.9
27840	ENSG00000228125.2	ENSG00000234396.3	ENSG00000175489.10
27841	ENSG00000228126.1	ENSG00000234394.8	ENSG00000058453.17
27842	ENSG00000228127.2	ENSG00000234393.1	ENSG00000121807.5
27843	ENSG00000228131.1	ENSG00000234391.1	ENSG00000260027.4
27844	ENSG00000228132.2	ENSG00000234390.4	ENSG00000262228.2
27845	ENSG00000228133.2	ENSG00000234389.1	ENSG00000120756.13
27846	ENSG00000228134.1	ENSG00000234388.2	ENSG00000188385.11
27847	ENSG00000228135.1	ENSG00000234387.1	ENSG00000279520.1
27848	ENSG00000228136.1	ENSG00000234386.4	ENSG00000252908.1
27849	ENSG00000228137.1	ENSG00000234385.1	ENSG00000176490.5
27850	ENSG00000228139.1	ENSG00000234384.1	ENSG00000225972.1
27851	ENSG00000228140.1	ENSG00000234383.1	ENSG00000234742.1
27852	ENSG00000228141.7	ENSG00000234382.2	ENSG00000285219.1
27853	ENSG00000228142.2	ENSG00000234381.1	ENSG00000276255.2
27854	ENSG00000228143.2	ENSG00000234380.2	ENSG00000277957.1
27855	ENSG00000228144.2	ENSG00000234379.1	ENSG00000166866.13
27856	ENSG00000228146.8	ENSG00000234378.1	ENSG00000133134.11
27857	ENSG00000228149.1	ENSG00000234377.7	ENSG00000136634.6
27858	ENSG00000228150.1	ENSG00000234376.4	ENSG00000088882.8
27859	ENSG00000228151.1	ENSG00000234373.1	ENSG00000080603.17
27860	ENSG00000228153.1	ENSG00000234371.6	ENSG00000162882.15
27861	ENSG00000228154.3	ENSG00000234369.1	ENSG00000137825.11
27862	ENSG00000228156.1	ENSG00000234367.1	ENSG00000170222.11
27863	ENSG00000228157.4	ENSG00000234363.1	ENSG00000175564.13
27864	ENSG00000228158.2	ENSG00000234362.5	ENSG00000101680.15
27865	ENSG00000228159.1	ENSG00000234361.1	ENSG00000247137.8
27866	ENSG00000228160.1	ENSG00000234360.1	ENSG00000102030.16
27867	ENSG00000228161.1	ENSG00000234359.3	ENSG00000214826.5
27868	ENSG00000228162.1	ENSG00000234358.1	ENSG00000275910.1
27869	ENSG00000228165.1	ENSG00000234356.1	ENSG00000237063.1
27870	ENSG00000228166.1	ENSG00000234355.3	ENSG00000280800.1
27871	ENSG00000228167.1	ENSG00000234354.3	ENSG00000181016.9
27872	ENSG00000228168.1	ENSG00000234353.2	ENSG00000115137.11
27873	ENSG00000228169.3	ENSG00000234352.9	ENSG00000232613.6
27874	ENSG00000228170.1	ENSG00000234350.4	ENSG00000145214.14
27875	ENSG00000228171.1	ENSG00000234349.1	ENSG00000274398.1

27876	ENSG00000228172.5	ENSG00000234345.2	ENSG00000111181.12
27877	ENSG00000228173.1	ENSG00000234340.1	ENSG00000178974.10
27878	ENSG00000228174.7	ENSG00000234338.1	ENSG00000177112.7
27879	ENSG00000228175.3	ENSG00000234337.4	ENSG00000158457.6
27880	ENSG00000228176.1	ENSG00000234336.6	ENSG00000179909.15
27881	ENSG00000228181.1	ENSG00000234335.1	ENSG00000161298.18
27882	ENSG00000228182.1	ENSG00000234333.2	ENSG00000085760.14
27883	ENSG00000228184.1	ENSG00000234332.1	ENSG00000235944.8
27884	ENSG00000228187.1	ENSG00000234329.1	ENSG00000225476.1
27885	ENSG00000228189.4	ENSG00000234327.7	ENSG00000130338.13
27886	ENSG00000228190.2	ENSG00000234325.1	ENSG00000198752.11
27887	ENSG00000228191.1	ENSG00000234324.1	ENSG00000256238.1
27888	ENSG00000228192.7	ENSG00000234322.1	ENSG00000197081.13
27889	ENSG00000228193.1	ENSG00000234319.1	ENSG00000171160.18
27890	ENSG00000228194.1	ENSG00000234318.1	ENSG00000114790.13
27891	ENSG00000228195.2	ENSG00000234315.1	ENSG00000162377.6
27892	ENSG00000228196.1	ENSG00000234312.1	ENSG00000224083.1
27893	ENSG00000228198.3	ENSG00000234311.1	ENSG00000236323.1
27894	ENSG00000228201.1	ENSG00000234309.1	ENSG00000271427.1
27895	ENSG00000228203.6	ENSG00000234308.2	ENSG00000206047.2
27896	ENSG00000228204.2	ENSG00000234306.1	ENSG00000230565.1
27897	ENSG00000228205.1	ENSG00000234304.2	ENSG00000115661.14
27898	ENSG00000228206.2	ENSG00000234303.2	ENSG00000007350.17
27899	ENSG00000228207.1	ENSG00000234300.1	ENSG00000231241.1
27900	ENSG00000228208.6	ENSG00000234297.2	ENSG00000274736.5
27901	ENSG00000228209.1	ENSG00000234296.1	ENSG00000149609.6
27902	ENSG00000228210.1	ENSG00000234293.1	ENSG00000159263.15
27903	ENSG00000228211.2	ENSG00000234292.3	ENSG00000211896.7
27904	ENSG00000228212.1	ENSG00000234290.2	ENSG00000236528.1
27905	ENSG00000228213.5	ENSG00000234289.5	ENSG00000139718.10
27906	ENSG00000228215.2	ENSG00000234288.2	ENSG00000230415.1
27907	ENSG00000228216.1	ENSG00000234287.1	ENSG00000279342.2
27908	ENSG00000228217.1	ENSG00000234286.1	ENSG00000254165.1
27909	ENSG00000228218.1	ENSG00000234285.1	ENSG00000123636.18
27910	ENSG00000228219.1	ENSG00000234284.6	ENSG00000057294.14
27911	ENSG00000228221.5	ENSG00000234283.1	ENSG00000196581.10
27912	ENSG00000228222.1	ENSG00000234281.5	ENSG00000107779.13
27913	ENSG00000228223.3	ENSG00000234279.1	ENSG00000176268.5
27914	ENSG00000228224.3	ENSG00000234278.3	ENSG00000175106.16
27915	ENSG00000228225.1	ENSG00000234277.2	ENSG00000064989.13
27916	ENSG00000228226.1	ENSG00000234276.1	ENSG00000181458.10
27917	ENSG00000228229.2	ENSG00000234275.3	ENSG00000136854.21
27918	ENSG00000228231.2	ENSG00000234274.1	ENSG00000258427.3
27919	ENSG00000228232.1	ENSG00000234273.1	ENSG00000236977.1
27920	ENSG00000228234.1	ENSG00000234272.1	ENSG00000134489.7
27921	ENSG00000228235.1	ENSG00000234271.1	ENSG00000273041.1
27922	ENSG00000228236.2	ENSG00000234270.1	ENSG00000224261.2
27923	ENSG00000228237.5	ENSG00000234269.2	ENSG00000156471.12
27924	ENSG00000228238.1	ENSG00000234268.1	ENSG00000262333.1
27925	ENSG00000228239.1	ENSG00000234267.3	ENSG00000184867.14
27926	ENSG00000228240.1	ENSG00000234265.1	ENSG00000255139.1
27927	ENSG00000228241.1	ENSG00000234264.1	ENSG00000173728.10
27928	ENSG00000228242.6	ENSG00000234263.1	ENSG00000253704.1

27929	ENSG00000228247.1	ENSG00000234262.1	ENSG00000132821.12
27930	ENSG00000228248.1	ENSG00000234261.3	ENSG00000101337.15
27931	ENSG00000228251.1	ENSG00000234259.3	ENSG00000116212.15
27932	ENSG00000228252.9	ENSG00000234257.1	ENSG00000211648.2
27933	ENSG00000228253.1	ENSG00000234256.2	ENSG00000169507.9
27934	ENSG00000228255.1	ENSG00000234255.8	ENSG00000271870.1
27935	ENSG00000228257.1	ENSG00000234253.1	ENSG00000258479.5
27936	ENSG00000228259.3	ENSG00000234248.1	ENSG00000272899.4
27937	ENSG00000228261.1	ENSG00000234247.1	ENSG00000131459.13
27938	ENSG00000228262.9	ENSG00000234244.1	ENSG00000253115.1
27939	ENSG00000228264.1	ENSG00000234241.1	ENSG00000185019.17
27940	ENSG00000228271.1	ENSG00000234239.1	ENSG00000213587.3
27941	ENSG00000228272.1	ENSG00000234238.1	ENSG00000168538.16
27942	ENSG00000228274.3	ENSG00000234235.1	ENSG00000239887.5
27943	ENSG00000228275.1	ENSG00000234234.1	ENSG00000261659.2
27944	ENSG00000228277.1	ENSG00000234233.1	ENSG00000166323.13
27945	ENSG00000228278.4	ENSG00000234232.7	ENSG00000156162.16
27946	ENSG00000228280.1	ENSG00000234231.4	ENSG00000158428.4
27947	ENSG00000228283.3	ENSG00000234230.1	ENSG00000279253.1
27948	ENSG00000228285.2	ENSG00000234229.8	ENSG00000105640.13
27949	ENSG00000228286.3	ENSG00000234228.1	ENSG00000234465.11
27950	ENSG00000228288.6	ENSG00000234227.1	ENSG00000185504.17
27951	ENSG00000228289.1	ENSG00000234226.5	ENSG00000235958.5
27952	ENSG00000228290.6	ENSG00000234225.2	ENSG00000227741.1
27953	ENSG00000228291.1	ENSG00000234224.2	ENSG00000276097.1
27954	ENSG00000228292.1	ENSG00000234223.2	ENSG00000111537.5
27955	ENSG00000228293.1	ENSG00000234222.6	ENSG00000230006.7
27956	ENSG00000228294.6	ENSG00000234219.1	ENSG00000270574.1
27957	ENSG00000228295.1	ENSG00000234217.2	ENSG00000215244.2
27958	ENSG00000228296.1	ENSG00000234215.2	ENSG00000111707.12
27959	ENSG00000228300.14	ENSG00000234213.1	ENSG00000177311.11
27960	ENSG00000228301.1	ENSG00000234211.2	ENSG00000279453.1
27961	ENSG00000228302.2	ENSG00000234210.1	ENSG00000266710.2
27962	ENSG00000228303.1	ENSG00000234208.1	ENSG00000178826.11
27963	ENSG00000228304.1	ENSG00000234207.1	ENSG00000260997.1
27964	ENSG00000228305.2	ENSG00000234206.5	ENSG00000270872.2
27965	ENSG00000228307.2	ENSG00000234204.1	ENSG00000174827.13
27966	ENSG00000228308.2	ENSG00000234203.1	ENSG00000181284.2
27967	ENSG00000228309.6	ENSG00000234202.2	ENSG00000235677.1
27968	ENSG00000228312.1	ENSG00000234201.1	ENSG00000182107.6
27969	ENSG00000228314.1	ENSG00000234200.2	ENSG00000277954.1
27970	ENSG00000228315.12	ENSG00000234197.1	ENSG00000270277.1
27971	ENSG00000228316.1	ENSG00000234193.1	ENSG00000266256.2
27972	ENSG00000228317.1	ENSG00000234192.1	ENSG00000279933.1
27973	ENSG00000228318.3	ENSG00000234191.1	ENSG00000238109.1
27974	ENSG00000228319.1	ENSG00000234190.1	ENSG00000279078.1
27975	ENSG00000228322.1	ENSG00000234189.1	ENSG00000211890.4
27976	ENSG00000228323.2	ENSG00000234187.1	ENSG00000273975.1
27977	ENSG00000228325.5	ENSG00000234186.2	ENSG00000136541.14
27978	ENSG00000228327.3	ENSG00000234185.2	ENSG00000271620.1
27979	ENSG00000228328.2	ENSG00000234184.5	ENSG00000185420.19
27980	ENSG00000228329.2	ENSG00000234183.1	ENSG00000158286.13
27981	ENSG00000228330.1	ENSG00000234182.1	ENSG00000011422.12

27982	ENSG00000228331.2	ENSG00000234181.1	ENSG00000105072.9
27983	ENSG00000228334.1	ENSG00000234179.1	ENSG00000283480.1
27984	ENSG00000228335.1	ENSG00000234178.1	ENSG00000250133.2
27985	ENSG00000228336.2	ENSG00000234177.5	ENSG00000087206.17
27986	ENSG00000228337.1	ENSG00000234176.1	ENSG00000251660.1
27987	ENSG00000228338.1	ENSG00000234175.1	ENSG00000236213.1
27988	ENSG00000228339.1	ENSG00000234174.1	ENSG00000112308.13
27989	ENSG00000228340.5	ENSG00000234173.1	ENSG00000116221.15
27990	ENSG00000228341.1	ENSG00000234172.1	ENSG00000255836.1
27991	ENSG00000228343.1	ENSG00000234171.2	ENSG00000196408.11
27992	ENSG00000228345.2	ENSG00000234170.5	ENSG00000285596.1
27993	ENSG00000228347.1	ENSG00000234169.1	ENSG00000103222.19
27994	ENSG00000228348.1	ENSG00000234168.2	ENSG00000155093.19
27995	ENSG00000228349.1	ENSG00000234167.1	ENSG00000278603.1
27996	ENSG00000228350.1	ENSG00000234166.1	ENSG00000093134.14
27997	ENSG00000228351.2	ENSG00000234163.1	ENSG00000100918.13
27998	ENSG00000228352.2	ENSG00000234162.2	ENSG00000158571.10
27999	ENSG00000228353.1	ENSG00000234161.1	ENSG00000279621.1
28000	ENSG00000228354.1	ENSG00000234160.1	ENSG00000144230.16
28001	ENSG00000228355.1	ENSG00000234159.1	ENSG00000285895.1
28002	ENSG00000228358.5	ENSG00000234158.1	ENSG00000248516.1
28003	ENSG00000228360.1	ENSG00000234156.2	ENSG00000284626.1
28004	ENSG00000228361.2	ENSG00000234155.1	ENSG00000137843.11
28005	ENSG00000228363.2	ENSG00000234152.1	ENSG00000066697.14
28006	ENSG00000228364.1	ENSG00000234149.1	ENSG00000185803.9
28007	ENSG00000228365.1	ENSG00000234148.2	ENSG00000140400.17
28008	ENSG00000228366.1	ENSG00000234147.1	ENSG00000279821.1
28009	ENSG00000228367.3	ENSG00000234146.1	ENSG00000172717.16
28010	ENSG00000228368.1	ENSG00000234145.1	ENSG00000237296.9
28011	ENSG00000228369.2	ENSG00000234144.1	ENSG00000246090.6
28012	ENSG00000228372.7	ENSG00000234143.1	ENSG00000129128.13
28013	ENSG00000228373.2	ENSG00000234142.1	ENSG00000253652.1
28014	ENSG00000228374.1	ENSG00000234141.1	ENSG00000180574.3
28015	ENSG00000228375.1	ENSG00000234139.3	ENSG00000127585.12
28016	ENSG00000228376.3	ENSG00000234136.1	ENSG00000278133.1
28017	ENSG00000228379.1	ENSG00000234135.2	ENSG00000090054.15
28018	ENSG00000228380.1	ENSG00000234132.2	ENSG00000237706.4
28019	ENSG00000228382.1	ENSG00000234131.1	ENSG00000258051.1
28020	ENSG00000228383.7	ENSG00000234130.2	ENSG00000178301.3
28021	ENSG00000228384.4	ENSG00000234129.7	ENSG00000205423.11
28022	ENSG00000228386.2	ENSG00000234127.9	ENSG00000132964.12
28023	ENSG00000228391.1	ENSG00000234125.5	ENSG00000135837.16
28024	ENSG00000228392.1	ENSG00000234124.6	ENSG00000214063.11
28025	ENSG00000228393.3	ENSG00000234123.1	ENSG00000164815.11
28026	ENSG00000228395.1	ENSG00000234122.1	ENSG00000228224.3
28027	ENSG00000228397.2	ENSG00000234121.1	ENSG00000259053.1
28028	ENSG00000228398.3	ENSG00000234120.1	ENSG00000225431.1
28029	ENSG00000228399.1	ENSG00000234119.1	ENSG00000241404.7
28030	ENSG00000228400.1	ENSG00000234118.1	ENSG00000065057.8
28031	ENSG00000228401.4	ENSG00000234117.2	ENSG00000227193.2
28032	ENSG00000228403.1	ENSG00000234116.1	ENSG00000167968.13
28033	ENSG00000228404.1	ENSG00000234115.2	ENSG00000166007.11
28034	ENSG00000228407.2	ENSG00000234112.2	ENSG00000260401.1

28035	ENSG00000228408.6	ENSG00000234111.6	ENSG00000285969.1
28036	ENSG00000228409.6	ENSG00000234110.1	ENSG00000258315.5
28037	ENSG00000228410.6	ENSG00000234109.1	ENSG00000173110.8
28038	ENSG00000228410.6_PAR_Y	ENSG00000234108.1	ENSG00000267811.1
28039	ENSG00000228411.1	ENSG00000234107.1	ENSG00000214659.4
28040	ENSG00000228412.8	ENSG00000234106.3	ENSG00000245148.2
28041	ENSG00000228413.1	ENSG00000234105.1	ENSG00000254815.5
28042	ENSG00000228414.6	ENSG00000234104.1	ENSG00000272578.5
28043	ENSG00000228415.3	ENSG00000234102.1	ENSG00000229924.3
28044	ENSG00000228417.1	ENSG00000234099.1	ENSG00000224389.9
28045	ENSG00000228420.1	ENSG00000234091.1	ENSG00000225914.2
28046	ENSG00000228421.2	ENSG00000234089.1	ENSG00000270016.1
28047	ENSG00000228422.4	ENSG00000234088.1	ENSG00000112697.16
28048	ENSG00000228423.2	ENSG00000234087.1	ENSG00000254779.4
28049	ENSG00000228426.1	ENSG00000234085.1	ENSG00000244217.1
28050	ENSG00000228427.2	ENSG00000234084.1	ENSG00000258794.3
28051	ENSG00000228429.1	ENSG00000234083.1	ENSG00000236790.5
28052	ENSG00000228430.9	ENSG00000234081.1	ENSG00000064309.14
28053	ENSG00000228431.1	ENSG00000234080.3	ENSG00000143365.18
28054	ENSG00000228432.1	ENSG00000234076.1	ENSG00000137502.10
28055	ENSG00000228433.2	ENSG00000234075.1	ENSG00000212916.4
28056	ENSG00000228434.1	ENSG00000234073.1	ENSG00000284753.1
28057	ENSG00000228436.2	ENSG00000234072.1	ENSG00000089639.11
28058	ENSG00000228437.5	ENSG00000234071.1	ENSG00000117385.15
28059	ENSG00000228438.1	ENSG00000234070.1	ENSG00000223899.1
28060	ENSG00000228440.1	ENSG00000234069.1	ENSG00000285006.1
28061	ENSG00000228444.2	ENSG00000234068.6	ENSG00000116783.15
28062	ENSG00000228445.1	ENSG00000234067.1	ENSG00000110921.14
28063	ENSG00000228446.2	ENSG00000234066.1	ENSG00000187189.10
28064	ENSG00000228450.2	ENSG00000234065.2	ENSG00000083097.14
28065	ENSG00000228451.4	ENSG00000234064.1	ENSG00000217241.1
28066	ENSG00000228452.1	ENSG00000234062.8	ENSG00000008056.14
28067	ENSG00000228453.1	ENSG00000234061.1	ENSG00000255553.1
28068	ENSG00000228459.3	ENSG00000234060.1	ENSG00000186283.14
28069	ENSG00000228460.1	ENSG00000234059.1	ENSG00000213090.2
28070	ENSG00000228462.1	ENSG00000234056.1	ENSG00000263400.7
28071	ENSG00000228463.10	ENSG00000234055.1	ENSG00000179766.19
28072	ENSG00000228464.1	ENSG00000234052.1	ENSG00000140939.14
28073	ENSG00000228465.1	ENSG00000234050.1	ENSG00000264350.1
28074	ENSG00000228466.1	ENSG00000234048.1	ENSG00000213625.9
28075	ENSG00000228467.2	ENSG00000234044.1	ENSG00000170325.14
28076	ENSG00000228470.1	ENSG00000234043.3	ENSG00000243260.3
28077	ENSG00000228471.1	ENSG00000234042.1	ENSG00000170919.15
28078	ENSG00000228473.1	ENSG00000234041.1	ENSG00000235946.1
28079	ENSG00000228474.6	ENSG00000234040.3	ENSG00000215644.10
28080	ENSG00000228476.1	ENSG00000234039.2	ENSG00000141959.17
28081	ENSG00000228477.1	ENSG00000234036.4	ENSG00000281920.1
28082	ENSG00000228478.1	ENSG00000234034.1	ENSG00000100154.14
28083	ENSG00000228480.1	ENSG00000234033.1	ENSG00000217236.2
28084	ENSG00000228481.1	ENSG00000234031.1	ENSG00000269293.2
28085	ENSG00000228482.1	ENSG00000234030.1	ENSG00000279488.1
28086	ENSG00000228484.2	ENSG00000234028.3	ENSG00000265727.2
28087	ENSG00000228485.1	ENSG00000234026.1	ENSG00000229677.1

28088	ENSG00000228486.10	ENSG00000234025.1	ENSG00000285851.1
28089	ENSG00000228487.2	ENSG00000234022.1	ENSG00000204469.12
28090	ENSG00000228488.1	ENSG00000234021.1	ENSG00000264714.1
28091	ENSG00000228489.1	ENSG00000234020.1	ENSG00000241769.7
28092	ENSG00000228492.2	ENSG00000234019.1	ENSG00000203288.3
28093	ENSG00000228495.1	ENSG00000234017.1	ENSG00000088356.6
28094	ENSG00000228496.1	ENSG00000234016.1	ENSG00000165916.8
28095	ENSG00000228499.1	ENSG00000234015.1	ENSG00000182902.14
28096	ENSG00000228501.2	ENSG00000234009.1	ENSG00000102119.10
28097	ENSG00000228502.1	ENSG00000234008.5	ENSG00000160226.16
28098	ENSG00000228503.1	ENSG00000234006.1	ENSG00000050555.18
28099	ENSG00000228504.1	ENSG00000234004.4	ENSG00000170266.16
28100	ENSG00000228505.5	ENSG00000234003.1	ENSG00000165698.16
28101	ENSG00000228506.2	ENSG00000234001.1	ENSG00000274177.1
28102	ENSG00000228507.1	ENSG00000233999.3	ENSG00000132793.11
28103	ENSG00000228509.5	ENSG00000233998.1	ENSG00000008282.8
28104	ENSG00000228513.1	ENSG00000233997.5	ENSG00000103494.14
28105	ENSG00000228517.2	ENSG00000233996.1	ENSG00000137642.13
28106	ENSG00000228518.1	ENSG00000233995.1	ENSG00000189409.13
28107	ENSG00000228519.3	ENSG00000233994.2	ENSG00000255284.1
28108	ENSG00000228521.2	ENSG00000233993.1	ENSG00000286162.1
28109	ENSG00000228522.2	ENSG00000233990.1	ENSG00000142694.6
28110	ENSG00000228523.1	ENSG00000233987.1	ENSG00000267801.1
28111	ENSG00000228525.1	ENSG00000233985.1	ENSG00000090263.16
28112	ENSG00000228526.7	ENSG00000233984.1	ENSG00000117410.14
28113	ENSG00000228527.1	ENSG00000233983.2	ENSG00000260279.3
28114	ENSG00000228528.1	ENSG00000233981.1	ENSG00000112701.18
28115	ENSG00000228530.1	ENSG00000233980.1	ENSG00000132294.15
28116	ENSG00000228532.4	ENSG00000233979.1	ENSG00000164171.11
28117	ENSG00000228536.2	ENSG00000233978.1	ENSG00000083896.12
28118	ENSG00000228538.6	ENSG00000233977.1	ENSG00000188229.6
28119	ENSG00000228539.1	ENSG00000233975.1	ENSG00000255557.1
28120	ENSG00000228540.1	ENSG00000233974.3	ENSG00000168575.10
28121	ENSG00000228541.1	ENSG00000233973.6	ENSG00000056736.10
28122	ENSG00000228543.1	ENSG00000233971.1	ENSG00000136636.13
28123	ENSG00000228544.1	ENSG00000233970.1	ENSG00000100253.13
28124	ENSG00000228546.2	ENSG00000233969.1	ENSG00000180233.11
28125	ENSG00000228547.5	ENSG00000233968.6	ENSG00000073670.14
28126	ENSG00000228548.1	ENSG00000233967.6	ENSG00000018510.15
28127	ENSG00000228549.3	ENSG00000233966.1	ENSG00000158482.10
28128	ENSG00000228550.1	ENSG00000233963.2	ENSG00000224003.1
28129	ENSG00000228551.1	ENSG00000233962.1	ENSG00000223561.6
28130	ENSG00000228553.1	ENSG00000233960.1	ENSG00000276248.1
28131	ENSG00000228554.1	ENSG00000233956.1	ENSG00000132463.14
28132	ENSG00000228557.1	ENSG00000233955.1	ENSG00000144395.18
28133	ENSG00000228559.1	ENSG00000233954.6	ENSG00000276707.1
28134	ENSG00000228560.1	ENSG00000233953.1	ENSG00000267296.2
28135	ENSG00000228561.2	ENSG00000233952.1	ENSG00000167767.14
28136	ENSG00000228563.1	ENSG00000233951.3	ENSG00000188321.13
28137	ENSG00000228564.2	ENSG00000233947.1	ENSG00000243071.1
28138	ENSG00000228566.1	ENSG00000233946.2	ENSG00000162881.6
28139	ENSG00000228567.3	ENSG00000233945.1	ENSG00000076053.10
28140	ENSG00000228568.1	ENSG00000233944.1	ENSG00000182504.11

28141	ENSG00000228570.7	ENSG00000233943.1	ENSG00000238287.1
28142	ENSG00000228571.1	ENSG00000233942.1	ENSG00000269897.5
28143	ENSG00000228572.7	ENSG00000233941.1	ENSG00000177275.4
28144	ENSG00000228572.7_PAR_Y	ENSG00000233940.1	ENSG00000233402.1
28145	ENSG00000228573.1	ENSG00000233939.1	ENSG00000283268.1
28146	ENSG00000228577.1	ENSG00000233937.6	ENSG00000259374.2
28147	ENSG00000228578.1	ENSG00000233936.1	ENSG00000220161.4
28148	ENSG00000228585.1	ENSG00000233934.2	ENSG00000186831.11
28149	ENSG00000228586.1	ENSG00000233933.2	ENSG00000213759.9
28150	ENSG00000228587.1	ENSG00000233932.6	ENSG00000164344.16
28151	ENSG00000228589.3	ENSG00000233930.3	ENSG00000260152.2
28152	ENSG00000228590.2	ENSG00000233929.1	ENSG00000060656.20
28153	ENSG00000228592.1	ENSG00000233928.5	ENSG00000156467.9
28154	ENSG00000228593.1	ENSG00000233927.5	ENSG00000255561.7
28155	ENSG00000228594.4	ENSG00000233926.1	ENSG00000279841.1
28156	ENSG00000228595.1	ENSG00000233924.1	ENSG00000241973.10
28157	ENSG00000228596.1	ENSG00000233922.2	ENSG00000101096.20
28158	ENSG00000228597.1	ENSG00000233921.2	ENSG00000132613.15
28159	ENSG00000228598.1	ENSG00000233920.2	ENSG00000235016.1
28160	ENSG00000228599.1	ENSG00000233919.1	ENSG00000129673.9
28161	ENSG00000228600.1	ENSG00000233918.1	ENSG00000171488.15
28162	ENSG00000228601.1	ENSG00000233917.8	ENSG00000237888.1
28163	ENSG00000228604.3	ENSG00000233916.1	ENSG00000055609.18
28164	ENSG00000228606.2	ENSG00000233915.4	ENSG00000270760.1
28165	ENSG00000228607.2	ENSG00000233913.7	ENSG00000267508.5
28166	ENSG00000228610.1	ENSG00000233912.1	ENSG00000171204.12
28167	ENSG00000228611.2	ENSG00000233910.1	ENSG00000272217.1
28168	ENSG00000228612.2	ENSG00000233909.1	ENSG00000116473.14
28169	ENSG00000228613.1	ENSG00000233908.1	ENSG00000279092.1
28170	ENSG00000228615.1	ENSG00000233907.1	ENSG00000166681.13
28171	ENSG00000228618.1	ENSG00000233906.1	ENSG00000240487.1
28172	ENSG00000228620.1	ENSG00000233905.1	ENSG00000161217.12
28173	ENSG00000228622.1	ENSG00000233902.1	ENSG00000158477.7
28174	ENSG00000228623.6	ENSG00000233901.5	ENSG00000126698.11
28175	ENSG00000228624.7	ENSG00000233900.1	ENSG00000279742.1
28176	ENSG00000228625.1	ENSG00000233896.2	ENSG00000242580.1
28177	ENSG00000228626.1	ENSG00000233894.1	ENSG00000100360.15
28178	ENSG00000228627.1	ENSG00000233893.2	ENSG00000105366.15
28179	ENSG00000228629.2	ENSG00000233892.1	ENSG00000279469.1
28180	ENSG00000228630.5	ENSG00000233891.7	ENSG00000010270.13
28181	ENSG00000228634.1	ENSG00000233888.2	ENSG00000238246.1
[truncated: 1,717,738 more chars]
