# Supplementary material for: Identification of Genes Associated with the Impairment of Olfactory and Gustatory Functions in COVID-19 via Machine-Learning Methods
Source: Life (Basel). 2023 Mar 15;13(3):798. doi: 10.3390/life13030798 (PMC10051382; doi:10.3390/life13030798)
Supplement: Supplementary file 1 [file life-13-00798-s001.zip › Table S2.pdf]

Supplementary Table S2: Performance of IFS with different classification algorithms.
#
(1) Performance of IFS with random forest on the list yielded by LASSO

Number of features	ACC	MCC	Precision	F1-measure
5	0.797	0.338	0.370	0.445
10	0.853	0.501	0.496	0.577
15	0.870	0.541	0.541	0.611
20	0.893	0.612	0.606	0.670
25	0.919	0.695	0.691	0.740
30	0.901	0.633	0.636	0.689
35	0.908	0.655	0.660	0.707
40	0.931	0.736	0.734	0.775
45	0.932	0.738	0.747	0.777
50	0.922	0.673	0.760	0.717
55	0.934	0.721	0.819	0.756
60	0.934	0.721	0.819	0.756
65	0.936	0.734	0.805	0.770
70	0.948	0.785	0.846	0.815
75	0.951	0.805	0.833	0.833
80	0.951	0.798	0.868	0.825
85	0.951	0.807	0.826	0.835
90	0.960	0.841	0.859	0.864
95	0.950	0.803	0.809	0.832
100	0.957	0.827	0.847	0.852
105	0.941	0.759	0.813	0.793
110	0.953	0.806	0.870	0.832
115	0.955	0.816	0.863	0.841
120	0.964	0.852	0.889	0.873
125	0.953	0.813	0.835	0.840
130	0.965	0.857	0.910	0.877
135	0.955	0.821	0.837	0.847
140	0.955	0.819	0.845	0.845
145	0.945	0.779	0.802	0.812
150	0.958	0.827	0.895	0.850
155	0.948	0.795	0.807	0.826
160	0.960	0.839	0.867	0.862
165	0.962	0.843	0.897	0.864
170	0.958	0.831	0.866	0.855
175	0.960	0.839	0.867	0.862
180	0.951	0.807	0.826	0.835
185	0.955	0.819	0.845	0.845
190	0.950	0.793	0.848	0.822
195	0.958	0.831	0.866	0.855

200	0.958	0.831	0.866	0.855
205	0.964	0.852	0.889	0.873
210	0.958	0.833	0.857	0.857
215	0.955	0.819	0.845	0.845
220	0.951	0.800	0.859	0.827
225	0.951	0.800	0.859	0.827
230	0.955	0.817	0.854	0.843
235	0.953	0.811	0.843	0.838
240	0.957	0.821	0.883	0.845
245	0.967	0.867	0.892	0.886
250	0.965	0.861	0.881	0.881
255	0.965	0.859	0.890	0.880
260	0.967	0.865	0.911	0.883
265	0.960	0.834	0.907	0.855
270	0.960	0.838	0.877	0.861
275	0.958	0.826	0.905	0.848
280	0.971	0.878	0.935	0.894
285	0.950	0.795	0.840	0.824
290	0.967	0.868	0.882	0.888
295	0.971	0.882	0.894	0.899
300	0.965	0.857	0.910	0.877
305	0.962	0.843	0.897	0.864
310	0.964	0.853	0.880	0.874
315	0.960	0.836	0.886	0.859
320	0.955	0.814	0.872	0.840
325	0.960	0.838	0.877	0.861
330	0.964	0.852	0.889	0.873
335	0.957	0.827	0.847	0.852
340	0.967	0.867	0.892	0.886
345	0.965	0.864	0.864	0.884
350	0.962	0.850	0.852	0.872
355	0.962	0.845	0.878	0.867
360	0.953	0.805	0.880	0.830
365	0.955	0.817	0.854	0.843
370	0.960	0.839	0.867	0.862
375	0.967	0.863	0.933	0.881
380	0.962	0.850	0.852	0.872
385	0.958	0.831	0.866	0.855
390	0.962	0.847	0.869	0.869
395	0.951	0.803	0.841	0.831
400	0.946	0.774	0.863	0.803
405	0.953	0.806	0.870	0.832
410	0.950	0.793	0.848	0.822

415	0.957	0.821	0.883	0.845
420	0.953	0.809	0.852	0.836
425	0.951	0.803	0.841	0.831
430	0.953	0.805	0.880	0.830
435	0.962	0.844	0.888	0.866
440	0.953	0.809	0.852	0.836
445	0.965	0.857	0.910	0.877
450	0.965	0.862	0.872	0.882
455	0.957	0.820	0.893	0.843
460	0.958	0.827	0.895	0.850
465	0.960	0.836	0.886	0.859
470	0.948	0.791	0.821	0.821
475	0.957	0.821	0.883	0.845
480	0.965	0.859	0.890	0.880
485	0.965	0.861	0.881	0.881
490	0.957	0.823	0.864	0.848
495	0.957	0.822	0.873	0.847
500	0.957	0.823	0.864	0.848
505	0.958	0.833	0.857	0.857
510	0.948	0.789	0.829	0.819
515	0.943	0.769	0.807	0.802
520	0.958	0.828	0.885	0.852
525	0.950	0.799	0.824	0.828
530	0.953	0.811	0.843	0.838
535	0.962	0.847	0.869	0.869
540	0.953	0.811	0.843	0.838
545	0.958	0.831	0.866	0.855
550	0.950	0.797	0.831	0.826
555	0.946	0.781	0.827	0.812
560	0.948	0.785	0.846	0.815
565	0.955	0.817	0.854	0.843
570	0.953	0.809	0.852	0.836
575	0.943	0.767	0.815	0.800
580	0.948	0.787	0.838	0.817
585	0.931	0.721	0.762	0.762
590	0.943	0.765	0.823	0.798
595	0.955	0.816	0.863	0.841
600	0.951	0.800	0.859	0.827
605	0.962	0.847	0.869	0.869
610	0.957	0.823	0.864	0.848
615	0.950	0.795	0.840	0.824
620	0.946	0.776	0.853	0.805
625	0.953	0.808	0.861	0.834

630	0.955	0.814	0.872	0.840
635	0.957	0.823	0.864	0.848
640	0.953	0.806	0.870	0.832
645	0.955	0.817	0.854	0.843
650	0.951	0.801	0.850	0.829
655	0.955	0.817	0.854	0.843
660	0.960	0.838	0.877	0.861
665	0.948	0.787	0.838	0.817
670	0.948	0.787	0.838	0.817
675	0.948	0.787	0.838	0.817
680	0.955	0.819	0.845	0.845
685	0.953	0.809	0.852	0.836
690	0.951	0.801	0.850	0.829
695	0.946	0.783	0.819	0.814
700	0.955	0.817	0.854	0.843
705	0.945	0.775	0.817	0.807
710	0.950	0.792	0.857	0.820
715	0.943	0.767	0.815	0.800
720	0.957	0.825	0.855	0.850
725	0.951	0.800	0.859	0.827
730	0.950	0.797	0.831	0.826
735	0.953	0.809	0.852	0.836
740	0.955	0.816	0.863	0.841
745	0.955	0.819	0.845	0.845
750	0.960	0.836	0.886	0.859
755	0.950	0.795	0.840	0.824
760	0.948	0.785	0.846	0.815
765	0.951	0.800	0.859	0.827
770	0.943	0.774	0.793	0.807
775	0.943	0.767	0.815	0.800
780	0.950	0.797	0.831	0.826
785	0.953	0.806	0.870	0.832
790	0.950	0.793	0.848	0.822
795	0.950	0.790	0.867	0.818
800	0.950	0.797	0.831	0.826
805	0.946	0.779	0.835	0.810
810	0.951	0.803	0.841	0.831
815	0.946	0.783	0.819	0.814
820	0.951	0.803	0.841	0.831
825	0.948	0.793	0.814	0.824
830	0.953	0.805	0.880	0.830
835	0.958	0.831	0.866	0.855
840	0.957	0.822	0.873	0.847

845	0.955	0.816	0.863	0.841
850	0.953	0.808	0.861	0.834
855	0.945	0.777	0.810	0.810
860	0.958	0.827	0.895	0.850
865	0.943	0.761	0.840	0.792
870	0.948	0.784	0.855	0.813
875	0.948	0.784	0.855	0.813
880	0.941	0.759	0.813	0.793
885	0.943	0.761	0.840	0.792
890	0.945	0.771	0.833	0.802
895	0.939	0.746	0.827	0.780
900	0.939	0.753	0.802	0.788
905	0.943	0.763	0.831	0.795
910	0.951	0.803	0.841	0.831
915	0.946	0.777	0.844	0.807
920	0.946	0.777	0.844	0.807
925	0.957	0.822	0.873	0.847
930	0.945	0.773	0.825	0.805
935	0.945	0.773	0.825	0.805
940	0.953	0.808	0.861	0.834
945	0.950	0.790	0.867	0.818
950	0.945	0.771	0.833	0.802
955	0.951	0.801	0.850	0.829
960	0.948	0.785	0.846	0.815
965	0.957	0.820	0.893	0.843
970	0.950	0.792	0.857	0.820
975	0.953	0.804	0.890	0.828
980	0.953	0.802	0.913	0.824
985	0.951	0.797	0.878	0.823
990	0.948	0.782	0.865	0.810
995	0.946	0.781	0.827	0.812
1000	0.955	0.813	0.882	0.838
1005	0.946	0.783	0.819	0.814
1010	0.953	0.804	0.890	0.828
1015	0.951	0.803	0.841	0.831
1020	0.950	0.789	0.877	0.815
1025	0.946	0.779	0.835	0.810
1030	0.955	0.817	0.854	0.843
1035	0.950	0.795	0.840	0.824
1040	0.936	0.734	0.805	0.770
1045	0.943	0.761	0.840	0.792
1050	0.950	0.790	0.867	0.818
1055	0.945	0.775	0.817	0.807

1060	0.950	0.795	0.840	0.824
1065	0.953	0.808	0.861	0.834
1070	0.950	0.799	0.824	0.828
1075	0.948	0.787	0.838	0.817
1080	0.955	0.814	0.872	0.840
1085	0.948	0.787	0.838	0.817
1090	0.945	0.773	0.825	0.805
1095	0.953	0.813	0.835	0.840
1100	0.943	0.767	0.815	0.800
1105	0.950	0.793	0.848	0.822
1110	0.941	0.763	0.798	0.798
1115	0.946	0.785	0.812	0.817
1120	0.951	0.800	0.859	0.827
1125	0.945	0.775	0.817	0.807
1130	0.946	0.787	0.805	0.819
1135	0.951	0.795	0.900	0.818
1140	0.948	0.781	0.875	0.808
1145	0.953	0.809	0.852	0.836
1150	0.941	0.761	0.805	0.795
1155	0.948	0.784	0.855	0.813
1160	0.943	0.763	0.831	0.795
1165	0.939	0.750	0.810	0.785
1170	0.945	0.771	0.833	0.802
1175	0.953	0.806	0.870	0.832
1180	0.950	0.797	0.831	0.826
1185	0.945	0.775	0.817	0.807
1190	0.946	0.781	0.827	0.812
1195	0.943	0.765	0.823	0.798
1200	0.945	0.775	0.817	0.807
1205	0.943	0.763	0.831	0.795
1210	0.950	0.793	0.848	0.822
1215	0.946	0.781	0.827	0.812
1220	0.945	0.782	0.795	0.814
1225	0.953	0.808	0.861	0.834
1230	0.955	0.816	0.863	0.841
1235	0.960	0.838	0.877	0.861
1240	0.943	0.767	0.815	0.800
1245	0.951	0.805	0.833	0.833
1250	0.941	0.755	0.829	0.788
1255	0.951	0.801	0.850	0.829
1260	0.948	0.785	0.846	0.815
1265	0.951	0.798	0.868	0.825
1270	0.951	0.800	0.859	0.827

1275	0.951	0.801	0.850	0.829
1280	0.946	0.781	0.827	0.812
1285	0.962	0.845	0.878	0.867
1290	0.946	0.781	0.827	0.812
1295	0.955	0.813	0.882	0.838
1300	0.950	0.793	0.848	0.822
1305	0.951	0.805	0.833	0.833
1310	0.950	0.792	0.857	0.820
1315	0.950	0.793	0.848	0.822
1320	0.950	0.793	0.848	0.822
1325	0.948	0.785	0.846	0.815
1330	0.951	0.803	0.841	0.831
1335	0.946	0.783	0.819	0.814
1340	0.951	0.798	0.868	0.825
1345	0.941	0.759	0.813	0.793
1350	0.953	0.806	0.870	0.832
1355	0.948	0.789	0.829	0.819
1360	0.955	0.814	0.872	0.840
1365	0.946	0.779	0.835	0.810
1370	0.946	0.781	0.827	0.812
1375	0.950	0.806	0.802	0.834
1380	0.955	0.823	0.830	0.849
1385	0.962	0.848	0.860	0.871
1390	0.957	0.825	0.855	0.850
1395	0.960	0.838	0.877	0.861
1400	0.958	0.833	0.857	0.857
1405	0.960	0.838	0.877	0.861
1410	0.967	0.865	0.911	0.883
1415	0.964	0.850	0.909	0.870
1420	0.955	0.817	0.854	0.843
1425	0.962	0.844	0.888	0.866
1430	0.969	0.871	0.934	0.888
1435	0.957	0.827	0.847	0.852
1440	0.969	0.875	0.893	0.893
1445	0.953	0.811	0.843	0.838
1450	0.962	0.845	0.878	0.867
1455	0.953	0.811	0.843	0.838
1460	0.964	0.851	0.899	0.871
1465	0.955	0.817	0.854	0.843
1470	0.960	0.835	0.896	0.857
1475	0.951	0.807	0.826	0.835
1480	0.965	0.858	0.900	0.878
1485	0.965	0.859	0.890	0.880

1490	0.958	0.835	0.849	0.859
1495	0.960	0.839	0.867	0.862
1500	0.962	0.844	0.888	0.866
1505	0.957	0.822	0.873	0.847
1510	0.953	0.808	0.861	0.834
1515	0.969	0.873	0.902	0.892
1520	0.955	0.819	0.845	0.845
1525	0.957	0.825	0.855	0.850
1530	0.955	0.816	0.863	0.841
1535	0.953	0.811	0.843	0.838
1540	0.955	0.819	0.845	0.845
1545	0.953	0.813	0.835	0.840
1550	0.948	0.789	0.829	0.819
1555	0.946	0.787	0.805	0.819
1560	0.962	0.845	0.878	0.867
1565	0.960	0.838	0.877	0.861
1570	0.967	0.865	0.911	0.883
1575	0.958	0.830	0.875	0.854
1580	0.950	0.793	0.848	0.822
1585	0.955	0.814	0.872	0.840
1590	0.953	0.809	0.852	0.836
1595	0.951	0.805	0.833	0.833
1600	0.958	0.831	0.866	0.855
1605	0.955	0.817	0.854	0.843
1610	0.962	0.844	0.888	0.866
1615	0.958	0.831	0.866	0.855
1620	0.958	0.833	0.857	0.857
1625	0.950	0.799	0.824	0.828
1630	0.955	0.821	0.837	0.847
1635	0.960	0.833	0.918	0.854
1640	0.946	0.785	0.812	0.817
1645	0.964	0.851	0.899	0.871
1650	0.958	0.831	0.866	0.855
1655	0.960	0.835	0.896	0.857
1660	0.957	0.822	0.873	0.847
1665	0.958	0.828	0.885	0.852
1670	0.964	0.856	0.862	0.877
1675	0.958	0.830	0.875	0.854
1680	0.967	0.868	0.882	0.888
1685	0.964	0.852	0.889	0.873
1690	0.962	0.845	0.878	0.867
1695	0.958	0.830	0.875	0.854
1700	0.960	0.839	0.867	0.862

1705	0.965	0.859	0.890	0.880
1710	0.953	0.808	0.861	0.834
1715	0.964	0.852	0.889	0.873
1720	0.950	0.795	0.840	0.824
1725	0.958	0.831	0.866	0.855
1730	0.967	0.865	0.911	0.883
1735	0.955	0.817	0.854	0.843
1740	0.951	0.807	0.826	0.835
1745	0.965	0.856	0.921	0.875
1750	0.955	0.816	0.863	0.841
1755	0.950	0.792	0.857	0.820
1760	0.953	0.813	0.835	0.840
1765	0.960	0.835	0.896	0.857
1770	0.945	0.777	0.810	0.810
1775	0.953	0.806	0.870	0.832
1780	0.946	0.790	0.798	0.821
1785	0.951	0.811	0.811	0.839
1790	0.962	0.843	0.897	0.864
1795	0.960	0.838	0.877	0.861
1800	0.962	0.844	0.888	0.866
1805	0.965	0.862	0.872	0.882
1810	0.955	0.819	0.845	0.845
1815	0.960	0.839	0.867	0.862
1820	0.960	0.841	0.859	0.864
1825	0.953	0.806	0.870	0.832
1830	0.957	0.829	0.839	0.854
1835	0.958	0.828	0.885	0.852
1840	0.962	0.844	0.888	0.866
1845	0.957	0.825	0.855	0.850
1850	0.957	0.827	0.847	0.852
1855	0.955	0.816	0.863	0.841
1860	0.950	0.789	0.877	0.815
1865	0.957	0.825	0.855	0.850
1870	0.946	0.781	0.827	0.812
1875	0.953	0.808	0.861	0.834
1880	0.951	0.803	0.841	0.831
1885	0.941	0.759	0.813	0.793
1890	0.953	0.805	0.880	0.830
1895	0.960	0.836	0.886	0.859
1900	0.948	0.791	0.821	0.821
1905	0.953	0.809	0.852	0.836
1910	0.951	0.805	0.833	0.833
1915	0.946	0.781	0.827	0.812

1920	0.948	0.785	0.846	0.815
1925	0.955	0.813	0.882	0.838
1930	0.953	0.813	0.835	0.840
1935	0.950	0.803	0.809	0.832
1940	0.958	0.833	0.857	0.857
1945	0.962	0.850	0.852	0.872
1950	0.958	0.838	0.833	0.862
1955	0.957	0.833	0.824	0.857
1960	0.948	0.795	0.807	0.826
1965	0.958	0.838	0.833	0.862
1970	0.962	0.847	0.869	0.869
1975	0.965	0.861	0.881	0.881
1980	0.953	0.815	0.828	0.842
1985	0.955	0.827	0.815	0.852
1990	0.958	0.835	0.849	0.859
1995	0.953	0.813	0.835	0.840
2000	0.962	0.850	0.852	0.872
2005	0.953	0.819	0.813	0.846
2010	0.962	0.848	0.860	0.871
2015	0.948	0.798	0.800	0.828
2020	0.955	0.821	0.837	0.847
2025	0.955	0.823	0.830	0.849
2030	0.958	0.835	0.849	0.859
2035	0.960	0.844	0.843	0.867
2040	0.951	0.803	0.841	0.831
2045	0.960	0.846	0.835	0.869
2050	0.960	0.844	0.843	0.867
2055	0.958	0.836	0.841	0.860
2060	0.957	0.831	0.831	0.855
2065	0.960	0.842	0.851	0.865
2070	0.957	0.827	0.847	0.852
2075	0.955	0.823	0.830	0.849
2080	0.957	0.831	0.831	0.855
2085	0.953	0.819	0.813	0.846
2090	0.960	0.841	0.859	0.864
2095	0.960	0.846	0.835	0.869
2100	0.951	0.811	0.811	0.839
2105	0.945	0.792	0.771	0.822
2110	0.962	0.848	0.860	0.871
2115	0.955	0.823	0.830	0.849
2120	0.960	0.842	0.851	0.865
2125	0.958	0.836	0.841	0.860
2130	0.965	0.861	0.881	0.881

2135	0.950	0.808	0.796	0.836
2140	0.955	0.821	0.837	0.847
2145	0.950	0.801	0.816	0.830
2150	0.951	0.811	0.811	0.839
2155	0.953	0.817	0.820	0.844
2160	0.962	0.850	0.852	0.872
2165	0.945	0.782	0.795	0.814
2170	0.951	0.811	0.811	0.839
2175	0.946	0.787	0.805	0.819
2180	0.958	0.840	0.826	0.864
2185	0.957	0.829	0.839	0.854
2190	0.960	0.842	0.851	0.865
2195	0.953	0.815	0.828	0.842
2200	0.962	0.850	0.852	0.872
2205	0.951	0.811	0.811	0.839
2210	0.958	0.836	0.841	0.860
2215	0.957	0.829	0.839	0.854
2220	0.953	0.821	0.806	0.847
2225	0.969	0.873	0.902	0.892
2230	0.967	0.866	0.901	0.885
2235	0.951	0.809	0.818	0.837
2240	0.955	0.821	0.837	0.847
2245	0.957	0.827	0.847	0.852
2250	0.953	0.815	0.828	0.842
2255	0.964	0.860	0.846	0.880
2260	0.964	0.854	0.871	0.876
2265	0.957	0.831	0.831	0.855
2270	0.965	0.865	0.856	0.885
2275	0.951	0.807	0.826	0.835
2280	0.962	0.848	0.860	0.871
2285	0.946	0.787	0.805	0.819
2290	0.960	0.844	0.843	0.867
2295	0.960	0.842	0.851	0.865
2300	0.965	0.865	0.856	0.885
2305	0.953	0.815	0.828	0.842
2310	0.958	0.833	0.857	0.857
2315	0.950	0.801	0.816	0.830
2320	0.951	0.814	0.804	0.841
2325	0.948	0.800	0.793	0.830
2330	0.955	0.819	0.845	0.845
2335	0.960	0.842	0.851	0.865
2340	0.951	0.811	0.811	0.839
2345	0.945	0.782	0.795	0.814

2350	0.946	0.792	0.791	0.823
2355	0.955	0.819	0.845	0.845
2360	0.955	0.821	0.837	0.847
2365	0.948	0.800	0.793	0.830
2370	0.953	0.811	0.843	0.838
2375	0.953	0.815	0.828	0.842
2380	0.951	0.807	0.826	0.835
2385	0.955	0.821	0.837	0.847
2390	0.955	0.823	0.830	0.849
2395	0.958	0.833	0.857	0.857
2400	0.955	0.823	0.830	0.849
2405	0.957	0.829	0.839	0.854
2410	0.955	0.817	0.854	0.843
2415	0.945	0.782	0.795	0.814
2420	0.955	0.821	0.837	0.847
2425	0.939	0.765	0.769	0.800
2430	0.950	0.799	0.824	0.828
2435	0.953	0.811	0.843	0.838
2440	0.948	0.798	0.800	0.828
2445	0.964	0.854	0.871	0.876
2450	0.946	0.792	0.791	0.823
2455	0.951	0.805	0.833	0.833
2460	0.957	0.827	0.847	0.852
2465	0.943	0.779	0.780	0.811
2470	0.953	0.813	0.835	0.840
2475	0.951	0.811	0.811	0.839
2480	0.969	0.876	0.884	0.894
2485	0.953	0.815	0.828	0.842
2490	0.955	0.821	0.837	0.847
2495	0.945	0.784	0.789	0.816
2500	0.955	0.825	0.822	0.851
2505	0.960	0.846	0.835	0.869
2510	0.955	0.823	0.830	0.849
2515	0.960	0.844	0.843	0.867
2520	0.960	0.841	0.859	0.864
2525	0.948	0.805	0.781	0.833
2530	0.953	0.817	0.820	0.844
2535	0.955	0.821	0.837	0.847
2540	0.943	0.787	0.763	0.818
2545	0.957	0.827	0.847	0.852
2550	0.953	0.815	0.828	0.842
2555	0.958	0.838	0.833	0.862
2560	0.955	0.823	0.830	0.849

2565	0.953	0.815	0.828	0.842
2570	0.960	0.848	0.828	0.870
2575	0.948	0.798	0.800	0.828
2580	0.953	0.817	0.820	0.844
2585	0.955	0.825	0.822	0.851
2590	0.953	0.819	0.813	0.846
2595	0.962	0.850	0.852	0.872
2600	0.953	0.817	0.820	0.844
2605	0.957	0.825	0.855	0.850
2610	0.945	0.779	0.802	0.812
2615	0.965	0.862	0.872	0.882
2620	0.946	0.790	0.798	0.821
2625	0.962	0.845	0.878	0.867
2630	0.955	0.819	0.845	0.845
2635	0.953	0.815	0.828	0.842
2640	0.960	0.841	0.859	0.864
2645	0.955	0.823	0.830	0.849
2650	0.955	0.825	0.822	0.851
2655	0.964	0.853	0.880	0.874
2660	0.948	0.798	0.800	0.828
2665	0.957	0.829	0.839	0.854
2670	0.957	0.829	0.839	0.854
2675	0.948	0.798	0.800	0.828
2680	0.957	0.827	0.847	0.852
2685	0.953	0.815	0.828	0.842
2690	0.953	0.813	0.835	0.840
2695	0.960	0.846	0.835	0.869
2700	0.964	0.856	0.862	0.877
2705	0.958	0.833	0.857	0.857
2710	0.960	0.839	0.867	0.862
2715	0.957	0.829	0.839	0.854
2720	0.950	0.799	0.824	0.828
2725	0.955	0.823	0.830	0.849
2730	0.962	0.850	0.852	0.872
2735	0.962	0.845	0.878	0.867
2740	0.955	0.819	0.845	0.845
2745	0.951	0.811	0.811	0.839
2750	0.964	0.860	0.846	0.880
2755	0.957	0.825	0.855	0.850
2760	0.960	0.841	0.859	0.864
2765	0.957	0.831	0.831	0.855
2770	0.950	0.806	0.802	0.834
2775	0.958	0.838	0.833	0.862

2780	0.955	0.825	0.822	0.851
2785	0.960	0.842	0.851	0.865
2790	0.957	0.829	0.839	0.854
2795	0.964	0.854	0.871	0.876
2800	0.946	0.790	0.798	0.821
2805	0.957	0.827	0.847	0.852
2810	0.955	0.821	0.837	0.847
2815	0.967	0.870	0.874	0.889
2820	0.958	0.835	0.849	0.859
2825	0.955	0.817	0.854	0.843
2830	0.955	0.825	0.822	0.851
2835	0.960	0.842	0.851	0.865
2840	0.958	0.836	0.841	0.860
2845	0.964	0.853	0.880	0.874
2850	0.960	0.846	0.835	0.869
2855	0.955	0.823	0.830	0.849
2860	0.965	0.861	0.881	0.881
2865	0.962	0.847	0.869	0.869
2870	0.964	0.858	0.854	0.879
2875	0.960	0.842	0.851	0.865
2880	0.960	0.844	0.843	0.867
2885	0.953	0.817	0.820	0.844
2890	0.958	0.831	0.866	0.855
2895	0.960	0.839	0.867	0.862
2900	0.950	0.808	0.796	0.836
2905	0.957	0.825	0.855	0.850
2910	0.957	0.829	0.839	0.854
2915	0.951	0.807	0.826	0.835
2920	0.957	0.833	0.824	0.857
2925	0.967	0.867	0.892	0.886
2930	0.950	0.806	0.802	0.834
2935	0.955	0.819	0.845	0.845
2940	0.953	0.824	0.800	0.849
2945	0.955	0.825	0.822	0.851
2950	0.958	0.838	0.833	0.862
2955	0.964	0.856	0.862	0.877
2960	0.945	0.782	0.795	0.814
2965	0.958	0.833	0.857	0.857
2970	0.958	0.833	0.857	0.857
2975	0.958	0.836	0.841	0.860
2980	0.953	0.817	0.820	0.844
2985	0.958	0.835	0.849	0.859
2990	0.955	0.817	0.854	0.843

2995	0.960	0.842	0.851	0.865
3000	0.957	0.825	0.855	0.850
3005	0.965	0.861	0.881	0.881
3010	0.965	0.859	0.890	0.880
3015	0.955	0.821	0.837	0.847
3020	0.958	0.836	0.841	0.860
3025	0.957	0.827	0.847	0.852
3030	0.957	0.823	0.864	0.848
3035	0.950	0.799	0.824	0.828
3040	0.960	0.841	0.859	0.864
3045	0.945	0.784	0.789	0.816
3050	0.950	0.799	0.824	0.828
3055	0.955	0.825	0.822	0.851
3060	0.955	0.827	0.815	0.852
3065	0.957	0.825	0.855	0.850
3070	0.957	0.831	0.831	0.855
3075	0.962	0.848	0.860	0.871
3080	0.950	0.799	0.824	0.828
3085	0.967	0.871	0.865	0.890
3090	0.957	0.827	0.847	0.852
3095	0.964	0.853	0.880	0.874
3100	0.960	0.841	0.859	0.864
3105	0.962	0.844	0.888	0.866
3110	0.950	0.799	0.824	0.828
3115	0.955	0.823	0.830	0.849
3120	0.960	0.838	0.877	0.861
3125	0.948	0.795	0.807	0.826
3130	0.955	0.825	0.822	0.851
3135	0.953	0.811	0.843	0.838
3140	0.950	0.799	0.824	0.828
3145	0.964	0.853	0.880	0.874
3150	0.946	0.792	0.791	0.823
3155	0.957	0.829	0.839	0.854
3160	0.965	0.859	0.890	0.880
3165	0.953	0.819	0.813	0.846
3170	0.955	0.821	0.837	0.847
3175	0.958	0.835	0.849	0.859
3180	0.953	0.815	0.828	0.842
3185	0.960	0.844	0.843	0.867
3190	0.964	0.854	0.871	0.876
3195	0.958	0.836	0.841	0.860
3200	0.955	0.823	0.830	0.849
3205	0.957	0.833	0.824	0.857

3210	0.960	0.841	0.859	0.864
3215	0.958	0.840	0.826	0.864
3220	0.958	0.831	0.866	0.855
3225	0.945	0.782	0.795	0.814
3230	0.962	0.852	0.844	0.874
3235	0.957	0.831	0.831	0.855
3240	0.960	0.844	0.843	0.867
3245	0.964	0.856	0.862	0.877
3250	0.965	0.865	0.856	0.885
3255	0.958	0.836	0.841	0.860
3260	0.960	0.841	0.859	0.864
3265	0.962	0.852	0.844	0.874
3270	0.960	0.846	0.835	0.869
3275	0.958	0.838	0.833	0.862
3280	0.955	0.827	0.815	0.852
3285	0.964	0.856	0.862	0.877
3290	0.960	0.844	0.843	0.867
3295	0.957	0.831	0.831	0.855
3300	0.969	0.879	0.867	0.897
3305	0.971	0.882	0.894	0.899
3310	0.971	0.882	0.894	0.899
3315	0.958	0.843	0.819	0.865
3320	0.958	0.840	0.826	0.864
3325	0.957	0.831	0.831	0.855
3330	0.964	0.861	0.839	0.881
3335	0.957	0.833	0.824	0.857
3340	0.955	0.825	0.822	0.851
3345	0.955	0.825	0.822	0.851
3350	0.958	0.840	0.826	0.864
3355	0.957	0.835	0.817	0.859
3360	0.957	0.831	0.831	0.855
3365	0.964	0.858	0.854	0.879
3370	0.965	0.865	0.856	0.885
3375	0.967	0.870	0.874	0.889
3380	0.962	0.850	0.852	0.872
3385	0.958	0.838	0.833	0.862
3390	0.962	0.848	0.860	0.871
3395	0.958	0.840	0.826	0.864
3400	0.969	0.873	0.902	0.892
3405	0.962	0.850	0.852	0.872
3410	0.950	0.808	0.796	0.836
3415	0.960	0.844	0.843	0.867
3420	0.965	0.865	0.856	0.885

3425	0.957	0.829	0.839	0.854
3430	0.953	0.824	0.800	0.849
3435	0.953	0.815	0.828	0.842
3440	0.958	0.836	0.841	0.860
3445	0.962	0.850	0.852	0.872
3450	0.962	0.854	0.837	0.875
3455	0.957	0.831	0.831	0.855
3460	0.950	0.806	0.802	0.834
3465	0.953	0.815	0.828	0.842
3470	0.962	0.850	0.852	0.872
3475	0.965	0.862	0.872	0.882
3480	0.962	0.847	0.869	0.869
3485	0.957	0.831	0.831	0.855
3490	0.965	0.867	0.848	0.886
3495	0.967	0.870	0.874	0.889
3500	0.974	0.896	0.906	0.911
3505	0.967	0.870	0.874	0.889
3510	0.976	0.902	0.927	0.916
3515	0.960	0.839	0.867	0.862
3520	0.971	0.883	0.885	0.901
3525	0.960	0.844	0.843	0.867
3530	0.967	0.868	0.882	0.888
3535	0.962	0.850	0.852	0.872
3540	0.958	0.835	0.849	0.859
3545	0.967	0.868	0.882	0.888
3550	0.962	0.848	0.860	0.871
3555	0.958	0.836	0.841	0.860
3560	0.958	0.831	0.866	0.855
3565	0.957	0.823	0.864	0.848
3570	0.957	0.827	0.847	0.852
3575	0.962	0.843	0.897	0.864
3580	0.969	0.875	0.893	0.893
3585	0.958	0.833	0.857	0.857
3590	0.969	0.877	0.875	0.895
3595	0.957	0.823	0.864	0.848
3600	0.964	0.854	0.871	0.876
3605	0.967	0.870	0.874	0.889
3610	0.962	0.845	0.878	0.867
3615	0.958	0.838	0.833	0.862
3620	0.958	0.835	0.849	0.859
3625	0.964	0.854	0.871	0.876
3630	0.958	0.833	0.857	0.857
3635	0.958	0.833	0.857	0.857

3640	0.962	0.845	0.878	0.867
3645	0.965	0.864	0.864	0.884
3650	0.962	0.854	0.837	0.875
3655	0.955	0.825	0.822	0.851
3660	0.958	0.840	0.826	0.864
3665	0.958	0.833	0.857	0.857
3670	0.962	0.845	0.878	0.867
3675	0.960	0.846	0.835	0.869
3680	0.965	0.864	0.864	0.884
3685	0.958	0.835	0.849	0.859
3690	0.965	0.861	0.881	0.881
3695	0.960	0.844	0.843	0.867
3700	0.960	0.842	0.851	0.865
3705	0.958	0.838	0.833	0.862
3710	0.955	0.827	0.815	0.852
3715	0.964	0.858	0.854	0.879
3720	0.958	0.831	0.866	0.855
3725	0.962	0.854	0.837	0.875
3730	0.965	0.859	0.890	0.880
3735	0.965	0.859	0.890	0.880
3740	0.960	0.844	0.843	0.867
3745	0.964	0.860	0.846	0.880
3750	0.957	0.835	0.817	0.859
3755	0.964	0.856	0.862	0.877
3760	0.962	0.854	0.837	0.875
3765	0.964	0.856	0.862	0.877
3770	0.965	0.861	0.881	0.881
3775	0.967	0.868	0.882	0.888
3780	0.965	0.865	0.856	0.885
3785	0.967	0.870	0.874	0.889
3790	0.967	0.870	0.874	0.889
3795	0.958	0.840	0.826	0.864
3800	0.965	0.862	0.872	0.882
3805	0.955	0.819	0.845	0.845
3810	0.969	0.873	0.902	0.892
3815	0.962	0.845	0.878	0.867
3820	0.955	0.821	0.837	0.847
3825	0.964	0.858	0.854	0.879
3830	0.958	0.838	0.833	0.862
3835	0.967	0.870	0.874	0.889
3840	0.967	0.867	0.892	0.886
3845	0.962	0.848	0.860	0.871
3850	0.967	0.867	0.892	0.886

3855	0.964	0.856	0.862	0.877
3860	0.964	0.853	0.880	0.874
3865	0.967	0.866	0.901	0.885
3870	0.955	0.821	0.837	0.847
3875	0.965	0.861	0.881	0.881
3880	0.972	0.889	0.905	0.905
3885	0.960	0.842	0.851	0.865
3890	0.965	0.861	0.881	0.881
3895	0.967	0.867	0.892	0.886
3900	0.951	0.811	0.811	0.839
3905	0.957	0.829	0.839	0.854
3910	0.964	0.858	0.854	0.879
3915	0.964	0.852	0.889	0.873
3920	0.962	0.854	0.837	0.875
3925	0.965	0.861	0.881	0.881
3930	0.960	0.842	0.851	0.865
3935	0.951	0.803	0.841	0.831
3940	0.964	0.853	0.880	0.874
3945	0.960	0.838	0.877	0.861
3950	0.957	0.827	0.847	0.852
3955	0.948	0.795	0.807	0.826
3960	0.965	0.859	0.890	0.880
3965	0.958	0.828	0.885	0.852
3970	0.962	0.845	0.878	0.867
3975	0.957	0.825	0.855	0.850
3980	0.962	0.845	0.878	0.867
3985	0.962	0.847	0.869	0.869
3990	0.950	0.792	0.857	0.820
3995	0.957	0.829	0.839	0.854
4000	0.955	0.825	0.822	0.851
4005	0.962	0.847	0.869	0.869
4010	0.969	0.873	0.902	0.892
4015	0.957	0.823	0.864	0.848
4020	0.957	0.825	0.855	0.850
4025	0.957	0.829	0.839	0.854
4030	0.960	0.836	0.886	0.859
4035	0.967	0.865	0.911	0.883
4040	0.955	0.825	0.822	0.851
4045	0.957	0.825	0.855	0.850
4050	0.964	0.851	0.899	0.871
4055	0.971	0.879	0.924	0.896
4060	0.953	0.815	0.828	0.842
4065	0.953	0.811	0.843	0.838

4070	0.958	0.831	0.866	0.855
4075	0.955	0.819	0.845	0.845
4080	0.964	0.851	0.899	0.871
4085	0.948	0.791	0.821	0.821
4090	0.962	0.847	0.869	0.869
4095	0.967	0.865	0.911	0.883
4100	0.965	0.858	0.900	0.878
4105	0.958	0.836	0.841	0.860
4110	0.958	0.833	0.857	0.857
4115	0.964	0.854	0.871	0.876
4120	0.964	0.856	0.862	0.877
4125	0.971	0.880	0.914	0.897
4130	0.964	0.854	0.871	0.876
4135	0.948	0.795	0.807	0.826
4140	0.964	0.854	0.871	0.876
4145	0.958	0.833	0.857	0.857
4150	0.958	0.831	0.866	0.855
4155	0.953	0.815	0.828	0.842
4160	0.964	0.853	0.880	0.874
4165	0.967	0.868	0.882	0.888
4170	0.965	0.858	0.900	0.878
4175	0.962	0.848	0.860	0.871
4180	0.967	0.868	0.882	0.888
4185	0.965	0.861	0.881	0.881
4190	0.964	0.854	0.871	0.876
4195	0.960	0.842	0.851	0.865
4200	0.957	0.827	0.847	0.852
4205	0.964	0.850	0.909	0.870
4210	0.962	0.843	0.897	0.864
4215	0.955	0.816	0.863	0.841
4220	0.958	0.830	0.875	0.854
4225	0.964	0.854	0.871	0.876
4230	0.958	0.830	0.875	0.854
4235	0.960	0.839	0.867	0.862
4240	0.953	0.811	0.843	0.838
4245	0.951	0.807	0.826	0.835
4250	0.964	0.854	0.871	0.876
4255	0.951	0.805	0.833	0.833
4260	0.953	0.811	0.843	0.838
4265	0.962	0.845	0.878	0.867
4270	0.964	0.853	0.880	0.874
4275	0.964	0.849	0.920	0.868
4280	0.960	0.842	0.851	0.865

4285	0.936	0.739	0.790	0.776
4290	0.962	0.848	0.860	0.871
4295	0.958	0.835	0.849	0.859
4300	0.962	0.845	0.878	0.867
4305	0.962	0.845	0.878	0.867
4310	0.957	0.823	0.864	0.848
4315	0.951	0.805	0.833	0.833
4320	0.951	0.803	0.841	0.831
4325	0.958	0.833	0.857	0.857
4330	0.948	0.795	0.807	0.826
4335	0.958	0.831	0.866	0.855
4340	0.957	0.827	0.847	0.852
4345	0.957	0.825	0.855	0.850
4350	0.946	0.787	0.805	0.819
4355	0.951	0.805	0.833	0.833
4360	0.955	0.817	0.854	0.843
4365	0.960	0.839	0.867	0.862
4370	0.953	0.811	0.843	0.838
4375	0.960	0.838	0.877	0.861
4380	0.946	0.790	0.798	0.821
4385	0.957	0.827	0.847	0.852
4390	0.967	0.868	0.882	0.888
4395	0.955	0.817	0.854	0.843
4400	0.955	0.825	0.822	0.851
4405	0.958	0.830	0.875	0.854
4410	0.950	0.795	0.840	0.824
4415	0.971	0.878	0.935	0.894
4420	0.958	0.833	0.857	0.857
4425	0.964	0.851	0.899	0.871
4430	0.957	0.821	0.883	0.845
4435	0.957	0.822	0.873	0.847
4440	0.953	0.815	0.828	0.842
4445	0.964	0.851	0.899	0.871
4450	0.962	0.847	0.869	0.869
4455	0.962	0.845	0.878	0.867
4460	0.958	0.830	0.875	0.854
4465	0.960	0.841	0.859	0.864
4470	0.960	0.839	0.867	0.862
4475	0.965	0.862	0.872	0.882
4480	0.957	0.825	0.855	0.850
4485	0.965	0.862	0.872	0.882
4490	0.962	0.844	0.888	0.866
4495	0.957	0.825	0.855	0.850

4500	0.964	0.852	0.889	0.873
4505	0.965	0.858	0.900	0.878
4510	0.958	0.831	0.866	0.855
4515	0.953	0.811	0.843	0.838
4520	0.962	0.847	0.869	0.869
4525	0.964	0.854	0.871	0.876
4530	0.957	0.822	0.873	0.847
4535	0.950	0.797	0.831	0.826
4540	0.967	0.868	0.882	0.888
4545	0.948	0.793	0.814	0.824
4550	0.951	0.807	0.826	0.835
4555	0.958	0.833	0.857	0.857
4560	0.967	0.870	0.874	0.889
4565	0.965	0.859	0.890	0.880
4570	0.955	0.817	0.854	0.843
4575	0.955	0.817	0.854	0.843
4580	0.955	0.816	0.863	0.841
4585	0.950	0.797	0.831	0.826
4590	0.958	0.833	0.857	0.857
4595	0.962	0.845	0.878	0.867
4600	0.958	0.827	0.895	0.850
4605	0.965	0.861	0.881	0.881
4610	0.958	0.830	0.875	0.854
4615	0.946	0.785	0.812	0.817
4620	0.965	0.859	0.890	0.880
4625	0.965	0.857	0.910	0.877
4630	0.957	0.827	0.847	0.852
4635	0.960	0.841	0.859	0.864
4640	0.951	0.803	0.841	0.831
4645	0.960	0.844	0.843	0.867
4650	0.964	0.853	0.880	0.874
4655	0.958	0.828	0.885	0.852
4660	0.946	0.779	0.835	0.810
4665	0.960	0.836	0.886	0.859
4670	0.955	0.819	0.845	0.845
4675	0.946	0.787	0.805	0.819
4680	0.958	0.831	0.866	0.855
4685	0.955	0.816	0.863	0.841
4690	0.955	0.819	0.845	0.845
4695	0.960	0.841	0.859	0.864
4700	0.951	0.805	0.833	0.833
4705	0.951	0.801	0.850	0.829
4710	0.964	0.851	0.899	0.871

4715	0.958	0.831	0.866	0.855
4720	0.962	0.844	0.888	0.866
4725	0.957	0.827	0.847	0.852
4730	0.948	0.791	0.821	0.821
4735	0.965	0.861	0.881	0.881
4740	0.960	0.841	0.859	0.864
4745	0.962	0.850	0.852	0.872
4750	0.945	0.777	0.810	0.810
4755	0.948	0.795	0.807	0.826
4760	0.960	0.838	0.877	0.861
4765	0.960	0.839	0.867	0.862
4770	0.960	0.836	0.886	0.859
4775	0.960	0.841	0.859	0.864
4780	0.958	0.836	0.841	0.860
4785	0.953	0.817	0.820	0.844
4790	0.951	0.805	0.833	0.833
4795	0.957	0.827	0.847	0.852
4800	0.964	0.852	0.889	0.873
4805	0.958	0.831	0.866	0.855
4810	0.958	0.831	0.866	0.855
4815	0.951	0.803	0.841	0.831
4820	0.945	0.777	0.810	0.810
4825	0.960	0.839	0.867	0.862
4830	0.951	0.801	0.850	0.829
4835	0.960	0.836	0.886	0.859
4840	0.957	0.827	0.847	0.852
4845	0.962	0.844	0.888	0.866
4850	0.957	0.822	0.873	0.847
4855	0.969	0.871	0.934	0.888
4860	0.955	0.819	0.845	0.845
4865	0.957	0.827	0.847	0.852
4870	0.960	0.838	0.877	0.861
4875	0.955	0.819	0.845	0.845
4880	0.951	0.807	0.826	0.835
4885	0.951	0.805	0.833	0.833
4890	0.958	0.836	0.841	0.860
4895	0.951	0.807	0.826	0.835
4900	0.957	0.821	0.883	0.845
4905	0.953	0.809	0.852	0.836
4910	0.965	0.858	0.900	0.878
4915	0.953	0.813	0.835	0.840
4920	0.953	0.806	0.870	0.832
4925	0.955	0.821	0.837	0.847

4930	0.957	0.822	0.873	0.847
4935	0.941	0.771	0.778	0.805
4940	0.953	0.813	0.835	0.840
4945	0.953	0.815	0.828	0.842
4950	0.960	0.839	0.867	0.862
4955	0.953	0.811	0.843	0.838
4960	0.957	0.825	0.855	0.850
4965	0.951	0.807	0.826	0.835
4970	0.953	0.815	0.828	0.842
4975	0.955	0.819	0.845	0.845
4980	0.965	0.864	0.864	0.884
4985	0.958	0.838	0.833	0.862
4990	0.958	0.831	0.866	0.855
4995	0.955	0.825	0.822	0.851
5000	0.948	0.789	0.829	0.819
5005	0.962	0.847	0.869	0.869
5010	0.958	0.835	0.849	0.859
5015	0.958	0.835	0.849	0.859
5020	0.964	0.854	0.871	0.876
5025	0.946	0.787	0.805	0.819
5030	0.962	0.842	0.908	0.863
5035	0.960	0.835	0.896	0.857
5040	0.955	0.823	0.830	0.849
5045	0.957	0.829	0.839	0.854
5050	0.967	0.867	0.892	0.886
5055	0.960	0.838	0.877	0.861
5060	0.957	0.825	0.855	0.850
5065	0.962	0.844	0.888	0.866
5070	0.960	0.839	0.867	0.862
5075	0.955	0.816	0.863	0.841
5080	0.955	0.819	0.845	0.845
5085	0.969	0.872	0.913	0.890
5090	0.958	0.833	0.857	0.857
5095	0.967	0.867	0.892	0.886
5100	0.967	0.868	0.882	0.888
5105	0.958	0.831	0.866	0.855
5110	0.964	0.852	0.889	0.873
5115	0.957	0.827	0.847	0.852
5120	0.962	0.848	0.860	0.871
5125	0.957	0.829	0.839	0.854
5130	0.964	0.853	0.880	0.874
5135	0.962	0.845	0.878	0.867
5140	0.953	0.809	0.852	0.836

5145	0.957	0.825	0.855	0.850
5150	0.960	0.841	0.859	0.864
5155	0.967	0.866	0.901	0.885
5160	0.958	0.833	0.857	0.857
5165	0.958	0.831	0.866	0.855
5170	0.953	0.813	0.835	0.840
5175	0.967	0.866	0.901	0.885
5180	0.958	0.833	0.857	0.857
5185	0.957	0.823	0.864	0.848
5190	0.965	0.859	0.890	0.880
5195	0.950	0.799	0.824	0.828
5200	0.946	0.783	0.819	0.814
5205	0.971	0.880	0.914	0.897
5210	0.955	0.817	0.854	0.843
5215	0.964	0.853	0.880	0.874
5220	0.958	0.835	0.849	0.859
5225	0.960	0.841	0.859	0.864
5230	0.950	0.803	0.809	0.832
5235	0.962	0.844	0.888	0.866
5240	0.958	0.830	0.875	0.854
5245	0.957	0.822	0.873	0.847
5250	0.960	0.838	0.877	0.861
5255	0.962	0.845	0.878	0.867
5260	0.962	0.844	0.888	0.866
5265	0.964	0.851	0.899	0.871
5270	0.962	0.848	0.860	0.871
5275	0.958	0.833	0.857	0.857
5280	0.960	0.842	0.851	0.865
5285	0.972	0.891	0.886	0.907
5290	0.955	0.817	0.854	0.843
5295	0.962	0.844	0.888	0.866
5300	0.962	0.843	0.897	0.864
5305	0.955	0.814	0.872	0.840
5310	0.967	0.866	0.901	0.885
5315	0.962	0.844	0.888	0.866
5320	0.960	0.842	0.851	0.865
5325	0.955	0.816	0.863	0.841
5330	0.951	0.801	0.850	0.829
5335	0.965	0.859	0.890	0.880
5340	0.962	0.845	0.878	0.867
5345	0.957	0.825	0.855	0.850
5350	0.965	0.859	0.890	0.880
5355	0.953	0.815	0.828	0.842

5360	0.957	0.829	0.839	0.854
5365	0.960	0.838	0.877	0.861
5370	0.955	0.819	0.845	0.845
5375	0.951	0.801	0.850	0.829
5380	0.943	0.774	0.793	0.807
5385	0.955	0.816	0.863	0.841
5390	0.953	0.811	0.843	0.838
5395	0.958	0.835	0.849	0.859
5400	0.955	0.816	0.863	0.841
5405	0.964	0.852	0.889	0.873
5410	0.953	0.809	0.852	0.836
5415	0.964	0.853	0.880	0.874
5420	0.955	0.821	0.837	0.847
5425	0.964	0.858	0.854	0.879
5430	0.960	0.839	0.867	0.862
5435	0.965	0.859	0.890	0.880
5440	0.957	0.829	0.839	0.854
5445	0.955	0.821	0.837	0.847
5450	0.965	0.861	0.881	0.881
5455	0.951	0.814	0.804	0.841
5460	0.957	0.823	0.864	0.848
5465	0.955	0.821	0.837	0.847
5470	0.964	0.854	0.871	0.876
5475	0.955	0.819	0.845	0.845
5480	0.957	0.822	0.873	0.847
5485	0.965	0.858	0.900	0.878
5490	0.958	0.828	0.885	0.852
5495	0.958	0.830	0.875	0.854
5500	0.965	0.857	0.910	0.877
5505	0.960	0.839	0.867	0.862
5510	0.969	0.872	0.923	0.889
5515	0.950	0.792	0.857	0.820
5520	0.958	0.833	0.857	0.857
5525	0.955	0.819	0.845	0.845
5530	0.957	0.822	0.873	0.847
5535	0.958	0.830	0.875	0.854
5540	0.962	0.842	0.908	0.863
5545	0.962	0.847	0.869	0.869
5550	0.960	0.836	0.886	0.859
5555	0.945	0.775	0.817	0.807
5560	0.946	0.783	0.819	0.814
5565	0.962	0.844	0.888	0.866
5570	0.958	0.831	0.866	0.855

5575	0.958	0.833	0.857	0.857
5580	0.964	0.851	0.899	0.871
5585	0.957	0.829	0.839	0.854
5590	0.962	0.847	0.869	0.869
5595	0.953	0.815	0.828	0.842
5600	0.953	0.815	0.828	0.842
5605	0.962	0.843	0.897	0.864
5610	0.951	0.805	0.833	0.833
5615	0.964	0.853	0.880	0.874
5620	0.948	0.793	0.814	0.824
5625	0.958	0.833	0.857	0.857
5630	0.965	0.864	0.864	0.884
5635	0.950	0.797	0.831	0.826
5640	0.950	0.799	0.824	0.828
5645	0.953	0.809	0.852	0.836
5650	0.950	0.795	0.840	0.824
5655	0.967	0.867	0.892	0.886
5660	0.957	0.823	0.864	0.848
5665	0.960	0.834	0.907	0.855
5670	0.962	0.844	0.888	0.866
5675	0.951	0.800	0.859	0.827
5680	0.953	0.808	0.861	0.834
5685	0.960	0.835	0.896	0.857
5690	0.955	0.819	0.845	0.845
5695	0.950	0.799	0.824	0.828
5700	0.948	0.795	0.807	0.826
5705	0.948	0.793	0.814	0.824
5710	0.955	0.819	0.845	0.845
5715	0.955	0.819	0.845	0.845
5720	0.965	0.857	0.910	0.877
5725	0.951	0.803	0.841	0.831
5730	0.951	0.801	0.850	0.829
5735	0.953	0.809	0.852	0.836
5740	0.948	0.791	0.821	0.821
5745	0.964	0.852	0.889	0.873
5750	0.953	0.808	0.861	0.834
5755	0.962	0.845	0.878	0.867
5760	0.953	0.813	0.835	0.840
5765	0.962	0.850	0.852	0.872
5770	0.957	0.827	0.847	0.852
5775	0.950	0.801	0.816	0.830
5780	0.957	0.827	0.847	0.852
5785	0.965	0.864	0.864	0.884

5790	0.958	0.833	0.857	0.857
5795	0.960	0.836	0.886	0.859
5800	0.967	0.866	0.901	0.885
5805	0.953	0.808	0.861	0.834
5810	0.965	0.862	0.872	0.882
5815	0.950	0.795	0.840	0.824
5820	0.955	0.814	0.872	0.840
5825	0.964	0.850	0.909	0.870
5830	0.943	0.771	0.800	0.805
5835	0.955	0.813	0.882	0.838
5840	0.950	0.795	0.840	0.824
5845	0.958	0.831	0.866	0.855
5850	0.948	0.798	0.800	0.828
5855	0.951	0.800	0.859	0.827
5860	0.958	0.830	0.875	0.854
5865	0.955	0.819	0.845	0.845
5870	0.965	0.858	0.900	0.878
5875	0.946	0.785	0.812	0.817
5880	0.955	0.816	0.863	0.841
5885	0.958	0.833	0.857	0.857
5890	0.955	0.819	0.845	0.845
5895	0.953	0.813	0.835	0.840
5900	0.962	0.848	0.860	0.871
5905	0.955	0.816	0.863	0.841
5910	0.951	0.805	0.833	0.833
5915	0.967	0.866	0.901	0.885
5920	0.957	0.827	0.847	0.852
5925	0.953	0.809	0.852	0.836
5930	0.965	0.861	0.881	0.881
5935	0.957	0.827	0.847	0.852
5940	0.960	0.838	0.877	0.861
5945	0.960	0.835	0.896	0.857
5950	0.945	0.775	0.817	0.807
5955	0.955	0.817	0.854	0.843
5960	0.953	0.805	0.880	0.830
5965	0.951	0.800	0.859	0.827
5970	0.958	0.830	0.875	0.854
5975	0.951	0.803	0.841	0.831
5980	0.950	0.792	0.857	0.820
5985	0.953	0.809	0.852	0.836
5990	0.955	0.816	0.863	0.841
5995	0.953	0.811	0.843	0.838
6000	0.950	0.795	0.840	0.824

6005	0.951	0.801	0.850	0.829
6010	0.965	0.859	0.890	0.880
6015	0.958	0.831	0.866	0.855
6020	0.958	0.831	0.866	0.855
6025	0.960	0.838	0.877	0.861
6030	0.953	0.809	0.852	0.836
6035	0.953	0.808	0.861	0.834
6040	0.957	0.823	0.864	0.848
6045	0.953	0.803	0.901	0.826
6050	0.958	0.830	0.875	0.854
6055	0.960	0.841	0.859	0.864
6060	0.953	0.809	0.852	0.836
6065	0.955	0.812	0.892	0.835
6070	0.950	0.799	0.824	0.828
6075	0.960	0.839	0.867	0.862
6080	0.960	0.835	0.896	0.857
6085	0.953	0.809	0.852	0.836
6090	0.957	0.821	0.883	0.845
6095	0.951	0.798	0.868	0.825
6100	0.951	0.803	0.841	0.831
6105	0.955	0.816	0.863	0.841
6110	0.950	0.792	0.857	0.820
6115	0.957	0.825	0.855	0.850
6120	0.955	0.819	0.845	0.845
6125	0.950	0.792	0.857	0.820
6130	0.955	0.814	0.872	0.840
6135	0.953	0.805	0.880	0.830
6140	0.958	0.828	0.885	0.852
6145	0.955	0.816	0.863	0.841
6150	0.962	0.844	0.888	0.866
6155	0.955	0.813	0.882	0.838
6160	0.948	0.787	0.838	0.817
6165	0.957	0.821	0.883	0.845
6170	0.953	0.806	0.870	0.832
6175	0.960	0.839	0.867	0.862
6180	0.958	0.830	0.875	0.854
6185	0.965	0.858	0.900	0.878
6190	0.955	0.817	0.854	0.843
6195	0.945	0.779	0.802	0.812
6200	0.955	0.819	0.845	0.845
6205	0.960	0.838	0.877	0.861
6210	0.955	0.816	0.863	0.841
6215	0.951	0.800	0.859	0.827

6220	0.955	0.816	0.863	0.841
6225	0.953	0.813	0.835	0.840
6230	0.953	0.806	0.870	0.832
6235	0.957	0.823	0.864	0.848
6240	0.953	0.806	0.870	0.832
6245	0.953	0.809	0.852	0.836
6250	0.964	0.849	0.920	0.868
6255	0.964	0.849	0.920	0.868
6260	0.958	0.827	0.895	0.850
6265	0.953	0.808	0.861	0.834
6270	0.953	0.808	0.861	0.834
6275	0.950	0.793	0.848	0.822
6280	0.948	0.781	0.875	0.808
6285	0.953	0.808	0.861	0.834
6290	0.946	0.781	0.827	0.812
6295	0.958	0.830	0.875	0.854
6300	0.960	0.835	0.896	0.857
6305	0.958	0.827	0.895	0.850
6310	0.953	0.808	0.861	0.834
6315	0.962	0.843	0.897	0.864
6320	0.945	0.773	0.825	0.805
6325	0.962	0.844	0.888	0.866
6330	0.958	0.827	0.895	0.850
6335	0.951	0.805	0.833	0.833
6340	0.948	0.787	0.838	0.817
6345	0.960	0.836	0.886	0.859
6350	0.948	0.789	0.829	0.819
6355	0.955	0.814	0.872	0.840
6360	0.955	0.816	0.863	0.841
6365	0.969	0.872	0.913	0.890
6370	0.965	0.856	0.921	0.875
6375	0.953	0.805	0.880	0.830
6380	0.955	0.814	0.872	0.840
6385	0.957	0.821	0.883	0.845
6390	0.950	0.795	0.840	0.824
6395	0.955	0.817	0.854	0.843
6400	0.958	0.830	0.875	0.854
6405	0.957	0.820	0.893	0.843
6410	0.955	0.812	0.892	0.835
6415	0.958	0.828	0.885	0.852
6420	0.958	0.828	0.885	0.852
6425	0.958	0.831	0.866	0.855
6430	0.955	0.810	0.914	0.831

6435	0.955	0.813	0.882	0.838
6440	0.946	0.785	0.812	0.817
6445	0.953	0.808	0.861	0.834
6450	0.950	0.792	0.857	0.820
6455	0.967	0.863	0.945	0.879
6460	0.953	0.809	0.852	0.836
6465	0.955	0.816	0.863	0.841
6470	0.962	0.844	0.888	0.866
6475	0.958	0.828	0.885	0.852
6480	0.950	0.793	0.848	0.822
6485	0.958	0.833	0.857	0.857
6490	0.957	0.821	0.883	0.845
6495	0.964	0.849	0.920	0.868
6500	0.953	0.806	0.870	0.832
6505	0.957	0.819	0.904	0.841
6510	0.958	0.827	0.895	0.850
6515	0.957	0.820	0.893	0.843
6520	0.955	0.813	0.882	0.838
6525	0.964	0.852	0.889	0.873
6530	0.955	0.812	0.892	0.835
6535	0.965	0.862	0.872	0.882
6540	0.951	0.805	0.833	0.833
6545	0.958	0.827	0.895	0.850
6550	0.965	0.861	0.881	0.881
6555	0.943	0.767	0.815	0.800
6560	0.962	0.844	0.888	0.866
6565	0.960	0.836	0.886	0.859
6570	0.955	0.816	0.863	0.841
6575	0.960	0.839	0.867	0.862
6580	0.955	0.814	0.872	0.840
6585	0.971	0.880	0.914	0.897
6590	0.958	0.828	0.885	0.852
6595	0.958	0.830	0.875	0.854
6600	0.960	0.834	0.907	0.855
6605	0.951	0.803	0.841	0.831
6610	0.953	0.809	0.852	0.836
6615	0.960	0.836	0.886	0.859
6620	0.953	0.811	0.843	0.838
6625	0.948	0.791	0.821	0.821
6630	0.955	0.812	0.892	0.835
6635	0.953	0.809	0.852	0.836
6640	0.955	0.816	0.863	0.841
6645	0.951	0.800	0.859	0.827

6650	0.960	0.839	0.867	0.862
6655	0.957	0.823	0.864	0.848
6660	0.957	0.825	0.855	0.850
6665	0.951	0.809	0.818	0.837
6670	0.951	0.801	0.850	0.829
6675	0.953	0.813	0.835	0.840
6680	0.960	0.834	0.907	0.855
6685	0.958	0.831	0.866	0.855
6690	0.962	0.847	0.869	0.869
6695	0.960	0.838	0.877	0.861
6700	0.964	0.851	0.899	0.871
6705	0.951	0.805	0.833	0.833
6710	0.958	0.826	0.917	0.846
6715	0.950	0.790	0.867	0.818
6720	0.958	0.827	0.895	0.850
6725	0.955	0.816	0.863	0.841
6730	0.951	0.807	0.826	0.835
6735	0.951	0.801	0.850	0.829
6740	0.960	0.839	0.867	0.862
6745	0.962	0.843	0.897	0.864
6750	0.962	0.843	0.897	0.864
6755	0.960	0.838	0.877	0.861
6760	0.955	0.821	0.837	0.847
6765	0.962	0.843	0.897	0.864
6770	0.955	0.814	0.872	0.840
6775	0.965	0.859	0.890	0.880
6780	0.960	0.836	0.886	0.859
6785	0.964	0.851	0.899	0.871
6790	0.960	0.835	0.896	0.857
6795	0.958	0.828	0.885	0.852
6800	0.962	0.845	0.878	0.867
6805	0.958	0.827	0.895	0.850
6810	0.964	0.851	0.899	0.871
6815	0.958	0.828	0.885	0.852
6820	0.960	0.838	0.877	0.861
6825	0.951	0.805	0.833	0.833
6830	0.962	0.845	0.878	0.867
6835	0.965	0.858	0.900	0.878
6840	0.965	0.857	0.910	0.877
6845	0.958	0.830	0.875	0.854
6850	0.960	0.839	0.867	0.862
6855	0.967	0.865	0.911	0.883
6860	0.953	0.819	0.813	0.846

6865	0.962	0.844	0.888	0.866
6870	0.965	0.857	0.910	0.877
6875	0.965	0.858	0.900	0.878
6880	0.964	0.851	0.899	0.871
6885	0.945	0.777	0.810	0.810
6890	0.962	0.844	0.888	0.866
6895	0.957	0.823	0.864	0.848
6900	0.955	0.823	0.830	0.849
6905	0.962	0.843	0.897	0.864
6910	0.960	0.838	0.877	0.861
6915	0.967	0.863	0.945	0.879
6920	0.960	0.835	0.896	0.857
6925	0.953	0.811	0.843	0.838
6930	0.950	0.793	0.848	0.822
6935	0.960	0.839	0.867	0.862
6940	0.962	0.845	0.878	0.867
6945	0.960	0.835	0.896	0.857
6950	0.957	0.825	0.855	0.850
6955	0.964	0.853	0.880	0.874
6960	0.969	0.873	0.902	0.892
6965	0.958	0.828	0.885	0.852
6970	0.964	0.853	0.880	0.874
6975	0.965	0.861	0.881	0.881
6980	0.960	0.836	0.886	0.859
6985	0.950	0.797	0.831	0.826
6990	0.960	0.842	0.851	0.865
6995	0.962	0.848	0.860	0.871
7000	0.960	0.838	0.877	0.861
7005	0.953	0.808	0.861	0.834
7010	0.962	0.844	0.888	0.866
7015	0.950	0.793	0.848	0.822
7020	0.971	0.878	0.935	0.894
7025	0.964	0.852	0.889	0.873
7030	0.958	0.830	0.875	0.854
7035	0.958	0.831	0.866	0.855
7040	0.957	0.821	0.883	0.845
7045	0.967	0.865	0.911	0.883
7050	0.955	0.813	0.882	0.838
7055	0.951	0.800	0.859	0.827
7060	0.953	0.805	0.880	0.830
7065	0.964	0.852	0.889	0.873
7070	0.948	0.793	0.814	0.824
7075	0.960	0.841	0.859	0.864

7080	0.955	0.819	0.845	0.845
7085	0.971	0.880	0.914	0.897
7090	0.958	0.831	0.866	0.855
7095	0.962	0.844	0.888	0.866
7100	0.953	0.813	0.835	0.840
7105	0.964	0.851	0.899	0.871
7110	0.960	0.836	0.886	0.859
7115	0.957	0.825	0.855	0.850
7120	0.948	0.787	0.838	0.817
7125	0.964	0.852	0.889	0.873
7130	0.958	0.827	0.895	0.850
7135	0.958	0.831	0.866	0.855
7140	0.964	0.854	0.871	0.876
7145	0.953	0.813	0.835	0.840
7150	0.958	0.831	0.866	0.855
7155	0.955	0.817	0.854	0.843
7160	0.957	0.827	0.847	0.852
7165	0.955	0.816	0.863	0.841
7170	0.955	0.823	0.830	0.849
7175	0.955	0.821	0.837	0.847
7180	0.957	0.822	0.873	0.847
7185	0.960	0.844	0.843	0.867
7190	0.948	0.784	0.855	0.813
7195	0.960	0.841	0.859	0.864
7200	0.967	0.864	0.922	0.882
7205	0.969	0.873	0.902	0.892
7210	0.967	0.866	0.901	0.885
7215	0.955	0.814	0.872	0.840
7220	0.962	0.844	0.888	0.866
7225	0.957	0.823	0.864	0.848
7230	0.953	0.808	0.861	0.834
7235	0.972	0.888	0.915	0.904
7240	0.948	0.785	0.846	0.815
7245	0.960	0.838	0.877	0.861
7250	0.962	0.843	0.897	0.864
7255	0.953	0.811	0.843	0.838
7260	0.958	0.833	0.857	0.857
7265	0.964	0.851	0.899	0.871
7270	0.964	0.854	0.871	0.876
7275	0.962	0.845	0.878	0.867
7280	0.957	0.829	0.839	0.854
7285	0.958	0.830	0.875	0.854
7290	0.965	0.859	0.890	0.880

7295	0.962	0.842	0.908	0.863
7300	0.957	0.829	0.839	0.854
7305	0.958	0.826	0.905	0.848
7310	0.967	0.867	0.892	0.886
7315	0.957	0.822	0.873	0.847
7320	0.957	0.825	0.855	0.850
7325	0.957	0.825	0.855	0.850
7330	0.958	0.831	0.866	0.855
7335	0.964	0.854	0.871	0.876
7340	0.962	0.845	0.878	0.867
7345	0.953	0.811	0.843	0.838
7350	0.957	0.821	0.883	0.845
7355	0.953	0.811	0.843	0.838
7360	0.953	0.811	0.843	0.838
7365	0.962	0.843	0.897	0.864
7370	0.955	0.814	0.872	0.840
7375	0.955	0.814	0.872	0.840
7380	0.948	0.787	0.838	0.817
7385	0.958	0.828	0.885	0.852
7390	0.951	0.803	0.841	0.831
7395	0.948	0.789	0.829	0.819
7400	0.960	0.836	0.886	0.859
7405	0.964	0.852	0.889	0.873
7410	0.950	0.793	0.848	0.822
7415	0.958	0.828	0.885	0.852
7420	0.953	0.804	0.890	0.828
7425	0.958	0.831	0.866	0.855
7430	0.960	0.838	0.877	0.861
7435	0.962	0.844	0.888	0.866
7440	0.969	0.872	0.913	0.890
7445	0.962	0.844	0.888	0.866
7450	0.958	0.828	0.885	0.852
7455	0.960	0.834	0.907	0.855
7460	0.964	0.851	0.899	0.871
7465	0.960	0.838	0.877	0.861
7470	0.955	0.814	0.872	0.840
7475	0.951	0.798	0.868	0.825
7480	0.955	0.816	0.863	0.841
7485	0.946	0.785	0.812	0.817
7490	0.960	0.838	0.877	0.861
7495	0.962	0.844	0.888	0.866
7500	0.964	0.849	0.920	0.868
7505	0.960	0.835	0.896	0.857

7510	0.948	0.789	0.829	0.819
7515	0.953	0.811	0.843	0.838
7520	0.960	0.838	0.877	0.861
7525	0.958	0.830	0.875	0.854
7530	0.953	0.809	0.852	0.836
7535	0.955	0.814	0.872	0.840
7540	0.965	0.859	0.890	0.880
7545	0.957	0.820	0.893	0.843
7550	0.957	0.823	0.864	0.848
7555	0.967	0.866	0.901	0.885
7560	0.955	0.819	0.845	0.845
7565	0.955	0.816	0.863	0.841
7570	0.953	0.808	0.861	0.834
7575	0.962	0.842	0.908	0.863
7580	0.955	0.816	0.863	0.841
7585	0.957	0.823	0.864	0.848
7590	0.957	0.820	0.893	0.843
7595	0.948	0.785	0.846	0.815
7600	0.951	0.800	0.859	0.827
7605	0.950	0.795	0.840	0.824
7610	0.964	0.851	0.899	0.871
7615	0.948	0.787	0.838	0.817
7620	0.957	0.823	0.864	0.848
7625	0.957	0.819	0.904	0.841
7630	0.953	0.805	0.880	0.830
7635	0.964	0.850	0.909	0.870
7640	0.962	0.843	0.897	0.864
7645	0.955	0.816	0.863	0.841
7650	0.955	0.817	0.854	0.843
7655	0.955	0.813	0.882	0.838
7660	0.951	0.800	0.859	0.827
7665	0.960	0.835	0.896	0.857
7670	0.960	0.836	0.886	0.859
7675	0.957	0.821	0.883	0.845
7680	0.953	0.813	0.835	0.840
7685	0.955	0.814	0.872	0.840
7690	0.964	0.853	0.880	0.874
7695	0.951	0.797	0.878	0.823
7700	0.958	0.830	0.875	0.854
7705	0.948	0.785	0.846	0.815
7710	0.962	0.845	0.878	0.867
7715	0.951	0.798	0.868	0.825
7720	0.955	0.814	0.872	0.840

7725	0.953	0.809	0.852	0.836
7730	0.948	0.791	0.821	0.821
7735	0.955	0.814	0.872	0.840
7740	0.960	0.838	0.877	0.861
7745	0.965	0.861	0.881	0.881
7750	0.953	0.806	0.870	0.832
7755	0.957	0.820	0.893	0.843
7760	0.955	0.816	0.863	0.841
7765	0.958	0.828	0.885	0.852
7770	0.960	0.835	0.896	0.857
7775	0.957	0.827	0.847	0.852
7780	0.958	0.830	0.875	0.854
7785	0.955	0.819	0.845	0.845
7790	0.957	0.821	0.883	0.845
7795	0.953	0.808	0.861	0.834
7800	0.958	0.827	0.895	0.850
7805	0.964	0.851	0.899	0.871
7810	0.962	0.844	0.888	0.866
7815	0.951	0.797	0.878	0.823
7820	0.951	0.801	0.850	0.829
7825	0.950	0.793	0.848	0.822
7830	0.948	0.784	0.855	0.813
7835	0.955	0.814	0.872	0.840
7840	0.953	0.808	0.861	0.834
7845	0.951	0.800	0.859	0.827
7850	0.958	0.828	0.885	0.852
7855	0.955	0.814	0.872	0.840
7860	0.962	0.843	0.897	0.864
7865	0.945	0.773	0.825	0.805
7870	0.948	0.787	0.838	0.817
7875	0.962	0.842	0.908	0.863
7880	0.957	0.825	0.855	0.850
7885	0.958	0.826	0.905	0.848
7890	0.951	0.798	0.868	0.825
7895	0.958	0.831	0.866	0.855
7900	0.951	0.798	0.868	0.825
7905	0.953	0.806	0.870	0.832
7910	0.953	0.808	0.861	0.834
7915	0.958	0.831	0.866	0.855
7920	0.951	0.801	0.850	0.829
7925	0.951	0.800	0.859	0.827
7930	0.955	0.813	0.882	0.838
7935	0.955	0.817	0.854	0.843

7940	0.950	0.795	0.840	0.824
7945	0.955	0.819	0.845	0.845
7950	0.953	0.808	0.861	0.834
7955	0.957	0.825	0.855	0.850
7960	0.962	0.844	0.888	0.866
7965	0.950	0.795	0.840	0.824
7970	0.967	0.863	0.933	0.881
7975	0.967	0.864	0.922	0.882
7980	0.971	0.879	0.924	0.896
7985	0.957	0.821	0.883	0.845
7990	0.962	0.842	0.908	0.863
7995	0.958	0.826	0.905	0.848
8000	0.955	0.814	0.872	0.840
8005	0.955	0.817	0.854	0.843
8010	0.958	0.833	0.857	0.857
8015	0.962	0.843	0.897	0.864
8020	0.953	0.813	0.835	0.840
8025	0.962	0.845	0.878	0.867
8030	0.946	0.779	0.835	0.810
8035	0.953	0.809	0.852	0.836
8040	0.955	0.819	0.845	0.845
8045	0.964	0.851	0.899	0.871
8050	0.950	0.793	0.848	0.822
8055	0.958	0.827	0.895	0.850
8060	0.955	0.816	0.863	0.841
8065	0.953	0.806	0.870	0.832
8070	0.960	0.836	0.886	0.859
8075	0.960	0.835	0.896	0.857
8080	0.950	0.801	0.816	0.830
8085	0.955	0.819	0.845	0.845
8090	0.951	0.803	0.841	0.831
8095	0.960	0.836	0.886	0.859
8100	0.948	0.787	0.838	0.817
8105	0.960	0.834	0.907	0.855
8110	0.957	0.822	0.873	0.847
8115	0.965	0.858	0.900	0.878
8120	0.962	0.845	0.878	0.867
8125	0.967	0.864	0.922	0.882
8130	0.964	0.850	0.909	0.870
8135	0.958	0.827	0.895	0.850
8140	0.945	0.773	0.825	0.805
8145	0.955	0.816	0.863	0.841
8150	0.965	0.857	0.910	0.877

8155	0.941	0.771	0.778	0.805
8160	0.948	0.787	0.838	0.817
8165	0.967	0.866	0.901	0.885
8170	0.965	0.859	0.890	0.880
8175	0.953	0.806	0.870	0.832
8180	0.958	0.830	0.875	0.854
8185	0.958	0.830	0.875	0.854
8190	0.953	0.808	0.861	0.834
8195	0.960	0.839	0.867	0.862
8200	0.964	0.852	0.889	0.873
8205	0.960	0.836	0.886	0.859
8210	0.955	0.813	0.882	0.838
8215	0.964	0.850	0.909	0.870
8220	0.964	0.852	0.889	0.873
8225	0.958	0.830	0.875	0.854
8230	0.960	0.836	0.886	0.859
8235	0.951	0.798	0.868	0.825
8240	0.957	0.819	0.904	0.841
8245	0.958	0.830	0.875	0.854
8250	0.950	0.795	0.840	0.824
8255	0.958	0.830	0.875	0.854
8260	0.955	0.814	0.872	0.840
8265	0.951	0.798	0.868	0.825
8270	0.964	0.850	0.909	0.870
8275	0.958	0.830	0.875	0.854
8280	0.957	0.820	0.893	0.843
8285	0.965	0.857	0.910	0.877
8290	0.951	0.800	0.859	0.827
8295	0.953	0.809	0.852	0.836
8300	0.957	0.819	0.904	0.841
8305	0.955	0.816	0.863	0.841
8310	0.962	0.845	0.878	0.867
8315	0.958	0.828	0.885	0.852
8320	0.958	0.836	0.841	0.860
8325	0.964	0.851	0.899	0.871
8330	0.957	0.821	0.883	0.845
8335	0.951	0.803	0.841	0.831
8340	0.958	0.831	0.866	0.855
8345	0.960	0.834	0.907	0.855
8350	0.955	0.812	0.892	0.835
8355	0.955	0.813	0.882	0.838
8360	0.958	0.831	0.866	0.855
8365	0.964	0.850	0.909	0.870

8370	0.957	0.822	0.873	0.847
8375	0.955	0.816	0.863	0.841
8380	0.951	0.797	0.878	0.823
8385	0.955	0.821	0.837	0.847
8390	0.960	0.836	0.886	0.859
8395	0.953	0.813	0.835	0.840
8400	0.957	0.825	0.855	0.850
8405	0.957	0.821	0.883	0.845
8410	0.957	0.825	0.855	0.850
8415	0.960	0.836	0.886	0.859
8420	0.943	0.769	0.807	0.802
8425	0.960	0.838	0.877	0.861
8430	0.953	0.809	0.852	0.836
8435	0.960	0.834	0.907	0.855
8440	0.951	0.800	0.859	0.827
8445	0.958	0.830	0.875	0.854
8450	0.951	0.800	0.859	0.827
8455	0.948	0.787	0.838	0.817
8460	0.951	0.801	0.850	0.829
8465	0.957	0.822	0.873	0.847
8470	0.950	0.793	0.848	0.822
8475	0.958	0.831	0.866	0.855
8480	0.951	0.798	0.868	0.825
8485	0.960	0.833	0.918	0.854
8490	0.958	0.828	0.885	0.852
8495	0.964	0.850	0.909	0.870
8500	0.957	0.821	0.883	0.845
8505	0.953	0.805	0.880	0.830
8510	0.969	0.873	0.902	0.892
8515	0.960	0.835	0.896	0.857
8520	0.948	0.789	0.829	0.819
8525	0.967	0.863	0.933	0.881
8530	0.960	0.834	0.907	0.855
8535	0.957	0.827	0.847	0.852
8540	0.964	0.852	0.889	0.873
8545	0.969	0.872	0.913	0.890
8550	0.962	0.843	0.897	0.864
8555	0.951	0.803	0.841	0.831
8560	0.960	0.838	0.877	0.861
8565	0.943	0.761	0.840	0.792
8570	0.955	0.819	0.845	0.845
8575	0.957	0.822	0.873	0.847
8580	0.969	0.873	0.902	0.892

8585	0.955	0.817	0.854	0.843
8590	0.960	0.838	0.877	0.861
8595	0.958	0.830	0.875	0.854
8600	0.967	0.864	0.922	0.882
8605	0.958	0.831	0.866	0.855
8610	0.957	0.821	0.883	0.845
8615	0.957	0.823	0.864	0.848
8620	0.950	0.797	0.831	0.826
8625	0.958	0.831	0.866	0.855
8630	0.964	0.850	0.909	0.870
8635	0.953	0.813	0.835	0.840
8640	0.965	0.858	0.900	0.878
8645	0.950	0.795	0.840	0.824
8650	0.967	0.863	0.933	0.881
8655	0.962	0.845	0.878	0.867
8660	0.969	0.872	0.913	0.890
8665	0.957	0.822	0.873	0.847
8670	0.960	0.836	0.886	0.859
8675	0.957	0.827	0.847	0.852
8680	0.958	0.828	0.885	0.852
8685	0.962	0.848	0.860	0.871
8690	0.965	0.857	0.910	0.877
8695	0.951	0.798	0.868	0.825
8700	0.964	0.852	0.889	0.873
8705	0.950	0.795	0.840	0.824
8710	0.958	0.828	0.885	0.852
8715	0.969	0.871	0.934	0.888
8720	0.967	0.864	0.922	0.882
8725	0.953	0.815	0.828	0.842
8730	0.962	0.840	0.931	0.859
8735	0.960	0.838	0.877	0.861
8740	0.965	0.857	0.910	0.877
8745	0.955	0.814	0.872	0.840
8750	0.960	0.838	0.877	0.861
8755	0.962	0.844	0.888	0.866
8760	0.964	0.852	0.889	0.873
8765	0.967	0.865	0.911	0.883
8770	0.960	0.838	0.877	0.861
8775	0.965	0.862	0.872	0.882
8780	0.948	0.793	0.814	0.824
8785	0.964	0.850	0.909	0.870
8790	0.955	0.817	0.854	0.843
8795	0.958	0.828	0.885	0.852

8800	0.957	0.822	0.873	0.847
8805	0.955	0.817	0.854	0.843
8810	0.960	0.835	0.896	0.857
8815	0.960	0.838	0.877	0.861
8820	0.960	0.839	0.867	0.862
8825	0.960	0.839	0.867	0.862
8830	0.962	0.847	0.869	0.869
8835	0.955	0.819	0.845	0.845
8840	0.955	0.812	0.892	0.835
8845	0.960	0.841	0.859	0.864
8850	0.957	0.820	0.893	0.843
8855	0.965	0.857	0.910	0.877
8860	0.962	0.844	0.888	0.866
8865	0.965	0.858	0.900	0.878
8870	0.958	0.831	0.866	0.855
8875	0.955	0.812	0.892	0.835
8880	0.964	0.852	0.889	0.873
8885	0.960	0.836	0.886	0.859
8890	0.964	0.850	0.909	0.870
8895	0.953	0.808	0.861	0.834
8900	0.957	0.823	0.864	0.848
8905	0.964	0.852	0.889	0.873
8910	0.964	0.850	0.909	0.870
8915	0.964	0.851	0.899	0.871
8920	0.960	0.839	0.867	0.862
8925	0.967	0.865	0.911	0.883
8930	0.964	0.852	0.889	0.873
8935	0.957	0.822	0.873	0.847
8940	0.964	0.852	0.889	0.873
8945	0.958	0.828	0.885	0.852
8950	0.955	0.813	0.882	0.838
8955	0.960	0.836	0.886	0.859
8960	0.957	0.822	0.873	0.847
8965	0.958	0.831	0.866	0.855
8970	0.958	0.833	0.857	0.857
8975	0.957	0.821	0.883	0.845
8980	0.955	0.816	0.863	0.841
8985	0.960	0.838	0.877	0.861
8990	0.965	0.858	0.900	0.878
8995	0.965	0.857	0.910	0.877
9000	0.946	0.781	0.827	0.812
9005	0.958	0.828	0.885	0.852
9010	0.962	0.843	0.897	0.864

9015	0.957	0.823	0.864	0.848
9020	0.955	0.814	0.872	0.840
9025	0.953	0.808	0.861	0.834
9030	0.948	0.789	0.829	0.819
9035	0.951	0.800	0.859	0.827
9040	0.951	0.801	0.850	0.829
9045	0.953	0.806	0.870	0.832
9050	0.958	0.833	0.857	0.857
9055	0.960	0.838	0.877	0.861
9060	0.958	0.828	0.885	0.852
9065	0.958	0.828	0.885	0.852
9070	0.950	0.797	0.831	0.826
9075	0.958	0.826	0.905	0.848
9080	0.962	0.841	0.919	0.861
9085	0.955	0.813	0.882	0.838
9090	0.958	0.830	0.875	0.854
9095	0.960	0.836	0.886	0.859
9100	0.946	0.783	0.819	0.814
9105	0.951	0.803	0.841	0.831
9110	0.950	0.797	0.831	0.826
9115	0.958	0.826	0.905	0.848
9120	0.964	0.851	0.899	0.871
9125	0.953	0.806	0.870	0.832
9130	0.958	0.828	0.885	0.852
9135	0.953	0.806	0.870	0.832
9140	0.958	0.828	0.885	0.852
9145	0.953	0.803	0.901	0.826
9150	0.955	0.816	0.863	0.841
9155	0.960	0.836	0.886	0.859
9160	0.958	0.828	0.885	0.852
9165	0.962	0.843	0.897	0.864
9170	0.957	0.821	0.883	0.845
9175	0.958	0.826	0.905	0.848
9180	0.958	0.828	0.885	0.852
9185	0.962	0.844	0.888	0.866
9190	0.958	0.831	0.866	0.855
9195	0.962	0.844	0.888	0.866
9200	0.955	0.816	0.863	0.841
9205	0.955	0.816	0.863	0.841
9210	0.957	0.822	0.873	0.847
9215	0.953	0.809	0.852	0.836
9220	0.964	0.848	0.932	0.866
9225	0.964	0.850	0.909	0.870

9230	0.962	0.844	0.888	0.866
9235	0.951	0.800	0.859	0.827
9240	0.962	0.845	0.878	0.867
9245	0.958	0.827	0.895	0.850
9250	0.953	0.805	0.880	0.830
9255	0.950	0.795	0.840	0.824
9260	0.955	0.816	0.863	0.841
9265	0.957	0.822	0.873	0.847
9270	0.960	0.836	0.886	0.859
9275	0.957	0.821	0.883	0.845
9280	0.950	0.795	0.840	0.824
9285	0.953	0.806	0.870	0.832
9290	0.951	0.801	0.850	0.829
9295	0.955	0.819	0.845	0.845
9300	0.960	0.835	0.896	0.857
9305	0.955	0.813	0.882	0.838
9310	0.955	0.814	0.872	0.840
9315	0.950	0.792	0.857	0.820
9320	0.953	0.809	0.852	0.836
9325	0.950	0.792	0.857	0.820
9330	0.955	0.813	0.882	0.838
9335	0.960	0.838	0.877	0.861
9340	0.957	0.821	0.883	0.845
9345	0.958	0.828	0.885	0.852
9350	0.955	0.819	0.845	0.845
9355	0.951	0.803	0.841	0.831
9360	0.955	0.813	0.882	0.838
9365	0.951	0.798	0.868	0.825
9370	0.948	0.785	0.846	0.815
9375	0.950	0.793	0.848	0.822
9380	0.951	0.800	0.859	0.827
9385	0.953	0.811	0.843	0.838
9390	0.958	0.828	0.885	0.852
9395	0.967	0.864	0.922	0.882
9400	0.951	0.798	0.868	0.825
9405	0.950	0.793	0.848	0.822
9410	0.962	0.842	0.908	0.863
9415	0.960	0.836	0.886	0.859
9420	0.964	0.850	0.909	0.870
9425	0.951	0.798	0.868	0.825
9430	0.953	0.808	0.861	0.834
9435	0.955	0.816	0.863	0.841
9440	0.957	0.825	0.855	0.850

9445	0.957	0.821	0.883	0.845
9450	0.960	0.835	0.896	0.857
9455	0.958	0.828	0.885	0.852
9460	0.960	0.834	0.907	0.855
9465	0.960	0.836	0.886	0.859
9470	0.958	0.828	0.885	0.852
9475	0.953	0.806	0.870	0.832
9480	0.951	0.800	0.859	0.827
9485	0.955	0.816	0.863	0.841
9490	0.946	0.776	0.853	0.805
9495	0.958	0.826	0.917	0.846
9500	0.948	0.785	0.846	0.815
9505	0.953	0.808	0.861	0.834
9510	0.962	0.840	0.931	0.859
9515	0.962	0.843	0.897	0.864
9520	0.953	0.806	0.870	0.832
9525	0.955	0.816	0.863	0.841
9530	0.957	0.822	0.873	0.847
9535	0.950	0.792	0.857	0.820
9540	0.948	0.781	0.875	0.808
9545	0.958	0.826	0.917	0.846
9550	0.957	0.820	0.893	0.843
9555	0.960	0.835	0.896	0.857
9560	0.951	0.797	0.878	0.823
9565	0.953	0.808	0.861	0.834
9570	0.951	0.800	0.859	0.827
9575	0.965	0.856	0.921	0.875
9580	0.948	0.787	0.838	0.817
9585	0.957	0.822	0.873	0.847
9590	0.957	0.823	0.864	0.848
9595	0.960	0.836	0.886	0.859
9600	0.951	0.798	0.868	0.825
9605	0.948	0.789	0.829	0.819
9610	0.958	0.828	0.885	0.852
9615	0.958	0.830	0.875	0.854
9620	0.953	0.811	0.843	0.838
9625	0.953	0.806	0.870	0.832
9630	0.950	0.795	0.840	0.824
9635	0.960	0.834	0.907	0.855
9640	0.960	0.835	0.896	0.857
9645	0.953	0.805	0.880	0.830
9650	0.958	0.827	0.895	0.850
9655	0.955	0.814	0.872	0.840

9660	0.953	0.804	0.890	0.828
9665	0.960	0.836	0.886	0.859
9670	0.957	0.821	0.883	0.845
9675	0.964	0.850	0.909	0.870
9680	0.960	0.835	0.896	0.857
9685	0.957	0.819	0.904	0.841
9690	0.953	0.808	0.861	0.834
9695	0.951	0.798	0.868	0.825
9700	0.953	0.806	0.870	0.832
9705	0.960	0.834	0.907	0.855
9710	0.960	0.833	0.918	0.854
9715	0.957	0.821	0.883	0.845
9720	0.951	0.801	0.850	0.829
9725	0.955	0.810	0.914	0.831
9730	0.951	0.798	0.868	0.825
9735	0.957	0.819	0.904	0.841
9740	0.960	0.836	0.886	0.859
9745	0.962	0.843	0.897	0.864
9750	0.957	0.819	0.904	0.841
9755	0.955	0.811	0.903	0.833
9760	0.951	0.801	0.850	0.829
9765	0.955	0.816	0.863	0.841
9770	0.955	0.811	0.903	0.833
9775	0.962	0.845	0.878	0.867
9780	0.950	0.792	0.857	0.820
9785	0.953	0.805	0.880	0.830
9790	0.958	0.830	0.875	0.854
9795	0.965	0.857	0.910	0.877
9800	0.953	0.804	0.890	0.828
9805	0.953	0.806	0.870	0.832
9810	0.953	0.805	0.880	0.830
9815	0.945	0.765	0.871	0.792
9820	0.953	0.806	0.870	0.832
9825	0.946	0.776	0.853	0.805
9830	0.951	0.803	0.841	0.831
9835	0.953	0.806	0.870	0.832
9840	0.955	0.813	0.882	0.838
9845	0.953	0.805	0.880	0.830
9850	0.958	0.828	0.885	0.852
9855	0.948	0.781	0.875	0.808
9860	0.958	0.826	0.905	0.848
9865	0.957	0.822	0.873	0.847
9870	0.957	0.822	0.873	0.847

9875	0.943	0.755	0.881	0.781
9880	0.955	0.813	0.882	0.838
9885	0.951	0.801	0.850	0.829
9890	0.950	0.792	0.857	0.820
9895	0.958	0.826	0.905	0.848
9900	0.945	0.773	0.825	0.805
9905	0.958	0.826	0.917	0.846
9910	0.957	0.825	0.855	0.850
9915	0.957	0.819	0.904	0.841
9920	0.951	0.796	0.889	0.821
9925	0.946	0.774	0.863	0.803
9930	0.941	0.755	0.829	0.788
9935	0.958	0.827	0.895	0.850
9940	0.948	0.789	0.829	0.819
9945	0.962	0.842	0.908	0.863
9950	0.951	0.795	0.900	0.818
9955	0.955	0.814	0.872	0.840
9960	0.957	0.821	0.883	0.845
9965	0.953	0.805	0.880	0.830
9970	0.950	0.790	0.867	0.818
9975	0.948	0.784	0.855	0.813
9980	0.948	0.781	0.875	0.808
9985	0.951	0.796	0.889	0.821
9990	0.953	0.806	0.870	0.832
9995	0.957	0.820	0.893	0.843
10000	0.946	0.774	0.863	0.803

(2) Performance of IFS with decision tree on the list yielded by LASSO

**Number of features**	**ACC**	**MCC**	**Precision**	**F1-measure**
5	0.756	0.259	0.303	0.384
10	0.776	0.347	0.351	0.451
15	0.764	0.337	0.338	0.443
20	0.797	0.389	0.385	0.485
25	0.787	0.412	0.379	0.498
30	0.787	0.399	0.376	0.490
35	0.776	0.379	0.360	0.473
40	0.776	0.353	0.353	0.456
45	0.806	0.445	0.408	0.525
50	0.797	0.401	0.388	0.494
55	0.787	0.361	0.366	0.463
60	0.771	0.359	0.350	0.459
65	0.789	0.345	0.362	0.450
70	0.797	0.457	0.400	0.530

75	0.801	0.413	0.395	0.502
80	0.787	0.361	0.366	0.463
85	0.797	0.370	0.380	0.471
90	0.794	0.403	0.384	0.494
95	0.795	0.411	0.388	0.500
100	0.804	0.424	0.401	0.511
105	0.757	0.335	0.331	0.440
110	0.775	0.370	0.356	0.467
115	0.764	0.298	0.324	0.414
120	0.778	0.375	0.361	0.471
125	0.799	0.435	0.397	0.517
130	0.783	0.394	0.371	0.486
135	0.790	0.404	0.381	0.494
140	0.783	0.369	0.364	0.468
145	0.768	0.342	0.342	0.446
150	0.794	0.409	0.386	0.498
155	0.787	0.355	0.364	0.458
160	0.794	0.384	0.379	0.480
165	0.802	0.421	0.399	0.509
170	0.752	0.315	0.321	0.426
175	0.778	0.323	0.345	0.434
180	0.794	0.427	0.390	0.510
185	0.771	0.327	0.340	0.436
190	0.794	0.390	0.381	0.485
195	0.769	0.344	0.344	0.448
200	0.789	0.414	0.381	0.500
205	0.761	0.352	0.339	0.452
210	0.790	0.373	0.372	0.472
215	0.794	0.458	0.396	0.530
220	0.789	0.402	0.378	0.492
225	0.809	0.438	0.411	0.522
230	0.782	0.322	0.348	0.432
235	0.816	0.443	0.421	0.527
240	0.780	0.390	0.366	0.482
245	0.809	0.432	0.410	0.518
250	0.789	0.420	0.383	0.504
255	0.771	0.327	0.340	0.436
260	0.775	0.395	0.363	0.484
265	0.766	0.320	0.333	0.430
270	0.771	0.391	0.359	0.480
275	0.759	0.298	0.320	0.414
280	0.773	0.368	0.354	0.465
285	0.754	0.264	0.304	0.388

290	0.787	0.380	0.371	0.477
295	0.775	0.357	0.353	0.458
300	0.806	0.396	0.397	0.491
305	0.775	0.331	0.345	0.440
310	0.756	0.306	0.321	0.420
315	0.799	0.435	0.397	0.517
320	0.766	0.378	0.351	0.471
325	0.792	0.381	0.377	0.478
330	0.768	0.302	0.329	0.417
335	0.771	0.300	0.331	0.416
340	0.789	0.357	0.366	0.460
345	0.776	0.294	0.333	0.411
350	0.766	0.346	0.342	0.449
355	0.780	0.299	0.338	0.415
360	0.782	0.341	0.354	0.447
365	0.759	0.257	0.305	0.382
370	0.785	0.372	0.367	0.470
375	0.795	0.393	0.384	0.487
380	0.783	0.375	0.366	0.473
385	0.797	0.383	0.383	0.480
390	0.821	0.433	0.427	0.521
395	0.783	0.356	0.361	0.459
400	0.794	0.359	0.372	0.462
405	0.823	0.442	0.432	0.528
410	0.795	0.342	0.369	0.449
415	0.816	0.466	0.426	0.543
420	0.799	0.385	0.386	0.482
425	0.809	0.401	0.403	0.495
430	0.809	0.358	0.392	0.461
435	0.780	0.377	0.363	0.473
440	0.776	0.379	0.360	0.473
445	0.783	0.344	0.357	0.449
450	0.802	0.353	0.381	0.457
455	0.799	0.366	0.381	0.468
460	0.766	0.346	0.342	0.449
465	0.804	0.412	0.399	0.502
470	0.778	0.343	0.351	0.448
475	0.768	0.374	0.351	0.468
480	0.811	0.441	0.414	0.524
485	0.815	0.416	0.414	0.507
490	0.769	0.324	0.338	0.434
495	0.789	0.383	0.373	0.479
500	0.789	0.402	0.378	0.492

505	0.792	0.375	0.375	0.474
510	0.773	0.329	0.342	0.438
515	0.768	0.367	0.349	0.464
520	0.818	0.415	0.419	0.507
525	0.771	0.340	0.344	0.445
530	0.783	0.375	0.366	0.473
535	0.787	0.374	0.369	0.472
540	0.771	0.372	0.354	0.468
545	0.773	0.361	0.352	0.461
550	0.794	0.403	0.384	0.494
555	0.811	0.441	0.414	0.524
560	0.785	0.390	0.372	0.483
565	0.789	0.376	0.372	0.474
570	0.792	0.362	0.371	0.464
575	0.764	0.298	0.324	0.414
580	0.783	0.394	0.371	0.486
585	0.801	0.407	0.393	0.498
590	0.730	0.213	0.269	0.350
595	0.773	0.374	0.356	0.470
600	0.794	0.409	0.386	0.498
605	0.783	0.394	0.371	0.486
610	0.804	0.412	0.399	0.502
615	0.782	0.354	0.358	0.457
620	0.801	0.425	0.397	0.511
625	0.802	0.434	0.401	0.517
630	0.799	0.385	0.386	0.482
635	0.792	0.362	0.371	0.464
640	0.768	0.329	0.338	0.437
645	0.787	0.355	0.364	0.458
650	0.801	0.431	0.399	0.515
655	0.785	0.378	0.368	0.475
660	0.792	0.375	0.375	0.474
665	0.787	0.361	0.366	0.463
670	0.782	0.354	0.358	0.457
675	0.797	0.383	0.383	0.480
680	0.752	0.315	0.321	0.426
685	0.795	0.405	0.387	0.496
690	0.808	0.441	0.409	0.524
695	0.787	0.387	0.373	0.481
700	0.776	0.327	0.345	0.437
705	0.790	0.360	0.369	0.462
710	0.802	0.421	0.399	0.509
715	0.761	0.293	0.320	0.410

720	0.789	0.351	0.364	0.455
725	0.802	0.403	0.394	0.496
730	0.799	0.373	0.382	0.473
735	0.821	0.445	0.430	0.530
740	0.797	0.389	0.385	0.485
745	0.818	0.445	0.424	0.529
750	0.801	0.382	0.387	0.480
755	0.768	0.316	0.333	0.427
760	0.783	0.324	0.350	0.434
765	0.780	0.345	0.354	0.450
770	0.809	0.395	0.402	0.491
775	0.785	0.378	0.368	0.475
780	0.795	0.380	0.380	0.478
785	0.811	0.416	0.409	0.507
790	0.801	0.350	0.378	0.455
795	0.794	0.390	0.381	0.485
800	0.839	0.469	0.463	0.551
805	0.785	0.320	0.351	0.431
810	0.780	0.352	0.356	0.455
815	0.792	0.400	0.382	0.492
820	0.761	0.346	0.337	0.448
825	0.795	0.399	0.385	0.491
830	0.802	0.421	0.399	0.509
835	0.795	0.368	0.377	0.468
840	0.790	0.354	0.367	0.457
845	0.792	0.388	0.378	0.483
850	0.804	0.412	0.399	0.502
855	0.797	0.364	0.378	0.466
860	0.785	0.365	0.365	0.466
865	0.802	0.409	0.396	0.500
870	0.787	0.322	0.353	0.433
875	0.763	0.309	0.327	0.422
880	0.778	0.323	0.345	0.434
885	0.738	0.210	0.272	0.346
890	0.778	0.310	0.341	0.423
895	0.754	0.291	0.314	0.408
900	0.756	0.273	0.309	0.395
905	0.724	0.235	0.275	0.367
910	0.733	0.211	0.270	0.347
915	0.776	0.347	0.351	0.451
920	0.787	0.342	0.360	0.448
925	0.776	0.327	0.345	0.437
930	0.761	0.326	0.331	0.434

935	0.799	0.366	0.381	0.468
940	0.763	0.268	0.312	0.391
945	0.747	0.255	0.296	0.381
950	0.757	0.282	0.313	0.402
955	0.787	0.342	0.360	0.448
960	0.757	0.227	0.291	0.358
965	0.785	0.346	0.359	0.451
970	0.776	0.301	0.336	0.416
975	0.768	0.255	0.311	0.380
980	0.771	0.307	0.333	0.421
985	0.792	0.369	0.373	0.469
990	0.776	0.281	0.328	0.400
995	0.785	0.333	0.355	0.441
1000	0.806	0.306	0.370	0.417
1005	0.761	0.259	0.307	0.384
1010	0.799	0.354	0.377	0.458
1015	0.756	0.259	0.303	0.384
1020	0.749	0.230	0.287	0.361
1025	0.787	0.322	0.353	0.433
1030	0.790	0.366	0.371	0.467
1035	0.783	0.331	0.353	0.439
1040	0.773	0.303	0.333	0.418
1045	0.780	0.390	0.366	0.482
1050	0.795	0.393	0.384	0.487
1055	0.771	0.307	0.333	0.421
1060	0.766	0.307	0.329	0.421
1065	0.778	0.343	0.351	0.448
1070	0.769	0.305	0.331	0.419
1075	0.813	0.413	0.410	0.505
1080	0.789	0.312	0.352	0.425
1085	0.778	0.323	0.345	0.434
1090	0.785	0.327	0.353	0.436
1095	0.789	0.370	0.370	0.470
1100	0.778	0.368	0.359	0.467
1105	0.764	0.291	0.322	0.409
1110	0.792	0.356	0.370	0.459
1115	0.775	0.325	0.342	0.435
1120	0.804	0.387	0.393	0.484
1125	0.775	0.305	0.336	0.420
1130	0.785	0.359	0.363	0.461
1135	0.775	0.331	0.345	0.440
1140	0.757	0.282	0.313	0.402
1145	0.780	0.332	0.350	0.441

1150	0.744	0.230	0.284	0.362
1155	0.827	0.424	0.434	0.515
1160	0.766	0.307	0.329	0.421
1165	0.766	0.384	0.353	0.475
1170	0.780	0.279	0.331	0.398
1175	0.801	0.350	0.378	0.455
1180	0.804	0.387	0.393	0.484
1185	0.790	0.360	0.369	0.462
1190	0.802	0.384	0.390	0.482
1195	0.789	0.357	0.366	0.460
1200	0.776	0.301	0.336	0.416
1205	0.761	0.300	0.322	0.415
1210	0.785	0.320	0.351	0.431
1215	0.771	0.280	0.324	0.400
1220	0.794	0.326	0.362	0.436
1225	0.794	0.371	0.376	0.471
1230	0.780	0.358	0.358	0.460
1235	0.780	0.326	0.348	0.436
1240	0.773	0.316	0.338	0.428
1245	0.801	0.388	0.388	0.484
1250	0.792	0.350	0.368	0.455
1255	0.761	0.300	0.322	0.415
1260	0.769	0.318	0.336	0.429
1265	0.773	0.303	0.333	0.418
1270	0.799	0.341	0.373	0.448
1275	0.761	0.238	0.299	0.367
1280	0.789	0.325	0.356	0.435
1285	0.794	0.326	0.362	0.436
1290	0.801	0.375	0.385	0.475
1295	0.775	0.331	0.345	0.440
1300	0.790	0.314	0.354	0.427
1305	0.790	0.379	0.374	0.476
1310	0.780	0.306	0.341	0.420
1315	0.776	0.308	0.338	0.422
1320	0.778	0.297	0.336	0.413
1325	0.785	0.340	0.357	0.446
1330	0.778	0.297	0.336	0.413
1335	0.782	0.288	0.336	0.406
1340	0.744	0.237	0.287	0.368
1345	0.778	0.330	0.347	0.439
1350	0.787	0.348	0.362	0.453
1355	0.756	0.232	0.292	0.362
1360	0.742	0.228	0.282	0.361

1365	0.787	0.348	0.362	0.453
1370	0.771	0.307	0.333	0.421
1375	0.789	0.332	0.358	0.440
1380	0.775	0.351	0.351	0.454
1385	0.778	0.356	0.355	0.458
1390	0.795	0.393	0.384	0.487
1395	0.801	0.369	0.383	0.470
1400	0.778	0.336	0.349	0.443
1405	0.804	0.368	0.388	0.469
1410	0.764	0.311	0.329	0.424
1415	0.768	0.275	0.319	0.396
1420	0.764	0.298	0.324	0.414
1425	0.782	0.281	0.333	0.400
1430	0.750	0.246	0.295	0.374
1435	0.775	0.285	0.328	0.404
1440	0.745	0.280	0.304	0.400
1445	0.754	0.229	0.290	0.360
1450	0.766	0.293	0.324	0.410
1455	0.766	0.307	0.329	0.421
1460	0.766	0.280	0.319	0.400
1465	0.778	0.310	0.341	0.423
1470	0.749	0.257	0.298	0.383
1475	0.783	0.324	0.350	0.434
1480	0.776	0.321	0.343	0.432
1485	0.778	0.303	0.338	0.418
1490	0.780	0.279	0.331	0.398
1495	0.744	0.305	0.312	0.417
1500	0.763	0.255	0.307	0.380
1505	0.780	0.306	0.341	0.420
1510	0.766	0.280	0.319	0.400
1515	0.761	0.293	0.320	0.410
1520	0.771	0.327	0.340	0.436
1525	0.768	0.309	0.331	0.422
1530	0.782	0.295	0.338	0.411
1535	0.780	0.312	0.343	0.425
1540	0.738	0.245	0.287	0.373
1545	0.802	0.384	0.390	0.482
1550	0.790	0.328	0.359	0.437
1555	0.802	0.378	0.388	0.477
1560	0.783	0.304	0.344	0.419
1565	0.802	0.366	0.385	0.467
1570	0.776	0.308	0.338	0.422
1575	0.785	0.273	0.333	0.392

1580	0.756	0.273	0.309	0.395
1585	0.766	0.313	0.331	0.426
1590	0.790	0.360	0.369	0.462
1595	0.775	0.351	0.351	0.454
1600	0.757	0.282	0.313	0.402
1605	0.792	0.388	0.378	0.483
1610	0.795	0.361	0.375	0.464
1615	0.745	0.253	0.294	0.380
1620	0.802	0.372	0.386	0.472
1625	0.775	0.325	0.342	0.435
1630	0.764	0.318	0.331	0.429
1635	0.759	0.298	0.320	0.414
1640	0.744	0.251	0.292	0.378
1645	0.778	0.283	0.331	0.402
1650	0.809	0.377	0.397	0.476
1655	0.795	0.348	0.371	0.454
1660	0.778	0.375	0.361	0.471
1665	0.780	0.319	0.345	0.430
1670	0.773	0.303	0.333	0.418
1675	0.804	0.424	0.401	0.511
1680	0.756	0.313	0.323	0.424
1685	0.776	0.334	0.347	0.442
1690	0.763	0.322	0.331	0.432
1695	0.757	0.262	0.306	0.386
1700	0.773	0.349	0.348	0.452
1705	0.794	0.359	0.372	0.462
1710	0.790	0.328	0.359	0.437
1715	0.750	0.266	0.303	0.390
1720	0.756	0.286	0.314	0.405
1725	0.813	0.425	0.413	0.514
1730	0.783	0.350	0.359	0.454
1735	0.764	0.304	0.327	0.419
1740	0.775	0.331	0.345	0.440
1745	0.761	0.273	0.313	0.395
1750	0.771	0.314	0.336	0.426
1755	0.783	0.324	0.350	0.434
1760	0.752	0.315	0.321	0.426
1765	0.764	0.243	0.303	0.370
1770	0.757	0.241	0.297	0.369
1775	0.742	0.207	0.273	0.344
1780	0.790	0.379	0.374	0.476
1785	0.780	0.345	0.354	0.450
1790	0.789	0.345	0.362	0.450

1795	0.769	0.331	0.340	0.439
1800	0.799	0.435	0.397	0.517
1805	0.733	0.252	0.287	0.379
1810	0.768	0.275	0.319	0.396
1815	0.783	0.317	0.348	0.429
1820	0.797	0.408	0.389	0.498
1825	0.787	0.348	0.362	0.453
1830	0.782	0.328	0.350	0.438
1835	0.783	0.331	0.353	0.439
1840	0.769	0.318	0.336	0.429
1845	0.792	0.330	0.362	0.439
1850	0.773	0.269	0.321	0.391
1855	0.776	0.260	0.320	0.383
1860	0.794	0.384	0.379	0.480
1865	0.763	0.295	0.322	0.412
1870	0.801	0.394	0.390	0.489
1875	0.816	0.431	0.419	0.518
1880	0.769	0.324	0.338	0.434
1885	0.797	0.383	0.383	0.480
1890	0.801	0.369	0.383	0.470
1895	0.792	0.375	0.375	0.474
1900	0.787	0.361	0.366	0.463
1905	0.778	0.290	0.333	0.407
1910	0.773	0.349	0.348	0.452
1915	0.776	0.314	0.340	0.427
1920	0.789	0.338	0.360	0.445
1925	0.782	0.367	0.362	0.466
1930	0.801	0.388	0.388	0.484
1935	0.789	0.332	0.358	0.440
1940	0.789	0.332	0.358	0.440
1945	0.789	0.414	0.381	0.500
1950	0.757	0.248	0.300	0.375
1955	0.787	0.368	0.367	0.468
1960	0.749	0.284	0.308	0.403
1965	0.769	0.291	0.326	0.409
1970	0.750	0.286	0.310	0.405
1975	0.778	0.317	0.343	0.429
1980	0.787	0.336	0.358	0.443
1985	0.787	0.342	0.360	0.448
1990	0.756	0.313	0.323	0.424
1995	0.740	0.247	0.288	0.375
2000	0.782	0.322	0.348	0.432
2005	0.737	0.256	0.290	0.382

2010	0.759	0.298	0.320	0.414
2015	0.769	0.337	0.342	0.444
2020	0.763	0.268	0.312	0.391
2025	0.794	0.346	0.368	0.452
2030	0.783	0.324	0.350	0.434
2035	0.782	0.348	0.356	0.452
2040	0.780	0.332	0.350	0.441
2045	0.771	0.300	0.331	0.416
2050	0.740	0.274	0.299	0.395
2055	0.804	0.406	0.397	0.498
2060	0.761	0.313	0.327	0.425
2065	0.780	0.364	0.359	0.464
2070	0.769	0.337	0.342	0.444
2075	0.757	0.262	0.306	0.386
2080	0.766	0.313	0.331	0.426
2085	0.757	0.275	0.311	0.397
2090	0.787	0.418	0.380	0.502
2095	0.766	0.320	0.333	0.430
2100	0.783	0.419	0.377	0.502
2105	0.771	0.320	0.338	0.431
2110	0.785	0.333	0.355	0.441
2115	0.782	0.308	0.343	0.422
2120	0.761	0.293	0.320	0.410
2125	0.773	0.303	0.333	0.418
2130	0.764	0.344	0.340	0.447
2135	0.794	0.333	0.364	0.441
2140	0.794	0.415	0.387	0.502
2145	0.782	0.308	0.343	0.422
2150	0.785	0.378	0.368	0.475
2155	0.790	0.341	0.363	0.447
2160	0.763	0.315	0.329	0.427
2165	0.778	0.336	0.349	0.443
2170	0.789	0.420	0.383	0.504
2175	0.782	0.354	0.358	0.457
2180	0.776	0.340	0.349	0.446
2185	0.778	0.362	0.357	0.462
2190	0.744	0.264	0.297	0.388
2195	0.776	0.308	0.338	0.422
2200	0.735	0.254	0.288	0.381
2205	0.773	0.323	0.340	0.433
2210	0.778	0.297	0.336	0.413
2215	0.737	0.283	0.300	0.402
2220	0.778	0.375	0.361	0.471

2225	0.787	0.336	0.358	0.443
2230	0.756	0.252	0.301	0.379
2235	0.775	0.298	0.333	0.414
2240	0.782	0.335	0.352	0.442
2245	0.776	0.334	0.347	0.442
2250	0.764	0.331	0.335	0.438
2255	0.782	0.295	0.338	0.411
2260	0.757	0.309	0.323	0.421
2265	0.778	0.356	0.355	0.458
2270	0.756	0.273	0.309	0.395
2275	0.752	0.302	0.317	0.416
2280	0.769	0.305	0.331	0.419
2285	0.757	0.262	0.306	0.386
2290	0.773	0.355	0.350	0.456
2295	0.775	0.331	0.345	0.440
2300	0.766	0.300	0.327	0.416
2305	0.801	0.363	0.382	0.465
2310	0.764	0.311	0.329	0.424
2315	0.780	0.377	0.363	0.473
2320	0.757	0.302	0.321	0.417
2325	0.771	0.294	0.329	0.411
2330	0.737	0.222	0.276	0.356
2335	0.768	0.354	0.346	0.455
2340	0.789	0.389	0.375	0.483
2345	0.794	0.371	0.376	0.471
2350	0.752	0.289	0.312	0.407
2355	0.792	0.388	0.378	0.483
2360	0.782	0.341	0.354	0.447
2365	0.780	0.358	0.358	0.460
2370	0.757	0.302	0.321	0.417
2375	0.782	0.328	0.350	0.438
2380	0.763	0.335	0.335	0.441
2385	0.771	0.327	0.340	0.436
2390	0.771	0.359	0.350	0.459
2395	0.776	0.360	0.355	0.460
2400	0.749	0.257	0.298	0.383
2405	0.769	0.324	0.338	0.434
2410	0.764	0.344	0.340	0.447
2415	0.766	0.339	0.340	0.444
2420	0.799	0.366	0.381	0.468
2425	0.752	0.255	0.299	0.381
2430	0.818	0.469	0.429	0.545
2435	0.738	0.298	0.306	0.412

2440	0.764	0.271	0.314	0.393
2445	0.782	0.373	0.364	0.471
2450	0.769	0.370	0.352	0.466
2455	0.738	0.292	0.304	0.408
2460	0.766	0.313	0.331	0.426
2465	0.737	0.242	0.285	0.372
2470	0.768	0.335	0.340	0.442
2475	0.742	0.276	0.301	0.397
2480	0.742	0.276	0.301	0.397
2485	0.789	0.376	0.372	0.474
2490	0.750	0.260	0.300	0.385
2495	0.789	0.370	0.370	0.470
2500	0.764	0.284	0.319	0.404
2505	0.740	0.280	0.301	0.400
2510	0.754	0.271	0.307	0.393
2515	0.775	0.351	0.351	0.454
2520	0.785	0.365	0.365	0.466
2525	0.790	0.347	0.365	0.452
2530	0.769	0.291	0.326	0.409
2535	0.747	0.234	0.288	0.365
2540	0.764	0.331	0.335	0.438
2545	0.780	0.364	0.359	0.464
2550	0.759	0.311	0.325	0.423
2555	0.759	0.298	0.320	0.414
2560	0.752	0.328	0.325	0.435
2565	0.785	0.372	0.367	0.470
2570	0.759	0.304	0.323	0.418
2575	0.783	0.356	0.361	0.459
2580	0.775	0.351	0.351	0.454
2585	0.782	0.373	0.364	0.471
2590	0.759	0.264	0.308	0.388
2595	0.766	0.365	0.347	0.462
2600	0.769	0.305	0.331	0.419
2605	0.730	0.241	0.280	0.371
2610	0.745	0.280	0.304	0.400
2615	0.783	0.363	0.362	0.464
2620	0.768	0.354	0.346	0.455
2625	0.759	0.337	0.333	0.442
2630	0.763	0.309	0.327	0.422
2635	0.794	0.371	0.376	0.471
2640	0.738	0.251	0.289	0.379
2645	0.766	0.320	0.333	0.430
2650	0.790	0.404	0.381	0.494

2655	0.752	0.275	0.307	0.397
2660	0.759	0.330	0.331	0.437
2665	0.763	0.315	0.329	0.427
2670	0.750	0.232	0.289	0.363
2675	0.782	0.360	0.360	0.462
2680	0.787	0.387	0.373	0.481
2685	0.771	0.359	0.350	0.459
2690	0.780	0.326	0.348	0.436
2695	0.759	0.298	0.320	0.414
2700	0.766	0.307	0.329	0.421
2705	0.766	0.339	0.340	0.444
2710	0.773	0.309	0.336	0.423
2715	0.757	0.275	0.311	0.397
2720	0.764	0.284	0.319	0.404
2725	0.740	0.219	0.277	0.353
2730	0.769	0.298	0.329	0.414
2735	0.782	0.335	0.352	0.442
2740	0.776	0.327	0.345	0.437
2745	0.783	0.388	0.369	0.481
2750	0.747	0.322	0.320	0.430
2755	0.769	0.344	0.344	0.448
2760	0.738	0.231	0.281	0.363
2765	0.771	0.314	0.336	0.426
2770	0.769	0.291	0.326	0.409
2775	0.773	0.289	0.328	0.407
2780	0.757	0.248	0.300	0.375
2785	0.775	0.318	0.340	0.430
2790	0.757	0.309	0.323	0.421
2795	0.766	0.307	0.329	0.421
2800	0.768	0.329	0.338	0.437
2805	0.749	0.271	0.303	0.393
2810	0.759	0.304	0.323	0.418
2815	0.783	0.369	0.364	0.468
2820	0.780	0.352	0.356	0.455
2825	0.749	0.317	0.320	0.427
2830	0.783	0.388	0.369	0.481
2835	0.792	0.337	0.364	0.444
2840	0.752	0.275	0.307	0.397
2845	0.773	0.329	0.342	0.438
2850	0.790	0.398	0.379	0.489
2855	0.766	0.293	0.324	0.410
2860	0.754	0.250	0.299	0.377
2865	0.747	0.341	0.326	0.443

2870	0.766	0.320	0.333	0.430
2875	0.783	0.344	0.357	0.449
2880	0.761	0.300	0.322	0.415
2885	0.740	0.205	0.271	0.342
2890	0.766	0.293	0.324	0.410
2895	0.754	0.264	0.304	0.388
2900	0.771	0.346	0.346	0.450
2905	0.756	0.306	0.321	0.420
2910	0.808	0.399	0.400	0.493
2915	0.773	0.323	0.340	0.433
2920	0.747	0.275	0.304	0.397
2925	0.782	0.373	0.364	0.471
2930	0.771	0.294	0.329	0.411
2935	0.738	0.292	0.304	0.408
2940	0.768	0.342	0.342	0.446
2945	0.728	0.273	0.291	0.394
2950	0.778	0.336	0.349	0.443
2955	0.750	0.266	0.303	0.390
2960	0.802	0.397	0.393	0.491
2965	0.771	0.320	0.338	0.431
2970	0.797	0.408	0.389	0.498
2975	0.763	0.275	0.315	0.396
2980	0.759	0.330	0.331	0.437
2985	0.769	0.285	0.324	0.404
2990	0.771	0.333	0.342	0.441
2995	0.782	0.348	0.356	0.452
3000	0.749	0.304	0.315	0.418
3005	0.776	0.353	0.353	0.456
3010	0.771	0.294	0.329	0.411
3015	0.785	0.313	0.348	0.426
3020	0.750	0.280	0.308	0.400
3025	0.775	0.364	0.354	0.463
3030	0.759	0.304	0.323	0.418
3035	0.783	0.311	0.346	0.424
3040	0.766	0.333	0.338	0.440
3045	0.783	0.382	0.368	0.477
3050	0.778	0.362	0.357	0.462
3055	0.742	0.214	0.276	0.349
3060	0.737	0.276	0.298	0.397
3065	0.757	0.315	0.325	0.426
3070	0.780	0.312	0.343	0.425
3075	0.775	0.325	0.342	0.435
3080	0.754	0.298	0.316	0.413

3085	0.759	0.298	0.320	0.414
3090	0.764	0.243	0.303	0.370
3095	0.782	0.360	0.360	0.462
3100	0.792	0.381	0.377	0.478
3105	0.783	0.394	0.371	0.486
3110	0.764	0.350	0.341	0.452
3115	0.794	0.409	0.386	0.498
3120	0.775	0.305	0.336	0.420
3125	0.747	0.328	0.322	0.434
3130	0.780	0.377	0.363	0.473
3135	0.745	0.280	0.304	0.400
3140	0.761	0.320	0.329	0.430
3145	0.764	0.356	0.343	0.456
3150	0.778	0.356	0.355	0.458
3155	0.778	0.317	0.343	0.429
3160	0.759	0.298	0.320	0.414
3165	0.773	0.323	0.340	0.433
3170	0.776	0.347	0.351	0.451
3175	0.757	0.289	0.316	0.407
3180	0.733	0.286	0.299	0.403
3185	0.775	0.331	0.345	0.440
3190	0.780	0.358	0.358	0.460
3195	0.769	0.350	0.346	0.453
3200	0.752	0.328	0.325	0.435
3205	0.761	0.286	0.318	0.405
3210	0.778	0.297	0.336	0.413
3215	0.742	0.276	0.301	0.397
3220	0.747	0.241	0.291	0.371
3225	0.773	0.336	0.344	0.443
3230	0.771	0.320	0.338	0.431
3235	0.747	0.269	0.301	0.392
3240	0.818	0.439	0.423	0.525
3245	0.787	0.316	0.351	0.428
3250	0.773	0.316	0.338	0.428
3255	0.790	0.379	0.374	0.476
3260	0.782	0.335	0.352	0.442
3265	0.789	0.338	0.360	0.445
3270	0.778	0.362	0.357	0.462
3275	0.782	0.354	0.358	0.457
3280	0.750	0.266	0.303	0.390
3285	0.780	0.332	0.350	0.441
3290	0.749	0.291	0.311	0.408
3295	0.775	0.331	0.345	0.440

3300	0.809	0.383	0.398	0.481
3305	0.761	0.306	0.325	0.420
3310	0.785	0.346	0.359	0.451
3315	0.757	0.289	0.316	0.407
3320	0.738	0.278	0.299	0.398
3325	0.804	0.393	0.394	0.489
3330	0.771	0.359	0.350	0.459
3335	0.776	0.366	0.357	0.465
3340	0.759	0.298	0.320	0.414
3345	0.776	0.321	0.343	0.432
3350	0.749	0.264	0.301	0.388
3355	0.773	0.296	0.331	0.413
3360	0.780	0.312	0.343	0.425
3365	0.766	0.259	0.311	0.384
3370	0.750	0.306	0.317	0.419
3375	0.773	0.316	0.338	0.428
3380	0.783	0.311	0.346	0.424
3385	0.801	0.337	0.374	0.444
3390	0.759	0.291	0.318	0.409
3395	0.787	0.303	0.346	0.417
3400	0.747	0.275	0.304	0.397
3405	0.789	0.332	0.358	0.440
3410	0.766	0.313	0.331	0.426
3415	0.769	0.318	0.336	0.429
3420	0.782	0.281	0.333	0.400
3425	0.776	0.321	0.343	0.432
3430	0.792	0.388	0.378	0.483
3435	0.754	0.304	0.319	0.418
3440	0.783	0.324	0.350	0.434
3445	0.763	0.348	0.339	0.450
3450	0.768	0.329	0.338	0.437
3455	0.742	0.242	0.288	0.371
3460	0.740	0.247	0.288	0.375
3465	0.757	0.295	0.318	0.412
3470	0.763	0.295	0.322	0.412
3475	0.768	0.309	0.331	0.422
3480	0.752	0.248	0.297	0.376
3485	0.757	0.262	0.306	0.386
3490	0.769	0.376	0.353	0.470
3495	0.771	0.307	0.333	0.421
3500	0.775	0.351	0.351	0.454
3505	0.742	0.256	0.293	0.382
3510	0.795	0.380	0.380	0.478

3515	0.752	0.255	0.299	0.381
3520	0.759	0.257	0.305	0.382
3525	0.785	0.352	0.361	0.456
3530	0.792	0.394	0.380	0.487
3535	0.773	0.276	0.323	0.396
3540	0.776	0.353	0.353	0.456
3545	0.802	0.397	0.393	0.491
3550	0.733	0.245	0.284	0.374
3555	0.792	0.350	0.368	0.455
3560	0.780	0.299	0.338	0.415
3565	0.795	0.348	0.371	0.454
3570	0.718	0.220	0.266	0.356
3575	0.787	0.303	0.346	0.417
3580	0.763	0.262	0.309	0.386
3585	0.768	0.262	0.313	0.385
3590	0.764	0.284	0.319	0.404
3595	0.763	0.282	0.317	0.402
3600	0.756	0.259	0.303	0.384
3605	0.747	0.269	0.301	0.392
3610	0.816	0.443	0.421	0.527
3615	0.730	0.220	0.272	0.355
3620	0.787	0.348	0.362	0.453
3625	0.750	0.273	0.305	0.395
3630	0.780	0.364	0.359	0.464
3635	0.789	0.395	0.377	0.487
3640	0.733	0.245	0.284	0.374
3645	0.773	0.349	0.348	0.452
3650	0.759	0.291	0.318	0.409
3655	0.780	0.383	0.365	0.477
3660	0.776	0.274	0.326	0.394
3665	0.773	0.329	0.342	0.438
3670	0.795	0.342	0.369	0.449
3675	0.733	0.265	0.292	0.389
3680	0.756	0.273	0.309	0.395
3685	0.744	0.278	0.302	0.398
3690	0.778	0.290	0.333	0.407
3695	0.766	0.293	0.324	0.410
3700	0.756	0.273	0.309	0.395
3705	0.728	0.218	0.270	0.354
3710	0.789	0.370	0.370	0.470
3715	0.768	0.289	0.324	0.407
3720	0.776	0.321	0.343	0.432
3725	0.749	0.250	0.295	0.378

3730	0.740	0.233	0.283	0.364
3735	0.776	0.308	0.338	0.422
3740	0.773	0.289	0.328	0.407
3745	0.737	0.236	0.282	0.367
3750	0.759	0.304	0.323	0.418
3755	0.778	0.343	0.351	0.448
3760	0.757	0.282	0.313	0.402
3765	0.778	0.310	0.341	0.423
3770	0.783	0.375	0.366	0.473
3775	0.763	0.322	0.331	0.432
3780	0.790	0.385	0.376	0.481
3785	0.763	0.315	0.329	0.427
3790	0.759	0.277	0.313	0.398
3795	0.799	0.422	0.395	0.508
3800	0.790	0.341	0.363	0.447
3805	0.766	0.313	0.331	0.426
3810	0.789	0.312	0.352	0.425
3815	0.764	0.304	0.327	0.419
3820	0.737	0.276	0.298	0.397
3825	0.780	0.332	0.350	0.441
3830	0.763	0.295	0.322	0.412
3835	0.783	0.331	0.353	0.439
3840	0.749	0.291	0.311	0.408
3845	0.735	0.198	0.265	0.338
3850	0.780	0.332	0.350	0.441
3855	0.744	0.223	0.281	0.357
3860	0.759	0.291	0.318	0.409
3865	0.726	0.284	0.294	0.402
3870	0.766	0.300	0.327	0.416
3875	0.754	0.271	0.307	0.393
3880	0.773	0.283	0.326	0.402
3885	0.757	0.255	0.303	0.381
3890	0.754	0.291	0.314	0.408
3895	0.771	0.300	0.331	0.416
3900	0.768	0.262	0.313	0.385
3905	0.768	0.329	0.338	0.437
3910	0.764	0.284	0.319	0.404
3915	0.757	0.282	0.313	0.402
3920	0.790	0.321	0.357	0.432
3925	0.776	0.308	0.338	0.422
3930	0.776	0.340	0.349	0.446
3935	0.763	0.255	0.307	0.380
3940	0.759	0.291	0.318	0.409

3945	0.745	0.300	0.311	0.414
3950	0.757	0.289	0.316	0.407
3955	0.794	0.421	0.389	0.506
3960	0.768	0.302	0.329	0.417
3965	0.782	0.341	0.354	0.447
3970	0.792	0.356	0.370	0.459
3975	0.776	0.347	0.351	0.451
3980	0.799	0.341	0.373	0.448
3985	0.757	0.268	0.308	0.391
3990	0.754	0.330	0.327	0.437
3995	0.764	0.304	0.327	0.419
4000	0.752	0.262	0.302	0.386
4005	0.773	0.309	0.336	0.423
4010	0.783	0.324	0.350	0.434
4015	0.759	0.291	0.318	0.409
4020	0.756	0.273	0.309	0.395
4025	0.787	0.355	0.364	0.458
4030	0.744	0.285	0.305	0.403
4035	0.740	0.274	0.299	0.395
4040	0.764	0.298	0.324	0.414
4045	0.757	0.289	0.316	0.407
4050	0.747	0.309	0.315	0.421
4055	0.742	0.262	0.296	0.387
4060	0.783	0.317	0.348	0.429
4065	0.756	0.280	0.311	0.400
4070	0.769	0.350	0.346	0.453
4075	0.771	0.287	0.326	0.405
4080	0.776	0.340	0.349	0.446
4085	0.775	0.376	0.358	0.472
4090	0.761	0.273	0.313	0.395
4095	0.782	0.354	0.358	0.457
4100	0.744	0.271	0.300	0.393
4105	0.768	0.289	0.324	0.407
4110	0.795	0.399	0.385	0.491
4115	0.737	0.236	0.282	0.367
4120	0.780	0.358	0.358	0.460
4125	0.740	0.253	0.291	0.380
4130	0.787	0.309	0.349	0.423
4135	0.773	0.303	0.333	0.418
4140	0.778	0.323	0.345	0.434
4145	0.768	0.361	0.348	0.460
4150	0.778	0.317	0.343	0.429
4155	0.776	0.314	0.340	0.427

4160	0.742	0.221	0.279	0.355
4165	0.761	0.280	0.315	0.400
4170	0.749	0.244	0.293	0.372
4175	0.780	0.292	0.336	0.409
4180	0.794	0.320	0.360	0.431
4185	0.759	0.271	0.310	0.393
4190	0.744	0.278	0.302	0.398
4195	0.749	0.291	0.311	0.408
4200	0.776	0.301	0.336	0.416
4205	0.768	0.309	0.331	0.422
4210	0.801	0.375	0.385	0.475
4215	0.731	0.257	0.287	0.382
4220	0.776	0.347	0.351	0.451
4225	0.745	0.300	0.311	0.414
4230	0.789	0.408	0.380	0.496
4235	0.782	0.335	0.352	0.442
4240	0.768	0.335	0.340	0.442
4245	0.789	0.389	0.375	0.483
4250	0.761	0.280	0.315	0.400
4255	0.768	0.282	0.321	0.402
4260	0.773	0.336	0.344	0.443
4265	0.795	0.411	0.388	0.500
4270	0.768	0.335	0.340	0.442
4275	0.782	0.328	0.350	0.438
4280	0.744	0.244	0.289	0.373
4285	0.757	0.234	0.294	0.364
4290	0.735	0.274	0.296	0.395
4295	0.790	0.366	0.371	0.467
4300	0.759	0.291	0.318	0.409
4305	0.794	0.390	0.381	0.485
4310	0.726	0.216	0.269	0.352
4315	0.723	0.226	0.271	0.360
4320	0.771	0.267	0.318	0.389
4325	0.756	0.326	0.327	0.434
4330	0.782	0.328	0.350	0.438
4335	0.764	0.291	0.322	0.409
4340	0.787	0.355	0.364	0.458
4345	0.778	0.323	0.345	0.434
4350	0.794	0.384	0.379	0.480
4355	0.773	0.316	0.338	0.428
4360	0.808	0.411	0.403	0.502
4365	0.766	0.259	0.311	0.384
4370	0.742	0.249	0.290	0.377

4375	0.747	0.262	0.299	0.387
4380	0.740	0.267	0.296	0.390
4385	0.745	0.280	0.304	0.400
4390	0.745	0.239	0.289	0.369
4395	0.750	0.286	0.310	0.405
4400	0.757	0.248	0.300	0.375
4405	0.780	0.339	0.352	0.445
4410	0.754	0.284	0.312	0.403
4415	0.771	0.307	0.333	0.421
4420	0.733	0.231	0.278	0.364
4425	0.761	0.320	0.329	0.430
4430	0.769	0.324	0.338	0.434
4435	0.757	0.309	0.323	0.421
4440	0.757	0.295	0.318	0.412
4445	0.778	0.323	0.345	0.434
4450	0.740	0.260	0.294	0.385
4455	0.794	0.403	0.384	0.494
4460	0.764	0.369	0.347	0.465
4465	0.797	0.370	0.380	0.471
4470	0.766	0.293	0.324	0.410
4475	0.754	0.229	0.290	0.360
4480	0.756	0.273	0.309	0.395
4485	0.756	0.300	0.318	0.415
4490	0.738	0.251	0.289	0.379
4495	0.795	0.374	0.379	0.473
4500	0.742	0.249	0.290	0.377
4505	0.724	0.200	0.261	0.340
4510	0.757	0.262	0.306	0.386
4515	0.769	0.324	0.338	0.434
4520	0.785	0.327	0.353	0.436
4525	0.747	0.275	0.304	0.397
4530	0.761	0.293	0.320	0.410
4535	0.764	0.291	0.322	0.409
4540	0.768	0.309	0.331	0.422
4545	0.783	0.356	0.361	0.459
4550	0.738	0.251	0.289	0.379
4555	0.789	0.364	0.368	0.465
4560	0.773	0.329	0.342	0.438
4565	0.771	0.333	0.342	0.441
4570	0.769	0.324	0.338	0.434
4575	0.757	0.354	0.337	0.453
4580	0.790	0.366	0.371	0.467
4585	0.768	0.302	0.329	0.417

4590	0.747	0.269	0.301	0.392
4595	0.789	0.332	0.358	0.440
4600	0.756	0.319	0.325	0.429
4605	0.782	0.360	0.360	0.462
4610	0.813	0.413	0.410	0.505
4615	0.759	0.264	0.308	0.388
4620	0.775	0.292	0.331	0.409
4625	0.797	0.370	0.380	0.471
4630	0.764	0.318	0.331	0.429
4635	0.773	0.309	0.336	0.423
4640	0.745	0.273	0.302	0.395
4645	0.768	0.309	0.331	0.422
4650	0.780	0.339	0.352	0.445
4655	0.775	0.292	0.331	0.409
4660	0.738	0.231	0.281	0.363
4665	0.804	0.349	0.382	0.454
4670	0.763	0.302	0.325	0.417
4675	0.778	0.323	0.345	0.434
4680	0.757	0.241	0.297	0.369
4685	0.775	0.325	0.342	0.435
4690	0.771	0.333	0.342	0.441
4695	0.769	0.257	0.313	0.381
4700	0.759	0.284	0.315	0.403
4705	0.783	0.356	0.361	0.459
4710	0.806	0.384	0.394	0.481
4715	0.745	0.239	0.289	0.369
4720	0.754	0.271	0.307	0.393
4725	0.747	0.269	0.301	0.392
4730	0.742	0.256	0.293	0.382
4735	0.756	0.319	0.325	0.429
4740	0.744	0.209	0.275	0.345
4745	0.773	0.323	0.340	0.433
4750	0.768	0.302	0.329	0.417
4755	0.766	0.333	0.338	0.440
4760	0.756	0.286	0.314	0.405
4765	0.749	0.237	0.290	0.367
4770	0.763	0.282	0.317	0.402
4775	0.787	0.316	0.351	0.428
4780	0.775	0.344	0.349	0.449
4785	0.792	0.400	0.382	0.492
4790	0.780	0.390	0.366	0.482
4795	0.757	0.268	0.308	0.391
4800	0.744	0.251	0.292	0.378

4805	0.768	0.309	0.331	0.422
4810	0.738	0.292	0.304	0.408
4815	0.776	0.327	0.345	0.437
4820	0.754	0.304	0.319	0.418
4825	0.754	0.271	0.307	0.393
4830	0.766	0.280	0.319	0.400
4835	0.771	0.359	0.350	0.459
4840	0.749	0.311	0.317	0.422
4845	0.731	0.250	0.285	0.378
4850	0.766	0.293	0.324	0.410
4855	0.768	0.361	0.348	0.460
4860	0.775	0.331	0.345	0.440
4865	0.782	0.348	0.356	0.452
4870	0.740	0.274	0.299	0.395
4875	0.769	0.298	0.329	0.414
4880	0.792	0.362	0.371	0.464
4885	0.749	0.244	0.293	0.372
4890	0.733	0.218	0.273	0.353
4895	0.792	0.343	0.366	0.450
4900	0.776	0.301	0.336	0.416
4905	0.752	0.309	0.319	0.421
4910	0.735	0.233	0.280	0.365
4915	0.747	0.282	0.306	0.402
4920	0.785	0.359	0.363	0.461
4925	0.768	0.302	0.329	0.417
4930	0.759	0.324	0.329	0.433
4935	0.761	0.293	0.320	0.410
4940	0.775	0.331	0.345	0.440
4945	0.761	0.280	0.315	0.400
4950	0.769	0.285	0.324	0.404
4955	0.766	0.280	0.319	0.400
4960	0.776	0.340	0.349	0.446
4965	0.804	0.381	0.391	0.479
4970	0.745	0.239	0.289	0.369
4975	0.780	0.339	0.352	0.445
4980	0.794	0.384	0.379	0.480
4985	0.787	0.322	0.353	0.433
4990	0.773	0.329	0.342	0.438
4995	0.780	0.326	0.348	0.436
5000	0.771	0.340	0.344	0.445
5005	0.790	0.321	0.357	0.432
5010	0.797	0.345	0.372	0.451
5015	0.773	0.289	0.328	0.407

5020	0.711	0.170	0.242	0.318
5025	0.750	0.286	0.310	0.405
5030	0.763	0.282	0.317	0.402
5035	0.756	0.259	0.303	0.384
5040	0.797	0.395	0.386	0.489
5045	0.750	0.273	0.305	0.395
5050	0.783	0.337	0.355	0.444
5055	0.759	0.291	0.318	0.409
5060	0.775	0.331	0.345	0.440
5065	0.795	0.380	0.380	0.478
5070	0.759	0.291	0.318	0.409
5075	0.783	0.369	0.364	0.468
5080	0.763	0.335	0.335	0.441
5085	0.816	0.413	0.415	0.505
5090	0.787	0.316	0.351	0.428
5095	0.750	0.266	0.303	0.390
5100	0.745	0.225	0.283	0.358
5105	0.783	0.304	0.344	0.419
5110	0.783	0.369	0.364	0.468
5115	0.747	0.302	0.313	0.416
5120	0.763	0.295	0.322	0.412
5125	0.761	0.286	0.318	0.405
5130	0.749	0.284	0.308	0.403
5135	0.787	0.322	0.353	0.433
5140	0.759	0.257	0.305	0.382
5145	0.776	0.340	0.349	0.446
5150	0.789	0.305	0.349	0.419
5155	0.763	0.282	0.317	0.402
5160	0.789	0.383	0.373	0.479
5165	0.771	0.300	0.331	0.416
5170	0.768	0.289	0.324	0.407
5175	0.764	0.291	0.322	0.409
5180	0.754	0.250	0.299	0.377
5185	0.744	0.251	0.292	0.378
5190	0.747	0.275	0.304	0.397
5195	0.776	0.301	0.336	0.416
5200	0.797	0.383	0.383	0.480
5205	0.757	0.289	0.316	0.407
5210	0.764	0.250	0.306	0.376
5215	0.782	0.360	0.360	0.462
5220	0.776	0.308	0.338	0.422
5225	0.766	0.287	0.322	0.405
5230	0.764	0.311	0.329	0.424

5235	0.749	0.257	0.298	0.383
5240	0.789	0.376	0.372	0.474
5245	0.750	0.286	0.310	0.405
5250	0.801	0.369	0.383	0.470
5255	0.757	0.275	0.311	0.397
5260	0.737	0.256	0.290	0.382
5265	0.744	0.278	0.302	0.398
5270	0.775	0.344	0.349	0.449
5275	0.757	0.289	0.316	0.407
5280	0.731	0.208	0.268	0.346
5285	0.794	0.346	0.368	0.452
5290	0.773	0.349	0.348	0.452
5295	0.773	0.329	0.342	0.438
5300	0.771	0.365	0.352	0.463
5305	0.761	0.300	0.322	0.415
5310	0.764	0.311	0.329	0.424
5315	0.750	0.306	0.317	0.419
5320	0.769	0.324	0.338	0.434
5325	0.763	0.275	0.315	0.396
5330	0.780	0.345	0.354	0.450
5335	0.773	0.336	0.344	0.443
5340	0.761	0.333	0.333	0.439
5345	0.744	0.285	0.305	0.403
5350	0.749	0.271	0.303	0.393
5355	0.764	0.311	0.329	0.424
5360	0.719	0.215	0.265	0.352
5365	0.744	0.230	0.284	0.362
5370	0.802	0.384	0.390	0.482
5375	0.742	0.269	0.298	0.392
5380	0.737	0.236	0.282	0.367
5385	0.775	0.357	0.353	0.458
5390	0.778	0.317	0.343	0.429
5395	0.733	0.238	0.281	0.369
5400	0.752	0.289	0.312	0.407
5405	0.761	0.273	0.313	0.395
5410	0.771	0.340	0.344	0.445
5415	0.761	0.313	0.327	0.425
5420	0.776	0.334	0.347	0.442
5425	0.764	0.324	0.333	0.433
5430	0.780	0.332	0.350	0.441
5435	0.773	0.323	0.340	0.433
5440	0.757	0.262	0.306	0.386
5445	0.776	0.347	0.351	0.451

5450	0.740	0.267	0.296	0.390
5455	0.764	0.324	0.333	0.433
5460	0.757	0.295	0.318	0.412
5465	0.726	0.216	0.269	0.352
5470	0.724	0.242	0.278	0.372
5475	0.775	0.344	0.349	0.449
5480	0.804	0.393	0.394	0.489
5485	0.771	0.359	0.350	0.459
5490	0.761	0.273	0.313	0.395
5495	0.799	0.385	0.386	0.482
5500	0.742	0.269	0.298	0.392
5505	0.768	0.380	0.353	0.472
5510	0.750	0.239	0.292	0.368
5515	0.754	0.304	0.319	0.418
5520	0.756	0.266	0.306	0.390
5525	0.744	0.271	0.300	0.393
5530	0.750	0.300	0.315	0.415
5535	0.756	0.319	0.325	0.429
5540	0.763	0.268	0.312	0.391
5545	0.785	0.333	0.355	0.441
5550	0.776	0.321	0.343	0.432
5555	0.745	0.293	0.309	0.410
5560	0.757	0.295	0.318	0.412
5565	0.795	0.355	0.373	0.459
5570	0.740	0.294	0.306	0.409
5575	0.775	0.292	0.331	0.409
5580	0.742	0.283	0.303	0.402
5585	0.749	0.298	0.313	0.413
5590	0.795	0.368	0.377	0.468
5595	0.769	0.311	0.333	0.424
5600	0.756	0.232	0.292	0.362
5605	0.752	0.302	0.317	0.416
5610	0.790	0.379	0.374	0.476
5615	0.776	0.334	0.347	0.442
5620	0.744	0.230	0.284	0.362
5625	0.756	0.313	0.323	0.424
5630	0.763	0.295	0.322	0.412
5635	0.742	0.256	0.293	0.382
5640	0.782	0.360	0.360	0.462
5645	0.771	0.327	0.340	0.436
5650	0.778	0.310	0.341	0.423
5655	0.794	0.403	0.384	0.494
5660	0.789	0.338	0.360	0.445

5665	0.790	0.379	0.374	0.476
5670	0.771	0.333	0.342	0.441
5675	0.766	0.273	0.317	0.395
5680	0.771	0.314	0.336	0.426
5685	0.768	0.322	0.336	0.432
5690	0.797	0.377	0.381	0.475
5695	0.769	0.344	0.344	0.448
5700	0.776	0.327	0.345	0.437
5705	0.749	0.284	0.308	0.403
5710	0.802	0.446	0.404	0.525
5715	0.771	0.307	0.333	0.421
5720	0.768	0.302	0.329	0.417
5725	0.757	0.289	0.316	0.407
5730	0.759	0.277	0.313	0.398
5735	0.761	0.266	0.310	0.389
5740	0.816	0.425	0.418	0.514
5745	0.761	0.306	0.325	0.420
5750	0.768	0.309	0.331	0.422
5755	0.768	0.296	0.326	0.412
5760	0.802	0.421	0.399	0.509
5765	0.785	0.378	0.368	0.475
5770	0.787	0.387	0.373	0.481
5775	0.771	0.294	0.329	0.411
5780	0.752	0.262	0.302	0.386
5785	0.806	0.377	0.392	0.477
5790	0.801	0.375	0.385	0.475
5795	0.782	0.354	0.358	0.457
5800	0.802	0.409	0.396	0.500
5805	0.773	0.342	0.346	0.447
5810	0.730	0.206	0.266	0.345
5815	0.787	0.329	0.356	0.438
5820	0.766	0.300	0.327	0.416
5825	0.778	0.303	0.338	0.418
5830	0.756	0.266	0.306	0.390
5835	0.773	0.283	0.326	0.402
5840	0.763	0.289	0.320	0.407
5845	0.750	0.239	0.292	0.368
5850	0.783	0.311	0.346	0.424
5855	0.769	0.305	0.331	0.419
5860	0.749	0.244	0.293	0.372
5865	0.754	0.215	0.284	0.349
5870	0.790	0.360	0.369	0.462
5875	0.821	0.427	0.426	0.516

5880	0.787	0.368	0.367	0.468
5885	0.802	0.391	0.391	0.486
5890	0.730	0.199	0.263	0.339
5895	0.780	0.251	0.319	0.374
5900	0.744	0.251	0.292	0.378
5905	0.742	0.221	0.279	0.355
5910	0.775	0.357	0.353	0.458
5915	0.771	0.260	0.315	0.383
5920	0.756	0.239	0.295	0.368
5925	0.768	0.296	0.326	0.412
5930	0.773	0.289	0.328	0.407
5935	0.768	0.329	0.338	0.437
5940	0.756	0.225	0.289	0.356
5945	0.733	0.238	0.281	0.369
5950	0.752	0.262	0.302	0.386
5955	0.731	0.208	0.268	0.346
5960	0.745	0.293	0.309	0.410
5965	0.769	0.331	0.340	0.439
5970	0.797	0.408	0.389	0.498
5975	0.761	0.273	0.313	0.395
5980	0.764	0.344	0.340	0.447
5985	0.766	0.246	0.305	0.372
5990	0.733	0.160	0.246	0.306
5995	0.757	0.262	0.306	0.386
6000	0.756	0.319	0.325	0.429
6005	0.783	0.311	0.346	0.424
6010	0.789	0.292	0.344	0.408
6015	0.742	0.200	0.270	0.338
6020	0.724	0.207	0.264	0.346
6025	0.785	0.273	0.333	0.392
6030	0.766	0.259	0.311	0.384
6035	0.752	0.282	0.310	0.402
6040	0.773	0.303	0.333	0.418
6045	0.769	0.271	0.319	0.393
6050	0.773	0.336	0.344	0.443
6055	0.787	0.316	0.351	0.428
6060	0.766	0.224	0.296	0.354
6065	0.782	0.308	0.343	0.422
6070	0.761	0.245	0.301	0.373
6075	0.771	0.273	0.321	0.394
6080	0.728	0.225	0.273	0.359
6085	0.754	0.243	0.296	0.372
6090	0.776	0.274	0.326	0.394

6095	0.775	0.331	0.345	0.440
6100	0.785	0.327	0.353	0.436
6105	0.756	0.232	0.292	0.362
6110	0.764	0.215	0.290	0.346
6115	0.744	0.223	0.281	0.357
6120	0.776	0.334	0.347	0.442
6125	0.790	0.321	0.357	0.432
6130	0.797	0.357	0.376	0.461
6135	0.811	0.429	0.411	0.516
6140	0.780	0.265	0.325	0.386
6145	0.757	0.282	0.313	0.402
6150	0.763	0.302	0.325	0.417
6155	0.764	0.264	0.312	0.387
6160	0.783	0.317	0.348	0.429
6165	0.775	0.292	0.331	0.409
6170	0.747	0.269	0.301	0.392
6175	0.756	0.232	0.292	0.362
6180	0.827	0.442	0.438	0.528
6185	0.723	0.155	0.240	0.304
6190	0.804	0.393	0.394	0.489
6195	0.719	0.194	0.256	0.336
6200	0.740	0.233	0.283	0.364
6205	0.761	0.266	0.310	0.389
6210	0.712	0.208	0.259	0.346
6215	0.738	0.210	0.272	0.346
6220	0.809	0.395	0.402	0.491
6225	0.775	0.292	0.331	0.409
6230	0.771	0.239	0.306	0.365
6235	0.752	0.248	0.297	0.376
6240	0.799	0.398	0.389	0.491
6245	0.816	0.394	0.411	0.490
6250	0.789	0.305	0.349	0.419
6255	0.750	0.232	0.289	0.363
6260	0.752	0.269	0.305	0.391
6265	0.757	0.275	0.311	0.397
6270	0.749	0.271	0.303	0.393
6275	0.749	0.250	0.295	0.378
6280	0.766	0.238	0.302	0.366
6285	0.749	0.250	0.295	0.378
6290	0.750	0.225	0.286	0.357
6295	0.794	0.326	0.362	0.436
6300	0.756	0.280	0.311	0.400
6305	0.757	0.241	0.297	0.369

6310	0.782	0.308	0.343	0.422
6315	0.761	0.252	0.304	0.378
6320	0.750	0.239	0.292	0.368
6325	0.752	0.241	0.294	0.370
6330	0.752	0.255	0.299	0.381
6335	0.740	0.233	0.283	0.364
6340	0.787	0.329	0.356	0.438
6345	0.790	0.404	0.381	0.494
6350	0.780	0.326	0.348	0.436
6355	0.757	0.241	0.297	0.369
6360	0.790	0.354	0.367	0.457
6365	0.766	0.259	0.311	0.384
6370	0.785	0.346	0.359	0.451
6375	0.775	0.271	0.323	0.393
6380	0.738	0.144	0.240	0.291
6385	0.797	0.345	0.372	0.451
6390	0.756	0.246	0.298	0.373
6395	0.749	0.271	0.303	0.393
6400	0.787	0.329	0.356	0.438
6405	0.764	0.278	0.317	0.398
6410	0.766	0.252	0.308	0.378
6415	0.750	0.253	0.297	0.379
6420	0.794	0.352	0.370	0.457
6425	0.744	0.251	0.292	0.378
6430	0.738	0.265	0.294	0.389
6435	0.768	0.316	0.333	0.427
6440	0.778	0.336	0.349	0.443
6445	0.806	0.402	0.399	0.495
6450	0.733	0.196	0.264	0.336
6455	0.757	0.248	0.300	0.375
6460	0.759	0.222	0.290	0.353
6465	0.757	0.268	0.308	0.391
6470	0.750	0.253	0.297	0.379
6475	0.761	0.217	0.289	0.349
6480	0.742	0.228	0.282	0.361
6485	0.737	0.164	0.250	0.309
6490	0.773	0.309	0.336	0.423
6495	0.787	0.303	0.346	0.417
6500	0.718	0.185	0.252	0.329
6505	0.764	0.291	0.322	0.409
6510	0.816	0.425	0.418	0.514
6515	0.778	0.368	0.359	0.467
6520	0.776	0.308	0.338	0.422

6525	0.731	0.215	0.271	0.351
6530	0.766	0.287	0.322	0.405
6535	0.719	0.151	0.236	0.302
6540	0.764	0.278	0.317	0.398
6545	0.766	0.238	0.302	0.366
6550	0.780	0.292	0.336	0.409
6555	0.775	0.285	0.328	0.404
6560	0.750	0.204	0.276	0.339
6565	0.785	0.365	0.365	0.466
6570	0.749	0.298	0.313	0.413
6575	0.745	0.197	0.270	0.335
6580	0.771	0.307	0.333	0.421
6585	0.799	0.347	0.375	0.453
6590	0.737	0.201	0.267	0.339
6595	0.747	0.255	0.296	0.381
6600	0.756	0.273	0.309	0.395
6605	0.789	0.408	0.380	0.496
6610	0.794	0.352	0.370	0.457
6615	0.780	0.319	0.345	0.430
6620	0.759	0.257	0.305	0.382
6625	0.782	0.335	0.352	0.442
6630	0.763	0.255	0.307	0.380
6635	0.785	0.352	0.361	0.456
6640	0.731	0.215	0.271	0.351
6645	0.785	0.300	0.344	0.415
6650	0.733	0.160	0.246	0.306
6655	0.778	0.381	0.363	0.475
6660	0.766	0.293	0.324	0.410
6665	0.790	0.328	0.359	0.437
6670	0.787	0.336	0.358	0.443
6675	0.787	0.405	0.377	0.494
6680	0.790	0.341	0.363	0.447
6685	0.775	0.298	0.333	0.414
6690	0.749	0.284	0.308	0.403
6695	0.771	0.287	0.326	0.405
6700	0.783	0.363	0.362	0.464
6705	0.759	0.250	0.302	0.377
6710	0.763	0.262	0.309	0.386
6715	0.766	0.273	0.317	0.395
6720	0.752	0.262	0.302	0.386
6725	0.775	0.285	0.328	0.404
6730	0.759	0.257	0.305	0.382
6735	0.799	0.379	0.384	0.477

6740	0.757	0.268	0.308	0.391
6745	0.775	0.278	0.326	0.398
6750	0.756	0.232	0.292	0.362
6755	0.766	0.287	0.322	0.405
6760	0.730	0.141	0.235	0.291
6765	0.757	0.322	0.327	0.431
6770	0.742	0.249	0.290	0.377
6775	0.764	0.284	0.319	0.404
6780	0.764	0.278	0.317	0.398
6785	0.757	0.227	0.291	0.358
6790	0.757	0.295	0.318	0.412
6795	0.776	0.327	0.345	0.437
6800	0.709	0.161	0.238	0.311
6805	0.752	0.262	0.302	0.386
6810	0.724	0.200	0.261	0.340
6815	0.766	0.293	0.324	0.410
6820	0.795	0.374	0.379	0.473
6825	0.752	0.248	0.297	0.376
6830	0.728	0.176	0.252	0.320
6835	0.778	0.330	0.347	0.439
6840	0.763	0.282	0.317	0.402
6845	0.764	0.311	0.329	0.424
6850	0.757	0.241	0.297	0.369
6855	0.782	0.360	0.360	0.462
6860	0.744	0.278	0.302	0.398
6865	0.794	0.339	0.366	0.447
6870	0.744	0.244	0.289	0.373
6875	0.721	0.203	0.261	0.343
6880	0.737	0.249	0.288	0.377
6885	0.768	0.275	0.319	0.396
6890	0.712	0.172	0.244	0.320
6895	0.763	0.289	0.320	0.407
6900	0.787	0.368	0.367	0.468
6905	0.763	0.282	0.317	0.402
6910	0.773	0.296	0.331	0.413
6915	0.771	0.307	0.333	0.421
6920	0.766	0.320	0.333	0.430
6925	0.747	0.228	0.285	0.360
6930	0.744	0.216	0.278	0.351
6935	0.733	0.224	0.276	0.358
6940	0.771	0.287	0.326	0.405
6945	0.754	0.264	0.304	0.388
6950	0.754	0.208	0.280	0.343

6955	0.757	0.248	0.300	0.375
6960	0.802	0.378	0.388	0.477
6965	0.761	0.313	0.327	0.425
6970	0.764	0.311	0.329	0.424
6975	0.761	0.217	0.289	0.349
6980	0.723	0.191	0.256	0.333
6985	0.782	0.302	0.341	0.417
6990	0.749	0.216	0.281	0.350
6995	0.761	0.245	0.301	0.373
7000	0.766	0.273	0.317	0.395
7005	0.747	0.282	0.306	0.402
7010	0.768	0.255	0.311	0.380
7015	0.750	0.225	0.286	0.357
7020	0.754	0.291	0.314	0.408
7025	0.759	0.264	0.308	0.388
7030	0.731	0.151	0.241	0.299
7035	0.744	0.237	0.287	0.368
7040	0.761	0.313	0.327	0.425
7045	0.724	0.200	0.261	0.340
7050	0.776	0.314	0.340	0.427
7055	0.745	0.246	0.291	0.374
7060	0.750	0.218	0.283	0.351
7065	0.761	0.245	0.301	0.373
7070	0.757	0.268	0.308	0.391
7075	0.763	0.262	0.309	0.386
7080	0.794	0.346	0.368	0.452
7085	0.766	0.300	0.327	0.416
7090	0.792	0.356	0.370	0.459
7095	0.792	0.356	0.370	0.459
7100	0.761	0.224	0.292	0.355
7105	0.757	0.255	0.303	0.381
7110	0.766	0.326	0.335	0.435
7115	0.771	0.320	0.338	0.431
7120	0.785	0.333	0.355	0.441
7125	0.797	0.332	0.368	0.440
7130	0.730	0.255	0.286	0.381
7135	0.775	0.338	0.347	0.444
7140	0.783	0.324	0.350	0.434
7145	0.776	0.294	0.333	0.411
7150	0.749	0.298	0.313	0.413
7155	0.771	0.307	0.333	0.421
7160	0.757	0.234	0.294	0.364
7165	0.724	0.200	0.261	0.340

7170	0.749	0.223	0.284	0.356
7175	0.733	0.224	0.276	0.358
7180	0.733	0.211	0.270	0.347
7185	0.775	0.338	0.347	0.444
7190	0.740	0.247	0.288	0.375
7195	0.738	0.188	0.262	0.329
7200	0.757	0.220	0.288	0.352
7205	0.776	0.366	0.357	0.465
7210	0.712	0.165	0.241	0.314
7215	0.750	0.225	0.286	0.357
7220	0.752	0.191	0.271	0.329
7225	0.778	0.290	0.333	0.407
7230	0.773	0.323	0.340	0.433
7235	0.757	0.227	0.291	0.358
7240	0.768	0.302	0.329	0.417
7245	0.769	0.305	0.331	0.419
7250	0.750	0.232	0.289	0.363
7255	0.752	0.248	0.297	0.376
7260	0.752	0.282	0.310	0.402
7265	0.709	0.190	0.250	0.333
7270	0.752	0.262	0.302	0.386
7275	0.792	0.369	0.373	0.469
7280	0.754	0.250	0.299	0.377
7285	0.773	0.316	0.338	0.428
7290	0.763	0.262	0.309	0.386
7295	0.747	0.241	0.291	0.371
7300	0.778	0.297	0.336	0.413
7305	0.768	0.241	0.305	0.368
7310	0.763	0.289	0.320	0.407
7315	0.768	0.219	0.295	0.350
7320	0.735	0.198	0.265	0.338
7325	0.769	0.311	0.333	0.424
7330	0.763	0.268	0.312	0.391
7335	0.789	0.318	0.354	0.430
7340	0.750	0.232	0.289	0.363
7345	0.738	0.217	0.275	0.352
7350	0.750	0.266	0.303	0.390
7355	0.733	0.218	0.273	0.353
7360	0.712	0.172	0.244	0.320
7365	0.792	0.375	0.375	0.474
7370	0.792	0.310	0.355	0.423
7375	0.730	0.156	0.243	0.304
7380	0.773	0.342	0.346	0.447

7385	0.782	0.386	0.367	0.479
7390	0.737	0.193	0.264	0.333
7395	0.752	0.227	0.288	0.359
7400	0.775	0.292	0.331	0.409
7405	0.764	0.318	0.331	0.429
7410	0.780	0.306	0.341	0.420
7415	0.754	0.243	0.296	0.372
7420	0.761	0.252	0.304	0.378
7425	0.726	0.202	0.263	0.342
7430	0.740	0.247	0.288	0.375
7435	0.757	0.241	0.297	0.369
7440	0.775	0.271	0.323	0.393
7445	0.775	0.305	0.336	0.420
7450	0.750	0.246	0.295	0.374
7455	0.799	0.347	0.375	0.453
7460	0.766	0.300	0.327	0.416
7465	0.768	0.335	0.340	0.442
7470	0.789	0.325	0.356	0.435
7475	0.769	0.285	0.324	0.404
7480	0.763	0.275	0.315	0.396
7485	0.723	0.177	0.250	0.322
7490	0.763	0.309	0.327	0.422
7495	0.757	0.295	0.318	0.412
7500	0.761	0.333	0.333	0.439
7505	0.750	0.239	0.292	0.368
7510	0.735	0.240	0.283	0.370
7515	0.757	0.268	0.308	0.391
7520	0.742	0.221	0.279	0.355
7525	0.756	0.196	0.276	0.332
7530	0.754	0.271	0.307	0.393
7535	0.747	0.248	0.293	0.376
7540	0.757	0.335	0.331	0.440
7545	0.766	0.246	0.305	0.372
7550	0.768	0.302	0.329	0.417
7555	0.811	0.404	0.406	0.498
7560	0.780	0.339	0.352	0.445
7565	0.782	0.341	0.354	0.447
7570	0.747	0.309	0.315	0.421
7575	0.704	0.134	0.223	0.290
7580	0.726	0.237	0.277	0.368
7585	0.763	0.302	0.325	0.417
7590	0.769	0.305	0.331	0.419
7595	0.787	0.316	0.351	0.428

7600	0.737	0.290	0.302	0.406
7605	0.763	0.302	0.325	0.417
7610	0.742	0.171	0.256	0.313
7615	0.778	0.317	0.343	0.429
7620	0.778	0.317	0.343	0.429
7625	0.782	0.328	0.350	0.438
7630	0.789	0.351	0.364	0.455
7635	0.776	0.347	0.351	0.451
7640	0.773	0.349	0.348	0.452
7645	0.740	0.280	0.301	0.400
7650	0.778	0.317	0.343	0.429
7655	0.733	0.218	0.273	0.353
7660	0.782	0.302	0.341	0.417
7665	0.756	0.280	0.311	0.400
7670	0.763	0.302	0.325	0.417
7675	0.768	0.302	0.329	0.417
7680	0.730	0.241	0.280	0.371
7685	0.737	0.249	0.288	0.377
7690	0.775	0.318	0.340	0.430
7695	0.792	0.381	0.377	0.478
7700	0.719	0.236	0.273	0.367
7705	0.768	0.296	0.326	0.412
7710	0.794	0.365	0.374	0.466
7715	0.766	0.266	0.314	0.389
7720	0.750	0.253	0.297	0.379
7725	0.747	0.248	0.293	0.376
7730	0.757	0.275	0.311	0.397
7735	0.723	0.191	0.256	0.333
7740	0.756	0.286	0.314	0.405
7745	0.744	0.251	0.292	0.378
7750	0.756	0.280	0.311	0.400
7755	0.742	0.221	0.279	0.355
7760	0.747	0.282	0.306	0.402
7765	0.733	0.265	0.292	0.389
7770	0.761	0.259	0.307	0.384
7775	0.769	0.298	0.329	0.414
7780	0.768	0.322	0.336	0.432
7785	0.740	0.267	0.296	0.390
7790	0.787	0.322	0.353	0.433
7795	0.738	0.258	0.292	0.384
7800	0.747	0.228	0.285	0.360
7805	0.787	0.336	0.358	0.443
7810	0.782	0.367	0.362	0.466

7815	0.785	0.307	0.346	0.421
7820	0.716	0.190	0.253	0.333
7825	0.697	0.141	0.224	0.297
7830	0.769	0.305	0.331	0.419
7835	0.761	0.300	0.322	0.415
7840	0.764	0.298	0.324	0.414
7845	0.778	0.310	0.341	0.423
7850	0.756	0.300	0.318	0.415
7855	0.737	0.236	0.282	0.367
7860	0.740	0.274	0.299	0.395
7865	0.752	0.255	0.299	0.381
7870	0.726	0.195	0.260	0.336
7875	0.799	0.328	0.369	0.437
7880	0.764	0.264	0.312	0.387
7885	0.754	0.250	0.299	0.377
7890	0.778	0.290	0.333	0.407
7895	0.775	0.285	0.328	0.404
7900	0.752	0.302	0.317	0.416
7905	0.737	0.249	0.288	0.377
7910	0.782	0.315	0.346	0.427
7915	0.756	0.239	0.295	0.368
7920	0.790	0.354	0.367	0.457
7925	0.747	0.302	0.313	0.416
7930	0.783	0.331	0.353	0.439
7935	0.763	0.282	0.317	0.402
7940	0.823	0.406	0.425	0.500
7945	0.747	0.282	0.306	0.402
7950	0.783	0.297	0.341	0.413
7955	0.769	0.243	0.307	0.370
7960	0.747	0.248	0.293	0.376
7965	0.797	0.401	0.388	0.494
7970	0.721	0.224	0.269	0.359
7975	0.783	0.350	0.359	0.454
7980	0.808	0.374	0.394	0.474
7985	0.780	0.339	0.352	0.445
7990	0.766	0.293	0.324	0.410
7995	0.745	0.246	0.291	0.374
8000	0.775	0.351	0.351	0.454
8005	0.795	0.417	0.390	0.504
8010	0.768	0.296	0.326	0.412
8015	0.771	0.314	0.336	0.426
8020	0.799	0.379	0.384	0.477
8025	0.728	0.239	0.279	0.369

8030	0.757	0.315	0.325	0.426
8035	0.742	0.262	0.296	0.387
8040	0.737	0.249	0.288	0.377
8045	0.754	0.264	0.304	0.388
8050	0.756	0.266	0.306	0.390
8055	0.783	0.331	0.353	0.439
8060	0.794	0.346	0.368	0.452
8065	0.740	0.219	0.277	0.353
8070	0.763	0.289	0.320	0.407
8075	0.780	0.332	0.350	0.441
8080	0.776	0.308	0.338	0.422
8085	0.775	0.331	0.345	0.440
8090	0.756	0.239	0.295	0.368
8095	0.785	0.365	0.365	0.466
8100	0.815	0.446	0.420	0.529
8105	0.747	0.255	0.296	0.381
8110	0.759	0.250	0.302	0.377
8115	0.783	0.311	0.346	0.424
8120	0.761	0.259	0.307	0.384
8125	0.756	0.246	0.298	0.373
8130	0.749	0.223	0.284	0.356
8135	0.768	0.296	0.326	0.412
8140	0.742	0.249	0.290	0.377
8145	0.750	0.239	0.292	0.368
8150	0.789	0.305	0.349	0.419
8155	0.742	0.228	0.282	0.361
8160	0.761	0.293	0.320	0.410
8165	0.750	0.239	0.292	0.368
8170	0.754	0.222	0.287	0.355
8175	0.740	0.240	0.286	0.370
8180	0.759	0.277	0.313	0.398
8185	0.747	0.241	0.291	0.371
8190	0.776	0.281	0.328	0.400
8195	0.768	0.361	0.348	0.460
8200	0.763	0.322	0.331	0.432
8205	0.754	0.236	0.293	0.366
8210	0.775	0.305	0.336	0.420
8215	0.782	0.335	0.352	0.442
8220	0.752	0.206	0.278	0.341
8225	0.792	0.362	0.371	0.464
8230	0.768	0.296	0.326	0.412
8235	0.792	0.369	0.373	0.469
8240	0.778	0.276	0.328	0.396

8245	0.778	0.297	0.336	0.413
8250	0.745	0.253	0.294	0.380
8255	0.778	0.263	0.323	0.385
8260	0.778	0.362	0.357	0.462
8265	0.752	0.248	0.297	0.376
8270	0.749	0.250	0.295	0.378
8275	0.780	0.292	0.336	0.409
8280	0.752	0.289	0.312	0.407
8285	0.785	0.286	0.339	0.404
8290	0.778	0.343	0.351	0.448
8295	0.763	0.248	0.304	0.374
8300	0.752	0.302	0.317	0.416
8305	0.764	0.311	0.329	0.424
8310	0.766	0.313	0.331	0.426
8315	0.756	0.252	0.301	0.379
8320	0.737	0.263	0.293	0.387
8325	0.763	0.268	0.312	0.391
8330	0.756	0.259	0.303	0.384
8335	0.749	0.264	0.301	0.388
8340	0.782	0.288	0.336	0.406
8345	0.728	0.176	0.252	0.320
8350	0.766	0.320	0.333	0.430
8355	0.749	0.257	0.298	0.383
8360	0.768	0.262	0.313	0.385
8365	0.806	0.365	0.389	0.467
8370	0.756	0.313	0.323	0.424
8375	0.759	0.257	0.305	0.382
8380	0.789	0.351	0.364	0.455
8385	0.752	0.220	0.285	0.353
8390	0.756	0.174	0.264	0.312
8395	0.759	0.298	0.320	0.414
8400	0.768	0.282	0.321	0.402
8405	0.756	0.259	0.303	0.384
8410	0.780	0.292	0.336	0.409
8415	0.771	0.300	0.331	0.416
8420	0.761	0.306	0.325	0.420
8425	0.785	0.346	0.359	0.451
8430	0.769	0.257	0.313	0.381
8435	0.768	0.269	0.316	0.391
8440	0.766	0.246	0.305	0.372
8445	0.750	0.253	0.297	0.379
8450	0.759	0.271	0.310	0.393
8455	0.773	0.283	0.326	0.402

8460	0.785	0.307	0.346	0.421
8465	0.776	0.308	0.338	0.422
8470	0.787	0.336	0.358	0.443
8475	0.744	0.188	0.265	0.327
8480	0.771	0.280	0.324	0.400
8485	0.783	0.331	0.353	0.439
8490	0.771	0.287	0.326	0.405
8495	0.744	0.237	0.287	0.368
8500	0.737	0.270	0.295	0.392
8505	0.756	0.293	0.316	0.410
8510	0.776	0.301	0.336	0.416
8515	0.768	0.289	0.324	0.407
8520	0.754	0.257	0.301	0.383
8525	0.763	0.255	0.307	0.380
8530	0.744	0.244	0.289	0.373
8535	0.740	0.198	0.268	0.336
8540	0.764	0.284	0.319	0.404
8545	0.785	0.365	0.365	0.466
8550	0.747	0.296	0.311	0.411
8555	0.789	0.370	0.370	0.470
8560	0.764	0.250	0.306	0.376
8565	0.783	0.350	0.359	0.454
8570	0.799	0.328	0.369	0.437
8575	0.763	0.268	0.312	0.391
8580	0.785	0.266	0.331	0.386
8585	0.773	0.329	0.342	0.438
8590	0.780	0.286	0.333	0.404
8595	0.759	0.257	0.305	0.382
8600	0.763	0.282	0.317	0.402
8605	0.756	0.266	0.306	0.390
8610	0.804	0.375	0.389	0.474
8615	0.768	0.309	0.331	0.422
8620	0.766	0.287	0.322	0.405
8625	0.761	0.300	0.322	0.415
8630	0.780	0.332	0.350	0.441
8635	0.766	0.313	0.331	0.426
8640	0.783	0.284	0.336	0.402
8645	0.761	0.326	0.331	0.434
8650	0.742	0.235	0.285	0.366
8655	0.759	0.298	0.320	0.414
8660	0.745	0.182	0.263	0.323
8665	0.740	0.247	0.288	0.375
8670	0.802	0.346	0.379	0.452

8675	0.759	0.271	0.310	0.393
8680	0.778	0.317	0.343	0.429
8685	0.768	0.309	0.331	0.422
8690	0.794	0.384	0.379	0.480
8695	0.745	0.218	0.280	0.352
8700	0.759	0.271	0.310	0.393
8705	0.754	0.243	0.296	0.372
8710	0.764	0.369	0.347	0.465
8715	0.775	0.278	0.326	0.398
8720	0.790	0.314	0.354	0.427
8725	0.776	0.321	0.343	0.432
8730	0.789	0.383	0.373	0.479
8735	0.735	0.240	0.283	0.370
8740	0.773	0.276	0.323	0.396
8745	0.757	0.275	0.311	0.397
8750	0.768	0.275	0.319	0.396
8755	0.735	0.206	0.268	0.343
8760	0.775	0.298	0.333	0.414
8765	0.782	0.322	0.348	0.432
8770	0.731	0.187	0.259	0.329
8775	0.745	0.287	0.307	0.405
8780	0.733	0.224	0.276	0.358
8785	0.757	0.234	0.294	0.364
8790	0.766	0.287	0.322	0.405
8795	0.757	0.282	0.313	0.402
8800	0.799	0.373	0.382	0.473
8805	0.785	0.320	0.351	0.431
8810	0.789	0.376	0.372	0.474
8815	0.795	0.355	0.373	0.459
8820	0.730	0.248	0.283	0.376
8825	0.769	0.264	0.316	0.387
8830	0.728	0.218	0.270	0.354
8835	0.775	0.331	0.345	0.440
8840	0.735	0.254	0.288	0.381
8845	0.756	0.217	0.286	0.350
8850	0.742	0.221	0.279	0.355
8855	0.749	0.250	0.295	0.378
8860	0.778	0.310	0.341	0.423
8865	0.737	0.222	0.276	0.356
8870	0.761	0.252	0.304	0.378
8875	0.769	0.271	0.319	0.393
8880	0.740	0.226	0.280	0.359
8885	0.776	0.301	0.336	0.416

8890	0.759	0.291	0.318	0.409
8895	0.759	0.264	0.308	0.388
8900	0.802	0.372	0.386	0.472
8905	0.759	0.284	0.315	0.403
8910	0.775	0.305	0.336	0.420
8915	0.759	0.229	0.293	0.359
8920	0.742	0.249	0.290	0.377
8925	0.738	0.245	0.287	0.373
8930	0.745	0.218	0.280	0.352
8935	0.789	0.345	0.362	0.450
8940	0.771	0.287	0.326	0.405
8945	0.785	0.372	0.367	0.470
8950	0.745	0.239	0.289	0.369
8955	0.769	0.318	0.336	0.429
8960	0.759	0.250	0.302	0.377
8965	0.776	0.321	0.343	0.432
8970	0.738	0.217	0.275	0.352
8975	0.742	0.221	0.279	0.355
8980	0.766	0.300	0.327	0.416
8985	0.783	0.317	0.348	0.429
8990	0.754	0.257	0.301	0.383
8995	0.783	0.311	0.346	0.424
9000	0.769	0.331	0.340	0.439
9005	0.811	0.379	0.400	0.478
9010	0.766	0.273	0.317	0.395
9015	0.790	0.360	0.369	0.462
9020	0.752	0.275	0.307	0.397
9025	0.740	0.212	0.274	0.348
9030	0.768	0.302	0.329	0.417
9035	0.776	0.301	0.336	0.416
9040	0.749	0.291	0.311	0.408
9045	0.775	0.312	0.338	0.425
9050	0.785	0.307	0.346	0.421
9055	0.776	0.308	0.338	0.422
9060	0.733	0.259	0.289	0.384
9065	0.785	0.340	0.357	0.446
9070	0.771	0.287	0.326	0.405
9075	0.723	0.170	0.247	0.316
9080	0.747	0.282	0.306	0.402
9085	0.802	0.391	0.391	0.486
9090	0.754	0.343	0.331	0.445
9095	0.806	0.390	0.396	0.486
9100	0.783	0.356	0.361	0.459

9105	0.776	0.321	0.343	0.432
9110	0.759	0.257	0.305	0.382
9115	0.782	0.335	0.352	0.442
9120	0.790	0.354	0.367	0.457
9125	0.726	0.174	0.250	0.319
9130	0.764	0.264	0.312	0.387
9135	0.759	0.257	0.305	0.382
9140	0.771	0.327	0.340	0.436
9145	0.750	0.266	0.303	0.390
9150	0.792	0.400	0.382	0.492
9155	0.764	0.318	0.331	0.429
9160	0.771	0.294	0.329	0.411
9165	0.768	0.289	0.324	0.407
9170	0.766	0.273	0.317	0.395
9175	0.754	0.243	0.296	0.372
9180	0.773	0.303	0.333	0.418
9185	0.797	0.383	0.383	0.480
9190	0.801	0.375	0.385	0.475
9195	0.802	0.346	0.379	0.452
9200	0.776	0.287	0.331	0.406
9205	0.776	0.334	0.347	0.442
9210	0.775	0.305	0.336	0.420
9215	0.763	0.282	0.317	0.402
9220	0.742	0.309	0.312	0.420
9225	0.766	0.320	0.333	0.430
9230	0.752	0.269	0.305	0.391
9235	0.749	0.216	0.281	0.350
9240	0.787	0.348	0.362	0.453
9245	0.766	0.326	0.335	0.435
9250	0.763	0.275	0.315	0.396
9255	0.802	0.359	0.383	0.462
9260	0.775	0.318	0.340	0.430
9265	0.764	0.324	0.333	0.433
9270	0.749	0.291	0.311	0.408
9275	0.750	0.326	0.324	0.433
9280	0.787	0.348	0.362	0.453
9285	0.794	0.359	0.372	0.462
9290	0.752	0.302	0.317	0.416
9295	0.754	0.257	0.301	0.383
9300	0.769	0.236	0.304	0.364
9305	0.794	0.352	0.370	0.457
9310	0.763	0.275	0.315	0.396
9315	0.771	0.307	0.333	0.421

9320	0.790	0.301	0.350	0.415
9325	0.730	0.199	0.263	0.339
9330	0.747	0.220	0.282	0.354
9335	0.747	0.262	0.299	0.387
9340	0.737	0.229	0.279	0.361
9345	0.789	0.305	0.349	0.419
9350	0.775	0.325	0.342	0.435
9355	0.776	0.340	0.349	0.446
9360	0.761	0.286	0.318	0.405
9365	0.756	0.259	0.303	0.384
9370	0.773	0.276	0.323	0.396
9375	0.756	0.203	0.279	0.338
9380	0.766	0.266	0.314	0.389
9385	0.821	0.433	0.427	0.521
9390	0.757	0.262	0.306	0.386
9395	0.759	0.264	0.308	0.388
9400	0.756	0.306	0.321	0.420
9405	0.789	0.351	0.364	0.455
9410	0.757	0.255	0.303	0.381
9415	0.782	0.308	0.343	0.422
9420	0.769	0.298	0.329	0.414
9425	0.731	0.187	0.259	0.329
9430	0.768	0.302	0.329	0.417
9435	0.733	0.224	0.276	0.358
9440	0.797	0.285	0.351	0.400
9445	0.726	0.209	0.266	0.347
9450	0.759	0.222	0.290	0.353
9455	0.764	0.304	0.327	0.419
9460	0.745	0.232	0.286	0.364
9465	0.776	0.274	0.326	0.394
9470	0.747	0.248	0.293	0.376
9475	0.759	0.257	0.305	0.382
9480	0.783	0.344	0.357	0.449
9485	0.754	0.257	0.301	0.383
9490	0.742	0.235	0.285	0.366
9495	0.775	0.292	0.331	0.409
9500	0.766	0.280	0.319	0.400
9505	0.761	0.280	0.315	0.400
9510	0.775	0.305	0.336	0.420
9515	0.771	0.280	0.324	0.400
9520	0.761	0.280	0.315	0.400
9525	0.775	0.305	0.336	0.420
9530	0.771	0.294	0.329	0.411

9535	0.763	0.268	0.312	0.391
9540	0.771	0.232	0.303	0.359
9545	0.738	0.238	0.284	0.368
9550	0.775	0.331	0.345	0.440
9555	0.775	0.331	0.345	0.440
9560	0.719	0.173	0.247	0.319
9565	0.768	0.302	0.329	0.417
9570	0.766	0.287	0.322	0.405
9575	0.766	0.273	0.317	0.395
9580	0.792	0.350	0.368	0.455
9585	0.757	0.255	0.303	0.381
9590	0.776	0.321	0.343	0.432
9595	0.764	0.298	0.324	0.414
9600	0.742	0.207	0.273	0.344
9605	0.792	0.310	0.355	0.423
9610	0.749	0.291	0.311	0.408
9615	0.735	0.184	0.259	0.326
9620	0.761	0.259	0.307	0.384
9625	0.783	0.263	0.328	0.384
9630	0.782	0.308	0.343	0.422
9635	0.773	0.355	0.350	0.456
9640	0.764	0.298	0.324	0.414
9645	0.752	0.302	0.317	0.416
9650	0.761	0.259	0.307	0.384
9655	0.747	0.275	0.304	0.397
9660	0.759	0.298	0.320	0.414
9665	0.742	0.221	0.279	0.355
9670	0.797	0.357	0.376	0.461
9675	0.761	0.273	0.313	0.395
9680	0.754	0.271	0.307	0.393
9685	0.752	0.248	0.297	0.376
9690	0.776	0.347	0.351	0.451
9695	0.776	0.334	0.347	0.442
9700	0.768	0.282	0.321	0.402
9705	0.757	0.282	0.313	0.402
9710	0.769	0.271	0.319	0.393
9715	0.752	0.241	0.294	0.370
9720	0.773	0.289	0.328	0.407
9725	0.778	0.310	0.341	0.423
9730	0.716	0.147	0.233	0.299
9735	0.747	0.213	0.279	0.348
9740	0.776	0.287	0.331	0.406
9745	0.780	0.312	0.343	0.425

9750	0.756	0.246	0.298	0.373
9755	0.738	0.210	0.272	0.346
9760	0.738	0.203	0.269	0.341
9765	0.768	0.309	0.331	0.422
9770	0.747	0.228	0.285	0.360
9775	0.763	0.248	0.304	0.374
9780	0.750	0.218	0.283	0.351
9785	0.747	0.228	0.285	0.360
9790	0.737	0.256	0.290	0.382
9795	0.775	0.298	0.333	0.414
9800	0.759	0.257	0.305	0.382
9805	0.750	0.232	0.289	0.363
9810	0.783	0.213	0.305	0.339
9815	0.768	0.269	0.316	0.391
9820	0.766	0.259	0.311	0.384
9825	0.764	0.284	0.319	0.404
9830	0.756	0.239	0.295	0.368
9835	0.771	0.287	0.326	0.405
9840	0.794	0.339	0.366	0.447
9845	0.754	0.215	0.284	0.349
9850	0.756	0.232	0.292	0.362
9855	0.759	0.257	0.305	0.382
9860	0.754	0.264	0.304	0.388
9865	0.802	0.353	0.381	0.457
9870	0.740	0.247	0.288	0.375
9875	0.768	0.296	0.326	0.412
9880	0.764	0.271	0.314	0.393
9885	0.754	0.298	0.316	0.413
9890	0.754	0.250	0.299	0.377
9895	0.768	0.262	0.313	0.385
9900	0.785	0.346	0.359	0.451
9905	0.737	0.263	0.293	0.387
9910	0.776	0.308	0.338	0.422
9915	0.778	0.283	0.331	0.402
9920	0.745	0.260	0.297	0.385
9925	0.754	0.304	0.319	0.418
9930	0.745	0.267	0.299	0.390
9935	0.742	0.235	0.285	0.366
9940	0.764	0.264	0.312	0.387
9945	0.782	0.348	0.356	0.452
9950	0.769	0.318	0.336	0.429
9955	0.780	0.306	0.341	0.420
9960	0.787	0.342	0.360	0.448

9965	0.769	0.250	0.310	0.376
9970	0.745	0.260	0.297	0.385
9975	0.745	0.253	0.294	0.380
9980	0.754	0.250	0.299	0.377
9985	0.782	0.308	0.343	0.422
9990	0.764	0.284	0.319	0.404
9995	0.763	0.335	0.335	0.441
10000	0.749	0.271	0.303	0.393

(3) Performance of IFS with random forest on the list yielded by LightGBM

Number of features	ACC	MCC	Precision	F1-measure
5	0.802	0.446	0.404	0.525
10	0.872	0.596	0.539	0.651
15	0.915	0.685	0.677	0.732
20	0.931	0.727	0.750	0.767
25	0.939	0.748	0.818	0.783
30	0.945	0.773	0.825	0.805
35	0.965	0.857	0.910	0.877
40	0.967	0.865	0.911	0.883
45	0.955	0.813	0.882	0.838
50	0.965	0.857	0.910	0.877
55	0.979	0.915	0.950	0.927
60	0.967	0.865	0.911	0.883
65	0.965	0.856	0.932	0.873
70	0.965	0.855	0.985	0.867
75	0.969	0.870	0.946	0.886
80	0.976	0.900	0.986	0.910
85	0.974	0.893	0.986	0.903
90	0.979	0.915	0.962	0.926
95	0.979	0.915	0.950	0.927
100	0.984	0.936	0.975	0.945
105	0.984	0.937	0.963	0.945
110	0.988	0.951	0.953	0.959
115	0.984	0.937	0.952	0.946
120	0.986	0.944	0.975	0.951
125	0.971	0.880	0.914	0.897
130	0.977	0.908	0.949	0.920
135	0.984	0.936	0.987	0.944
140	0.983	0.929	0.987	0.938
145	0.974	0.893	0.960	0.906
150	0.971	0.878	0.959	0.892
155	0.969	0.870	0.946	0.886
160	0.974	0.893	0.960	0.906

165	0.977	0.907	0.973	0.918
170	0.977	0.907	0.973	0.918
175	0.977	0.907	0.986	0.917
180	0.962	0.840	0.943	0.857
185	0.976	0.900	0.973	0.911
190	0.977	0.908	0.949	0.920
195	0.974	0.893	0.960	0.906
200	0.972	0.885	0.972	0.897
205	0.971	0.878	0.947	0.893
210	0.974	0.893	0.960	0.906
215	0.972	0.886	0.936	0.901
220	0.974	0.893	0.948	0.907
225	0.974	0.893	0.948	0.907
230	0.971	0.878	0.935	0.894
235	0.977	0.907	0.961	0.919
240	0.976	0.901	0.938	0.915
245	0.967	0.863	0.958	0.877
250	0.974	0.893	0.948	0.907
255	0.981	0.922	0.962	0.933
260	0.979	0.916	0.939	0.928
265	0.984	0.937	0.952	0.946
270	0.979	0.915	0.986	0.924
275	0.977	0.907	0.961	0.919
280	0.979	0.915	0.950	0.927
285	0.979	0.915	0.950	0.927
290	0.977	0.907	0.961	0.919
295	0.983	0.929	0.974	0.938
300	0.983	0.929	0.974	0.938
305	0.977	0.908	0.949	0.920
310	0.979	0.916	0.939	0.928
315	0.986	0.944	0.975	0.951
320	0.983	0.929	0.963	0.939
325	0.977	0.910	0.918	0.923
330	0.979	0.915	0.986	0.924
335	0.983	0.930	0.951	0.940
340	0.990	0.958	0.964	0.964
345	0.984	0.936	0.987	0.944
350	0.977	0.907	0.973	0.918
355	0.981	0.924	0.929	0.935
360	0.984	0.936	0.975	0.945
365	0.979	0.915	0.962	0.926
370	0.983	0.930	0.951	0.940
375	0.984	0.936	0.975	0.945

380	0.983	0.930	0.951	0.940
385	0.983	0.930	0.951	0.940
390	0.979	0.915	0.974	0.925
395	0.986	0.944	0.963	0.952
400	0.977	0.907	0.961	0.919
405	0.981	0.922	0.951	0.933
410	0.977	0.908	0.938	0.921
415	0.986	0.944	0.963	0.952
420	0.984	0.936	0.975	0.945
425	0.977	0.908	0.938	0.921
430	0.977	0.910	0.918	0.923
435	0.972	0.888	0.915	0.904
440	0.979	0.915	0.962	0.926
445	0.981	0.922	0.987	0.931
450	0.983	0.929	0.963	0.939
455	0.977	0.907	0.973	0.918
460	0.977	0.908	0.949	0.920
465	0.977	0.909	0.928	0.922
470	0.984	0.936	0.987	0.944
475	0.981	0.922	0.987	0.931
480	0.972	0.886	0.936	0.901
485	0.976	0.900	0.973	0.911
490	0.983	0.929	0.974	0.938
495	0.976	0.900	0.949	0.914
500	0.976	0.900	0.949	0.914
505	0.974	0.893	0.948	0.907
510	0.976	0.902	0.927	0.916
515	0.983	0.929	0.987	0.938
520	0.981	0.922	0.951	0.933
525	0.981	0.922	0.962	0.933
530	0.976	0.900	0.949	0.914
535	0.983	0.929	0.963	0.939
540	0.977	0.909	0.928	0.922
545	0.986	0.944	0.975	0.951
550	0.988	0.951	0.975	0.958
555	0.988	0.951	0.975	0.958
560	0.971	0.878	0.959	0.892
565	0.974	0.893	0.960	0.906
570	0.979	0.915	0.986	0.924
575	0.979	0.915	0.962	0.926
580	0.976	0.901	0.938	0.915
585	0.981	0.922	0.962	0.933
590	0.972	0.885	0.959	0.899

595	0.974	0.892	0.973	0.904
600	0.983	0.929	0.974	0.938
605	0.979	0.915	0.962	0.926
610	0.984	0.936	0.975	0.945
615	0.979	0.915	0.974	0.925
620	0.974	0.893	0.948	0.907
625	0.977	0.907	0.961	0.919
630	0.976	0.900	0.973	0.911
635	0.976	0.900	0.949	0.914
640	0.977	0.907	0.961	0.919
645	0.974	0.892	0.973	0.904
650	0.983	0.929	0.974	0.938
655	0.976	0.900	0.986	0.910
660	0.974	0.893	0.960	0.906
665	0.981	0.922	0.962	0.933
670	0.986	0.944	1.000	0.950
675	0.974	0.893	0.948	0.907
680	0.977	0.907	0.961	0.919
685	0.983	0.929	0.963	0.939
690	0.977	0.907	0.973	0.918
695	0.981	0.922	0.974	0.932
700	0.977	0.907	0.961	0.919
705	0.976	0.900	0.973	0.911
710	0.974	0.893	0.960	0.906
715	0.977	0.907	0.986	0.917
720	0.972	0.885	0.986	0.896
725	0.969	0.870	0.958	0.885
730	0.965	0.855	0.985	0.867
735	0.986	0.944	1.000	0.950
740	0.953	0.802	0.913	0.824
745	0.964	0.848	0.944	0.865
750	0.964	0.848	0.944	0.865
755	0.958	0.824	0.955	0.840
760	0.960	0.832	0.942	0.850
765	0.964	0.848	0.932	0.866
770	0.969	0.871	1.000	0.880
775	0.969	0.870	0.971	0.883
780	0.962	0.840	0.970	0.853
785	0.962	0.840	0.943	0.857
790	0.960	0.832	0.969	0.846
795	0.964	0.848	0.944	0.865
800	0.958	0.825	0.941	0.842
805	0.960	0.832	0.955	0.848

810	0.964	0.847	0.970	0.861
815	0.962	0.840	0.956	0.855
820	0.969	0.870	0.946	0.886
825	0.969	0.870	0.985	0.882
830	0.965	0.855	0.971	0.868
835	0.962	0.840	0.970	0.853
840	0.972	0.885	0.986	0.896
845	0.971	0.878	0.959	0.892
850	0.967	0.863	0.971	0.876
855	0.967	0.863	0.971	0.876
860	0.971	0.878	1.000	0.887
865	0.965	0.855	0.944	0.872
870	0.971	0.878	0.959	0.892
875	0.964	0.847	0.957	0.863
880	0.972	0.885	0.972	0.897
885	0.972	0.885	0.986	0.896
890	0.967	0.863	0.958	0.877
895	0.972	0.885	0.986	0.896
900	0.960	0.833	0.930	0.852
905	0.974	0.893	0.986	0.903
910	0.969	0.870	0.971	0.883
915	0.969	0.870	0.946	0.886
920	0.962	0.840	0.984	0.851
925	0.974	0.892	0.973	0.904
930	0.962	0.840	0.984	0.851
935	0.972	0.885	0.972	0.897
940	0.953	0.803	0.901	0.826
945	0.965	0.856	1.000	0.865
950	0.960	0.832	0.969	0.846
955	0.960	0.832	0.984	0.844
960	0.969	0.870	0.958	0.885
965	0.965	0.855	0.957	0.870
970	0.965	0.855	0.957	0.870
975	0.962	0.840	0.970	0.853
980	0.962	0.840	0.956	0.855
985	0.964	0.847	0.970	0.861
990	0.965	0.855	0.957	0.870
995	0.967	0.863	0.985	0.874
1000	0.969	0.870	0.958	0.885
1005	0.960	0.832	0.969	0.846
1010	0.964	0.847	0.970	0.861
1015	0.964	0.847	0.957	0.863
1020	0.965	0.855	0.985	0.867

1025	0.960	0.832	0.955	0.848
1030	0.965	0.855	0.985	0.867
1035	0.965	0.856	1.000	0.865
1040	0.962	0.840	0.956	0.855
1045	0.964	0.848	0.985	0.859
1050	0.962	0.840	0.931	0.859
1055	0.958	0.824	0.955	0.840
1060	0.960	0.832	0.955	0.848
1065	0.960	0.832	0.984	0.844
1070	0.962	0.840	0.956	0.855
1075	0.965	0.855	0.971	0.868
1080	0.969	0.870	0.985	0.882
1085	0.967	0.863	0.971	0.876
1090	0.960	0.832	0.984	0.844
1095	0.960	0.832	0.984	0.844
1100	0.962	0.840	0.956	0.855
1105	0.958	0.825	1.000	0.833
1110	0.958	0.824	0.955	0.840
1115	0.962	0.840	0.943	0.857
1120	0.958	0.824	0.955	0.840
1125	0.965	0.855	0.957	0.870
1130	0.967	0.863	0.985	0.874
1135	0.964	0.847	0.957	0.863
1140	0.965	0.855	0.985	0.867
1145	0.962	0.840	0.956	0.855
1150	0.972	0.885	0.959	0.899
1155	0.967	0.863	0.971	0.876
1160	0.962	0.840	0.984	0.851
1165	0.957	0.817	0.984	0.828
1170	0.960	0.832	0.955	0.848
1175	0.971	0.878	0.972	0.890
1180	0.971	0.878	0.959	0.892
1185	0.962	0.840	0.970	0.853
1190	0.969	0.870	0.971	0.883
1195	0.972	0.885	0.972	0.897
1200	0.969	0.870	0.985	0.882
1205	0.974	0.892	0.973	0.904
1210	0.962	0.840	0.970	0.853
1215	0.971	0.878	0.959	0.892
1220	0.967	0.863	0.958	0.877
1225	0.962	0.840	0.970	0.853
1230	0.972	0.885	0.986	0.896
1235	0.976	0.900	0.986	0.910

1240	0.974	0.892	0.973	0.904
1245	0.977	0.907	0.986	0.917
1250	0.972	0.885	0.972	0.897
1255	0.969	0.870	0.946	0.886
1260	0.971	0.878	0.959	0.892
1265	0.967	0.863	0.971	0.876
1270	0.965	0.855	0.944	0.872
1275	0.971	0.878	0.959	0.892
1280	0.972	0.885	0.959	0.899
1285	0.967	0.863	0.945	0.879
1290	0.971	0.878	0.972	0.890
1295	0.965	0.855	0.944	0.872
1300	0.967	0.863	0.945	0.879
1305	0.971	0.878	0.959	0.892
1310	0.969	0.870	0.946	0.886
1315	0.971	0.878	0.972	0.890
1320	0.960	0.833	0.930	0.852
1325	0.965	0.856	0.932	0.873
1330	0.965	0.856	0.932	0.873
1335	0.969	0.870	0.946	0.886
1340	0.960	0.833	0.930	0.852
1345	0.958	0.824	0.969	0.838
1350	0.967	0.863	0.985	0.874
1355	0.962	0.840	0.956	0.855
1360	0.965	0.855	0.971	0.868
1365	0.969	0.871	0.934	0.888
1370	0.955	0.810	0.914	0.831
1375	0.965	0.855	0.957	0.870
1380	0.962	0.841	0.919	0.861
1385	0.962	0.841	0.919	0.861
1390	0.974	0.893	0.960	0.906
1395	0.962	0.840	0.956	0.855
1400	0.962	0.841	0.919	0.861
1405	0.974	0.892	0.973	0.904
1410	0.965	0.855	0.944	0.872
1415	0.967	0.863	0.971	0.876
1420	0.976	0.900	0.973	0.911
1425	0.971	0.878	0.972	0.890
1430	0.967	0.863	0.958	0.877
1435	0.969	0.870	0.958	0.885
1440	0.971	0.878	0.972	0.890
1445	0.971	0.878	0.959	0.892
1450	0.976	0.900	0.973	0.911

1455	0.972	0.885	0.959	0.899
1460	0.960	0.832	0.969	0.846
1465	0.964	0.848	0.944	0.865
1470	0.965	0.855	0.957	0.870
1475	0.969	0.870	0.985	0.882
1480	0.964	0.848	0.944	0.865
1485	0.974	0.893	0.986	0.903
1490	0.972	0.885	0.972	0.897
1495	0.971	0.878	0.947	0.893
1500	0.971	0.878	0.959	0.892
1505	0.967	0.863	0.945	0.879
1510	0.972	0.885	1.000	0.895
1515	0.972	0.885	0.986	0.896
1520	0.971	0.878	0.959	0.892
1525	0.969	0.870	0.985	0.882
1530	0.967	0.863	0.933	0.881
1535	0.971	0.878	0.947	0.893
1540	0.972	0.885	0.959	0.899
1545	0.971	0.878	0.947	0.893
1550	0.976	0.900	0.973	0.911
1555	0.965	0.855	0.944	0.872
1560	0.972	0.885	0.947	0.900
1565	0.977	0.907	0.961	0.919
1570	0.967	0.863	0.945	0.879
1575	0.964	0.848	0.944	0.865
1580	0.974	0.893	0.948	0.907
1585	0.967	0.863	0.933	0.881
1590	0.972	0.885	0.986	0.896
1595	0.969	0.870	0.958	0.885
1600	0.965	0.855	0.944	0.872
1605	0.969	0.870	0.946	0.886
1610	0.981	0.922	1.000	0.930
1615	0.965	0.855	0.944	0.872
1620	0.976	0.900	0.973	0.911
1625	0.979	0.915	0.986	0.924
1630	0.969	0.870	0.946	0.886
1635	0.972	0.885	0.947	0.900
1640	0.974	0.893	1.000	0.902
1645	0.976	0.900	0.986	0.910
1650	0.974	0.892	0.973	0.904
1655	0.972	0.885	0.959	0.899
1660	0.972	0.885	0.972	0.897
1665	0.976	0.900	0.986	0.910

1670	0.981	0.922	1.000	0.930
1675	0.969	0.870	0.946	0.886
1680	0.965	0.856	0.932	0.873
1685	0.969	0.870	0.958	0.885
1690	0.972	0.885	0.959	0.899
1695	0.965	0.856	0.932	0.873
1700	0.972	0.885	0.959	0.899
1705	0.965	0.855	0.957	0.870
1710	0.965	0.855	0.944	0.872
1715	0.974	0.893	0.986	0.903
1720	0.964	0.848	0.944	0.865
1725	0.967	0.863	0.958	0.877
1730	0.969	0.870	0.958	0.885
1735	0.971	0.878	0.986	0.889
1740	0.969	0.870	0.946	0.886
1745	0.969	0.870	0.971	0.883
1750	0.974	0.893	0.960	0.906
1755	0.965	0.855	0.944	0.872
1760	0.967	0.863	0.958	0.877
1765	0.969	0.870	0.985	0.882
1770	0.971	0.878	0.959	0.892
1775	0.971	0.878	0.972	0.890
1780	0.967	0.863	0.985	0.874
1785	0.967	0.863	0.945	0.879
1790	0.976	0.900	1.000	0.909
1795	0.965	0.855	0.985	0.867
1800	0.974	0.893	0.960	0.906
1805	0.964	0.848	0.944	0.865
1810	0.971	0.878	0.935	0.894
1815	0.972	0.885	0.986	0.896
1820	0.969	0.870	0.958	0.885
1825	0.960	0.833	0.918	0.854
1830	0.962	0.840	0.943	0.857
1835	0.958	0.825	0.941	0.842
1840	0.964	0.847	0.970	0.861
1845	0.974	0.892	0.973	0.904
1850	0.965	0.855	0.971	0.868
1855	0.971	0.878	0.972	0.890
1860	0.969	0.870	0.958	0.885
1865	0.965	0.855	0.944	0.872
1870	0.971	0.878	0.959	0.892
1875	0.971	0.878	0.986	0.889
1880	0.974	0.893	0.986	0.903

1885	0.965	0.858	0.900	0.878
1890	0.964	0.848	0.985	0.859
1895	0.974	0.892	0.973	0.904
1900	0.962	0.840	0.970	0.853
1905	0.958	0.825	0.941	0.842
1910	0.969	0.870	0.971	0.883
1915	0.972	0.885	0.986	0.896
1920	0.964	0.848	0.944	0.865
1925	0.962	0.840	0.943	0.857
1930	0.972	0.885	1.000	0.895
1935	0.965	0.855	0.957	0.870
1940	0.964	0.847	0.970	0.861
1945	0.972	0.885	0.986	0.896
1950	0.969	0.870	0.985	0.882
1955	0.965	0.855	0.985	0.867
1960	0.971	0.878	0.947	0.893
1965	0.972	0.885	0.986	0.896
1970	0.971	0.878	0.986	0.889
1975	0.971	0.878	1.000	0.887
1980	0.960	0.833	0.930	0.852
1985	0.969	0.871	1.000	0.880
1990	0.965	0.855	0.957	0.870
1995	0.958	0.826	0.905	0.848
2000	0.962	0.840	0.956	0.855
2005	0.957	0.817	0.954	0.832
2010	0.962	0.840	0.970	0.853
2015	0.974	0.893	0.986	0.903
2020	0.958	0.826	0.905	0.848
2025	0.964	0.847	0.957	0.863
2030	0.960	0.833	0.918	0.854
2035	0.967	0.863	0.958	0.877
2040	0.972	0.885	0.959	0.899
2045	0.957	0.817	0.984	0.828
2050	0.965	0.855	0.957	0.870
2055	0.969	0.870	0.958	0.885
2060	0.962	0.840	0.943	0.857
2065	0.971	0.878	0.972	0.890
2070	0.958	0.824	0.955	0.840
2075	0.957	0.818	0.915	0.839
2080	0.971	0.878	0.986	0.889
2085	0.965	0.855	0.957	0.870
2090	0.967	0.863	0.985	0.874
2095	0.958	0.824	0.955	0.840

2100	0.964	0.847	0.970	0.861
2105	0.965	0.855	0.971	0.868
2110	0.960	0.833	0.930	0.852
2115	0.967	0.863	0.971	0.876
2120	0.964	0.848	0.944	0.865
2125	0.967	0.863	0.945	0.879
2130	0.964	0.847	0.970	0.861
2135	0.964	0.848	1.000	0.857
2140	0.965	0.855	0.944	0.872
2145	0.958	0.825	0.941	0.842
2150	0.962	0.840	0.984	0.851
2155	0.962	0.840	0.956	0.855
2160	0.958	0.826	0.917	0.846
2165	0.960	0.832	0.955	0.848
2170	0.969	0.870	0.958	0.885
2175	0.967	0.863	0.985	0.874
2180	0.962	0.840	0.984	0.851
2185	0.972	0.885	0.972	0.897
2190	0.960	0.832	0.969	0.846
2195	0.967	0.863	0.958	0.877
2200	0.969	0.870	0.946	0.886
2205	0.969	0.870	0.958	0.885
2210	0.958	0.825	0.929	0.844
2215	0.972	0.885	0.986	0.896
2220	0.967	0.863	0.945	0.879
2225	0.972	0.885	0.959	0.899
2230	0.962	0.840	0.984	0.851
2235	0.971	0.878	0.972	0.890
2240	0.967	0.863	0.958	0.877
2245	0.964	0.847	0.970	0.861
2250	0.964	0.847	0.970	0.861
2255	0.965	0.855	0.944	0.872
2260	0.957	0.817	0.940	0.834
2265	0.962	0.841	0.919	0.861
2270	0.969	0.870	0.958	0.885
2275	0.964	0.849	0.920	0.868
2280	0.971	0.878	0.986	0.889
2285	0.960	0.832	0.955	0.848
2290	0.957	0.817	0.940	0.834
2295	0.969	0.871	1.000	0.880
2300	0.967	0.863	0.971	0.876
2305	0.953	0.801	0.967	0.814
2310	0.960	0.832	0.955	0.848

2315	0.967	0.863	1.000	0.872
2320	0.967	0.863	0.971	0.876
2325	0.962	0.840	0.943	0.857
2330	0.964	0.848	0.944	0.865
2335	0.957	0.817	0.940	0.834
2340	0.969	0.870	0.958	0.885
2345	0.971	0.878	0.972	0.890
2350	0.955	0.809	0.939	0.827
2355	0.960	0.832	0.984	0.844
2360	0.960	0.832	0.942	0.850
2365	0.969	0.870	0.985	0.882
2370	0.964	0.848	0.944	0.865
2375	0.953	0.801	0.983	0.811
2380	0.964	0.847	0.957	0.863
2385	0.962	0.840	0.970	0.853
2390	0.960	0.832	0.942	0.850
2395	0.957	0.817	0.928	0.837
2400	0.962	0.840	0.970	0.853
2405	0.964	0.847	0.970	0.861
2410	0.962	0.840	0.956	0.855
2415	0.964	0.847	0.957	0.863
2420	0.948	0.777	0.950	0.792
2425	0.967	0.863	0.971	0.876
2430	0.962	0.840	0.943	0.857
2435	0.967	0.863	0.971	0.876
2440	0.960	0.833	0.930	0.852
2445	0.967	0.863	0.958	0.877
2450	0.964	0.848	0.985	0.859
2455	0.965	0.855	0.971	0.868
2460	0.957	0.817	0.954	0.832
2465	0.960	0.832	0.942	0.850
2470	0.950	0.785	0.966	0.797
2475	0.951	0.793	0.967	0.806
2480	0.965	0.855	0.971	0.868
2485	0.957	0.817	0.940	0.834
2490	0.953	0.801	0.952	0.816
2495	0.957	0.817	0.928	0.837
2500	0.962	0.840	0.956	0.855
2505	0.962	0.840	0.970	0.853
2510	0.962	0.840	0.943	0.857
2515	0.965	0.856	1.000	0.865
2520	0.951	0.793	0.924	0.813
2525	0.964	0.847	0.957	0.863

2530	0.962	0.840	0.970	0.853
2535	0.958	0.824	0.955	0.840
2540	0.957	0.817	0.968	0.830
2545	0.967	0.863	0.971	0.876
2550	0.965	0.855	0.985	0.867
2555	0.967	0.863	0.971	0.876
2560	0.967	0.863	0.958	0.877
2565	0.962	0.840	0.931	0.859
2570	0.953	0.801	0.967	0.814
2575	0.960	0.833	0.918	0.854
2580	0.960	0.832	0.984	0.844
2585	0.946	0.772	0.884	0.797
2590	0.958	0.825	0.929	0.844
2595	0.965	0.855	0.957	0.870
2600	0.969	0.871	1.000	0.880
2605	0.958	0.824	0.955	0.840
2610	0.969	0.871	1.000	0.880
2615	0.955	0.809	0.968	0.822
2620	0.953	0.801	0.938	0.819
2625	0.964	0.848	0.944	0.865
2630	0.960	0.833	0.930	0.852
2635	0.958	0.825	0.984	0.836
2640	0.950	0.785	0.951	0.800
2645	0.964	0.848	0.944	0.865
2650	0.958	0.824	0.969	0.838
2655	0.955	0.809	0.939	0.827
2660	0.960	0.832	0.969	0.846
2665	0.957	0.817	0.984	0.828
2670	0.960	0.832	0.942	0.850
2675	0.965	0.855	0.985	0.867
2680	0.964	0.849	0.920	0.868
2685	0.958	0.825	0.941	0.842
2690	0.964	0.847	0.970	0.861
2695	0.960	0.832	0.984	0.844
2700	0.960	0.832	0.969	0.846
2705	0.953	0.801	0.938	0.819
2710	0.955	0.809	0.968	0.822
2715	0.958	0.825	0.929	0.844
2720	0.955	0.811	0.903	0.833
2725	0.957	0.817	0.928	0.837
2730	0.960	0.832	0.955	0.848
2735	0.960	0.832	0.942	0.850
2740	0.948	0.777	0.922	0.797

2745	0.962	0.840	0.931	0.859
2750	0.960	0.832	0.969	0.846
2755	0.958	0.825	0.941	0.842
2760	0.964	0.848	0.985	0.859
2765	0.974	0.893	0.986	0.903
2770	0.972	0.885	1.000	0.895
2775	0.957	0.817	0.928	0.837
2780	0.960	0.832	0.969	0.846
2785	0.964	0.847	0.970	0.861
2790	0.957	0.817	0.954	0.832
2795	0.969	0.870	0.985	0.882
2800	0.964	0.847	0.957	0.863
2805	0.958	0.824	0.955	0.840
2810	0.960	0.832	0.969	0.846
2815	0.964	0.847	0.957	0.863
2820	0.958	0.826	0.917	0.846
2825	0.948	0.777	0.935	0.795
2830	0.955	0.809	0.968	0.822
2835	0.951	0.793	0.938	0.811
2840	0.958	0.825	0.929	0.844
2845	0.955	0.809	0.939	0.827
2850	0.958	0.824	0.969	0.838
2855	0.955	0.809	0.953	0.824
2860	0.953	0.801	0.925	0.821
2865	0.964	0.848	0.944	0.865
2870	0.967	0.863	0.958	0.877
2875	0.958	0.825	0.941	0.842
2880	0.964	0.848	1.000	0.857
2885	0.955	0.809	0.968	0.822
2890	0.965	0.856	1.000	0.865
2895	0.946	0.769	0.934	0.786
2900	0.960	0.833	0.930	0.852
2905	0.951	0.793	0.938	0.811
2910	0.967	0.863	0.971	0.876
2915	0.962	0.840	0.970	0.853
2920	0.953	0.801	0.952	0.816
2925	0.969	0.870	0.971	0.883
2930	0.957	0.817	0.928	0.837
2935	0.955	0.809	0.953	0.824
2940	0.962	0.840	0.931	0.859
2945	0.960	0.832	0.942	0.850
2950	0.964	0.848	0.944	0.865
2955	0.974	0.893	1.000	0.902

2960	0.958	0.824	0.969	0.838
2965	0.955	0.809	0.953	0.824
2970	0.955	0.809	0.926	0.829
2975	0.972	0.885	0.972	0.897
2980	0.962	0.840	0.970	0.853
2985	0.960	0.832	0.969	0.846
2990	0.964	0.847	0.970	0.861
2995	0.958	0.824	0.955	0.840
3000	0.960	0.832	0.969	0.846
3005	0.955	0.809	0.953	0.824
3010	0.951	0.793	0.967	0.806
3015	0.962	0.840	0.984	0.851
3020	0.948	0.777	0.935	0.795
3025	0.958	0.825	0.941	0.842
3030	0.960	0.832	0.955	0.848
3035	0.950	0.786	0.910	0.808
3040	0.965	0.855	0.985	0.867
3045	0.958	0.824	0.969	0.838
3050	0.960	0.832	0.955	0.848
3055	0.967	0.863	0.971	0.876
3060	0.965	0.855	0.971	0.868
3065	0.957	0.817	0.940	0.834
3070	0.958	0.824	0.955	0.840
3075	0.960	0.832	0.969	0.846
3080	0.957	0.818	0.915	0.839
3085	0.955	0.809	0.926	0.829
3090	0.971	0.878	0.972	0.890
3095	0.958	0.825	0.941	0.842
3100	0.965	0.855	0.957	0.870
3105	0.964	0.847	0.970	0.861
3110	0.958	0.824	0.969	0.838
3115	0.957	0.817	0.954	0.832
3120	0.960	0.832	0.969	0.846
3125	0.960	0.832	0.969	0.846
3130	0.958	0.824	0.969	0.838
3135	0.958	0.824	0.955	0.840
3140	0.955	0.809	0.968	0.822
3145	0.951	0.795	0.900	0.818
3150	0.957	0.817	0.968	0.830
3155	0.960	0.832	0.955	0.848
3160	0.964	0.847	0.957	0.863
3165	0.960	0.832	0.969	0.846
3170	0.957	0.818	0.915	0.839

3175	0.958	0.824	0.955	0.840
3180	0.965	0.855	0.985	0.867
3185	0.965	0.855	0.944	0.872
3190	0.957	0.817	0.928	0.837
3195	0.960	0.832	0.969	0.846
3200	0.969	0.870	0.971	0.883
3205	0.962	0.840	0.956	0.855
3210	0.957	0.817	0.928	0.837
3215	0.962	0.840	0.956	0.855
3220	0.955	0.809	0.939	0.827
3225	0.955	0.809	0.953	0.824
3230	0.953	0.801	0.925	0.821
3235	0.960	0.832	0.984	0.844
3240	0.951	0.795	0.900	0.818
3245	0.962	0.840	0.956	0.855
3250	0.951	0.793	0.952	0.808
3255	0.964	0.847	0.970	0.861
3260	0.964	0.848	0.985	0.859
3265	0.957	0.817	0.954	0.832
3270	0.955	0.809	0.939	0.827
3275	0.964	0.847	0.957	0.863
3280	0.962	0.840	0.970	0.853
3285	0.957	0.817	0.954	0.832
3290	0.957	0.817	0.984	0.828
3295	0.957	0.817	0.968	0.830
3300	0.960	0.832	0.984	0.844
3305	0.953	0.801	0.952	0.816
3310	0.951	0.793	0.952	0.808
3315	0.964	0.847	0.970	0.861
3320	0.960	0.832	0.955	0.848
3325	0.955	0.809	0.983	0.819
3330	0.953	0.801	0.952	0.816
3335	0.953	0.801	0.952	0.816
3340	0.962	0.840	0.956	0.855
3345	0.965	0.855	0.971	0.868
3350	0.953	0.801	0.952	0.816
3355	0.958	0.824	0.969	0.838
3360	0.953	0.801	0.925	0.821
3365	0.958	0.824	0.969	0.838
3370	0.962	0.840	0.956	0.855
3375	0.955	0.810	1.000	0.817
3380	0.962	0.840	0.970	0.853
3385	0.964	0.848	0.985	0.859

3390	0.964	0.847	0.957	0.863
3395	0.962	0.840	0.943	0.857
3400	0.965	0.856	1.000	0.865
3405	0.960	0.832	0.969	0.846
3410	0.951	0.793	0.952	0.808
3415	0.960	0.832	0.984	0.844
3420	0.958	0.825	0.984	0.836
3425	0.958	0.824	0.969	0.838
3430	0.960	0.832	0.955	0.848
3435	0.946	0.769	0.921	0.789
3440	0.953	0.801	0.925	0.821
3445	0.958	0.825	0.941	0.842
3450	0.951	0.794	0.983	0.803
3455	0.957	0.817	0.940	0.834
3460	0.964	0.848	0.985	0.859
3465	0.957	0.817	0.940	0.834
3470	0.957	0.817	0.954	0.832
3475	0.965	0.855	0.971	0.868
3480	0.941	0.745	0.917	0.764
3485	0.958	0.824	0.955	0.840
3490	0.948	0.777	0.922	0.797
3495	0.965	0.855	0.971	0.868
3500	0.953	0.801	0.983	0.811
3505	0.962	0.840	0.956	0.855
3510	0.953	0.801	0.967	0.814
3515	0.955	0.809	0.953	0.824
3520	0.960	0.832	0.984	0.844
3525	0.958	0.826	0.917	0.846
3530	0.953	0.801	0.938	0.819
3535	0.957	0.817	0.928	0.837
3540	0.953	0.801	0.938	0.819
3545	0.965	0.855	0.971	0.868
3550	0.950	0.785	0.951	0.800
3555	0.951	0.793	0.952	0.808
3560	0.951	0.794	0.912	0.816
3565	0.955	0.809	0.968	0.822
3570	0.965	0.855	0.985	0.867
3575	0.960	0.832	0.942	0.850
3580	0.960	0.832	0.955	0.848
3585	0.957	0.817	0.984	0.828
3590	0.941	0.745	0.917	0.764
3595	0.962	0.840	0.970	0.853
3600	0.960	0.833	1.000	0.841

3605	0.960	0.832	0.969	0.846
3610	0.960	0.832	0.955	0.848
3615	0.953	0.801	0.967	0.814
3620	0.962	0.840	0.970	0.853
3625	0.965	0.855	0.985	0.867
3630	0.962	0.840	0.970	0.853
3635	0.948	0.777	0.950	0.792
3640	0.951	0.793	0.924	0.813
3645	0.955	0.809	0.953	0.824
3650	0.945	0.761	0.933	0.778
3655	0.960	0.832	0.984	0.844
3660	0.955	0.809	0.968	0.822
3665	0.953	0.801	0.952	0.816
3670	0.960	0.832	0.955	0.848
3675	0.958	0.824	0.969	0.838
3680	0.948	0.777	0.922	0.797
3685	0.950	0.785	0.966	0.797
3690	0.957	0.817	0.968	0.830
3695	0.953	0.801	0.952	0.816
3700	0.948	0.777	0.935	0.795
3705	0.957	0.817	0.940	0.834
3710	0.958	0.825	1.000	0.833
3715	0.964	0.847	0.970	0.861
3720	0.948	0.777	0.950	0.792
3725	0.962	0.840	0.956	0.855
3730	0.957	0.817	0.954	0.832
3735	0.955	0.809	0.983	0.819
3740	0.965	0.856	1.000	0.865
3745	0.946	0.769	0.949	0.783
3750	0.962	0.840	0.956	0.855
3755	0.957	0.817	0.954	0.832
3760	0.962	0.840	0.970	0.853
3765	0.948	0.777	0.966	0.789
3770	0.955	0.810	0.914	0.831
3775	0.955	0.809	0.939	0.827
3780	0.948	0.777	0.922	0.797
3785	0.955	0.809	0.968	0.822
3790	0.955	0.809	0.939	0.827
3795	0.958	0.824	0.969	0.838
3800	0.965	0.855	0.971	0.868
3805	0.958	0.825	1.000	0.833
3810	0.960	0.832	0.969	0.846
3815	0.964	0.848	0.985	0.859

3820	0.958	0.825	0.984	0.836
3825	0.964	0.847	0.957	0.863
3830	0.957	0.817	0.928	0.837
3835	0.964	0.847	0.957	0.863
3840	0.955	0.809	0.968	0.822
3845	0.953	0.801	0.983	0.811
3850	0.953	0.801	0.967	0.814
3855	0.960	0.832	0.969	0.846
3860	0.953	0.801	0.925	0.821
3865	0.953	0.801	0.938	0.819
3870	0.951	0.794	0.983	0.803
3875	0.957	0.817	0.954	0.832
3880	0.955	0.809	0.983	0.819
3885	0.955	0.809	0.953	0.824
3890	0.957	0.817	0.984	0.828
3895	0.953	0.801	0.967	0.814
3900	0.953	0.801	0.983	0.811
3905	0.955	0.809	0.968	0.822
3910	0.958	0.825	1.000	0.833
3915	0.960	0.832	0.969	0.846
3920	0.951	0.793	0.938	0.811
3925	0.955	0.809	0.983	0.819
3930	0.960	0.833	1.000	0.841
3935	0.938	0.728	0.944	0.739
3940	0.957	0.817	0.954	0.832
3945	0.951	0.793	0.967	0.806
3950	0.948	0.777	0.935	0.795
3955	0.953	0.801	0.952	0.816
3960	0.957	0.817	0.940	0.834
3965	0.958	0.825	1.000	0.833
3970	0.951	0.793	0.967	0.806
3975	0.962	0.840	0.970	0.853
3980	0.955	0.809	0.968	0.822
3985	0.958	0.824	0.955	0.840
3990	0.957	0.817	0.954	0.832
3995	0.951	0.793	0.938	0.811
4000	0.957	0.817	0.984	0.828
4005	0.948	0.777	0.966	0.789
4010	0.960	0.832	0.942	0.850
4015	0.964	0.848	1.000	0.857
4020	0.953	0.801	0.938	0.819
4025	0.951	0.793	0.967	0.806
4030	0.953	0.801	0.952	0.816

4035	0.948	0.777	0.966	0.789
4040	0.962	0.840	0.984	0.851
4045	0.948	0.777	0.950	0.792
4050	0.946	0.769	0.934	0.786
4055	0.948	0.777	0.966	0.789
4060	0.951	0.793	0.924	0.813
4065	0.953	0.801	0.938	0.819
4070	0.950	0.785	0.966	0.797
4075	0.955	0.809	0.983	0.819
4080	0.953	0.801	0.952	0.816
4085	0.955	0.809	0.953	0.824
4090	0.957	0.818	1.000	0.825
4095	0.946	0.769	0.949	0.783
4100	0.950	0.785	0.966	0.797
4105	0.964	0.848	0.985	0.859
4110	0.958	0.824	0.969	0.838
4115	0.960	0.833	1.000	0.841
4120	0.948	0.777	0.966	0.789
4125	0.957	0.817	0.984	0.828
4130	0.953	0.801	0.967	0.814
4135	0.958	0.825	1.000	0.833
4140	0.955	0.809	0.968	0.822
4145	0.951	0.793	0.967	0.806
4150	0.955	0.809	0.983	0.819
4155	0.951	0.793	0.967	0.806
4160	0.955	0.810	1.000	0.817
4165	0.964	0.847	0.970	0.861
4170	0.951	0.793	0.938	0.811
4175	0.955	0.809	0.953	0.824
4180	0.951	0.794	1.000	0.800
4185	0.960	0.832	0.984	0.844
4190	0.943	0.753	0.964	0.763
4195	0.958	0.825	0.984	0.836
4200	0.964	0.848	1.000	0.857
4205	0.953	0.801	0.938	0.819
4210	0.958	0.824	0.969	0.838
4215	0.950	0.785	0.966	0.797
4220	0.960	0.832	0.984	0.844
4225	0.957	0.817	0.968	0.830
4230	0.958	0.824	0.969	0.838
4235	0.962	0.840	0.943	0.857
4240	0.951	0.793	0.967	0.806
4245	0.964	0.848	0.985	0.859

4250	0.958	0.824	0.955	0.840
4255	0.955	0.809	0.968	0.822
4260	0.955	0.809	0.953	0.824
4265	0.964	0.848	0.985	0.859
4270	0.957	0.817	0.984	0.828
4275	0.962	0.841	1.000	0.849
4280	0.964	0.848	1.000	0.857
4285	0.955	0.809	0.968	0.822
4290	0.957	0.817	0.968	0.830
4295	0.941	0.745	0.917	0.764
4300	0.951	0.793	0.967	0.806
4305	0.941	0.745	0.963	0.754
4310	0.953	0.801	0.967	0.814
4315	0.955	0.809	0.968	0.822
4320	0.948	0.777	0.966	0.789
4325	0.945	0.761	0.919	0.781
4330	0.953	0.801	0.967	0.814
4335	0.953	0.801	0.938	0.819
4340	0.955	0.809	0.983	0.819
4345	0.957	0.817	0.984	0.828
4350	0.951	0.793	0.952	0.808
4355	0.948	0.778	0.982	0.786
4360	0.958	0.825	0.984	0.836
4365	0.948	0.777	0.966	0.789
4370	0.953	0.801	0.967	0.814
4375	0.948	0.777	0.950	0.792
4380	0.951	0.794	0.983	0.803
4385	0.957	0.818	1.000	0.825
4390	0.948	0.777	0.950	0.792
4395	0.958	0.825	0.984	0.836
4400	0.945	0.761	0.964	0.771
4405	0.948	0.777	0.966	0.789
4410	0.948	0.778	0.982	0.786
4415	0.950	0.786	0.982	0.794
4420	0.950	0.786	0.982	0.794
4425	0.957	0.817	0.984	0.828
4430	0.950	0.785	0.966	0.797
4435	0.946	0.770	0.982	0.777
4440	0.957	0.817	0.984	0.828
4445	0.955	0.809	0.983	0.819
4450	0.953	0.801	0.983	0.811
4455	0.958	0.825	1.000	0.833
4460	0.946	0.769	0.949	0.783

4465	0.962	0.841	1.000	0.849
4470	0.941	0.744	0.931	0.761
4475	0.960	0.832	0.942	0.850
4480	0.953	0.801	0.967	0.814
4485	0.951	0.793	0.952	0.808
4490	0.960	0.832	0.984	0.844
4495	0.946	0.770	0.982	0.777
4500	0.955	0.809	0.983	0.819
4505	0.953	0.801	0.983	0.811
4510	0.951	0.794	0.983	0.803
4515	0.958	0.825	0.984	0.836
4520	0.957	0.817	0.954	0.832
4525	0.962	0.840	0.984	0.851
4530	0.943	0.753	0.964	0.763
4535	0.951	0.793	0.967	0.806
4540	0.950	0.786	1.000	0.791
4545	0.951	0.794	0.983	0.803
4550	0.948	0.778	0.982	0.786
4555	0.951	0.793	0.967	0.806
4560	0.951	0.793	0.967	0.806
4565	0.951	0.793	0.967	0.806
4570	0.960	0.833	1.000	0.841
4575	0.955	0.809	0.939	0.827
4580	0.958	0.824	0.969	0.838
4585	0.957	0.817	0.984	0.828
4590	0.953	0.801	0.983	0.811
4595	0.955	0.809	0.968	0.822
4600	0.953	0.801	0.967	0.814
4605	0.967	0.863	1.000	0.872
4610	0.960	0.833	1.000	0.841
4615	0.943	0.753	0.932	0.769
4620	0.950	0.785	0.966	0.797
4625	0.950	0.785	0.951	0.800
4630	0.964	0.848	0.985	0.859
4635	0.962	0.840	0.970	0.853
4640	0.951	0.793	0.952	0.808
4645	0.946	0.769	0.965	0.780
4650	0.948	0.777	0.966	0.789
4655	0.955	0.809	0.953	0.824
4660	0.955	0.809	0.968	0.822
4665	0.948	0.777	0.966	0.789
4670	0.951	0.794	0.983	0.803
4675	0.958	0.824	0.955	0.840

4680	0.946	0.769	0.965	0.780
4685	0.946	0.770	0.982	0.777
4690	0.957	0.817	0.954	0.832
4695	0.946	0.770	0.982	0.777
4700	0.955	0.809	0.953	0.824
4705	0.958	0.825	0.984	0.836
4710	0.953	0.801	0.967	0.814
4715	0.958	0.825	0.984	0.836
4720	0.951	0.793	0.952	0.808
4725	0.953	0.801	0.983	0.811
4730	0.950	0.786	0.982	0.794
4735	0.951	0.793	0.967	0.806
4740	0.948	0.778	0.982	0.786
4745	0.946	0.769	0.934	0.786
4750	0.946	0.769	0.965	0.780
4755	0.948	0.777	0.950	0.792
4760	0.946	0.769	0.965	0.780
4765	0.945	0.761	0.964	0.771
4770	0.967	0.863	1.000	0.872
4775	0.950	0.785	0.966	0.797
4780	0.960	0.833	1.000	0.841
4785	0.948	0.777	0.935	0.795
4790	0.948	0.777	0.935	0.795
4795	0.941	0.745	0.963	0.754
4800	0.955	0.809	0.968	0.822
4805	0.951	0.793	0.967	0.806
4810	0.946	0.769	0.949	0.783
4815	0.945	0.762	0.981	0.768
4820	0.958	0.825	1.000	0.833
4825	0.946	0.769	0.965	0.780
4830	0.945	0.762	0.981	0.768
4835	0.951	0.793	0.952	0.808
4840	0.951	0.794	0.983	0.803
4845	0.948	0.777	0.966	0.789
4850	0.957	0.817	0.984	0.828
4855	0.950	0.785	0.966	0.797
4860	0.943	0.753	0.932	0.769
4865	0.955	0.809	0.939	0.827
4870	0.946	0.770	0.982	0.777
4875	0.953	0.801	0.952	0.816
4880	0.957	0.817	0.954	0.832
4885	0.950	0.785	0.937	0.803
4890	0.950	0.785	0.923	0.805

4895	0.943	0.753	0.932	0.769
4900	0.953	0.801	0.967	0.814
4905	0.948	0.777	0.950	0.792
4910	0.950	0.785	0.951	0.800
4915	0.945	0.762	0.906	0.784
4920	0.962	0.840	0.970	0.853
4925	0.955	0.810	1.000	0.817
4930	0.960	0.833	1.000	0.841
4935	0.955	0.809	0.968	0.822
4940	0.945	0.761	0.948	0.775
4945	0.957	0.817	0.954	0.832
4950	0.962	0.841	1.000	0.849
4955	0.950	0.786	0.982	0.794
4960	0.943	0.753	0.947	0.766
4965	0.955	0.809	0.983	0.819
4970	0.950	0.786	0.982	0.794
4975	0.948	0.777	0.950	0.792
4980	0.948	0.777	0.950	0.792
4985	0.945	0.762	0.981	0.768
4990	0.948	0.778	1.000	0.783
4995	0.950	0.785	0.951	0.800
5000	0.957	0.817	0.968	0.830
5005	0.958	0.825	1.000	0.833
5010	0.945	0.762	1.000	0.765
5015	0.945	0.761	0.948	0.775
5020	0.957	0.817	0.984	0.828
5025	0.936	0.720	0.912	0.738
5030	0.957	0.817	0.984	0.828
5035	0.950	0.785	0.923	0.805
5040	0.945	0.761	0.933	0.778
5045	0.946	0.770	0.982	0.777
5050	0.950	0.785	0.951	0.800
5055	0.953	0.801	0.938	0.819
5060	0.953	0.801	0.983	0.811
5065	0.951	0.793	0.967	0.806
5070	0.951	0.794	0.912	0.816
5075	0.948	0.777	0.966	0.789
5080	0.955	0.809	0.968	0.822
5085	0.946	0.769	0.965	0.780
5090	0.951	0.793	0.952	0.808
5095	0.943	0.753	0.964	0.763
5100	0.950	0.785	0.966	0.797
5105	0.946	0.769	0.965	0.780

5110	0.946	0.770	0.982	0.777
5115	0.951	0.793	0.938	0.811
5120	0.953	0.802	1.000	0.809
5125	0.955	0.810	1.000	0.817
5130	0.962	0.840	0.956	0.855
5135	0.945	0.761	0.933	0.778
5140	0.958	0.825	0.984	0.836
5145	0.945	0.761	0.948	0.775
5150	0.948	0.778	1.000	0.783
5155	0.946	0.769	0.965	0.780
5160	0.955	0.809	0.968	0.822
5165	0.948	0.777	0.966	0.789
5170	0.955	0.809	0.968	0.822
5175	0.955	0.810	1.000	0.817
5180	0.958	0.825	0.984	0.836
5185	0.939	0.737	0.915	0.755
5190	0.951	0.793	0.938	0.811
5195	0.946	0.769	0.965	0.780
5200	0.943	0.753	0.981	0.759
5205	0.945	0.761	0.964	0.771
5210	0.953	0.801	0.967	0.814
5215	0.957	0.817	0.954	0.832
5220	0.955	0.809	0.983	0.819
5225	0.948	0.777	0.935	0.795
5230	0.948	0.778	0.982	0.786
5235	0.951	0.793	0.967	0.806
5240	0.950	0.786	0.982	0.794
5245	0.955	0.809	0.968	0.822
5250	0.941	0.745	0.917	0.764
5255	0.950	0.785	0.951	0.800
5260	0.955	0.809	0.983	0.819
5265	0.945	0.761	0.964	0.771
5270	0.951	0.793	0.967	0.806
5275	0.945	0.762	1.000	0.765
5280	0.945	0.761	0.948	0.775
5285	0.946	0.769	0.965	0.780
5290	0.957	0.817	0.968	0.830
5295	0.945	0.761	0.964	0.771
5300	0.953	0.801	0.952	0.816
5305	0.957	0.817	0.984	0.828
5310	0.945	0.761	0.933	0.778
5315	0.955	0.810	1.000	0.817
5320	0.948	0.778	0.909	0.800

5325	0.957	0.817	0.984	0.828
5330	0.951	0.793	0.967	0.806
5335	0.953	0.801	0.983	0.811
5340	0.955	0.809	0.983	0.819
5345	0.948	0.777	0.966	0.789
5350	0.953	0.801	0.938	0.819
5355	0.953	0.801	0.952	0.816
5360	0.946	0.769	0.965	0.780
5365	0.945	0.762	0.981	0.768
5370	0.936	0.719	0.927	0.734
5375	0.953	0.801	0.967	0.814
5380	0.953	0.801	0.967	0.814
5385	0.958	0.825	0.984	0.836
5390	0.950	0.786	0.982	0.794
5395	0.951	0.793	0.967	0.806
5400	0.953	0.801	0.952	0.816
5405	0.953	0.801	0.983	0.811
5410	0.948	0.777	0.966	0.789
5415	0.955	0.810	1.000	0.817
5420	0.946	0.769	0.965	0.780
5425	0.948	0.777	0.950	0.792
5430	0.967	0.863	0.985	0.874
5435	0.943	0.753	0.947	0.766
5440	0.953	0.801	0.952	0.816
5445	0.950	0.786	0.982	0.794
5450	0.945	0.762	0.981	0.768
5455	0.939	0.736	0.962	0.745
5460	0.948	0.777	0.966	0.789
5465	0.960	0.832	0.969	0.846
5470	0.960	0.832	0.984	0.844
5475	0.951	0.794	0.983	0.803
5480	0.948	0.778	0.982	0.786
5485	0.951	0.793	0.967	0.806
5490	0.941	0.744	0.946	0.757
5495	0.951	0.794	1.000	0.800
5500	0.943	0.753	0.964	0.763
5505	0.951	0.794	0.983	0.803
5510	0.957	0.817	0.984	0.828
5515	0.943	0.753	0.964	0.763
5520	0.945	0.762	1.000	0.765
5525	0.948	0.777	0.922	0.797
5530	0.962	0.840	0.956	0.855
5535	0.958	0.825	1.000	0.833

5540	0.945	0.761	0.964	0.771
5545	0.943	0.753	0.981	0.759
5550	0.948	0.777	0.966	0.789
5555	0.948	0.778	0.982	0.786
5560	0.946	0.770	0.982	0.777
5565	0.955	0.809	0.968	0.822
5570	0.946	0.769	0.965	0.780
5575	0.950	0.786	0.982	0.794
5580	0.950	0.785	0.966	0.797
5585	0.948	0.777	0.966	0.789
5590	0.951	0.794	0.983	0.803
5595	0.943	0.753	0.964	0.763
5600	0.958	0.824	0.969	0.838
5605	0.938	0.728	0.962	0.735
5610	0.950	0.785	0.966	0.797
5615	0.953	0.801	0.983	0.811
5620	0.948	0.777	0.966	0.789
5625	0.950	0.785	0.951	0.800
5630	0.945	0.762	0.981	0.768
5635	0.955	0.809	0.968	0.822
5640	0.939	0.736	0.962	0.745
5645	0.946	0.769	0.965	0.780
5650	0.950	0.785	0.966	0.797
5655	0.943	0.753	0.932	0.769
5660	0.946	0.769	0.949	0.783
5665	0.932	0.703	0.895	0.723
5670	0.943	0.753	0.932	0.769
5675	0.941	0.744	0.931	0.761
5680	0.951	0.794	0.983	0.803
5685	0.950	0.785	0.951	0.800
5690	0.943	0.753	0.964	0.763
5695	0.943	0.753	0.932	0.769
5700	0.958	0.825	1.000	0.833
5705	0.951	0.793	0.967	0.806
5710	0.946	0.770	0.982	0.777
5715	0.948	0.777	0.966	0.789
5720	0.946	0.770	0.982	0.777
5725	0.948	0.777	0.966	0.789
5730	0.951	0.794	0.983	0.803
5735	0.946	0.769	0.921	0.789
5740	0.943	0.753	0.932	0.769
5745	0.941	0.744	0.931	0.761
5750	0.946	0.769	0.949	0.783

5755	0.945	0.761	0.948	0.775
5760	0.950	0.786	0.982	0.794
5765	0.955	0.809	0.953	0.824
5770	0.946	0.769	0.965	0.780
5775	0.950	0.785	0.966	0.797
5780	0.946	0.769	0.934	0.786
5785	0.955	0.809	0.968	0.822
5790	0.965	0.856	1.000	0.865
5795	0.946	0.769	0.921	0.789
5800	0.948	0.777	0.966	0.789
5805	0.953	0.801	0.983	0.811
5810	0.941	0.745	0.963	0.754
5815	0.946	0.769	0.965	0.780
5820	0.958	0.825	1.000	0.833
5825	0.951	0.793	0.938	0.811
5830	0.948	0.777	0.950	0.792
5835	0.957	0.817	0.968	0.830
5840	0.948	0.778	0.982	0.786
5845	0.938	0.728	0.929	0.743
5850	0.945	0.761	0.933	0.778
5855	0.948	0.778	0.982	0.786
5860	0.945	0.761	0.964	0.771
5865	0.957	0.818	1.000	0.825
5870	0.950	0.786	0.982	0.794
5875	0.941	0.745	0.963	0.754
5880	0.953	0.801	0.983	0.811
5885	0.951	0.793	0.967	0.806
5890	0.945	0.761	0.933	0.778
5895	0.943	0.753	0.964	0.763
5900	0.955	0.810	1.000	0.817
5905	0.950	0.785	0.951	0.800
5910	0.945	0.761	0.933	0.778
5915	0.953	0.801	0.952	0.816
5920	0.951	0.794	0.983	0.803
5925	0.948	0.777	0.966	0.789
5930	0.955	0.809	0.983	0.819
5935	0.948	0.777	0.950	0.792
5940	0.953	0.802	1.000	0.809
5945	0.941	0.745	0.917	0.764
5950	0.948	0.777	0.966	0.789
5955	0.946	0.769	0.934	0.786
5960	0.958	0.824	0.969	0.838
5965	0.955	0.809	0.983	0.819

5970	0.948	0.777	0.966	0.789
5975	0.945	0.761	0.933	0.778
5980	0.948	0.777	0.966	0.789
5985	0.951	0.794	0.983	0.803
5990	0.953	0.801	0.952	0.816
5995	0.958	0.825	0.984	0.836
6000	0.950	0.786	1.000	0.791
6005	0.957	0.817	0.968	0.830
6010	0.948	0.778	0.982	0.786
6015	0.951	0.793	0.967	0.806
6020	0.950	0.786	0.982	0.794
6025	0.945	0.762	0.981	0.768
6030	0.943	0.753	0.981	0.759
6035	0.939	0.736	0.962	0.745
6040	0.946	0.770	0.982	0.777
6045	0.950	0.786	0.982	0.794
6050	0.948	0.777	0.966	0.789
6055	0.946	0.769	0.965	0.780
6060	0.948	0.778	0.982	0.786
6065	0.964	0.847	0.970	0.861
6070	0.950	0.786	1.000	0.791
6075	0.953	0.801	0.952	0.816
6080	0.955	0.809	0.968	0.822
6085	0.948	0.778	0.982	0.786
6090	0.950	0.786	1.000	0.791
6095	0.943	0.754	1.000	0.756
6100	0.951	0.793	0.967	0.806
6105	0.951	0.794	0.983	0.803
6110	0.953	0.802	1.000	0.809
6115	0.950	0.786	1.000	0.791
6120	0.943	0.753	0.964	0.763
6125	0.948	0.777	0.966	0.789
6130	0.957	0.818	1.000	0.825
6135	0.939	0.736	0.945	0.748
6140	0.948	0.778	1.000	0.783
6145	0.948	0.777	0.935	0.795
6150	0.950	0.785	0.966	0.797
6155	0.946	0.770	0.982	0.777
6160	0.945	0.761	0.964	0.771
6165	0.957	0.818	1.000	0.825
6170	0.946	0.769	0.965	0.780
6175	0.962	0.841	1.000	0.849
6180	0.955	0.810	1.000	0.817

6185	0.945	0.762	1.000	0.765
6190	0.951	0.794	1.000	0.800
6195	0.953	0.801	0.983	0.811
6200	0.953	0.801	0.967	0.814
6205	0.950	0.785	0.966	0.797
6210	0.948	0.778	1.000	0.783
6215	0.953	0.801	0.983	0.811
6220	0.945	0.761	0.948	0.775
6225	0.946	0.770	0.982	0.777
6230	0.962	0.840	0.984	0.851
6235	0.950	0.786	0.982	0.794
6240	0.950	0.786	0.982	0.794
6245	0.955	0.810	1.000	0.817
6250	0.941	0.744	0.931	0.761
6255	0.939	0.736	0.945	0.748
6260	0.939	0.736	0.930	0.752
6265	0.946	0.770	0.982	0.777
6270	0.951	0.794	0.983	0.803
6275	0.953	0.802	1.000	0.809
6280	0.955	0.809	0.983	0.819
6285	0.946	0.770	0.982	0.777
6290	0.941	0.744	0.931	0.761
6295	0.951	0.794	0.983	0.803
6300	0.951	0.794	1.000	0.800
6305	0.950	0.785	0.966	0.797
6310	0.951	0.793	0.967	0.806
6315	0.936	0.719	0.927	0.734
6320	0.950	0.785	0.966	0.797
6325	0.948	0.777	0.950	0.792
6330	0.945	0.761	0.933	0.778
6335	0.941	0.745	0.963	0.754
6340	0.938	0.728	0.914	0.746
6345	0.946	0.769	0.965	0.780
6350	0.953	0.801	0.983	0.811
6355	0.943	0.753	0.981	0.759
6360	0.950	0.785	0.951	0.800
6365	0.955	0.809	0.968	0.822
6370	0.946	0.770	0.982	0.777
6375	0.953	0.801	0.952	0.816
6380	0.948	0.777	0.966	0.789
6385	0.946	0.769	0.965	0.780
6390	0.951	0.793	0.967	0.806
6395	0.943	0.753	0.981	0.759

6400	0.945	0.762	1.000	0.765
6405	0.950	0.785	0.951	0.800
6410	0.941	0.744	0.931	0.761
6415	0.945	0.761	0.964	0.771
6420	0.957	0.817	0.984	0.828
6425	0.951	0.793	0.952	0.808
6430	0.950	0.786	0.982	0.794
6435	0.951	0.793	0.967	0.806
6440	0.950	0.786	0.982	0.794
6445	0.955	0.809	0.953	0.824
6450	0.946	0.770	0.982	0.777
6455	0.945	0.761	0.948	0.775
6460	0.943	0.753	0.932	0.769
6465	0.951	0.794	1.000	0.800
6470	0.951	0.794	1.000	0.800
6475	0.945	0.761	0.964	0.771
6480	0.946	0.769	0.949	0.783
6485	0.943	0.753	0.932	0.769
6490	0.951	0.794	1.000	0.800
6495	0.953	0.802	1.000	0.809
6500	0.934	0.711	0.960	0.716
6505	0.953	0.801	0.983	0.811
6510	0.939	0.736	0.962	0.745
6515	0.953	0.801	0.967	0.814
6520	0.948	0.778	0.982	0.786
6525	0.948	0.777	0.950	0.792
6530	0.941	0.745	0.963	0.754
6535	0.951	0.794	0.983	0.803
6540	0.943	0.754	0.905	0.776
6545	0.946	0.769	0.965	0.780
6550	0.950	0.786	0.982	0.794
6555	0.950	0.786	0.982	0.794
6560	0.946	0.770	1.000	0.774
6565	0.950	0.786	0.982	0.794
6570	0.958	0.825	0.984	0.836
6575	0.950	0.786	0.982	0.794
6580	0.946	0.770	0.982	0.777
6585	0.945	0.761	0.948	0.775
6590	0.948	0.777	0.935	0.795
6595	0.945	0.762	0.981	0.768
6600	0.945	0.761	0.948	0.775
6605	0.941	0.744	0.946	0.757
6610	0.943	0.753	0.964	0.763

6615	0.938	0.728	0.962	0.735
6620	0.953	0.801	0.983	0.811
6625	0.945	0.762	0.981	0.768
6630	0.948	0.777	0.966	0.789
6635	0.943	0.753	0.964	0.763
6640	0.950	0.786	0.982	0.794
6645	0.948	0.777	0.966	0.789
6650	0.948	0.777	0.935	0.795
6655	0.953	0.801	0.967	0.814
6660	0.953	0.801	0.967	0.814
6665	0.951	0.794	1.000	0.800
6670	0.957	0.818	1.000	0.825
6675	0.953	0.801	0.952	0.816
6680	0.953	0.801	0.983	0.811
6685	0.948	0.777	0.966	0.789
6690	0.939	0.736	0.945	0.748
6695	0.953	0.801	0.967	0.814
6700	0.955	0.809	0.968	0.822
6705	0.943	0.753	0.964	0.763
6710	0.946	0.770	1.000	0.774
6715	0.948	0.778	0.982	0.786
6720	0.945	0.761	0.933	0.778
6725	0.943	0.753	0.947	0.766
6730	0.948	0.778	0.982	0.786
6735	0.948	0.777	0.966	0.789
6740	0.936	0.719	0.943	0.730
6745	0.953	0.802	1.000	0.809
6750	0.953	0.801	0.967	0.814
6755	0.950	0.786	0.982	0.794
6760	0.945	0.762	0.981	0.768
6765	0.941	0.745	0.981	0.750
6770	0.951	0.794	1.000	0.800
6775	0.951	0.793	0.967	0.806
6780	0.943	0.753	0.932	0.769
6785	0.957	0.817	0.984	0.828
6790	0.945	0.761	0.964	0.771
6795	0.946	0.769	0.965	0.780
6800	0.946	0.769	0.934	0.786
6805	0.953	0.801	0.967	0.814
6810	0.945	0.761	0.964	0.771
6815	0.946	0.769	0.949	0.783
6820	0.948	0.777	0.966	0.789
6825	0.943	0.753	0.964	0.763

6830	0.955	0.809	0.968	0.822
6835	0.948	0.777	0.950	0.792
6840	0.938	0.728	0.962	0.735
6845	0.945	0.761	0.964	0.771
6850	0.946	0.769	0.965	0.780
6855	0.945	0.761	0.964	0.771
6860	0.950	0.786	1.000	0.791
6865	0.950	0.786	1.000	0.791
6870	0.948	0.777	0.966	0.789
6875	0.951	0.794	1.000	0.800
6880	0.948	0.778	0.982	0.786
6885	0.948	0.778	0.982	0.786
6890	0.939	0.737	0.915	0.755
6895	0.958	0.824	0.969	0.838
6900	0.950	0.785	0.966	0.797
6905	0.945	0.761	0.964	0.771
6910	0.945	0.761	0.964	0.771
6915	0.946	0.770	0.982	0.777
6920	0.946	0.770	0.982	0.777
6925	0.946	0.770	0.982	0.777
6930	0.945	0.761	0.948	0.775
6935	0.948	0.777	0.966	0.789
6940	0.950	0.785	0.966	0.797
6945	0.948	0.778	0.982	0.786
6950	0.946	0.770	0.982	0.777
6955	0.941	0.745	0.981	0.750
6960	0.946	0.770	0.982	0.777
6965	0.950	0.786	0.982	0.794
6970	0.948	0.777	0.966	0.789
6975	0.946	0.770	0.982	0.777
6980	0.953	0.802	1.000	0.809
6985	0.941	0.745	0.963	0.754
6990	0.955	0.809	0.983	0.819
6995	0.955	0.809	0.968	0.822
7000	0.950	0.785	0.966	0.797
7005	0.948	0.778	0.982	0.786
7010	0.950	0.786	0.982	0.794
7015	0.953	0.802	1.000	0.809
7020	0.948	0.778	0.982	0.786
7025	0.946	0.769	0.934	0.786
7030	0.953	0.801	0.938	0.819
7035	0.946	0.769	0.949	0.783
7040	0.953	0.801	0.983	0.811

7045	0.945	0.761	0.964	0.771
7050	0.951	0.793	0.938	0.811
7055	0.939	0.737	0.980	0.741
7060	0.957	0.817	0.984	0.828
7065	0.945	0.762	1.000	0.765
7070	0.953	0.801	0.983	0.811
7075	0.955	0.809	0.983	0.819
7080	0.948	0.777	0.966	0.789
7085	0.946	0.769	0.949	0.783
7090	0.948	0.777	0.966	0.789
7095	0.943	0.753	0.981	0.759
7100	0.951	0.794	0.983	0.803
7105	0.953	0.801	0.983	0.811
7110	0.950	0.785	0.966	0.797
7115	0.945	0.761	0.948	0.775
7120	0.943	0.753	0.981	0.759
7125	0.938	0.728	0.914	0.746
7130	0.945	0.761	0.948	0.775
7135	0.950	0.785	0.966	0.797
7140	0.953	0.801	0.983	0.811
7145	0.943	0.753	0.981	0.759
7150	0.945	0.762	1.000	0.765
7155	0.941	0.745	0.963	0.754
7160	0.931	0.694	0.907	0.710
7165	0.941	0.745	0.963	0.754
7170	0.941	0.745	0.981	0.750
7175	0.948	0.777	0.966	0.789
7180	0.945	0.761	0.964	0.771
7185	0.927	0.676	0.938	0.682
7190	0.955	0.810	1.000	0.817
7195	0.946	0.770	0.982	0.777
7200	0.939	0.737	0.980	0.741
7205	0.958	0.825	1.000	0.833
7210	0.955	0.809	0.968	0.822
7215	0.948	0.778	1.000	0.783
7220	0.941	0.745	0.917	0.764
7225	0.950	0.785	0.966	0.797
7230	0.948	0.778	0.982	0.786
7235	0.951	0.794	1.000	0.800
7240	0.943	0.753	0.964	0.763
7245	0.943	0.753	0.981	0.759
7250	0.941	0.745	0.963	0.754
7255	0.948	0.778	1.000	0.783

7260	0.950	0.785	0.966	0.797
7265	0.948	0.777	0.966	0.789
7270	0.943	0.753	0.964	0.763
7275	0.945	0.761	0.948	0.775
7280	0.943	0.753	0.981	0.759
7285	0.938	0.728	0.962	0.735
7290	0.946	0.769	0.965	0.780
7295	0.958	0.825	1.000	0.833
7300	0.943	0.753	0.981	0.759
7305	0.941	0.744	0.946	0.757
7310	0.941	0.745	0.981	0.750
7315	0.946	0.769	0.949	0.783
7320	0.936	0.720	0.980	0.722
7325	0.941	0.745	0.981	0.750
7330	0.960	0.833	1.000	0.841
7335	0.941	0.744	0.946	0.757
7340	0.943	0.753	0.964	0.763
7345	0.948	0.777	0.966	0.789
7350	0.945	0.762	0.981	0.768
7355	0.936	0.719	0.943	0.730
7360	0.948	0.777	0.966	0.789
7365	0.945	0.762	1.000	0.765
7370	0.946	0.770	0.982	0.777
7375	0.945	0.761	0.964	0.771
7380	0.948	0.778	0.982	0.786
7385	0.943	0.753	0.964	0.763
7390	0.939	0.736	0.930	0.752
7395	0.946	0.769	0.965	0.780
7400	0.936	0.719	0.927	0.734
7405	0.941	0.746	1.000	0.746
7410	0.938	0.728	0.962	0.735
7415	0.948	0.778	0.982	0.786
7420	0.943	0.753	0.918	0.772
7425	0.945	0.761	0.964	0.771
7430	0.939	0.736	0.962	0.745
7435	0.955	0.810	1.000	0.817
7440	0.951	0.794	0.983	0.803
7445	0.948	0.778	0.982	0.786
7450	0.943	0.753	0.947	0.766
7455	0.950	0.786	0.982	0.794
7460	0.951	0.793	0.952	0.808
7465	0.946	0.769	0.949	0.783
7470	0.946	0.770	0.982	0.777

7475	0.951	0.794	0.983	0.803
7480	0.938	0.730	1.000	0.727
7485	0.943	0.753	0.981	0.759
7490	0.946	0.769	0.965	0.780
7495	0.946	0.770	0.982	0.777
7500	0.941	0.744	0.931	0.761
7505	0.938	0.729	0.980	0.731
7510	0.943	0.753	0.947	0.766
7515	0.951	0.793	0.967	0.806
7520	0.951	0.794	0.983	0.803
7525	0.948	0.777	0.966	0.789
7530	0.958	0.825	0.984	0.836
7535	0.948	0.777	0.966	0.789
7540	0.945	0.762	0.981	0.768
7545	0.946	0.770	0.982	0.777
7550	0.957	0.818	1.000	0.825
7555	0.939	0.736	0.962	0.745
7560	0.939	0.736	0.962	0.745
7565	0.946	0.770	1.000	0.774
7570	0.951	0.794	0.983	0.803
7575	0.951	0.794	0.983	0.803
7580	0.938	0.728	0.944	0.739
7585	0.939	0.736	0.945	0.748
7590	0.946	0.769	0.949	0.783
7595	0.955	0.809	0.968	0.822
7600	0.941	0.745	0.917	0.764
7605	0.939	0.737	0.980	0.741
7610	0.945	0.761	0.964	0.771
7615	0.951	0.794	0.983	0.803
7620	0.948	0.778	0.982	0.786
7625	0.955	0.809	0.968	0.822
7630	0.948	0.778	0.982	0.786
7635	0.945	0.761	0.919	0.781
7640	0.950	0.785	0.951	0.800
7645	0.945	0.761	0.948	0.775
7650	0.936	0.720	0.961	0.726
7655	0.948	0.777	0.966	0.789
7660	0.941	0.744	0.931	0.761
7665	0.946	0.770	0.982	0.777
7670	0.945	0.761	0.933	0.778
7675	0.953	0.801	0.983	0.811
7680	0.950	0.786	0.982	0.794
7685	0.953	0.801	0.983	0.811

7690	0.946	0.769	0.965	0.780
7695	0.951	0.793	0.967	0.806
7700	0.950	0.785	0.966	0.797
7705	0.948	0.777	0.966	0.789
7710	0.945	0.762	0.981	0.768
7715	0.953	0.801	0.983	0.811
7720	0.950	0.785	0.966	0.797
7725	0.948	0.778	0.982	0.786
7730	0.950	0.785	0.951	0.800
7735	0.946	0.770	0.982	0.777
7740	0.950	0.785	0.951	0.800
7745	0.955	0.809	0.968	0.822
7750	0.945	0.761	0.964	0.771
7755	0.960	0.832	0.969	0.846
7760	0.948	0.777	0.950	0.792
7765	0.948	0.777	0.966	0.789
7770	0.945	0.762	0.981	0.768
7775	0.960	0.832	0.984	0.844
7780	0.950	0.785	0.966	0.797
7785	0.945	0.761	0.933	0.778
7790	0.957	0.817	0.968	0.830
7795	0.950	0.785	0.966	0.797
7800	0.958	0.824	0.969	0.838
7805	0.939	0.736	0.930	0.752
7810	0.941	0.745	0.981	0.750
7815	0.951	0.793	0.967	0.806
7820	0.950	0.786	0.982	0.794
7825	0.939	0.736	0.962	0.745
7830	0.953	0.801	0.938	0.819
7835	0.951	0.794	0.983	0.803
7840	0.950	0.785	0.966	0.797
7845	0.945	0.762	1.000	0.765
7850	0.948	0.778	1.000	0.783
7855	0.946	0.770	0.982	0.777
7860	0.948	0.777	0.935	0.795
7865	0.945	0.761	0.948	0.775
7870	0.951	0.794	0.983	0.803
7875	0.946	0.770	0.982	0.777
7880	0.946	0.770	0.982	0.777
7885	0.948	0.777	0.966	0.789
7890	0.946	0.770	0.982	0.777
7895	0.955	0.809	0.953	0.824
7900	0.936	0.720	0.961	0.726

7905	0.945	0.761	0.964	0.771
7910	0.943	0.753	0.964	0.763
7915	0.950	0.786	0.982	0.794
7920	0.946	0.770	0.982	0.777
7925	0.950	0.785	0.966	0.797
7930	0.955	0.809	0.968	0.822
7935	0.955	0.809	0.968	0.822
7940	0.953	0.801	0.967	0.814
7945	0.943	0.754	1.000	0.756
7950	0.953	0.801	0.983	0.811
7955	0.941	0.744	0.946	0.757
7960	0.946	0.770	0.982	0.777
7965	0.955	0.809	0.983	0.819
7970	0.943	0.753	0.981	0.759
7975	0.950	0.785	0.951	0.800
7980	0.946	0.769	0.949	0.783
7985	0.948	0.778	0.982	0.786
7990	0.946	0.769	0.965	0.780
7995	0.951	0.794	1.000	0.800
8000	0.951	0.794	1.000	0.800
8005	0.948	0.777	0.950	0.792
8010	0.950	0.785	0.966	0.797
8015	0.951	0.794	1.000	0.800
8020	0.953	0.801	0.952	0.816
8025	0.945	0.761	0.964	0.771
8030	0.941	0.745	0.963	0.754
8035	0.953	0.801	0.967	0.814
8040	0.941	0.745	0.981	0.750
8045	0.939	0.736	0.945	0.748
8050	0.951	0.794	0.983	0.803
8055	0.941	0.745	0.981	0.750
8060	0.951	0.794	0.983	0.803
8065	0.948	0.778	0.982	0.786
8070	0.934	0.711	0.960	0.716
8075	0.946	0.769	0.949	0.783
8080	0.951	0.794	1.000	0.800
8085	0.945	0.762	0.981	0.768
8090	0.953	0.801	0.983	0.811
8095	0.936	0.720	0.980	0.722
8100	0.946	0.770	0.982	0.777
8105	0.955	0.809	0.983	0.819
8110	0.941	0.745	0.981	0.750
8115	0.943	0.753	0.932	0.769

8120	0.946	0.770	0.982	0.777
8125	0.946	0.770	0.982	0.777
8130	0.943	0.753	0.947	0.766
8135	0.943	0.753	0.947	0.766
8140	0.953	0.801	0.967	0.814
8145	0.948	0.778	0.982	0.786
8150	0.945	0.762	0.981	0.768
8155	0.946	0.770	0.982	0.777
8160	0.939	0.736	0.945	0.748
8165	0.943	0.754	1.000	0.756
8170	0.953	0.802	1.000	0.809
8175	0.945	0.762	1.000	0.765
8180	0.948	0.778	1.000	0.783
8185	0.938	0.728	0.929	0.743
8190	0.945	0.762	0.981	0.768
8195	0.948	0.777	0.966	0.789
8200	0.946	0.770	0.982	0.777
8205	0.946	0.770	0.982	0.777
8210	0.945	0.761	0.964	0.771
8215	0.946	0.770	1.000	0.774
8220	0.943	0.753	0.947	0.766
8225	0.943	0.753	0.981	0.759
8230	0.950	0.785	0.966	0.797
8235	0.953	0.802	1.000	0.809
8240	0.943	0.754	1.000	0.756
8245	0.953	0.801	0.983	0.811
8250	0.950	0.785	0.966	0.797
8255	0.936	0.720	0.980	0.722
8260	0.948	0.777	0.966	0.789
8265	0.941	0.745	0.963	0.754
8270	0.951	0.794	0.983	0.803
8275	0.948	0.777	0.950	0.792
8280	0.941	0.745	0.981	0.750
8285	0.962	0.841	1.000	0.849
8290	0.958	0.825	0.984	0.836
8295	0.927	0.677	0.889	0.696
8300	0.939	0.737	0.980	0.741
8305	0.941	0.745	0.963	0.754
8310	0.948	0.777	0.966	0.789
8315	0.950	0.786	0.982	0.794
8320	0.957	0.817	0.968	0.830
8325	0.950	0.786	0.982	0.794
8330	0.946	0.769	0.949	0.783

8335	0.955	0.809	0.983	0.819
8340	0.943	0.753	0.964	0.763
8345	0.958	0.825	1.000	0.833
8350	0.950	0.785	0.923	0.805
8355	0.946	0.770	0.982	0.777
8360	0.941	0.745	0.963	0.754
8365	0.945	0.762	0.981	0.768
8370	0.939	0.736	0.962	0.745
8375	0.953	0.801	0.967	0.814
8380	0.936	0.719	0.943	0.730
8385	0.946	0.769	0.949	0.783
8390	0.958	0.824	0.969	0.838
8395	0.936	0.720	0.980	0.722
8400	0.946	0.769	0.965	0.780
8405	0.951	0.794	1.000	0.800
8410	0.939	0.736	0.930	0.752
8415	0.953	0.802	1.000	0.809
8420	0.946	0.770	0.982	0.777
8425	0.941	0.745	0.981	0.750
8430	0.950	0.786	1.000	0.791
8435	0.946	0.770	0.982	0.777
8440	0.951	0.793	0.967	0.806
8445	0.951	0.794	1.000	0.800
8450	0.934	0.712	0.979	0.712
8455	0.945	0.761	0.964	0.771
8460	0.946	0.769	0.934	0.786
8465	0.945	0.762	0.981	0.768
8470	0.948	0.777	0.966	0.789
8475	0.941	0.744	0.946	0.757
8480	0.939	0.736	0.962	0.745
8485	0.946	0.770	0.982	0.777
8490	0.950	0.786	0.982	0.794
8495	0.943	0.753	0.981	0.759
8500	0.955	0.809	0.968	0.822
8505	0.941	0.745	0.981	0.750
8510	0.948	0.778	1.000	0.783
8515	0.951	0.794	1.000	0.800
8520	0.953	0.802	1.000	0.809
8525	0.946	0.770	0.982	0.777
8530	0.943	0.753	0.947	0.766
8535	0.951	0.793	0.967	0.806
8540	0.939	0.736	0.962	0.745
8545	0.951	0.794	1.000	0.800

8550	0.938	0.728	0.962	0.735
8555	0.953	0.801	0.983	0.811
8560	0.941	0.745	0.981	0.750
8565	0.950	0.786	1.000	0.791
8570	0.943	0.753	0.981	0.759
8575	0.941	0.744	0.946	0.757
8580	0.938	0.728	0.944	0.739
8585	0.948	0.777	0.966	0.789
8590	0.943	0.753	0.964	0.763
8595	0.939	0.736	0.962	0.745
8600	0.938	0.728	0.944	0.739
8605	0.950	0.786	1.000	0.791
8610	0.939	0.736	0.945	0.748
8615	0.938	0.729	0.900	0.750
8620	0.948	0.778	0.982	0.786
8625	0.946	0.770	0.982	0.777
8630	0.950	0.786	0.982	0.794
8635	0.955	0.810	1.000	0.817
8640	0.948	0.778	1.000	0.783
8645	0.946	0.769	0.965	0.780
8650	0.951	0.794	1.000	0.800
8655	0.946	0.770	0.982	0.777
8660	0.945	0.761	0.964	0.771
8665	0.950	0.785	0.966	0.797
8670	0.951	0.794	1.000	0.800
8675	0.953	0.801	0.967	0.814
8680	0.941	0.745	0.981	0.750
8685	0.950	0.785	0.966	0.797
8690	0.939	0.736	0.945	0.748
8695	0.946	0.770	1.000	0.774
8700	0.936	0.720	0.980	0.722
8705	0.953	0.802	1.000	0.809
8710	0.943	0.753	0.981	0.759
8715	0.941	0.745	0.981	0.750
8720	0.951	0.794	1.000	0.800
8725	0.945	0.761	0.964	0.771
8730	0.950	0.785	0.966	0.797
8735	0.941	0.744	0.931	0.761
8740	0.945	0.762	0.981	0.768
8745	0.934	0.711	0.926	0.725
8750	0.958	0.824	0.969	0.838
8755	0.945	0.762	0.981	0.768
8760	0.953	0.802	1.000	0.809

8765	0.941	0.746	1.000	0.746
8770	0.948	0.778	1.000	0.783
8775	0.955	0.809	0.983	0.819
8780	0.941	0.744	0.946	0.757
8785	0.951	0.793	0.967	0.806
8790	0.955	0.809	0.968	0.822
8795	0.943	0.753	0.964	0.763
8800	0.941	0.745	0.963	0.754
8805	0.934	0.711	0.960	0.716
8810	0.943	0.753	0.964	0.763
8815	0.934	0.711	0.960	0.716
8820	0.946	0.770	1.000	0.774
8825	0.950	0.786	1.000	0.791
8830	0.945	0.762	0.981	0.768
8835	0.939	0.737	0.915	0.755
8840	0.948	0.778	0.982	0.786
8845	0.943	0.754	1.000	0.756
8850	0.951	0.793	0.967	0.806
8855	0.945	0.762	0.981	0.768
8860	0.950	0.785	0.966	0.797
8865	0.936	0.719	0.943	0.730
8870	0.943	0.753	0.947	0.766
8875	0.950	0.785	0.951	0.800
8880	0.943	0.753	0.964	0.763
8885	0.948	0.777	0.950	0.792
8890	0.943	0.753	0.981	0.759
8895	0.938	0.728	0.929	0.743
8900	0.945	0.762	1.000	0.765
8905	0.943	0.753	0.964	0.763
8910	0.943	0.753	0.981	0.759
8915	0.941	0.744	0.946	0.757
8920	0.948	0.778	1.000	0.783
8925	0.934	0.712	0.979	0.712
8930	0.941	0.745	0.981	0.750
8935	0.946	0.769	0.949	0.783
8940	0.941	0.745	0.963	0.754
8945	0.953	0.801	0.967	0.814
8950	0.934	0.712	0.979	0.712
8955	0.946	0.770	0.982	0.777
8960	0.945	0.761	0.948	0.775
8965	0.948	0.778	1.000	0.783
8970	0.951	0.794	1.000	0.800
8975	0.943	0.753	0.981	0.759

8980	0.960	0.832	0.984	0.844
8985	0.948	0.778	1.000	0.783
8990	0.941	0.746	1.000	0.746
8995	0.948	0.778	1.000	0.783
9000	0.945	0.762	1.000	0.765
9005	0.939	0.736	0.930	0.752
9010	0.938	0.729	0.980	0.731
9015	0.945	0.761	0.948	0.775
9020	0.934	0.711	0.960	0.716
9025	0.943	0.754	1.000	0.756
9030	0.946	0.770	0.982	0.777
9035	0.943	0.754	1.000	0.756
9040	0.943	0.753	0.981	0.759
9045	0.945	0.762	0.981	0.768
9050	0.948	0.778	0.982	0.786
9055	0.941	0.745	0.963	0.754
9060	0.943	0.753	0.981	0.759
9065	0.943	0.753	0.981	0.759
9070	0.946	0.770	1.000	0.774
9075	0.943	0.754	1.000	0.756
9080	0.951	0.794	1.000	0.800
9085	0.941	0.746	1.000	0.746
9090	0.950	0.786	1.000	0.791
9095	0.941	0.745	0.963	0.754
9100	0.945	0.762	0.981	0.768
9105	0.941	0.744	0.931	0.761
9110	0.945	0.762	1.000	0.765
9115	0.932	0.703	0.959	0.707
9120	0.939	0.737	0.980	0.741
9125	0.946	0.770	0.982	0.777
9130	0.941	0.745	0.981	0.750
9135	0.943	0.753	0.964	0.763
9140	0.948	0.777	0.950	0.792
9145	0.948	0.778	1.000	0.783
9150	0.948	0.778	0.982	0.786
9155	0.950	0.786	0.982	0.794
9160	0.946	0.770	1.000	0.774
9165	0.941	0.745	0.981	0.750
9170	0.948	0.777	0.966	0.789
9175	0.941	0.744	0.931	0.761
9180	0.941	0.745	0.981	0.750
9185	0.950	0.785	0.966	0.797
9190	0.945	0.762	0.981	0.768

9195	0.946	0.770	0.982	0.777
9200	0.950	0.786	0.982	0.794
9205	0.948	0.778	1.000	0.783
9210	0.948	0.778	1.000	0.783
9215	0.941	0.745	0.963	0.754
9220	0.939	0.737	0.980	0.741
9225	0.938	0.728	0.962	0.735
9230	0.936	0.719	0.927	0.734
9235	0.941	0.745	0.963	0.754
9240	0.936	0.721	1.000	0.718
9245	0.941	0.745	0.981	0.750
9250	0.938	0.728	0.944	0.739
9255	0.931	0.694	0.958	0.697
9260	0.950	0.786	1.000	0.791
9265	0.936	0.720	0.961	0.726
9270	0.939	0.737	0.980	0.741
9275	0.946	0.770	1.000	0.774
9280	0.943	0.753	0.981	0.759
9285	0.948	0.777	0.950	0.792
9290	0.936	0.720	0.961	0.726
9295	0.943	0.753	0.981	0.759
9300	0.945	0.762	0.981	0.768
9305	0.941	0.745	0.981	0.750
9310	0.946	0.770	1.000	0.774
9315	0.945	0.762	1.000	0.765
9320	0.948	0.778	0.982	0.786
9325	0.945	0.761	0.933	0.778
9330	0.939	0.736	0.962	0.745
9335	0.948	0.778	1.000	0.783
9340	0.948	0.778	1.000	0.783
9345	0.939	0.736	0.962	0.745
9350	0.946	0.769	0.965	0.780
9355	0.938	0.728	0.944	0.739
9360	0.936	0.721	1.000	0.718
9365	0.938	0.728	0.962	0.735
9370	0.953	0.801	0.983	0.811
9375	0.946	0.769	0.949	0.783
9380	0.948	0.778	0.982	0.786
9385	0.939	0.737	0.980	0.741
9390	0.950	0.785	0.966	0.797
9395	0.951	0.794	0.983	0.803
9400	0.943	0.753	0.981	0.759
9405	0.939	0.737	0.980	0.741

9410	0.941	0.745	0.963	0.754
9415	0.946	0.770	1.000	0.774
9420	0.945	0.761	0.948	0.775
9425	0.943	0.753	0.964	0.763
9430	0.945	0.762	0.981	0.768
9435	0.939	0.737	0.980	0.741
9440	0.931	0.694	0.958	0.697
9445	0.946	0.770	0.982	0.777
9450	0.941	0.745	0.963	0.754
9455	0.936	0.720	0.961	0.726
9460	0.943	0.753	0.981	0.759
9465	0.941	0.745	0.981	0.750
9470	0.948	0.778	0.982	0.786
9475	0.951	0.794	0.983	0.803
9480	0.939	0.736	0.962	0.745
9485	0.945	0.761	0.964	0.771
9490	0.948	0.778	0.982	0.786
9495	0.950	0.786	1.000	0.791
9500	0.943	0.753	0.964	0.763
9505	0.946	0.770	0.982	0.777
9510	0.927	0.678	0.977	0.672
9515	0.938	0.730	1.000	0.727
9520	0.936	0.719	0.927	0.734
9525	0.941	0.745	0.981	0.750
9530	0.938	0.728	0.962	0.735
9535	0.945	0.762	0.981	0.768
9540	0.943	0.753	0.981	0.759
9545	0.948	0.778	1.000	0.783
9550	0.945	0.761	0.948	0.775
9555	0.948	0.778	1.000	0.783
9560	0.941	0.745	0.981	0.750
9565	0.936	0.719	0.943	0.730
9570	0.943	0.753	0.964	0.763
9575	0.938	0.729	0.980	0.731
9580	0.939	0.738	1.000	0.737
9585	0.945	0.762	0.906	0.784
9590	0.936	0.720	0.961	0.726
9595	0.939	0.736	0.930	0.752
9600	0.931	0.694	0.907	0.710
9605	0.948	0.778	0.982	0.786
9610	0.946	0.769	0.965	0.780
9615	0.945	0.761	0.948	0.775
9620	0.948	0.778	1.000	0.783

9625	0.934	0.711	0.960	0.716
9630	0.939	0.736	0.945	0.748
9635	0.938	0.728	0.962	0.735
9640	0.939	0.737	0.980	0.741
9645	0.938	0.728	0.962	0.735
9650	0.946	0.770	1.000	0.774
9655	0.945	0.762	0.981	0.768
9660	0.941	0.745	0.963	0.754
9665	0.946	0.770	1.000	0.774
9670	0.948	0.777	0.966	0.789
9675	0.946	0.769	0.965	0.780
9680	0.934	0.711	0.926	0.725
9685	0.938	0.730	1.000	0.727
9690	0.939	0.737	0.980	0.741
9695	0.939	0.736	0.945	0.748
9700	0.938	0.729	0.980	0.731
9705	0.941	0.745	0.981	0.750
9710	0.945	0.761	0.964	0.771
9715	0.938	0.729	0.980	0.731
9720	0.948	0.778	0.982	0.786
9725	0.945	0.762	0.981	0.768
9730	0.943	0.753	0.964	0.763
9735	0.939	0.737	0.980	0.741
9740	0.945	0.762	0.981	0.768
9745	0.941	0.745	0.981	0.750
9750	0.943	0.753	0.964	0.763
9755	0.950	0.786	0.982	0.794
9760	0.953	0.802	1.000	0.809
9765	0.941	0.745	0.963	0.754
9770	0.941	0.745	0.963	0.754
9775	0.948	0.777	0.950	0.792
9780	0.948	0.778	0.982	0.786
9785	0.939	0.738	1.000	0.737
9790	0.939	0.737	0.980	0.741
9795	0.948	0.778	0.982	0.786
9800	0.950	0.786	1.000	0.791
9805	0.945	0.762	1.000	0.765
9810	0.939	0.737	0.980	0.741
9815	0.939	0.736	0.962	0.745
9820	0.938	0.728	0.962	0.735
9825	0.931	0.694	0.940	0.701
9830	0.946	0.770	1.000	0.774
9835	0.945	0.762	1.000	0.765

9840	0.936	0.720	0.980	0.722
9845	0.941	0.746	1.000	0.746
9850	0.939	0.736	0.962	0.745
9855	0.945	0.762	0.981	0.768
9860	0.941	0.745	0.981	0.750
9865	0.948	0.778	0.982	0.786
9870	0.939	0.736	0.962	0.745
9875	0.941	0.746	1.000	0.746
9880	0.934	0.711	0.942	0.721
9885	0.939	0.737	0.980	0.741
9890	0.941	0.745	0.981	0.750
9895	0.938	0.728	0.962	0.735
9900	0.941	0.745	0.981	0.750
9905	0.936	0.720	0.961	0.726
9910	0.938	0.728	0.944	0.739
9915	0.929	0.685	0.939	0.692
9920	0.945	0.762	0.981	0.768
9925	0.938	0.728	0.944	0.739
9930	0.939	0.737	0.980	0.741
9935	0.938	0.728	0.962	0.735
9940	0.934	0.711	0.942	0.721
9945	0.936	0.720	0.961	0.726
9950	0.936	0.719	0.943	0.730
9955	0.953	0.801	0.983	0.811
9960	0.943	0.753	0.981	0.759
9965	0.938	0.729	0.980	0.731
9970	0.939	0.736	0.962	0.745
9975	0.941	0.745	0.981	0.750
9980	0.939	0.736	0.962	0.745
9985	0.938	0.728	0.962	0.735
9990	0.943	0.753	0.964	0.763
9995	0.929	0.686	0.978	0.682
10000	0.948	0.778	0.982	0.786

(4) Performance of IFS with decision tree on the list yielded by LightGBM

Number of features	ACC	MCC	Precision	F1-measure
5	0.808	0.405	0.401	0.498
10	0.821	0.469	0.434	0.546
15	0.860	0.530	0.513	0.601
20	0.844	0.529	0.478	0.595
25	0.847	0.529	0.485	0.596
30	0.839	0.492	0.466	0.567
35	0.832	0.463	0.450	0.545

40	0.872	0.570	0.542	0.634
45	0.863	0.579	0.519	0.636
50	0.853	0.479	0.495	0.560
55	0.825	0.451	0.436	0.535
60	0.847	0.507	0.484	0.581
65	0.818	0.403	0.416	0.498
70	0.825	0.439	0.434	0.526
75	0.837	0.477	0.461	0.557
80	0.813	0.443	0.417	0.526
85	0.835	0.497	0.460	0.570
90	0.854	0.531	0.500	0.600
95	0.856	0.529	0.504	0.599
100	0.830	0.471	0.448	0.550
105	0.861	0.550	0.516	0.615
110	0.837	0.528	0.466	0.591
115	0.841	0.534	0.472	0.596
120	0.808	0.429	0.407	0.515
125	0.806	0.427	0.404	0.513
130	0.828	0.451	0.442	0.535
135	0.815	0.422	0.415	0.511
140	0.813	0.370	0.400	0.471
145	0.816	0.413	0.415	0.505
150	0.811	0.398	0.405	0.493
155	0.802	0.366	0.385	0.467
160	0.797	0.383	0.383	0.480
165	0.815	0.397	0.409	0.493
170	0.823	0.412	0.426	0.505
175	0.813	0.382	0.403	0.481
180	0.778	0.356	0.355	0.458
185	0.806	0.384	0.394	0.481
190	0.795	0.348	0.371	0.454
195	0.808	0.386	0.397	0.484
200	0.820	0.412	0.421	0.505
205	0.804	0.368	0.388	0.469
210	0.828	0.439	0.440	0.526
215	0.815	0.379	0.405	0.478
220	0.806	0.371	0.391	0.472
225	0.797	0.401	0.388	0.494
230	0.802	0.391	0.391	0.486
235	0.801	0.394	0.390	0.489
240	0.830	0.430	0.442	0.520
245	0.806	0.396	0.397	0.491
250	0.790	0.379	0.374	0.476

255	0.787	0.361	0.366	0.463
260	0.811	0.367	0.397	0.468
265	0.797	0.364	0.378	0.466
270	0.802	0.320	0.371	0.430
275	0.783	0.317	0.348	0.429
280	0.783	0.344	0.357	0.449
285	0.775	0.331	0.345	0.440
290	0.782	0.315	0.346	0.427
295	0.816	0.413	0.415	0.505
300	0.783	0.337	0.355	0.444
305	0.801	0.394	0.390	0.489
310	0.797	0.401	0.388	0.494
315	0.830	0.477	0.449	0.555
320	0.806	0.427	0.404	0.513
325	0.789	0.383	0.373	0.479
330	0.806	0.371	0.391	0.472
335	0.764	0.271	0.314	0.393
340	0.789	0.325	0.356	0.435
345	0.815	0.410	0.412	0.502
350	0.797	0.318	0.364	0.429
355	0.801	0.331	0.372	0.439
360	0.783	0.356	0.361	0.459
365	0.809	0.438	0.411	0.522
370	0.802	0.384	0.390	0.482
375	0.799	0.398	0.389	0.491
380	0.769	0.311	0.333	0.424
385	0.799	0.360	0.379	0.463
390	0.802	0.353	0.381	0.457
395	0.790	0.301	0.350	0.415
400	0.794	0.346	0.368	0.452
405	0.775	0.331	0.345	0.440
410	0.790	0.341	0.363	0.447
415	0.804	0.343	0.380	0.449
420	0.789	0.345	0.362	0.450
425	0.804	0.399	0.396	0.493
430	0.815	0.416	0.414	0.507
435	0.816	0.419	0.417	0.509
440	0.780	0.339	0.352	0.445
445	0.815	0.416	0.414	0.507
450	0.818	0.415	0.419	0.507
455	0.782	0.341	0.354	0.447
460	0.792	0.362	0.371	0.464
465	0.789	0.312	0.352	0.425

470	0.773	0.309	0.336	0.423
475	0.773	0.323	0.340	0.433
480	0.799	0.360	0.379	0.463
485	0.769	0.291	0.326	0.409
490	0.773	0.262	0.318	0.385
495	0.780	0.312	0.343	0.425
500	0.775	0.331	0.345	0.440
505	0.764	0.278	0.317	0.398
510	0.756	0.266	0.306	0.390
515	0.764	0.215	0.290	0.346
520	0.797	0.389	0.385	0.485
525	0.823	0.412	0.426	0.505
530	0.764	0.324	0.333	0.433
535	0.771	0.340	0.344	0.445
540	0.785	0.333	0.355	0.441
545	0.809	0.364	0.393	0.466
550	0.806	0.377	0.392	0.477
555	0.794	0.339	0.366	0.447
560	0.782	0.341	0.354	0.447
565	0.813	0.363	0.398	0.465
570	0.780	0.312	0.343	0.425
575	0.792	0.337	0.364	0.444
580	0.799	0.373	0.382	0.473
585	0.756	0.306	0.321	0.420
590	0.773	0.276	0.323	0.396
595	0.790	0.366	0.371	0.467
600	0.775	0.298	0.333	0.414
605	0.808	0.355	0.388	0.459
610	0.794	0.378	0.378	0.476
615	0.782	0.328	0.350	0.438
620	0.782	0.322	0.348	0.432
625	0.780	0.332	0.350	0.441
630	0.792	0.283	0.345	0.400
635	0.809	0.351	0.390	0.455
640	0.775	0.338	0.347	0.444
645	0.775	0.271	0.323	0.393
650	0.771	0.300	0.331	0.416
655	0.785	0.320	0.351	0.431
660	0.769	0.278	0.321	0.398
665	0.789	0.305	0.349	0.419
670	0.766	0.266	0.314	0.389
675	0.808	0.374	0.394	0.474
680	0.804	0.349	0.382	0.454

685	0.768	0.296	0.326	0.412
690	0.799	0.385	0.386	0.482
695	0.794	0.371	0.376	0.471
700	0.773	0.276	0.323	0.396
705	0.766	0.293	0.324	0.410
710	0.783	0.344	0.357	0.449
715	0.809	0.370	0.395	0.471
720	0.776	0.281	0.328	0.400
725	0.794	0.384	0.379	0.480
730	0.787	0.361	0.366	0.463
735	0.802	0.327	0.373	0.436
740	0.773	0.283	0.326	0.402
745	0.750	0.232	0.289	0.363
750	0.749	0.216	0.281	0.350
755	0.795	0.342	0.369	0.449
760	0.787	0.309	0.349	0.423
765	0.754	0.264	0.304	0.388
770	0.795	0.348	0.371	0.454
775	0.775	0.278	0.326	0.398
780	0.761	0.224	0.292	0.355
785	0.792	0.350	0.368	0.455
790	0.790	0.354	0.367	0.457
795	0.792	0.356	0.370	0.459
800	0.763	0.309	0.327	0.422
805	0.763	0.248	0.304	0.374
810	0.799	0.328	0.369	0.437
815	0.795	0.329	0.365	0.438
820	0.780	0.332	0.350	0.441
825	0.797	0.377	0.381	0.475
830	0.802	0.403	0.394	0.496
835	0.769	0.318	0.336	0.429
840	0.795	0.393	0.384	0.487
845	0.787	0.309	0.349	0.423
850	0.782	0.302	0.341	0.417
855	0.775	0.370	0.356	0.467
860	0.775	0.331	0.345	0.440
865	0.773	0.309	0.336	0.423
870	0.790	0.373	0.372	0.472
875	0.766	0.307	0.329	0.421
880	0.782	0.348	0.356	0.452
885	0.780	0.339	0.352	0.445
890	0.775	0.305	0.336	0.420
895	0.775	0.298	0.333	0.414

900	0.768	0.316	0.333	0.427
905	0.778	0.263	0.323	0.385
910	0.785	0.359	0.363	0.461
915	0.780	0.326	0.348	0.436
920	0.799	0.392	0.387	0.487
925	0.820	0.406	0.419	0.500
930	0.795	0.355	0.373	0.459
935	0.768	0.309	0.331	0.422
940	0.794	0.390	0.381	0.485
945	0.795	0.368	0.377	0.468
950	0.811	0.404	0.406	0.498
955	0.804	0.375	0.389	0.474
960	0.792	0.343	0.366	0.450
965	0.769	0.324	0.338	0.434
970	0.783	0.344	0.357	0.449
975	0.797	0.332	0.368	0.440
980	0.821	0.463	0.433	0.542
985	0.775	0.312	0.338	0.425
990	0.750	0.239	0.292	0.368
995	0.789	0.370	0.370	0.470
1000	0.799	0.385	0.386	0.482
1005	0.771	0.314	0.336	0.426
1010	0.787	0.309	0.349	0.423
1015	0.794	0.346	0.368	0.452
1020	0.804	0.368	0.388	0.469
1025	0.757	0.268	0.308	0.391
1030	0.787	0.342	0.360	0.448
1035	0.802	0.403	0.394	0.496
1040	0.780	0.326	0.348	0.436
1045	0.789	0.318	0.354	0.430
1050	0.764	0.278	0.317	0.398
1055	0.769	0.298	0.329	0.414
1060	0.795	0.342	0.369	0.449
1065	0.738	0.265	0.294	0.389
1070	0.794	0.359	0.372	0.462
1075	0.757	0.262	0.306	0.386
1080	0.799	0.404	0.390	0.496
1085	0.782	0.295	0.338	0.411
1090	0.802	0.372	0.386	0.472
1095	0.766	0.259	0.311	0.384
1100	0.757	0.289	0.316	0.407
1105	0.804	0.368	0.388	0.469
1110	0.809	0.370	0.395	0.471

1115	0.799	0.360	0.379	0.463
1120	0.802	0.403	0.394	0.496
1125	0.809	0.305	0.375	0.415
1130	0.783	0.291	0.339	0.408
1135	0.804	0.393	0.394	0.489
1140	0.787	0.361	0.366	0.463
1145	0.782	0.341	0.354	0.447
1150	0.785	0.378	0.368	0.475
1155	0.801	0.382	0.387	0.480
1160	0.795	0.361	0.375	0.464
1165	0.797	0.351	0.374	0.456
1170	0.795	0.289	0.351	0.404
1175	0.783	0.304	0.344	0.419
1180	0.797	0.345	0.372	0.451
1185	0.802	0.372	0.386	0.472
1190	0.778	0.303	0.338	0.418
1195	0.809	0.389	0.400	0.486
1200	0.789	0.298	0.347	0.413
1205	0.778	0.297	0.336	0.413
1210	0.757	0.275	0.311	0.397
1215	0.771	0.327	0.340	0.436
1220	0.787	0.296	0.344	0.411
1225	0.780	0.332	0.350	0.441
1230	0.782	0.322	0.348	0.432
1235	0.766	0.293	0.324	0.410
1240	0.785	0.333	0.355	0.441
1245	0.763	0.262	0.309	0.386
1250	0.769	0.291	0.326	0.409
1255	0.778	0.349	0.353	0.453
1260	0.778	0.303	0.338	0.418
1265	0.761	0.293	0.320	0.410
1270	0.773	0.276	0.323	0.396
1275	0.782	0.281	0.333	0.400
1280	0.769	0.264	0.316	0.387
1285	0.780	0.272	0.328	0.392
1290	0.768	0.335	0.340	0.442
1295	0.792	0.324	0.359	0.434
1300	0.785	0.346	0.359	0.451
1305	0.769	0.311	0.333	0.424
1310	0.809	0.383	0.398	0.481
1315	0.794	0.415	0.387	0.502
1320	0.768	0.262	0.313	0.385
1325	0.759	0.304	0.323	0.418

1330	0.768	0.275	0.319	0.396
1335	0.790	0.385	0.376	0.481
1340	0.792	0.343	0.366	0.450
1345	0.815	0.440	0.418	0.524
1350	0.787	0.348	0.362	0.453
1355	0.757	0.262	0.306	0.386
1360	0.825	0.457	0.437	0.539
1365	0.802	0.391	0.391	0.486
1370	0.754	0.250	0.299	0.377
1375	0.768	0.302	0.329	0.417
1380	0.804	0.412	0.399	0.502
1385	0.759	0.257	0.305	0.382
1390	0.806	0.345	0.383	0.451
1395	0.821	0.409	0.423	0.502
1400	0.771	0.320	0.338	0.431
1405	0.769	0.337	0.342	0.444
1410	0.795	0.380	0.380	0.478
1415	0.790	0.347	0.365	0.452
1420	0.769	0.363	0.350	0.462
1425	0.790	0.360	0.369	0.462
1430	0.811	0.354	0.393	0.458
1435	0.792	0.369	0.373	0.469
1440	0.801	0.394	0.390	0.489
1445	0.768	0.289	0.324	0.407
1450	0.778	0.317	0.343	0.429
1455	0.776	0.287	0.331	0.406
1460	0.773	0.269	0.321	0.391
1465	0.785	0.352	0.361	0.456
1470	0.783	0.331	0.353	0.439
1475	0.823	0.442	0.432	0.528
1480	0.790	0.354	0.367	0.457
1485	0.808	0.399	0.400	0.493
1490	0.776	0.347	0.351	0.451
1495	0.794	0.286	0.348	0.402
1500	0.776	0.353	0.353	0.456
1505	0.808	0.392	0.398	0.488
1510	0.799	0.410	0.392	0.500
1515	0.769	0.285	0.324	0.404
1520	0.801	0.413	0.395	0.502
1525	0.763	0.289	0.320	0.407
1530	0.806	0.408	0.400	0.500
1535	0.783	0.324	0.350	0.434
1540	0.787	0.368	0.367	0.468

1545	0.771	0.287	0.326	0.405
1550	0.778	0.343	0.351	0.448
1555	0.821	0.439	0.429	0.525
1560	0.783	0.356	0.361	0.459
1565	0.792	0.356	0.370	0.459
1570	0.792	0.350	0.368	0.455
1575	0.802	0.409	0.396	0.500
1580	0.821	0.445	0.430	0.530
1585	0.809	0.408	0.404	0.500
1590	0.790	0.373	0.372	0.472
1595	0.802	0.409	0.396	0.500
1600	0.773	0.309	0.336	0.423
1605	0.785	0.359	0.363	0.461
1610	0.789	0.357	0.366	0.460
1615	0.768	0.302	0.329	0.417
1620	0.792	0.350	0.368	0.455
1625	0.785	0.333	0.355	0.441
1630	0.754	0.257	0.301	0.383
1635	0.816	0.400	0.413	0.495
1640	0.797	0.370	0.380	0.471
1645	0.797	0.345	0.372	0.451
1650	0.776	0.321	0.343	0.432
1655	0.834	0.489	0.456	0.564
1660	0.811	0.435	0.413	0.520
1665	0.782	0.302	0.341	0.417
1670	0.785	0.327	0.353	0.436
1675	0.789	0.332	0.358	0.440
1680	0.780	0.345	0.354	0.450
1685	0.811	0.398	0.405	0.493
1690	0.795	0.355	0.373	0.459
1695	0.789	0.338	0.360	0.445
1700	0.756	0.252	0.301	0.379
1705	0.775	0.325	0.342	0.435
1710	0.808	0.392	0.398	0.488
1715	0.794	0.396	0.383	0.489
1720	0.802	0.391	0.391	0.486
1725	0.785	0.327	0.353	0.436
1730	0.797	0.383	0.383	0.480
1735	0.804	0.387	0.393	0.484
1740	0.763	0.295	0.322	0.412
1745	0.771	0.294	0.329	0.411
1750	0.799	0.373	0.382	0.473
1755	0.802	0.384	0.390	0.482

1760	0.780	0.345	0.354	0.450
1765	0.773	0.276	0.323	0.396
1770	0.780	0.312	0.343	0.425
1775	0.745	0.267	0.299	0.390
1780	0.757	0.262	0.306	0.386
1785	0.780	0.332	0.350	0.441
1790	0.802	0.378	0.388	0.477
1795	0.771	0.294	0.329	0.411
1800	0.799	0.366	0.381	0.468
1805	0.804	0.356	0.384	0.459
1810	0.761	0.300	0.322	0.415
1815	0.802	0.403	0.394	0.496
1820	0.780	0.377	0.363	0.473
1825	0.756	0.300	0.318	0.415
1830	0.790	0.366	0.371	0.467
1835	0.768	0.289	0.324	0.407
1840	0.802	0.359	0.383	0.462
1845	0.775	0.305	0.336	0.420
1850	0.789	0.364	0.368	0.465
1855	0.780	0.352	0.356	0.455
1860	0.794	0.365	0.374	0.466
1865	0.776	0.360	0.355	0.460
1870	0.799	0.366	0.381	0.468
1875	0.768	0.262	0.313	0.385
1880	0.787	0.380	0.371	0.477
1885	0.766	0.280	0.319	0.400
1890	0.775	0.338	0.347	0.444
1895	0.773	0.309	0.336	0.423
1900	0.795	0.361	0.375	0.464
1905	0.771	0.327	0.340	0.436
1910	0.787	0.329	0.356	0.438
1915	0.780	0.364	0.359	0.464
1920	0.761	0.266	0.310	0.389
1925	0.789	0.383	0.373	0.479
1930	0.776	0.347	0.351	0.451
1935	0.785	0.333	0.355	0.441
1940	0.778	0.330	0.347	0.439
1945	0.783	0.344	0.357	0.449
1950	0.771	0.294	0.329	0.411
1955	0.768	0.309	0.331	0.422
1960	0.795	0.348	0.371	0.454
1965	0.754	0.284	0.312	0.403
1970	0.801	0.344	0.376	0.450

1975	0.775	0.325	0.342	0.435
1980	0.766	0.307	0.329	0.421
1985	0.780	0.339	0.352	0.445
1990	0.780	0.332	0.350	0.441
1995	0.782	0.308	0.343	0.422
2000	0.773	0.342	0.346	0.447
2005	0.785	0.307	0.346	0.421
2010	0.785	0.340	0.357	0.446
2015	0.773	0.309	0.336	0.423
2020	0.776	0.308	0.338	0.422
2025	0.802	0.384	0.390	0.482
2030	0.783	0.317	0.348	0.429
2035	0.785	0.333	0.355	0.441
2040	0.763	0.268	0.312	0.391
2045	0.768	0.275	0.319	0.396
2050	0.764	0.318	0.331	0.429
2055	0.766	0.300	0.327	0.416
2060	0.761	0.259	0.307	0.384
2065	0.764	0.284	0.319	0.404
2070	0.768	0.255	0.311	0.380
2075	0.794	0.371	0.376	0.471
2080	0.794	0.365	0.374	0.466
2085	0.808	0.423	0.406	0.511
2090	0.789	0.376	0.372	0.474
2095	0.799	0.404	0.390	0.496
2100	0.799	0.379	0.384	0.477
2105	0.752	0.289	0.312	0.407
2110	0.820	0.406	0.419	0.500
2115	0.790	0.347	0.365	0.452
2120	0.726	0.195	0.260	0.336
2125	0.785	0.372	0.367	0.470
2130	0.785	0.333	0.355	0.441
2135	0.797	0.377	0.381	0.475
2140	0.795	0.368	0.377	0.468
2145	0.776	0.308	0.338	0.422
2150	0.799	0.392	0.387	0.487
2155	0.756	0.280	0.311	0.400
2160	0.785	0.320	0.351	0.431
2165	0.795	0.380	0.380	0.478
2170	0.785	0.320	0.351	0.431
2175	0.759	0.317	0.327	0.428
2180	0.809	0.420	0.407	0.509
2185	0.801	0.337	0.374	0.444

2190	0.749	0.244	0.293	0.372
2195	0.790	0.341	0.363	0.447
2200	0.789	0.351	0.364	0.455
2205	0.775	0.292	0.331	0.409
2210	0.763	0.309	0.327	0.422
2215	0.780	0.306	0.341	0.420
2220	0.808	0.405	0.401	0.498
2225	0.766	0.313	0.331	0.426
2230	0.763	0.268	0.312	0.391
2235	0.766	0.313	0.331	0.426
2240	0.775	0.370	0.356	0.467
2245	0.775	0.278	0.326	0.398
2250	0.754	0.271	0.307	0.393
2255	0.780	0.345	0.354	0.450
2260	0.808	0.355	0.388	0.459
2265	0.761	0.300	0.322	0.415
2270	0.763	0.275	0.315	0.396
2275	0.761	0.313	0.327	0.425
2280	0.778	0.336	0.349	0.443
2285	0.825	0.457	0.437	0.539
2290	0.771	0.307	0.333	0.421
2295	0.787	0.374	0.369	0.472
2300	0.818	0.451	0.426	0.533
2305	0.771	0.287	0.326	0.405
2310	0.778	0.317	0.343	0.429
2315	0.745	0.267	0.299	0.390
2320	0.771	0.340	0.344	0.445
2325	0.740	0.240	0.286	0.370
2330	0.794	0.306	0.355	0.420
2335	0.802	0.421	0.399	0.509
2340	0.785	0.365	0.365	0.466
2345	0.797	0.389	0.385	0.485
2350	0.759	0.284	0.315	0.403
2355	0.795	0.380	0.380	0.478
2360	0.773	0.349	0.348	0.452
2365	0.773	0.309	0.336	0.423
2370	0.785	0.365	0.365	0.466
2375	0.797	0.401	0.388	0.494
2380	0.750	0.286	0.310	0.405
2385	0.780	0.332	0.350	0.441
2390	0.766	0.313	0.331	0.426
2395	0.821	0.409	0.423	0.502
2400	0.771	0.340	0.344	0.445

2405	0.789	0.325	0.356	0.435
2410	0.768	0.289	0.324	0.407
2415	0.792	0.356	0.370	0.459
2420	0.787	0.322	0.353	0.433
2425	0.782	0.281	0.333	0.400
2430	0.792	0.350	0.368	0.455
2435	0.780	0.345	0.354	0.450
2440	0.789	0.364	0.368	0.465
2445	0.764	0.284	0.319	0.404
2450	0.773	0.276	0.323	0.396
2455	0.750	0.286	0.310	0.405
2460	0.776	0.301	0.336	0.416
2465	0.775	0.331	0.345	0.440
2470	0.789	0.351	0.364	0.455
2475	0.763	0.282	0.317	0.402
2480	0.749	0.264	0.301	0.388
2485	0.792	0.381	0.377	0.478
2490	0.778	0.310	0.341	0.423
2495	0.778	0.323	0.345	0.434
2500	0.789	0.332	0.358	0.440
2505	0.825	0.439	0.434	0.526
2510	0.769	0.264	0.316	0.387
2515	0.782	0.341	0.354	0.447
2520	0.795	0.361	0.375	0.464
2525	0.790	0.373	0.372	0.472
2530	0.775	0.395	0.363	0.484
2535	0.747	0.248	0.293	0.376
2540	0.773	0.323	0.340	0.433
2545	0.764	0.324	0.333	0.433
2550	0.768	0.316	0.333	0.427
2555	0.794	0.371	0.376	0.471
2560	0.773	0.336	0.344	0.443
2565	0.835	0.428	0.451	0.518
2570	0.775	0.325	0.342	0.435
2575	0.759	0.271	0.310	0.393
2580	0.785	0.384	0.370	0.479
2585	0.754	0.236	0.293	0.366
2590	0.776	0.340	0.349	0.446
2595	0.787	0.336	0.358	0.443
2600	0.763	0.289	0.320	0.407
2605	0.797	0.383	0.383	0.480
2610	0.771	0.300	0.331	0.416
2615	0.783	0.297	0.341	0.413

2620	0.756	0.286	0.314	0.405
2625	0.801	0.413	0.395	0.502
2630	0.785	0.333	0.355	0.441
2635	0.816	0.394	0.411	0.490
2640	0.797	0.332	0.368	0.440
2645	0.783	0.331	0.353	0.439
2650	0.756	0.293	0.316	0.410
2655	0.724	0.200	0.261	0.340
2660	0.787	0.336	0.358	0.443
2665	0.785	0.320	0.351	0.431
2670	0.740	0.267	0.296	0.390
2675	0.782	0.322	0.348	0.432
2680	0.801	0.375	0.385	0.475
2685	0.823	0.436	0.431	0.523
2690	0.768	0.269	0.316	0.391
2695	0.799	0.354	0.377	0.458
2700	0.768	0.309	0.331	0.422
2705	0.775	0.318	0.340	0.430
2710	0.787	0.309	0.349	0.423
2715	0.747	0.241	0.291	0.371
2720	0.790	0.366	0.371	0.467
2725	0.787	0.329	0.356	0.438
2730	0.757	0.289	0.316	0.407
2735	0.766	0.320	0.333	0.430
2740	0.785	0.346	0.359	0.451
2745	0.782	0.398	0.370	0.488
2750	0.783	0.375	0.366	0.473
2755	0.797	0.377	0.381	0.475
2760	0.766	0.339	0.340	0.444
2765	0.778	0.310	0.341	0.423
2770	0.799	0.392	0.387	0.487
2775	0.740	0.253	0.291	0.380
2780	0.785	0.340	0.357	0.446
2785	0.794	0.378	0.378	0.476
2790	0.785	0.352	0.361	0.456
2795	0.773	0.349	0.348	0.452
2800	0.790	0.373	0.372	0.472
2805	0.775	0.285	0.328	0.404
2810	0.763	0.302	0.325	0.417
2815	0.769	0.331	0.340	0.439
2820	0.761	0.266	0.310	0.389
2825	0.761	0.280	0.315	0.400
2830	0.778	0.303	0.338	0.418

2835	0.787	0.329	0.356	0.438
2840	0.801	0.369	0.383	0.470
2845	0.773	0.329	0.342	0.438
2850	0.795	0.355	0.373	0.459
2855	0.787	0.355	0.364	0.458
2860	0.752	0.289	0.312	0.407
2865	0.749	0.237	0.290	0.367
2870	0.806	0.396	0.397	0.491
2875	0.759	0.250	0.302	0.377
2880	0.783	0.369	0.364	0.468
2885	0.787	0.374	0.369	0.472
2890	0.738	0.258	0.292	0.384
2895	0.763	0.295	0.322	0.412
2900	0.773	0.349	0.348	0.452
2905	0.795	0.374	0.379	0.473
2910	0.759	0.284	0.315	0.403
2915	0.809	0.420	0.407	0.509
2920	0.787	0.361	0.366	0.463
2925	0.756	0.306	0.321	0.420
2930	0.773	0.289	0.328	0.407
2935	0.775	0.312	0.338	0.425
2940	0.802	0.415	0.397	0.504
2945	0.769	0.271	0.319	0.393
2950	0.783	0.291	0.339	0.408
2955	0.787	0.361	0.366	0.463
2960	0.804	0.424	0.401	0.511
2965	0.789	0.338	0.360	0.445
2970	0.780	0.292	0.336	0.409
2975	0.749	0.244	0.293	0.372
2980	0.773	0.262	0.318	0.385
2985	0.797	0.345	0.372	0.451
2990	0.802	0.372	0.386	0.472
2995	0.787	0.336	0.358	0.443
3000	0.764	0.278	0.317	0.398
3005	0.778	0.310	0.341	0.423
3010	0.768	0.275	0.319	0.396
3015	0.754	0.215	0.284	0.349
3020	0.771	0.294	0.329	0.411
3025	0.771	0.353	0.348	0.455
3030	0.790	0.354	0.367	0.457
3035	0.778	0.330	0.347	0.439
3040	0.787	0.336	0.358	0.443
3045	0.799	0.366	0.381	0.468

3050	0.776	0.294	0.333	0.411
3055	0.782	0.386	0.367	0.479
3060	0.776	0.347	0.351	0.451
3065	0.789	0.383	0.373	0.479
3070	0.795	0.361	0.375	0.464
3075	0.763	0.248	0.304	0.374
3080	0.776	0.308	0.338	0.422
3085	0.797	0.357	0.376	0.461
3090	0.757	0.289	0.316	0.407
3095	0.783	0.337	0.355	0.444
3100	0.775	0.325	0.342	0.435
3105	0.790	0.334	0.361	0.442
3110	0.719	0.173	0.247	0.319
3115	0.747	0.206	0.275	0.342
3120	0.806	0.402	0.399	0.495
3125	0.754	0.243	0.296	0.372
3130	0.806	0.371	0.391	0.472
3135	0.789	0.325	0.356	0.435
3140	0.754	0.236	0.293	0.366
3145	0.776	0.308	0.338	0.422
3150	0.766	0.293	0.324	0.410
3155	0.769	0.311	0.333	0.424
3160	0.808	0.386	0.397	0.484
3165	0.773	0.329	0.342	0.438
3170	0.780	0.326	0.348	0.436
3175	0.771	0.327	0.340	0.436
3180	0.813	0.419	0.412	0.509
3185	0.776	0.308	0.338	0.422
3190	0.759	0.304	0.323	0.418
3195	0.731	0.310	0.306	0.419
3200	0.763	0.322	0.331	0.432
3205	0.808	0.380	0.395	0.479
3210	0.820	0.388	0.415	0.485
3215	0.780	0.345	0.354	0.450
3220	0.808	0.405	0.401	0.498
3225	0.773	0.309	0.336	0.423
3230	0.745	0.232	0.286	0.364
3235	0.771	0.307	0.333	0.421
3240	0.782	0.335	0.352	0.442
3245	0.780	0.299	0.338	0.415
3250	0.794	0.371	0.376	0.471
3255	0.780	0.292	0.336	0.409
3260	0.790	0.360	0.369	0.462

3265	0.756	0.300	0.318	0.415
3270	0.797	0.377	0.381	0.475
3275	0.794	0.346	0.368	0.452
3280	0.787	0.303	0.346	0.417
3285	0.754	0.250	0.299	0.377
3290	0.776	0.327	0.345	0.437
3295	0.773	0.316	0.338	0.428
3300	0.797	0.332	0.368	0.440
3305	0.757	0.241	0.297	0.369
3310	0.750	0.239	0.292	0.368
3315	0.794	0.333	0.364	0.441
3320	0.776	0.239	0.311	0.365
3325	0.801	0.394	0.390	0.489
3330	0.754	0.264	0.304	0.388
3335	0.731	0.229	0.277	0.362
3340	0.761	0.266	0.310	0.389
3345	0.761	0.333	0.333	0.439
3350	0.787	0.342	0.360	0.448
3355	0.806	0.402	0.399	0.495
3360	0.804	0.362	0.386	0.464
3365	0.795	0.282	0.348	0.398
3370	0.764	0.337	0.338	0.443
3375	0.768	0.296	0.326	0.412
3380	0.789	0.351	0.364	0.455
3385	0.769	0.264	0.316	0.387
3390	0.761	0.273	0.313	0.395
3395	0.799	0.366	0.381	0.468
3400	0.795	0.355	0.373	0.459
3405	0.759	0.243	0.299	0.371
3410	0.776	0.379	0.360	0.473
3415	0.778	0.297	0.336	0.413
3420	0.778	0.276	0.328	0.396
3425	0.761	0.306	0.325	0.420
3430	0.761	0.245	0.301	0.373
3435	0.801	0.407	0.393	0.498
3440	0.759	0.277	0.313	0.398
3445	0.749	0.291	0.311	0.408
3450	0.745	0.267	0.299	0.390
3455	0.752	0.295	0.314	0.412
3460	0.769	0.278	0.321	0.398
3465	0.768	0.316	0.333	0.427
3470	0.801	0.350	0.378	0.455
3475	0.776	0.314	0.340	0.427

3480	0.789	0.370	0.370	0.470
3485	0.761	0.313	0.327	0.425
3490	0.742	0.235	0.285	0.366
3495	0.763	0.328	0.333	0.436
3500	0.773	0.276	0.323	0.396
3505	0.769	0.298	0.329	0.414
3510	0.771	0.294	0.329	0.411
3515	0.789	0.332	0.358	0.440
3520	0.797	0.383	0.383	0.480
3525	0.776	0.294	0.333	0.411
3530	0.782	0.328	0.350	0.438
3535	0.776	0.314	0.340	0.427
3540	0.795	0.368	0.377	0.468
3545	0.756	0.326	0.327	0.434
3550	0.792	0.317	0.357	0.429
3555	0.740	0.253	0.291	0.380
3560	0.768	0.302	0.329	0.417
3565	0.776	0.321	0.343	0.432
3570	0.769	0.357	0.348	0.457
3575	0.764	0.250	0.306	0.376
3580	0.769	0.324	0.338	0.434
3585	0.766	0.307	0.329	0.421
3590	0.789	0.364	0.368	0.465
3595	0.776	0.327	0.345	0.437
3600	0.764	0.324	0.333	0.433
3605	0.776	0.347	0.351	0.451
3610	0.773	0.323	0.340	0.433
3615	0.808	0.355	0.388	0.459
3620	0.761	0.300	0.322	0.415
3625	0.783	0.356	0.361	0.459
3630	0.769	0.311	0.333	0.424
3635	0.761	0.210	0.286	0.343
3640	0.785	0.300	0.344	0.415
3645	0.750	0.280	0.308	0.400
3650	0.780	0.319	0.345	0.430
3655	0.799	0.410	0.392	0.500
3660	0.818	0.403	0.416	0.498
3665	0.735	0.233	0.280	0.365
3670	0.773	0.316	0.338	0.428
3675	0.799	0.360	0.379	0.463
3680	0.730	0.261	0.288	0.386
3685	0.752	0.255	0.299	0.381
3690	0.778	0.297	0.336	0.413

3695	0.782	0.288	0.336	0.406
3700	0.754	0.291	0.314	0.408
3705	0.768	0.275	0.319	0.396
3710	0.766	0.326	0.335	0.435
3715	0.759	0.236	0.296	0.365
3720	0.754	0.243	0.296	0.372
3725	0.757	0.255	0.303	0.381
3730	0.764	0.298	0.324	0.414
3735	0.759	0.264	0.308	0.388
3740	0.792	0.362	0.371	0.464
3745	0.782	0.315	0.346	0.427
3750	0.783	0.331	0.353	0.439
3755	0.773	0.296	0.331	0.413
3760	0.768	0.316	0.333	0.427
3765	0.799	0.354	0.377	0.458
3770	0.794	0.378	0.378	0.476
3775	0.757	0.295	0.318	0.412
3780	0.757	0.262	0.306	0.386
3785	0.804	0.393	0.394	0.489
3790	0.783	0.317	0.348	0.429
3795	0.795	0.348	0.371	0.454
3800	0.771	0.300	0.331	0.416
3805	0.787	0.329	0.356	0.438
3810	0.766	0.313	0.331	0.426
3815	0.752	0.234	0.291	0.364
3820	0.768	0.289	0.324	0.407
3825	0.787	0.289	0.341	0.406
3830	0.795	0.348	0.371	0.454
3835	0.768	0.329	0.338	0.437
3840	0.769	0.305	0.331	0.419
3845	0.790	0.379	0.374	0.476
3850	0.780	0.312	0.343	0.425
3855	0.794	0.390	0.381	0.485
3860	0.763	0.262	0.309	0.386
3865	0.776	0.281	0.328	0.400
3870	0.782	0.288	0.336	0.406
3875	0.756	0.203	0.279	0.338
3880	0.766	0.280	0.319	0.400
3885	0.782	0.360	0.360	0.462
3890	0.752	0.248	0.297	0.376
3895	0.783	0.311	0.346	0.424
3900	0.757	0.302	0.321	0.417
3905	0.771	0.287	0.326	0.405

3910	0.797	0.338	0.370	0.445
3915	0.754	0.257	0.301	0.383
3920	0.776	0.327	0.345	0.437
3925	0.752	0.241	0.294	0.370
3930	0.795	0.342	0.369	0.449
3935	0.775	0.325	0.342	0.435
3940	0.787	0.336	0.358	0.443
3945	0.802	0.403	0.394	0.496
3950	0.761	0.231	0.295	0.361
3955	0.756	0.252	0.301	0.379
3960	0.792	0.330	0.362	0.439
3965	0.776	0.334	0.347	0.442
3970	0.804	0.399	0.396	0.493
3975	0.759	0.257	0.305	0.382
3980	0.756	0.259	0.303	0.384
3985	0.776	0.321	0.343	0.432
3990	0.766	0.273	0.317	0.395
3995	0.769	0.285	0.324	0.404
4000	0.769	0.298	0.329	0.414
4005	0.738	0.245	0.287	0.373
4010	0.778	0.368	0.359	0.467
4015	0.780	0.332	0.350	0.441
4020	0.756	0.246	0.298	0.373
4025	0.799	0.347	0.375	0.453
4030	0.745	0.253	0.294	0.380
4035	0.776	0.353	0.353	0.456
4040	0.754	0.304	0.319	0.418
4045	0.785	0.320	0.351	0.431
4050	0.773	0.303	0.333	0.418
4055	0.768	0.329	0.338	0.437
4060	0.776	0.321	0.343	0.432
4065	0.771	0.273	0.321	0.394
4070	0.761	0.286	0.318	0.405
4075	0.768	0.289	0.324	0.407
4080	0.783	0.337	0.355	0.444
4085	0.756	0.286	0.314	0.405
4090	0.776	0.294	0.333	0.411
4095	0.756	0.232	0.292	0.362
4100	0.768	0.275	0.319	0.396
4105	0.815	0.379	0.405	0.478
4110	0.821	0.409	0.423	0.502
4115	0.771	0.294	0.329	0.411
4120	0.761	0.266	0.310	0.389

4125	0.782	0.328	0.350	0.438
4130	0.808	0.386	0.397	0.484
4135	0.783	0.344	0.357	0.449
4140	0.782	0.341	0.354	0.447
4145	0.761	0.286	0.318	0.405
4150	0.789	0.318	0.354	0.430
4155	0.754	0.291	0.314	0.408
4160	0.738	0.217	0.275	0.352
4165	0.801	0.350	0.378	0.455
4170	0.787	0.355	0.364	0.458
4175	0.775	0.251	0.315	0.375
4180	0.745	0.273	0.302	0.395
4185	0.795	0.275	0.345	0.392
4190	0.757	0.262	0.306	0.386
4195	0.764	0.318	0.331	0.429
4200	0.780	0.364	0.359	0.464
4205	0.790	0.334	0.361	0.442
4210	0.766	0.313	0.331	0.426
4215	0.782	0.295	0.338	0.411
4220	0.787	0.322	0.353	0.433
4225	0.754	0.264	0.304	0.388
4230	0.775	0.305	0.336	0.420
4235	0.771	0.320	0.338	0.431
4240	0.778	0.330	0.347	0.439
4245	0.783	0.311	0.346	0.424
4250	0.761	0.300	0.322	0.415
4255	0.785	0.340	0.357	0.446
4260	0.790	0.366	0.371	0.467
4265	0.764	0.257	0.309	0.382
4270	0.775	0.271	0.323	0.393
4275	0.795	0.342	0.369	0.449
4280	0.752	0.220	0.285	0.353
4285	0.792	0.330	0.362	0.439
4290	0.747	0.241	0.291	0.371
4295	0.750	0.280	0.308	0.400
4300	0.754	0.277	0.309	0.398
4305	0.785	0.320	0.351	0.431
4310	0.778	0.323	0.345	0.434
4315	0.776	0.314	0.340	0.427
4320	0.763	0.262	0.309	0.386
4325	0.759	0.243	0.299	0.371
4330	0.787	0.282	0.339	0.400
4335	0.801	0.382	0.387	0.480

4340	0.771	0.346	0.346	0.450
4345	0.780	0.326	0.348	0.436
4350	0.740	0.240	0.286	0.370
4355	0.749	0.271	0.303	0.393
4360	0.783	0.356	0.361	0.459
4365	0.737	0.270	0.295	0.392
4370	0.766	0.246	0.305	0.372
4375	0.761	0.306	0.325	0.420
4380	0.780	0.332	0.350	0.441
4385	0.782	0.354	0.358	0.457
4390	0.769	0.278	0.321	0.398
4395	0.785	0.346	0.359	0.451
4400	0.823	0.460	0.435	0.541
4405	0.787	0.336	0.358	0.443
4410	0.790	0.321	0.357	0.432
4415	0.769	0.271	0.319	0.393
4420	0.782	0.315	0.346	0.427
4425	0.799	0.373	0.382	0.473
4430	0.775	0.344	0.349	0.449
4435	0.761	0.266	0.310	0.389
4440	0.747	0.296	0.311	0.411
4445	0.794	0.326	0.362	0.436
4450	0.757	0.309	0.323	0.421
4455	0.738	0.285	0.302	0.403
4460	0.764	0.298	0.324	0.414
4465	0.789	0.325	0.356	0.435
4470	0.789	0.383	0.373	0.479
4475	0.776	0.287	0.331	0.406
4480	0.766	0.273	0.317	0.395
4485	0.766	0.300	0.327	0.416
4490	0.782	0.328	0.350	0.438
4495	0.789	0.376	0.372	0.474
4500	0.768	0.219	0.295	0.350
4505	0.771	0.320	0.338	0.431
4510	0.764	0.278	0.317	0.398
4515	0.776	0.301	0.336	0.416
4520	0.778	0.297	0.336	0.413
4525	0.773	0.262	0.318	0.385
4530	0.787	0.322	0.353	0.433
4535	0.776	0.308	0.338	0.422
4540	0.775	0.258	0.317	0.381
4545	0.752	0.295	0.314	0.412
4550	0.780	0.279	0.331	0.398

4555	0.773	0.316	0.338	0.428
4560	0.795	0.374	0.379	0.473
4565	0.783	0.317	0.348	0.429
4570	0.792	0.343	0.366	0.450
4575	0.768	0.269	0.316	0.391
4580	0.801	0.413	0.395	0.502
4585	0.778	0.310	0.341	0.423
4590	0.738	0.174	0.255	0.317
4595	0.794	0.352	0.370	0.457
4600	0.773	0.349	0.348	0.452
4605	0.750	0.253	0.297	0.379
4610	0.757	0.289	0.316	0.407
4615	0.750	0.266	0.303	0.390
4620	0.768	0.309	0.331	0.422
4625	0.749	0.216	0.281	0.350
4630	0.747	0.228	0.285	0.360
4635	0.820	0.388	0.415	0.485
4640	0.738	0.238	0.284	0.368
4645	0.769	0.278	0.321	0.398
4650	0.775	0.298	0.333	0.414
4655	0.763	0.282	0.317	0.402
4660	0.783	0.304	0.344	0.419
4665	0.776	0.334	0.347	0.442
4670	0.775	0.305	0.336	0.420
4675	0.801	0.331	0.372	0.439
4680	0.802	0.333	0.375	0.441
4685	0.764	0.324	0.333	0.433
4690	0.768	0.255	0.311	0.380
4695	0.745	0.260	0.297	0.385
4700	0.778	0.310	0.341	0.423
4705	0.778	0.276	0.328	0.396
4710	0.764	0.264	0.312	0.387
4715	0.799	0.404	0.390	0.496
4720	0.728	0.211	0.268	0.349
4725	0.778	0.263	0.323	0.385
4730	0.775	0.325	0.342	0.435
4735	0.768	0.275	0.319	0.396
4740	0.782	0.341	0.354	0.447
4745	0.795	0.302	0.356	0.416
4750	0.778	0.336	0.349	0.443
4755	0.792	0.375	0.375	0.474
4760	0.776	0.210	0.297	0.338
4765	0.790	0.308	0.352	0.421

4770	0.785	0.327	0.353	0.436
4775	0.733	0.224	0.276	0.358
4780	0.754	0.284	0.312	0.403
4785	0.764	0.236	0.300	0.364
4790	0.754	0.229	0.290	0.360
4795	0.759	0.257	0.305	0.382
4800	0.773	0.289	0.328	0.407
4805	0.764	0.243	0.303	0.370
4810	0.763	0.282	0.317	0.402
4815	0.771	0.307	0.333	0.421
4820	0.738	0.188	0.262	0.329
4825	0.761	0.280	0.315	0.400
4830	0.783	0.317	0.348	0.429
4835	0.785	0.327	0.353	0.436
4840	0.783	0.317	0.348	0.429
4845	0.769	0.285	0.324	0.404
4850	0.783	0.350	0.359	0.454
4855	0.759	0.271	0.310	0.393
4860	0.769	0.229	0.301	0.357
4865	0.754	0.277	0.309	0.398
4870	0.771	0.294	0.329	0.411
4875	0.744	0.264	0.297	0.388
4880	0.747	0.248	0.293	0.376
4885	0.815	0.397	0.409	0.493
4890	0.780	0.292	0.336	0.409
4895	0.773	0.289	0.328	0.407
4900	0.783	0.311	0.346	0.424
4905	0.763	0.248	0.304	0.374
4910	0.789	0.338	0.360	0.445
4915	0.773	0.276	0.323	0.396
4920	0.778	0.336	0.349	0.443
4925	0.769	0.236	0.304	0.364
4930	0.740	0.198	0.268	0.336
4935	0.749	0.271	0.303	0.393
4940	0.776	0.321	0.343	0.432
4945	0.759	0.298	0.320	0.414
4950	0.792	0.388	0.378	0.483
4955	0.780	0.319	0.345	0.430
4960	0.785	0.320	0.351	0.431
4965	0.764	0.250	0.306	0.376
4970	0.816	0.431	0.419	0.518
4975	0.754	0.277	0.309	0.398
4980	0.768	0.255	0.311	0.380

4985	0.778	0.283	0.331	0.402
4990	0.782	0.261	0.325	0.382
4995	0.780	0.251	0.319	0.374
5000	0.778	0.290	0.333	0.407
5005	0.764	0.271	0.314	0.393
5010	0.761	0.259	0.307	0.384
5015	0.768	0.269	0.316	0.391
5020	0.745	0.197	0.270	0.335
5025	0.752	0.241	0.294	0.370
5030	0.775	0.292	0.331	0.409
5035	0.794	0.352	0.370	0.457
5040	0.750	0.211	0.279	0.345
5045	0.782	0.302	0.341	0.417
5050	0.785	0.320	0.351	0.431
5055	0.785	0.320	0.351	0.431
5060	0.747	0.269	0.301	0.392
5065	0.768	0.302	0.329	0.417
5070	0.745	0.267	0.299	0.390
5075	0.766	0.287	0.322	0.405
5080	0.775	0.351	0.351	0.454
5085	0.768	0.302	0.329	0.417
5090	0.759	0.311	0.325	0.423
5095	0.780	0.292	0.336	0.409
5100	0.778	0.290	0.333	0.407
5105	0.797	0.318	0.364	0.429
5110	0.764	0.284	0.319	0.404
5115	0.780	0.299	0.338	0.415
5120	0.776	0.321	0.343	0.432
5125	0.744	0.244	0.289	0.373
5130	0.780	0.319	0.345	0.430
5135	0.752	0.234	0.291	0.364
5140	0.782	0.308	0.343	0.422
5145	0.785	0.365	0.365	0.466
5150	0.816	0.406	0.414	0.500
5155	0.744	0.251	0.292	0.378
5160	0.752	0.241	0.294	0.370
5165	0.780	0.312	0.343	0.425
5170	0.757	0.282	0.313	0.402
5175	0.783	0.270	0.331	0.390
5180	0.768	0.296	0.326	0.412
5185	0.806	0.390	0.396	0.486
5190	0.780	0.292	0.336	0.409
5195	0.797	0.389	0.385	0.485

5200	0.775	0.292	0.331	0.409
5205	0.802	0.397	0.393	0.491
5210	0.795	0.342	0.369	0.449
5215	0.776	0.314	0.340	0.427
5220	0.738	0.210	0.272	0.346
5225	0.766	0.307	0.329	0.421
5230	0.775	0.298	0.333	0.414
5235	0.769	0.318	0.336	0.429
5240	0.794	0.339	0.366	0.447
5245	0.749	0.264	0.301	0.388
5250	0.778	0.303	0.338	0.418
5255	0.769	0.291	0.326	0.409
5260	0.756	0.225	0.289	0.356
5265	0.763	0.262	0.309	0.386
5270	0.780	0.279	0.331	0.398
5275	0.787	0.322	0.353	0.433
5280	0.773	0.296	0.331	0.413
5285	0.782	0.315	0.346	0.427
5290	0.726	0.230	0.274	0.363
5295	0.764	0.298	0.324	0.414
5300	0.773	0.355	0.350	0.456
5305	0.775	0.305	0.336	0.420
5310	0.806	0.371	0.391	0.472
5315	0.773	0.283	0.326	0.402
5320	0.775	0.325	0.342	0.435
5325	0.773	0.276	0.323	0.396
5330	0.766	0.217	0.293	0.348
5335	0.790	0.354	0.367	0.457
5340	0.737	0.229	0.279	0.361
5345	0.802	0.333	0.375	0.441
5350	0.752	0.309	0.319	0.421
5355	0.754	0.264	0.304	0.388
5360	0.776	0.267	0.323	0.389
5365	0.768	0.296	0.326	0.412
5370	0.771	0.273	0.321	0.394
5375	0.787	0.309	0.349	0.423
5380	0.806	0.384	0.394	0.481
5385	0.794	0.359	0.372	0.462
5390	0.802	0.340	0.377	0.447
5395	0.808	0.355	0.388	0.459
5400	0.769	0.291	0.326	0.409
5405	0.773	0.355	0.350	0.456
5410	0.778	0.303	0.338	0.418

5415	0.756	0.246	0.298	0.373
5420	0.757	0.255	0.303	0.381
5425	0.764	0.278	0.317	0.398
5430	0.752	0.248	0.297	0.376
5435	0.768	0.262	0.313	0.385
5440	0.780	0.251	0.319	0.374
5445	0.792	0.337	0.364	0.444
5450	0.763	0.248	0.304	0.374
5455	0.764	0.264	0.312	0.387
5460	0.775	0.265	0.320	0.387
5465	0.775	0.344	0.349	0.449
5470	0.776	0.301	0.336	0.416
5475	0.785	0.300	0.344	0.415
5480	0.797	0.377	0.381	0.475
5485	0.776	0.294	0.333	0.411
5490	0.776	0.301	0.336	0.416
5495	0.773	0.323	0.340	0.433
5500	0.768	0.335	0.340	0.442
5505	0.792	0.304	0.352	0.417
5510	0.790	0.328	0.359	0.437
5515	0.789	0.305	0.349	0.419
5520	0.757	0.248	0.300	0.375
5525	0.794	0.359	0.372	0.462
5530	0.769	0.318	0.336	0.429
5535	0.747	0.255	0.296	0.381
5540	0.787	0.322	0.353	0.433
5545	0.792	0.310	0.355	0.423
5550	0.775	0.312	0.338	0.425
5555	0.771	0.246	0.310	0.371
5560	0.780	0.326	0.348	0.436
5565	0.759	0.236	0.296	0.365
5570	0.771	0.307	0.333	0.421
5575	0.773	0.303	0.333	0.418
5580	0.766	0.273	0.317	0.395
5585	0.802	0.340	0.377	0.447
5590	0.763	0.315	0.329	0.427
5595	0.771	0.232	0.303	0.359
5600	0.792	0.324	0.359	0.434
5605	0.768	0.275	0.319	0.396
5610	0.773	0.303	0.333	0.418
5615	0.787	0.355	0.364	0.458
5620	0.790	0.360	0.369	0.462
5625	0.778	0.283	0.331	0.402

5630	0.776	0.232	0.308	0.358
5635	0.785	0.313	0.348	0.426
5640	0.778	0.323	0.345	0.434
5645	0.752	0.255	0.299	0.381
5650	0.761	0.231	0.295	0.361
5655	0.780	0.312	0.343	0.425
5660	0.756	0.232	0.292	0.362
5665	0.744	0.230	0.284	0.362
5670	0.730	0.213	0.269	0.350
5675	0.787	0.316	0.351	0.428
5680	0.778	0.263	0.323	0.385
5685	0.769	0.278	0.321	0.398
5690	0.747	0.255	0.296	0.381
5695	0.763	0.255	0.307	0.380
5700	0.778	0.330	0.347	0.439
5705	0.771	0.253	0.313	0.377
5710	0.794	0.352	0.370	0.457
5715	0.750	0.266	0.303	0.390
5720	0.773	0.316	0.338	0.428
5725	0.754	0.277	0.309	0.398
5730	0.787	0.336	0.358	0.443
5735	0.768	0.322	0.336	0.432
5740	0.759	0.250	0.302	0.377
5745	0.775	0.285	0.328	0.404
5750	0.761	0.252	0.304	0.378
5755	0.757	0.241	0.297	0.369
5760	0.785	0.327	0.353	0.436
5765	0.783	0.331	0.353	0.439
5770	0.795	0.355	0.373	0.459
5775	0.802	0.366	0.385	0.467
5780	0.740	0.267	0.296	0.390
5785	0.749	0.244	0.293	0.372
5790	0.756	0.259	0.303	0.384
5795	0.740	0.260	0.294	0.385
5800	0.769	0.298	0.329	0.414
5805	0.773	0.289	0.328	0.407
5810	0.783	0.304	0.344	0.419
5815	0.771	0.273	0.321	0.394
5820	0.782	0.295	0.338	0.411
5825	0.769	0.236	0.304	0.364
5830	0.749	0.264	0.301	0.388
5835	0.761	0.217	0.289	0.349
5840	0.749	0.244	0.293	0.372

5845	0.787	0.296	0.344	0.411
5850	0.811	0.404	0.406	0.498
5855	0.761	0.273	0.313	0.395
5860	0.756	0.259	0.303	0.384
5865	0.816	0.394	0.411	0.490
5870	0.771	0.333	0.342	0.441
5875	0.752	0.241	0.294	0.370
5880	0.787	0.275	0.336	0.394
5885	0.757	0.213	0.285	0.346
5890	0.745	0.239	0.289	0.369
5895	0.792	0.343	0.366	0.450
5900	0.789	0.278	0.339	0.396
5905	0.766	0.300	0.327	0.416
5910	0.759	0.222	0.290	0.353
5915	0.799	0.373	0.382	0.473
5920	0.769	0.285	0.324	0.404
5925	0.771	0.273	0.321	0.394
5930	0.766	0.238	0.302	0.366
5935	0.804	0.336	0.378	0.443
5940	0.761	0.252	0.304	0.378
5945	0.754	0.229	0.290	0.360
5950	0.761	0.210	0.286	0.343
5955	0.790	0.385	0.376	0.481
5960	0.769	0.243	0.307	0.370
5965	0.797	0.345	0.372	0.451
5970	0.790	0.321	0.357	0.432
5975	0.787	0.309	0.349	0.423
5980	0.783	0.317	0.348	0.429
5985	0.768	0.289	0.324	0.407
5990	0.769	0.278	0.321	0.398
5995	0.782	0.288	0.336	0.406
6000	0.780	0.292	0.336	0.409
6005	0.761	0.231	0.295	0.361
6010	0.783	0.304	0.344	0.419
6015	0.747	0.228	0.285	0.360
6020	0.749	0.209	0.277	0.344
6025	0.749	0.194	0.271	0.332
6030	0.773	0.303	0.333	0.418
6035	0.794	0.313	0.358	0.425
6040	0.811	0.348	0.391	0.452
6045	0.769	0.271	0.319	0.393
6050	0.795	0.355	0.373	0.459
6055	0.785	0.300	0.344	0.415

6060	0.783	0.311	0.346	0.424
6065	0.785	0.340	0.357	0.446
6070	0.744	0.291	0.307	0.408
6075	0.763	0.268	0.312	0.391
6080	0.744	0.223	0.281	0.357
6085	0.771	0.232	0.303	0.359
6090	0.769	0.285	0.324	0.404
6095	0.795	0.348	0.371	0.454
6100	0.790	0.341	0.363	0.447
6105	0.794	0.359	0.372	0.462
6110	0.768	0.212	0.292	0.343
6115	0.782	0.335	0.352	0.442
6120	0.787	0.275	0.336	0.394
6125	0.754	0.264	0.304	0.388
6130	0.785	0.300	0.344	0.415
6135	0.783	0.297	0.341	0.413
6140	0.771	0.267	0.318	0.389
6145	0.783	0.270	0.331	0.390
6150	0.773	0.276	0.323	0.396
6155	0.768	0.282	0.321	0.402
6160	0.756	0.203	0.279	0.338
6165	0.775	0.312	0.338	0.425
6170	0.769	0.278	0.321	0.398
6175	0.797	0.325	0.366	0.435
6180	0.790	0.334	0.361	0.442
6185	0.783	0.311	0.346	0.424
6190	0.778	0.276	0.328	0.396
6195	0.787	0.316	0.351	0.428
6200	0.787	0.316	0.351	0.428
6205	0.775	0.285	0.328	0.404
6210	0.792	0.330	0.362	0.439
6215	0.750	0.253	0.297	0.379
6220	0.775	0.285	0.328	0.404
6225	0.787	0.342	0.360	0.448
6230	0.813	0.407	0.409	0.500
6235	0.761	0.266	0.310	0.389
6240	0.771	0.327	0.340	0.436
6245	0.783	0.270	0.331	0.390
6250	0.761	0.238	0.299	0.367
6255	0.782	0.348	0.356	0.452
6260	0.768	0.329	0.338	0.437
6265	0.797	0.325	0.366	0.435
6270	0.764	0.304	0.327	0.419

6275	0.783	0.311	0.346	0.424
6280	0.737	0.242	0.285	0.372
6285	0.790	0.334	0.361	0.442
6290	0.795	0.335	0.367	0.443
6295	0.787	0.336	0.358	0.443
6300	0.745	0.218	0.280	0.352
6305	0.769	0.331	0.340	0.439
6310	0.771	0.300	0.331	0.416
6315	0.795	0.322	0.363	0.433
6320	0.766	0.246	0.305	0.372
6325	0.799	0.347	0.375	0.453
6330	0.775	0.285	0.328	0.404
6335	0.789	0.332	0.358	0.440
6340	0.790	0.354	0.367	0.457
6345	0.782	0.254	0.322	0.376
6350	0.756	0.225	0.289	0.356
6355	0.754	0.277	0.309	0.398
6360	0.756	0.239	0.295	0.368
6365	0.756	0.239	0.295	0.368
6370	0.776	0.340	0.349	0.446
6375	0.813	0.344	0.393	0.449
6380	0.789	0.312	0.352	0.425
6385	0.780	0.319	0.345	0.430
6390	0.757	0.275	0.311	0.397
6395	0.776	0.321	0.343	0.432
6400	0.782	0.281	0.333	0.400
6405	0.763	0.262	0.309	0.386
6410	0.782	0.295	0.338	0.411
6415	0.776	0.334	0.347	0.442
6420	0.768	0.255	0.311	0.380
6425	0.773	0.234	0.306	0.361
6430	0.799	0.328	0.369	0.437
6435	0.764	0.257	0.309	0.382
6440	0.771	0.300	0.331	0.416
6445	0.750	0.204	0.276	0.339
6450	0.761	0.280	0.315	0.400
6455	0.795	0.393	0.384	0.487
6460	0.795	0.302	0.356	0.416
6465	0.735	0.220	0.275	0.354
6470	0.771	0.253	0.313	0.377
6475	0.785	0.340	0.357	0.446
6480	0.764	0.284	0.319	0.404
6485	0.771	0.267	0.318	0.389

6490	0.771	0.300	0.331	0.416
6495	0.789	0.312	0.352	0.425
6500	0.771	0.273	0.321	0.394
6505	0.780	0.292	0.336	0.409
6510	0.801	0.413	0.395	0.502
6515	0.789	0.325	0.356	0.435
6520	0.775	0.318	0.340	0.430
6525	0.764	0.311	0.329	0.424
6530	0.797	0.338	0.370	0.445
6535	0.742	0.214	0.276	0.349
6540	0.778	0.290	0.333	0.407
6545	0.764	0.284	0.319	0.404
6550	0.769	0.278	0.321	0.398
6555	0.771	0.300	0.331	0.416
6560	0.747	0.199	0.272	0.336
6565	0.780	0.306	0.341	0.420
6570	0.754	0.284	0.312	0.403
6575	0.752	0.227	0.288	0.359
6580	0.775	0.285	0.328	0.404
6585	0.768	0.282	0.321	0.402
6590	0.785	0.340	0.357	0.446
6595	0.775	0.285	0.328	0.404
6600	0.763	0.268	0.312	0.391
6605	0.731	0.173	0.252	0.317
6610	0.773	0.303	0.333	0.418
6615	0.789	0.325	0.356	0.435
6620	0.783	0.311	0.346	0.424
6625	0.782	0.328	0.350	0.438
6630	0.763	0.234	0.298	0.363
6635	0.773	0.309	0.336	0.423
6640	0.763	0.255	0.307	0.380
6645	0.776	0.253	0.317	0.377
6650	0.775	0.351	0.351	0.454
6655	0.775	0.305	0.336	0.420
6660	0.780	0.332	0.350	0.441
6665	0.783	0.297	0.341	0.413
6670	0.735	0.206	0.268	0.343
6675	0.780	0.312	0.343	0.425
6680	0.794	0.352	0.370	0.457
6685	0.749	0.244	0.293	0.372
6690	0.773	0.289	0.328	0.407
6695	0.775	0.229	0.305	0.356
6700	0.785	0.286	0.339	0.404

6705	0.761	0.195	0.279	0.330
6710	0.771	0.273	0.321	0.394
6715	0.799	0.347	0.375	0.453
6720	0.759	0.291	0.318	0.409
6725	0.769	0.311	0.333	0.424
6730	0.801	0.350	0.378	0.455
6735	0.778	0.303	0.338	0.418
6740	0.771	0.273	0.321	0.394
6745	0.733	0.211	0.270	0.347
6750	0.782	0.302	0.341	0.417
6755	0.782	0.254	0.322	0.376
6760	0.794	0.326	0.362	0.436
6765	0.764	0.284	0.319	0.404
6770	0.735	0.184	0.259	0.326
6775	0.771	0.260	0.315	0.383
6780	0.802	0.384	0.390	0.482
6785	0.789	0.318	0.354	0.430
6790	0.756	0.286	0.314	0.405
6795	0.790	0.354	0.367	0.457
6800	0.769	0.271	0.319	0.393
6805	0.768	0.322	0.336	0.432
6810	0.778	0.336	0.349	0.443
6815	0.785	0.320	0.351	0.431
6820	0.790	0.301	0.350	0.415
6825	0.797	0.408	0.389	0.498
6830	0.757	0.282	0.313	0.402
6835	0.773	0.329	0.342	0.438
6840	0.783	0.337	0.355	0.444
6845	0.809	0.383	0.398	0.481
6850	0.785	0.307	0.346	0.421
6855	0.787	0.316	0.351	0.428
6860	0.742	0.242	0.288	0.371
6865	0.787	0.316	0.351	0.428
6870	0.789	0.264	0.333	0.384
6875	0.780	0.279	0.331	0.398
6880	0.750	0.273	0.305	0.395
6885	0.785	0.293	0.341	0.410
6890	0.750	0.239	0.292	0.368
6895	0.763	0.241	0.301	0.369
6900	0.776	0.287	0.331	0.406
6905	0.771	0.267	0.318	0.389
6910	0.763	0.255	0.307	0.380
6915	0.782	0.295	0.338	0.411

6920	0.787	0.336	0.358	0.443
6925	0.768	0.227	0.298	0.356
6930	0.795	0.368	0.377	0.468
6935	0.790	0.288	0.345	0.404
6940	0.742	0.163	0.252	0.307
6945	0.764	0.243	0.303	0.370
6950	0.783	0.304	0.344	0.419
6955	0.792	0.362	0.371	0.464
6960	0.795	0.368	0.377	0.468
6965	0.764	0.264	0.312	0.387
6970	0.789	0.338	0.360	0.445
6975	0.764	0.257	0.309	0.382
6980	0.745	0.211	0.277	0.347
6985	0.754	0.229	0.290	0.360
6990	0.757	0.268	0.308	0.391
6995	0.763	0.275	0.315	0.396
7000	0.799	0.341	0.373	0.448
7005	0.749	0.244	0.293	0.372
7010	0.768	0.296	0.326	0.412
7015	0.771	0.287	0.326	0.405
7020	0.750	0.260	0.300	0.385
7025	0.761	0.280	0.315	0.400
7030	0.787	0.261	0.330	0.382
7035	0.744	0.244	0.289	0.373
7040	0.790	0.314	0.354	0.427
7045	0.769	0.285	0.324	0.404
7050	0.749	0.216	0.281	0.350
7055	0.775	0.258	0.317	0.381
7060	0.769	0.264	0.316	0.387
7065	0.787	0.309	0.349	0.423
7070	0.745	0.232	0.286	0.364
7075	0.782	0.315	0.346	0.427
7080	0.778	0.317	0.343	0.429
7085	0.818	0.353	0.404	0.456
7090	0.825	0.421	0.431	0.512
7095	0.724	0.207	0.264	0.346
7100	0.775	0.271	0.323	0.393
7105	0.771	0.294	0.329	0.411
7110	0.785	0.307	0.346	0.421
7115	0.752	0.255	0.299	0.381
7120	0.724	0.172	0.248	0.318
7125	0.771	0.273	0.321	0.394
7130	0.744	0.264	0.297	0.388

7135	0.768	0.269	0.316	0.391
7140	0.792	0.317	0.357	0.429
7145	0.780	0.292	0.336	0.409
7150	0.797	0.332	0.368	0.440
7155	0.754	0.236	0.293	0.366
7160	0.738	0.210	0.272	0.346
7165	0.790	0.328	0.359	0.437
7170	0.768	0.335	0.340	0.442
7175	0.754	0.250	0.299	0.377
7180	0.771	0.280	0.324	0.400
7185	0.771	0.267	0.318	0.389
7190	0.789	0.332	0.358	0.440
7195	0.749	0.250	0.295	0.378
7200	0.764	0.264	0.312	0.387
7205	0.778	0.317	0.343	0.429
7210	0.750	0.225	0.286	0.357
7215	0.756	0.225	0.289	0.356
7220	0.771	0.327	0.340	0.436
7225	0.761	0.252	0.304	0.378
7230	0.773	0.289	0.328	0.407
7235	0.787	0.254	0.327	0.376
7240	0.775	0.305	0.336	0.420
7245	0.747	0.234	0.288	0.365
7250	0.761	0.252	0.304	0.378
7255	0.773	0.276	0.323	0.396
7260	0.726	0.216	0.269	0.352
7265	0.808	0.367	0.392	0.469
7270	0.794	0.326	0.362	0.436
7275	0.769	0.298	0.329	0.414
7280	0.761	0.286	0.318	0.405
7285	0.735	0.191	0.262	0.332
7290	0.799	0.354	0.377	0.458
7295	0.769	0.285	0.324	0.404
7300	0.794	0.346	0.368	0.452
7305	0.756	0.239	0.295	0.368
7310	0.754	0.277	0.309	0.398
7315	0.778	0.310	0.341	0.423
7320	0.782	0.295	0.338	0.411
7325	0.768	0.255	0.311	0.380
7330	0.766	0.293	0.324	0.410
7335	0.757	0.198	0.278	0.333
7340	0.769	0.291	0.326	0.409
7345	0.756	0.246	0.298	0.373

7350	0.749	0.264	0.301	0.388
7355	0.750	0.280	0.308	0.400
7360	0.752	0.206	0.278	0.341
7365	0.783	0.311	0.346	0.424
7370	0.771	0.239	0.306	0.365
7375	0.769	0.311	0.333	0.424
7380	0.778	0.323	0.345	0.434
7385	0.783	0.344	0.357	0.449
7390	0.759	0.298	0.320	0.414
7395	0.763	0.262	0.309	0.386
7400	0.763	0.262	0.309	0.386
7405	0.756	0.239	0.295	0.368
7410	0.769	0.278	0.321	0.398
7415	0.808	0.399	0.400	0.493
7420	0.799	0.366	0.381	0.468
7425	0.783	0.297	0.341	0.413
7430	0.768	0.269	0.316	0.391
7435	0.763	0.275	0.315	0.396
7440	0.737	0.215	0.273	0.350
7445	0.769	0.271	0.319	0.393
7450	0.768	0.282	0.321	0.402
7455	0.749	0.264	0.301	0.388
7460	0.757	0.241	0.297	0.369
7465	0.794	0.320	0.360	0.431
7470	0.761	0.280	0.315	0.400
7475	0.776	0.274	0.326	0.394
7480	0.769	0.337	0.342	0.444
7485	0.789	0.312	0.352	0.425
7490	0.766	0.287	0.322	0.405
7495	0.782	0.281	0.333	0.400
7500	0.766	0.273	0.317	0.395
7505	0.759	0.236	0.296	0.365
7510	0.783	0.297	0.341	0.413
7515	0.780	0.279	0.331	0.398
7520	0.757	0.248	0.300	0.375
7525	0.787	0.303	0.346	0.417
7530	0.761	0.313	0.327	0.425
7535	0.763	0.220	0.291	0.351
7540	0.780	0.299	0.338	0.415
7545	0.782	0.302	0.341	0.417
7550	0.790	0.373	0.372	0.472
7555	0.735	0.191	0.262	0.332
7560	0.761	0.306	0.325	0.420

7565	0.754	0.250	0.299	0.377
7570	0.750	0.218	0.283	0.351
7575	0.771	0.267	0.318	0.389
7580	0.776	0.287	0.331	0.406
7585	0.769	0.291	0.326	0.409
7590	0.749	0.201	0.274	0.338
7595	0.780	0.265	0.325	0.386
7600	0.771	0.307	0.333	0.421
7605	0.811	0.404	0.406	0.498
7610	0.797	0.318	0.364	0.429
7615	0.799	0.315	0.364	0.426
7620	0.792	0.330	0.362	0.439
7625	0.759	0.250	0.302	0.377
7630	0.768	0.282	0.321	0.402
7635	0.773	0.255	0.315	0.379
7640	0.776	0.308	0.338	0.422
7645	0.766	0.266	0.314	0.389
7650	0.787	0.254	0.327	0.376
7655	0.785	0.266	0.331	0.386
7660	0.778	0.276	0.328	0.396
7665	0.775	0.258	0.317	0.381
7670	0.792	0.343	0.366	0.450
7675	0.782	0.348	0.356	0.452
7680	0.745	0.267	0.299	0.390
7685	0.769	0.250	0.310	0.376
7690	0.763	0.262	0.309	0.386
7695	0.790	0.347	0.365	0.452
7700	0.766	0.252	0.308	0.378
7705	0.768	0.275	0.319	0.396
7710	0.794	0.326	0.362	0.436
7715	0.780	0.312	0.343	0.425
7720	0.782	0.302	0.341	0.417
7725	0.750	0.246	0.295	0.374
7730	0.792	0.324	0.359	0.434
7735	0.782	0.295	0.338	0.411
7740	0.757	0.255	0.303	0.381
7745	0.763	0.227	0.295	0.357
7750	0.789	0.332	0.358	0.440
7755	0.792	0.304	0.352	0.417
7760	0.775	0.285	0.328	0.404
7765	0.766	0.252	0.308	0.378
7770	0.769	0.271	0.319	0.393
7775	0.785	0.313	0.348	0.426

7780	0.771	0.246	0.310	0.371
7785	0.785	0.333	0.355	0.441
7790	0.766	0.246	0.305	0.372
7795	0.769	0.285	0.324	0.404
7800	0.780	0.312	0.343	0.425
7805	0.782	0.302	0.341	0.417
7810	0.806	0.371	0.391	0.472
7815	0.756	0.286	0.314	0.405
7820	0.789	0.312	0.352	0.425
7825	0.790	0.341	0.363	0.447
7830	0.783	0.324	0.350	0.434
7835	0.773	0.289	0.328	0.407
7840	0.783	0.291	0.339	0.408
7845	0.756	0.259	0.303	0.384
7850	0.792	0.350	0.368	0.455
7855	0.794	0.365	0.374	0.466
7860	0.750	0.196	0.273	0.333
7865	0.797	0.312	0.361	0.424
7870	0.789	0.312	0.352	0.425
7875	0.783	0.304	0.344	0.419
7880	0.794	0.300	0.353	0.414
7885	0.769	0.271	0.319	0.393
7890	0.789	0.285	0.342	0.402
7895	0.816	0.406	0.414	0.500
7900	0.768	0.269	0.316	0.391
7905	0.787	0.316	0.351	0.428
7910	0.823	0.418	0.427	0.510
7915	0.789	0.305	0.349	0.419
7920	0.790	0.360	0.369	0.462
7925	0.795	0.368	0.377	0.468
7930	0.757	0.220	0.288	0.352
7935	0.768	0.219	0.295	0.350
7940	0.802	0.327	0.373	0.436
7945	0.780	0.299	0.338	0.415
7950	0.809	0.389	0.400	0.486
7955	0.780	0.286	0.333	0.404
7960	0.790	0.308	0.352	0.421
7965	0.771	0.273	0.321	0.394
7970	0.764	0.236	0.300	0.364
7975	0.771	0.314	0.336	0.426
7980	0.801	0.324	0.370	0.433
7985	0.790	0.308	0.352	0.421
7990	0.797	0.332	0.368	0.440

7995	0.789	0.325	0.356	0.435
8000	0.816	0.344	0.398	0.448
8005	0.768	0.248	0.308	0.374
8010	0.782	0.275	0.331	0.394
8015	0.775	0.258	0.317	0.381
8020	0.801	0.344	0.376	0.450
8025	0.789	0.345	0.362	0.450
8030	0.778	0.256	0.320	0.379
8035	0.785	0.327	0.353	0.436
8040	0.763	0.220	0.291	0.351
8045	0.802	0.333	0.375	0.441
8050	0.778	0.269	0.325	0.390
8055	0.783	0.311	0.346	0.424
8060	0.799	0.281	0.352	0.396
8065	0.787	0.268	0.333	0.388
8070	0.789	0.338	0.360	0.445
8075	0.780	0.299	0.338	0.415
8080	0.785	0.333	0.355	0.441
8085	0.787	0.316	0.351	0.428
8090	0.775	0.331	0.345	0.440
8095	0.778	0.269	0.325	0.390
8100	0.756	0.252	0.301	0.379
8105	0.763	0.262	0.309	0.386
8110	0.763	0.255	0.307	0.380
8115	0.775	0.271	0.323	0.393
8120	0.787	0.303	0.346	0.417
8125	0.776	0.267	0.323	0.389
8130	0.724	0.157	0.241	0.306
8135	0.771	0.273	0.321	0.394
8140	0.795	0.316	0.361	0.427
8145	0.782	0.328	0.350	0.438
8150	0.761	0.231	0.295	0.361
8155	0.783	0.277	0.333	0.396
8160	0.769	0.305	0.331	0.419
8165	0.773	0.248	0.312	0.373
8170	0.780	0.286	0.333	0.404
8175	0.780	0.265	0.325	0.386
8180	0.731	0.208	0.268	0.346
8185	0.783	0.291	0.339	0.408
8190	0.790	0.301	0.350	0.415
8195	0.782	0.261	0.325	0.382
8200	0.795	0.335	0.367	0.443
8205	0.785	0.313	0.348	0.426

8210	0.787	0.316	0.351	0.428
8215	0.766	0.224	0.296	0.354
8220	0.790	0.354	0.367	0.457
8225	0.789	0.364	0.368	0.465
8230	0.792	0.317	0.357	0.429
8235	0.813	0.344	0.393	0.449
8240	0.752	0.191	0.271	0.329
8245	0.756	0.280	0.311	0.400
8250	0.775	0.271	0.323	0.393
8255	0.749	0.223	0.284	0.356
8260	0.730	0.156	0.243	0.304
8265	0.752	0.241	0.294	0.370
8270	0.804	0.387	0.393	0.484
8275	0.789	0.332	0.358	0.440
8280	0.794	0.300	0.353	0.414
8285	0.794	0.346	0.368	0.452
8290	0.763	0.241	0.301	0.369
8295	0.778	0.310	0.341	0.423
8300	0.775	0.271	0.323	0.393
8305	0.806	0.371	0.391	0.472
8310	0.768	0.227	0.298	0.356
8315	0.794	0.339	0.366	0.447
8320	0.775	0.292	0.331	0.409
8325	0.757	0.183	0.270	0.320
8330	0.797	0.318	0.364	0.429
8335	0.768	0.282	0.321	0.402
8340	0.785	0.340	0.357	0.446
8345	0.780	0.306	0.341	0.420
8350	0.794	0.339	0.366	0.447
8355	0.754	0.229	0.290	0.360
8360	0.766	0.246	0.305	0.372
8365	0.761	0.273	0.313	0.395
8370	0.782	0.302	0.341	0.417
8375	0.776	0.281	0.328	0.400
8380	0.756	0.217	0.286	0.350
8385	0.773	0.283	0.326	0.402
8390	0.794	0.326	0.362	0.436
8395	0.757	0.248	0.300	0.375
8400	0.768	0.241	0.305	0.368
8405	0.776	0.314	0.340	0.427
8410	0.797	0.325	0.366	0.435
8415	0.780	0.279	0.331	0.398
8420	0.756	0.225	0.289	0.356

8425	0.809	0.364	0.393	0.466
8430	0.785	0.307	0.346	0.421
8435	0.768	0.262	0.313	0.385
8440	0.742	0.185	0.263	0.326
8445	0.804	0.330	0.376	0.438
8450	0.750	0.260	0.300	0.385
8455	0.780	0.306	0.341	0.420
8460	0.795	0.309	0.358	0.422
8465	0.771	0.239	0.306	0.365
8470	0.806	0.384	0.394	0.481
8475	0.794	0.320	0.360	0.431
8480	0.776	0.287	0.331	0.406
8485	0.785	0.286	0.339	0.404
8490	0.778	0.336	0.349	0.443
8495	0.768	0.248	0.308	0.374
8500	0.766	0.252	0.308	0.378
8505	0.764	0.215	0.290	0.346
8510	0.754	0.271	0.307	0.393
8515	0.768	0.282	0.321	0.402
8520	0.804	0.316	0.372	0.426
8525	0.778	0.276	0.328	0.396
8530	0.782	0.322	0.348	0.432
8535	0.756	0.210	0.282	0.344
8540	0.787	0.355	0.364	0.458
8545	0.790	0.347	0.365	0.452
8550	0.776	0.267	0.323	0.389
8555	0.797	0.298	0.357	0.412
8560	0.797	0.312	0.361	0.424
8565	0.761	0.266	0.310	0.389
8570	0.783	0.304	0.344	0.419
8575	0.776	0.301	0.336	0.416
8580	0.776	0.287	0.331	0.406
8585	0.763	0.268	0.312	0.391
8590	0.804	0.375	0.389	0.474
8595	0.802	0.333	0.375	0.441
8600	0.776	0.301	0.336	0.416
8605	0.780	0.299	0.338	0.415
8610	0.773	0.269	0.321	0.391
8615	0.775	0.331	0.345	0.440
8620	0.775	0.251	0.315	0.375
8625	0.776	0.287	0.331	0.406
8630	0.769	0.278	0.321	0.398
8635	0.752	0.227	0.288	0.359

8640	0.789	0.298	0.347	0.413
8645	0.776	0.327	0.345	0.437
8650	0.764	0.243	0.303	0.370
8655	0.799	0.347	0.375	0.453
8660	0.782	0.288	0.336	0.406
8665	0.764	0.264	0.312	0.387
8670	0.745	0.232	0.286	0.364
8675	0.769	0.257	0.313	0.381
8680	0.795	0.289	0.351	0.404
8685	0.764	0.291	0.322	0.409
8690	0.775	0.251	0.315	0.375
8695	0.750	0.253	0.297	0.379
8700	0.813	0.344	0.393	0.449
8705	0.787	0.336	0.358	0.443
8710	0.797	0.351	0.374	0.456
8715	0.773	0.241	0.309	0.367
8720	0.785	0.293	0.341	0.410
8725	0.764	0.271	0.314	0.393
8730	0.769	0.298	0.329	0.414
8735	0.780	0.292	0.336	0.409
8740	0.782	0.302	0.341	0.417
8745	0.776	0.301	0.336	0.416
8750	0.759	0.229	0.293	0.359
8755	0.752	0.213	0.281	0.347
8760	0.780	0.265	0.325	0.386
8765	0.789	0.305	0.349	0.419
8770	0.761	0.266	0.310	0.389
8775	0.769	0.243	0.307	0.370
8780	0.782	0.315	0.346	0.427
8785	0.750	0.225	0.286	0.357
8790	0.778	0.276	0.328	0.396
8795	0.789	0.298	0.347	0.413
8800	0.747	0.248	0.293	0.376
8805	0.792	0.255	0.333	0.375
8810	0.721	0.139	0.231	0.291
8815	0.794	0.339	0.366	0.447
8820	0.771	0.287	0.326	0.405
8825	0.769	0.243	0.307	0.370
8830	0.744	0.251	0.292	0.378
8835	0.764	0.278	0.317	0.398
8840	0.716	0.190	0.253	0.333
8845	0.785	0.293	0.341	0.410
8850	0.744	0.209	0.275	0.345

8855	0.757	0.268	0.308	0.391
8860	0.766	0.224	0.296	0.354
8865	0.792	0.317	0.357	0.429
8870	0.718	0.157	0.238	0.306
8875	0.778	0.263	0.323	0.385
8880	0.766	0.246	0.305	0.372
8885	0.782	0.281	0.333	0.400
8890	0.768	0.248	0.308	0.374
8895	0.776	0.321	0.343	0.432
8900	0.789	0.264	0.333	0.384
8905	0.716	0.155	0.237	0.305
8910	0.776	0.281	0.328	0.400
8915	0.799	0.347	0.375	0.453
8920	0.782	0.308	0.343	0.422
8925	0.790	0.341	0.363	0.447
8930	0.783	0.284	0.336	0.402
8935	0.787	0.275	0.336	0.394
8940	0.763	0.248	0.304	0.374
8945	0.775	0.278	0.326	0.398
8950	0.754	0.257	0.301	0.383
8955	0.799	0.334	0.371	0.442
8960	0.764	0.264	0.312	0.387
8965	0.773	0.316	0.338	0.428
8970	0.790	0.294	0.347	0.410
8975	0.763	0.234	0.298	0.363
8980	0.768	0.234	0.302	0.362
8985	0.768	0.275	0.319	0.396
8990	0.744	0.258	0.295	0.383
8995	0.775	0.305	0.336	0.420
9000	0.761	0.293	0.320	0.410
9005	0.820	0.400	0.418	0.495
9010	0.776	0.308	0.338	0.422
9015	0.789	0.351	0.364	0.455
9020	0.825	0.378	0.422	0.477
9025	0.768	0.289	0.324	0.407
9030	0.780	0.299	0.338	0.415
9035	0.769	0.264	0.316	0.387
9040	0.802	0.314	0.368	0.424
9045	0.794	0.326	0.362	0.436
9050	0.782	0.254	0.322	0.376
9055	0.764	0.222	0.294	0.352
9060	0.802	0.353	0.381	0.457
9065	0.759	0.229	0.293	0.359

9070	0.745	0.225	0.283	0.358
9075	0.782	0.295	0.338	0.411
9080	0.776	0.301	0.336	0.416
9085	0.785	0.273	0.333	0.392
9090	0.752	0.206	0.278	0.341
9095	0.764	0.243	0.303	0.370
9100	0.757	0.248	0.300	0.375
9105	0.766	0.280	0.319	0.400
9110	0.749	0.264	0.301	0.388
9115	0.761	0.286	0.318	0.405
9120	0.773	0.255	0.315	0.379
9125	0.742	0.193	0.266	0.332
9130	0.778	0.283	0.331	0.402
9135	0.782	0.348	0.356	0.452
9140	0.754	0.229	0.290	0.360
9145	0.750	0.246	0.295	0.374
9150	0.792	0.263	0.336	0.381
9155	0.768	0.275	0.319	0.396
9160	0.757	0.234	0.294	0.364
9165	0.806	0.332	0.379	0.440
9170	0.797	0.345	0.372	0.451
9175	0.764	0.222	0.294	0.352
9180	0.783	0.263	0.328	0.384
9185	0.811	0.373	0.398	0.473
9190	0.724	0.186	0.255	0.329
9195	0.801	0.382	0.387	0.480
9200	0.797	0.325	0.366	0.435
9205	0.745	0.197	0.270	0.335
9210	0.775	0.305	0.336	0.420
9215	0.733	0.153	0.243	0.300
9220	0.744	0.244	0.289	0.373
9225	0.789	0.312	0.352	0.425
9230	0.778	0.317	0.343	0.429
9235	0.735	0.184	0.259	0.326
9240	0.763	0.234	0.298	0.363
9245	0.808	0.329	0.381	0.437
9250	0.749	0.216	0.281	0.350
9255	0.780	0.319	0.345	0.430
9260	0.744	0.209	0.275	0.345
9265	0.764	0.236	0.300	0.364
9270	0.792	0.283	0.345	0.400
9275	0.776	0.327	0.345	0.437
9280	0.778	0.269	0.325	0.390

9285	0.764	0.243	0.303	0.370
9290	0.766	0.313	0.331	0.426
9295	0.789	0.338	0.360	0.445
9300	0.724	0.120	0.222	0.274
9305	0.780	0.319	0.345	0.430
9310	0.778	0.276	0.328	0.396
9315	0.775	0.292	0.331	0.409
9320	0.756	0.217	0.286	0.350
9325	0.794	0.293	0.350	0.408
9330	0.776	0.287	0.331	0.406
9335	0.790	0.288	0.345	0.404
9340	0.750	0.246	0.295	0.374
9345	0.757	0.213	0.285	0.346
9350	0.776	0.281	0.328	0.400
9355	0.789	0.345	0.362	0.450
9360	0.735	0.206	0.268	0.343
9365	0.776	0.274	0.326	0.394
9370	0.769	0.291	0.326	0.409
9375	0.742	0.214	0.276	0.349
9380	0.787	0.329	0.356	0.438
9385	0.775	0.325	0.342	0.435
9390	0.801	0.356	0.380	0.460
9395	0.794	0.339	0.366	0.447
9400	0.775	0.298	0.333	0.414
9405	0.771	0.260	0.315	0.383
9410	0.790	0.341	0.363	0.447
9415	0.764	0.278	0.317	0.398
9420	0.799	0.373	0.382	0.473
9425	0.797	0.338	0.370	0.445
9430	0.764	0.278	0.317	0.398
9435	0.766	0.273	0.317	0.395
9440	0.763	0.282	0.317	0.402
9445	0.783	0.331	0.353	0.439
9450	0.769	0.271	0.319	0.393
9455	0.776	0.281	0.328	0.400
9460	0.813	0.376	0.402	0.476
9465	0.797	0.345	0.372	0.451
9470	0.797	0.318	0.364	0.429
9475	0.771	0.294	0.329	0.411
9480	0.769	0.257	0.313	0.381
9485	0.759	0.277	0.313	0.398
9490	0.754	0.250	0.299	0.377
9495	0.764	0.257	0.309	0.382

9500	0.768	0.275	0.319	0.396
9505	0.802	0.384	0.390	0.482
9510	0.790	0.301	0.350	0.415
9515	0.785	0.333	0.355	0.441
9520	0.756	0.232	0.292	0.362
9525	0.776	0.287	0.331	0.406
9530	0.764	0.264	0.312	0.387
9535	0.780	0.286	0.333	0.404
9540	0.750	0.204	0.276	0.339
9545	0.756	0.225	0.289	0.356
9550	0.792	0.317	0.357	0.429
9555	0.792	0.297	0.350	0.412
9560	0.785	0.313	0.348	0.426
9565	0.783	0.284	0.336	0.402
9570	0.771	0.287	0.326	0.405
9575	0.801	0.317	0.368	0.428
9580	0.757	0.268	0.308	0.391
9585	0.802	0.340	0.377	0.447
9590	0.771	0.300	0.331	0.416
9595	0.752	0.234	0.291	0.364
9600	0.773	0.296	0.331	0.413
9605	0.756	0.259	0.303	0.384
9610	0.797	0.332	0.368	0.440
9615	0.783	0.284	0.336	0.402
9620	0.759	0.236	0.296	0.365
9625	0.757	0.234	0.294	0.364
9630	0.756	0.246	0.298	0.373
9635	0.782	0.275	0.331	0.394
9640	0.769	0.305	0.331	0.419
9645	0.794	0.286	0.348	0.402
9650	0.752	0.248	0.297	0.376
9655	0.769	0.250	0.310	0.376
9660	0.759	0.243	0.299	0.371
9665	0.769	0.271	0.319	0.393
9670	0.782	0.275	0.331	0.394
9675	0.771	0.260	0.315	0.383
9680	0.775	0.305	0.336	0.420
9685	0.747	0.220	0.282	0.354
9690	0.782	0.288	0.336	0.406
9695	0.750	0.225	0.286	0.357
9700	0.773	0.269	0.321	0.391
9705	0.799	0.360	0.379	0.463
9710	0.740	0.219	0.277	0.353

9715	0.785	0.307	0.346	0.421
9720	0.771	0.273	0.321	0.394
9725	0.771	0.287	0.326	0.405
9730	0.735	0.206	0.268	0.343
9735	0.754	0.250	0.299	0.377
9740	0.783	0.317	0.348	0.429
9745	0.768	0.269	0.316	0.391
9750	0.768	0.248	0.308	0.374
9755	0.735	0.220	0.275	0.354
9760	0.783	0.256	0.325	0.378
9765	0.783	0.263	0.328	0.384
9770	0.723	0.103	0.212	0.259
9775	0.761	0.231	0.295	0.361
9780	0.731	0.236	0.280	0.367
9785	0.789	0.332	0.358	0.440
9790	0.806	0.339	0.381	0.446
9795	0.757	0.213	0.285	0.346
9800	0.759	0.215	0.287	0.347
9805	0.789	0.338	0.360	0.445
9810	0.750	0.204	0.276	0.339
9815	0.752	0.241	0.294	0.370
9820	0.782	0.302	0.341	0.417
9825	0.752	0.262	0.302	0.386
9830	0.780	0.279	0.331	0.398
9835	0.761	0.252	0.304	0.378
9840	0.782	0.302	0.341	0.417
9845	0.740	0.161	0.250	0.306
9850	0.752	0.199	0.275	0.335
9855	0.780	0.306	0.341	0.420
9860	0.754	0.229	0.290	0.360
9865	0.750	0.232	0.289	0.363
9870	0.763	0.262	0.309	0.386
9875	0.752	0.213	0.281	0.347
9880	0.735	0.213	0.272	0.349
9885	0.808	0.342	0.385	0.448
9890	0.764	0.250	0.306	0.376
9895	0.759	0.236	0.296	0.365
9900	0.776	0.301	0.336	0.416
9905	0.792	0.276	0.342	0.394
9910	0.764	0.264	0.312	0.387
9915	0.825	0.439	0.434	0.526
9920	0.811	0.348	0.391	0.452
9925	0.766	0.246	0.305	0.372

9930	0.752	0.262	0.302	0.386
9935	0.775	0.258	0.317	0.381
9940	0.801	0.375	0.385	0.475
9945	0.768	0.241	0.305	0.368
9950	0.827	0.406	0.431	0.500
9955	0.775	0.298	0.333	0.414
9960	0.780	0.286	0.333	0.404
9965	0.759	0.257	0.305	0.382
9970	0.787	0.322	0.353	0.433
9975	0.747	0.241	0.291	0.371
9980	0.763	0.220	0.291	0.351
9985	0.728	0.183	0.255	0.326
9990	0.742	0.156	0.248	0.300
9995	0.794	0.333	0.364	0.441
10000	0.778	0.276	0.328	0.396

(5) Performance of IFS with random forest on the list yielded by MCFS

**Number of features**	**ACC**	**MCC**	**Precision**	**F1-measure**
5	0.841	0.506	0.470	0.578
10	0.865	0.577	0.523	0.636
15	0.880	0.603	0.563	0.660
20	0.889	0.617	0.589	0.673
25	0.906	0.688	0.629	0.730
30	0.908	0.675	0.645	0.723
35	0.912	0.680	0.660	0.727
40	0.917	0.701	0.673	0.745
45	0.919	0.695	0.691	0.740
50	0.920	0.693	0.707	0.739
55	0.932	0.741	0.742	0.780
60	0.939	0.765	0.769	0.800
65	0.936	0.746	0.770	0.784
70	0.936	0.736	0.797	0.773
75	0.941	0.757	0.821	0.790
80	0.957	0.825	0.855	0.850
85	0.946	0.785	0.812	0.817
90	0.955	0.819	0.845	0.845
95	0.951	0.809	0.818	0.837
100	0.953	0.813	0.835	0.840
105	0.945	0.787	0.783	0.818
110	0.958	0.830	0.875	0.854
115	0.958	0.833	0.857	0.857
120	0.951	0.805	0.833	0.833
125	0.955	0.821	0.837	0.847

130	0.960	0.841	0.859	0.864
135	0.953	0.815	0.828	0.842
140	0.967	0.867	0.892	0.886
145	0.962	0.847	0.869	0.869
150	0.958	0.835	0.849	0.859
155	0.967	0.868	0.882	0.888
160	0.957	0.825	0.855	0.850
165	0.960	0.838	0.877	0.861
170	0.953	0.808	0.861	0.834
175	0.960	0.841	0.859	0.864
180	0.960	0.836	0.886	0.859
185	0.965	0.858	0.900	0.878
190	0.960	0.839	0.867	0.862
195	0.964	0.853	0.880	0.874
200	0.962	0.842	0.908	0.863
205	0.962	0.845	0.878	0.867
210	0.957	0.823	0.864	0.848
215	0.962	0.845	0.878	0.867
220	0.969	0.873	0.902	0.892
225	0.967	0.866	0.901	0.885
230	0.965	0.859	0.890	0.880
235	0.960	0.839	0.867	0.862
240	0.962	0.845	0.878	0.867
245	0.955	0.819	0.845	0.845
250	0.962	0.844	0.888	0.866
255	0.958	0.831	0.866	0.855
260	0.950	0.795	0.840	0.824
265	0.967	0.863	0.933	0.881
270	0.957	0.822	0.873	0.847
275	0.960	0.835	0.896	0.857
280	0.969	0.870	0.946	0.886
285	0.962	0.844	0.888	0.866
290	0.950	0.795	0.840	0.824
295	0.957	0.820	0.893	0.843
300	0.974	0.893	0.937	0.908
305	0.960	0.836	0.886	0.859
310	0.958	0.826	0.905	0.848
315	0.960	0.833	0.918	0.854
320	0.964	0.849	0.920	0.868
325	0.965	0.856	0.932	0.873
330	0.957	0.823	0.864	0.848
335	0.960	0.834	0.907	0.855
340	0.955	0.812	0.892	0.835

345	0.958	0.828	0.885	0.852
350	0.955	0.813	0.882	0.838
355	0.962	0.840	0.931	0.859
360	0.964	0.850	0.909	0.870
365	0.957	0.822	0.873	0.847
370	0.965	0.858	0.900	0.878
375	0.971	0.878	0.947	0.893
380	0.965	0.857	0.910	0.877
385	0.962	0.842	0.908	0.863
390	0.958	0.827	0.895	0.850
395	0.962	0.840	0.931	0.859
400	0.958	0.830	0.875	0.854
405	0.960	0.834	0.907	0.855
410	0.964	0.848	0.932	0.866
415	0.957	0.821	0.883	0.845
420	0.958	0.828	0.885	0.852
425	0.967	0.863	0.958	0.877
430	0.967	0.863	0.945	0.879
435	0.965	0.855	0.944	0.872
440	0.958	0.826	0.905	0.848
445	0.967	0.863	0.933	0.881
450	0.969	0.870	0.946	0.886
455	0.972	0.885	0.959	0.899
460	0.958	0.828	0.885	0.852
465	0.964	0.849	0.920	0.868
470	0.967	0.864	0.922	0.882
475	0.965	0.856	0.932	0.873
480	0.960	0.834	0.907	0.855
485	0.969	0.871	0.934	0.888
490	0.960	0.836	0.886	0.859
495	0.962	0.841	0.919	0.861
500	0.962	0.841	0.919	0.861
505	0.967	0.863	0.945	0.879
510	0.955	0.814	0.872	0.840
515	0.965	0.855	0.944	0.872
520	0.972	0.885	0.972	0.897
525	0.960	0.834	0.907	0.855
530	0.953	0.804	0.890	0.828
535	0.962	0.841	0.919	0.861
540	0.969	0.872	0.923	0.889
545	0.962	0.840	0.931	0.859
550	0.958	0.825	0.929	0.844
555	0.962	0.840	0.943	0.857

560	0.964	0.849	0.920	0.868
565	0.948	0.780	0.886	0.805
570	0.964	0.849	0.920	0.868
575	0.960	0.834	0.907	0.855
580	0.965	0.855	0.944	0.872
585	0.955	0.810	0.914	0.831
590	0.960	0.833	0.918	0.854
595	0.955	0.810	0.914	0.831
600	0.957	0.820	0.893	0.843
605	0.962	0.840	0.931	0.859
610	0.965	0.855	0.944	0.872
615	0.965	0.857	0.910	0.877
620	0.965	0.856	0.921	0.875
625	0.962	0.840	0.931	0.859
630	0.960	0.834	0.907	0.855
635	0.955	0.810	0.914	0.831
640	0.962	0.840	0.931	0.859
645	0.958	0.826	0.905	0.848
650	0.962	0.840	0.931	0.859
655	0.962	0.840	0.931	0.859
660	0.965	0.855	0.957	0.870
665	0.957	0.820	0.893	0.843
670	0.955	0.812	0.892	0.835
675	0.962	0.840	0.931	0.859
680	0.960	0.833	0.930	0.852
685	0.964	0.849	0.920	0.868
690	0.957	0.820	0.893	0.843
695	0.967	0.863	0.933	0.881
700	0.965	0.856	0.932	0.873
705	0.965	0.856	0.932	0.873
710	0.964	0.847	0.957	0.863
715	0.960	0.833	0.918	0.854
720	0.962	0.840	0.931	0.859
725	0.958	0.826	0.905	0.848
730	0.960	0.832	0.955	0.848
735	0.960	0.833	0.918	0.854
740	0.957	0.817	0.928	0.837
745	0.965	0.855	0.971	0.868
750	0.957	0.818	0.915	0.839
755	0.953	0.803	0.901	0.826
760	0.953	0.801	0.925	0.821
765	0.960	0.832	0.984	0.844
770	0.967	0.863	0.971	0.876

775	0.958	0.824	0.955	0.840
780	0.960	0.833	0.918	0.854
785	0.965	0.855	0.957	0.870
790	0.967	0.863	0.958	0.877
795	0.965	0.855	0.944	0.872
800	0.962	0.840	0.956	0.855
805	0.962	0.840	0.956	0.855
810	0.967	0.863	0.945	0.879
815	0.958	0.824	0.969	0.838
820	0.955	0.809	0.939	0.827
825	0.958	0.825	0.941	0.842
830	0.960	0.833	0.918	0.854
835	0.960	0.832	0.955	0.848
840	0.964	0.848	0.932	0.866
845	0.960	0.832	0.942	0.850
850	0.964	0.847	0.970	0.861
855	0.964	0.847	0.957	0.863
860	0.962	0.840	0.931	0.859
865	0.965	0.855	0.957	0.870
870	0.965	0.855	0.944	0.872
875	0.955	0.809	0.926	0.829
880	0.960	0.832	0.955	0.848
885	0.965	0.855	0.957	0.870
890	0.962	0.840	0.956	0.855
895	0.967	0.863	0.971	0.876
900	0.965	0.855	0.944	0.872
905	0.958	0.824	0.955	0.840
910	0.955	0.809	0.939	0.827
915	0.960	0.833	0.918	0.854
920	0.964	0.848	0.932	0.866
925	0.965	0.855	0.985	0.867
930	0.964	0.847	0.970	0.861
935	0.965	0.855	0.971	0.868
940	0.964	0.847	0.970	0.861
945	0.962	0.840	0.943	0.857
950	0.962	0.840	0.943	0.857
955	0.957	0.817	0.940	0.834
960	0.960	0.832	0.969	0.846
965	0.965	0.856	1.000	0.865
970	0.960	0.832	0.969	0.846
975	0.953	0.801	0.925	0.821
980	0.953	0.802	0.913	0.824
985	0.950	0.785	0.937	0.803

990	0.962	0.840	0.970	0.853
995	0.957	0.817	0.954	0.832
1000	0.957	0.817	0.940	0.834
1005	0.951	0.793	0.924	0.813
1010	0.964	0.847	0.957	0.863
1015	0.964	0.848	0.985	0.859
1020	0.958	0.825	0.984	0.836
1025	0.957	0.817	0.968	0.830
1030	0.964	0.847	0.957	0.863
1035	0.950	0.785	0.937	0.803
1040	0.957	0.817	0.940	0.834
1045	0.953	0.801	0.952	0.816
1050	0.964	0.847	0.957	0.863
1055	0.960	0.832	0.969	0.846
1060	0.967	0.863	0.958	0.877
1065	0.958	0.825	0.941	0.842
1070	0.955	0.809	0.939	0.827
1075	0.964	0.847	0.957	0.863
1080	0.967	0.863	0.945	0.879
1085	0.971	0.878	0.972	0.890
1090	0.957	0.818	0.915	0.839
1095	0.967	0.863	0.958	0.877
1100	0.969	0.870	0.971	0.883
1105	0.955	0.809	0.968	0.822
1110	0.967	0.863	0.971	0.876
1115	0.960	0.833	0.930	0.852
1120	0.955	0.809	0.939	0.827
1125	0.953	0.801	0.938	0.819
1130	0.953	0.801	0.952	0.816
1135	0.960	0.832	0.942	0.850
1140	0.964	0.847	0.970	0.861
1145	0.951	0.794	0.912	0.816
1150	0.955	0.809	0.939	0.827
1155	0.958	0.825	0.941	0.842
1160	0.960	0.832	0.942	0.850
1165	0.962	0.840	0.943	0.857
1170	0.962	0.840	0.970	0.853
1175	0.955	0.809	0.953	0.824
1180	0.964	0.847	0.957	0.863
1185	0.958	0.825	0.929	0.844
1190	0.957	0.817	0.940	0.834
1195	0.957	0.817	0.968	0.830
1200	0.955	0.809	0.926	0.829

1205	0.955	0.809	0.968	0.822
1210	0.960	0.832	0.942	0.850
1215	0.950	0.785	0.923	0.805
1220	0.958	0.825	0.984	0.836
1225	0.958	0.824	0.969	0.838
1230	0.964	0.847	0.957	0.863
1235	0.958	0.824	0.969	0.838
1240	0.958	0.824	0.955	0.840
1245	0.969	0.870	0.958	0.885
1250	0.951	0.793	0.938	0.811
1255	0.958	0.824	0.955	0.840
1260	0.945	0.761	0.933	0.778
1265	0.958	0.824	0.955	0.840
1270	0.965	0.855	0.971	0.868
1275	0.951	0.793	0.967	0.806
1280	0.957	0.817	0.968	0.830
1285	0.955	0.809	0.926	0.829
1290	0.967	0.863	1.000	0.872
1295	0.955	0.809	0.953	0.824
1300	0.965	0.855	0.985	0.867
1305	0.962	0.840	0.943	0.857
1310	0.967	0.863	0.971	0.876
1315	0.972	0.885	0.986	0.896
1320	0.962	0.840	0.956	0.855
1325	0.948	0.777	0.922	0.797
1330	0.960	0.832	0.942	0.850
1335	0.955	0.809	0.953	0.824
1340	0.962	0.840	0.956	0.855
1345	0.960	0.832	0.955	0.848
1350	0.964	0.847	0.957	0.863
1355	0.955	0.809	0.953	0.824
1360	0.958	0.824	0.955	0.840
1365	0.958	0.824	0.969	0.838
1370	0.967	0.863	0.971	0.876
1375	0.957	0.817	0.940	0.834
1380	0.962	0.840	0.931	0.859
1385	0.960	0.832	0.942	0.850
1390	0.960	0.832	0.955	0.848
1395	0.957	0.817	0.954	0.832
1400	0.965	0.855	0.944	0.872
1405	0.965	0.855	0.985	0.867
1410	0.962	0.840	0.956	0.855
1415	0.964	0.847	0.957	0.863

1420	0.958	0.824	0.969	0.838
1425	0.965	0.855	0.971	0.868
1430	0.965	0.855	0.971	0.868
1435	0.955	0.809	0.968	0.822
1440	0.958	0.824	0.969	0.838
1445	0.964	0.848	0.985	0.859
1450	0.953	0.801	0.952	0.816
1455	0.965	0.855	0.944	0.872
1460	0.950	0.785	0.951	0.800
1465	0.965	0.855	0.985	0.867
1470	0.960	0.832	0.942	0.850
1475	0.967	0.863	0.971	0.876
1480	0.960	0.832	0.955	0.848
1485	0.962	0.840	0.943	0.857
1490	0.965	0.855	0.971	0.868
1495	0.957	0.817	0.940	0.834
1500	0.958	0.824	0.955	0.840
1505	0.962	0.840	0.956	0.855
1510	0.955	0.809	0.953	0.824
1515	0.964	0.847	0.970	0.861
1520	0.962	0.840	0.956	0.855
1525	0.950	0.785	0.923	0.805
1530	0.962	0.840	0.970	0.853
1535	0.955	0.809	0.953	0.824
1540	0.958	0.824	0.955	0.840
1545	0.957	0.817	0.928	0.837
1550	0.958	0.825	0.929	0.844
1555	0.955	0.809	0.953	0.824
1560	0.960	0.832	0.942	0.850
1565	0.953	0.801	0.925	0.821
1570	0.958	0.825	0.941	0.842
1575	0.957	0.817	0.940	0.834
1580	0.962	0.840	0.984	0.851
1585	0.965	0.855	0.957	0.870
1590	0.958	0.825	0.941	0.842
1595	0.962	0.840	0.970	0.853
1600	0.958	0.824	0.955	0.840
1605	0.965	0.855	0.957	0.870
1610	0.964	0.847	0.957	0.863
1615	0.958	0.824	0.955	0.840
1620	0.960	0.832	0.955	0.848
1625	0.960	0.832	0.969	0.846
1630	0.958	0.824	0.969	0.838

1635	0.951	0.793	0.938	0.811
1640	0.960	0.832	0.955	0.848
1645	0.960	0.833	0.930	0.852
1650	0.957	0.818	0.915	0.839
1655	0.962	0.840	0.984	0.851
1660	0.957	0.817	0.928	0.837
1665	0.955	0.811	0.903	0.833
1670	0.962	0.840	0.956	0.855
1675	0.957	0.817	0.954	0.832
1680	0.960	0.832	0.955	0.848
1685	0.964	0.847	0.970	0.861
1690	0.958	0.824	0.969	0.838
1695	0.948	0.778	0.909	0.800
1700	0.960	0.832	0.942	0.850
1705	0.957	0.818	0.915	0.839
1710	0.960	0.832	0.955	0.848
1715	0.953	0.801	0.952	0.816
1720	0.962	0.840	0.956	0.855
1725	0.958	0.825	0.929	0.844
1730	0.951	0.794	0.912	0.816
1735	0.958	0.824	0.969	0.838
1740	0.962	0.840	0.970	0.853
1745	0.958	0.824	0.955	0.840
1750	0.951	0.793	0.938	0.811
1755	0.953	0.802	0.913	0.824
1760	0.958	0.824	0.969	0.838
1765	0.955	0.809	0.968	0.822
1770	0.964	0.847	0.957	0.863
1775	0.962	0.840	0.943	0.857
1780	0.955	0.809	0.953	0.824
1785	0.950	0.785	0.923	0.805
1790	0.957	0.817	0.940	0.834
1795	0.958	0.824	0.955	0.840
1800	0.958	0.824	0.969	0.838
1805	0.964	0.848	0.985	0.859
1810	0.958	0.825	0.941	0.842
1815	0.957	0.817	0.968	0.830
1820	0.962	0.840	0.956	0.855
1825	0.965	0.855	0.985	0.867
1830	0.943	0.754	0.905	0.776
1835	0.962	0.840	0.956	0.855
1840	0.960	0.832	0.942	0.850
1845	0.965	0.855	0.957	0.870

1850	0.969	0.871	1.000	0.880
1855	0.953	0.801	0.938	0.819
1860	0.964	0.848	0.944	0.865
1865	0.957	0.817	0.954	0.832
1870	0.960	0.832	0.942	0.850
1875	0.964	0.847	0.970	0.861
1880	0.964	0.847	0.970	0.861
1885	0.953	0.801	0.938	0.819
1890	0.964	0.848	0.985	0.859
1895	0.958	0.824	0.955	0.840
1900	0.953	0.801	0.938	0.819
1905	0.964	0.847	0.957	0.863
1910	0.960	0.832	0.942	0.850
1915	0.962	0.840	0.970	0.853
1920	0.960	0.832	0.955	0.848
1925	0.957	0.817	0.928	0.837
1930	0.962	0.840	0.956	0.855
1935	0.953	0.801	0.938	0.819
1940	0.964	0.847	0.970	0.861
1945	0.962	0.840	0.943	0.857
1950	0.962	0.840	0.956	0.855
1955	0.953	0.801	0.938	0.819
1960	0.958	0.825	0.941	0.842
1965	0.964	0.847	0.970	0.861
1970	0.957	0.817	0.928	0.837
1975	0.958	0.826	0.917	0.846
1980	0.962	0.840	0.943	0.857
1985	0.962	0.840	0.931	0.859
1990	0.964	0.847	0.970	0.861
1995	0.955	0.809	0.939	0.827
2000	0.955	0.809	0.939	0.827
2005	0.955	0.809	0.953	0.824
2010	0.962	0.840	0.956	0.855
2015	0.965	0.855	0.971	0.868
2020	0.948	0.777	0.950	0.792
2025	0.965	0.855	0.944	0.872
2030	0.951	0.793	0.967	0.806
2035	0.964	0.847	0.957	0.863
2040	0.955	0.809	0.968	0.822
2045	0.969	0.870	0.985	0.882
2050	0.969	0.871	0.934	0.888
2055	0.948	0.777	0.922	0.797
2060	0.962	0.840	0.970	0.853

2065	0.960	0.833	0.930	0.852
2070	0.962	0.840	0.956	0.855
2075	0.960	0.832	0.969	0.846
2080	0.958	0.825	0.941	0.842
2085	0.969	0.870	0.971	0.883
2090	0.958	0.825	0.941	0.842
2095	0.962	0.840	0.970	0.853
2100	0.962	0.840	0.970	0.853
2105	0.948	0.779	0.897	0.803
2110	0.960	0.832	0.969	0.846
2115	0.957	0.817	0.940	0.834
2120	0.969	0.870	0.985	0.882
2125	0.962	0.840	0.956	0.855
2130	0.955	0.809	0.939	0.827
2135	0.957	0.817	0.954	0.832
2140	0.955	0.809	0.953	0.824
2145	0.957	0.817	0.984	0.828
2150	0.958	0.825	0.941	0.842
2155	0.960	0.832	0.969	0.846
2160	0.964	0.848	0.985	0.859
2165	0.967	0.863	0.985	0.874
2170	0.965	0.855	0.957	0.870
2175	0.960	0.832	0.942	0.850
2180	0.962	0.840	0.956	0.855
2185	0.962	0.840	0.931	0.859
2190	0.962	0.840	0.956	0.855
2195	0.958	0.825	0.929	0.844
2200	0.962	0.840	0.943	0.857
2205	0.960	0.832	0.955	0.848
2210	0.957	0.817	0.954	0.832
2215	0.957	0.817	0.940	0.834
2220	0.967	0.863	0.971	0.876
2225	0.964	0.848	0.944	0.865
2230	0.962	0.840	0.970	0.853
2235	0.969	0.870	0.971	0.883
2240	0.969	0.871	1.000	0.880
2245	0.960	0.833	0.918	0.854
2250	0.957	0.817	0.954	0.832
2255	0.962	0.840	0.970	0.853
2260	0.965	0.855	0.985	0.867
2265	0.962	0.840	0.931	0.859
2270	0.960	0.832	0.942	0.850
2275	0.958	0.824	0.969	0.838

2280	0.960	0.833	0.930	0.852
2285	0.964	0.847	0.970	0.861
2290	0.965	0.855	0.971	0.868
2295	0.965	0.855	0.944	0.872
2300	0.957	0.817	0.928	0.837
2305	0.965	0.855	0.971	0.868
2310	0.962	0.840	0.943	0.857
2315	0.955	0.809	0.926	0.829
2320	0.955	0.809	0.953	0.824
2325	0.958	0.825	0.929	0.844
2330	0.960	0.833	0.930	0.852
2335	0.958	0.825	0.941	0.842
2340	0.964	0.847	0.970	0.861
2345	0.967	0.863	0.958	0.877
2350	0.965	0.855	0.944	0.872
2355	0.965	0.855	0.957	0.870
2360	0.960	0.832	0.942	0.850
2365	0.955	0.809	0.968	0.822
2370	0.958	0.824	0.969	0.838
2375	0.964	0.847	0.957	0.863
2380	0.958	0.826	0.917	0.846
2385	0.967	0.863	0.971	0.876
2390	0.957	0.817	0.954	0.832
2395	0.964	0.847	0.970	0.861
2400	0.960	0.832	0.984	0.844
2405	0.969	0.870	0.985	0.882
2410	0.964	0.847	0.957	0.863
2415	0.960	0.833	0.930	0.852
2420	0.967	0.863	0.971	0.876
2425	0.958	0.824	0.969	0.838
2430	0.969	0.870	0.985	0.882
2435	0.964	0.847	0.957	0.863
2440	0.964	0.847	0.970	0.861
2445	0.967	0.863	0.971	0.876
2450	0.962	0.840	0.943	0.857
2455	0.955	0.809	0.926	0.829
2460	0.960	0.832	0.955	0.848
2465	0.965	0.855	0.957	0.870
2470	0.958	0.825	0.929	0.844
2475	0.957	0.817	0.984	0.828
2480	0.964	0.847	0.970	0.861
2485	0.964	0.847	0.957	0.863
2490	0.960	0.832	0.969	0.846

2495	0.955	0.809	0.953	0.824
2500	0.962	0.840	0.970	0.853
2505	0.964	0.847	0.957	0.863
2510	0.967	0.863	0.971	0.876
2515	0.971	0.878	0.986	0.889
2520	0.967	0.863	0.971	0.876
2525	0.960	0.832	0.969	0.846
2530	0.962	0.840	0.970	0.853
2535	0.958	0.825	0.929	0.844
2540	0.960	0.832	0.955	0.848
2545	0.965	0.855	0.985	0.867
2550	0.964	0.848	0.985	0.859
2555	0.957	0.817	0.928	0.837
2560	0.967	0.863	0.958	0.877
2565	0.969	0.871	1.000	0.880
2570	0.955	0.810	0.914	0.831
2575	0.965	0.855	0.957	0.870
2580	0.962	0.840	0.956	0.855
2585	0.969	0.870	0.971	0.883
2590	0.964	0.847	0.957	0.863
2595	0.957	0.817	0.954	0.832
2600	0.969	0.870	0.971	0.883
2605	0.964	0.847	0.957	0.863
2610	0.964	0.848	0.944	0.865
2615	0.958	0.825	0.941	0.842
2620	0.962	0.840	0.956	0.855
2625	0.969	0.870	0.971	0.883
2630	0.960	0.832	0.969	0.846
2635	0.955	0.809	0.939	0.827
2640	0.964	0.847	0.970	0.861
2645	0.960	0.832	0.955	0.848
2650	0.960	0.832	0.955	0.848
2655	0.960	0.833	1.000	0.841
2660	0.955	0.809	0.953	0.824
2665	0.955	0.809	0.953	0.824
2670	0.964	0.847	0.957	0.863
2675	0.958	0.825	0.984	0.836
2680	0.962	0.840	0.956	0.855
2685	0.971	0.878	0.986	0.889
2690	0.967	0.863	0.958	0.877
2695	0.951	0.793	0.924	0.813
2700	0.960	0.832	0.984	0.844
2705	0.955	0.809	0.968	0.822

2710	0.958	0.824	0.955	0.840
2715	0.958	0.824	0.955	0.840
2720	0.964	0.847	0.957	0.863
2725	0.962	0.840	0.984	0.851
2730	0.957	0.817	0.968	0.830
2735	0.960	0.832	0.969	0.846
2740	0.962	0.840	0.931	0.859
2745	0.965	0.855	0.944	0.872
2750	0.958	0.825	0.941	0.842
2755	0.953	0.801	0.967	0.814
2760	0.957	0.817	0.940	0.834
2765	0.962	0.841	0.919	0.861
2770	0.964	0.847	0.970	0.861
2775	0.965	0.855	0.944	0.872
2780	0.967	0.863	0.958	0.877
2785	0.955	0.809	0.939	0.827
2790	0.962	0.840	0.970	0.853
2795	0.964	0.847	0.957	0.863
2800	0.964	0.848	0.985	0.859
2805	0.964	0.848	0.985	0.859
2810	0.960	0.833	0.918	0.854
2815	0.958	0.824	0.969	0.838
2820	0.958	0.825	0.941	0.842
2825	0.950	0.785	0.937	0.803
2830	0.960	0.832	0.942	0.850
2835	0.955	0.809	0.926	0.829
2840	0.953	0.801	0.938	0.819
2845	0.958	0.824	0.955	0.840
2850	0.953	0.801	0.925	0.821
2855	0.965	0.855	0.957	0.870
2860	0.969	0.870	0.985	0.882
2865	0.953	0.801	0.952	0.816
2870	0.960	0.832	0.955	0.848
2875	0.960	0.832	0.955	0.848
2880	0.972	0.885	0.986	0.896
2885	0.964	0.847	0.970	0.861
2890	0.958	0.825	0.941	0.842
2895	0.964	0.847	0.957	0.863
2900	0.958	0.824	0.969	0.838
2905	0.958	0.824	0.955	0.840
2910	0.965	0.855	0.971	0.868
2915	0.955	0.811	0.903	0.833
2920	0.962	0.840	0.970	0.853

2925	0.960	0.832	0.984	0.844
2930	0.955	0.809	0.953	0.824
2935	0.965	0.855	0.957	0.870
2940	0.960	0.833	0.930	0.852
2945	0.955	0.809	0.953	0.824
2950	0.960	0.832	0.942	0.850
2955	0.955	0.809	0.968	0.822
2960	0.951	0.793	0.952	0.808
2965	0.958	0.824	0.969	0.838
2970	0.967	0.863	0.985	0.874
2975	0.965	0.855	0.957	0.870
2980	0.960	0.832	0.942	0.850
2985	0.960	0.832	0.984	0.844
2990	0.962	0.841	1.000	0.849
2995	0.957	0.817	0.940	0.834
3000	0.957	0.817	0.954	0.832
3005	0.965	0.855	0.957	0.870
3010	0.953	0.801	0.925	0.821
3015	0.955	0.810	0.914	0.831
3020	0.971	0.878	0.972	0.890
3025	0.953	0.801	0.952	0.816
3030	0.958	0.824	0.969	0.838
3035	0.960	0.832	0.955	0.848
3040	0.958	0.826	0.917	0.846
3045	0.951	0.794	0.912	0.816
3050	0.962	0.840	0.956	0.855
3055	0.962	0.840	0.931	0.859
3060	0.967	0.863	0.985	0.874
3065	0.958	0.824	0.969	0.838
3070	0.958	0.824	0.955	0.840
3075	0.958	0.824	0.969	0.838
3080	0.960	0.832	0.942	0.850
3085	0.962	0.840	0.956	0.855
3090	0.967	0.863	0.985	0.874
3095	0.960	0.832	0.955	0.848
3100	0.964	0.848	0.985	0.859
3105	0.962	0.840	0.943	0.857
3110	0.957	0.817	0.940	0.834
3115	0.957	0.817	0.940	0.834
3120	0.953	0.801	0.952	0.816
3125	0.958	0.825	0.984	0.836
3130	0.964	0.847	0.957	0.863
3135	0.958	0.825	0.941	0.842

3140	0.955	0.809	0.939	0.827
3145	0.960	0.832	0.942	0.850
3150	0.960	0.832	0.955	0.848
3155	0.965	0.855	0.957	0.870
3160	0.960	0.832	0.942	0.850
3165	0.955	0.809	0.968	0.822
3170	0.958	0.826	0.917	0.846
3175	0.962	0.840	0.984	0.851
3180	0.960	0.832	0.942	0.850
3185	0.950	0.785	0.923	0.805
3190	0.951	0.793	0.952	0.808
3195	0.962	0.840	0.984	0.851
3200	0.972	0.885	0.986	0.896
3205	0.962	0.840	0.984	0.851
3210	0.969	0.870	0.958	0.885
3215	0.957	0.817	0.954	0.832
3220	0.964	0.847	0.957	0.863
3225	0.962	0.840	0.984	0.851
3230	0.972	0.885	0.959	0.899
3235	0.951	0.793	0.952	0.808
3240	0.957	0.817	0.954	0.832
3245	0.957	0.817	0.954	0.832
3250	0.953	0.801	0.952	0.816
3255	0.953	0.801	0.983	0.811
3260	0.951	0.793	0.924	0.813
3265	0.958	0.824	0.969	0.838
3270	0.948	0.778	0.982	0.786
3275	0.948	0.777	0.922	0.797
3280	0.951	0.793	0.938	0.811
3285	0.955	0.809	0.939	0.827
3290	0.957	0.817	0.940	0.834
3295	0.957	0.817	0.968	0.830
3300	0.955	0.809	0.953	0.824
3305	0.950	0.785	0.966	0.797
3310	0.957	0.817	0.968	0.830
3315	0.957	0.817	0.968	0.830
3320	0.955	0.809	0.983	0.819
3325	0.962	0.840	0.956	0.855
3330	0.958	0.824	0.955	0.840
3335	0.960	0.832	0.969	0.846
3340	0.957	0.817	0.954	0.832
3345	0.955	0.809	0.953	0.824
3350	0.958	0.825	1.000	0.833

3355	0.958	0.825	1.000	0.833
3360	0.953	0.801	0.925	0.821
3365	0.965	0.856	1.000	0.865
3370	0.958	0.824	0.955	0.840
3375	0.962	0.840	0.943	0.857
3380	0.962	0.840	0.956	0.855
3385	0.955	0.809	0.968	0.822
3390	0.950	0.785	0.966	0.797
3395	0.953	0.801	0.938	0.819
3400	0.950	0.785	0.937	0.803
3405	0.953	0.801	0.925	0.821
3410	0.965	0.855	0.957	0.870
3415	0.962	0.840	0.970	0.853
3420	0.951	0.793	0.938	0.811
3425	0.960	0.832	0.984	0.844
3430	0.953	0.801	0.967	0.814
3435	0.962	0.840	0.970	0.853
3440	0.955	0.809	0.939	0.827
3445	0.957	0.817	0.954	0.832
3450	0.955	0.809	0.968	0.822
3455	0.960	0.832	0.942	0.850
3460	0.958	0.824	0.969	0.838
3465	0.955	0.809	0.953	0.824
3470	0.951	0.793	0.967	0.806
3475	0.958	0.824	0.969	0.838
3480	0.950	0.785	0.966	0.797
3485	0.960	0.832	0.942	0.850
3490	0.964	0.847	0.957	0.863
3495	0.951	0.793	0.938	0.811
3500	0.965	0.855	0.957	0.870
3505	0.953	0.801	0.952	0.816
3510	0.953	0.801	0.967	0.814
3515	0.958	0.824	0.969	0.838
3520	0.958	0.824	0.955	0.840
3525	0.962	0.841	1.000	0.849
3530	0.953	0.801	0.952	0.816
3535	0.955	0.809	0.953	0.824
3540	0.958	0.825	0.941	0.842
3545	0.953	0.802	0.913	0.824
3550	0.960	0.832	0.942	0.850
3555	0.955	0.809	0.968	0.822
3560	0.957	0.817	0.954	0.832
3565	0.962	0.840	0.943	0.857

3570	0.951	0.793	0.924	0.813
3575	0.957	0.817	0.940	0.834
3580	0.951	0.793	0.952	0.808
3585	0.958	0.825	0.984	0.836
3590	0.960	0.832	0.955	0.848
3595	0.950	0.785	0.951	0.800
3600	0.955	0.809	0.939	0.827
3605	0.964	0.847	0.970	0.861
3610	0.960	0.832	0.984	0.844
3615	0.958	0.824	0.955	0.840
3620	0.950	0.785	0.937	0.803
3625	0.962	0.840	0.956	0.855
3630	0.950	0.785	0.937	0.803
3635	0.955	0.809	0.983	0.819
3640	0.948	0.777	0.922	0.797
3645	0.960	0.832	0.942	0.850
3650	0.958	0.825	0.984	0.836
3655	0.958	0.825	0.941	0.842
3660	0.955	0.809	0.983	0.819
3665	0.953	0.801	0.952	0.816
3670	0.960	0.832	0.955	0.848
3675	0.957	0.817	0.968	0.830
3680	0.955	0.809	0.953	0.824
3685	0.958	0.825	0.984	0.836
3690	0.964	0.848	1.000	0.857
3695	0.953	0.801	0.925	0.821
3700	0.953	0.801	0.925	0.821
3705	0.946	0.769	0.934	0.786
3710	0.955	0.809	0.983	0.819
3715	0.946	0.769	0.965	0.780
3720	0.951	0.793	0.952	0.808
3725	0.955	0.809	0.953	0.824
3730	0.957	0.817	0.954	0.832
3735	0.953	0.801	0.952	0.816
3740	0.950	0.785	0.937	0.803
3745	0.960	0.832	0.955	0.848
3750	0.951	0.793	0.952	0.808
3755	0.951	0.793	0.938	0.811
3760	0.950	0.785	0.966	0.797
3765	0.953	0.801	0.952	0.816
3770	0.957	0.817	0.940	0.834
3775	0.955	0.809	0.983	0.819
3780	0.957	0.817	0.954	0.832

3785	0.953	0.801	0.952	0.816
3790	0.950	0.785	0.937	0.803
3795	0.948	0.777	0.935	0.795
3800	0.950	0.785	0.923	0.805
3805	0.955	0.809	0.968	0.822
3810	0.950	0.785	0.951	0.800
3815	0.955	0.809	0.968	0.822
3820	0.960	0.832	0.969	0.846
3825	0.950	0.785	0.966	0.797
3830	0.955	0.809	0.939	0.827
3835	0.953	0.801	0.938	0.819
3840	0.951	0.793	0.952	0.808
3845	0.945	0.762	0.906	0.784
3850	0.957	0.817	0.984	0.828
3855	0.958	0.824	0.955	0.840
3860	0.955	0.809	0.968	0.822
3865	0.958	0.824	0.969	0.838
3870	0.957	0.817	0.968	0.830
3875	0.958	0.824	0.969	0.838
3880	0.955	0.809	0.983	0.819
3885	0.953	0.801	0.967	0.814
3890	0.958	0.824	0.969	0.838
3895	0.945	0.761	0.964	0.771
3900	0.953	0.801	0.967	0.814
3905	0.957	0.817	0.940	0.834
3910	0.938	0.728	0.929	0.743
3915	0.948	0.777	0.966	0.789
3920	0.950	0.786	0.982	0.794
3925	0.948	0.777	0.935	0.795
3930	0.955	0.809	0.926	0.829
3935	0.953	0.802	0.913	0.824
3940	0.953	0.801	0.938	0.819
3945	0.957	0.817	0.968	0.830
3950	0.950	0.785	0.923	0.805
3955	0.964	0.848	0.985	0.859
3960	0.962	0.840	0.970	0.853
3965	0.955	0.809	0.953	0.824
3970	0.953	0.801	0.983	0.811
3975	0.957	0.817	0.968	0.830
3980	0.958	0.825	0.984	0.836
3985	0.950	0.785	0.937	0.803
3990	0.953	0.801	0.938	0.819
3995	0.955	0.809	0.953	0.824

4000	0.950	0.785	0.966	0.797
4005	0.960	0.832	0.969	0.846
4010	0.951	0.794	0.983	0.803
4015	0.946	0.769	0.949	0.783
4020	0.953	0.804	0.890	0.828
4025	0.953	0.801	0.952	0.816
4030	0.955	0.809	0.926	0.829
4035	0.958	0.824	0.969	0.838
4040	0.957	0.817	0.940	0.834
4045	0.951	0.793	0.967	0.806
4050	0.953	0.801	0.952	0.816
4055	0.955	0.809	0.939	0.827
4060	0.957	0.817	0.954	0.832
4065	0.951	0.793	0.952	0.808
4070	0.955	0.809	0.983	0.819
4075	0.948	0.777	0.935	0.795
4080	0.953	0.801	0.967	0.814
4085	0.960	0.832	0.969	0.846
4090	0.960	0.832	0.955	0.848
4095	0.958	0.825	0.984	0.836
4100	0.957	0.817	0.954	0.832
4105	0.951	0.793	0.952	0.808
4110	0.946	0.771	0.896	0.795
4115	0.950	0.785	0.937	0.803
4120	0.950	0.785	0.937	0.803
4125	0.948	0.778	0.982	0.786
4130	0.946	0.769	0.934	0.786
4135	0.946	0.769	0.934	0.786
4140	0.950	0.785	0.966	0.797
4145	0.953	0.801	0.983	0.811
4150	0.945	0.761	0.919	0.781
4155	0.953	0.801	0.925	0.821
4160	0.958	0.824	0.969	0.838
4165	0.957	0.817	0.954	0.832
4170	0.951	0.793	0.938	0.811
4175	0.948	0.777	0.922	0.797
4180	0.945	0.762	0.906	0.784
4185	0.953	0.801	0.952	0.816
4190	0.948	0.777	0.966	0.789
4195	0.960	0.832	0.969	0.846
4200	0.953	0.801	0.983	0.811
4205	0.946	0.769	0.949	0.783
4210	0.951	0.793	0.938	0.811

4215	0.950	0.785	0.937	0.803
4220	0.943	0.753	0.964	0.763
4225	0.946	0.769	0.965	0.780
4230	0.955	0.809	0.953	0.824
4235	0.953	0.801	0.967	0.814
4240	0.953	0.801	0.967	0.814
4245	0.962	0.840	0.984	0.851
4250	0.945	0.761	0.919	0.781
4255	0.951	0.794	0.983	0.803
4260	0.950	0.785	0.966	0.797
4265	0.962	0.840	0.984	0.851
4270	0.955	0.809	0.968	0.822
4275	0.946	0.769	0.934	0.786
4280	0.955	0.809	0.953	0.824
4285	0.946	0.769	0.921	0.789
4290	0.951	0.793	0.967	0.806
4295	0.950	0.785	0.951	0.800
4300	0.965	0.855	0.985	0.867
4305	0.965	0.855	0.944	0.872
4310	0.964	0.847	0.970	0.861
4315	0.951	0.793	0.938	0.811
4320	0.953	0.801	0.925	0.821
4325	0.951	0.793	0.924	0.813
4330	0.964	0.847	0.957	0.863
4335	0.953	0.801	0.952	0.816
4340	0.967	0.863	0.971	0.876
4345	0.951	0.793	0.938	0.811
4350	0.955	0.809	0.939	0.827
4355	0.950	0.785	0.923	0.805
4360	0.950	0.785	0.923	0.805
4365	0.962	0.840	0.956	0.855
4370	0.955	0.809	0.953	0.824
4375	0.948	0.777	0.935	0.795
4380	0.962	0.841	1.000	0.849
4385	0.948	0.777	0.922	0.797
4390	0.955	0.809	0.953	0.824
4395	0.958	0.824	0.969	0.838
4400	0.943	0.753	0.918	0.772
4405	0.948	0.777	0.922	0.797
4410	0.955	0.809	0.939	0.827
4415	0.950	0.786	0.910	0.808
4420	0.962	0.840	0.970	0.853
4425	0.953	0.801	0.938	0.819

4430	0.955	0.809	0.968	0.822
4435	0.948	0.777	0.950	0.792
4440	0.958	0.824	0.955	0.840
4445	0.960	0.832	0.984	0.844
4450	0.957	0.817	0.954	0.832
4455	0.948	0.777	0.922	0.797
4460	0.957	0.817	0.940	0.834
4465	0.948	0.779	0.897	0.803
4470	0.958	0.824	0.969	0.838
4475	0.950	0.786	0.910	0.808
4480	0.960	0.833	0.918	0.854
4485	0.955	0.809	0.953	0.824
4490	0.958	0.825	0.984	0.836
4495	0.957	0.817	0.968	0.830
4500	0.950	0.785	0.937	0.803
4505	0.950	0.785	0.937	0.803
4510	0.962	0.840	0.956	0.855
4515	0.950	0.785	0.951	0.800
4520	0.964	0.848	0.985	0.859
4525	0.958	0.824	0.969	0.838
4530	0.957	0.817	0.954	0.832
4535	0.950	0.785	0.937	0.803
4540	0.960	0.832	0.984	0.844
4545	0.955	0.809	0.983	0.819
4550	0.939	0.736	0.945	0.748
4555	0.955	0.809	0.953	0.824
4560	0.953	0.801	0.952	0.816
4565	0.951	0.793	0.924	0.813
4570	0.948	0.777	0.950	0.792
4575	0.950	0.785	0.937	0.803
4580	0.955	0.813	0.882	0.838
4585	0.960	0.832	0.984	0.844
4590	0.953	0.801	0.952	0.816
4595	0.960	0.832	0.969	0.846
4600	0.962	0.840	0.956	0.855
4605	0.960	0.832	0.984	0.844
4610	0.951	0.793	0.967	0.806
4615	0.951	0.793	0.952	0.808
4620	0.960	0.832	0.969	0.846
4625	0.950	0.785	0.951	0.800
4630	0.957	0.817	0.984	0.828
4635	0.948	0.777	0.950	0.792
4640	0.953	0.801	0.983	0.811

4645	0.943	0.754	0.892	0.779
4650	0.957	0.817	0.940	0.834
4655	0.955	0.809	0.983	0.819
4660	0.958	0.824	0.969	0.838
4665	0.955	0.809	0.953	0.824
4670	0.953	0.801	0.952	0.816
4675	0.960	0.832	0.955	0.848
4680	0.957	0.817	0.984	0.828
4685	0.948	0.778	0.909	0.800
4690	0.958	0.824	0.969	0.838
4695	0.953	0.801	0.967	0.814
4700	0.958	0.824	0.955	0.840
4705	0.955	0.809	0.983	0.819
4710	0.953	0.801	0.938	0.819
4715	0.960	0.833	1.000	0.841
4720	0.955	0.809	0.953	0.824
4725	0.955	0.809	0.939	0.827
4730	0.967	0.863	0.985	0.874
4735	0.953	0.801	0.967	0.814
4740	0.960	0.832	0.969	0.846
4745	0.946	0.769	0.949	0.783
4750	0.964	0.848	1.000	0.857
4755	0.951	0.793	0.924	0.813
4760	0.948	0.777	0.922	0.797
4765	0.958	0.824	0.955	0.840
4770	0.957	0.817	0.968	0.830
4775	0.953	0.801	0.967	0.814
4780	0.960	0.832	0.969	0.846
4785	0.951	0.793	0.952	0.808
4790	0.953	0.801	0.938	0.819
4795	0.964	0.848	1.000	0.857
4800	0.950	0.785	0.966	0.797
4805	0.953	0.801	0.967	0.814
4810	0.950	0.785	0.951	0.800
4815	0.951	0.793	0.952	0.808
4820	0.953	0.801	0.967	0.814
4825	0.950	0.785	0.951	0.800
4830	0.951	0.793	0.967	0.806
4835	0.958	0.824	0.969	0.838
4840	0.951	0.793	0.938	0.811
4845	0.958	0.824	0.955	0.840
4850	0.958	0.825	0.984	0.836
4855	0.953	0.801	0.967	0.814

4860	0.951	0.793	0.952	0.808
4865	0.948	0.777	0.950	0.792
4870	0.965	0.855	0.971	0.868
4875	0.951	0.793	0.952	0.808
4880	0.955	0.809	0.953	0.824
4885	0.958	0.824	0.969	0.838
4890	0.957	0.817	0.968	0.830
4895	0.967	0.863	1.000	0.872
4900	0.957	0.817	0.954	0.832
4905	0.955	0.809	0.953	0.824
4910	0.955	0.809	0.968	0.822
4915	0.953	0.801	0.967	0.814
4920	0.953	0.801	0.967	0.814
4925	0.955	0.809	0.953	0.824
4930	0.951	0.793	0.952	0.808
4935	0.960	0.833	1.000	0.841
4940	0.945	0.761	0.948	0.775
4945	0.953	0.801	0.952	0.816
4950	0.960	0.832	0.969	0.846
4955	0.957	0.817	0.968	0.830
4960	0.953	0.801	0.967	0.814
4965	0.951	0.793	0.952	0.808
4970	0.950	0.785	0.951	0.800
4975	0.953	0.801	0.952	0.816
4980	0.955	0.809	0.968	0.822
4985	0.950	0.785	0.937	0.803
4990	0.950	0.785	0.937	0.803
4995	0.964	0.848	1.000	0.857
5000	0.964	0.848	0.985	0.859
5005	0.946	0.770	0.982	0.777
5010	0.962	0.840	0.970	0.853
5015	0.953	0.802	0.913	0.824
5020	0.950	0.785	0.937	0.803
5025	0.957	0.817	0.984	0.828
5030	0.957	0.817	0.984	0.828
5035	0.948	0.777	0.966	0.789
5040	0.950	0.785	0.951	0.800
5045	0.955	0.809	0.968	0.822
5050	0.957	0.817	0.984	0.828
5055	0.953	0.801	0.967	0.814
5060	0.941	0.744	0.931	0.761
5065	0.953	0.801	0.952	0.816
5070	0.948	0.777	0.966	0.789

5075	0.967	0.863	0.985	0.874
5080	0.941	0.745	0.903	0.767
5085	0.955	0.809	0.983	0.819
5090	0.955	0.809	0.968	0.822
5095	0.960	0.832	0.984	0.844
5100	0.960	0.832	0.969	0.846
5105	0.953	0.801	0.967	0.814
5110	0.953	0.801	0.952	0.816
5115	0.962	0.840	0.984	0.851
5120	0.943	0.753	0.932	0.769
5125	0.953	0.801	0.952	0.816
5130	0.962	0.840	0.956	0.855
5135	0.955	0.809	0.983	0.819
5140	0.950	0.785	0.966	0.797
5145	0.958	0.825	0.984	0.836
5150	0.948	0.777	0.935	0.795
5155	0.958	0.825	0.984	0.836
5160	0.948	0.778	0.982	0.786
5165	0.955	0.809	0.968	0.822
5170	0.958	0.824	0.969	0.838
5175	0.953	0.801	0.952	0.816
5180	0.950	0.785	0.937	0.803
5185	0.960	0.832	0.969	0.846
5190	0.957	0.818	0.915	0.839
5195	0.958	0.824	0.969	0.838
5200	0.957	0.817	0.954	0.832
5205	0.950	0.785	0.937	0.803
5210	0.953	0.801	0.952	0.816
5215	0.951	0.793	0.952	0.808
5220	0.941	0.745	0.903	0.767
5225	0.953	0.801	0.983	0.811
5230	0.957	0.817	0.968	0.830
5235	0.946	0.769	0.934	0.786
5240	0.960	0.832	0.984	0.844
5245	0.950	0.785	0.966	0.797
5250	0.960	0.832	0.984	0.844
5255	0.960	0.832	0.969	0.846
5260	0.948	0.777	0.950	0.792
5265	0.955	0.810	1.000	0.817
5270	0.955	0.809	0.926	0.829
5275	0.948	0.777	0.935	0.795
5280	0.951	0.793	0.924	0.813
5285	0.943	0.753	0.947	0.766

5290	0.946	0.769	0.934	0.786
5295	0.960	0.832	0.942	0.850
5300	0.948	0.777	0.966	0.789
5305	0.943	0.753	0.947	0.766
5310	0.953	0.802	0.913	0.824
5315	0.948	0.777	0.950	0.792
5320	0.950	0.786	0.982	0.794
5325	0.938	0.729	0.900	0.750
5330	0.943	0.753	0.918	0.772
5335	0.950	0.786	0.982	0.794
5340	0.951	0.793	0.952	0.808
5345	0.951	0.793	0.967	0.806
5350	0.946	0.769	0.934	0.786
5355	0.955	0.810	1.000	0.817
5360	0.951	0.794	0.983	0.803
5365	0.950	0.785	0.951	0.800
5370	0.951	0.793	0.924	0.813
5375	0.953	0.801	0.983	0.811
5380	0.950	0.785	0.966	0.797
5385	0.951	0.793	0.952	0.808
5390	0.953	0.801	0.938	0.819
5395	0.951	0.793	0.952	0.808
5400	0.953	0.801	0.952	0.816
5405	0.951	0.793	0.952	0.808
5410	0.948	0.778	0.982	0.786
5415	0.955	0.809	0.968	0.822
5420	0.945	0.761	0.948	0.775
5425	0.953	0.801	0.967	0.814
5430	0.957	0.817	0.968	0.830
5435	0.951	0.793	0.924	0.813
5440	0.953	0.801	0.967	0.814
5445	0.957	0.817	0.954	0.832
5450	0.950	0.785	0.966	0.797
5455	0.955	0.809	0.983	0.819
5460	0.951	0.793	0.967	0.806
5465	0.948	0.777	0.950	0.792
5470	0.957	0.817	0.968	0.830
5475	0.951	0.794	0.983	0.803
5480	0.945	0.764	0.882	0.789
5485	0.951	0.793	0.952	0.808
5490	0.941	0.744	0.931	0.761
5495	0.953	0.801	0.967	0.814
5500	0.953	0.801	0.938	0.819

5505	0.950	0.785	0.951	0.800
5510	0.953	0.801	0.952	0.816
5515	0.951	0.793	0.967	0.806
5520	0.945	0.761	0.964	0.771
5525	0.955	0.809	0.953	0.824
5530	0.941	0.745	0.981	0.750
5535	0.950	0.785	0.966	0.797
5540	0.948	0.777	0.966	0.789
5545	0.939	0.736	0.945	0.748
5550	0.953	0.801	0.967	0.814
5555	0.960	0.832	0.955	0.848
5560	0.957	0.817	0.954	0.832
5565	0.953	0.801	0.967	0.814
5570	0.960	0.832	0.984	0.844
5575	0.957	0.818	1.000	0.825
5580	0.946	0.769	0.934	0.786
5585	0.953	0.801	0.952	0.816
5590	0.955	0.809	0.939	0.827
5595	0.953	0.801	0.967	0.814
5600	0.943	0.753	0.964	0.763
5605	0.951	0.793	0.938	0.811
5610	0.938	0.731	0.875	0.757
5615	0.951	0.793	0.967	0.806
5620	0.962	0.840	0.970	0.853
5625	0.946	0.769	0.949	0.783
5630	0.950	0.785	0.951	0.800
5635	0.951	0.793	0.952	0.808
5640	0.951	0.793	0.938	0.811
5645	0.950	0.785	0.966	0.797
5650	0.948	0.777	0.966	0.789
5655	0.953	0.801	0.967	0.814
5660	0.948	0.777	0.966	0.789
5665	0.953	0.802	1.000	0.809
5670	0.948	0.777	0.950	0.792
5675	0.951	0.794	0.983	0.803
5680	0.953	0.801	0.967	0.814
5685	0.948	0.777	0.935	0.795
5690	0.957	0.817	0.984	0.828
5695	0.957	0.817	0.968	0.830
5700	0.955	0.809	0.983	0.819
5705	0.950	0.785	0.966	0.797
5710	0.951	0.793	0.952	0.808
5715	0.955	0.809	0.983	0.819

5720	0.953	0.801	0.938	0.819
5725	0.955	0.809	0.983	0.819
5730	0.962	0.840	0.970	0.853
5735	0.958	0.824	0.969	0.838
5740	0.955	0.809	0.953	0.824
5745	0.951	0.793	0.967	0.806
5750	0.945	0.761	0.919	0.781
5755	0.953	0.801	0.983	0.811
5760	0.945	0.761	0.948	0.775
5765	0.953	0.801	0.967	0.814
5770	0.948	0.777	0.935	0.795
5775	0.941	0.744	0.946	0.757
5780	0.950	0.785	0.951	0.800
5785	0.953	0.801	0.983	0.811
5790	0.951	0.793	0.938	0.811
5795	0.950	0.785	0.937	0.803
5800	0.938	0.728	0.914	0.746
5805	0.955	0.809	0.953	0.824
5810	0.955	0.809	0.983	0.819
5815	0.957	0.817	0.984	0.828
5820	0.955	0.809	0.953	0.824
5825	0.950	0.785	0.951	0.800
5830	0.950	0.785	0.951	0.800
5835	0.958	0.824	0.955	0.840
5840	0.946	0.769	0.934	0.786
5845	0.943	0.753	0.918	0.772
5850	0.951	0.793	0.967	0.806
5855	0.960	0.833	1.000	0.841
5860	0.953	0.801	0.925	0.821
5865	0.953	0.801	0.952	0.816
5870	0.957	0.818	1.000	0.825
5875	0.953	0.801	0.983	0.811
5880	0.946	0.769	0.949	0.783
5885	0.945	0.761	0.948	0.775
5890	0.950	0.785	0.951	0.800
5895	0.955	0.809	0.953	0.824
5900	0.950	0.785	0.966	0.797
5905	0.950	0.785	0.951	0.800
5910	0.955	0.809	0.953	0.824
5915	0.953	0.801	0.925	0.821
5920	0.957	0.817	0.954	0.832
5925	0.953	0.801	0.952	0.816
5930	0.951	0.793	0.952	0.808

5935	0.955	0.809	0.983	0.819
5940	0.960	0.832	0.969	0.846
5945	0.943	0.753	0.947	0.766
5950	0.957	0.817	0.968	0.830
5955	0.960	0.832	0.984	0.844
5960	0.951	0.793	0.924	0.813
5965	0.945	0.761	0.964	0.771
5970	0.948	0.777	0.935	0.795
5975	0.951	0.793	0.967	0.806
5980	0.950	0.785	0.951	0.800
5985	0.941	0.744	0.946	0.757
5990	0.946	0.769	0.965	0.780
5995	0.955	0.809	0.983	0.819
6000	0.945	0.761	0.919	0.781
6005	0.955	0.809	0.926	0.829
6010	0.964	0.847	0.970	0.861
6015	0.948	0.777	0.935	0.795
6020	0.948	0.778	0.909	0.800
6025	0.941	0.744	0.931	0.761
6030	0.948	0.777	0.922	0.797
6035	0.950	0.785	0.951	0.800
6040	0.951	0.793	0.952	0.808
6045	0.951	0.793	0.952	0.808
6050	0.946	0.769	0.934	0.786
6055	0.943	0.753	0.918	0.772
6060	0.951	0.793	0.924	0.813
6065	0.955	0.809	0.968	0.822
6070	0.950	0.785	0.966	0.797
6075	0.948	0.777	0.950	0.792
6080	0.948	0.778	0.982	0.786
6085	0.958	0.825	0.984	0.836
6090	0.946	0.770	0.982	0.777
6095	0.953	0.801	0.967	0.814
6100	0.939	0.736	0.945	0.748
6105	0.951	0.793	0.952	0.808
6110	0.953	0.801	0.952	0.816
6115	0.955	0.809	0.953	0.824
6120	0.945	0.761	0.919	0.781
6125	0.945	0.761	0.948	0.775
6130	0.955	0.809	0.953	0.824
6135	0.950	0.785	0.966	0.797
6140	0.951	0.793	0.952	0.808
6145	0.957	0.818	1.000	0.825

6150	0.943	0.753	0.964	0.763
6155	0.951	0.794	0.912	0.816
6160	0.960	0.832	0.969	0.846
6165	0.945	0.761	0.964	0.771
6170	0.957	0.817	0.968	0.830
6175	0.951	0.793	0.952	0.808
6180	0.941	0.744	0.931	0.761
6185	0.948	0.777	0.966	0.789
6190	0.951	0.793	0.952	0.808
6195	0.948	0.777	0.950	0.792
6200	0.943	0.753	0.964	0.763
6205	0.945	0.762	0.906	0.784
6210	0.960	0.832	0.984	0.844
6215	0.951	0.794	0.983	0.803
6220	0.946	0.769	0.934	0.786
6225	0.941	0.745	0.917	0.764
6230	0.946	0.769	0.965	0.780
6235	0.953	0.801	0.983	0.811
6240	0.951	0.793	0.952	0.808
6245	0.948	0.777	0.966	0.789
6250	0.958	0.825	0.984	0.836
6255	0.957	0.817	0.968	0.830
6260	0.948	0.777	0.950	0.792
6265	0.950	0.785	0.966	0.797
6270	0.955	0.809	0.953	0.824
6275	0.962	0.841	1.000	0.849
6280	0.946	0.769	0.949	0.783
6285	0.950	0.785	0.966	0.797
6290	0.953	0.801	0.967	0.814
6295	0.950	0.785	0.951	0.800
6300	0.951	0.793	0.938	0.811
6305	0.958	0.825	0.984	0.836
6310	0.958	0.825	0.984	0.836
6315	0.948	0.777	0.966	0.789
6320	0.955	0.809	0.968	0.822
6325	0.950	0.785	0.966	0.797
6330	0.953	0.801	0.967	0.814
6335	0.948	0.777	0.935	0.795
6340	0.945	0.761	0.948	0.775
6345	0.948	0.777	0.922	0.797
6350	0.948	0.777	0.922	0.797
6355	0.958	0.824	0.969	0.838
6360	0.945	0.761	0.933	0.778

6365	0.958	0.824	0.969	0.838
6370	0.962	0.840	0.984	0.851
6375	0.953	0.801	0.983	0.811
6380	0.953	0.801	0.952	0.816
6385	0.964	0.847	0.970	0.861
6390	0.953	0.801	0.938	0.819
6395	0.948	0.777	0.966	0.789
6400	0.950	0.786	0.910	0.808
6405	0.951	0.793	0.952	0.808
6410	0.967	0.863	0.971	0.876
6415	0.958	0.824	0.955	0.840
6420	0.964	0.848	0.985	0.859
6425	0.955	0.809	0.953	0.824
6430	0.955	0.809	0.983	0.819
6435	0.948	0.777	0.950	0.792
6440	0.955	0.809	0.983	0.819
6445	0.953	0.801	0.967	0.814
6450	0.948	0.778	0.982	0.786
6455	0.950	0.785	0.951	0.800
6460	0.950	0.785	0.966	0.797
6465	0.941	0.744	0.946	0.757
6470	0.948	0.778	0.909	0.800
6475	0.951	0.793	0.952	0.808
6480	0.953	0.801	0.938	0.819
6485	0.948	0.777	0.966	0.789
6490	0.951	0.793	0.952	0.808
6495	0.958	0.824	0.969	0.838
6500	0.945	0.762	0.981	0.768
6505	0.953	0.801	0.983	0.811
6510	0.960	0.832	0.955	0.848
6515	0.939	0.736	0.945	0.748
6520	0.951	0.793	0.952	0.808
6525	0.951	0.793	0.952	0.808
6530	0.953	0.801	0.967	0.814
6535	0.953	0.801	0.952	0.816
6540	0.950	0.785	0.966	0.797
6545	0.953	0.801	0.938	0.819
6550	0.960	0.832	0.984	0.844
6555	0.953	0.801	0.952	0.816
6560	0.951	0.793	0.967	0.806
6565	0.950	0.786	0.982	0.794
6570	0.955	0.809	0.968	0.822
6575	0.960	0.832	0.984	0.844

6580	0.946	0.769	0.921	0.789
6585	0.948	0.777	0.950	0.792
6590	0.955	0.809	0.953	0.824
6595	0.946	0.769	0.949	0.783
6600	0.955	0.809	0.953	0.824
6605	0.955	0.809	0.939	0.827
6610	0.943	0.753	0.947	0.766
6615	0.950	0.786	0.982	0.794
6620	0.946	0.769	0.965	0.780
6625	0.953	0.801	0.983	0.811
6630	0.943	0.753	0.964	0.763
6635	0.946	0.769	0.921	0.789
6640	0.958	0.824	0.969	0.838
6645	0.953	0.801	0.925	0.821
6650	0.950	0.786	1.000	0.791
6655	0.953	0.801	0.967	0.814
6660	0.957	0.817	0.984	0.828
6665	0.946	0.769	0.949	0.783
6670	0.938	0.728	0.914	0.746
6675	0.957	0.817	0.984	0.828
6680	0.955	0.809	0.939	0.827
6685	0.950	0.785	0.966	0.797
6690	0.953	0.801	0.983	0.811
6695	0.943	0.753	0.932	0.769
6700	0.960	0.832	0.984	0.844
6705	0.948	0.777	0.966	0.789
6710	0.948	0.777	0.966	0.789
6715	0.943	0.753	0.964	0.763
6720	0.950	0.785	0.966	0.797
6725	0.950	0.785	0.937	0.803
6730	0.943	0.753	0.964	0.763
6735	0.953	0.801	0.925	0.821
6740	0.953	0.801	0.983	0.811
6745	0.946	0.769	0.965	0.780
6750	0.958	0.824	0.955	0.840
6755	0.958	0.825	0.941	0.842
6760	0.948	0.778	0.909	0.800
6765	0.951	0.793	0.952	0.808
6770	0.950	0.786	0.982	0.794
6775	0.951	0.793	0.938	0.811
6780	0.957	0.817	0.968	0.830
6785	0.946	0.769	0.949	0.783
6790	0.953	0.801	0.967	0.814

6795	0.945	0.761	0.948	0.775
6800	0.962	0.841	1.000	0.849
6805	0.946	0.770	0.982	0.777
6810	0.955	0.809	0.953	0.824
6815	0.946	0.769	0.934	0.786
6820	0.953	0.801	0.967	0.814
6825	0.950	0.785	0.966	0.797
6830	0.953	0.801	0.983	0.811
6835	0.945	0.762	1.000	0.765
6840	0.953	0.801	0.967	0.814
6845	0.948	0.777	0.966	0.789
6850	0.950	0.785	0.966	0.797
6855	0.955	0.810	1.000	0.817
6860	0.958	0.824	0.969	0.838
6865	0.946	0.769	0.949	0.783
6870	0.950	0.786	0.982	0.794
6875	0.951	0.793	0.967	0.806
6880	0.953	0.801	0.983	0.811
6885	0.958	0.825	1.000	0.833
6890	0.946	0.769	0.965	0.780
6895	0.951	0.793	0.952	0.808
6900	0.951	0.794	0.983	0.803
6905	0.951	0.794	0.983	0.803
6910	0.951	0.793	0.967	0.806
6915	0.951	0.793	0.967	0.806
6920	0.955	0.809	0.953	0.824
6925	0.957	0.818	1.000	0.825
6930	0.962	0.840	0.984	0.851
6935	0.958	0.824	0.969	0.838
6940	0.955	0.809	0.983	0.819
6945	0.951	0.794	0.983	0.803
6950	0.946	0.769	0.949	0.783
6955	0.951	0.793	0.967	0.806
6960	0.946	0.770	0.982	0.777
6965	0.945	0.762	0.981	0.768
6970	0.951	0.793	0.967	0.806
6975	0.962	0.840	0.970	0.853
6980	0.953	0.802	1.000	0.809
6985	0.941	0.745	0.963	0.754
6990	0.953	0.801	0.967	0.814
6995	0.957	0.817	0.954	0.832
7000	0.946	0.769	0.934	0.786
7005	0.950	0.785	0.951	0.800

7010	0.946	0.769	0.949	0.783
7015	0.950	0.785	0.966	0.797
7020	0.945	0.761	0.933	0.778
7025	0.948	0.777	0.966	0.789
7030	0.950	0.785	0.951	0.800
7035	0.953	0.801	0.983	0.811
7040	0.951	0.794	1.000	0.800
7045	0.953	0.801	0.938	0.819
7050	0.951	0.793	0.952	0.808
7055	0.936	0.719	0.943	0.730
7060	0.943	0.753	0.947	0.766
7065	0.950	0.785	0.951	0.800
7070	0.950	0.786	1.000	0.791
7075	0.951	0.793	0.938	0.811
7080	0.943	0.753	0.947	0.766
7085	0.950	0.785	0.937	0.803
7090	0.939	0.736	0.962	0.745
7095	0.945	0.762	0.981	0.768
7100	0.945	0.761	0.948	0.775
7105	0.955	0.809	0.983	0.819
7110	0.948	0.777	0.950	0.792
7115	0.953	0.801	0.983	0.811
7120	0.946	0.769	0.949	0.783
7125	0.950	0.786	0.982	0.794
7130	0.957	0.817	0.968	0.830
7135	0.953	0.801	0.983	0.811
7140	0.957	0.817	0.968	0.830
7145	0.951	0.793	0.952	0.808
7150	0.945	0.761	0.919	0.781
7155	0.948	0.777	0.950	0.792
7160	0.953	0.802	1.000	0.809
7165	0.946	0.769	0.949	0.783
7170	0.943	0.753	0.947	0.766
7175	0.951	0.794	1.000	0.800
7180	0.953	0.801	0.983	0.811
7185	0.953	0.801	0.983	0.811
7190	0.951	0.794	0.983	0.803
7195	0.960	0.832	0.984	0.844
7200	0.950	0.785	0.937	0.803
7205	0.943	0.753	0.947	0.766
7210	0.951	0.794	1.000	0.800
7215	0.946	0.769	0.934	0.786
7220	0.950	0.785	0.923	0.805

7225	0.957	0.817	0.954	0.832
7230	0.945	0.761	0.948	0.775
7235	0.950	0.785	0.966	0.797
7240	0.939	0.736	0.930	0.752
7245	0.936	0.720	0.912	0.738
7250	0.945	0.761	0.919	0.781
7255	0.953	0.801	0.938	0.819
7260	0.948	0.777	0.935	0.795
7265	0.953	0.801	0.983	0.811
7270	0.955	0.810	0.914	0.831
7275	0.960	0.833	1.000	0.841
7280	0.948	0.777	0.966	0.789
7285	0.955	0.810	1.000	0.817
7290	0.953	0.801	0.983	0.811
7295	0.945	0.761	0.948	0.775
7300	0.945	0.761	0.948	0.775
7305	0.943	0.753	0.964	0.763
7310	0.943	0.753	0.918	0.772
7315	0.939	0.736	0.945	0.748
7320	0.957	0.817	0.984	0.828
7325	0.946	0.769	0.949	0.783
7330	0.950	0.785	0.937	0.803
7335	0.946	0.770	0.982	0.777
7340	0.953	0.802	1.000	0.809
7345	0.943	0.753	0.947	0.766
7350	0.953	0.801	0.983	0.811
7355	0.941	0.744	0.946	0.757
7360	0.953	0.801	0.967	0.814
7365	0.946	0.769	0.949	0.783
7370	0.964	0.848	0.985	0.859
7375	0.941	0.744	0.946	0.757
7380	0.951	0.793	0.938	0.811
7385	0.948	0.777	0.950	0.792
7390	0.948	0.777	0.966	0.789
7395	0.953	0.801	0.967	0.814
7400	0.955	0.809	0.983	0.819
7405	0.955	0.809	0.983	0.819
7410	0.939	0.736	0.945	0.748
7415	0.946	0.769	0.949	0.783
7420	0.951	0.793	0.924	0.813
7425	0.948	0.777	0.966	0.789
7430	0.950	0.786	0.982	0.794
7435	0.943	0.753	0.964	0.763

7440	0.957	0.817	0.984	0.828
7445	0.953	0.801	0.983	0.811
7450	0.950	0.785	0.951	0.800
7455	0.953	0.802	1.000	0.809
7460	0.955	0.809	0.939	0.827
7465	0.943	0.753	0.932	0.769
7470	0.946	0.772	0.884	0.797
7475	0.950	0.786	0.982	0.794
7480	0.945	0.761	0.964	0.771
7485	0.957	0.817	0.968	0.830
7490	0.943	0.753	0.947	0.766
7495	0.955	0.809	0.926	0.829
7500	0.943	0.753	0.932	0.769
7505	0.946	0.769	0.934	0.786
7510	0.957	0.817	0.968	0.830
7515	0.955	0.809	0.953	0.824
7520	0.958	0.825	1.000	0.833
7525	0.951	0.793	0.938	0.811
7530	0.945	0.761	0.948	0.775
7535	0.946	0.769	0.949	0.783
7540	0.955	0.809	0.983	0.819
7545	0.945	0.761	0.964	0.771
7550	0.948	0.777	0.950	0.792
7555	0.951	0.793	0.952	0.808
7560	0.941	0.745	0.963	0.754
7565	0.953	0.801	0.967	0.814
7570	0.945	0.762	0.906	0.784
7575	0.955	0.809	0.968	0.822
7580	0.957	0.818	1.000	0.825
7585	0.955	0.809	0.953	0.824
7590	0.953	0.801	0.983	0.811
7595	0.939	0.736	0.945	0.748
7600	0.948	0.777	0.950	0.792
7605	0.945	0.761	0.964	0.771
7610	0.955	0.810	1.000	0.817
7615	0.953	0.801	0.967	0.814
7620	0.943	0.753	0.947	0.766
7625	0.945	0.761	0.964	0.771
7630	0.953	0.801	0.952	0.816
7635	0.953	0.802	1.000	0.809
7640	0.955	0.809	0.953	0.824
7645	0.953	0.801	0.952	0.816
7650	0.948	0.777	0.950	0.792

7655	0.957	0.817	0.968	0.830
7660	0.948	0.777	0.922	0.797
7665	0.946	0.769	0.965	0.780
7670	0.953	0.801	0.983	0.811
7675	0.953	0.801	0.967	0.814
7680	0.958	0.825	0.984	0.836
7685	0.941	0.744	0.946	0.757
7690	0.946	0.769	0.965	0.780
7695	0.958	0.825	0.984	0.836
7700	0.950	0.786	0.982	0.794
7705	0.953	0.801	0.938	0.819
7710	0.943	0.753	0.947	0.766
7715	0.943	0.753	0.964	0.763
7720	0.958	0.824	0.969	0.838
7725	0.945	0.761	0.964	0.771
7730	0.953	0.801	0.983	0.811
7735	0.945	0.761	0.948	0.775
7740	0.948	0.778	0.982	0.786
7745	0.960	0.833	1.000	0.841
7750	0.958	0.824	0.969	0.838
7755	0.945	0.761	0.919	0.781
7760	0.957	0.817	0.984	0.828
7765	0.953	0.801	0.967	0.814
7770	0.948	0.778	0.982	0.786
7775	0.950	0.785	0.966	0.797
7780	0.951	0.793	0.967	0.806
7785	0.946	0.769	0.965	0.780
7790	0.950	0.785	0.966	0.797
7795	0.950	0.786	0.982	0.794
7800	0.948	0.777	0.966	0.789
7805	0.953	0.802	1.000	0.809
7810	0.950	0.785	0.966	0.797
7815	0.946	0.769	0.949	0.783
7820	0.953	0.801	0.967	0.814
7825	0.946	0.770	1.000	0.774
7830	0.951	0.794	1.000	0.800
7835	0.962	0.841	1.000	0.849
7840	0.955	0.809	0.983	0.819
7845	0.943	0.753	0.932	0.769
7850	0.946	0.769	0.965	0.780
7855	0.946	0.769	0.965	0.780
7860	0.957	0.817	0.984	0.828
7865	0.946	0.769	0.921	0.789

7870	0.950	0.786	1.000	0.791
7875	0.946	0.770	0.982	0.777
7880	0.946	0.770	1.000	0.774
7885	0.948	0.777	0.966	0.789
7890	0.946	0.769	0.965	0.780
7895	0.951	0.793	0.967	0.806
7900	0.957	0.817	0.984	0.828
7905	0.945	0.761	0.948	0.775
7910	0.953	0.801	0.983	0.811
7915	0.950	0.786	0.982	0.794
7920	0.955	0.809	0.968	0.822
7925	0.948	0.777	0.966	0.789
7930	0.951	0.794	0.983	0.803
7935	0.953	0.801	0.983	0.811
7940	0.948	0.778	1.000	0.783
7945	0.948	0.777	0.966	0.789
7950	0.953	0.801	0.967	0.814
7955	0.943	0.753	0.981	0.759
7960	0.950	0.785	0.966	0.797
7965	0.931	0.695	0.893	0.714
7970	0.955	0.809	0.983	0.819
7975	0.953	0.801	0.983	0.811
7980	0.945	0.761	0.964	0.771
7985	0.948	0.777	0.950	0.792
7990	0.951	0.794	1.000	0.800
7995	0.951	0.794	0.983	0.803
8000	0.939	0.736	0.945	0.748
8005	0.945	0.761	0.964	0.771
8010	0.938	0.730	1.000	0.727
8015	0.931	0.694	0.958	0.697
8020	0.939	0.737	0.980	0.741
8025	0.939	0.737	0.980	0.741
8030	0.946	0.770	1.000	0.774
8035	0.938	0.730	1.000	0.727
8040	0.934	0.711	0.960	0.716
8045	0.941	0.746	1.000	0.746
8050	0.941	0.745	0.981	0.750
8055	0.943	0.753	0.964	0.763
8060	0.932	0.703	0.979	0.702
8065	0.939	0.736	0.945	0.748
8070	0.938	0.728	0.962	0.735
8075	0.925	0.668	0.902	0.681
8080	0.932	0.702	0.941	0.711

8085	0.951	0.794	0.983	0.803
8090	0.945	0.762	1.000	0.765
8095	0.932	0.703	0.979	0.702
8100	0.943	0.753	0.981	0.759
8105	0.932	0.702	0.925	0.715
8110	0.938	0.729	0.980	0.731
8115	0.936	0.720	0.980	0.722
8120	0.929	0.685	0.939	0.692
8125	0.936	0.720	0.961	0.726
8130	0.927	0.676	0.920	0.687
8135	0.941	0.746	1.000	0.746
8140	0.934	0.711	0.942	0.721
8145	0.934	0.711	0.960	0.716
8150	0.939	0.738	1.000	0.737
8155	0.936	0.719	0.943	0.730
8160	0.936	0.720	0.980	0.722
8165	0.931	0.694	0.907	0.710
8170	0.945	0.761	0.964	0.771
8175	0.948	0.778	1.000	0.783
8180	0.934	0.711	0.960	0.716
8185	0.934	0.711	0.960	0.716
8190	0.950	0.785	0.951	0.800
8195	0.951	0.793	0.967	0.806
8200	0.941	0.745	0.981	0.750
8205	0.953	0.801	0.967	0.814
8210	0.955	0.810	1.000	0.817
8215	0.948	0.777	0.950	0.792
8220	0.948	0.777	0.966	0.789
8225	0.953	0.802	1.000	0.809
8230	0.958	0.825	0.984	0.836
8235	0.948	0.777	0.966	0.789
8240	0.943	0.753	0.918	0.772
8245	0.948	0.777	0.922	0.797
8250	0.941	0.745	0.963	0.754
8255	0.946	0.770	0.982	0.777
8260	0.948	0.778	0.982	0.786
8265	0.946	0.769	0.949	0.783
8270	0.951	0.794	1.000	0.800
8275	0.945	0.762	0.981	0.768
8280	0.946	0.770	0.982	0.777
8285	0.950	0.785	0.966	0.797
8290	0.945	0.761	0.964	0.771
8295	0.945	0.761	0.948	0.775

8300	0.948	0.777	0.935	0.795
8305	0.939	0.736	0.945	0.748
8310	0.941	0.744	0.931	0.761
8315	0.945	0.761	0.964	0.771
8320	0.939	0.736	0.945	0.748
8325	0.957	0.817	0.968	0.830
8330	0.938	0.728	0.914	0.746
8335	0.943	0.753	0.947	0.766
8340	0.955	0.810	1.000	0.817
8345	0.948	0.778	0.982	0.786
8350	0.962	0.840	0.984	0.851
8355	0.945	0.761	0.948	0.775
8360	0.943	0.753	0.981	0.759
8365	0.951	0.793	0.952	0.808
8370	0.939	0.737	0.915	0.755
8375	0.941	0.744	0.946	0.757
8380	0.950	0.786	0.982	0.794
8385	0.945	0.761	0.964	0.771
8390	0.939	0.736	0.962	0.745
8395	0.948	0.778	0.982	0.786
8400	0.946	0.769	0.965	0.780
8405	0.946	0.769	0.949	0.783
8410	0.951	0.794	0.983	0.803
8415	0.951	0.793	0.952	0.808
8420	0.943	0.753	0.964	0.763
8425	0.943	0.753	0.947	0.766
8430	0.948	0.778	0.982	0.786
8435	0.945	0.761	0.933	0.778
8440	0.943	0.753	0.932	0.769
8445	0.950	0.785	0.951	0.800
8450	0.946	0.769	0.949	0.783
8455	0.951	0.794	0.983	0.803
8460	0.955	0.809	0.983	0.819
8465	0.945	0.761	0.964	0.771
8470	0.951	0.793	0.967	0.806
8475	0.943	0.753	0.947	0.766
8480	0.945	0.761	0.948	0.775
8485	0.948	0.777	0.950	0.792
8490	0.948	0.777	0.966	0.789
8495	0.945	0.761	0.964	0.771
8500	0.939	0.737	0.902	0.759
8505	0.950	0.786	0.982	0.794
8510	0.945	0.761	0.948	0.775

8515	0.953	0.801	0.952	0.816
8520	0.946	0.769	0.949	0.783
8525	0.955	0.809	0.983	0.819
8530	0.951	0.793	0.952	0.808
8535	0.939	0.736	0.962	0.745
8540	0.939	0.737	0.915	0.755
8545	0.948	0.777	0.950	0.792
8550	0.948	0.777	0.950	0.792
8555	0.953	0.801	0.967	0.814
8560	0.943	0.753	0.964	0.763
8565	0.953	0.801	0.983	0.811
8570	0.943	0.753	0.947	0.766
8575	0.943	0.753	0.964	0.763
8580	0.943	0.753	0.932	0.769
8585	0.950	0.785	0.951	0.800
8590	0.943	0.753	0.932	0.769
8595	0.939	0.736	0.945	0.748
8600	0.943	0.754	1.000	0.756
8605	0.941	0.745	0.917	0.764
8610	0.958	0.824	0.969	0.838
8615	0.941	0.745	0.963	0.754
8620	0.943	0.753	0.981	0.759
8625	0.943	0.753	0.964	0.763
8630	0.948	0.778	0.982	0.786
8635	0.936	0.719	0.927	0.734
8640	0.946	0.769	0.949	0.783
8645	0.953	0.801	0.925	0.821
8650	0.946	0.769	0.934	0.786
8655	0.945	0.761	0.964	0.771
8660	0.955	0.809	0.983	0.819
8665	0.929	0.685	0.939	0.692
8670	0.950	0.785	0.966	0.797
8675	0.946	0.769	0.949	0.783
8680	0.945	0.761	0.964	0.771
8685	0.941	0.744	0.946	0.757
8690	0.941	0.744	0.946	0.757
8695	0.950	0.786	0.982	0.794
8700	0.950	0.785	0.951	0.800
8705	0.950	0.785	0.937	0.803
8710	0.950	0.785	0.966	0.797
8715	0.943	0.753	0.981	0.759
8720	0.943	0.753	0.932	0.769
8725	0.941	0.744	0.946	0.757

8730	0.957	0.818	1.000	0.825
8735	0.945	0.762	0.981	0.768
8740	0.945	0.761	0.948	0.775
8745	0.938	0.730	1.000	0.727
8750	0.948	0.777	0.966	0.789
8755	0.950	0.785	0.966	0.797
8760	0.936	0.719	0.927	0.734
8765	0.946	0.770	0.982	0.777
8770	0.953	0.801	0.952	0.816
8775	0.951	0.793	0.952	0.808
8780	0.948	0.778	0.982	0.786
8785	0.951	0.793	0.952	0.808
8790	0.945	0.762	0.981	0.768
8795	0.950	0.785	0.966	0.797
8800	0.946	0.769	0.965	0.780
8805	0.943	0.753	0.964	0.763
8810	0.946	0.769	0.965	0.780
8815	0.950	0.785	0.951	0.800
8820	0.943	0.753	0.932	0.769
8825	0.946	0.769	0.949	0.783
8830	0.950	0.785	0.937	0.803
8835	0.941	0.744	0.931	0.761
8840	0.939	0.736	0.930	0.752
8845	0.948	0.778	1.000	0.783
8850	0.939	0.736	0.962	0.745
8855	0.939	0.736	0.962	0.745
8860	0.936	0.719	0.943	0.730
8865	0.948	0.778	0.982	0.786
8870	0.939	0.737	0.980	0.741
8875	0.939	0.736	0.962	0.745
8880	0.948	0.778	1.000	0.783
8885	0.936	0.720	0.961	0.726
8890	0.941	0.745	0.981	0.750
8895	0.938	0.728	0.914	0.746
8900	0.934	0.712	0.979	0.712
8905	0.946	0.769	0.949	0.783
8910	0.946	0.770	1.000	0.774
8915	0.938	0.728	0.944	0.739
8920	0.939	0.736	0.930	0.752
8925	0.951	0.793	0.967	0.806
8930	0.938	0.729	0.980	0.731
8935	0.943	0.754	1.000	0.756
8940	0.932	0.702	0.941	0.711

8945	0.948	0.777	0.966	0.789
8950	0.943	0.753	0.981	0.759
8955	0.938	0.730	1.000	0.727
8960	0.945	0.762	0.981	0.768
8965	0.943	0.753	0.964	0.763
8970	0.943	0.753	0.964	0.763
8975	0.938	0.729	0.980	0.731
8980	0.950	0.786	1.000	0.791
8985	0.932	0.703	0.959	0.707
8990	0.943	0.753	0.964	0.763
8995	0.948	0.777	0.950	0.792
9000	0.941	0.744	0.946	0.757
9005	0.941	0.745	0.963	0.754
9010	0.939	0.736	0.945	0.748
9015	0.946	0.770	0.982	0.777
9020	0.938	0.728	0.962	0.735
9025	0.939	0.738	1.000	0.737
9030	0.939	0.736	0.945	0.748
9035	0.945	0.762	0.981	0.768
9040	0.936	0.719	0.943	0.730
9045	0.945	0.761	0.964	0.771
9050	0.941	0.745	0.963	0.754
9055	0.939	0.737	0.980	0.741
9060	0.941	0.745	0.917	0.764
9065	0.946	0.769	0.965	0.780
9070	0.936	0.719	0.943	0.730
9075	0.945	0.761	0.948	0.775
9080	0.941	0.745	0.963	0.754
9085	0.934	0.711	0.942	0.721
9090	0.946	0.770	0.982	0.777
9095	0.941	0.745	0.981	0.750
9100	0.938	0.728	0.929	0.743
9105	0.946	0.769	0.965	0.780
9110	0.946	0.769	0.965	0.780
9115	0.936	0.719	0.943	0.730
9120	0.946	0.770	1.000	0.774
9125	0.932	0.703	0.959	0.707
9130	0.946	0.769	0.949	0.783
9135	0.951	0.794	1.000	0.800
9140	0.936	0.720	0.961	0.726
9145	0.943	0.753	0.981	0.759
9150	0.945	0.761	0.964	0.771
9155	0.948	0.777	0.950	0.792

9160	0.932	0.702	0.925	0.715
9165	0.955	0.810	1.000	0.817
9170	0.945	0.762	1.000	0.765
9175	0.943	0.753	0.947	0.766
9180	0.948	0.777	0.966	0.789
9185	0.941	0.744	0.946	0.757
9190	0.943	0.753	0.932	0.769
9195	0.938	0.728	0.929	0.743
9200	0.948	0.777	0.966	0.789
9205	0.936	0.720	0.980	0.722
9210	0.941	0.746	1.000	0.746
9215	0.939	0.736	0.930	0.752
9220	0.953	0.801	0.983	0.811
9225	0.941	0.745	0.903	0.767
9230	0.939	0.737	0.915	0.755
9235	0.938	0.728	0.962	0.735
9240	0.943	0.753	0.932	0.769
9245	0.941	0.744	0.931	0.761
9250	0.950	0.785	0.966	0.797
9255	0.945	0.762	0.981	0.768
9260	0.939	0.736	0.945	0.748
9265	0.931	0.694	0.958	0.697
9270	0.936	0.719	0.943	0.730
9275	0.943	0.753	0.981	0.759
9280	0.950	0.786	1.000	0.791
9285	0.929	0.687	0.877	0.709
9290	0.946	0.769	0.965	0.780
9295	0.939	0.736	0.962	0.745
9300	0.939	0.736	0.945	0.748
9305	0.939	0.736	0.930	0.752
9310	0.950	0.785	0.951	0.800
9315	0.932	0.703	0.895	0.723
9320	0.945	0.761	0.933	0.778
9325	0.953	0.801	0.983	0.811
9330	0.934	0.711	0.926	0.725
9335	0.934	0.711	0.960	0.716
9340	0.939	0.736	0.962	0.745
9345	0.946	0.769	0.965	0.780
9350	0.938	0.728	0.914	0.746
9355	0.939	0.736	0.962	0.745
9360	0.941	0.744	0.931	0.761
9365	0.945	0.762	0.981	0.768
9370	0.950	0.785	0.951	0.800

9375	0.945	0.761	0.933	0.778
9380	0.945	0.762	0.981	0.768
9385	0.945	0.762	0.981	0.768
9390	0.938	0.728	0.962	0.735
9395	0.941	0.744	0.946	0.757
9400	0.943	0.753	0.964	0.763
9405	0.953	0.801	0.983	0.811
9410	0.948	0.778	0.982	0.786
9415	0.943	0.753	0.981	0.759
9420	0.946	0.769	0.965	0.780
9425	0.941	0.745	0.981	0.750
9430	0.953	0.801	0.967	0.814
9435	0.939	0.737	0.980	0.741
9440	0.948	0.777	0.966	0.789
9445	0.945	0.761	0.948	0.775
9450	0.941	0.745	0.963	0.754
9455	0.943	0.753	0.964	0.763
9460	0.945	0.761	0.964	0.771
9465	0.938	0.728	0.962	0.735
9470	0.939	0.736	0.930	0.752
9475	0.941	0.744	0.946	0.757
9480	0.945	0.761	0.964	0.771
9485	0.943	0.753	0.947	0.766
9490	0.939	0.736	0.930	0.752
9495	0.943	0.753	0.947	0.766
9500	0.943	0.754	1.000	0.756
9505	0.943	0.753	0.964	0.763
9510	0.950	0.786	1.000	0.791
9515	0.943	0.753	0.918	0.772
9520	0.936	0.719	0.943	0.730
9525	0.946	0.769	0.934	0.786
9530	0.938	0.728	0.962	0.735
9535	0.945	0.761	0.933	0.778
9540	0.934	0.711	0.911	0.729
9545	0.945	0.761	0.964	0.771
9550	0.948	0.777	0.950	0.792
9555	0.938	0.728	0.914	0.746
9560	0.950	0.786	1.000	0.791
9565	0.943	0.753	0.947	0.766
9570	0.948	0.778	1.000	0.783
9575	0.946	0.769	0.965	0.780
9580	0.938	0.728	0.929	0.743
9585	0.938	0.728	0.962	0.735

9590	0.936	0.720	0.961	0.726
9595	0.945	0.761	0.964	0.771
9600	0.927	0.676	0.938	0.682
9605	0.950	0.786	0.982	0.794
9610	0.950	0.786	1.000	0.791
9615	0.939	0.736	0.962	0.745
9620	0.948	0.777	0.966	0.789
9625	0.943	0.753	0.964	0.763
9630	0.943	0.753	0.932	0.769
9635	0.948	0.777	0.950	0.792
9640	0.936	0.720	0.961	0.726
9645	0.943	0.753	0.981	0.759
9650	0.946	0.769	0.949	0.783
9655	0.939	0.736	0.930	0.752
9660	0.943	0.753	0.964	0.763
9665	0.948	0.777	0.966	0.789
9670	0.946	0.770	0.982	0.777
9675	0.943	0.753	0.964	0.763
9680	0.939	0.737	0.980	0.741
9685	0.946	0.770	1.000	0.774
9690	0.934	0.711	0.942	0.721
9695	0.943	0.753	0.964	0.763
9700	0.938	0.729	0.980	0.731
9705	0.951	0.793	0.952	0.808
9710	0.948	0.778	1.000	0.783
9715	0.932	0.703	0.959	0.707
9720	0.941	0.744	0.946	0.757
9725	0.945	0.761	0.948	0.775
9730	0.946	0.770	0.982	0.777
9735	0.945	0.762	0.981	0.768
9740	0.943	0.754	1.000	0.756
9745	0.943	0.753	0.947	0.766
9750	0.946	0.770	0.982	0.777
9755	0.943	0.753	0.964	0.763
9760	0.941	0.744	0.946	0.757
9765	0.938	0.728	0.929	0.743
9770	0.943	0.753	0.918	0.772
9775	0.945	0.761	0.948	0.775
9780	0.936	0.720	0.961	0.726
9785	0.946	0.769	0.965	0.780
9790	0.948	0.778	1.000	0.783
9795	0.943	0.753	0.947	0.766
9800	0.939	0.736	0.945	0.748

9805	0.929	0.685	0.922	0.696
9810	0.948	0.778	1.000	0.783
9815	0.945	0.762	1.000	0.765
9820	0.939	0.736	0.962	0.745
9825	0.934	0.711	0.942	0.721
9830	0.939	0.736	0.945	0.748
9835	0.941	0.745	0.981	0.750
9840	0.943	0.753	0.947	0.766
9845	0.943	0.753	0.964	0.763
9850	0.955	0.810	1.000	0.817
9855	0.953	0.801	0.983	0.811
9860	0.941	0.744	0.931	0.761
9865	0.941	0.745	0.963	0.754
9870	0.939	0.736	0.945	0.748
9875	0.945	0.762	0.981	0.768
9880	0.962	0.841	1.000	0.849
9885	0.938	0.728	0.962	0.735
9890	0.939	0.736	0.945	0.748
9895	0.934	0.711	0.942	0.721
9900	0.943	0.753	0.964	0.763
9905	0.934	0.713	1.000	0.708
9910	0.931	0.694	0.907	0.710
9915	0.946	0.770	1.000	0.774
9920	0.939	0.737	0.980	0.741
9925	0.950	0.785	0.966	0.797
9930	0.936	0.719	0.943	0.730
9935	0.939	0.736	0.962	0.745
9940	0.941	0.745	0.981	0.750
9945	0.941	0.745	0.963	0.754
9950	0.945	0.761	0.964	0.771
9955	0.939	0.736	0.962	0.745
9960	0.946	0.769	0.965	0.780
9965	0.946	0.769	0.965	0.780
9970	0.932	0.703	0.959	0.707
9975	0.951	0.794	1.000	0.800
9980	0.932	0.703	0.959	0.707
9985	0.945	0.762	1.000	0.765
9990	0.939	0.737	0.980	0.741
9995	0.948	0.778	0.982	0.786
10000	0.936	0.720	0.980	0.722

(6) Performance of IFS with decision tree on the list yielded by MCFS

Number of features	ACC	MCC	Precision	F1-measure

5	0.806	0.445	0.408	0.525
10	0.823	0.436	0.431	0.523
15	0.799	0.404	0.390	0.496
20	0.811	0.435	0.413	0.520
25	0.825	0.474	0.440	0.551
30	0.825	0.433	0.433	0.521
35	0.828	0.451	0.442	0.535
40	0.804	0.399	0.396	0.493
45	0.795	0.361	0.375	0.464
50	0.802	0.378	0.388	0.477
55	0.818	0.385	0.412	0.483
60	0.816	0.425	0.418	0.514
65	0.802	0.391	0.391	0.486
70	0.844	0.473	0.475	0.554
75	0.853	0.528	0.496	0.597
80	0.809	0.377	0.397	0.476
85	0.820	0.394	0.417	0.490
90	0.816	0.455	0.424	0.535
95	0.811	0.410	0.407	0.502
100	0.809	0.364	0.393	0.466
105	0.816	0.406	0.414	0.500
110	0.851	0.519	0.492	0.590
115	0.808	0.399	0.400	0.493
120	0.827	0.436	0.437	0.524
125	0.832	0.427	0.444	0.517
130	0.823	0.418	0.427	0.510
135	0.795	0.342	0.369	0.449
140	0.828	0.385	0.430	0.482
145	0.816	0.413	0.415	0.505
150	0.802	0.403	0.394	0.496
155	0.789	0.351	0.364	0.455
160	0.801	0.425	0.397	0.511
165	0.853	0.512	0.496	0.585
170	0.816	0.406	0.414	0.500
175	0.790	0.347	0.365	0.452
180	0.809	0.401	0.403	0.495
185	0.827	0.418	0.433	0.510
190	0.813	0.413	0.410	0.505
195	0.825	0.457	0.437	0.539
200	0.787	0.316	0.351	0.428
205	0.797	0.364	0.378	0.466
210	0.790	0.347	0.365	0.452
215	0.830	0.418	0.440	0.510

220	0.830	0.477	0.449	0.555
225	0.804	0.399	0.396	0.493
230	0.809	0.383	0.398	0.481
235	0.775	0.376	0.358	0.472
240	0.818	0.403	0.416	0.498
245	0.834	0.494	0.457	0.568
250	0.820	0.394	0.417	0.490
255	0.842	0.470	0.471	0.552
260	0.828	0.403	0.434	0.497
265	0.832	0.457	0.449	0.540
270	0.811	0.392	0.403	0.488
275	0.823	0.448	0.433	0.532
280	0.808	0.380	0.395	0.479
285	0.799	0.354	0.377	0.458
290	0.811	0.404	0.406	0.498
295	0.834	0.419	0.446	0.510
300	0.818	0.385	0.412	0.483
305	0.815	0.373	0.403	0.473
310	0.818	0.409	0.417	0.502
315	0.832	0.451	0.448	0.536
320	0.801	0.369	0.383	0.470
325	0.809	0.389	0.400	0.486
330	0.827	0.430	0.435	0.519
335	0.813	0.357	0.397	0.460
340	0.834	0.448	0.451	0.534
345	0.820	0.442	0.426	0.527
350	0.827	0.424	0.434	0.515
355	0.813	0.407	0.409	0.500
360	0.789	0.402	0.378	0.492
365	0.815	0.385	0.407	0.483
370	0.806	0.384	0.394	0.481
375	0.834	0.442	0.450	0.529
380	0.802	0.378	0.388	0.477
385	0.795	0.335	0.367	0.443
390	0.825	0.433	0.433	0.521
395	0.827	0.412	0.432	0.505
400	0.804	0.343	0.380	0.449
405	0.825	0.421	0.431	0.512
410	0.820	0.436	0.425	0.523
415	0.839	0.469	0.463	0.551
420	0.825	0.409	0.429	0.502
425	0.801	0.350	0.378	0.455
430	0.821	0.433	0.427	0.521

435	0.795	0.309	0.358	0.422
440	0.806	0.345	0.383	0.451
445	0.825	0.409	0.429	0.502
450	0.799	0.366	0.381	0.468
455	0.809	0.395	0.402	0.491
460	0.834	0.454	0.452	0.538
465	0.804	0.393	0.394	0.489
470	0.849	0.500	0.488	0.576
475	0.757	0.248	0.300	0.375
480	0.789	0.376	0.372	0.474
485	0.823	0.436	0.431	0.523
490	0.823	0.442	0.432	0.528
495	0.839	0.486	0.465	0.563
500	0.792	0.369	0.373	0.469
505	0.815	0.404	0.411	0.498
510	0.815	0.416	0.414	0.507
515	0.795	0.329	0.365	0.438
520	0.787	0.342	0.360	0.448
525	0.801	0.344	0.376	0.450
530	0.804	0.330	0.376	0.438
535	0.820	0.430	0.424	0.519
540	0.783	0.331	0.353	0.439
545	0.769	0.229	0.301	0.357
550	0.802	0.378	0.388	0.477
555	0.827	0.448	0.438	0.533
560	0.795	0.348	0.371	0.454
565	0.815	0.391	0.408	0.488
570	0.837	0.472	0.460	0.552
575	0.811	0.398	0.405	0.493
580	0.795	0.386	0.382	0.482
585	0.802	0.378	0.388	0.477
590	0.789	0.305	0.349	0.419
595	0.789	0.357	0.366	0.460
600	0.808	0.355	0.388	0.459
605	0.794	0.306	0.355	0.420
610	0.830	0.442	0.444	0.529
615	0.785	0.307	0.346	0.421
620	0.799	0.360	0.379	0.463
625	0.789	0.357	0.366	0.460
630	0.801	0.363	0.382	0.465
635	0.787	0.342	0.360	0.448
640	0.783	0.331	0.353	0.439
645	0.806	0.306	0.370	0.417

650	0.768	0.296	0.326	0.412
655	0.804	0.387	0.393	0.484
660	0.815	0.379	0.405	0.478
665	0.794	0.378	0.378	0.476
670	0.795	0.374	0.379	0.473
675	0.816	0.394	0.411	0.490
680	0.804	0.368	0.388	0.469
685	0.799	0.334	0.371	0.442
690	0.782	0.315	0.346	0.427
695	0.816	0.413	0.415	0.505
700	0.804	0.393	0.394	0.489
705	0.790	0.373	0.372	0.472
710	0.789	0.325	0.356	0.435
715	0.789	0.389	0.375	0.483
720	0.809	0.351	0.390	0.455
725	0.809	0.364	0.393	0.466
730	0.802	0.366	0.385	0.467
735	0.795	0.361	0.375	0.464
740	0.790	0.334	0.361	0.442
745	0.785	0.313	0.348	0.426
750	0.818	0.385	0.412	0.483
755	0.809	0.358	0.392	0.461
760	0.809	0.351	0.390	0.455
765	0.795	0.361	0.375	0.464
770	0.818	0.397	0.415	0.493
775	0.771	0.287	0.326	0.405
780	0.794	0.339	0.366	0.447
785	0.804	0.362	0.386	0.464
790	0.785	0.313	0.348	0.426
795	0.809	0.370	0.395	0.471
800	0.802	0.333	0.375	0.441
805	0.790	0.301	0.350	0.415
810	0.821	0.463	0.433	0.542
815	0.766	0.231	0.299	0.360
820	0.763	0.262	0.309	0.386
825	0.778	0.276	0.328	0.396
830	0.782	0.308	0.343	0.422
835	0.790	0.328	0.359	0.437
840	0.804	0.296	0.364	0.408
845	0.789	0.357	0.366	0.460
850	0.785	0.266	0.331	0.386
855	0.804	0.381	0.391	0.479
860	0.766	0.231	0.299	0.360

865	0.820	0.381	0.414	0.480
870	0.809	0.383	0.398	0.481
875	0.799	0.392	0.387	0.487
880	0.790	0.334	0.361	0.442
885	0.785	0.307	0.346	0.421
890	0.801	0.356	0.380	0.460
895	0.768	0.234	0.302	0.362
900	0.776	0.253	0.317	0.377
905	0.835	0.410	0.449	0.503
910	0.769	0.285	0.324	0.404
915	0.787	0.296	0.344	0.411
920	0.808	0.342	0.385	0.448
925	0.773	0.262	0.318	0.385
930	0.808	0.367	0.392	0.469
935	0.769	0.271	0.319	0.393
940	0.769	0.257	0.313	0.381
945	0.816	0.394	0.411	0.490
950	0.775	0.292	0.331	0.409
955	0.804	0.393	0.394	0.489
960	0.828	0.427	0.438	0.517
965	0.795	0.329	0.365	0.438
970	0.789	0.298	0.347	0.413
975	0.783	0.331	0.353	0.439
980	0.808	0.361	0.390	0.464
985	0.820	0.424	0.423	0.514
990	0.782	0.341	0.354	0.447
995	0.830	0.454	0.445	0.538
1000	0.806	0.365	0.389	0.467
1005	0.787	0.296	0.344	0.411
1010	0.801	0.382	0.387	0.480
1015	0.806	0.365	0.389	0.467
1020	0.799	0.392	0.387	0.487
1025	0.808	0.392	0.398	0.488
1030	0.766	0.273	0.317	0.395
1035	0.816	0.376	0.407	0.475
1040	0.799	0.347	0.375	0.453
1045	0.780	0.339	0.352	0.445
1050	0.818	0.445	0.424	0.529
1055	0.787	0.348	0.362	0.453
1060	0.794	0.352	0.370	0.457
1065	0.815	0.404	0.411	0.498
1070	0.818	0.427	0.421	0.516
1075	0.811	0.410	0.407	0.502

1080	0.766	0.293	0.324	0.410
1085	0.806	0.414	0.401	0.504
1090	0.820	0.418	0.422	0.509
1095	0.809	0.351	0.390	0.455
1100	0.789	0.338	0.360	0.445
1105	0.778	0.317	0.343	0.429
1110	0.782	0.315	0.346	0.427
1115	0.801	0.350	0.378	0.455
1120	0.783	0.311	0.346	0.424
1125	0.778	0.241	0.314	0.366
1130	0.780	0.251	0.319	0.374
1135	0.797	0.318	0.364	0.429
1140	0.801	0.356	0.380	0.460
1145	0.801	0.311	0.365	0.422
1150	0.799	0.360	0.379	0.463
1155	0.764	0.278	0.317	0.398
1160	0.794	0.346	0.368	0.452
1165	0.789	0.357	0.366	0.460
1170	0.785	0.293	0.341	0.410
1175	0.792	0.375	0.375	0.474
1180	0.808	0.380	0.395	0.479
1185	0.782	0.275	0.331	0.394
1190	0.794	0.313	0.358	0.425
1195	0.809	0.351	0.390	0.455
1200	0.766	0.217	0.293	0.348
1205	0.820	0.363	0.409	0.464
1210	0.813	0.382	0.403	0.481
1215	0.806	0.365	0.389	0.467
1220	0.768	0.262	0.313	0.385
1225	0.802	0.359	0.383	0.462
1230	0.790	0.308	0.352	0.421
1235	0.794	0.339	0.366	0.447
1240	0.811	0.360	0.395	0.463
1245	0.795	0.342	0.369	0.449
1250	0.763	0.234	0.298	0.363
1255	0.783	0.344	0.357	0.449
1260	0.792	0.304	0.352	0.417
1265	0.778	0.269	0.325	0.390
1270	0.782	0.268	0.328	0.388
1275	0.802	0.397	0.393	0.491
1280	0.768	0.227	0.298	0.356
1285	0.801	0.350	0.378	0.455
1290	0.789	0.298	0.347	0.413

1295	0.818	0.340	0.400	0.444
1300	0.778	0.276	0.328	0.396
1305	0.790	0.314	0.354	0.427
1310	0.763	0.234	0.298	0.363
1315	0.780	0.272	0.328	0.392
1320	0.797	0.345	0.372	0.451
1325	0.809	0.377	0.397	0.476
1330	0.780	0.258	0.322	0.380
1335	0.775	0.312	0.338	0.425
1340	0.789	0.318	0.354	0.430
1345	0.820	0.424	0.423	0.514
1350	0.797	0.332	0.368	0.440
1355	0.769	0.264	0.316	0.387
1360	0.797	0.370	0.380	0.471
1365	0.790	0.334	0.361	0.442
1370	0.797	0.332	0.368	0.440
1375	0.813	0.413	0.410	0.505
1380	0.806	0.319	0.375	0.429
1385	0.789	0.298	0.347	0.413
1390	0.797	0.332	0.368	0.440
1395	0.783	0.277	0.333	0.396
1400	0.806	0.402	0.399	0.495
1405	0.766	0.259	0.311	0.384
1410	0.811	0.367	0.397	0.468
1415	0.785	0.280	0.336	0.398
1420	0.782	0.315	0.346	0.427
1425	0.818	0.409	0.417	0.502
1430	0.808	0.392	0.398	0.488
1435	0.776	0.260	0.320	0.383
1440	0.792	0.350	0.368	0.455
1445	0.799	0.385	0.386	0.482
1450	0.795	0.342	0.369	0.449
1455	0.771	0.287	0.326	0.405
1460	0.759	0.236	0.296	0.365
1465	0.782	0.360	0.360	0.462
1470	0.799	0.354	0.377	0.458
1475	0.804	0.362	0.386	0.464
1480	0.804	0.393	0.394	0.489
1485	0.754	0.236	0.293	0.366
1490	0.802	0.378	0.388	0.477
1495	0.808	0.348	0.387	0.453
1500	0.778	0.276	0.328	0.396
1505	0.766	0.266	0.314	0.389

1510	0.782	0.268	0.328	0.388
1515	0.787	0.289	0.341	0.406
1520	0.799	0.334	0.371	0.442
1525	0.801	0.369	0.383	0.470
1530	0.783	0.270	0.331	0.390
1535	0.783	0.331	0.353	0.439
1540	0.778	0.317	0.343	0.429
1545	0.792	0.337	0.364	0.444
1550	0.780	0.292	0.336	0.409
1555	0.806	0.352	0.385	0.456
1560	0.776	0.321	0.343	0.432
1565	0.780	0.345	0.354	0.450
1570	0.808	0.380	0.395	0.479
1575	0.790	0.341	0.363	0.447
1580	0.799	0.334	0.371	0.442
1585	0.782	0.281	0.333	0.400
1590	0.815	0.354	0.398	0.457
1595	0.789	0.318	0.354	0.430
1600	0.797	0.325	0.366	0.435
1605	0.802	0.384	0.390	0.482
1610	0.782	0.335	0.352	0.442
1615	0.797	0.395	0.386	0.489
1620	0.790	0.314	0.354	0.427
1625	0.815	0.328	0.390	0.434
1630	0.799	0.360	0.379	0.463
1635	0.815	0.410	0.412	0.502
1640	0.792	0.337	0.364	0.444
1645	0.790	0.354	0.367	0.457
1650	0.780	0.279	0.331	0.398
1655	0.775	0.298	0.333	0.414
1660	0.766	0.259	0.311	0.384
1665	0.811	0.410	0.407	0.502
1670	0.799	0.341	0.373	0.448
1675	0.804	0.368	0.388	0.469
1680	0.783	0.344	0.357	0.449
1685	0.802	0.314	0.368	0.424
1690	0.809	0.395	0.402	0.491
1695	0.832	0.445	0.447	0.531
1700	0.815	0.360	0.400	0.462
1705	0.795	0.374	0.379	0.473
1710	0.804	0.362	0.386	0.464
1715	0.813	0.388	0.405	0.486
1720	0.808	0.380	0.395	0.479

1725	0.801	0.337	0.374	0.444
1730	0.754	0.264	0.304	0.388
1735	0.804	0.356	0.384	0.459
1740	0.789	0.345	0.362	0.450
1745	0.794	0.306	0.355	0.420
1750	0.804	0.412	0.399	0.502
1755	0.790	0.334	0.361	0.442
1760	0.769	0.298	0.329	0.414
1765	0.783	0.270	0.331	0.390
1770	0.799	0.392	0.387	0.487
1775	0.780	0.312	0.343	0.425
1780	0.797	0.351	0.374	0.456
1785	0.785	0.340	0.357	0.446
1790	0.799	0.354	0.377	0.458
1795	0.780	0.319	0.345	0.430
1800	0.806	0.352	0.385	0.456
1805	0.794	0.365	0.374	0.466
1810	0.801	0.425	0.397	0.511
1815	0.802	0.378	0.388	0.477
1820	0.802	0.353	0.381	0.457
1825	0.823	0.400	0.424	0.495
1830	0.783	0.337	0.355	0.444
1835	0.827	0.388	0.427	0.485
1840	0.823	0.406	0.425	0.500
1845	0.808	0.361	0.390	0.464
1850	0.820	0.375	0.412	0.475
1855	0.801	0.350	0.378	0.455
1860	0.820	0.375	0.412	0.475
1865	0.811	0.373	0.398	0.473
1870	0.808	0.392	0.398	0.488
1875	0.792	0.375	0.375	0.474
1880	0.806	0.384	0.394	0.481
1885	0.802	0.378	0.388	0.477
1890	0.808	0.392	0.398	0.488
1895	0.789	0.318	0.354	0.430
1900	0.776	0.260	0.320	0.383
1905	0.802	0.340	0.377	0.447
1910	0.821	0.359	0.411	0.461
1915	0.823	0.436	0.431	0.523
1920	0.806	0.377	0.392	0.477
1925	0.804	0.368	0.388	0.469
1930	0.821	0.385	0.417	0.482
1935	0.801	0.382	0.387	0.480

1940	0.789	0.338	0.360	0.445
1945	0.771	0.340	0.344	0.445
1950	0.816	0.419	0.417	0.509
1955	0.792	0.343	0.366	0.450
1960	0.797	0.377	0.381	0.475
1965	0.797	0.370	0.380	0.471
1970	0.789	0.305	0.349	0.419
1975	0.837	0.483	0.462	0.561
1980	0.792	0.337	0.364	0.444
1985	0.795	0.342	0.369	0.449
1990	0.809	0.377	0.397	0.476
1995	0.794	0.333	0.364	0.441
2000	0.801	0.350	0.378	0.455
2005	0.828	0.457	0.443	0.540
2010	0.816	0.419	0.417	0.509
2015	0.802	0.333	0.375	0.441
2020	0.799	0.301	0.360	0.414
2025	0.794	0.346	0.368	0.452
2030	0.827	0.436	0.437	0.524
2035	0.804	0.343	0.380	0.449
2040	0.811	0.386	0.402	0.483
2045	0.806	0.352	0.385	0.456
2050	0.832	0.445	0.447	0.531
2055	0.792	0.362	0.371	0.464
2060	0.815	0.379	0.405	0.478
2065	0.811	0.373	0.398	0.473
2070	0.815	0.391	0.408	0.488
2075	0.813	0.401	0.408	0.495
2080	0.809	0.358	0.392	0.461
2085	0.806	0.377	0.392	0.477
2090	0.789	0.325	0.356	0.435
2095	0.809	0.408	0.404	0.500
2100	0.813	0.382	0.403	0.481
2105	0.809	0.389	0.400	0.486
2110	0.789	0.332	0.358	0.440
2115	0.806	0.358	0.387	0.462
2120	0.837	0.431	0.455	0.520
2125	0.811	0.410	0.407	0.502
2130	0.801	0.331	0.372	0.439
2135	0.811	0.373	0.398	0.473
2140	0.802	0.384	0.390	0.482
2145	0.830	0.406	0.438	0.500
2150	0.813	0.388	0.405	0.486

2155	0.825	0.451	0.436	0.535
2160	0.818	0.415	0.419	0.507
2165	0.787	0.336	0.358	0.443
2170	0.806	0.332	0.379	0.440
2175	0.789	0.345	0.362	0.450
2180	0.834	0.442	0.450	0.529
2185	0.821	0.427	0.426	0.516
2190	0.802	0.340	0.377	0.447
2195	0.813	0.344	0.393	0.449
2200	0.837	0.419	0.454	0.510
2205	0.802	0.372	0.386	0.472
2210	0.801	0.394	0.390	0.489
2215	0.795	0.348	0.371	0.454
2220	0.804	0.368	0.388	0.469
2225	0.778	0.310	0.341	0.423
2230	0.802	0.293	0.361	0.406
2235	0.792	0.362	0.371	0.464
2240	0.832	0.463	0.450	0.545
2245	0.823	0.436	0.431	0.523
2250	0.764	0.271	0.314	0.393
2255	0.802	0.366	0.385	0.467
2260	0.806	0.396	0.397	0.491
2265	0.809	0.383	0.398	0.481
2270	0.790	0.334	0.361	0.442
2275	0.834	0.442	0.450	0.529
2280	0.790	0.341	0.363	0.447
2285	0.794	0.339	0.366	0.447
2290	0.841	0.455	0.466	0.540
2295	0.797	0.389	0.385	0.485
2300	0.799	0.341	0.373	0.448
2305	0.789	0.345	0.362	0.450
2310	0.801	0.375	0.385	0.475
2315	0.780	0.339	0.352	0.445
2320	0.795	0.361	0.375	0.464
2325	0.809	0.432	0.410	0.518
2330	0.809	0.345	0.388	0.450
2335	0.818	0.397	0.415	0.493
2340	0.815	0.360	0.400	0.462
2345	0.847	0.457	0.481	0.542
2350	0.795	0.302	0.356	0.416
2355	0.818	0.378	0.410	0.478
2360	0.809	0.351	0.390	0.455
2365	0.802	0.372	0.386	0.472

2370	0.821	0.403	0.421	0.498
2375	0.794	0.359	0.372	0.462
2380	0.795	0.348	0.371	0.454
2385	0.823	0.388	0.421	0.485
2390	0.809	0.351	0.390	0.455
2395	0.811	0.360	0.395	0.463
2400	0.832	0.403	0.440	0.497
2405	0.783	0.317	0.348	0.429
2410	0.794	0.326	0.362	0.436
2415	0.809	0.383	0.398	0.481
2420	0.827	0.442	0.438	0.528
2425	0.815	0.391	0.408	0.488
2430	0.808	0.335	0.383	0.442
2435	0.785	0.293	0.341	0.410
2440	0.792	0.337	0.364	0.444
2445	0.783	0.304	0.344	0.419
2450	0.795	0.316	0.361	0.427
2455	0.813	0.413	0.410	0.505
2460	0.794	0.333	0.364	0.441
2465	0.789	0.285	0.342	0.402
2470	0.782	0.354	0.358	0.457
2475	0.821	0.403	0.421	0.498
2480	0.797	0.370	0.380	0.471
2485	0.808	0.399	0.400	0.493
2490	0.768	0.289	0.324	0.407
2495	0.783	0.356	0.361	0.459
2500	0.799	0.366	0.381	0.468
2505	0.787	0.361	0.366	0.463
2510	0.773	0.296	0.331	0.413
2515	0.801	0.344	0.376	0.450
2520	0.790	0.366	0.371	0.467
2525	0.787	0.355	0.364	0.458
2530	0.794	0.371	0.376	0.471
2535	0.809	0.395	0.402	0.491
2540	0.789	0.332	0.358	0.440
2545	0.813	0.382	0.403	0.481
2550	0.820	0.424	0.423	0.514
2555	0.799	0.366	0.381	0.468
2560	0.783	0.344	0.357	0.449
2565	0.802	0.359	0.383	0.462
2570	0.813	0.437	0.415	0.522
2575	0.806	0.365	0.389	0.467
2580	0.797	0.357	0.376	0.461

2585	0.797	0.357	0.376	0.461
2590	0.835	0.445	0.454	0.532
2595	0.775	0.305	0.336	0.420
2600	0.830	0.412	0.439	0.505
2605	0.830	0.424	0.441	0.515
2610	0.811	0.386	0.402	0.483
2615	0.811	0.422	0.410	0.511
2620	0.795	0.348	0.371	0.454
2625	0.787	0.309	0.349	0.423
2630	0.801	0.363	0.382	0.465
2635	0.801	0.363	0.382	0.465
2640	0.782	0.335	0.352	0.442
2645	0.825	0.397	0.426	0.492
2650	0.789	0.285	0.342	0.402
2655	0.809	0.364	0.393	0.466
2660	0.776	0.287	0.331	0.406
2665	0.799	0.347	0.375	0.453
2670	0.789	0.312	0.352	0.425
2675	0.794	0.293	0.350	0.408
2680	0.795	0.296	0.353	0.410
2685	0.795	0.335	0.367	0.443
2690	0.801	0.356	0.380	0.460
2695	0.821	0.421	0.425	0.512
2700	0.806	0.377	0.392	0.477
2705	0.818	0.391	0.413	0.488
2710	0.821	0.409	0.423	0.502
2715	0.780	0.292	0.336	0.409
2720	0.795	0.335	0.367	0.443
2725	0.804	0.375	0.389	0.474
2730	0.808	0.392	0.398	0.488
2735	0.816	0.382	0.408	0.480
2740	0.813	0.395	0.406	0.491
2745	0.754	0.264	0.304	0.388
2750	0.806	0.365	0.389	0.467
2755	0.811	0.398	0.405	0.493
2760	0.790	0.321	0.357	0.432
2765	0.813	0.376	0.402	0.476
2770	0.785	0.333	0.355	0.441
2775	0.792	0.283	0.345	0.400
2780	0.809	0.389	0.400	0.486
2785	0.802	0.359	0.383	0.462
2790	0.823	0.418	0.427	0.510
2795	0.771	0.287	0.326	0.405

2800	0.780	0.279	0.331	0.398
2805	0.820	0.436	0.425	0.523
2810	0.813	0.363	0.398	0.465
2815	0.820	0.424	0.423	0.514
2820	0.821	0.372	0.414	0.472
2825	0.797	0.377	0.381	0.475
2830	0.821	0.397	0.420	0.493
2835	0.818	0.433	0.422	0.521
2840	0.813	0.388	0.405	0.486
2845	0.808	0.355	0.388	0.459
2850	0.811	0.386	0.402	0.483
2855	0.815	0.404	0.411	0.498
2860	0.797	0.345	0.372	0.451
2865	0.802	0.397	0.393	0.491
2870	0.834	0.436	0.449	0.525
2875	0.795	0.329	0.365	0.438
2880	0.792	0.297	0.350	0.412
2885	0.811	0.367	0.397	0.468
2890	0.801	0.356	0.380	0.460
2895	0.835	0.440	0.453	0.527
2900	0.830	0.454	0.445	0.538
2905	0.820	0.418	0.422	0.509
2910	0.789	0.312	0.352	0.425
2915	0.797	0.332	0.368	0.440
2920	0.820	0.375	0.412	0.475
2925	0.844	0.467	0.474	0.550
2930	0.809	0.389	0.400	0.486
2935	0.804	0.336	0.378	0.443
2940	0.823	0.412	0.426	0.505
2945	0.820	0.412	0.421	0.505
2950	0.790	0.354	0.367	0.457
2955	0.790	0.288	0.345	0.404
2960	0.802	0.346	0.379	0.452
2965	0.795	0.322	0.363	0.433
2970	0.801	0.344	0.376	0.450
2975	0.795	0.335	0.367	0.443
2980	0.808	0.386	0.397	0.484
2985	0.802	0.384	0.390	0.482
2990	0.783	0.344	0.357	0.449
2995	0.783	0.291	0.339	0.408
3000	0.801	0.350	0.378	0.455
3005	0.821	0.421	0.425	0.512
3010	0.818	0.385	0.412	0.483

3015	0.811	0.367	0.397	0.468
3020	0.804	0.387	0.393	0.484
3025	0.801	0.382	0.387	0.480
3030	0.804	0.349	0.382	0.454
3035	0.794	0.306	0.355	0.420
3040	0.802	0.391	0.391	0.486
3045	0.773	0.296	0.331	0.413
3050	0.815	0.360	0.400	0.462
3055	0.780	0.286	0.333	0.404
3060	0.804	0.343	0.380	0.449
3065	0.801	0.356	0.380	0.460
3070	0.813	0.401	0.408	0.495
3075	0.809	0.351	0.390	0.455
3080	0.797	0.338	0.370	0.445
3085	0.799	0.341	0.373	0.448
3090	0.802	0.320	0.371	0.430
3095	0.787	0.309	0.349	0.423
3100	0.804	0.381	0.391	0.479
3105	0.778	0.263	0.323	0.385
3110	0.771	0.307	0.333	0.421
3115	0.797	0.305	0.359	0.418
3120	0.780	0.332	0.350	0.441
3125	0.769	0.264	0.316	0.387
3130	0.794	0.339	0.366	0.447
3135	0.780	0.306	0.341	0.420
3140	0.804	0.323	0.374	0.432
3145	0.776	0.260	0.320	0.383
3150	0.816	0.406	0.414	0.500
3155	0.790	0.253	0.330	0.373
3160	0.769	0.250	0.310	0.376
3165	0.790	0.341	0.363	0.447
3170	0.795	0.342	0.369	0.449
3175	0.776	0.321	0.343	0.432
3180	0.787	0.336	0.358	0.443
3185	0.790	0.301	0.350	0.415
3190	0.763	0.275	0.315	0.396
3195	0.806	0.377	0.392	0.477
3200	0.821	0.378	0.416	0.477
3205	0.795	0.342	0.369	0.449
3210	0.787	0.268	0.333	0.388
3215	0.789	0.338	0.360	0.445
3220	0.792	0.270	0.339	0.388
3225	0.790	0.288	0.345	0.404

3230	0.804	0.330	0.376	0.438
3235	0.792	0.297	0.350	0.412
3240	0.795	0.342	0.369	0.449
3245	0.797	0.351	0.374	0.456
3250	0.782	0.315	0.346	0.427
3255	0.757	0.262	0.306	0.386
3260	0.802	0.340	0.377	0.447
3265	0.808	0.386	0.397	0.484
3270	0.795	0.348	0.371	0.454
3275	0.790	0.321	0.357	0.432
3280	0.811	0.348	0.391	0.452
3285	0.773	0.283	0.326	0.402
3290	0.821	0.385	0.417	0.482
3295	0.794	0.279	0.345	0.396
3300	0.823	0.430	0.430	0.519
3305	0.797	0.312	0.361	0.424
3310	0.790	0.294	0.347	0.410
3315	0.823	0.424	0.429	0.514
3320	0.785	0.333	0.355	0.441
3325	0.776	0.314	0.340	0.427
3330	0.799	0.301	0.360	0.414
3335	0.776	0.260	0.320	0.383
3340	0.792	0.317	0.357	0.429
3345	0.789	0.318	0.354	0.430
3350	0.827	0.418	0.433	0.510
3355	0.776	0.267	0.323	0.389
3360	0.750	0.232	0.289	0.363
3365	0.792	0.290	0.347	0.406
3370	0.787	0.296	0.344	0.411
3375	0.801	0.337	0.374	0.444
3380	0.783	0.270	0.331	0.390
3385	0.783	0.311	0.346	0.424
3390	0.782	0.275	0.331	0.394
3395	0.776	0.294	0.333	0.411
3400	0.782	0.268	0.328	0.388
3405	0.790	0.301	0.350	0.415
3410	0.780	0.332	0.350	0.441
3415	0.764	0.236	0.300	0.364
3420	0.752	0.213	0.281	0.347
3425	0.797	0.351	0.374	0.456
3430	0.792	0.310	0.355	0.423
3435	0.815	0.341	0.394	0.446
3440	0.776	0.253	0.317	0.377

3445	0.809	0.318	0.380	0.427
3450	0.797	0.292	0.354	0.406
3455	0.757	0.255	0.303	0.381
3460	0.785	0.259	0.328	0.380
3465	0.789	0.318	0.354	0.430
3470	0.821	0.409	0.423	0.502
3475	0.811	0.373	0.398	0.473
3480	0.759	0.229	0.293	0.359
3485	0.813	0.351	0.395	0.455
3490	0.787	0.329	0.356	0.438
3495	0.806	0.345	0.383	0.451
3500	0.773	0.303	0.333	0.418
3505	0.790	0.328	0.359	0.437
3510	0.789	0.325	0.356	0.435
3515	0.766	0.280	0.319	0.400
3520	0.823	0.375	0.418	0.474
3525	0.806	0.352	0.385	0.456
3530	0.842	0.475	0.471	0.556
3535	0.747	0.248	0.293	0.376
3540	0.795	0.309	0.358	0.422
3545	0.787	0.329	0.356	0.438
3550	0.771	0.246	0.310	0.371
3555	0.771	0.267	0.318	0.389
3560	0.785	0.286	0.339	0.404
3565	0.799	0.392	0.387	0.487
3570	0.799	0.301	0.360	0.414
3575	0.799	0.301	0.360	0.414
3580	0.795	0.355	0.373	0.459
3585	0.761	0.238	0.299	0.367
3590	0.785	0.320	0.351	0.431
3595	0.764	0.284	0.319	0.404
3600	0.776	0.294	0.333	0.411
3605	0.763	0.268	0.312	0.391
3610	0.768	0.248	0.308	0.374
3615	0.790	0.260	0.333	0.379
3620	0.771	0.246	0.310	0.371
3625	0.806	0.365	0.389	0.467
3630	0.769	0.236	0.304	0.364
3635	0.766	0.287	0.322	0.405
3640	0.802	0.327	0.373	0.436
3645	0.785	0.352	0.361	0.456
3650	0.783	0.256	0.325	0.378
3655	0.785	0.280	0.336	0.398

3660	0.778	0.297	0.336	0.413
3665	0.785	0.293	0.341	0.410
3670	0.785	0.320	0.351	0.431
3675	0.835	0.463	0.456	0.545
3680	0.787	0.355	0.364	0.458
3685	0.792	0.330	0.362	0.439
3690	0.801	0.394	0.390	0.489
3695	0.769	0.311	0.333	0.424
3700	0.816	0.388	0.410	0.485
3705	0.766	0.273	0.317	0.395
3710	0.787	0.329	0.356	0.438
3715	0.778	0.263	0.323	0.385
3720	0.799	0.294	0.357	0.408
3725	0.801	0.344	0.376	0.450
3730	0.785	0.280	0.336	0.398
3735	0.808	0.348	0.387	0.453
3740	0.787	0.342	0.360	0.448
3745	0.828	0.391	0.431	0.487
3750	0.801	0.324	0.370	0.433
3755	0.802	0.320	0.371	0.430
3760	0.787	0.316	0.351	0.428
3765	0.790	0.328	0.359	0.437
3770	0.757	0.248	0.300	0.375
3775	0.792	0.324	0.359	0.434
3780	0.790	0.366	0.371	0.467
3785	0.794	0.346	0.368	0.452
3790	0.801	0.331	0.372	0.439
3795	0.771	0.253	0.313	0.377
3800	0.771	0.195	0.286	0.327
3805	0.778	0.269	0.325	0.390
3810	0.775	0.244	0.311	0.369
3815	0.764	0.222	0.294	0.352
3820	0.773	0.262	0.318	0.385
3825	0.787	0.289	0.341	0.406
3830	0.773	0.309	0.336	0.423
3835	0.780	0.279	0.331	0.398
3840	0.809	0.364	0.393	0.466
3845	0.783	0.297	0.341	0.413
3850	0.802	0.340	0.377	0.447
3855	0.778	0.310	0.341	0.423
3860	0.816	0.376	0.407	0.475
3865	0.802	0.327	0.373	0.436
3870	0.785	0.300	0.344	0.415

3875	0.776	0.327	0.345	0.437
3880	0.799	0.410	0.392	0.500
3885	0.837	0.413	0.453	0.505
3890	0.749	0.244	0.293	0.372
3895	0.808	0.342	0.385	0.448
3900	0.776	0.294	0.333	0.411
3905	0.783	0.311	0.346	0.424
3910	0.747	0.228	0.285	0.360
3915	0.813	0.376	0.402	0.476
3920	0.806	0.352	0.385	0.456
3925	0.802	0.353	0.381	0.457
3930	0.792	0.324	0.359	0.434
3935	0.771	0.232	0.303	0.359
3940	0.792	0.263	0.336	0.381
3945	0.780	0.292	0.336	0.409
3950	0.789	0.312	0.352	0.425
3955	0.837	0.460	0.459	0.544
3960	0.795	0.361	0.375	0.464
3965	0.787	0.309	0.349	0.423
3970	0.780	0.258	0.322	0.380
3975	0.773	0.234	0.306	0.361
3980	0.778	0.303	0.338	0.418
3985	0.780	0.312	0.343	0.425
3990	0.778	0.297	0.336	0.413
3995	0.813	0.376	0.402	0.476
4000	0.785	0.293	0.341	0.410
4005	0.821	0.415	0.424	0.507
4010	0.790	0.308	0.352	0.421
4015	0.778	0.227	0.307	0.354
4020	0.820	0.375	0.412	0.475
4025	0.776	0.274	0.326	0.394
4030	0.795	0.316	0.361	0.427
4035	0.769	0.278	0.321	0.398
4040	0.811	0.379	0.400	0.478
4045	0.773	0.248	0.312	0.373
4050	0.801	0.337	0.374	0.444
4055	0.808	0.374	0.394	0.474
4060	0.790	0.294	0.347	0.410
4065	0.809	0.332	0.384	0.439
4070	0.769	0.243	0.307	0.370
4075	0.778	0.310	0.341	0.423
4080	0.813	0.351	0.395	0.455
4085	0.806	0.396	0.397	0.491

4090	0.771	0.280	0.324	0.400
4095	0.799	0.347	0.375	0.453
4100	0.799	0.308	0.362	0.420
4105	0.797	0.357	0.376	0.461
4110	0.816	0.419	0.417	0.509
4115	0.775	0.285	0.328	0.404
4120	0.775	0.312	0.338	0.425
4125	0.799	0.379	0.384	0.477
4130	0.804	0.349	0.382	0.454
4135	0.735	0.109	0.220	0.261
4140	0.756	0.239	0.295	0.368
4145	0.773	0.269	0.321	0.391
4150	0.764	0.236	0.300	0.364
4155	0.794	0.306	0.355	0.420
4160	0.775	0.298	0.333	0.414
4165	0.818	0.433	0.422	0.521
4170	0.804	0.303	0.367	0.415
4175	0.773	0.241	0.309	0.367
4180	0.797	0.318	0.364	0.429
4185	0.789	0.285	0.342	0.402
4190	0.775	0.298	0.333	0.414
4195	0.789	0.305	0.349	0.419
4200	0.771	0.253	0.313	0.377
4205	0.778	0.297	0.336	0.413
4210	0.821	0.334	0.404	0.437
4215	0.759	0.257	0.305	0.382
4220	0.790	0.274	0.339	0.392
4225	0.752	0.220	0.285	0.353
4230	0.801	0.337	0.374	0.444
4235	0.799	0.315	0.364	0.426
4240	0.775	0.325	0.342	0.435
4245	0.785	0.286	0.339	0.404
4250	0.804	0.362	0.386	0.464
4255	0.787	0.322	0.353	0.433
4260	0.801	0.304	0.363	0.416
4265	0.756	0.259	0.303	0.384
4270	0.769	0.298	0.329	0.414
4275	0.790	0.308	0.352	0.421
4280	0.804	0.356	0.384	0.459
4285	0.809	0.395	0.402	0.491
4290	0.764	0.250	0.306	0.376
4295	0.801	0.375	0.385	0.475
4300	0.778	0.297	0.336	0.413

4305	0.750	0.239	0.292	0.368
4310	0.754	0.236	0.293	0.366
4315	0.801	0.375	0.385	0.475
4320	0.801	0.317	0.368	0.428
4325	0.780	0.286	0.333	0.404
4330	0.771	0.273	0.321	0.394
4335	0.789	0.285	0.342	0.402
4340	0.792	0.310	0.355	0.423
4345	0.809	0.358	0.392	0.461
4350	0.766	0.300	0.327	0.416
4355	0.783	0.311	0.346	0.424
4360	0.780	0.265	0.325	0.386
4365	0.809	0.364	0.393	0.466
4370	0.775	0.251	0.315	0.375
4375	0.780	0.299	0.338	0.415
4380	0.766	0.217	0.293	0.348
4385	0.775	0.298	0.333	0.414
4390	0.776	0.281	0.328	0.400
4395	0.763	0.234	0.298	0.363
4400	0.804	0.323	0.374	0.432
4405	0.773	0.227	0.303	0.355
4410	0.792	0.310	0.355	0.423
4415	0.768	0.289	0.324	0.407
4420	0.782	0.288	0.336	0.406
4425	0.801	0.382	0.387	0.480
4430	0.771	0.253	0.313	0.377
4435	0.766	0.273	0.317	0.395
4440	0.771	0.273	0.321	0.394
4445	0.804	0.356	0.384	0.459
4450	0.816	0.406	0.414	0.500
4455	0.785	0.273	0.333	0.392
4460	0.787	0.342	0.360	0.448
4465	0.821	0.372	0.414	0.472
4470	0.795	0.302	0.356	0.416
4475	0.776	0.253	0.317	0.377
4480	0.764	0.243	0.303	0.370
4485	0.802	0.353	0.381	0.457
4490	0.785	0.259	0.328	0.380
4495	0.782	0.295	0.338	0.411
4500	0.759	0.215	0.287	0.347
4505	0.747	0.234	0.288	0.365
4510	0.802	0.314	0.368	0.424
4515	0.735	0.206	0.268	0.343

4520	0.816	0.376	0.407	0.475
4525	0.775	0.265	0.320	0.387
4530	0.782	0.275	0.331	0.394
4535	0.711	0.149	0.232	0.301
4540	0.735	0.191	0.262	0.332
4545	0.776	0.267	0.323	0.389
4550	0.804	0.349	0.382	0.454
4555	0.769	0.250	0.310	0.376
4560	0.776	0.321	0.343	0.432
4565	0.794	0.326	0.362	0.436
4570	0.778	0.303	0.338	0.418
4575	0.754	0.222	0.287	0.355
4580	0.825	0.378	0.422	0.477
4585	0.764	0.284	0.319	0.404
4590	0.783	0.284	0.336	0.402
4595	0.773	0.255	0.315	0.379
4600	0.804	0.310	0.369	0.421
4605	0.809	0.395	0.402	0.491
4610	0.771	0.320	0.338	0.431
4615	0.754	0.222	0.287	0.355
4620	0.778	0.283	0.331	0.402
4625	0.750	0.174	0.262	0.314
4630	0.813	0.388	0.405	0.486
4635	0.799	0.334	0.371	0.442
4640	0.764	0.284	0.319	0.404
4645	0.766	0.273	0.317	0.395
4650	0.813	0.357	0.397	0.460
4655	0.830	0.460	0.446	0.542
4660	0.808	0.348	0.387	0.453
4665	0.782	0.302	0.341	0.417
4670	0.766	0.280	0.319	0.400
4675	0.794	0.352	0.370	0.457
4680	0.757	0.255	0.303	0.381
4685	0.809	0.401	0.403	0.495
4690	0.806	0.390	0.396	0.486
4695	0.783	0.277	0.333	0.396
4700	0.782	0.328	0.350	0.438
4705	0.806	0.352	0.385	0.456
4710	0.823	0.394	0.422	0.490
4715	0.787	0.316	0.351	0.428
4720	0.816	0.363	0.404	0.465
4725	0.792	0.330	0.362	0.439
4730	0.815	0.404	0.411	0.498

4735	0.802	0.327	0.373	0.436
4740	0.815	0.347	0.396	0.451
4745	0.790	0.294	0.347	0.410
4750	0.809	0.401	0.403	0.495
4755	0.785	0.273	0.333	0.392
4760	0.792	0.330	0.362	0.439
4765	0.783	0.291	0.339	0.408
4770	0.797	0.345	0.372	0.451
4775	0.764	0.207	0.287	0.340
4780	0.790	0.360	0.369	0.462
4785	0.756	0.217	0.286	0.350
4790	0.768	0.241	0.305	0.368
4795	0.763	0.282	0.317	0.402
4800	0.792	0.263	0.336	0.381
4805	0.783	0.304	0.344	0.419
4810	0.782	0.268	0.328	0.388
4815	0.795	0.296	0.353	0.410
4820	0.783	0.270	0.331	0.390
4825	0.787	0.303	0.346	0.417
4830	0.775	0.338	0.347	0.444
4835	0.764	0.264	0.312	0.387
4840	0.792	0.324	0.359	0.434
4845	0.795	0.329	0.365	0.438
4850	0.799	0.341	0.373	0.448
4855	0.782	0.268	0.328	0.388
4860	0.782	0.302	0.341	0.417
4865	0.761	0.181	0.271	0.317
4870	0.790	0.334	0.361	0.442
4875	0.780	0.292	0.336	0.409
4880	0.780	0.230	0.310	0.355
4885	0.769	0.291	0.326	0.409
4890	0.773	0.241	0.309	0.367
4895	0.804	0.362	0.386	0.464
4900	0.795	0.316	0.361	0.427
4905	0.778	0.276	0.328	0.396
4910	0.787	0.296	0.344	0.411
4915	0.775	0.285	0.328	0.404
4920	0.757	0.227	0.291	0.358
4925	0.744	0.195	0.268	0.333
4930	0.750	0.239	0.292	0.368
4935	0.769	0.236	0.304	0.364
4940	0.768	0.255	0.311	0.380
4945	0.794	0.313	0.358	0.425

4950	0.773	0.283	0.326	0.402
4955	0.789	0.298	0.347	0.413
4960	0.780	0.332	0.350	0.441
4965	0.821	0.366	0.413	0.466
4970	0.761	0.217	0.289	0.349
4975	0.763	0.205	0.285	0.338
4980	0.778	0.330	0.347	0.439
4985	0.790	0.294	0.347	0.410
4990	0.775	0.298	0.333	0.414
4995	0.802	0.346	0.379	0.452
5000	0.808	0.367	0.392	0.469
5005	0.794	0.371	0.376	0.471
5010	0.785	0.327	0.353	0.436
5015	0.759	0.222	0.290	0.353
5020	0.773	0.336	0.344	0.443
5025	0.764	0.257	0.309	0.382
5030	0.789	0.305	0.349	0.419
5035	0.750	0.174	0.262	0.314
5040	0.771	0.287	0.326	0.405
5045	0.780	0.251	0.319	0.374
5050	0.744	0.216	0.278	0.351
5055	0.747	0.199	0.272	0.336
5060	0.785	0.293	0.341	0.410
5065	0.768	0.269	0.316	0.391
5070	0.773	0.255	0.315	0.379
5075	0.775	0.251	0.315	0.375
5080	0.787	0.268	0.333	0.388
5085	0.752	0.199	0.275	0.335
5090	0.766	0.217	0.293	0.348
5095	0.744	0.209	0.275	0.345
5100	0.785	0.320	0.351	0.431
5105	0.761	0.231	0.295	0.361
5110	0.773	0.248	0.312	0.373
5115	0.771	0.232	0.303	0.359
5120	0.785	0.252	0.325	0.374
5125	0.799	0.334	0.371	0.442
5130	0.775	0.285	0.328	0.404
5135	0.764	0.200	0.283	0.333
5140	0.783	0.311	0.346	0.424
5145	0.769	0.250	0.310	0.376
5150	0.802	0.378	0.388	0.477
5155	0.782	0.295	0.338	0.411
5160	0.808	0.348	0.387	0.453

5165	0.789	0.318	0.354	0.430
5170	0.785	0.352	0.361	0.456
5175	0.776	0.260	0.320	0.383
5180	0.809	0.389	0.400	0.486
5185	0.785	0.313	0.348	0.426
5190	0.825	0.421	0.431	0.512
5195	0.775	0.222	0.302	0.350
5200	0.780	0.251	0.319	0.374
5205	0.750	0.273	0.305	0.395
5210	0.744	0.237	0.287	0.368
5215	0.775	0.298	0.333	0.414
5220	0.771	0.273	0.321	0.394
5225	0.790	0.301	0.350	0.415
5230	0.773	0.296	0.331	0.413
5235	0.794	0.346	0.368	0.452
5240	0.742	0.249	0.290	0.377
5245	0.778	0.323	0.345	0.434
5250	0.792	0.381	0.377	0.478
5255	0.797	0.338	0.370	0.445
5260	0.773	0.316	0.338	0.428
5265	0.792	0.317	0.357	0.429
5270	0.797	0.271	0.346	0.387
5275	0.787	0.322	0.353	0.433
5280	0.778	0.276	0.328	0.396
5285	0.794	0.352	0.370	0.457
5290	0.731	0.187	0.259	0.329
5295	0.778	0.297	0.336	0.413
5300	0.801	0.356	0.380	0.460
5305	0.785	0.245	0.321	0.367
5310	0.808	0.348	0.387	0.453
5315	0.792	0.324	0.359	0.434
5320	0.780	0.306	0.341	0.420
5325	0.787	0.309	0.349	0.423
5330	0.787	0.322	0.353	0.433
5335	0.782	0.328	0.350	0.438
5340	0.804	0.375	0.389	0.474
5345	0.754	0.243	0.296	0.372
5350	0.759	0.243	0.299	0.371
5355	0.795	0.316	0.361	0.427
5360	0.794	0.352	0.370	0.457
5365	0.839	0.446	0.461	0.533
5370	0.766	0.266	0.314	0.389
5375	0.801	0.331	0.372	0.439

5380	0.816	0.382	0.408	0.480
5385	0.794	0.313	0.358	0.425
5390	0.783	0.291	0.339	0.408
5395	0.799	0.379	0.384	0.477
5400	0.789	0.351	0.364	0.455
5405	0.795	0.322	0.363	0.433
5410	0.782	0.275	0.331	0.394
5415	0.785	0.286	0.339	0.404
5420	0.799	0.328	0.369	0.437
5425	0.801	0.356	0.380	0.460
5430	0.787	0.303	0.346	0.417
5435	0.764	0.250	0.306	0.376
5440	0.806	0.358	0.387	0.462
5445	0.750	0.246	0.295	0.374
5450	0.785	0.286	0.339	0.404
5455	0.783	0.297	0.341	0.413
5460	0.799	0.334	0.371	0.442
5465	0.806	0.352	0.385	0.456
5470	0.775	0.305	0.336	0.420
5475	0.757	0.255	0.303	0.381
5480	0.804	0.343	0.380	0.449
5485	0.764	0.193	0.280	0.327
5490	0.799	0.347	0.375	0.453
5495	0.813	0.388	0.405	0.486
5500	0.766	0.266	0.314	0.389
5505	0.785	0.273	0.333	0.392
5510	0.794	0.326	0.362	0.436
5515	0.745	0.190	0.267	0.329
5520	0.775	0.271	0.323	0.393
5525	0.815	0.397	0.409	0.493
5530	0.771	0.246	0.310	0.371
5535	0.780	0.272	0.328	0.392
5540	0.782	0.341	0.354	0.447
5545	0.785	0.359	0.363	0.461
5550	0.783	0.344	0.357	0.449
5555	0.763	0.227	0.295	0.357
5560	0.763	0.227	0.295	0.357
5565	0.783	0.331	0.353	0.439
5570	0.759	0.222	0.290	0.353
5575	0.830	0.424	0.441	0.515
5580	0.783	0.337	0.355	0.444
5585	0.759	0.171	0.265	0.308
5590	0.823	0.418	0.427	0.510

5595	0.731	0.208	0.268	0.346
5600	0.795	0.302	0.356	0.416
5605	0.773	0.269	0.321	0.391
5610	0.795	0.368	0.377	0.468
5615	0.808	0.329	0.381	0.437
5620	0.804	0.343	0.380	0.449
5625	0.778	0.256	0.320	0.379
5630	0.801	0.369	0.383	0.470
5635	0.787	0.309	0.349	0.423
5640	0.782	0.302	0.341	0.417
5645	0.811	0.373	0.398	0.473
5650	0.782	0.288	0.336	0.406
5655	0.815	0.366	0.402	0.468
5660	0.825	0.427	0.432	0.517
5665	0.776	0.327	0.345	0.437
5670	0.792	0.330	0.362	0.439
5675	0.797	0.338	0.370	0.445
5680	0.799	0.341	0.373	0.448
5685	0.778	0.297	0.336	0.413
5690	0.778	0.297	0.336	0.413
5695	0.787	0.336	0.358	0.443
5700	0.776	0.274	0.326	0.394
5705	0.801	0.363	0.382	0.465
5710	0.795	0.355	0.373	0.459
5715	0.754	0.171	0.262	0.311
5720	0.787	0.275	0.336	0.394
5725	0.738	0.144	0.240	0.291
5730	0.802	0.372	0.386	0.472
5735	0.761	0.245	0.301	0.373
5740	0.759	0.229	0.293	0.359
5745	0.804	0.310	0.369	0.421
5750	0.802	0.340	0.377	0.447
5755	0.820	0.394	0.417	0.490
5760	0.795	0.335	0.367	0.443
5765	0.789	0.236	0.321	0.358
5770	0.790	0.301	0.350	0.415
5775	0.766	0.293	0.324	0.410
5780	0.806	0.365	0.389	0.467
5785	0.797	0.357	0.376	0.461
5790	0.792	0.297	0.350	0.412
5795	0.750	0.204	0.276	0.339
5800	0.768	0.269	0.316	0.391
5805	0.747	0.199	0.272	0.336

5810	0.778	0.317	0.343	0.429
5815	0.768	0.269	0.316	0.391
5820	0.761	0.210	0.286	0.343
5825	0.789	0.318	0.354	0.430
5830	0.771	0.246	0.310	0.371
5835	0.775	0.258	0.317	0.381
5840	0.790	0.334	0.361	0.442
5845	0.773	0.269	0.321	0.391
5850	0.768	0.296	0.326	0.412
5855	0.801	0.304	0.363	0.416
5860	0.789	0.338	0.360	0.445
5865	0.789	0.264	0.333	0.384
5870	0.750	0.232	0.289	0.363
5875	0.792	0.290	0.347	0.406
5880	0.790	0.288	0.345	0.404
5885	0.787	0.296	0.344	0.411
5890	0.766	0.266	0.314	0.389
5895	0.757	0.262	0.306	0.386
5900	0.806	0.365	0.389	0.467
5905	0.789	0.305	0.349	0.419
5910	0.771	0.307	0.333	0.421
5915	0.780	0.230	0.310	0.355
5920	0.808	0.386	0.397	0.484
5925	0.766	0.246	0.305	0.372
5930	0.773	0.255	0.315	0.379
5935	0.808	0.361	0.390	0.464
5940	0.792	0.310	0.355	0.423
5945	0.804	0.330	0.376	0.438
5950	0.780	0.306	0.341	0.420
5955	0.809	0.358	0.392	0.461
5960	0.827	0.448	0.438	0.533
5965	0.771	0.267	0.318	0.389
5970	0.790	0.347	0.365	0.452
5975	0.783	0.324	0.350	0.434
5980	0.776	0.274	0.326	0.394
5985	0.773	0.309	0.336	0.423
5990	0.787	0.296	0.344	0.411
5995	0.785	0.352	0.361	0.456
6000	0.789	0.332	0.358	0.440
6005	0.787	0.296	0.344	0.411
6010	0.775	0.338	0.347	0.444
6015	0.802	0.320	0.371	0.430
6020	0.802	0.359	0.383	0.462

6025	0.799	0.328	0.369	0.437
6030	0.792	0.324	0.359	0.434
6035	0.752	0.241	0.294	0.370
6040	0.771	0.253	0.313	0.377
6045	0.776	0.267	0.323	0.389
6050	0.754	0.257	0.301	0.383
6055	0.782	0.302	0.341	0.417
6060	0.754	0.250	0.299	0.377
6065	0.823	0.424	0.429	0.514
6070	0.771	0.294	0.329	0.411
6075	0.811	0.354	0.393	0.458
6080	0.811	0.386	0.402	0.483
6085	0.794	0.333	0.364	0.441
6090	0.782	0.335	0.352	0.442
6095	0.782	0.281	0.333	0.400
6100	0.809	0.364	0.393	0.466
6105	0.802	0.346	0.379	0.452
6110	0.763	0.234	0.298	0.363
6115	0.776	0.301	0.336	0.416
6120	0.776	0.327	0.345	0.437
6125	0.785	0.286	0.339	0.404
6130	0.794	0.300	0.353	0.414
6135	0.749	0.216	0.281	0.350
6140	0.782	0.261	0.325	0.382
6145	0.804	0.336	0.378	0.443
6150	0.780	0.272	0.328	0.392
6155	0.802	0.372	0.386	0.472
6160	0.771	0.232	0.303	0.359
6165	0.821	0.372	0.414	0.472
6170	0.785	0.293	0.341	0.410
6175	0.811	0.367	0.397	0.468
6180	0.820	0.388	0.415	0.485
6185	0.795	0.355	0.373	0.459
6190	0.801	0.324	0.370	0.433
6195	0.799	0.315	0.364	0.426
6200	0.790	0.301	0.350	0.415
6205	0.787	0.322	0.353	0.433
6210	0.806	0.306	0.370	0.417
6215	0.821	0.397	0.420	0.493
6220	0.797	0.318	0.364	0.429
6225	0.761	0.245	0.301	0.373
6230	0.797	0.338	0.370	0.445
6235	0.756	0.151	0.252	0.291

6240	0.778	0.290	0.333	0.407
6245	0.790	0.301	0.350	0.415
6250	0.785	0.313	0.348	0.426
6255	0.764	0.278	0.317	0.398
6260	0.775	0.298	0.333	0.414
6265	0.790	0.360	0.369	0.462
6270	0.773	0.262	0.318	0.385
6275	0.773	0.289	0.328	0.407
6280	0.789	0.312	0.352	0.425
6285	0.802	0.372	0.386	0.472
6290	0.735	0.206	0.268	0.343
6295	0.766	0.246	0.305	0.372
6300	0.782	0.328	0.350	0.438
6305	0.752	0.206	0.278	0.341
6310	0.799	0.360	0.379	0.463
6315	0.775	0.265	0.320	0.387
6320	0.766	0.252	0.308	0.378
6325	0.780	0.279	0.331	0.398
6330	0.802	0.346	0.379	0.452
6335	0.782	0.322	0.348	0.432
6340	0.797	0.318	0.364	0.429
6345	0.754	0.243	0.296	0.372
6350	0.782	0.308	0.343	0.422
6355	0.804	0.330	0.376	0.438
6360	0.816	0.406	0.414	0.500
6365	0.778	0.317	0.343	0.429
6370	0.768	0.241	0.305	0.368
6375	0.792	0.283	0.345	0.400
6380	0.780	0.279	0.331	0.398
6385	0.790	0.267	0.336	0.386
6390	0.787	0.303	0.346	0.417
6395	0.769	0.271	0.319	0.393
6400	0.808	0.386	0.397	0.484
6405	0.801	0.317	0.368	0.428
6410	0.780	0.299	0.338	0.415
6415	0.757	0.255	0.303	0.381
6420	0.776	0.314	0.340	0.427
6425	0.782	0.288	0.336	0.406
6430	0.795	0.329	0.365	0.438
6435	0.794	0.352	0.370	0.457
6440	0.764	0.298	0.324	0.414
6445	0.834	0.431	0.448	0.520
6450	0.780	0.312	0.343	0.425

6455	0.818	0.391	0.413	0.488
6460	0.801	0.337	0.374	0.444
6465	0.783	0.324	0.350	0.434
6470	0.750	0.225	0.286	0.357
6475	0.754	0.250	0.299	0.377
6480	0.778	0.317	0.343	0.429
6485	0.792	0.356	0.370	0.459
6490	0.804	0.336	0.378	0.443
6495	0.766	0.259	0.311	0.384
6500	0.744	0.237	0.287	0.368
6505	0.780	0.279	0.331	0.398
6510	0.790	0.308	0.352	0.421
6515	0.785	0.286	0.339	0.404
6520	0.790	0.294	0.347	0.410
6525	0.783	0.311	0.346	0.424
6530	0.792	0.324	0.359	0.434
6535	0.752	0.199	0.275	0.335
6540	0.794	0.320	0.360	0.431
6545	0.802	0.320	0.371	0.430
6550	0.744	0.237	0.287	0.368
6555	0.775	0.305	0.336	0.420
6560	0.795	0.335	0.367	0.443
6565	0.795	0.342	0.369	0.449
6570	0.827	0.394	0.429	0.490
6575	0.801	0.356	0.380	0.460
6580	0.804	0.336	0.378	0.443
6585	0.795	0.374	0.379	0.473
6590	0.763	0.241	0.301	0.369
6595	0.769	0.243	0.307	0.370
6600	0.776	0.308	0.338	0.422
6605	0.811	0.328	0.385	0.435
6610	0.794	0.359	0.372	0.462
6615	0.801	0.375	0.385	0.475
6620	0.749	0.250	0.295	0.378
6625	0.787	0.322	0.353	0.433
6630	0.780	0.292	0.336	0.409
6635	0.778	0.283	0.331	0.402
6640	0.790	0.314	0.354	0.427
6645	0.768	0.302	0.329	0.417
6650	0.775	0.271	0.323	0.393
6655	0.763	0.295	0.322	0.412
6660	0.783	0.324	0.350	0.434
6665	0.806	0.339	0.381	0.446

6670	0.756	0.232	0.292	0.362
6675	0.782	0.302	0.341	0.417
6680	0.790	0.288	0.345	0.404
6685	0.759	0.222	0.290	0.353
6690	0.787	0.322	0.353	0.433
6695	0.750	0.239	0.292	0.368
6700	0.790	0.321	0.357	0.432
6705	0.773	0.296	0.331	0.413
6710	0.789	0.305	0.349	0.419
6715	0.752	0.289	0.312	0.407
6720	0.783	0.297	0.341	0.413
6725	0.749	0.230	0.287	0.361
6730	0.776	0.308	0.338	0.422
6735	0.790	0.321	0.357	0.432
6740	0.768	0.269	0.316	0.391
6745	0.795	0.309	0.358	0.422
6750	0.773	0.262	0.318	0.385
6755	0.771	0.287	0.326	0.405
6760	0.769	0.271	0.319	0.393
6765	0.763	0.262	0.309	0.386
6770	0.804	0.330	0.376	0.438
6775	0.787	0.316	0.351	0.428
6780	0.771	0.300	0.331	0.416
6785	0.792	0.324	0.359	0.434
6790	0.802	0.359	0.383	0.462
6795	0.790	0.341	0.363	0.447
6800	0.750	0.266	0.303	0.390
6805	0.782	0.322	0.348	0.432
6810	0.766	0.293	0.324	0.410
6815	0.787	0.361	0.366	0.463
6820	0.763	0.248	0.304	0.374
6825	0.806	0.377	0.392	0.477
6830	0.795	0.309	0.358	0.422
6835	0.801	0.350	0.378	0.455
6840	0.780	0.286	0.333	0.404
6845	0.764	0.236	0.300	0.364
6850	0.790	0.366	0.371	0.467
6855	0.780	0.299	0.338	0.415
6860	0.815	0.410	0.412	0.502
6865	0.782	0.335	0.352	0.442
6870	0.775	0.244	0.311	0.369
6875	0.780	0.326	0.348	0.436
6880	0.780	0.312	0.343	0.425

6885	0.785	0.320	0.351	0.431
6890	0.776	0.321	0.343	0.432
6895	0.789	0.278	0.339	0.396
6900	0.785	0.346	0.359	0.451
6905	0.808	0.374	0.394	0.474
6910	0.787	0.329	0.356	0.438
6915	0.768	0.234	0.302	0.362
6920	0.761	0.286	0.318	0.405
6925	0.783	0.317	0.348	0.429
6930	0.792	0.388	0.378	0.483
6935	0.783	0.317	0.348	0.429
6940	0.792	0.330	0.362	0.439
6945	0.790	0.347	0.365	0.452
6950	0.771	0.260	0.315	0.383
6955	0.789	0.298	0.347	0.413
6960	0.769	0.250	0.310	0.376
6965	0.795	0.355	0.373	0.459
6970	0.776	0.287	0.331	0.406
6975	0.776	0.260	0.320	0.383
6980	0.778	0.234	0.310	0.360
6985	0.783	0.311	0.346	0.424
6990	0.782	0.308	0.343	0.422
6995	0.794	0.320	0.360	0.431
7000	0.783	0.291	0.339	0.408
7005	0.790	0.328	0.359	0.437
7010	0.764	0.264	0.312	0.387
7015	0.785	0.327	0.353	0.436
7020	0.799	0.385	0.386	0.482
7025	0.776	0.340	0.349	0.446
7030	0.795	0.289	0.351	0.404
7035	0.775	0.244	0.311	0.369
7040	0.764	0.264	0.312	0.387
7045	0.780	0.306	0.341	0.420
7050	0.816	0.388	0.410	0.485
7055	0.795	0.348	0.371	0.454
7060	0.776	0.287	0.331	0.406
7065	0.757	0.248	0.300	0.375
7070	0.757	0.248	0.300	0.375
7075	0.797	0.351	0.374	0.456
7080	0.802	0.403	0.394	0.496
7085	0.783	0.291	0.339	0.408
7090	0.763	0.262	0.309	0.386
7095	0.783	0.324	0.350	0.434

7100	0.783	0.324	0.350	0.434
7105	0.795	0.329	0.365	0.438
7110	0.764	0.222	0.294	0.352
7115	0.754	0.250	0.299	0.377
7120	0.775	0.305	0.336	0.420
7125	0.792	0.276	0.342	0.394
7130	0.827	0.388	0.427	0.485
7135	0.795	0.335	0.367	0.443
7140	0.759	0.222	0.290	0.353
7145	0.802	0.353	0.381	0.457
7150	0.778	0.297	0.336	0.413
7155	0.775	0.305	0.336	0.420
7160	0.756	0.252	0.301	0.379
7165	0.780	0.332	0.350	0.441
7170	0.778	0.256	0.320	0.379
7175	0.797	0.364	0.378	0.466
7180	0.776	0.239	0.311	0.365
7185	0.794	0.339	0.366	0.447
7190	0.761	0.252	0.304	0.378
7195	0.744	0.180	0.261	0.321
7200	0.771	0.273	0.321	0.394
7205	0.764	0.318	0.331	0.429
7210	0.769	0.278	0.321	0.398
7215	0.783	0.356	0.361	0.459
7220	0.795	0.302	0.356	0.416
7225	0.787	0.322	0.353	0.433
7230	0.763	0.289	0.320	0.407
7235	0.808	0.355	0.388	0.459
7240	0.795	0.316	0.361	0.427
7245	0.809	0.370	0.395	0.471
7250	0.763	0.282	0.317	0.402
7255	0.801	0.290	0.358	0.404
7260	0.801	0.304	0.363	0.416
7265	0.785	0.352	0.361	0.456
7270	0.757	0.255	0.303	0.381
7275	0.764	0.243	0.303	0.370
7280	0.787	0.329	0.356	0.438
7285	0.790	0.321	0.357	0.432
7290	0.790	0.321	0.357	0.432
7295	0.778	0.336	0.349	0.443
7300	0.778	0.297	0.336	0.413
7305	0.780	0.286	0.333	0.404
7310	0.763	0.268	0.312	0.391

7315	0.773	0.241	0.309	0.367
7320	0.799	0.334	0.371	0.442
7325	0.776	0.260	0.320	0.383
7330	0.780	0.312	0.343	0.425
7335	0.754	0.229	0.290	0.360
7340	0.808	0.386	0.397	0.484
7345	0.801	0.331	0.372	0.439
7350	0.790	0.341	0.363	0.447
7355	0.795	0.368	0.377	0.468
7360	0.785	0.340	0.357	0.446
7365	0.795	0.329	0.365	0.438
7370	0.773	0.262	0.318	0.385
7375	0.764	0.243	0.303	0.370
7380	0.769	0.278	0.321	0.398
7385	0.766	0.259	0.311	0.384
7390	0.782	0.341	0.354	0.447
7395	0.766	0.273	0.317	0.395
7400	0.790	0.321	0.357	0.432
7405	0.792	0.304	0.352	0.417
7410	0.782	0.254	0.322	0.376
7415	0.756	0.232	0.292	0.362
7420	0.797	0.285	0.351	0.400
7425	0.785	0.307	0.346	0.421
7430	0.782	0.322	0.348	0.432
7435	0.778	0.317	0.343	0.429
7440	0.816	0.369	0.405	0.470
7445	0.769	0.278	0.321	0.398
7450	0.799	0.347	0.375	0.453
7455	0.763	0.302	0.325	0.417
7460	0.769	0.285	0.324	0.404
7465	0.790	0.308	0.352	0.421
7470	0.795	0.329	0.365	0.438
7475	0.780	0.292	0.336	0.409
7480	0.802	0.359	0.383	0.462
7485	0.761	0.203	0.282	0.337
7490	0.761	0.293	0.320	0.410
7495	0.775	0.292	0.331	0.409
7500	0.794	0.339	0.366	0.447
7505	0.782	0.275	0.331	0.394
7510	0.790	0.321	0.357	0.432
7515	0.785	0.307	0.346	0.421
7520	0.768	0.309	0.331	0.422
7525	0.795	0.361	0.375	0.464

7530	0.757	0.275	0.311	0.397
7535	0.811	0.367	0.397	0.468
7540	0.754	0.264	0.304	0.388
7545	0.766	0.259	0.311	0.384
7550	0.782	0.302	0.341	0.417
7555	0.756	0.246	0.298	0.373
7560	0.778	0.330	0.347	0.439
7565	0.754	0.250	0.299	0.377
7570	0.766	0.300	0.327	0.416
7575	0.754	0.243	0.296	0.372
7580	0.785	0.320	0.351	0.431
7585	0.768	0.282	0.321	0.402
7590	0.757	0.213	0.285	0.346
7595	0.756	0.273	0.309	0.395
7600	0.750	0.225	0.286	0.357
7605	0.787	0.289	0.341	0.406
7610	0.783	0.242	0.319	0.365
7615	0.785	0.293	0.341	0.410
7620	0.764	0.304	0.327	0.419
7625	0.820	0.394	0.417	0.490
7630	0.782	0.302	0.341	0.417
7635	0.797	0.325	0.366	0.435
7640	0.778	0.283	0.331	0.402
7645	0.776	0.274	0.326	0.394
7650	0.815	0.391	0.408	0.488
7655	0.757	0.234	0.294	0.364
7660	0.778	0.303	0.338	0.418
7665	0.806	0.326	0.377	0.434
7670	0.778	0.310	0.341	0.423
7675	0.771	0.287	0.326	0.405
7680	0.759	0.284	0.315	0.403
7685	0.730	0.261	0.288	0.386
7690	0.769	0.264	0.316	0.387
7695	0.782	0.341	0.354	0.447
7700	0.754	0.250	0.299	0.377
7705	0.778	0.269	0.325	0.390
7710	0.780	0.312	0.343	0.425
7715	0.750	0.246	0.295	0.374
7720	0.757	0.262	0.306	0.386
7725	0.759	0.171	0.265	0.308
7730	0.809	0.383	0.398	0.481
7735	0.778	0.290	0.333	0.407
7740	0.797	0.292	0.354	0.406

7745	0.806	0.377	0.392	0.477
7750	0.757	0.227	0.291	0.358
7755	0.768	0.296	0.326	0.412
7760	0.785	0.313	0.348	0.426
7765	0.747	0.228	0.285	0.360
7770	0.756	0.259	0.303	0.384
7775	0.761	0.238	0.299	0.367
7780	0.747	0.228	0.285	0.360
7785	0.785	0.273	0.333	0.392
7790	0.808	0.367	0.392	0.469
7795	0.768	0.282	0.321	0.402
7800	0.759	0.236	0.296	0.365
7805	0.799	0.360	0.379	0.463
7810	0.763	0.262	0.309	0.386
7815	0.761	0.238	0.299	0.367
7820	0.773	0.283	0.326	0.402
7825	0.764	0.236	0.300	0.364
7830	0.768	0.296	0.326	0.412
7835	0.775	0.222	0.302	0.350
7840	0.768	0.296	0.326	0.412
7845	0.821	0.397	0.420	0.493
7850	0.769	0.278	0.321	0.398
7855	0.754	0.298	0.316	0.413
7860	0.771	0.287	0.326	0.405
7865	0.783	0.324	0.350	0.434
7870	0.804	0.362	0.386	0.464
7875	0.763	0.268	0.312	0.391
7880	0.818	0.385	0.412	0.483
7885	0.769	0.229	0.301	0.357
7890	0.794	0.352	0.370	0.457
7895	0.801	0.311	0.365	0.422
7900	0.754	0.236	0.293	0.366
7905	0.724	0.165	0.245	0.312
7910	0.782	0.232	0.313	0.357
7915	0.757	0.227	0.291	0.358
7920	0.801	0.350	0.378	0.455
7925	0.773	0.309	0.336	0.423
7930	0.785	0.252	0.325	0.374
7935	0.731	0.208	0.268	0.346
7940	0.775	0.285	0.328	0.404
7945	0.768	0.289	0.324	0.407
7950	0.768	0.227	0.298	0.356
7955	0.761	0.238	0.299	0.367

7960	0.761	0.231	0.295	0.361
7965	0.754	0.250	0.299	0.377
7970	0.778	0.297	0.336	0.413
7975	0.790	0.288	0.345	0.404
7980	0.764	0.298	0.324	0.414
7985	0.773	0.296	0.331	0.413
7990	0.773	0.323	0.340	0.433
7995	0.752	0.248	0.297	0.376
8000	0.783	0.270	0.331	0.390
8005	0.776	0.314	0.340	0.427
8010	0.773	0.283	0.326	0.402
8015	0.780	0.279	0.331	0.398
8020	0.771	0.260	0.315	0.383
8025	0.806	0.306	0.370	0.417
8030	0.761	0.259	0.307	0.384
8035	0.783	0.263	0.328	0.384
8040	0.771	0.246	0.310	0.371
8045	0.790	0.314	0.354	0.427
8050	0.766	0.210	0.289	0.341
8055	0.792	0.297	0.350	0.412
8060	0.797	0.305	0.359	0.418
8065	0.752	0.241	0.294	0.370
8070	0.815	0.354	0.398	0.457
8075	0.776	0.260	0.320	0.383
8080	0.773	0.289	0.328	0.407
8085	0.797	0.338	0.370	0.445
8090	0.771	0.273	0.321	0.394
8095	0.757	0.227	0.291	0.358
8100	0.787	0.296	0.344	0.411
8105	0.776	0.239	0.311	0.365
8110	0.773	0.262	0.318	0.385
8115	0.773	0.234	0.306	0.361
8120	0.745	0.197	0.270	0.335
8125	0.763	0.241	0.301	0.369
8130	0.756	0.225	0.289	0.356
8135	0.792	0.304	0.352	0.417
8140	0.802	0.333	0.375	0.441
8145	0.747	0.228	0.285	0.360
8150	0.787	0.316	0.351	0.428
8155	0.782	0.295	0.338	0.411
8160	0.778	0.263	0.323	0.385
8165	0.773	0.241	0.309	0.367
8170	0.795	0.348	0.371	0.454

8175	0.775	0.278	0.326	0.398
8180	0.792	0.297	0.350	0.412
8185	0.757	0.227	0.291	0.358
8190	0.790	0.334	0.361	0.442
8195	0.780	0.292	0.336	0.409
8200	0.773	0.283	0.326	0.402
8205	0.790	0.301	0.350	0.415
8210	0.790	0.301	0.350	0.415
8215	0.790	0.314	0.354	0.427
8220	0.780	0.265	0.325	0.386
8225	0.778	0.290	0.333	0.407
8230	0.799	0.354	0.377	0.458
8235	0.764	0.271	0.314	0.393
8240	0.778	0.283	0.331	0.402
8245	0.815	0.373	0.403	0.473
8250	0.813	0.376	0.402	0.476
8255	0.802	0.333	0.375	0.441
8260	0.802	0.346	0.379	0.452
8265	0.783	0.263	0.328	0.384
8270	0.808	0.380	0.395	0.479
8275	0.773	0.255	0.315	0.379
8280	0.789	0.312	0.352	0.425
8285	0.797	0.312	0.361	0.424
8290	0.776	0.281	0.328	0.400
8295	0.797	0.364	0.378	0.466
8300	0.825	0.403	0.427	0.498
8305	0.785	0.293	0.341	0.410
8310	0.813	0.338	0.391	0.443
8315	0.771	0.300	0.331	0.416
8320	0.757	0.234	0.294	0.364
8325	0.764	0.271	0.314	0.393
8330	0.802	0.314	0.368	0.424
8335	0.775	0.271	0.323	0.393
8340	0.794	0.293	0.350	0.408
8345	0.778	0.283	0.331	0.402
8350	0.790	0.314	0.354	0.427
8355	0.769	0.285	0.324	0.404
8360	0.808	0.386	0.397	0.484
8365	0.785	0.333	0.355	0.441
8370	0.771	0.307	0.333	0.421
8375	0.776	0.301	0.336	0.416
8380	0.801	0.356	0.380	0.460
8385	0.776	0.301	0.336	0.416

8390	0.804	0.362	0.386	0.464
8395	0.766	0.252	0.308	0.378
8400	0.795	0.316	0.361	0.427
8405	0.799	0.334	0.371	0.442
8410	0.776	0.281	0.328	0.400
8415	0.797	0.345	0.372	0.451
8420	0.789	0.285	0.342	0.402
8425	0.782	0.275	0.331	0.394
8430	0.787	0.289	0.341	0.406
8435	0.799	0.347	0.375	0.453
8440	0.795	0.296	0.353	0.410
8445	0.795	0.342	0.369	0.449
8450	0.787	0.348	0.362	0.453
8455	0.769	0.257	0.313	0.381
8460	0.823	0.400	0.424	0.495
8465	0.799	0.334	0.371	0.442
8470	0.763	0.275	0.315	0.396
8475	0.804	0.349	0.382	0.454
8480	0.801	0.317	0.368	0.428
8485	0.797	0.351	0.374	0.456
8490	0.785	0.313	0.348	0.426
8495	0.789	0.292	0.344	0.408
8500	0.759	0.229	0.293	0.359
8505	0.768	0.241	0.305	0.368
8510	0.794	0.320	0.360	0.431
8515	0.778	0.256	0.320	0.379
8520	0.768	0.289	0.324	0.407
8525	0.763	0.309	0.327	0.422
8530	0.776	0.260	0.320	0.383
8535	0.763	0.220	0.291	0.351
8540	0.797	0.345	0.372	0.451
8545	0.809	0.358	0.392	0.461
8550	0.776	0.308	0.338	0.422
8555	0.768	0.289	0.324	0.407
8560	0.790	0.360	0.369	0.462
8565	0.775	0.271	0.323	0.393
8570	0.789	0.318	0.354	0.430
8575	0.768	0.282	0.321	0.402
8580	0.773	0.262	0.318	0.385
8585	0.797	0.351	0.374	0.456
8590	0.754	0.257	0.301	0.383
8595	0.757	0.227	0.291	0.358
8600	0.795	0.316	0.361	0.427

8605	0.778	0.269	0.325	0.390
8610	0.792	0.304	0.352	0.417
8615	0.780	0.279	0.331	0.398
8620	0.782	0.295	0.338	0.411
8625	0.780	0.279	0.331	0.398
8630	0.787	0.309	0.349	0.423
8635	0.815	0.366	0.402	0.468
8640	0.809	0.338	0.386	0.444
8645	0.785	0.307	0.346	0.421
8650	0.754	0.284	0.312	0.403
8655	0.797	0.318	0.364	0.429
8660	0.775	0.271	0.323	0.393
8665	0.802	0.359	0.383	0.462
8670	0.787	0.309	0.349	0.423
8675	0.797	0.351	0.374	0.456
8680	0.771	0.260	0.315	0.383
8685	0.797	0.332	0.368	0.440
8690	0.789	0.285	0.342	0.402
8695	0.789	0.236	0.321	0.358
8700	0.794	0.300	0.353	0.414
8705	0.794	0.359	0.372	0.462
8710	0.795	0.296	0.353	0.410
8715	0.785	0.327	0.353	0.436
8720	0.787	0.275	0.336	0.394
8725	0.795	0.329	0.365	0.438
8730	0.783	0.304	0.344	0.419
8735	0.799	0.354	0.377	0.458
8740	0.768	0.282	0.321	0.402
8745	0.816	0.369	0.405	0.470
8750	0.768	0.248	0.308	0.374
8755	0.802	0.340	0.377	0.447
8760	0.785	0.300	0.344	0.415
8765	0.808	0.367	0.392	0.469
8770	0.754	0.194	0.273	0.330
8775	0.776	0.267	0.323	0.389
8780	0.778	0.310	0.341	0.423
8785	0.769	0.285	0.324	0.404
8790	0.785	0.327	0.353	0.436
8795	0.804	0.368	0.388	0.469
8800	0.789	0.345	0.362	0.450
8805	0.795	0.342	0.369	0.449
8810	0.778	0.269	0.325	0.390
8815	0.785	0.313	0.348	0.426

8820	0.752	0.262	0.302	0.386
8825	0.802	0.366	0.385	0.467
8830	0.785	0.320	0.351	0.431
8835	0.782	0.281	0.333	0.400
8840	0.780	0.272	0.328	0.392
8845	0.780	0.286	0.333	0.404
8850	0.773	0.283	0.326	0.402
8855	0.790	0.308	0.352	0.421
8860	0.771	0.280	0.324	0.400
8865	0.752	0.227	0.288	0.359
8870	0.761	0.273	0.313	0.395
8875	0.783	0.304	0.344	0.419
8880	0.787	0.316	0.351	0.428
8885	0.757	0.234	0.294	0.364
8890	0.776	0.225	0.304	0.352
8895	0.756	0.232	0.292	0.362
8900	0.782	0.302	0.341	0.417
8905	0.768	0.269	0.316	0.391
8910	0.790	0.314	0.354	0.427
8915	0.768	0.262	0.313	0.385
8920	0.723	0.155	0.240	0.304
8925	0.780	0.286	0.333	0.404
8930	0.771	0.267	0.318	0.389
8935	0.769	0.285	0.324	0.404
8940	0.785	0.327	0.353	0.436
8945	0.757	0.213	0.285	0.346
8950	0.768	0.248	0.308	0.374
8955	0.789	0.292	0.344	0.408
8960	0.782	0.315	0.346	0.427
8965	0.769	0.298	0.329	0.414
8970	0.776	0.301	0.336	0.416
8975	0.773	0.234	0.306	0.361
8980	0.771	0.280	0.324	0.400
8985	0.742	0.214	0.276	0.349
8990	0.808	0.355	0.388	0.459
8995	0.769	0.243	0.307	0.370
9000	0.759	0.222	0.290	0.353
9005	0.792	0.276	0.342	0.394
9010	0.797	0.345	0.372	0.451
9015	0.771	0.217	0.297	0.347
9020	0.778	0.256	0.320	0.379
9025	0.795	0.335	0.367	0.443
9030	0.780	0.299	0.338	0.415

9035	0.783	0.284	0.336	0.402
9040	0.775	0.292	0.331	0.409
9045	0.773	0.255	0.315	0.379
9050	0.756	0.217	0.286	0.350
9055	0.825	0.427	0.432	0.517
9060	0.787	0.329	0.356	0.438
9065	0.802	0.359	0.383	0.462
9070	0.757	0.213	0.285	0.346
9075	0.776	0.314	0.340	0.427
9080	0.790	0.308	0.352	0.421
9085	0.787	0.329	0.356	0.438
9090	0.766	0.259	0.311	0.384
9095	0.816	0.388	0.410	0.485
9100	0.782	0.268	0.328	0.388
9105	0.778	0.290	0.333	0.407
9110	0.773	0.262	0.318	0.385
9115	0.759	0.264	0.308	0.388
9120	0.764	0.271	0.314	0.393
9125	0.797	0.377	0.381	0.475
9130	0.787	0.309	0.349	0.423
9135	0.759	0.277	0.313	0.398
9140	0.763	0.234	0.298	0.363
9145	0.768	0.212	0.292	0.343
9150	0.795	0.309	0.358	0.422
9155	0.799	0.301	0.360	0.414
9160	0.782	0.275	0.331	0.394
9165	0.808	0.342	0.385	0.448
9170	0.768	0.282	0.321	0.402
9175	0.787	0.303	0.346	0.417
9180	0.759	0.243	0.299	0.371
9185	0.759	0.229	0.293	0.359
9190	0.773	0.248	0.312	0.373
9195	0.790	0.328	0.359	0.437
9200	0.749	0.278	0.306	0.398
9205	0.780	0.265	0.325	0.386
9210	0.769	0.264	0.316	0.387
9215	0.763	0.212	0.288	0.344
9220	0.783	0.324	0.350	0.434
9225	0.745	0.225	0.283	0.358
9230	0.768	0.275	0.319	0.396
9235	0.754	0.257	0.301	0.383
9240	0.780	0.279	0.331	0.398
9245	0.790	0.314	0.354	0.427

9250	0.782	0.275	0.331	0.394
9255	0.775	0.236	0.308	0.363
9260	0.782	0.275	0.331	0.394
9265	0.785	0.273	0.333	0.392
9270	0.778	0.256	0.320	0.379
9275	0.783	0.263	0.328	0.384
9280	0.780	0.292	0.336	0.409
9285	0.775	0.278	0.326	0.398
9290	0.783	0.304	0.344	0.419
9295	0.776	0.267	0.323	0.389
9300	0.775	0.265	0.320	0.387
9305	0.754	0.236	0.293	0.366
9310	0.790	0.321	0.357	0.432
9315	0.787	0.296	0.344	0.411
9320	0.775	0.251	0.315	0.375
9325	0.799	0.321	0.367	0.431
9330	0.773	0.283	0.326	0.402
9335	0.778	0.303	0.338	0.418
9340	0.790	0.328	0.359	0.437
9345	0.761	0.224	0.292	0.355
9350	0.757	0.227	0.291	0.358
9355	0.790	0.308	0.352	0.421
9360	0.766	0.273	0.317	0.395
9365	0.771	0.300	0.331	0.416
9370	0.804	0.375	0.389	0.474
9375	0.759	0.215	0.287	0.347
9380	0.782	0.295	0.338	0.411
9385	0.769	0.285	0.324	0.404
9390	0.750	0.225	0.286	0.357
9395	0.785	0.307	0.346	0.421
9400	0.761	0.245	0.301	0.373
9405	0.804	0.362	0.386	0.464
9410	0.757	0.255	0.303	0.381
9415	0.778	0.317	0.343	0.429
9420	0.785	0.280	0.336	0.398
9425	0.780	0.292	0.336	0.409
9430	0.787	0.289	0.341	0.406
9435	0.761	0.245	0.301	0.373
9440	0.773	0.255	0.315	0.379
9445	0.790	0.314	0.354	0.427
9450	0.813	0.376	0.402	0.476
9455	0.764	0.278	0.317	0.398
9460	0.795	0.322	0.363	0.433

9465	0.757	0.262	0.306	0.386
9470	0.787	0.303	0.346	0.417
9475	0.771	0.260	0.315	0.383
9480	0.776	0.287	0.331	0.406
9485	0.763	0.268	0.312	0.391
9490	0.744	0.223	0.281	0.357
9495	0.747	0.206	0.275	0.342
9500	0.789	0.264	0.333	0.384
9505	0.787	0.240	0.321	0.363
9510	0.797	0.338	0.370	0.445
9515	0.790	0.301	0.350	0.415
9520	0.787	0.275	0.336	0.394
9525	0.787	0.322	0.353	0.433
9530	0.759	0.243	0.299	0.371
9535	0.771	0.287	0.326	0.405
9540	0.768	0.234	0.302	0.362
9545	0.750	0.189	0.269	0.327
9550	0.773	0.248	0.312	0.373
9555	0.783	0.317	0.348	0.429
9560	0.768	0.296	0.326	0.412
9565	0.776	0.294	0.333	0.411
9570	0.783	0.277	0.333	0.396
9575	0.761	0.245	0.301	0.373
9580	0.768	0.275	0.319	0.396
9585	0.785	0.307	0.346	0.421
9590	0.776	0.281	0.328	0.400
9595	0.799	0.328	0.369	0.437
9600	0.801	0.324	0.370	0.433
9605	0.773	0.241	0.309	0.367
9610	0.790	0.328	0.359	0.437
9615	0.782	0.322	0.348	0.432
9620	0.789	0.305	0.349	0.419
9625	0.764	0.271	0.314	0.393
9630	0.799	0.328	0.369	0.437
9635	0.790	0.281	0.342	0.398
9640	0.783	0.291	0.339	0.408
9645	0.764	0.278	0.317	0.398
9650	0.780	0.299	0.338	0.415
9655	0.776	0.294	0.333	0.411
9660	0.761	0.210	0.286	0.343
9665	0.782	0.315	0.346	0.427
9670	0.761	0.259	0.307	0.384
9675	0.776	0.294	0.333	0.411

9680	0.775	0.292	0.331	0.409
9685	0.763	0.241	0.301	0.369
9690	0.794	0.333	0.364	0.441
9695	0.804	0.316	0.372	0.426
9700	0.787	0.329	0.356	0.438
9705	0.764	0.222	0.294	0.352
9710	0.797	0.345	0.372	0.451
9715	0.737	0.179	0.257	0.321
9720	0.792	0.317	0.357	0.429
9725	0.792	0.304	0.352	0.417
9730	0.785	0.286	0.339	0.404
9735	0.789	0.351	0.364	0.455
9740	0.782	0.302	0.341	0.417
9745	0.759	0.193	0.276	0.329
9750	0.775	0.236	0.308	0.363
9755	0.742	0.200	0.270	0.338
9760	0.773	0.241	0.309	0.367
9765	0.768	0.269	0.316	0.391
9770	0.766	0.231	0.299	0.360
9775	0.768	0.282	0.321	0.402
9780	0.759	0.250	0.302	0.377
9785	0.787	0.289	0.341	0.406
9790	0.782	0.288	0.336	0.406
9795	0.785	0.252	0.325	0.374
9800	0.776	0.314	0.340	0.427
9805	0.759	0.277	0.313	0.398
9810	0.792	0.324	0.359	0.434
9815	0.773	0.289	0.328	0.407
9820	0.757	0.198	0.278	0.333
9825	0.795	0.335	0.367	0.443
9830	0.766	0.231	0.299	0.360
9835	0.778	0.330	0.347	0.439
9840	0.795	0.335	0.367	0.443
9845	0.794	0.384	0.379	0.480
9850	0.752	0.241	0.294	0.370
9855	0.759	0.291	0.318	0.409
9860	0.759	0.236	0.296	0.365
9865	0.778	0.249	0.317	0.373
9870	0.795	0.329	0.365	0.438
9875	0.756	0.259	0.303	0.384
9880	0.799	0.301	0.360	0.414
9885	0.775	0.251	0.315	0.375
9890	0.766	0.217	0.293	0.348

9895	0.794	0.279	0.345	0.396
9900	0.790	0.347	0.365	0.452
9905	0.799	0.347	0.375	0.453
9910	0.780	0.272	0.328	0.392
9915	0.778	0.263	0.323	0.385
9920	0.761	0.224	0.292	0.355
9925	0.790	0.288	0.345	0.404
9930	0.806	0.345	0.383	0.451
9935	0.769	0.229	0.301	0.357
9940	0.769	0.264	0.316	0.387
9945	0.769	0.298	0.329	0.414
9950	0.778	0.241	0.314	0.366
9955	0.759	0.208	0.283	0.341
9960	0.771	0.287	0.326	0.405
9965	0.776	0.260	0.320	0.383
9970	0.773	0.289	0.328	0.407
9975	0.759	0.243	0.299	0.371
9980	0.763	0.212	0.288	0.344
9985	0.787	0.303	0.346	0.417
9990	0.795	0.289	0.351	0.404
9995	0.795	0.296	0.353	0.410
10000	0.773	0.269	0.321	0.391

#
