# Supplementary material for: Identification of Genes Associated with the Impairment of Olfactory and Gustatory Functions in COVID-19 via Machine-Learning Methods
Source: Life (Basel). 2023 Mar 15;13(3):798. doi: 10.3390/life13030798 (PMC10051382; doi:10.3390/life13030798)
Supplement: Supplementary file 1 [file life-13-00798-s001.zip › Table S3.pdf]

Supplementary Table S3: Intersection of LASSO, LightGBM, and MCFS for the optimal subset of features. The features that appear in the 3, 2, and 1 optimal feature subsets are shown.

Features found by one method	Features found by two methods	Features found by three methods
ENSG00000254624.1	ENSG00000188375.5	ENSG00000234134.1
ENSG00000223379.1	ENSG00000260693.1	
ENSG00000168528.12	ENSG00000210191.1	
ENSG00000278931.1	ENSG00000258926.1	
ENSG00000203581.7	ENSG00000165609.13	
ENSG00000229596.3	ENSG00000225611.1	
ENSG00000261707.1	ENSG00000104973.18	
ENSG00000229190.1	ENSG00000010361.13	
ENSG00000183562.3	ENSG00000163032.11	
ENSG00000180104.16	ENSG00000149798.5	
ENSG00000199719.1	ENSG00000101544.9	
ENSG00000260135.6	ENSG00000165406.16	
ENSG00000250781.1	ENSG00000260231.2	
ENSG00000279315.1	ENSG00000149260.17	
ENSG00000240445.3	ENSG00000158710.14	
ENSG00000111305.19	ENSG00000171360.3	
ENSG00000269886.1	ENSG00000264204.2	
ENSG00000267708.1	ENSG00000240403.5	
ENSG00000162594.15	ENSG00000185551.15	
ENSG00000267643.1		
ENSG00000189184.11		
ENSG00000259895.1		
ENSG00000122786.20		
ENSG00000237310.1		
ENSG00000273680.1		
ENSG00000286220.1		
ENSG00000167985.6		
ENSG00000233834.6		
ENSG00000272274.1		
ENSG00000273262.1		
ENSG00000248468.1		
ENSG00000143297.19		
ENSG00000101294.17		
ENSG00000170959.14		
ENSG00000166450.13		
ENSG00000105976.15		
ENSG00000126266.3		
ENSG00000228742.10		
ENSG00000269982.1		
ENSG00000278084.1		
ENSG00000254911.3		
ENSG00000106341.11		
ENSG00000255355.1		
ENSG00000196972.9		
ENSG00000178860.8		
ENSG00000188037.11		
ENSG00000279859.2		
ENSG00000226650.5		
ENSG00000247151.7		

ENSG00000271787.1		
ENSG00000048540.15		
ENSG00000267023.5		
ENSG00000253475.1		
ENSG00000236204.6		
ENSG00000267749.1		
ENSG00000242580.1		
ENSG00000285918.1		
ENSG00000000003.14		
ENSG00000000005.6		
ENSG00000000419.12		
ENSG00000000457.14		
ENSG00000000460.17		
ENSG00000000938.13		
ENSG00000000971.15		
ENSG00000001036.13		
ENSG00000001084.12		
ENSG00000001167.14		
ENSG00000001460.18		
ENSG00000001461.17		
ENSG00000001497.16		
ENSG00000001561.7		
ENSG00000001617.12		
ENSG00000001626.15		
ENSG00000001629.10		
ENSG00000001630.17		
ENSG00000001631.15		
ENSG00000002016.17		
ENSG00000002079.14		
ENSG00000002330.13		
ENSG00000002549.12		
ENSG00000002586.20		
ENSG00000002586.20		
ENSG00000002587.10		
ENSG00000002726.20		
ENSG00000259515.1		
ENSG00000236496.2		
ENSG00000259674.1		
ENSG00000140941.13		
ENSG00000229007.1		
ENSG00000196933.5		
ENSG00000022556.16		
ENSG00000270157.1		
ENSG00000267598.1		
ENSG00000267226.2		
ENSG00000211689.7		
ENSG00000172425.10		
ENSG00000163121.10		
ENSG00000125656.10		
ENSG00000267984.1		
ENSG00000244734.4		
ENSG00000240137.5		
ENSG00000239975.2		

ENSG00000164308.16		
ENSG00000124839.13		
ENSG00000105889.15		
ENSG00000054179.12		
ENSG00000250067.12		
ENSG00000235052.1		
ENSG00000226855.1		
ENSG00000197111.15		
ENSG00000165730.16		
ENSG00000150045.12		
ENSG00000137267.6		
ENSG00000286122.1		
ENSG00000267082.1		
ENSG00000239203.1		
ENSG00000230433.1		
ENSG00000197182.14		
ENSG00000184361.13		
ENSG00000184292.6		
ENSG00000124549.14		
ENSG00000230526.1		
ENSG00000092054.13		
ENSG00000131370.16		
ENSG00000130522.5		
ENSG00000261402.1		
ENSG00000176009.3		
ENSG00000012048.22		
ENSG00000104904.12		
ENSG00000254990.5		
ENSG00000224328.1		
ENSG00000185177.12		
ENSG00000271412.1		
ENSG00000175398.2		
ENSG00000235975.1		
ENSG00000198858.10		
ENSG00000214374.2		
ENSG00000026950.17		
ENSG00000173825.7		
ENSG00000182809.10		
ENSG00000277288.4		
ENSG00000258763.5		
ENSG00000286058.1		
ENSG00000244411.3		
ENSG00000073737.16		
ENSG00000271071.1		
ENSG00000227621.1		
ENSG00000201988.2		
ENSG00000285000.1		
ENSG00000253204.1		
ENSG00000099250.18		
ENSG00000155307.18		
ENSG00000233456.1		
ENSG00000258819.1		
ENSG00000096088.16		

ENSG00000241935.9		
ENSG00000133606.11		
ENSG00000233242.2		
ENSG00000235399.1		
ENSG00000189430.12		
ENSG00000165495.16		
ENSG00000128283.7		
ENSG00000180304.14		
ENSG00000257951.2		
ENSG00000238243.3		
ENSG00000173239.13		
ENSG00000196998.18		
ENSG00000196553.15		
ENSG00000255356.2		
ENSG00000259341.1		
ENSG00000198963.11		
ENSG00000283202.1		
ENSG00000273776.1		
ENSG00000253677.1		
ENSG00000198901.14		
ENSG00000163346.17		
ENSG00000239590.1		
ENSG00000250961.1		
ENSG00000103381.12		
ENSG00000254664.1		
ENSG00000285685.1		
ENSG00000229807.11		
ENSG00000285064.1		
ENSG00000013306.16		
