# Supplementary material for: Identification of Genes Associated with the Impairment of Olfactory and Gustatory Functions in COVID-19 via Machine-Learning Methods
Source: Life (Basel). 2023 Mar 15;13(3):798. doi: 10.3390/life13030798 (PMC10051382; doi:10.3390/life13030798)
Supplement: Supplementary file 1 [file life-13-00798-s001.zip › Table S4.pdf]

Supplementary Table S4: Classification rules generated by the optimal DT classifier.

(1) Rules on list yielded by LASSO

Rules_0	passed counts:354
node_0: feature_name=ENSG00000012048.22	feature_id[371].value <= threshold=13.43457317352295
node_1: feature_name=ENSG00000047578.13	feature_id[684].value <= threshold=53.576541900634766
node_2: feature_name=ENSG00000007952.18	feature_id[257].value <= threshold=0.4518554210662842
node_3: feature_name=ENSG00000026297.15	feature_id[517].value > threshold=274.33460998535156
node_9: feature_name=ENSG00000008277.14	feature_id[270].value <= threshold=4.677093505859375
node_10: feature_name=ENSG00000049192.15	feature_id[715].value <= threshold=2.364746928215027
node_11: feature_name=ENSG00000011638.10	feature_id[369].value <= threshold=10.54316234588623
node_12: feature_name=ENSG00000033627.16	feature_id[566].value <= threshold=148.1126251220703
node_13: feature_name=ENSG00000002746.15	feature_id[91].value <= threshold=0.17109555006027222
node_14: feature_name=ENSG00000033100.16	feature_id[561].value <= threshold=121.37638854980469
node_15: feature_name=ENSG00000025434.19	feature_id[508].value <= threshold=16.221430778503418
node_16: feature_name=ENSG00000028310.18	feature_id[533].value <= threshold=73.98308944702148
node_17: feature_name=ENSG00000031081.10	feature_id[550].value <= threshold=35.2840518951416
node_18: feature_name=ENSG00000035720.8	feature_id[585].value <= threshold=24.809341430664062
node_19: feature_name=ENSG00000016402.13	feature_id[430].value <= threshold=0.4444928616285324
node_20: feature_name=ENSG00000003137.8	feature_id[98].value <= threshold=9.946229934692383
node_21: feature_name=ENSG00000014216.15	feature_id[410].value > threshold=136.36983489990234
node_25: feature_name=ENSG00000039600.11	feature_id[623].value <= threshold=1.2662832140922546
node_26: feature_name=ENSG00000000460.17	feature_id[67].value <= threshold=6.493743658065796
node_27: feature_name=ENSG00000037757.14	feature_id[603].value <= threshold=63.50008010864258
node_28: feature_name=ENSG00000004846.16	feature_id[127].value <= threshold=0.09349484369158745

node_29: feature_name=ENSG00000041353.10	feature_id[635].value > threshold=2.9915499687194824
node_33: feature_name=ENSG00000005189.19	feature_id[149].value <= threshold=2.6748963594436646
Class: 1	
Rules_1	passed counts:47
node_0: feature_name=ENSG00000012048.22	feature_id[371].value > threshold=13.43457317352295
node_72: feature_name=ENSG00000162594.15	feature_id[20].value > threshold=0.23572762310504913
node_88: feature_name=ENSG00000009950.16	feature_id[304].value <= threshold=0.2558544874191284
node_89: feature_name=ENSG00000051128.19	feature_id[750].value <= threshold=43.44260597229004
Class: 1	
Rules_2	passed counts:29
node_0: feature_name=ENSG00000012048.22	feature_id[371].value > threshold=13.43457317352295
node_72: feature_name=ENSG00000162594.15	feature_id[20].value <= threshold=0.23572762310504913
node_73: feature_name=ENSG00000042317.17	feature_id[646].value <= threshold=1.344666063785553
node_74: feature_name=ENSG00000042753.11	feature_id[650].value <= threshold=41.77660369873047
node_75: feature_name=ENSG00000019582.15	feature_id[455].value > threshold=2379.6041259765625
node_77: feature_name=ENSG00000014824.14	feature_id[413].value <= threshold=18.987863540649414
Class: 1	
Rules_3	passed counts:29
node_0: feature_name=ENSG00000012048.22	feature_id[371].value <= threshold=13.43457317352295
node_1: feature_name=ENSG00000047578.13	feature_id[684].value <= threshold=53.576541900634766
node_2: feature_name=ENSG00000007952.18	feature_id[257].value <= threshold=0.4518554210662842
node_3: feature_name=ENSG00000026297.15	feature_id[517].value > threshold=274.33460998535156

node_9: feature_name=ENSG00000008277.14	feature_id[270].value <= threshold=4.677093505859375
node_10: feature_name=ENSG00000049192.15	feature_id[715].value <= threshold=2.364746928215027
node_11: feature_name=ENSG00000011638.10	feature_id[369].value <= threshold=10.54316234588623
node_12: feature_name=ENSG00000033627.16	feature_id[566].value <= threshold=148.1126251220703
node_13: feature_name=ENSG00000002746.15	feature_id[91].value <= threshold=0.17109555006027222
node_14: feature_name=ENSG00000033100.16	feature_id[561].value <= threshold=121.37638854980469
node_15: feature_name=ENSG00000025434.19	feature_id[508].value <= threshold=16.221430778503418
node_16: feature_name=ENSG00000028310.18	feature_id[533].value <= threshold=73.98308944702148
node_17: feature_name=ENSG00000031081.10	feature_id[550].value <= threshold=35.2840518951416
node_18: feature_name=ENSG00000035720.8	feature_id[585].value <= threshold=24.809341430664062
node_19: feature_name=ENSG00000016402.13	feature_id[430].value <= threshold=0.4444928616285324
node_20: feature_name=ENSG00000003137.8	feature_id[98].value <= threshold=9.946229934692383
node_21: feature_name=ENSG00000014216.15	feature_id[410].value > threshold=136.36983489990234
node_25: feature_name=ENSG00000039600.11	feature_id[623].value <= threshold=1.2662832140922546
node_26: feature_name=ENSG00000000460.17	feature_id[67].value <= threshold=6.493743658065796
node_27: feature_name=ENSG00000037757.14	feature_id[603].value <= threshold=63.50008010864258
node_28: feature_name=ENSG00000004846.16	feature_id[127].value > threshold=0.09349484369158745
node_38: feature_name=ENSG00000010327.10	feature_id[322].value > threshold=115.10892868041992
node_40: feature_name=ENSG00000018280.17	feature_id[440].value > threshold=316.47174072265625
Class: 1	
Rules_4	passed counts:21
node_0: feature_name=ENSG00000012048.22	feature_id[371].value > threshold=13.43457317352295
node_72: feature_name=ENSG00000162594.15	feature_id[20].value <= threshold=0.23572762310504913
node_73: feature_name=ENSG00000042317.17	feature_id[646].value > threshold=1.344666063785553

node_83: feature_name=ENSG00000269886.1	feature_id[18].value <= threshold=0.25252923369407654
node_84: feature_name=ENSG00000006638.11	feature_id[210].value <= threshold=15.165156841278076
Class: 0	
Rules_5	passed counts:8
node_0: feature_name=ENSG00000012048.22	feature_id[371].value > threshold=13.43457317352295
node_72: feature_name=ENSG00000162594.15	feature_id[20].value <= threshold=0.23572762310504913
node_73: feature_name=ENSG00000042317.17	feature_id[646].value <= threshold=1.344666063785553
node_74: feature_name=ENSG00000042753.11	feature_id[650].value > threshold=41.77660369873047
node_80: feature_name=ENSG00000025772.8	feature_id[511].value <= threshold=13.427343845367432
Class: 0	
Rules_6	passed counts:7
node_0: feature_name=ENSG00000012048.22	feature_id[371].value <= threshold=13.43457317352295
node_1: feature_name=ENSG00000047578.13	feature_id[684].value <= threshold=53.576541900634766
node_2: feature_name=ENSG00000007952.18	feature_id[257].value <= threshold=0.4518554210662842
node_3: feature_name=ENSG00000026297.15	feature_id[517].value <= threshold=274.33460998535156
node_4: feature_name=ENSG00000025423.11	feature_id[507].value > threshold=0.0394274340942502
node_6: feature_name=ENSG00000043591.5	feature_id[661].value > threshold=0.21197742223739624
Class: 1	
Rules_7	passed counts:5
node_0: feature_name=ENSG00000012048.22	feature_id[371].value > threshold=13.43457317352295
node_72: feature_name=ENSG00000162594.15	feature_id[20].value > threshold=0.23572762310504913
node_88: feature_name=ENSG00000009950.16	feature_id[304].value > threshold=0.2558544874191284

node_92: feature_name=ENSG00000015153.14	feature_id[417].value <= threshold=13.395617961883545
Class: 0	
Rules_8	passed counts:5
node_0: feature_name=ENSG00000012048.22	feature_id[371].value <= threshold=13.43457317352295
node_1: feature_name=ENSG00000047578.13	feature_id[684].value <= threshold=53.576541900634766
node_2: feature_name=ENSG00000007952.18	feature_id[257].value <= threshold=0.4518554210662842
node_3: feature_name=ENSG00000026297.15	feature_id[517].value <= threshold=274.33460998535156
node_4: feature_name=ENSG00000025423.11	feature_id[507].value <= threshold=0.0394274340942502
Class: 0	
Rules_9	passed counts:4
node_0: feature_name=ENSG00000012048.22	feature_id[371].value > threshold=13.43457317352295
node_72: feature_name=ENSG00000162594.15	feature_id[20].value <= threshold=0.23572762310504913
node_73: feature_name=ENSG00000042317.17	feature_id[646].value > threshold=1.344666063785553
node_83: feature_name=ENSG00000269886.1	feature_id[18].value > threshold=0.25252923369407654
Class: 1	
Rules_10	passed counts:4
node_0: feature_name=ENSG00000012048.22	feature_id[371].value > threshold=13.43457317352295
node_72: feature_name=ENSG00000162594.15	feature_id[20].value <= threshold=0.23572762310504913
node_73: feature_name=ENSG00000042317.17	feature_id[646].value <= threshold=1.344666063785553
node_74: feature_name=ENSG00000042753.11	feature_id[650].value <= threshold=41.77660369873047
node_75: feature_name=ENSG00000019582.15	feature_id[455].value <= threshold=2379.6041259765625
Class: 0	

Rules_11	passed counts:4
node_0: feature_name=ENSG00000012048.22	feature_id[371].value <= threshold=13.43457317352295
node_1: feature_name=ENSG00000047578.13	feature_id[684].value <= threshold=53.576541900634766
node_2: feature_name=ENSG00000007952.18	feature_id[257].value <= threshold=0.4518554210662842
node_3: feature_name=ENSG00000026297.15	feature_id[517].value > threshold=274.33460998535156
node_9: feature_name=ENSG00000008277.14	feature_id[270].value <= threshold=4.677093505859375
node_10: feature_name=ENSG00000049192.15	feature_id[715].value <= threshold=2.364746928215027
node_11: feature_name=ENSG00000011638.10	feature_id[369].value <= threshold=10.54316234588623
node_12: feature_name=ENSG00000033627.16	feature_id[566].value <= threshold=148.1126251220703
node_13: feature_name=ENSG00000002746.15	feature_id[91].value > threshold=0.17109555006027222
node_59: feature_name=ENSG00000003436.16	feature_id[104].value <= threshold=0.8902605473995209
Class: 1	
Rules_12	passed counts:3
node_0: feature_name=ENSG00000012048.22	feature_id[371].value <= threshold=13.43457317352295
node_1: feature_name=ENSG00000047578.13	feature_id[684].value > threshold=53.576541900634766
Class: 0	
Rules_13	passed counts:3
node_0: feature_name=ENSG00000012048.22	feature_id[371].value <= threshold=13.43457317352295
node_1: feature_name=ENSG00000047578.13	feature_id[684].value <= threshold=53.576541900634766
node_2: feature_name=ENSG00000007952.18	feature_id[257].value > threshold=0.4518554210662842
Class: 0	

Rules_14	passed counts:3
node_0: feature_name=ENSG00000012048.22	feature_id[371].value <= threshold=13.43457317352295
node_1: feature_name=ENSG00000047578.13	feature_id[684].value <= threshold=53.576541900634766
node_2: feature_name=ENSG00000007952.18	feature_id[257].value <= threshold=0.4518554210662842
node_3: feature_name=ENSG00000026297.15	feature_id[517].value > threshold=274.33460998535156
node_9: feature_name=ENSG00000008277.14	feature_id[270].value <= threshold=4.677093505859375
node_10: feature_name=ENSG00000049192.15	feature_id[715].value <= threshold=2.364746928215027
node_11: feature_name=ENSG00000011638.10	feature_id[369].value > threshold=10.54316234588623
node_65: feature_name=ENSG00000033100.16	feature_id[561].value <= threshold=94.66749572753906
Class: 0	
Rules_15	passed counts:3
node_0: feature_name=ENSG00000012048.22	feature_id[371].value <= threshold=13.43457317352295
node_1: feature_name=ENSG00000047578.13	feature_id[684].value <= threshold=53.576541900634766
node_2: feature_name=ENSG00000007952.18	feature_id[257].value <= threshold=0.4518554210662842
node_3: feature_name=ENSG00000026297.15	feature_id[517].value > threshold=274.33460998535156
node_9: feature_name=ENSG00000008277.14	feature_id[270].value <= threshold=4.677093505859375
node_10: feature_name=ENSG00000049192.15	feature_id[715].value <= threshold=2.364746928215027
node_11: feature_name=ENSG00000011638.10	feature_id[369].value <= threshold=10.54316234588623
node_12: feature_name=ENSG00000033627.16	feature_id[566].value > threshold=148.1126251220703
node_62: feature_name=ENSG00000054118.15	feature_id[780].value > threshold=140.7735366821289
Class: 0	
Rules_16	passed counts:3
node_0: feature_name=ENSG00000012048.22	feature_id[371].value <= threshold=13.43457317352295

node_1: feature_name=ENSG00000047578.13	feature_id[684].value <= threshold=53.576541900634766
node_2: feature_name=ENSG00000007952.18	feature_id[257].value <= threshold=0.4518554210662842
node_3: feature_name=ENSG00000026297.15	feature_id[517].value > threshold=274.33460998535156
node_9: feature_name=ENSG00000008277.14	feature_id[270].value <= threshold=4.677093505859375
node_10: feature_name=ENSG00000049192.15	feature_id[715].value <= threshold=2.364746928215027
node_11: feature_name=ENSG00000011638.10	feature_id[369].value <= threshold=10.54316234588623
node_12: feature_name=ENSG00000033627.16	feature_id[566].value > threshold=148.1126251220703
node_62: feature_name=ENSG00000054118.15	feature_id[780].value <= threshold=140.7735366821289
Class: 1	
Rules_17	passed counts:3
node_0: feature_name=ENSG00000012048.22	feature_id[371].value <= threshold=13.43457317352295
node_1: feature_name=ENSG00000047578.13	feature_id[684].value <= threshold=53.576541900634766
node_2: feature_name=ENSG00000007952.18	feature_id[257].value <= threshold=0.4518554210662842
node_3: feature_name=ENSG00000026297.15	feature_id[517].value > threshold=274.33460998535156
node_9: feature_name=ENSG00000008277.14	feature_id[270].value <= threshold=4.677093505859375
node_10: feature_name=ENSG00000049192.15	feature_id[715].value <= threshold=2.364746928215027
node_11: feature_name=ENSG00000011638.10	feature_id[369].value <= threshold=10.54316234588623
node_12: feature_name=ENSG00000033627.16	feature_id[566].value <= threshold=148.1126251220703
node_13: feature_name=ENSG00000002746.15	feature_id[91].value > threshold=0.17109555006027222
node_59: feature_name=ENSG00000003436.16	feature_id[104].value > threshold=0.8902605473995209
Class: 0	
Rules_18	passed counts:3
node_0: feature_name=ENSG00000012048.22	feature_id[371].value <= threshold=13.43457317352295

node_1: feature_name=ENSG00000047578.13	feature_id[684].value <= threshold=53.576541900634766
node_2: feature_name=ENSG00000007952.18	feature_id[257].value <= threshold=0.4518554210662842
node_3: feature_name=ENSG00000026297.15	feature_id[517].value > threshold=274.33460998535156
node_9: feature_name=ENSG00000008277.14	feature_id[270].value <= threshold=4.677093505859375
node_10: feature_name=ENSG00000049192.15	feature_id[715].value <= threshold=2.364746928215027
node_11: feature_name=ENSG00000011638.10	feature_id[369].value <= threshold=10.54316234588623
node_12: feature_name=ENSG00000033627.16	feature_id[566].value <= threshold=148.1126251220703
node_13: feature_name=ENSG00000002746.15	feature_id[91].value <= threshold=0.17109555006027222
node_14: feature_name=ENSG00000033100.16	feature_id[561].value <= threshold=121.37638854980469
node_15: feature_name=ENSG00000025434.19	feature_id[508].value <= threshold=16.221430778503418
node_16: feature_name=ENSG00000028310.18	feature_id[533].value <= threshold=73.98308944702148
node_17: feature_name=ENSG00000031081.10	feature_id[550].value <= threshold=35.2840518951416
node_18: feature_name=ENSG00000035720.8	feature_id[585].value <= threshold=24.809341430664062
node_19: feature_name=ENSG00000016402.13	feature_id[430].value <= threshold=0.4444928616285324
node_20: feature_name=ENSG00000003137.8	feature_id[98].value <= threshold=9.946229934692383
node_21: feature_name=ENSG00000014216.15	feature_id[410].value > threshold=136.36983489990234
node_25: feature_name=ENSG00000039600.11	feature_id[623].value <= threshold=1.2662832140922546
node_26: feature_name=ENSG00000000460.17	feature_id[67].value <= threshold=6.493743658065796
node_27: feature_name=ENSG00000037757.14	feature_id[603].value <= threshold=63.50008010864258
node_28: feature_name=ENSG00000004846.16	feature_id[127].value > threshold=0.09349484369158745
node_38: feature_name=ENSG00000010327.10	feature_id[322].value <= threshold=115.10892868041992
Class: 0	
Rules_19	passed counts:3
node_0: feature_name=ENSG00000012048.22	feature_id[371].value <= threshold=13.43457317352295

node_1: feature_name=ENSG00000047578.13	feature_id[684].value <= threshold=53.576541900634766
node_2: feature_name=ENSG00000007952.18	feature_id[257].value <= threshold=0.4518554210662842
node_3: feature_name=ENSG00000026297.15	feature_id[517].value > threshold=274.33460998535156
node_9: feature_name=ENSG00000008277.14	feature_id[270].value <= threshold=4.677093505859375
node_10: feature_name=ENSG00000049192.15	feature_id[715].value <= threshold=2.364746928215027
node_11: feature_name=ENSG00000011638.10	feature_id[369].value <= threshold=10.54316234588623
node_12: feature_name=ENSG00000033627.16	feature_id[566].value <= threshold=148.1126251220703
node_13: feature_name=ENSG00000002746.15	feature_id[91].value <= threshold=0.17109555006027222
node_14: feature_name=ENSG00000033100.16	feature_id[561].value <= threshold=121.37638854980469
node_15: feature_name=ENSG00000025434.19	feature_id[508].value <= threshold=16.221430778503418
node_16: feature_name=ENSG00000028310.18	feature_id[533].value <= threshold=73.98308944702148
node_17: feature_name=ENSG00000031081.10	feature_id[550].value <= threshold=35.2840518951416
node_18: feature_name=ENSG00000035720.8	feature_id[585].value <= threshold=24.809341430664062
node_19: feature_name=ENSG00000016402.13	feature_id[430].value <= threshold=0.4444928616285324
node_20: feature_name=ENSG00000003137.8	feature_id[98].value <= threshold=9.946229934692383
node_21: feature_name=ENSG00000014216.15	feature_id[410].value <= threshold=136.36983489990234
node_22: feature_name=ENSG00000009694.13	feature_id[296].value <= threshold=1.6295068264007568
Class: 1	
Rules_20	passed counts:2
node_0: feature_name=ENSG00000012048.22	feature_id[371].value > threshold=13.43457317352295
node_72: feature_name=ENSG00000162594.15	feature_id[20].value > threshold=0.23572762310504913
node_88: feature_name=ENSG00000009950.16	feature_id[304].value > threshold=0.2558544874191284
node_92: feature_name=ENSG00000015153.14	feature_id[417].value > threshold=13.395617961883545
Class: 1	

Rules_21	passed counts:2
node_0: feature_name=ENSG00000012048.22	feature_id[371].value > threshold=13.43457317352295
node_72: feature_name=ENSG00000162594.15	feature_id[20].value <= threshold=0.23572762310504913
node_73: feature_name=ENSG00000042317.17	feature_id[646].value <= threshold=1.344666063785553
node_74: feature_name=ENSG00000042753.11	feature_id[650].value <= threshold=41.77660369873047
node_75: feature_name=ENSG00000019582.15	feature_id[455].value > threshold=2379.6041259765625
node_77: feature_name=ENSG00000014824.14	feature_id[413].value > threshold=18.987863540649414
Class: 0	
Rules_22	passed counts:2
node_0: feature_name=ENSG00000012048.22	feature_id[371].value <= threshold=13.43457317352295
node_1: feature_name=ENSG00000047578.13	feature_id[684].value <= threshold=53.576541900634766
node_2: feature_name=ENSG00000007952.18	feature_id[257].value <= threshold=0.4518554210662842
node_3: feature_name=ENSG00000026297.15	feature_id[517].value > threshold=274.33460998535156
node_9: feature_name=ENSG00000008277.14	feature_id[270].value > threshold=4.677093505859375
Class: 0	
Rules_23	passed counts:2
node_0: feature_name=ENSG00000012048.22	feature_id[371].value <= threshold=13.43457317352295
node_1: feature_name=ENSG00000047578.13	feature_id[684].value <= threshold=53.576541900634766
node_2: feature_name=ENSG00000007952.18	feature_id[257].value <= threshold=0.4518554210662842
node_3: feature_name=ENSG00000026297.15	feature_id[517].value > threshold=274.33460998535156
node_9: feature_name=ENSG00000008277.14	feature_id[270].value <= threshold=4.677093505859375
node_10: feature_name=ENSG00000049192.15	feature_id[715].value > threshold=2.364746928215027

Class: 0	
Rules_24	passed counts:2
node_0: feature_name=ENSG00000012048.22	feature_id[371].value <= threshold=13.43457317352295
node_1: feature_name=ENSG00000047578.13	feature_id[684].value <= threshold=53.576541900634766
node_2: feature_name=ENSG00000007952.18	feature_id[257].value <= threshold=0.4518554210662842
node_3: feature_name=ENSG00000026297.15	feature_id[517].value > threshold=274.33460998535156
node_9: feature_name=ENSG00000008277.14	feature_id[270].value <= threshold=4.677093505859375
node_10: feature_name=ENSG00000049192.15	feature_id[715].value <= threshold=2.364746928215027
node_11: feature_name=ENSG00000011638.10	feature_id[369].value > threshold=10.54316234588623
node_65: feature_name=ENSG00000033100.16	feature_id[561].value > threshold=94.66749572753906
Class: 1	
Rules_25	passed counts:2
node_0: feature_name=ENSG00000012048.22	feature_id[371].value <= threshold=13.43457317352295
node_1: feature_name=ENSG00000047578.13	feature_id[684].value <= threshold=53.576541900634766
node_2: feature_name=ENSG00000007952.18	feature_id[257].value <= threshold=0.4518554210662842
node_3: feature_name=ENSG00000026297.15	feature_id[517].value > threshold=274.33460998535156
node_9: feature_name=ENSG00000008277.14	feature_id[270].value <= threshold=4.677093505859375
node_10: feature_name=ENSG00000049192.15	feature_id[715].value <= threshold=2.364746928215027
node_11: feature_name=ENSG00000011638.10	feature_id[369].value <= threshold=10.54316234588623
node_12: feature_name=ENSG00000033627.16	feature_id[566].value <= threshold=148.1126251220703
node_13: feature_name=ENSG00000002746.15	feature_id[91].value <= threshold=0.17109555006027222
node_14: feature_name=ENSG00000033100.16	feature_id[561].value <= threshold=121.37638854980469
node_15: feature_name=ENSG00000025434.19	feature_id[508].value <= threshold=16.221430778503418

node_16: feature_name=ENSG00000028310.18	feature_id[533].value <= threshold=73.98308944702148
node_17: feature_name=ENSG00000031081.10	feature_id[550].value <= threshold=35.2840518951416
node_18: feature_name=ENSG00000035720.8	feature_id[585].value <= threshold=24.809341430664062
node_19: feature_name=ENSG00000016402.13	feature_id[430].value <= threshold=0.4444928616285324
node_20: feature_name=ENSG00000003137.8	feature_id[98].value <= threshold=9.946229934692383
node_21: feature_name=ENSG00000014216.15	feature_id[410].value > threshold=136.36983489990234
node_25: feature_name=ENSG00000039600.11	feature_id[623].value <= threshold=1.2662832140922546
node_26: feature_name=ENSG00000000460.17	feature_id[67].value <= threshold=6.493743658065796
node_27: feature_name=ENSG00000037757.14	feature_id[603].value <= threshold=63.50008010864258
node_28: feature_name=ENSG00000004846.16	feature_id[127].value <= threshold=0.09349484369158745
node_29: feature_name=ENSG00000041353.10	feature_id[635].value > threshold=2.9915499687194824
node_33: feature_name=ENSG00000005189.19	feature_id[149].value > threshold=2.6748963594436646
node_35: feature_name=ENSG00000047188.16	feature_id[676].value <= threshold=19.489907264709473
Class: 1	
Rules_26	passed counts:2
node_0: feature_name=ENSG00000012048.22	feature_id[371].value <= threshold=13.43457317352295
node_1: feature_name=ENSG00000047578.13	feature_id[684].value <= threshold=53.576541900634766
node_2: feature_name=ENSG00000007952.18	feature_id[257].value <= threshold=0.4518554210662842
node_3: feature_name=ENSG00000026297.15	feature_id[517].value > threshold=274.33460998535156
node_9: feature_name=ENSG00000008277.14	feature_id[270].value <= threshold=4.677093505859375
node_10: feature_name=ENSG00000049192.15	feature_id[715].value <= threshold=2.364746928215027
node_11: feature_name=ENSG00000011638.10	feature_id[369].value <= threshold=10.54316234588623
node_12: feature_name=ENSG00000033627.16	feature_id[566].value <= threshold=148.1126251220703
node_13: feature_name=ENSG00000002746.15	feature_id[91].value <= threshold=0.17109555006027222

node_14: feature_name=ENSG00000033100.16	feature_id[561].value <= threshold=121.37638854980469
node_15: feature_name=ENSG00000025434.19	feature_id[508].value <= threshold=16.221430778503418
node_16: feature_name=ENSG00000028310.18	feature_id[533].value <= threshold=73.98308944702148
node_17: feature_name=ENSG00000031081.10	feature_id[550].value <= threshold=35.2840518951416
node_18: feature_name=ENSG00000035720.8	feature_id[585].value <= threshold=24.809341430664062
node_19: feature_name=ENSG00000016402.13	feature_id[430].value <= threshold=0.4444928616285324
node_20: feature_name=ENSG00000003137.8	feature_id[98].value <= threshold=9.946229934692383
node_21: feature_name=ENSG00000014216.15	feature_id[410].value > threshold=136.36983489990234
node_25: feature_name=ENSG00000039600.11	feature_id[623].value <= threshold=1.2662832140922546
node_26: feature_name=ENSG00000000460.17	feature_id[67].value <= threshold=6.493743658065796
node_27: feature_name=ENSG00000037757.14	feature_id[603].value <= threshold=63.50008010864258
node_28: feature_name=ENSG00000004846.16	feature_id[127].value <= threshold=0.09349484369158745
node_29: feature_name=ENSG00000041353.10	feature_id[635].value <= threshold=2.9915499687194824
node_30: feature_name=ENSG00000005882.11	feature_id[172].value <= threshold=20.62182331085205
Class: 1	
Rules_27	passed counts:2
node_0: feature_name=ENSG00000012048.22	feature_id[371].value <= threshold=13.43457317352295
node_1: feature_name=ENSG00000047578.13	feature_id[684].value <= threshold=53.576541900634766
node_2: feature_name=ENSG00000007952.18	feature_id[257].value <= threshold=0.4518554210662842
node_3: feature_name=ENSG00000026297.15	feature_id[517].value > threshold=274.33460998535156
node_9: feature_name=ENSG00000008277.14	feature_id[270].value <= threshold=4.677093505859375
node_10: feature_name=ENSG00000049192.15	feature_id[715].value <= threshold=2.364746928215027
node_11: feature_name=ENSG00000011638.10	feature_id[369].value <= threshold=10.54316234588623
node_12: feature_name=ENSG00000033627.16	feature_id[566].value <= threshold=148.1126251220703

node_13: feature_name=ENSG00000002746.15	feature_id[91].value <= threshold=0.17109555006027222
node_14: feature_name=ENSG00000033100.16	feature_id[561].value <= threshold=121.37638854980469
node_15: feature_name=ENSG00000025434.19	feature_id[508].value <= threshold=16.221430778503418
node_16: feature_name=ENSG00000028310.18	feature_id[533].value <= threshold=73.98308944702148
node_17: feature_name=ENSG00000031081.10	feature_id[550].value <= threshold=35.2840518951416
node_18: feature_name=ENSG00000035720.8	feature_id[585].value <= threshold=24.809341430664062
node_19: feature_name=ENSG00000016402.13	feature_id[430].value <= threshold=0.4444928616285324
node_20: feature_name=ENSG00000003137.8	feature_id[98].value <= threshold=9.946229934692383
node_21: feature_name=ENSG00000014216.15	feature_id[410].value <= threshold=136.36983489990234
node_22: feature_name=ENSG00000009694.13	feature_id[296].value > threshold=1.6295068264007568
Class: 0	
Rules_28	passed counts:1
node_0: feature_name=ENSG00000012048.22	feature_id[371].value > threshold=13.43457317352295
node_72: feature_name=ENSG00000162594.15	feature_id[20].value > threshold=0.23572762310504913
node_88: feature_name=ENSG00000009950.16	feature_id[304].value <= threshold=0.2558544874191284
node_89: feature_name=ENSG00000051128.19	feature_id[750].value > threshold=43.44260597229004
Class: 0	
Rules_29	passed counts:1
node_0: feature_name=ENSG00000012048.22	feature_id[371].value > threshold=13.43457317352295
node_72: feature_name=ENSG00000162594.15	feature_id[20].value <= threshold=0.23572762310504913
node_73: feature_name=ENSG00000042317.17	feature_id[646].value > threshold=1.344666063785553
node_83: feature_name=ENSG00000269886.1	feature_id[18].value <= threshold=0.25252923369407654
node_84: feature_name=ENSG00000006638.11	feature_id[210].value > threshold=15.165156841278076

Class: 1	
Rules_30	passed counts:1
node_0: feature_name=ENSG00000012048.22	feature_id[371].value > threshold=13.43457317352295
node_72: feature_name=ENSG00000162594.15	feature_id[20].value <= threshold=0.23572762310504913
node_73: feature_name=ENSG00000042317.17	feature_id[646].value <= threshold=1.344666063785553
node_74: feature_name=ENSG00000042753.11	feature_id[650].value > threshold=41.77660369873047
node_80: feature_name=ENSG00000025772.8	feature_id[511].value > threshold=13.427343845367432
Class: 1	
Rules_31	passed counts:1
node_0: feature_name=ENSG00000012048.22	feature_id[371].value <= threshold=13.43457317352295
node_1: feature_name=ENSG00000047578.13	feature_id[684].value <= threshold=53.576541900634766
node_2: feature_name=ENSG00000007952.18	feature_id[257].value <= threshold=0.4518554210662842
node_3: feature_name=ENSG00000026297.15	feature_id[517].value > threshold=274.33460998535156
node_9: feature_name=ENSG00000008277.14	feature_id[270].value <= threshold=4.677093505859375
node_10: feature_name=ENSG00000049192.15	feature_id[715].value <= threshold=2.364746928215027
node_11: feature_name=ENSG00000011638.10	feature_id[369].value <= threshold=10.54316234588623
node_12: feature_name=ENSG00000033627.16	feature_id[566].value <= threshold=148.1126251220703
node_13: feature_name=ENSG00000002746.15	feature_id[91].value <= threshold=0.17109555006027222
node_14: feature_name=ENSG00000033100.16	feature_id[561].value > threshold=121.37638854980469
Class: 0	
Rules_32	passed counts:1
node_0: feature_name=ENSG00000012048.22	feature_id[371].value <= threshold=13.43457317352295

node_1: feature_name=ENSG00000047578.13	feature_id[684].value <= threshold=53.576541900634766
node_2: feature_name=ENSG00000007952.18	feature_id[257].value <= threshold=0.4518554210662842
node_3: feature_name=ENSG00000026297.15	feature_id[517].value > threshold=274.33460998535156
node_9: feature_name=ENSG00000008277.14	feature_id[270].value <= threshold=4.677093505859375
node_10: feature_name=ENSG00000049192.15	feature_id[715].value <= threshold=2.364746928215027
node_11: feature_name=ENSG00000011638.10	feature_id[369].value <= threshold=10.54316234588623
node_12: feature_name=ENSG00000033627.16	feature_id[566].value <= threshold=148.1126251220703
node_13: feature_name=ENSG00000002746.15	feature_id[91].value <= threshold=0.17109555006027222
node_14: feature_name=ENSG00000033100.16	feature_id[561].value <= threshold=121.37638854980469
node_15: feature_name=ENSG00000025434.19	feature_id[508].value > threshold=16.221430778503418
Class: 0	
Rules_33	passed counts:1
node_0: feature_name=ENSG00000012048.22	feature_id[371].value <= threshold=13.43457317352295
node_1: feature_name=ENSG00000047578.13	feature_id[684].value <= threshold=53.576541900634766
node_2: feature_name=ENSG00000007952.18	feature_id[257].value <= threshold=0.4518554210662842
node_3: feature_name=ENSG00000026297.15	feature_id[517].value > threshold=274.33460998535156
node_9: feature_name=ENSG00000008277.14	feature_id[270].value <= threshold=4.677093505859375
node_10: feature_name=ENSG00000049192.15	feature_id[715].value <= threshold=2.364746928215027
node_11: feature_name=ENSG00000011638.10	feature_id[369].value <= threshold=10.54316234588623
node_12: feature_name=ENSG00000033627.16	feature_id[566].value <= threshold=148.1126251220703
node_13: feature_name=ENSG00000002746.15	feature_id[91].value <= threshold=0.17109555006027222
node_14: feature_name=ENSG00000033100.16	feature_id[561].value <= threshold=121.37638854980469
node_15: feature_name=ENSG00000025434.19	feature_id[508].value <= threshold=16.221430778503418
node_16: feature_name=ENSG00000028310.18	feature_id[533].value > threshold=73.98308944702148

Class: 0	
Rules_34	passed counts:1
node_0: feature_name=ENSG00000012048.22	feature_id[371].value <= threshold=13.43457317352295
node_1: feature_name=ENSG00000047578.13	feature_id[684].value <= threshold=53.576541900634766
node_2: feature_name=ENSG00000007952.18	feature_id[257].value <= threshold=0.4518554210662842
node_3: feature_name=ENSG00000026297.15	feature_id[517].value > threshold=274.33460998535156
node_9: feature_name=ENSG00000008277.14	feature_id[270].value <= threshold=4.677093505859375
node_10: feature_name=ENSG00000049192.15	feature_id[715].value <= threshold=2.364746928215027
node_11: feature_name=ENSG00000011638.10	feature_id[369].value <= threshold=10.54316234588623
node_12: feature_name=ENSG00000033627.16	feature_id[566].value <= threshold=148.1126251220703
node_13: feature_name=ENSG00000002746.15	feature_id[91].value <= threshold=0.17109555006027222
node_14: feature_name=ENSG00000033100.16	feature_id[561].value <= threshold=121.37638854980469
node_15: feature_name=ENSG00000025434.19	feature_id[508].value <= threshold=16.221430778503418
node_16: feature_name=ENSG00000028310.18	feature_id[533].value <= threshold=73.98308944702148
node_17: feature_name=ENSG00000031081.10	feature_id[550].value > threshold=35.2840518951416
Class: 0	
Rules_35	passed counts:1
node_0: feature_name=ENSG00000012048.22	feature_id[371].value <= threshold=13.43457317352295
node_1: feature_name=ENSG00000047578.13	feature_id[684].value <= threshold=53.576541900634766
node_2: feature_name=ENSG00000007952.18	feature_id[257].value <= threshold=0.4518554210662842
node_3: feature_name=ENSG00000026297.15	feature_id[517].value > threshold=274.33460998535156
node_9: feature_name=ENSG00000008277.14	feature_id[270].value <= threshold=4.677093505859375
node_10: feature_name=ENSG00000049192.15	feature_id[715].value <= threshold=2.364746928215027

node_11: feature_name=ENSG00000011638.10	feature_id[369].value <= threshold=10.54316234588623
node_12: feature_name=ENSG00000033627.16	feature_id[566].value <= threshold=148.1126251220703
node_13: feature_name=ENSG00000002746.15	feature_id[91].value <= threshold=0.17109555006027222
node_14: feature_name=ENSG00000033100.16	feature_id[561].value <= threshold=121.37638854980469
node_15: feature_name=ENSG00000025434.19	feature_id[508].value <= threshold=16.221430778503418
node_16: feature_name=ENSG00000028310.18	feature_id[533].value <= threshold=73.98308944702148
node_17: feature_name=ENSG00000031081.10	feature_id[550].value <= threshold=35.2840518951416
node_18: feature_name=ENSG00000035720.8	feature_id[585].value > threshold=24.809341430664062
Class: 0	
Rules_36	passed counts:1
node_0: feature_name=ENSG00000012048.22	feature_id[371].value <= threshold=13.43457317352295
node_1: feature_name=ENSG00000047578.13	feature_id[684].value <= threshold=53.576541900634766
node_2: feature_name=ENSG00000007952.18	feature_id[257].value <= threshold=0.4518554210662842
node_3: feature_name=ENSG00000026297.15	feature_id[517].value > threshold=274.33460998535156
node_9: feature_name=ENSG00000008277.14	feature_id[270].value <= threshold=4.677093505859375
node_10: feature_name=ENSG00000049192.15	feature_id[715].value <= threshold=2.364746928215027
node_11: feature_name=ENSG00000011638.10	feature_id[369].value <= threshold=10.54316234588623
node_12: feature_name=ENSG00000033627.16	feature_id[566].value <= threshold=148.1126251220703
node_13: feature_name=ENSG00000002746.15	feature_id[91].value <= threshold=0.17109555006027222
node_14: feature_name=ENSG00000033100.16	feature_id[561].value <= threshold=121.37638854980469
node_15: feature_name=ENSG00000025434.19	feature_id[508].value <= threshold=16.221430778503418
node_16: feature_name=ENSG00000028310.18	feature_id[533].value <= threshold=73.98308944702148
node_17: feature_name=ENSG00000031081.10	feature_id[550].value <= threshold=35.2840518951416
node_18: feature_name=ENSG00000035720.8	feature_id[585].value <= threshold=24.809341430664062

node_19: feature_name=ENSG00000016402.13	feature_id[430].value > threshold=0.4444928616285324
Class: 0	
Rules_37	passed counts:1
node_0: feature_name=ENSG00000012048.22	feature_id[371].value <= threshold=13.43457317352295
node_1: feature_name=ENSG00000047578.13	feature_id[684].value <= threshold=53.576541900634766
node_2: feature_name=ENSG00000007952.18	feature_id[257].value <= threshold=0.4518554210662842
node_3: feature_name=ENSG00000026297.15	feature_id[517].value > threshold=274.33460998535156
node_9: feature_name=ENSG00000008277.14	feature_id[270].value <= threshold=4.677093505859375
node_10: feature_name=ENSG00000049192.15	feature_id[715].value <= threshold=2.364746928215027
node_11: feature_name=ENSG00000011638.10	feature_id[369].value <= threshold=10.54316234588623
node_12: feature_name=ENSG00000033627.16	feature_id[566].value <= threshold=148.1126251220703
node_13: feature_name=ENSG00000002746.15	feature_id[91].value <= threshold=0.17109555006027222
node_14: feature_name=ENSG00000033100.16	feature_id[561].value <= threshold=121.37638854980469
node_15: feature_name=ENSG00000025434.19	feature_id[508].value <= threshold=16.221430778503418
node_16: feature_name=ENSG00000028310.18	feature_id[533].value <= threshold=73.98308944702148
node_17: feature_name=ENSG00000031081.10	feature_id[550].value <= threshold=35.2840518951416
node_18: feature_name=ENSG00000035720.8	feature_id[585].value <= threshold=24.809341430664062
node_19: feature_name=ENSG00000016402.13	feature_id[430].value <= threshold=0.4444928616285324
node_20: feature_name=ENSG00000003137.8	feature_id[98].value > threshold=9.946229934692383
Class: 0	
Rules_38	passed counts:1
node_0: feature_name=ENSG00000012048.22	feature_id[371].value <= threshold=13.43457317352295
node_1: feature_name=ENSG00000047578.13	feature_id[684].value <= threshold=53.576541900634766

node_2: feature_name=ENSG00000007952.18	feature_id[257].value <= threshold=0.4518554210662842
node_3: feature_name=ENSG00000026297.15	feature_id[517].value > threshold=274.33460998535156
node_9: feature_name=ENSG00000008277.14	feature_id[270].value <= threshold=4.677093505859375
node_10: feature_name=ENSG00000049192.15	feature_id[715].value <= threshold=2.364746928215027
node_11: feature_name=ENSG00000011638.10	feature_id[369].value <= threshold=10.54316234588623
node_12: feature_name=ENSG00000033627.16	feature_id[566].value <= threshold=148.1126251220703
node_13: feature_name=ENSG00000002746.15	feature_id[91].value <= threshold=0.17109555006027222
node_14: feature_name=ENSG00000033100.16	feature_id[561].value <= threshold=121.37638854980469
node_15: feature_name=ENSG00000025434.19	feature_id[508].value <= threshold=16.221430778503418
node_16: feature_name=ENSG00000028310.18	feature_id[533].value <= threshold=73.98308944702148
node_17: feature_name=ENSG00000031081.10	feature_id[550].value <= threshold=35.2840518951416
node_18: feature_name=ENSG00000035720.8	feature_id[585].value <= threshold=24.809341430664062
node_19: feature_name=ENSG00000016402.13	feature_id[430].value <= threshold=0.4444928616285324
node_20: feature_name=ENSG00000003137.8	feature_id[98].value <= threshold=9.946229934692383
node_21: feature_name=ENSG00000014216.15	feature_id[410].value > threshold=136.36983489990234
node_25: feature_name=ENSG00000039600.11	feature_id[623].value > threshold=1.2662832140922546
node_49: feature_name=ENSG00000005801.18	feature_id[168].value > threshold=9.061001062393188
Class: 0	
Rules_39	passed counts:1
node_0: feature_name=ENSG00000012048.22	feature_id[371].value <= threshold=13.43457317352295
node_1: feature_name=ENSG00000047578.13	feature_id[684].value <= threshold=53.576541900634766
node_2: feature_name=ENSG00000007952.18	feature_id[257].value <= threshold=0.4518554210662842
node_3: feature_name=ENSG00000026297.15	feature_id[517].value > threshold=274.33460998535156
node_9: feature_name=ENSG00000008277.14	feature_id[270].value <= threshold=4.677093505859375

node_10: feature_name=ENSG00000049192.15	feature_id[715].value <= threshold=2.364746928215027
node_11: feature_name=ENSG00000011638.10	feature_id[369].value <= threshold=10.54316234588623
node_12: feature_name=ENSG00000033627.16	feature_id[566].value <= threshold=148.1126251220703
node_13: feature_name=ENSG00000002746.15	feature_id[91].value <= threshold=0.17109555006027222
node_14: feature_name=ENSG00000033100.16	feature_id[561].value <= threshold=121.37638854980469
node_15: feature_name=ENSG00000025434.19	feature_id[508].value <= threshold=16.221430778503418
node_16: feature_name=ENSG00000028310.18	feature_id[533].value <= threshold=73.98308944702148
node_17: feature_name=ENSG00000031081.10	feature_id[550].value <= threshold=35.2840518951416
node_18: feature_name=ENSG00000035720.8	feature_id[585].value <= threshold=24.809341430664062
node_19: feature_name=ENSG00000016402.13	feature_id[430].value <= threshold=0.4444928616285324
node_20: feature_name=ENSG00000003137.8	feature_id[98].value <= threshold=9.946229934692383
node_21: feature_name=ENSG00000014216.15	feature_id[410].value > threshold=136.36983489990234
node_25: feature_name=ENSG00000039600.11	feature_id[623].value > threshold=1.2662832140922546
node_49: feature_name=ENSG00000005801.18	feature_id[168].value <= threshold=9.061001062393188
Class: 1	
Rules_40	passed counts:1
node_0: feature_name=ENSG00000012048.22	feature_id[371].value <= threshold=13.43457317352295
node_1: feature_name=ENSG00000047578.13	feature_id[684].value <= threshold=53.576541900634766
node_2: feature_name=ENSG00000007952.18	feature_id[257].value <= threshold=0.4518554210662842
node_3: feature_name=ENSG00000026297.15	feature_id[517].value > threshold=274.33460998535156
node_9: feature_name=ENSG00000008277.14	feature_id[270].value <= threshold=4.677093505859375
node_10: feature_name=ENSG00000049192.15	feature_id[715].value <= threshold=2.364746928215027
node_11: feature_name=ENSG00000011638.10	feature_id[369].value <= threshold=10.54316234588623
node_12: feature_name=ENSG00000033627.16	feature_id[566].value <= threshold=148.1126251220703

node_13: feature_name=ENSG00000002746.15	feature_id[91].value <= threshold=0.17109555006027222
node_14: feature_name=ENSG00000033100.16	feature_id[561].value <= threshold=121.37638854980469
node_15: feature_name=ENSG00000025434.19	feature_id[508].value <= threshold=16.221430778503418
node_16: feature_name=ENSG00000028310.18	feature_id[533].value <= threshold=73.98308944702148
node_17: feature_name=ENSG00000031081.10	feature_id[550].value <= threshold=35.2840518951416
node_18: feature_name=ENSG00000035720.8	feature_id[585].value <= threshold=24.809341430664062
node_19: feature_name=ENSG00000016402.13	feature_id[430].value <= threshold=0.4444928616285324
node_20: feature_name=ENSG00000003137.8	feature_id[98].value <= threshold=9.946229934692383
node_21: feature_name=ENSG00000014216.15	feature_id[410].value > threshold=136.36983489990234
node_25: feature_name=ENSG00000039600.11	feature_id[623].value <= threshold=1.2662832140922546
node_26: feature_name=ENSG00000000460.17	feature_id[67].value > threshold=6.493743658065796
node_46: feature_name=ENSG00000007392.16	feature_id[246].value > threshold=86.06796264648438
Class: 1	
Rules_41	passed counts:1
node_0: feature_name=ENSG00000012048.22	feature_id[371].value <= threshold=13.43457317352295
node_1: feature_name=ENSG00000047578.13	feature_id[684].value <= threshold=53.576541900634766
node_2: feature_name=ENSG00000007952.18	feature_id[257].value <= threshold=0.4518554210662842
node_3: feature_name=ENSG00000026297.15	feature_id[517].value > threshold=274.33460998535156
node_9: feature_name=ENSG00000008277.14	feature_id[270].value <= threshold=4.677093505859375
node_10: feature_name=ENSG00000049192.15	feature_id[715].value <= threshold=2.364746928215027
node_11: feature_name=ENSG00000011638.10	feature_id[369].value <= threshold=10.54316234588623
node_12: feature_name=ENSG00000033627.16	feature_id[566].value <= threshold=148.1126251220703
node_13: feature_name=ENSG00000002746.15	feature_id[91].value <= threshold=0.17109555006027222
node_14: feature_name=ENSG00000033100.16	feature_id[561].value <= threshold=121.37638854980469

node_15: feature_name=ENSG00000025434.19	feature_id[508].value <= threshold=16.221430778503418
node_16: feature_name=ENSG00000028310.18	feature_id[533].value <= threshold=73.98308944702148
node_17: feature_name=ENSG00000031081.10	feature_id[550].value <= threshold=35.2840518951416
node_18: feature_name=ENSG00000035720.8	feature_id[585].value <= threshold=24.809341430664062
node_19: feature_name=ENSG00000016402.13	feature_id[430].value <= threshold=0.4444928616285324
node_20: feature_name=ENSG00000003137.8	feature_id[98].value <= threshold=9.946229934692383
node_21: feature_name=ENSG00000014216.15	feature_id[410].value > threshold=136.36983489990234
node_25: feature_name=ENSG00000039600.11	feature_id[623].value <= threshold=1.2662832140922546
node_26: feature_name=ENSG00000000460.17	feature_id[67].value > threshold=6.493743658065796
node_46: feature_name=ENSG00000007392.16	feature_id[246].value <= threshold=86.06796264648438
Class: 0	
Rules_42	passed counts:1
node_0: feature_name=ENSG00000012048.22	feature_id[371].value <= threshold=13.43457317352295
node_1: feature_name=ENSG00000047578.13	feature_id[684].value <= threshold=53.576541900634766
node_2: feature_name=ENSG00000007952.18	feature_id[257].value <= threshold=0.4518554210662842
node_3: feature_name=ENSG00000026297.15	feature_id[517].value > threshold=274.33460998535156
node_9: feature_name=ENSG00000008277.14	feature_id[270].value <= threshold=4.677093505859375
node_10: feature_name=ENSG00000049192.15	feature_id[715].value <= threshold=2.364746928215027
node_11: feature_name=ENSG00000011638.10	feature_id[369].value <= threshold=10.54316234588623
node_12: feature_name=ENSG00000033627.16	feature_id[566].value <= threshold=148.1126251220703
node_13: feature_name=ENSG00000002746.15	feature_id[91].value <= threshold=0.17109555006027222
node_14: feature_name=ENSG00000033100.16	feature_id[561].value <= threshold=121.37638854980469
node_15: feature_name=ENSG00000025434.19	feature_id[508].value <= threshold=16.221430778503418
node_16: feature_name=ENSG00000028310.18	feature_id[533].value <= threshold=73.98308944702148

node_17: feature_name=ENSG00000031081.10	feature_id[550].value <= threshold=35.2840518951416
node_18: feature_name=ENSG00000035720.8	feature_id[585].value <= threshold=24.809341430664062
node_19: feature_name=ENSG00000016402.13	feature_id[430].value <= threshold=0.4444928616285324
node_20: feature_name=ENSG00000003137.8	feature_id[98].value <= threshold=9.946229934692383
node_21: feature_name=ENSG00000014216.15	feature_id[410].value > threshold=136.36983489990234
node_25: feature_name=ENSG00000039600.11	feature_id[623].value <= threshold=1.2662832140922546
node_26: feature_name=ENSG00000000460.17	feature_id[67].value <= threshold=6.493743658065796
node_27: feature_name=ENSG00000037757.14	feature_id[603].value > threshold=63.50008010864258
node_43: feature_name=ENSG00000039523.20	feature_id[620].value > threshold=119.8829116821289
Class: 0	
Rules_43	passed counts:1
node_0: feature_name=ENSG00000012048.22	feature_id[371].value <= threshold=13.43457317352295
node_1: feature_name=ENSG00000047578.13	feature_id[684].value <= threshold=53.576541900634766
node_2: feature_name=ENSG00000007952.18	feature_id[257].value <= threshold=0.4518554210662842
node_3: feature_name=ENSG00000026297.15	feature_id[517].value > threshold=274.33460998535156
node_9: feature_name=ENSG00000008277.14	feature_id[270].value <= threshold=4.677093505859375
node_10: feature_name=ENSG00000049192.15	feature_id[715].value <= threshold=2.364746928215027
node_11: feature_name=ENSG00000011638.10	feature_id[369].value <= threshold=10.54316234588623
node_12: feature_name=ENSG00000033627.16	feature_id[566].value <= threshold=148.1126251220703
node_13: feature_name=ENSG00000002746.15	feature_id[91].value <= threshold=0.17109555006027222
node_14: feature_name=ENSG00000033100.16	feature_id[561].value <= threshold=121.37638854980469
node_15: feature_name=ENSG00000025434.19	feature_id[508].value <= threshold=16.221430778503418
node_16: feature_name=ENSG00000028310.18	feature_id[533].value <= threshold=73.98308944702148
node_17: feature_name=ENSG00000031081.10	feature_id[550].value <= threshold=35.2840518951416

node_18: feature_name=ENSG00000035720.8	feature_id[585].value <= threshold=24.809341430664062
node_19: feature_name=ENSG00000016402.13	feature_id[430].value <= threshold=0.4444928616285324
node_20: feature_name=ENSG00000003137.8	feature_id[98].value <= threshold=9.946229934692383
node_21: feature_name=ENSG00000014216.15	feature_id[410].value > threshold=136.36983489990234
node_25: feature_name=ENSG00000039600.11	feature_id[623].value <= threshold=1.2662832140922546
node_26: feature_name=ENSG00000000460.17	feature_id[67].value <= threshold=6.493743658065796
node_27: feature_name=ENSG00000037757.14	feature_id[603].value > threshold=63.50008010864258
node_43: feature_name=ENSG00000039523.20	feature_id[620].value <= threshold=119.8829116821289
Class: 1	
Rules_44	passed counts:1
node_0: feature_name=ENSG00000012048.22	feature_id[371].value <= threshold=13.43457317352295
node_1: feature_name=ENSG00000047578.13	feature_id[684].value <= threshold=53.576541900634766
node_2: feature_name=ENSG00000007952.18	feature_id[257].value <= threshold=0.4518554210662842
node_3: feature_name=ENSG00000026297.15	feature_id[517].value > threshold=274.33460998535156
node_9: feature_name=ENSG00000008277.14	feature_id[270].value <= threshold=4.677093505859375
node_10: feature_name=ENSG00000049192.15	feature_id[715].value <= threshold=2.364746928215027
node_11: feature_name=ENSG00000011638.10	feature_id[369].value <= threshold=10.54316234588623
node_12: feature_name=ENSG00000033627.16	feature_id[566].value <= threshold=148.1126251220703
node_13: feature_name=ENSG00000002746.15	feature_id[91].value <= threshold=0.17109555006027222
node_14: feature_name=ENSG00000033100.16	feature_id[561].value <= threshold=121.37638854980469
node_15: feature_name=ENSG00000025434.19	feature_id[508].value <= threshold=16.221430778503418
node_16: feature_name=ENSG00000028310.18	feature_id[533].value <= threshold=73.98308944702148
node_17: feature_name=ENSG00000031081.10	feature_id[550].value <= threshold=35.2840518951416
node_18: feature_name=ENSG00000035720.8	feature_id[585].value <= threshold=24.809341430664062

node_19: feature_name=ENSG00000016402.13	feature_id[430].value <= threshold=0.4444928616285324
node_20: feature_name=ENSG00000003137.8	feature_id[98].value <= threshold=9.946229934692383
node_21: feature_name=ENSG00000014216.15	feature_id[410].value > threshold=136.36983489990234
node_25: feature_name=ENSG00000039600.11	feature_id[623].value <= threshold=1.2662832140922546
node_26: feature_name=ENSG00000000460.17	feature_id[67].value <= threshold=6.493743658065796
node_27: feature_name=ENSG00000037757.14	feature_id[603].value <= threshold=63.50008010864258
node_28: feature_name=ENSG00000004846.16	feature_id[127].value > threshold=0.09349484369158745
node_38: feature_name=ENSG00000010327.10	feature_id[322].value > threshold=115.10892868041992
node_40: feature_name=ENSG00000018280.17	feature_id[440].value <= threshold=316.47174072265625
Class: 0	
Rules_45	passed counts:1
node_0: feature_name=ENSG00000012048.22	feature_id[371].value <= threshold=13.43457317352295
node_1: feature_name=ENSG00000047578.13	feature_id[684].value <= threshold=53.576541900634766
node_2: feature_name=ENSG00000007952.18	feature_id[257].value <= threshold=0.4518554210662842
node_3: feature_name=ENSG00000026297.15	feature_id[517].value > threshold=274.33460998535156
node_9: feature_name=ENSG00000008277.14	feature_id[270].value <= threshold=4.677093505859375
node_10: feature_name=ENSG00000049192.15	feature_id[715].value <= threshold=2.364746928215027
node_11: feature_name=ENSG00000011638.10	feature_id[369].value <= threshold=10.54316234588623
node_12: feature_name=ENSG00000033627.16	feature_id[566].value <= threshold=148.1126251220703
node_13: feature_name=ENSG00000002746.15	feature_id[91].value <= threshold=0.17109555006027222
node_14: feature_name=ENSG00000033100.16	feature_id[561].value <= threshold=121.37638854980469
node_15: feature_name=ENSG00000025434.19	feature_id[508].value <= threshold=16.221430778503418
node_16: feature_name=ENSG00000028310.18	feature_id[533].value <= threshold=73.98308944702148
node_17: feature_name=ENSG00000031081.10	feature_id[550].value <= threshold=35.2840518951416

node_18: feature_name=ENSG00000035720.8	feature_id[585].value <= threshold=24.809341430664062
node_19: feature_name=ENSG00000016402.13	feature_id[430].value <= threshold=0.4444928616285324
node_20: feature_name=ENSG00000003137.8	feature_id[98].value <= threshold=9.946229934692383
node_21: feature_name=ENSG00000014216.15	feature_id[410].value > threshold=136.36983489990234
node_25: feature_name=ENSG00000039600.11	feature_id[623].value <= threshold=1.2662832140922546
node_26: feature_name=ENSG00000000460.17	feature_id[67].value <= threshold=6.493743658065796
node_27: feature_name=ENSG00000037757.14	feature_id[603].value <= threshold=63.50008010864258
node_28: feature_name=ENSG00000004846.16	feature_id[127].value <= threshold=0.09349484369158745
node_29: feature_name=ENSG00000041353.10	feature_id[635].value > threshold=2.9915499687194824
node_33: feature_name=ENSG00000005189.19	feature_id[149].value > threshold=2.6748963594436646
node_35: feature_name=ENSG00000047188.16	feature_id[676].value > threshold=19.489907264709473
Class: 0	
Rules_46	passed counts:1
node_0: feature_name=ENSG00000012048.22	feature_id[371].value <= threshold=13.43457317352295
node_1: feature_name=ENSG00000047578.13	feature_id[684].value <= threshold=53.576541900634766
node_2: feature_name=ENSG00000007952.18	feature_id[257].value <= threshold=0.4518554210662842
node_3: feature_name=ENSG00000026297.15	feature_id[517].value > threshold=274.33460998535156
node_9: feature_name=ENSG00000008277.14	feature_id[270].value <= threshold=4.677093505859375
node_10: feature_name=ENSG00000049192.15	feature_id[715].value <= threshold=2.364746928215027
node_11: feature_name=ENSG00000011638.10	feature_id[369].value <= threshold=10.54316234588623
node_12: feature_name=ENSG00000033627.16	feature_id[566].value <= threshold=148.1126251220703
node_13: feature_name=ENSG00000002746.15	feature_id[91].value <= threshold=0.17109555006027222
node_14: feature_name=ENSG00000033100.16	feature_id[561].value <= threshold=121.37638854980469
node_15: feature_name=ENSG00000025434.19	feature_id[508].value <= threshold=16.221430778503418

node_16: feature_name=ENSG00000028310.18	feature_id[533].value <= threshold=73.98308944702148
node_17: feature_name=ENSG00000031081.10	feature_id[550].value <= threshold=35.2840518951416
node_18: feature_name=ENSG00000035720.8	feature_id[585].value <= threshold=24.809341430664062
node_19: feature_name=ENSG00000016402.13	feature_id[430].value <= threshold=0.4444928616285324
node_20: feature_name=ENSG00000003137.8	feature_id[98].value <= threshold=9.946229934692383
node_21: feature_name=ENSG00000014216.15	feature_id[410].value > threshold=136.36983489990234
node_25: feature_name=ENSG00000039600.11	feature_id[623].value <= threshold=1.2662832140922546
node_26: feature_name=ENSG00000000460.17	feature_id[67].value <= threshold=6.493743658065796
node_27: feature_name=ENSG00000037757.14	feature_id[603].value <= threshold=63.50008010864258
node_28: feature_name=ENSG00000004846.16	feature_id[127].value <= threshold=0.09349484369158745
node_29: feature_name=ENSG00000041353.10	feature_id[635].value <= threshold=2.9915499687194824
node_30: feature_name=ENSG00000005882.11	feature_id[172].value > threshold=20.62182331085205
Class: 0	
Rules_47	passed counts:1
node_0: feature_name=ENSG00000012048.22	feature_id[371].value <= threshold=13.43457317352295
node_1: feature_name=ENSG00000047578.13	feature_id[684].value <= threshold=53.576541900634766
node_2: feature_name=ENSG00000007952.18	feature_id[257].value <= threshold=0.4518554210662842
node_3: feature_name=ENSG00000026297.15	feature_id[517].value <= threshold=274.33460998535156
node_4: feature_name=ENSG00000025423.11	feature_id[507].value > threshold=0.0394274340942502
node_6: feature_name=ENSG00000043591.5	feature_id[661].value <= threshold=0.21197742223739624
Class: 0	

(2) Rules on list yielded by LightGBM

Rules_0	passed counts:142

node_0: feature_name=ENSG00000149798.5	feature_id[6].value > threshold=34.76214790344238
node_16: feature_name=ENSG00000101544.9	feature_id[7].value <= threshold=11.966409683227539
node_17: feature_name=ENSG00000250067.12	feature_id[37].value <= threshold=25.887118339538574
Class: 1	
Rules_1	passed counts:139
node_0: feature_name=ENSG00000149798.5	feature_id[6].value > threshold=34.76214790344238
node_16: feature_name=ENSG00000101544.9	feature_id[7].value > threshold=11.966409683227539
node_20: feature_name=ENSG00000105889.15	feature_id[35].value > threshold=0.6272584795951843
node_56: feature_name=ENSG00000171360.3	feature_id[32].value <= threshold=0.37142980098724365
node_57: feature_name=ENSG00000229007.1	feature_id[14].value <= threshold=0.3132975101470947
node_58: feature_name=ENSG00000165730.16	feature_id[41].value <= threshold=1.240545928478241
node_59: feature_name=ENSG00000196933.5	feature_id[17].value <= threshold=0.6400375366210938
Class: 1	
Rules_2	passed counts:49
node_0: feature_name=ENSG00000149798.5	feature_id[6].value > threshold=34.76214790344238
node_16: feature_name=ENSG00000101544.9	feature_id[7].value > threshold=11.966409683227539
node_20: feature_name=ENSG00000105889.15	feature_id[35].value <= threshold=0.6272584795951843
node_21: feature_name=ENSG00000259674.1	feature_id[8].value > threshold=0.18670745193958282
node_37: feature_name=ENSG00000104973.18	feature_id[2].value > threshold=162.33904266357422
node_43: feature_name=ENSG00000101544.9	feature_id[7].value > threshold=14.32283878326416
node_53: feature_name=ENSG00000226855.1	feature_id[39].value <= threshold=0.6071135699748993
Class: 1	

Rules_3	passed counts:35
node_0: feature_name=ENSG00000149798.5	feature_id[6].value > threshold=34.76214790344238
node_16: feature_name=ENSG00000101544.9	feature_id[7].value > threshold=11.966409683227539
node_20: feature_name=ENSG00000105889.15	feature_id[35].value > threshold=0.6272584795951843
node_56: feature_name=ENSG00000171360.3	feature_id[32].value <= threshold=0.37142980098724365
node_57: feature_name=ENSG00000229007.1	feature_id[14].value > threshold=0.3132975101470947
node_69: feature_name=ENSG00000125656.10	feature_id[27].value <= threshold=39.06645393371582
node_70: feature_name=ENSG00000260693.1	feature_id[13].value > threshold=0.7929590046405792
node_76: feature_name=ENSG00000210191.1	feature_id[16].value <= threshold=0.47084370255470276
Class: 1	
Rules_4	passed counts:25
node_0: feature_name=ENSG00000149798.5	feature_id[6].value <= threshold=34.76214790344238
node_1: feature_name=ENSG00000125656.10	feature_id[27].value <= threshold=38.85020446777344
node_2: feature_name=ENSG00000226855.1	feature_id[39].value <= threshold=0.12361128628253937
node_3: feature_name=ENSG00000267598.1	feature_id[21].value <= threshold=0.9463811218738556
node_4: feature_name=ENSG00000172425.10	feature_id[24].value <= threshold=0.32587555050849915
Class: 0	
Rules_5	passed counts:20
node_0: feature_name=ENSG00000149798.5	feature_id[6].value > threshold=34.76214790344238
node_16: feature_name=ENSG00000101544.9	feature_id[7].value > threshold=11.966409683227539
node_20: feature_name=ENSG00000105889.15	feature_id[35].value <= threshold=0.6272584795951843
node_21: feature_name=ENSG00000259674.1	feature_id[8].value > threshold=0.18670745193958282
node_37: feature_name=ENSG00000104973.18	feature_id[2].value > threshold=162.33904266357422

node_43: feature_name=ENSG00000101544.9	feature_id[7].value <= threshold=14.32283878326416
node_44: feature_name=ENSG00000137267.6	feature_id[43].value <= threshold=20.68922519683838
node_45: feature_name=ENSG00000267984.1	feature_id[28].value <= threshold=0.23421459645032883
node_46: feature_name=ENSG00000229007.1	feature_id[14].value <= threshold=0.4686327427625656
Class: 1	
Rules_6	passed counts:20
node_0: feature_name=ENSG00000149798.5	feature_id[6].value <= threshold=34.76214790344238
node_1: feature_name=ENSG00000125656.10	feature_id[27].value > threshold=38.85020446777344
Class: 1	
Rules_7	passed counts:14
node_0: feature_name=ENSG00000149798.5	feature_id[6].value <= threshold=34.76214790344238
node_1: feature_name=ENSG00000125656.10	feature_id[27].value <= threshold=38.85020446777344
node_2: feature_name=ENSG00000226855.1	feature_id[39].value > threshold=0.12361128628253937
node_10: feature_name=ENSG00000101544.9	feature_id[7].value <= threshold=19.272947311401367
Class: 1	
Rules_8	passed counts:12
node_0: feature_name=ENSG00000149798.5	feature_id[6].value > threshold=34.76214790344238
node_16: feature_name=ENSG00000101544.9	feature_id[7].value > threshold=11.966409683227539
node_20: feature_name=ENSG00000105889.15	feature_id[35].value <= threshold=0.6272584795951843
node_21: feature_name=ENSG00000259674.1	feature_id[8].value <= threshold=0.18670745193958282
node_22: feature_name=ENSG00000022556.16	feature_id[19].value <= threshold=14.754841327667236
node_23: feature_name=ENSG00000270157.1	feature_id[20].value <= threshold=0.4186003506183624

node_24: feature_name=ENSG00000229007.1	feature_id[14].value <= threshold=0.4799928367137909
Class: 1	
Rules_9	passed counts:11
node_0: feature_name=ENSG00000149798.5	feature_id[6].value > threshold=34.76214790344238
node_16: feature_name=ENSG00000101544.9	feature_id[7].value > threshold=11.966409683227539
node_20: feature_name=ENSG00000105889.15	feature_id[35].value > threshold=0.6272584795951843
node_56: feature_name=ENSG00000171360.3	feature_id[32].value <= threshold=0.37142980098724365
node_57: feature_name=ENSG00000229007.1	feature_id[14].value <= threshold=0.3132975101470947
node_58: feature_name=ENSG00000165730.16	feature_id[41].value > threshold=1.240545928478241
node_64: feature_name=ENSG00000259515.1	feature_id[1].value > threshold=0.14896193519234657
node_66: feature_name=ENSG00000244734.4	feature_id[29].value > threshold=341.78221130371094
Class: 1	
Rules_10	passed counts:10
node_0: feature_name=ENSG00000149798.5	feature_id[6].value > threshold=34.76214790344238
node_16: feature_name=ENSG00000101544.9	feature_id[7].value > threshold=11.966409683227539
node_20: feature_name=ENSG00000105889.15	feature_id[35].value > threshold=0.6272584795951843
node_56: feature_name=ENSG00000171360.3	feature_id[32].value <= threshold=0.37142980098724365
node_57: feature_name=ENSG00000229007.1	feature_id[14].value > threshold=0.3132975101470947
node_69: feature_name=ENSG00000125656.10	feature_id[27].value <= threshold=39.06645393371582
node_70: feature_name=ENSG00000260693.1	feature_id[13].value <= threshold=0.7929590046405792
node_71: feature_name=ENSG00000211689.7	feature_id[23].value <= threshold=12.181947231292725
Class: 1	

Rules_11	passed counts:9
node_0: feature_name=ENSG00000149798.5	feature_id[6].value > threshold=34.76214790344238
node_16: feature_name=ENSG00000101544.9	feature_id[7].value > threshold=11.966409683227539
node_20: feature_name=ENSG00000105889.15	feature_id[35].value <= threshold=0.6272584795951843
node_21: feature_name=ENSG00000259674.1	feature_id[8].value <= threshold=0.18670745193958282
node_22: feature_name=ENSG00000022556.16	feature_id[19].value <= threshold=14.754841327667236
node_23: feature_name=ENSG00000270157.1	feature_id[20].value > threshold=0.4186003506183624
node_29: feature_name=ENSG00000137267.6	feature_id[43].value > threshold=3.641284704208374
node_33: feature_name=ENSG00000022556.16	feature_id[19].value > threshold=3.750759482383728
Class: 0	
Rules_12	passed counts:8
node_0: feature_name=ENSG00000149798.5	feature_id[6].value > threshold=34.76214790344238
node_16: feature_name=ENSG00000101544.9	feature_id[7].value > threshold=11.966409683227539
node_20: feature_name=ENSG00000105889.15	feature_id[35].value > threshold=0.6272584795951843
node_56: feature_name=ENSG00000171360.3	feature_id[32].value <= threshold=0.37142980098724365
node_57: feature_name=ENSG00000229007.1	feature_id[14].value <= threshold=0.3132975101470947
node_58: feature_name=ENSG00000165730.16	feature_id[41].value <= threshold=1.240545928478241
node_59: feature_name=ENSG00000196933.5	feature_id[17].value > threshold=0.6400375366210938
node_61: feature_name=ENSG00000164308.16	feature_id[33].value > threshold=59.54672622680664
Class: 1	
Rules_13	passed counts:7
node_0: feature_name=ENSG00000149798.5	feature_id[6].value > threshold=34.76214790344238
node_16: feature_name=ENSG00000101544.9	feature_id[7].value > threshold=11.966409683227539

node_20: feature_name=ENSG00000105889.15	feature_id[35].value <= threshold=0.6272584795951843
node_21: feature_name=ENSG00000259674.1	feature_id[8].value > threshold=0.18670745193958282
node_37: feature_name=ENSG00000104973.18	feature_id[2].value <= threshold=162.33904266357422
node_38: feature_name=ENSG00000165730.16	feature_id[41].value > threshold=0.12023531645536423
node_40: feature_name=ENSG00000054179.12	feature_id[36].value <= threshold=0.4876798391342163
Class: 0	
Rules_14	passed counts:7
node_0: feature_name=ENSG00000149798.5	feature_id[6].value > threshold=34.76214790344238
node_16: feature_name=ENSG00000101544.9	feature_id[7].value > threshold=11.966409683227539
node_20: feature_name=ENSG00000105889.15	feature_id[35].value <= threshold=0.6272584795951843
node_21: feature_name=ENSG00000259674.1	feature_id[8].value <= threshold=0.18670745193958282
node_22: feature_name=ENSG00000022556.16	feature_id[19].value > threshold=14.754841327667236
Class: 0	
Rules_15	passed counts:6
node_0: feature_name=ENSG00000149798.5	feature_id[6].value > threshold=34.76214790344238
node_16: feature_name=ENSG00000101544.9	feature_id[7].value > threshold=11.966409683227539
node_20: feature_name=ENSG00000105889.15	feature_id[35].value <= threshold=0.6272584795951843
node_21: feature_name=ENSG00000259674.1	feature_id[8].value > threshold=0.18670745193958282
node_37: feature_name=ENSG00000104973.18	feature_id[2].value <= threshold=162.33904266357422
node_38: feature_name=ENSG00000165730.16	feature_id[41].value <= threshold=0.12023531645536423
Class: 1	
Rules_16	passed counts:6

node_0: feature_name=ENSG00000149798.5	feature_id[6].value <= threshold=34.76214790344238
node_1: feature_name=ENSG00000125656.10	feature_id[27].value <= threshold=38.85020446777344
node_2: feature_name=ENSG00000226855.1	feature_id[39].value <= threshold=0.12361128628253937
node_3: feature_name=ENSG00000267598.1	feature_id[21].value > threshold=0.9463811218738556
node_7: feature_name=ENSG00000239975.2	feature_id[31].value <= threshold=0.4101337790489197
Class: 1	
Rules_17	passed counts:5
node_0: feature_name=ENSG00000149798.5	feature_id[6].value > threshold=34.76214790344238
node_16: feature_name=ENSG00000101544.9	feature_id[7].value > threshold=11.966409683227539
node_20: feature_name=ENSG00000105889.15	feature_id[35].value > threshold=0.6272584795951843
node_56: feature_name=ENSG00000171360.3	feature_id[32].value <= threshold=0.37142980098724365
node_57: feature_name=ENSG00000229007.1	feature_id[14].value > threshold=0.3132975101470947
node_69: feature_name=ENSG00000125656.10	feature_id[27].value > threshold=39.06645393371582
node_79: feature_name=ENSG00000150045.12	feature_id[42].value > threshold=19.185542583465576
Class: 0	
Rules_18	passed counts:5
node_0: feature_name=ENSG00000149798.5	feature_id[6].value > threshold=34.76214790344238
node_16: feature_name=ENSG00000101544.9	feature_id[7].value > threshold=11.966409683227539
node_20: feature_name=ENSG00000105889.15	feature_id[35].value > threshold=0.6272584795951843
node_56: feature_name=ENSG00000171360.3	feature_id[32].value <= threshold=0.37142980098724365
node_57: feature_name=ENSG00000229007.1	feature_id[14].value > threshold=0.3132975101470947
node_69: feature_name=ENSG00000125656.10	feature_id[27].value > threshold=39.06645393371582
node_79: feature_name=ENSG00000150045.12	feature_id[42].value <= threshold=19.185542583465576

node_80: feature_name=ENSG00000105889.15	feature_id[35].value <= threshold=2.010471761226654
Class: 1	
Rules_19	passed counts:5
node_0: feature_name=ENSG00000149798.5	feature_id[6].value > threshold=34.76214790344238
node_16: feature_name=ENSG00000101544.9	feature_id[7].value > threshold=11.966409683227539
node_20: feature_name=ENSG00000105889.15	feature_id[35].value > threshold=0.6272584795951843
node_56: feature_name=ENSG00000171360.3	feature_id[32].value <= threshold=0.37142980098724365
node_57: feature_name=ENSG00000229007.1	feature_id[14].value > threshold=0.3132975101470947
node_69: feature_name=ENSG00000125656.10	feature_id[27].value <= threshold=39.06645393371582
node_70: feature_name=ENSG00000260693.1	feature_id[13].value <= threshold=0.7929590046405792
node_71: feature_name=ENSG00000211689.7	feature_id[23].value > threshold=12.181947231292725
node_73: feature_name=ENSG00000149798.5	feature_id[6].value <= threshold=87.111083984375
Class: 0	
Rules_20	passed counts:5
node_0: feature_name=ENSG00000149798.5	feature_id[6].value > threshold=34.76214790344238
node_16: feature_name=ENSG00000101544.9	feature_id[7].value > threshold=11.966409683227539
node_20: feature_name=ENSG00000105889.15	feature_id[35].value <= threshold=0.6272584795951843
node_21: feature_name=ENSG00000259674.1	feature_id[8].value <= threshold=0.18670745193958282
node_22: feature_name=ENSG00000022556.16	feature_id[19].value <= threshold=14.754841327667236
node_23: feature_name=ENSG00000270157.1	feature_id[20].value > threshold=0.4186003506183624
node_29: feature_name=ENSG00000137267.6	feature_id[43].value <= threshold=3.641284704208374
node_30: feature_name=ENSG00000171360.3	feature_id[32].value <= threshold=0.12518571689724922
Class: 1	

Rules_21	passed counts:3
node_0: feature_name=ENSG00000149798.5	feature_id[6].value > threshold=34.76214790344238
node_16: feature_name=ENSG00000101544.9	feature_id[7].value > threshold=11.966409683227539
node_20: feature_name=ENSG00000105889.15	feature_id[35].value <= threshold=0.6272584795951843
node_21: feature_name=ENSG00000259674.1	feature_id[8].value > threshold=0.18670745193958282
node_37: feature_name=ENSG00000104973.18	feature_id[2].value > threshold=162.33904266357422
node_43: feature_name=ENSG00000101544.9	feature_id[7].value <= threshold=14.32283878326416
node_44: feature_name=ENSG00000137267.6	feature_id[43].value > threshold=20.68922519683838
Class: 0	
Rules_22	passed counts:3
node_0: feature_name=ENSG00000149798.5	feature_id[6].value > threshold=34.76214790344238
node_16: feature_name=ENSG00000101544.9	feature_id[7].value > threshold=11.966409683227539
node_20: feature_name=ENSG00000105889.15	feature_id[35].value <= threshold=0.6272584795951843
node_21: feature_name=ENSG00000259674.1	feature_id[8].value > threshold=0.18670745193958282
node_37: feature_name=ENSG00000104973.18	feature_id[2].value > threshold=162.33904266357422
node_43: feature_name=ENSG00000101544.9	feature_id[7].value <= threshold=14.32283878326416
node_44: feature_name=ENSG00000137267.6	feature_id[43].value <= threshold=20.68922519683838
node_45: feature_name=ENSG00000267984.1	feature_id[28].value > threshold=0.23421459645032883
node_49: feature_name=ENSG00000158710.14	feature_id[26].value <= threshold=1072.6016845703125
Class: 0	
Rules_23	passed counts:3
node_0: feature_name=ENSG00000149798.5	feature_id[6].value <= threshold=34.76214790344238

node_1: feature_name=ENSG00000125656.10	feature_id[27].value <= threshold=38.85020446777344
node_2: feature_name=ENSG00000226855.1	feature_id[39].value > threshold=0.12361128628253937
node_10: feature_name=ENSG00000101544.9	feature_id[7].value > threshold=19.272947311401367
node_12: feature_name=ENSG00000250067.12	feature_id[37].value <= threshold=15.292912483215332
Class: 0	
Rules_24	passed counts:3
node_0: feature_name=ENSG00000149798.5	feature_id[6].value <= threshold=34.76214790344238
node_1: feature_name=ENSG00000125656.10	feature_id[27].value <= threshold=38.85020446777344
node_2: feature_name=ENSG00000226855.1	feature_id[39].value <= threshold=0.12361128628253937
node_3: feature_name=ENSG00000267598.1	feature_id[21].value > threshold=0.9463811218738556
node_7: feature_name=ENSG00000239975.2	feature_id[31].value > threshold=0.4101337790489197
Class: 0	
Rules_25	passed counts:2
node_0: feature_name=ENSG00000149798.5	feature_id[6].value > threshold=34.76214790344238
node_16: feature_name=ENSG00000101544.9	feature_id[7].value > threshold=11.966409683227539
node_20: feature_name=ENSG00000105889.15	feature_id[35].value > threshold=0.6272584795951843
node_56: feature_name=ENSG00000171360.3	feature_id[32].value > threshold=0.37142980098724365
Class: 0	
Rules_26	passed counts:2
node_0: feature_name=ENSG00000149798.5	feature_id[6].value > threshold=34.76214790344238
node_16: feature_name=ENSG00000101544.9	feature_id[7].value > threshold=11.966409683227539
node_20: feature_name=ENSG00000105889.15	feature_id[35].value > threshold=0.6272584795951843

node_56: feature_name=ENSG00000171360.3	feature_id[32].value <= threshold=0.37142980098724365
node_57: feature_name=ENSG00000229007.1	feature_id[14].value <= threshold=0.3132975101470947
node_58: feature_name=ENSG00000165730.16	feature_id[41].value > threshold=1.240545928478241
node_64: feature_name=ENSG00000259515.1	feature_id[1].value <= threshold=0.14896193519234657
Class: 0	
Rules_27	passed counts:2
node_0: feature_name=ENSG00000149798.5	feature_id[6].value > threshold=34.76214790344238
node_16: feature_name=ENSG00000101544.9	feature_id[7].value > threshold=11.966409683227539
node_20: feature_name=ENSG00000105889.15	feature_id[35].value <= threshold=0.6272584795951843
node_21: feature_name=ENSG00000259674.1	feature_id[8].value > threshold=0.18670745193958282
node_37: feature_name=ENSG00000104973.18	feature_id[2].value > threshold=162.33904266357422
node_43: feature_name=ENSG00000101544.9	feature_id[7].value <= threshold=14.32283878326416
node_44: feature_name=ENSG00000137267.6	feature_id[43].value <= threshold=20.68922519683838
node_45: feature_name=ENSG00000267984.1	feature_id[28].value > threshold=0.23421459645032883
node_49: feature_name=ENSG00000158710.14	feature_id[26].value > threshold=1072.6016845703125
Class: 1	
Rules_28	passed counts:2
node_0: feature_name=ENSG00000149798.5	feature_id[6].value > threshold=34.76214790344238
node_16: feature_name=ENSG00000101544.9	feature_id[7].value > threshold=11.966409683227539
node_20: feature_name=ENSG00000105889.15	feature_id[35].value <= threshold=0.6272584795951843
node_21: feature_name=ENSG00000259674.1	feature_id[8].value > threshold=0.18670745193958282
node_37: feature_name=ENSG00000104973.18	feature_id[2].value <= threshold=162.33904266357422
node_38: feature_name=ENSG00000165730.16	feature_id[41].value > threshold=0.12023531645536423

node_40: feature_name=ENSG00000054179.12	feature_id[36].value > threshold=0.4876798391342163
Class: 1	
Rules_29	passed counts:2
node_0: feature_name=ENSG00000149798.5	feature_id[6].value > threshold=34.76214790344238
node_16: feature_name=ENSG00000101544.9	feature_id[7].value > threshold=11.966409683227539
node_20: feature_name=ENSG00000105889.15	feature_id[35].value <= threshold=0.6272584795951843
node_21: feature_name=ENSG00000259674.1	feature_id[8].value <= threshold=0.18670745193958282
node_22: feature_name=ENSG00000022556.16	feature_id[19].value <= threshold=14.754841327667236
node_23: feature_name=ENSG00000270157.1	feature_id[20].value > threshold=0.4186003506183624
node_29: feature_name=ENSG00000137267.6	feature_id[43].value > threshold=3.641284704208374
node_33: feature_name=ENSG00000022556.16	feature_id[19].value <= threshold=3.750759482383728
Class: 1	
Rules_30	passed counts:2
node_0: feature_name=ENSG00000149798.5	feature_id[6].value > threshold=34.76214790344238
node_16: feature_name=ENSG00000101544.9	feature_id[7].value > threshold=11.966409683227539
node_20: feature_name=ENSG00000105889.15	feature_id[35].value <= threshold=0.6272584795951843
node_21: feature_name=ENSG00000259674.1	feature_id[8].value <= threshold=0.18670745193958282
node_22: feature_name=ENSG00000022556.16	feature_id[19].value <= threshold=14.754841327667236
node_23: feature_name=ENSG00000270157.1	feature_id[20].value <= threshold=0.4186003506183624
node_24: feature_name=ENSG00000229007.1	feature_id[14].value > threshold=0.4799928367137909
node_26: feature_name=ENSG00000158710.14	feature_id[26].value > threshold=674.1937255859375
Class: 0	

Rules_31	passed counts:2
node_0: feature_name=ENSG00000149798.5	feature_id[6].value <= threshold=34.76214790344238
node_1: feature_name=ENSG00000125656.10	feature_id[27].value <= threshold=38.85020446777344
node_2: feature_name=ENSG00000226855.1	feature_id[39].value <= threshold=0.12361128628253937
node_3: feature_name=ENSG00000267598.1	feature_id[21].value <= threshold=0.9463811218738556
node_4: feature_name=ENSG00000172425.10	feature_id[24].value > threshold=0.32587555050849915
Class: 1	
Rules_32	passed counts:1
node_0: feature_name=ENSG00000149798.5	feature_id[6].value > threshold=34.76214790344238
node_16: feature_name=ENSG00000101544.9	feature_id[7].value > threshold=11.966409683227539
node_20: feature_name=ENSG00000105889.15	feature_id[35].value > threshold=0.6272584795951843
node_56: feature_name=ENSG00000171360.3	feature_id[32].value <= threshold=0.37142980098724365
node_57: feature_name=ENSG00000229007.1	feature_id[14].value > threshold=0.3132975101470947
node_69: feature_name=ENSG00000125656.10	feature_id[27].value > threshold=39.06645393371582
node_79: feature_name=ENSG00000150045.12	feature_id[42].value <= threshold=19.185542583465576
node_80: feature_name=ENSG00000105889.15	feature_id[35].value > threshold=2.010471761226654
Class: 0	
Rules_33	passed counts:1
node_0: feature_name=ENSG00000149798.5	feature_id[6].value > threshold=34.76214790344238
node_16: feature_name=ENSG00000101544.9	feature_id[7].value > threshold=11.966409683227539
node_20: feature_name=ENSG00000105889.15	feature_id[35].value > threshold=0.6272584795951843
node_56: feature_name=ENSG00000171360.3	feature_id[32].value <= threshold=0.37142980098724365
node_57: feature_name=ENSG00000229007.1	feature_id[14].value > threshold=0.3132975101470947

node_69: feature_name=ENSG00000125656.10	feature_id[27].value <= threshold=39.06645393371582
node_70: feature_name=ENSG00000260693.1	feature_id[13].value > threshold=0.7929590046405792
node_76: feature_name=ENSG00000210191.1	feature_id[16].value > threshold=0.47084370255470276
Class: 0	
Rules_34	passed counts:1
node_0: feature_name=ENSG00000149798.5	feature_id[6].value > threshold=34.76214790344238
node_16: feature_name=ENSG00000101544.9	feature_id[7].value > threshold=11.966409683227539
node_20: feature_name=ENSG00000105889.15	feature_id[35].value > threshold=0.6272584795951843
node_56: feature_name=ENSG00000171360.3	feature_id[32].value <= threshold=0.37142980098724365
node_57: feature_name=ENSG00000229007.1	feature_id[14].value > threshold=0.3132975101470947
node_69: feature_name=ENSG00000125656.10	feature_id[27].value <= threshold=39.06645393371582
node_70: feature_name=ENSG00000260693.1	feature_id[13].value <= threshold=0.7929590046405792
node_71: feature_name=ENSG00000211689.7	feature_id[23].value > threshold=12.181947231292725
node_73: feature_name=ENSG00000149798.5	feature_id[6].value > threshold=87.111083984375
Class: 1	
Rules_35	passed counts:1
node_0: feature_name=ENSG00000149798.5	feature_id[6].value > threshold=34.76214790344238
node_16: feature_name=ENSG00000101544.9	feature_id[7].value > threshold=11.966409683227539
node_20: feature_name=ENSG00000105889.15	feature_id[35].value > threshold=0.6272584795951843
node_56: feature_name=ENSG00000171360.3	feature_id[32].value <= threshold=0.37142980098724365
node_57: feature_name=ENSG00000229007.1	feature_id[14].value <= threshold=0.3132975101470947
node_58: feature_name=ENSG00000165730.16	feature_id[41].value > threshold=1.240545928478241
node_64: feature_name=ENSG00000259515.1	feature_id[1].value > threshold=0.14896193519234657

node_66: feature_name=ENSG00000244734.4	feature_id[29].value <= threshold=341.78221130371094
Class: 0	
Rules_36	passed counts:1
node_0: feature_name=ENSG00000149798.5	feature_id[6].value > threshold=34.76214790344238
node_16: feature_name=ENSG00000101544.9	feature_id[7].value > threshold=11.966409683227539
node_20: feature_name=ENSG00000105889.15	feature_id[35].value > threshold=0.6272584795951843
node_56: feature_name=ENSG00000171360.3	feature_id[32].value <= threshold=0.37142980098724365
node_57: feature_name=ENSG00000229007.1	feature_id[14].value <= threshold=0.3132975101470947
node_58: feature_name=ENSG00000165730.16	feature_id[41].value <= threshold=1.240545928478241
node_59: feature_name=ENSG00000196933.5	feature_id[17].value > threshold=0.6400375366210938
node_61: feature_name=ENSG00000164308.16	feature_id[33].value <= threshold=59.54672622680664
Class: 0	
Rules_37	passed counts:1
node_0: feature_name=ENSG00000149798.5	feature_id[6].value > threshold=34.76214790344238
node_16: feature_name=ENSG00000101544.9	feature_id[7].value > threshold=11.966409683227539
node_20: feature_name=ENSG00000105889.15	feature_id[35].value <= threshold=0.6272584795951843
node_21: feature_name=ENSG00000259674.1	feature_id[8].value > threshold=0.18670745193958282
node_37: feature_name=ENSG00000104973.18	feature_id[2].value > threshold=162.33904266357422
node_43: feature_name=ENSG00000101544.9	feature_id[7].value > threshold=14.32283878326416
node_53: feature_name=ENSG00000226855.1	feature_id[39].value > threshold=0.6071135699748993
Class: 0	
Rules_38	passed counts:1

node_0: feature_name=ENSG00000149798.5	feature_id[6].value > threshold=34.76214790344238
node_16: feature_name=ENSG00000101544.9	feature_id[7].value > threshold=11.966409683227539
node_20: feature_name=ENSG00000105889.15	feature_id[35].value <= threshold=0.6272584795951843
node_21: feature_name=ENSG00000259674.1	feature_id[8].value > threshold=0.18670745193958282
node_37: feature_name=ENSG00000104973.18	feature_id[2].value > threshold=162.33904266357422
node_43: feature_name=ENSG00000101544.9	feature_id[7].value <= threshold=14.32283878326416
node_44: feature_name=ENSG00000137267.6	feature_id[43].value <= threshold=20.68922519683838
node_45: feature_name=ENSG00000267984.1	feature_id[28].value <= threshold=0.23421459645032883
node_46: feature_name=ENSG00000229007.1	feature_id[14].value > threshold=0.4686327427625656
Class: 0	
Rules_39	passed counts:1
node_0: feature_name=ENSG00000149798.5	feature_id[6].value > threshold=34.76214790344238
node_16: feature_name=ENSG00000101544.9	feature_id[7].value > threshold=11.966409683227539
node_20: feature_name=ENSG00000105889.15	feature_id[35].value <= threshold=0.6272584795951843
node_21: feature_name=ENSG00000259674.1	feature_id[8].value <= threshold=0.18670745193958282
node_22: feature_name=ENSG00000022556.16	feature_id[19].value <= threshold=14.754841327667236
node_23: feature_name=ENSG00000270157.1	feature_id[20].value > threshold=0.4186003506183624
node_29: feature_name=ENSG00000137267.6	feature_id[43].value <= threshold=3.641284704208374
node_30: feature_name=ENSG00000171360.3	feature_id[32].value > threshold=0.12518571689724922
Class: 0	
Rules_40	passed counts:1
node_0: feature_name=ENSG00000149798.5	feature_id[6].value > threshold=34.76214790344238
node_16: feature_name=ENSG00000101544.9	feature_id[7].value > threshold=11.966409683227539

node_20: feature_name=ENSG00000105889.15	feature_id[35].value <= threshold=0.6272584795951843
node_21: feature_name=ENSG00000259674.1	feature_id[8].value <= threshold=0.18670745193958282
node_22: feature_name=ENSG00000022556.16	feature_id[19].value <= threshold=14.754841327667236
node_23: feature_name=ENSG00000270157.1	feature_id[20].value <= threshold=0.4186003506183624
node_24: feature_name=ENSG00000229007.1	feature_id[14].value > threshold=0.4799928367137909
node_26: feature_name=ENSG00000158710.14	feature_id[26].value <= threshold=674.1937255859375
Class: 1	
Rules_41	passed counts:1
node_0: feature_name=ENSG00000149798.5	feature_id[6].value > threshold=34.76214790344238
node_16: feature_name=ENSG00000101544.9	feature_id[7].value <= threshold=11.966409683227539
node_17: feature_name=ENSG00000250067.12	feature_id[37].value > threshold=25.887118339538574
Class: 0	
Rules_42	passed counts:1
node_0: feature_name=ENSG00000149798.5	feature_id[6].value <= threshold=34.76214790344238
node_1: feature_name=ENSG00000125656.10	feature_id[27].value <= threshold=38.85020446777344
node_2: feature_name=ENSG00000226855.1	feature_id[39].value > threshold=0.12361128628253937
node_10: feature_name=ENSG00000101544.9	feature_id[7].value > threshold=19.272947311401367
node_12: feature_name=ENSG00000250067.12	feature_id[37].value > threshold=15.292912483215332
Class: 1	

(3) Rules on list yielded by MCFS

Rules_0	passed counts:347
node_0: feature_name=ENSG00000149798.5	feature_id[1].value > threshold=34.76214790344238

node_20: feature_name=ENSG00000163346.17	feature_id[70].value > threshold=237.0936508178711
node_34: feature_name=ENSG00000277288.4	feature_id[30].value <= threshold=0.08194983750581741
node_35: feature_name=ENSG00000258763.5	feature_id[31].value <= threshold=0.7273186147212982
node_36: feature_name=ENSG00000254990.5	feature_id[17].value <= threshold=0.1868050992488861
node_37: feature_name=ENSG00000257951.2	feature_id[58].value <= threshold=0.04973463900387287
node_38: feature_name=ENSG00000130522.5	feature_id[10].value > threshold=156.51981353759766
node_42: feature_name=ENSG00000099250.18	feature_id[42].value <= threshold=4.655871629714966
node_43: feature_name=ENSG00000241935.9	feature_id[48].value <= threshold=0.2611011117696762
node_44: feature_name=ENSG00000012048.22	feature_id[14].value <= threshold=19.74557590484619
node_45: feature_name=ENSG00000173239.13	feature_id[60].value <= threshold=0.22336432337760925
node_46: feature_name=ENSG00000239590.1	feature_id[71].value <= threshold=0.1118115596473217
node_47: feature_name=ENSG00000233242.2	feature_id[51].value <= threshold=0.0636320523917675
node_48: feature_name=ENSG00000198901.14	feature_id[69].value > threshold=0.39943213760852814
node_52: feature_name=ENSG00000171360.3	feature_id[0].value <= threshold=0.37142980098724365
node_53: feature_name=ENSG00000273776.1	feature_id[67].value <= threshold=0.11133104935288429
node_54: feature_name=ENSG00000255356.2	feature_id[63].value <= threshold=0.04708113335072994
node_55: feature_name=ENSG00000260231.2	feature_id[49].value > threshold=6.197221994400024
Class: 1	
Rules_1	passed counts:28
node_0: feature_name=ENSG00000149798.5	feature_id[1].value > threshold=34.76214790344238
node_20: feature_name=ENSG00000163346.17	feature_id[70].value <= threshold=237.0936508178711
node_21: feature_name=ENSG00000099250.18	feature_id[42].value <= threshold=1.921088457107544
node_22: feature_name=ENSG00000201988.2	feature_id[37].value <= threshold=0.06192629598081112
node_23: feature_name=ENSG00000258763.5	feature_id[31].value <= threshold=0.13121316581964493

node_24: feature_name=ENSG00000261402.1	feature_id[12].value <= threshold=0.026882583275437355
node_25: feature_name=ENSG00000264204.2	feature_id[9].value > threshold=1.6955653429031372
Class: 1	
Rules_2	passed counts:25
node_0: feature_name=ENSG00000149798.5	feature_id[1].value <= threshold=34.76214790344238
node_1: feature_name=ENSG00000073737.16	feature_id[34].value <= threshold=56.694297790527344
node_2: feature_name=ENSG00000238243.3	feature_id[59].value <= threshold=3.4281924962997437
node_3: feature_name=ENSG00000155307.18	feature_id[43].value <= threshold=19.13234519958496
Class: 1	
Rules_3	passed counts:14
node_0: feature_name=ENSG00000149798.5	feature_id[1].value > threshold=34.76214790344238
node_20: feature_name=ENSG00000163346.17	feature_id[70].value > threshold=237.0936508178711
node_34: feature_name=ENSG00000277288.4	feature_id[30].value <= threshold=0.08194983750581741
node_35: feature_name=ENSG00000258763.5	feature_id[31].value <= threshold=0.7273186147212982
node_36: feature_name=ENSG00000254990.5	feature_id[17].value <= threshold=0.1868050992488861
node_37: feature_name=ENSG00000257951.2	feature_id[58].value <= threshold=0.04973463900387287
node_38: feature_name=ENSG00000130522.5	feature_id[10].value > threshold=156.51981353759766
node_42: feature_name=ENSG00000099250.18	feature_id[42].value <= threshold=4.655871629714966
node_43: feature_name=ENSG00000241935.9	feature_id[48].value <= threshold=0.2611011117696762
node_44: feature_name=ENSG00000012048.22	feature_id[14].value <= threshold=19.74557590484619
node_45: feature_name=ENSG00000173239.13	feature_id[60].value <= threshold=0.22336432337760925
node_46: feature_name=ENSG00000239590.1	feature_id[71].value <= threshold=0.1118115596473217
node_47: feature_name=ENSG00000233242.2	feature_id[51].value <= threshold=0.0636320523917675

node_48: feature_name=ENSG00000198901.14	feature_id[69].value > threshold=0.39943213760852814
node_52: feature_name=ENSG00000171360.3	feature_id[0].value <= threshold=0.37142980098724365
node_53: feature_name=ENSG00000273776.1	feature_id[67].value <= threshold=0.11133104935288429
node_54: feature_name=ENSG00000255356.2	feature_id[63].value <= threshold=0.04708113335072994
node_55: feature_name=ENSG00000260231.2	feature_id[49].value <= threshold=6.197221994400024
node_56: feature_name=ENSG00000260231.2	feature_id[49].value <= threshold=6.1267735958099365
Class: 1	
Rules_4	passed counts:13
node_0: feature_name=ENSG00000149798.5	feature_id[1].value <= threshold=34.76214790344238
node_1: feature_name=ENSG00000073737.16	feature_id[34].value <= threshold=56.694297790527344
node_2: feature_name=ENSG00000238243.3	feature_id[59].value > threshold=3.4281924962997437
node_10: feature_name=ENSG00000103381.12	feature_id[73].value > threshold=217.66256713867188
node_16: feature_name=ENSG00000158710.14	feature_id[8].value <= threshold=886.7076416015625
Class: 0	
Rules_5	passed counts:12
node_0: feature_name=ENSG00000149798.5	feature_id[1].value > threshold=34.76214790344238
node_20: feature_name=ENSG00000163346.17	feature_id[70].value > threshold=237.0936508178711
node_34: feature_name=ENSG00000277288.4	feature_id[30].value <= threshold=0.08194983750581741
node_35: feature_name=ENSG00000258763.5	feature_id[31].value <= threshold=0.7273186147212982
node_36: feature_name=ENSG00000254990.5	feature_id[17].value <= threshold=0.1868050992488861
node_37: feature_name=ENSG00000257951.2	feature_id[58].value <= threshold=0.04973463900387287
node_38: feature_name=ENSG00000130522.5	feature_id[10].value > threshold=156.51981353759766
node_42: feature_name=ENSG00000099250.18	feature_id[42].value <= threshold=4.655871629714966

node_43: feature_name=ENSG00000241935.9	feature_id[48].value <= threshold=0.2611011117696762
node_44: feature_name=ENSG00000012048.22	feature_id[14].value <= threshold=19.74557590484619
node_45: feature_name=ENSG00000173239.13	feature_id[60].value <= threshold=0.22336432337760925
node_46: feature_name=ENSG00000239590.1	feature_id[71].value > threshold=0.1118115596473217
node_74: feature_name=ENSG00000158710.14	feature_id[8].value > threshold=756.7427062988281
node_76: feature_name=ENSG00000185551.15	feature_id[16].value <= threshold=0.10993906855583191
Class: 1	
Rules_6	passed counts:11
node_0: feature_name=ENSG00000149798.5	feature_id[1].value > threshold=34.76214790344238
node_20: feature_name=ENSG00000163346.17	feature_id[70].value > threshold=237.0936508178711
node_34: feature_name=ENSG00000277288.4	feature_id[30].value <= threshold=0.08194983750581741
node_35: feature_name=ENSG00000258763.5	feature_id[31].value <= threshold=0.7273186147212982
node_36: feature_name=ENSG00000254990.5	feature_id[17].value <= threshold=0.1868050992488861
node_37: feature_name=ENSG00000257951.2	feature_id[58].value <= threshold=0.04973463900387287
node_38: feature_name=ENSG00000130522.5	feature_id[10].value > threshold=156.51981353759766
node_42: feature_name=ENSG00000099250.18	feature_id[42].value <= threshold=4.655871629714966
node_43: feature_name=ENSG00000241935.9	feature_id[48].value <= threshold=0.2611011117696762
node_44: feature_name=ENSG00000012048.22	feature_id[14].value <= threshold=19.74557590484619
node_45: feature_name=ENSG00000173239.13	feature_id[60].value <= threshold=0.22336432337760925
node_46: feature_name=ENSG00000239590.1	feature_id[71].value <= threshold=0.1118115596473217
node_47: feature_name=ENSG00000233242.2	feature_id[51].value <= threshold=0.0636320523917675
node_48: feature_name=ENSG00000198901.14	feature_id[69].value > threshold=0.39943213760852814
node_52: feature_name=ENSG00000171360.3	feature_id[0].value <= threshold=0.37142980098724365
node_53: feature_name=ENSG00000273776.1	feature_id[67].value <= threshold=0.11133104935288429

node_54: feature_name=ENSG00000255356.2	feature_id[63].value > threshold=0.04708113335072994
node_60: feature_name=ENSG00000189430.12	feature_id[53].value <= threshold=9.082543849945068
Class: 1	
Rules_7	passed counts:9
node_0: feature_name=ENSG00000149798.5	feature_id[1].value > threshold=34.76214790344238
node_20: feature_name=ENSG00000163346.17	feature_id[70].value > threshold=237.0936508178711
node_34: feature_name=ENSG00000277288.4	feature_id[30].value <= threshold=0.08194983750581741
node_35: feature_name=ENSG00000258763.5	feature_id[31].value <= threshold=0.7273186147212982
node_36: feature_name=ENSG00000254990.5	feature_id[17].value <= threshold=0.1868050992488861
node_37: feature_name=ENSG00000257951.2	feature_id[58].value > threshold=0.04973463900387287
node_83: feature_name=ENSG00000224328.1	feature_id[18].value <= threshold=0.12363792955875397
node_84: feature_name=ENSG00000225611.1	feature_id[4].value <= threshold=4.301597833633423
Class: 1	
Rules_8	passed counts:9
node_0: feature_name=ENSG00000149798.5	feature_id[1].value <= threshold=34.76214790344238
node_1: feature_name=ENSG00000073737.16	feature_id[34].value > threshold=56.694297790527344
Class: 0	
Rules_9	passed counts:9
node_0: feature_name=ENSG00000149798.5	feature_id[1].value <= threshold=34.76214790344238
node_1: feature_name=ENSG00000073737.16	feature_id[34].value <= threshold=56.694297790527344
node_2: feature_name=ENSG00000238243.3	feature_id[59].value > threshold=3.4281924962997437
node_10: feature_name=ENSG00000103381.12	feature_id[73].value <= threshold=217.66256713867188

node_11: feature_name=ENSG00000104973.18	feature_id[11].value > threshold=80.44169235229492
node_13: feature_name=ENSG00000180304.14	feature_id[56].value <= threshold=127.41180419921875
Class: 1	
Rules_10	passed counts:7
node_0: feature_name=ENSG00000149798.5	feature_id[1].value > threshold=34.76214790344238
node_20: feature_name=ENSG00000163346.17	feature_id[70].value <= threshold=237.0936508178711
node_21: feature_name=ENSG00000099250.18	feature_id[42].value > threshold=1.921088457107544
node_31: feature_name=ENSG00000264204.2	feature_id[9].value > threshold=5.726377010345459
Class: 0	
Rules_11	passed counts:6
node_0: feature_name=ENSG00000149798.5	feature_id[1].value > threshold=34.76214790344238
node_20: feature_name=ENSG00000163346.17	feature_id[70].value > threshold=237.0936508178711
node_34: feature_name=ENSG00000277288.4	feature_id[30].value > threshold=0.08194983750581741
node_96: feature_name=ENSG00000198963.11	feature_id[65].value > threshold=0.05539559945464134
Class: 0	
Rules_12	passed counts:6
node_0: feature_name=ENSG00000149798.5	feature_id[1].value > threshold=34.76214790344238
node_20: feature_name=ENSG00000163346.17	feature_id[70].value > threshold=237.0936508178711
node_34: feature_name=ENSG00000277288.4	feature_id[30].value > threshold=0.08194983750581741
node_96: feature_name=ENSG00000198963.11	feature_id[65].value <= threshold=0.05539559945464134
node_97: feature_name=ENSG00000180304.14	feature_id[56].value > threshold=124.1164779663086
Class: 1	

Rules_13	passed counts:6
node_0: feature_name=ENSG00000149798.5	feature_id[1].value > threshold=34.76214790344238
node_20: feature_name=ENSG00000163346.17	feature_id[70].value > threshold=237.0936508178711
node_34: feature_name=ENSG00000277288.4	feature_id[30].value <= threshold=0.08194983750581741
node_35: feature_name=ENSG00000258763.5	feature_id[31].value <= threshold=0.7273186147212982
node_36: feature_name=ENSG00000254990.5	feature_id[17].value <= threshold=0.1868050992488861
node_37: feature_name=ENSG00000257951.2	feature_id[58].value <= threshold=0.04973463900387287
node_38: feature_name=ENSG00000130522.5	feature_id[10].value > threshold=156.51981353759766
node_42: feature_name=ENSG00000099250.18	feature_id[42].value <= threshold=4.655871629714966
node_43: feature_name=ENSG00000241935.9	feature_id[48].value <= threshold=0.2611011117696762
node_44: feature_name=ENSG00000012048.22	feature_id[14].value <= threshold=19.74557590484619
node_45: feature_name=ENSG00000173239.13	feature_id[60].value <= threshold=0.22336432337760925
node_46: feature_name=ENSG00000239590.1	feature_id[71].value <= threshold=0.1118115596473217
node_47: feature_name=ENSG00000233242.2	feature_id[51].value > threshold=0.0636320523917675
node_69: feature_name=ENSG00000233242.2	feature_id[51].value > threshold=0.06934208422899246
Class: 1	
Rules_14	passed counts:5
node_0: feature_name=ENSG00000149798.5	feature_id[1].value <= threshold=34.76214790344238
node_1: feature_name=ENSG00000073737.16	feature_id[34].value <= threshold=56.694297790527344
node_2: feature_name=ENSG00000238243.3	feature_id[59].value <= threshold=3.4281924962997437
node_3: feature_name=ENSG00000155307.18	feature_id[43].value > threshold=19.13234519958496
node_5: feature_name=ENSG00000165406.16	feature_id[25].value > threshold=104.79007339477539
Class: 0	

Rules_15	passed counts:5
node_0: feature_name=ENSG00000149798.5	feature_id[1].value <= threshold=34.76214790344238
node_1: feature_name=ENSG00000073737.16	feature_id[34].value <= threshold=56.694297790527344
node_2: feature_name=ENSG00000238243.3	feature_id[59].value <= threshold=3.4281924962997437
node_3: feature_name=ENSG00000155307.18	feature_id[43].value > threshold=19.13234519958496
node_5: feature_name=ENSG00000165406.16	feature_id[25].value <= threshold=104.79007339477539
node_6: feature_name=ENSG00000158710.14	feature_id[8].value > threshold=572.8588562011719
Class: 1	
Rules_16	passed counts:4
node_0: feature_name=ENSG00000149798.5	feature_id[1].value > threshold=34.76214790344238
node_20: feature_name=ENSG00000163346.17	feature_id[70].value > threshold=237.0936508178711
node_34: feature_name=ENSG00000277288.4	feature_id[30].value <= threshold=0.08194983750581741
node_35: feature_name=ENSG00000258763.5	feature_id[31].value <= threshold=0.7273186147212982
node_36: feature_name=ENSG00000254990.5	feature_id[17].value <= threshold=0.1868050992488861
node_37: feature_name=ENSG00000257951.2	feature_id[58].value <= threshold=0.04973463900387287
node_38: feature_name=ENSG00000130522.5	feature_id[10].value <= threshold=156.51981353759766
node_39: feature_name=ENSG00000198963.11	feature_id[65].value <= threshold=0.013976069167256355
Class: 1	
Rules_17	passed counts:4
node_0: feature_name=ENSG00000149798.5	feature_id[1].value > threshold=34.76214790344238
node_20: feature_name=ENSG00000163346.17	feature_id[70].value <= threshold=237.0936508178711
node_21: feature_name=ENSG00000099250.18	feature_id[42].value <= threshold=1.921088457107544

node_22: feature_name=ENSG00000201988.2	feature_id[37].value > threshold=0.0619262959808112
Class: 0	
Rules_18	passed counts:4
node_0: feature_name=ENSG00000149798.5	feature_id[1].value <= threshold=34.76214790344238
node_1: feature_name=ENSG00000073737.16	feature_id[34].value <= threshold=56.694297790527344
node_2: feature_name=ENSG00000238243.3	feature_id[59].value > threshold=3.4281924962997437
node_10: feature_name=ENSG00000103381.12	feature_id[73].value > threshold=217.66256713867188
node_16: feature_name=ENSG00000158710.14	feature_id[8].value > threshold=886.7076416015625
Class: 1	
Rules_19	passed counts:3
node_0: feature_name=ENSG00000149798.5	feature_id[1].value > threshold=34.76214790344238
node_20: feature_name=ENSG00000163346.17	feature_id[70].value > threshold=237.0936508178711
node_34: feature_name=ENSG00000277288.4	feature_id[30].value <= threshold=0.08194983750581741
node_35: feature_name=ENSG00000258763.5	feature_id[31].value > threshold=0.7273186147212982
node_93: feature_name=ENSG00000012048.22	feature_id[14].value > threshold=9.985532760620117
Class: 0	
Rules_20	passed counts:3
node_0: feature_name=ENSG00000149798.5	feature_id[1].value > threshold=34.76214790344238
node_20: feature_name=ENSG00000163346.17	feature_id[70].value > threshold=237.0936508178711
node_34: feature_name=ENSG00000277288.4	feature_id[30].value <= threshold=0.08194983750581741
node_35: feature_name=ENSG00000258763.5	feature_id[31].value <= threshold=0.7273186147212982
node_36: feature_name=ENSG00000254990.5	feature_id[17].value > threshold=0.1868050992488861

node_90: feature_name=ENSG00000026950.17	feature_id[27].value > threshold=348.0993347167969
Class: 1	
Rules_21	passed counts:3
node_0: feature_name=ENSG00000149798.5	feature_id[1].value > threshold=34.76214790344238
node_20: feature_name=ENSG00000163346.17	feature_id[70].value > threshold=237.0936508178711
node_34: feature_name=ENSG00000277288.4	feature_id[30].value <= threshold=0.08194983750581741
node_35: feature_name=ENSG00000258763.5	feature_id[31].value <= threshold=0.7273186147212982
node_36: feature_name=ENSG00000254990.5	feature_id[17].value > threshold=0.1868050992488861
node_90: feature_name=ENSG00000026950.17	feature_id[27].value <= threshold=348.0993347167969
Class: 0	
Rules_22	passed counts:3
node_0: feature_name=ENSG00000149798.5	feature_id[1].value > threshold=34.76214790344238
node_20: feature_name=ENSG00000163346.17	feature_id[70].value > threshold=237.0936508178711
node_34: feature_name=ENSG00000277288.4	feature_id[30].value <= threshold=0.08194983750581741
node_35: feature_name=ENSG00000258763.5	feature_id[31].value <= threshold=0.7273186147212982
node_36: feature_name=ENSG00000254990.5	feature_id[17].value <= threshold=0.1868050992488861
node_37: feature_name=ENSG00000257951.2	feature_id[58].value > threshold=0.04973463900387287
node_83: feature_name=ENSG00000224328.1	feature_id[18].value > threshold=0.12363792955875397
Class: 0	
Rules_23	passed counts:3
node_0: feature_name=ENSG00000149798.5	feature_id[1].value > threshold=34.76214790344238
node_20: feature_name=ENSG00000163346.17	feature_id[70].value > threshold=237.0936508178711

node_34: feature_name=ENSG00000277288.4	feature_id[30].value <= threshold=0.08194983750581741
node_35: feature_name=ENSG00000258763.5	feature_id[31].value <= threshold=0.7273186147212982
node_36: feature_name=ENSG00000254990.5	feature_id[17].value <= threshold=0.1868050992488861
node_37: feature_name=ENSG00000257951.2	feature_id[58].value <= threshold=0.04973463900387287
node_38: feature_name=ENSG00000130522.5	feature_id[10].value > threshold=156.51981353759766
node_42: feature_name=ENSG00000099250.18	feature_id[42].value <= threshold=4.655871629714966
node_43: feature_name=ENSG00000241935.9	feature_id[48].value <= threshold=0.2611011117696762
node_44: feature_name=ENSG00000012048.22	feature_id[14].value <= threshold=19.74557590484619
node_45: feature_name=ENSG00000173239.13	feature_id[60].value <= threshold=0.22336432337760925
node_46: feature_name=ENSG00000239590.1	feature_id[71].value > threshold=0.1118115596473217
node_74: feature_name=ENSG00000158710.14	feature_id[8].value <= threshold=756.7427062988281
Class: 0	
Rules_24	passed counts:3
node_0: feature_name=ENSG00000149798.5	feature_id[1].value > threshold=34.76214790344238
node_20: feature_name=ENSG00000163346.17	feature_id[70].value > threshold=237.0936508178711
node_34: feature_name=ENSG00000277288.4	feature_id[30].value <= threshold=0.08194983750581741
node_35: feature_name=ENSG00000258763.5	feature_id[31].value <= threshold=0.7273186147212982
node_36: feature_name=ENSG00000254990.5	feature_id[17].value <= threshold=0.1868050992488861
node_37: feature_name=ENSG00000257951.2	feature_id[58].value <= threshold=0.04973463900387287
node_38: feature_name=ENSG00000130522.5	feature_id[10].value > threshold=156.51981353759766
node_42: feature_name=ENSG00000099250.18	feature_id[42].value <= threshold=4.655871629714966
node_43: feature_name=ENSG00000241935.9	feature_id[48].value <= threshold=0.2611011117696762
node_44: feature_name=ENSG00000012048.22	feature_id[14].value <= threshold=19.74557590484619
node_45: feature_name=ENSG00000173239.13	feature_id[60].value <= threshold=0.22336432337760925

node_46: feature_name=ENSG00000239590.1	feature_id[71].value <= threshold=0.1118115596473217
node_47: feature_name=ENSG00000233242.2	feature_id[51].value > threshold=0.0636320523917675
node_69: feature_name=ENSG00000233242.2	feature_id[51].value <= threshold=0.06934208422899246
node_70: feature_name=ENSG00000182809.10	feature_id[29].value <= threshold=21.95294761657715
Class: 0	
Rules_25	passed counts:3
node_0: feature_name=ENSG00000149798.5	feature_id[1].value > threshold=34.76214790344238
node_20: feature_name=ENSG00000163346.17	feature_id[70].value > threshold=237.0936508178711
node_34: feature_name=ENSG00000277288.4	feature_id[30].value <= threshold=0.08194983750581741
node_35: feature_name=ENSG00000258763.5	feature_id[31].value <= threshold=0.7273186147212982
node_36: feature_name=ENSG00000254990.5	feature_id[17].value <= threshold=0.1868050992488861
node_37: feature_name=ENSG00000257951.2	feature_id[58].value <= threshold=0.04973463900387287
node_38: feature_name=ENSG00000130522.5	feature_id[10].value > threshold=156.51981353759766
node_42: feature_name=ENSG00000099250.18	feature_id[42].value <= threshold=4.655871629714966
node_43: feature_name=ENSG00000241935.9	feature_id[48].value <= threshold=0.2611011117696762
node_44: feature_name=ENSG00000012048.22	feature_id[14].value <= threshold=19.74557590484619
node_45: feature_name=ENSG00000173239.13	feature_id[60].value <= threshold=0.22336432337760925
node_46: feature_name=ENSG00000239590.1	feature_id[71].value <= threshold=0.1118115596473217
node_47: feature_name=ENSG00000233242.2	feature_id[51].value <= threshold=0.0636320523917675
node_48: feature_name=ENSG00000198901.14	feature_id[69].value > threshold=0.39943213760852814
node_52: feature_name=ENSG00000171360.3	feature_id[0].value <= threshold=0.37142980098724365
node_53: feature_name=ENSG00000273776.1	feature_id[67].value > threshold=0.11133104935288429
node_63: feature_name=ENSG00000240403.5	feature_id[40].value <= threshold=3.764121174812317
Class: 1	

Rules_26	passed counts:3
node_0: feature_name=ENSG00000149798.5	feature_id[1].value > threshold=34.76214790344238
node_20: feature_name=ENSG00000163346.17	feature_id[70].value > threshold=237.0936508178711
node_34: feature_name=ENSG00000277288.4	feature_id[30].value <= threshold=0.08194983750581741
node_35: feature_name=ENSG00000258763.5	feature_id[31].value <= threshold=0.7273186147212982
node_36: feature_name=ENSG00000254990.5	feature_id[17].value <= threshold=0.1868050992488861
node_37: feature_name=ENSG00000257951.2	feature_id[58].value <= threshold=0.04973463900387287
node_38: feature_name=ENSG00000130522.5	feature_id[10].value <= threshold=156.51981353759766
node_39: feature_name=ENSG00000198963.11	feature_id[65].value > threshold=0.013976069167256355
Class: 0	
Rules_27	passed counts:2
node_0: feature_name=ENSG00000149798.5	feature_id[1].value > threshold=34.76214790344238
node_20: feature_name=ENSG00000163346.17	feature_id[70].value > threshold=237.0936508178711
node_34: feature_name=ENSG00000277288.4	feature_id[30].value <= threshold=0.08194983750581741
node_35: feature_name=ENSG00000258763.5	feature_id[31].value > threshold=0.7273186147212982
node_93: feature_name=ENSG00000012048.22	feature_id[14].value <= threshold=9.985532760620117
Class: 1	
Rules_28	passed counts:2
node_0: feature_name=ENSG00000149798.5	feature_id[1].value > threshold=34.76214790344238
node_20: feature_name=ENSG00000163346.17	feature_id[70].value > threshold=237.0936508178711
node_34: feature_name=ENSG00000277288.4	feature_id[30].value <= threshold=0.08194983750581741
node_35: feature_name=ENSG00000258763.5	feature_id[31].value <= threshold=0.7273186147212982

node_36: feature_name=ENSG00000254990.5	feature_id[17].value <= threshold=0.1868050992488861
node_37: feature_name=ENSG00000257951.2	feature_id[58].value > threshold=0.04973463900387287
node_83: feature_name=ENSG00000224328.1	feature_id[18].value <= threshold=0.12363792955875397
node_84: feature_name=ENSG00000225611.1	feature_id[4].value > threshold=4.301597833633423
node_86: feature_name=ENSG00000163346.17	feature_id[70].value <= threshold=469.8916015625
Class: 0	
Rules_29	passed counts:2
node_0: feature_name=ENSG00000149798.5	feature_id[1].value > threshold=34.76214790344238
node_20: feature_name=ENSG00000163346.17	feature_id[70].value <= threshold=237.0936508178711
node_21: feature_name=ENSG00000099250.18	feature_id[42].value <= threshold=1.921088457107544
node_22: feature_name=ENSG00000201988.2	feature_id[37].value <= threshold=0.06192629598081112
node_23: feature_name=ENSG00000258763.5	feature_id[31].value > threshold=0.13121316581964493
Class: 0	
Rules_30	passed counts:2
node_0: feature_name=ENSG00000149798.5	feature_id[1].value > threshold=34.76214790344238
node_20: feature_name=ENSG00000163346.17	feature_id[70].value <= threshold=237.0936508178711
node_21: feature_name=ENSG00000099250.18	feature_id[42].value <= threshold=1.921088457107544
node_22: feature_name=ENSG00000201988.2	feature_id[37].value <= threshold=0.06192629598081112
node_23: feature_name=ENSG00000258763.5	feature_id[31].value <= threshold=0.13121316581964493
node_24: feature_name=ENSG00000261402.1	feature_id[12].value > threshold=0.026882583275437355
Class: 0	
Rules_31	passed counts:2

node_0: feature_name=ENSG00000149798.5	feature_id[1].value <= threshold=34.76214790344238
node_1: feature_name=ENSG00000073737.16	feature_id[34].value <= threshold=56.694297790527344
node_2: feature_name=ENSG00000238243.3	feature_id[59].value > threshold=3.4281924962997437
node_10: feature_name=ENSG00000103381.12	feature_id[73].value <= threshold=217.66256713867188
node_11: feature_name=ENSG00000104973.18	feature_id[11].value <= threshold=80.44169235229492
Class: 0	
Rules_32	passed counts:1
node_0: feature_name=ENSG00000149798.5	feature_id[1].value > threshold=34.76214790344238
node_20: feature_name=ENSG00000163346.17	feature_id[70].value > threshold=237.0936508178711
node_34: feature_name=ENSG00000277288.4	feature_id[30].value > threshold=0.08194983750581741
node_96: feature_name=ENSG00000198963.11	feature_id[65].value <= threshold=0.05539559945464134
node_97: feature_name=ENSG00000180304.14	feature_id[56].value <= threshold=124.1164779663086
Class: 0	
Rules_33	passed counts:1
node_0: feature_name=ENSG00000149798.5	feature_id[1].value > threshold=34.76214790344238
node_20: feature_name=ENSG00000163346.17	feature_id[70].value > threshold=237.0936508178711
node_34: feature_name=ENSG00000277288.4	feature_id[30].value <= threshold=0.08194983750581741
node_35: feature_name=ENSG00000258763.5	feature_id[31].value <= threshold=0.7273186147212982
node_36: feature_name=ENSG00000254990.5	feature_id[17].value <= threshold=0.1868050992488861
node_37: feature_name=ENSG00000257951.2	feature_id[58].value > threshold=0.04973463900387287
node_83: feature_name=ENSG00000224328.1	feature_id[18].value <= threshold=0.12363792955875397
node_84: feature_name=ENSG00000225611.1	feature_id[4].value > threshold=4.301597833633423
node_86: feature_name=ENSG00000163346.17	feature_id[70].value > threshold=469.8916015625

Class: 1	
Rules_34	passed counts:1
node_0: feature_name=ENSG00000149798.5	feature_id[1].value > threshold=34.76214790344238
node_20: feature_name=ENSG00000163346.17	feature_id[70].value > threshold=237.0936508178711
node_34: feature_name=ENSG00000277288.4	feature_id[30].value <= threshold=0.08194983750581741
node_35: feature_name=ENSG00000258763.5	feature_id[31].value <= threshold=0.7273186147212982
node_36: feature_name=ENSG00000254990.5	feature_id[17].value <= threshold=0.1868050992488861
node_37: feature_name=ENSG00000257951.2	feature_id[58].value <= threshold=0.04973463900387287
node_38: feature_name=ENSG00000130522.5	feature_id[10].value > threshold=156.51981353759766
node_42: feature_name=ENSG00000099250.18	feature_id[42].value > threshold=4.655871629714966
Class: 0	
Rules_35	passed counts:1
node_0: feature_name=ENSG00000149798.5	feature_id[1].value > threshold=34.76214790344238
node_20: feature_name=ENSG00000163346.17	feature_id[70].value > threshold=237.0936508178711
node_34: feature_name=ENSG00000277288.4	feature_id[30].value <= threshold=0.08194983750581741
node_35: feature_name=ENSG00000258763.5	feature_id[31].value <= threshold=0.7273186147212982
node_36: feature_name=ENSG00000254990.5	feature_id[17].value <= threshold=0.1868050992488861
node_37: feature_name=ENSG00000257951.2	feature_id[58].value <= threshold=0.04973463900387287
node_38: feature_name=ENSG00000130522.5	feature_id[10].value > threshold=156.51981353759766
node_42: feature_name=ENSG00000099250.18	feature_id[42].value <= threshold=4.655871629714966
node_43: feature_name=ENSG00000241935.9	feature_id[48].value > threshold=0.2611011117696762
Class: 0	

Rules_36	passed counts:1
node_0: feature_name=ENSG00000149798.5	feature_id[1].value > threshold=34.76214790344238
node_20: feature_name=ENSG00000163346.17	feature_id[70].value > threshold=237.0936508178711
node_34: feature_name=ENSG00000277288.4	feature_id[30].value <= threshold=0.08194983750581741
node_35: feature_name=ENSG00000258763.5	feature_id[31].value <= threshold=0.7273186147212982
node_36: feature_name=ENSG00000254990.5	feature_id[17].value <= threshold=0.1868050992488861
node_37: feature_name=ENSG00000257951.2	feature_id[58].value <= threshold=0.04973463900387287
node_38: feature_name=ENSG00000130522.5	feature_id[10].value > threshold=156.51981353759766
node_42: feature_name=ENSG00000099250.18	feature_id[42].value <= threshold=4.655871629714966
node_43: feature_name=ENSG00000241935.9	feature_id[48].value <= threshold=0.2611011117696762
node_44: feature_name=ENSG00000012048.22	feature_id[14].value > threshold=19.74557590484619
Class: 0	
Rules_37	passed counts:1
node_0: feature_name=ENSG00000149798.5	feature_id[1].value > threshold=34.76214790344238
node_20: feature_name=ENSG00000163346.17	feature_id[70].value > threshold=237.0936508178711
node_34: feature_name=ENSG00000277288.4	feature_id[30].value <= threshold=0.08194983750581741
node_35: feature_name=ENSG00000258763.5	feature_id[31].value <= threshold=0.7273186147212982
node_36: feature_name=ENSG00000254990.5	feature_id[17].value <= threshold=0.1868050992488861
node_37: feature_name=ENSG00000257951.2	feature_id[58].value <= threshold=0.04973463900387287
node_38: feature_name=ENSG00000130522.5	feature_id[10].value > threshold=156.51981353759766
node_42: feature_name=ENSG00000099250.18	feature_id[42].value <= threshold=4.655871629714966
node_43: feature_name=ENSG00000241935.9	feature_id[48].value <= threshold=0.2611011117696762
node_44: feature_name=ENSG00000012048.22	feature_id[14].value <= threshold=19.74557590484619
node_45: feature_name=ENSG00000173239.13	feature_id[60].value > threshold=0.22336432337760925

Class: 0	
Rules_38	passed counts:1
node_0: feature_name=ENSG00000149798.5	feature_id[1].value > threshold=34.76214790344238
node_20: feature_name=ENSG00000163346.17	feature_id[70].value > threshold=237.0936508178711
node_34: feature_name=ENSG00000277288.4	feature_id[30].value <= threshold=0.08194983750581741
node_35: feature_name=ENSG00000258763.5	feature_id[31].value <= threshold=0.7273186147212982
node_36: feature_name=ENSG00000254990.5	feature_id[17].value <= threshold=0.1868050992488861
node_37: feature_name=ENSG00000257951.2	feature_id[58].value <= threshold=0.04973463900387287
node_38: feature_name=ENSG00000130522.5	feature_id[10].value > threshold=156.51981353759766
node_42: feature_name=ENSG00000099250.18	feature_id[42].value <= threshold=4.655871629714966
node_43: feature_name=ENSG00000241935.9	feature_id[48].value <= threshold=0.2611011117696762
node_44: feature_name=ENSG00000012048.22	feature_id[14].value <= threshold=19.74557590484619
node_45: feature_name=ENSG00000173239.13	feature_id[60].value <= threshold=0.22336432337760925
node_46: feature_name=ENSG00000239590.1	feature_id[71].value > threshold=0.1118115596473217
node_74: feature_name=ENSG00000158710.14	feature_id[8].value > threshold=756.7427062988281
node_76: feature_name=ENSG00000185551.15	feature_id[16].value > threshold=0.10993906855583191
Class: 0	
Rules_39	passed counts:1
node_0: feature_name=ENSG00000149798.5	feature_id[1].value > threshold=34.76214790344238
node_20: feature_name=ENSG00000163346.17	feature_id[70].value > threshold=237.0936508178711
node_34: feature_name=ENSG00000277288.4	feature_id[30].value <= threshold=0.08194983750581741
node_35: feature_name=ENSG00000258763.5	feature_id[31].value <= threshold=0.7273186147212982
node_36: feature_name=ENSG00000254990.5	feature_id[17].value <= threshold=0.1868050992488861

node_37: feature_name=ENSG00000257951.2	feature_id[58].value <= threshold=0.04973463900387287
node_38: feature_name=ENSG00000130522.5	feature_id[10].value > threshold=156.51981353759766
node_42: feature_name=ENSG00000099250.18	feature_id[42].value <= threshold=4.655871629714966
node_43: feature_name=ENSG00000241935.9	feature_id[48].value <= threshold=0.2611011117696762
node_44: feature_name=ENSG00000012048.22	feature_id[14].value <= threshold=19.74557590484619
node_45: feature_name=ENSG00000173239.13	feature_id[60].value <= threshold=0.22336432337760925
node_46: feature_name=ENSG00000239590.1	feature_id[71].value <= threshold=0.1118115596473217
node_47: feature_name=ENSG00000233242.2	feature_id[51].value > threshold=0.0636320523917675
node_69: feature_name=ENSG00000233242.2	feature_id[51].value <= threshold=0.06934208422899246
node_70: feature_name=ENSG00000182809.10	feature_id[29].value > threshold=21.95294761657715
Class: 1	
Rules_40	passed counts:1
node_0: feature_name=ENSG00000149798.5	feature_id[1].value > threshold=34.76214790344238
node_20: feature_name=ENSG00000163346.17	feature_id[70].value > threshold=237.0936508178711
node_34: feature_name=ENSG00000277288.4	feature_id[30].value <= threshold=0.08194983750581741
node_35: feature_name=ENSG00000258763.5	feature_id[31].value <= threshold=0.7273186147212982
node_36: feature_name=ENSG00000254990.5	feature_id[17].value <= threshold=0.1868050992488861
node_37: feature_name=ENSG00000257951.2	feature_id[58].value <= threshold=0.04973463900387287
node_38: feature_name=ENSG00000130522.5	feature_id[10].value > threshold=156.51981353759766
node_42: feature_name=ENSG00000099250.18	feature_id[42].value <= threshold=4.655871629714966
node_43: feature_name=ENSG00000241935.9	feature_id[48].value <= threshold=0.2611011117696762
node_44: feature_name=ENSG00000012048.22	feature_id[14].value <= threshold=19.74557590484619
node_45: feature_name=ENSG00000173239.13	feature_id[60].value <= threshold=0.22336432337760925
node_46: feature_name=ENSG00000239590.1	feature_id[71].value <= threshold=0.1118115596473217

node_47: feature_name=ENSG00000233242.2	feature_id[51].value <= threshold=0.0636320523917675
node_48: feature_name=ENSG00000198901.14	feature_id[69].value > threshold=0.39943213760852814
node_52: feature_name=ENSG00000171360.3	feature_id[0].value > threshold=0.37142980098724365
node_66: feature_name=ENSG00000158710.14	feature_id[8].value > threshold=1152.2646484375
Class: 1	
Rules_41	passed counts:1
node_0: feature_name=ENSG00000149798.5	feature_id[1].value > threshold=34.76214790344238
node_20: feature_name=ENSG00000163346.17	feature_id[70].value > threshold=237.0936508178711
node_34: feature_name=ENSG00000277288.4	feature_id[30].value <= threshold=0.08194983750581741
node_35: feature_name=ENSG00000258763.5	feature_id[31].value <= threshold=0.7273186147212982
node_36: feature_name=ENSG00000254990.5	feature_id[17].value <= threshold=0.1868050992488861
node_37: feature_name=ENSG00000257951.2	feature_id[58].value <= threshold=0.04973463900387287
node_38: feature_name=ENSG00000130522.5	feature_id[10].value > threshold=156.51981353759766
node_42: feature_name=ENSG00000099250.18	feature_id[42].value <= threshold=4.655871629714966
node_43: feature_name=ENSG00000241935.9	feature_id[48].value <= threshold=0.2611011117696762
node_44: feature_name=ENSG00000012048.22	feature_id[14].value <= threshold=19.74557590484619
node_45: feature_name=ENSG00000173239.13	feature_id[60].value <= threshold=0.22336432337760925
node_46: feature_name=ENSG00000239590.1	feature_id[71].value <= threshold=0.1118115596473217
node_47: feature_name=ENSG00000233242.2	feature_id[51].value <= threshold=0.0636320523917675
node_48: feature_name=ENSG00000198901.14	feature_id[69].value > threshold=0.39943213760852814
node_52: feature_name=ENSG00000171360.3	feature_id[0].value > threshold=0.37142980098724365
node_66: feature_name=ENSG00000158710.14	feature_id[8].value <= threshold=1152.2646484375
Class: 0	

Rules_42	passed counts:1
node_0: feature_name=ENSG00000149798.5	feature_id[1].value > threshold=34.76214790344238
node_20: feature_name=ENSG00000163346.17	feature_id[70].value > threshold=237.0936508178711
node_34: feature_name=ENSG00000277288.4	feature_id[30].value <= threshold=0.08194983750581741
node_35: feature_name=ENSG00000258763.5	feature_id[31].value <= threshold=0.7273186147212982
node_36: feature_name=ENSG00000254990.5	feature_id[17].value <= threshold=0.1868050992488861
node_37: feature_name=ENSG00000257951.2	feature_id[58].value <= threshold=0.04973463900387287
node_38: feature_name=ENSG00000130522.5	feature_id[10].value > threshold=156.51981353759766
node_42: feature_name=ENSG00000099250.18	feature_id[42].value <= threshold=4.655871629714966
node_43: feature_name=ENSG00000241935.9	feature_id[48].value <= threshold=0.2611011117696762
node_44: feature_name=ENSG00000012048.22	feature_id[14].value <= threshold=19.74557590484619
node_45: feature_name=ENSG00000173239.13	feature_id[60].value <= threshold=0.22336432337760925
node_46: feature_name=ENSG00000239590.1	feature_id[71].value <= threshold=0.1118115596473217
node_47: feature_name=ENSG00000233242.2	feature_id[51].value <= threshold=0.0636320523917675
node_48: feature_name=ENSG00000198901.14	feature_id[69].value > threshold=0.39943213760852814
node_52: feature_name=ENSG00000171360.3	feature_id[0].value <= threshold=0.37142980098724365
node_53: feature_name=ENSG00000273776.1	feature_id[67].value > threshold=0.11133104935288429
node_63: feature_name=ENSG00000240403.5	feature_id[40].value > threshold=3.764121174812317
Class: 0	
Rules_43	passed counts:1
node_0: feature_name=ENSG00000149798.5	feature_id[1].value > threshold=34.76214790344238
node_20: feature_name=ENSG00000163346.17	feature_id[70].value > threshold=237.0936508178711
node_34: feature_name=ENSG00000277288.4	feature_id[30].value <= threshold=0.08194983750581741
node_35: feature_name=ENSG00000258763.5	feature_id[31].value <= threshold=0.7273186147212982

node_36: feature_name=ENSG00000254990.5	feature_id[17].value <= threshold=0.1868050992488861
node_37: feature_name=ENSG00000257951.2	feature_id[58].value <= threshold=0.04973463900387287
node_38: feature_name=ENSG00000130522.5	feature_id[10].value > threshold=156.51981353759766
node_42: feature_name=ENSG00000099250.18	feature_id[42].value <= threshold=4.655871629714966
node_43: feature_name=ENSG00000241935.9	feature_id[48].value <= threshold=0.2611011117696762
node_44: feature_name=ENSG00000012048.22	feature_id[14].value <= threshold=19.74557590484619
node_45: feature_name=ENSG00000173239.13	feature_id[60].value <= threshold=0.22336432337760925
node_46: feature_name=ENSG00000239590.1	feature_id[71].value <= threshold=0.1118115596473217
node_47: feature_name=ENSG00000233242.2	feature_id[51].value <= threshold=0.0636320523917675
node_48: feature_name=ENSG00000198901.14	feature_id[69].value > threshold=0.39943213760852814
node_52: feature_name=ENSG00000171360.3	feature_id[0].value <= threshold=0.37142980098724365
node_53: feature_name=ENSG00000273776.1	feature_id[67].value <= threshold=0.11133104935288429
node_54: feature_name=ENSG00000255356.2	feature_id[63].value > threshold=0.04708113335072994
node_60: feature_name=ENSG00000189430.12	feature_id[53].value > threshold=9.082543849945068
Class: 0	
Rules_44	passed counts:1
node_0: feature_name=ENSG00000149798.5	feature_id[1].value > threshold=34.76214790344238
node_20: feature_name=ENSG00000163346.17	feature_id[70].value > threshold=237.0936508178711
node_34: feature_name=ENSG00000277288.4	feature_id[30].value <= threshold=0.08194983750581741
node_35: feature_name=ENSG00000258763.5	feature_id[31].value <= threshold=0.7273186147212982
node_36: feature_name=ENSG00000254990.5	feature_id[17].value <= threshold=0.1868050992488861
node_37: feature_name=ENSG00000257951.2	feature_id[58].value <= threshold=0.04973463900387287
node_38: feature_name=ENSG00000130522.5	feature_id[10].value > threshold=156.51981353759766
node_42: feature_name=ENSG00000099250.18	feature_id[42].value <= threshold=4.655871629714966

node_43: feature_name=ENSG00000241935.9	feature_id[48].value <= threshold=0.2611011117696762
node_44: feature_name=ENSG00000012048.22	feature_id[14].value <= threshold=19.74557590484619
node_45: feature_name=ENSG00000173239.13	feature_id[60].value <= threshold=0.22336432337760925
node_46: feature_name=ENSG00000239590.1	feature_id[71].value <= threshold=0.1118115596473217
node_47: feature_name=ENSG00000233242.2	feature_id[51].value <= threshold=0.0636320523917675
node_48: feature_name=ENSG00000198901.14	feature_id[69].value > threshold=0.39943213760852814
node_52: feature_name=ENSG00000171360.3	feature_id[0].value <= threshold=0.37142980098724365
node_53: feature_name=ENSG00000273776.1	feature_id[67].value <= threshold=0.11133104935288429
node_54: feature_name=ENSG00000255356.2	feature_id[63].value <= threshold=0.04708113335072994
node_55: feature_name=ENSG00000260231.2	feature_id[49].value <= threshold=6.197221994400024
node_56: feature_name=ENSG00000260231.2	feature_id[49].value > threshold=6.1267735958099365
Class: 0	
Rules_45	passed counts:1
node_0: feature_name=ENSG00000149798.5	feature_id[1].value > threshold=34.76214790344238
node_20: feature_name=ENSG00000163346.17	feature_id[70].value > threshold=237.0936508178711
node_34: feature_name=ENSG00000277288.4	feature_id[30].value <= threshold=0.08194983750581741
node_35: feature_name=ENSG00000258763.5	feature_id[31].value <= threshold=0.7273186147212982
node_36: feature_name=ENSG00000254990.5	feature_id[17].value <= threshold=0.1868050992488861
node_37: feature_name=ENSG00000257951.2	feature_id[58].value <= threshold=0.04973463900387287
node_38: feature_name=ENSG00000130522.5	feature_id[10].value > threshold=156.51981353759766
node_42: feature_name=ENSG00000099250.18	feature_id[42].value <= threshold=4.655871629714966
node_43: feature_name=ENSG00000241935.9	feature_id[48].value <= threshold=0.2611011117696762
node_44: feature_name=ENSG00000012048.22	feature_id[14].value <= threshold=19.74557590484619
node_45: feature_name=ENSG00000173239.13	feature_id[60].value <= threshold=0.22336432337760925

node_46: feature_name=ENSG00000239590.1	feature_id[71].value <= threshold=0.1118115596473217
node_47: feature_name=ENSG00000233242.2	feature_id[51].value <= threshold=0.0636320523917675
node_48: feature_name=ENSG00000198901.14	feature_id[69].value <= threshold=0.39943213760852814
node_49: feature_name=ENSG00000149798.5	feature_id[1].value > threshold=69.77110290527344
Class: 1	
Rules_46	passed counts:1
node_0: feature_name=ENSG00000149798.5	feature_id[1].value > threshold=34.76214790344238
node_20: feature_name=ENSG00000163346.17	feature_id[70].value > threshold=237.0936508178711
node_34: feature_name=ENSG00000277288.4	feature_id[30].value <= threshold=0.08194983750581741
node_35: feature_name=ENSG00000258763.5	feature_id[31].value <= threshold=0.7273186147212982
node_36: feature_name=ENSG00000254990.5	feature_id[17].value <= threshold=0.1868050992488861
node_37: feature_name=ENSG00000257951.2	feature_id[58].value <= threshold=0.04973463900387287
node_38: feature_name=ENSG00000130522.5	feature_id[10].value > threshold=156.51981353759766
node_42: feature_name=ENSG00000099250.18	feature_id[42].value <= threshold=4.655871629714966
node_43: feature_name=ENSG00000241935.9	feature_id[48].value <= threshold=0.2611011117696762
node_44: feature_name=ENSG00000012048.22	feature_id[14].value <= threshold=19.74557590484619
node_45: feature_name=ENSG00000173239.13	feature_id[60].value <= threshold=0.22336432337760925
node_46: feature_name=ENSG00000239590.1	feature_id[71].value <= threshold=0.1118115596473217
node_47: feature_name=ENSG00000233242.2	feature_id[51].value <= threshold=0.0636320523917675
node_48: feature_name=ENSG00000198901.14	feature_id[69].value <= threshold=0.39943213760852814
node_49: feature_name=ENSG00000149798.5	feature_id[1].value <= threshold=69.77110290527344
Class: 0	
Rules_47	passed counts:1

node_0: feature_name=ENSG00000149798.5	feature_id[1].value > threshold=34.76214790344238
node_20: feature_name=ENSG00000163346.17	feature_id[70].value <= threshold=237.0936508178711
node_21: feature_name=ENSG00000099250.18	feature_id[42].value > threshold=1.921088457107544
node_31: feature_name=ENSG00000264204.2	feature_id[9].value <= threshold=5.726377010345459
Class: 1	
Rules_48	passed counts:1
node_0: feature_name=ENSG00000149798.5	feature_id[1].value > threshold=34.76214790344238
node_20: feature_name=ENSG00000163346.17	feature_id[70].value <= threshold=237.0936508178711
node_21: feature_name=ENSG00000099250.18	feature_id[42].value <= threshold=1.921088457107544
node_22: feature_name=ENSG00000201988.2	feature_id[37].value <= threshold=0.06192629598081112
node_23: feature_name=ENSG00000258763.5	feature_id[31].value <= threshold=0.13121316581964493
node_24: feature_name=ENSG00000261402.1	feature_id[12].value <= threshold=0.026882583275437355
node_25: feature_name=ENSG00000264204.2	feature_id[9].value <= threshold=1.6955653429031372
Class: 0	
Rules_49	passed counts:1
node_0: feature_name=ENSG00000149798.5	feature_id[1].value <= threshold=34.76214790344238
node_1: feature_name=ENSG00000073737.16	feature_id[34].value <= threshold=56.694297790527344
node_2: feature_name=ENSG00000238243.3	feature_id[59].value > threshold=3.4281924962997437
node_10: feature_name=ENSG00000103381.12	feature_id[73].value <= threshold=217.66256713867188
node_11: feature_name=ENSG00000104973.18	feature_id[11].value > threshold=80.44169235229492
node_13: feature_name=ENSG00000180304.14	feature_id[56].value > threshold=127.41180419921875
Class: 0	

Rules_50	passed counts:1
node_0: feature_name=ENSG00000149798.5	feature_id[1].value <= threshold=34.76214790344238
node_1: feature_name=ENSG00000073737.16	feature_id[34].value <= threshold=56.694297790527344
node_2: feature_name=ENSG00000238243.3	feature_id[59].value <= threshold=3.4281924962997437
node_3: feature_name=ENSG00000155307.18	feature_id[43].value > threshold=19.13234519958496
node_5: feature_name=ENSG00000165406.16	feature_id[25].value <= threshold=104.79007339477539
node_6: feature_name=ENSG00000158710.14	feature_id[8].value <= threshold=572.8588562011719
Class: 0	

#
